# An Integrative Transcriptomic and Metabolomic Study Revealed That Melatonin Plays a Protective Role in Chronic Lung Inflammation by Reducing Necroptosis

**DOI:** 10.3389/fimmu.2021.668002

**Published:** 2021-05-04

**Authors:** Kaimin Mao, Ping Luo, Wei Geng, Juanjuan Xu, Yuhan Liao, Hua Zhong, Pei Ma, Qi Tan, Hui Xia, Limin Duan, Siwei Song, Danling Long, Yuqi Liu, Tinglin Yang, Yali Wu, Yang Jin

**Affiliations:** ^1^ Department of Respiratory and Critical Care Medicine, Key Laboratory of Pulmonary Diseases of the National Health Commission Union Hospital, Tongji Medical College, Huazhong University of Science and Technology, Wuhan, China; ^2^ Department of Critical Care Medicine, Renji Hospital, School of Medicine, Shanghai Jiaotong University, Shanghai, China; ^3^ Center for Translational Medicine, Union Hospital, Tongji Medical College, Huazhong University of Science and Technology, Wuhan, China; ^4^ College of Life Sciences, Wuhan University, Wuhan, China; ^5^ Department of Stomatology, Taihe Hospital, Hubei University of Medicine, Shiyan, China; ^6^ Department of Cardiovascular Surgery, Union Hospital, Tongji Medical College, Huazhong University of Science and Technology, Wuhan, China

**Keywords:** melatonin, COPD, chronic lung inflammation, transcriptomic, metabolomic, necroptosis

## Abstract

It has been reported that melatonin can relieve the symptoms of chronic obstructive pulmonary disease (COPD) by improving sleep quality, that is to say, the pineal secreted hormone melatonin has a protective effect in the pathogenesis of COPD, but its underlying mechanism remains unclear. In this study, we recruited 73 people into control (n = 22), stable COPD (n = 20), and acute exacerbation of COPD (n = 31) groups to detect the serum melatonin levels. Then, through the mouse model, we employed a systematic study based on the metabolomic and transcriptomic analyses to investigate the molecular mechanisms involved in the progression of the disease. Circulating melatonin in acute exacerbation of COPD patients was decreased compared with that in healthy donors and stable COPD patients. The serum melatonin level was positively correlated with lung function parameters, such as FEV1, FEV1/FVC, and FEV1% predicted in acute exacerbation of COPD patients. Animal experiments showed that melatonin can not only alleviate chronic lipopolysaccharide (LPS)-induced mouse lung destruction and chronic lung inflammation but also reduce necroptosis (RIP1/RIP3/MLKL), a programmed cell death process in bronchial epithelial cells. The protective effect of melatonin on chronic lung inflammation was further suggested to be dependent on targeting its membrane receptor MT1/MT2. In addition, transcriptomic and metabolomic profiling in the lungs of mice indicated that LPS can induce perturbations of the mainstream metabolites associated with amino acid and energy metabolism. Melatonin may reduce the necroptosis by modifying the disordered pathways of alanine, aspartate, and glutamate metabolism caused by LPS. This study suggests that melatonin may act as a potential therapeutic agent for alleviating the chronic inflammation associated with COPD.

## Introduction

The main feature of chronic obstructive pulmonary disease (COPD) is a common respiratory disease with airflow limitation that cannot be completely reversed (1, 2). It has been well acknowledged that cigarette smoke is the most widely associated environmental risk factor for COPD because 90% of patients are smokers or ex-smokers (3). However, in addition to cigarette smoke, some other factors, especially lipopolysaccharide (LPS)-involved burning biomass, are important risk factors for this disease, which may be the cause of a rise in inflammation during acute exacerbation of COPD (4). Delaying or preventing the progression of aberrant inflammatory responses is essential for successful resolution of returning to lung homeostasis. However, it has been acknowledged that chronic lung inflammation responds poorly to current anti-inflammatory treatments. Although antibiotics and glucocorticoids have a function of stabilizing COPD, these therapies cannot arrest the progressive disease or reverse the pathological changes in the lungs.

Melatonin (N-acetyl-5-methoxy-tryptamine) is produced by the pineal gland and can be secreted into the blood circulation (5). Melatonin has various biological activities that improve the health of an individual. In a double-blind, randomized, placebo controlled study, researchers showed that melatonin administration could reduce oxidative stress and improve dyspnea in COPD (6). In addition, it has been reported that melatonin attenuates apoptosis and endoplasmic reticulum (ER) stress in the lung tissues of rats with cigarette smoke- and lipopolysaccharide-induced COPD (7). Melatonin works through receptor-independent mechanisms and receptor-mediated processes, and membrane proteins and nuclear binding sites can affect receptor mediation. Membrane receptors of melatonin include high affinity melatonin receptor 1 (MT1), MT2, and MT3. Interestingly, the activation of MT1/MT2 has been shown to protect against acute lung injury in animal models (8) and polluted air-induced rat lung injury (9). However, until now, no study has elucidated the effect of melatonin on non-cigarette smoke-induced chronic pulmonary inflammation or investigated its underlying molecular mechanisms.

Some existing omics studies on COPD have discovered the differential expression of certain genes, proteins, or metabolites through methods such as genomics, proteomics, or metabolomics, which provide potential sites for COPD screening or typing (10–12). However, no research on the potential genomic-metabolomic changes in chronic lung inflammation has been reported to date. Therefore, clarifying the gene-metabolite profiles during the progression of chronic lung inflammation is of great importance, especially for the development of more effective treatments for the chronic lung inflammation associated with COPD. Common pathogenic pollutants contributing to COPD include LPS, cigarette smoke, air pollution, and organic dust. Among them, LPS is a strong inflammatory stimulant. To further study the role of LPS in COPD, we made mice repeatedly inhale LPS to induce bronchitis and create a COPD-like inflammation mouse model. In this study, we aimed to identify the potential therapeutic effect and underlying mechanism of melatonin. Our animal experiments showed that melatonin can reduce the mouse lung destruction, inflammation, and necroptosis, a programmed cell death process in bronchial epithelial cells, caused by LPS. Integrated metabolomics and transcriptomics analysis revealed that melatonin may reduce necroptosis by modifying the disordered pathways of alanine, aspartate, and glutamate metabolism caused by LPS, which provides an in-depth understanding of the pathophysiology of chronic lung inflammation and may facilitate the development of new treatments for COPD.

## Materials and Methods

### Study Population

In this study, all 73 subjects were enrolled if they conformed to the 2020 classification of the Global Initiative for Chronic Obstructive Lung Disease (GOLD) (13). They were divided into three groups: control (n = 22), stable COPD (n = 20), and acute COPD (n = 31). The control group did not smoke and had no respiratory, circulatory, digestive, or other diseases. Subjects with FEV1/FVC ≤0.7, and no smoking history, no other lung diseases or serious diseases in the major systems, such as bronchiectasis, bronchial asthma, lung cancer, or diseases in the nervous, respiratory, digestive, urinary, and hematological systems, were considered to have COPD. **Table S1** shows the basic information of the patients, including gender, age, routine blood test (including RBC, Hb, WBC, PLT, and serum CRP and PCT), liver and kidney function-related items (including BUN, serum Cr, Ccr, ALT, and AST), and lung function-related items (including FEV1 and FEV1/FVC%).

### Enzyme-Linked Immunosorbent Assay (ELISA)

Human serum samples were melted at room temperature. Then, according to the instructions, a human melatonin ELISA kit (CSB-E08132h, CUSABIO) was used to measure the concentration of melatonin. Detection was performed with a microplate reader (Thermo Scientific, USA) at a wavelength of 450 nm.

### Animal Study Design

Specific pathogen-free (SPF) male C57BL/6 mice were purchased from Charles River (Beijing, China) and housed in the Experimental Animal Center of Tongji Medical College, Huazhong University of Science and Technology. Mice were subjected to light for 12 h/darkness for 12 h, normal diet, and water under SPF conditions with the ambient temperature of 24 ± 2°C. All experiments involving animals were carried out according to the National Institutes of Health Guidelines on the Use of Laboratory Animals, and were approved by the Animal Ethics Committee of Huazhong University of Science and Technology. Male C57BL/6 mice at the age of 8 weeks were randomly divided into four groups. Chronic pulmonary inflammation was induced by an aerosol of phosphate-buffer saline (PBS) alone or PBS containing *Escherichia coli* LPS (0.5 mg/ml; L2880, Sigma) in a custom-built chamber for 2 h daily, 6 days per week for 2 months. At the same time, one group of LPS-exposed mice was treated with vehicle (10% ethanol), and the other two groups of mice were given melatonin (100 mg/L) in drinking water. According to the average amount of water the mice drink per day (5 ml), the daily intake of melatonin should be 0.5 mg. Melatonin-treated mice received vehicle (DMSO) or MT1/MT2 receptor competitive antagonist luzindole (2-benzyl-N-acetyltryptamine, dissolved in DMSO) intraperitoneal injection (5 mg/kg body weight daily) until the end of the experiment. The dosages of melatonin treatment and luzindole treatment were chosen according to their pharmacokinetic profile in mice and because the same dosages had been used in other studies (14, 15). The control group C57BL/6 mice were exposed to air, given water containing 10% ethanol, and received a DMSO intraperitoneal injection daily. The four groups were as follows: control (Con), con+lps (Lps), con+lps+mel (Mel), and con+lps+mel+luz (Luz).

### Microcomputed Tomography (micro-CT)

Following the LPS exposure, mice were sacrificed by the intraperitoneal injection of an overdose of 5% pentobarbital sodium. Mice were placed in the prone position, and the lungs of mice were scanned by microcomputed tomography (Bruker Skyscan 1176). The scanning mode (2000*1336, Al 1 mm) at a resolution of 18 um was chosen. The dynamic image range of the reconstructed CT image is a minimal value of 0 and a maximal value of 0.020385.

### Transmission Electron Microscopy

The tissue block (1 mm) was placed in 0.1 M potassium sodium phosphate buffer containing 2% glutaraldehyde at temperature of 4°C for more than 24 h, and then, a series of graded ethanol solutions were used for tissue dehydration. The tissue block was embedded with epoxy resin, and ultrathin sections were stained with lead citrate. Finally, pictures of the prepared samples were taken according to the operation manual of the JEM1200EX electron microscope (HITACHI, 229 JAPAN) at 80 keV.

### Flow Cytometry Analysis

The mice were euthanized, and the lung tissues were removed, rinsed, and minced in RPMI medium containing 1 mg/ml type 4 collagenase (Sigma) at 37°C for 45 min. Then, the lung cell suspension was filtered with a 100 mm cell strainer, following resuspended, and red blood cells lysing. Subsequently, we immunolabeled the collected cells with the antibodies against CD45, NKP46, Ly6G, CD11c, CD11b, TCRb, CD8a, CD4, CD24 (BioLegend), Siglec-F (BD Biosciences), and Class II major histocompatibility antigen complex (MHC II). The gating strategy for the immune subsets was performed as follows: CD4 (CD45+/TCRb+/CD4), CD8 (CD45+/TCRb+/CD8), neutrophils (CD45+/Ly6G+), NK cells (CD45+/NKp46+), alveolar macrophages (CD45+/CD11c+, Siglec-F+), and interstitial macrophages (CD45+/MHCII+, CD11c+, Siglec-F−, CD11b+, CD24). An LSRFortessaTM X-20 flow cytometer (BD Biosciences) was used to analyze these immunolabeled cells.

### Bronchoalveolar Lavage Fluid Analysis

The lungs were lavaged three times through an intratracheal cannula with 1 ml of phosphate buffer (PBS). Then, bronchoalveolar lavage fluid (BALF) was collected and centrifuged at 700 g for 6 min at 4°C. The supernatant was stored at −80°C for further analysis. Following the instructions, the mouse cytokine/chemokine magnetic bead plate kit (HCYTOMAG-60K, Millipore, Billerica, MA, USA) was used to detect the levels of cytokines. The BALF samples and a series of dilution standard solutions were added to a 96-well plate and incubated overnight at 4°C, with three replicates for each sample. Then, xPONENT 3.1 software was used on the Luminex 200TM machine to detect and evaluate the differences in inflammatory cells by flow cytometry.

### Western Blot Analysis

RIPA buffer, phosphatase, and protease inhibitors (MEC, China) was added to mouse lung or cell samples, and being lysed on ice plate. An equal amount of protein sample was added to each well for electrophoresis in SDS-PAGE, and then transferred to a polyvinylidene fluoride membrane (Millipore, USA). At room temperature, the membrane was washed with TBST and sealed in 5% skimmed milk for 60 min. The membrane was washed three times for 5 min each time and then incubated with primary antibodies against RIP1 (1:500 dilution, 3493, Cell Signaling Technology, USA), RIP3 (phospho S232) (1:500 dilution, ab195117, Abcam, USA), RIP3 (1:500 dilution, sc-374639, Santa-Cruz, USA), MLKL (phospho S345) (1:1,000 dilution, ab196436, Abcam, USA), MLKL (1:500 dilution, sc-293201, Santa-Cruz, USA), and GAPDH (1:1,000–1:2,000 dilution, CST, USA) at 4°C overnight. After membranes were incubated with appropriate secondary antibodies for 1 h at room temperature, protein bands were visualized using enhanced chemiluminescence (Biosharp, Shanghai, China). Signal quantification was performed with ImageJ software.

### Immunohistochemistry and Immunofluorescence Staining

One lung lobe of the mouse was fixed in 4% paraformaldehyde for 24 h, embedded in paraffin, and cut into 5-µm-thick sections. After deparaffinization and hydration, sections were incubated with primary antibodies against CD45 (1:100 dilution, ab10558, Abcam), CD11b (1:100 dilution, ab133357, Abcam), CD11c (1:100 dilution, 97585, Cell Signaling Technology), and F4/80 (1:100 dilution, ab111101, Abcam) at appropriate concentrations. After washing the sections with PBS, diluted biotinylated secondary antibody was added and incubated at room temperature for 30 min. After washing the sections with PBS, streptavidin-biotin complex (SABC, BOSTER, Wuhan, China) was added and incubated for 20 min. Finally, the sections were washed with PBS and then treated with diaminobenzidine (DAB) substrate solution to obtain the desired color intensity.

For tissue immunofluorescence, sections were incubated with primary antibodies against cytokeratin 5 (1:200 dilution, ab52635, Abcam, USA) and MLKL (Phospho-S345) (1:200 dilution, ab196436, Abcam, USA) at appropriate concentrations. After incubation at 4°C overnight, Alexa Fluor 488- or 594-conjugated anti-mouse or anti-rabbit IgG was added, and sections were incubated at room temperature for 60 min. Finally, 4’,6-diamidino-2-phenylindole (DAPI) was added to stain nuclei. Photos were evaluated by an inverted fluorescence microscope (Nikon, Japan).

### Real-Time Polymerase Chain Reaction

Total RNA was extracted from mouse lung samples with TRIzol reagent (Invitrogen, USA). The RNA concentration was measured with a NanoDrop 2000 (Thermo Scientific, USA). Total RNA was reverse transcribed into cDNA using a reverse transcription kit (Takara, Japan), and then, Takara PCR Thermal Cycler Dice (Takara, Japan) was used for detection. GAPDH was used as the internal control, and each sample was repeated. The data were analyzed using the comparative threshold cycle method; that is, the average fold change of the gene in the treated sample relative to the control was used to express the fold change value. **Table S2** lists all of the upstream and downstream primer sequences used in this study in detail.

### Metabolomics Extraction

One hundred microliters of liquid sample were accurately weighed, and 500 ul of H_2_O: methanol (1:4, v/v) solution containing 2% L-2 chlorophenylalanine was used to extract metabolites. Then, 200 ul of chloroform was added and homogenized at −10°C at 50 Hz for 3 min. After vortexing and mixing, ultrasonic extraction was performed in an ice water bath for 10*3 min. The mixture was allowed to settle for 30 min at −20°C after centrifugation at 12,000 rcf at 4°C for 20 min, and the supernatant was placed in a glass bottle and vacuum dried. Then, 80 ul of methoxyamine hydrochloride (15 mg/ml in pyridine) was added, and the sample was shaken for 2 min, followed by incubated at 37°C for 90 min. For derivatization, 80 ul of bis (trimethylsilyl) trifuoroacetamide (BSTFA) reagent with 1% trimethylchlorosilane (TMCS) and 20 ul n-hexane was added, and the mixture was placed at 70°C for 60 min after shaking for 2 min. The samples were kept at room temperature for 30 min and analyzed by GC-MS.

### Gas Chromatography-Mass Spectrometry (GC-MS) Analysis

The analysis was conducted using an Agilent 8890B gas chromatography coupled to an Agilent 5977B mass selective detector, and the ionization voltage was 70 eV (Agilent, USA). Analyte compounds were separated with an HP-5MS (30 m × 0.25 mm × 0.25 µm) capillary column. The GC column temperature was set to hold at 60°C, then increased to 310°C at a rate of 8°C/min and kept at the final temperature for 6 min. The injection volume of derivatives was 1 µl. The temperatures of the ion sources and quadrupole were 230 and 270°C, respectively. Data acquisition was performed in full scan mode with a range of 50–500 m/z.

### Metabolite Identification

GC-MS data were processed with MassHunter workstation Quantitative Analysis (version v10.0.707.0), including raw peak extraction, baselines calibration of data, and deconvolution analysis. The resulting matrix that detected at least 80% in any set of samples was retained. After filtering, minimum metabolite values were inputed, and each metabolic feature was normalized by the sum. Mass spectra of these metabolic features were identified through the Fehin database and NIST database. A total of 1,475 characteristic peaks were detected, and 275 raw metabolites were identified. After preprocessing (RSD <30% and the internal reference is 3,4-dichlorophenylalanine), 180 metabolites could be used for subsequent analysis. A Similarity Score greater than 60 points was chosen, but the Similarity Score from subsequent KEGG annotated differential metabolite is greater than 80 points. Among them, according to the KEGG Compound First Category, 25 peptides, 8 carbohydrates, 7 organic acids, 6 lipids, 3 nucleic acids, 3 vitamins and cofactors, and 2 steroids were identified. The following composition analysis was implemented by the Majorbio cloud platform (https://cloud.majorbio.com/). All metabolite variables were scaled to unit variances prior to PCA (principal component analysis) and scaled to Pareto scaling prior to PLS-DA (Partial Least Squares-Discriminant Analysis). The model validity was estimated from model parameters R2 and Q2. The variable importance in the projection (VIP) was calculated in the PLS-DA model.

### Transcriptome Profiling

Prepared mRNA was sequenced using the Illumina HiSeq 2000 (Illumina, San Diego, CA, USA) platform. Alignment to the musculus reference genome (GRCm38) was performed using Bowtie2 version 2.3.5. Alignment files were sorted and indexed with SAMtools version 1.9. Differential expression analyses were estimated with DESeq2 version 1.24. Genes were then filtered based on the fold change (FC) and *p* value, whereby a *p* value <0.05 and |log2(fold change) | > 1 were considered significant. The Majorbio cloud platform (https://cloud.majorbio.com/) was utilized for subsequent functional annotation and enrichment analysis.

### Integrated Transcriptomics and Metabolomics Data Analysis

The comprehensive analysis of transcriptomics and metabolomics datasets and enrichment analysis of metabolomics data were performed using the “joint pathway analysis” function in the MetaboAnalyst website (www.metaboanalyst.ca). The integrated metabolic pathways include pathways containing both metabolites and metabolic genes. The hypergeometric test was used for the enrichment analysis, as the topology was measured with “Degree Centrality.” Finally, tight integration by combining queries was selected.

### Statistical Analysis

All statistical analyses were performed using Prism 7 (GraphPad, USA) and SPSS Statistics Version 22 (USA). Briefly, all data are presented as the mean ± SEM. Studies comparing two groups were analyzed by the two-sided Student’s t-test. Studies comparing more than two groups were analyzed by ordinary one-way ANOVA with Tukey’s multiple comparisons. All statistical analyses considered p < 0.05 significant. The peak intensities of metabolites and the signal values of gene expression were statistically analyzed by the Student’s t-test between two groups and by the Kruskal-Wallis H test among multiple groups. A multivariate statistical analysis was performed using the “ropls” (Version 1.6.2, http://bioconductor.org/packages/release/bioc/html/ropls.html) R package from Bioconductor on the Majorbio Cloud Platform (https://cloud.majorbio.com). All of the data are presented as the mean, with a level of probability of 0.05 as the criterion for significance. The fold change is a measure describing how much a quantity changes going from the initial to the final value, which was recorded as log2 transformed.

## Results

### Circulating Melatonin Was Decreased in Serum and Was Correlated With Lung Function in Acute Exacerbation of COPD Patients

**Figure 1:**
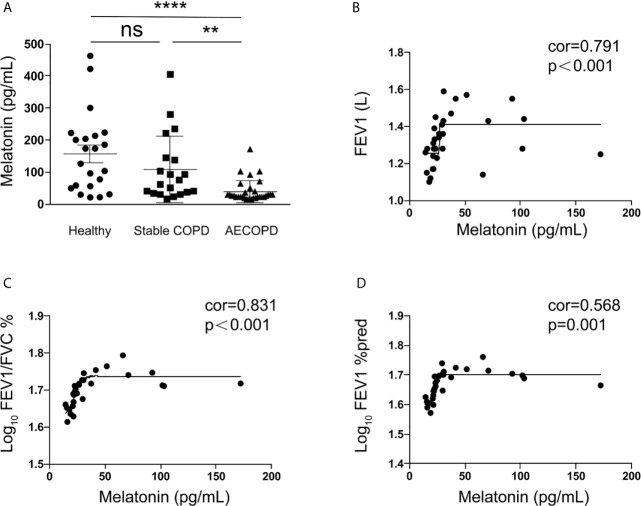
Melatonin was decreased in the serum of acute exacerbation of COPD patients. **(A)** Melatonin in acute exacerbation of COPD patients (40.35 ± 34.87 pg/ml, n = 31) was significantly lower than that in healthy donors (156.90 ± 122.75 pg/ml, n = 22) and stable COPD patients (107.68 ± 103.83 pg/ml, n = 20). **(B–D)** The circulating melatonin level had positive correlation with FEV1 (correlation = 0.791, p < 0.001) and log_10_ (FEV1/FVC) (correlation = 0.831, p < 0.001) and log_10_ (FEV1%pred) (correlation = 0.568, p = 0.001). **p < 0.01, ****p < 0.0001. ns, no significance.

The circulating melatonin in three cohorts, including healthy donors, stable COPD patients, and acute exacerbation of COPD (AECOPD) patients, was determined and compared. As shown in **Figure 1A**, we found that the serum melatonin in AECOPD patients (40.35±34.87 pg/ml) was significantly lower than that in healthy donors (156.90 ± 122.75 pg/ml) and stable COPD patients (107.68 ± 103.83 pg/ml). In addition, the correlations between serum melatonin and FEV1, FEV1/FVC, and FEV1% predicted in AECOPD patients were also analyzed. Association between melatonin and FEV1 was assessed with Spearman correlation analysis, and we found there was a positive correction between serum melatonin and FEV1 (correlation = 0.791, p < 0.001, **Figure 1B**). In order to convert FEV1/FVC and FEV1% pred into normal distributions, FEV1/FVC and FEV1% pred were converted to logarithms. Then association between melatonin with log_10_ (FEV1/FVC) and log_10_ (FEV1% pred) were assessed with Spearman correlation coefficients. We found that melatonin levels also had positive correlation with log_10_ (FEV1/FVC) (correlation = 0.831, p < 0.001, **Figure 1C**) and log_10_ (FEV1% pred) (correlation = 0.568, p = 0.001, **Figure 1D**).

### Melatonin Protects Against LPS-Induced Chronic Lung Inflammation *via* MT1/MT2

**Figure 2:**
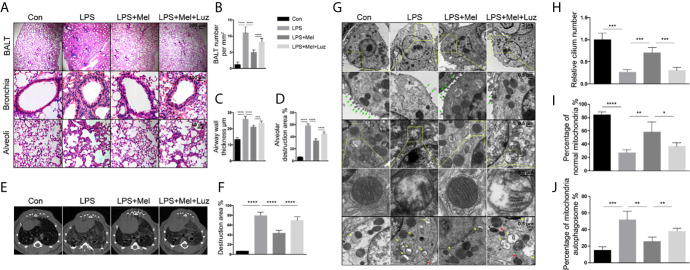
Melatonin protects against chronic LPS-induced mouse lung destruction by the targeting MT1/MT2 receptor. **(A–D)** Melatonin inhibited BALT formation and bronchial thickening as well as alveolar enlargement compared with LPS treatment. Mice receiving luzindole (MT1/MT2 inhibitor) intraperitoneal injection showed diminished protective effects of melatonin in their lungs. N = 5–7 in each group. **(E, F)** Micro-CT indicated that mice treated with melatonin had less destruction in their lungs under chronic LPS exposure. However, luzindole (MT1/MT2 inhibitor) increased the amount of destruction in the lungs even when mice were treated with melatonin. N = 5–7 in each group. **(G–J)** Melatonin preserved the number of cilia and normal morphology of mitochondrial autophagosomes in mouse bronchial epithelial cells under LPS exposure. However, luzindole (MT1/MT2 inhibitor) intraperitoneal injection reduced the cilia and increased the percentage of abnormal mitochondria and mitochondrial autophagosomes, although mice received melatonin orally daily. N = 4 in each group. The green arrow shows cilia, the yellow arrow shows mitochondrial autophagosomes, the red arrow shows autophagosomes, and the black arrow shows lysosomes. *p < 0.05, **p < 0.01, ***p < 0.001, ****p < 0.0001.

Bronchial thickening and alveolar destruction are important characteristics of chronic pulmonary inflammation. After inhaling aerosolized LPS or saline 2 h daily, 6 days per week for 2 months, mice were sacrificed, and lung samples were obtained. We observed a significant increase in the bronchial wall width and airspace enlargement in LPS-exposed C57/BL6 mice compared with mice exposed to saline. However, we found that mice that received melatonin orally daily along with LPS inhaling had smaller bronchial walls and airspaces than LPS-challenged control mice (**Figures 2A–D**). In addition, lymphoid follicles or inducible bronchus-associated lymphoid tissue (iBALT), which is composed of T and B lymphocytes, was significantly increased in the lungs of LPS-exposed mice, and the number of BALT cells in the lungs of mice treated with melatonin was much lower than that in mice exposed to LPS. Surprisingly, however, mice that received an intraperitoneal injection of luzindole had increased bronchial wall thickness and airspace enlargement, although they were given melatonin orally daily (**Figures 2A–D**). In addition, as shown by the micro-CT, the daily oral administration of melatonin effectively prevented the percentage of lung structure destruction induced by LPS. However, the MT1/MT2 inhibitor luzindole diminished the protective effect of melatonin, as we observed much larger destructive in the lungs of this group of mice than in the lungs of melatonin-treated mice (**Figures 2E, F**).

To study the effect of chronic LPS on ultrastructural morphological changes in animal pulmonary cells, we applied transmission electron microscopy to scan the bronchial epithelial cells of mouse lungs. After 2 months of LPS exposure, mouse bronchial epithelial cells had significantly decreased numbers of cilia compared with the unexposed group of mice (**Figures 2G, H**). We also found abnormal mitochondrial changes, such as mitochondrial swelling, and a decrease or disappearance of mitochondrial cristae in LPS-exposed bronchial epithelial cells (**Figures 2G, I**). In addition, electron microscopy indicted that mitophagy can be induced under chronic LPS stimulation because significantly more mitochondrial autophagosomes and autophagolysosomes were found in the lungs of these mice (**Figures 2G, J**). However, oral melatonin treatment preserved the mitochondrial integrity and cilia numbers of bronchial epithelial cells and reduced mitochondrial autophagosome and autophagolysosome formation. When mice were given luzindole intraperitoneally along with melatonin treatment, disrupted mitochondria and more signatures of mitophagy were observed under electron microscopy (**Figure 2G**).

**Figure 3:**
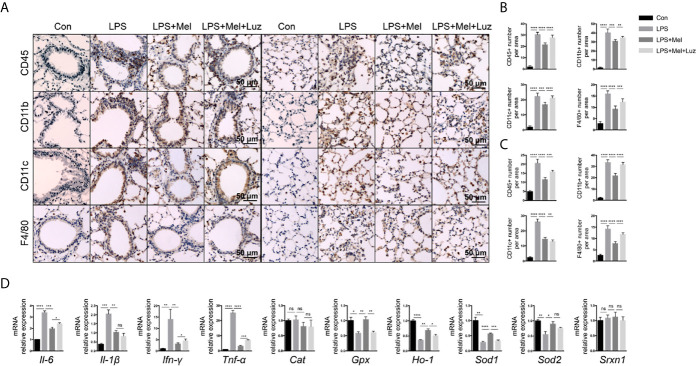
Presenting melatonin at histochemical and RNA levels inhibits chronic inflammation in the lungs of mice exposed to LPS. **(A–C)** Significantly fewer CD45+ T cells, CD11b+ and CD11c+ and F4/80+ macrophages infiltrated into mouse lung bronchi and alveoli when LPS-exposed mice were given melatonin orally daily than non-treated mice. Luzindole (MT1/MT2 inhibitor) increased the infiltration of these inflammatory cells into the mouse lung, even when the mice were treated with melatonin. N = 5–7. **(D)** Mice treated with melatonin expressed lower levels of Il-6, Il-1β, Ifn-γ, and Tnf-α mRNAs as well as more Gpx, Ho-1, Sod1, and Sod2 mRNAs in the lungs than compared with LPS-exposed mice. Mice treated with luzindole (MT1/MT2 inhibitor) along with melatonin had higher levels of Il-6, Ifn-γ, and Tnf-α and decreased Gpx, Ho-1, and Sod1 mRNA expression in the lungs. N = 4–5. *p < 0.05, **p < 0.01, ***p < 0.001, ****p < 0.0001, ns, no significance.

Chronic lung inflammation-related cells, such as CD45+ cells and CD11b+, CD11c+, and F4/80+ macrophages, were stained by immunochemistry in mouse lung sections. We found that there were significantly more CD45+ cells and CD11b+, CD11c+, and F4/80+ macrophages infiltrating the bronchi and alveoli of mice under the chronic exposure to aerosolized LPS. The daily oral administration of melatonin reduced the infiltration of inflammatory cells in mouse lungs. However, when mice were given luzindole, an MT1/MT2 inhibitor, significantly more CD45+ cells and CD11b+, CD11c+, and F4/80+ macrophages were found in the lungs, although mice received oral melatonin daily (**Figures 3A–C**). Inflammatory-related molecules in the lungs of mice were determine. As shown in the mRNA assay in **Figure 3D**, mice treated with melatonin expressed significantly lower levels of *Il-6*, *Il-1β*, *Ifn-γ*, and *Tnf-α* as well as more antioxidant-related mRNAs, such as *Gpx*, *Ho-1*, *Sod1*, and *Sod2*, in the lungs than LPS-exposed mice. However, mice treated with luzindole along with melatonin had higher levels of *Il-6*, *Ifn-γ*, and *Tnf-α* and decreased *Gpx*, *Ho-1*, and *Sod1* mRNAs expression in the lungs. These results indicated a protective effect of melatonin on chronic LPS-induced chronic lung inflammation *via* its membrane receptor MT1/MT2.

**Figure 4:**
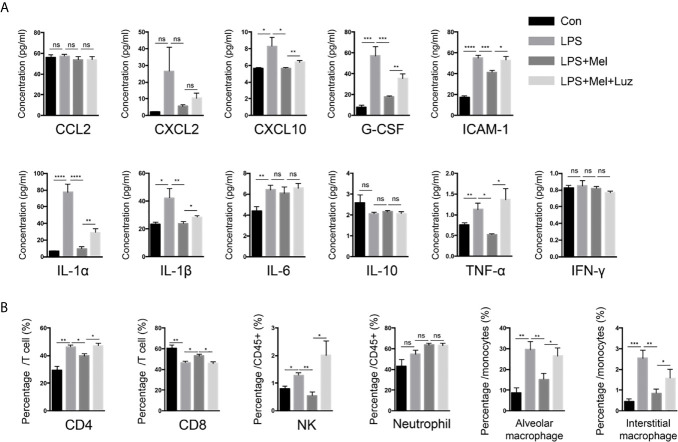
Melatonin decreases chronic LPS exposure-induced inflammatory cells and cytokines in the BALF of mouse lungs. **(A)** A Luminex assay showed that melatonin decreased CXCL10, G-CSF, ICAM-1, and IL-1α, IL-1β, and TNF-α levels in the BALF of LPS-exposed mice. However, luzindole (MT1/MT2 inhibitor) increased these inflammatory factors in BALF. N = 4–5 in each group. **(B)** We determined a higher percentage of CD4+ and CD8+ T cells and NK cells as well as alveolar and interstitial macrophages in the BALF of LPS-exposed mice. Melatonin reduced these cells in BALF, but intraperitoneal luzindole (MT1/MT2 inhibitor) injection abolished the effects of melatonin. *p < 0.05, **p < 0.01, ***p < 0.001, ****p < 0.0001. ns, no significance. N = 4 in each group.

Various lymphoid and myeloid immune cell populations in the bronchoalveolar lavage fluid (BALF) were detected by flow cytometry. Chronic exposure to LPS resulted in a significant influx of CD4+, and CD8+ T cells, interstitial macrophages and alveolar macrophages, and NK cells into the BALF. In melatonin-treated mice, however, the accumulation of each of these immune cell types in the BALF was significantly reduced. Furthermore, we found that the number of these immune cells in the BALF was decreased when mice received luzindole intraperitoneal injection, even with oral melatonin treatment (**Figure 4B**). Representative staining for **Figure 4B** can be seen in the supplementary materials (from **Figures S3–1**–**S3–5**). In addition, inflammatory cytokines in the BALF were also determined with a mouse cytokine/chemokine magnetic bead panel kit. As shown in **Figure 4A**, chronic LPS inhalation significantly upregulated the IL-6, IL-1α, IL-1β, TNF-α, ICAM-1, CXCL2, CXCL10, and G-CSF levels in the mouse BALF, while BALF from melatonin-treated mice had reduced levels of IL-1α, IL-1β, TNF-α, ICAM-1, CXCL2, CXCL10, and G-CSF. Surprisingly, the MT1/MT2 inhibitor luzindole diminished the anti-inflammatory effect of melatonin because we found that these cytokines and chemokines were downregulated in this group of mice.

### Metabolic Signatures Among the Con, LPS, Mel, and Luz Groups

To further investigate the metabolic distinction between the four treatments, we performed PLS-DA analyses. As shown in **Figure S1A**, the score plots of samples were clustered into distinct groups, showing that the discriminations were more obvious. After 200 permutation tests (**Figure S1B**), the low values of the Q2 intercept implied the robustness of the models with a low risk of overfitting and reliability.

**Figure 5:**
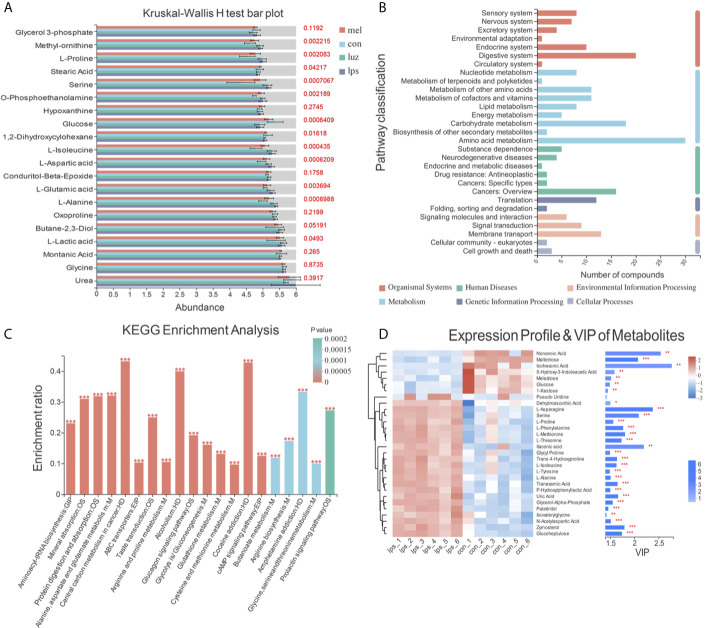
Metabolic signatures among the Con, LPS, Mel, and Luz groups. **(A)** Bar plot of the top 20 presented metabolites in the Kruskal-Wallis H test among the Con, LPS, Mel, and Luz four groups. The y-axis represents the names of metabolites, the x-axis represents the average relative abundance of metabolites in different groups, and the columns with different colors represent different groups. The right-hand side is the p value. **(B)** KEGG enrichment pathway classification of 93 differentially altered metabolites among the Con, LPS, Mel, and Luz groups. The x-axis represents number of compounds, and the y-axis represents the pathway classification name. **(C)** KEGG enrichment results of significantly changed metabolites among Con, LPS, Mel, and Luz. The x-axis represents the top 20 pathway terms, and the y-axis represents the enrichment rate, indicating the ratio between the metabolite number and background number annotated in the pathway. The color gradient of the column indicates the significance of enrichment, where a p value <0.001 is marked as ***, a p value <0.01 as **, and a p value <0.05 as *. **(D)** Hierarchical clustering heatmap of top 30 presented metabolites between the Mel and LPS groups. Each column represents a sample and each row represents a metabolite, and the color represents the relative expression quantity of the metabolite. The corresponding relationship between the color gradient and the numerical value is shown in the gradient color block. On the right is a VIP bar chart for metabolites. The length of the bar indicates the VIP values between the two groups. The default VIP value >1. The bar color indicates a significant difference (p value). N = 6–8 in each group.

Then, multigroup variance analysis and the Kruskal-Wallis H test were performed to identify differential metabolites. After calculating the FDR for multiple tests, the metabolite with FDR < 0.2 was regarded as statistical significance. Consequently, 93 differentially altered metabolites were totally identified among the four groups (**Table S3**). Notably, compared with the Con group, 51 metabolites, such as methyl-ornithine, l-proline, and serine, were enriched in the LPS group, significantly decreased after Mel treatment and finally rebounded after Luz treatment (**Figure 5A**). Moreover, KEGG functional annotation analysis (http://www.genome.jp/kegg/) was applied to search for the metabolic pathways. The KEGG functional enrichment results showed that those differentially presented metabolites were mainly annotated in the aminoacyl-tRNA biosynthesis, alanine, aspartate, and glutamate metabolism, arginine and proline metabolism, and glucagon signaling pathways, which were classified as amino acid metabolism, carbohydrate metabolism, metabolism of cofactors and vitamins, and so on (**Figures 5B, C**). Of note, alanine, aspartate, and glutamate metabolism was one of the key components in necroptosis metabolic pathway (KEGG pathway ID: map04217).

Then, we conducted hierarchical clustering of the top 30 discriminatory metabolites between five pairwise comparisons, and the heatmaps are illustrated in **Figures 5D** and **S2**. The first principal component of VIP was also obtained, and VIP values >1.0 were first selected as the discriminatory metabolites. As shown in **Figure 5D**, increased metabolites, such as isohexonic acid, methoxyamine, and alpha-d-glucose, and decreased metabolites, including l-methionine and l-alanine, were identified in the Mel *vs* LPS comparisons. Meanwhile, there were disparate metabolic profiles among the LPS *vs* Con and Luz *vs* Mel groups (**Figures S2A, B**). Compared with the Con group, nine metabolites (l-isoleucine, l-proline, l-asparagine, l-methionine, l-phenylalanine, serine, l-threonine, l-tyrosine, and itaconic acid) accumulated in the LPS group, were downregulated after Mel treatment and finally rebounded after Luz treatment.

### Transcriptional Profiles Among the Con, LPS, Mel, and Luz Groups

**Figure 6:**
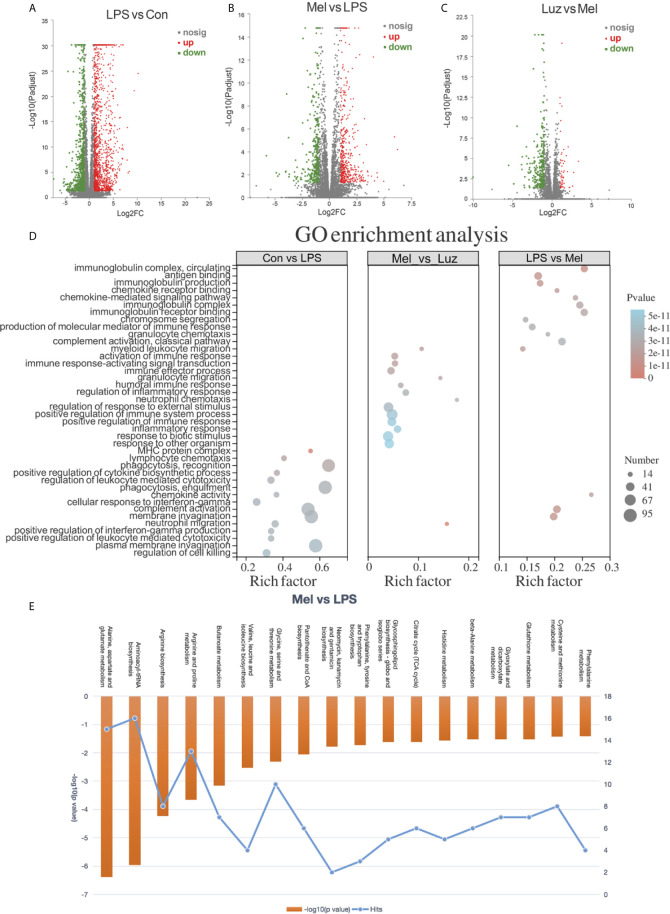
Transcriptional profiles among the Con, LPS, Mel, and Luz groups. Volcano plots show the differentially expressed genes among the LPS *vs* Con **(A)**, Mel *vs* LPS **(B)**, and Luz *vs* Mel **(C)** three comparisons. The x-coordinate represents the log2FC and the y-coordinate represents -log10 (p value). Red points indicate significantly upregulated genes, blue points indicate significantly downregulated genes, and gray points indicate non-significantly different genes. **(D)** Functional GO enrichment analysis of differentially expressed genes among LPS *vs* Con, Mel *vs* LPS, and Luz *vs* Mel. **(E)** Histogram of significantly enriched metabolic pathways by MetaboAnalyst between Mel *vs* LPS (p value <0.05). The left ordinate represents −log10 (p value) and the right ordinate represents the number of genes in the metabolic pathway, and the upper abscissa represents the enhanced metabolic pathways. N = 6–8 in each group.

To detect the role of metabolites playing in transcriptional regulation with the observed alterations, we analyzed transcriptome datasets obtained with the Illumina sequencing platform. In the results, 2,559, 811, and 360 significantly altered (p value <0.05) genes were tested among the LPS *vs* Con, Mel *vs* LPS, and Luz *vs* Mel comparisons, respectively (**Figures 6A–C**). After Mel treatment, the expression levels of 331 genes were upregulated (log2FC > 1) and 480 were downregulated (log2FC < 1) compared with those in the LPS group. Of these, we found that Tcrg-C2, Aif1, and Tnf were suppressed, which were reported to be related to necroptosis (**Table S4**). In addition, we also found that 26 and 5 differentially expressed genes were linked with necroptosis, such as Naip1, Ctsb, and IL6, among the LPS *vs* Con and Luz *vs* Mel comparisons, respectively (**Table S4**).

To gain insight into the biological function of the groups, we implemented gene ontology (GO) analysis based on the differentially expressed genes between groups. GO functional analyses showed that differentially expressed genes between the LPS *vs* Con group were associated with cellular structure, biotic stimulus, and the regulation of cell killing. Between the Mel *vs* LPS groups, GO analysis of differentially expressed datasets denoted alterations in regulation of immune system process and regulation of response to external stimulus. Moreover, GO terms between Luz *vs* Mel were mainly enriched in the immune response-regulating signaling pathway and granulocyte chemotaxis (**Figure 6D**).

### Melatonin Prevents Necroptosis (RIP1/RIP3/MLKL) in Bronchial Epithelial Cells by Targeting the MT1/MT2 Receptor

**Figure 7:**
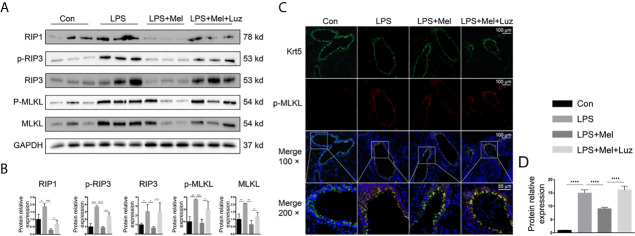
Melatonin inhibits chronic LPS-induced necroptosis in mouse bronchial epithelial cells. **(A, B)** Melatonin downregulated necroptosis-related protein expression, such as RIP1, p-RIP3, total RIP3, and phosphorylated and total MLKL, in LPS-exposed mouse lungs. However, luzindole (MT1/MT2 inhibitor) promoted necroptosis- related protein expression in mouse lungs. **(C, D)** Immunofluorescence in lung tissues showed that melatonin decreased p-MLKL protein expression in LPS-treated mouse bronchial epithelial cells, while luzindole (MT1/MT2 inhibitor) reversed this effect. N = 3 in each group. *p < 0.05, **p < 0.01, ***p < 0.001, ****p < 0.0001.

Necroptosis is a genetically programmed and regulated form of necrosis that is involved in cigarette smoke-induced COPD. Moreover, necroptosis has been reported to participate in mitophagy and mitochondrial dysfunction in bronchial epithelial cells in the pathological process of COPD. We have found significantly more mitophagy-related phenotypes in LPS-treated mouse lungs than in saline-inhaled mice (**Figures 2G, J**). Moreover, the transcriptomic and metabolomic profiling suggested that necroptosis was involved in the progression of chronic lung inflammation. As a result, we assessed necroptosis in animal lungs. According to the protein immunoblotting assay in **Figures 7A, B** chronic LPS inhalation increased phosphorylated and total necroptosis-related receptor-interacting protein-1 and -3 (RIP1/3) kinase and MLKL protein expression. The daily oral administration of melatonin downregulated necroptosis-related protein levels in mouse lung lysates. However, in the luzindole-treated group of mouse lungs, significantly more phosphorylated and total RIP3 and MLKL were detected. Furthermore, we located necroptosis-related p-MLKL (phospho S345) protein by using immunofluorescence staining. The results indicated that bronchial epithelial cells, which were specifically labeled with cytokeratin 5 (krt5), had differential p-MLKL protein expression in each group of mouse lungs (**Figures 7C, D**), suggesting that melatonin prevented LPS- induced necroptosis in bronchial epithelial cells *via* the membrane MT1/MT2 receptor.

### Integration of Metabolomics and Transcriptomics Revealed the Changes in Necroptosis-Related Alanine, Aspartate, and Glutamate Metabolism Pathways

To further confirm our metabolomics results, we comprehensively analyzed the transcriptomics and metabolomics database using the MetaboAnalyst software. Integrated analysis of both genes and metabolites among three comparisons validated the impact of LPS, Mel, and Luz on necroptosis metabolism (**Table S5**). Among the 37 common enriched pathways in the three comparisons, one key pathway was important to necroptosis, namely, alanine, aspartate, and glutamate metabolism (map00250). For example, between the Mel *vs* LPS group, alanine, aspartate, and glutamate metabolism had high enrichment and significance in enriched pathways involved in necroptosis metabolism (**Figure 6E**). In this pathway, seven metabolites and one gene were enriched (N-acetyl-L-aspartate, L-Aspartate, L-Asparagine, L-Alanine, L-Glutamate, 4-Aminobutanoate, N-Carbamoyl-L-aspartate, and Il4i1).

**Figure 8:**
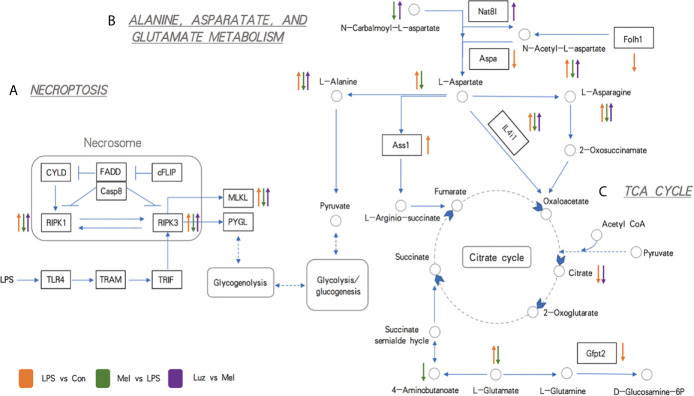
Schematic representations of the most relevant metabolic and transcriptional differences between three pairwise comparisons. **(A)** Metabolic pathways related to necroptosis. **(B)** Metabolic pathways related to alanine, aspartate, and glutamate metabolism. **(C)** Metabolic pathways related to the TCA cycle. The up arrow indicates significantly higher concentrations of metabolites and expression levels of genes, while the down arrow indicates significantly lower concentrations of metabolites and expression levels of genes. The circles indicate metabolites, the rectangles indicate genes (the blue background rectangles highlight genes tested further by experiments), and the underlined italic letters indicate pathways. Orange, green, and purple arrows represent LPS *vs* Con, Mel *vs* LPS, and Luz *vs* Mel comparisons, respectively.

For the alanine, aspartate, and glutamate metabolism pathways, we found that amino acid metabolites, including l-alanine, l-aspartate, l-asparagine, l-glutamate, n-carbalmoyl-l-aspartate, and n-acetyl-l-aspartate, and the related enzyme IL4i1 were significantly altered in the three comparisons. In addition, the pyruvate from the alanine, aspartate, and glutamate metabolism then participates in the TCA cycle. Pyruvate is converted into acetyl-CoA and condensed with oxaloacetate to form citrate (**Figure 8**). Our findings showed that perturbations of the mainstream metabolites were associated with changes in amino acid and energy metabolism.

## Discussion

COPD is associated with chronic inflammation of the airway and lung parenchyma. The inflammation is further aggravated during the acute exacerbation of the disease and is also associated with systemic inflammation (16). Even after long-term smoking cessation, inflammation will continue to exist in COPD patients. Its molecular and cellular mechanisms are still unclear, but this may be a new treatment target for this disease (17). In a study of the effect of melatonin on COPD, researchers found that melatonin can decrease oxidative stress, improve the symptoms of dyspnea, and improve the sleep quality, sleep latency, sleep efficacy, and sleep duration of COPD patients but has no effect on the lung function and exercise capacity (6, 18, 19). We tested the serum melatonin level of patients with active COPD and found that it was significantly lower than that in healthy people and patients with stable COPD. In addition, we also found that in COPD patients with an aggravated status, the circulating melatonin level had obvious positive correlations with the FEV1/FVC ratios and FEV1% predicted. It has been reported that melatonin can bind to membrane receptors MT1/MT2 to exert a protective effect (20). In this study, we proved that melatonin can reduce the structural destruction of mouse lung tissue by chronic LPS inhalation, as well as reduce lung inflammation, protect bronchial epithelial cell mitochondrial function, and inhibit mitophagy. These protective effects disappear after the cell membrane receptors MT1/MT2 are blocked, indicating that melatonin plays a protective role *via* membrane receptors MT1/MT2 in LPS-induced chronic lung inflammation.

Furthermore, to explore the genomic and metabolomic changes in melatonin in the process of COPD-related chronic lung inflammation, we screened the metabolic and transcriptional profiles of the LPS *versus* Con group, Mel *versus* LPS group, and Luz *versus* Mel group in mouse models. We discovered that 51 metabolites were significantly decreased after melatonin treatment and finally rebounded after luzindole treatment, most of which were involved in amino acid metabolism, carbohydrate metabolism, and the metabolism of cofactors and vitamins. Moreover, 2,559, 811, and 360 differentially expressed genes were recognized among the LPS *versus* Con, Mel *versus* LPS, and Luz *versus* Mel comparisons, respectively. The enrichment of immune response-related pathways was also indicated by the presence of multiple pathways in GO functional enrichment analyses. Through transcriptomic and metabonomic sequencing of mouse lung tissue, a series of different small molecules and metabolites were found, including CXCL10, IL-1β, IL-6, TNF-α, G-CSF, Sod1, CatmRNA, RIP1/3, and MLKL, were found, and KEGG analysis found 72 metabolites involved in ABC transporter, protein digestion and absorption, the glucagon signaling pathway, and glycine, serine, and threonine metabolism (including 1-serine, 1-aspartate, and pyroglutamate). Another 370 transcripts were found to be significantly and differentially expressed (118 upregulated and 152 downregulated) through the Illumina sequencing platform.

Among the genomic and metabonomic findings, we found that the necroptosis-related pathway is involved in the disease process. Cell death is closely related to the life activities of living organisms. The balance between cell death, proliferation, and differentiation is essential for maintaining the homeostasis of the entire living system (21). Recently, some studies have indicated the mechanistic link between chronic lung inflammation and a well-designed pro-inflammatory form of programmed cell death (22). Necroptosis is defined as RIP3-dependent and caspase-independent programmed necrosis, which can be caused by activation of TNF receptor 1 (TNFR1). In this process, RIP1 phosphorylates RIP3 to form necrosomes. After assembly, RIP3 phosphorylates mixed-lineage kinase domain-like pseudokinase (MLKL). Phosphorylated MLKL oligomerizes and translocates to the cell membrane, forming a plasma membrane pore and causing cell swelling (22). The use of necrostatin-1 (RIP1 antagonist) can alleviate the neutrophil inflammation in a mouse model induced by cigarette smoke (23). Studies have pointed out that the expression of PINK1 and RIP3 is increased in the lungs of COPD patients. These two proteins are related to mitophagy and necroptosis pathways. In addition, necroptosis was observed to directly induce inflammation through the release of massive DAMPs, which promoted the progression of COPD (24, 25). This evidence indicated that necroptosis-associated cell death was a potential therapeutic target for unsolved lung problems. Studies have shown that mitophagy of lung epithelial cells depends on necroptosis and is involved in the pathogenesis of COPD (24, 26). Our previous electron microscopy results showed that chronic LPS inhalation can promote the formation of mitochondrial autophagosomes in mouse lung bronchial epithelial cells, and melatonin can reduce the formation of such autophagosomes. Experiments in mice have shown that melatonin can inhibit the expression and phosphorylation of RIP1, RIP3, and MLKL in bronchial epithelial cells under chronic LPS exposure, while blocking the membrane receptor MT1/MT2 of melatonin reversed this effect. The suggests that melatonin can inhibit the necroptosis-related pathways in the process of chronic lung inflammation *via* the membrane receptors MT1/MT2.

In addition, Qiu et al. has demonstrated that the consumption of pyruvate, the inhibition of pyruvate transporters in mitochondria, and the inhibition of pyruvate transport proteins in mitochondria can all inhibit TNF-mediated necroptosis (27). ERK is a downstream molecule of RIP1, and RIP1 is an important protein that mediates necroptosis. L-Glutamate treatment can enhance the phosphorylation level of ERK1/2 (28). Lou et al. pointed out that abnormal expression of N-acetyl-L-aspartate (NAA) was detected in non-small cell lung cancer cell (NSCLC) lines. Further experiments proved the cancer specificity of NAA and its synthase (Nat8l) (29). Patients with lung adenocarcinoma have a poor prognosis and high mortality. It has been reported that a new treatment method is proposed to eliminate autophagy and L-asparagine (30). Citrate can act on the IGF-1R-AKT-PTEN-eIF2a pathway to inhibit the growth of A549 lung cancer, and metabolic profile analysis has shown that it inhibits glycolysis and the tricarboxylic acid cycle in tumor cells both *in vivo* and *in vitro* (31).

Prostate-specific membrane antigen (PSMA) can be detected on the plasma membrane of normal human prostate and prostate cancer. The homolog in mice is FOLH1 (32). The study by Wang et al. found that PSMA is also expressed in tumor cells of most NSCLC patients and is a good marker (33). The aspergillus mold passes through alveolar epithelial cells (AECs) when it infects the lungs, of which AEC II is more important. In the proteomics analysis, a study reported that the abundance of IL4i1 increased significantly (34). Among the 37 common enriched pathways in the three comparisons, one key pathway was alanine, aspartate, and glutamate metabolism, one of the important parts of necroptosis metabolism. In the alanine, aspartate, and glutamate metabolism pathways, the key enzyme IL4i1 and mainstream metabolites, such as L-alanine and L-asparagine, were significantly attenuated under melatonin treatment and enhanced after luzindole treatment. This outcome indicates that the suppressed alanine and glutamate metabolism may decrease to supply the raw materials for amino acid synthesis under melatonin treatment.

Our study still requires further exploration. First, this study did not explore in depth how melatonin MT1/MT2 inhibits the necroptotic pathway in depth. Second, a clinical trial of melatonin in the treatment of chronic inflammation in COPD patients is our future plan.

## Conclusion

The results of various experiments in this study show that melatonin has a protective effect on chronic LPS-induced bronchial thickening and alveolar destruction, while blocking MT1/MT2 with luzindole can reduce this protective effect. In addition, melatonin can reduce LPS-induced chronic lung inflammation, preserve the cilia and mitochondria of bronchial epithelial cells, reduce mitophagy under chronic LPS exposure, and what’s more, prevent bronchial epithelial cell necroptosis. The integrated results of our metabolomics and transcriptomics experiments indicated that LPS may alter the alanine, aspartate, and glutamate metabolism signaling pathways, including pyruvate participating in the TCA cycle, which is related to necroptosis, to regulate further cellular processes. Therefore, melatonin may be a novel drug for alleviating chronic inflammation in COPD.

## Data Availability Statement

The datasets presented in this study can be found in online repositories. The names of the repository/repositories and accession number(s) can be found in the article/**Supplementary Material**.

## Ethics Statement

The studies involving human participants were reviewed and approved by Ethics Review Board at Union Hospital, Huazhong University of Science and Technology. The patients/participants provided their written informed consent to participate in this study. The animal study was reviewed and approved by Ethics Review Board at Union Hospital, Huazhong University of Science and Technology. Written informed consent was obtained from the individual(s) for the publication of any potentially identifiable images or data included in this article.

## Author Contributions

KM: Conceptualization, Methodology, Formal analysis, Writing—Original Draft. PL: Data Curation, Visualization. WG: Investigation, Writing—Original Draft. JX: Resources, Supervision. YL: Formal analysis. HZ: Data Curation. PM: Methodology. QT: Methodology. HX: Resources. LD: Visualization. SS: Visualization. DL: Formal analysis. YQL: Resources. TY: Writing—Original Draft. YW: Formal analysis. YJ: Funding acquisition, Writing—Review and Editing. All authors contributed to the article and approved the submitted version.

## Funding

The study was supported by grants from the National Major Scientific and Technological Special Project for “Significant New Drugs Development” (No. 2019ZX09301-001), the National Science Foundation of China (Nos. 81770096, 81802113), Key Laboratory Open Fund of Hubei Province (No: F016.02004.20003.082).

## Conflict of Interest

The authors declare that the research was conducted in the absence of any commercial or financial relationships that could be construed as a potential conflict of interest.

## Supplementary Material

Supplementary Figure 1 Score plots of PLS-DA based on the metabolic profile of COPD. **(A)** The plot of PLS-DA scores showing almost complete separation of Mel (red circles), Luz (blue triangles), LPS (green rhombi), and Con (gray squares). The classification parameters were R2X (cum) = 0.65, R2Y (cum) = 0.871, and Q2 (cum) = 0.536. **(B)** Validation model of PLS-DA. The R2 and Q2 intercept values were 0.4356 and −0.4317, respectively, after 200 permutations.

Supplementary Figure 2 Hierarchical clustering heatmaps of the top 30 presented metabolites between LPS *vs* Con **(A)** and Luz *vs* Mel **(B)**.

Supplementary Figure 3–1 Representative staining of CD4/CD8 in **Figure 4B**.

Supplementary Figure 3–2 Representative staining of neutrophils in **Figure 4B**.

Supplementary Figure 3–3 Representative staining of NK cells in **Figure 4B**.

Supplementary Figure 3–4 Representative staining of alveolar macrophages in **Figure 4B**.

Supplementary Figure 3–5 Representative staining of interstitial macrophages in **Figure 4B**.

Supplementary Table 1 Clinical characteristics of patients and healthy donors in this study.

Supplementary Table 2 The primer pairs for real-time PCR used in this study.

Supplementary Table 3 Differentially altered metabolites among the LPS vs Con, Mel vs LPS, and Luz vs Mel groups.

Supplementary Table 4 Differentially expressed genes among the LPS vs Con, Mel vs LPS, and Luz vs Mel groups. The red background highlights necroptosis-related genes.

Supplementary Table 5 Integrative metabolic pathway enrichment analysis results by MetaboAnalyst.

## Abbreviations

COPD, chronic obstructive pulmonary disease; LPS, lipopolysaccharide; ER, endoplasmic reticulum; MT1/MT2, melatonin receptor 1/2; GOLD, Global Initiative for Chronic Obstructive Lung Disease; FEV1/FVC, ratio of forced expiratory volume in 1 s to forced vital capacity; ELISA, Enzyme-linked immunosorbent assay; SPF, Specific Pathogen-Free; micro-CT, micro-computed tomography; PBS, phosphate buffer; BALF, Bronchoalveolar Lavage Fluid; GAPDH, glyceraldehyde-3-phosphate dehydrogenase; DAB, diaminobenzidine; DAPI, 4’,6-diamidino-2-phenylindole; BSTFA, Bis (trimethylsilyl) Trifluoroacetamide; TMCS, Trimethylchlorosilane; GC-MS, Gas Chromatography-Mass Spectrometry; EI, electron impact ionization; QC, quality control; RSD, relative standard deviation; PLS-DA, Partial Least Squares-Discriminant Analysis; VIP, variable importance projection; KEGG, Kyoto Encyclopedia of Genes and Genomes; ANOVA, Analysis of Variance; AECOPD, acute exacerbation chronic obstructive pulmonary disease; iBALT, inducible bronchus-associated lymphoid tissue; Con, control; Mel, melatonin; Luz, luzidole; RIP, receptor-interacting protein; krt5, cytokeratin 5; TNFR1, tumor necrosis factor receptor 1; MLKL, mixed-lineage kinase domain-like pseudokinase; NAA, N-Acetyl-L-aspartate; NSCLC, non-small cell lung cancer cell; PSMA, prostate-specific membrane antigen; AEC, alveolar epithelial cells.
