# Supplementary material for: An Integrative Transcriptomic and Metabolomic Study Revealed That Melatonin Plays a Protective Role in Chronic Lung Inflammation by Reducing Necroptosis
Source: Front Immunol. 2021 May 4;12:668002. doi: 10.3389/fimmu.2021.668002 (PMC8129533; doi:10.3389/fimmu.2021.668002)
Supplement: Supplementary Figure 1 — Score plots of PLS-DA based on the metabolic profile of COPD. (A) The plot of PLS-DA scores showing almost complete separation of Mel (red circles), Luz (blue triangles), LPS (green rhombi), and Con (gray squares). The classification parameters were R2X (cum) = 0.65, R2Y (cum) = 0.871, and Q2 (cum) = 0.536. (B) Validation model of PLS-DA. The R2 and Q2 intercept values were 0.4356 and −0.4317, respectively, after 200 permutations. [file DataSheet_1.zip › Table S1.pdf]

Table S1. Clinical characteristics of patients and healthy donors in this study.

Characteristics	Healthy (n=22)	Stable COPD (n=20)	Acute Exacerbation COPD (n=31)
Age （year）	68.32±6.88	65.40±5.76	64.90±7.96
Gender （male/female）	15/7	13/7	22/9
Smoke (n)	17	16	23
BMI（kg/m^2）	26.1±1.1	24.2±1.3	25.7±1.4
RBC（$10^{12}/L$）	4.8±0.3	4.0±0.6	4.2±0.7
Hb（g/L）	147±20	154±13	165.94±13.95
WBC（$10^9/L$）	6.86±1.04	5.66±1.05	10.23±1.79
PLT（$10^9/L$）	242±75	225±66	211±59
Serum CRP（mg/L）	2.06±1.32	6.51±0.92	15.16±1.15
PCT（ng/mL）	0.21±0.09	0.52±0.07	0.87±0.14
BUN（mmol/L）	5.0±1.3	6.9±1.0	5.7±2.3
Serum Cr（umol/L）	70.9±38.2	71.7±11.9	75.0±12.3
Ccr （mL/min）	105.7±10.7	112.7±3.9	90.8±15.6
ALT（U/L）	19.9±11.7	28.3±17.8	27.5±15.1
AST（U/L）	22.1±6.5	23.5±9.4	23.0±10.4
FEV1（L）	2.71±0.18	1.44±0.11*	1.33±0.14#&
FEV1/FVC%	85.51±6.33	61.97±4.21*	50.41±4.84#&
FEV1%pred	94.82±1.51	67.73±3.54*	47.03±5.01#&

BMI: body mass index; RBC: red blood cell; Hb: hemoglobin; WBC: white blood cell; PLT: platelet; CRP: C-reactive protein; PCT: procalcitonin; BUN: blood urea nitrogen; Cr: creatinine; Ccr: creatinine clearance; ALT: alanine aminotransferase; AST: aspartate aminotransferase; FEV1: forced expiratory volume in one second; FVC: forced vital capacity; % pred: % predicted

*: significantly different in stable COPD versus healthy donors; #: significantly different in acute exacerbation COPD versus healthy donors; &: significantly different in acute exacerbation COPD versus stable COPD.
