# Supplementary material for: An Integrative Transcriptomic and Metabolomic Study Revealed That Melatonin Plays a Protective Role in Chronic Lung Inflammation by Reducing Necroptosis
Source: Front Immunol. 2021 May 4;12:668002. doi: 10.3389/fimmu.2021.668002 (PMC8129533; doi:10.3389/fimmu.2021.668002)
Supplement: Supplementary Figure 1 — Score plots of PLS-DA based on the metabolic profile of COPD. (A) The plot of PLS-DA scores showing almost complete separation of Mel (red circles), Luz (blue triangles), LPS (green rhombi), and Con (gray squares). The classification parameters were R2X (cum) = 0.65, R2Y (cum) = 0.871, and Q2 (cum) = 0.536. (B) Validation model of PLS-DA. The R2 and Q2 intercept values were 0.4356 and −0.4317, respectively, after 200 permutations. [file DataSheet_1.zip › Table S2.pdf]

Table S2. The primer pairs for Real-time PCR used in this study.

Gene ID	Name	Primer sequence 5'-3'
14433	GAPDH	AGGTCGGTGTGAACGGATTTG
		TGTAGACCATGTAGTTGAGGTCA
21926	TNF-α	CCCTCCTGGCCAACGGCATG
		TCGGGGCAGCCTTGTCCCTT
16176	IL-1β	GCCTCGTGCTGTCGGACCCATAT
		TCCTTTGAGGCCCAAGGCCACA
16193	IL-6	AGACAAAGCCAGAGTCCTTCAGAGA
		GCCACTCCTTCTGTGACTCCAGC
15978	IFN-γ	GCTGTTACTGCCACGGCACAGT
		CACCATCCTTTTGCCAGTTCCTCC
16153	IL-10	GGCTGGACGAGAGCCGAACG
		CCCGGGGTGTAGGCACCACT
14775	Gpx1	AGTCCACCGTGTATGCCTTCT
		GAGACGCGACATTCTCAATGA
20655	SOD1	AACCAGTTGTGTTGTCAGGAC
		CCACCATGTTTCTTAGAGTGAGG
20656	SOD2	CAGACCTGCCTTACGACTATGG
		CTCGGTGGCGTTGAGATTGTT
12359	CAT	AGCGACCAGATGAAGCAGTG
		TCCGCTCTCTGTCAAAGTGTG
15368	HO-1	AAGCCGAGAATGCTGAGTTCA
		GCCGTGTAGATATGGTACAAGGA
76650	SRXN1	ATCGTGGTGCTGGATTGATTC
		CACCCCAGAGATAAGATTACCCA
