# Supplementary material for: An Integrative Transcriptomic and Metabolomic Study Revealed That Melatonin Plays a Protective Role in Chronic Lung Inflammation by Reducing Necroptosis
Source: Front Immunol. 2021 May 4;12:668002. doi: 10.3389/fimmu.2021.668002 (PMC8129533; doi:10.3389/fimmu.2021.668002)
Supplement: Supplementary Figure 1 — Score plots of PLS-DA based on the metabolic profile of COPD. (A) The plot of PLS-DA scores showing almost complete separation of Mel (red circles), Luz (blue triangles), LPS (green rhombi), and Con (gray squares). The classification parameters were R2X (cum) = 0.65, R2Y (cum) = 0.871, and Q2 (cum) = 0.536. (B) Validation model of PLS-DA. The R2 and Q2 intercept values were 0.4356 and −0.4317, respectively, after 200 permutations. [file DataSheet_1.zip › Table S3.pdf]

Table S3. 93 differentially altered metabolites among Con, LPS, MeL and Luz groups.

Metabolite	P_value	FDR	mel_mean	mel_SD	con_mean	con_SD	luz_mean	luz_SD	lps_mean	lps_SD
Sophorose	0.02979	0.0654	2.438	0.08343	2.331	0.04366	2.401	0.0719	2.376	0.03257
Maleimide	0.005788	0.01975	2.031	0.1249	2.148	0.7329	2.255	0.2604	2.39	0.1354
Guanidinosuccinate	0.003161	0.01293	2.262	0.1654	2.011	0.1777	2.366	0.07741	2.391	0.08208
Glucoheptulose	0.0003069	0.005531	2.215	0.1162	1.904	0.105	2.288	0.1553	2.414	0.06009
Serotonin	0.002826	0.01223	2.284	0.09704	2.168	0.1329	2.446	0.1144	2.453	0.09685
N,N-Bis(hydroxyethyl)-2-aminoethanesulfonic acid	0.01716	0.04542	2.375	0.09763	2.321	0.05713	2.347	0.1056	2.529	0.08775
Trans-4-Hydroxyproline	0.000632	0.005531	2.407	0.132	2.073	0.1632	2.615	0.1482	2.558	0.08779
Homoserine	0.001351	0.008708	2.415	0.1487	2.346	0.09441	2.571	0.123	2.662	0.1188
D-Glyceraldehyde 3-phosphate	0.0122	0.03543	2.551	0.1447	2.695	0.1307	2.43	0.1519	2.766	0.3653
1-Monoheptadecanoyl Glyceride	0.03112	0.06669	2.882	0.1162	2.69	0.1304	2.88	0.1089	2.785	0.1178
2-Ketoglucose Dimethylacetal	0.007073	0.02315	2.903	0.0856	2.634	0.1607	2.824	0.07367	2.84	0.064
N-Acetylaspartic Acid	0.000116	0.004638	2.746	0.08756	2.461	0.1603	3	0.1329	2.88	0.06683
Tranexamic Acid	0.002203	0.01022	2.813	0.1453	2.404	0.1485	2.889	0.1647	2.884	0.06384
2-Hydroxy-2-	0.009439	0.02883	2.71	0.1756	2.604	0.1824	2.858	0.1293	2.904	0.1038

Methylbutanoic Acid										
Adenosine	0.0407	0.08286	2.991	0.07152	2.814	0.1906	2.873	0.06927	2.919	0.1018
Galactitol	0.006221	0.02074	2.873	0.09197	2.781	0.09396	2.976	0.08192	2.969	0.08199
Phenol	0.03088	0.06669	3.029	0.05186	2.966	0.1685	2.947	0.04212	2.992	0.08348
1,3-Dihydroxypyridine	0.04573	0.09046	2.983	0.04831	2.941	0.08523	2.954	0.04657	3.008	0.03935
N-Acetylputrescine	0.03268	0.0688	3.083	0.1012	3.042	0.0715	3.167	0.05349	3.083	0.112
D-Ribose-5-Phosphate	0.01741	0.04542	3.178	0.07845	2.957	0.1803	3.118	0.08147	3.083	0.04305
Cis-Gondoic Acid	0.04097	0.08286	3.164	0.04098	3.059	0.1261	3.122	0.06481	3.092	0.08753
D-Xylitol	0.01423	0.04002	2.999	0.1013	2.935	0.1462	2.989	0.08376	3.21	0.1149
Beta-Gentiobiose	0.02626	0.05962	2.997	0.0874	2.757	0.1747	2.953	0.06607	3.225	0.7031
Zymosterol	0.0004044	0.005531	3.057	0.137	2.69	0.1228	3.19	0.1162	3.23	0.07064
Palatinitol	0.001355	0.008708	3.165	0.1146	2.885	0.05559	3.445	0.6204	3.245	0.06352
Amrinone	0.04821	0.09433	3.181	0.1335	3.027	0.5071	3.126	0.1245	3.254	0.06182
Adenine	0.007828	0.02516	3.144	0.09517	3.059	0.1028	3.169	0.1499	3.342	0.1552
Glycyl Proline	0.001489	0.009242	3.523	0.1496	2.937	0.2067	3.398	0.1318	3.365	0.07087
2-Monostearin	0.02489	0.05818	3.511	0.1728	3.273	0.1257	3.387	0.03952	3.366	0.06104
Pseudo Uridine	0.02687	0.05972	2.596	0.2223	2.777	0.6829	2.639	0.6254	3.381	0.9375
2,5-Dihydroxy-pyrazine	0.0005032	0.005531	3.426	0.0686	3.199	0.09867	3.376	0.06994	3.476	0.02153
Udp-Glucuronic Acid	0.02427	0.05749	3.46	0.09883	3.384	0.1908	3.531	0.07346	3.557	0.07735
Alpha-Tocopherol	0.004064	0.01465	3.595	0.06366	3.445	0.03867	3.671	0.1697	3.572	0.08645
Octadecylglyc	0.003832	0.01465	3.623	0.06486	3.363	0.1267	3.619	0.04404	3.578	0.03867

erol										
Itaconic acid	0.003929	0.01465	3.109	0.2355	2.621	0.5469	3.51	0.1457	3.586	0.1791
Putrescine	0.000104	0.004638	3.534	0.0818	3.291	0.07633	3.822	0.2164	3.604	0.09661
P-Hydroxylphenyllactic Acid	0.00235	0.01058	3.581	0.09452	3.233	0.1134	3.537	0.1207	3.629	0.07535
4-Hydroxybutanoic acid	0.0006747	0.005531	3.583	0.0863	3.401	0.104	3.803	0.1513	3.658	0.1172
Inosine-5'-Monophosphate	0.02199	0.0535	3.677	0.09162	3.509	0.0982	3.699	0.06855	3.68	0.1046
Isovalerylglycine	0.008812	0.02783	3.642	0.1133	3.291	0.21	3.666	0.09967	3.699	0.104
Alpha-Aminoadipic Acid	0.005415	0.01911	3.652	0.09484	3.475	0.2035	3.698	0.1149	3.755	0.04882
Ornithine	0.001329	0.008708	3.61	0.1295	3.495	0.1201	3.761	0.06759	3.804	0.09229
N-Methylglutamic Acid	0.001826	0.009741	3.864	0.09461	3.509	0.1586	3.911	0.1194	3.833	0.05895
3-Hydroxybutyric acid	0.03634	0.07518	3.637	0.1525	3.595	0.2872	3.795	0.1847	3.847	0.116
Catechin	0.001793	0.009741	3.77	0.09943	3.588	0.155	3.933	0.07265	3.901	0.07599
beta-Alanine	0.002923	0.01224	3.859	0.123	3.688	0.09624	3.943	0.09871	3.945	0.07915
4-Aminobutyric Acid	0.01333	0.0381	3.802	0.1417	3.753	0.3264	4.107	0.6292	3.962	0.09591
5-Methoxytryptamine	0.03287	0.0688	4.022	0.02731	3.951	0.1612	4.053	0.08758	3.964	0.05887
Glycerol-Alpha-Phosphate	0.0005272	0.005531	3.852	0.1009	3.566	0.07899	3.906	0.09415	3.969	0.04138
L-Asparagine	0.0001044	0.004638	3.776	0.1752	3.171	0.3528	4.106	0.1732	4.179	0.08697
Fumaric acid	0.02154	0.05312	4.162	0.11	4.092	0.05933	4.189	0.03718	4.234	0.05359
Uric Acid	0.0003632	0.005531	4.163	0.1686	3.87	0.113	4.443	0.1178	4.352	0.1028
N-Carbamoyla	0.02246	0.05391	4.181	0.1899	4.315	0.7308	4.384	0.1338	4.455	0.1593

spartate										
Dehydroascorbic Acid	0.00184	0.009741	4.378	0.106	3.881	0.5376	4.356	0.09708	4.455	0.06532
L-Methionine	0.0003735	0.005531	4.324	0.1285	3.971	0.1967	4.531	0.1406	4.551	0.07802
Uracil	0.0213	0.05312	4.603	0.1268	4.46	0.1579	4.688	0.06888	4.657	0.09452
Malic acid	0.002184	0.01022	4.583	0.07184	4.556	0.04766	4.692	0.055	4.673	0.06897
L-Tyrosine	0.001178	0.008479	4.467	0.1376	4.282	0.1608	4.627	0.1415	4.689	0.06356
L-Phenylalanine	0.0002456	0.005531	4.56	0.1004	4.21	0.1627	4.755	0.1314	4.758	0.0764
L-Threonine	0.0004837	0.005531	4.551	0.1423	4.24	0.1885	4.739	0.1172	4.777	0.06768
Ethanolamine	0.001169	0.008479	4.738	0.1159	4.549	0.08085	4.856	0.06262	4.853	0.1116
Methyl-ornithine	0.002215	0.01022	4.755	0.1229	4.613	0.1653	4.916	0.1011	4.937	0.06021
O-Phosphoethanolamine	0.002189	0.01022	4.953	0.09345	4.742	0.05652	5.001	0.06697	5.001	0.1195
L-Proline	0.002083	0.01022	4.775	0.158	4.532	0.2306	4.98	0.1286	5.007	0.09575
Serine	0.0007067	0.005531	4.917	0.178	4.341	0.4223	5.141	0.1331	5.18	0.06459
L-Aspartic acid	0.0006209	0.005531	5.116	0.09875	4.962	0.1295	5.179	0.07985	5.265	0.08101
L-Isoleucine	0.000435	0.005531	5.061	0.1222	4.796	0.1815	5.269	0.1369	5.282	0.09074
L-Glutamic acid	0.003694	0.0146	5.132	0.1114	5.07	0.0599	5.232	0.106	5.301	0.0747
L-Alanine	0.0006988	0.005531	5.194	0.1362	5.004	0.1292	5.341	0.09524	5.405	0.04666
L-Lactic acid	0.0493	0.09542	5.514	0.1095	5.401	0.2754	5.627	0.09435	5.569	0.09651
1,2-Dihydroxycylohexane	0.01618	0.04347	5.089	0.02538	5.156	0.1284	5.06	0.02792	5.071	0.05401
Glucose	0.0006409	0.005531	5.177	0.1297	5.37	0.2395	4.906	0.1538	4.918	0.1149
Stearic Acid	0.04217	0.08434	4.926	0.02356	4.91	0.1163	4.867	0.05564	4.856	0.04459
L-Cysteine	0.003731	0.0146	4.009	0.08177	4.205	0.07311	4.027	0.06852	3.969	0.06903
Erythronic acid	0.02638	0.05962	3.993	0.1495	4.137	0.1644	3.963	0.08164	3.849	0.1279

Citric acid	0.01611	0.04347	3.674	0.1985	3.882	0.2754	3.444	0.2159	3.507	0.1778
Creatinine	0.001679	0.009741	3.465	0.05892	3.911	0.5271	3.484	0.05414	3.536	0.07908
Alpha-D-Glucose	0.0265	0.05962	3.702	0.3209	3.688	0.3052	3.428	0.2492	3.347	0.1044
1-Kestose	0.002854	0.01223	3.546	0.2088	3.656	0.2658	3.262	0.1836	3.214	0.09833
2-Hydroxypyrazinyl-2-Propenoic Acid	0.004069	0.01465	3.307	0.01635	3.395	0.1519	3.294	0.02037	3.291	0.02644
Melezitose	0.009449	0.02883	3.416	0.238	3.575	0.2565	3.167	0.2379	3.105	0.1173
Phosphenodiimidic amide	0.01047	0.03142	3.244	0.03075	3.364	0.1782	3.219	0.04336	3.291	0.09025
Maltotriose	0.0004503	0.005531	3.319	0.2538	3.714	0.1696	2.894	0.2399	2.995	0.1215
2-Oxo-propanoic acid	0.0001336	0.004638	3.14	0.04811	3.295	0.2283	2.988	0.05578	3.086	0.04866
6-Hydroxy-2-aminohexanoic acid	0.0001546	0.004638	3.056	0.06754	3.339	0.6976	2.895	0.06652	2.864	0.03874
5-Hydroxy-3-Indoleacetic Acid	0.0004063	0.005531	2.98	0.07102	3.328	0.3655	2.911	0.1632	2.799	0.05345
DL-Dopa	0.005814	0.01975	2.928	0.1328	3.174	0.08412	2.928	0.1122	2.909	0.06384
Methylphosphonic acid	0.02145	0.05312	2.942	0.02193	2.994	0.1555	2.907	0.02192	2.94	0.02754
Citraconic acid	0.02001	0.05146	2.428	0.1787	3.104	0.7691	2.773	0.1113	2.795	0.1588
Nonanoic Acid	0.0161	0.04347	2.258	0.1041	3.515	0.7212	2.165	0.1273	2.227	0.05566
Methoxyamine	0.001691	0.009741	2.531	0.05326	2.617	0.2019	2.465	0.1185	2.326	0.05927
Isohexonic Acid	0.0000371	0.004638	2.148	0.05044	3.48	0.972	2.093	0.0372	1.992	0.05309

Stigmasterol	0.01214	0.03543	2.354	0.6284	2.245	0.156	2.563	0.2692	2.197	0.06076
