# Supplementary material for: An Integrative Transcriptomic and Metabolomic Study Revealed That Melatonin Plays a Protective Role in Chronic Lung Inflammation by Reducing Necroptosis
Source: Front Immunol. 2021 May 4;12:668002. doi: 10.3389/fimmu.2021.668002 (PMC8129533; doi:10.3389/fimmu.2021.668002)
Supplement: Supplementary Figure 1 — Score plots of PLS-DA based on the metabolic profile of COPD. (A) The plot of PLS-DA scores showing almost complete separation of Mel (red circles), Luz (blue triangles), LPS (green rhombi), and Con (gray squares). The classification parameters were R2X (cum) = 0.65, R2Y (cum) = 0.871, and Q2 (cum) = 0.536. (B) Validation model of PLS-DA. The R2 and Q2 intercept values were 0.4356 and −0.4317, respectively, after 200 permutations. [file DataSheet_1.zip › Table S4.pdf]

Table S4-1. 2559 differentially expressed genes between LPS and Con group.

Gene_id	Gene name	Gene description	FC(lps/con)	Log2FC(lps/con)	Pvalue	Padjust	Significant	Regulate	con	lps	con_2	con_3	con_4	con_5	con_6	lps_1	lps_3	lps_4	lps_5	lps_6
ENSMUSG00000036469	1-Mar	membrane-associated ring finger (C3HC4) 1 [Source:MGI Symbol;Acc:MGI:1920175]	2.665505466	1.41440914	1.95E-38	1.29E-36	yes	up	8.142	17.726	7.62	9.85	8.39	7.17	7.68	19.38	17.09	16.82	17.21	18.13
ENSMUSG00000092203	1110038B12Rik	RIKEN cDNA 1110038B12 gene [Source:MGI Symbol;Acc:MGI:1916013]	2.094704643	1.066746836	9.08E-09	8.33E-08	yes	up	13.504	24.772	12.24	13.66	11.9	13.74	15.98	30.68	20.62	27.67	24.54	20.35
ENSMUSG00000087684	1200007C13Rik	RIKEN cDNA 1200007C13 gene [Source:MGI Symbol;Acc:MGI:1921369]	0.149289794	-2.743812558	2.46E-12	3.30E-11	yes	down	1.586	0.198	2.07	1.34	1.06	1.49	1.97	0.36	0.25	0.1	0.11	0.17
ENSMUSG00000112545	1300014J16Rik	RIKEN cDNA 1300014J16 gene [Source:MGI Symbol;Acc:MGI:1921410]	2.469674074	1.30432066	0.00139554	0.005275706	yes	up	0.562	1.156	0.62	0.64	0.58	0.54	0.43	1.27	0.74	1.68	1.08	1.01
ENSMUSG00000059631	1500035N22Rik	RIKEN cDNA 1500035N22 gene [Source:MGI Symbol;Acc:MGI:1917508]	0.058120902	-4.104799103	0.007037627	0.022190492	yes	down	0.454	0.016	0.2	0	0.84	0.44	0.79	0	0	0.08	0	0
ENSMUSG00000101585	1600010M07Rik	RIKEN cDNA 1600010M07 gene [Source:MGI Symbol;Acc:MGI:1917031]	2.224021969	1.153171039	4.31E-05	0.000222926	yes	up	4.282	8.202	4	3.74	3.77	5.33	4.57	7.81	8.65	6.18	8.09	10.28
ENSMUSG00000029182	1700001C02Rik	RIKEN cDNA 1700001C02 gene [Source:MGI Symbol;Acc:MGI:1922684]	0.462349321	-1.112944824	0.000200311	0.000913516	yes	down	11.012	4.326	12.31	10.49	6.69	10.34	15.23	4.61	4.69	5.94	2.19	4.2
ENSMUSG00000021534	1700001L19Rik	RIKEN cDNA 1700001L19 gene [Source:MGI Symbol;Acc:MGI:1916565]	0.496007556	-1.011565998	0.001087137	0.004223131	yes	down	7.488	3.208	9.63	7.4	5.18	6.61	8.62	4.56	2.19	4.68	1.42	3.19
ENSMUSG00000096573	1700009J07Rik	RIKEN cDNA 1700009J07 gene [Source:MGI Symbol;Acc:MGI:1914938]	4.342181511	2.118420034	0.00527197	0.017200564	yes	up	0.19	0.672	0	0.29	0.3	0.13	0.23	0.24	0.75	0.78	0.86	0.73
ENSMUSG00000031927	1700012B09Rik	RIKEN cDNA 1700012B09 gene [Source:MGI Symbol;Acc:MGI:1916575]	0.281190556	-1.830379953	0.001542275	0.00577033	yes	down	4.5	1.11	3.23	1.75	4.23	6.94	6.35	0.28	1.29	2.42	0.28	1.28
ENSMUSG00000027886	1700013F07Rik	RIKEN cDNA 1700013F07 gene [Source:MGI Symbol;Acc:MGI:1922754]	0.454711296	-1.136977252	0.00063011	0.002572328	yes	down	7.29	3.088	7.91	6.04	5.52	7.62	9.36	3.75	4.36	4.3	1.05	1.98
ENSMUSG00000032300	1700017B05Rik	RIKEN cDNA 1700017B05 gene [Source:MGI Symbol;Acc:MGI:1921461]	2.394626172	1.259800453	1.07E-24	3.66E-23	yes	up	8.41	15.908	7.1	11.83	9.47	6.93	6.72	16.11	17.86	14.97	11.37	19.23
ENSMUSG00000033053	1700028P14Rik	RIKEN cDNA 1700028P14 gene [Source:MGI Symbol;Acc:MGI:1914733]	0.47156883	-1.084459733	0.001759367	0.006495191	yes	down	6.096	2.426	4.41	9.24	4.01	6.28	6.54	1.94	1.96	4.48	1.77	1.98
ENSMUSG00000054822	1700041G16Rik	RIKEN cDNA 1700041G16 gene [Source:MGI Symbol;Acc:MGI:1920549]	0.317396762	-1.655640686	0.01160125	0.03410933	yes	down	0.532	0.22	0.58	0.71	0.35	0.73	0.29	0.19	0.09	0	0.55	0.27

ENSMUSG00000102060	1700061E17Rik	RIKEN cDNA 1700061E17 gene [Source:MGI Symbol;Acc:MGI:3642196]	0.217853022	-2.198572971	0.017945962	0.049482517	yes	down	4.818	0.89	6.3	3.57	0	5.01	9.21	0.75	2.02	0.24	0	1.44
ENSMUSG00000024209	1700061G19Rik	RIKEN cDNA 1700061G19 gene [Source:MGI Symbol;Acc:MGI:1925875]	2.640137035	1.400612814	0.015938066	0.044788037	yes	up	0.118	0.266	0.12	0.18	0.15	0.04	0.1	0.16	0.36	0.19	0.23	0.39
ENSMUSG00000090307	1700071M16Rik	RIKEN cDNA 1700071M16 gene [Source:MGI Symbol;Acc:MGI:1920754]	3.591292785	1.844503276	0.001919808	0.007017341	yes	up	0.134	0.416	0.11	0.16	0.03	0.3	0.07	0.57	0.33	0.49	0.2	0.49
ENSMUSG00000097080	1700086O06Rik	RIKEN cDNA 1700086O06 gene [Source:MGI Symbol;Acc:MGI:1920766]	0.475275288	-1.073164703	0.007317232	0.022957199	yes	down	2.266	0.998	2.07	3.27	2.26	2.37	1.36	0.97	0.56	1.17	1.14	1.15
ENSMUSG00000085407	1700095J03Rik	RIKEN cDNA 1700095J03 gene [Source:MGI Symbol;Acc:MGI:1921543]	0.267029275	-1.90493018	0.000219489	0.000995043	yes	down	0.768	0.172	0.85	0.48	0.64	1.11	0.76	0.16	0.18	0.3	0.16	0.06
ENSMUSG00000100094	1810008I18Rik	RIKEN cDNA 1810008I18 gene [Source:MGI Symbol;Acc:MGI:1920875]	0.463239018	-1.110171321	1.06E-05	6.13E-05	yes	down	2.662	1.186	2.74	2.47	2.58	1.94	3.58	0.82	1.19	1.8	0.66	1.46
ENSMUSG00000078607	1810010H24Rik	RIKEN cDNA 1810010H24 gene [Source:MGI Symbol;Acc:MGI:1916316]	0.472296038	-1.082236663	5.22E-16	9.75E-15	yes	down	47.484	19.416	50.86	38.1	46.47	61.46	40.53	22.25	18.99	19.75	19.57	16.52
ENSMUSG00000027713	1810062G17Rik	RIKEN cDNA 1810062G17 gene [Source:MGI Symbol;Acc:MGI:1919532]	0.188554948	-2.406943089	0.000392634	0.001682068	yes	down	2.014	0.322	3.84	1.83	1.45	0.65	2.3	0.08	0.14	0.87	0.16	0.36
ENSMUSG00000051606	2010001K21Rik	RIKEN cDNA 2010001K21 gene [Source:MGI Symbol;Acc:MGI:1917079]	0.476778497	-1.068608925	2.99E-06	1.92E-05	yes	down	5.44	1.576	4.84	8.85	5.95	3.05	4.51	1.64	2.28	2	1.05	0.91
ENSMUSG00000107761	2010008C14Rik	RIKEN cDNA 2010008C14 gene [Source:MGI Symbol;Acc:MGI:1917109]	20.18409341	4.335146883	0.003530333	0.012021883	yes	up	0.05	0.874	0	0	0.25	0	0	0	0.37	1.16	1.33	1.51
ENSMUSG00000091575	2010016I18Rik	RIKEN cDNA 2010016I18 gene [Source:MGI Symbol;Acc:MGI:1916456]	2.205711307	1.141243977	0.002202604	0.007921238	yes	up	2.476	4.498	3.01	1.69	1.43	4.9	1.35	5.94	6.51	4.26	1.55	4.23
ENSMUSG00000063018	2010204K13Rik	RIKEN cDNA 2010204K13 gene [Source:MGI Symbol;Acc:MGI:1922859]	0.356675415	-1.48731632	0.000246468	0.001107966	yes	down	7.094	2.052	5.85	11.03	4.02	9.2	5.37	1.25	4	1.84	1.75	1.42
ENSMUSG00000101268	2010310C07Rik	RIKEN cDNA 2010310C07 gene [Source:MGI Symbol;Acc:MGI:1919381]	0.111716313	-3.16208823	0.001102647	0.004272534	yes	down	1.646	0.176	2.38	1.68	0.34	2.86	0.97	0.33	0.02	0.14	0.31	0.08
ENSMUSG00000109036	2210406H18Rik	RIKEN cDNA 2210406H18 gene [Source:MGI Symbol;Acc:MGI:1917383]	4.980032969	2.316155293	5.47E-07	3.89E-06	yes	up	0.402	1.68	0.57	0.37	0.52	0.37	0.18	2.41	1.73	1.11	1.62	1.53
ENSMUSG00000097457	2310031A07Rik	RIKEN cDNA 2310031A07 gene [Source:MGI Symbol;Acc:MGI:1916871]	6.328741119	2.661918555	0.000206157	0.000937778	yes	up	0.222	1.12	0	0.65	0.36	0.1	0	1.11	1.68	1.08	0.73	1
ENSMUSG00000101655	2310040G24Rik	RIKEN cDNA 2310040G24 gene [Source:MGI Symbol;Acc:MGI:1916897]	2.8554871	1.513736867	0.000120015	0.000572554	yes	up	1.622	3.284	1.47	2.23	2.11	0.95	1.35	3.16	3.5	4.14	2.34	3.28

ENSMUSG00000038311	2410017I17Rik	RIKEN cDNA 2410017I17 gene [Source:MGI Symbol;Acc:MGI:1916967]	16.06208842	4.005587581	0.009802641	0.029514039	yes	up	1.312	13.044	0	0	3.94	0	2.62	0	0	13.88	30.28	21.06
ENSMUSG00000087013	2610027K06Rik	RIKEN cDNA 2610027K06 gene [Source:MGI Symbol;Acc:MGI:1917159]	0.429573268	-1.219023876	2.91E-08	2.50E-07	yes	down	7.096	2.46	6.74	6.97	8.32	6.61	6.84	2.55	2.85	2.77	2.44	1.69
ENSMUSG00000079671	2610203C22Rik	RIKEN cDNA 2610203C22 gene [Source:MGI Symbol;Acc:MGI:1919731]	0.469068688	-1.092128896	3.40E-05	0.000178714	yes	down	7.49	2.596	6.14	5.96	7.11	9.14	9.1	2.53	1.67	2.36	5.07	1.35
ENSMUSG00000107479	2610300M13Rik	RIKEN cDNA 2610300M13 gene [Source:MGI Symbol;Acc:MGI:1919734]	11.89466045	3.572242184	0.001604055	0.005975376	yes	up	0.032	0.578	0	0.04	0.12	0	0	0.07	0.34	0.36	1.14	0.98
ENSMUSG00000041789	2700046A07Rik	RIKEN cDNA 2700046A07 gene [Source:MGI Symbol;Acc:MGI:1919803]	3.416394934	1.772474759	1.92E-05	0.000106715	yes	up	1.368	2.814	1.25	3.46	0.77	1.22	0.14	1.93	3.41	3.02	3.5	2.21
ENSMUSG00000099338	2810030D12Rik	RIKEN cDNA 2810030D12 gene [Source:MGI Symbol;Acc:MGI:1925585]	0.479640548	-1.059974468	0.001739125	0.006429327	yes	down	4.62	1.924	5.48	4.32	3.76	6	3.54	2.36	1.06	2.42	1.23	2.55
ENSMUSG00000087484	2900089D17Rik	RIKEN cDNA 2900089D17 gene [Source:MGI Symbol;Acc:MGI:1914527]	2.356905915	1.236894169	0.002828409	0.009897582	yes	up	1.106	1.194	0.68	1.46	0.11	1.52	1.76	1.26	0.92	1.09	1.16	1.54
ENSMUSG00000074635	3110070M22Rik	RIKEN cDNA 3110070M22 gene [Source:MGI Symbol;Acc:MGI:1914554]	0.413620032	-1.273622038	0.015507683	0.043739305	yes	down	0.938	0.326	0.71	0.8	0.8	1.29	1.09	0.35	0.33	0.43	0.35	0.17
ENSMUSG00000033731	3300002A11Rik	RIKEN cDNA 3300002A11 gene [Source:MGI Symbol;Acc:MGI:1919073]	0.411933407	-1.279516963	4.22E-08	3.53E-07	yes	down	11.288	4.898	11.17	13.02	4.58	8.61	19.06	5.38	4.51	8.54	3.16	2.9
ENSMUSG00000102705	4632432E15Rik	RIKEN cDNA 4632432E15 gene [Source:MGI Symbol;Acc:MGI:1921296]	0.127036557	-2.97668438	0.00499115	0.016369136	yes	down	0.246	0.026	0.18	0.2	0.32	0.32	0.21	0	0	0	0	0.13
ENSMUSG00000097350	4732491K20Rik	RIKEN cDNA 4732491K20 gene [Source:MGI Symbol;Acc:MGI:3026975]	0.372079924	-1.426315545	0.001064291	0.004142651	yes	down	0.61	0.192	0.49	0.57	0.71	0.71	0.57	0.27	0.32	0.22	0.07	0.08
ENSMUSG00000032057	4833427G06Rik	RIKEN cDNA 4833427G06 gene [Source:MGI Symbol;Acc:MGI:1921013]	0.457456899	-1.128292274	5.02E-06	3.08E-05	yes	down	12.032	4.798	13.07	13.61	6.92	11.25	15.31	5.14	4.1	6.85	3.17	4.73
ENSMUSG00000087192	4930412L05Rik	RIKEN cDNA 4930412L05 gene [Source:MGI Symbol;Acc:MGI:1921191]	0.037947954	-4.719834068	0.010026705	0.030104013	yes	down	13.666	0	67.18	0	0.44	0.71	0	0	0	0	0	0
ENSMUSG00000085295	4930430E12Rik	RIKEN cDNA 4930430E12 gene [Source:MGI Symbol;Acc:MGI:1918889]	10.28978517	3.363140957	1.47E-32	7.79E-31	yes	up	0.69	6.254	0.7	0.36	1.21	0.91	0.27	7.37	5.56	4.64	6.04	7.66
ENSMUSG00000046958	4930432E11Rik	RIKEN cDNA 4930432E11 gene [Source:MGI Symbol;Acc:MGI:3045259]	11.27361233	3.494877958	0.003698784	0.012534879	yes	up	0.002	0.028	0.01	0	0	0	0	0.02	0.01	0.06	0.02	0.03
ENSMUSG00000086969	4930443O20Rik	RIKEN cDNA 4930443O20 gene [Source:MGI Symbol;Acc:MGI:1921937]	0.057834956	-4.11191446	0.000467168	0.001969462	yes	down	1.164	0.024	3.71	0.16	0.42	0.22	1.31	0	0.08	0	0	0.04

ENSMUSG00000090202	4930503B20Rik	RIKEN cDNA 4930503B20 gene [Source:MGI Symbol;Acc:MGI:1922264]	46.93462814	5.552580824	0.000272958	0.001214304	yes	up	0	0.356	0	0	0	0	0	0.78	0.2	0	0.22	0.58
ENSMUSG00000107622	4930512J16Rik	RIKEN cDNA 4930512J16 gene [Source:MGI Symbol;Acc:MGI:1922381]	8.387301759	3.068206762	0.000135462	0.000640553	yes	up	0.13	0.788	0.04	0	0.12	0	0.49	0.28	0.44	2.41	0.25	0.56
ENSMUSG00000005131	4930550C14Rik	RIKEN cDNA 4930550C14 gene [Source:MGI Symbol;Acc:MGI:1922561]	0.473597608	-1.078266301	0.001400828	0.005291115	yes	down	2.018	1.03	1.32	3.03	1.85	1.82	2.07	0.62	1.86	0.44	1.52	0.71
ENSMUSG00000079460	4933403O08Rik	RIKEN cDNA 4933403O08 gene [Source:MGI Symbol;Acc:MGI:1918280]	0.066225998	-3.916458521	0.000253895	0.001136822	yes	down	0.792	0.054	1.18	0.62	0.68	1.41	0.07	0	0.13	0	0.07	0.07
ENSMUSG00000110427	4933406B17Rik	RIKEN cDNA 4933406B17 gene [Source:MGI Symbol;Acc:MGI:1918290]	0.307766768	-1.700090635	2.98E-07	2.21E-06	yes	down	11.892	0.324	0.96	0.51	55.53	0.85	1.61	0.18	0.54	0.33	0.13	0.44
ENSMUSG00000097494	4933406C10Rik	RIKEN cDNA 4933406C10 gene [Source:MGI Symbol;Acc:MGI:1921326]	0.253897077	-1.977684307	0.000199138	0.000908746	yes	down	1.014	0.846	1.17	0.98	1.02	1.28	0.62	3.38	0.37	0.03	0.34	0.11
ENSMUSG00000103006	4933417C20Rik	RIKEN cDNA 4933417C20 gene [Source:MGI Symbol;Acc:MGI:1918378]	17.24055907	4.107734653	0.007263882	0.022803181	yes	up	0	0.094	0	0	0	0	0	0.09	0.09	0.11	0.06	0.12
ENSMUSG00000058046	4933430I17Rik	RIKEN cDNA 4933430I17 gene [Source:MGI Symbol;Acc:MGI:3045314]	6.199681314	2.632194058	1.44E-05	8.13E-05	yes	up	0.41	1.098	0.12	0	1.73	0.07	0.13	0.99	0.55	1.93	0.82	1.2
ENSMUSG00000112276	5033421B08Rik	RIKEN cDNA 5033421B08 gene [Source:MGI Symbol;Acc:MGI:1923240]	0.401347732	-1.317075351	1.15E-07	9.02E-07	yes	down	3.21	1.132	4.12	2.51	3.75	3.29	2.38	0.45	1.39	1.37	1.2	1.25
ENSMUSG00000097097	5033426O07Rik	RIKEN cDNA 5033426O07 gene [Source:MGI Symbol;Acc:MGI:1923251]	0.24562961	-2.025443613	0.01499099	0.042438473	yes	down	0.31	0.064	0.1	0.31	0.31	0.66	0.17	0.03	0.08	0	0.11	0.1
ENSMUSG00000025058	5430427O19Rik	RIKEN cDNA 5430427O19 gene [Source:MGI Symbol;Acc:MGI:1918648]	2.669271664	1.416446143	2.74E-09	2.66E-08	yes	up	1.514	3.276	1.99	1.65	1.7	1.31	0.92	4.12	3.91	2.82	2.57	2.96
ENSMUSG00000110018	5430437J10Rik	RIKEN cDNA 5430437J10 gene [Source:MGI Symbol;Acc:MGI:1918682]	2.781550664	1.475889383	0.014612096	0.041519524	yes	up	0.462	1.186	0.45	0.98	0	0.45	0.43	0.49	1.94	1.04	1.08	1.38
ENSMUSG00000054672	5830411N06Rik	RIKEN cDNA 5830411N06 gene [Source:MGI Symbol;Acc:MGI:2443685]	4.455238292	2.155502597	6.22E-07	4.40E-06	yes	up	0.42	1.328	0.87	0.12	0.25	0.61	0.25	1.94	0.82	1.46	0.52	1.9
ENSMUSG00000102895	5830415G21Rik	RIKEN cDNA 5830415G21 gene [Source:MGI Symbol;Acc:MGI:1923259]	124.0074527	6.954283017	1.51E-11	1.85E-10	yes	up	0	1.036	0	0	0	0	0	1.42	0.97	0.78	0.99	1.02
ENSMUSG00000106219	5830416I19Rik	RIKEN cDNA 5830416I19 gene [Source:MGI Symbol;Acc:MGI:1922007]	18.19257225	4.185277635	4.72E-10	4.96E-09	yes	up	0.04	0.578	0.03	0.03	0.03	0.11	0	0.48	0.7	0.54	0.42	0.75
ENSMUSG00000086236	5830418P13Rik	RIKEN cDNA 5830418P13 gene [Source:MGI Symbol;Acc:MGI:3604110]	16.23605527	4.021129251	0.000318032	0.001392554	yes	up	0.058	0.678	0	0.29	0	0	0	0.76	0.44	0.43	0.16	1.6

ENSMUSG00000097471	5830432E09Rik	RIKEN cDNA 5830432E09 gene [Source:MGI Symbol;Acc:MGI:1915015]	2.706044113	1.436185358	2.59E-34	1.47E-32	yes	up	4.202	9.854	4.39	4.61	4.25	4	3.76	8.39	10.48	10.4	9.62	10.38
ENSMUSG00000046463	5930403N24Rik	RIKEN cDNA 5930403N24 gene [Source:MGI Symbol;Acc:MGI:2444171]	0.44266814	-1.175702554	0.002565155	0.009084728	yes	down	1.566	0.474	1.58	1.39	1.79	2.17	0.9	0.83	0.35	0.21	0.62	0.36
ENSMUSG00000100301	6030407O03Rik	RIKEN cDNA 6030407O03 gene [Source:MGI Symbol;Acc:MGI:1924410]	0.061260187	-4.028906412	0.002385414	0.008517176	yes	down	0.39	0.02	0.88	0.45	0.13	0.44	0.05	0	0.05	0	0.05	0
ENSMUSG00000032680	6820408C15Rik	RIKEN cDNA 6820408C15 gene [Source:MGI Symbol;Acc:MGI:3045333]	0.481496693	-1.054402207	9.40E-05	0.000457957	yes	down	8.93	3.884	6.77	8.7	9.28	8.43	11.47	2.43	4.5	6.91	2.74	2.84
ENSMUSG00000102674	8030442B05Rik	RIKEN cDNA 8030442B05 gene [Source:MGI Symbol;Acc:MGI:1924793]	0.239389743	-2.062566755	2.83E-09	2.74E-08	yes	down	2.308	0.464	2.78	3.06	1.62	2.04	2.04	0.84	0.37	0.17	0.57	0.37
ENSMUSG00000085743	8430419K02Rik	RIKEN cDNA 8430419K02 gene [Source:MGI Symbol;Acc:MGI:1921768]	0.221662723	-2.173561925	0.017924069	0.049444656	yes	down	0.392	0.128	0.07	0.5	0.42	0.45	0.52	0	0.49	0	0.12	0.03
ENSMUSG00000115970	8430426J06Rik	RIKEN cDNA 8430426J06 gene [Source:MGI Symbol;Acc:MGI:1925352]	0.470717136	-1.087067721	0.00767894	0.023927277	yes	down	3.688	1.444	4.23	2.46	1.95	4.62	5.18	1.51	1.02	1.98	0.94	1.77
ENSMUSG00000086141	9030622O22Rik	RIKEN cDNA 9030622O22 gene [Source:MGI Symbol;Acc:MGI:1918820]	0.423145469	-1.240774376	2.85E-08	2.45E-07	yes	down	6.936	3.486	5.73	3.84	9.39	8.08	7.64	1.76	3.49	5.74	4.62	1.82
ENSMUSG00000054598	9130230L23Rik	RIKEN cDNA 9130230L23 gene [Source:MGI Symbol;Acc:MGI:3041166]	0.454094396	-1.138935861	0.000271678	0.001209113	yes	down	5.766	1.204	16.12	3.3	3.18	2.89	3.34	1.48	1.13	1.21	0.83	1.37
ENSMUSG00000111761	9230112J17Rik	RIKEN cDNA 9230112J17 gene [Source:MGI Symbol;Acc:MGI:1924999]	0.363859248	-1.458547615	0.008565736	0.026292648	yes	down	0.85	0.188	1.82	0.36	0.68	1.07	0.32	0.17	0.29	0.04	0.27	0.17
ENSMUSG00000091050	9330020H09Rik	RIKEN cDNA 9330020H09 gene [Source:MGI Symbol;Acc:MGI:3704457]	3.07521089	1.62068535	0.002561236	0.00907235	yes	up	0.174	0.456	0.1	0.2	0.16	0.22	0.19	0.45	0.43	0.22	0.8	0.38
ENSMUSG00000004360	9330159F19Rik	RIKEN cDNA 9330159F19 gene [Source:MGI Symbol;Acc:MGI:3036239]	0.185001221	-2.434393301	6.80E-38	4.38E-36	yes	down	3.33	0.45	2.78	3.92	3.53	3.61	2.81	0.32	0.57	0.34	0.69	0.33
ENSMUSG00000097177	9330159M07Rik	RIKEN cDNA 9330159M07 gene [Source:MGI Symbol;Acc:MGI:2442519]	0.457681858	-1.127582988	0.00649743	0.020697449	yes	down	3.846	1.222	2.93	3.58	7.72	3.46	1.54	1.21	0.57	0.44	3.05	0.84
ENSMUSG00000097194	9330175E14Rik	RIKEN cDNA 9330175E14 gene [Source:MGI Symbol;Acc:MGI:2443913]	3.279062354	1.713283336	1.17E-05	6.73E-05	yes	up	0.51	1.072	0.56	0.36	0.75	0.44	0.44	1.77	0.9	1.19	0.54	0.96
ENSMUSG00000101799	9330175M20Rik	RIKEN cDNA 9330175M20 gene [Source:MGI Symbol;Acc:MGI:3045285]	0.463447659	-1.109521682	0.000314932	0.001380955	yes	down	2.352	0.916	3.37	1.01	2.48	3.07	1.83	1.07	0.89	0.66	1.33	0.63
ENSMUSG00000108402	9430064I24Rik	RIKEN cDNA 9430064I24 gene [Source:MGI Symbol;Acc:MGI:3704302]	2.473763799	1.306707755	0.007593805	0.023702152	yes	up	0.23	0.486	0.2	0.07	0.29	0.31	0.28	0.62	0.76	0.3	0.22	0.53

ENSMUSG00000085982	9530051G07Rik	RIKEN cDNA 9530051G07 gene [Source:MGI Symbol;Acc:MGI:2442715]	0.288615388	-1.792779876	0.01083942	0.032123005	yes	down	0.212	0.05	0.11	0.09	0.41	0.24	0.21	0.08	0	0.04	0.1	0.03
ENSMUSG00000045065	9930022D16Rik	RIKEN cDNA 9930022D16 gene [Source:MGI Symbol;Acc:MGI:2444178]	16.4247218	4.03779703	0.004011769	0.013482535	yes	up	0.01	0.132	0	0.03	0	0	0.02	0	0.3	0.15	0.04	0.17
ENSMUSG00000027596	a	nonagouti [Source:MGI Symbol;Acc:MGI:87853]	0.264696106	-1.917591123	1.53E-22	4.56E-21	yes	down	9.39	3.044	9.22	11.93	7.87	6.99	10.94	2.43	3.39	3.32	2.16	3.92
ENSMUSG00000112095	A130077B15Rik	RIKEN cDNA A130077B15 gene [Source:MGI Symbol;Acc:MGI:2441782]	2.331246368	1.221101478	3.50E-06	2.22E-05	yes	up	0.776	1.518	0.84	0.61	0.96	0.89	0.58	1.69	1.52	1.38	1.94	1.06
ENSMUSG00000096960	A230028O05Rik	RIKEN cDNA A230028O05 gene [Source:MGI Symbol;Acc:MGI:2442126]	13.12467457	3.714209746	0.004767879	0.015730842	yes	up	0.008	0.102	0	0	0	0	0.04	0.12	0.11	0.14	0.1	0.04
ENSMUSG00000030111	A2m	alpha-2-macroglobulin [Source:MGI Symbol;Acc:MGI:2449119]	13.85721771	3.792565714	1.18E-07	9.28E-07	yes	up	0.05	0.586	0.01	0.13	0.04	0.01	0.06	0.16	0.29	1.67	0.19	0.62
ENSMUSG00000096929	A330023F24Rik	RIKEN cDNA A330023F24 gene [Source:MGI Symbol;Acc:MGI:2443958]	0.350113414	-1.514105757	0.000940191	0.003710176	yes	down	0.828	0.244	1.15	0.22	0.71	1.16	0.9	0.27	0.32	0.41	0.12	0.1
ENSMUSG00000086213	A330040F15Rik	RIKEN cDNA A330040F15 gene [Source:MGI Symbol;Acc:MGI:3698434]	5.086239917	2.346599515	0.00943311	0.028578227	yes	up	0.092	0.392	0	0.04	0.11	0.31	0	0.36	0.57	0.25	0.24	0.54
ENSMUSG00000109321	A330076H08Rik	RIKEN cDNA A330076H08 gene [Source:MGI Symbol;Acc:MGI:2443193]	0.398234173	-1.328311068	0.007200304	0.022633451	yes	down	5.448	0.886	13.79	1.12	6.41	3.9	2.02	2.39	0.52	0.67	0.4	0.45
ENSMUSG00000086851	A430108G06Rik	RIKEN cDNA A430108G06 gene [Source:MGI Symbol;Acc:MGI:2442478]	0.489099541	-1.031799984	0.017017022	0.047354021	yes	down	1.476	0.596	1.8	1.46	1.52	1.29	1.31	1.07	0.34	0.87	0.32	0.38
ENSMUSG00000006462	A530013C23Rik	RIKEN cDNA A530013C23 gene [Source:MGI Symbol;Acc:MGI:3041178]	0.398692994	-1.326649841	0.000109344	0.000525635	yes	down	1.622	0.544	2.32	0.72	1.46	1.57	2.04	0.5	0.61	0.67	0.28	0.66
ENSMUSG00000105160	A530030E21Rik	RIKEN cDNA A530030E21 gene [Source:MGI Symbol;Acc:MGI:2444617]	3.369003388	1.752321879	0.000511102	0.00213534	yes	up	0.76	2.122	0.75	1.07	0.83	1.05	0.1	4.43	1.35	1.82	1.74	1.27
ENSMUSG00000086395	A630014C17Rik	RIKEN cDNA A630014C17 gene [Source:MGI Symbol;Acc:MGI:3026989]	0.428608265	-1.222268425	0.004626092	0.015317315	yes	down	0.552	0.202	0.31	0.52	0.38	0.7	0.85	0.2	0.29	0.2	0.15	0.17
ENSMUSG00000097924	A730020E08Rik	RIKEN cDNA A730020E08 gene [Source:MGI Symbol;Acc:MGI:2444999]	0.427052073	-1.227516098	0.000115361	0.000551588	yes	down	1.514	0.364	0.89	2.4	1.39	1.04	1.85	0.4	0.35	0.51	0.35	0.21
ENSMUSG00000045238	A730035I17Rik	RIKEN cDNA A730035I17 gene [Source:MGI Symbol;Acc:MGI:3696858]	0.158864252	-2.654133574	0.010207438	0.03055241	yes	down	0.308	0.034	0.46	0.27	0.2	0.35	0.26	0.07	0.07	0.03	0	0
ENSMUSG00000085139	A730046J19Rik	RIKEN cDNA A730046J19 gene [Source:MGI Symbol;Acc:MGI:2442684]	0.255238233	-1.970083642	3.15E-08	2.68E-07	yes	down	0.626	0.136	0.59	0.53	0.53	0.68	0.8	0.11	0.17	0.18	0.17	0.05
ENSMUSG00000048636	A730049H05Rik	RIKEN cDNA A730049H05 gene [Source:MGI	0.135630379	-2.882247736	1.91E-05	0.000105793	yes	down	0.686	0.078	0.66	0.58	0.71	0.29	1.19	0.11	0.18	0	0.08	0.02

		Symbol;Acc:MGI:1921766]																		
ENSMUSG00000057715	A830018L16Rik	RIKEN cDNA A830018L16 gene [Source:MGI Symbol;Acc:MGI:2444149]	0.478087629	-1.064653021	0.000254654	0.001139744	yes	down	0.874	0.438	0.97	0.95	0.95	0.74	0.76	0.79	0.15	0.53	0.26	0.46
ENSMUSG00000097855	A930007I19Rik	RIKEN cDNA A930007I19 gene [Source:MGI Symbol;Acc:MGI:1925029]	4.418569631	2.143579419	4.37E-21	1.19E-19	yes	up	2.066	11.47	2.38	1.71	2.92	2.25	1.07	7.88	9.08	6.35	28.12	5.92
ENSMUSG00000054181	A930012O16Rik	RIKEN cDNA A930012O16 gene [Source:MGI Symbol;Acc:MGI:2442255]	0.353109453	-1.501812651	0.008053312	0.024952218	yes	down	0.746	0.224	1.13	0.5	0.63	0.68	0.79	0.2	0.34	0.05	0.35	0.18
ENSMUSG00000113342	AA414992	expressed sequence AA414992 [Source:MGI Symbol;Acc:MGI:2142939]	0.324802403	-1.62236579	2.29E-07	1.73E-06	yes	down	2.932	0.808	3.37	4.88	1.64	1.4	3.37	0.74	0.84	0.74	0.65	1.07
ENSMUSG00000033213	AA467197	expressed sequence AA467197 [Source:MGI Symbol;Acc:MGI:3034182]	15.19995948	3.925995573	1.63E-28	7.03E-27	yes	up	5.386	69.058	4.51	11.03	4.96	3	3.43	55.56	77.16	51.9	72.33	88.34
ENSMUSG00000027761	Aadac	arylacetamide deacetylase [Source:MGI Symbol;Acc:MGI:1915008]	6.304043742	2.656277545	0.004490007	0.014908216	yes	up	0.09	0.984	0	0.35	0	0.05	0.05	0.38	0.49	2.78	0.38	0.89
ENSMUSG00000068522	Aard	alanine and arginine rich domain containing protein [Source:MGI Symbol;Acc:MGI:2181621]	0.415763514	-1.266164938	1.70E-12	2.31E-11	yes	down	13.74	4.83	17.56	13.57	10.65	15.16	11.76	4.97	5.79	4.32	5.53	3.54
ENSMUSG00000029695	Aass	aminoadipate-semialdehyde synthase [Source:MGI Symbol;Acc:MGI:1353573]	0.36836758	-1.440782	6.86E-19	1.63E-17	yes	down	5.198	1.724	5.68	3.81	5.5	6.53	4.47	1.59	1.5	1.51	1.96	2.06
ENSMUSG00000057191	AB124611	cDNA sequence AB124611 [Source:MGI Symbol;Acc:MGI:3043001]	2.210928234	1.144652197	9.09E-17	1.86E-15	yes	up	10.026	18.69	12.04	10.38	9.52	11.03	7.16	19.19	17.21	18.67	17.65	20.73
ENSMUSG00000015243	Abca1	ATP-binding cassette, sub-family A (ABC1), member 1 [Source:MGI Symbol;Acc:MGI:99607]	2.230025965	1.157060508	9.87E-25	3.37E-23	yes	up	16.768	31.25	19.29	12.32	18.35	18.75	15.13	35.13	28.16	31.79	30.17	31
ENSMUSG00000062017	Abca14	ATP-binding cassette, sub-family A (ABC1), member 14 [Source:MGI Symbol;Acc:MGI:2388708]	0.199643454	-2.324502324	0.00157586	0.005878527	yes	down	0.662	0.048	0.17	0.58	1.09	1	0.47	0.05	0	0.07	0.08	0.04
ENSMUSG00000018800	Abca5	ATP-binding cassette, sub-family A (ABC1), member 5 [Source:MGI Symbol;Acc:MGI:2386607]	0.477797028	-1.065530215	2.14E-26	8.07E-25	yes	down	13.364	5.394	14.15	12.96	12.7	15.07	11.94	5.63	5.91	4.4	6.28	4.75
ENSMUSG00000024030	Abcg1	ATP binding cassette subfamily G member 1 [Source:MGI Symbol;Acc:MGI:107704]	3.383245391	1.758407822	1.10E-41	8.09E-40	yes	up	23.042	64.73	28.04	19.41	25.79	17.45	24.52	50.14	74.39	64.08	66.71	68.33
ENSMUSG00000032046	Abhd12	abhydrolase domain containing 12 [Source:MGI Symbol;Acc:MGI:1923442]	2.869655641	1.520877623	1.54E-50	1.52E-48	yes	up	43.84	107.108	38.77	45.44	49.25	38.75	46.99	88.09	118.37	99.98	112.87	116.23
ENSMUSG00000035258	Abi3bp	ABI gene family, member 3 (NESH) binding protein [Source:MGI Symbol;Acc:MGI:2444583]	0.446547559	-1.163114258	1.02E-23	3.27E-22	yes	down	31.566	12.572	34.48	29.6	29.46	34.64	29.65	10.56	13.68	15.14	13.7	9.78
ENSMUSG00000042895	Abra	actin-binding Rho activating protein [Source:MGI Symbol;Acc:MGI:2444891]	0.334957383	-1.577950541	1.25E-05	7.14E-05	yes	down	1.18	0.336	0.86	1.71	0.74	0.95	1.64	0.31	0.27	0.51	0.35	0.24

ENSMUSG00000117538	AC096777.1	TEC	0.093058995	-3.425710582	0.000306661	0.001348551	yes	down	0.554	0.044	0.54	0.28	0.83	0.52	0.6	0.09	0	0	0	0.13
ENSMUSG00000117905	AC109138.8	novel protein	2.115691432	1.08112923	4.56E-06	2.82E-05	yes	up	3.796	6.888	3.38	3.25	4.74	5.08	2.53	9.5	5.65	7.19	3.52	8.58
ENSMUSG00000117964	AC119228.1	novel transcript, antisense to Cd274	3.162198036	1.660927721	3.80E-06	2.39E-05	yes	up	1.24	2.068	0.24	1.79	0.46	1.74	1.97	1.99	2.87	3.01	0.54	1.93
ENSMUSG00000118232	AC122252.3	novel transcript	0.116674953	-3.099433205	1.19E-06	8.05E-06	yes	down	13.036	0.062	12.44	0.3	0.53	1.99	49.92	0.05	0.07	0.07	0.12	0
ENSMUSG00000118219	AC124108.1	novel protein	5.424716536	2.439547753	0.017218429	0.047834366	yes	up	0.024	0.11	0	0.05	0	0.07	0	0.07	0.14	0.15	0.01	0.18
ENSMUSG00000117662	AC125110.1	novel transcript	0.486791971	-1.038622722	0.014912517	0.042244241	yes	down	12.982	5.206	10.22	17.13	17.06	15.33	5.17	4	6.18	5.63	7.34	2.88
ENSMUSG00000079800	AC125149.3		7.354240725	2.878576401	1.65E-07	1.27E-06	yes	up	0.738	4.788	0.34	0.61	1.84	0.73	0.17	9.13	5	3.99	2.4	3.42
ENSMUSG00000117028	AC126937.1	novel transcript, antisense to Def6	4.973154835	2.314161349	0.009420764	0.02854486	yes	up	0.14	0.55	0.4	0.08	0.08	0.14	0	0.67	0.61	0.55	0.8	0.12
ENSMUSG00000118330	AC132148.1	novel transcript, antisense to Dagla	20.75825028	4.375612938	0.000183941	0.00084534	yes	up	0.018	0.286	0	0	0.09	0	0	0.54	0.11	0.15	0.12	0.51
ENSMUSG00000117263	AC132460.3	novel transcript, antisense to Anks1	0.235184269	-2.08813653	0.00709337	0.022333374	yes	down	0.568	0.112	0.7	0.48	0.3	0.71	0.65	0.29	0.09	0.13	0	0.05
ENSMUSG00000079190	AC133103.1	sp110 nuclear body protein-like [Source:NCBI gene;Acc:100041057]	6.034501061	2.593234492	0.001202001	0.004614925	yes	up	0.098	0.576	0	0.15	0.16	0.04	0.14	0.66	0.17	0.26	0.69	1.1
ENSMUSG00000117399	AC134555.1	novel transcript	20.70636332	4.372002289	0.002876063	0.010049562	yes	up	0	0.132	0	0	0	0	0	0.16	0.11	0.16	0.16	0.07
ENSMUSG00000117980	AC138228.3	novel transcript	0.280976183	-1.83148025	3.95E-07	2.87E-06	yes	down	23.066	4.742	22.55	22.47	15	29.83	25.48	6	5.24	6.47	2.95	3.05
ENSMUSG00000117234	AC154707.1	novel C2H2 zinc finger pseudogene	2.952152446	1.561767223	0.016048413	0.045068557	yes	up	0.178	0.456	0.08	0.25	0.08	0.1	0.38	0.44	0.51	0.34	0.2	0.79
ENSMUSG00000117764	AC157784.1	novel transcript, sense intronic to Ccbe1	16.83166916	4.073106347	2.79E-18	6.31E-17	yes	up	0.27	3.022	0.41	0.53	0.04	0.3	0.07	4	2.56	2.44	3.72	2.39
ENSMUSG00000117604	AC161438.1	novel transcript	0.284984569	-1.811044292	5.28E-13	7.51E-12	yes	down	52.662	12.842	50.23	77.8	35.35	30.89	69.04	10.88	15.05	14.23	12.67	11.38
ENSMUSG00000117575	AC163635.1	novel transcript	3.153218239	1.656825025	0.000195537	0.000894409	yes	up	0.158	1.866	0.22	0.12	0.15	0.19	0.11	1.38	0.27	0.54	0.21	6.93
ENSMUSG00000117239	AC166110.3	G protein-coupled receptor 31, D17Leh66b region (Gpr31b) pseudogene	6.824078961	2.77063434	2.86E-12	3.81E-11	yes	up	0.12	0.71	0.07	0.12	0.11	0.14	0.16	0.88	0.38	0.77	0.53	0.99
ENSMUSG00000079808	AC168977.1	sp110 nuclear body protein-like [Source:NCBI gene;Acc:102638047]	4.904145405	2.294001755	1.25E-07	9.78E-07	yes	up	7.142	27.966	8.84	8.18	10.64	4.33	3.72	62.41	20.2	21.43	10.92	24.87
ENSMUSG00000117063	AC174800.1	novel transcript	0.344946811	-1.535554173	7.45E-05	0.000370024	yes	down	5.786	0.704	0.96	6.19	14.17	3.84	3.77	0.32	0.57	0.3	0.26	2.07
ENSMUSG00000010651	Acaa1b	acetyl-Coenzyme A acyltransferase 1B [Source:MGI Symbol;Acc:MGI:3605455]	0.275973784	-1.85739687	2.50E-07	1.87E-06	yes	down	4.232	1.084	3.99	3.13	4.83	2.47	6.74	0.68	1.57	1.57	1.12	0.48
ENSMUSG00000038007	Acer2	alkaline ceramidase 2 [Source:MGI Symbol;Acc:MGI:1920932]	0.479753147	-1.059635826	3.13E-18	7.04E-17	yes	down	184.118	73.198	207.49	169.67	160.59	207.22	175.62	70.84	79.71	63.77	94.08	57.59
ENSMUSG00000037872	Ackr1	atypical chemokine receptor 1 (Duffy blood group) [Source:MGI Symbol;Acc:MGI:1097689]	3.790101081	1.922236325	2.41E-05	0.000131307	yes	up	0.79	2.498	0.96	1.48	0.92	0.47	0.12	2.89	2.57	1.52	2.98	2.53
ENSMUSG00000022126	Acod1	aconitate decarboxylase 1 [Source:MGI Symbol;Acc:MGI:103206]	20.91386342	4.38638769	9.53E-29	4.19E-27	yes	up	0.246	4.322	0.25	0.41	0.16	0.26	0.15	4.09	2.92	3.08	2.44	9.08
ENSMUSG00000072949	Acot1	acyl-CoA thioesterase 1 [Source:MGI Symbol;Acc:MGI:1349396]	0.37986212	-1.396452242	7.08E-06	4.24E-05	yes	down	61.828	16.982	91.99	42.26	65.5	40.98	68.41	14.04	19.99	15.07	26.06	9.75
ENSMUSG00000029098	Acox3	acyl-Coenzyme A oxidase 3, pristanoyl [Source:MGI Symbol;Acc:MGI:1933156]	2.01991926	1.014297627	3.04E-23	9.53E-22	yes	up	18.236	30.704	16.57	20.82	17.14	19.07	17.58	28.38	37.56	26	28.97	32.61
ENSMUSG00000027380	Acoxl	acyl-Coenzyme A oxidase-like [Source:MGI Symbol;Acc:MGI:1921371]	0.356372369	-1.488542611	1.91E-08	1.68E-07	yes	down	49.946	11.358	55.49	58.28	44.36	49.18	42.42	6.4	13.28	14.38	13.67	9.06

ENSMUSG00000001348	Acp5	acid phosphatase 5, tartrate resistant [Source:MGI Symbol;Acc:MGI:87883]	11.40202854	3.511218613	5.50E-51	5.51E-49	yes	up	23.406	219.14	18.02	28.93	35.11	15.86	19.11	209.42	229.35	196.2	219.77	240.96
ENSMUSG00000032561	Acpp	acid phosphatase, prostate [Source:MGI Symbol;Acc:MGI:1928480]	4.718661153	2.238377575	1.28E-55	1.46E-53	yes	up	2.78	11.124	3.37	3.2	1.96	2.63	2.74	11.91	10.94	8.94	11.47	12.36
ENSMUSG00000022622	Acr	acrosin prepropeptide [Source:MGI Symbol;Acc:MGI:87884]	0.090155135	-3.47144652	0.008226482	0.02541156	yes	down	0.318	0.026	0.97	0.15	0.06	0.24	0.17	0	0	0	0.09	0.04
ENSMUSG00000032281	Acsbg1	acyl-CoA synthetase bubblegum family member 1 [Source:MGI Symbol;Acc:MGI:2385656]	2.37439237	1.247558361	4.25E-09	4.05E-08	yes	up	0.972	1.948	1.05	1.14	0.7	1.18	0.79	1.78	2.26	2.06	1.63	2.01
ENSMUSG00000033533	Acsm1	acyl-CoA synthetase medium-chain family member 1 [Source:MGI Symbol;Acc:MGI:2152200]	0.324676732	-1.622924097	2.15E-07	1.63E-06	yes	down	8.288	2.066	8.15	9.77	3.51	10.51	9.5	1.98	2.44	3.26	0.73	1.92
ENSMUSG00000030935	Acsm3	acyl-CoA synthetase medium-chain family member 3 [Source:MGI Symbol;Acc:MGI:99538]	0.107338478	-3.219760757	4.49E-07	3.25E-06	yes	down	2.214	0.168	0.5	2.06	6.59	0.57	1.35	0.37	0.02	0.24	0.08	0.13
ENSMUSG00000030972	Acsm5	acyl-CoA synthetase medium-chain family member 5 [Source:MGI Symbol;Acc:MGI:2444086]	0.163936565	-2.608790417	0.003847585	0.012982491	yes	down	0.39	0.054	0.22	0.66	0.34	0.19	0.54	0.11	0	0.16	0	0
ENSMUSG00000035783	Acta2	actin, alpha 2, smooth muscle, aorta [Source:MGI Symbol;Acc:MGI:87909]	0.42400202	-1.237856958	1.31E-06	8.80E-06	yes	down	154.228	55.204	142.39	230.74	88.58	157.15	152.28	45.31	61.19	67.11	39.61	62.8
ENSMUSG00000059430	Actg2	actin, gamma 2, smooth muscle, enteric [Source:MGI Symbol;Acc:MGI:104589]	0.325663964	-1.618544008	4.83E-05	0.00024728	yes	down	28.584	8.328	22.78	47.48	15.93	27.72	29.01	4.76	8.93	14.5	5.34	8.11
ENSMUSG00000008438	Adam21	a disintegrin and metallopeptidase domain 21 [Source:MGI Symbol;Acc:MGI:1861229]	0.07834693	-3.67397945	0.010771712	0.031961023	yes	down	0.074	0.004	0.09	0.09	0.11	0.04	0.04	0	0	0	0	0.02
ENSMUSG00000031553	Adam3	a disintegrin and metallopeptidase domain 3 (cyritestin) [Source:MGI Symbol;Acc:MGI:102518]	0.255955135	-1.966037147	1.50E-09	1.50E-08	yes	down	4.65	1.15	4.18	5.88	4.33	5.34	3.52	1.58	1.81	0.32	1.54	0.5
ENSMUSG00000025473	Adam8	a disintegrin and metallopeptidase domain 8 [Source:MGI Symbol;Acc:MGI:107825]	3.730043789	1.899192567	7.00E-10	7.22E-09	yes	up	11.052	24.902	7.69	17.82	5.63	16.45	7.67	34.25	21.08	29.09	16.6	23.49
ENSMUSG00000033453	Adamts15	a disintegrin-like and metallopeptidase (reprolysin type) with thrombospondin type 1 motif, 15 [Source:MGI Symbol;Acc:MGI:2449569]	0.344864199	-1.535899729	2.04E-27	8.14E-26	yes	down	30.764	8.986	33.77	33.2	21.97	32.82	32.06	7.05	10.66	11.29	7.34	8.59
ENSMUSG00000022894	Adamts5	a disintegrin-like and metallopeptidase (reprolysin type) with thrombospondin type 1 motif, 5 (aggrecanase-2) [Source:MGI Symbol;Acc:MGI:1346321]	0.426473905	-1.229470625	1.92E-21	5.30E-20	yes	down	3.244	1.17	3.15	3.32	2.98	3.56	3.21	1.15	1.27	1.49	0.95	0.99
ENSMUSG00000030022	Adamts9	a disintegrin-like and metallopeptidase (reprolysin type) with thrombospondin type 1 motif, 9 [Source:MGI Symbol;Acc:MGI:1916320]	0.435548133	-1.199095932	2.30E-17	4.87E-16	yes	down	15.966	5.878	15.88	18.15	12.86	21.17	11.77	5.75	5.69	4.46	6.99	6.5
ENSMUSG00000043822	Adamtsl5	ADAMTS-like 5 [Source:MGI Symbol;Acc:MGI:1913798]	0.473310552	-1.07914101	5.51E-11	6.36E-10	yes	down	20.326	9.072	26.07	21.68	10.83	24.24	18.81	3.57	5.85	17.8	10.35	7.79

ENSMUSG00000056413	Adap1	ArfGAP with dual PH domains 1 [Source:MGI Symbol;Acc:MGI:2442201]	4.23722758	2.083120618	4.75E-53	5.11E-51	yes	up	3.856	13.068	3.49	4.63	4.52	3.24	3.4	11.7	12.47	12.02	12.76	16.39
ENSMUSG00000020431	Adcy1	adenylate cyclase 1 [Source:MGI Symbol;Acc:MGI:99677]	0.319785324	-1.644824365	9.90E-08	7.86E-07	yes	down	0.312	0.088	0.38	0.32	0.33	0.32	0.21	0.07	0.06	0.13	0.1	0.08
ENSMUSG00000020654	Adcy3	adenylate cyclase 3 [Source:MGI Symbol;Acc:MGI:99675]	2.251158463	1.170667614	5.76E-20	1.46E-18	yes	up	8.374	18.446	8.62	8.3	8.9	7.94	8.11	12.01	21.18	19.56	17.88	21.6
ENSMUSG00000031659	Adcy7	adenylate cyclase 7 [Source:MGI Symbol;Acc:MGI:102891]	2.007191387	1.005178185	5.64E-37	3.50E-35	yes	up	20.03	33.682	18.51	20.25	21.2	20.39	19.8	29.11	36.84	34.23	30.28	37.95
ENSMUSG00000004730	Adgre1	adhesion G protein-coupled receptor E1 [Source:MGI Symbol;Acc:MGI:106912]	6.429743953	2.684761287	6.65E-73	1.33E-70	yes	up	17.132	94.536	18.85	20.1	18.52	9.86	18.33	88.14	86.36	82.3	103.97	111.91
ENSMUSG00000061577	Adgrg5	adhesion G protein-coupled receptor G5 [Source:MGI Symbol;Acc:MGI:2685955]	2.353212765	1.234631767	1.81E-07	1.38E-06	yes	up	1.15	2.214	1.2	1.04	1.2	1.47	0.84	2.18	2.88	1.92	1.78	2.31
ENSMUSG00000037605	Adgrl3	adhesion G protein-coupled receptor L3 [Source:MGI Symbol;Acc:MGI:2441950]	0.492640857	-1.021391812	5.16E-25	1.79E-23	yes	down	26.948	8.866	23.11	37.46	22.55	26.03	25.59	9.15	9.28	8.46	8.46	8.98
ENSMUSG00000069170	Adgrv1	adhesion G protein-coupled receptor V1 [Source:MGI Symbol;Acc:MGI:1274784]	0.369017746	-1.438237897	0.000259248	0.001158366	yes	down	1.416	0.498	1.6	0.48	1.19	2.65	1.16	0.32	0.28	0.84	0.47	0.58
ENSMUSG00000030790	Adm	adrenomedullin [Source:MGI Symbol;Acc:MGI:108058]	0.316918824	-1.657814743	3.27E-10	3.50E-09	yes	down	14.38	3.814	13.9	19.65	10.35	15.6	12.4	5.94	5.03	3.54	1.83	2.73
ENSMUSG00000042429	Adora1	adenosine A1 receptor [Source:MGI Symbol;Acc:MGI:99401]	2.095168452	1.067066242	1.50E-06	1.01E-05	yes	up	1.442	2.714	1.19	1.6	2.16	1	1.26	1.83	3.17	3.13	2.04	3.4
ENSMUSG00000018500	Adora2b	adenosine A2b receptor [Source:MGI Symbol;Acc:MGI:99403]	2.452975812	1.294533008	3.28E-12	4.33E-11	yes	up	6.814	14.222	7.15	7.67	6.74	3.91	8.6	11.64	17.31	14.55	13.59	14.02
ENSMUSG00000000562	Adora3	adenosine A3 receptor [Source:MGI Symbol;Acc:MGI:104847]	4.644775421	2.215608843	6.41E-12	8.23E-11	yes	up	0.558	2.204	0.37	0.96	0.75	0.35	0.36	2.91	2.18	1.24	2.05	2.64
ENSMUSG00000045875	Adra1a	adrenergic receptor, alpha 1a [Source:MGI Symbol;Acc:MGI:104773]	0.311030293	-1.684872994	2.44E-08	2.11E-07	yes	down	9.09	2.516	9.13	6.06	8.79	13.8	7.67	2.98	3.96	2.37	2.01	1.26
ENSMUSG00000050541	Adra1b	adrenergic receptor, alpha 1b [Source:MGI Symbol;Acc:MGI:104774]	0.299913892	-1.737379745	0.001168397	0.004495577	yes	down	1.1	0.228	0.81	1.78	0.33	1.14	1.44	0.31	0.31	0.36	0.07	0.09
ENSMUSG00000031489	Adrb3	adrenergic receptor, beta 3 [Source:MGI Symbol;Acc:MGI:87939]	0.337637754	-1.56645186	2.50E-08	2.16E-07	yes	down	4.314	1.292	3.32	3.3	6.3	4.28	4.37	1.69	1.35	1.87	1.01	0.54
ENSMUSG00000054932	Afp	alpha fetoprotein [Source:MGI Symbol;Acc:MGI:87951]	2.676495354	1.420345148	0.010184231	0.030487209	yes	up	0.304	0.752	0.37	0.68	0.24	0.06	0.17	1.13	0.36	0.51	0.8	0.96
ENSMUSG00000036231	Agr3	anterior gradient 3 [Source:MGI Symbol;Acc:MGI:2685734]	0.477722962	-1.065753871	2.80E-05	0.000150685	yes	down	12.81	4.974	9.38	17.24	9.73	15.29	12.41	4.89	5.51	8.51	1.71	4.25
ENSMUSG00000031980	Agt	angiotensinogen (serpin peptidase inhibitor, clade A, member 8) [Source:MGI Symbol;Acc:MGI:87963]	0.146492873	-2.77109762	8.89E-05	0.000435465	yes	down	0.986	0.122	0.55	0.68	2.6	0.26	0.84	0.15	0.23	0.14	0	0.09
ENSMUSG00000049115	Agtr1a	angiotensin II receptor, type 1a [Source:MGI Symbol;Acc:MGI:87964]	0.417650413	-1.259632229	8.41E-15	1.43E-13	yes	down	16.788	5.744	22.01	13.02	15.14	16.21	17.56	5.68	6.19	6.54	6.07	4.24
ENSMUSG00000072812	Ahnak2	AHNAK nucleoprotein 2 [Source:MGI Symbol;Acc:MGI:2144831]	2.673051908	1.418487854	1.48E-18	3.41E-17	yes	up	8.396	17.734	6.31	7.32	9.72	7.27	11.36	16.71	18.39	19.01	18.29	16.27
ENSMUSG00000086712	AI427809	expressed sequence AI427809 [Source:MGI Symbol;Acc:MGI:2140270]	8.36016239	3.063530966	3.61E-11	4.25E-10	yes	up	0.402	2.21	0.16	0.52	0.16	0.83	0.34	2.27	2.25	1.52	2.57	2.44
ENSMUSG00000045165	AI467606	expressed sequence AI467606 [Source:MGI Symbol;Acc:MGI:2141979]	2.176122006	1.121759445	8.28E-36	4.91E-34	yes	up	14.43	26.542	12.37	15.78	14.39	15	14.61	24.1	27.6	25.38	27.65	27.98
ENSMUSG00000056145	AI504432	expressed sequence AI504432 [Source:MGI Symbol;Acc:MGI:2139742]	2.714695373	1.440790317	5.60E-31	2.72E-29	yes	up	4.43	10.134	4.49	3.56	5.76	4.54	3.8	10.63	11.94	9.31	8.95	9.84
ENSMUSG00000087107	AI662270	expressed sequence AI662270 [Source:MGI Symbol;Acc:MGI:2144254]	3.83707802	1.9400081	2.34E-41	1.69E-39	yes	up	19.548	56.706	9.65	22.88	29.59	22.14	13.48	71.88	51.86	48.53	54.14	57.12
ENSMUSG00000040627	Aicda	activation-induced cytidine deaminase [Source:MGI	8.148571493	3.026547166	0.000192121	0.000880475	yes	up	0.098	0.624	0.12	0.17	0.06	0.14	0	1.4	0.43	0.11	0.12	1.06

	Symbol;Acc:MGI:1342279]																		
ENSMUSG00000024397 Aif1	allograft inflammatory factor 1 [Source:MGI Symbol;Acc:MGI:1343098]	5.582209344	2.480836229	1.05E-31	5.28E-30	yes	up	6.184	29.138	6.16	7.15	6.76	6.38	4.47	27.33	22.59	29.45	22.68	43.64
ENSMUSG00000037860 Aim2	absent in melanoma 2 [Source:MGI Symbol;Acc:MGI:2686159]	3.298099689	1.721635006	6.88E-26	2.51E-24	yes	up	7.712	20.914	9.06	6.32	10.81	8.69	3.68	21.64	23.22	14.96	22.21	22.54
ENSMUSG00000029419 Ajm1	apical junction component 1 [Source:MGI Symbol;Acc:MGI:2685842]	35.48055896	5.148956833	0.000745684	0.003005187	yes	up	0	0.102	0	0	0	0	0	0.09	0.07	0.29	0	0.06
ENSMUSG00000038587 Akap12	A kinase (PRKA) anchor protein (gravin) 12 [Source:MGI Symbol;Acc:MGI:1932576]	0.384705347	-1.378174213	4.70E-20	1.20E-18	yes	down	62.96	20.356	62.16	71.59	43.9	91.23	45.92	18.48	22.8	21.21	19.76	19.53
ENSMUSG00000021057 Akap5	A kinase (PRKA) anchor protein 5 [Source:MGI Symbol;Acc:MGI:2685104]	0.491087581	-1.025947755	1.67E-16	3.31E-15	yes	down	168.864	77.384	177.83	170.96	158.74	196.91	139.88	50.83	86.74	69.27	111.86	68.22
ENSMUSG00000061603 Akap6	A kinase (PRKA) anchor protein 6 [Source:MGI Symbol;Acc:MGI:3050566]	0.430552733	-1.21573815	0.003141883	0.010845676	yes	down	1.052	2.07	0.93	2.6	0.25	0.8	0.68	9.3	0.33	0.34	0.06	0.32
ENSMUSG00000052131 Akr1b7	aldo-keto reductase family 1, member B7 [Source:MGI Symbol;Acc:MGI:101918]	0.483778229	-1.047582247	0.003292943	0.0113088	yes	down	2.34	0.954	2.97	2.63	1.95	2.63	1.52	1.09	1.29	0.59	1.29	0.51
ENSMUSG00000025270 Alas2	aminolevulinic acid synthase 2, erythroid [Source:MGI Symbol;Acc:MGI:87990]	0.258683116	-1.9507422	8.51E-28	3.48E-26	yes	down	73.796	16.062	80.41	53.57	69.51	98.89	66.6	11.65	16.47	11.25	22.62	18.32
ENSMUSG00000029368 Alb	albumin [Source:MGI Symbol;Acc:MGI:87991]	0.197218949	-2.34212992	2.31E-05	0.00012601	yes	down	0.738	0.122	1.01	0.52	0.28	0.7	1.18	0.14	0.13	0.1	0.11	0.13
ENSMUSG00000053279 Aldh1a1	aldehyde dehydrogenase family 1, subfamily A1 [Source:MGI Symbol;Acc:MGI:1353450]	0.466058282	-1.101417716	2.37E-24	7.88E-23	yes	down	1091.362	426.938	1145.6	1067.67	1021	1072.08	1150.46	488.95	377.54	500.43	418.77	349
ENSMUSG00000013584 Aldh1a2	aldehyde dehydrogenase family 1, subfamily A2 [Source:MGI Symbol;Acc:MGI:107928]	0.458578868	-1.124758221	1.14E-14	1.91E-13	yes	down	18.948	7.318	20.47	20.05	17.65	20.24	16.33	6.65	6.04	8.94	9.23	5.73
ENSMUSG00000015134 Aldh1a3	aldehyde dehydrogenase family 1, subfamily A3 [Source:MGI Symbol;Acc:MGI:1861722]	0.487865723	-1.03544397	0.003950893	0.013299643	yes	down	0.85	0.422	0.81	1.11	0.84	0.59	0.9	0.28	0.48	0.63	0.5	0.22
ENSMUSG00000035561 Aldh1b1	aldehyde dehydrogenase 1 family, member B1 [Source:MGI Symbol;Acc:MGI:1919785]	3.758634604	1.91020867	0.001649342	0.00612809	yes	up	1.22	3.876	0.21	3.41	0.9	0.66	0.92	3.69	2.96	4.51	4.38	3.84
ENSMUSG00000019102 Aldh3a1	aldehyde dehydrogenase family 3, subfamily A1 [Source:MGI Symbol;Acc:MGI:1353451]	0.296363495	-1.754560344	1.55E-06	1.03E-05	yes	down	23.668	5.902	25.71	31.37	13.52	20.82	26.92	5.98	3.34	11.07	4.27	4.85
ENSMUSG00000017390 Aldoc	aldolase C, fructose-bisphosphate [Source:MGI Symbol;Acc:MGI:101863]	3.681764337	1.880397285	1.91E-22	5.67E-21	yes	up	3.802	11.116	2.68	4.9	4.21	3.39	3.83	7.12	13.76	10.16	14.29	10.25
ENSMUSG00000000320 Alox12	arachidonate 12-lipoxygenase [Source:MGI Symbol;Acc:MGI:87998]	0.328186377	-1.607412742	7.37E-11	8.42E-10	yes	down	5.33	1.512	4.37	9.12	3.86	4.99	4.31	1.49	1.36	1.03	1.99	1.69
ENSMUSG00000018924 Alox15	arachidonate 15-lipoxygenase [Source:MGI Symbol;Acc:MGI:87997]	0.405496427	-1.302238893	0.004677613	0.015473549	yes	down	1.342	0.64	0.92	3.03	1.25	1.01	0.5	0.18	1.38	0.56	0.49	0.59
ENSMUSG00000060063 Alox5ap	arachidonate 5-lipoxygenase activating protein [Source:MGI Symbol;Acc:MGI:107505]	2.621787293	1.390550644	1.16E-43	9.17E-42	yes	up	69.79	155.006	65.78	80.29	69.74	58.08	75.06	146.45	158.62	148.6	152.95	168.41

ENSMUSG00000038763 Alpk3	alpha-kinase 3 [Source:MGI Symbol;Acc:MGI:2151224]	0.440006918	-1.184401888	0.007657004	0.02386587	yes	down	0.392	0.142	0.35	0.54	0.45	0.34	0.28	0.06	0.11	0.35	0.08	0.11
ENSMUSG00000036820 Amdhd2	amidohydrolase domain containing 2 [Source:MGI Symbol;Acc:MGI:2443978]	2.015653393	1.011247578	6.24E-12	8.03E-11	yes	up	13.114	22.108	13.11	15.58	11.88	12.51	12.49	15.04	24.97	23.19	20.9	26.44
ENSMUSG00000021314 Amph	amphiphysin [Source:MGI Symbol;Acc:MGI:103574]	0.335413261	-1.575988365	3.11E-32	1.63E-30	yes	down	9.816	2.854	9.34	9.46	8.86	12.34	9.08	2.47	3.24	3	3.06	2.5
ENSMUSG00000050022 Amz1	archaelysin family metallopeptidase 1 [Source:MGI Symbol;Acc:MGI:2442258]	2.929242094	1.550527433	2.83E-59	3.65E-57	yes	up	5.612	14.634	4.81	5.89	6.7	5.23	5.43	11.83	14.68	16.8	16.02	13.84
ENSMUSG00000027460 Angpt4	angiopoietin 4 [Source:MGI Symbol;Acc:MGI:1336887]	0.238172533	-2.069921049	0.000347384	0.001508126	yes	down	0.972	0.198	0.86	1.86	0.15	0.72	1.27	0.29	0.31	0.09	0.12	0.18
ENSMUSG00000028553 Angptl3	angiopoietin-like 3 [Source:MGI Symbol;Acc:MGI:1353627]	3.756229518	1.909285219	0.002940957	0.01024956	yes	up	0.194	0.84	0.4	0.05	0.1	0.18	0.24	1.09	0.55	0.19	1.98	0.39
ENSMUSG00000002289 Angptl4	angiopoietin-like 4 [Source:MGI Symbol;Acc:MGI:1888999]	0.316649066	-1.659043271	5.41E-08	4.45E-07	yes	down	64.9	17.186	63.63	53.75	59.3	73.98	73.84	16.87	30.12	10.75	18.56	9.63
ENSMUSG00000074771 Ankef1	ankyrin repeat and EF-hand domain containing 1 [Source:MGI Symbol;Acc:MGI:2441685]	0.372778687	-1.423608716	0.002089969	0.007558451	yes	down	1.444	0.418	0.68	1.45	2.41	0.56	2.12	0.17	0.41	1	0.38	0.13
ENSMUSG00000046295 Ankle1	ankyrin repeat and LEM domain containing 1 [Source:MGI Symbol;Acc:MGI:1918775]	2.871381521	1.521745034	0.005986394	0.019244163	yes	up	0.204	0.578	0.24	0.22	0.1	0.31	0.15	0.49	0.38	0.17	1.03	0.82
ENSMUSG00000034212 Ankmy1	ankyrin repeat and MYND domain containing 1 [Source:MGI Symbol;Acc:MGI:3045261]	0.394671674	-1.341275116	0.004568914	0.015149074	yes	down	1.134	0.354	1.28	1.02	0.53	1.3	1.54	0.21	0.66	0.62	0.07	0.21
ENSMUSG00000057766 Ankrd29	ankyrin repeat domain 29 [Source:MGI Symbol;Acc:MGI:2687055]	0.479892133	-1.059217933	6.60E-18	1.46E-16	yes	down	13.17	5.172	11.56	14.3	13.8	11.94	14.25	3.79	4.82	5.48	5.34	6.43
ENSMUSG00000045034 Ankrd34b	ankyrin repeat domain 34B [Source:MGI Symbol;Acc:MGI:2443245]	11.73036382	3.552175855	1.47E-11	1.81E-10	yes	up	0.076	0.746	0.09	0	0.18	0.1	0.01	0.45	0.66	0.68	0.7	1.24
ENSMUSG00000050914 Ankrd37	ankyrin repeat domain 37 [Source:MGI Symbol;Acc:MGI:3603344]	2.763350681	1.466418658	1.94E-07	1.48E-06	yes	up	3.77	7.664	2.65	4.48	5.59	3.34	2.79	6.72	7.04	8.9	4.43	11.23
ENSMUSG00000049985 Ankrd55	ankyrin repeat domain 55 [Source:MGI Symbol;Acc:MGI:1924568]	7.252190779	2.858416878	1.24E-09	1.25E-08	yes	up	0.568	5.73	0.47	0.47	1.3	0.2	0.4	3.66	2.12	1.91	17.86	3.1
ENSMUSG00000078137 Ankrd63	ankyrin repeat domain 63 [Source:MGI Symbol;Acc:MGI:2686183]	0.352829617	-1.502956429	2.81E-10	3.03E-09	yes	down	4.442	1.324	4.29	2.31	4.93	5.75	4.93	1.43	1.6	1.04	1.75	0.8
ENSMUSG00000021322 Aoah	acyloxyacyl hydrolase [Source:MGI Symbol;Acc:MGI:1350928]	16.33910754	4.030257279	3.09E-24	1.02E-22	yes	up	0.92	13.76	0.43	1.56	1.43	0.51	0.67	15.32	12.68	10.43	12.25	18.12
ENSMUSG00000019326 Aoc3	amine oxidase, copper containing 3 [Source:MGI Symbol;Acc:MGI:1306797]	0.397525555	-1.330880489	0.008036362	0.024906908	yes	down	3.452	1.156	2.31	5.51	4.45	2.1	2.89	0.54	0.98	2.58	0.51	1.17
ENSMUSG00000063558 Aox1	aldehyde oxidase 1 [Source:MGI Symbol;Acc:MGI:88035]	0.488128234	-1.034667892	7.43E-23	2.26E-21	yes	down	15.064	6.252	17.3	13.29	14.88	15.89	13.96	5.35	6.47	7.24	6.77	5.43
ENSMUSG00000024480 Ap3s1	adaptor-related protein complex 3, sigma 1 subunit [Source:MGI Symbol;Acc:MGI:1337062]	2.463092674	1.300470911	2.19E-30	1.03E-28	yes	up	30.352	62.244	27.8	28.94	41.32	27.01	26.69	69.44	57.03	57.74	68.78	58.23
ENSMUSG00000030519 Apba2	amyloid beta (A4) precursor protein-binding, family A, member 2 [Source:MGI Symbol;Acc:MGI:1261791]	2.016130258	1.011588852	0.004894916	0.01607568	yes	up	0.842	0.984	0.78	0.36	0.83	0.34	1.9	1.06	0.87	1.64	0.7	0.65
ENSMUSG00000026786 Apbb1ip	amyloid beta (A4) precursor protein-binding, family B, member 1 interacting protein [Source:MGI Symbol;Acc:MGI:1861354]	2.933979124	1.552858606	7.61E-58	9.53E-56	yes	up	13.806	34.112	14.77	15.24	15.26	11.55	12.21	32.79	38.53	33.67	33.04	32.53
ENSMUSG00000037010 Apln	apelin [Source:MGI Symbol;Acc:MGI:1353624]	0.385766094	-1.374201748	3.01E-22	8.76E-21	yes	down	28.156	9.054	33.45	28.67	23.07	33.75	21.84	7.5	10.73	7.74	11.04	8.26

ENSMUSG00000020609 Apob	apolipoprotein B [Source:MGI Symbol;Acc:MGI:88052]	0.470987118	-1.086240493	0.010474152	0.031245916	yes	down	0.336	0.166	0.12	0.18	0.33	0.69	0.36	0.13	0.21	0.19	0.18	0.12
ENSMUSG00000040613 Apobec1	apolipoprotein B mRNA editing enzyme, catalytic polypeptide 1 [Source:MGI Symbol;Acc:MGI:103298]	4.955340881	2.308984305	9.34E-125	6.06E-122	yes	up	12.2	62.466	11.41	12.99	11.79	10.88	13.93	58.17	68.81	65.52	50.02	69.81
ENSMUSG00000042759 Apobr	apolipoprotein B receptor [Source:MGI Symbol;Acc:MGI:2176230]	2.900630409	1.536366483	2.65E-58	3.36E-56	yes	up	8.768	20.608	7.25	9.12	8.26	9.79	9.42	18.73	19.9	19.82	19.6	24.99
ENSMUSG00000040564 Apoc1	apolipoprotein C-I [Source:MGI Symbol;Acc:MGI:88053]	2.715770776	1.441361715	1.88E-19	4.63E-18	yes	up	73.698	164.79	50.97	91.47	87.15	76.57	62.33	182.57	140.37	163.91	190.85	146.25
ENSMUSG00000002992 Apoc2	apolipoprotein C-II [Source:MGI Symbol;Acc:MGI:88054]	4.697006342	2.231741541	7.76E-11	8.85E-10	yes	up	4.264	16.842	3.57	3.61	2.61	8.71	2.82	20.01	11.31	18.33	10.97	23.59
ENSMUSG00000002985 Apoe	apolipoprotein E [Source:MGI Symbol;Acc:MGI:88057]	5.92425541	2.56663384	5.73E-124	3.61E-121	yes	up	1492.014	7545.254	1330.31	1438.85	1793.48	1488.62	1408.81	8534.9	6570.84	7534.35	7042.04	8044.14
ENSMUSG00000068252 Apol7b	apolipoprotein L 7b [Source:MGI Symbol;Acc:MGI:3583950]	6.141983908	2.618704733	0.000109263	0.000525363	yes	up	0.216	0.66	0.09	0	0.08	0.68	0.23	0.56	0.37	1.26	0.17	0.94
ENSMUSG00000044309 Apol7c	apolipoprotein L 7c [Source:MGI Symbol;Acc:MGI:1920912]	4.542618566	2.18352417	3.80E-15	6.63E-14	yes	up	1.714	5.754	2.51	1.44	2.11	1.18	1.33	6.26	5.11	5.34	2.72	9.34
ENSMUSG00000071716 Apol7e	apolipoprotein L 7e [Source:MGI Symbol;Acc:MGI:3704456]	2.279062597	1.18844055	1.82E-06	1.20E-05	yes	up	2.196	5.582	1.6	2.52	2.43	2.73	1.7	6.9	5.21	3.74	4.93	7.13
ENSMUSG00000090698 Apold1	apolipoprotein L domain containing 1 [Source:MGI Symbol;Acc:MGI:2685921]	0.275615419	-1.859271495	0.003482902	0.011882164	yes	down	3.558	0.826	1.38	9.87	2.37	2.11	2.06	0.7	0.6	1.78	0.42	0.63
ENSMUSG00000006589 Aprt	adenine phosphoribosyl transferase [Source:MGI Symbol;Acc:MGI:88061]	2.088324009	1.062345567	1.78E-28	7.64E-27	yes	up	47.496	84.41	45.2	51.86	49.24	46.2	44.98	80.64	78.93	79.96	98.8	83.72
ENSMUSG00000041219 Arhgap11a	Rho GTPase activating protein 11A [Source:MGI Symbol;Acc:MGI:2444300]	2.533136876	1.340925034	8.21E-16	1.51E-14	yes	up	3.222	6.936	2.85	3.41	4.09	2.17	3.59	6.81	7.4	6.69	5.84	7.94
ENSMUSG00000025154 Arhgap19	Rho GTPase activating protein 19 [Source:MGI Symbol;Acc:MGI:1918335]	3.132063262	1.647113353	1.06E-41	7.83E-40	yes	up	3.832	11.392	2.8	3.57	5.27	5.22	2.3	12.63	13.14	8.96	9.58	12.65
ENSMUSG00000030047 Arhgap25	Rho GTPase activating protein 25 [Source:MGI Symbol;Acc:MGI:2443687]	3.133258816	1.647663945	1.96E-63	2.89E-61	yes	up	13.028	31.268	14.6	12.78	14.79	11.41	11.56	29.46	34.65	29.34	33.2	29.69
ENSMUSG00000048865 Arhgap30	Rho GTPase activating protein 30 [Source:MGI Symbol;Acc:MGI:2684948]	2.749042701	1.458929317	4.77E-55	5.44E-53	yes	up	14.202	32.858	13.12	15.96	15.23	14.95	11.75	36.6	33.29	29.85	30.86	33.69
ENSMUSG00000040345 Arhgap9	Rho GTPase activating protein 9 [Source:MGI Symbol;Acc:MGI:2143764]	2.507944643	1.326505504	1.23E-53	1.36E-51	yes	up	12.466	26.594	11.85	12.64	14.09	11.79	11.96	27.2	28.15	24.71	25.31	27.6
ENSMUSG00000030220 Arhgdib	Rho, GDP dissociation inhibitor (GDI) beta [Source:MGI Symbol;Acc:MGI:101940]	2.163822502	1.11358216	6.89E-30	3.18E-28	yes	up	110.582	202.042	105.29	118.93	115.43	128.05	85.21	202.66	190.85	195.92	192.51	228.27
ENSMUSG00000025656 Arhgef9	CDC42 guanine nucleotide exchange factor (GEF) 9 [Source:MGI Symbol;Acc:MGI:2442233]	0.439488873	-1.186101456	1.79E-12	2.43E-11	yes	down	2.49	1.096	2.38	2.29	2.39	3.14	2.25	0.72	1.22	1.18	1.38	0.98
ENSMUSG00000019564 Arid3a	AT rich interactive domain 3A (BRIGHT-like) [Source:MGI Symbol;Acc:MGI:1328360]	2.131306007	1.091737746	1.50E-16	3.00E-15	yes	up	2.95	5.418	2.49	2.67	3.29	3.66	2.64	5.97	5.7	4.69	4.57	6.16
ENSMUSG00000043157 Arl11	ADP-ribosylation factor-like 11 [Source:MGI	5.924761396	2.566757055	1.67E-60	2.21E-58	yes	up	8.576	34.452	10.89	9.57	9.86	4.63	7.93	28.21	40.23	35.79	34.73	33.3

	Symbol;Acc:MGI:2444054]																		
ENSMUSG00000034936 Arl4d	ADP-ribosylation factor-like 4D [Source:MGI Symbol;Acc:MGI:1933155]	0.22323298	-2.163377912	1.95E-66	3.10E-64	yes	down	183.376	34.596	192.71	188.38	152.55	196.18	187.06	37.4	39.11	39.47	33.14	23.86
ENSMUSG00000037683 Armc3	armadillo repeat containing 3 [Source:MGI Symbol;Acc:MGI:1918132]	0.461844618	-1.11452054	9.43E-07	6.49E-06	yes	down	3.02	1.18	2.95	2.98	2.62	3	3.55	1.15	1.45	1.58	0.66	1.06
ENSMUSG00000061802 Armc4	armadillo repeat containing 4 [Source:MGI Symbol;Acc:MGI:1922184]	0.491459903	-1.024854381	0.000473425	0.001993876	yes	down	2.76	1.056	2.73	2.84	1.72	2.1	4.41	1.02	0.92	1.76	0.62	0.96
ENSMUSG00000057219 Armc7	armadillo repeat containing 7 [Source:MGI Symbol;Acc:MGI:2679719]	2.130376003	1.091108083	3.74E-12	4.90E-11	yes	up	4.898	9.284	3.97	6.18	5.49	5.31	3.54	12.23	9.38	7	9.01	8.8
ENSMUSG00000036242 Armh4	armadillo-like helical domain containing 4 [Source:MGI Symbol;Acc:MGI:1914669]	0.436132324	-1.197162176	3.61E-07	2.64E-06	yes	down	2.972	1.08	3.19	2.7	3.44	2.38	3.15	0.64	1.1	1.64	1.08	0.94
ENSMUSG00000015709 Arnt2	aryl hydrocarbon receptor nuclear translocator 2 [Source:MGI Symbol;Acc:MGI:107188]	4.784980718	2.258513111	1.68E-12	2.28E-11	yes	up	0.236	5.796	0.31	0.28	0.2	0.29	0.1	25.48	0.76	0.51	1.16	1.07
ENSMUSG00000055116 Arntl	aryl hydrocarbon receptor nuclear translocator-like [Source:MGI Symbol;Acc:MGI:1096381]	0.411117066	-1.282378835	1.85E-15	3.32E-14	yes	down	24.974	8.714	24.01	22.09	26.05	27.95	24.77	8.25	12.93	8.06	8.72	5.61
ENSMUSG00000060216 Arrb2	arrestin, beta 2 [Source:MGI Symbol;Acc:MGI:99474]	2.109617607	1.076981517	3.32E-28	1.39E-26	yes	up	30.276	53.196	30.6	31.7	35.31	26.49	27.28	59.72	57.07	46.64	48.49	54.06
ENSMUSG00000074794 Arrdc3	arrestin domain containing 3 [Source:MGI Symbol;Acc:MGI:2145242]	0.388161759	-1.365270101	3.40E-35	1.98E-33	yes	down	125.244	40.948	117.26	136.77	110.09	147.96	114.14	37.64	52.49	37.11	43.84	33.66
ENSMUSG00000020604 Arsg	arylsulfatase G [Source:MGI Symbol;Acc:MGI:1921258]	2.145716848	1.101459708	1.74E-13	2.63E-12	yes	up	2.546	4.818	2.4	2.62	2.41	2.52	2.78	3.49	5.81	3.91	6.32	4.56
ENSMUSG00000046561 Arsj	arylsulfatase J [Source:MGI Symbol;Acc:MGI:2443513]	0.396424144	-1.334883262	5.30E-08	4.37E-07	yes	down	1.682	0.564	1.22	2.25	1.72	1.79	1.43	0.37	0.49	0.57	0.69	0.7
ENSMUSG00000092517 Art2a-ps	ADP-ribosyltransferase 2a, pseudogene [Source:MGI Symbol;Acc:MGI:107546]	2.901716552	1.5369066	2.46E-06	1.60E-05	yes	up	0.34	1.264	0.22	0.28	0.23	0.79	0.18	1.72	1.33	1.34	0.4	1.53
ENSMUSG00000048040 Arxes2	adipocyte-related X-chromosome expressed sequence 2 [Source:MGI Symbol;Acc:MGI:1924226]	0.452243407	-1.144828624	4.09E-09	3.91E-08	yes	down	15.772	6.012	14.68	14.28	23.75	15.6	10.55	6.3	5.96	4.59	7.32	5.89
ENSMUSG00000021898 Asb14	ankyrin repeat and SOCS box-containing 14 [Source:MGI Symbol;Acc:MGI:2655107]	0.349242366	-1.517699515	0.006569639	0.020911918	yes	down	1.346	0.46	0.65	1.09	1.56	1.76	1.67	0.31	0.48	1.23	0.23	0.05
ENSMUSG00000009248 Ascl2	achaete-scute family bHLH transcription factor 2 [Source:MGI Symbol;Acc:MGI:96920]	4.131507283	2.046668211	0.006946199	0.021940973	yes	up	0.172	0.452	0.09	0.05	0.05	0.29	0.38	0.57	0.42	0.69	0.26	0.32
ENSMUSG00000005470 Asf1b	anti-silencing function 1B histone chaperone [Source:MGI Symbol;Acc:MGI:1914179]	2.325969478	1.217832165	5.21E-09	4.91E-08	yes	up	2.722	6.044	2.16	1.9	3.06	2.73	3.76	5.9	4.09	3.99	5.27	10.97
ENSMUSG00000020884 Asgr1	asialoglycoprotein receptor 1 [Source:MGI Symbol;Acc:MGI:88081]	0.085421778	-3.54925226	1.78E-09	1.76E-08	yes	down	2.936	0.24	4.99	1.76	1.14	3.03	3.76	0.32	0.1	0.56	0.17	0.05
ENSMUSG00000020774 Aspa	aspartoacylase [Source:MGI Symbol;Acc:MGI:87914]	0.42863881	-1.222165613	1.53E-05	8.59E-05	yes	down	6.534	2.672	4.2	8.47	7.89	7.86	4.25	2.81	4.22	1.92	2	2.41
ENSMUSG00000033952 Aspm	abnormal spindle microtubule assembly [Source:MGI Symbol;Acc:MGI:1334448]	3.131881511	1.647029632	1.65E-09	1.64E-08	yes	up	0.576	1.282	0.53	0.52	0.68	0.42	0.73	1.54	0.94	1.08	0.95	1.9
ENSMUSG00000076441 Ass1	argininosuccinate synthetase 1 [Source:MGI Symbol;Acc:MGI:88090]	3.140356235	1.650928224	9.14E-46	7.89E-44	yes	up	23.94	63.224	25.03	22.63	23.22	25.47	23.35	63.95	53.66	57.71	58.36	82.44

ENSMUSG00000034958	Atcay	ataxia, cerebellar, Cayman type [Source:MGI Symbol;Acc:MGI:2448730]	4.78781521	2.259367471	0.000835008	0.003330079	yes	up	0.136	0.21	0.05	0.05	0.51	0.07	0	0.17	0.08	0.28	0.24	0.28
ENSMUSG00000025324	Atp10a	ATPase, class V, type 10A [Source:MGI Symbol;Acc:MGI:1330809]	2.069676299	1.049405146	2.96E-13	4.32E-12	yes	up	5.052	8.86	4.29	5.98	5.31	3.7	5.98	7.37	9.19	9.79	9	8.95
ENSMUSG00000038094	Atp13a4	ATPase type 13A4 [Source:MGI Symbol;Acc:MGI:1924456]	0.459359304	-1.122305045	9.52E-06	5.55E-05	yes	down	2.806	1.168	4.41	2.59	1.49	2.8	2.74	0.41	1.03	2.4	1.03	0.97
ENSMUSG00000048939	Atp13a5	ATPase type 13A5 [Source:MGI Symbol;Acc:MGI:2444068]	0.066559976	-3.909201272	0.001275301	0.004865819	yes	down	5.562	0.008	0.05	0.27	27.31	0.15	0.03	0.01	0.03	0	0	0
ENSMUSG00000007097	Atp1a2	ATPase, Na+/K+ transporting, alpha 2 polypeptide [Source:MGI Symbol;Acc:MGI:88106]	0.466150175	-1.101133285	1.31E-24	4.41E-23	yes	down	27.612	10.77	25.72	24.5	30.89	33.68	23.27	10.34	11.37	12.37	9.95	9.82
ENSMUSG00000040907	Atp1a3	ATPase, Na+/K+ transporting, alpha 3 polypeptide [Source:MGI Symbol;Acc:MGI:88107]	8.367120951	3.06473129	1.76E-28	7.59E-27	yes	up	2.972	20.888	3.04	3.65	1.42	4.45	2.3	21.35	18.8	16.83	21.31	26.15
ENSMUSG00000034112	Atp2c2	ATPase, Ca++ transporting, type 2C, member 2 [Source:MGI Symbol;Acc:MGI:1916297]	0.48386511	-1.047323179	0.000716925	0.002899652	yes	down	2.878	1.178	3.14	2.7	2.41	2.23	3.91	0.89	1.04	2.39	0.69	0.88
ENSMUSG00000038600	Atp6v0a4	ATPase, H+ transporting, lysosomal V0 subunit A4 [Source:MGI Symbol;Acc:MGI:2153480]	0.438497473	-1.189359565	1.98E-07	1.51E-06	yes	down	2.912	1.018	3.04	2.89	3.78	1.99	2.86	0.75	1.55	0.87	1.19	0.73
ENSMUSG00000024121	Atp6v0c	ATPase, H+ transporting, lysosomal V0 subunit C [Source:MGI Symbol;Acc:MGI:88116]	2.112266612	1.078791945	5.99E-61	8.02E-59	yes	up	214.176	381.894	218.85	220.48	207.77	208.26	215.52	347.79	388.94	363.11	385.28	424.35
ENSMUSG00000028238	Atp6v0d2	ATPase, H+ transporting, lysosomal V0 subunit D2 [Source:MGI Symbol;Acc:MGI:1924415]	7.467369163	2.900600055	2.39E-42	1.79E-40	yes	up	29.292	182.158	30.36	30.81	36.3	17.21	31.78	179.56	201.56	164.9	209.03	155.74
ENSMUSG00000006273	Atp6v1b2	ATPase, H+ transporting, lysosomal V1 subunit B2 [Source:MGI Symbol;Acc:MGI:109618]	2.1814147	1.125264061	5.58E-49	5.33E-47	yes	up	76.072	141.098	70.07	75.92	81.25	71.23	81.89	128.96	148.59	139.97	151.03	136.94
ENSMUSG00000060131	Atp8b4	ATPase, class I, type 8B, member 4 [Source:MGI Symbol;Acc:MGI:1859664]	2.608213808	1.383062139	1.80E-19	4.43E-18	yes	up	2.942	6.136	3	3.12	4.21	1.88	2.5	7.25	6.01	5.74	5.75	5.93
ENSMUSG00000028457	Atp8b5	ATPase, class I, type 8B, member 5 [Source:MGI Symbol;Acc:MGI:2444287]	0.312014017	-1.680317251	0.015665733	0.044121164	yes	down	0.324	0.076	0.13	0.5	0.24	0.27	0.48	0.05	0.09	0.08	0.12	0.04
ENSMUSG00000097415	AU020206	expressed sequence AU020206 [Source:MGI Symbol;Acc:MGI:2142134]	2.611679397	1.384977806	5.56E-46	4.86E-44	yes	up	58.676	123.812	62.56	51.41	64.25	60.59	54.57	138.04	135.05	111.85	110.37	123.75
ENSMUSG00000115230	AU022793	expressed sequence AU022793 [Source:MGI Symbol;Acc:MGI:2146144]	10.66317767	3.414565525	4.99E-26	1.84E-24	yes	up	0.512	4.762	0.88	0.19	0.82	0.3	0.37	3.47	6.51	4.05	3.32	6.46
ENSMUSG00000027496	Aurka	aurora kinase A [Source:MGI Symbol;Acc:MGI:894678]	2.191519282	1.131931372	5.37E-05	0.000272187	yes	up	2.16	4.09	2.92	1.35	3.28	1.72	1.53	5.1	3.08	3.37	3.27	5.63
ENSMUSG00000020897	Aurkb	aurora kinase B [Source:MGI Symbol;Acc:MGI:107168]	2.902055531	1.537075126	5.18E-06	3.17E-05	yes	up	1.902	4.814	1.42	0.73	3.04	1.63	2.69	6.07	3.91	3.19	3.02	7.88
ENSMUSG00000020123	Avpr1a	arginine vasopressin receptor 1A [Source:MGI Symbol;Acc:MGI:1859216]	0.271690521	-1.879963863	3.12E-08	2.65E-07	yes	down	1.684	0.388	1.66	1.98	1.37	2.02	1.39	0.49	0.5	0.21	0.58	0.16

ENSMUSG00000075010 AW112010	expressed sequence AW112010 [Source:MGI Symbol;Acc:MGI:2147706]	2.6704132	1.417062991	4.26E-21	1.16E-19	yes	up	134.158	311.818	133.27	141.75	138.87	134.22	122.68	319.59	224.33	291.61	265.33	458.23
ENSMUSG00000085998 AW822252	expressed sequence AW822252 [Source:MGI Symbol;Acc:MGI:2148030]	10.75163682	3.426484407	0.003657194	0.012412436	yes	up	0.022	0.5	0	0.06	0	0.05	0	0.24	0.31	0.38	1.36	0.21
ENSMUSG00000015665 Awat1	acyl-CoA wax alcohol acyltransferase 1 [Source:MGI Symbol;Acc:MGI:3588200]	10.9546195	3.453467469	0.001005533	0.003938278	yes	up	0.066	0.59	0	0.33	0	0	0	0.22	0.29	0.53	0.94	0.97
ENSMUSG00000002602 Axl	AXL receptor tyrosine kinase [Source:MGI Symbol;Acc:MGI:1347244]	2.710734258	1.438683688	5.88E-38	3.80E-36	yes	up	59.352	138.714	56.79	59.67	68.18	46.17	65.95	106.36	150.34	143.04	140.54	153.29
ENSMUSG00000037053 Azgp1	alpha-2-glycoprotein 1, zinc [Source:MGI Symbol;Acc:MGI:103163]	0.486924134	-1.038231087	9.56E-06	5.57E-05	yes	down	5.968	2.456	5.37	4.94	6.64	7.61	5.28	3.12	2.19	3.05	1.49	2.43
ENSMUSG00000045746 B230317F23Rik	RIKEN cDNA B230317F23 gene [Source:MGI Symbol;Acc:MGI:2443920]	18.98979896	4.247152727	0.015548213	0.0438363	yes	up	0	0.074	0	0	0	0	0	0.18	0.04	0.04	0.11	0
ENSMUSG00000060802 B2m	beta-2 microglobulin [Source:MGI Symbol;Acc:MGI:88127]	2.648604809	1.405232599	1.36E-64	2.08E-62	yes	up	3365.596	7544.694	3420.96	3159.45	3797.93	3065.25	3384.39	7655.63	7239.86	7023.32	7260.85	8543.81
ENSMUSG00000074892 B3galt5	UDP-Gal:betaGlcNAc beta 1,3-galactosyltransferase, polypeptide 5 [Source:MGI Symbol;Acc:MGI:2136878]	6.9814813	2.803533174	1.56E-08	1.39E-07	yes	up	0.186	0.864	0.4	0.04	0.02	0.36	0.11	0.5	0.39	1.55	0.66	1.22
ENSMUSG00000079445 B3gnt7	UDP-GlcNAc:betaGal beta-1,3-N-acetylglucosaminyltransferase 7 [Source:MGI Symbol;Acc:MGI:2384394]	3.134600699	1.648281677	1.22E-19	3.03E-18	yes	up	3.548	9.452	3.93	3.47	4.42	2.18	3.74	7.68	11.58	8.3	10.64	9.06
ENSMUSG00000059479 B3gnt8	UDP-GlcNAc:betaGal beta-1,3-N-acetylglucosaminyltransferase 8 [Source:MGI Symbol;Acc:MGI:2385269]	2.009619047	1.006922043	1.73E-10	1.90E-09	yes	up	4.324	7.158	4.78	4.61	2.95	5.28	4	5.9	7.49	6.19	8.1	8.11
ENSMUSG00000084960 B430010I23Rik	RIKEN cDNA B430010I23 gene [Source:MGI Symbol;Acc:MGI:1926099]	0.384002935	-1.380810759	6.58E-11	7.55E-10	yes	down	48.33	13.582	49.09	38.18	39.71	65.72	48.95	15.88	17.48	11.52	9.53	13.5
ENSMUSG00000043740 B430306N03Rik	RIKEN cDNA B430306N03 gene [Source:MGI Symbol;Acc:MGI:2443478]	3.992884117	1.997431202	9.69E-17	1.97E-15	yes	up	0.576	1.808	0.45	0.7	0.63	0.64	0.46	2.13	1.68	1.19	2.03	2.01
ENSMUSG00000006731 B4galnt1	beta-1,4-N-acetyl-galactosaminyl transferase 1 [Source:MGI Symbol;Acc:MGI:1342057]	2.510047439	1.327714631	2.68E-48	2.49E-46	yes	up	40.69	85.99	37.15	44.61	39.51	39.97	42.21	72.61	94.05	92.1	79.13	92.06
ENSMUSG00000055629 B4galnt4	beta-1,4-N-acetyl-galactosaminyl transferase 4 [Source:MGI Symbol;Acc:MGI:2652891]	3.307125565	1.725577823	1.17E-05	6.73E-05	yes	up	0.37	1.082	0.32	0.28	0.32	0.4	0.53	1.2	0.92	1.65	0.58	1.06
ENSMUSG00000017929 B4galt5	UDP-Gal:betaGlcNAc beta 1,4-galactosyltransferase, polypeptide 5 [Source:MGI Symbol;Acc:MGI:1927169]	2.452629095	1.294329075	1.28E-42	9.78E-41	yes	up	11.644	23.484	9.49	11.52	14.4	12.35	10.46	22.89	24.93	21.08	26.39	22.13
ENSMUSG00000056124 B4galt6	UDP-Gal:betaGlcNAc beta 1,4-galactosyltransferase, polypeptide 6 [Source:MGI Symbol;Acc:MGI:1928380]	2.286349232	1.193045787	3.44E-16	6.56E-15	yes	up	1.792	3.45	1.58	1.73	2.39	1.59	1.67	4.02	3.09	3.46	3.37	3.31
ENSMUSG00000044633 B530045E10Rik	RIKEN cDNA B530045E10 gene [Source:MGI Symbol;Acc:MGI:2445145]	0.167553817	-2.577303543	5.11E-08	4.22E-07	yes	down	1.06	0.152	1.68	0.58	1.08	1.11	0.85	0.12	0.14	0.31	0.17	0.02

ENSMUSG00000045763 Basp1	brain abundant, membrane attached signal protein 1 [Source:MGI Symbol;Acc:MGI:1917600]	2.163949075	1.113666548	0.002861002	0.010005098	yes	up	8.464	15.494	6.13	15.47	6.51	4.86	9.35	20.97	12.61	17.12	9.04	17.73
ENSMUSG00000034266 Batf	basic leucine zipper transcription factor, ATF-like [Source:MGI Symbol;Acc:MGI:1859147]	2.894859616	1.533493388	3.06E-27	1.20E-25	yes	up	8.402	20.748	9.27	6.66	9.69	8.75	7.64	20.68	21.69	18.01	20.42	22.94
ENSMUSG00000026630 Batf3	basic leucine zipper transcription factor, ATF-like 3 [Source:MGI Symbol;Acc:MGI:1925491]	4.29408022	2.102349143	3.63E-14	5.80E-13	yes	up	3.104	10.596	1.84	5.29	3.5	2.62	2.27	9.71	14.4	9.85	7.78	11.24
ENSMUSG00000041660 Bbox1	butyrobetaine (gamma), 2-oxoglutarate dioxygenase 1 (gamma-butyrobetaine hydroxylase) [Source:MGI Symbol;Acc:MGI:1891372]	0.450890045	-1.149152437	0.003783596	0.012794803	yes	down	2.122	0.954	2.72	2.99	1.8	0.67	2.43	0.22	0.66	2.79	0.12	0.98
ENSMUSG00000063388 BC023105	cDNA sequence BC023105 [Source:MGI Symbol;Acc:MGI:2384767]	4.230097091	2.080690777	1.22E-06	8.26E-06	yes	up	0.742	2.638	0.44	1.11	0.52	1.1	0.54	4.3	1.33	1.95	2.07	3.54
ENSMUSG00000074300 BC030870	cDNA sequence BC030870 [Source:MGI Symbol;Acc:MGI:3039572]	0.317999343	-1.652904312	1.87E-06	1.23E-05	yes	down	3.118	0.834	2.71	4.13	2.72	2.9	3.13	0.56	0.6	1.71	0.73	0.57
ENSMUSG00000090486 BC035947	cDNA sequence BC035947 [Source:MGI Symbol;Acc:MGI:2652858]	0.083651597	-3.579463104	0.013532931	0.038865662	yes	down	0.102	0.008	0.04	0.16	0.18	0.13	0	0	0	0.02	0.02	0
ENSMUSG00000057246 BC051142	cDNA sequence BC051142 [Source:MGI Symbol;Acc:MGI:3039565]	2.807611499	1.489343317	0.000584574	0.00241079	yes	up	0.96	2.11	0.91	1.03	0.49	1.49	0.88	2.78	2.15	1.78	0.66	3.18
ENSMUSG00000086199 Bcas3os1	breast carcinoma amplified sequence 3, opposite strand 1 [Source:MGI Symbol;Acc:MGI:1918739]	0.299057065	-1.741507296	0.01506084	0.04261368	yes	down	1.176	0.204	1.47	1.47	0.2	1.96	0.78	0.32	0.28	0.2	0	0.22
ENSMUSG00000085514 Bcas3os2	breast carcinoma amplified sequence 3 opposite strand 2 [Source:MGI Symbol;Acc:MGI:1925670]	0.258540291	-1.951538967	0.003698217	0.012534879	yes	down	2.512	0.546	4.53	2.03	0.67	3.23	2.1	0.55	0.13	1.12	0.55	0.38
ENSMUSG00000030268 Bcat1	branched chain aminotransferase 1, cytosolic [Source:MGI Symbol;Acc:MGI:104861]	4.102027102	2.036337024	0.002801518	0.009814707	yes	up	0.204	0.468	0.03	0.18	0.7	0.08	0.03	0.41	1.06	0.36	0.04	0.47
ENSMUSG00000027792 Bche	butyrylcholinesterase [Source:MGI Symbol;Acc:MGI:894278]	0.228286327	-2.131083642	5.47E-18	1.22E-16	yes	down	3.034	0.576	2.85	4.01	3.33	2.4	2.58	0.44	0.79	0.84	0.51	0.3
ENSMUSG00000102037 Bcl2a1a	B cell leukemia/lymphoma 2 related protein A1a [Source:MGI Symbol;Acc:MGI:102687]	3.330469489	1.735725565	1.49E-51	1.53E-49	yes	up	15.838	44.682	17.65	15.2	15.35	13.85	17.14	45.17	45.11	42.22	43.18	47.73
ENSMUSG00000089929 Bcl2a1b	B cell leukemia/lymphoma 2 related protein A1b [Source:MGI Symbol;Acc:MGI:1278326]	3.674144641	1.877408423	5.95E-44	4.77E-42	yes	up	40.412	125.882	39.49	43.19	50.69	27.99	40.7	123.69	141.07	108.3	128.77	127.58
ENSMUSG00000099974 Bcl2a1d	B cell leukemia/lymphoma 2 related protein A1d [Source:MGI Symbol;Acc:MGI:1278325]	3.245196589	1.698305877	2.15E-22	6.35E-21	yes	up	22.862	62.148	23.04	28.37	30.2	12.97	19.73	58.43	60.59	66.08	60.45	65.19
ENSMUSG00000044165 Bcl2l15	BCL2-like 15 [Source:MGI Symbol;Acc:MGI:2685412]	13.01442702	3.702039891	0.000196068	0.000896455	yes	up	0.09	0.736	0.16	0.17	0	0.09	0.03	0.66	0.22	0.65	1.24	0.91
ENSMUSG00000099757 BE692007	expressed sequence BE692007 [Source:MGI Symbol;Acc:MGI:3035348]	2.392410945	1.258465223	2.04E-06	1.33E-05	yes	up	3.962	6.224	3.54	2.83	3.29	5.32	4.83	11.1	5.94	5.99	2.91	5.18
ENSMUSG00000042182 Bend6	BEN domain containing 6 [Source:MGI Symbol;Acc:MGI:2444572]	3.209379491	1.68229439	5.27E-12	6.83E-11	yes	up	0.896	2.552	0.8	1	0.62	1.06	1	1.75	1.83	4.27	2.69	2.22
ENSMUSG00000037418 Best1	bestrophin 1 [Source:MGI Symbol;Acc:MGI:1346332]	3.517357631	1.81449203	1.01E-17	2.18E-16	yes	up	1.938	6.256	1.92	1.87	1.76	1.31	2.83	4.71	8.49	7.12	5.73	5.23
ENSMUSG00000020169 Best3	bestrophin 3 [Source:MGI Symbol;Acc:MGI:3580298]	0.462226134	-1.113329264	1.41E-07	1.09E-06	yes	down	1.352	0.528	1.14	1.13	1.11	2.11	1.27	0.51	0.52	0.44	0.54	0.63

ENSMUSG00000050071 Bex1	brain expressed X-linked 1 [Source:MGI Symbol;Acc:MGI:1328321]	0.208303846	-2.263238616	3.86E-08	3.24E-07	yes	down	4.336	0.788	5.5	4.14	2.91	3.45	5.68	1.29	0.66	0.5	0.99	0.5
ENSMUSG00000042750 Bex2	brain expressed X-linked 2 [Source:MGI Symbol;Acc:MGI:1338017]	0.143112783	-2.804775557	3.44E-32	1.80E-30	yes	down	40.774	4.92	40.3	35.66	33.22	61.25	33.44	2.9	4.71	6.12	7.55	3.32
ENSMUSG00000047844 Bex4	brain expressed X-linked 4 [Source:MGI Symbol;Acc:MGI:3606746]	0.265110889	-1.915332167	4.16E-40	2.87E-38	yes	down	79.502	17.902	84.43	68.25	74.05	77.58	93.2	19.1	19.45	16.94	21.47	12.55
ENSMUSG00000052271 Bhlha15	basic helix-loop-helix family, member a15 [Source:MGI Symbol;Acc:MGI:891976]	6.677676091	2.739346115	4.48E-27	1.75E-25	yes	up	0.55	3.068	0.4	0.59	0.55	0.85	0.36	4.14	2.41	2.87	2.38	3.54
ENSMUSG00000025128 Bhlhe22	basic helix-loop-helix family, member e22 [Source:MGI Symbol;Acc:MGI:1930001]	3.478598611	1.798506219	5.38E-06	3.28E-05	yes	up	0.238	0.682	0.1	0.36	0.38	0.15	0.2	0.72	0.46	0.84	0.74	0.65
ENSMUSG00000030103 Bhlhe40	basic helix-loop-helix family, member e40 [Source:MGI Symbol;Acc:MGI:1097714]	2.419069202	1.274452041	2.21E-25	7.88E-24	yes	up	18.904	38.922	20.24	24.3	17.62	15.86	16.5	37.33	35.47	35.92	40.84	45.05
ENSMUSG00000030256 Bhlhe41	basic helix-loop-helix family, member e41 [Source:MGI Symbol;Acc:MGI:1930704]	5.746043676	2.522568959	3.73E-122	2.28E-119	yes	up	2.998	14.218	2.98	3	3.08	3.02	2.91	15.45	13.36	13.56	14.19	14.53
ENSMUSG00000017716 Birc5	baculoviral IAP repeat-containing 5 [Source:MGI Symbol;Acc:MGI:1203517]	3.06659162	1.616636055	1.76E-10	1.93E-09	yes	up	4.092	9.94	3.55	3.53	6.36	2.55	4.47	10.3	8.26	7.93	9.95	13.26
ENSMUSG00000061132 Blnk	B cell linker [Source:MGI Symbol;Acc:MGI:96878]	4.430348131	2.147420068	1.97E-47	1.78E-45	yes	up	4.47	17.976	4.15	5.28	4.99	4.53	3.4	21.26	17.9	15.75	14.16	20.81
ENSMUSG00000001999 Blvra	biliverdin reductase A [Source:MGI Symbol;Acc:MGI:88170]	2.04310728	1.030764959	3.97E-23	1.23E-21	yes	up	25.736	43.996	23.92	27.07	30.67	23.8	23.22	34.76	48.22	37.41	51.13	48.46
ENSMUSG00000039004 Bmp6	bone morphogenetic protein 6 [Source:MGI Symbol;Acc:MGI:88182]	0.377025737	-1.407265084	8.33E-37	5.14E-35	yes	down	87.85	28.016	89.12	90.71	71.66	109.35	78.41	23.78	33.03	23.89	30.93	28.45
ENSMUSG00000052430 Bmpr1b	bone morphogenetic protein receptor, type 1B [Source:MGI Symbol;Acc:MGI:107191]	0.423243275	-1.240440949	6.73E-10	6.95E-09	yes	down	2.59	0.878	3.06	2.85	2.16	2.02	2.86	0.82	0.63	1.02	0.77	1.15
ENSMUSG00000017146 Brca1	breast cancer 1, early onset [Source:MGI Symbol;Acc:MGI:104537]	2.181213783	1.125131177	7.46E-06	4.44E-05	yes	up	0.692	1.17	0.77	0.59	0.61	0.94	0.55	1.55	1.08	0.68	0.9	1.64
ENSMUSG00000032589 Bsn	bassoon [Source:MGI Symbol;Acc:MGI:1277955]	2.285621481	1.192586501	0.000227819	0.001030407	yes	up	0.132	0.242	0.1	0.14	0.15	0.07	0.2	0.22	0.29	0.26	0.25	0.19
ENSMUSG00000029082 Bst1	bone marrow stromal cell antigen 1 [Source:MGI Symbol;Acc:MGI:105370]	4.521234643	2.176716792	8.69E-62	1.19E-59	yes	up	21.136	78.328	22.66	18.95	23.17	13.92	26.98	78.73	77.27	69.55	87.98	78.11
ENSMUSG00000020423 Btg2	B cell translocation gene 2, anti-proliferative [Source:MGI Symbol;Acc:MGI:108384]	0.354886831	-1.494569054	2.96E-07	2.20E-06	yes	down	268.216	80.032	232.02	445.3	186.85	301.82	175.09	88.56	75.34	99.85	69.41	67
ENSMUSG00000032056 Btg4	B cell translocation gene 4 [Source:MGI Symbol;Acc:MGI:1860140]	0.108697478	-3.201609629	0.000104546	0.00050461	yes	down	0.79	0.262	0.3	1.85	0.43	0.41	0.96	0.08	0.11	1.08	0	0.04
ENSMUSG00000031264 Btk	Bruton agammaglobulinemia tyrosine kinase [Source:MGI Symbol;Acc:MGI:88216]	3.222279934	1.688081833	5.35E-31	2.61E-29	yes	up	5.962	16.128	6.06	7.43	5.95	5.5	4.87	20.08	14.23	12.56	16.52	17.25
ENSMUSG00000058435 Btnl4	butyrophilin-like 4 [Source:MGI Symbol;Acc:MGI:1932036]	254.3122959	7.990457407	7.92E-16	1.46E-14	yes	up	0	1.328	0	0	0	0	0	1.13	1.41	0.86	1.06	2.18
ENSMUSG00000073420 Btnl5-ps	butyrophilin-like 5, pseudogene [Source:MGI Symbol;Acc:MGI:1932037]	12.89764563	3.689035831	0.002199045	0.007909765	yes	up	0.084	0.77	0	0.35	0	0.07	0	0.68	0.8	0.35	0.27	1.75
ENSMUSG00000092618 Btnl6	butyrophilin-like 6 [Source:MGI Symbol;Acc:MGI:1932038]	120.7995155	6.916470859	3.08E-12	4.08E-11	yes	up	0.018	1.652	0	0.09	0	0	0	1.31	1.92	0.84	1.82	2.37

ENSMUSG00000040084 Bub1b	BUB1B, mitotic checkpoint serine/threonine kinase [Source:MGI Symbol;Acc:MGI:1333889]	3.261482958	1.70552809	8.95E-22	2.51E-20	yes	up	2.948	11.434	3.38	2.08	3.82	2.13	3.33	9	10.78	15	12.15	10.24
ENSMUSG00000071317 Bves	blood vessel epicardial substance [Source:MGI Symbol;Acc:MGI:1346013]	0.211253223	-2.242954743	0.001837592	0.006753711	yes	down	0.374	0.066	0.12	0.95	0.25	0.28	0.27	0.07	0.02	0.16	0	0.08
ENSMUSG00000087574 C030037D09Rik	RIKEN cDNA C030037D09 gene [Source:MGI Symbol;Acc:MGI:1924865]	0.436225299	-1.196854653	0.00177037	0.006529059	yes	down	1.43	0.488	2.55	0.59	1.31	1.54	1.16	0.49	0.58	0.42	0.51	0.44
ENSMUSG00000052477 C130026I21Rik	RIKEN cDNA C130026I21 gene [Source:MGI Symbol;Acc:MGI:3612702]	4.270457143	2.094390515	1.33E-13	2.02E-12	yes	up	5.036	19.236	3.52	7.11	8.2	3.61	2.74	28.61	17.83	14.55	15.32	19.87
ENSMUSG00000085154 C130046K22Rik	RIKEN cDNA C130046K22 gene [Source:MGI Symbol;Acc:MGI:3026935]	0.479186031	-1.061342243	8.37E-07	5.81E-06	yes	down	4.88	2.81	3.89	4.1	5.92	4.45	6.04	2.33	4.99	1.58	1.94	3.21
ENSMUSG00000036887 C1qa	complement component 1, q subcomponent, alpha polypeptide [Source:MGI Symbol;Acc:MGI:88223]	5.354851165	2.420846477	5.56E-24	1.81E-22	yes	up	64.582	292.918	58.42	79.84	79.74	36.42	68.49	334.08	244.31	262.93	261.62	361.65
ENSMUSG00000036905 C1qb	complement component 1, q subcomponent, beta polypeptide [Source:MGI Symbol;Acc:MGI:88224]	7.508423635	2.908510051	1.47E-39	1.00E-37	yes	up	54.16	344.482	48.89	69.25	65.21	31.16	56.29	368.2	297.41	314.16	301.89	440.75
ENSMUSG00000036896 C1qc	complement component 1, q subcomponent, C chain [Source:MGI Symbol;Acc:MGI:88225]	6.658584064	2.735215423	1.30E-62	1.82E-60	yes	up	40.43	226.742	38.08	48.31	51.75	26.18	37.83	236.42	198.45	209.65	193.01	296.18
ENSMUSG00000046491 C1qtnf2	C1q and tumor necrosis factor related protein 2 [Source:MGI Symbol;Acc:MGI:1916433]	0.43372569	-1.205145197	3.25E-11	3.86E-10	yes	down	21.218	7.904	18.19	22.05	22.03	28.23	15.59	7.26	8.99	6.34	8.92	8.01
ENSMUSG00000058914 C1qtnf3	C1q and tumor necrosis factor related protein 3 [Source:MGI Symbol;Acc:MGI:1932136]	2.186173457	1.128407873	1.07E-06	7.27E-06	yes	up	2.44	4.54	2.32	2.63	2.68	1.71	2.86	2.4	4.6	4.55	5.08	6.07
ENSMUSG00000087461 C230014O12Rik	RIKEN cDNA C230014O12 gene [Source:MGI Symbol;Acc:MGI:3045377]	2.421065702	1.275642231	0.005492698	0.017828375	yes	up	0.274	0.568	0.3	0.28	0.18	0.15	0.46	0.23	0.69	0.53	0.81	0.58
ENSMUSG00000040552 C3ar1	complement component 3a receptor 1 [Source:MGI Symbol;Acc:MGI:1097680]	10.94118475	3.451697061	7.86E-77	1.68E-74	yes	up	1.748	16.054	1.4	1.7	2.9	1.39	1.35	16.47	13.62	14.29	15.61	20.28
ENSMUSG00000087365 C430049B03Rik	RIKEN cDNA C430049B03 gene [Source:MGI Symbol;Acc:MGI:1924903]	0.442556672	-1.176065881	0.000129599	0.000614589	yes	down	6.956	2.63	8.66	4.85	8.17	9.08	4.02	2.93	2.64	2.52	3.26	1.8
ENSMUSG00000036377 C530008M17Rik	RIKEN cDNA C530008M17 gene [Source:MGI Symbol;Acc:MGI:2444817]	2.441821812	1.287957926	0.001794082	0.006609679	yes	up	1.804	3.708	1.69	1.75	2.06	1.14	2.38	2.73	5.49	4.11	3.65	2.56
ENSMUSG00000049130 C5ar1	complement component 5a receptor 1 [Source:MGI Symbol;Acc:MGI:88232]	2.448181538	1.291710541	7.20E-16	1.33E-14	yes	up	11.862	22.488	13.16	13.92	15.03	5.17	12.03	23.11	22	20.91	25.24	21.18
ENSMUSG00000079105 C7	complement component 7 [Source:MGI Symbol;Acc:MGI:88235]	0.464649841	-1.105784182	1.14E-16	2.29E-15	yes	down	200.042	78.58	206.15	172.75	227.41	192.72	201.18	73.03	88.1	80.06	99.29	52.42

ENSMUSG00000097574	C920006O11Rik	RIKEN cDNA C920006O11 gene [Source:MGI Symbol;Acc:MGI:2443759]	0.482122379	-1.052528697	0.002714986	0.009544343	yes	down	1.93	0.784	2.09	1.78	2.09	2.39	1.3	0.64	0.87	0.86	1.19	0.36
ENSMUSG00000097352	C920009B18Rik	RIKEN cDNA C920009B18 gene [Source:MGI Symbol;Acc:MGI:3583961]	4.358917527	2.123969908	1.11E-12	1.53E-11	yes	up	0.478	1.576	0.34	0.45	0.31	0.8	0.49	1.59	1.45	1.14	0.64	3.06
ENSMUSG00000004110	Cacna1e	calcium channel, voltage-dependent, R type, alpha 1E subunit [Source:MGI Symbol;Acc:MGI:106217]	3.000186971	1.585052412	3.57E-08	3.01E-07	yes	up	0.288	3.632	0.26	0.38	0.24	0.42	0.14	1.32	0.93	2.28	13.05	0.58
ENSMUSG00000024112	Cacna1h	calcium channel, voltage-dependent, T type, alpha 1H subunit [Source:MGI Symbol;Acc:MGI:1928842]	6.465796851	2.69282818	1.01E-07	7.98E-07	yes	up	1.088	2.662	2.22	1.76	0.34	0.62	0.5	2.39	3.21	2.59	1.46	3.66
ENSMUSG00000026407	Cacna1s	calcium channel, voltage-dependent, L type, alpha 1S subunit [Source:MGI Symbol;Acc:MGI:88294]	3.004304662	1.587031122	6.23E-07	4.40E-06	yes	up	2.46	6.408	1.45	4.11	2.48	1.39	2.87	6.81	5.43	7.24	4.39	8.17
ENSMUSG00000041460	Cacna2d4	calcium channel, voltage-dependent, alpha 2/delta subunit 4 [Source:MGI Symbol;Acc:MGI:2442632]	0.275478014	-1.859990914	5.04E-16	9.43E-15	yes	down	5.46	1.296	7.2	4.59	4.08	8.04	3.39	0.92	1.99	0.83	1.64	1.1
ENSMUSG00000040373	Cacng5	calcium channel, voltage-dependent, gamma subunit 5 [Source:MGI Symbol;Acc:MGI:2157946]	14.14492941	3.822213072	0.017774188	0.049115203	yes	up	0.002	0.052	0	0	0	0	0.01	0	0.05	0.09	0.01	0.11
ENSMUSG00000064115	Cadm2	cell adhesion molecule 2 [Source:MGI Symbol;Acc:MGI:2442722]	0.266733668	-1.906528158	5.58E-08	4.59E-07	yes	down	0.738	0.34	0.38	0.8	1.1	0.56	0.85	0.85	0.4	0.2	0.08	0.17
ENSMUSG00000030666	Calcb	calcitonin-related polypeptide, beta [Source:MGI Symbol;Acc:MGI:2151254]	0.210015325	-2.251433488	0.006708245	0.021293879	yes	down	1.052	0.16	0.96	1.45	0.36	0.53	1.96	0.29	0	0.07	0.37	0.07
ENSMUSG00000046031	Calhm6	calcium homeostasis modulator family member 6 [Source:MGI Symbol;Acc:MGI:2443082]	11.34600682	3.504112731	8.66E-21	2.32E-19	yes	up	0.912	8.77	0.95	0.68	0.6	1.27	1.06	10.23	5.18	6.63	5.2	16.61
ENSMUSG00000063130	Calml3	calmodulin-like 3 [Source:MGI Symbol;Acc:MGI:1917655]	0.243020863	-2.040847923	0.013001579	0.037596788	yes	down	4.282	0.84	3.01	11.25	0.8	2	4.35	0.22	0.44	2.32	0.65	0.57
ENSMUSG00000039145	Camk1d	calcium/calmodulin-dependent protein kinase ID [Source:MGI Symbol;Acc:MGI:2442190]	2.064988396	1.046133675	2.68E-28	1.14E-26	yes	up	56.114	78.488	46.39	42.52	52.44	54.31	84.91	59.64	98.89	98.13	78.15	57.63
ENSMUSG00000056737	Capg	capping protein (actin filament), gelsolin-like [Source:MGI Symbol;Acc:MGI:1098259]	4.218202961	2.076628513	1.89E-68	3.34E-66	yes	up	60.504	226.306	54.17	70.34	69.22	48.39	60.4	186.38	227.23	219.03	219.52	279.37
ENSMUSG00000035694	Caps2	calcyphosphine 2 [Source:MGI Symbol;Acc:MGI:2441980]	0.452547306	-1.143859486	0.002273771	0.008152532	yes	down	0.952	0.378	0.77	1.12	0.53	1.41	0.93	0.44	0.51	0.33	0.35	0.26
ENSMUSG00000027555	Car13	carbonic anhydrase 13 [Source:MGI Symbol;Acc:MGI:1931322]	2.304244996	1.204294118	6.94E-12	8.88E-11	yes	up	3.964	7.704	3.46	5.09	4.05	3.23	3.99	8.42	5.87	6.78	9.36	8.09
ENSMUSG00000036526	Card11	caspase recruitment domain family, member 11 [Source:MGI Symbol;Acc:MGI:1916978]	2.600990624	1.379061199	2.46E-43	1.94E-41	yes	up	9.4	19.59	9.01	9.23	9.7	8.95	10.11	16.93	21.93	20.09	18.14	20.86
ENSMUSG00000026928	Card9	caspase recruitment domain family, member 9 [Source:MGI Symbol;Acc:MGI:2685628]	2.140107442	1.097683228	5.30E-20	1.34E-18	yes	up	8.446	12.678	8.74	7.57	11.21	7.94	6.77	12.42	13.59	13.52	11.44	12.42
ENSMUSG00000097324	Carmn	cardiac mesoderm enhancer-associated non-coding RNA [Source:MGI	0.136859995	-2.869227295	1.38E-06	9.26E-06	yes	down	1.87	0.212	2.41	1.11	1.47	1.76	2.6	0.07	0.28	0.64	0.07	0

	Symbol;Acc:MGI:4439832]																		
ENSMUSG00000025888 Casp1	caspase 1 [Source:MGI Symbol;Acc:MGI:96544]	3.411004418	1.770196623	1.00E-28	4.40E-27	yes	up	8.486	24.634	5.97	10.09	11.31	8.24	6.82	25.51	21.91	25.36	23.72	26.67
ENSMUSG00000073650 Catip	ciliogenesis associated TTC17 interacting protein [Source:MGI Symbol;Acc:MGI:2685062]	0.481626438	-1.054013506	1.82E-05	0.000101403	yes	down	5.966	3.038	5.79	6.38	3.57	5.47	8.62	3	3.48	3.32	2.82	2.57
ENSMUSG00000045954 Cavin2	caveolae associated 2 [Source:MGI Symbol;Acc:MGI:99513]	0.453842264	-1.139737129	1.76E-49	1.70E-47	yes	down	393.166	150.652	413.97	381.77	390.18	402.8	377.11	147.89	155.13	127	174.5	148.74
ENSMUSG00000031654 Cbln1	cerebellin 1 precursor protein [Source:MGI Symbol;Acc:MGI:88281]	660.6264955	9.367691022	2.35E-23	7.42E-22	yes	up	0	3.454	0	0	0	0	0	3.07	3.18	3.52	3.49	4.01
ENSMUSG00000024039 Cbs	cystathionine beta-synthase [Source:MGI Symbol;Acc:MGI:88285]	0.463801309	-1.108421202	3.70E-06	2.33E-05	yes	down	3.46	0.94	3.66	3.06	5.17	3.1	2.31	1.02	0.92	1.24	0.79	0.73
ENSMUSG00000064280 Ccdc146	coiled-coil domain containing 146 [Source:MGI Symbol;Acc:MGI:1922422]	0.4554325	-1.134690849	0.00319293	0.011007693	yes	down	8.68	2.596	9.03	6.64	14.63	5.07	8.03	2.74	1.58	4.58	1.12	2.96
ENSMUSG00000025983 Ccdc150	coiled-coil domain containing 150 [Source:MGI Symbol;Acc:MGI:1925266]	0.255082445	-1.970964482	0.002421981	0.00863191	yes	down	0.452	0.08	0.28	0.82	0.39	0.35	0.42	0.03	0.16	0.16	0	0.05
ENSMUSG00000091119 Ccdc152	coiled-coil domain containing 152 [Source:MGI Symbol;Acc:MGI:3641617]	0.39074803	-1.355689497	0.000494597	0.002075362	yes	down	3.352	1.098	3.63	4.18	3.24	2.55	3.16	0.56	0.61	2.12	1.63	0.57
ENSMUSG00000070306 Ccdc153	coiled-coil domain containing 153 [Source:MGI Symbol;Acc:MGI:2448587]	0.466410534	-1.100327721	1.20E-08	1.09E-07	yes	down	151.642	59.128	144.19	174.53	121.35	141.53	176.61	45.11	62.43	99.62	39.14	49.34
ENSMUSG00000038292 Ccdc155	coiled-coil domain containing 155 [Source:MGI Symbol;Acc:MGI:2687329]	0.449476733	-1.153681658	0.005808743	0.018726485	yes	down	0.864	0.336	0.63	0.37	1.04	1.01	1.27	0.27	0.25	0.3	0.44	0.42
ENSMUSG00000075225 Ccdc162	coiled-coil domain containing 162 [Source:MGI Symbol;Acc:MGI:1923223]	0.476841434	-1.068418495	3.39E-06	2.15E-05	yes	down	15.668	3.134	8.87	6.91	45.62	6.68	10.26	3.14	2.45	5.81	1.86	2.41
ENSMUSG00000019767 Ccdc170	coiled-coil domain containing 170 [Source:MGI Symbol;Acc:MGI:2685067]	0.439775202	-1.18516184	3.23E-06	2.06E-05	yes	down	5.642	2.006	5.82	5.76	5.84	4.17	6.62	1.75	1.89	3.28	0.77	2.34
ENSMUSG00000052407 Ccdc171	coiled-coil domain containing 171 [Source:MGI Symbol;Acc:MGI:1922152]	0.379836755	-1.396548579	3.54E-09	3.40E-08	yes	down	53.278	16.46	60.32	47.92	45.52	37.56	75.07	16.48	17.11	16.33	22.45	9.93
ENSMUSG00000035539 Ccdc180	coiled-coil domain containing 180 [Source:MGI Symbol;Acc:MGI:2685871]	0.413921621	-1.272570485	3.69E-06	2.32E-05	yes	down	4.452	1.78	5.03	3.58	3.95	4.11	5.59	1.81	1.58	3.03	0.67	1.81
ENSMUSG00000029875 Ccdc184	coiled-coil domain containing 184 [Source:MGI Symbol;Acc:MGI:2146066]	0.490084326	-1.028898087	4.00E-06	2.50E-05	yes	down	2.524	1.046	2.85	3.2	1.95	2.24	2.38	0.98	1.16	0.67	1.13	1.29
ENSMUSG00000048038 Ccdc187	coiled-coil domain containing 187 [Source:MGI Symbol;Acc:MGI:3045295]	0.46811545	-1.095063713	4.95E-09	4.68E-08	yes	down	4.592	1.816	4.84	3.96	3.97	5.34	4.85	1.61	2.05	2.63	1.01	1.78
ENSMUSG00000026676 Ccdc3	coiled-coil domain containing 3 [Source:MGI Symbol;Acc:MGI:1921436]	0.357140424	-1.485436655	4.44E-06	2.75E-05	yes	down	2.024	0.61	2.08	3.31	1.82	1.1	1.81	0.72	0.88	0.51	0.54	0.4

ENSMUSG00000045915	Ccdc42	coiled-coil domain containing 42 [Source:MGI Symbol;Acc:MGI:3045254]	0.345849562	-1.531783466	0.002338616	0.008365427	yes	down	5.152	3.488	5.45	4.5	3.04	10.81	1.96	1.67	0.19	12.71	1.75	1.12
ENSMUSG00000041617	Ccdc74a	coiled-coil domain containing 74A [Source:MGI Symbol;Acc:MGI:1919565]	0.432110619	-1.210527409	0.001083553	0.004213029	yes	down	5.8	1.78	8.47	4.62	2.66	8.49	4.76	0.79	3.37	2.79	1	0.95
ENSMUSG00000022665	Ccdc80	coiled-coil domain containing 80 [Source:MGI Symbol;Acc:MGI:1915146]	2.547508161	1.349086768	2.53E-06	1.63E-05	yes	up	14.844	30.996	10.25	18.98	21.56	8.32	15.11	20.74	33.36	40.18	28.56	32.14
ENSMUSG00000039391	Ccdc81	coiled-coil domain containing 81 [Source:MGI Symbol;Acc:MGI:1918134]	0.494516999	-1.015907982	7.96E-05	0.000393608	yes	down	4.072	1.722	4.6	4.53	2.82	4.91	3.5	0.98	2.06	2.26	1.06	2.25
ENSMUSG00000032878	Ccdc85a	coiled-coil domain containing 85A [Source:MGI Symbol;Acc:MGI:2445069]	0.456177112	-1.13233403	3.61E-06	2.28E-05	yes	down	23.178	8.942	30.23	16.38	25.77	15.36	28.15	9.71	10.19	7.13	10.16	7.52
ENSMUSG00000047810	Ccdc88b	coiled-coil domain containing 88B [Source:MGI Symbol;Acc:MGI:1925567]	2.555116854	1.353389272	1.20E-31	6.00E-30	yes	up	5.542	13.55	5.76	4.79	5.2	7.19	4.77	10.51	18.99	10.46	12.51	15.28
ENSMUSG00000029193	Cckar	cholecystokinin A receptor [Source:MGI Symbol;Acc:MGI:99478]	0.453743142	-1.140052256	2.61E-14	4.22E-13	yes	down	40.698	15.322	42.98	27.28	36.73	56.61	39.89	18.66	13.46	22.47	8.83	13.19
ENSMUSG00000020702	Ccl1	chemokine (C-C motif) ligand 1 [Source:MGI Symbol;Acc:MGI:98258]	16.91640206	4.08035085	0.013470085	0.038716324	yes	up	0.104	1.442	0.52	0	0	0	0	2.59	0	0	1.32	3.3
ENSMUSG00000035352	Ccl12	chemokine (C-C motif) ligand 12 [Source:MGI Symbol;Acc:MGI:108224]	3.72449201	1.897043667	1.76E-08	1.55E-07	yes	up	5.04	15.872	5.64	6.42	3.88	5.35	3.91	22.67	8.56	17.63	10.13	20.37
ENSMUSG00000031780	Ccl17	chemokine (C-C motif) ligand 17 [Source:MGI Symbol;Acc:MGI:1329039]	0.433904969	-1.204548986	0.000155711	0.000726831	yes	down	23.022	8.478	23.27	28.05	18.69	17.22	27.88	4.02	7.59	10.8	5.74	14.24
ENSMUSG00000071005	Ccl19	chemokine (C-C motif) ligand 19 [Source:MGI Symbol;Acc:MGI:1346316]	2.91838722	1.545171317	7.48E-05	0.000371294	yes	up	5.612	13.048	2.26	6.65	9.89	4.17	5.09	16.42	8.95	11.38	6.41	22.08
ENSMUSG00000035385	Ccl2	chemokine (C-C motif) ligand 2 [Source:MGI Symbol;Acc:MGI:98259]	3.667722532	1.874884501	8.99E-05	0.000439804	yes	up	1.082	3.314	0.67	1.63	0.69	0.59	1.83	3.69	2.24	3.37	1.7	5.57
ENSMUSG00000026166	Ccl20	chemokine (C-C motif) ligand 20 [Source:MGI Symbol;Acc:MGI:1329031]	11.38968286	3.509655671	5.63E-07	4.00E-06	yes	up	1.026	9.598	0.47	1.7	0.84	0.3	1.82	10.43	3.81	24.21	5.04	4.5
ENSMUSG00000004814	Ccl24	chemokine (C-C motif) ligand 24 [Source:MGI Symbol;Acc:MGI:1928953]	0.2030223	-2.300289891	0.005634742	0.018247942	yes	down	1.772	0.302	0.86	2.67	3.54	0.94	0.85	0.55	0.09	0.5	0.37	0
ENSMUSG00000000982	Ccl3	chemokine (C-C motif) ligand 3 [Source:MGI Symbol;Acc:MGI:98260]	7.747511176	2.953732931	1.23E-06	8.32E-06	yes	up	2.458	16.104	2.22	7.03	0.38	1.18	1.48	19.04	17.53	13.37	15.23	15.35
ENSMUSG00000035042	Ccl5	chemokine (C-C motif) ligand 5 [Source:MGI Symbol;Acc:MGI:98262]	5.119695304	2.356057951	3.12E-18	7.01E-17	yes	up	136.938	593.68	157.67	121.11	122.28	176.2	107.43	691.81	475.81	423.16	420.1	957.52
ENSMUSG00000018927	Ccl6	chemokine (C-C motif) ligand 6 [Source:MGI Symbol;Acc:MGI:98263]	6.014237651	2.588381877	1.37E-23	4.34E-22	yes	up	238.398	1202.03	252.28	274.74	284.73	112.98	267.26	1156.95	1248.31	1048.4	1431.1	1125.39
ENSMUSG00000009185	Ccl8	chemokine (C-C motif) ligand 8 [Source:MGI Symbol;Acc:MGI:101878]	13.31249909	3.734709521	2.08E-15	3.72E-14	yes	up	13.416	151.908	13.1	13.25	20.29	3.44	17	209.68	94.36	144.1	87.91	223.49
ENSMUSG00000019122	Ccl9	chemokine (C-C motif) ligand 9 [Source:MGI Symbol;Acc:MGI:104533]	3.306678141	1.725382626	8.95E-26	3.24E-24	yes	up	23.67	67.098	21.39	28.88	17.25	19.82	31.01	60.12	66.68	89.08	58.55	61.06
ENSMUSG00000028195	Ccn1	cellular communication network factor 1 [Source:MGI	0.270773009	-1.884844157	5.36E-13	7.63E-12	yes	down	230.488	51.22	251.61	363.65	136.56	227.45	173.17	58.3	49.2	40.1	51.84	56.66

	Symbol;Acc:MGI:88613]																			
ENSMUSG00000027715 Ccna2	cyclin A2 [Source:MGI Symbol;Acc:MGI:108069]	2.724602149	1.446045581	2.13E-11	2.58E-10	yes	up	3.314	7.354	2.87	2.76	4.13	3.04	3.77	9.69	6.48	6.58	4.91	9.11
ENSMUSG00000041431 Ccnb1	cyclin B1 [Source:MGI Symbol;Acc:MGI:88302]	3.217499718	1.685940022	7.15E-12	9.12E-11	yes	up	1.706	4.712	1.53	1.34	1.84	2.23	1.59	5.73	2.87	3.54	3.24	8.18
ENSMUSG00000032218 Ccnb2	cyclin B2 [Source:MGI Symbol;Acc:MGI:88311]	2.454000036	1.29513527	1.02E-05	5.93E-05	yes	up	2.828	5.85	3.18	1.25	4.13	2.55	3.03	5.86	5.19	4.45	4.36	9.39
ENSMUSG00000002068 Ccne1	cyclin E1 [Source:MGI Symbol;Acc:MGI:88316]	3.299042916	1.722047545	6.77E-08	5.49E-07	yes	up	1.008	2.152	1.02	0.96	1.8	0.45	0.81	2.96	2.48	1.65	1.35	2.32
ENSMUSG00000072082 Ccnf	cyclin F [Source:MGI Symbol;Acc:MGI:102551]	3.343179271	1.741220718	5.56E-10	5.81E-09	yes	up	1.048	2.012	1.3	0.73	1.93	0.59	0.69	2.48	1.75	1.79	1.28	2.76
ENSMUSG00000025804 Ccr1	chemokine (C-C motif) receptor 1 [Source:MGI Symbol;Acc:MGI:104618]	4.90129793	2.293163845	2.04E-93	6.14E-91	yes	up	5.772	23.922	5.19	7.14	5.43	4.96	6.14	24.34	25.98	22.04	24.41	22.84
ENSMUSG00000044052 Ccr10	chemokine (C-C motif) receptor 10 [Source:MGI Symbol;Acc:MGI:1096320]	6.415929553	2.681658301	3.03E-19	7.35E-18	yes	up	0.682	3.696	0.94	0.21	0.92	0.68	0.66	3.28	3.31	4.51	3.01	4.37
ENSMUSG00000049103 Ccr2	chemokine (C-C motif) receptor 2 [Source:MGI Symbol;Acc:MGI:106185]	2.290781986	1.195840164	5.53E-21	1.49E-19	yes	up	13.528	26.354	10.96	15.29	15.65	14.93	10.81	32.64	23.69	28.51	23.3	23.63
ENSMUSG00000047898 Ccr4	chemokine (C-C motif) receptor 4 [Source:MGI Symbol;Acc:MGI:107824]	3.482484542	1.800116949	2.74E-05	0.000147664	yes	up	0.316	0.936	0.45	0.1	0.17	0.26	0.6	0.95	0.98	0.97	0.57	1.21
ENSMUSG00000079227 Ccr5	chemokine (C-C motif) receptor 5 [Source:MGI Symbol;Acc:MGI:107182]	8.322725624	3.057056076	1.07E-92	3.13E-90	yes	up	2.884	19.752	2.28	3.67	4.05	1.95	2.47	18.76	19.28	17.73	18.55	24.44
ENSMUSG00000040899 Ccr6	chemokine (C-C motif) receptor 6 [Source:MGI Symbol;Acc:MGI:1333797]	2.304772385	1.20462428	7.35E-07	5.14E-06	yes	up	4.676	8.214	2.25	5.23	4.56	6.87	4.47	8.1	3.5	10.87	9.47	9.13
ENSMUSG00000042262 Ccr8	chemokine (C-C motif) receptor 8 [Source:MGI Symbol;Acc:MGI:1201402]	4.045925798	2.016469861	0.000159676	0.000743398	yes	up	0.648	2.184	0.4	1.1	1.23	0.06	0.45	2.61	1.55	2.2	1.22	3.34
ENSMUSG00000051439 Cd14	CD14 antigen [Source:MGI Symbol;Acc:MGI:88318]	3.039498587	1.603833348	2.68E-12	3.58E-11	yes	up	46.62	118.144	45.45	69.67	38.89	28.38	50.71	120.63	110.41	133.15	112.45	114.08
ENSMUSG00000008845 Cd163	CD163 antigen [Source:MGI Symbol;Acc:MGI:2135946]	0.441790126	-1.178566919	0.016199781	0.045416221	yes	down	5.378	2.02	4.56	4.69	6.69	2.79	8.16	1.6	2.12	4.16	1.3	0.92
ENSMUSG00000025461 Cd163l1	CD163 molecule-like 1 [Source:MGI Symbol;Acc:MGI:2443796]	2.259823029	1.176209797	4.70E-05	0.000241322	yes	up	1.304	2.406	1.4	1	1.12	1.63	1.37	2.88	1.46	2.44	1.38	3.87
ENSMUSG00000052212 Cd177	CD177 antigen [Source:MGI Symbol;Acc:MGI:1916141]	13.34647809	3.738387184	8.44E-08	6.76E-07	yes	up	1.948	21.858	1.09	3.05	1.12	2.61	1.87	12.47	9.92	66.58	4.56	15.76
ENSMUSG00000021624 Cd180	CD180 antigen [Source:MGI Symbol;Acc:MGI:1194924]	4.490375741	2.16683617	2.90E-40	2.02E-38	yes	up	5.248	18.478	4.48	6.57	5.35	6.52	3.32	20.04	13.7	18.07	17.54	23.04
ENSMUSG00000028076 Cd1d1	CD1d1 antigen [Source:MGI Symbol;Acc:MGI:107674]	2.271786147	1.183827034	3.01E-23	9.44E-22	yes	up	12.436	23.658	14.83	11.01	12.43	10.47	13.44	20.45	23.52	21.36	21.49	31.47
ENSMUSG00000022667 Cd200r1	CD200 receptor 1 [Source:MGI Symbol;Acc:MGI:1889024]	11.26728248	3.494067694	1.25E-109	5.56E-107	yes	up	3.996	38.438	4.2	3.19	5.85	2.42	4.32	34.43	40.72	35.96	43.59	37.49
ENSMUSG00000090176 Cd200r2	Cd200 receptor 2 [Source:MGI Symbol;Acc:MGI:3042847]	5.936712997	2.569664369	0.003989489	0.013416911	yes	up	0.098	0.484	0	0.25	0.21	0	0.03	0.2	0.75	0.12	0.88	0.47
ENSMUSG00000036172 Cd200r3	CD200 receptor 3 [Source:MGI Symbol;Acc:MGI:1921853]	2.120957782	1.084715904	2.83E-08	2.43E-07	yes	up	4.2	7.692	5.27	3.73	3.03	3.98	4.99	9.03	7.19	6.18	7.5	8.56
ENSMUSG00000062082 Cd200r4	CD200 receptor 4 [Source:MGI Symbol;Acc:MGI:3036289]	12.39022106	3.631130023	6.48E-72	1.25E-69	yes	up	4.326	45.294	3.37	5.88	6.17	2.17	4.04	41.01	45.75	40.98	51.27	47.46
ENSMUSG00000065987 Cd209b	CD209b antigen [Source:MGI Symbol;Acc:MGI:1916415]	0.132245694	-2.918707351	1.67E-05	9.34E-05	yes	down	1.066	0.09	0.62	1.18	1.11	1.22	1.2	0.06	0.16	0.23	0	0

ENSMUSG00000030577 Cd22	CD22 antigen [Source:MGI Symbol;Acc:MGI:88322]	4.243283974	2.085181232	2.44E-63	3.58E-61	yes	up	12.282	39.486	14.53	13.01	14.45	13.69	5.73	38.78	39.55	37.2	38.55	43.35
ENSMUSG00000016496 Cd274	CD274 antigen [Source:MGI Symbol;Acc:MGI:1926446]	5.124561967	2.357428693	8.18E-99	2.78E-96	yes	up	11.174	48.342	10.17	11.43	13.49	9.93	10.85	45.41	46.2	40.49	53.1	56.51
ENSMUSG00000035914 Cd276	CD276 antigen [Source:MGI Symbol;Acc:MGI:2183926]	2.355197193	1.235847857	3.13E-11	3.72E-10	yes	up	2.564	4.276	4.11	2.17	1.85	2.02	2.67	3.51	4.19	4.25	4.53	4.9
ENSMUSG00000034652 Cd300a	CD300A molecule [Source:MGI Symbol;Acc:MGI:2443411]	4.241403411	2.084541708	1.38E-64	2.10E-62	yes	up	4.698	20.918	4.22	5.35	3.94	6.27	3.71	23.61	21.03	16.65	23.73	19.57
ENSMUSG00000044811 Cd300c2	CD300C molecule 2 [Source:MGI Symbol;Acc:MGI:2153249]	5.766317568	2.527650293	4.95E-105	2.04E-102	yes	up	17.718	87.134	16.71	17.89	20.82	13.75	19.42	83.37	85.08	77.81	93.66	95.75
ENSMUSG00000048498 Cd300e	CD300E molecule [Source:MGI Symbol;Acc:MGI:2387602]	0.451908914	-1.145896079	6.29E-05	0.000316569	yes	down	3.18	1.212	3.12	3.71	2.58	4.55	1.94	1.22	1.41	0.54	1.43	1.46
ENSMUSG00000063193 Cd300lb	CD300 molecule like family member B [Source:MGI Symbol;Acc:MGI:2685099]	6.448262591	2.688910496	2.99E-50	2.94E-48	yes	up	2.812	16.084	2.71	3.95	2.46	3.27	1.67	15	14.94	12.01	23.9	14.57
ENSMUSG00000034641 Cd300ld	CD300 molecule like family member d [Source:MGI Symbol;Acc:MGI:2442358]	2.094976701	1.066934199	3.50E-13	5.08E-12	yes	up	5.67	10.01	5.45	6.71	5.3	5.56	5.33	12.77	9.15	8.15	11.06	8.92
ENSMUSG00000069607 Cd300ld3	CD300 molecule like family member D3 [Source:MGI Symbol;Acc:MGI:2687214]	2.401123245	1.263709456	1.67E-20	4.39E-19	yes	up	9.246	19.07	6.36	9.02	13.37	8.88	8.6	15.72	18.61	17.9	19.99	23.13
ENSMUSG00000069609 Cd300ld4	CD300 molecule like family member D4 [Source:MGI Symbol;Acc:MGI:3702658]	4.603661268	2.202781685	0.000157953	0.000736173	yes	up	0.376	2.294	0.42	0.18	0.23	0.31	0.74	2.86	0.85	2.37	3.2	2.19
ENSMUSG00000089722 Cd300ld5	CD300 molecule like family member D5 [Source:MGI Symbol;Acc:MGI:3702661]	2.654709634	1.408554071	4.69E-28	1.96E-26	yes	up	3.932	8.87	4.7	4.27	3.42	3.61	3.66	10.2	7.49	9.46	9.03	8.17
ENSMUSG00000047798 Cd300lf	CD300 molecule like family member F [Source:MGI Symbol;Acc:MGI:2442359]	4.181876529	2.064150467	2.17E-68	3.81E-66	yes	up	7.196	26.3	8.08	8.43	7.17	6.13	6.17	28.04	26.92	23.24	23.76	29.54
ENSMUSG00000017309 Cd300lg	CD300 molecule like family member G [Source:MGI Symbol;Acc:MGI:1289168]	0.365975263	-1.450181957	1.19E-22	3.57E-21	yes	down	12.482	3.622	13.58	11.99	12.7	14.96	9.18	3.32	3.88	3.25	4.97	2.69
ENSMUSG00000004609 Cd33	CD33 antigen [Source:MGI Symbol;Acc:MGI:99440]	3.184198179	1.670930129	1.54E-48	1.45E-46	yes	up	7.372	19.31	6.09	8.73	7.44	7.92	6.68	21.92	21.82	13.81	21.73	17.27
ENSMUSG00000030798 Cd37	CD37 antigen [Source:MGI Symbol;Acc:MGI:88330]	2.461636754	1.29961789	5.39E-23	1.66E-21	yes	up	27.776	58.408	23.4	35.28	26.88	32.99	20.33	59.51	61.16	50.11	58.76	62.5
ENSMUSG00000032093 Cd3e	CD3 antigen, epsilon polypeptide [Source:MGI Symbol;Acc:MGI:88332]	2.351275658	1.233443687	8.55E-20	2.14E-18	yes	up	7.7	14.46	7.44	8.09	7.57	9.68	5.72	17.31	13.41	14.2	11.96	15.42
ENSMUSG00000002033 Cd3g	CD3 antigen, gamma polypeptide [Source:MGI Symbol;Acc:MGI:88333]	2.347096591	1.230877215	4.41E-17	9.17E-16	yes	up	20.608	40.916	19.58	22.38	23.33	21.75	16	47.88	32.13	37	39.24	48.33
ENSMUSG00000023274 Cd4	CD4 antigen [Source:MGI Symbol;Acc:MGI:88335]	3.434183914	1.779967303	3.58E-21	9.76E-20	yes	up	5.3	15.464	4.21	6.44	6.43	4.72	4.7	22.72	13.98	16.96	9.08	14.58
ENSMUSG00000005087 Cd44	CD44 antigen [Source:MGI Symbol;Acc:MGI:88338]	2.407583521	1.267585847	1.01E-39	6.90E-38	yes	up	59.31	126.536	59.67	64.56	61.1	48.47	62.75	107.48	140.37	122.74	129.87	132.22
ENSMUSG00000015355 Cd48	CD48 antigen [Source:MGI Symbol;Acc:MGI:88339]	3.801571897	1.926596076	2.99E-53	3.24E-51	yes	up	20.046	65.346	16.06	24.69	24.56	19.13	15.79	68.08	63.03	58.94	66.69	69.99
ENSMUSG00000024669 Cd5	CD5 antigen [Source:MGI Symbol;Acc:MGI:88340]	2.542157471	1.346053399	3.67E-13	5.31E-12	yes	up	3.434	7.256	3.95	2.73	3.54	3.69	3.26	8.43	5.26	8.14	6.55	7.9
ENSMUSG00000000682 Cd52	CD52 antigen [Source:MGI Symbol;Acc:MGI:1346088]	4.112655706	2.0400703	2.24E-57	2.76E-55	yes	up	221.128	770.474	211.83	256.82	238.94	217.58	180.47	910.64	642.58	708.16	662.8	928.19
ENSMUSG00000040747 Cd53	CD53 antigen [Source:MGI Symbol;Acc:MGI:88341]	2.980476809	1.575543148	1.67E-57	2.08E-55	yes	up	54.754	137.5	53.67	58.59	62.42	52.22	46.87	158.77	127.37	122.77	143.07	135.52

ENSMUSG00000024670 Cd6	CD6 antigen [Source:MGI Symbol;Acc:MGI:103566]	2.665403924	1.41435418	5.76E-12	7.44E-11	yes	up	2.66	6.212	2.6	2.1	3.28	2.24	3.08	7.91	4.04	6.45	5.66	7
ENSMUSG00000025351 Cd63	CD63 antigen [Source:MGI Symbol;Acc:MGI:99529]	2.592286496	1.374225172	3.45E-46	3.05E-44	yes	up	247.716	545.246	222.07	285.73	258.74	211.92	260.12	500.34	535.7	516.97	600.86	572.36
ENSMUSG00000018774 Cd68	CD68 antigen [Source:MGI Symbol;Acc:MGI:88342]	9.722487144	3.281325422	2.99E-113	1.45E-110	yes	up	47.828	376.102	39.77	51.71	57.02	34.47	56.17	336.9	405.2	326.56	391.95	419.9
ENSMUSG00000028459 Cd72	CD72 antigen [Source:MGI Symbol;Acc:MGI:88345]	2.829684527	1.50064122	1.80E-14	2.95E-13	yes	up	13.144	30.408	9.81	12.61	19.82	15.04	8.44	40.4	22.32	36.81	16.84	35.67
ENSMUSG00000024610 Cd74	CD74 antigen (invariant polypeptide of major histocompatibility complex, class II antigen-associated) [Source:MGI Symbol;Acc:MGI:96534]	3.464455324	1.792628552	6.94E-43	5.37E-41	yes	up	1652.384	4839.83	1468.61	1705.73	1868.25	1359.73	1859.6	4557.48	4397.25	4669.84	4114.59	6459.99
ENSMUSG00000075122 Cd80	CD80 antigen [Source:MGI Symbol;Acc:MGI:101775]	2.65456176	1.408473707	1.19E-11	1.48E-10	yes	up	1.77	4.608	1.66	2.05	1.86	1.3	1.98	5.74	4.21	4.55	4.48	4.06
ENSMUSG00000038147 Cd84	CD84 antigen [Source:MGI Symbol;Acc:MGI:1336885]	7.285861205	2.865099512	2.30E-104	8.95E-102	yes	up	18.674	118.382	18.45	22.72	21.77	13.44	16.99	108.05	126.11	112.24	126.63	118.88
ENSMUSG00000022901 Cd86	CD86 antigen [Source:MGI Symbol;Acc:MGI:101773]	3.301750011	1.723230892	7.73E-24	2.49E-22	yes	up	5.056	15.118	5.66	5.25	6.5	3.19	4.68	18.21	16.55	12.31	12	16.52
ENSMUSG00000006398 Cdc20	cell division cycle 20 [Source:MGI Symbol;Acc:MGI:1859866]	2.805859009	1.488442517	2.93E-12	3.90E-11	yes	up	4.504	10.728	2.7	4.75	5.05	4.42	5.6	10.09	10.65	10.24	7.42	15.24
ENSMUSG00000044201 Cdc25c	cell division cycle 25C [Source:MGI Symbol;Acc:MGI:88350]	3.573232313	1.837229713	0.000426213	0.001811786	yes	up	0.24	1.036	0.21	0.37	0.44	0.09	0.09	1.1	1	2.07	0.4	0.61
ENSMUSG00000024769 Cdc42bpg	CDC42 binding protein kinase gamma (DMPK-like) [Source:MGI Symbol;Acc:MGI:2652845]	0.498321709	-1.004850669	3.54E-29	1.57E-27	yes	down	74.926	31.528	83.18	62.4	70.12	82.74	76.19	29.18	32.29	31.36	35.74	29.07
ENSMUSG00000017499 Cdc6	cell division cycle 6 [Source:MGI Symbol;Acc:MGI:1345150]	2.354570973	1.23546421	4.87E-06	2.99E-05	yes	up	0.726	1.184	0.78	0.46	0.85	0.43	1.11	1.33	0.87	1.17	0.96	1.59
ENSMUSG00000048922 Cdca2	cell division cycle associated 2 [Source:MGI Symbol;Acc:MGI:1919787]	2.887667826	1.529904796	3.35E-07	2.46E-06	yes	up	1.022	2.262	1.92	0.68	0.89	0.8	0.82	1.87	1.74	2.71	1.08	3.91
ENSMUSG00000023505 Cdca3	cell division cycle associated 3 [Source:MGI Symbol;Acc:MGI:1315198]	2.34381252	1.228857174	2.20E-06	1.44E-05	yes	up	2.448	4.762	1.37	2.16	2.74	2.58	3.39	4.84	4.75	3.47	3.67	7.08
ENSMUSG00000024791 Cdca5	cell division cycle associated 5 [Source:MGI Symbol;Acc:MGI:1915099]	3.532097581	1.820525201	4.65E-06	2.87E-05	yes	up	0.688	1.984	0.27	1.01	0.68	0.91	0.57	2.86	1.74	1.12	1.11	3.09
ENSMUSG00000028873 Cdca8	cell division cycle associated 8 [Source:MGI Symbol;Acc:MGI:1196274]	2.692274621	1.428825577	2.03E-08	1.78E-07	yes	up	3.394	7.426	4.07	4.07	2.62	1.6	4.61	9.53	7.33	5.86	5.2	9.21
ENSMUSG00000031841 Cdh13	cadherin 13 [Source:MGI Symbol;Acc:MGI:99551]	0.415274226	-1.267863761	2.17E-13	3.23E-12	yes	down	17.53	6.322	14.08	18.27	17.26	15.62	22.42	3.67	6.13	8.1	7.12	6.59
ENSMUSG00000028217 Cdh17	cadherin 17 [Source:MGI Symbol;Acc:MGI:1095414]	5.224814741	2.385379884	3.96E-06	2.48E-05	yes	up	0.146	0.632	0.11	0.29	0.19	0.11	0.03	1.23	0.43	0.36	0.5	0.64
ENSMUSG00000047216 Cdh19	cadherin 19, type 2 [Source:MGI Symbol;Acc:MGI:3588198]	0.229032775	-2.126374029	6.29E-05	0.00031688	yes	down	0.536	0.2	0.21	1.17	0.58	0.42	0.3	0.22	0.49	0.16	0.05	0.08
ENSMUSG00000039155 Cdh26	cadherin-like 26 [Source:MGI Symbol;Acc:MGI:2685856]	0.105958641	-3.238426844	0.013563809	0.038938677	yes	down	0.66	0.06	0.16	2.47	0.09	0.2	0.38	0	0.09	0.19	0	0.02
ENSMUSG00000021803 Cdhr1	cadherin-related family member 1 [Source:MGI Symbol;Acc:MGI:2157782]	4.366457578	2.126463324	0.00233575	0.008357963	yes	up	0.082	6.582	0.07	0.19	0.03	0.03	0.09	0.29	31.58	0.59	0.04	0.41
ENSMUSG00000019942 Cdk1	cyclin-dependent kinase 1 [Source:MGI Symbol;Acc:MGI:88351]	2.534037541	1.341437898	3.56E-10	3.80E-09	yes	up	7.968	19.956	9.18	3.46	12.41	5.41	9.38	20.3	17.62	15.4	16.53	29.93
ENSMUSG00000048895 Cdk5r1	cyclin-dependent kinase 5, regulatory subunit 1 (p35) [Source:MGI Symbol;Acc:MGI:101764]	2.984701958	1.577586876	0.000266796	0.001189118	yes	up	0.24	0.394	0.14	0.17	0.19	0.66	0.04	0.48	0.4	0.25	0.35	0.49
ENSMUSG00000023067 Cdkn1a	cyclin-dependent kinase inhibitor 1A (P21) [Source:MGI Symbol;Acc:MGI:104556]	0.371699384	-1.427791797	5.02E-42	3.75E-40	yes	down	214.096	69.47	227.16	235.81	161.1	215.27	231.14	65.99	77.86	62.54	73.6	67.36
ENSMUSG00000037664 Cdkn1c	cyclin-dependent kinase inhibitor 1C (P57) [Source:MGI	0.492119804	-1.02291852	1.84E-25	6.55E-24	yes	down	43.816	16.732	51.03	38.49	36.53	51.64	41.39	14.95	16.95	16.32	17.29	18.15

	Symbol;Acc:MGI:104564]																		
ENSMUSG00000044303 Cdkn2a	cyclin dependent kinase inhibitor 2A [Source:MGI Symbol;Acc:MGI:104738]	3.733817764	1.900651516	2.41E-06	1.56E-05	yes	up	1.154	3.714	2.12	0.63	0.94	0.79	1.29	3.58	4.29	4.68	1.96	4.06
ENSMUSG00000054169 Ceacam10	carcinoembryonic antigen-related cell adhesion molecule 10 [Source:MGI Symbol;Acc:MGI:1347248]	7.286183945	2.865163417	0.001142344	0.00441198	yes	up	0.264	1.402	0	0.17	0.34	0.27	0.54	0.76	0.18	4.57	0.74	0.76
ENSMUSG00000049848 Ceacam19	carcinoembryonic antigen-related cell adhesion molecule 19 [Source:MGI Symbol;Acc:MGI:2443001]	7.327506359	2.873322315	0.000323957	0.001415018	yes	up	0.03	0.18	0	0.02	0.06	0.02	0.05	0.14	0.18	0.12	0.14	0.32
ENSMUSG00000071637 Cebpd	CCAAT/enhancer binding protein (C/EBP), delta [Source:MGI Symbol;Acc:MGI:103573]	0.365326505	-1.452741668	5.68E-08	4.66E-07	yes	down	80.986	25.294	58.69	95.18	57.85	105.29	87.92	31.6	24.73	34.68	14.65	20.81
ENSMUSG00000023031 Cela1	chymotrypsin-like elastase family, member 1 [Source:MGI Symbol;Acc:MGI:95314]	2.17297284	1.119670142	0.001525906	0.005714075	yes	up	2.758	5.302	1.34	2.51	3.32	5.3	1.32	4.01	7.91	6.37	3.5	4.72
ENSMUSG00000023473 Celsr3	cadherin, EGF LAG seven-pass G-type receptor 3 [Source:MGI Symbol;Acc:MGI:1858236]	4.225365935	2.07907629	6.73E-07	4.74E-06	yes	up	0.084	0.258	0.16	0.13	0.03	0.05	0.05	0.25	0.19	0.3	0.41	0.14
ENSMUSG00000045328 Cenpe	centromere protein E [Source:MGI Symbol;Acc:MGI:1098230]	2.903518509	1.537802231	3.62E-09	3.48E-08	yes	up	0.93	2.118	1.27	0.96	1.25	0.44	0.73	2.03	2.91	2.9	1.06	1.69
ENSMUSG00000026605 Cenpf	centromere protein F [Source:MGI Symbol;Acc:MGI:1313302]	3.024159959	1.596534451	3.78E-13	5.47E-12	yes	up	0.756	2.222	0.81	0.46	1.29	0.46	0.76	3.26	2.36	1.35	2.08	2.06
ENSMUSG00000021714 Cenpk	centromere protein K [Source:MGI Symbol;Acc:MGI:1926210]	2.034177206	1.024445364	0.008386278	0.025826938	yes	up	3.618	4.492	2.09	6.7	1.93	4.54	2.83	6.34	3.98	1.93	2.92	7.29
ENSMUSG00000068101 Cenpm	centromere protein M [Source:MGI Symbol;Acc:MGI:1913820]	2.073686904	1.052198085	0.009304282	0.028223867	yes	up	1.174	3.868	0.9	0.93	1.37	2.04	0.63	7.59	2.05	2.34	1.08	6.28
ENSMUSG00000024989 Cep55	centrosomal protein 55 [Source:MGI Symbol;Acc:MGI:1921357]	3.659630119	1.871697842	7.14E-12	9.11E-11	yes	up	1.416	3.638	1.44	0.8	2.59	1.01	1.24	4.88	2.57	3.37	2.39	4.98
ENSMUSG00000075256 Cerkl	ceramide kinase-like [Source:MGI Symbol;Acc:MGI:3037816]	3.236849079	1.6945901	0.003410121	0.011658868	yes	up	2.804	2.566	0.17	0	0.56	0.35	12.94	2.42	3.37	1.47	5.12	0.45
ENSMUSG00000078964 Ces1b	carboxylesterase 1B [Source:MGI Symbol;Acc:MGI:3779470]	0.417317722	-1.260781905	0.010173375	0.030461168	yes	down	1.892	8.204	0.64	2.05	2.36	2.61	1.8	0.38	0.05	38.83	0.68	1.08
ENSMUSG00000061959 Ces1e	carboxylesterase 1E [Source:MGI Symbol;Acc:MGI:95432]	0.429928889	-1.217830041	2.12E-15	3.79E-14	yes	down	8.322	3.218	8.83	7.79	7.28	9.36	8.35	3.65	2.68	3.5	2.76	3.5
ENSMUSG00000031725 Ces1f	carboxylesterase 1F [Source:MGI Symbol;Acc:MGI:2142687]	0.235979755	-2.083264998	6.12E-12	7.88E-11	yes	down	11.526	1.998	11.14	16.51	9.71	9.92	10.35	1.68	1.37	4.2	1.41	1.33
ENSMUSG00000057074 Ces1g	carboxylesterase 1G [Source:MGI Symbol;Acc:MGI:88378]	0.258158107	-1.953673193	6.58E-27	2.56E-25	yes	down	8.778	1.906	9.63	8.74	6.18	12.01	7.33	1.75	1.89	1.68	1.99	2.22
ENSMUSG00000061825 Ces2c	carboxylesterase 2C [Source:MGI Symbol;Acc:MGI:2385905]	3.32618985	1.733870516	6.17E-08	5.04E-07	yes	up	0.548	1.526	0.81	0.4	0.72	0.36	0.45	1.26	1.99	1.52	1.51	1.35
ENSMUSG00000011154 Cfap161	cilia and flagella associated protein 161 [Source:MGI Symbol;Acc:MGI:1922806]	0.425741473	-1.231950459	1.59E-07	1.23E-06	yes	down	13.756	4.962	15.77	14.22	9.62	12.73	16.44	5.13	4.68	8.1	3.36	3.54
ENSMUSG00000028294 Cfap206	cilia and flagella associated protein 206 [Source:MGI Symbol;Acc:MGI:1916579]	0.486978115	-1.038071156	8.70E-07	6.01E-06	yes	down	15.344	6.262	15.88	16.02	11.78	14.64	18.4	4.48	5.6	10.56	4.46	6.21
ENSMUSG00000036962 Cfap221	cilia and flagella associated protein 221 [Source:MGI Symbol;Acc:MGI:2684947]	0.491770496	-1.023942912	0.007795084	0.024250365	yes	down	2.722	0.706	2.52	3.93	1.69	2.35	3.12	0.83	0.59	1.28	0.22	0.61
ENSMUSG00000071550 Cfap44	cilia and flagella associated protein 44 [Source:MGI	0.496279222	-1.010776041	0.000665919	0.002709218	yes	down	9.116	4.878	9.88	8.35	5.75	9.7	11.9	4.77	3.99	8.26	1.84	5.53

		Symbol;Acc:MGI:1277238]																		
ENSMUSG00000020014	Cfap54	cilia and flagella associated protein 54 [Source:MGI Symbol;Acc:MGI:1922208]	0.496735578	-1.009450014	3.20E-05	0.000169455	yes	down	3.412	1.28	2.78	3.42	2.07	4.39	4.4	1.29	1.06	2.37	0.62	1.06
ENSMUSG00000046585	Cfap58	cilia and flagella associated protein 58 [Source:MGI Symbol;Acc:MGI:2685815]	0.482214115	-1.052254215	2.89E-05	0.000155033	yes	down	4.992	2.062	5.77	4.02	5.23	3.91	6.03	1.48	1.52	3.35	2.05	1.91
ENSMUSG00000094282	Cfap73	cilia and flagella associated protein 73 [Source:MGI Symbol;Acc:MGI:3779542]	0.445118049	-1.167740095	0.007543705	0.023574492	yes	down	3.222	1.212	2.05	3.46	3.05	3.56	3.99	1.41	1.45	2.22	0.39	0.59
ENSMUSG00000090231	Cfb	complement factor B [Source:MGI Symbol;Acc:MGI:105975]	8.800960539	3.137660988	2.09E-95	6.53E-93	yes	up	29.226	226.768	29.4	27.58	32.68	24.59	31.88	196.39	205.92	210.21	170.64	350.68
ENSMUSG00000001128	Cfp	complement factor properdin [Source:MGI Symbol;Acc:MGI:97545]	2.409496198	1.268731524	2.90E-25	1.02E-23	yes	up	11.802	24.852	10.06	12.53	13.87	11.74	10.81	26.15	21.61	24.82	25.37	26.31
ENSMUSG00000032344	Cgas	cyclic GMP-AMP synthase [Source:MGI Symbol;Acc:MGI:2442261]	2.131115199	1.091608581	3.53E-08	2.99E-07	yes	up	1.066	1.894	1.19	0.97	1.3	0.9	0.97	2.38	1.9	1.81	1.5	1.88
ENSMUSG00000050370	Ch25h	cholesterol 25-hydroxylase [Source:MGI Symbol;Acc:MGI:1333869]	5.146823245	2.363682237	9.91E-32	4.99E-30	yes	up	4.996	21.716	5.47	8.01	3.95	2.98	4.57	20.56	21.17	23.58	20.74	22.53
ENSMUSG00000032113	Chek1	checkpoint kinase 1 [Source:MGI Symbol;Acc:MGI:1202065]	2.315683643	1.211438173	0.003770286	0.012759868	yes	up	0.85	0.952	1.08	0.54	1.08	1.24	0.31	1.6	0.72	0.75	0.47	1.22
ENSMUSG00000029521	Chek2	checkpoint kinase 2 [Source:MGI Symbol;Acc:MGI:1355321]	2.354111012	1.235182355	3.53E-10	3.76E-09	yes	up	4.01	6.234	4.25	1.86	4.17	3.05	6.72	7.86	5.38	4.27	6.3	7.36
ENSMUSG00000064246	Chil1	chitinase-like 1 [Source:MGI Symbol;Acc:MGI:1340899]	2.545661116	1.348040377	1.22E-23	3.89E-22	yes	up	661.088	1426.87	562.21	839.71	642.62	505.24	755.66	1177.24	1424.04	1583.27	1427.68	1522.12
ENSMUSG00000040809	Chil3	chitinase-like 3 [Source:MGI Symbol;Acc:MGI:1330860]	7.324405731	2.872711711	2.27E-41	1.64E-39	yes	up	658.996	4130.12	654.58	653.5	860.89	356.73	769.28	4085.97	4797.84	3877.22	4612.76	3276.81
ENSMUSG00000063779	Chil4	chitinase-like 4 [Source:MGI Symbol;Acc:MGI:1341098]	11.01304755	3.461141844	0.000109683	0.000527028	yes	up	0.07	0.67	0	0.05	0	0.2	0.1	1	0.83	0.62	0.1	0.8
ENSMUSG00000030077	Chl1	cell adhesion molecule L1-like [Source:MGI Symbol;Acc:MGI:1098266]	4.804166326	2.2642861	5.87E-05	0.000296394	yes	up	0.326	1.286	0.1	0.88	0.2	0.31	0.14	1.37	1.22	1.12	1.05	1.67
ENSMUSG00000022860	Chodl	chondrolectin [Source:MGI Symbol;Acc:MGI:2179069]	4.319179187	2.11075717	0.008606231	0.026401811	yes	up	0.094	0.316	0	0.09	0.21	0.14	0.03	0.15	0.56	0.4	0.28	0.19
ENSMUSG00000006958	Chrd	chordin [Source:MGI Symbol;Acc:MGI:1313268]	0.490387576	-1.028005667	2.23E-05	0.000121926	yes	down	1.47	0.58	1.65	1.83	1.16	1.28	1.43	0.69	0.66	0.47	0.54	0.54
ENSMUSG00000031283	Chrdl1	chordin-like 1 [Source:MGI Symbol;Acc:MGI:1933172]	0.414943605	-1.269012822	1.39E-14	2.31E-13	yes	down	12.67	4.402	14.94	11.28	11.65	14.48	11	5.52	4.04	4.4	5.2	2.85
ENSMUSG00000045613	Chrm2	cholinergic receptor, muscarinic 2, cardiac [Source:MGI Symbol;Acc:MGI:88397]	0.475413381	-1.072745584	3.10E-09	3.00E-08	yes	down	5.164	2.074	3.91	7.57	4.85	4.38	5.11	2.18	2.03	2.41	1.58	2.17
ENSMUSG00000046159	Chrm3	cholinergic receptor, muscarinic 3, cardiac [Source:MGI Symbol;Acc:MGI:88398]	0.265278116	-1.914422427	1.86E-07	1.42E-06	yes	down	1.748	0.344	1.37	2.6	1.33	2.34	1.1	0.47	0.14	0.54	0.13	0.44
ENSMUSG00000035930	Chst4	carbohydrate sulfotransferase 4 [Source:MGI Symbol;Acc:MGI:1349479]	0.318092645	-1.652481082	2.63E-11	3.16E-10	yes	down	5.98	1.972	3.99	6.92	5.2	5.68	8.11	1.33	2.7	2.43	2.5	0.9
ENSMUSG00000060402	Chst8	carbohydrate sulfotransferase 8 [Source:MGI Symbol;Acc:MGI:1916197]	0.30877392	-1.695377193	6.03E-09	5.63E-08	yes	down	3.792	1.282	4.43	1.83	5.75	4.61	2.34	1.05	0.83	0.91	1.39	2.23
ENSMUSG00000047161	Chst9	carbohydrate (N-acetylgalactosamine 4-0) sulfotransferase 9 [Source:MGI Symbol;Acc:MGI:1918617]	0.052512178	-4.251204162	0.006840858	0.021662548	yes	down	0.14	0	0.04	0.32	0.15	0.08	0.11	0	0	0	0	0
ENSMUSG00000037493	Cib2	calcium and integrin binding family member 2 [Source:MGI	4.250389474	2.087595045	2.69E-39	1.82E-37	yes	up	6.852	24.724	7	6.57	7.12	5.32	8.25	17.84	29.08	28.45	25.42	22.83

		Symbol;Acc:MGI:1929293]																		
ENSMUSG00000022219	Cideb	cell death-inducing DNA fragmentation factor, alpha subunit-like effector B [Source:MGI Symbol;Acc:MGI:1270844]	5.053507909	2.337285187	8.54E-13	1.19E-11	yes	up	0.898	3.816	0.87	1.12	0.62	1.37	0.51	2.59	4.97	3.13	4.36	4.03
ENSMUSG00000022504	Ciita	class II transactivator [Source:MGI Symbol;Acc:MGI:108445]	2.795297018	1.483001587	2.41E-23	7.60E-22	yes	up	10.464	24.528	9.84	9.18	13.39	9.13	10.78	25.53	30.74	23.35	16.63	26.39
ENSMUSG00000042254	Cilp	cartilage intermediate layer protein, nucleotide pyrophosphohydrolase [Source:MGI Symbol;Acc:MGI:2444507]	4.303997456	2.105677225	3.15E-07	2.33E-06	yes	up	1.344	4.888	0.63	1.91	1.75	0.81	1.62	4.55	4.12	7.18	2.33	6.26
ENSMUSG00000033031	Cip2a	cell proliferation regulating inhibitor of protein phosphatase 2A [Source:MGI Symbol;Acc:MGI:2146335]	2.20066735	1.137941086	7.85E-07	5.47E-06	yes	up	1.716	3.164	1.21	1.73	2.58	1.62	1.44	3.5	3.21	3.16	2.2	3.75
ENSMUSG00000070803	Cited4	Cbp/p300-interacting transactivator, with Glu/Asp-rich carboxy-terminal domain, 4 [Source:MGI Symbol;Acc:MGI:1861694]	2.219020492	1.14992299	0.01165326	0.034241785	yes	up	0.604	1.13	0.74	0.7	0.31	0.59	0.68	1.18	1.14	1.89	0.67	0.77
ENSMUSG00000037725	Ckap2	cytoskeleton associated protein 2 [Source:MGI Symbol;Acc:MGI:1931797]	2.527683378	1.33781576	0.000754138	0.003033497	yes	up	1.62	2.728	1.88	1.1	1.88	1.84	1.4	0.69	4.2	2.4	2.32	4.03
ENSMUSG00000048327	Ckap2l	cytoskeleton associated protein 2-like [Source:MGI Symbol;Acc:MGI:1917716]	2.414795892	1.271901252	4.35E-10	4.59E-09	yes	up	1.834	3.73	2.05	1.7	1.97	1.32	2.13	4.34	3.86	3.29	2.49	4.67
ENSMUSG00000028044	Cks1b	CDC28 protein kinase 1b [Source:MGI Symbol;Acc:MGI:1889208]	2.089864259	1.063409239	3.35E-08	2.84E-07	yes	up	9.058	16.108	8.71	9.56	10.34	7.4	9.28	14.37	16.34	13.51	13.4	22.92
ENSMUSG00000062248	Cks2	CDC28 protein kinase regulatory subunit 2 [Source:MGI Symbol;Acc:MGI:1913447]	2.170678513	1.118146072	9.93E-07	6.79E-06	yes	up	10.758	19.806	7.41	9.62	14.07	11.24	11.45	28.68	15.42	15.45	18.83	20.65
ENSMUSG00000036636	Clcn7	chloride channel, voltage-sensitive 7 [Source:MGI Symbol;Acc:MGI:1347048]	2.059353555	1.042191537	1.63E-27	6.54E-26	yes	up	15.594	26.67	16.72	13.95	15.07	16.01	16.22	23.47	27.31	24.56	25.44	32.57
ENSMUSG00000022132	Cldn10	claudin 10 [Source:MGI Symbol;Acc:MGI:1913101]	0.342874158	-1.544248921	8.79E-27	3.38E-25	yes	down	14.714	4.252	15.38	13.59	13.8	14.51	16.29	4.43	4.03	5.52	3.29	3.99
ENSMUSG00000001739	Cldn15	claudin 15 [Source:MGI Symbol;Acc:MGI:1913103]	0.413716281	-1.27328636	5.47E-11	6.32E-10	yes	down	10.254	3.608	9.9	8.57	12.11	9.75	10.94	3.41	2.81	3.17	5.71	2.94
ENSMUSG00000041378	Cldn5	claudin 5 [Source:MGI Symbol;Acc:MGI:1276112]	0.476916467	-1.068191497	3.37E-19	8.16E-18	yes	down	502.46	202.246	500.54	462.85	417.78	684.98	446.15	189.33	250.41	171.08	193.45	206.96
ENSMUSG00000050520	Cldn8	claudin 8 [Source:MGI Symbol;Acc:MGI:1859286]	0.31718261	-1.656614418	3.55E-07	2.60E-06	yes	down	2.51	0.672	2	3.43	1.46	3.02	2.64	1.04	0.86	0.42	0.64	0.4
ENSMUSG00000053063	Clec12a	C-type lectin domain family 12, member a [Source:MGI Symbol;Acc:MGI:3040968]	6.006563946	2.586539933	2.81E-51	2.87E-49	yes	up	6.636	33.576	4.38	8.25	8.82	6.47	5.26	32.61	33.2	28.05	32.07	41.95
ENSMUSG00000030158	Clec12b	C-type lectin domain family 12, member B [Source:MGI Symbol;Acc:MGI:1918433]	0.463281506	-1.110039004	9.89E-05	0.000479359	yes	down	2.728	1.112	2.87	2.12	2.95	2.44	3.26	0.96	1.27	1.1	1.48	0.75
ENSMUSG00000049037	Clec4a1	C-type lectin domain family 4, member a1 [Source:MGI Symbol;Acc:MGI:3036291]	3.510586156	1.811711935	3.63E-22	1.05E-20	yes	up	5.94	17.494	6.26	6.21	5.18	7.9	4.15	24.67	15.21	15.41	14.81	17.37
ENSMUSG00000030148	Clec4a2	C-type lectin domain family 4, member a2 [Source:MGI Symbol;Acc:MGI:1349412]	6.964631736	2.800047071	7.47E-82	1.68E-79	yes	up	9.338	53.082	7.94	11.01	13.98	6.56	7.2	47.19	54.04	53.06	58.26	52.86

ENSMUSG00000043832	Clec4a3	C-type lectin domain family 4, member a3 [Source:MGI Symbol;Acc:MGI:1920399]	4.150195457	2.053179283	9.19E-79	1.99E-76	yes	up	18.228	65.018	17.12	17.63	24.28	17.3	14.81	63.7	66.64	64.57	71.66	58.52
ENSMUSG00000030147	Clec4b1	C-type lectin domain family 4, member b1 [Source:MGI Symbol;Acc:MGI:1917060]	2.604185427	1.380832177	6.12E-06	3.70E-05	yes	up	3.018	6.652	3.26	4.68	2.89	2.25	2.01	7.59	5.15	7.67	6.92	5.93
ENSMUSG00000030144	Clec4d	C-type lectin domain family 4, member d [Source:MGI Symbol;Acc:MGI:1298389]	17.52396642	4.13125745	4.65E-138	3.69E-135	yes	up	8.106	117.83	8.86	9.68	6.44	9.76	5.79	162.45	103.66	93.75	121.8	107.49
ENSMUSG00000030142	Clec4e	C-type lectin domain family 4, member e [Source:MGI Symbol;Acc:MGI:1861232]	6.12164759	2.613919995	6.42E-27	2.50E-25	yes	up	2.81	12.564	2.71	3.77	1.28	4.51	1.78	16.94	9.67	9.68	10.43	16.1
ENSMUSG00000074491	Clec4g	C-type lectin domain family 4, member g [Source:MGI Symbol;Acc:MGI:1923113]	0.357004593	-1.485985458	0.000520864	0.002172736	yes	down	1.546	0.448	1.54	1.26	2.56	1.18	1.19	0.44	0.38	0.43	0.31	0.68
ENSMUSG00000023349	Clec4n	C-type lectin domain family 4, member n [Source:MGI Symbol;Acc:MGI:1861231]	8.934280956	3.159351624	1.38E-88	3.78E-86	yes	up	19.156	147.12	16.45	24.61	23.94	11.67	19.11	146.14	137.57	159.55	146.66	145.68
ENSMUSG00000029915	Clec5a	C-type lectin domain family 5, member a [Source:MGI Symbol;Acc:MGI:1345151]	7.356712932	2.879061297	3.84E-49	3.68E-47	yes	up	2.75	20.64	2.46	3.64	2.39	1.85	3.41	18.9	17.83	19.77	17.58	29.12
ENSMUSG00000079293	Clec7a	C-type lectin domain family 7, member a [Source:MGI Symbol;Acc:MGI:1861431]	16.98623898	4.086294547	5.29E-79	1.16E-76	yes	up	48.116	692.848	41.46	70.32	60.97	28.38	39.45	636.88	704.94	661.7	746.33	714.39
ENSMUSG00000002190	Clgn	calmegin [Source:MGI Symbol;Acc:MGI:107472]	11.30023827	3.498281288	0.000489458	0.002054935	yes	up	0.018	0.244	0	0	0.05	0	0.04	0.55	0.09	0.27	0.05	0.26
ENSMUSG00000024059	Clip4	CAP-GLY domain containing linker protein family, member 4 [Source:MGI Symbol;Acc:MGI:1919100]	2.082605483	1.058389569	1.29E-09	1.30E-08	yes	up	16.292	34.134	15.39	23.19	13.46	10.86	18.56	29.28	31.74	36.21	39.55	33.89
ENSMUSG00000032024	Clmp	CXADR-like membrane protein [Source:MGI Symbol;Acc:MGI:1918816]	4.215215898	2.075606526	3.43E-99	1.20E-96	yes	up	5.2	18.336	4.83	6.17	4.77	5.92	4.31	18.65	19.66	16.84	18.6	17.93
ENSMUSG00000039315	Clnk	cytokine-dependent hematopoietic cell linker [Source:MGI Symbol;Acc:MGI:1351468]	2.14931348	1.103875917	0.003343987	0.011455105	yes	up	0.566	1.03	0.42	0.56	0.85	0.36	0.64	0.84	0.78	1.06	1.19	1.28
ENSMUSG00000042489	Clspn	claspin [Source:MGI Symbol;Acc:MGI:2445153]	2.173879848	1.120272204	1.21E-05	6.94E-05	yes	up	1.134	2.17	1.63	1.07	1.04	0.7	1.23	2.36	1.94	2	0.93	3.62
ENSMUSG00000022037	Clu	clusterin [Source:MGI Symbol;Acc:MGI:88423]	2.004338374	1.003126086	1.15E-14	1.92E-13	yes	up	201.45	320.75	196.25	237.03	201.05	153.11	219.81	261.04	289.01	369.7	323.33	360.67
ENSMUSG00000022225	Cma1	chymase 1, mast cell [Source:MGI Symbol;Acc:MGI:96941]	0.40740399	-1.295467984	0.00355702	0.012103138	yes	down	2.88	0.994	2.1	6.27	1.69	2.48	1.86	0.63	1.58	0.69	1.13	0.94
ENSMUSG00000038085	Cnbd2	cyclic nucleotide binding domain containing 2 [Source:MGI Symbol;Acc:MGI:1918123]	2.529239697	1.338703768	0.004886058	0.016056438	yes	up	4.328	8.842	6.48	2.65	4.62	3.89	4	3.94	6.83	3.68	19.38	10.38
ENSMUSG00000005864	Cnga2	cyclic nucleotide gated channel alpha 2 [Source:MGI Symbol;Acc:MGI:108040]	0.172944202	-2.531621446	2.15E-16	4.21E-15	yes	down	1.322	0.244	1.21	1.45	1.43	1.4	1.12	0.18	0.52	0.11	0.19	0.22
ENSMUSG00000025658	Cnksr2	connector enhancer of kinase suppressor of Ras 2 [Source:MGI	0.407278623	-1.295912003	0.000175922	0.00081127	yes	down	0.638	0.22	0.6	0.43	0.66	0.75	0.75	0.3	0.14	0.14	0.38	0.14

	Symbol;Acc:MGI:2661175]																		
ENSMUSG00000001349 Cnn1	calponin 1 [Source:MGI Symbol;Acc:MGI:104979]	0.295726285	-1.757665615	0.000194001	0.00088779	yes	down	47.226	10.958	33.19	94.41	21.1	39.2	48.23	10.73	9.29	21.1	6.32	7.35
ENSMUSG00000062585 Cnr2	cannabinoid receptor 2 (macrophage) [Source:MGI Symbol;Acc:MGI:104650]	2.068653166	1.048691781	8.14E-20	2.04E-18	yes	up	4.614	7.986	4.32	4.53	4.72	5.43	4.07	9.01	8.04	6.58	7.77	8.53
ENSMUSG00000028444 Cntfr	ciliary neurotrophic factor receptor [Source:MGI Symbol;Acc:MGI:99605]	0.466236991	-1.100864624	4.67E-06	2.88E-05	yes	down	3.858	1.546	4.03	4.08	3.65	3.87	3.66	1.22	2.8	1.11	1.38	1.22
ENSMUSG00000055022 Cntn1	contactin 1 [Source:MGI Symbol;Acc:MGI:105980]	0.298969734	-1.741928654	6.72E-05	0.00033701	yes	down	0.832	0.23	0.48	1.23	0.36	1.11	0.98	0.38	0.16	0.14	0.13	0.34
ENSMUSG00000032332 Col12a1	collagen, type XII, alpha 1 [Source:MGI Symbol;Acc:MGI:88448]	0.388996714	-1.362170127	8.86E-22	2.49E-20	yes	down	19.734	6.16	18.67	19.17	17.6	23.59	19.64	4.95	4.81	5.6	7.57	7.87
ENSMUSG00000058806 Col13a1	collagen, type XIII, alpha 1 [Source:MGI Symbol;Acc:MGI:1277201]	0.461333135	-1.116119178	8.65E-32	4.40E-30	yes	down	36.254	14.204	38.74	37.69	35.08	39.58	30.18	12.47	16.31	12.39	16.12	13.73
ENSMUSG00000025064 Col17a1	collagen, type XVII, alpha 1 [Source:MGI Symbol;Acc:MGI:88450]	0.156098046	-2.679475615	4.27E-18	9.54E-17	yes	down	1.58	0.394	1.29	1.59	1.18	2.55	1.29	0.1	1.08	0.29	0.18	0.32
ENSMUSG00000016356 Col20a1	collagen, type XX, alpha 1 [Source:MGI Symbol;Acc:MGI:1920618]	0.49070785	-1.027063743	0.000733574	0.002959168	yes	down	2.962	12.22	3.27	3.23	0.61	7.03	0.67	0.36	4.18	2.08	54.32	0.16
ENSMUSG00000028197 Col24a1	collagen, type XXIV, alpha 1 [Source:MGI Symbol;Acc:MGI:1918605]	19.04217536	4.251126395	8.97E-55	1.02E-52	yes	up	0.172	2.45	0.14	0.33	0.18	0.17	0.04	1.85	2	3.18	2.76	2.46
ENSMUSG00000068794 Col28a1	collagen, type XXVIII, alpha 1 [Source:MGI Symbol;Acc:MGI:2685312]	3.784410094	1.920068433	5.68E-05	0.000287395	yes	up	1.404	4.548	1.09	1.49	2.03	0.67	1.74	1.16	5.85	3.42	5.59	6.72
ENSMUSG00000032572 Col6a4	collagen, type VI, alpha 4 [Source:MGI Symbol;Acc:MGI:1915803]	4.058870263	2.021078227	4.32E-08	3.60E-07	yes	up	0.304	0.89	0.29	0.39	0.53	0.27	0.04	0.66	0.66	0.97	0.81	1.35
ENSMUSG00000091345 Col6a5	collagen, type VI, alpha 5 [Source:MGI Symbol;Acc:MGI:3648134]	0.273780035	-1.868910849	0.001943024	0.007098567	yes	down	0.272	0.084	0.27	0.5	0.29	0.21	0.09	0.02	0.19	0.09	0.1	0.02
ENSMUSG00000043719 Col6a6	collagen, type VI, alpha 6 [Source:MGI Symbol;Acc:MGI:2444259]	0.316528591	-1.659592276	0.015076432	0.042646529	yes	down	0.096	0.026	0.08	0.18	0.09	0.11	0.02	0.01	0.03	0.05	0.03	0.01
ENSMUSG00000032649 Colgalt2	collagen beta(1-O)galactosyltransferase 2 [Source:MGI Symbol;Acc:MGI:2138232]	0.418410335	-1.257009609	4.80E-24	1.57E-22	yes	down	12.7	4.336	14.55	11.86	10.89	14.96	11.24	4.54	5.1	3.57	3.92	4.55
ENSMUSG00000057606 Colq	collagen-like tail subunit (single strand of homotrimer) of asymmetric acetylcholinesterase [Source:MGI Symbol;Acc:MGI:1338761]	0.134494118	-2.894385018	2.87E-65	4.52E-63	yes	down	15.136	3.51	13.39	16.43	13.58	19.48	12.8	2.3	1.62	10.8	1.11	1.72
ENSMUSG00000031827 Cotl1	coactosin-like 1 (Dictyostelium) [Source:MGI Symbol;Acc:MGI:1919292]	3.285394606	1.716066662	2.16E-71	4.05E-69	yes	up	52.356	147.714	50.01	59.17	58.5	46.24	47.86	127.03	152.91	142.97	148.66	167
ENSMUSG00000025488 Cox8b	cytochrome c oxidase subunit 8B [Source:MGI Symbol;Acc:MGI:105958]	0.221158054	-2.17685031	0.002557963	0.009062255	yes	down	17.158	3.19	4.08	48.06	12.06	9.13	12.46	3.68	0.56	6.97	3.62	1.12
ENSMUSG00000001865 Cpa3	carboxypeptidase A3, mast cell [Source:MGI Symbol;Acc:MGI:88479]	0.326684975	-1.61402799	3.07E-11	3.66E-10	yes	down	12.822	3.786	15.18	16.08	10.08	14.86	7.91	3.74	4.41	3.23	4.14	3.41
ENSMUSG00000011463 Cpb1	carboxypeptidase B1 (tissue) [Source:MGI Symbol;Acc:MGI:1923953]	0.254562028	-1.973910863	0.001523425	0.005706143	yes	down	0.596	0.1	0.83	0.5	0.65	0.63	0.37	0	0.17	0.15	0.12	0.06
ENSMUSG00000024008 Cpne5	copine V [Source:MGI Symbol;Acc:MGI:2385908]	0.263715341	-1.922946595	5.66E-10	5.90E-09	yes	down	3.124	1.154	3.69	3.24	4.23	1.72	2.74	1.15	0.75	1.11	1.45	1.31
ENSMUSG00000027408 Cpxm1	carboxypeptidase X 1 (M14 family) [Source:MGI Symbol;Acc:MGI:1934569]	2.937174994	1.554429223	1.03E-21	2.89E-20	yes	up	15.654	41.584	16.87	15.61	20.32	10.62	14.85	37.82	39.43	57.15	32.21	41.31
ENSMUSG00000095207 CR974586.1	protein FAM205A-like [Source:NCBI gene;Acc:100862043]	0.289849726	-1.786622973	0.001795684	0.006614444	yes	down	1.38	0.334	2.26	0.91	1.78	0.93	1.02	0.41	0.03	0.77	0.09	0.37
ENSMUSG00000096506 CR974586.5		5.526519298	2.466371131	1.35E-05	7.69E-05	yes	up	0.85	2.97	0	1.38	1.02	0.74	1.11	2.63	1.38	4.96	2.15	3.73

ENSMUSG00000035403 Crb2	crumbs family member 2 [Source:MGI Symbol;Acc:MGI:2679260]	0.455607347	-1.134137083	0.007605528	0.023729603	yes	down	0.366	0.168	0.35	0.17	0.48	0.28	0.55	0.06	0.17	0.2	0.32	0.09
ENSMUSG00000035041 Creb3l3	cAMP responsive element binding protein 3-like 3 [Source:MGI Symbol;Acc:MGI:2384786]	7.299257357	2.867749688	0.006308216	0.020148658	yes	up	0.028	0.176	0.03	0.03	0.03	0	0.05	0.05	0.25	0.16	0.37	0.05
ENSMUSG00000040713 Creg1	cellular repressor of E1A-stimulated genes 1 [Source:MGI Symbol;Acc:MGI:1344382]	2.39536745	1.260246983	9.23E-56	1.07E-53	yes	up	77.948	157.842	76.79	72.61	79.15	80.36	80.83	159.31	159.43	138.38	177.19	154.9
ENSMUSG00000050967 Creg2	cellular repressor of E1A-stimulated genes 2 [Source:MGI Symbol;Acc:MGI:1928333]	23.83339126	4.574912343	1.03E-67	1.73E-65	yes	up	0.17	3.404	0.09	0.25	0.25	0.05	0.21	3.67	3.11	2.92	4.19	3.13
ENSMUSG00000023272 Creld2	cysteine-rich with EGF-like domains 2 [Source:MGI Symbol;Acc:MGI:1923987]	2.210113267	1.144120309	5.07E-22	1.45E-20	yes	up	49.206	91.886	56.19	40.1	51.85	43.39	54.5	107.24	82.95	90.71	86.74	91.79
ENSMUSG00000025776 Crispld1	cysteine-rich secretory protein LCCL domain containing 1 [Source:MGI Symbol;Acc:MGI:1934666]	0.49833851	-1.004802031	0.000295814	0.001304771	yes	down	1.222	0.412	0.83	1.13	1.62	1.28	1.25	0.27	0.43	0.63	0.35	0.38
ENSMUSG00000031825 Crispld2	cysteine-rich secretory protein LCCL domain containing 2 [Source:MGI Symbol;Acc:MGI:1926142]	0.243965682	-2.035249875	3.00E-53	3.24E-51	yes	down	62.532	12.972	77.26	49.62	65.39	55.16	65.23	11.77	11.76	13.31	16.74	11.28
ENSMUSG00000033467 Crlf2	cytokine receptor-like factor 2 [Source:MGI Symbol;Acc:MGI:1889506]	3.077687968	1.621846971	1.44E-13	2.18E-12	yes	up	13.844	35.156	9.66	11.8	13.35	17.92	16.49	37.07	37.43	38.24	17.99	45.05
ENSMUSG00000029121 Crmp1	collapsin response mediator protein 1 [Source:MGI Symbol;Acc:MGI:107793]	3.216502458	1.685492791	5.98E-06	3.63E-05	yes	up	0.8	1.682	0.27	0.44	1.18	1.22	0.89	1.54	1.03	1.01	1.73	3.1
ENSMUSG00000084989 Crocc2	ciliary rootlet coiled-coil, rootletin family member 2 [Source:MGI Symbol;Acc:MGI:3045962]	0.382692382	-1.385742912	3.14E-05	0.000166858	yes	down	1.078	0.348	0.84	0.97	0.77	1.43	1.38	0.26	0.29	0.62	0.13	0.44
ENSMUSG00000066975 Cryba4	crystallin, beta A4 [Source:MGI Symbol;Acc:MGI:102716]	3.417395373	1.772897169	0.012326895	0.03593205	yes	up	0.442	1.25	0.63	0.59	0	0.44	0.55	1.34	0.69	2.6	0.64	0.98
ENSMUSG00000029352 Crybb3	crystallin, beta B3 [Source:MGI Symbol;Acc:MGI:102717]	0.353396274	-1.500641268	0.016597464	0.046330982	yes	down	2.698	0.612	3.28	1.85	2.79	1.15	4.42	0.92	1.15	0.79	0.11	0.09
ENSMUSG00000030905 Crym	crystallin, mu [Source:MGI Symbol;Acc:MGI:102675]	0.494281405	-1.016595461	1.52E-06	1.02E-05	yes	down	4.096	1.7	4.2	3.92	4.08	3.83	4.45	1.84	1.97	1.66	1.44	1.59
ENSMUSG00000024621 Csf1r	colony stimulating factor 1 receptor [Source:MGI Symbol;Acc:MGI:1339758]	2.258510003	1.175371303	9.86E-93	2.93E-90	yes	up	47	105.96	44.92	45.76	48.96	50.57	44.79	110.6	105.83	111.3	92.93	109.14
ENSMUSG00000059326 Csf2ra	colony stimulating factor 2 receptor, alpha, low-affinity (granulocyte-macrophage) [Source:MGI Symbol;Acc:MGI:1339754]	4.729300686	2.24162687	2.56E-68	4.43E-66	yes	up	26.162	106.116	18.49	22.1	25.22	34.17	30.83	110.31	129.63	100.22	74.28	116.14
ENSMUSG00000071713 Csf2rb	colony stimulating factor 2 receptor, beta, low-affinity (granulocyte-macrophage) [Source:MGI Symbol;Acc:MGI:1339759]	4.776264198	2.255882641	1.77E-120	1.06E-117	yes	up	15.27	56.786	12.73	19.2	14.18	10.03	20.21	69.32	61.03	51.09	49.63	52.86
ENSMUSG00000071714 Csf2rb2	colony stimulating factor 2 receptor, beta 2, low-affinity (granulocyte-macrophage) [Source:MGI Symbol;Acc:MGI:1339760]	6.728071724	2.750193085	6.41E-105	2.59E-102	yes	up	4.766	24.124	4.15	4.97	5.79	3.26	5.66	22.63	24.48	24.58	22.66	26.27
ENSMUSG00000036356 Csgalnact1	chondroitin sulfate N-acetylgalactosaminyltransferase 1 [Source:MGI Symbol;Acc:MGI:2442354]	0.43453894	-1.202442629	9.15E-26	3.31E-24	yes	down	16.514	5.7	17.62	14.9	18.74	14.48	16.83	4.69	7.19	5.21	6.24	5.17

ENSMUSG00000060924 Csmd1	CUB and Sushi multiple domains 1 [Source:MGI Symbol;Acc:MGI:2137383]	5.284221774	2.401691016	8.18E-19	1.93E-17	yes	up	0.17	0.632	0.14	0.29	0.18	0.1	0.14	0.72	0.46	0.76	0.39	0.83
ENSMUSG00000062783 Csprs	component of Sp100-rs [Source:NCBI gene;Acc:114564]	2.10176741	1.071603024	0.000311648	0.001367957	yes	up	2.38	3.46	1.08	3.56	5.76	0.87	0.63	11.63	1.46	1.28	1.08	1.85
ENSMUSG00000032515 Csrnp1	cysteine-serine-rich nuclear protein 1 [Source:MGI Symbol;Acc:MGI:2387989]	0.255221114	-1.970180408	3.91E-15	6.81E-14	yes	down	64.376	13.672	67.13	96.24	39.83	60.6	58.08	14.6	12.43	18.53	10.72	12.08
ENSMUSG00000024846 Cst6	cystatin E/M [Source:MGI Symbol;Acc:MGI:1920970]	4.137576773	2.048786081	0.008502564	0.02613245	yes	up	0.05	0.922	0	0.07	0	0.07	0.11	1.44	2.62	0.14	0.08	0.33
ENSMUSG00000068129 Cst7	cystatin F (leukocystatin) [Source:MGI Symbol;Acc:MGI:1298217]	3.613451565	1.853377557	1.12E-06	7.60E-06	yes	up	3.136	9.548	1.6	4.27	3.63	2.86	3.32	13.25	5.2	7.73	3.96	17.6
ENSMUSG00000027442 Cst8	cystatin 8 (cystatin-related epididymal spermatogenic) [Source:MGI Symbol;Acc:MGI:107161]	0.046565359	-4.42459908	2.94E-27	1.16E-25	yes	down	11.892	0.548	11.03	12.34	8.69	14.95	12.45	0.1	1.45	0.58	0.42	0.19
ENSMUSG00000005054 Cstb	cystatin B [Source:MGI Symbol;Acc:MGI:109514]	2.958088804	1.564665364	9.74E-56	1.13E-53	yes	up	141.562	355.514	143.91	164.84	138.59	117.53	142.94	340.1	350.34	322.87	371.31	392.95
ENSMUSG00000117003 CT010496.1	novel transcript	0.261325093	-1.936082432	0.000120371	0.000573623	yes	down	0.666	0.148	0.67	0.47	1.08	0.56	0.55	0.02	0.28	0.15	0.1	0.19
ENSMUSG00000074874 Ctla2b	cytotoxic T lymphocyte-associated protein 2 beta [Source:MGI Symbol;Acc:MGI:88555]	0.489880958	-1.029496881	1.85E-10	2.03E-09	yes	down	51.184	20.458	62.15	60.31	39.22	56.94	37.3	21.83	23.56	16.32	22.65	17.93
ENSMUSG00000026011 Ctla4	cytotoxic T-lymphocyte-associated protein 4 [Source:MGI Symbol;Acc:MGI:88556]	2.175419815	1.121293841	0.000497532	0.002085563	yes	up	3.244	6.074	3.69	2.98	4	3.05	2.5	6.73	4.98	12.26	2.63	3.77
ENSMUSG00000017760 Ctsa	cathepsin A [Source:MGI Symbol;Acc:MGI:97748]	2.791556729	1.481069875	1.69E-84	4.20E-82	yes	up	127.044	314.26	128.78	132.07	130	123.11	121.26	289.81	332.97	289.15	307.33	352.04
ENSMUSG00000021939 Ctsb	cathepsin B [Source:MGI Symbol;Acc:MGI:88561]	4.224168102	2.078667248	3.73E-183	7.26E-180	yes	up	218.85	790.074	229.41	232.39	230.09	193.93	208.43	761.4	776.98	722.49	819.25	870.25
ENSMUSG00000030560 Ctsc	cathepsin C [Source:MGI Symbol;Acc:MGI:109553]	3.034907773	1.601652676	6.43E-82	1.46E-79	yes	up	195.844	498.812	204.9	188.11	223.6	173.48	189.13	470.34	473.96	476.32	527.59	545.85
ENSMUSG00000007891 Ctsd	cathepsin D [Source:MGI Symbol;Acc:MGI:88562]	8.488603078	3.085527157	1.54E-197	4.70E-194	yes	up	454.866	3279.108	453.53	459.24	516.62	353.85	491.09	3200.65	3403.86	2953.8	3588.02	3249.21
ENSMUSG00000004552 Ctse	cathepsin E [Source:MGI Symbol;Acc:MGI:107361]	3.064070878	1.61544967	2.93E-22	8.57E-21	yes	up	7.458	19.59	7.52	7.54	7.32	9.3	5.61	26.64	17.12	18.39	13.98	21.82
ENSMUSG00000032359 Ctsh	cathepsin H [Source:MGI Symbol;Acc:MGI:107285]	2.037669419	1.026920015	7.01E-18	1.54E-16	yes	up	380.674	653.612	370.6	368.18	420.03	300.46	444.1	561.69	640.51	691.96	648.41	725.49
ENSMUSG00000028111 Ctsk	cathepsin K [Source:MGI Symbol;Acc:MGI:107823]	15.40976743	3.945773183	1.60E-71	3.04E-69	yes	up	22.914	304.554	18.54	27.93	24.8	13.89	29.41	220	338.03	351.41	317.11	296.22
ENSMUSG00000038642 Ctss	cathepsin S [Source:MGI Symbol;Acc:MGI:107341]	13.56179181	3.761475898	3.02E-165	4.31E-162	yes	up	260.678	2993.674	224.2	297.29	332.4	187.82	261.68	2930.53	2879.05	2499.49	3214.93	3444.37
ENSMUSG00000016256 Ctsz	cathepsin Z [Source:MGI Symbol;Acc:MGI:1891190]	4.323951353	2.112350292	8.10E-126	5.78E-123	yes	up	144.568	523.408	139.01	148.27	159.49	128.53	147.54	452.94	533.15	483.24	537.76	609.95
ENSMUSG00000000416 Cttnbp2	cortactin binding protein 2 [Source:MGI Symbol;Acc:MGI:1353467]	0.488671728	-1.033062456	3.32E-05	0.000175168	yes	down	2.774	1.514	2.22	3.23	2.24	4.36	1.82	0.73	1.73	2.44	1.46	1.21
ENSMUSG00000052336 Cx3cr1	chemokine (C-X3-C motif) receptor 1 [Source:MGI Symbol;Acc:MGI:1333815]	2.706214082	1.436275972	6.63E-26	2.43E-24	yes	up	13.63	20.982	15.59	12.33	14.29	13.89	12.05	19.85	19.15	21.7	16.54	27.67
ENSMUSG00000034855 Cxcl10	chemokine (C-X-C motif) ligand 10 [Source:MGI Symbol;Acc:MGI:1352450]	9.828788209	3.297013558	7.13E-13	9.99E-12	yes	up	2.602	21.416	4.31	2.72	2.07	2.22	1.69	30.89	9.11	18.07	11.81	37.2
ENSMUSG00000018920 Cxcl16	chemokine (C-X-C motif) ligand 16 [Source:MGI Symbol;Acc:MGI:1932682]	2.863627875	1.517844028	1.22E-62	1.71E-60	yes	up	50.338	121.664	48.66	49.75	51.24	50.95	51.09	115.94	117.97	112.41	114.9	147.1

ENSMUSG00000029379	Cxcl3	chemokine (C-X-C motif) ligand 3 [Source:MGI Symbol;Acc:MGI:3037818]	25.91809265	4.695887647	3.56E-23	1.11E-21	yes	up	0.272	5.976	0.4	0.17	0.17	0	0.62	5.36	5.87	8.77	4	5.88
ENSMUSG00000029417	Cxcl9	chemokine (C-X-C motif) ligand 9 [Source:MGI Symbol;Acc:MGI:1352449]	28.08617366	4.811788186	1.45E-17	3.11E-16	yes	up	1.63	38.258	2.24	1.84	2.3	1.04	0.73	59.77	15.44	19.86	17.89	78.33
ENSMUSG00000048480	Cxcr1	chemokine (C-X-C motif) receptor 1 [Source:MGI Symbol;Acc:MGI:2448715]	4.489362301	2.16651053	3.46E-08	2.93E-07	yes	up	0.856	3.234	0.99	0.73	0.79	1.47	0.3	2.83	4.16	1.68	4.07	3.43
ENSMUSG00000050232	Cxcr3	chemokine (C-X-C motif) receptor 3 [Source:MGI Symbol;Acc:MGI:1277207]	3.657918604	1.871022973	2.59E-20	6.73E-19	yes	up	3.658	11.258	2.61	3.82	4.98	3.65	3.23	15.77	10.43	9.23	8.94	11.92
ENSMUSG00000048521	Cxcr6	chemokine (C-X-C motif) receptor 6 [Source:MGI Symbol;Acc:MGI:1934582]	2.588591187	1.37216714	1.44E-11	1.78E-10	yes	up	7.048	15.356	5.2	7.08	9.45	6.9	6.61	17.18	10.54	12.64	13.94	22.48
ENSMUSG00000044365	Cxxc4	CXXC finger 4 [Source:MGI Symbol;Acc:MGI:2442112]	0.463526211	-1.109277172	0.001320333	0.005015299	yes	down	0.594	0.298	0.81	0.65	0.36	0.53	0.62	0.44	0.22	0.41	0.19	0.23
ENSMUSG00000006519	Cyba	cytochrome b-245, alpha polypeptide [Source:MGI Symbol;Acc:MGI:1316658]	3.691148811	1.884069902	1.63E-100	5.92E-98	yes	up	145.616	453.956	141.43	146.68	153.18	144.84	141.95	379.81	450.36	418.71	493.06	527.84
ENSMUSG00000015340	Cybb	cytochrome b-245, beta polypeptide [Source:MGI Symbol;Acc:MGI:88574]	11.37498812	3.507793133	0	0	yes	up	30.214	274.728	28.74	32.72	34.93	28.24	26.44	250.79	280.57	255.33	281.72	305.23
ENSMUSG00000039294	Cybc1	cytochrome b 245 chaperone 1 [Source:MGI Symbol;Acc:MGI:2384959]	2.02461	1.017644029	1.36E-29	6.16E-28	yes	up	25.914	45.136	24.64	29.07	27.07	23.04	25.75	50.31	49.13	37.19	45.08	43.97
ENSMUSG00000024365	Cyp21a1	cytochrome P450, family 21, subfamily a, polypeptide 1 [Source:MGI Symbol;Acc:MGI:88591]	20.0261057	4.323809995	0.002904669	0.010136204	yes	up	0.006	0.208	0	0	0	0	0.03	0	0.2	0.38	0.17	0.29
ENSMUSG00000025197	Cyp2c23	cytochrome P450, family 2, subfamily c, polypeptide 23 [Source:MGI Symbol;Acc:MGI:1888897]	0.211854134	-2.238856815	0.000452108	0.001911994	yes	down	0.544	0.106	0.47	0.52	0.74	0.31	0.68	0.09	0.18	0.17	0.09	0
ENSMUSG00000061740	Cyp2d22	cytochrome P450, family 2, subfamily d, polypeptide 22 [Source:MGI Symbol;Acc:MGI:1929474]	0.414455122	-1.270712202	3.00E-31	1.48E-29	yes	down	29.916	9.942	28.31	25.12	30.06	36.58	29.51	10	12.52	9.04	10.17	7.98
ENSMUSG00000052974	Cyp2f2	cytochrome P450, family 2, subfamily f, polypeptide 2 [Source:MGI Symbol;Acc:MGI:88608]	0.378896995	-1.400122395	1.65E-24	5.53E-23	yes	down	1895.548	586.056	1855.05	1832.46	1677.46	2096.14	2016.63	609.66	558.41	786.99	418.66	556.56
ENSMUSG00000070419	Cyp3a57	cytochrome P450, family 3, subfamily a, polypeptide 57 [Source:MGI Symbol;Acc:MGI:3646373]	0.092916168	-3.427926531	0.013442802	0.038648282	yes	down	0.176	0.014	0.03	0.45	0.1	0.21	0.09	0.04	0.03	0	0	0
ENSMUSG00000028713	Cyp4b1	cytochrome P450, family 4, subfamily b, polypeptide 1 [Source:MGI Symbol;Acc:MGI:103225]	0.345615189	-1.532761473	9.93E-52	1.03E-49	yes	down	515.248	152.16	554.02	445.32	474.11	581.75	521.04	140.03	181.74	129.85	175.71	133.47
ENSMUSG00000073424	Cyp4f15	cytochrome P450, family 4, subfamily f, polypeptide 15 [Source:MGI Symbol;Acc:MGI:2146921]	0.356461157	-1.488183216	7.55E-11	8.61E-10	yes	down	4.516	1.552	4.38	6.17	3.3	4.36	4.37	0.88	2.87	1.35	0.76	1.9
ENSMUSG00000003484	Cyp4f18	cytochrome P450, family 4, subfamily f, polypeptide 18 [Source:MGI	2.418071022	1.273856619	1.32E-25	4.74E-24	yes	up	10.43	21.078	10.71	11.11	10.03	11.73	8.57	22.71	21.5	19.07	16.88	25.23

	Symbol;Acc:MGI:1919304]																		
ENSMUSG00000079057 Cyp4v3	cytochrome P450, family 4, subfamily v, polypeptide 3 [Source:MGI Symbol;Acc:MGI:2142763]	2.089578029	1.063211633	7.06E-32	3.61E-30	yes	up	23.18	40.982	23.03	25.56	23.22	19.76	24.33	37.28	42.45	38.26	44.77	42.15
ENSMUSG00000018008 Cyth4	cytohesin 4 [Source:MGI Symbol;Acc:MGI:2441702]	4.239861547	2.084017154	5.96E-107	2.61E-104	yes	up	19.318	67.578	18.22	20.88	22.69	16.69	18.11	73.02	72.63	62.12	60.5	69.62
ENSMUSG00000062329 Cytl1	cytokine-like 1 [Source:MGI Symbol;Acc:MGI:2684993]	0.383153545	-1.38400544	1.48E-11	1.82E-10	yes	down	25.652	8.354	19.06	27.6	18.31	28.29	35	7.07	10.78	9.38	7.92	6.62
ENSMUSG00000097131 D230017M19Rik	RIKEN cDNA D230017M19 gene [Source:MGI Symbol;Acc:MGI:2445071]	0.494493139	-1.015977591	0.002121101	0.00766287	yes	down	1.316	3.498	1.21	1.41	1.54	1.27	1.15	0.48	0.94	0.32	15.45	0.3
ENSMUSG00000042498 D330045A20Rik	RIKEN cDNA D330045A20 gene [Source:MGI Symbol;Acc:MGI:2147848]	9.629921719	3.267524071	2.07E-20	5.44E-19	yes	up	0.11	0.926	0.1	0.09	0.12	0.09	0.15	0.77	0.82	1.01	1.03	1
ENSMUSG00000037813 D630003M21Rik	RIKEN cDNA D630003M21 gene [Source:MGI Symbol;Acc:MGI:3606579]	2.369440629	1.244546512	0.000177065	0.000816017	yes	up	0.564	1.086	0.35	0.4	0.51	0.72	0.84	0.59	1.21	1.41	0.73	1.49
ENSMUSG00000085772 D630024D03Rik	RIKEN cDNA D630024D03 gene [Source:MGI Symbol;Acc:MGI:3041224]	0.495182314	-1.013968307	0.006050983	0.019422639	yes	down	4.134	1.67	4.31	4.89	2.84	4.35	4.28	1.8	1.63	2.62	0.81	1.49
ENSMUSG00000031294 D630029K05Rik	RIKEN cDNA D630029K05 gene [Source:MGI Symbol;Acc:MGI:2143561]	0.338360654	-1.563366281	2.04E-05	0.000112384	yes	down	2.054	0.546	1.54	4.03	1.43	2.12	1.15	0.35	0.9	0.53	0.4	0.55
ENSMUSG00000063455 D630045J12Rik	RIKEN cDNA D630045J12 gene [Source:MGI Symbol;Acc:MGI:2669829]	0.484879634	-1.044301437	1.44E-12	1.98E-11	yes	down	3.732	1.564	4.57	3.04	3.11	4.21	3.73	1.65	1.26	1.21	1.75	1.95
ENSMUSG00000090891 D6Ertd527e	DNA segment, Chr 6, ERATO Doi 527, expressed [Source:MGI Symbol;Acc:MGI:1261919]	2.934000614	1.552869173	7.06E-05	0.00035204	yes	up	0.362	0.918	0.28	0.42	0.35	0.44	0.32	0.39	0.95	0.96	1.16	1.13
ENSMUSG00000100029 D7Ertd128e	DNA segment, Chr 7, ERATO Doi 128, expressed [Source:MGI Symbol;Acc:MGI:1098716]	9.920101054	3.310354817	5.35E-07	3.82E-06	yes	up	0.21	1.768	0	0.1	0.47	0.16	0.32	1.47	2.3	1.42	2.78	0.87
ENSMUSG00000085171 D830026I12Rik	RIKEN cDNA D830026I12 gene [Source:MGI Symbol;Acc:MGI:2442531]	0.354976184	-1.494205859	1.40E-06	9.37E-06	yes	down	2.586	0.798	2.75	2.56	2.69	3.25	1.68	0.79	1.09	0.72	0.43	0.96
ENSMUSG00000097083 D930019O06Rik	RIKEN cDNA D930019O06 [Source:MGI Symbol;Acc:MGI:3043219]	26.76315006	4.742176028	8.66E-05	0.000424896	yes	up	0.006	0.154	0	0	0	0.03	0	0.34	0.06	0.14	0.09	0.14
ENSMUSG00000028519 Dab1	disabled 1 [Source:MGI Symbol;Acc:MGI:108554]	0.151692732	-2.720776135	0.000140049	0.000660053	yes	down	0.278	0.02	0.62	0.28	0.25	0.16	0.08	0.04	0.01	0.02	0.02	0.01
ENSMUSG00000039206 Daglb	diacylglycerol lipase, beta [Source:MGI Symbol;Acc:MGI:2442032]	2.48600528	1.31382936	1.57E-33	8.73E-32	yes	up	6.52	13.522	5.79	6.57	6.51	6.64	7.09	10.31	13.99	14.44	14.04	14.83
ENSMUSG00000053161 Daw1	dynein assembly factor with WDR repeat domains 1 [Source:MGI Symbol;Acc:MGI:1923089]	0.40717729	-1.296270997	0.000492529	0.002067423	yes	down	2.12	0.72	3.44	1.63	1.15	2.4	1.98	0.8	0.5	0.99	0.45	0.86
ENSMUSG00000059824 Dbp	D site albumin promoter binding protein [Source:MGI Symbol;Acc:MGI:94866]	11.65745218	3.543180606	2.59E-18	5.85E-17	yes	up	3.54	24.098	1.55	3.1	5.07	5.24	2.74	21.9	12.11	24.46	26.15	35.87
ENSMUSG00000035910 Dcdc2a	doublecortin domain containing 2a [Source:MGI	0.279427889	-1.839452074	6.45E-13	9.08E-12	yes	down	7.514	1.264	9.42	7.86	2.96	7.31	10.02	0.97	0.87	2.91	0.65	0.92

		Symbol;Acc:MGI:2652818]																		
ENSMUSG00000078552	Dcdc2b	doublecortin domain containing 2b [Source:MGI Symbol;Acc:MGI:2686212]	0.47241801	-1.081864129	0.004097964	0.013736421	yes	down	5.688	2.088	3.04	6.23	4.84	8.37	5.96	1.54	2.85	3.79	0.81	1.45
ENSMUSG00000074981	Dcdc5	doublecortin domain containing 5 [Source:MGI Symbol;Acc:MGI:3045363]	0.445180749	-1.167536886	0.007775404	0.024199687	yes	down	1.118	0.45	1.16	1.16	0.69	1.32	1.26	0.26	0.7	0.74	0.09	0.46
ENSMUSG00000022303	Dcstamp	dendrocyte expressed seven transmembrane protein [Source:MGI Symbol;Acc:MGI:1923016]	33.40365978	5.061934271	2.88E-41	2.06E-39	yes	up	0.248	8.712	0.05	0.39	0.22	0.05	0.53	8.08	5.51	8.49	5.01	16.47
ENSMUSG00000020182	Ddc	dopa decarboxylase [Source:MGI Symbol;Acc:MGI:94876]	0.327625232	-1.609881625	2.30E-07	1.73E-06	yes	down	2.962	0.694	3.52	2.99	2.54	3.74	2.02	0.48	1.02	0.91	0.27	0.79
ENSMUSG00000037697	Ddhd1	DDHD domain containing 1 [Source:MGI Symbol;Acc:MGI:2150302]	2.525900054	1.336797555	8.84E-49	8.34E-47	yes	up	21.49	45.91	22.2	21.93	21.92	18.68	22.72	47.62	53.12	40.87	43.19	44.75
ENSMUSG00000020108	Ddit4	DNA-damage-inducible transcript 4 [Source:MGI Symbol;Acc:MGI:1921997]	0.402757597	-1.312016293	1.88E-07	1.44E-06	yes	down	80.806	27.594	71.03	72.29	73.09	75.33	112.29	25.59	30.48	41.72	18.18	22
ENSMUSG00000028175	Depdc1a	DEP domain containing 1a [Source:MGI Symbol;Acc:MGI:1923381]	3.635044525	1.861973036	3.11E-08	2.65E-07	yes	up	0.46	1.532	0.53	0.36	0.44	0.61	0.36	1.71	1.61	0.97	0.89	2.48
ENSMUSG00000009092	Derl3	Der1-like domain family, member 3 [Source:MGI Symbol;Acc:MGI:1917627]	8.946235456	3.161280729	2.36E-33	1.31E-31	yes	up	4.606	23.314	5.18	3.95	6.46	3.38	4.06	35.41	19.19	21.28	14.04	26.65
ENSMUSG00000026208	Des	desmin [Source:MGI Symbol;Acc:MGI:94885]	0.462055395	-1.113862272	1.43E-11	1.76E-10	yes	down	50.604	19.38	45.45	66.72	38.27	52.92	49.66	17.54	17.63	26.78	13.93	21.02
ENSMUSG00000062393	Dgkk	diacylglycerol kinase kappa [Source:MGI Symbol;Acc:MGI:3580254]	15.77636756	3.979693164	0.000979658	0.003848193	yes	up	0.006	0.064	0	0	0	0.01	0.02	0.1	0.03	0.11	0.01	0.07
ENSMUSG00000058454	Dhcr7	7-dehydrocholesterol reductase [Source:MGI Symbol;Acc:MGI:1298378]	2.002468677	1.001779676	1.03E-14	1.72E-13	yes	up	11.166	17.478	11.15	14.65	7.72	11.22	11.09	16.36	19.66	15.43	16.49	19.45
ENSMUSG00000007682	Dio2	deiodinase, iodothyronine, type II [Source:MGI Symbol;Acc:MGI:1338833]	0.414731832	-1.269749311	0.000604035	0.00247864	yes	down	0.566	0.196	0.38	0.83	0.57	0.54	0.51	0.28	0.12	0.32	0.1	0.16
ENSMUSG00000028031	Dkk2	dickkopf WNT signaling pathway inhibitor 2 [Source:MGI Symbol;Acc:MGI:1890663]	2.036306059	1.025954417	0.000744677	0.003001694	yes	up	0.446	0.768	0.43	0.53	0.35	0.55	0.37	0.46	0.74	0.94	0.63	1.07
ENSMUSG00000037544	Dlgap5	DLG associated protein 5 [Source:MGI Symbol;Acc:MGI:2183453]	3.195621449	1.676096518	9.92E-08	7.87E-07	yes	up	3.2	3.008	2.03	8.79	2.33	0.46	2.39	3.2	2.27	2.05	2.17	5.35
ENSMUSG00000043753	Dmrta1	doublesex and mab-3 related transcription factor like family A1 [Source:MGI Symbol;Acc:MGI:2653627]	3.359885669	1.748412141	0.016945048	0.047178255	yes	up	0.052	0.234	0.06	0.05	0.08	0.07	0	0.21	0.5	0.2	0.15	0.11
ENSMUSG00000041268	Dmxl2	Dmx-like 2 [Source:MGI Symbol;Acc:MGI:2444630]	2.63549202	1.398072324	6.72E-39	4.50E-37	yes	up	7.646	16.86	7.2	6.73	9.5	7.36	7.44	14.16	21.56	16.17	15.58	16.83
ENSMUSG00000036875	Dna2	DNA replication helicase/nuclease 2 [Source:MGI Symbol;Acc:MGI:2443732]	2.142624024	1.099378716	3.67E-12	4.82E-11	yes	up	3.03	5.53	2.68	3.89	3.37	2.91	2.3	4.25	5.89	6.21	4.97	6.33
ENSMUSG00000031831	Dnaaf1	dynein, axonemal assembly factor 1 [Source:MGI Symbol;Acc:MGI:1915520]	0.489716325	-1.029981804	0.000598469	0.00245721	yes	down	3.208	1.328	3.08	2.93	2.74	3.26	4.03	0.96	1.21	2.51	0.58	1.38
ENSMUSG00000019027	Dnah1	dynein, axonemal, heavy chain 1 [Source:MGI	0.383680912	-1.382021102	2.35E-08	2.04E-07	yes	down	2.876	0.72	2.3	2.6	3.87	2.42	3.19	0.43	0.69	1.24	0.5	0.74

	Symbol;Acc:MGI:107721]																	
ENSMUSG00000038011 Dnah10	dynein, axonemal, heavy chain 10 [Source:MGI Symbol;Acc:MGI:1860299]	0.472939978	-1.080270996	0.001933836	0.007066205	yes	down	3.618	1.394	4.57	2.95	2.37	4.09	4.11	0.94	1.41	2.29	0.54	1.79
ENSMUSG00000021879 Dnah12	dynein, axonemal, heavy chain 12 [Source:MGI Symbol;Acc:MGI:107720]	0.442532396	-1.176145021	1.28E-09	1.29E-08	yes	down	3.468	1.418	3.2	3.2	3.9	3.68	3.36	1.83	1.15	2.38	0.66	1.07
ENSMUSG00000052861 Dnah6	dynein, axonemal, heavy chain 6 [Source:MGI Symbol;Acc:MGI:107744]	0.484828832	-1.044452598	0.000296036	0.001305045	yes	down	6.616	2.708	6.95	5.95	5.46	6.68	8.04	2.45	2.26	4.74	1.58	2.51
ENSMUSG00000101337 Dnah7c	dynein, axonemal, heavy chain 7C [Source:MGI Symbol;Acc:MGI:3639762]	0.41728417	-1.260897904	1.25E-05	7.15E-05	yes	down	12.022	0.314	0.87	1.03	17.85	39.35	1.01	0.22	0.26	0.62	0.19	0.28
ENSMUSG00000056752 Dnah9	dynein, axonemal, heavy chain 9 [Source:MGI Symbol;Acc:MGI:1289279]	0.492714069	-1.021177427	0.00273341	0.009602807	yes	down	4.764	2.072	4.26	4.26	5.68	4.44	5.18	1.8	1.85	3.82	0.81	2.08
ENSMUSG00000025279 Dnase1l3	deoxyribonuclease 1-like 3 [Source:MGI Symbol;Acc:MGI:1314633]	9.073615029	3.181677451	8.85E-31	4.26E-29	yes	up	0.698	5.304	0.54	0.77	1.03	0.6	0.55	7.62	3.63	3.43	3.67	8.17
ENSMUSG00000003812 Dnase2a	deoxyribonuclease II alpha [Source:MGI Symbol;Acc:MGI:1329019]	2.415944036	1.272587036	7.18E-36	4.27E-34	yes	up	15.44	30.674	13.54	16.53	19.59	13.31	14.23	27.29	32.34	27.77	33.11	32.86
ENSMUSG00000036766 Dner	delta/notch-like EGF repeat containing [Source:MGI Symbol;Acc:MGI:2152889]	0.2586464	-1.950946982	0.002074781	0.007512121	yes	down	0.34	0.076	0.37	0.51	0.07	0.35	0.4	0.03	0.1	0.16	0.03	0.06
ENSMUSG00000078190 Dnm3os	dynamin 3, opposite strand [Source:MGI Symbol;Acc:MGI:3052332]	0.422414754	-1.243267868	0.000604275	0.002478961	yes	down	0.396	0.288	0.49	0.15	0.37	0.5	0.47	0.12	0.75	0.12	0.3	0.15
ENSMUSG00000073242 Dnmt3aos	DNA methyltransferase 3A, opposite strand [Source:MGI Symbol;Acc:MGI:3641689]	19.90837871	4.315303831	0.011273009	0.033267572	yes	up	0	0.156	0	0	0	0	0	0.27	0	0.08	0.18	0.25
ENSMUSG00000082079 Dnmt3c	DNA methyltransferase 3C [Source:MGI Symbol;Acc:MGI:3649996]	0.062146878	-4.00817426	0.010526581	0.031367367	yes	down	0.128	0.006	0.34	0.06	0	0.08	0.16	0	0	0	0.03	0
ENSMUSG00000020848 Doc2b	double C2, beta [Source:MGI Symbol;Acc:MGI:1100497]	0.20193793	-2.308016176	1.32E-09	1.33E-08	yes	down	1.038	0.178	0.83	1.37	1.02	0.5	1.47	0.17	0.12	0.26	0.24	0.1
ENSMUSG00000038608 Dock10	dedicator of cytokinesis 10 [Source:MGI Symbol;Acc:MGI:2146320]	2.172710574	1.119496007	4.32E-51	4.37E-49	yes	up	17.122	29.83	16.17	16.48	20.12	18.4	14.44	31.76	31.92	29.46	26.75	29.26
ENSMUSG00000020143 Dock2	dedicator of cyto-kinesis 2 [Source:MGI Symbol;Acc:MGI:2149010]	2.677067599	1.420653569	2.99E-86	7.78E-84	yes	up	12.766	28.804	12.02	13.61	13.61	12.94	11.65	29.18	27.79	28.61	27.25	31.19
ENSMUSG00000039716 Dock3	dedicator of cyto-kinesis 3 [Source:MGI Symbol;Acc:MGI:2429763]	2.808148863	1.489619417	0.002634631	0.009292367	yes	up	0.08	5.188	0.12	0.12	0.09	0.03	0.04	0.29	24.92	0.09	0.21	0.43
ENSMUSG00000035711 Dok3	docking protein 3 [Source:MGI Symbol;Acc:MGI:1351490]	2.279257857	1.188564149	2.32E-12	3.12E-11	yes	up	10.292	20.304	8.92	12.69	10.11	11.41	8.33	24.32	22.25	15.56	15.54	23.85
ENSMUSG00000027560 Dok5	docking protein 5 [Source:MGI Symbol;Acc:MGI:1924079]	5.557023969	2.474312463	0.006800956	0.021552128	yes	up	0.054	0.254	0	0.16	0.08	0	0.03	0.33	0.22	0.21	0.2	0.31
ENSMUSG00000073514 Dok6	docking protein 6 [Source:MGI Symbol;Acc:MGI:3639495]	11.99591564	3.584471376	0.002982733	0.010377141	yes	up	0.004	0.034	0.02	0	0	0	0	0.02	0.02	0.06	0.03	0.04
ENSMUSG00000019278 Dpep1	dipeptidase 1 [Source:MGI Symbol;Acc:MGI:94917]	0.433173251	-1.206983938	4.40E-21	1.19E-19	yes	down	107.346	39.304	120.02	94.79	110.04	101.91	109.97	40.9	44.93	43.14	41.62	25.93
ENSMUSG00000053687 Dpep2	dipeptidase 2 [Source:MGI Symbol;Acc:MGI:2442042]	5.54273423	2.470597833	4.58E-68	7.85E-66	yes	up	7.944	35.392	8.48	9.33	8.47	6.09	7.35	25.89	42.74	33.81	38.08	36.44
ENSMUSG00000115067 Dpep2	dipeptidase 2 [Source:MGI Symbol;Acc:MGI:2442042]	3.89892327	1.963075763	0.00054422	0.002258722	yes	up	1.068	3.484	0.64	1.7	0	2.2	0.8	2.41	1.44	3.52	4.18	5.87
ENSMUSG00000026958 Dpp7	dipeptidylpeptidase 7 [Source:MGI Symbol;Acc:MGI:1933213]	2.530569956	1.339462357	5.21E-42	3.88E-40	yes	up	15.43	32.202	21.4	13.51	13.88	12.79	15.57	30.78	34.88	34.01	29.42	31.92
ENSMUSG00000046323 Dppa3	developmental pluripotency-associated 3 [Source:MGI	20.11795896	4.330412041	0.000293138	0.001293702	yes	up	0.06	0.908	0.15	0.15	0	0	0	0.87	0.68	0.33	1.97	0.69

	Symbol;Acc:MGI:1920958]																		
ENSMUSG00000029168 Dpysl5	dihydropyrimidinase-like 5 [Source:MGI Symbol;Acc:MGI:1929772]	0.239081178	-2.064427541	4.95E-25	1.72E-23	yes	down	4.9	0.912	5.58	2.96	3.83	7.14	4.99	1.01	1.21	0.67	0.94	0.73
ENSMUSG00000029005 Draxin	dorsal inhibitory axon guidance protein [Source:MGI Symbol;Acc:MGI:1917683]	0.458904687	-1.123733554	0.002008299	0.007307123	yes	down	0.37	0.144	0.41	0.41	0.31	0.37	0.35	0.12	0.16	0.12	0.08	0.24
ENSMUSG00000050272 Dscam	DS cell adhesion molecule [Source:MGI Symbol;Acc:MGI:1196281]	10.40558134	3.379285663	6.34E-07	4.47E-06	yes	up	0.014	0.262	0.01	0.02	0.01	0.03	0	0.24	0.07	0.38	0.08	0.54
ENSMUSG00000037474 Dtl	denticleless E3 ubiquitin protein ligase [Source:MGI Symbol;Acc:MGI:1924093]	2.413457572	1.271101465	2.98E-06	1.91E-05	yes	up	0.608	1.208	0.41	0.83	0.83	0.58	0.39	1.32	0.94	1.01	1.21	1.56
ENSMUSG00000033268 Duox1	dual oxidase 1 [Source:MGI Symbol;Acc:MGI:2139422]	21.05932081	4.396387003	0.01022193	0.030587235	yes	up	0	0.036	0	0	0	0	0	0	0.06	0.02	0.02	0.08
ENSMUSG00000024190 Dusp1	dual specificity phosphatase 1 [Source:MGI Symbol;Acc:MGI:105120]	0.375271082	-1.413994973	6.91E-06	4.15E-05	yes	down	404.696	126.096	273.16	698.78	303.14	474.53	273.87	144.28	158.37	134.82	97.57	95.44
ENSMUSG00000021768 Dusp13	dual specificity phosphatase 13 [Source:MGI Symbol;Acc:MGI:1351599]	17.65130912	4.141703281	2.03E-05	0.00011231	yes	up	0.136	0.98	0.06	0	0.56	0	0.06	0.3	0.76	0.59	0.74	2.51
ENSMUSG00000037887 Dusp8	dual specificity phosphatase 8 [Source:MGI Symbol;Acc:MGI:106626]	0.432481634	-1.209289227	4.50E-11	5.26E-10	yes	down	10.138	3.746	10.11	12.32	6.45	14.62	7.19	3.87	4.1	2.95	4.14	3.67
ENSMUSG00000029757 Dync1i1	dynein cytoplasmic 1 intermediate chain 1 [Source:MGI Symbol;Acc:MGI:107743]	0.300719803	-1.733508219	4.41E-07	3.19E-06	yes	down	3.026	0.894	2.54	2.9	1.86	5.05	2.78	0.7	0.4	1.96	0.44	0.97
ENSMUSG00000030345 Dyrk4	dual-specificity tyrosine-(Y)-phosphorylation regulated kinase 4 [Source:MGI Symbol;Acc:MGI:1330292]	3.856845637	1.947421406	0.004392161	0.014603713	yes	up	0.158	0.554	0.1	0.07	0.03	0.11	0.48	1.25	0.44	0.44	0.27	0.37
ENSMUSG00000086843 E030013I19Rik	RIKEN cDNA E030013I19 gene [Source:MGI Symbol;Acc:MGI:2443735]	0.446572672	-1.163033126	3.57E-12	4.70E-11	yes	down	5.676	2.11	5.6	4.95	5.59	7.87	4.37	2.16	1.96	2.07	2.36	2
ENSMUSG00000097706 E030037K01Rik	RIKEN cDNA E030037K01 gene [Source:MGI Symbol;Acc:MGI:3704310]	13.81819706	3.788497486	0.007017155	0.022129199	yes	up	0.016	0.182	0	0.04	0	0	0.04	0.11	0.21	0.21	0	0.38
ENSMUSG00000066170 E230001N04Rik	RIKEN cDNA E230001N04 gene [Source:MGI Symbol;Acc:MGI:2443549]	0.442832261	-1.175167767	0.00485796	0.015973909	yes	down	0.89	0.348	0.41	1.38	0.93	0.9	0.83	0.53	0.26	0.33	0.36	0.26
ENSMUSG00000051832 E230016K23Rik	RIKEN cDNA E230016K23 gene [Source:MGI Symbol;Acc:MGI:3041204]	3.757759771	1.90987284	1.53E-08	1.37E-07	yes	up	0.62	2.48	0.31	0.9	0.42	0.57	0.9	2.75	3.81	1.93	1.87	2.04
ENSMUSG00000109251 E230032D23Rik	RIKEN cDNA E230032D23 gene [Source:MGI Symbol;Acc:MGI:3041205]	11.21779856	3.487717677	1.24E-08	1.12E-07	yes	up	0.822	7.232	0.78	1.5	1.83	0	0	6.7	7.71	8.47	6.25	7.03
ENSMUSG00000046179 E2f8	E2F transcription factor 8 [Source:MGI Symbol;Acc:MGI:1922038]	3.40809558	1.768965796	1.03E-11	1.30E-10	yes	up	0.72	2.032	0.75	1.04	0.82	0.46	0.53	2.31	2.33	1.74	1.4	2.38
ENSMUSG00000110264 E330018M18Rik	RIKEN cDNA E330018M18 gene [Source:MGI Symbol;Acc:MGI:3041231]	0.14728856	-2.763282715	0.00077195	0.003095895	yes	down	2.068	0.068	0.65	0.33	0.83	5.86	2.67	0.13	0.08	0	0	0.13
ENSMUSG00000073538 E330020D12Rik	Riken cDNA E330020D12 gene [Source:MGI Symbol;Acc:MGI:3761270]	2.440657836	1.287270053	0.000977124	0.00383965	yes	up	1.114	3.586	1.58	0.75	0.64	1.92	0.68	5	3.96	3.89	1.34	3.74

ENSMUSG00000022838 Eaf2	ELL associated factor 2 [Source:MGI Symbol;Acc:MGI:2146616]	6.301464149	2.655687079	3.12E-13	4.54E-12	yes	up	5.142	16.286	2.24	2.31	17.78	0.76	2.62	37.24	10.66	12.71	8.43	12.39
ENSMUSG00000072601 Ear1	eosinophil-associated, ribonuclease A family, member 1 [Source:MGI Symbol;Acc:MGI:108021]	9.154158033	3.194427198	2.61E-85	6.59E-83	yes	up	79.96	623.348	87.22	63.74	98.06	59.33	91.45	414.13	782.55	657.53	696.12	566.41
ENSMUSG00000072596 Ear2	eosinophil-associated, ribonuclease A family, member 2 [Source:MGI Symbol;Acc:MGI:108020]	4.681925739	2.227102052	2.61E-20	6.79E-19	yes	up	230.682	921.016	249.45	183.42	321.08	136.52	262.94	650.82	1134.82	958.61	1049.12	811.71
ENSMUSG00000062148 Ear6	eosinophil-associated, ribonuclease A family, member 6 [Source:MGI Symbol;Acc:MGI:1890463]	15.7359806	3.975995179	4.17E-13	6.02E-12	yes	up	0.454	6.078	0	1.22	0.57	0.24	0.24	7.86	8.46	4.5	3.27	6.3
ENSMUSG00000022053 Ebf2	early B cell factor 2 [Source:MGI Symbol;Acc:MGI:894332]	0.199164159	-2.327970049	0.001428419	0.005384796	yes	down	0.302	0.05	0.22	0.55	0.49	0.04	0.21	0.01	0.02	0.13	0.02	0.07
ENSMUSG00000003206 Ebi3	Epstein-Barr virus induced gene 3 [Source:MGI Symbol;Acc:MGI:1354171]	5.531958247	2.467790268	7.32E-56	8.57E-54	yes	up	3.274	15.316	2.79	3.65	2.93	3.22	3.78	15.8	15.27	15.1	16.06	14.35
ENSMUSG00000043631 Ecm2	extracellular matrix protein 2, female organ and adipocyte specific [Source:MGI Symbol;Acc:MGI:3039578]	0.461247042	-1.116388436	2.74E-26	1.02E-24	yes	down	15.74	6.108	14.41	17.06	14.19	17.91	15.13	5.77	6.21	7.24	5.93	5.39
ENSMUSG00000027699 Ect2	ect2 oncogene [Source:MGI Symbol;Acc:MGI:95281]	2.012670896	1.009111288	1.09E-09	1.10E-08	yes	up	2.808	3.816	2.45	2.37	3.15	2.9	3.17	3.62	4.2	4.22	2.53	4.51
ENSMUSG00000071392 Ect2l	epithelial cell transforming sequence 2 oncogene-like [Source:MGI Symbol;Acc:MGI:3641723]	0.434744074	-1.201761732	0.00015465	0.000722667	yes	down	3.292	1.314	3.38	4.14	1.58	3.4	3.96	2.27	0.94	1.61	0.68	1.07
ENSMUSG00000059327 Eda	ectodysplasin-A [Source:MGI Symbol;Acc:MGI:1195272]	0.268171629	-1.898771479	3.54E-23	1.10E-21	yes	down	1.854	0.416	2.09	1.6	1.68	2.1	1.8	0.39	0.46	0.43	0.47	0.33
ENSMUSG00000030104 Edem1	ER degradation enhancer, mannosidase alpha-like 1 [Source:MGI Symbol;Acc:MGI:2180139]	2.633291096	1.396867012	6.14E-72	1.20E-69	yes	up	24.866	59.54	22.72	24.35	28	25.94	23.32	74.51	56.59	53.8	53.99	58.81
ENSMUSG00000038312 Edem2	ER degradation enhancer, mannosidase alpha-like 2 [Source:MGI Symbol;Acc:MGI:1915540]	2.080950316	1.05724252	5.88E-48	5.45E-46	yes	up	30.994	53.258	28.87	30.86	32.21	32.31	30.72	54.15	57	50.83	48.19	56.12
ENSMUSG00000034488 Edil3	EGF-like repeats and discoidin I-like domains 3 [Source:MGI Symbol;Acc:MGI:1329025]	0.411147039	-1.282273657	7.62E-23	2.31E-21	yes	down	8.456	2.952	10.35	7.61	7.88	8.18	8.26	3.24	3.48	2.66	2.98	2.4
ENSMUSG00000022122 Ednrb	endothelin receptor type B [Source:MGI Symbol;Acc:MGI:102720]	0.335801451	-1.574319629	7.78E-30	3.57E-28	yes	down	44.402	12.616	45.17	42.02	45.67	50.93	38.22	10.97	16.34	9.45	15.97	10.35
ENSMUSG00000080115 Eef1akmt3	EEF1A lysine methyltransferase 3 [Source:MGI Symbol;Acc:MGI:3645330]	3.067722951	1.617168198	0.012507436	0.036364275	yes	up	0.182	0.468	0.15	0.02	0.45	0.02	0.27	0.54	0.39	0.73	0.26	0.42
ENSMUSG00000020562 Efcab10	EF-hand calcium binding domain 10 [Source:MGI Symbol;Acc:MGI:1922290]	0.369718151	-1.43550222	0.002728745	0.009587992	yes	down	7.294	2.278	8.4	10.81	2.83	4.53	9.9	1.69	1.44	5.06	1.03	2.17
ENSMUSG00000020467 Efemp1	epidermal growth factor-containing fibulin-like extracellular matrix protein 1 [Source:MGI Symbol;Acc:MGI:1339998]	0.46879856	-1.092959957	6.83E-32	3.50E-30	yes	down	131.132	52.23	139.38	121.97	131.2	132.44	130.67	50.56	55.32	50.06	62.79	42.42
ENSMUSG00000025038 Efhc2	EF-hand domain (C-terminal) containing 2 [Source:MGI Symbol;Acc:MGI:1921655]	0.354269287	-1.497081697	0.013358222	0.038472253	yes	down	1.918	0.158	0.55	0.69	7.74	0.32	0.29	0.32	0.14	0.24	0.07	0.02

ENSMUSG00000028039 Efna3	ephrin A3 [Source:MGI Symbol;Acc:MGI:106644]	2.858622144	1.515319935	0.000197378	0.00090148	yes	up	4.122	8.408	1.3	0.09	11.76	5.7	1.76	22.35	1.34	5.98	6.5	5.87
ENSMUSG00000031217 Efnb1	ephrin B1 [Source:MGI Symbol;Acc:MGI:102708]	0.452867334	-1.142839614	5.63E-34	3.15E-32	yes	down	87.048	33.5	92.49	87.27	71.21	96.39	87.88	34.2	38.11	28.11	34.04	33.04
ENSMUSG00000001300 Efnb2	ephrin B2 [Source:MGI Symbol;Acc:MGI:105097]	0.479157306	-1.061428728	9.30E-32	4.71E-30	yes	down	89.634	36.062	96.17	87.3	81.78	106.63	76.29	35.61	42.64	32.26	36.73	33.07
ENSMUSG00000063600 Egfem1	EGF-like and EMI domain containing 1 [Source:MGI Symbol;Acc:MGI:1922990]	5.963968546	2.576272649	5.58E-26	2.05E-24	yes	up	1.076	4.11	1.57	0.76	0.8	0.53	1.72	3.51	3.42	3.4	5.26	4.96
ENSMUSG00000020122 Egfr	epidermal growth factor receptor [Source:MGI Symbol;Acc:MGI:95294]	0.404128154	-1.307115233	4.61E-31	2.26E-29	yes	down	8.894	2.78	9.85	9.9	8.3	7.4	9.02	2.45	3.37	2.97	2.89	2.22
ENSMUSG00000038418 Egr1	early growth response 1 [Source:MGI Symbol;Acc:MGI:95295]	0.147927593	-2.757036907	3.88E-13	5.61E-12	yes	down	60.824	7.46	53.7	109.88	32.14	73.39	35.01	6.81	6.5	11.99	5.95	6.05
ENSMUSG00000037868 Egr2	early growth response 2 [Source:MGI Symbol;Acc:MGI:95296]	2.299875818	1.201555965	3.50E-15	6.13E-14	yes	up	3.266	6.39	3.13	4.18	2.39	3.13	3.5	5.42	6.62	5.86	6.81	7.24
ENSMUSG00000006390 Elovl1	elongation of very long chain fatty acids (FEN1/Elo2, SUR4/Elo3, yeast)-like 1 [Source:MGI Symbol;Acc:MGI:1858959]	2.004349272	1.00313393	8.59E-15	1.46E-13	yes	up	232.526	409.718	195	311.52	211.9	208.85	235.36	314.1	398.12	422.22	454.23	459.92
ENSMUSG00000039055 Eme1	essential meiotic structure-specific endonuclease 1 [Source:MGI Symbol;Acc:MGI:3576783]	2.178148813	1.123102523	0.010657259	0.031703828	yes	up	0.382	0.69	0.25	0.25	0.26	0.6	0.55	0.87	0.67	0.56	0.34	1.01
ENSMUSG00000024053 Emilin2	elastin microfibril interfacer 2 [Source:MGI Symbol;Acc:MGI:2389136]	2.046922416	1.033456421	6.89E-12	8.83E-11	yes	up	7.104	10.866	4.6	7.24	6.72	11.69	5.27	15.99	8.98	8.91	9.9	10.55
ENSMUSG00000040212 Emp3	epithelial membrane protein 3 [Source:MGI Symbol;Acc:MGI:1098729]	2.139879889	1.09752982	1.76E-27	7.07E-26	yes	up	114.188	199.088	113.55	111.69	116.55	111.69	117.46	155.17	202.98	193.57	205.96	237.76
ENSMUSG00000026814 Eng	endoglin [Source:MGI Symbol;Acc:MGI:95392]	0.49060366	-1.027370098	1.97E-18	4.50E-17	yes	down	269.592	112.51	304.89	215.76	230.2	348.88	248.23	104.17	138.84	92.6	124.2	102.74
ENSMUSG00000028445 Enho	energy homeostasis associated [Source:MGI Symbol;Acc:MGI:1916888]	0.353040845	-1.502092989	1.76E-13	2.64E-12	yes	down	11.816	3.54	11.11	11.33	13.91	10.02	12.71	3.06	4.48	2.03	4.02	4.11
ENSMUSG00000026679 Enkur	enkurin, TRPC channel interacting protein [Source:MGI Symbol;Acc:MGI:1918483]	0.465007268	-1.104674829	7.62E-08	6.14E-07	yes	down	19.024	7.626	19.62	22.76	15.96	16.26	20.52	7.69	6.54	9.26	5.18	9.46
ENSMUSG00000004267 Eno2	enolase 2, gamma neuronal [Source:MGI Symbol;Acc:MGI:95394]	2.737980735	1.453112296	1.82E-10	1.99E-09	yes	up	1.464	2.436	1.13	1.68	1.58	1.46	1.47	3.27	2.51	1.87	1.35	3.18
ENSMUSG00000048029 Eno4	enolase 4 [Source:MGI Symbol;Acc:MGI:2441717]	0.376599267	-1.408897901	5.48E-10	5.73E-09	yes	down	7.734	2.46	8.14	8.07	5.18	8.18	9.1	2.74	2	3.66	1.46	2.44
ENSMUSG00000037370 Enpp1	ectonucleotide pyrophosphatase/phosphodiesterase 1 [Source:MGI Symbol;Acc:MGI:97370]	3.923500216	1.97214128	5.64E-63	8.00E-61	yes	up	2.594	10.23	2.39	3.02	3.08	1.71	2.77	10.55	10.61	9.91	11.08	9
ENSMUSG00000038173 Enpp6	ectonucleotide pyrophosphatase/phosphodiesterase 6 [Source:MGI Symbol;Acc:MGI:2445171]	0.301025829	-1.732040815	5.36E-10	5.62E-09	yes	down	1.64	0.476	1.47	2.64	1.06	1.22	1.81	0.34	0.33	0.52	1.03	0.16
ENSMUSG00000050439 Enthd1	ENTH domain containing 1 [Source:MGI Symbol;Acc:MGI:2686088]	23.92064054	4.580184117	8.68E-05	0.000425576	yes	up	0.012	0.256	0.03	0.03	0	0	0	0.22	0.55	0.17	0.16	0.18
ENSMUSG00000041608 Entpd3	ectonucleoside triphosphate diphosphohydrolase 3 [Source:MGI Symbol;Acc:MGI:1321386]	0.350588168	-1.512150788	2.98E-08	2.55E-07	yes	down	1.624	0.462	1.55	1.85	1.37	1.4	1.95	0.5	0.51	0.63	0.23	0.44
ENSMUSG00000024140 Epas1	endothelial PAS domain protein 1 [Source:MGI Symbol;Acc:MGI:109169]	0.466223052	-1.100907756	1.36E-26	5.18E-25	yes	down	1011.438	400.052	1143.64	881.01	945.51	1031.41	1055.62	397.2	471.85	343.8	453.22	334.19
ENSMUSG00000113346 Eprn	ephemeron, early developmental lncRNA [Source:MGI Symbol;Acc:MGI:3583897]	0.075367686	-3.729910082	0.000311185	0.001366204	yes	down	0.584	0.038	0.29	1.19	0.59	0.55	0.3	0.1	0.09	0	0	0

ENSMUSG00000022014 Epsti1	epithelial stromal interaction 1 (breast) [Source:MGI Symbol;Acc:MGI:1915168]	2.006372374	1.004589388	2.60E-14	4.21E-13	yes	up	12.068	20.182	9.96	13.36	12.66	12.28	12.08	22.32	17.28	22.18	16.36	22.77
ENSMUSG00000028967 Errfi1	ERBB receptor feedback inhibitor 1 [Source:MGI Symbol;Acc:MGI:1921405]	0.230038941	-2.120049994	2.53E-40	1.77E-38	yes	down	306.952	59.678	339.73	333.29	239.51	261.75	360.48	65.8	56.48	76.72	60.17	39.22
ENSMUSG00000022034 Esco2	establishment of sister chromatid cohesion N-acetyltransferase 2 [Source:MGI Symbol;Acc:MGI:1919238]	2.171124044	1.118442154	0.000972217	0.003823679	yes	up	0.762	1.376	1.12	0.53	1.13	0.36	0.67	1.55	1.41	1.1	0.82	2
ENSMUSG00000042379 Esm1	endothelial cell-specific molecule 1 [Source:MGI Symbol;Acc:MGI:1918940]	0.382501752	-1.38646174	1.29E-10	1.43E-09	yes	down	18.296	5.91	21.12	17.25	19.18	22.23	11.7	3.99	7.56	3.56	8.46	5.98
ENSMUSG00000058290 Espl1	extra spindle pole bodies 1, separase [Source:MGI Symbol;Acc:MGI:2146156]	2.626604638	1.393199059	1.05E-14	1.76E-13	yes	up	2.132	3.53	2.15	0.85	3.14	1.06	3.46	3.42	5.32	2.07	2.75	4.09
ENSMUSG00000021055 Esr2	estrogen receptor 2 (beta) [Source:MGI Symbol;Acc:MGI:109392]	0.321492651	-1.637142337	1.79E-09	1.77E-08	yes	down	1.516	0.42	1.57	1.05	1.93	1.91	1.12	0.45	0.54	0.32	0.41	0.38
ENSMUSG00000026610 Esrrg	estrogen-related receptor gamma [Source:MGI Symbol;Acc:MGI:1347056]	0.477590055	-1.0661553	0.010560869	0.031452032	yes	down	0.782	0.318	0.7	0.86	0.46	0.85	1.04	0.28	0.32	0.68	0.08	0.23
ENSMUSG00000037681 Esyt3	extended synaptotagmin-like protein 3 [Source:MGI Symbol;Acc:MGI:1098699]	0.487231839	-1.037319684	2.19E-09	2.15E-08	yes	down	4.076	1.466	3.31	4.79	3.66	4.74	3.88	1.51	1.61	1.8	1.31	1.1
ENSMUSG00000070354 Evi2	ecotropic viral integration site 2 [Source:MGI Symbol;Acc:MGI:5439444]	3.603939328	1.849574724	1.46E-10	1.62E-09	yes	up	0.628	1.892	0.4	0.41	1.12	0.85	0.36	2.25	1.97	1.89	1.57	1.78
ENSMUSG00000078771 Evi2a	ecotropic viral integration site 2a [Source:MGI Symbol;Acc:MGI:95458]	3.143504971	1.652374045	2.39E-49	2.30E-47	yes	up	16.128	41.112	14.68	18.41	16.97	13.64	16.94	42.98	40.19	38.45	39.1	44.84
ENSMUSG00000093938 Evi2b	ecotropic viral integration site 2b [Source:MGI Symbol;Acc:MGI:1890682]	2.442872325	1.288578464	7.04E-25	2.42E-23	yes	up	14.772	30.424	10.87	17.61	15.97	16.1	13.31	34.74	29.69	30.04	28.11	29.54
ENSMUSG00000021262 Evl	Ena-vasodilator stimulated phosphoprotein [Source:MGI Symbol;Acc:MGI:1194884]	2.538356444	1.343894671	1.03E-45	8.89E-44	yes	up	54.6	109.56	59.04	52.47	61.33	46.28	53.88	88.86	121.57	114.72	105.45	117.2
ENSMUSG00000034282 Evpl	envoplakin [Source:MGI Symbol;Acc:MGI:107507]	0.458184145	-1.126000558	3.88E-10	4.12E-09	yes	down	3.108	1.202	2.87	2.73	3.59	3.19	3.16	0.85	0.85	1.49	1.41	1.41
ENSMUSG00000039748 Exo1	exonuclease 1 [Source:MGI Symbol;Acc:MGI:1349427]	2.425120922	1.278056685	0.000758794	0.003048271	yes	up	0.654	0.85	0.84	0.12	0.13	1.22	0.96	0.77	0.72	0.17	1.27	1.32
ENSMUSG00000109941 Exosc6	exosome component 6 [Source:MGI Symbol;Acc:MGI:1919794]	17.47084589	4.126877556	6.79E-05	0.000339905	yes	up	0.044	0.62	0	0	0	0	0.22	0.66	1.14	0.27	0.59	0.44
ENSMUSG00000010461 Eya4	EYA transcriptional coactivator and phosphatase 4 [Source:MGI Symbol;Acc:MGI:1337104]	2.568312192	1.360820581	0.011820969	0.034636457	yes	up	0.09	0.2	0.05	0.18	0.08	0.09	0.05	0.18	0.2	0.35	0.12	0.15
ENSMUSG00000031444 F10	coagulation factor X [Source:MGI Symbol;Acc:MGI:103107]	5.350661619	2.419717295	5.04E-56	5.93E-54	yes	up	5.322	23.526	5.96	6.57	6.23	3.7	4.15	18.89	27.11	25.11	22.69	23.83
ENSMUSG00000028128 F3	coagulation factor III [Source:MGI Symbol;Acc:MGI:88381]	0.340817399	-1.552929106	3.22E-22	9.33E-21	yes	down	61.882	17.636	58.25	89.91	49.89	50.6	60.76	16.09	15.9	21.33	18.39	16.47
ENSMUSG00000026579 F5	coagulation factor V [Source:MGI Symbol;Acc:MGI:88382]	0.38618689	-1.372628904	5.31E-05	0.000269684	yes	down	1.626	0.53	1.16	3.56	1.28	1.27	0.86	0.58	0.55	0.77	0.43	0.32
ENSMUSG00000031443 F7	coagulation factor VII [Source:MGI Symbol;Acc:MGI:109325]	4.260151631	2.090904781	1.71E-10	1.88E-09	yes	up	10.866	39.092	8.17	13.88	13.52	4.28	14.48	27.27	45.38	39.31	42.43	41.07

ENSMUSG00000090942	F830016B08Rik	RIKEN cDNA F830016B08 gene [Source:MGI Symbol;Acc:MGI:3588218]	2.606605829	1.382172435	2.39E-14	3.89E-13	yes	up	2.094	4.61	2.08	1.76	2.41	2.09	2.13	6.65	4.04	3.5	3.26	5.6
ENSMUSG00000031138	F9	coagulation factor IX [Source:MGI Symbol;Acc:MGI:88384]	4.50231659	2.170667507	7.58E-06	4.51E-05	yes	up	0.14	0.54	0.14	0.2	0.07	0.04	0.25	0.68	0.45	0.54	0.48	0.55
ENSMUSG00000054422	Fabp1	fatty acid binding protein 1, liver [Source:MGI Symbol;Acc:MGI:95479]	0.212770849	-2.232627592	1.06E-09	1.08E-08	yes	down	22.748	4.156	30.97	14.62	18.7	18.38	31.07	4.37	4.72	2.22	7.71	1.76
ENSMUSG00000027533	Fabp5	fatty acid binding protein 5, epidermal [Source:MGI Symbol;Acc:MGI:101790]	2.796824141	1.483789543	4.27E-24	1.40E-22	yes	up	170.192	404.328	155.75	248.63	136.82	159.85	149.91	444.82	368.87	328.36	413.38	466.21
ENSMUSG00000019874	Fabp7	fatty acid binding protein 7, brain [Source:MGI Symbol;Acc:MGI:101916]	39.14150633	5.290627373	3.52E-16	6.69E-15	yes	up	0.154	5.026	0	0.47	0	0.3	0	5.77	6.1	3.14	5.37	4.75
ENSMUSG00000023011	Faim2	Fas apoptotic inhibitory molecule 2 [Source:MGI Symbol;Acc:MGI:1919643]	0.273960982	-1.867957656	1.61E-35	9.52E-34	yes	down	9.564	2.158	12.46	8.33	8.71	11.45	6.87	2.04	2.02	2.23	2.26	2.24
ENSMUSG00000021750	Fam107a	family with sequence similarity 107, member A [Source:MGI Symbol;Acc:MGI:3041256]	0.249898045	-2.000588482	2.63E-14	4.25E-13	yes	down	36.326	7.504	45.63	27.18	33.15	22.02	53.65	9.63	9.3	6.62	7.46	4.51
ENSMUSG00000024691	Fam111a	family with sequence similarity 111, member A [Source:MGI Symbol;Acc:MGI:1915508]	2.35626463	1.236501576	3.23E-24	1.06E-22	yes	up	11.36	22.59	10.61	11.32	13.76	11.53	9.58	27.46	22.46	18.49	21.94	22.6
ENSMUSG00000034871	Fam151a	family with sequence simliarity 151, member A [Source:MGI Symbol;Acc:MGI:2657115]	0.039955854	-4.645449317	0.013739747	0.039361066	yes	down	0.156	0	0.3	0	0.4	0	0.08	0	0	0	0	0
ENSMUSG00000050493	Fam167b	family with sequence similarity 167, member B [Source:MGI Symbol;Acc:MGI:2668032]	5.468341387	2.451103313	3.94E-09	3.77E-08	yes	up	0.636	3.186	0.94	0.68	0.58	0.66	0.32	1.74	2.87	4.42	2.93	3.97
ENSMUSG00000046337	Fam178b	family with sequence similarity 178, member B [Source:MGI Symbol;Acc:MGI:3026913]	0.427472484	-1.226096535	3.15E-05	0.000167231	yes	down	5.266	11.034	3.34	6.71	6.85	5.07	4.36	1.53	47.9	1.91	1.68	2.15
ENSMUSG00000047420	Fam180a	family with sequence similarity 180, member A [Source:MGI Symbol;Acc:MGI:3039626]	2.041170389	1.029396618	0.000313773	0.001376717	yes	up	3.878	6.678	3.98	4.42	5.36	1.9	3.73	8.69	4.93	3.85	6.76	9.16
ENSMUSG00000096753	Fam181a	family with sequence similarity 181, member A [Source:MGI Symbol;Acc:MGI:3647570]	2.975740968	1.573248948	0.007239704	0.022740615	yes	up	0.472	1.062	0	0.4	1.01	0.33	0.62	1.19	0.8	1.14	1.65	0.53
ENSMUSG00000049154	Fam183b	family with sequence similarity 183, member B [Source:MGI Symbol;Acc:MGI:1922679]	0.429288867	-1.219979336	3.32E-08	2.82E-07	yes	down	63.404	23.138	63.91	64.99	46.8	63.3	78.02	21.63	21.47	36.7	14.43	21.46
ENSMUSG00000027955	Fam198b	family with sequence similarity 198, member B [Source:MGI Symbol;Acc:MGI:1915909]	0.497306084	-1.007794014	2.44E-27	9.68E-26	yes	down	20.794	9.046	20.03	22.7	19.96	23.52	17.76	6.69	9.94	8.37	8.84	11.39
ENSMUSG00000050141	Fam205c	family with sequence similarity 205, member C [Source:MGI Symbol;Acc:MGI:2679716]	0.04547205	-4.458876131	0.002223064	0.00798945	yes	down	0.404	0	0.35	0.69	0.34	0.37	0.27	0	0	0	0	0
ENSMUSG00000025854	Fam20c	family with sequence similarity 20, member C [Source:MGI	6.137517071	2.617655132	1.72E-114	8.77E-112	yes	up	10.55	34.074	11.93	12.01	9.52	9.72	9.57	31.99	34.01	34.55	30.35	39.47

		Symbol;Acc:MGI:2136853]																		
ENSMUSG00000079652	Fam71f2	family with sequence similarity 71, member F2 [Source:MGI Symbol;Acc:MGI:2141439]	0.343639501	-1.541032212	2.09E-11	2.54E-10	yes	down	2.61	0.864	3.98	1.72	1.62	2.17	3.56	1.38	0.78	0.33	0.74	1.09
ENSMUSG00000027654	Fam83d	family with sequence similarity 83, member D [Source:MGI Symbol;Acc:MGI:1919128]	2.412710168	1.270654619	0.000283664	0.001256454	yes	up	0.68	1.396	0.48	0.92	0.6	0.68	0.72	1.51	1.59	0.78	0.83	2.27
ENSMUSG00000022408	Fam83f	family with sequence similarity 83, member F [Source:MGI Symbol;Acc:MGI:2146227]	2.294498762	1.198179028	1.52E-05	8.54E-05	yes	up	0.6	1.166	0.37	0.5	0.66	0.69	0.78	1.41	1.2	1.27	0.9	1.05
ENSMUSG00000024939	Fam89b	family with sequence similarity 89, member B [Source:MGI Symbol;Acc:MGI:106595]	2.092832317	1.065456724	0.000442741	0.00187646	yes	up	5.264	9.294	2.2	4.83	4.19	6.77	8.33	10.3	11.02	9.22	10.81	5.12
ENSMUSG00000042269	Fam92b	family with sequence similarity 92, member B [Source:MGI Symbol;Acc:MGI:3588213]	0.38253696	-1.386328951	5.76E-07	4.09E-06	yes	down	9.028	2.902	8.39	9.66	4.24	10.08	12.77	2.31	2.9	4.29	2.14	2.87
ENSMUSG00000053111	Fank1	fibronectin type 3 and ankyrin repeat domains 1 [Source:MGI Symbol;Acc:MGI:1914180]	0.454766204	-1.136803051	0.000143362	0.000674331	yes	down	3.9	1.494	5.22	2.83	3.13	3.7	4.62	1.14	1.33	2.59	0.77	1.64
ENSMUSG00000074505	Fat3	FAT atypical cadherin 3 [Source:MGI Symbol;Acc:MGI:2444314]	0.487356151	-1.036951642	1.55E-09	1.54E-08	yes	down	3.106	1.402	4.05	1.84	3.16	3.6	2.88	1.49	2.01	0.99	1.56	0.96
ENSMUSG00000034532	Fbxo16	F-box protein 16 [Source:MGI Symbol;Acc:MGI:1354706]	0.498579289	-1.004105139	0.000475484	0.002001368	yes	down	2.378	1.01	1.51	3.18	2.55	2.5	2.15	1.14	0.21	1.04	1.72	0.94
ENSMUSG00000070336	Fbxo47	F-box protein 47 [Source:MGI Symbol;Acc:MGI:1920223]	0.31817795	-1.652094238	0.002437016	0.008682605	yes	down	0.886	0.182	0.91	0.98	0.89	0.94	0.71	0.28	0.24	0.12	0.17	0.1
ENSMUSG00000026415	Fcamr	Fc receptor, IgA, IgM, high affinity [Source:MGI Symbol;Acc:MGI:1927803]	2.507994083	1.326533944	0.013586481	0.038994409	yes	up	0.576	1.256	0.49	0.53	0.07	0.53	1.26	1.3	0.82	2.18	0.57	1.41
ENSMUSG00000058715	Fcer1g	Fc receptor, IgE, high affinity I, gamma polypeptide [Source:MGI Symbol;Acc:MGI:95496]	5.027612592	2.329873486	7.45E-125	4.99E-122	yes	up	106.566	454.64	97.93	115.46	127.49	92.82	99.13	415.66	467.92	423.49	457.37	508.76
ENSMUSG00000015947	Fcgr1	Fc receptor, IgG, high affinity I [Source:MGI Symbol;Acc:MGI:95498]	7.813280738	2.965928452	3.04E-82	6.99E-80	yes	up	3.54	19.57	4.3	2.47	2.52	2.93	5.48	18.73	16.21	17.06	21.2	24.65
ENSMUSG00000026656	Fcgr2b	Fc receptor, IgG, low affinity IIb [Source:MGI Symbol;Acc:MGI:95499]	6.103306841	2.609591123	8.15E-113	3.79E-110	yes	up	32.502	160.758	27.5	36.29	34.92	28.8	35	183.1	162.69	135.39	149.36	173.25
ENSMUSG00000059498	Fcgr3	Fc receptor, IgG, low affinity III [Source:MGI Symbol;Acc:MGI:95500]	8.767313584	3.13213485	1.62E-179	2.88E-176	yes	up	51.81	384.454	53.29	60.5	54.81	39.21	51.24	371.31	386.57	337.41	418.16	408.82
ENSMUSG00000059089	Fcgr4	Fc receptor, IgG, low affinity IV [Source:MGI Symbol;Acc:MGI:2179523]	6.050296508	2.597005847	4.66E-56	5.51E-54	yes	up	11.378	58.212	14.42	13.18	10.29	8.3	10.7	53.73	54.4	47.14	56.83	78.96
ENSMUSG00000070000	Fcho1	FCH domain only 1 [Source:MGI Symbol;Acc:MGI:1921265]	2.866604871	1.519343059	5.76E-13	8.15E-12	yes	up	4.278	10.66	3.27	4.16	5.45	4.93	3.58	11.4	8.79	9.69	9.32	14.1
ENSMUSG00000089665	Fcor	Foxo1 corepressor [Source:MGI Symbol;Acc:MGI:1915484]	2.675826113	1.419984366	0.001124003	0.004348202	yes	up	4.648	10.558	5.21	6.73	4.33	4.62	2.35	6.49	10.13	11	14.58	10.59
ENSMUSG00000070524	Fcrlb	Fc receptor-like B [Source:MGI Symbol;Acc:MGI:3576487]	9.818051215	3.295436692	1.70E-08	1.50E-07	yes	up	0.194	1.596	0.24	0	0.48	0.1	0.15	2.08	1.38	1.87	0.74	1.91
ENSMUSG00000015852	Fcrls	Fc receptor-like S, scavenger receptor [Source:MGI Symbol;Acc:MGI:1933397]	2.449730831	1.292623239	1.15E-12	1.58E-11	yes	up	6.726	13.178	8.27	6.73	6.44	4.51	7.68	9.8	13.52	14.03	16.69	11.85
ENSMUSG00000097336	Fendrr	Foxf1 adjacent non-coding developmental regulatory RNA [Source:MGI	0.429938328	-1.217798366	0.000172464	0.000797041	yes	down	52.36	18.796	55.04	35.8	47.37	79.39	44.2	18.4	26.29	16	13.85	19.44

	Symbol;Acc:MGI:1916040]																		
ENSMUSG00000037712 Fermt2	fermitin family member 2 [Source:MGI Symbol;Acc:MGI:2385001]	0.481381073	-1.054748677	1.19E-41	8.76E-40	yes	down	193.306	75.984	216.3	183.04	184.56	197.75	184.88	70.75	83.65	70.36	84.21	70.95
ENSMUSG00000024965 Fermt3	fermitin family member 3 [Source:MGI Symbol;Acc:MGI:2147790]	2.311856621	1.209051926	4.03E-33	2.19E-31	yes	up	16.374	32.566	14.45	18.49	15.45	17.57	15.91	29.07	37.61	30.85	27.86	37.44
ENSMUSG00000053158 Fes	feline sarcoma oncogene [Source:MGI Symbol;Acc:MGI:95514]	2.003043107	1.002193469	1.07E-28	4.68E-27	yes	up	19.146	31.594	16.45	21.54	25.15	15.47	17.12	34.81	35.89	28.37	25.99	32.91
ENSMUSG00000032118 Fez1	fasciculation and elongation protein zeta 1 (zygin I) [Source:MGI Symbol;Acc:MGI:2670976]	0.232014745	-2.107711599	2.19E-05	0.000120407	yes	down	1.604	0.358	1.03	2.61	1.16	1.99	1.23	0.15	0.3	0.51	0.74	0.09
ENSMUSG00000024013 Fgd2	FYVE, RhoGEF and PH domain containing 2 [Source:MGI Symbol;Acc:MGI:1347084]	2.016971446	1.01219066	1.19E-16	2.38E-15	yes	up	11.948	18.634	8.12	13.7	12.68	13.16	12.08	19.19	18.19	21.14	18.58	16.07
ENSMUSG00000037946 Fgd3	FYVE, RhoGEF and PH domain containing 3 [Source:MGI Symbol;Acc:MGI:1353657]	2.164996112	1.114364434	7.77E-18	1.71E-16	yes	up	3.842	7.132	3.71	4.44	3.46	3.97	3.63	8.65	7.34	7.14	5.53	7
ENSMUSG00000048373 Fgfbp1	fibroblast growth factor binding protein 1 [Source:MGI Symbol;Acc:MGI:1096350]	0.376150972	-1.410616276	1.06E-15	1.93E-14	yes	down	20.65	6.588	19.5	22.49	19.54	16.74	24.98	7.72	7.42	6.79	6.55	4.46
ENSMUSG00000054252 Fgfr3	fibroblast growth factor receptor 3 [Source:MGI Symbol;Acc:MGI:95524]	0.467962316	-1.095535737	2.30E-32	1.21E-30	yes	down	83.538	34.812	85.28	68.98	79.43	102.87	81.13	39.46	40.99	32.1	35.11	26.4
ENSMUSG00000031594 Fgl1	fibrinogen-like protein 1 [Source:MGI Symbol;Acc:MGI:102795]	3.590442068	1.844161485	0.015403157	0.043478847	yes	up	0.35	1.056	0.14	0.51	0.8	0.12	0.18	0.12	1.32	2.51	0.83	0.5
ENSMUSG00000039899 Fgl2	fibrinogen-like protein 2 [Source:MGI Symbol;Acc:MGI:103266]	2.288940983	1.194680266	9.67E-15	1.63E-13	yes	up	26.938	52.016	21.45	33.95	28.74	23.14	27.41	56.05	40.52	52.79	42.43	68.29
ENSMUSG00000028874 Fgr	FGR proto-oncogene, Src family tyrosine kinase [Source:MGI Symbol;Acc:MGI:95527]	2.769486574	1.469618545	5.69E-33	3.06E-31	yes	up	7.554	17.808	6.8	10	7.24	7.85	5.88	15.05	18.74	18.35	17.18	19.72
ENSMUSG00000028259 Fhl5	four and a half LIM domains 5 [Source:MGI Symbol;Acc:MGI:1913192]	3.78238734	1.919297112	0.007521478	0.023511891	yes	up	0.212	0.664	0	0.45	0.3	0.19	0.12	0.42	0.56	0.78	0.48	1.08
ENSMUSG00000074971 Fibin	fin bud initiation factor homolog (zebrafish) [Source:MGI Symbol;Acc:MGI:1914856]	0.278469122	-1.844410732	8.23E-76	1.73E-73	yes	down	61.858	14.562	69.32	56.72	59.57	62.47	61.21	14.55	16.98	13.99	15.78	11.51
ENSMUSG00000075324 Fign	fidgetin [Source:MGI Symbol;Acc:MGI:1890647]	0.429629003	-1.218836707	3.99E-11	4.68E-10	yes	down	7.658	0.94	3.15	1.04	2.91	29.84	1.35	0.5	0.54	2.34	0.55	0.77
ENSMUSG00000035455 Fignl1	fidgetin-like 1 [Source:MGI Symbol;Acc:MGI:1890648]	2.027469236	1.019680024	0.000303976	0.001337295	yes	up	1.046	1.782	0.82	1.33	0.89	1.09	1.1	2.14	1.16	1.57	1.26	2.78
ENSMUSG00000003355 Fkbp11	FK506 binding protein 11 [Source:MGI Symbol;Acc:MGI:1913370]	2.203492358	1.139791893	6.01E-06	3.64E-05	yes	up	6.444	12.49	7.11	8.22	7.65	4.69	4.55	14.98	10.04	9.29	12.4	15.74
ENSMUSG00000038074 Fkbp14	FK506 binding protein 14 [Source:MGI Symbol;Acc:MGI:2387639]	0.389546927	-1.36013096	4.07E-20	1.05E-18	yes	down	27.396	9.066	34.18	25.36	28.49	18.92	30.03	10.2	9.46	8.4	8.24	9.03
ENSMUSG00000020635 Fkbp1b	FK506 binding protein 1b [Source:MGI Symbol;Acc:MGI:1336205]	2.160181685	1.111152658	1.55E-06	1.03E-05	yes	up	8.442	8.95	4.02	19.08	2.9	10.71	5.5	8.33	11.62	8.3	6.39	10.11
ENSMUSG00000042817 Flt3	FMS-like tyrosine kinase 3 [Source:MGI Symbol;Acc:MGI:95559]	2.486936785	1.314369836	5.48E-18	1.22E-16	yes	up	3.138	6.56	2.66	3.68	3.99	2.67	2.69	6.77	6.31	7.83	5.76	6.13
ENSMUSG00000044042 Fmn1	formin 1 [Source:MGI Symbol;Acc:MGI:101815]	2.488541654	1.315300535	3.22E-33	1.77E-31	yes	up	2.148	3.578	1.36	2.66	2.66	1.67	2.39	3.13	3.68	3.21	4.61	3.26
ENSMUSG00000028354 Fmn2	formin 2 [Source:MGI Symbol;Acc:MGI:1859252]	0.350750105	-1.51148456	4.73E-11	5.51E-10	yes	down	6.364	1.532	4.97	4.72	6.17	13.02	2.94	1.83	1.73	1.37	1.56	1.17
ENSMUSG00000055805 Fmnl1	formin-like 1 [Source:MGI Symbol;Acc:MGI:1888994]	2.033458853	1.023935798	1.49E-26	5.68E-25	yes	up	23.736	36.736	30.7	21.75	24.28	22.54	19.41	43.16	33.43	38.6	28.93	39.56
ENSMUSG00000040170 Fmo2	flavin containing monooxygenase 2 [Source:MGI	0.468021209	-1.095354185	1.54E-19	3.79E-18	yes	down	481.812	201.248	554.53	390.54	503.56	525.53	434.9	214.55	199.76	182.22	257.46	152.25

		Symbol;Acc:MGI:1916776]																		
ENSMUSG00000026691	Fmo3	flavin containing monooxygenase 3 [Source:MGI Symbol;Acc:MGI:1100496]	0.330923144	-1.5954319	2.07E-08	1.81E-07	yes	down	45.644	12.718	49.16	50.84	36.35	41.94	49.93	18.54	9.92	17.48	7.97	9.68
ENSMUSG00000026692	Fmo4	flavin containing monooxygenase 4 [Source:MGI Symbol;Acc:MGI:2429497]	0.48573767	-1.041750721	0.00058657	0.002417159	yes	down	2.602	1.208	1.55	3.79	2.49	2.88	2.3	1.07	2.21	0.57	0.65	1.54
ENSMUSG00000026193	Fn1	fibronectin 1 [Source:MGI Symbol;Acc:MGI:95566]	4.375301059	2.12938229	5.02E-86	1.28E-83	yes	up	99.726	365.81	100.1	98.75	87.91	109.03	102.84	262.42	426.71	350.17	395.77	393.98
ENSMUSG00000048721	Fndc9	fibronectin type III domain containing 9 [Source:MGI Symbol;Acc:MGI:2443410]	4.460940668	2.15734796	0.000485045	0.002038943	yes	up	0.132	0.49	0.12	0.27	0.09	0.03	0.15	0.91	0.43	0.34	0.22	0.55
ENSMUSG00000001773	Folh1	folate hydrolase 1 [Source:MGI Symbol;Acc:MGI:1858193]	0.129509655	-2.948868437	6.74E-05	0.000337685	yes	down	0.536	0.038	0.19	1.61	0.4	0.18	0.3	0.03	0.08	0.04	0.04	0
ENSMUSG00000021250	Fos	FBJ osteosarcoma oncogene [Source:MGI Symbol;Acc:MGI:95574]	0.318727713	-1.649603628	0.000836279	0.003333097	yes	down	124.348	35.036	61.3	292.52	67.25	140.74	59.93	35.64	31.47	50.61	25.45	32.01
ENSMUSG00000003545	Fosb	FBJ osteosarcoma oncogene B [Source:MGI Symbol;Acc:MGI:95575]	0.092106497	-3.440553257	8.53E-11	9.68E-10	yes	down	12.508	0.856	13.7	24.92	5.75	7.79	10.38	0.66	1.44	1.35	0.51	0.32
ENSMUSG00000042812	Foxf1	forkhead box F1 [Source:MGI Symbol;Acc:MGI:1347470]	0.402376135	-1.313383355	5.44E-38	3.53E-36	yes	down	161.014	54.728	176.11	171.83	133.02	189.61	134.5	56.94	59.9	46.96	56.45	53.39
ENSMUSG00000001517	Foxm1	forkhead box M1 [Source:MGI Symbol;Acc:MGI:1347487]	2.381437259	1.25183254	5.61E-10	5.86E-09	yes	up	1.562	3.346	1.12	1.97	2.26	1.18	1.28	2.88	3.91	3.09	2.33	4.52
ENSMUSG00000039521	Foxp3	forkhead box P3 [Source:MGI Symbol;Acc:MGI:1891436]	10.27498253	3.361064035	3.16E-38	2.07E-36	yes	up	0.376	2.664	0.4	0.22	0.67	0.18	0.41	2.43	2.12	3.82	2.27	2.68
ENSMUSG00000045551	Fpr1	formyl peptide receptor 1 [Source:MGI Symbol;Acc:MGI:107443]	2.699462571	1.432672214	6.64E-09	6.18E-08	yes	up	11.142	25.634	8.08	10.26	15.03	6.47	15.87	20.68	31.14	26.24	34.29	15.82
ENSMUSG00000052270	Fpr2	formyl peptide receptor 2 [Source:MGI Symbol;Acc:MGI:1278319]	6.446637844	2.688546939	4.47E-72	8.86E-70	yes	up	11.628	62.092	10.85	11.36	12.91	9.06	13.96	50.62	69.97	53.66	63.65	72.56
ENSMUSG00000079700	Fpr3	formyl peptide receptor 3 [Source:MGI Symbol;Acc:MGI:1194495]	14.51012857	3.858988397	2.76E-11	3.31E-10	yes	up	0.258	3.15	0.12	0.81	0	0	0.36	3.32	3.41	1.85	3.77	3.4
ENSMUSG00000034687	Fras1	Fraser extracellular matrix complex subunit 1 [Source:MGI Symbol;Acc:MGI:2385368]	0.442769	-1.175373878	7.85E-10	8.06E-09	yes	down	0.998	0.292	0.69	1.95	0.65	1.04	0.66	0.25	0.27	0.37	0.34	0.23
ENSMUSG00000027004	Frzb	frizzled-related protein [Source:MGI Symbol;Acc:MGI:892032]	3.438449112	1.781757994	0.003947426	0.013292151	yes	up	0.16	0.414	0.1	0.21	0.33	0.04	0.12	0.32	0.42	0.69	0.1	0.54
ENSMUSG00000029581	Fscn1	fascin actin-bundling protein 1 [Source:MGI Symbol;Acc:MGI:1352745]	2.66658909	1.414995529	6.99E-13	9.81E-12	yes	up	10.95	23.162	9.59	8.6	12.05	11.62	12.89	22.77	15.09	28.08	15.46	34.41
ENSMUSG00000027344	Fsip1	fibrous sheath-interacting protein 1 [Source:MGI Symbol;Acc:MGI:1918563]	0.471804715	-1.083738259	5.94E-05	0.000299794	yes	down	2.772	1.106	2.88	3.2	1.9	2.79	3.09	1.01	1.49	1.33	1	0.7
ENSMUSG00000024661	Fth1	ferritin heavy polypeptide 1 [Source:MGI Symbol;Acc:MGI:95588]	3.205122929	1.680379691	1.27E-39	8.69E-38	yes	up	3562.502	10120.628	2761.99	2839.79	3369.08	4221.1	4620.55	11611.98	11059.7	9763.91	6866.26	11301.29
ENSMUSG00000050708	Ftl1	ferritin light polypeptide 1 [Source:MGI Symbol;Acc:MGI:95589]	2.954446706	1.562887975	9.61E-163	1.29E-159	yes	up	2358.438	5893.114	2318.28	2467.55	2397.41	2313.68	2295.27	5816.51	5998.26	5329.54	6216.44	6104.82
ENSMUSG00000062382	Ftl1-ps1	ferritin light polypeptide 1, pseudogene 1 [Source:MGI Symbol;Acc:MGI:3779109]	2.898612453	1.535362457	4.73E-09	4.47E-08	yes	up	2.558	6.042	2.21	2.01	3.5	3.09	1.98	6.34	7.89	5.84	5.79	4.35
ENSMUSG00000008461	Fut1	fucosyltransferase 1 [Source:MGI Symbol;Acc:MGI:109375]	8.693394668	3.119919642	0.000941599	0.00371505	yes	up	0.176	1.25	0	0.13	0.53	0	0.22	1.71	1	1.11	1.12	1.31
ENSMUSG00000049307	Fut4	fucosyltransferase 4 [Source:MGI Symbol;Acc:MGI:95594]	2.212174872	1.145465435	7.87E-11	8.96E-10	yes	up	2.826	5.276	1.87	3.87	3	2.34	3.05	5.65	4.54	5.44	5.31	5.44
ENSMUSG00000036587	Fut7	fucosyltransferase 7 [Source:MGI Symbol;Acc:MGI:107692]	4.181498605	2.064020082	6.18E-11	7.11E-10	yes	up	0.76	3.292	0.75	0.6	1.01	0.33	1.11	4.88	3.2	2.62	2.34	3.42
ENSMUSG00000036570	Fxyd1	FXYD domain-containing ion transport regulator 1 [Source:MGI	0.379043755	-1.399563701	4.71E-22	1.35E-20	yes	down	135.158	39.69	121.73	188.38	113.21	149.73	102.74	33.29	42.24	44.87	48.13	29.92

	Symbol;Acc:MGI:1889273]																		
ENSMUSG00000004988 Fxyd4	FXYD domain-containing ion transport regulator 4 [Source:MGI Symbol;Acc:MGI:1889005]	8.304144127	3.053831483	3.64E-07	2.66E-06	yes	up	0.282	2.57	0.34	0.12	0.46	0.39	0.1	1.56	3.71	3.17	1.5	2.91
ENSMUSG00000009687 Fxyd5	FXYD domain-containing ion transport regulator 5 [Source:MGI Symbol;Acc:MGI:1201785]	2.568659196	1.36101549	7.30E-51	7.23E-49	yes	up	215.226	480.288	209.3	246.47	214.23	200.82	205.31	402.81	491.13	459.29	485.52	562.69
ENSMUSG00000022148 Fyb	FYN binding protein [Source:MGI Symbol;Acc:MGI:1346327]	3.184931288	1.671262248	5.72E-24	1.86E-22	yes	up	17.234	36.108	15.21	21.85	18.06	17.08	13.97	40.95	36.83	28.39	29.88	44.49
ENSMUSG00000049791 Fzd4	frizzled class receptor 4 [Source:MGI Symbol;Acc:MGI:108520]	0.488168424	-1.034549114	1.23E-40	8.62E-39	yes	down	55.198	22.732	62.29	52.17	53.54	53.82	54.17	23.41	24.78	22.33	22.99	20.15
ENSMUSG00000009633 G0s2	G0/G1 switch gene 2 [Source:MGI Symbol;Acc:MGI:1316737]	0.39492686	-1.340342603	4.39E-19	1.05E-17	yes	down	102.344	34.008	90.47	112.6	96.06	140.05	72.54	36.9	30.99	35.06	38.76	28.33
ENSMUSG00000097694 G730013B05Rik	RIKEN cDNA G730013B05 gene [Source:MGI Symbol;Acc:MGI:3588276]	2.498253168	1.320919684	0.013820887	0.03955474	yes	up	0.432	0.978	0.78	0.24	0.25	0.29	0.6	1.95	1.09	0.62	0.72	0.51
ENSMUSG00000115009 G930009F23Rik	RIKEN cDNA G930009F23 gene [Source:MGI Symbol;Acc:MGI:3642701]	0.454238754	-1.138477297	0.000786132	0.003147242	yes	down	2.916	0.846	2.54	2.04	4.54	3.6	1.86	0.82	0.65	0.71	1	1.05
ENSMUSG00000031343 Gabra3	gamma-aminobutyric acid (GABA) A receptor, subunit alpha 3 [Source:MGI Symbol;Acc:MGI:95615]	0.471272038	-1.08536801	4.91E-08	4.06E-07	yes	down	6.52	2.554	6.05	6.61	6.81	6.39	6.74	1.92	2.33	3.87	2.63	2.02
ENSMUSG00000031340 Gabre	gamma-aminobutyric acid (GABA) A receptor, subunit epsilon [Source:MGI Symbol;Acc:MGI:1330235]	0.26423226	-1.920121481	0.00013595	0.000642717	yes	down	6.978	2.804	6.49	7.12	10.09	7.64	3.55	5.76	5.56	2.58	0.07	0.05
ENSMUSG00000020159 Gabrp	gamma-aminobutyric acid (GABA) A receptor, pi [Source:MGI Symbol;Acc:MGI:2387597]	0.454846663	-1.136547826	3.62E-12	4.77E-11	yes	down	36.852	14.12	37.6	38.23	29.57	40.39	38.47	12.51	13.91	21.8	10.77	11.61
ENSMUSG00000031344 Gabrq	gamma-aminobutyric acid (GABA) A receptor, subunit theta [Source:MGI Symbol;Acc:MGI:1888498]	0.453108217	-1.142072442	0.012969056	0.037512871	yes	down	0.434	0.172	0.44	0.56	0.39	0.27	0.51	0.05	0.25	0.23	0.24	0.09
ENSMUSG00000024907 Gal	galanin [Source:MGI Symbol;Acc:MGI:95637]	0.083059663	-3.589708164	6.21E-09	5.80E-08	yes	down	3.526	0.244	3.83	3.78	3.14	2.25	4.63	0.2	0.46	0	0	0.56
ENSMUSG00000015027 Galns	galactosamine (N-acetyl)-6-sulfate sulfatase [Source:MGI Symbol;Acc:MGI:1355303]	2.973778621	1.572297252	3.68E-39	2.47E-37	yes	up	6.47	16.628	5.23	7.54	7	6.18	6.4	14.91	17.23	13.66	19.36	17.98
ENSMUSG00000060988 Galnt13	polypeptide N-acetylgalactosaminyltransferase 13 [Source:MGI Symbol;Acc:MGI:2139447]	0.392303856	-1.34995658	1.81E-06	1.19E-05	yes	down	0.864	0.19	0.94	0.58	0.66	1.43	0.71	0.13	0.16	0.11	0.23	0.32
ENSMUSG00000021903 Galnt15	polypeptide N-acetylgalactosaminyltransferase 15 [Source:MGI Symbol;Acc:MGI:1926004]	0.306793214	-1.704661523	2.17E-29	9.71E-28	yes	down	16.576	4.336	14.18	15.99	17.81	13.29	21.61	3.58	5.16	5.16	4.11	3.67
ENSMUSG00000026828 Galnt5	polypeptide N-acetylgalactosaminyltransferase 5 [Source:MGI Symbol;Acc:MGI:2179403]	0.380316219	-1.39472863	7.55E-05	0.000374445	yes	down	0.986	0.32	0.78	1.29	0.83	1.18	0.85	0.28	0.17	0.61	0.19	0.35
ENSMUSG00000037280 Galnt6	polypeptide N-acetylgalactosaminyltransferase 6 [Source:MGI Symbol;Acc:MGI:1891640]	2.790884728	1.480722538	3.17E-33	1.74E-31	yes	up	5.062	12.022	5.1	5.56	5.36	3.62	5.67	11.25	12.87	11.56	11.93	12.5

ENSMUSG00000031608 Galnt7	polypeptide N-acetylgalactosaminyltransferase 7 [Source:MGI Symbol;Acc:MGI:1349449]	2.942913676	1.557245225	2.10E-57	2.60E-55	yes	up	5.54	13.702	5.17	5.93	6.2	5.16	5.24	13.67	14.25	15.01	12.47	13.11
ENSMUSG00000033316 Galnt9	polypeptide N-acetylgalactosaminyltransferase 9 [Source:MGI Symbol;Acc:MGI:2677965]	2.182382383	1.125903904	0.012580965	0.036538371	yes	up	0.508	1.028	0.56	0.57	0.48	0.69	0.24	1.8	1.25	0.63	0.87	0.59
ENSMUSG00000114755 Galr3	galanin receptor 3 [Source:MGI Symbol;Acc:MGI:1329003]	28.87097281	4.851547815	0.001590979	0.005932859	yes	up	0	0.26	0	0	0	0	0	0.36	0.42	0.31	0	0.21
ENSMUSG00000074802 Gas2l3	growth arrest-specific 2 like 3 [Source:MGI Symbol;Acc:MGI:1918780]	5.302476915	2.406666435	2.48E-37	1.56E-35	yes	up	1.358	2.482	0.57	0.58	4.77	0.41	0.46	2.97	2.42	2.09	2.11	2.82
ENSMUSG00000033066 Gas7	growth arrest specific 7 [Source:MGI Symbol;Acc:MGI:1202388]	2.21057259	1.14442011	7.37E-18	1.62E-16	yes	up	8.146	15.486	8.52	9.21	8.43	6.37	8.2	12.53	18.44	16.17	12.07	18.22
ENSMUSG00000015053 Gata2	GATA binding protein 2 [Source:MGI Symbol;Acc:MGI:95662]	0.452102433	-1.145278413	3.03E-27	1.19E-25	yes	down	82.95	34.722	77.88	63.11	72.78	92.83	108.15	43.22	42.86	32.08	24.3	31.15
ENSMUSG00000021944 Gata4	GATA binding protein 4 [Source:MGI Symbol;Acc:MGI:95664]	0.39841699	-1.327648923	0.005265816	0.017184711	yes	down	1.356	0.382	0.91	1.72	1.37	1.95	0.83	0.28	0.46	0.58	0.48	0.11
ENSMUSG00000027199 Gatm	glycine amidinotransferase (L-arginine:glycine amidinotransferase) [Source:MGI Symbol;Acc:MGI:1914342]	3.186925019	1.672165076	3.75E-21	1.02E-19	yes	up	3.668	9.498	3.12	4.41	4.45	3.71	2.65	8.59	8.65	8.58	9.08	12.59
ENSMUSG00000026829 Gbgt1	globoside alpha-1,3-N-acetylgalactosaminyltransferase 1 [Source:MGI Symbol;Acc:MGI:2449143]	3.377886879	1.756121015	6.71E-44	5.36E-42	yes	up	3.11	9.35	2.55	4.57	3.28	2.54	2.61	6.06	10.47	9.55	12.88	7.79
ENSMUSG00000105096 Gbp10	guanylate-binding protein 10 [Source:MGI Symbol;Acc:MGI:4359647]	2.895152975	1.53363958	6.26E-06	3.78E-05	yes	up	1.87	3.914	1.98	0.88	2.93	1.17	2.39	4.66	4.23	1.74	3.99	4.95
ENSMUSG00000028270 Gbp2	guanylate binding protein 2 [Source:MGI Symbol;Acc:MGI:102772]	2.073804186	1.052279678	6.10E-06	3.69E-05	yes	up	54.956	95.936	59.71	45.18	59.56	56.87	53.46	105.86	68.28	68.17	90.57	146.8
ENSMUSG00000040264 Gbp2b	guanylate binding protein 2b [Source:MGI Symbol;Acc:MGI:95666]	3.576605746	1.838591097	5.49E-05	0.000278286	yes	up	0.246	0.744	0.09	0.38	0.26	0.22	0.28	1.06	0.79	0.39	0.39	1.09
ENSMUSG00000105504 Gbp5	guanylate binding protein 5 [Source:MGI Symbol;Acc:MGI:2429943]	2.6657436	1.414538024	4.90E-07	3.52E-06	yes	up	8.928	20.032	8.79	5.59	9.7	9.83	10.73	24.07	12.66	17.08	15.73	30.62
ENSMUSG00000034438 Gbp8	guanylate-binding protein 8 [Source:MGI Symbol;Acc:MGI:1923324]	2.633217364	1.396826617	9.15E-14	1.41E-12	yes	up	5.986	13.274	6.73	4.54	6.08	6.02	6.56	17.26	9.17	11.86	11.06	17.02
ENSMUSG00000116378 Gcat	glycine C-acetyltransferase (2-amino-3-ketobutyrate-coenzyme A ligase) [Source:MGI Symbol;Acc:MGI:1349389]	0.272005396	-1.878292822	0.000751096	0.003023586	yes	down	2.62	0.602	3.31	1.51	2.69	2.8	2.79	0	1.13	0.15	0.84	0.89
ENSMUSG00000091387 Gcnt4	glucosaminyl (N-acetyl) transferase 4, core 2 (beta-1,6-N-acetylglucosaminyltransferase) [Source:MGI Symbol;Acc:MGI:2684919]	3.395923485	1.763803953	0.000760058	0.003052202	yes	up	0.084	0.242	0.09	0.14	0.04	0.06	0.09	0.28	0.12	0.29	0.14	0.38
ENSMUSG00000022659 Gcsam	germinal center associated, signaling and motility [Source:MGI Symbol;Acc:MGI:102969]	2.844226625	1.508036422	9.95E-10	1.01E-08	yes	up	1.284	3.118	1.52	1.56	1.75	0.83	0.76	4	2.65	2.51	2.89	3.54
ENSMUSG00000058624 Gda	guanine deaminase [Source:MGI Symbol;Acc:MGI:95678]	2.142930628	1.099585147	3.83E-17	8.00E-16	yes	up	12.444	22.714	12.89	14.18	14.03	8.99	12.13	25.11	24.46	18.48	23.83	21.69
ENSMUSG00000072625 Gdf2	growth differentiation factor 2 [Source:MGI Symbol;Acc:MGI:1321394]	0.292277026	-1.774591663	0.000297009	0.001309065	yes	down	0.688	0.17	0.63	0.74	0.74	0.63	0.7	0.04	0.1	0.37	0.25	0.09
ENSMUSG00000030117 Gdf3	growth differentiation factor 3 [Source:MGI Symbol;Acc:MGI:95686]	12.16318641	3.604449318	1.10E-10	1.23E-09	yes	up	0.092	0.928	0.03	0.1	0.13	0.17	0.03	1.26	0.55	1.11	0.64	1.08
ENSMUSG00000051279 Gdf6	growth differentiation factor 6 [Source:MGI Symbol;Acc:MGI:95689]	4.087536663	2.031231671	1.31E-09	1.32E-08	yes	up	0.288	0.986	0.31	0.35	0.48	0.11	0.19	0.88	1.07	1.01	1.04	0.93
ENSMUSG00000019359 Gdpd2	glycerophosphodiester phosphodiesterase domain containing 2 [Source:MGI Symbol;Acc:MGI:1918834]	0.252515483	-1.985556243	4.06E-19	9.75E-18	yes	down	10.692	2.386	12.14	7.91	7.46	11.87	14.08	1.95	2.11	2.81	2.34	2.72

ENSMUSG00000029275 Gfi1	growth factor independent 1 transcription repressor [Source:MGI Symbol;Acc:MGI:103170]	3.27583657	1.711863383	0.000139276	0.0006567	yes	up	0.45	1.258	0.27	0.73	0.74	0.26	0.25	1.18	1.98	0.73	0.69	1.71
ENSMUSG00000020363 Gfpt2	glutamine fructose-6-phosphate transaminase 2 [Source:MGI Symbol;Acc:MGI:1338883]	0.485311511	-1.043017016	4.45E-08	3.71E-07	yes	down	9.94	4.074	8.95	13.81	9.26	7.06	10.62	3.39	3.66	5.63	3.92	3.77
ENSMUSG00000025089 Gfra1	glial cell line derived neurotrophic factor family receptor alpha 1 [Source:MGI Symbol;Acc:MGI:1100842]	0.490760301	-1.026909544	5.14E-15	8.84E-14	yes	down	8.196	4.612	7.75	7.32	7.79	9.14	8.98	3.3	4.9	5.09	2.34	7.43
ENSMUSG00000024366 Gfra3	glial cell line derived neurotrophic factor family receptor alpha 3 [Source:MGI Symbol;Acc:MGI:1201403]	0.130236449	-2.940794824	0.002167182	0.007805646	yes	down	0.35	0.036	0.52	0.85	0.11	0.18	0.09	0.03	0.05	0.05	0	0.05
ENSMUSG00000006345 Ggt1	gamma-glutamyltransferase 1 [Source:MGI Symbol;Acc:MGI:95706]	0.339916873	-1.556746117	0.000408333	0.001743741	yes	down	2.248	0.606	1.6	1.15	1.79	2.02	4.68	0.65	0.37	1.04	0.36	0.61
ENSMUSG00000043931 Gimap7	GTPase, IMAP family member 7 [Source:MGI Symbol;Acc:MGI:1349657]	2.463221909	1.300546604	2.89E-08	2.48E-07	yes	up	5.672	11.788	5.83	4.46	7.45	6.57	4.05	18.74	8.72	10.35	9.93	11.2
ENSMUSG00000046623 Gjb4	gap junction protein, beta 4 [Source:MGI Symbol;Acc:MGI:95722]	2.756255286	1.462709518	0.011627352	0.03417669	yes	up	0.234	0.542	0.31	0.16	0.31	0.13	0.26	0.34	0.59	0.74	0.72	0.32
ENSMUSG00000040055 Gjb6	gap junction protein, beta 6 [Source:MGI Symbol;Acc:MGI:107588]	0.460069897	-1.120075033	9.77E-11	1.10E-09	yes	down	7.592	2.984	8.5	6.48	7.48	6.91	8.59	2.97	4.09	3.08	2.49	2.29
ENSMUSG00000031266 Gla	galactosidase, alpha [Source:MGI Symbol;Acc:MGI:1347344]	4.237674129	2.083272651	5.79E-64	8.61E-62	yes	up	6.568	25.128	7.28	6.71	7.81	5.18	5.86	24.81	29.24	20.5	27.74	23.35
ENSMUSG00000045594 Glb1	galactosidase, beta 1 [Source:MGI Symbol;Acc:MGI:88151]	2.255022997	1.173142147	2.24E-47	2.01E-45	yes	up	14.528	27.664	14.64	15.19	15.51	13.68	13.62	25.11	30.27	29.2	26.01	27.73
ENSMUSG00000036395 Glb1l2	galactosidase, beta 1-like 2 [Source:MGI Symbol;Acc:MGI:2388283]	0.318277627	-1.651642347	9.01E-37	5.54E-35	yes	down	18.328	4.774	17.07	17.21	20.84	19.72	16.8	4.21	6.21	5.68	4.15	3.62
ENSMUSG00000056888 Glipr1	GLI pathogenesis-related 1 (glioma) [Source:MGI Symbol;Acc:MGI:1920940]	6.668482615	2.73735852	2.78E-116	1.45E-113	yes	up	28.384	160.37	21.14	35.94	29.64	25.56	29.64	189.05	152.01	149.62	159.48	151.69
ENSMUSG00000057802 Gm10030	predicted gene 10030 [Source:MGI Symbol;Acc:MGI:3641798]	0.037749341	-4.727404741	0.000619593	0.002532775	yes	down	0.53	0.002	0.02	2.43	0.07	0.07	0.06	0	0	0	0	0.01
ENSMUSG00000100954 Gm10138	predicted gene 10138 [Source:MGI Symbol;Acc:MGI:3708522]	5.222516571	2.384745165	0.000840259	0.003346037	yes	up	0.066	0.284	0.11	0.06	0	0.05	0.11	0.33	0.3	0.41	0.11	0.27
ENSMUSG00000064032 Gm10143	predicted gene 10143 [Source:MGI Symbol;Acc:MGI:3704492]	10.42594659	3.382106469	0.012744634	0.036968589	yes	up	0.024	0.202	0	0	0.07	0.05	0	0.22	0.5	0	0	0.29
ENSMUSG00000071036 Gm10309	predicted gene 10309 [Source:MGI Symbol;Acc:MGI:3641941]	0.228394656	-2.130399199	3.13E-06	2.00E-05	yes	down	1.172	0.23	1.37	0.67	1.09	0.89	1.84	0.2	0.35	0.32	0.09	0.19
ENSMUSG00000105044 Gm10416	predicted pseudogene 10416 [Source:MGI Symbol;Acc:MGI:3645037]	0.195153757	-2.357316861	0.005927605	0.019078087	yes	down	1.266	0.21	0.82	1.03	1.19	1.44	1.85	0.14	0.52	0.25	0.14	0
ENSMUSG00000073486 Gm10518	predicted gene 10518 [Source:MGI Symbol;Acc:MGI:3704205]	5.433396142	2.441854236	0.017659759	0.048863231	yes	up	0.078	0.358	0	0.12	0.12	0	0.15	0.72	0.09	0.51	0.1	0.37
ENSMUSG00000073492 Gm10521	predicted gene 10521 [Source:MGI Symbol;Acc:MGI:3642358]	4.398237131	2.136925389	4.03E-13	5.81E-12	yes	up	0.682	2.528	0.95	0.53	0.82	0.63	0.48	2.62	4.19	1.7	1.89	2.24
ENSMUSG00000083929 Gm10600	predicted gene 10600 [Source:MGI Symbol;Acc:MGI:3710628]	0.482905096	-1.050188408	0.001054105	0.004107482	yes	down	1.918	0.786	2.43	1.64	1.77	2.37	1.38	0.62	1.4	0.49	0.85	0.57
ENSMUSG00000090174 Gm10612	predicted gene 10612 [Source:MGI Symbol;Acc:MGI:3708796]	5.160780379	2.367589237	0.00032232	0.0014096	yes	up	0.12	0.524	0.13	0.09	0.09	0.07	0.22	0.64	0.27	0.92	0.39	0.4
ENSMUSG00000074219 Gm10644	predicted gene 10644 [Source:MGI Symbol;Acc:MGI:3704314]	0.469609933	-1.090465171	0.012065525	0.035246934	yes	down	0.766	0.304	0.47	1.04	0.72	1.1	0.5	0.2	0.29	0.16	0.26	0.61
ENSMUSG00000074461 Gm10699	predicted gene 10699 [Source:MGI Symbol;Acc:MGI:3642530]	0.318174747	-1.652108758	0.00098557	0.003868579	yes	down	0.602	0.16	0.54	0.69	0.49	0.56	0.73	0.16	0.17	0.12	0.05	0.3
ENSMUSG00000073018 Gm1070	predicted gene 1070 [Source:MGI Symbol;Acc:MGI:2685916]	2.950530352	1.560974299	0.016688785	0.046555558	yes	up	0.118	0.292	0.14	0.07	0	0.26	0.12	0.21	0.44	0.42	0.23	0.16
ENSMUSG00000112431 Gm10741	predicted gene 10741 [Source:MGI Symbol;Acc:MGI:3642299]	0.061348255	-4.026833885	0.001107212	0.004288761	yes	down	0.256	0.012	0.08	0.21	0.29	0.39	0.31	0	0	0.03	0	0.03

ENSMUSG00000079414	Gm11110	predicted gene 11110 [Source:MGI Symbol;Acc:MGI:3779362]	2.248603429	1.169029245	0.002439911	0.008691472	yes	up	0.856	1.626	0.75	0.29	1.03	1.13	1.08	2.05	0.99	1.62	1.93	1.54
ENSMUSG00000083161	Gm11427	predicted gene 11427 [Source:MGI Symbol;Acc:MGI:3651774]	6.412499995	2.68088692	3.76E-20	9.67E-19	yes	up	2.254	11.526	6.93	0.74	0.75	1.13	1.72	3.75	6.29	40.52	3.18	3.89
ENSMUSG00000087057	Gm11730	predicted gene 11730 [Source:MGI Symbol;Acc:MGI:3650941]	0.310244923	-1.688520494	1.40E-07	1.09E-06	yes	down	4.08	1.274	5.5	2.33	4.64	4.6	3.33	0.72	0.93	1.09	2.02	1.61
ENSMUSG00000086765	Gm11827	predicted gene 11827 [Source:MGI Symbol;Acc:MGI:3649801]	0.211906271	-2.238501814	0.000749838	0.003019656	yes	down	0.818	0.152	0.55	1.18	1.03	0.76	0.57	0.05	0.15	0.33	0.18	0.05
ENSMUSG00000085885	Gm11906	predicted gene 11906 [Source:MGI Symbol;Acc:MGI:3705114]	3.834502458	1.939039394	0.002181688	0.007853929	yes	up	0.302	0.976	0.1	0.55	0.62	0	0.24	1.08	0.93	0.74	0.38	1.75
ENSMUSG00000083178	Gm12187	predicted gene 12187 [Source:MGI Symbol;Acc:MGI:3651944]	3.757711862	1.909854446	0.018013272	0.049642548	yes	up	0.122	0.392	0.16	0.11	0.11	0.09	0.14	0.77	0.29	0.42	0.05	0.43
ENSMUSG00000085162	Gm12295	predicted gene 12295 [Source:MGI Symbol;Acc:MGI:3650535]	0.04498313	-4.474472142	0.008802523	0.026921722	yes	down	0.08	0	0.04	0.21	0.04	0.11	0	0	0	0	0	0
ENSMUSG00000053957	Gm12474	predicted gene 12474 [Source:MGI Symbol;Acc:MGI:3649378]	0.476315942	-1.070009258	0.011290544	0.033305549	yes	down	1.578	0.828	1.59	0.89	1.36	2.4	1.65	0.65	0.62	1.85	0.31	0.71
ENSMUSG00000095042	Gm12537	predicted gene 12537 [Source:MGI Symbol;Acc:MGI:3650720]	4.03825782	2.013733021	0.005446796	0.017700847	yes	up	0.354	1.234	0	0	0.51	0.72	0.54	0.95	1.61	0.51	1.16	1.94
ENSMUSG00000081169	Gm12551	predicted gene 12551 [Source:MGI Symbol;Acc:MGI:3651664]	19.85742891	4.311606934	2.08E-07	1.58E-06	yes	up	0.13	1.836	0	0.42	0	0.23	0	1.45	1.97	2.56	1.93	1.27
ENSMUSG00000085181	Gm12709	predicted gene 12709 [Source:MGI Symbol;Acc:MGI:3651946]	0.218272416	-2.195798273	0.00056525	0.002335594	yes	down	2.012	0.468	2.77	1.13	1.51	1.38	3.27	0.08	0.98	0.52	0.05	0.71
ENSMUSG00000086320	Gm12840	predicted gene 12840 [Source:MGI Symbol;Acc:MGI:3650917]	0.27529723	-1.860938003	4.41E-15	7.64E-14	yes	down	11.202	3.442	8.75	14.83	10.43	10.54	11.46	2.19	4.57	3.44	3.93	3.08
ENSMUSG00000078502	Gm13212	predicted gene 13212 [Source:MGI Symbol;Acc:MGI:3651014]	2.861532843	1.516788165	5.92E-16	1.10E-14	yes	up	8.322	12.882	8.45	11.41	10.43	5.52	5.8	16.02	14.77	13.36	11.14	9.12
ENSMUSG00000073716	Gm13241	predicted gene 13241 [Source:MGI Symbol;Acc:MGI:3649920]	8.915501377	3.156315932	0.000403561	0.001725428	yes	up	0.164	1.328	0.12	0	0	0.21	0.49	1.52	1.78	1.35	0.2	1.79
ENSMUSG00000086358	Gm13270	predicted gene 13270 [Source:MGI Symbol;Acc:MGI:3651704]	5.268463944	2.397382396	0.013856636	0.039630571	yes	up	0.066	0.236	0.02	0.22	0.05	0	0.04	0.35	0.49	0.09	0.04	0.21
ENSMUSG00000094271	Gm13290	predicted gene 13290 [Source:MGI Symbol;Acc:MGI:3701990]	19.37522348	4.276141046	0.013022244	0.037641299	yes	up	0	0.262	0	0	0	0	0	0	0.34	0.22	0.61	0.14
ENSMUSG00000096862	Gm13301	predicted gene 13301 [Source:MGI Symbol;Acc:MGI:3707230]	0.18656663	-2.422237133	0.016207762	0.045432647	yes	down	0.51	0.08	0.98	0.11	0.81	0.59	0.06	0	0.21	0	0	0.19
ENSMUSG00000073878	Gm13304	predicted gene 13304 [Source:MGI Symbol;Acc:MGI:3710514]	8.123891666	3.022171001	0.001299542	0.004946851	yes	up	0.186	1.326	0	0.18	0.21	0	0.54	1.49	1.51	1.93	1.7	0
ENSMUSG00000085730	Gm13307	predicted gene 13307 [Source:MGI Symbol;Acc:MGI:3701135]	0.12051412	-3.05272591	0.003773432	0.012766481	yes	down	2.018	0.216	2.97	0.81	1.59	4.37	0.35	0	0	0.66	0.05	0.37
ENSMUSG00000087028	Gm13387	predicted gene 13387 [Source:MGI Symbol;Acc:MGI:3651242]	0.190875432	-2.389296672	3.19E-09	3.08E-08	yes	down	12.624	1.75	17.36	10.19	9.07	14.83	11.67	2	2.71	0.93	2.39	0.72
ENSMUSG00000086109	Gm13391	predicted gene 13391 [Source:MGI Symbol;Acc:MGI:3651526]	11.50678476	3.524412865	0.004153493	0.013902971	yes	up	0.092	0.956	0	0.12	0.34	0	0	1.5	1.44	0	1.31	0.53
ENSMUSG00000086862	Gm13546	predicted gene 13546 [Source:MGI Symbol;Acc:MGI:3649666]	0.336122616	-1.572940477	0.001392975	0.005266942	yes	down	1.57	0.446	1.85	1.22	1.81	1.39	1.58	0.71	0.39	0.65	0.28	0.2
ENSMUSG00000085543	Gm13568	predicted gene 13568 [Source:MGI Symbol;Acc:MGI:3702049]	0.411064919	-1.28256184	0.004876563	0.016027696	yes	down	10.734	3.714	8.02	7.28	11.7	19.28	7.39	4.15	2.93	3.01	6.87	1.61
ENSMUSG00000086196	Gm13571	predicted gene 13571 [Source:MGI Symbol;Acc:MGI:3652223]	23.8356378	4.575048325	2.65E-11	3.18E-10	yes	up	0.06	1.29	0	0	0	0.13	0.17	1.02	1.68	1.13	1.31	1.31
ENSMUSG00000075325	Gm13582	predicted gene 13582 [Source:MGI Symbol;Acc:MGI:3642495]	0.379130226	-1.399234615	3.59E-06	2.27E-05	yes	down	1.318	0.416	1.07	1.72	1.42	1.3	1.08	0.32	0.3	0.3	0.54	0.62
ENSMUSG00000075312	Gm13597	predicted gene 13597 [Source:MGI Symbol;Acc:MGI:3651139]	0.285411243	-1.808885928	0.001048825	0.004089136	yes	down	29.264	7.074	18.83	39.55	22.69	31.25	34	20.86	2.78	7.92	2.48	1.33
ENSMUSG00000082345	Gm13622	predicted gene 13622 [Source:MGI Symbol;Acc:MGI:3652182]	15.90239373	3.99117204	0.0114553	0.033721884	yes	up	0.078	1.436	0.39	0	0	0	0	2.42	2.52	0	0.34	1.9
ENSMUSG00000082152	Gm13655	predicted gene 13655 [Source:MGI Symbol;Acc:MGI:3650029]	0.183716692	-2.444445385	0.002621849	0.00925796	yes	down	1.208	0.192	1.54	0.67	0.93	1.35	1.55	0.44	0.1	0	0.11	0.31
ENSMUSG00000085976	Gm13816	predicted gene 13816 [Source:MGI Symbol;Acc:MGI:3649317]	0.424587779	-1.235865249	0.00032194	0.001408223	yes	down	6.128	2.174	8.64	5.59	4.39	7.5	4.52	3.27	1.62	2.34	2.22	1.42
ENSMUSG00000084897	Gm14226	predicted gene 14226 [Source:MGI Symbol;Acc:MGI:3649244]	0.220587286	-2.180578454	3.02E-06	1.93E-05	yes	down	1.1	0.206	1.93	0.83	0.45	0.92	1.37	0.32	0.28	0.24	0.05	0.14

ENSMUSG00000078868	Gm14412	predicted gene 14412 [Source:MGI Symbol;Acc:MGI:3652251]	0.481017909	-1.055837488	0.002869612	0.010033569	yes	down	14.176	8.354	2.44	0.89	6.54	41.95	19.06	0.82	8.01	7.91	9.01	16.02
ENSMUSG00000082724	Gm14416	predicted gene 14416 [Source:MGI Symbol;Acc:MGI:3702407]	0.02392488	-5.385344483	0.000299299	0.001317801	yes	down	0.216	0	0	0.05	0.28	0.34	0.41	0	0	0	0	0
ENSMUSG00000083111	Gm14421	predicted gene 14421 [Source:MGI Symbol;Acc:MGI:3652254]	0.467084674	-1.098243986	0.000236051	0.001064716	yes	down	1.628	0.634	1.49	1.94	1.67	1.86	1.18	0.81	0.6	0.4	0.73	0.63
ENSMUSG00000054510	Gm14461	predicted gene 14461 [Source:MGI Symbol;Acc:MGI:3651589]	49.98450942	5.643409157	6.34E-06	3.82E-05	yes	up	0	0.6	0	0	0	0	0	1.13	0.64	0.88	0.26	0.09
ENSMUSG00000081680	Gm14473	predicted gene 14473 [Source:MGI Symbol;Acc:MGI:3650130]	0.026937147	-5.21425914	0.001470996	0.005529726	yes	down	1.154	0	4.44	0.56	0	0.46	0.31	0	0	0	0	0
ENSMUSG00000074417	Gm14548	predicted gene 14548 [Source:MGI Symbol;Acc:MGI:3709645]	2.500171274	1.32202693	6.43E-09	6.00E-08	yes	up	1.9	4.33	0.92	1.8	2.02	2.81	1.95	3.86	3.86	4.11	3.79	6.03
ENSMUSG00000091556	Gm14569	predicted gene 14569 [Source:MGI Symbol;Acc:MGI:3705243]	0.479606348	-1.06007734	0.013765522	0.039421601	yes	down	0.946	0.412	0.81	1.28	0.88	0.51	1.25	0.89	0.17	0.08	0.27	0.65
ENSMUSG00000052188	Gm14964	predicted gene 14964 [Source:MGI Symbol;Acc:MGI:3641621]	0.106318499	-3.233535458	7.61E-21	2.04E-19	yes	down	9.008	0.962	6.91	7.45	8.56	10.02	12.1	1.75	0.56	0.38	1.49	0.63
ENSMUSG00000079261	Gm15217	predicted gene 15217 [Source:MGI Symbol;Acc:MGI:3705233]	0.097518594	-3.358178861	0.017909977	0.04942216	yes	down	0.526	0.042	0.13	0.14	0.41	1.04	0.91	0	0	0.1	0.11	0
ENSMUSG00000086480	Gm15287	predicted gene 15287 [Source:MGI Symbol;Acc:MGI:3705156]	0.103343952	-3.274474141	0.012124994	0.035396521	yes	down	0.708	0.064	1.57	0.13	0.91	0.71	0.22	0	0.2	0.12	0	0
ENSMUSG00000074419	Gm15448	predicted gene 15448 [Source:MGI Symbol;Acc:MGI:3705216]	6.449785341	2.689251146	4.08E-05	0.000212179	yes	up	0.22	1.062	0	0.39	0.51	0.2	0	1.15	1.15	1.34	1.21	0.46
ENSMUSG00000087001	Gm15475	predicted gene 15475 [Source:MGI Symbol;Acc:MGI:3705288]	0.264636736	-1.917914747	1.74E-08	1.54E-07	yes	down	1.612	0.25	1.05	3.77	1.48	0.88	0.88	0.17	0.22	0.3	0.16	0.4
ENSMUSG00000086291	Gm15513	predicted gene 15513 [Source:MGI Symbol;Acc:MGI:3782961]	3.374918225	1.754852546	0.000725297	0.002929089	yes	up	0.246	0.644	0.16	0.06	0.39	0.41	0.21	0.48	0.95	0.57	0.34	0.88
ENSMUSG00000087371	Gm15541	predicted gene 15541 [Source:MGI Symbol;Acc:MGI:3782989]	0.406376066	-1.29911266	2.94E-05	0.000157248	yes	down	1.87	0.466	1.54	2.34	1.46	2.68	1.33	0.28	0.62	0.5	0.34	0.59
ENSMUSG00000083396	Gm15542	predicted gene 15542 [Source:MGI Symbol;Acc:MGI:3782990]	3.445675776	1.784786956	0.015807778	0.044486119	yes	up	0.406	1.102	0.32	0.17	0.8	0.74	0	2.25	1.55	0.67	0.68	0.36
ENSMUSG00000085913	Gm15601	predicted gene 15601 [Source:MGI Symbol;Acc:MGI:3783048]	4.993525598	2.320058767	2.00E-13	2.98E-12	yes	up	0.37	1.552	0.46	0.32	0.31	0.49	0.27	2.11	2.02	1.23	0.71	1.69
ENSMUSG00000086414	Gm15645	predicted gene 15645 [Source:MGI Symbol;Acc:MGI:3783089]	2.770698094	1.470249518	0.00744915	0.023326657	yes	up	14.79	35.122	19.95	18.1	0	25.71	10.19	52.59	54.61	21.78	23.96	22.67
ENSMUSG00000090214	Gm15657	predicted gene 15657 [Source:MGI Symbol;Acc:MGI:3783100]	0.476113999	-1.070621046	0.00060569	0.002483526	yes	down	1.78	0.72	2.69	1.13	2.16	0.99	1.93	0.74	0.99	0.4	1.04	0.43
ENSMUSG00000079462	Gm15737	predicted gene 15737 [Source:MGI Symbol;Acc:MGI:3783179]	0.117644074	-3.087499439	0.004516748	0.014990032	yes	down	0.084	0.008	0.06	0.11	0.05	0.09	0.11	0.03	0	0	0.01	0
ENSMUSG00000086868	Gm15883	predicted gene 15883 [Source:MGI Symbol;Acc:MGI:3801875]	0.387160956	-1.368994626	0.001026504	0.004010874	yes	down	3.766	1.232	3.87	1.73	3.02	7.56	2.65	1.19	2.68	0.65	0.81	0.83
ENSMUSG00000085881	Gm15912	predicted gene 15912 [Source:MGI Symbol;Acc:MGI:3801895]	0.419202887	-1.254279441	0.011299839	0.033328379	yes	down	1.94	0.67	3.34	2.32	1.63	0.87	1.54	0.33	1.32	0.91	0.33	0.46
ENSMUSG00000081665	Gm15922	predicted gene 15922 [Source:MGI Symbol;Acc:MGI:3802148]	4.498059839	2.169302854	2.59E-25	9.17E-24	yes	up	1.312	4.952	1.4	1.84	1.17	1.44	0.71	4.01	6.06	5.85	4.55	4.29
ENSMUSG00000081723	Gm15931	predicted gene 15931 [Source:MGI Symbol;Acc:MGI:3805553]	3.421665744	1.774698832	3.41E-63	4.94E-61	yes	up	2.206	7.122	2.2	1.85	2.19	2.48	2.31	6.33	7.67	5.5	8.21	7.9
ENSMUSG00000085055	Gm15958	predicted gene 15958 [Source:MGI Symbol;Acc:MGI:3801839]	0.0444853	-4.490527505	0.000557494	0.002308449	yes	down	0.666	0.008	0.1	0.76	0.07	0.9	1.5	0	0.04	0	0	0
ENSMUSG00000085337	Gm15964	predicted gene 15964 [Source:MGI Symbol;Acc:MGI:3802003]	3.465069244	1.792884183	8.20E-05	0.000404603	yes	up	0.198	0.56	0.22	0.25	0.21	0.1	0.21	0.77	0.4	0.28	0.62	0.73
ENSMUSG00000090002	Gm16006	predicted gene 16006 [Source:MGI Symbol;Acc:MGI:3801853]	0.305778982	-1.709438851	0.00774981	0.02413055	yes	down	3.88	1.008	4.64	1.55	2.17	3.32	7.72	0.72	0.67	1.79	0.18	1.68
ENSMUSG00000081343	Gm16071	predicted gene 16071 [Source:MGI Symbol;Acc:MGI:3801777]	19.40243858	4.278166083	0.011206401	0.033089242	yes	up	0	0.336	0	0	0	0	0	0.39	0.46	0.54	0.29	0
ENSMUSG00000086245	Gm16170	predicted gene 16170 [Source:MGI Symbol;Acc:MGI:3805548]	2.343092685	1.228414023	0.013737041	0.039361066	yes	up	0.19	0.37	0.07	0.25	0.32	0.24	0.07	0.49	0.43	0.48	0.19	0.26
ENSMUSG00000085368	Gm16188	predicted gene 16188 [Source:MGI Symbol;Acc:MGI:3802067]	0.451726929	-1.146477176	0.005995846	0.019268762	yes	down	2.746	0.702	1.46	1.61	1.17	7.54	1.95	0.22	0.73	1.58	0.57	0.41
ENSMUSG00000085174	Gm16206	predicted gene 16206 [Source:MGI Symbol;Acc:MGI:3801870]	4.035765203	2.012842242	0.003253234	0.011192171	yes	up	0.104	0.356	0.04	0.07	0.11	0.18	0.12	0.61	0.44	0.13	0.32	0.28

ENSMUSG00000086709	Gm16263	predicted gene 16263 [Source:MGI Symbol;Acc:MGI:3826536]	0.490044968	-1.029013954	0.001440376	0.005423189	yes	down	1.804	1.596	1.51	1.79	2.43	1.42	1.87	0.41	1.71	3.48	1.09	1.29
ENSMUSG00000084862	Gm16278	predicted gene 16278 [Source:MGI Symbol;Acc:MGI:3826602]	3.34193498	1.740683665	0.000817894	0.003264262	yes	up	0.196	0.552	0.26	0.14	0.05	0.26	0.27	0.27	0.63	0.68	0.41	0.77
ENSMUSG00000090024	Gm16350	predicted gene 16350 [Source:MGI Symbol;Acc:MGI:3840133]	23.06743854	4.527785908	0.005964537	0.019187148	yes	up	0	1.274	0	0	0	0	0	0.96	0	0.85	2.51	2.05
ENSMUSG00000082154	Gm16464	predicted gene 16464 [Source:MGI Symbol;Acc:MGI:3643716]	0.360810738	-1.47068582	0.014456294	0.041147793	yes	down	0.996	0.296	1.22	0.39	1.13	1.08	1.16	0.61	0.27	0.35	0.09	0.16
ENSMUSG00000108255	Gm16499	predicted gene 16499 [Source:MGI Symbol;Acc:MGI:3704267]	0.255082025	-1.970966855	0.00186838	0.006849167	yes	down	0.662	0.142	0.38	0.27	1.01	0.72	0.93	0.1	0.28	0.11	0	0.22
ENSMUSG00000087646	Gm1667	predicted gene 1667 [Source:MGI Symbol;Acc:MGI:2686513]	0.294927624	-1.761567137	0.007116159	0.022395247	yes	down	3.108	0.88	4.52	2.21	3.69	3.61	1.51	1.09	1.06	0.21	1.83	0.21
ENSMUSG00000097804	Gm16685	predicted gene, 16685 [Source:MGI Symbol;Acc:MGI:4439609]	2.215640874	1.147724059	0.007414213	0.023235515	yes	up	11.974	0.964	0.35	0.7	0.32	0.14	58.36	1.56	0.98	0.81	0.87	0.6
ENSMUSG00000097077	Gm16712	predicted gene, 16712 [Source:MGI Symbol;Acc:MGI:4439636]	7.143386576	2.836608197	7.79E-05	0.000386031	yes	up	0.084	0.496	0.18	0.11	0.07	0.06	0	0.38	0.91	0.43	0.18	0.58
ENSMUSG00000097665	Gm16853	predicted gene, 16853 [Source:MGI Symbol;Acc:MGI:4439777]	0.036781006	-4.764895261	0.000706128	0.002858683	yes	down	0.162	0	0.07	0.21	0.14	0.09	0.3	0	0	0	0	0
ENSMUSG00000090576	Gm17055	predicted gene 17055 [Source:MGI Symbol;Acc:MGI:4937882]	0.341325639	-1.550779307	0.007804825	0.024273617	yes	down	0.4	0.12	0.45	0.52	0.19	0.39	0.45	0.08	0.23	0.14	0.05	0.1
ENSMUSG00000091509	Gm17066	predicted gene 17066 [Source:MGI Symbol;Acc:MGI:4937893]	0.362648343	-1.463356839	0.008958891	0.027331	yes	down	2.944	0.704	2.46	2.54	4.07	5.13	0.52	1.16	0.7	1.26	0.09	0.31
ENSMUSG00000091730	Gm17230	predicted gene 17230 [Source:MGI Symbol;Acc:MGI:4938057]	27.28353767	4.769958816	0.000285954	0.001266074	yes	up	0.002	0.096	0	0	0	0.01	0	0.21	0.1	0.01	0.07	0.09
ENSMUSG00000097347	Gm17275	predicted gene, 17275 [Source:MGI Symbol;Acc:MGI:4936909]	2.474525218	1.307151745	0.000107894	0.000519599	yes	up	1.61	3.352	0.48	1.93	1.95	1.98	1.71	5.45	4.02	2.2	2.25	2.84
ENSMUSG00000097805	Gm17473	predicted gene, 17473 [Source:MGI Symbol;Acc:MGI:4937107]	0.213477602	-2.227843382	1.78E-06	1.17E-05	yes	down	1.058	0.192	1.33	1.32	0.42	1.33	0.89	0.21	0.12	0.14	0.34	0.15
ENSMUSG00000091514	Gm17484	predicted gene, 17484 [Source:MGI Symbol;Acc:MGI:4937118]	3.50205773	1.808202866	0.010081099	0.030233414	yes	up	0.12	0.362	0.14	0.05	0.23	0.14	0.04	0.24	0.58	0.07	0.58	0.34
ENSMUSG00000111114	Gm18703	predicted gene, 18703 [Source:MGI Symbol;Acc:MGI:5010888]	0.041304618	-4.597553108	0.014761794	0.041889303	yes	down	3.98	0	0	11.72	0	3.21	4.97	0	0	0	0	0
ENSMUSG00000092438	Gm18734	predicted gene, 18734 [Source:MGI Symbol;Acc:MGI:5010919]	2.213980235	1.146642343	7.43E-05	0.000369044	yes	up	10.934	19.808	10.25	6.94	10.75	17.43	9.3	24.99	25.46	22.02	7.89	18.68
ENSMUSG00000116605	Gm19142	predicted gene, 19142 [Source:MGI Symbol;Acc:MGI:5011327]	29.10112794	4.863003167	4.84E-08	4.01E-07	yes	up	0.034	0.834	0.04	0	0.05	0	0.08	1.31	0.77	1.1	0.6	0.39
ENSMUSG00000111556	Gm19299	predicted gene, 19299 [Source:MGI Symbol;Acc:MGI:5011484]	0.384428106	-1.37921428	0.001123646	0.004347605	yes	down	1.03	0.332	0.86	1.31	1.14	0.65	1.19	0.21	0.38	0.41	0.24	0.42
ENSMUSG00000115426	Gm19510	predicted gene, 19510 [Source:MGI Symbol;Acc:MGI:5011695]	20.51108809	4.358332122	0.01477281	0.04191501	yes	up	0	0.112	0	0	0	0	0	0.12	0.03	0.06	0	0.35
ENSMUSG00000116903	Gm19522	predicted gene, 19522 [Source:MGI Symbol;Acc:MGI:5011707]	0.404959009	-1.304152213	2.36E-05	0.000128531	yes	down	1.838	0.616	2.31	1.64	1.94	1.42	1.88	0.55	0.5	0.6	1.04	0.39
ENSMUSG00000073902	Gm1966	predicted gene 1966 [Source:MGI Symbol;Acc:MGI:3584360]	2.322958904	1.215963631	1.75E-13	2.63E-12	yes	up	4.854	9.478	3.53	5.14	5.67	6.23	3.7	13.11	8.12	8.64	7.36	10.16
ENSMUSG00000103983	Gm20045	predicted gene, 20045 [Source:MGI Symbol;Acc:MGI:5012230]	3.308998974	1.726394844	1.61E-06	1.07E-05	yes	up	0.184	0.514	0.27	0.1	0.14	0.24	0.17	0.8	0.55	0.4	0.32	0.5
ENSMUSG00000103847	Gm20056	predicted gene, 20056 [Source:MGI Symbol;Acc:MGI:5012241]	29.13784925	4.864822487	3.32E-07	2.44E-06	yes	up	0.178	4.66	0	0	0	0.89	0	8.96	2.41	3.74	4.54	3.65
ENSMUSG00000110245	Gm20100	predicted gene, 20100 [Source:MGI Symbol;Acc:MGI:5012285]	6.054234441	2.597944542	0.009642572	0.029105755	yes	up	0.034	0.17	0.06	0.03	0	0.05	0.03	0.3	0.14	0.27	0.02	0.12
ENSMUSG00000092176	Gm20460	predicted gene 20460 [Source:MGI Symbol;Acc:MGI:5141925]	5.16185352	2.367889202	4.48E-08	3.73E-07	yes	up	3.606	15.448	3.14	2.89	4.78	0.95	6.27	15.76	15.05	13.92	14.65	17.86
ENSMUSG00000093765	Gm20658	predicted gene 20658 [Source:MGI Symbol;Acc:MGI:5313105]	8.692171953	3.119716715	0.015767812	0.044387797	yes	up	0.02	0.162	0	0	0	0.03	0.07	0.3	0.21	0.08	0.16	0.06
ENSMUSG00000106461	Gm20755	predicted gene, 20755 [Source:MGI Symbol;Acc:MGI:5434111]	0.306880344	-1.704251852	0.005209024	0.01701834	yes	down	4.354	0.224	17.78	2.5	0.43	0.51	0.55	0.03	0.37	0.38	0.19	0.15
ENSMUSG00000097789	Gm2115	predicted gene 2115 [Source:MGI Symbol;Acc:MGI:3780284]	0.371000704	-1.430506169	1.85E-09	1.82E-08	yes	down	2.038	0.816	1.91	2.35	2.22	1.65	2.06	0.3	1.19	1.44	0.58	0.57
ENSMUSG00000095609	Gm21188	predicted gene, 21188 [Source:MGI Symbol;Acc:MGI:5434543]	5.640814263	2.495903434	2.50E-25	8.87E-24	yes	up	2.984	11.554	2.24	3.84	2.61	4.23	2	12.23	8.42	12.48	9.98	14.66

ENSMUSG00000096327	Gm21980	predicted gene 21980 [Source:MGI Symbol;Acc:MGI:5439449]	0.237735781	-2.072569038	1.96E-10	2.14E-09	yes	down	6.87	1.362	6.62	7.34	4.23	11.22	4.94	1.84	1.39	1.59	1.38	0.61
ENSMUSG00000098369	Gm2274	predicted gene 2274 [Source:MGI Symbol;Acc:MGI:3780444]	0.47287577	-1.080466874	0.000550991	0.002282882	yes	down	4.396	1.758	1.69	3.27	4.81	4.69	7.52	2.56	1.46	1.61	1.39	1.77
ENSMUSG00000086119	Gm2415	predicted gene 2415 [Source:MGI Symbol;Acc:MGI:3780582]	0.450423933	-1.150644608	5.72E-13	8.10E-12	yes	down	8.268	5.442	7.9	7.61	6.53	10.5	8.8	3.18	3.28	14.25	2.87	3.63
ENSMUSG00000092849	Gm24327	predicted gene, 24327 [Source:MGI Symbol;Acc:MGI:5454104]	0.277545856	-1.849201945	6.89E-07	4.84E-06	yes	down	4.178	0.984	4.42	4.48	3.71	4.37	3.91	0.13	0.77	1.3	1.76	0.96
ENSMUSG00000091938	Gm2564	predicted gene 2564 [Source:MGI Symbol;Acc:MGI:3708691]	2.337084508	1.224709903	0.017665298	0.048872241	yes	up	1.3	2.504	0.89	1.59	1.08	1.8	1.14	1.9	1.07	3.79	1.47	4.29
ENSMUSG00000088646	Gm26486	predicted gene, 26486 [Source:MGI Symbol;Acc:MGI:5456263]	7.685961362	2.942225724	0.012528007	0.036414197	yes	up	0.116	0.746	0	0.4	0.1	0	0.08	0.41	0	0.6	0.57	2.15
ENSMUSG00000097423	Gm26522	predicted gene, 26522 [Source:MGI Symbol;Acc:MGI:5477016]	9.5874172	3.261142213	4.82E-05	0.000246822	yes	up	0.098	0.778	0.05	0.19	0.05	0.2	0	1.71	0.83	0.75	0.16	0.44
ENSMUSG00000097737	Gm26530	predicted gene, 26530 [Source:MGI Symbol;Acc:MGI:5477024]	3.547792242	1.826921528	0.002235634	0.008029236	yes	up	0.08	0.224	0.18	0.02	0.07	0.09	0.04	0.32	0.29	0.22	0.15	0.14
ENSMUSG00000097501	Gm26536	predicted gene, 26536 [Source:MGI Symbol;Acc:MGI:5477030]	14.30826829	3.83877717	0.008905585	0.027183866	yes	up	0.028	0.308	0.07	0	0	0.07	0	0.36	0.75	0.05	0	0.38
ENSMUSG00000096945	Gm26546	predicted gene, 26546 [Source:MGI Symbol;Acc:MGI:5477040]	2.305518459	1.205091217	0.008338975	0.02569974	yes	up	0.176	0.342	0.3	0.27	0.03	0.13	0.15	0.33	0.37	0.3	0.32	0.39
ENSMUSG00000097217	Gm26549	predicted gene, 26549 [Source:MGI Symbol;Acc:MGI:5477043]	4.269522182	2.094074622	0.002877554	0.010051491	yes	up	0.212	0.76	0.13	0.46	0.2	0.11	0.16	1.29	0.9	0.49	0.16	0.96
ENSMUSG00000096958	Gm26573	predicted gene, 26573 [Source:MGI Symbol;Acc:MGI:5477067]	4.908745907	2.29535449	3.02E-11	3.60E-10	yes	up	35.392	78.438	27.77	74.15	37.52	13.82	23.7	67.46	89.68	48.8	62.89	123.36
ENSMUSG00000097474	Gm26584	predicted gene, 26584 [Source:MGI Symbol;Acc:MGI:5477078]	3.650383997	1.868048234	3.95E-31	1.93E-29	yes	up	0.698	2.138	0.75	0.59	0.99	0.61	0.55	2	2.31	2.17	2.24	1.97
ENSMUSG00000097339	Gm26671	predicted gene, 26671 [Source:MGI Symbol;Acc:MGI:5477165]	0.354794223	-1.494945575	3.09E-05	0.000164147	yes	down	5.694	1.604	6.92	4.46	4.33	5.62	7.14	1.72	3.34	1.12	0.53	1.31
ENSMUSG00000097213	Gm26745	predicted gene, 26745 [Source:MGI Symbol;Acc:MGI:5477239]	3.036936812	1.602616891	0.000629883	0.002571891	yes	up	3.466	9.756	2.53	1.33	5.61	2.94	4.92	14.3	5.68	11	9.37	8.43
ENSMUSG00000097484	Gm26807	predicted gene, 26807 [Source:MGI Symbol;Acc:MGI:5477301]	84.2944381	6.397365538	4.97E-08	4.11E-07	yes	up	0	0.182	0	0	0	0	0	0.05	0.33	0.23	0.07	0.23
ENSMUSG00000097722	Gm26841	predicted gene, 26841 [Source:MGI Symbol;Acc:MGI:5477335]	0.202487373	-2.304096152	0.000180587	0.000830996	yes	down	0.73	0.116	0.39	0.53	1.41	0.75	0.57	0.05	0.21	0.05	0.06	0.21
ENSMUSG00000097087	Gm26878	predicted gene, 26878 [Source:MGI Symbol;Acc:MGI:5477372]	0.373749633	-1.41985593	0.000375909	0.001618836	yes	down	2	0.632	1.7	2.95	1.11	2.94	1.3	0.62	1.11	0.4	0.62	0.41
ENSMUSG00000097558	Gm26902	predicted gene, 26902 [Source:MGI Symbol;Acc:MGI:5477396]	2.579612235	1.367154218	0.002778096	0.009739025	yes	up	0.262	0.782	0.19	0.22	0.52	0.09	0.29	0.41	0.45	1.29	1.09	0.67
ENSMUSG00000097835	Gm26910	predicted gene, 26910 [Source:MGI Symbol;Acc:MGI:5477404]	0.37570135	-1.412341797	0.014861768	0.042122767	yes	down	2.33	0.748	3.59	1.34	3.24	1.5	1.98	0.85	1.24	0.22	1.09	0.34
ENSMUSG00000097971	Gm26917	predicted gene, 26917 [Source:MGI Symbol;Acc:MGI:5504032]	4.960556697	2.310502036	2.36E-07	1.78E-06	yes	up	5.074	18.134	4.66	7.35	3.98	5.55	3.83	30.18	30.99	10.59	10.57	8.34
ENSMUSG00000097248	Gm2694	predicted gene 2694 [Source:MGI Symbol;Acc:MGI:3780864]	46.51741502	5.539699023	6.46E-06	3.89E-05	yes	up	0	1.734	0	0	0	0	0	1.56	1.48	0.51	2.24	2.88
ENSMUSG00000098306	Gm28040	predicted gene, 28040 [Source:MGI Symbol;Acc:MGI:5547776]	0.026241054	-5.252030506	0.000161609	0.000751252	yes	down	0.53	0.006	1.45	0	0.38	0.33	0.49	0	0	0.03	0	0
ENSMUSG00000084902	Gm281	predicted gene 281 [Source:MGI Symbol;Acc:MGI:2685127]	0.458136592	-1.126150299	0.000477517	0.002009138	yes	down	4.704	1.824	4.71	5.98	3.12	3.47	6.24	1.51	1.37	3.78	0.9	1.56
ENSMUSG00000100108	Gm28100	predicted gene 28100 [Source:MGI Symbol;Acc:MGI:5578806]	16.6141344	4.054339226	0.005126109	0.016775787	yes	up	0.03	0.46	0	0	0	0	0.15	1.05	0.45	0.35	0.13	0.32
ENSMUSG00000101628	Gm28177	predicted gene 28177 [Source:MGI Symbol;Acc:MGI:5578883]	2.073306063	1.051933104	0.00014992	0.000703018	yes	up	23.758	33.854	13.88	17.23	40.42	27.56	19.7	36.67	25.55	29.68	24.66	52.71
ENSMUSG00000100502	Gm28286	predicted gene 28286 [Source:MGI Symbol;Acc:MGI:5578992]	26.06491676	4.704037348	0.001329159	0.005046139	yes	up	0.034	1.136	0	0	0	0.17	0	2.82	1.23	0	1.32	0.31
ENSMUSG00000101711	Gm28306	predicted gene 28306 [Source:MGI Symbol;Acc:MGI:5579012]	19.46684625	4.282947272	0.017391446	0.048232267	yes	up	0	0.722	0	0	0	0	0	0.23	0	0.38	0.87	2.13
ENSMUSG00000099767	Gm28884	predicted gene 28884 [Source:MGI Symbol;Acc:MGI:5579590]	6.903755672	2.787381408	0.001281869	0.004888269	yes	up	0.02	0.12	0.03	0	0	0.04	0.03	0.15	0.06	0.15	0.13	0.11
ENSMUSG00000100037	Gm29103	predicted gene 29103 [Source:MGI Symbol;Acc:MGI:5579809]	0.140204424	-2.834396224	0.000714344	0.002889759	yes	down	2.8	0.33	1.98	3.29	5.96	0.64	2.13	0	0.41	0.52	0.72	0

ENSMUSG00000101249 Gm29216	predicted gene 29216 [Source:MGI Symbol;Acc:MGI:5579922]	3.367530936	1.751691199	0.002750153	0.009647382	yes	up	497.94	1428.276	82.63	359.59	239.06	663.14	1145.28	797.39	1508.57	1875.51	1330.06	1629.85
ENSMUSG00000101903 Gm29291	predicted gene 29291 [Source:MGI Symbol;Acc:MGI:5579997]	6.632312979	2.729512089	0.004586453	0.015202521	yes	up	0.202	1.172	0	0	0	0.72	0.29	1.7	1.44	0.64	0.93	1.15
ENSMUSG00000101625 Gm29371	predicted gene 29371 [Source:MGI Symbol;Acc:MGI:5580077]	0.271770579	-1.879538812	3.44E-16	6.56E-15	yes	down	6.262	1.444	5.9	9.21	5.2	3.5	7.5	1.32	1.54	1.93	1.23	1.2
ENSMUSG00000099553 Gm29538	predicted gene 29538 [Source:MGI Symbol;Acc:MGI:5580244]	0.481070836	-1.055678752	0.008758844	0.026816213	yes	down	11.728	4.676	8.72	17.45	10.29	7.86	14.32	3.59	5.79	6.43	1.67	5.9
ENSMUSG00000000594 Gm2a	GM2 ganglioside activator protein [Source:MGI Symbol;Acc:MGI:95762]	3.107048819	1.63554491	2.11E-95	6.53E-93	yes	up	67.926	175.872	59.31	75.33	69.72	65.88	69.39	164.89	176.7	162.27	181.58	193.92
ENSMUSG00000107318 Gm30003	predicted gene, 30003 [Source:MGI Symbol;Acc:MGI:5589162]	0.080504019	-3.634795389	0.013373668	0.038501121	yes	down	0.166	0.008	0.15	0.05	0.32	0.18	0.13	0	0.04	0	0	0
ENSMUSG00000105646 Gm30211	predicted gene, 30211 [Source:MGI Symbol;Acc:MGI:5589370]	10.21276002	3.352300905	5.03E-13	7.19E-12	yes	up	20.61	233.23	5.82	24.29	32.6	34	6.34	290.72	190.9	317.84	179.08	187.61
ENSMUSG00000110388 Gm30329	predicted gene, 30329 [Source:MGI Symbol;Acc:MGI:5589488]	9.254877573	3.210213906	0.001613203	0.006006322	yes	up	0.02	0.158	0.02	0.02	0.02	0	0.04	0.25	0.34	0.08	0.09	0.03
ENSMUSG00000106515 Gm30382	predicted gene, 30382 [Source:MGI Symbol;Acc:MGI:5589541]	0.12475411	-3.002840753	0.017065826	0.047458996	yes	down	0.656	0.14	0.23	2.6	0.08	0.27	0.1	0	0	0.03	0	0.67
ENSMUSG00000106139 Gm30648	predicted gene, 30648 [Source:MGI Symbol;Acc:MGI:5589807]	0.152738821	-2.710861299	3.21E-10	3.44E-09	yes	down	2.478	0.392	2.45	2.56	1.21	3.86	2.31	0.03	1.03	0.17	0.42	0.31
ENSMUSG00000108417 Gm30928	predicted gene, 30928 [Source:MGI Symbol;Acc:MGI:5590087]	0.487356693	-1.036950038	4.72E-09	4.46E-08	yes	down	8.514	8.11	6.8	8.27	11.79	4.24	11.47	10.76	1.91	12.94	13.41	1.53
ENSMUSG00000109587 Gm31105	predicted gene, 31105 [Source:MGI Symbol;Acc:MGI:5590264]	0.38602882	-1.373219536	0.014858013	0.042122767	yes	down	2.25	0.742	4.57	1.46	2.07	1.08	2.07	0.77	0.6	0.58	0.88	0.88
ENSMUSG00000110697 Gm31718	predicted gene, 31718 [Source:MGI Symbol;Acc:MGI:5590877]	3.113071998	1.638338943	0.002975741	0.010357301	yes	up	0.62	2.486	0.09	0.31	0.35	1.81	0.54	2.87	1.15	6.51	0.55	1.35
ENSMUSG00000115869 Gm31814	predicted gene, 31814 [Source:MGI Symbol;Acc:MGI:5590973]	3.93221043	1.975340529	0.003606712	0.012264426	yes	up	0.63	2.1	0.15	0.54	1.57	0.44	0.45	4.6	1.24	0.92	1.55	2.19
ENSMUSG00000111602 Gm32014	predicted gene, 32014 [Source:MGI Symbol;Acc:MGI:5591173]	0.451752072	-1.146396877	3.84E-08	3.23E-07	yes	down	7.408	2.61	7.91	8.88	4.48	8.51	7.26	3.76	2.4	1.64	2.57	2.68
ENSMUSG00000114055 Gm32089	predicted gene, 32089 [Source:MGI Symbol;Acc:MGI:5591248]	18.82532297	4.234602711	0.016447991	0.045991667	yes	up	0	0.18	0	0	0	0	0	0.49	0.2	0	0.11	0.1
ENSMUSG00000091275 Gm3248	predicted gene 3248 [Source:MGI Symbol;Acc:MGI:3781426]	0.240330311	-2.056909476	0.005208834	0.01701834	yes	down	0.474	0.096	0.5	0.38	0.34	0.55	0.6	0	0.03	0.17	0.25	0.03
ENSMUSG00000115536 Gm32857	predicted gene, 32857 [Source:MGI Symbol;Acc:MGI:5592016]	3.273080438	1.710649058	0.000351837	0.001525911	yes	up	0.932	2.17	0.42	0.23	1.81	1.31	0.89	3.79	1.21	2.11	1.89	1.85
ENSMUSG00000111474 Gm32926	predicted gene, 32926 [Source:MGI Symbol;Acc:MGI:5592085]	0.060355842	-4.050362772	0.006912557	0.021860502	yes	down	4.16	0.012	20.29	0.14	0.14	0.06	0.17	0.06	0	0	0	0
ENSMUSG00000115142 Gm33251	predicted gene, 33251 [Source:MGI Symbol;Acc:MGI:5592410]	7.619381494	2.929673891	0.004809612	0.015844124	yes	up	0.076	0.47	0	0.23	0.15	0	0	0.37	0.92	0.28	0.49	0.29
ENSMUSG00000113010 Gm34084	predicted gene, 34084 [Source:MGI Symbol;Acc:MGI:5593243]	2.298148214	1.200471845	0.000463834	0.001958106	yes	up	0.996	1.886	0.54	1.16	0.81	0.94	1.53	2.94	1.51	1.95	1.63	1.4
ENSMUSG00000116780 Gm3417	predicted gene 3417 [Source:MGI Symbol;Acc:MGI:3781595]	0.117942311	-3.083846724	0.012414779	0.036163615	yes	down	2.108	0.154	2.16	1.54	0	5.81	1.03	0	0	0.3	0.47	0
ENSMUSG00000111094 Gm34425	predicted gene, 34425 [Source:MGI Symbol;Acc:MGI:5593584]	9.107889222	3.187116744	3.19E-05	0.000169266	yes	up	0.08	0.534	0	0.23	0.02	0	0.15	0.62	0.56	0.5	0.5	0.49
ENSMUSG00000115855 Gm34643	predicted gene, 34643 [Source:MGI Symbol;Acc:MGI:5593802]	20.45776943	4.354576947	2.89E-05	0.000155124	yes	up	0.018	0.292	0	0.03	0.03	0.03	0	0.47	0.3	0.14	0.32	0.23
ENSMUSG00000115762 Gm34907	predicted gene, 34907 [Source:MGI Symbol;Acc:MGI:5594066]	0.461012086	-1.117123523	0.010718287	0.031845564	yes	down	1.854	0.674	2.87	0.9	0.99	1.97	2.54	1.2	0.98	0.56	0.36	0.27
ENSMUSG00000107985 Gm35037	predicted gene, 35037 [Source:MGI Symbol;Acc:MGI:5594196]	6.704160954	2.745056786	0.000242431	0.001090964	yes	up	0.194	1.03	0.07	0.23	0.48	0.19	0	1.83	0.85	1.46	0.67	0.34
ENSMUSG00000114053 Gm35330	predicted gene, 35330 [Source:MGI Symbol;Acc:MGI:5594489]	0.177189747	-2.496632971	0.000732167	0.002954604	yes	down	4.56	0.32	0.58	14.63	3.53	1.25	2.81	0.03	0.95	0.56	0.03	0.03
ENSMUSG00000105686 Gm35394	predicted gene, 35394 [Source:MGI Symbol;Acc:MGI:5594553]	0.193767069	-2.367604688	0.013744693	0.039368243	yes	down	0.188	0.032	0.22	0.16	0.02	0.29	0.25	0	0.09	0.02	0.05	0
ENSMUSG00000112831 Gm35533	predicted gene, 35533 [Source:MGI Symbol;Acc:MGI:5594692]	0.04418848	-4.500185876	0.00568696	0.018397573	yes	down	0.604	0	0.91	0.79	0	0.93	0.39	0	0	0	0	0

ENSMUSG00000116114	Gm35853	predicted gene, 35853 [Source:MGI Symbol;Acc:MGI:5595012]	0.470916856	-1.086455731	2.22E-15	3.96E-14	yes	down	13.254	4.304	15.66	13.87	10.34	10.46	15.94	5.63	4.51	3.47	4.36	3.55
ENSMUSG00000116010	Gm36026	predicted gene, 36026 [Source:MGI Symbol;Acc:MGI:5595185]	0.174237019	-2.520876917	0.013211541	0.038121579	yes	down	0.234	0.036	0.17	0.22	0.22	0.26	0.3	0.07	0.07	0	0.04	0
ENSMUSG00000067017	Gm3608	predicted gene 3608 [Source:MGI Symbol;Acc:MGI:3804932]	2.297614467	1.200136739	0.003346008	0.011459797	yes	up	0.354	0.69	0.53	0.47	0.3	0.11	0.36	0.66	0.56	0.52	0.81	0.9
ENSMUSG00000114608	Gm36161	predicted gene, 36161 [Source:MGI Symbol;Acc:MGI:5595320]	4.286482944	2.099794403	4.41E-15	7.64E-14	yes	up	2.974	10.704	1	4.08	3.37	4.3	2.12	12.16	9.29	11.24	9.92	10.91
ENSMUSG00000116582	Gm36169	predicted gene, 36169 [Source:MGI Symbol;Acc:MGI:5595328]	0.144290733	-2.792949447	0.000648953	0.002644213	yes	down	1.572	0.134	0.61	2.25	2.34	0.79	1.87	0.19	0.12	0.12	0	0.24
ENSMUSG00000112532	Gm36283	predicted gene, 36283 [Source:MGI Symbol;Acc:MGI:5595442]	0.45218608	-1.145011514	1.06E-07	8.35E-07	yes	down	4.23	2.052	7.79	2.87	3.54	5.34	1.61	3.15	0.63	1.55	2.73	2.2
ENSMUSG00000111933	Gm36660	predicted gene, 36660 [Source:MGI Symbol;Acc:MGI:5595819]	0.402046728	-1.314564907	0.001050766	0.004095214	yes	down	0.462	0.156	0.53	0.62	0.37	0.54	0.25	0.1	0.2	0.19	0.14	0.15
ENSMUSG00000113669	Gm36723	predicted gene, 36723 [Source:MGI Symbol;Acc:MGI:5595882]	2.29533738	1.198706224	0.000230977	0.001043806	yes	up	1.624	3.508	1.75	2.72	1.13	1.54	0.98	3.67	5.35	3.08	1.35	4.09
ENSMUSG00000102496	Gm36989	predicted gene, 36989 [Source:MGI Symbol;Acc:MGI:5610217]	3.49843972	1.806711633	0.003321378	0.011388208	yes	up	0.146	0.434	0.19	0.16	0	0.25	0.13	0.71	0.51	0.41	0.24	0.3
ENSMUSG00000102286	Gm37004	predicted gene, 37004 [Source:MGI Symbol;Acc:MGI:5610232]	29.61918055	4.888459822	0.009926753	0.029849951	yes	up	0	13.386	0	0	0	0	0	0	0	22.25	42.34	2.34
ENSMUSG00000103174	Gm37168	predicted gene, 37168 [Source:MGI Symbol;Acc:MGI:5610396]	5.698754585	2.510646665	0.015488647	0.043691367	yes	up	0.06	0.28	0.05	0	0.16	0	0.09	0.34	0.35	0.43	0.04	0.24
ENSMUSG00000104068	Gm37199	predicted gene, 37199 [Source:MGI Symbol;Acc:MGI:5610427]	38.52011018	5.267539925	3.39E-05	0.000178611	yes	up	0.004	0.132	0	0.02	0	0	0	0.27	0.21	0.1	0.01	0.07
ENSMUSG00000103620	Gm37359	predicted gene, 37359 [Source:MGI Symbol;Acc:MGI:5610587]	0.165371003	-2.596221804	0.005286833	0.017243803	yes	down	1.386	0.196	0.99	2.48	0.84	0.68	1.94	0.34	0	0.5	0.14	0
ENSMUSG00000102155	Gm37468	predicted gene, 37468 [Source:MGI Symbol;Acc:MGI:5610696]	5.13078819	2.359180469	2.30E-05	0.00012574	yes	up	8.086	36.496	1.73	3.39	12.52	8.57	14.22	29.98	44.43	52.26	36.03	19.78
ENSMUSG00000104376	Gm37516	predicted gene, 37516 [Source:MGI Symbol;Acc:MGI:5610744]	0.358034671	-1.481828794	2.22E-11	2.69E-10	yes	down	5.902	1.852	5.39	5.28	6.65	6.73	5.46	1.93	1.9	1.29	2.76	1.38
ENSMUSG00000103199	Gm37648	predicted gene, 37648 [Source:MGI Symbol;Acc:MGI:5610876]	16.17125801	4.01536001	0.013012018	0.037621896	yes	up	0	0.06	0	0	0	0	0	0.06	0.04	0.06	0.02	0.12
ENSMUSG00000103473	Gm37696	predicted gene, 37696 [Source:MGI Symbol;Acc:MGI:5610924]	4.314811814	2.109297639	0.007603375	0.023726342	yes	up	0.05	0.174	0.06	0	0.14	0.05	0	0.16	0.22	0.12	0.22	0.15
ENSMUSG00000103219	Gm37787	predicted gene, 37787 [Source:MGI Symbol;Acc:MGI:5611015]	61.85477494	5.950813065	3.01E-07	2.24E-06	yes	up	0	1.838	0	0	0	0	0	2.88	1.62	2.22	0.87	1.6
ENSMUSG00000102813	Gm37795	predicted gene, 37795 [Source:MGI Symbol;Acc:MGI:5611023]	3.610029935	1.8520108	2.55E-06	1.65E-05	yes	up	0.37	1.132	0.38	0.28	0.28	0.52	0.39	1.54	1.66	0.9	0.45	1.11
ENSMUSG00000103928	Gm37893	predicted gene, 37893 [Source:MGI Symbol;Acc:MGI:5611121]	2.067842563	1.048126349	0.004647766	0.01537719	yes	up	0.732	1.274	0.52	0.79	0.59	1.15	0.61	1.69	2.21	0.97	0.73	0.77
ENSMUSG00000102962	Gm37944	predicted gene, 37944 [Source:MGI Symbol;Acc:MGI:5611172]	21.85086188	4.449618281	8.88E-10	9.07E-09	yes	up	11.356	209.774	8.41	22.97	20.43	0.49	4.48	280.53	197.88	133.93	134.88	301.65
ENSMUSG00000104164	Gm38248	predicted gene, 38248 [Source:MGI Symbol;Acc:MGI:5611476]	11.01755082	3.461731646	2.23E-08	1.94E-07	yes	up	0.046	0.428	0.01	0	0.11	0.07	0.04	0.71	0.61	0.31	0.13	0.38
ENSMUSG00000104000	Gm38335	predicted gene, 38335 [Source:MGI Symbol;Acc:MGI:5611563]	17.24312798	4.107949604	0.008893687	0.027151509	yes	up	0	0.1	0	0	0	0	0	0.1	0.12	0.15	0.1	0.03
ENSMUSG00000108010	Gm38708	predicted gene, 38708 [Source:MGI Symbol;Acc:MGI:5621593]	0.460684642	-1.118148592	0.001876131	0.006874117	yes	down	2.602	1.302	2.93	1.27	3.04	3.7	2.07	2.61	1.44	0.72	0.5	1.24
ENSMUSG00000108371	Gm38832	predicted gene, 38832 [Source:MGI Symbol;Acc:MGI:5621717]	22.24837362	4.475627972	0.007266561	0.022808246	yes	up	0	1.67	0	0	0	0	0	3.49	0	1.92	2.14	0.8
ENSMUSG00000111229	Gm39323	predicted gene, 39323 [Source:MGI Symbol;Acc:MGI:5622208]	2.968285044	1.569629641	0.008108234	0.025107857	yes	up	0.11	0.266	0.07	0.09	0.23	0.12	0.04	0.33	0.21	0.27	0.15	0.37
ENSMUSG00000111533	Gm39458	predicted gene, 39458 [Source:MGI Symbol;Acc:MGI:5622343]	15.01416904	3.908252726	0.001513983	0.005674391	yes	up	0.044	0.502	0	0.14	0	0	0.08	0.46	0.42	0.65	0.26	0.72
ENSMUSG00000113553	Gm40932	predicted gene, 40932 [Source:MGI Symbol;Acc:MGI:5623817]	51.50099753	5.686528471	9.05E-06	5.30E-05	yes	up	0	2.098	0	0	0	0	0	3.76	1.27	0.42	1.4	3.64
ENSMUSG00000115835	Gm41253	predicted gene, 41253 [Source:MGI Symbol;Acc:MGI:5624138]	0.491756707	-1.023983365	0.000106231	0.000512167	yes	down	1.808	0.706	2.41	1.58	2.27	1.46	1.32	0.51	0.57	0.66	0.93	0.86
ENSMUSG00000116946	Gm41442	predicted gene, 41442 [Source:MGI Symbol;Acc:MGI:5624327]	0.460290489	-1.119383463	5.97E-09	5.58E-08	yes	down	2.396	0.928	2.81	2.44	2.24	2.52	1.97	1.19	1.01	0.95	0.91	0.58

ENSMUSG00000108348	Gm42372	predicted gene, 42372 [Source:MGI Symbol;Acc:MGI:5625257]	2.887505045	1.529823467	0.000184513	0.000847606	yes	up	0.122	0.298	0.07	0.22	0.09	0.14	0.09	0.31	0.25	0.34	0.23	0.36
ENSMUSG00000098178	Gm42418	predicted gene, 42418 [Source:MGI Symbol;Acc:MGI:5649005]	2.376980355	1.24912998	0.000144426	0.000678893	yes	up	45.06	90.978	22.65	49.6	47.46	50.82	54.77	98.5	139.4	76.88	45.19	94.92
ENSMUSG00000097056	Gm4262	predicted gene 4262 [Source:MGI Symbol;Acc:MGI:3782439]	0.416863281	-1.262353797	0.00018254	0.000839259	yes	down	1.944	0.692	1.81	1.43	1.49	2.16	2.83	0.93	0.54	0.86	0.38	0.75
ENSMUSG00000106892	Gm42791	predicted gene 42791 [Source:MGI Symbol;Acc:MGI:5662928]	0.294785055	-1.762264711	0.00234298	0.008379635	yes	down	6.762	2.212	5.34	6.42	1.61	8.6	11.84	2.03	3.8	0.12	3.4	1.71
ENSMUSG00000104822	Gm42967	predicted gene 42967 [Source:MGI Symbol;Acc:MGI:5663104]	6.104845064	2.609954681	0.01533817	0.043329673	yes	up	0.044	0.224	0.09	0.03	0	0.1	0	0.49	0.45	0.07	0.02	0.09
ENSMUSG00000107029	Gm43123	predicted gene 43123 [Source:MGI Symbol;Acc:MGI:5663260]	0.428691518	-1.221988221	0.005773739	0.018636079	yes	down	13.21	4.82	15.46	10.78	10.53	8.12	21.16	7.75	5.69	3.51	5.56	1.59
ENSMUSG00000104927	Gm43388	predicted gene 43388 [Source:MGI Symbol;Acc:MGI:5663525]	6.493374553	2.69896843	4.88E-06	3.00E-05	yes	up	0.208	1.122	0.25	0.26	0.26	0.22	0.05	1.37	1.08	1.6	0.42	1.14
ENSMUSG00000104761	Gm43511	predicted gene 43511 [Source:MGI Symbol;Acc:MGI:5663648]	7.497440641	2.906398195	0.001087964	0.004225578	yes	up	0.038	0.242	0	0.06	0.03	0.03	0.07	0.46	0.18	0.32	0.07	0.18
ENSMUSG00000105607	Gm43513	predicted gene 43513 [Source:MGI Symbol;Acc:MGI:5663650]	8.846766383	3.145150226	0.016244269	0.045511155	yes	up	0.038	0.314	0	0	0	0	0.19	0.38	0.47	0.12	0.25	0.35
ENSMUSG00000105403	Gm43618	predicted gene 43618 [Source:MGI Symbol;Acc:MGI:5663755]	0.389180468	-1.361488789	0.001864795	0.006837668	yes	down	0.574	0.19	0.44	0.54	0.69	0.85	0.35	0.23	0.32	0.18	0.16	0.06
ENSMUSG00000108126	Gm43909	predicted gene, 43909 [Source:MGI Symbol;Acc:MGI:5690301]	3.944641311	1.979894121	0.004781454	0.015761847	yes	up	0.176	0.58	0.33	0.04	0.42	0	0.09	0.93	0.41	0.41	0.54	0.61
ENSMUSG00000107811	Gm44000	predicted gene, 44000 [Source:MGI Symbol;Acc:MGI:5690392]	0.413760155	-1.273133374	0.006802763	0.021554662	yes	down	2.352	0.818	1.68	3.47	1.88	2.41	2.32	0.5	0.54	2.21	0.41	0.43
ENSMUSG00000108216	Gm44153	predicted gene, 44153 [Source:MGI Symbol;Acc:MGI:5690545]	0.375155546	-1.414439209	0.004552869	0.015102886	yes	down	0.954	0.304	1.36	0.8	0.61	1.18	0.82	0.27	0.53	0.39	0.08	0.25
ENSMUSG00000107428	Gm44154	predicted gene, 44154 [Source:MGI Symbol;Acc:MGI:5690546]	6.580739606	2.718249737	0.005729926	0.018516992	yes	up	0.028	0.158	0.05	0.07	0.02	0	0	0.24	0.28	0.07	0.12	0.08
ENSMUSG00000108132	Gm44175	predicted gene, 44175 [Source:MGI Symbol;Acc:MGI:5690567]	3.276500345	1.712155684	0.009868179	0.029682152	yes	up	0.084	0.228	0.03	0.14	0.03	0.18	0.04	0.39	0.36	0.1	0.2	0.09
ENSMUSG00000108184	Gm44198	predicted gene, 44198 [Source:MGI Symbol;Acc:MGI:5690590]	19.7645356	4.304842152	0.004836658	0.015911195	yes	up	0	0.074	0	0	0	0	0	0.13	0.06	0.02	0.08	0.08
ENSMUSG00000107429	Gm44206	predicted gene, 44206 [Source:MGI Symbol;Acc:MGI:5690598]	11.12917056	3.47627417	0.000764014	0.003066366	yes	up	0.544	3.456	2.08	0	0	0	0.64	3.46	4.09	1.1	4.99	3.64
ENSMUSG00000107451	Gm44421	predicted gene, 44421 [Source:MGI Symbol;Acc:MGI:5690813]	0.302765501	-1.723727272	0.001950094	0.007118324	yes	down	1.682	0.432	1.45	2.41	1.55	1.76	1.24	0.9	0.59	0	0.37	0.3
ENSMUSG00000108064	Gm44423	predicted gene, 44423 [Source:MGI Symbol;Acc:MGI:5690815]	3.537793569	1.822849869	8.54E-05	0.000420425	yes	up	0.202	0.612	0.16	0.06	0.15	0.36	0.28	0.63	0.99	0.55	0.29	0.6
ENSMUSG00000107872	Gm44511	predicted gene 44511 [Source:MGI Symbol;Acc:MGI:5753087]	2.876976542	1.524553458	8.33E-06	4.93E-05	yes	up	5.132	12.612	3.79	2.33	6.22	6.28	7.04	10.23	10.07	17.57	14.11	11.08
ENSMUSG00000109371	Gm44626	predicted gene 44626 [Source:MGI Symbol;Acc:MGI:5753202]	0.076939094	-3.700139343	0.017815737	0.049218502	yes	down	0.91	0.056	0	2.79	0.37	0.93	0.46	0	0.28	0	0	0
ENSMUSG00000108473	Gm44739	predicted gene 44739 [Source:MGI Symbol;Acc:MGI:5753315]	7.007126809	2.808823005	0.006688583	0.021249471	yes	up	0.098	0.59	0	0.05	0.17	0	0.27	0.51	1.79	0.14	0.06	0.45
ENSMUSG00000109350	Gm44805	predicted gene 44805 [Source:MGI Symbol;Acc:MGI:5753381]	11.79807518	3.560479603	7.15E-10	7.36E-09	yes	up	0.164	1.604	0.07	0.32	0.21	0.22	0	1.4	1.21	1.4	2.17	1.84
ENSMUSG00000108897	Gm44861	predicted gene 44861 [Source:MGI Symbol;Acc:MGI:5753437]	6.435568072	2.686067502	1.17E-05	6.73E-05	yes	up	0.21	1.136	0.25	0.2	0.39	0.16	0.05	0.85	1.97	0.88	1.48	0.5
ENSMUSG00000109008	Gm44899	predicted gene 44899 [Source:MGI Symbol;Acc:MGI:5753475]	0.400015052	-1.321873807	0.000106422	0.000512972	yes	down	7.452	0.774	28.14	1.96	2.64	2.7	1.82	0.8	0.96	0.65	0.89	0.57
ENSMUSG00000108536	Gm45059	predicted gene 45059 [Source:MGI Symbol;Acc:MGI:5753635]	7.948732047	2.990724745	0.011976245	0.035043485	yes	up	0.054	0.37	0	0.08	0	0.19	0	0.5	0.41	0.29	0.06	0.59
ENSMUSG00000108308	Gm45218	predicted gene 45218 [Source:MGI Symbol;Acc:MGI:5753794]	18.14998941	4.181896801	0.006780134	0.021489321	yes	up	0	0.116	0	0	0	0	0	0.04	0.13	0.13	0.11	0.17
ENSMUSG00000110317	Gm45250	predicted gene 45250 [Source:MGI Symbol;Acc:MGI:5791086]	2.104246839	1.07330395	0.009337089	0.028315364	yes	up	0.33	0.58	0.39	0.2	0.55	0.36	0.15	0.9	0.76	0.36	0.49	0.39
ENSMUSG00000110020	Gm45441	predicted gene 45441 [Source:MGI Symbol;Acc:MGI:5791277]	3.543532736	1.825188377	0.000308698	0.001356674	yes	up	0.648	1.736	0.32	0.3	0.36	1.36	0.9	1.39	2.54	1.65	2.05	1.05
ENSMUSG00000109780	Gm45447	predicted gene 45447 [Source:MGI Symbol;Acc:MGI:5791283]	0.227459965	-2.136315457	0.007052242	0.022228259	yes	down	0.392	0.076	0.58	0.36	0.12	0.4	0.5	0.16	0.03	0	0.1	0.09

ENSMUSG00000109674	Gm45470	predicted gene 45470 [Source:MGI Symbol;Acc:MGI:5791306]	0.326716425	-1.613889111	0.001510845	0.005664611	yes	down	0.75	0.238	0.78	0.43	0.4	1.49	0.65	0.16	0.22	0.58	0.12	0.11
ENSMUSG00000109715	Gm45606	predicted gene 45606 [Source:MGI Symbol;Acc:MGI:5791442]	2.373008155	1.246717058	5.21E-07	3.73E-06	yes	up	1.152	2.29	1.11	0.74	0.98	1.68	1.25	2.85	2.14	1.69	1.95	2.82
ENSMUSG00000110141	Gm45684	predicted gene 45684 [Source:MGI Symbol;Acc:MGI:5791520]	3.940741089	1.978466966	0.012091109	0.035307226	yes	up	0.126	0.422	0.09	0.05	0.09	0.28	0.12	0.84	0.33	0.25	0.08	0.61
ENSMUSG00000110481	Gm45705	predicted gene 45705 [Source:MGI Symbol;Acc:MGI:5804820]	8.270292671	3.047938385	0.00112206	0.004343823	yes	up	0.046	0.336	0	0.07	0	0	0.16	0.08	0.35	0.37	0.48	0.4
ENSMUSG00000110588	Gm45774	predicted gene 45774 [Source:MGI Symbol;Acc:MGI:5804889]	0.15417599	-2.697349984	8.18E-05	0.000403702	yes	down	0.512	0.064	0.42	1.1	0.38	0.43	0.23	0.04	0.13	0.11	0.04	0
ENSMUSG00000110626	Gm45805	predicted gene 45805 [Source:MGI Symbol;Acc:MGI:5804920]	0.337892098	-1.565365481	0.004833903	0.015904574	yes	down	0.562	0.162	0.5	0.44	0.48	0.4	0.99	0.06	0.23	0.23	0.08	0.21
ENSMUSG00000110641	Gm45826	predicted gene 45826 [Source:MGI Symbol;Acc:MGI:5804941]	0.073002151	-3.775917219	0.000521436	0.0021747	yes	down	2.204	0.146	2.59	0.36	1.07	2.91	4.09	0.55	0	0	0.18	0
ENSMUSG00000108825	Gm45838	predicted gene 45838 [Source:MGI Symbol;Acc:MGI:5804953]	2.965646969	1.568346869	0.00013938	0.000657045	yes	up	0.374	0.936	0.33	0.22	0.48	0.54	0.3	1.43	1.23	0.84	0.39	0.79
ENSMUSG00000109243	Gm45867	predicted gene 45867 [Source:MGI Symbol;Acc:MGI:5804982]	2.576971951	1.365676834	0.003917995	0.013201349	yes	up	0.29	0.636	0.2	0.27	0.15	0.53	0.3	0.53	1.19	0.38	0.46	0.62
ENSMUSG00000112843	Gm46224	predicted gene, 46224 [Source:MGI Symbol;Acc:MGI:5825861]	8.32814917	3.05799591	2.79E-05	0.000150222	yes	up	0.144	1.016	0.06	0.25	0.25	0	0.16	1.65	1.59	0.28	0.78	0.78
ENSMUSG00000112844	Gm47036	predicted gene, 47036 [Source:MGI Symbol;Acc:MGI:6095735]	0.331539617	-1.59274682	0.004309361	0.014359618	yes	down	0.47	0.132	0.32	0.72	0.69	0.37	0.25	0.17	0.18	0.12	0	0.19
ENSMUSG00000114493	Gm47071	predicted gene, 47071 [Source:MGI Symbol;Acc:MGI:6095789]	2.840893236	1.506344615	0.003222744	0.011103326	yes	up	0.276	0.674	0.11	0.39	0.17	0.5	0.21	0.7	1.3	0.51	0.29	0.57
ENSMUSG00000111340	Gm47171	predicted gene, 47171 [Source:MGI Symbol;Acc:MGI:6095951]	4.276081367	2.096289306	0.001094889	0.004248622	yes	up	0.15	0.54	0.07	0.28	0.15	0.21	0.04	0.77	1.01	0.49	0.08	0.35
ENSMUSG00000114761	Gm47242	predicted gene, 47242 [Source:MGI Symbol;Acc:MGI:6096064]	3.26078865	1.705220935	1.37E-09	1.37E-08	yes	up	0.986	1.706	0.36	1.95	0.57	0.98	1.07	1.9	1.26	1.31	1.44	2.62
ENSMUSG00000111362	Gm47389	predicted gene, 47389 [Source:MGI Symbol;Acc:MGI:6096310]	14.27518557	3.835437595	2.13E-05	0.000116916	yes	up	0.086	1.072	0.19	0	0	0.08	0.16	0.55	1.39	1.95	0.71	0.76
ENSMUSG00000113757	Gm47507	predicted gene, 47507 [Source:MGI Symbol;Acc:MGI:6096493]	4.704290927	2.233977283	7.50E-07	5.24E-06	yes	up	0.602	2.39	0.36	1.05	0.3	0.19	1.11	1.89	2.66	2.4	2.17	2.83
ENSMUSG00000114033	Gm47705	predicted gene, 47705 [Source:MGI Symbol;Acc:MGI:6096822]	2.860151459	1.516091547	5.63E-06	3.43E-05	yes	up	0.772	1.884	0.89	0.92	0.69	0.79	0.57	1.77	1.34	1.72	2.71	1.88
ENSMUSG00000111970	Gm47715	predicted gene, 47715 [Source:MGI Symbol;Acc:MGI:6096840]	0.39083343	-1.35537422	0.010565386	0.031458432	yes	down	3.408	1.136	1.96	3.45	5.74	2.5	3.39	2.18	1.66	0.35	0.89	0.6
ENSMUSG00000114573	Gm47732	predicted gene, 47732 [Source:MGI Symbol;Acc:MGI:6096868]	0.179674095	-2.476545676	0.000459177	0.001939977	yes	down	2.268	0.344	1.42	2.22	1.64	4.02	2.04	0.45	0.56	0.41	0.3	0
ENSMUSG00000112597	Gm4777	predicted gene 4777 [Source:MGI Symbol;Acc:MGI:3644085]	0.328065424	-1.607944545	0.000160073	0.000744596	yes	down	4.99	1.388	6.51	5.53	4.64	4.86	3.41	0.89	0.31	0.96	1.38	3.4
ENSMUSG00000111171	Gm47815	predicted gene, 47815 [Source:MGI Symbol;Acc:MGI:6097001]	2.339189008	1.226008437	0.000391388	0.001677064	yes	up	1.204	2.794	1.46	1	1.89	0.55	1.12	2.27	3.25	1.49	2.6	4.36
ENSMUSG00000113811	Gm47882	predicted gene, 47882 [Source:MGI Symbol;Acc:MGI:6097109]	11.15071594	3.479064437	0.017521723	0.048556575	yes	up	2.056	21.284	0	2.96	0	7.29	0.03	36.59	14.72	32.62	22.49	0
ENSMUSG00000111116	Gm48065	predicted gene, 48065 [Source:MGI Symbol;Acc:MGI:6097392]	3.981978369	1.993485384	0.014936558	0.042301153	yes	up	0.032	0.104	0	0.02	0.05	0.08	0.01	0.11	0.15	0.07	0.09	0.1
ENSMUSG00000111551	Gm48128	predicted gene, 48128 [Source:MGI Symbol;Acc:MGI:6097487]	3.398536538	1.764913634	0.002482262	0.008833521	yes	up	0.254	0.738	0.15	0.03	0.32	0.19	0.58	1.18	0.85	0.66	0.13	0.87
ENSMUSG00000112770	Gm48210	predicted gene, 48210 [Source:MGI Symbol;Acc:MGI:6097604]	14.50981381	3.858957102	0.007476882	0.02339639	yes	up	0.012	0.194	0.06	0	0	0	0	0.2	0.05	0.33	0.1	0.29
ENSMUSG00000114036	Gm48228	predicted gene, 48228 [Source:MGI Symbol;Acc:MGI:6097631]	0.448955662	-1.155355121	1.20E-05	6.87E-05	yes	down	4.162	1.724	4.97	4.76	4.49	3.61	2.98	2.2	1.26	2.57	1.31	1.28
ENSMUSG00000068606	Gm4841	predicted gene 4841 [Source:MGI Symbol;Acc:MGI:3643814]	10.68943908	3.418114245	4.09E-17	8.53E-16	yes	up	0.168	1.51	0.28	0.21	0.09	0.08	0.18	1.79	0.95	1.19	1.25	2.37
ENSMUSG00000114566	Gm48500	predicted gene, 48500 [Source:MGI Symbol;Acc:MGI:6098026]	0.215133044	-2.21669896	0.007570267	0.023640252	yes	down	0.546	0.1	1.01	0.38	0.1	0.73	0.51	0.19	0.19	0.08	0.04	0
ENSMUSG00000114004	Gm48552	predicted gene, 48552 [Source:MGI Symbol;Acc:MGI:6098105]	0.314084723	-1.670774322	0.007627829	0.02378532	yes	down	2.604	0.846	1.21	3.53	3.72	0.48	4.08	0.47	0.78	0.96	0.41	1.61
ENSMUSG00000111345	Gm48562	predicted gene, 48562 [Source:MGI Symbol;Acc:MGI:6098119]	13.821862	3.788880075	0.004974001	0.016317893	yes	up	0.07	0.844	0	0	0.13	0.22	0	0.97	2.2	0.3	0.75	0

ENSMUSG00000111291 Gm48604	predicted gene, 48604 [Source:MGI Symbol;Acc:MGI:6098186]	2.37374951	1.247167702	0.017357189	0.048167633	yes	up	0.314	0.64	0.31	0.02	0.48	0.44	0.32	0.43	1.1	0.37	0.86	0.44
ENSMUSG00000111292 Gm48627	predicted gene, 48627 [Source:MGI Symbol;Acc:MGI:6098227]	19.55821098	4.289702505	0.011883317	0.0347985	yes	up	0	0.16	0	0	0	0	0	0.28	0.09	0.17	0	0.26
ENSMUSG00000105163 Gm4873	predicted gene 4873 [Source:MGI Symbol;Acc:MGI:3648522]	32.80574444	5.035876555	0.000176952	0.000815669	yes	up	0.594	17.034	1.53	0	0	0	1.44	4.28	10.46	25.98	0	44.45
ENSMUSG00000111929 Gm48780	predicted gene, 48780 [Source:MGI Symbol;Acc:MGI:6098477]	0.114169378	-3.13075235	0.017369643	0.048178769	yes	down	0.424	0.044	0.44	0.94	0	0.36	0.38	0.09	0	0.06	0	0.07
ENSMUSG00000112794 Gm48878	predicted gene, 48878 [Source:MGI Symbol;Acc:MGI:6098630]	0.249018355	-2.005676008	0.002744698	0.009634559	yes	down	0.36	0.074	0.59	0.35	0.56	0.15	0.15	0.06	0.04	0.11	0.12	0.04
ENSMUSG00000112441 Gm48898	predicted gene, 48898 [Source:MGI Symbol;Acc:MGI:6098663]	4.004599867	2.001658098	2.82E-05	0.000151468	yes	up	0.154	0.512	0.12	0.23	0.2	0.09	0.13	0.58	0.27	0.51	0.7	0.5
ENSMUSG00000115729 Gm48957	predicted gene, 48957 [Source:MGI Symbol;Acc:MGI:6118287]	0.032691826	-4.93492622	0.000545631	0.002263703	yes	down	9.922	0	48.97	0.38	0.16	0.08	0.02	0	0	0	0	0
ENSMUSG00000115148 Gm49125	predicted gene, 49125 [Source:MGI Symbol;Acc:MGI:6118530]	4.627455466	2.210219106	1.62E-05	9.06E-05	yes	up	0.518	2.016	0.23	0.6	0.9	0.71	0.15	2.35	3.19	0.94	0.93	2.67
ENSMUSG00000115739 Gm49254	predicted gene, 49254 [Source:MGI Symbol;Acc:MGI:6118725]	23.63864325	4.563075329	0.00218587	0.007867664	yes	up	0	0.12	0	0	0	0	0	0.03	0.05	0.21	0.15	0.16
ENSMUSG00000062593 Gm49339	predicted gene, 49339 [Source:MGI Symbol;Acc:MGI:6121530]	8.559944448	3.097601434	3.30E-96	1.05E-93	yes	up	14.052	104.536	10.46	19.67	14.75	9.98	15.4	101.38	115.04	84.32	109	112.94
ENSMUSG00000114470 Gm49395	predicted gene, 49395 [Source:MGI Symbol;Acc:MGI:6121627]	0.466362649	-1.100475847	0.001273899	0.004861337	yes	down	6.334	2.656	4.08	5.12	6.56	8.12	7.79	3.87	3.51	1.82	1.07	3.01
ENSMUSG00000115157 Gm49397	predicted gene, 49397 [Source:MGI Symbol;Acc:MGI:6121631]	0.448694657	-1.156194092	0.014514	0.041273647	yes	down	7.608	2.932	6.73	6.76	11.03	5.91	7.61	0.86	3.69	2.85	3.35	3.91
ENSMUSG00000115186 Gm49417	predicted gene, 49417 [Source:MGI Symbol;Acc:MGI:6155048]	0.442792331	-1.17529786	1.11E-11	1.39E-10	yes	down	10.79	3.8	13.77	8.47	12.3	8.96	10.45	4.41	4.3	2.87	3.06	4.36
ENSMUSG00000116226 Gm49502	predicted gene, 49502 [Source:MGI Symbol;Acc:MGI:6155188]	3.293372745	1.719565806	0.015405306	0.043479182	yes	up	0.092	0.254	0.1	0.1	0.07	0.11	0.08	0.46	0.28	0.1	0.13	0.3
ENSMUSG00000116528 Gm49508	predicted gene, 49508 [Source:MGI Symbol;Acc:MGI:6155198]	78.90777451	6.302095546	6.18E-07	4.38E-06	yes	up	0	8.324	0	0	0	0	0	13.93	4.42	0.53	16.03	6.71
ENSMUSG00000025936 Gm4956	predicted gene 4956 [Source:MGI Symbol;Acc:MGI:3647976]	0.334351005	-1.58056464	5.09E-20	1.29E-18	yes	down	117.398	33.118	129.06	87.66	119.19	126.35	124.73	38.1	34.68	26.94	43.77	22.1
ENSMUSG00000116594 Gm49601	predicted gene, 49601 [Source:MGI Symbol;Acc:MGI:6215011]	0.302996241	-1.722628198	0.013879524	0.039680134	yes	down	0.696	0.518	0.36	0.86	0.49	0.91	0.86	0.38	1.41	0.45	0.13	0.22
ENSMUSG00000116579 Gm49701	predicted gene, 49701 [Source:MGI Symbol;Acc:MGI:6215160]	0.246075945	-2.02282446	0.002354891	0.008418019	yes	down	1.83	0.41	1.02	1.16	2.25	2.18	2.54	0.73	0.58	0.47	0.15	0.12
ENSMUSG00000116829 Gm49704	predicted gene, 49704 [Source:MGI Symbol;Acc:MGI:6215166]	0.119869963	-3.060457898	0.001297624	0.004941306	yes	down	1.526	0.158	2.09	0.84	0.66	1.12	2.92	0.41	0.13	0.25	0	0
ENSMUSG00000116617 Gm49767	predicted gene, 49767 [Source:MGI Symbol;Acc:MGI:6215277]	0.420906063	-1.248429805	5.43E-09	5.10E-08	yes	down	2.516	0.926	2.63	3.07	2.01	2.61	2.26	0.94	0.76	1.38	0.59	0.96
ENSMUSG00000116657 Gm49774	predicted gene, 49774 [Source:MGI Symbol;Acc:MGI:6215288]	9.522628381	3.251359833	0.011440964	0.033687518	yes	up	0.004	0.026	0.01	0	0	0	0.01	0.06	0.03	0.02	0.01	0.01
ENSMUSG00000116896 Gm49792	predicted gene, 49792 [Source:MGI Symbol;Acc:MGI:6215320]	11.46544973	3.519221041	3.34E-07	2.45E-06	yes	up	3.804	39.15	0	0	5.57	6.55	6.9	65.63	24.33	19.28	43.82	42.69
ENSMUSG00000116827 Gm5063	predicted gene 5063 [Source:MGI Symbol;Acc:MGI:3648069]	12.72097001	3.669136779	6.01E-07	4.25E-06	yes	up	0.118	1.244	0.17	0.09	0.26	0	0.07	1.37	1.13	0.86	1.65	1.21
ENSMUSG00000110235 Gm5086	predicted gene 5086 [Source:MGI Symbol;Acc:MGI:3644417]	3.391392498	1.761877762	8.17E-06	4.84E-05	yes	up	0.736	2.132	0.7	0.36	0.76	1.29	0.57	1.73	1.55	3.28	1.96	2.14
ENSMUSG00000115240 Gm5144	predicted gene 5144 [Source:MGI Symbol;Acc:MGI:3779468]	0.211890431	-2.238609656	5.89E-06	3.58E-05	yes	down	5.198	0.94	7.21	6.27	4.72	5.41	2.38	1.1	1.09	0.54	1.87	0.1
ENSMUSG00000078780 Gm5150	predicted gene 5150 [Source:MGI Symbol;Acc:MGI:3779469]	2.484560219	1.312990509	5.39E-09	5.07E-08	yes	up	4.48	9.494	4.42	5.92	3.02	4.9	4.14	11.09	8.02	10.43	10.96	6.97
ENSMUSG00000098050 Gm5345	predicted gene 5345 [Source:MGI Symbol;Acc:MGI:3644320]	3.370692799	1.753045148	0.001501614	0.005634934	yes	up	0.632	1.832	1.42	0.47	0	0.72	0.55	1.44	2.19	2.24	2.17	1.12
ENSMUSG00000097245 Gm5421	predicted gene 5421 [Source:MGI Symbol;Acc:MGI:3645718]	21.70550522	4.439989099	0.000444312	0.001882371	yes	up	0.012	0.202	0.06	0	0	0	0	0.19	0.11	0.44	0.05	0.22
ENSMUSG00000058163 Gm5431	predicted gene 5431 [Source:MGI Symbol;Acc:MGI:3645205]	3.627299623	1.85889592	8.43E-27	3.26E-25	yes	up	1.944	5.956	1.78	1.86	2.21	1.43	2.44	5.96	5.58	6.11	4.98	7.15
ENSMUSG00000104835 Gm5547	predicted gene 5547 [Source:MGI Symbol;Acc:MGI:3648206]	3.361628251	1.749160192	5.33E-09	5.02E-08	yes	up	3.608	7.576	1.31	2.48	7.4	4.34	2.51	7.74	7.07	6.85	5.55	10.67

ENSMUSG00000113750	Gm5628	predicted gene 5628 [Source:MGI Symbol;Acc:MGI:3779506]	0.207918609	-2.265909204	0.002746483	0.009639247	yes	down	0.99	0.172	1.08	0.89	0.48	1.5	1	0.32	0.42	0.06	0	0.06
ENSMUSG00000097260	Gm5706	predicted gene 5706 [Source:MGI Symbol;Acc:MGI:3643223]	0.196070784	-2.350553518	6.38E-30	2.95E-28	yes	down	1.716	0.352	1.89	1.44	1.99	1.55	1.71	0.18	0.3	0.71	0.36	0.21
ENSMUSG00000114858	Gm5790	predicted gene 5790 [Source:MGI Symbol;Acc:MGI:3779518]	0.315433206	-1.664593554	0.005463685	0.017747652	yes	down	1.384	0.374	1.02	0.96	1.54	1.22	2.18	0.15	0.12	0.89	0.08	0.63
ENSMUSG00000102715	Gm6209	predicted gene 6209 [Source:MGI Symbol;Acc:MGI:3643374]	77.7111972	6.280050583	1.34E-15	2.42E-14	yes	up	0.058	6.272	0	0.08	0	0.21	0	6.14	5.36	6.54	5.11	8.21
ENSMUSG00000102795	Gm6236	predicted gene 6236 [Source:MGI Symbol;Acc:MGI:3779577]	2.342540146	1.228073773	0.006969957	0.022003036	yes	up	0.214	0.42	0.17	0.26	0.23	0.13	0.28	0.39	0.5	0.34	0.62	0.25
ENSMUSG00000089716	Gm6264	predicted gene 6264 [Source:MGI Symbol;Acc:MGI:3779580]	5.834777284	2.544677589	1.12E-28	4.89E-27	yes	up	3.616	12.524	4.32	5.04	3.57	3.5	1.65	17.24	9.66	10.75	11.37	13.6
ENSMUSG00000061833	Gm6311	predicted gene 6311 [Source:MGI Symbol;Acc:MGI:3644735]	9.361781709	3.226783126	0.002876838	0.010050629	yes	up	0.098	0.77	0	0.49	0	0	0	0.57	0.36	0.99	0.77	1.16
ENSMUSG00000100141	Gm6445	predicted gene 6445 [Source:MGI Symbol;Acc:MGI:3779598]	9.612800032	3.264956723	0.016242643	0.045511155	yes	up	0.036	0.31	0	0	0	0.12	0.06	0	0.39	0.5	0.46	0.2
ENSMUSG00000097252	Gm6634	predicted gene 6634 [Source:MGI Symbol;Acc:MGI:3643829]	0.258283267	-1.952973914	8.16E-06	4.83E-05	yes	down	7.09	1.542	3.81	17.81	3.89	4.86	5.08	2.44	1.12	1.74	1.73	0.68
ENSMUSG00000090881	Gm6904	predicted gene 6904 [Source:MGI Symbol;Acc:MGI:3644962]	10.11649577	3.33863774	0.011762835	0.034503887	yes	up	0.058	0.426	0.1	0.19	0	0	0	0.24	0.3	0.87	0.16	0.56
ENSMUSG00000035983	Gm7008	predicted gene 7008 [Source:MGI Symbol;Acc:MGI:3647211]	0.356571438	-1.487736948	0.00052427	0.002185461	yes	down	1.57	0.466	1.57	2.05	1.73	1.36	1.14	0.14	0.35	0.55	0.71	0.58
ENSMUSG00000073077	Gm7173	predicted gene 7173 [Source:MGI Symbol;Acc:MGI:3645063]	0.499814891	-1.000534209	0.000104244	0.000503267	yes	down	0.868	3.308	0.88	0.8	0.72	1.04	0.9	14.19	0.7	0.31	0.66	0.68
ENSMUSG00000083573	Gm7312	predicted gene 7312 [Source:MGI Symbol;Acc:MGI:3646347]	0.078557622	-3.670104929	0.011878868	0.034791827	yes	down	1.628	0.106	0.96	1.36	1.32	4.5	0	0	0	0	0.27	0.26
ENSMUSG00000090957	Gm7535	predicted gene 7535 [Source:MGI Symbol;Acc:MGI:3644434]	20.61794838	4.365828878	0.00418956	0.014001813	yes	up	0.006	0.182	0	0	0	0	0.03	0	0.34	0.03	0.23	0.31
ENSMUSG00000079457	Gm7609	predicted pseudogene 7609 [Source:MGI Symbol;Acc:MGI:3644536]	3.080845198	1.623326194	0.001330083	0.005048752	yes	up	0.836	2.052	0.73	0.07	1.49	1.09	0.8	3.14	1.64	1.34	1.64	2.5
ENSMUSG00000091568	Gm8206	predicted gene 8206 [Source:MGI Symbol;Acc:MGI:3779788]	0.371325325	-1.429244383	0.015835186	0.044551544	yes	down	0.914	0.278	0.57	2.54	0.64	0.47	0.35	0.16	0.03	0.31	0.09	0.8
ENSMUSG00000090379	Gm8229	predicted gene 8229 [Source:MGI Symbol;Acc:MGI:3646979]	2.158189711	1.109821687	0.003321384	0.011388208	yes	up	2.006	3.62	2.57	1.26	2.14	1.64	2.42	4.24	3.64	5.69	1.52	3.01
ENSMUSG00000063286	Gm8995	predicted gene 8995 [Source:MGI Symbol;Acc:MGI:3644223]	2.364544312	1.241562178	2.65E-20	6.87E-19	yes	up	16.24	32.142	14.21	16.38	18.31	19.8	12.5	39.74	34.03	29.79	26.32	30.83
ENSMUSG00000105242	Gm9236	predicted gene 9236 [Source:MGI Symbol;Acc:MGI:3645301]	128.8898342	7.009994669	2.60E-09	2.53E-08	yes	up	0	18.628	0	0	0	0	0	10.74	9.53	7.9	13.9	51.07
ENSMUSG00000047361	Gm973	predicted gene 973 [Source:MGI Symbol;Acc:MGI:2685819]	0.342966301	-1.543861265	1.48E-07	1.15E-06	yes	down	8.926	2.63	10.79	12.45	5.65	4.93	10.81	2.97	1.54	4.88	2.2	1.56
ENSMUSG00000095332	Gm9821	predicted gene 9821 [Source:MGI Symbol;Acc:MGI:3704208]	2.396652437	1.261020705	2.97E-05	0.0001584	yes	up	0.862	1.746	0.5	0.73	1.01	1.23	0.84	2.12	2.03	1.28	1.33	1.97
ENSMUSG00000052005	Gm9864	predicted gene 9864 [Source:MGI Symbol;Acc:MGI:3708663]	2.032110002	1.0229785	0.001859496	0.006821336	yes	up	1.544	2.626	1.52	1.08	1.05	2.93	1.14	3.41	3.12	2.98	1.84	1.78
ENSMUSG00000052769	Gm9889	predicted gene 9889 [Source:MGI Symbol;Acc:MGI:3642568]	0.180105626	-2.473084844	3.66E-13	5.30E-12	yes	down	2.258	0.344	2.28	2.44	2.24	2.66	1.67	0.27	0.32	0.28	0.38	0.47
ENSMUSG00000053214	Gm9899	predicted gene 9899 [Source:MGI Symbol;Acc:MGI:3708711]	0.461555364	-1.115424383	0.000412308	0.001758612	yes	down	0.832	0.352	0.59	0.98	0.64	1.17	0.78	0.45	0.6	0.23	0.26	0.22
ENSMUSG00000103469	Gm9910	predicted gene 9910 [Source:MGI Symbol;Acc:MGI:3642577]	0.4088987	-1.29018462	7.14E-06	4.27E-05	yes	down	3.838	1.328	3.62	2.56	3.45	4.4	5.16	1	1.88	1.63	0.79	1.34
ENSMUSG00000097099	Gm9917	predicted gene 9917 [Source:MGI Symbol;Acc:MGI:3704343]	0.497377612	-1.007586525	2.73E-21	7.48E-20	yes	down	33.568	13.39	36.2	36.06	24.88	39.64	31.06	18.59	13.08	9.27	11.52	14.49
ENSMUSG00000054618	Gm9951	predicted gene 9951 [Source:MGI Symbol;Acc:MGI:3641827]	0.177578632	-2.493470106	0.00097917	0.003846983	yes	down	0.41	0.126	0.48	0.49	0.47	0.41	0.2	0.07	0.09	0.03	0.03	0.41
ENSMUSG00000055370	Gm9968	predicted gene 9968 [Source:MGI Symbol;Acc:MGI:3642146]	0.269783723	-1.890124789	0.001061897	0.00413709	yes	down	0.358	0.082	0.57	0.27	0.45	0.26	0.24	0.15	0.03	0.05	0.11	0.07
ENSMUSG00000068428	Gmnc	geminin coiled-coil domain containing [Source:MGI Symbol;Acc:MGI:2685452]	0.41524736	-1.267957099	3.63E-05	0.000190151	yes	down	1.172	0.422	1.31	1.05	1.02	1.49	0.99	0.19	0.58	0.6	0.22	0.52

ENSMUSG00000034792 Gna15	guanine nucleotide binding protein, alpha 15 [Source:MGI Symbol;Acc:MGI:95770]	3.888003606	1.959029557	2.49E-22	7.33E-21	yes	up	4.86	15.292	4.3	5.27	6.67	2.9	5.16	12.65	14.95	12.49	17.85	18.52
ENSMUSG00000031748 Gnao1	guanine nucleotide binding protein, alpha O [Source:MGI Symbol;Acc:MGI:95775]	0.471888157	-1.083483129	2.28E-25	8.09E-24	yes	down	16.374	6.668	14.84	15.03	15.65	18.11	18.24	5.71	7.65	7.24	6.73	6.01
ENSMUSG00000009108 Gnat2	guanine nucleotide binding protein, alpha transducing 2 [Source:MGI Symbol;Acc:MGI:95779]	6.453454289	2.690071587	0.008708276	0.026669016	yes	up	0.132	0.536	0	0	0.14	0.52	0	1.09	0.28	0.06	0.95	0.3
ENSMUSG00000002769 Gnmt	glycine N-methyltransferase [Source:MGI Symbol;Acc:MGI:1202304]	0.346111152	-1.530692669	8.04E-10	8.25E-09	yes	down	6.494	1.906	5.28	7.25	5.66	7.06	7.22	1.79	1.41	1.76	2.71	1.86
ENSMUSG00000052102 Gnpda1	glucosamine-6-phosphate deaminase 1 [Source:MGI Symbol;Acc:MGI:1347054]	2.905122424	1.538598961	4.25E-65	6.65E-63	yes	up	10.966	26.528	9.18	12.85	12.99	10.71	9.1	26.34	29.61	21.68	24.46	30.55
ENSMUSG00000035311 Gnptab	N-acetylglucosamine-1-phosphate transferase, alpha and beta subunits [Source:MGI Symbol;Acc:MGI:3643902]	2.091378988	1.064454523	9.01E-46	7.82E-44	yes	up	24.388	43.578	23.72	26.5	25.19	19.59	26.94	47.3	43.66	42.56	40.12	44.25
ENSMUSG00000034707 Gns	glucosamine (N-acetyl)-6-sulfatase [Source:MGI Symbol;Acc:MGI:1922862]	2.495754238	1.319475876	8.26E-67	1.34E-64	yes	up	78.252	165.93	79.26	75.85	83.79	72.67	79.69	152.96	170.57	151.42	170.03	184.67
ENSMUSG00000050675 Gp1ba	glycoprotein 1b, alpha polypeptide [Source:MGI Symbol;Acc:MGI:1333744]	0.4082177	-1.292589359	8.69E-05	0.000426333	yes	down	1.09	0.364	0.95	1.35	0.99	1.48	0.68	0.4	0.3	0.45	0.25	0.42
ENSMUSG00000050761 Gp1bb	glycoprotein Ib, beta polypeptide [Source:MGI Symbol;Acc:MGI:107852]	0.149597472	-2.740842301	0.002716033	0.009546457	yes	down	0.598	0.076	0.34	0.88	0.78	0.15	0.84	0.09	0.04	0.02	0.06	0.17
ENSMUSG00000047953 Gp5	glycoprotein 5 (platelet) [Source:MGI Symbol;Acc:MGI:1096363]	0.282273429	-1.824834765	2.85E-07	2.12E-06	yes	down	2.036	0.484	1.95	3.52	1.39	1.85	1.47	0.51	0.77	0.4	0.26	0.48
ENSMUSG00000078810 Gp6	glycoprotein 6 (platelet) [Source:MGI Symbol;Acc:MGI:1889810]	0.318268599	-1.651683271	3.03E-05	0.000161227	yes	down	0.748	0.182	0.56	1.77	0.36	0.51	0.54	0.18	0.11	0.15	0.15	0.32
ENSMUSG00000030054 Gp9	glycoprotein 9 (platelet) [Source:MGI Symbol;Acc:MGI:1860137]	0.239489508	-2.061965643	3.59E-07	2.63E-06	yes	down	6.314	1.278	3.77	12.33	5.09	5.01	5.37	1.92	1.47	1.45	1	0.55
ENSMUSG00000031517 Gpm6a	glycoprotein m6a [Source:MGI Symbol;Acc:MGI:107671]	0.425038023	-1.234336188	3.17E-22	9.21E-21	yes	down	53.8	19.29	59.52	56.02	51.88	53.32	48.26	19.5	17.88	17.85	26.31	14.91
ENSMUSG00000029816 Gpnmb	glycoprotein (transmembrane) nmb [Source:MGI Symbol;Acc:MGI:1934765]	36.11620782	5.174574514	3.91E-285	2.79E-281	yes	up	9.702	285.586	8.74	9.37	11.36	6.64	12.4	319.63	291.57	212.22	304.02	300.49
ENSMUSG00000053101 Gpr141	G protein-coupled receptor 141 [Source:MGI Symbol;Acc:MGI:2672983]	2.612173296	1.385250611	5.62E-09	5.27E-08	yes	up	3.734	6.614	3.19	6.44	1.93	5.01	2.1	6.71	4.79	7.28	6.3	7.99
ENSMUSG00000047462 Gpr141b	G protein-coupled receptor 141B [Source:MGI Symbol;Acc:MGI:2441809]	2.582454973	1.368743195	3.20E-07	2.36E-06	yes	up	1.55	3.372	0.91	1.85	2.46	1.16	1.37	2.77	3.43	4.81	2.38	3.47
ENSMUSG00000047293 Gpr15	G protein-coupled receptor 15 [Source:MGI Symbol;Acc:MGI:1918473]	4.809834221	2.26598717	2.15E-07	1.63E-06	yes	up	0.4	1.6	0.31	0.72	0.18	0.57	0.22	2.04	0.92	1.85	1.2	1.99
ENSMUSG00000047875 Gpr157	G protein-coupled receptor 157 [Source:MGI Symbol;Acc:MGI:2442046]	2.132517583	1.092557637	5.55E-12	7.18E-11	yes	up	2.034	3.656	1.37	2.43	2.11	2.2	2.06	3.87	3.63	3.46	3.05	4.27
ENSMUSG00000037661 Gpr160	G protein-coupled receptor 160 [Source:MGI	2.714697516	1.440791456	9.47E-15	1.60E-13	yes	up	3.386	7.162	4.34	2.47	5.03	2.64	2.45	7.63	9.26	6.54	5.73	6.65

	Symbol;Acc:MGI:1919112]																		
ENSMUSG00000038390 Gpr162	G protein-coupled receptor 162 [Source:MGI Symbol;Acc:MGI:1315214]	6.524912085	2.705958463	8.10E-24	2.61E-22	yes	up	0.764	4.452	0.41	1.04	0.82	0.99	0.56	4.72	3.39	4	3.34	6.81
ENSMUSG00000052229 Gpr17	G protein-coupled receptor 17 [Source:MGI Symbol;Acc:MGI:3584514]	0.341986232	-1.547989849	0.000358388	0.001550553	yes	down	0.726	0.21	1.01	0.52	0.6	1.03	0.47	0.25	0.45	0.18	0.1	0.07
ENSMUSG00000040133 Gpr176	G protein-coupled receptor 176 [Source:MGI Symbol;Acc:MGI:2685858]	7.704307129	2.945665217	2.53E-23	7.95E-22	yes	up	0.434	2.8	0.23	0.79	0.55	0.25	0.35	3.51	2.3	2.31	2.41	3.47
ENSMUSG00000070337 Gpr179	G protein-coupled receptor 179 [Source:MGI Symbol;Acc:MGI:2443409]	0.499804227	-1.000564992	5.07E-05	0.000258502	yes	down	12.818	0.976	61.89	0.44	0.56	0.61	0.59	3.89	0.23	0.16	0.25	0.35
ENSMUSG00000049649 Gpr3	G-protein coupled receptor 3 [Source:MGI Symbol;Acc:MGI:101908]	0.049870586	-4.325667029	0.002069147	0.007498311	yes	down	0.22	0.012	0.42	0.37	0.15	0.14	0.02	0	0	0	0	0.06
ENSMUSG00000116935 Gpr31a	G protein-coupled receptor 31, D17Leh66a region [Source:MGI Symbol;Acc:MGI:1354370]	21.67849568	4.438192743	0.007995206	0.024790117	yes	up	0	0.28	0	0	0	0	0	0.58	0.41	0.27	0	0.14
ENSMUSG00000035148 Gpr33	G protein-coupled receptor 33 [Source:MGI Symbol;Acc:MGI:1277106]	12.07642182	3.59412115	6.71E-08	5.45E-07	yes	up	0.106	1.052	0.21	0.11	0.11	0.05	0.05	0.94	1.46	1.61	0.49	0.76
ENSMUSG00000040229 Gpr34	G protein-coupled receptor 34 [Source:MGI Symbol;Acc:MGI:1346334]	2.292827464	1.197127796	8.28E-10	8.49E-09	yes	up	3.088	6.036	2.39	2.92	4.51	2.26	3.36	5.18	6.29	5.54	5.86	7.31
ENSMUSG00000026271 Gpr35	G protein-coupled receptor 35 [Source:MGI Symbol;Acc:MGI:1929509]	3.320474213	1.731389294	1.88E-27	7.50E-26	yes	up	2.732	6.706	2.27	3.62	2.13	2.67	2.97	6.91	5.75	7.6	5.56	7.71
ENSMUSG00000049608 Gpr55	G protein-coupled receptor 55 [Source:MGI Symbol;Acc:MGI:2685064]	3.539831051	1.823680505	5.59E-14	8.79E-13	yes	up	0.922	2.76	0.76	0.75	1.39	0.57	1.14	2.29	2.97	2.19	3.5	2.85
ENSMUSG00000021886 Gpr65	G-protein coupled receptor 65 [Source:MGI Symbol;Acc:MGI:108031]	2.87285553	1.522485445	5.40E-25	1.87E-23	yes	up	5.864	14.172	6.26	5.68	6.98	5.28	5.12	18.41	13.4	13.61	11.87	13.57
ENSMUSG00000047415 Gpr68	G protein-coupled receptor 68 [Source:MGI Symbol;Acc:MGI:2441763]	3.84412111	1.942653789	1.70E-26	6.48E-25	yes	up	2.084	6.056	2.47	1.8	2	2.69	1.46	7.06	4.42	7.07	4.86	6.87
ENSMUSG00000047678 Gpr82	G protein-coupled receptor 82 [Source:MGI Symbol;Acc:MGI:2441734]	2.541987357	1.345956855	0.000332939	0.001450117	yes	up	0.358	0.764	0.21	0.52	0.49	0.32	0.25	0.93	0.74	0.96	0.58	0.61
ENSMUSG00000063234 Gpr84	G protein-coupled receptor 84 [Source:MGI Symbol;Acc:MGI:1934129]	14.41679782	3.849678851	0.000653322	0.002659989	yes	up	0.028	0.384	0.11	0	0	0	0.03	0.69	0.19	0.35	0.03	0.66
ENSMUSG00000048216 Gpr85	G protein-coupled receptor 85 [Source:MGI Symbol;Acc:MGI:1927851]	6.117867049	2.613028755	2.61E-11	3.14E-10	yes	up	0.206	1.18	0.25	0.14	0.16	0.18	0.3	0.82	1.96	0.86	1.34	0.92
ENSMUSG00000068696 Gpr88	G-protein coupled receptor 88 [Source:MGI Symbol;Acc:MGI:1927653]	0.441119062	-1.180759989	0.001013229	0.00396407	yes	down	0.964	0.374	0.44	1.43	1.02	0.9	1.03	0.22	0.32	0.63	0.26	0.44
ENSMUSG00000019905 Gprc6a	G protein-coupled receptor, family C, group 6, member A [Source:MGI Symbol;Acc:MGI:2429498]	0.395134994	-1.339582474	1.18E-13	1.80E-12	yes	down	4.212	1.408	5.39	3.37	4.09	3.72	4.49	1.49	1.46	1.41	1.53	1.15
ENSMUSG00000034786 Gpsm3	G-protein signalling modulator 3 (AGS3-like, C. elegans) [Source:MGI Symbol;Acc:MGI:2146785]	2.236487452	1.161234664	9.10E-13	1.27E-11	yes	up	13.736	25.912	12.55	11.14	14.14	19.26	11.59	30.98	21.97	23.38	23.41	29.82
ENSMUSG00000042808 Gpx2	glutathione peroxidase 2 [Source:MGI Symbol;Acc:MGI:106609]	0.415008303	-1.268787893	3.83E-10	4.07E-09	yes	down	27.766	9.786	29.05	28.43	18.05	32.4	30.9	8.58	8.46	14.99	8.62	8.28
ENSMUSG00000074934 Grem1	gremlin 1, DAN family BMP antagonist [Source:MGI Symbol;Acc:MGI:1344337]	2.991442015	1.580841098	0.000773015	0.003099585	yes	up	0.38	0.956	0.41	0.86	0.2	0.37	0.06	0.67	0.7	1.67	0.76	0.98
ENSMUSG00000020656 Grhl1	grainyhead like transcription factor 1 [Source:MGI Symbol;Acc:MGI:2182540]	0.466753791	-1.099266353	3.23E-16	6.20E-15	yes	down	4.832	1.84	4.81	4.78	4.97	4.77	4.83	1.91	1.6	1.99	1.72	1.98
ENSMUSG00000020524 Gria1	glutamate receptor, ionotropic, AMPA1 (alpha 1) [Source:MGI	0.078765317	-3.666295686	1.03E-79	2.28E-77	yes	down	5.166	0.348	4.21	4.84	5.44	6	5.34	0.22	0.41	0.54	0.32	0.25

	Symbol;Acc:MGI:95808]																		
ENSMUSG00000056073 Grik2	glutamate receptor, ionotropic, kainate 2 (beta 2) [Source:MGI Symbol;Acc:MGI:95815]	0.439168756	-1.187152675	0.001163362	0.004480231	yes	down	9.016	0.34	1.21	42.48	0.49	0.56	0.34	0.35	0.19	0.41	0.67	0.08
ENSMUSG00000001985 Grik3	glutamate receptor, ionotropic, kainate 3 [Source:MGI Symbol;Acc:MGI:95816]	2.556974618	1.35443784	0.000109707	0.000527028	yes	up	129.468	329.748	118.48	260.58	89.53	79.03	99.72	447.58	292.33	233.98	341.65	333.2
ENSMUSG00000032017 Grik4	glutamate receptor, ionotropic, kainate 4 [Source:MGI Symbol;Acc:MGI:95817]	0.237184646	-2.075917474	0.000291302	0.001286559	yes	down	7.916	0.062	0.19	38.24	0.46	0.39	0.3	0.03	0.16	0.06	0.02	0.04
ENSMUSG00000034813 Grip1	glutamate receptor interacting protein 1 [Source:MGI Symbol;Acc:MGI:1921303]	0.484497679	-1.045438339	3.03E-05	0.000161255	yes	down	1.17	0.512	1.39	0.85	1.01	1.05	1.55	0.56	0.56	0.56	0.51	0.37
ENSMUSG00000030098 Grip2	glutamate receptor interacting protein 2 [Source:MGI Symbol;Acc:MGI:2681173]	0.375649696	-1.412540161	0.005984419	0.01924359	yes	down	0.352	0.1	0.3	0.26	0.55	0.37	0.28	0.17	0.11	0.03	0.08	0.11
ENSMUSG00000034708 Grn	granulin [Source:MGI Symbol;Acc:MGI:95832]	2.72373111	1.445584286	2.42E-68	4.21E-66	yes	up	109.2	255.38	111.73	101.92	106.38	110.55	115.42	216.97	268.7	242.87	248.67	299.69
ENSMUSG00000039934 Gsap	gamma-secretase activating protein [Source:MGI Symbol;Acc:MGI:2442259]	2.268047338	1.181450752	4.72E-38	3.07E-36	yes	up	22.652	42.11	22.55	24.7	23.49	18.7	23.82	37.73	47.39	41	40.28	44.15
ENSMUSG00000046182 Gsg1l	GSG1-like [Source:MGI Symbol;Acc:MGI:2685483]	0.38729471	-1.3684963	5.96E-06	3.62E-05	yes	down	1.644	0.538	1.25	1.62	1.83	2.23	1.29	0.19	0.64	0.61	0.82	0.43
ENSMUSG00000026879 Gsn	gelsolin [Source:MGI Symbol;Acc:MGI:95851]	0.410953546	-1.282952773	9.26E-35	5.33E-33	yes	down	2088.982	723.05	1961.93	2000.77	2021.71	2129.25	2331.25	559.89	832.81	785.51	810.77	626.27
ENSMUSG00000057933 Gsta2	glutathione S-transferase, alpha 2 (Yc2) [Source:MGI Symbol;Acc:MGI:95863]	0.332716888	-1.587632999	3.46E-06	2.19E-05	yes	down	9.926	2.84	9.73	9.49	7.73	12.48	10.2	5.62	2.36	2.15	2.8	1.27
ENSMUSG00000025934 Gsta3	glutathione S-transferase, alpha 3 [Source:MGI Symbol;Acc:MGI:95856]	0.288880636	-1.791454592	3.89E-36	2.35E-34	yes	down	319.856	75.47	373.77	301.61	291.88	366.05	265.97	88.2	68.94	78.11	91.44	50.66
ENSMUSG00000040562 Gstm2	glutathione S-transferase, mu 2 [Source:MGI Symbol;Acc:MGI:95861]	0.419759816	-1.252364031	1.25E-83	3.01E-81	yes	down	220.1	78.174	225.06	221.05	209.45	241.94	203	69.66	82.07	77.68	82.02	79.44
ENSMUSG00000004038 Gstm3	glutathione S-transferase, mu 3 [Source:MGI Symbol;Acc:MGI:106026]	0.466121591	-1.101221754	6.12E-20	1.54E-18	yes	down	374.936	148.472	386.71	384.72	296.49	410.5	396.26	161.45	172.95	147.98	149.98	110
ENSMUSG00000068762 Gstm6	glutathione S-transferase, mu 6 [Source:MGI Symbol;Acc:MGI:1309467]	0.336468056	-1.571458552	1.58E-08	1.40E-07	yes	down	4.71	1.4	4.36	6.53	3.7	4.71	4.25	1.54	1.5	1.68	1.03	1.25
ENSMUSG00000004035 Gstm7	glutathione S-transferase, mu 7 [Source:MGI Symbol;Acc:MGI:1915562]	0.403882984	-1.307990731	2.95E-08	2.53E-07	yes	down	6.39	1.962	5.02	5.58	5.82	7.19	8.34	1.72	2.03	2.29	1.22	2.55
ENSMUSG00000038155 Gstp2	glutathione S-transferase, pi 2 [Source:MGI Symbol;Acc:MGI:95864]	0.005749485	-7.442351453	3.93E-05	0.00020453	yes	down	5.866	0.048	11.1	15.36	1.87	0.74	0.26	0	0	0.24	0	0
ENSMUSG00000022385 Gtse1	G two S phase expressed protein 1 [Source:MGI Symbol;Acc:MGI:1352755]	3.696722398	1.88624671	7.92E-10	8.13E-09	yes	up	0.63	1.968	0.45	0.4	0.9	0.65	0.75	1.81	1.93	1.46	1.44	3.2
ENSMUSG00000025534 Gusb	glucuronidase, beta [Source:MGI Symbol;Acc:MGI:95872]	3.152351039	1.656428199	1.31E-75	2.71E-73	yes	up	33.716	88.236	35.01	33.63	37.17	29.1	33.67	85.31	89.07	82.41	85.3	99.09
ENSMUSG00000023132 Gzma	granzyme A [Source:MGI Symbol;Acc:MGI:109266]	0.443397097	-1.17332877	1.31E-12	1.81E-11	yes	down	59.316	22.262	77.43	50.1	48.46	62.8	57.79	22.09	21.55	15.06	26.48	26.13
ENSMUSG00000015437 Gzmb	granzyme B [Source:MGI Symbol;Acc:MGI:109267]	0.384895379	-1.377461746	9.82E-21	2.63E-19	yes	down	12.816	4.168	14.99	12.58	11.2	12.88	12.43	5.02	4.11	3.91	3.73	4.07

ENSMUSG00000042385	Gzmk	granzyme K [Source:MGI Symbol;Acc:MGI:1298232]	2.351929068	1.233844551	0.006074126	0.019485244	yes	up	1.964	3.664	1.11	0.74	2.92	4.04	1.01	3.87	2.73	3.14	6.2	2.38
ENSMUSG00000036594	H2-Aa	histocompatibility 2, class II antigen A, alpha [Source:MGI Symbol;Acc:MGI:95895]	3.144984886	1.653053084	1.59E-37	1.01E-35	yes	up	710.02	1892.646	620.34	760.67	814.03	598.91	756.15	1791.47	1656.39	1863.39	1622.1	2529.88
ENSMUSG00000073421	H2-Ab1	histocompatibility 2, class II antigen A, beta 1 [Source:MGI Symbol;Acc:MGI:103070]	3.185390856	1.671470406	1.71E-29	7.68E-28	yes	up	606.944	1637.706	507.41	646.3	722.38	500.99	657.64	1488.21	1450.11	1648.12	1300.35	2301.74
ENSMUSG00000049932	H2afx	H2A histone family, member X [Source:MGI Symbol;Acc:MGI:102688]	2.394154237	1.259516097	0.00147641	0.005547158	yes	up	8.93	18.082	4.95	5.21	8.76	10.97	14.76	23.31	16.13	19.6	8.32	23.05
ENSMUSG00000073411	H2-D1	histocompatibility 2, D region locus 1 [Source:MGI Symbol;Acc:MGI:95896]	2.731690432	1.449794	1.09E-38	7.28E-37	yes	up	1135.974	2537.17	1002.83	929.73	1195.34	1200.67	1351.3	2394.74	2679.98	2459.6	2048.66	3102.87
ENSMUSG00000037649	H2-DMa	histocompatibility 2, class II, locus DMa [Source:MGI Symbol;Acc:MGI:95921]	2.624994468	1.392314383	4.90E-22	1.40E-20	yes	up	79.522	161.258	65.05	101.96	86.07	58.12	86.41	132.33	146.1	153.44	156.04	218.38
ENSMUSG00000079547	H2-DMb1	histocompatibility 2, class II, locus Mb1 [Source:MGI Symbol;Acc:MGI:95922]	2.412133734	1.270309896	3.76E-26	1.40E-24	yes	up	72.346	148.306	56.42	76.07	77.07	68.66	83.51	125.79	143.22	141.23	152.28	179.01
ENSMUSG00000060586	H2-Eb1	histocompatibility 2, class II antigen E beta [Source:MGI Symbol;Acc:MGI:95901]	3.192370179	1.674627952	1.88E-30	8.87E-29	yes	up	598.782	1556.042	531.24	665.49	721.25	474.51	601.42	1545.7	1340.79	1526.42	1238.8	2128.5
ENSMUSG00000067341	H2-Eb2	histocompatibility 2, class II antigen E beta2 [Source:MGI Symbol;Acc:MGI:95902]	3.0996388	1.632100108	1.38E-13	2.10E-12	yes	up	2.006	5.166	1.83	1.91	2.81	2.19	1.29	6.66	3.55	5.83	3.85	5.94
ENSMUSG00000061232	H2-K1	histocompatibility 2, K1, K region [Source:MGI Symbol;Acc:MGI:95904]	2.849423168	1.510669892	5.82E-58	7.33E-56	yes	up	1460.332	3686.298	1313.32	1447.28	1543.62	1472.19	1525.25	3297.42	3561.57	3630.2	3301.65	4640.65
ENSMUSG00000016283	H2-M2	histocompatibility 2, M region locus 2 [Source:MGI Symbol;Acc:MGI:95914]	126.2980709	6.980688793	2.69E-105	1.13E-102	yes	up	1.464	168.386	1.36	1.86	2.62	0.47	1.01	157.72	154.22	158.76	153	218.23
ENSMUSG00000016206	H2-M3	histocompatibility 2, M region locus 3 [Source:MGI Symbol;Acc:MGI:95915]	2.157466514	1.109338167	3.10E-23	9.68E-22	yes	up	18.852	34.352	17.83	18.95	21.15	17.53	18.8	31.28	34.29	31.58	31.88	42.73
ENSMUSG00000079507	H2-Q1	histocompatibility 2, Q region locus 1 [Source:MGI Symbol;Acc:MGI:95928]	0.225661429	-2.147768249	1.36E-07	1.05E-06	yes	down	1.376	0.24	1.18	1.52	1.32	2	0.86	0.39	0.21	0.09	0.35	0.16
ENSMUSG00000055413	H2-Q5	histocompatibility 2, Q region locus 5 [Source:MGI Symbol;Acc:MGI:95934]	2.117347302	1.08225793	4.57E-12	5.95E-11	yes	up	50.334	55.406	20.45	55.88	80.34	49.01	45.99	45.31	49.83	40.98	80.63	60.28
ENSMUSG00000073409	H2-Q6	histocompatibility 2, Q region locus 6 [Source:MGI Symbol;Acc:MGI:95935]	2.071892864	1.050949404	1.06E-18	2.48E-17	yes	up	137.86	240.048	132.88	109.98	136.89	159.66	149.89	232.54	239.45	215.14	203.78	309.33
ENSMUSG00000060550	H2-Q7	histocompatibility 2, Q region locus 7 [Source:MGI Symbol;Acc:MGI:95936]	2.448425599	1.291854357	7.40E-25	2.54E-23	yes	up	180.948	362.358	173.2	156.59	188.79	196.05	190.11	349.28	372.09	331.81	283.48	475.13
ENSMUSG00000073405	H2-T-ps	histocompatibility 2, T region locus, pseudogene [Source:MGI	2.480669425	1.310729494	0.000401148	0.001716137	yes	up	1.35	2.864	1.44	1.33	0.54	1.05	2.39	2.57	2.65	2.57	2.6	3.93

		Symbol;Acc:MGI:2442805]																	
ENSMUSG00000075297	H60b	histocompatibility 60b [Source:MGI Symbol;Acc:MGI:3649078]	15.19128648	3.925172145	0.007577499	0.023655936	yes	up	0.012	0.262	0	0	0	0.06	0	0.44	0.56	0.06	0.12	0.13
ENSMUSG00000091618	H60c	histocompatibility 60c [Source:MGI Symbol;Acc:MGI:3774845]	0.188279327	-2.409053492	2.46E-07	1.85E-06	yes	down	1.346	0.25	1.17	1.07	1.06	1.26	2.17	0.02	0.38	0.44	0.12	0.29
ENSMUSG00000028497	Hacd4	3-hydroxyacyl-CoA dehydratase 4 [Source:MGI Symbol;Acc:MGI:1914025]	2.568759547	1.361071851	1.85E-45	1.58E-43	yes	up	8.352	18.636	8.23	10.12	7.96	7.74	7.71	18.61	19.95	16.59	19.42	18.61
ENSMUSG00000003665	Has1	hyaluronan synthase 1 [Source:MGI Symbol;Acc:MGI:106590]	0.441526815	-1.179427036	0.010476101	0.031247378	yes	down	1.892	0.69	2.27	3.17	0.68	0.92	2.42	0.44	0.76	1.3	0.69	0.26
ENSMUSG00000050107	Haspin	histone H3 associated protein kinase [Source:MGI Symbol;Acc:MGI:1194498]	2.629924713	1.3950215	1.49E-06	9.94E-06	yes	up	0.936	2.072	0.63	0.84	1.12	1.34	0.75	2.43	2.05	2.03	1.03	2.82
ENSMUSG00000020399	Havcr2	hepatitis A virus cellular receptor 2 [Source:MGI Symbol;Acc:MGI:2159682]	2.958226536	1.564732536	3.98E-26	1.48E-24	yes	up	7.832	19.858	8.16	9.08	10.1	4.57	7.25	18.91	17.65	20.6	19.74	22.39
ENSMUSG00000069919	Hba-a1	hemoglobin alpha, adult chain 1 [Source:MGI Symbol;Acc:MGI:96015]	0.271226695	-1.882428916	1.97E-13	2.95E-12	yes	down	3924.478	898.95	3891.33	2916.04	3221.91	6078.32	3514.79	580.24	940.44	717.81	1157.05	1099.21
ENSMUSG00000069917	Hba-a2	hemoglobin alpha, adult chain 2 [Source:MGI Symbol;Acc:MGI:96016]	0.273038985	-1.872821141	9.63E-15	1.62E-13	yes	down	11232.294	2599.942	11214.67	8187.03	9296.28	16678.96	10784.53	1716.24	2663.77	2112.6	3381.06	3126.04
ENSMUSG00000052305	Hbb-bs	hemoglobin, beta adult s chain [Source:MGI Symbol;Acc:MGI:5474852]	0.273568925	-1.87002373	4.68E-16	8.76E-15	yes	down	9428.25	2181.538	9361.81	7317.61	8125.8	13636.69	8699.34	1458.73	2153.67	1798.96	2950.2	2546.13
ENSMUSG00000073940	Hbb-bt	hemoglobin, beta adult t chain [Source:MGI Symbol;Acc:MGI:5474850]	0.280486946	-1.833994467	4.39E-12	5.72E-11	yes	down	2594.484	603.866	2673.55	1899.92	2206.51	3826.36	2366.08	379.67	549.41	486.52	852.75	750.98
ENSMUSG00000026874	Hc	hemolytic complement [Source:MGI Symbol;Acc:MGI:96031]	2.055175619	1.03926168	6.90E-19	1.63E-17	yes	up	262.838	480.086	269.99	281.12	287.27	209.99	265.82	401.52	528.61	525.16	537.3	407.84
ENSMUSG00000045502	Hcar2	hydroxycarboxylic acid receptor 2 [Source:MGI Symbol;Acc:MGI:1933383]	2.640340477	1.40072398	3.32E-10	3.56E-09	yes	up	4.04	9.034	3.23	6.98	2.94	2.96	4.09	8.91	9.53	7.37	9.14	10.22
ENSMUSG00000022831	Hcls1	hematopoietic cell specific Lyn substrate 1 [Source:MGI Symbol;Acc:MGI:104568]	2.770200691	1.469990498	4.87E-43	3.79E-41	yes	up	32.278	75.292	28.48	34.65	38.68	31.58	28	74.17	71.84	74.16	66.72	89.57
ENSMUSG00000004698	Hdac9	histone deacetylase 9 [Source:MGI Symbol;Acc:MGI:1931221]	2.310560088	1.208242609	5.10E-18	1.14E-16	yes	up	3.07	21.788	2.62	3.63	2.89	2.69	3.52	56.38	8.06	34.73	4.82	4.95
ENSMUSG00000042770	Hebp1	heme binding protein 1 [Source:MGI Symbol;Acc:MGI:1333880]	2.19939186	1.137104669	3.34E-11	3.96E-10	yes	up	26.298	47.656	23.77	29.4	36.26	18.94	23.12	42.37	49.68	47.12	58.27	40.84
ENSMUSG00000019853	Hebp2	heme binding protein 2 [Source:MGI Symbol;Acc:MGI:1860084]	0.399559531	-1.323517626	0.004124275	0.01381494	yes	down	0.636	0.238	0.73	0.49	0.35	0.52	1.09	0.26	0.24	0.31	0.11	0.27
ENSMUSG00000042807	Hecw2	HECT, C2 and WW domain containing E3 ubiquitin protein ligase 2 [Source:MGI Symbol;Acc:MGI:2685817]	0.389536403	-1.360169936	1.80E-36	1.11E-34	yes	down	17.342	6.566	18.71	17.07	14.36	18.36	18.21	5.43	10	3.67	8.2	5.53
ENSMUSG00000046240	Hepacam	hepatocyte cell adhesion molecule [Source:MGI Symbol;Acc:MGI:1920177]	0.337201761	-1.568316026	0.001716447	0.006355366	yes	down	11.96	0.402	19.24	37.44	0.65	0.98	1.49	0.34	0.51	0.88	0.02	0.26
ENSMUSG00000028940	Hes2	hes family bHLH transcription factor 2 [Source:MGI Symbol;Acc:MGI:1098624]	0.147245245	-2.763707051	2.31E-08	2.01E-07	yes	down	2.176	0.186	1.37	1.59	2.62	3.19	2.11	0.46	0.12	0.12	0.23	0
ENSMUSG00000025232	Hexa	hexosaminidase A [Source:MGI Symbol;Acc:MGI:96073]	3.443611695	1.783922472	1.14E-100	4.29E-98	yes	up	84.27	245.372	86.87	87.57	91.74	70.53	84.64	234.81	242.26	235.83	253.09	260.87
ENSMUSG00000021665	Hexb	hexosaminidase B [Source:MGI Symbol;Acc:MGI:96074]	2.730375876	1.449099573	2.30E-44	1.89E-42	yes	up	50.192	115.148	41.13	53.53	60.48	47.06	48.76	114.01	120.51	113.45	100.19	127.58
ENSMUSG00000004328	Hif3a	hypoxia inducible factor 3, alpha subunit [Source:MGI	0.330856349	-1.595723131	5.17E-11	5.99E-10	yes	down	2.302	0.428	3.18	3.33	1.6	1.37	2.03	0.62	0.48	0.38	0.23	0.43

	Symbol;Acc:MGI:1859778]																		
ENSMUSG00000052565 Hist1h1d	histone cluster 1, H1d [Source:MGI Symbol;Acc:MGI:107502]	3.000945096	1.585416924	0.002508915	0.008904695	yes	up	0.058	0.142	0.05	0.11	0.08	0.01	0.04	0.13	0.18	0.14	0.12	0.14
ENSMUSG00000067455 Hist1h4j	histone cluster 1, H4j [Source:MGI Symbol;Acc:MGI:2448436]	2.466715117	1.302591107	0.011167796	0.032998002	yes	up	0.844	1.752	0.61	1.26	0.99	0.21	1.15	1.64	1.42	1.5	2.24	1.96
ENSMUSG00000081058 Hist2h3c2	histone cluster 2, H3c2 [Source:MGI Symbol;Acc:MGI:2448357]	2.042509337	1.030342674	0.00579915	0.018704005	yes	up	1.874	3.256	1.81	0.88	0.94	3.02	2.72	3.56	2.66	3.52	4.14	2.4
ENSMUSG00000091405 Hist2h4	histone cluster 2, H4 [Source:MGI Symbol;Acc:MGI:2140113]	2.80078907	1.485833337	0.013933712	0.039808482	yes	up	0.392	0.932	0	0.5	0.4	0.57	0.49	1.43	1.6	0.47	0.4	0.76
ENSMUSG00000025877 Hk3	hexokinase 3 [Source:MGI Symbol;Acc:MGI:2670962]	9.46348231	3.242371155	1.30E-56	1.57E-54	yes	up	1.486	12.37	1.47	2.15	1.47	1.01	1.33	11.66	13.04	8.49	10.12	18.54
ENSMUSG00000097295 Hmgb1-ps8	high mobility group box 1, pseudogene 8 [Source:MGI Symbol;Acc:MGI:96114]	3.435293072	1.780433184	0.006045913	0.019415097	yes	up	1.232	3.58	0	0.91	1.61	2.13	1.51	6.82	3.97	4.02	0.88	2.21
ENSMUSG00000027875 Hmgcs2	3-hydroxy-3-methylglutaryl-Coenzyme A synthase 2 [Source:MGI Symbol;Acc:MGI:101939]	0.310886266	-1.685541212	2.66E-16	5.15E-15	yes	down	18.536	4.882	19.62	11.46	25.61	16.1	19.89	5.69	5.78	5.69	4.4	2.85
ENSMUSG00000020330 Hmmr	hyaluronan mediated motility receptor (RHAMM) [Source:MGI Symbol;Acc:MGI:104667]	2.806448762	1.48874572	1.54E-09	1.54E-08	yes	up	0.964	2.27	1.52	1.01	1.04	0.65	0.6	2.33	2.31	1.92	1.87	2.92
ENSMUSG00000005413 Hmox1	heme oxygenase 1 [Source:MGI Symbol;Acc:MGI:96163]	4.041324948	2.014828358	5.51E-64	8.24E-62	yes	up	29.278	99.936	28.48	30.45	33.59	26.2	27.67	120.35	88.1	80.57	103.23	107.43
ENSMUSG00000025176 Hogal	4-hydroxy-2-oxoglutarate aldolase 1 [Source:MGI Symbol;Acc:MGI:1914682]	0.41259742	-1.277193297	1.94E-07	1.48E-06	yes	down	4.428	1.584	4.46	4.67	4.18	5.48	3.35	1.03	1.92	1.98	2.28	0.71
ENSMUSG00000059325 Hopx	HOP homeobox [Source:MGI Symbol;Acc:MGI:1916782]	0.460607479	-1.118390258	3.43E-35	2.00E-33	yes	down	1645.106	640.252	1789.73	1478.55	1536.05	1773.2	1648	552.77	696.38	574.81	771.76	605.54
ENSMUSG00000085696 Hoxaas3	Hoxa cluster antisense RNA 3 [Source:MGI Symbol;Acc:MGI:1919878]	0.283819567	-1.81695404	0.000465961	0.00196476	yes	down	2.89	0.748	2.76	4.12	3.01	3.01	1.55	0.93	0.43	0.41	1.13	0.84
ENSMUSG00000038700 Hoxb5	homeobox B5 [Source:MGI Symbol;Acc:MGI:96186]	0.432950692	-1.207725366	4.37E-19	1.05E-17	yes	down	13.032	4.624	14.93	12.19	13.21	14.11	10.72	5.02	4.77	3.84	5.32	4.17
ENSMUSG00000031613 Hpgd	hydroxyprostaglandin dehydrogenase 15 (NAD) [Source:MGI Symbol;Acc:MGI:108085]	0.446925999	-1.161892122	1.21E-47	1.11E-45	yes	down	741.788	280.018	812.22	688.45	755.5	730.66	722.11	313.36	301.41	250.28	281.02	254.02
ENSMUSG00000029919 Hpgds	hematopoietic prostaglandin D synthase [Source:MGI Symbol;Acc:MGI:1859384]	4.34172804	2.11826936	1.33E-27	5.38E-26	yes	up	1.396	7.982	1.2	3.03	1.09	0.81	0.85	9.73	4.44	9.32	3	13.42
ENSMUSG00000035273 Hpse	heparanase [Source:MGI Symbol;Acc:MGI:1343124]	4.23259552	2.081542627	5.34E-26	1.96E-24	yes	up	4.288	15.258	2.92	5.63	5.29	4.1	3.5	18.84	12.34	10.92	14.43	19.76
ENSMUSG00000071001 Hrct1	histidine rich carboxyl terminus 1 [Source:MGI Symbol;Acc:MGI:1917945]	0.385622138	-1.374740219	3.51E-10	3.75E-09	yes	down	10.448	3.41	10.68	8.81	7.83	12.88	12.04	3.76	4.49	3.85	3	1.95
ENSMUSG00000039628 Hs3st6	heparan sulfate (glucosamine) 3-O-sulfotransferase 6 [Source:MGI Symbol;Acc:MGI:3580487]	0.415096946	-1.268479776	0.000773979	0.003102872	yes	down	1.944	0.67	2.44	1.71	2.01	1.72	1.84	1.05	0.6	0.77	0.58	0.35
ENSMUSG00000016194 Hsd11b1	hydroxysteroid 11-beta dehydrogenase 1 [Source:MGI Symbol;Acc:MGI:103562]	0.434149397	-1.203736514	1.27E-27	5.16E-26	yes	down	311.636	114.57	368.88	277.19	302.72	338.42	270.97	109.19	122.39	91.66	140.47	109.14
ENSMUSG00000019301 Hsd17b1	hydroxysteroid (17-beta) dehydrogenase 1 [Source:MGI Symbol;Acc:MGI:105077]	3.627841597	1.859111465	0.013375436	0.038501121	yes	up	0.174	0.528	0.17	0.06	0.4	0	0.24	0.61	0.26	0.6	0.38	0.79

ENSMUSG00000026228 Htr2b	5-hydroxytryptamine (serotonin) receptor 2B [Source:MGI Symbol;Acc:MGI:109323]	4.56799431	2.191560854	1.83E-24	6.09E-23	yes	up	1.138	4.686	1.29	1.23	0.94	1.34	0.89	5.41	5.89	3.19	4.55	4.39
ENSMUSG00000024798 Htr7	5-hydroxytryptamine (serotonin) receptor 7 [Source:MGI Symbol;Acc:MGI:99841]	6.475983625	2.695099338	2.71E-10	2.92E-09	yes	up	0.192	0.87	0.42	0.17	0.11	0.06	0.2	0.85	0.51	0.86	1.06	1.07
ENSMUSG00000064267 Hvcn1	hydrogen voltage-gated channel 1 [Source:MGI Symbol;Acc:MGI:1921346]	5.324698224	2.412699763	1.61E-149	1.64E-146	yes	up	19.84	91.846	21.56	19.95	21.83	17.53	18.33	85.56	90.69	92.05	88.31	102.62
ENSMUSG00000059854 Hydin	HYDIN, axonemal central pair apparatus protein [Source:MGI Symbol;Acc:MGI:2389007]	0.486741091	-1.038773521	0.001179653	0.004535629	yes	down	3.63	1.56	3.16	3.25	2.89	4.43	4.42	1.31	1.33	2.67	0.86	1.63
ENSMUSG00000026018 Ica1l	islet cell autoantigen 1-like [Source:MGI Symbol;Acc:MGI:1917625]	0.399738563	-1.322871339	0.012852463	0.037225906	yes	down	0.412	0.554	0.66	0.23	0.56	0.34	0.27	0.66	0.04	0.17	1.8	0.1
ENSMUSG00000032174 Icam5	intercellular adhesion molecule 5, telencephalin [Source:MGI Symbol;Acc:MGI:109430]	3.628143361	1.859231463	6.51E-05	0.000327279	yes	up	0.17	0.516	0.24	0.07	0.25	0.12	0.17	0.29	0.48	0.44	0.55	0.82
ENSMUSG00000026009 Icos	inducible T cell co-stimulator [Source:MGI Symbol;Acc:MGI:1858745]	2.374920641	1.247879306	1.96E-09	1.93E-08	yes	up	2.828	4.918	3.32	2.88	2.7	3.05	2.19	6.85	4.2	5.92	2.88	4.74
ENSMUSG00000042745 Id1	inhibitor of DNA binding 1 [Source:MGI Symbol;Acc:MGI:96396]	0.315933945	-1.662305141	1.02E-66	1.64E-64	yes	down	270.47	71.686	233.99	304.35	231.51	314.88	267.62	66.53	77.64	68.68	76.12	69.46
ENSMUSG00000020644 Id2	inhibitor of DNA binding 2 [Source:MGI Symbol;Acc:MGI:96397]	2.079003501	1.055892188	1.38E-17	2.96E-16	yes	up	73.478	129.332	72.37	70.81	99.23	60.33	64.65	124.44	121.46	117.99	136.65	146.12
ENSMUSG00000053560 Ier2	immediate early response 2 [Source:MGI Symbol;Acc:MGI:104815]	0.490778772	-1.026855246	1.11E-11	1.39E-10	yes	down	60.438	25.032	51.15	77.49	45.19	70.04	58.32	25.63	26.68	30.33	19.31	23.21
ENSMUSG00000073489 Ifi204	interferon activated gene 204 [Source:MGI Symbol;Acc:MGI:96429]	3.534442611	1.821482717	3.52E-30	1.65E-28	yes	up	6.058	18.34	5.85	8.78	5.95	5.56	4.15	21.1	18.06	15.7	17.44	19.4
ENSMUSG00000073490 Ifi207	interferon activated gene 207 [Source:MGI Symbol;Acc:MGI:2138302]	3.581121435	1.840411441	2.13E-44	1.76E-42	yes	up	7.1	23.612	6.27	8.94	6.07	6.8	7.42	24.75	27.05	18.04	23.47	24.75
ENSMUSG00000026536 Ifi211	interferon activated gene 211 [Source:MGI Symbol;Acc:MGI:3041120]	3.794783378	1.924017533	9.77E-26	3.52E-24	yes	up	8.112	25.958	7.05	12.03	9.34	5.4	6.74	26.44	22.82	25.51	23.66	31.36
ENSMUSG00000031838 Ifi30	interferon gamma inducible protein 30 [Source:MGI Symbol;Acc:MGI:2137648]	3.087521305	1.626449092	1.00E-56	1.22E-54	yes	up	107.812	281.552	102.28	105.29	124.19	93.46	113.84	290.73	257.13	280.5	255.91	323.49
ENSMUSG00000045777 Ifitm10	interferon induced transmembrane protein 10 [Source:MGI Symbol;Acc:MGI:2444776]	0.385962456	-1.373467578	9.12E-12	1.15E-10	yes	down	15.86	5.518	19.44	11.81	12.47	17.32	18.26	6.43	6.36	4.36	6.78	3.66
ENSMUSG00000055170 Ifng	interferon gamma [Source:MGI Symbol;Acc:MGI:107656]	5.928724175	2.567721679	4.70E-06	2.90E-05	yes	up	0.398	2.328	0.52	0.46	0.46	0.28	0.27	2.68	2.64	2.4	1.5	2.42
ENSMUSG00000020053 Igf1	insulin-like growth factor 1 [Source:MGI Symbol;Acc:MGI:96432]	3.874051584	1.953843164	2.98E-38	1.95E-36	yes	up	8.68	43.334	6.31	9.11	11.87	8.63	7.48	41.51	44.6	41.31	44.4	44.85
ENSMUSG00000048583 Igf2	insulin-like growth factor 2 [Source:MGI Symbol;Acc:MGI:96434]	0.432079723	-1.210630567	2.95E-14	4.74E-13	yes	down	8.294	3.228	7.74	8.5	7.42	9.2	8.61	2.77	2.39	4.69	2.27	4.02
ENSMUSG00000029814 Igf2bp3	insulin-like growth factor 2 mRNA binding protein 3 [Source:MGI Symbol;Acc:MGI:1890359]	2.684041359	1.424406902	0.008365103	0.025765432	yes	up	0.966	1.426	3.08	0.44	0.44	0.73	0.14	3.16	0.45	0.73	1.94	0.85
ENSMUSG00000020427 Igfbp3	insulin-like growth factor binding protein 3 [Source:MGI Symbol;Acc:MGI:96438]	0.227204567	-2.137936258	1.48E-90	4.27E-88	yes	down	93.26	17.97	86.51	85.76	88.21	99.46	106.36	14.84	21.19	15.33	19	19.49
ENSMUSG00000026185 Igfbp5	insulin-like growth factor binding protein 5 [Source:MGI Symbol;Acc:MGI:96440]	0.425388788	-1.233146086	8.58E-18	1.88E-16	yes	down	91.594	32.952	82.87	113.28	78.38	88.46	94.98	28.05	32.96	45.13	33.86	24.76

ENSMUSG00000023046	Igfbp6	insulin-like growth factor binding protein 6 [Source:MGI Symbol;Acc:MGI:96441]	0.262801461	-1.9279548	1.68E-53	1.85E-51	yes	down	330.812	73.714	329.1	318.22	321.82	313.3	371.62	53.55	77.82	83.21	91.48	62.51
ENSMUSG00000095079	Igha	immunoglobulin heavy constant alpha [Source:MGI Symbol;Acc:MGI:96444]	20.80439742	4.378816598	1.24E-27	5.04E-26	yes	up	803.628	14310.362	536.78	1573.52	584.1	389.67	934.07	15952.38	15262.6	15892.8	10029.59	14414.44
ENSMUSG00000087642	Ighe	Immunoglobulin heavy constant epsilon [Source:MGI Symbol;Acc:MGI:2685746]	281.3172387	8.136054151	2.13E-06	1.39E-05	yes	up	0	2.244	0	0	0	0	0	3.51	0.17	0.08	1.37	6.09
ENSMUSG00000076614	Ighg1	immunoglobulin heavy constant gamma 1 (G1m marker) [Source:MGI Symbol;Acc:MGI:96446]	30.0267445	4.908176161	6.57E-07	4.63E-06	yes	up	60.232	1518.768	27.02	83.92	179.24	1.08	9.9	2440	772.12	756.92	1001.49	2623.31
ENSMUSG00000076613	Ighg2b	immunoglobulin heavy constant gamma 2B [Source:MGI Symbol;Acc:MGI:96445]	12.52642577	3.646902917	5.74E-07	4.07E-06	yes	up	120.446	1289.742	16.04	199.04	321.76	24.39	41	1861.04	913.43	1159.79	1272.38	1242.07
ENSMUSG00000076612	Ighg2c	immunoglobulin heavy constant gamma 2C [Source:MGI Symbol;Acc:MGI:2686979]	23.07868353	4.528489026	2.61E-16	5.08E-15	yes	up	168.776	3292.356	110	190.3	389.44	36.57	117.57	4703.31	3148.1	2135.17	4010.23	2464.97
ENSMUSG00000095981	Ighv10-1	immunoglobulin heavy variable 10-1 [Source:MGI Symbol;Acc:MGI:4439620]	4.759955111	2.250947968	1.38E-10	1.54E-09	yes	up	14.198	57.888	13.74	13.44	20.04	6.15	17.62	70.07	56.85	84.99	29.86	47.67
ENSMUSG00000095700	Ighv10-3	immunoglobulin heavy variable V10-3 [Source:MGI Symbol;Acc:MGI:3648785]	28.11429428	4.813231928	4.71E-08	3.91E-07	yes	up	14.078	349.286	22.89	25.37	11.6	6.27	4.26	159.3	1257.68	85.16	64.05	180.24
ENSMUSG00000095416	Ighv1-12	immunoglobulin heavy variable V1-12 [Source:MGI Symbol;Acc:MGI:3646284]	16.20351492	4.018234896	7.19E-09	6.67E-08	yes	up	28.672	400.91	32.74	48.44	13.05	13.1	36.03	415.96	80.61	179.37	1039.51	289.1
ENSMUSG00000096108	Ighv11-2	immunoglobulin heavy variable V11-2 [Source:MGI Symbol;Acc:MGI:4947968]	2.471941148	1.305644396	0.016991199	0.047294452	yes	up	15.986	201.89	3.01	50.12	21.78	2.06	2.96	105.23	336.07	65.73	374.26	128.16
ENSMUSG00000103254	Ighv1-15	immunoglobulin heavy variable 1-15 [Source:MGI Symbol;Acc:MGI:4439782]	5.04472629	2.334775996	0.011019315	0.032595257	yes	up	43.478	186.244	2.96	140.44	28.58	9.97	35.44	391.31	73.08	142.21	56.08	268.54
ENSMUSG00000076695	Ighv1-18	immunoglobulin heavy variable V1-18 [Source:MGI Symbol;Acc:MGI:4439780]	19.024183	4.249762593	3.49E-15	6.12E-14	yes	up	41.368	678.384	28.02	73.26	67.97	9.3	28.29	754.74	302.18	601.64	832.91	900.45
ENSMUSG00000096410	Ighv1-19	immunoglobulin heavy variable V1-19 [Source:MGI Symbol;Acc:MGI:4439779]	12.93539577	3.69325229	3.54E-08	2.99E-07	yes	up	300.052	3305.21	252.27	197.43	812.03	47.45	191.08	5264.26	2310.61	2533.22	2817.98	3599.98
ENSMUSG00000095761	Ighv1-20	immunoglobulin heavy variable V1-20 [Source:MGI Symbol;Acc:MGI:3644607]	35.13204182	5.134715521	2.56E-07	1.92E-06	yes	up	2.43	72.274	1.48	7.26	0	0	3.41	122.25	12.79	73.1	117.37	35.86
ENSMUSG00000094561	Ighv1-22	immunoglobulin heavy variable 1-22 [Source:MGI Symbol;Acc:MGI:4439784]	17.28418084	4.111380325	8.74E-12	1.10E-10	yes	up	19.114	286.43	8.15	46.01	17.76	7.97	15.68	262.13	617.42	97.87	212.44	242.29
ENSMUSG00000094546	Ighv1-26	immunoglobulin heavy variable 1-26 [Source:MGI Symbol;Acc:MGI:4439641]	16.34815911	4.031056285	1.45E-14	2.40E-13	yes	up	121.988	1718.184	80.15	138.21	257.05	29.23	105.3	2765.11	1269.92	1965.26	1541.35	1049.28

ENSMUSG00000093955	Ighv1-34	immunoglobulin heavy variable 1-34 [Source:MGI Symbol;Acc:MGI:4439659]	18.7040078	4.225275531	1.95E-26	7.37E-25	yes	up	157.798	2533.52	85.64	225.96	253.81	61.47	162.11	3925.49	2216.32	2169.01	2071.9	2284.88
ENSMUSG00000094051	Ighv1-36	immunoglobulin heavy variable 1-36 [Source:MGI Symbol;Acc:MGI:4439639]	42.39501033	5.405822572	7.92E-05	0.000391569	yes	up	1.414	50.886	0	1.66	5.41	0	0	171.17	15.23	5.85	38.31	23.87
ENSMUSG00000095923	Ighv1-37	immunoglobulin heavy variable 1-37 [Source:MGI Symbol;Acc:MGI:4439640]	10.42294721	3.381691369	0.003518279	0.011986553	yes	up	1.082	9.686	0	0	5.41	0	0	25.41	8.53	1.81	2.05	10.63
ENSMUSG00000095130	Ighv1-39	immunoglobulin heavy variable 1-39 [Source:MGI Symbol;Acc:MGI:4439888]	16.49817539	4.044234574	2.65E-06	1.71E-05	yes	up	38.788	545.514	2.96	130.75	23.17	16.61	20.45	841.01	799.64	647.85	146.46	292.61
ENSMUSG00000095442	Ighv1-4	immunoglobulin heavy variable 1-4 [Source:MGI Symbol;Acc:MGI:4439618]	19.71918579	4.301528079	1.16E-16	2.32E-15	yes	up	11.696	208.342	6.64	19.48	18.37	7.82	6.17	188.44	321.5	210.49	56.07	265.21
ENSMUSG00000094509	Ighv14-1	immunoglobulin heavy variable 14-1 [Source:MGI Symbol;Acc:MGI:4439920]	12.24406367	3.614010547	0.001182365	0.00454524	yes	up	5.578	57.898	0	16.95	0.77	1.99	8.18	95.17	20.13	21.1	10.56	142.53
ENSMUSG00000095583	Ighv14-2	immunoglobulin heavy variable 14-2 [Source:MGI Symbol;Acc:MGI:4439607]	8.591403287	3.102893795	9.26E-07	6.38E-06	yes	up	36.4	268.4	61.49	65.38	31.56	10.62	12.95	321.76	96.48	467.64	305.46	150.66
ENSMUSG00000095642	Ighv14-3	immunoglobulin heavy variable V14-3 [Source:MGI Symbol;Acc:MGI:4439764]	13.53553584	3.758680096	8.59E-11	9.74E-10	yes	up	105.774	1232.418	107.24	102.46	254.61	25.41	39.15	1180.85	1681.49	1416.03	843.06	1040.66
ENSMUSG00000076666	Ighv14-4	immunoglobulin heavy variable 14-4 [Source:MGI Symbol;Acc:MGI:4439765]	6.887944211	2.784073458	0.011681022	0.034292647	yes	up	32.17	194.72	3.7	15.06	138.06	1.99	2.04	109.71	276.52	109.69	294.86	182.82
ENSMUSG00000076709	Ighv1-47	immunoglobulin heavy variable 1-47 [Source:MGI Symbol;Acc:MGI:4439890]	10.15604128	3.344266259	9.17E-06	5.37E-05	yes	up	2.058	17.396	2.01	2.7	4.69	0.44	0.45	4.91	8.93	41.93	3.09	28.12
ENSMUSG00000096499	Ighv1-5	immunoglobulin heavy variable V1-5 [Source:MGI Symbol;Acc:MGI:3704121]	45.24673489	5.499741783	1.14E-23	3.65E-22	yes	up	9.808	359.988	12.22	8.76	17.1	3.16	7.8	227.83	248.88	731.29	449.97	141.97
ENSMUSG00000094198	Ighv1-50	immunoglobulin heavy variable 1-50 [Source:MGI Symbol;Acc:MGI:4439753]	9.832763362	3.297596923	0.000248533	0.001116315	yes	up	200.196	1659.996	46.58	716.17	160.15	24.16	53.92	3240.15	816.34	1021.49	2417.54	804.46
ENSMUSG00000076688	Ighv15-2	immunoglobulin heavy variable V15-2 [Source:MGI Symbol;Acc:MGI:4947963]	81.25614851	6.344405078	3.17E-07	2.34E-06	yes	up	0.15	13.434	0	0	0.75	0	0	33.65	1.83	20.31	9.6	1.78
ENSMUSG00000095204	Ighv1-52	immunoglobulin heavy variable 1-52 [Source:MGI Symbol;Acc:MGI:4439752]	32.09989863	5.004496836	1.58E-29	7.11E-28	yes	up	9.84	270.61	9.63	9.66	11.59	8.1	10.22	349.89	80.58	236.44	432.24	253.9
ENSMUSG00000093894	Ighv1-53	immunoglobulin heavy variable 1-53 [Source:MGI Symbol;Acc:MGI:3576502]	10.57230473	3.402218009	2.79E-16	5.39E-15	yes	up	79.878	723.23	58.53	150.13	51.85	48.1	90.78	964.94	482.3	479.83	522.02	1167.06
ENSMUSG00000094787	Ighv1-54	immunoglobulin heavy variable V1-54 [Source:MGI Symbol;Acc:MGI:3647133]	24.1158555	4.591910085	1.30E-15	2.36E-14	yes	up	10.298	213.276	4.7	5.07	22.59	2.61	16.52	239.73	153.81	201.17	142.55	329.12

ENSMUSG00000095589 Ighv1-55	immunoglobulin heavy variable 1-55 [Source:MGI Symbol;Acc:MGI:4439716]	16.25032797	4.02239693	6.26E-18	1.39E-16	yes	up	132.504	1834.154	42.83	241.29	85.71	77.06	215.63	2070.1	1543.95	1996.84	1239.32	2320.56
ENSMUSG00000094862 Ighv1-56	immunoglobulin heavy variable 1-56 [Source:MGI Symbol;Acc:MGI:4439715]	86.11415299	6.428178461	0.009241058	0.028079809	yes	up	1.516	73.466	3.05	0	0	4.53	0	4.03	0	259.5	0	103.8
ENSMUSG00000095889 Ighv1-58	immunoglobulin heavy variable 1-58 [Source:MGI Symbol;Acc:MGI:4439557]	20.86002783	4.382669177	3.55E-16	6.75E-15	yes	up	3.064	56.186	0	1.61	1.54	3.99	8.18	101.35	32.89	29.85	67.29	49.55
ENSMUSG00000095197 Ighv1-59	immunoglobulin heavy variable V1-59 [Source:MGI Symbol;Acc:MGI:3644474]	79.53352015	6.313491121	1.21E-11	1.50E-10	yes	up	4.738	325.012	2.96	3.23	3.86	1.99	11.65	827.72	23.24	130.59	559.04	84.47
ENSMUSG00000094087 Ighv1-61	immunoglobulin heavy variable 1-61 [Source:MGI Symbol;Acc:MGI:4439824]	14.79642941	3.887177169	1.80E-06	1.19E-05	yes	up	11.18	140.78	8.15	21.89	5.41	0	20.45	193.86	62.24	233.79	90.4	123.61
ENSMUSG00000096078 Ighv1-62-2	immunoglobulin heavy variable 1-62-2 [Source:MGI Symbol;Acc:MGI:3644968]	97.77553513	6.611401622	1.63E-34	9.34E-33	yes	up	3.704	303.108	0	7.28	1.82	4.66	4.76	248.86	454.25	376.28	156.21	279.94
ENSMUSG00000096767 Ighv1-62-3	immunoglobulin heavy variable 1-62-3 [Source:MGI Symbol;Acc:MGI:3648544]	30.08441035	4.910944175	0.017393688	0.048232267	yes	up	0.308	7.546	0	1.54	0	0	0	30.91	0.79	1.94	4.09	0
ENSMUSG00000096672 Ighv1-63	immunoglobulin heavy variable V1-63 [Source:MGI Symbol;Acc:MGI:3642755]	24.05166597	4.588064923	4.66E-19	1.11E-17	yes	up	2.598	53.924	2.22	1.61	3.09	2.66	3.41	40.28	73.38	97.8	25.73	32.43
ENSMUSG00000094088 Ighv1-64	immunoglobulin heavy variable 1-64 [Source:MGI Symbol;Acc:MGI:4439789]	15.17314082	3.923447847	2.31E-12	3.11E-11	yes	up	143.8	1881.952	54.98	207.33	232.83	36.69	187.17	1711.24	2943.42	999.62	1174.91	2580.57
ENSMUSG00000095519 Ighv1-66	immunoglobulin heavy variable 1-66 [Source:MGI Symbol;Acc:MGI:4439825]	31.44933789	4.974957739	3.43E-22	9.94E-21	yes	up	41.792	1122.084	16.73	67.61	69.48	17.53	37.61	1594.2	902.87	683.24	580.56	1849.55
ENSMUSG00000094502 Ighv1-69	immunoglobulin heavy variable 1-69 [Source:MGI Symbol;Acc:MGI:4439632]	37.23518603	5.21859466	1.26E-14	2.09E-13	yes	up	17.728	565.052	19.26	12.91	30.9	12.62	12.95	1421.31	139.33	760.04	214.63	289.95
ENSMUSG00000095200 Ighv1-7	immunoglobulin heavy variable V1-7 [Source:MGI Symbol;Acc:MGI:3704122]	11.56837064	3.532113776	1.35E-09	1.36E-08	yes	up	51.084	507.384	15.04	125.58	21.22	35.8	57.78	489.15	940.31	345.77	297.47	464.22
ENSMUSG00000096577 Ighv1-71	immunoglobulin heavy variable 1-71 [Source:MGI Symbol;Acc:MGI:3643033]	111.7780176	6.804492684	1.82E-09	1.79E-08	yes	up	0.39	26.698	1.37	0.58	0	0	0	15.78	60.31	45.22	5.56	6.62
ENSMUSG00000096074 Ighv1-72	immunoglobulin heavy variable 1-72 [Source:MGI Symbol;Acc:MGI:4439633]	25.5487354	4.675179978	3.83E-36	2.32E-34	yes	up	110.06	2402.276	58.69	126.04	110.84	55.13	199.6	2412.1	1799.94	2977.68	2419.5	2402.16
ENSMUSG00000094124 Ighv1-74	immunoglobulin heavy variable V1-74 [Source:MGI Symbol;Acc:MGI:4439634]	12.02626151	3.58811633	9.85E-05	0.000477563	yes	up	32.1	333.83	6.67	97.66	20.08	1.33	34.76	518.95	609.9	120.05	127.86	292.39
ENSMUSG00000096020 Ighv1-75	immunoglobulin heavy variable 1-75 [Source:MGI Symbol;Acc:MGI:4439735]	40.99988008	5.357547785	3.37E-16	6.44E-15	yes	up	20.6	730.292	5.54	28.16	51.57	7.58	10.15	373.82	1190.37	362.86	750.14	974.27

ENSMUSG00000093896	Ighv1-76	immunoglobulin heavy variable 1-76 [Source:MGI Symbol;Acc:MGI:4439737]	27.77240544	4.795580233	8.28E-19	1.95E-17	yes	up	32.318	770.092	10.14	35.17	52.79	7.89	55.6	1155.16	640.43	726.24	705.42	623.21
ENSMUSG00000096452	Ighv1-77	immunoglobulin heavy variable 1-77 [Source:MGI Symbol;Acc:MGI:4439670]	27.67745388	4.790639327	4.06E-32	2.10E-30	yes	up	12.258	289.916	14.1	17.01	12.63	7.92	9.63	324.19	194.06	520.22	134.81	276.3
ENSMUSG00000096326	Ighv1-78	immunoglobulin heavy variable 1-78 [Source:MGI Symbol;Acc:MGI:4439736]	80.04703411	6.322776044	9.07E-18	1.98E-16	yes	up	11.136	646.818	11.84	23.91	1.85	15.29	2.79	1259.13	306.92	920.62	243.32	504.1
ENSMUSG00000094075	Ighv1-80	immunoglobulin heavy variable 1-80 [Source:MGI Symbol;Acc:MGI:4439738]	33.04330801	5.046286219	1.10E-21	3.07E-20	yes	up	33.494	968.94	40.75	48.43	24.72	10.63	42.94	773.27	1872.71	425.46	1311.74	461.52
ENSMUSG00000094689	Ighv1-81	immunoglobulin heavy variable 1-81 [Source:MGI Symbol;Acc:MGI:4439635]	14.27759328	3.835680905	3.77E-23	1.17E-21	yes	up	66.858	828.914	68.9	94.43	63.34	29.23	78.39	723.81	1545.75	802.69	450.66	621.66
ENSMUSG00000095127	Ighv1-82	immunoglobulin heavy variable 1-82 [Source:MGI Symbol;Acc:MGI:4439671]	23.63656021	4.562948193	2.73E-25	9.65E-24	yes	up	135.936	2786.522	144.05	108.8	262.74	44.14	119.95	3518.93	3614.34	1203.05	2944.31	2651.98
ENSMUSG00000094940	Ighv1-84	immunoglobulin heavy variable 1-84 [Source:MGI Symbol;Acc:MGI:3644235]	21.56092715	4.430347313	1.26E-07	9.87E-07	yes	up	6.874	130.34	8.15	12.91	3.86	3.32	6.13	31.14	407.1	34.11	27.11	152.24
ENSMUSG00000096150	Ighv1-85	immunoglobulin heavy variable 1-85 [Source:MGI Symbol;Acc:MGI:3645723]	41.13753715	5.362383519	4.35E-06	2.70E-05	yes	up	8.188	289.694	0.74	6.46	26.26	0.66	6.82	151.04	74.3	74.91	37.61	1110.61
ENSMUSG00000094694	Ighv1-9	immunoglobulin heavy variable V1-9 [Source:MGI Symbol;Acc:MGI:4439621]	18.19277573	4.185293771	2.19E-21	6.01E-20	yes	up	61.714	963.47	60.01	102.51	35.53	22.59	87.93	1498.79	674.43	1136.52	694.05	813.56
ENSMUSG00000096464	Ighv2-2	immunoglobulin heavy variable 2-2 [Source:MGI Symbol;Acc:MGI:4439894]	36.03256388	5.171229405	7.78E-14	1.21E-12	yes	up	13.81	423.654	7.03	40.17	7.34	5.69	8.82	377.8	389.17	875.93	105.43	369.94
ENSMUSG00000094164	Ighv2-3	immunoglobulin heavy variable 2-3 [Source:MGI Symbol;Acc:MGI:4439872]	10.13983315	3.341962008	1.12E-12	1.55E-11	yes	up	8.082	71.36	19.26	3.36	7.23	3.46	7.1	58.04	107.22	84.1	22.69	84.75
ENSMUSG00000096498	Ighv2-5	immunoglobulin heavy variable 2-5 [Source:MGI Symbol;Acc:MGI:4439517]	29.46753908	4.881054677	3.52E-23	1.10E-21	yes	up	24.622	681.184	15.52	40.15	21.01	15.6	30.83	629.11	686.98	1322.42	238.38	529.03
ENSMUSG00000096670	Ighv2-6	immunoglobulin heavy variable 2-6 [Source:MGI Symbol;Acc:MGI:4439518]	18.61790981	4.218619209	1.80E-06	1.19E-05	yes	up	9.514	153.358	1.5	1.63	9.36	1.34	33.74	86.11	216.56	219.9	145.97	98.25
ENSMUSG00000095565	Ighv2-9-1	immunoglobulin heavy variable 2-9-1 [Source:MGI Symbol;Acc:MGI:4439519]	23.73705622	4.569069123	1.62E-18	3.73E-17	yes	up	57.224	1173.802	23.87	58.52	126.55	31.38	45.8	1985.54	1310.26	623.16	1347.07	602.98
ENSMUSG00000093838	Ighv3-1	immunoglobulin heavy variable 3-1 [Source:MGI Symbol;Acc:MGI:4439534]	24.27719051	4.60152957	5.93E-09	5.55E-08	yes	up	18.786	391.914	9.37	16.82	40.59	6.94	20.21	1050.19	455.64	298.92	62.44	92.38
ENSMUSG00000076672	Ighv3-6	immunoglobulin heavy variable 3-6 [Source:MGI Symbol;Acc:MGI:4439856]	11.64309035	3.541402129	7.15E-20	1.80E-18	yes	up	55.828	556.5	35.16	44.02	49.92	108.03	42.01	697.72	397.43	433.89	911.13	342.33

ENSMUSG00000076655	Ighv4-1	immunoglobulin heavy variable 4-1 [Source:MGI Symbol;Acc:MGI:4439536]	20.57589302	4.362883141	5.16E-11	5.99E-10	yes	up	16.726	241.828	5.78	15.22	29.35	27.71	5.57	495.54	151.77	196.5	174.97	190.36
ENSMUSG00000095429	Ighv5-12	immunoglobulin heavy variable 5-12 [Source:MGI Symbol;Acc:MGI:4439516]	14.16157725	3.82391005	4.45E-08	3.71E-07	yes	up	17.738	221.424	12.03	6.79	42.98	9.22	17.67	106.79	73.78	551.99	138.09	236.47
ENSMUSG00000094194	Ighv5-16	immunoglobulin heavy variable 5-16 [Source:MGI Symbol;Acc:MGI:4439556]	49.78139179	5.637534661	1.14E-13	1.74E-12	yes	up	7.13	299.722	12.6	6.8	10.57	1.53	4.15	273.56	26.07	318.21	295.44	585.33
ENSMUSG00000095571	Ighv5-17	immunoglobulin heavy variable 5-17 [Source:MGI Symbol;Acc:MGI:4439533]	9.887878645	3.305661037	0.00012004	0.000572554	yes	up	61.734	521.126	7.1	111.16	155.74	12.57	22.1	349.8	322.74	223.16	511.7	1198.23
ENSMUSG00000095612	Ighv5-4	immunoglobulin heavy variable 5-4 [Source:MGI Symbol;Acc:MGI:4439895]	17.17257867	4.102034788	1.08E-10	1.22E-09	yes	up	41.282	602.362	14.67	91.28	39.38	12.77	48.31	896.21	470.6	648.66	171.41	824.93
ENSMUSG00000094951	Ighv5-6	immunoglobulin heavy variable 5-6 [Source:MGI Symbol;Acc:MGI:4439815]	22.24582497	4.475462696	2.67E-11	3.21E-10	yes	up	8.096	153.43	0	15.03	13.64	8.47	3.34	260.3	129.53	99.83	163.57	113.92
ENSMUSG00000095285	Ighv5-9	immunoglobulin heavy variable 5-9 [Source:MGI Symbol;Acc:MGI:4439873]	6.071604385	2.60207779	6.79E-06	4.08E-05	yes	up	21.174	109.494	9.52	28.69	37.27	14.91	15.48	256.88	75.03	31.33	96.13	88.1
ENSMUSG00000095210	Ighv5-9-1	immunoglobulin heavy variable 5-9-1 [Source:MGI Symbol;Acc:MGI:4439810]	35.76712791	5.16056237	1.73E-10	1.91E-09	yes	up	5.764	164.22	3.13	8.66	7.44	3.17	6.42	93.64	128.69	533.02	26.98	38.77
ENSMUSG00000076677	Ighv6-3	immunoglobulin heavy variable 6-3 [Source:MGI Symbol;Acc:MGI:4439854]	32.60435672	5.026992851	5.58E-11	6.44E-10	yes	up	11.952	330.266	7.92	19.21	18.4	1.27	12.96	632.65	68.37	575.54	145.59	229.18
ENSMUSG00000094174	Ighv6-4	immunoglobulin heavy variable V6-4 [Source:MGI Symbol;Acc:MGI:3704120]	22.56440992	4.495977146	2.75E-07	2.06E-06	yes	up	1.072	13.51	0	1.46	0.94	2.53	0.43	14.56	0.75	36.12	7.29	8.83
ENSMUSG00000076680	Ighv6-6	immunoglobulin heavy variable 6-6 [Source:MGI Symbol;Acc:MGI:4439619]	27.37299399	4.774681337	1.62E-10	1.79E-09	yes	up	16.31	382.548	28.54	18.26	19.69	2.51	12.55	111.84	188.77	1048.36	202.87	360.9
ENSMUSG00000076665	Ighv7-1	immunoglobulin heavy variable 7-1 [Source:MGI Symbol;Acc:MGI:4439622]	15.69887489	3.972589262	0.000989893	0.003883192	yes	up	0.394	5.216	0	0	1.38	0.59	0	2.98	0.54	8.86	8.8	4.9
ENSMUSG00000076653	Ighv7-2	immunoglobulin heavy variable 7-2 [Source:MGI Symbol;Acc:MGI:4439623]	13.3102029	3.734460658	0.013125919	0.037905163	yes	up	0.128	2.292	0	0	0	0.64	0	6.06	2.36	0.57	1.28	1.19
ENSMUSG00000076652	Ighv7-3	immunoglobulin heavy variable 7-3 [Source:MGI Symbol;Acc:MGI:4439766]	7.524584435	2.911611908	0.000145353	0.000682798	yes	up	10.108	64.838	0.67	10.25	18.27	10.86	10.49	47.45	27.61	82.34	151.82	14.97
ENSMUSG00000076668	Ighv7-4	immunoglobulin heavy variable 7-4 [Source:MGI Symbol;Acc:MGI:4439763]	10.81238964	3.434613502	1.43E-10	1.58E-09	yes	up	26.146	231.666	5.01	45.7	45.71	15.54	18.77	221.43	138.76	298.57	300.37	199.2
ENSMUSG00000076731	Ighv8-12	immunoglobulin heavy variable V8-12 [Source:MGI Symbol;Acc:MGI:3642873]	9.163570388	3.195909823	3.07E-06	1.96E-05	yes	up	84.18	658.518	58.94	263.02	53.13	24.2	21.61	843.26	610.35	918.38	543.31	377.29

ENSMUSG00000076733	Ighv8-13	immunoglobulin heavy variable 8-13 [Source:MGI Symbol;Acc:MGI:4439734]	31.36227549	4.970958333	7.38E-09	6.84E-08	yes	up	1.062	28.126	1.27	2.83	0	0	1.21	33.2	35.5	16.45	18.74	36.74
ENSMUSG00000102301	Ighv8-2	immunoglobulin heavy variable V8-2 [Source:MGI Symbol;Acc:MGI:3647474]	54.41647558	5.765971615	2.28E-05	0.000124796	yes	up	0	5.792	0	0	0	0	0	0.62	10.69	11.91	1.22	4.52
ENSMUSG00000102364	Ighv8-5	immunoglobulin heavy variable V8-5 [Source:MGI Symbol;Acc:MGI:3645478]	240.7486411	7.911383844	4.33E-09	4.12E-08	yes	up	0.718	146.55	2.08	1.51	0	0	0	437.73	5.14	74.54	58.92	156.42
ENSMUSG00000104452	Ighv8-8	immunoglobulin heavy variable 8-8 [Source:MGI Symbol;Acc:MGI:3815333]	41.04718382	5.359211339	1.91E-32	1.01E-30	yes	up	39.21	1380.804	16.64	31.65	46.58	20.48	80.7	2000.59	1304.77	1542.33	792.04	1264.29
ENSMUSG00000095117	Ighv8-9	immunoglobulin heavy variable V8-9 [Source:MGI Symbol;Acc:MGI:3644475]	24.50076487	4.614754883	1.10E-30	5.28E-29	yes	up	24.094	504.244	17.89	43.85	16.56	9.47	32.7	642.22	398.26	605.35	366.53	508.86
ENSMUSG00000096805	Ighv9-1	immunoglobulin heavy variable 9-1 [Source:MGI Symbol;Acc:MGI:4439911]	11.59741278	3.535731091	9.88E-07	6.76E-06	yes	up	115.18	1145.276	29.03	166.68	84.75	15.25	280.19	1828.13	850.95	921.24	658.23	1467.83
ENSMUSG00000094102	Ighv9-2	immunoglobulin heavy variable V9-2 [Source:MGI Symbol;Acc:MGI:3643816]	21.18619843	4.405052834	2.66E-10	2.87E-09	yes	up	52.114	706.118	23.39	64.08	40.59	5.31	127.2	1059.3	602.39	684.73	330.27	853.9
ENSMUSG00000096459	Ighv9-3	immunoglobulin heavy variable V9-3 [Source:MGI Symbol;Acc:MGI:3642720]	11.83267042	3.564703795	3.58E-08	3.02E-07	yes	up	69.562	708.246	18.98	163.94	93.87	13.29	57.73	977.6	512.82	414.96	637.88	997.97
ENSMUSG00000094322	Ighv9-4	immunoglobulin heavy variable 9-4 [Source:MGI Symbol;Acc:MGI:3646379]	17.47593938	4.127298102	0.000177387	0.000817146	yes	up	3.932	47.216	0	15.38	1.51	0	2.77	46.74	57.2	50.92	15.31	65.91
ENSMUSG00000076609	Igkc	immunoglobulin kappa constant [Source:MGI Symbol;Acc:MGI:96495]	16.42147709	4.037511996	6.20E-33	3.33E-31	yes	up	1699.804	23747.662	993.63	2289.33	2798.95	752.26	1664.85	28126.18	23344.62	24142.52	19773.53	23351.46
ENSMUSG00000096490	Igkv10-94	immunoglobulin kappa variable 10-94 [Source:MGI Symbol;Acc:MGI:3646140]	25.39248739	4.66632982	3.75E-16	7.11E-15	yes	up	83.122	1836.824	32.47	114.34	113.15	21.07	134.58	2524.27	3178.12	956.63	1514.63	1010.47
ENSMUSG00000094902	Igkv10-95	immunoglobulin kappa variable 10-95 [Source:MGI Symbol;Acc:MGI:3644598]	18.61993311	4.218775985	0.000322418	0.001409618	yes	up	0.756	15.644	2.31	0	0	1.47	0	20.28	33.21	1.74	1.93	21.06
ENSMUSG00000094420	Igkv10-96	immunoglobulin kappa variable 10-96 [Source:MGI Symbol;Acc:MGI:4439561]	12.50069411	3.643936299	1.23E-07	9.66E-07	yes	up	639.336	6871.174	264.97	766.6	1719.4	125.67	320.04	8788.2	5108.51	9083.74	6555.97	4819.45
ENSMUSG00000093861	Igkv1-110	immunoglobulin kappa variable 1-110 [Source:MGI Symbol;Acc:MGI:4439558]	11.64961504	3.542210377	4.62E-09	4.38E-08	yes	up	118.902	1182.776	166.07	246.54	90.93	26.31	64.66	1371.62	983.91	1661.25	395.64	1501.46
ENSMUSG00000095737	Igkv11-125	immunoglobulin kappa variable 11-125 [Source:MGI Symbol;Acc:MGI:3642338]	90.56020084	6.500805253	6.58E-18	1.46E-16	yes	up	3.096	241.61	0	0	7.58	4.56	3.34	269.04	162.96	122.2	227.58	426.27
ENSMUSG00000094335	Igkv1-117	immunoglobulin kappa variable 1-117 [Source:MGI Symbol;Acc:MGI:4439721]	11.78224895	3.558543036	4.31E-12	5.63E-11	yes	up	264.86	2637.47	133.96	289.27	593.86	100.96	206.25	3297.19	3702.42	2749.5	1481.68	1956.56

ENSMUSG00000094491	Igkv1-133	immunoglobulin kappa variable 1-133 [Source:MGI Symbol;Acc:MGI:3648380]	14.66506569	3.874311629	5.36E-10	5.62E-09	yes	up	7.204	90.454	6.62	9.76	5.01	6.97	7.66	171.28	108.85	105.37	10.08	56.69
ENSMUSG00000096336	Igkv1-135	immunoglobulin kappa variable 1-135 [Source:MGI Symbol;Acc:MGI:3819952]	17.23665283	4.107407741	1.22E-11	1.52E-10	yes	up	83.254	1218.282	37.96	122.49	176.43	39.61	39.78	1300.99	568.12	1497.95	595.27	2129.08
ENSMUSG00000076570	Igkv12-38	immunoglobulin kappa chain variable 12-38 [Source:MGI Symbol;Acc:MGI:4439614]	63.36327399	5.985574976	1.02E-10	1.15E-09	yes	up	1.388	74.354	0	3.36	0	1.38	2.2	183.22	52.03	21.33	24.06	91.13
ENSMUSG00000095007	Igkv12-41	immunoglobulin kappa chain variable 12-41 [Source:MGI Symbol;Acc:MGI:4439772]	12.58654217	3.653810089	0.000160001	0.000744423	yes	up	22.74	250.672	58.8	26.9	16.16	8.29	3.55	223.32	49.21	18.36	689.23	273.24
ENSMUSG00000096422	Igkv12-44	immunoglobulin kappa variable 12-44 [Source:MGI Symbol;Acc:MGI:4439775]	12.17698924	3.606085566	1.53E-12	2.08E-11	yes	up	96.544	1008.988	108.22	172.03	118.84	29.66	53.97	1383.49	680.53	440.4	1425.67	1114.85
ENSMUSG00000076564	Igkv12-46	immunoglobulin kappa variable 12-46 [Source:MGI Symbol;Acc:MGI:4439773]	20.51453032	4.358574219	5.71E-28	2.37E-26	yes	up	110.434	1949.526	94.09	86.37	170.96	58.02	142.73	1579.64	2823.27	2941.08	1347.46	1056.18
ENSMUSG00000105747	Igkv13-64	immunoglobulin kappa chain variable 13-64 [Source:MGI Symbol;Acc:MGI:5009834]	62.01894892	5.954637171	2.15E-20	5.62E-19	yes	up	2.852	149.888	0	0	7.38	5.66	1.22	127.11	73.77	122.16	94.73	331.67
ENSMUSG00000076538	Igkv13-84	immunoglobulin kappa chain variable 13-84 [Source:MGI Symbol;Acc:MGI:96514]	15.81644341	3.983353317	3.26E-08	2.77E-07	yes	up	18.748	252.85	1.59	32.78	24.5	5.53	29.34	530.73	169.07	123.5	188.93	252.02
ENSMUSG00000079543	Igkv13-85	immunoglobulin kappa chain variable 13-85 [Source:MGI Symbol;Acc:MGI:4439827]	7.284922283	2.864913581	0.00017389	0.000802594	yes	up	49.508	314.164	122.45	26.9	25.32	3.46	69.41	209.86	237.62	553.54	178.2	391.6
ENSMUSG00000096515	Igkv14-100	immunoglobulin kappa chain variable 14-100 [Source:MGI Symbol;Acc:MGI:4439559]	25.21393768	4.656149538	1.33E-09	1.33E-08	yes	up	9.236	198.406	5.81	8.7	6.82	20.84	4.01	511.11	35.81	49.34	157.75	238.02
ENSMUSG00000095771	Igkv14-111	immunoglobulin kappa variable 14-111 [Source:MGI Symbol;Acc:MGI:4439863]	8.653084835	3.113214547	0.000204738	0.000931916	yes	up	424.818	3223.994	41.66	1285.03	323.4	32.49	441.51	4362.37	3618.52	2020.91	3407.75	2710.42
ENSMUSG00000094345	Igkv14-126	immunoglobulin kappa variable 14-126 [Source:MGI Symbol;Acc:MGI:3643131]	14.06911158	3.814459325	0.002070828	0.007501497	yes	up	17.928	216.526	4.85	28.64	50.57	5.58	0	665.58	23.3	36.02	108.29	249.44
ENSMUSG00000096461	Igkv14-130	immunoglobulin kappa variable 14-130 [Source:MGI Symbol;Acc:MGI:3645770]	28.86378965	4.851188825	4.09E-07	2.97E-06	yes	up	2.994	75.108	0	1.58	0.76	2.61	10.02	163.14	50.14	16.64	114.44	31.18
ENSMUSG00000076523	Igkv15-103	immunoglobulin kappa chain variable 15-103 [Source:MGI Symbol;Acc:MGI:96513]	21.1309505	4.401285758	2.91E-13	4.25E-12	yes	up	100.686	1820.11	45.36	121.32	55.89	32.97	247.89	2387.24	1001.72	1852.21	998.15	2861.23
ENSMUSG00000076522	Igkv16-104	immunoglobulin kappa variable 16-104 [Source:MGI Symbol;Acc:MGI:2685913]	15.14390165	3.920665041	1.97E-19	4.83E-18	yes	up	43.678	567.436	37.84	76.72	51.47	24.36	28	980.35	628.42	488.86	500.77	238.78
ENSMUSG00000076514	Igkv17-121	immunoglobulin kappa variable 17-121 [Source:MGI Symbol;Acc:MGI:3647671]	42.2764851	5.40178353	1.53E-12	2.09E-11	yes	up	26.028	949.956	6	20.85	5.16	15.71	82.42	2007.52	854.03	520.99	651.82	715.42

ENSMUSG00000076508	Igkv17-127	immunoglobulin kappa variable 17-127 [Source:MGI Symbol;Acc:MGI:3646891]	13.0836153	3.70968934	3.85E-09	3.68E-08	yes	up	242.388	2423.794	90.25	210.46	646.18	46.91	218.14	4012.57	1515.65	1616.34	2552.5	2421.91
ENSMUSG00000076572	Igkv18-36	immunoglobulin kappa chain variable 18-36 [Source:MGI Symbol;Acc:MGI:4439613]	9.782255816	3.290167193	0.000219074	0.000993369	yes	up	1.538	12.588	2.63	2.35	0	2.71	0	17.04	8.82	33.81	1.14	2.13
ENSMUSG00000076535	Igkv1-88	immunoglobulin kappa chain variable 1-88 [Source:MGI Symbol;Acc:MGI:4439828]	56.65421496	5.824111389	1.61E-06	1.07E-05	yes	up	6.772	322.932	1.34	21.77	4.18	6.57	0	37.96	74.39	513.12	934.25	54.94
ENSMUSG00000076525	Igkv1-99	immunoglobulin kappa variable 1-99 [Source:MGI Symbol;Acc:MGI:4439724]	58.00697835	5.858154565	2.05E-15	3.67E-14	yes	up	10.624	525.392	7.69	1.28	31.41	8.44	4.3	510.19	225.85	696.52	299.59	894.81
ENSMUSG00000098814	Igkv19-93	immunoglobulin kappa chain variable 19-93 [Source:MGI Symbol;Acc:MGI:107617]	31.07916662	4.957875914	2.31E-22	6.80E-21	yes	up	74.734	2002.916	80.34	72.15	72.73	37.74	110.71	3410.31	3350	703.94	1600.4	949.93
ENSMUSG00000105606	Igkv2-109	immunoglobulin kappa variable 2-109 [Source:MGI Symbol;Acc:MGI:3642626]	20.5007855	4.357607283	5.24E-15	8.99E-14	yes	up	33.894	595.174	54.17	61.84	10.65	20.92	21.89	749.73	202.99	436.49	730.73	855.93
ENSMUSG00000076518	Igkv2-112	immunoglobulin kappa variable 2-112 [Source:MGI Symbol;Acc:MGI:3644894]	52.7221478	5.720337239	1.36E-05	7.72E-05	yes	up	3.162	142.224	0.69	0.73	7.73	5.43	1.23	9.12	71.26	15.94	4.18	610.62
ENSMUSG00000076501	Igkv2-137	immunoglobulin kappa chain variable 2-137 [Source:MGI Symbol;Acc:MGI:4439879]	11.98071191	3.582641733	4.34E-06	2.70E-05	yes	up	26.794	271.65	15.89	68.48	11.6	4.56	33.44	619.71	85.71	291.07	98.11	263.65
ENSMUSG00000076596	Igkv3-10	immunoglobulin kappa variable 3-10 [Source:MGI Symbol;Acc:MGI:1330821]	19.51564736	4.286559415	2.61E-11	3.14E-10	yes	up	17.64	291.122	4.21	3.73	33.59	32.04	14.63	345.33	212.57	471.36	143.83	282.52
ENSMUSG00000105499	Igkv3-11	immunoglobulin kappa variable 3-11 [Source:MGI Symbol;Acc:MGI:1330820]	21.25467442	4.409708255	3.10E-20	8.04E-19	yes	up	4.096	74.118	2.2	2.98	7.42	4.89	2.99	180.74	42.55	69.96	35.03	42.31
ENSMUSG00000094117	Igkv3-12	immunoglobulin kappa variable 3-12 [Source:MGI Symbol;Acc:MGI:1330815]	21.42314575	4.421098434	3.38E-15	5.94E-14	yes	up	38.722	706.562	17.86	102.24	21.4	26.95	25.16	1249.79	654.16	815.17	445.88	367.81
ENSMUSG00000095351	Igkv3-2	immunoglobulin kappa variable 3-2 [Source:MGI Symbol;Acc:MGI:1330850]	13.63688071	3.769441776	3.14E-06	2.00E-05	yes	up	204.562	2389.412	17.46	414.81	447.83	51.83	90.88	4145.13	1647.17	1642.63	3191.92	1320.21
ENSMUSG00000094478	Igkv3-3	immunoglobulin kappa variable 3-3 [Source:MGI Symbol;Acc:MGI:1330849]	23.88400909	4.577973118	0.008673885	0.026571293	yes	up	0	2.55	0	0	0	0	0	1.24	0	0.54	3.05	7.92
ENSMUSG00000096715	Igkv3-4	immunoglobulin kappa variable 3-4 [Source:MGI Symbol;Acc:MGI:1330855]	18.51429721	4.210567882	1.87E-16	3.69E-15	yes	up	215.37	3374.764	71.09	329.72	392.41	101.09	182.54	5993.07	2712.36	2941.5	2767.89	2459
ENSMUSG00000095335	Igkv3-5	immunoglobulin kappa chain variable 3-5 [Source:MGI Symbol;Acc:MGI:1330854]	10.89585636	3.445707684	2.41E-07	1.81E-06	yes	up	152.152	1403.758	21.13	65.06	298.65	313.32	62.6	1776.58	1261.46	1719.8	950.91	1310.04
ENSMUSG00000076598	Igkv3-7	immunoglobulin kappa variable 3-7 [Source:MGI Symbol;Acc:MGI:1330852]	8.90773542	3.155058707	0.002395746	0.008548368	yes	up	36.812	281.972	3.44	5.97	140.29	10.45	23.91	420.5	44.46	170	142.44	632.46

ENSMUSG00000095338	Igkv3-9	immunoglobulin kappa variable 3-9 [Source:MGI Symbol;Acc:MGI:1330856]	100.8931062	6.656683791	2.87E-08	2.47E-07	yes	up	0.126	16.242	0	0	0	0	0.63	17.36	9.57	0.54	39.61	14.13
ENSMUSG00000094993	Igkv4-51	immunoglobulin kappa chain variable 4-51 [Source:MGI Symbol;Acc:MGI:5009829]	16.65481456	4.057867386	2.33E-05	0.000127244	yes	up	18.042	256.422	12.82	4.05	65.03	1.37	6.94	558.18	60.37	282.68	222.5	158.38
ENSMUSG00000096833	Igkv4-55	immunoglobulin kappa variable 4-55 [Source:MGI Symbol;Acc:MGI:2686370]	30.06802592	4.910158247	2.06E-28	8.80E-27	yes	up	75.23	1969.032	27.5	111.01	117.9	63.79	55.95	2112.57	1546.14	1181.68	3544.1	1460.67
ENSMUSG00000076556	Igkv4-57	immunoglobulin kappa variable 4-57 [Source:MGI Symbol;Acc:MGI:2685035]	10.92145321	3.449092929	6.23E-09	5.81E-08	yes	up	116.128	1147.458	41.95	182.18	213.88	31.48	111.15	1403.44	1188.23	640.45	770.65	1734.52
ENSMUSG00000076555	Igkv4-57-1	immunoglobulin kappa variable 4-57-1 [Source:MGI Symbol;Acc:MGI:2686264]	13.09512468	3.710957891	2.09E-10	2.28E-09	yes	up	21.592	242.058	14.57	56.48	10.74	16.23	9.94	367.59	235.05	76.24	331.77	199.64
ENSMUSG00000095633	Igkv4-58	immunoglobulin kappa variable 4-58 [Source:MGI Symbol;Acc:MGI:2685923]	18.60242566	4.217418849	5.15E-05	0.000262299	yes	up	7.09	113.268	0	3.77	25.66	1.86	4.16	248.06	19.79	122.97	106.86	68.66
ENSMUSG00000094006	Igkv4-59	immunoglobulin kappa variable 4-59 [Source:MGI Symbol;Acc:MGI:3646808]	14.4629442	3.854289364	7.63E-07	5.32E-06	yes	up	110.784	1379.938	38.72	41.17	297.22	27.91	148.9	1490.27	645.8	605.89	800.78	3356.95
ENSMUSG00000104679	Igkv4-60	immunoglobulin kappa variable 4-60 [Source:MGI Symbol;Acc:MGI:3708737]	21.6992198	4.439571266	0.009186722	0.027950396	yes	up	0	2.358	0	0	0	0	0	1.88	1.14	0	2.46	6.31
ENSMUSG00000076552	Igkv4-61	immunoglobulin kappa chain variable 4-61 [Source:MGI Symbol;Acc:MGI:4439819]	14.80290973	3.887808882	9.12E-10	9.31E-09	yes	up	65	769.234	35.64	153.64	66.59	20.8	48.33	1755.49	654.65	359.27	468.79	607.97
ENSMUSG00000076549	Igkv4-68	immunoglobulin kappa variable 4-68 [Source:MGI Symbol;Acc:MGI:2686265]	48.94580039	5.613113175	3.87E-11	4.55E-10	yes	up	70.03	2865.358	37.88	229.71	24.53	44.27	13.76	1225.86	1679.05	8129.56	1768.21	1524.11
ENSMUSG00000076548	Igkv4-69	immunoglobulin kappa variable 4-69 [Source:MGI Symbol;Acc:MGI:3648668]	15.77004504	3.979114875	0.010168474	0.03045281	yes	up	1.838	25.23	4.58	2.5	0	0	2.11	84.44	0	2.41	28.58	10.72
ENSMUSG00000073028	Igkv4-71	immunoglobulin kappa chain variable 4-71 [Source:MGI Symbol;Acc:MGI:4439654]	22.90663579	4.517693686	0.001657557	0.006156476	yes	up	0	4.34	0	0	0	0	0	5.92	3.91	2.63	4.57	4.67
ENSMUSG00000076545	Igkv4-72	immunoglobulin kappa chain variable 4-72 [Source:MGI Symbol;Acc:MGI:2686345]	23.08485694	4.528874887	2.29E-13	3.39E-12	yes	up	59.266	1171.752	13.54	93.94	106.92	20.64	61.29	1886.18	941.4	732.93	1758.88	539.37
ENSMUSG00000076543	Igkv4-74	immunoglobulin kappa variable 4-74 [Source:MGI Symbol;Acc:MGI:3779447]	37.26928444	5.219915215	7.87E-12	1.00E-10	yes	up	21.272	677.018	12.41	43.99	28.92	14.77	6.27	1251.78	596.53	76.33	1193.62	266.83
ENSMUSG00000105462	Igkv4-77	immunoglobulin kappa variable 4-77 [Source:MGI Symbol;Acc:MGI:3647937]	12.39168919	3.631300958	0.000289867	0.001280751	yes	up	0.688	7.472	1.47	0	0	1.97	0	3.99	10.77	15.08	3.29	4.23
ENSMUSG00000076541	Igkv4-79	immunoglobulin kappa variable 4-79 [Source:MGI Symbol;Acc:MGI:2685040]	12.45849511	3.639057907	2.85E-05	0.000152754	yes	up	4.18	43.998	0.69	15.07	0.72	1.24	3.18	50.09	22.59	16.33	42.46	88.52

ENSMUSG00000076540 Igkv4-80	immunoglobulin kappa variable 4-80 [Source:MGI Symbol;Acc:MGI:4439653]	42.21901016	5.399820849	4.33E-07	3.14E-06	yes	up	21.608	790.634	0.77	1.68	44.99	3.46	57.14	400.92	1592.46	209.96	889.01	860.82
ENSMUSG00000076533 Igkv4-90	immunoglobulin kappa chain variable 4-90 [Source:MGI Symbol;Acc:MGI:4439830]	322.675656	8.333940929	1.01E-06	6.87E-06	yes	up	0.32	82.09	0	1.6	0	0	0	203.32	10.25	5.79	4.57	186.52
ENSMUSG00000076532 Igkv4-91	immunoglobulin kappa chain variable 4-91 [Source:MGI Symbol;Acc:MGI:3642277]	15.38824813	3.943757093	4.81E-07	3.46E-06	yes	up	44.992	601.164	6.93	118.32	59.97	12.41	27.33	529.71	1414.48	304.97	549.46	207.2
ENSMUSG00000076531 Igkv4-92	immunoglobulin kappa variable 4-92 [Source:MGI Symbol;Acc:MGI:2686254]	9.487063969	3.245961675	0.002208268	0.007938939	yes	up	0.67	3.078	1.51	0	0	1.33	0.51	6.03	1.15	4.49	3.72	0
ENSMUSG00000076569 Igkv5-39	immunoglobulin kappa variable 5-39 [Source:MGI Symbol;Acc:MGI:2686255]	27.14051977	4.762376445	1.83E-10	2.01E-09	yes	up	14.728	336.696	8.5	10.41	37.61	7.08	10.04	770.86	100.95	527.41	94.05	190.21
ENSMUSG00000094433 Igkv5-43	immunoglobulin kappa chain variable 5-43 [Source:MGI Symbol;Acc:MGI:4943320]	18.28287412	4.192420979	1.10E-16	2.22E-15	yes	up	43.728	683.092	22.11	51.75	82.83	47.28	14.67	914.93	608.71	322.49	454.91	1114.42
ENSMUSG00000076563 Igkv5-48	immunoglobulin kappa variable 5-48 [Source:MGI Symbol;Acc:MGI:3642817]	12.73606121	3.67084727	9.23E-06	5.40E-05	yes	up	67.604	739.524	7.49	50.28	143.96	7.91	128.38	643.63	417.54	1018.87	1263.71	353.87
ENSMUSG00000076594 Igkv6-13	immunoglobulin kappa variable 6-13 [Source:MGI Symbol;Acc:MGI:1330829]	16.50757579	4.045056364	3.59E-20	9.27E-19	yes	up	51.47	713.758	31.94	58.39	82.36	28.6	56.06	1021.13	272.64	939.55	494.21	841.26
ENSMUSG00000096844 Igkv6-14	immunoglobulin kappa variable 6-14 [Source:MGI Symbol;Acc:MGI:1330830]	14.63264874	3.871119039	5.21E-05	0.000264996	yes	up	16.248	198.14	1.75	15.53	12.28	3.77	47.91	281.27	26.97	323.21	45.15	314.1
ENSMUSG00000094797 Igkv6-15	immunoglobulin kappa variable 6-15 [Source:MGI Symbol;Acc:MGI:1330831]	15.99498665	3.999547883	1.23E-08	1.11E-07	yes	up	89.124	1220.662	18.49	119.35	212.11	35.28	60.39	2285.09	833.08	1728.16	639.76	617.22
ENSMUSG00000095794 Igkv6-17	immunoglobulin kappa variable 6-17 [Source:MGI Symbol;Acc:MGI:1330833]	15.90441902	3.991355767	1.09E-28	4.76E-27	yes	up	304.466	4116.328	245.29	544.5	353.29	105.9	273.35	4638.63	3991.36	4015.69	3157.77	4778.19
ENSMUSG00000076587 Igkv6-20	immunoglobulin kappa variable 6-20 [Source:MGI Symbol;Acc:MGI:1330836]	28.86156074	4.851077413	5.54E-25	1.91E-23	yes	up	22.77	560.626	11.94	24.52	25.46	34.03	17.9	1102.67	276.55	232.29	637.58	554.04
ENSMUSG00000095630 Igkv6-23	immunoglobulin kappa variable 6-23 [Source:MGI Symbol;Acc:MGI:3711980]	15.34352463	3.939558023	9.53E-12	1.20E-10	yes	up	382.522	5081.414	258.21	834.21	402.64	110.99	306.56	5094.66	10102.2	4389.38	3068.63	2752.2
ENSMUSG00000094930 Igkv6-25	immunoglobulin kappa chain variable 6-25 [Source:MGI Symbol;Acc:MGI:4439867]	41.1897675	5.364214078	6.82E-18	1.50E-16	yes	up	63.568	2260.61	142.43	82.4	25.02	28.54	39.45	4224.71	1131.88	1042.91	1503.51	3400.04
ENSMUSG00000076576 Igkv6-32	immunoglobulin kappa variable 6-32 [Source:MGI Symbol;Acc:MGI:3641634]	11.03972338	3.464632118	7.22E-08	5.84E-07	yes	up	145.406	1385.91	89	327.94	140.36	17.85	151.88	2078.78	1611.36	857.72	1740.23	641.46
ENSMUSG00000096594 Igkv8-19	immunoglobulin kappa variable 8-19 [Source:MGI Symbol;Acc:MGI:1330844]	30.93655278	4.951240543	1.97E-26	7.44E-25	yes	up	17.652	471.052	18.06	16.86	9.08	26.67	17.59	263.6	843.39	360.64	186.33	701.3

ENSMUSG00000076586	Igkv8-21	immunoglobulin kappa variable 8-21 [Source:MGI Symbol;Acc:MGI:1330840]	7.787561082	2.961171574	7.21E-05	0.000359205	yes	up	41.802	278.364	36.06	137.44	11.15	8.02	16.34	200.08	237.15	210.19	431.47	312.93
ENSMUSG00000076583	Igkv8-24	immunoglobulin kappa chain variable 8-24 [Source:MGI Symbol;Acc:MGI:4947958]	20.80253295	4.378687299	1.07E-14	1.80E-13	yes	up	33.772	607.902	26.03	35.28	74.54	11.62	21.39	398.9	446.74	270.27	1242	681.6
ENSMUSG00000076580	Igkv8-27	immunoglobulin kappa chain variable 8-27 [Source:MGI Symbol;Acc:MGI:4439868]	11.9612307	3.580293932	4.75E-06	2.92E-05	yes	up	17.77	164.672	3.4	23.59	26.95	9.13	25.78	187.95	17.84	65.97	156.3	395.3
ENSMUSG00000094356	Igkv8-28	immunoglobulin kappa variable 8-28 [Source:MGI Symbol;Acc:MGI:3642251]	16.96467967	4.084462284	1.48E-07	1.15E-06	yes	up	25.33	362.728	28.03	60.69	11.02	15	11.91	469.28	107.2	693.29	45.62	498.25
ENSMUSG00000076577	Igkv8-30	immunoglobulin kappa chain variable 8-30 [Source:MGI Symbol;Acc:MGI:3642250]	17.74738319	4.149534413	1.43E-22	4.27E-21	yes	up	107.996	1640.924	109.28	166.49	106.37	52.32	105.52	2763.85	1617.31	1275.24	610.87	1937.35
ENSMUSG00000104769	Igkv8-34	immunoglobulin kappa variable 8-34 [Source:MGI Symbol;Acc:MGI:4949914]	49.40043877	5.626451951	0.000158894	0.000739916	yes	up	0	4.986	0	0	0	0	0	1.18	0	4.1	13.24	6.41
ENSMUSG00000094872	Igkv9-120	immunoglobulin kappa chain variable 9-120 [Source:MGI Symbol;Acc:MGI:3647784]	23.03389291	4.525686353	2.02E-11	2.46E-10	yes	up	34.732	681.94	45.79	46.68	47.01	21.49	12.69	553.6	67.01	1149.66	642.39	997.04
ENSMUSG00000096632	Igkv9-124	immunoglobulin kappa chain variable 9-124 [Source:MGI Symbol;Acc:MGI:3646892]	8.565055111	3.098462529	4.65E-07	3.35E-06	yes	up	38.502	275.396	15.26	21.36	98.15	14.33	43.41	381.14	195.63	278.93	99.83	421.45
ENSMUSG00000093906	Igkv9-129	immunoglobulin kappa variable 9-129 [Source:MGI Symbol;Acc:MGI:3525017]	40.19624585	5.328988861	6.14E-06	3.71E-05	yes	up	1.006	35.032	1.5	1.58	0	1.95	0	55.91	91.33	2.36	5.17	20.39
ENSMUSG00000076937	Iglc2	immunoglobulin lambda constant 2 [Source:MGI Symbol;Acc:MGI:99547]	5.978655383	2.579821055	1.13E-15	2.07E-14	yes	up	72.788	374.09	68.27	90.2	92.92	60.94	51.61	399.83	285.08	323.81	241.55	620.18
ENSMUSG00000105547	Iglc3	immunoglobulin lambda constant 3 [Source:MGI Symbol;Acc:MGI:99886]	5.138778868	2.361425572	1.52E-06	1.01E-05	yes	up	21.166	92.072	15.1	22.38	22.17	26.57	19.61	125.63	48.28	58.59	32.06	195.8
ENSMUSG00000076934	Iglv1	immunoglobulin lambda variable 1 [Source:MGI Symbol;Acc:MGI:96530]	11.63116223	3.539923358	1.17E-17	2.53E-16	yes	up	231.628	2612.854	224.79	323.48	382.25	117.5	110.12	2538.17	1789.59	2178.61	2689.76	3868.14
ENSMUSG00000076939	Iglv3	immunoglobulin lambda variable 3 [Source:MGI Symbol;Acc:MGI:3645509]	11.4345991	3.51533388	2.45E-15	4.37E-14	yes	up	18.244	180.922	11.47	21.61	22.42	9.96	25.76	91.64	137.46	150.24	140.17	385.1
ENSMUSG00000022790	Igsf11	immunoglobulin superfamily, member 11 [Source:MGI Symbol;Acc:MGI:2388477]	0.408841396	-1.290386817	0.001359857	0.005150076	yes	down	0.816	0.274	0.91	0.94	0.36	0.75	1.12	0.23	0.13	0.47	0.23	0.31
ENSMUSG00000035004	Igsf6	immunoglobulin superfamily, member 6 [Source:MGI Symbol;Acc:MGI:1891393]	4.465406683	2.158791573	2.15E-53	2.35E-51	yes	up	7.2	27.012	5.78	8.19	7.35	9.08	5.6	32.71	23.94	25.26	25.59	27.56
ENSMUSG00000038034	Igsf8	immunoglobulin superfamily, member 8 [Source:MGI Symbol;Acc:MGI:2154090]	2.677619038	1.420950714	2.59E-43	2.04E-41	yes	up	13.404	30.266	13.48	13.85	14.55	12.57	12.57	24.82	29.04	34.14	30.04	33.29

ENSMUSG00000054072 Iigp1	interferon inducible GTPase 1 [Source:MGI Symbol;Acc:MGI:1926259]	2.8203293	1.495863621	1.05E-08	9.52E-08	yes	up	24.102	57.038	24.43	19.59	30.8	16.5	29.19	67.07	43.67	47.21	46.49	80.75
ENSMUSG00000042349 Ikbke	inhibitor of kappaB kinase epsilon [Source:MGI Symbol;Acc:MGI:1929612]	2.594731303	1.375585148	2.65E-19	6.48E-18	yes	up	4.354	10.356	4.21	4.45	3.65	5.9	3.56	8.81	14.91	8.96	8.26	10.84
ENSMUSG00000025997 Ikzf2	IKAROS family zinc finger 2 [Source:MGI Symbol;Acc:MGI:1342541]	2.156770508	1.108872674	1.57E-11	1.93E-10	yes	up	1.248	2.562	1.33	1.23	1.05	1.36	1.27	3.57	1.78	3.55	1.79	2.12
ENSMUSG00000032089 Il10ra	interleukin 10 receptor, alpha [Source:MGI Symbol;Acc:MGI:96538]	2.50298016	1.323646856	1.20E-31	6.00E-30	yes	up	10.79	22.738	9.61	13.2	10.09	12.01	9.04	24.37	21.5	21.81	20.28	25.73
ENSMUSG00000004371 Il11	interleukin 11 [Source:MGI Symbol;Acc:MGI:107613]	0.330623129	-1.59674044	0.002039753	0.007406467	yes	down	3.508	1.386	0.5	0.69	15.07	0.59	0.69	0.27	5.79	0.14	0.09	0.64
ENSMUSG00000027776 Il12a	interleukin 12a [Source:MGI Symbol;Acc:MGI:96539]	0.35908891	-1.477586997	0.002415265	0.008615142	yes	down	3.642	0.858	2.17	4.37	3.12	6.89	1.66	0.3	0.8	0.28	0.64	2.27
ENSMUSG00000004296 Il12b	interleukin 12b [Source:MGI Symbol;Acc:MGI:96540]	2.17168636	1.118815761	0.000346755	0.001505697	yes	up	1.028	1.88	1.05	1.31	1.15	0.55	1.08	1.81	1.19	2.65	1.81	1.94
ENSMUSG00000000791 Il12rb1	interleukin 12 receptor, beta 1 [Source:MGI Symbol;Acc:MGI:104579]	3.377027755	1.755754037	4.34E-06	2.70E-05	yes	up	0.88	1.682	2.27	0.19	0.69	0.56	0.69	1.48	0.83	1.37	2.47	2.26
ENSMUSG00000018341 Il12rb2	interleukin 12 receptor, beta 2 [Source:MGI Symbol;Acc:MGI:1270861]	2.158633386	1.110118243	3.13E-09	3.03E-08	yes	up	1.302	2.384	1.63	1.11	1.25	1.12	1.4	2.34	2.78	2.06	2.11	2.63
ENSMUSG00000025929 Il17a	interleukin 17A [Source:MGI Symbol;Acc:MGI:107364]	9.421058777	3.235889205	0.00471606	0.015582999	yes	up	0.092	0.714	0.33	0	0.07	0.06	0	0.61	0.16	1.33	0	1.47
ENSMUSG00000015966 Il17rb	interleukin 17 receptor B [Source:MGI Symbol;Acc:MGI:1355292]	2.288722077	1.194542285	0.001980572	0.007223421	yes	up	0.472	0.862	0.28	0.29	0.45	0.99	0.35	0.71	1.17	0.64	0.55	1.24
ENSMUSG00000070427 Il18bp	interleukin 18 binding protein [Source:MGI Symbol;Acc:MGI:1333800]	4.17838831	2.062946572	1.70E-17	3.63E-16	yes	up	6.828	24.124	5.41	5.81	7.67	11.62	3.63	24.74	17.65	19.27	17.77	41.19
ENSMUSG00000027399 Il1a	interleukin 1 alpha [Source:MGI Symbol;Acc:MGI:96542]	2.186697555	1.128753693	8.06E-08	6.48E-07	yes	up	2.524	4.646	3.46	2.77	2.61	2	1.78	5.3	4.96	3.63	5.37	3.97
ENSMUSG00000026981 Il1rn	interleukin 1 receptor antagonist [Source:MGI Symbol;Acc:MGI:96547]	4.008473924	2.003053089	3.63E-54	4.07E-52	yes	up	11.12	31.254	12.81	11.3	11.92	7.79	11.78	32.9	32.39	30.09	30.66	30.23
ENSMUSG00000027718 Il21	interleukin 21 [Source:MGI Symbol;Acc:MGI:1890474]	39.92257937	5.31913303	1.09E-05	6.32E-05	yes	up	0.01	0.302	0	0.03	0.02	0	0	0.77	0.02	0.28	0.19	0.25
ENSMUSG00000030745 Il21r	interleukin 21 receptor [Source:MGI Symbol;Acc:MGI:1890475]	3.506293005	1.809946561	1.37E-19	3.39E-18	yes	up	4.27	12.408	4.74	4.58	5.2	4.53	2.3	13.36	10.11	12.05	10.58	15.94
ENSMUSG00000037157 Il22ra1	interleukin 22 receptor, alpha 1 [Source:MGI Symbol;Acc:MGI:2663588]	0.432858043	-1.208034128	0.011342201	0.033439506	yes	down	0.892	0.324	0.52	0.58	0.9	1.3	1.16	0.43	0.14	0.68	0.19	0.18
ENSMUSG00000039760 Il22ra2	interleukin 22 receptor, alpha 2 [Source:MGI Symbol;Acc:MGI:2665114]	0.217477064	-2.201064836	3.07E-06	1.96E-05	yes	down	1.204	0.22	1.23	0.96	1.47	1	1.36	0.11	0.41	0.43	0.07	0.08
ENSMUSG00000049093 Il23r	interleukin 23 receptor [Source:MGI Symbol;Acc:MGI:2181693]	2.51584088	1.331040679	0.009612542	0.029039666	yes	up	0.326	0.684	0.19	0.17	0.47	0.6	0.2	0.62	0.27	0.91	0.4	1.22
ENSMUSG00000044701 Il27	interleukin 27 [Source:MGI Symbol;Acc:MGI:2384409]	3.160471079	1.660139613	0.012984577	0.037552693	yes	up	0.258	0.686	0.16	0.42	0.08	0.49	0.14	0.88	0.5	0.68	0.61	0.76
ENSMUSG00000005465 Il27ra	interleukin 27 receptor, alpha [Source:MGI Symbol;Acc:MGI:1355318]	2.050562186	1.036019496	3.47E-09	3.34E-08	yes	up	3.624	6.058	3.06	3.39	4.36	4.18	3.13	6.22	5.85	5.98	4.33	7.91
ENSMUSG00000050377 Il31ra	interleukin 31 receptor A [Source:MGI Symbol;Acc:MGI:2180511]	0.147172313	-2.76442181	0.007826118	0.02432218	yes	down	0.166	0.04	0.33	0.05	0.08	0.12	0.25	0	0.04	0.03	0	0.13
ENSMUSG00000074141 Il4i1	interleukin 4 induced 1 [Source:MGI Symbol;Acc:MGI:109552]	4.878437571	2.286419166	2.45E-13	3.62E-12	yes	up	2.08	6.848	2.51	1.93	1.73	1.9	2.33	7.93	4.04	9.46	2.45	10.36
ENSMUSG00000025746 Il6	interleukin 6 [Source:MGI Symbol;Acc:MGI:96559]	0.294425648	-1.764024742	0.015363565	0.043380519	yes	down	1.042	0.322	0.7	2.79	0.39	1.03	0.3	0.22	0.42	0.31	0.53	0.13
ENSMUSG00000040329 Il7	interleukin 7 [Source:MGI Symbol;Acc:MGI:96561]	2.271673921	1.183755763	1.23E-07	9.63E-07	yes	up	11.61	4.214	2.89	48.78	3.03	1.12	2.23	4.23	5.26	3.94	4.13	3.51
ENSMUSG00000003882 Il7r	interleukin 7 receptor [Source:MGI Symbol;Acc:MGI:96562]	2.104621287	1.073560653	1.01E-16	2.05E-15	yes	up	11.086	18.696	14.74	10.83	9.11	11.1	9.65	24.46	15.63	18.09	16.21	19.09
ENSMUSG00000020279 Il9r	interleukin 9 receptor [Source:MGI Symbol;Acc:MGI:96564]	2.905150634	1.53861297	9.30E-07	6.40E-06	yes	up	0.9	2.024	0.72	1.22	1.63	0.54	0.39	1.93	1.94	2.77	1.44	2.04

ENSMUSG00000035270	Impg2	interphotoreceptor matrix proteoglycan 2 [Source:MGI Symbol;Acc:MGI:3044955]	2.081274982	1.057467589	0.005290915	0.017254491	yes	up	0.134	0.238	0.06	0.12	0.2	0.16	0.13	0.29	0.26	0.27	0.22	0.15
ENSMUSG00000041324	Inhba	inhibin beta-A [Source:MGI Symbol;Acc:MGI:96570]	7.542242378	2.914993514	7.45E-12	9.49E-11	yes	up	0.77	5.926	0.31	1.19	1.33	0.54	0.48	7.29	4.57	4.95	4.8	8.02
ENSMUSG00000003477	Inmt	indolethylamine N-methyltransferase [Source:MGI Symbol;Acc:MGI:102963]	0.33621965	-1.572524053	1.74E-31	8.63E-30	yes	down	1915.15	545.348	2009.77	1721.61	1819.68	2359.91	1664.78	469.53	629.01	499.01	729.52	399.67
ENSMUSG00000050957	Insl6	insulin-like 6 [Source:MGI Symbol;Acc:MGI:1351595]	7.61713485	2.929248437	2.03E-15	3.63E-14	yes	up	1.428	9.102	1.54	0.73	3.17	0.61	1.09	9.14	8.78	7.77	8.97	10.85
ENSMUSG00000064065	Ipcef1	interaction protein for cytohesin exchange factors 1 [Source:MGI Symbol;Acc:MGI:2444159]	2.307854857	1.206552494	8.60E-15	1.46E-13	yes	up	2.038	3.796	1.46	2.33	2.55	2.31	1.54	4.53	3.38	3.76	3.25	4.06
ENSMUSG00000026301	Iqca	IQ motif containing with AAA domain [Source:MGI Symbol;Acc:MGI:1922168]	0.478107603	-1.064592748	5.39E-06	3.29E-05	yes	down	6.874	2.732	7.02	6.81	4.1	6.29	10.15	1.95	3.26	4.34	1.81	2.3
ENSMUSG00000029601	Iqcd	IQ motif containing D [Source:MGI Symbol;Acc:MGI:1922982]	0.228643126	-2.128830551	7.07E-10	7.29E-09	yes	down	2.124	0.402	1.53	2.3	2.09	2.09	2.61	0.26	0.46	0.51	0.28	0.5
ENSMUSG00000110622	Iqcn	IQ motif containing N [Source:MGI Symbol;Acc:MGI:3708784]	0.192190597	-2.379390339	8.97E-05	0.000439072	yes	down	1.166	0.208	2.12	0.16	1.15	1.2	1.2	0.23	0.13	0.41	0.11	0.16
ENSMUSG00000021676	Iqgap2	IQ motif containing GTPase activating protein 2 [Source:MGI Symbol;Acc:MGI:2449975]	2.34011254	1.226577913	7.22E-27	2.80E-25	yes	up	5.558	10.96	4.9	6.52	5.33	4.81	6.23	9.99	11.57	12.15	10.42	10.67
ENSMUSG00000028068	Iqgap3	IQ motif containing GTPase activating protein 3 [Source:MGI Symbol;Acc:MGI:3028642]	3.24504727	1.698239494	3.07E-07	2.27E-06	yes	up	0.68	1.924	0.48	0.46	1.3	0.47	0.69	1.7	2.06	1.24	1.67	2.95
ENSMUSG00000020227	Irak3	interleukin-1 receptor-associated kinase 3 [Source:MGI Symbol;Acc:MGI:1921164]	2.115791877	1.081197722	8.79E-22	2.47E-20	yes	up	6.17	10.71	4.86	6.4	7.52	5.75	6.32	10.27	10.67	10.03	11.02	11.56
ENSMUSG00000021356	Irf4	interferon regulatory factor 4 [Source:MGI Symbol;Acc:MGI:1096873]	3.057371596	1.61229191	5.45E-14	8.59E-13	yes	up	2.888	7.408	1.73	4.08	3.56	3.15	1.92	9.91	6.88	6.56	5.83	7.86
ENSMUSG00000029771	Irf5	interferon regulatory factor 5 [Source:MGI Symbol;Acc:MGI:1350924]	2.578775118	1.366685968	3.45E-33	1.88E-31	yes	up	15.824	33.098	14.6	17.77	17.95	15.52	13.28	30.92	30.2	35.87	28.23	40.27
ENSMUSG00000041515	Irf8	interferon regulatory factor 8 [Source:MGI Symbol;Acc:MGI:96395]	2.767646337	1.4686596	4.01E-51	4.07E-49	yes	up	26.458	61.728	25.32	27.31	29.92	26.34	23.4	56.57	59.99	59.85	57.89	74.34
ENSMUSG00000034664	Itga2b	integrin alpha 2b [Source:MGI Symbol;Acc:MGI:96601]	0.284898975	-1.811477665	5.98E-12	7.71E-11	yes	down	7.35	2.62	5.95	12.59	5.31	7.37	5.53	2.78	3.01	1.54	4.28	1.49
ENSMUSG00000070369	Itgad	integrin, alpha D [Source:MGI Symbol;Acc:MGI:3578624]	0.256222463	-1.964531135	7.35E-05	0.000365352	yes	down	0.654	0.196	0.41	0.71	1.13	0.49	0.53	0.1	0.53	0.08	0.15	0.12
ENSMUSG00000005947	Itgae	integrin alpha E, epithelial-associated [Source:MGI Symbol;Acc:MGI:1298377]	3.270327508	1.709435122	1.61E-22	4.80E-21	yes	up	3.458	9.586	2.54	3.56	4.22	2.45	4.52	8.27	9.24	10.42	8.96	11.04
ENSMUSG00000030830	Itgal	integrin alpha L [Source:MGI Symbol;Acc:MGI:96606]	3.035235712	1.601808558	1.74E-83	4.14E-81	yes	up	24.01	60.642	22.22	23.97	24.54	29.17	20.15	55.9	59.72	58.83	58.61	70.15
ENSMUSG00000030786	Itgam	integrin alpha M [Source:MGI Symbol;Acc:MGI:96607]	5.413610099	2.436590985	7.62E-68	1.30E-65	yes	up	8.39	38.228	7.36	10.84	7.14	8.65	7.96	39.39	34.2	30.57	37.34	49.64
ENSMUSG00000030789	Itgax	integrin alpha X [Source:MGI Symbol;Acc:MGI:96609]	8.322962606	3.057097155	4.70E-99	1.62E-96	yes	up	19.498	138.608	19.11	19.9	23.3	12.5	22.68	114.11	162.26	135.14	145.2	136.33
ENSMUSG00000031312	Itgb1bp2	integrin beta 1 binding protein 2 [Source:MGI Symbol;Acc:MGI:1353420]	0.335145746	-1.577139474	0.0107446	0.031906039	yes	down	1.478	0.426	0.8	2.86	1.06	1.25	1.42	0.1	0.68	1.02	0.24	0.09
ENSMUSG00000000290	Itgb2	integrin beta 2 [Source:MGI Symbol;Acc:MGI:96611]	6.611201682	2.724912526	2.89E-155	3.44E-152	yes	up	60.154	334.048	56.34	66.42	68.94	49.73	59.34	286.33	356.72	309.74	342.64	374.81

ENSMUSG00000022817 Itgb5	integrin beta 5 [Source:MGI Symbol;Acc:MGI:96614]	2.004585694	1.003304093	3.82E-63	5.49E-61	yes	up	76.04	128.828	77.3	72.07	78.48	73.94	78.41	118.11	137.11	124.83	131.68	132.41
ENSMUSG00000037254 Itih2	inter-alpha trypsin inhibitor, heavy chain 2 [Source:MGI Symbol;Acc:MGI:96619]	0.342969439	-1.543848066	5.34E-05	0.000270992	yes	down	1.736	0.5	0.96	1.29	1.03	1.66	3.74	0.4	0.18	0.74	0.93	0.25
ENSMUSG00000021922 Itih4	inter alpha-trypsin inhibitor, heavy chain 4 [Source:MGI Symbol;Acc:MGI:109536]	2.260339045	1.17653919	0.000474926	0.001999415	yes	up	40.094	76.454	32.06	69.37	38.17	21.7	39.17	64.42	85.01	84.95	71.48	76.41
ENSMUSG00000037973 Itprid1	ITPR interacting domain containing 1 [Source:MGI Symbol;Acc:MGI:2685304]	0.358673563	-1.479256683	4.07E-09	3.89E-08	yes	down	4.096	1.222	2.89	4.62	4.02	4.38	4.57	1.45	1.16	1.88	0.58	1.04
ENSMUSG00000049128 Ivl	involucrin [Source:MGI Symbol;Acc:MGI:96626]	0.236400553	-2.080694684	0.009737715	0.029347438	yes	down	0.628	0.126	0.54	0.67	0.44	1.03	0.46	0.03	0.36	0	0.24	0
ENSMUSG00000019762 Iyd	iodotyrosine deiodinase [Source:MGI Symbol;Acc:MGI:1917587]	0.245548691	-2.025918961	1.81E-13	2.71E-12	yes	down	8.302	1.704	13.81	8.91	5.19	6.82	6.78	1.16	3.63	1.89	0.84	1
ENSMUSG00000031933 Izumo1r	IZUMO1 receptor, JUNO [Source:MGI Symbol;Acc:MGI:1929185]	4.280752532	2.097864437	7.90E-07	5.50E-06	yes	up	1.258	3.276	2.28	0.63	1.4	0.9	1.08	5.11	2.37	2.77	2.26	3.87
ENSMUSG00000048534 Jaml	junction adhesion molecule like [Source:MGI Symbol;Acc:MGI:2685484]	2.081721405	1.057777007	4.96E-14	7.85E-13	yes	up	5.374	9.746	4.69	5.91	5.91	6.35	4.01	9.91	10.5	8.78	10.29	9.25
ENSMUSG00000067149 Jchain	immunoglobulin joining chain [Source:MGI Symbol;Acc:MGI:96493]	14.7546139	3.883094263	7.47E-22	2.11E-20	yes	up	292.966	3672.634	174.79	518.04	416.01	94.28	261.71	4775.53	3600.68	3657.05	2947.37	3382.54
ENSMUSG00000033182 Kbtbd12	kelch repeat and BTB (POZ) domain containing 12 [Source:MGI Symbol;Acc:MGI:1918481]	12.60835414	3.656308058	3.31E-05	0.00017471	yes	up	0.072	0.736	0	0.36	0	0	0	0.62	0.89	0.67	0.89	0.61
ENSMUSG00000075502 Kbtbd6	kelch repeat and BTB (POZ) domain containing 6 [Source:MGI Symbol;Acc:MGI:3643058]	5.946334335	2.572000582	2.49E-05	0.00013527	yes	up	0.07	0.334	0.02	0.12	0.05	0.06	0.1	0.47	0.36	0.18	0.23	0.43
ENSMUSG00000047976 Kcna1	potassium voltage-gated channel, shaker-related subfamily, member 1 [Source:MGI Symbol;Acc:MGI:96654]	0.177041074	-2.497843984	1.67E-05	9.37E-05	yes	down	0.468	0.078	0.09	0.86	0.64	0.4	0.35	0.07	0.09	0.11	0.03	0.09
ENSMUSG00000040724 Kcna2	potassium voltage-gated channel, shaker-related subfamily, member 2 [Source:MGI Symbol;Acc:MGI:96659]	0.212096109	-2.237209944	4.39E-54	4.89E-52	yes	down	3.778	0.666	3.03	3.39	4.11	4.31	4.05	0.73	0.8	0.72	0.55	0.53
ENSMUSG00000047959 Kcna3	potassium voltage-gated channel, shaker-related subfamily, member 3 [Source:MGI Symbol;Acc:MGI:96660]	2.76985327	1.469809553	9.55E-17	1.95E-15	yes	up	3.284	7.686	2.74	2.37	4.3	3.68	3.33	8.06	9.5	6.26	7.25	7.36
ENSMUSG00000045534 Kcna5	potassium voltage-gated channel, shaker-related subfamily, member 5 [Source:MGI Symbol;Acc:MGI:96662]	0.394434181	-1.342143517	0.011548002	0.033966765	yes	down	0.414	0.138	0.34	0.4	0.7	0.2	0.43	0.08	0.11	0.26	0.13	0.11
ENSMUSG00000028931 Kcnab2	potassium voltage-gated channel, shaker-related subfamily, beta member 2 [Source:MGI Symbol;Acc:MGI:109239]	2.435073332	1.283965219	1.08E-51	1.11E-49	yes	up	7.172	15.378	7.01	7.36	7.19	8.5	5.8	13.91	15.19	13.46	14.35	19.98
ENSMUSG00000009731 Kcnd1	potassium voltage-gated channel, Shal-related family, member 1 [Source:MGI Symbol;Acc:MGI:96671]	2.753378951	1.461203184	7.11E-05	0.00035413	yes	up	0.156	0.362	0.13	0.17	0.22	0.06	0.2	0.31	0.36	0.39	0.33	0.42
ENSMUSG00000040896 Kcnd3	potassium voltage-gated channel, Shal-related family, member 3 [Source:MGI Symbol;Acc:MGI:1928743]	0.420040156	-1.251400838	0.001885251	0.006902809	yes	down	0.592	0.26	0.66	0.61	0.34	0.57	0.78	0.16	0.27	0.6	0.19	0.08

ENSMUSG00000047330 Kcne4	potassium voltage-gated channel, Isk-related subfamily, gene 4 [Source:MGI Symbol;Acc:MGI:1891125]	0.389123552	-1.361699793	5.39E-10	5.64E-09	yes	down	14.802	4.868	12.82	24.54	11.33	14.49	10.83	4.06	6.21	5.32	3.17	5.58
ENSMUSG00000029088 Kcnip4	Kv channel interacting protein 4 [Source:MGI Symbol;Acc:MGI:1933131]	0.462478359	-1.112542236	0.009862644	0.029669671	yes	down	1.552	0.604	1.9	1.33	0.96	2.18	1.39	0.63	0.75	0.78	0.78	0.08
ENSMUSG00000044708 Kcnj10	potassium inwardly-rectifying channel, subfamily J, member 10 [Source:MGI Symbol;Acc:MGI:1194504]	11.60057522	3.536124439	3.50E-32	1.82E-30	yes	up	0.19	1.832	0.08	0.38	0.2	0.14	0.15	2.1	1.71	1.9	1.3	2.15
ENSMUSG00000038026 Kcnj9	potassium inwardly-rectifying channel, subfamily J, member 9 [Source:MGI Symbol;Acc:MGI:108007]	4.725951189	2.240604729	0.007831069	0.024330408	yes	up	0.054	0.144	0.03	0.06	0.15	0	0.03	0.21	0.17	0.11	0.19	0.04
ENSMUSG00000063142 Kcnma1	potassium large conductance calcium-activated channel, subfamily M, alpha member 1 [Source:MGI Symbol;Acc:MGI:99923]	0.339521569	-1.558424866	0.00115251	0.004444033	yes	down	0.35	0.112	0.3	0.41	0.31	0.36	0.37	0.06	0.06	0.16	0.04	0.24
ENSMUSG00000000794 Kcnn3	potassium intermediate/small conductance calcium-activated channel, subfamily N, member 3 [Source:MGI Symbol;Acc:MGI:2153183]	4.374152762	2.129003606	2.42E-14	3.92E-13	yes	up	4.432	17.082	4.08	4.17	6.11	1.95	5.85	13.9	21.74	15.84	18.7	15.23
ENSMUSG00000054342 Kcnn4	potassium intermediate/small conductance calcium-activated channel, subfamily N, member 4 [Source:MGI Symbol;Acc:MGI:1277957]	4.961406116	2.310749054	4.59E-44	3.72E-42	yes	up	4.026	17.56	3.69	4.54	3.57	3.65	4.68	21.07	17.69	17.18	12.01	19.85
ENSMUSG00000037653 Kctd8	potassium channel tetramerisation domain containing 8 [Source:MGI Symbol;Acc:MGI:2443804]	0.494007729	-1.01739448	0.000921448	0.003644273	yes	down	10.546	0.6	1.17	1.34	1.48	46.95	1.79	0.76	0.76	0.78	0.27	0.43
ENSMUSG00000012443 Kif11	kinesin family member 11 [Source:MGI Symbol;Acc:MGI:1098231]	2.766514936	1.468069713	2.65E-15	4.69E-14	yes	up	1.676	3.9	1.98	1.55	1.84	1.53	1.48	4.59	3.68	3.53	2.56	5.14
ENSMUSG00000051378 Kif18b	kinesin family member 18B [Source:MGI Symbol;Acc:MGI:2446979]	3.165149441	1.662273617	8.52E-07	5.90E-06	yes	up	0.468	1.25	0.47	0.32	0.79	0.27	0.49	1.05	1.38	1.1	0.79	1.93
ENSMUSG00000003779 Kif20a	kinesin family member 20A [Source:MGI Symbol;Acc:MGI:1201682]	3.135679979	1.648778329	7.59E-12	9.67E-11	yes	up	1.662	4.158	2.07	1.15	2.27	1.24	1.58	4.55	3.97	2.85	3	6.42
ENSMUSG00000022629 Kif21a	kinesin family member 21A [Source:MGI Symbol;Acc:MGI:109188]	0.496940029	-1.008856339	9.49E-12	1.19E-10	yes	down	10.158	5.49	9.44	13.64	8.7	8.58	10.43	5.03	4.39	7.22	5.17	5.64
ENSMUSG00000030677 Kif22	kinesin family member 22 [Source:MGI Symbol;Acc:MGI:109233]	2.278402318	1.188022519	2.20E-05	0.000120661	yes	up	2.338	4.796	2.54	1.48	4.03	2.02	1.62	5.72	4.67	3.62	2.77	7.2
ENSMUSG00000060176 Kif27	kinesin family member 27 [Source:MGI Symbol;Acc:MGI:1922300]	0.462564261	-1.112274291	2.77E-07	2.07E-06	yes	down	5.224	2.942	6.33	4.05	4.01	5.11	6.62	3.5	3.51	3.47	1.22	3.01
ENSMUSG00000028678 Kif2c	kinesin family member 2C [Source:MGI Symbol;Acc:MGI:1921054]	4.451816544	2.154394142	4.26E-14	6.79E-13	yes	up	0.61	2.286	0.58	0.59	0.87	0.38	0.63	2.53	2.2	1.99	1.42	3.29
ENSMUSG00000074657 Kif5a	kinesin family member 5A [Source:MGI Symbol;Acc:MGI:109564]	2.461476678	1.299524071	6.60E-05	0.000330867	yes	up	0.21	0.43	0.28	0.25	0.2	0.19	0.13	0.29	0.5	0.52	0.28	0.56
ENSMUSG00000026764 Kif5c	kinesin family member 5C [Source:MGI Symbol;Acc:MGI:1098269]	2.18652225	1.12863803	4.19E-05	0.000217383	yes	up	0.588	0.766	0.85	0.34	0.51	0.44	0.8	0.94	0.51	0.77	0.77	0.84
ENSMUSG00000023999 Kif6	kinesin family member 6 [Source:MGI Symbol;Acc:MGI:1098238]	0.420731967	-1.249026658	0.00023674	0.001067372	yes	down	2.088	0.71	2.59	2.58	1.1	2.02	2.15	0.66	0.48	1.37	0.3	0.74
ENSMUSG00000079553 Kifc1	kinesin family member C1 [Source:MGI Symbol;Acc:MGI:109596]	2.851231483	1.511585172	3.71E-09	3.56E-08	yes	up	1.378	3.23	1.6	0.94	1.9	1.17	1.28	3.61	3.21	2.64	2.22	4.47
ENSMUSG00000024301 Kifc5b	kinesin family member C5B [Source:MGI Symbol;Acc:MGI:2137414]	2.044255839	1.031575761	0.003328686	0.011409595	yes	up	0.754	2.594	0.67	0.66	1.16	0.54	0.74	2.33	1.73	1.05	0.66	7.2
ENSMUSG00000029195 Klb	klotho beta [Source:MGI Symbol;Acc:MGI:1932466]	0.121527077	-3.040650308	0.003459688	0.011811742	yes	down	0.27	0.026	0.02	0.5	0.61	0.03	0.19	0.02	0.01	0.07	0	0.03
ENSMUSG00000030087 Klf15	Kruppel-like factor 15 [Source:MGI Symbol;Acc:MGI:1929988]	0.313988153	-1.671217969	1.02E-61	1.39E-59	yes	down	47.632	12.578	46.37	43.99	44.74	56.4	46.66	12.62	12.38	14.91	11.07	11.91
ENSMUSG00000003032 Klf4	Kruppel-like factor 4 (gut) [Source:MGI Symbol;Acc:MGI:1342287]	0.422952889	-1.241431117	1.30E-17	2.80E-16	yes	down	107.59	38.136	103.71	149.35	81.3	120.18	83.41	42.71	38.96	34.68	40.15	34.18

ENSMUSG00000033863	Klf9	Kruppel-like factor 9 [Source:MGI Symbol;Acc:MGI:1333856]	0.442258829	-1.177037151	8.97E-40	6.18E-38	yes	down	101.52	37.914	105.57	107.2	91.64	106.3	96.89	38.54	37.06	43.58	38.84	31.55
ENSMUSG00000040263	Klhdc4	kelch domain containing 4 [Source:MGI Symbol;Acc:MGI:2384569]	2.138527678	1.09661788	4.46E-29	1.98E-27	yes	up	31.904	73.522	28.44	34.32	38.09	26.65	32.02	55.9	73.45	75.52	81.03	81.71
ENSMUSG00000036782	Klhl13	kelch-like 13 [Source:MGI Symbol;Acc:MGI:1914705]	0.456755037	-1.130507455	8.93E-14	1.38E-12	yes	down	13.092	5.422	14.02	13.11	11.98	13.69	12.66	5.47	6.87	5.49	5.4	3.88
ENSMUSG00000043008	Klhl6	kelch-like 6 [Source:MGI Symbol;Acc:MGI:2686922]	2.589792404	1.372836457	1.08E-43	8.62E-42	yes	up	9.062	20.526	8.73	8.76	8.41	10.15	9.26	22.59	20.6	19.84	18.24	21.36
ENSMUSG00000030187	Klra2	killer cell lectin-like receptor, subfamily A, member 2 [Source:MGI Symbol;Acc:MGI:101906]	2.905392605	1.538733128	1.06E-20	2.82E-19	yes	up	9.178	23.306	8.98	12.21	10.33	7.34	7.03	25.03	20.75	21.82	21.46	27.47
ENSMUSG00000079852	Klra4	killer cell lectin-like receptor, subfamily A, member 4 [Source:MGI Symbol;Acc:MGI:101904]	0.406749437	-1.297787745	0.000171714	0.000793746	yes	down	2.96	1.02	2.9	2.9	1.95	4.51	2.54	1.46	1.16	0.69	0.55	1.24
ENSMUSG00000089727	Klra8	killer cell lectin-like receptor, subfamily A, member 8 [Source:MGI Symbol;Acc:MGI:102968]	0.496690457	-1.009581066	0.000158511	0.000738452	yes	down	4.308	1.658	4.04	3.68	6.19	4.77	2.86	1.69	1.28	1.23	1.94	2.15
ENSMUSG00000033024	Klra9	killer cell lectin-like receptor subfamily A, member 9 [Source:MGI Symbol;Acc:MGI:1321153]	0.495301952	-1.013619787	0.005039794	0.016518552	yes	down	2.744	1.178	3.3	3.51	3.08	1.87	1.96	1.77	1.66	0.98	0.47	1.01
ENSMUSG00000030325	Klrb1c	killer cell lectin-like receptor subfamily B member 1C [Source:MGI Symbol;Acc:MGI:107538]	0.358945383	-1.478163755	3.04E-16	5.85E-15	yes	down	8.55	2.07	8.73	13.3	7.62	6.93	6.17	1.93	1.85	2.1	2.74	1.73
ENSMUSG00000027326	Knl1	kinetochore scaffold 1 [Source:MGI Symbol;Acc:MGI:1923714]	3.684481689	1.881461684	7.11E-14	1.11E-12	yes	up	0.36	1.154	0.29	0.41	0.38	0.26	0.46	1.43	0.87	0.72	1.27	1.48
ENSMUSG00000027331	Knstrn	kinetochore-localized astrin/SPAG5 binding [Source:MGI Symbol;Acc:MGI:1289298]	2.442535852	1.288379739	4.36E-08	3.64E-07	yes	up	3.67	25.796	3.37	2.25	7.06	2.96	2.71	27.95	68.35	7.18	7	18.5
ENSMUSG00000029414	Kntc1	kinetochore associated 1 [Source:MGI Symbol;Acc:MGI:2673709]	2.043664226	1.031158181	0.000348432	0.001512061	yes	up	1.5	2.162	0.4	5.03	1.27	0.34	0.46	2.63	1.53	3.87	1.15	1.63
ENSMUSG00000035775	Krt20	keratin 20 [Source:MGI Symbol;Acc:MGI:1914059]	15.24499003	3.930263302	0.004613246	0.01527714	yes	up	0.014	0.18	0	0	0.04	0.03	0	0.3	0.03	0.06	0.06	0.45
ENSMUSG00000050463	Krt78	keratin 78 [Source:MGI Symbol;Acc:MGI:1917529]	10.92294398	3.449289843	1.18E-08	1.07E-07	yes	up	0.048	0.448	0.04	0.02	0.04	0.08	0.06	0.2	0.46	0.82	0.31	0.45
ENSMUSG00000061397	Krt79	keratin 79 [Source:MGI Symbol;Acc:MGI:2385030]	2.898494808	1.535303901	0.000388066	0.001664829	yes	up	13.24	35.832	8.98	7.8	16.43	5.52	27.47	16.41	54.5	40.38	38.03	29.84
ENSMUSG00000037185	Krt80	keratin 80 [Source:MGI Symbol;Acc:MGI:1921377]	0.492503346	-1.02179457	1.20E-18	2.81E-17	yes	down	146.002	60.908	175.51	120.17	142.92	136.87	154.54	68.06	63.31	49.32	68.16	55.69
ENSMUSG00000067613	Krt83	keratin 83 [Source:MGI Symbol;Acc:MGI:3690448]	3.36050231	1.748676896	0.006778675	0.021487876	yes	up	0.366	0.948	0.4	0.8	0.4	0.2	0.03	1.23	0.42	1.23	0.33	1.53
ENSMUSG00000061578	Ksr2	kinase suppressor of ras 2 [Source:MGI Symbol;Acc:MGI:3610315]	11.52843156	3.527124344	1.29E-16	2.58E-15	yes	up	0.032	0.274	0.06	0	0.04	0.02	0.04	0.3	0.31	0.17	0.37	0.22
ENSMUSG00000026866	Kynu	kynureninase [Source:MGI Symbol;Acc:MGI:1918039]	9.353547755	3.225513676	3.49E-87	9.34E-85	yes	up	2.308	18.164	1.78	2.95	2.72	1.8	2.29	16.21	18.45	15.61	18.65	21.9
ENSMUSG00000044350	Lacc1	laccase domain containing 1 [Source:MGI Symbol;Acc:MGI:2445077]	2.218873475	1.149827404	3.64E-17	7.64E-16	yes	up	4.542	8.52	4.9	4.59	4.9	4.89	3.43	7.66	9.08	7.68	7.83	10.35
ENSMUSG00000030124	Lag3	lymphocyte-activation gene 3 [Source:MGI Symbol;Acc:MGI:106588]	6.199616046	2.632178869	1.82E-14	2.99E-13	yes	up	0.852	4.424	0.62	1.03	1.37	0.48	0.76	5.7	2.62	3.7	2.84	7.26
ENSMUSG00000055541	Lair1	leukocyte-associated Ig-like receptor 1 [Source:MGI Symbol;Acc:MGI:105492]	5.17063168	2.370340541	8.95E-76	1.86E-73	yes	up	6.614	28.418	6.86	8.13	6.03	4.94	7.11	30.77	28.53	22.25	22.8	37.74
ENSMUSG00000032796	Lama1	laminin, alpha 1 [Source:MGI Symbol;Acc:MGI:99892]	2.164182613	1.113822239	5.73E-07	4.07E-06	yes	up	0.878	1.998	0.86	1.16	0.94	0.48	0.95	1.84	3.53	0.74	1.91	1.97
ENSMUSG00000028581	Laptm5	lysosomal-associated protein transmembrane 5 [Source:MGI	4.28459776	2.09915977	2.17E-143	1.86E-140	yes	up	100.232	358.218	99.03	104.63	103.93	89.18	104.39	323.45	392.35	327.43	365.14	382.72

		Symbol;Acc:MGI:108046]																		
ENSMUSG00000040751	Lat2	linker for activation of T cells family, member 2 [Source:MGI Symbol;Acc:MGI:1926479]	3.556053267	1.830276935	5.13E-21	1.39E-19	yes	up	9.714	25.72	10.09	10.26	12.09	8.36	7.77	31.37	20.71	24.51	20.95	31.06
ENSMUSG00000051998	Lax1	lymphocyte transmembrane adaptor 1 [Source:MGI Symbol;Acc:MGI:2443362]	4.479633066	2.163380564	1.01E-13	1.56E-12	yes	up	5.416	18.604	5.7	5.38	6.71	5.67	3.62	29.07	15.6	18.71	9.94	19.7
ENSMUSG00000026822	Lcn2	lipocalin 2 [Source:MGI Symbol;Acc:MGI:96757]	7.70129962	2.945101926	1.94E-26	7.34E-25	yes	up	142.366	929.328	106.56	223.94	147.68	81.95	151.7	851.9	848.84	1022.01	765.53	1158.36
ENSMUSG00000021998	Lcp1	lymphocyte cytosolic protein 1 [Source:MGI Symbol;Acc:MGI:104808]	2.494767719	1.318905497	9.61E-65	1.48E-62	yes	up	124.68	260.706	123.35	136.67	135.05	117.6	110.73	267.44	248.37	250.03	254.49	283.2
ENSMUSG00000002699	Lcp2	lymphocyte cytosolic protein 2 [Source:MGI Symbol;Acc:MGI:1321402]	3.289997181	1.718086348	6.44E-60	8.46E-58	yes	up	8.6	25.104	9.17	8.56	9.01	8.33	7.93	28.57	25.1	23.49	19.66	28.7
ENSMUSG00000070877	Ldlrad1	low density lipoprotein receptor class A domain containing 1 [Source:MGI Symbol;Acc:MGI:3652166]	0.477580519	-1.066184106	1.45E-07	1.12E-06	yes	down	13.402	5.424	12.07	14.93	10.69	13.11	16.21	4.3	5.58	8.57	3.35	5.32
ENSMUSG00000024972	Lgals12	lectin, galactose binding, soluble 12 [Source:MGI Symbol;Acc:MGI:1929094]	0.290303424	-1.784366506	3.93E-05	0.000204372	yes	down	1.78	0.43	1.07	2.5	2.83	1.66	0.84	0.25	0.26	0.86	0.2	0.58
ENSMUSG00000043501	Lgals2	lectin, galactose-binding, soluble 2 [Source:MGI Symbol;Acc:MGI:895068]	0.428383203	-1.223026183	0.002285904	0.008193292	yes	down	3.812	1.638	6.9	3.56	3.06	4	1.54	0.53	0.99	2.27	3.73	0.67
ENSMUSG00000050335	Lgals3	lectin, galactose binding, soluble 3 [Source:MGI Symbol;Acc:MGI:96778]	4.717638434	2.238064853	1.94E-70	3.61E-68	yes	up	126.76	509.81	123.26	155.49	119.28	93.03	142.74	470	510.32	481.01	525.4	562.32
ENSMUSG00000033880	Lgals3bp	lectin, galactoside-binding, soluble, 3 binding protein [Source:MGI Symbol;Acc:MGI:99554]	3.180670267	1.669330819	6.68E-48	6.17E-46	yes	up	82.292	224.588	87.18	79.71	76.85	76.15	91.57	205.57	214.55	225.39	190.72	286.71
ENSMUSG00000021190	Lgmn	legumain [Source:MGI Symbol;Acc:MGI:1330838]	2.464727798	1.301428326	1.66E-60	2.21E-58	yes	up	98.886	206.14	98.3	104.06	103.67	92.39	96.01	178.19	218.1	190.94	209.75	233.72
ENSMUSG00000045312	Lhfpl2	lipoma HMGIC fusion partner-like 2 [Source:MGI Symbol;Acc:MGI:2145236]	12.21979443	3.61114811	1.05E-147	9.76E-145	yes	up	3.498	36.656	3.45	4.66	3.83	2.65	2.9	41.24	33.7	31.44	40.61	36.29
ENSMUSG00000112023	Lilr4b	leukocyte immunoglobulin-like receptor, subfamily B, member 4B [Source:MGI Symbol;Acc:MGI:102702]	4.204907713	2.072074137	9.92E-29	4.35E-27	yes	up	7.182	27.792	7.12	8.13	6.37	8.25	6.04	36.64	30.94	21.63	27.24	22.51
ENSMUSG00000070873	Lilra5	leukocyte immunoglobulin-like receptor, subfamily A (with TM domain), member 5 [Source:MGI Symbol;Acc:MGI:3647196]	4.037578701	2.013490381	1.31E-21	3.63E-20	yes	up	7.708	26.338	7.56	8.11	10.39	3.45	9.03	20.27	30.66	27.46	28.2	25.1
ENSMUSG00000112148	Lilrb4a	leukocyte immunoglobulin-like receptor, subfamily B, member 4A [Source:MGI Symbol;Acc:MGI:102701]	5.32843215	2.413711094	3.31E-19	8.03E-18	yes	up	13.132	61.83	14.55	16.62	12.04	16.37	6.08	70.8	75.02	51.09	49.18	63.06
ENSMUSG00000037736	Limch1	LIM and calponin homology domains 1 [Source:MGI Symbol;Acc:MGI:1924819]	0.474635085	-1.075109347	2.94E-41	2.10E-39	yes	down	282.202	118.522	291.16	251.09	286.24	316.11	266.41	105.02	134.36	108.9	134.03	110.3
ENSMUSG00000024395	Lims2	LIM and senescent cell antigen like domains 2 [Source:MGI Symbol;Acc:MGI:2385067]	0.446615627	-1.162894363	1.67E-24	5.58E-23	yes	down	93.876	33.302	107.06	76.24	82.27	94.66	109.15	34.24	36.69	27.85	35.88	31.85

ENSMUSG00000024781 Lipa	lysosomal acid lipase A [Source:MGI Symbol;Acc:MGI:96789]	3.770163852	1.914627225	4.96E-114	2.47E-111	yes	up	47.778	152.884	49.02	47.41	52.03	42.79	47.64	132.97	170.96	139.76	170.34	150.39
ENSMUSG00000032207 Lipc	lipase, hepatic [Source:MGI Symbol;Acc:MGI:96216]	4.090645456	2.032328501	0.016377027	0.045817167	yes	up	0.086	0.49	0	0.04	0.04	0.21	0.14	0.24	0.16	0.16	0.41	1.48
ENSMUSG00000024768 Lipf	lipase, gastric [Source:MGI Symbol;Acc:MGI:1914967]	3.899630293	1.963337355	4.75E-06	2.92E-05	yes	up	0.788	2.676	1.07	0.5	1.15	0.1	1.12	2.28	3.16	2.22	3.01	2.71
ENSMUSG00000053846 Lipg	lipase, endothelial [Source:MGI Symbol;Acc:MGI:1341803]	0.45973181	-1.121135602	2.13E-27	8.47E-26	yes	down	37.294	14.428	40.55	31.05	35.79	41.52	37.56	15.5	17.35	12.97	13.88	12.44
ENSMUSG00000024770 Lipn	lipase, family member N [Source:MGI Symbol;Acc:MGI:1917416]	107.1682637	6.743733926	2.13E-11	2.58E-10	yes	up	0.036	1.868	0	0	0.18	0	0	1.78	1.72	2.34	2.06	1.44
ENSMUSG00000054966 Lmntd1	lamin tail domain containing 1 [Source:MGI Symbol;Acc:MGI:1921321]	0.198708149	-2.331277053	6.40E-11	7.35E-10	yes	down	2.694	0.574	2.1	4.04	1.38	3.49	2.46	0.42	0.58	0.34	1.1	0.43
ENSMUSG00000116305 Lncppara	long noncoding RNA near Ppara [Source:MGI Symbol;Acc:MGI:5568843]	0.385065188	-1.376825394	0.00906954	0.027613486	yes	down	5.366	5.79	1.22	16.33	6.38	1.96	0.94	0.26	0.59	27.72	0.09	0.29
ENSMUSG00000029228 Lnx1	ligand of numb-protein X 1 [Source:MGI Symbol;Acc:MGI:1278335]	0.418254625	-1.2575466	4.40E-06	2.73E-05	yes	down	1.876	8.928	1.89	2.04	1.68	2.1	1.67	42.13	0.66	0.6	0.39	0.86
ENSMUSG00000098318 Lockd	lncRNA downstream of Cdkn1b [Source:MGI Symbol;Acc:MGI:1915081]	2.595553935	1.376042467	0.001063796	0.004142228	yes	up	0.452	0.254	1.15	0.06	0.78	0.13	0.14	0.38	0.16	0.25	0.19	0.29
ENSMUSG00000049929 Lpar4	lysophosphatidic acid receptor 4 [Source:MGI Symbol;Acc:MGI:1925384]	0.301449916	-1.730009765	2.52E-06	1.63E-05	yes	down	0.8	0.206	0.72	0.78	1.25	0.67	0.58	0.15	0.37	0.16	0.24	0.11
ENSMUSG00000067714 Lpar5	lysophosphatidic acid receptor 5 [Source:MGI Symbol;Acc:MGI:2685918]	4.835351188	2.273620675	9.44E-32	4.77E-30	yes	up	1.464	6.088	0.77	1.87	2.8	1.07	0.81	7.1	5.38	5.65	7.29	5.02
ENSMUSG00000033192 Lpcat2	lysophosphatidylcholine acyltransferase 2 [Source:MGI Symbol;Acc:MGI:3606214]	6.504584626	2.701456931	7.99E-120	4.63E-117	yes	up	4.418	23.48	4.25	5.33	4.14	4.76	3.61	21.97	24.5	23.05	21.13	26.75
ENSMUSG00000015568 Lpl	lipoprotein lipase [Source:MGI Symbol;Acc:MGI:96820]	2.952575703	1.56197405	1.09E-25	3.92E-24	yes	up	103.568	263.878	101.91	109.66	137.19	66.73	102.35	205.93	287.98	295.37	280.15	249.96
ENSMUSG00000024696 Lpxn	leupaxin [Source:MGI Symbol;Acc:MGI:2147677]	3.610546692	1.8522173	7.41E-28	3.05E-26	yes	up	6.184	18.744	5.53	6.52	7.56	6.17	5.14	24.29	14.36	18.89	15.34	20.84
ENSMUSG00000028003 Lrat	lecithin-retinol acyltransferase (phosphatidylcholine-retinol-O-acyltransferase) [Source:MGI Symbol;Acc:MGI:1891259]	0.232185233	-2.106651876	4.10E-25	1.43E-23	yes	down	13.182	2.588	18.03	11.91	10.09	15.5	10.38	1.99	3.61	1.9	3.57	1.87
ENSMUSG00000037095 Lrg1	leucine-rich alpha-2-glycoprotein 1 [Source:MGI Symbol;Acc:MGI:1924155]	3.029093039	1.598885891	1.81E-05	0.000100741	yes	up	43.354	112.086	22.25	71.48	41.06	20.61	61.37	97.08	131.32	122.22	77.89	131.92
ENSMUSG00000022305 Lrp12	low density lipoprotein-related protein 12 [Source:MGI Symbol;Acc:MGI:2443132]	5.012104128	2.325416387	3.01E-86	7.78E-84	yes	up	6.186	26.36	6.03	6.45	6.8	4.74	6.91	26.73	26.1	26.07	29.62	23.28
ENSMUSG00000090291 Lrrc10b	leucine rich repeat containing 10B [Source:MGI Symbol;Acc:MGI:2685551]	0.483327811	-1.048926083	0.000378736	0.001629046	yes	down	8.294	3.348	6.92	8.55	5.83	8.53	11.64	2.55	2.55	6.28	1.62	3.74
ENSMUSG00000039883 Lrrc17	leucine rich repeat containing 17 [Source:MGI Symbol;Acc:MGI:1921761]	0.315312715	-1.665144745	4.30E-08	3.60E-07	yes	down	4.35	1.152	3.1	6.53	3.78	6	2.34	0.63	0.96	1.36	1.42	1.39
ENSMUSG00000030125 Lrrc23	leucine rich repeat containing 23 [Source:MGI	0.458380437	-1.12538262	2.03E-08	1.78E-07	yes	down	38.948	13.736	38.31	50.78	28.73	39.74	37.18	8.54	10.97	22.4	10.17	16.6

	Symbol;Acc:MGI:1315192]																	
ENSMUSG00000049988 Lrrc25	leucine rich repeat containing 25 [Source:MGI Symbol;Acc:MGI:2445284]	3.61902612	1.85560152	2.94E-27	1.16E-25	yes	up	3.278	9.772	2.33	4.63	3.65	3.36	2.42	11.04	9.45	8.82	9.06	10.49
ENSMUSG00000026961 Lrrc26	leucine rich repeat containing 26 [Source:MGI Symbol;Acc:MGI:2385129]	0.369007521	-1.438277874	1.71E-08	1.52E-07	yes	down	12.338	3.854	10.91	11.65	9.24	15.66	14.23	1.91	4.93	5.78	2.9	3.75
ENSMUSG00000027961 Lrrc39	leucine rich repeat containing 39 [Source:MGI Symbol;Acc:MGI:1924557]	0.452394534	-1.144346598	0.016193123	0.045410241	yes	down	1.35	0.59	0.71	1.79	1.48	1.53	1.24	0.14	0.92	0.24	0.54	1.11
ENSMUSG00000050587 Lrrc4c	leucine rich repeat containing 4C [Source:MGI Symbol;Acc:MGI:2442636]	0.467419771	-1.097209337	0.000687581	0.002789926	yes	down	1.01	0.838	1.27	1.06	0.49	1.12	1.11	0.21	0.46	0.41	1.5	1.61
ENSMUSG00000040485 Lrrc52	leucine rich repeat containing 52 [Source:MGI Symbol;Acc:MGI:1924118]	0.081678659	-3.61389701	0.016065697	0.045105264	yes	down	0.216	0.01	0.21	0.07	0.21	0.12	0.47	0	0	0	0	0.05
ENSMUSG00000022375 Lrrc6	leucine rich repeat containing 6 (testis) [Source:MGI Symbol;Acc:MGI:1859553]	0.490858369	-1.026621283	0.000509572	0.002130194	yes	down	4.002	1.67	4.6	4.12	2.08	3.9	5.31	2.02	1.17	2.68	1.12	1.36
ENSMUSG00000022759 Lrrc74b	leucine rich repeat containing 74B [Source:MGI Symbol;Acc:MGI:1921935]	0.416882893	-1.262285923	1.76E-09	1.74E-08	yes	down	9.132	3.01	9.73	11.58	7.14	7.17	10.04	3.13	2.45	4.34	1.77	3.36
ENSMUSG00000021090 Lrrc9	leucine rich repeat containing 9 [Source:MGI Symbol;Acc:MGI:1925507]	0.452837614	-1.142934297	0.005060625	0.016579217	yes	down	0.63	0.26	0.68	0.7	0.36	0.61	0.8	0.43	0.17	0.2	0.11	0.39
ENSMUSG00000019892 Lrriq1	leucine-rich repeats and IQ motif containing 1 [Source:MGI Symbol;Acc:MGI:1922228]	0.406074253	-1.300184537	9.26E-11	1.04E-09	yes	down	1.83	0.594	1.16	1.74	1.46	3.15	1.64	0.45	0.44	0.87	0.81	0.4
ENSMUSG00000043110 Lrrn4	leucine rich repeat neuronal 4 [Source:MGI Symbol;Acc:MGI:2445154]	0.351736405	-1.507433432	9.44E-06	5.51E-05	yes	down	8.724	2.59	9.03	7.79	8.45	9.68	8.67	2.42	2.11	2.38	4.95	1.09
ENSMUSG00000055003 Lrtm2	leucine-rich repeats and transmembrane domains 2 [Source:MGI Symbol;Acc:MGI:2141485]	0.427904856	-1.224638043	1.99E-06	1.30E-05	yes	down	5.388	1.88	5.63	5.33	4.16	7.84	3.98	1.84	1.83	0.93	3.12	1.68
ENSMUSG00000061080 Lsamp	limbic system-associated membrane protein [Source:MGI Symbol;Acc:MGI:1261760]	0.2567119	-1.961777918	0.011131061	0.032898537	yes	down	0.136	0.064	0.1	0.18	0.12	0.14	0.14	0	0.03	0.25	0	0.04
ENSMUSG00000073412 Lst1	leukocyte specific transcript 1 [Source:MGI Symbol;Acc:MGI:1096324]	2.247281603	1.168180918	4.63E-05	0.000237794	yes	up	22.57	43.006	19.42	26.93	23.36	26.54	16.6	51.1	30.57	45.74	22.63	64.99
ENSMUSG00000024402 Lta	lymphotoxin A [Source:MGI Symbol;Acc:MGI:104797]	4.164615227	2.05818321	3.44E-05	0.000181105	yes	up	0.408	1.41	0.22	0.55	0.66	0.47	0.14	2.07	0.71	0.91	1.71	1.65
ENSMUSG00000024399 Ltb	lymphotoxin B [Source:MGI Symbol;Acc:MGI:104796]	2.526720788	1.33726625	8.96E-11	1.01E-09	yes	up	15.714	33.37	11.23	15.96	19.04	17.85	14.49	45.18	19.88	37.22	26.29	38.28
ENSMUSG00000002020 Ltbp2	latent transforming growth factor beta binding protein 2 [Source:MGI Symbol;Acc:MGI:99502]	2.926020046	1.548939653	3.14E-35	1.84E-33	yes	up	29.922	71.694	31.08	22.72	32.59	31.65	31.57	65.57	75.39	60.27	63.49	93.75
ENSMUSG00000040488 Ltbp4	latent transforming growth factor beta binding protein 4 [Source:MGI Symbol;Acc:MGI:1321395]	0.398913125	-1.325853503	5.28E-27	2.06E-25	yes	down	224.4	83.574	228.99	187.91	209.03	261.99	234.08	59.47	105.94	78.8	99.26	74.4

ENSMUSG00000020377 Ltc4s	leukotriene C4 synthase [Source:MGI Symbol;Acc:MGI:107498]	2.553965945	1.352739288	1.53E-13	2.32E-12	yes	up	21.29	46.586	19.8	21.51	19.97	17.18	27.99	32.11	54.79	44.96	60.72	40.35
ENSMUSG00000022584 Ly6c2	lymphocyte antigen 6 complex, locus C2 [Source:MGI Symbol;Acc:MGI:3712069]	3.872471535	1.953254634	1.86E-42	1.41E-40	yes	up	59.168	230.608	41.63	82.56	72.17	53.2	46.28	278.92	206.6	186.03	259.03	222.46
ENSMUSG00000092586 Ly6g6c	lymphocyte antigen 6 complex, locus G6C [Source:MGI Symbol;Acc:MGI:2148930]	0.296800322	-1.752435437	0.001160535	0.004470951	yes	down	2.234	0.62	1.31	3.66	0.9	3.8	1.5	0.66	0.8	0.99	0.44	0.21
ENSMUSG00000022586 Ly6i	lymphocyte antigen 6 complex, locus I [Source:MGI Symbol;Acc:MGI:1888480]	12.86667744	3.685567649	4.44E-21	1.20E-19	yes	up	6.742	99.394	8.63	5.88	6.66	4.41	8.13	102.77	59.85	110.62	48.07	175.66
ENSMUSG00000044678 Ly6k	lymphocyte antigen 6 complex, locus K [Source:MGI Symbol;Acc:MGI:1923736]	2.430374903	1.281178877	0.014592166	0.041473899	yes	up	1.36	2.88	0.58	3.28	1.32	0.31	1.31	2.8	4.2	2.43	2.95	2.02
ENSMUSG00000026980 Ly75	lymphocyte antigen 75 [Source:MGI Symbol;Acc:MGI:106662]	3.749883718	1.906845859	1.47E-58	1.87E-56	yes	up	5.838	18.566	5.79	6.61	6.59	4.34	5.86	15.61	18.9	17.64	19.56	21.12
ENSMUSG00000021423 Ly86	lymphocyte antigen 86 [Source:MGI Symbol;Acc:MGI:1321404]	4.918624575	2.298254942	2.65E-70	4.89E-68	yes	up	17.06	70.478	15.34	17.96	21.7	17.37	12.93	80.72	63.38	71.41	63.2	73.68
ENSMUSG00000004707 Ly9	lymphocyte antigen 9 [Source:MGI Symbol;Acc:MGI:96885]	5.840488563	2.546089057	3.36E-110	1.53E-107	yes	up	6.944	33.868	7.61	6.29	7.9	6.68	6.24	36.97	29.68	30.49	34.29	37.91
ENSMUSG00000025779 Ly96	lymphocyte antigen 96 [Source:MGI Symbol;Acc:MGI:1341909]	2.348680189	1.231850281	8.13E-08	6.54E-07	yes	up	11.416	21.894	7.86	16.71	13.32	9.47	9.72	27.43	19.08	23.75	21.8	17.41
ENSMUSG00000030787 Lyve1	lymphatic vessel endothelial hyaluronan receptor 1 [Source:MGI Symbol;Acc:MGI:2136348]	0.368497351	-1.440273846	7.85E-09	7.25E-08	yes	down	434.258	137.76	529.09	336.1	440.43	372.08	493.59	151.38	165.69	108.79	193.11	69.83
ENSMUSG00000069516 Lyz2	lysozyme 2 [Source:MGI Symbol;Acc:MGI:96897]	2.844937137	1.508396774	5.44E-36	3.26E-34	yes	up	7857.876	18964.116	7402.83	8679.42	8282.64	6053.36	8871.13	15301.67	18827.92	18588.12	22778.26	19324.61
ENSMUSG00000044313 Mab21l3	mab-21-like 3 (C. elegans) [Source:MGI Symbol;Acc:MGI:2446273]	8.52480141	3.091666226	1.08E-10	1.21E-09	yes	up	0.144	0.824	0.15	0.15	0.23	0.04	0.15	0.94	0.92	0.91	0.29	1.06
ENSMUSG00000074622 Mafb	v-maf musculoaponeurotic fibrosarcoma oncogene family, protein B (avian) [Source:MGI Symbol;Acc:MGI:104555]	3.884405072	1.957693655	1.06E-46	9.43E-45	yes	up	5.452	17.932	5.35	5.51	5.49	4.14	6.77	18.99	17.31	19.33	15.03	19
ENSMUSG00000036634 Mag	myelin-associated glycoprotein [Source:MGI Symbol;Acc:MGI:96912]	4.633025918	2.211954753	7.01E-30	3.22E-28	yes	up	0.886	3.458	0.97	0.97	0.93	0.84	0.72	3.2	3.35	3.08	3.6	4.06
ENSMUSG00000032688 Malt1	MALT1 paracaspase [Source:MGI Symbol;Acc:MGI:2445027]	2.400988751	1.263628644	5.79E-45	4.88E-43	yes	up	12.696	24.372	12.49	14.91	12.22	13.12	10.74	23.28	32.43	19.01	20.49	26.65
ENSMUSG00000005142 Man2b1	mannosidase 2, alpha B1 [Source:MGI Symbol;Acc:MGI:107286]	3.875811535	1.95449842	9.73E-185	2.31E-181	yes	up	36.272	116.162	33.92	33.43	37.89	37.5	38.62	106.8	121.04	111.61	114.4	126.96
ENSMUSG00000028862 Map3k6	mitogen-activated protein kinase kinase kinase 6 [Source:MGI Symbol;Acc:MGI:1855691]	0.265646373	-1.912421078	4.98E-14	7.88E-13	yes	down	42.664	9.6	52.89	36.55	39.14	36.62	48.12	13.3	11.39	7.05	8.76	7.5
ENSMUSG00000025610 Map3k7cl	Map3k7 C-terminal like [Source:MGI Symbol;Acc:MGI:2446584]	0.439818806	-1.185018802	7.32E-06	4.37E-05	yes	down	2.324	0.866	2.29	2.53	1.9	2.51	2.39	1.12	0.87	0.83	0.83	0.68
ENSMUSG00000037337 Map4k1	mitogen-activated protein kinase kinase kinase kinase 1 [Source:MGI Symbol;Acc:MGI:1346882]	2.536984701	1.34311482	3.36E-24	1.11E-22	yes	up	5.896	14.278	6.45	5.01	6.63	6.12	5.27	16.16	11.55	17.44	8.56	17.68
ENSMUSG00000032577 Mapkapk3	mitogen-activated protein kinase-activated protein kinase 3 [Source:MGI Symbol;Acc:MGI:2143163]	2.136825399	1.095469029	1.38E-43	1.09E-41	yes	up	16.556	29.986	17.46	15.77	16.62	16.19	16.74	27.52	33.54	29.07	29	30.8
ENSMUSG00000018411 Mapt	microtubule-associated protein tau [Source:MGI Symbol;Acc:MGI:97180]	0.455708595	-1.133816512	4.08E-18	9.13E-17	yes	down	53.52	20.372	65.11	37.97	48.68	63.72	52.12	26.32	22.86	17.62	19.2	15.86

ENSMUSG00000047945 Marcksl1	MARCKS-like 1 [Source:MGI Symbol;Acc:MGI:97143]	2.283436769	1.19120684	1.36E-09	1.36E-08	yes	up	10.3	19.782	7.38	11.01	9.3	13.33	10.48	25.24	15.19	20.3	13.42	24.76
ENSMUSG00000022887 Masp1	mannan-binding lectin serine peptidase 1 [Source:MGI Symbol;Acc:MGI:88492]	0.400712565	-1.319360348	1.40E-10	1.55E-09	yes	down	1.816	0.61	1.88	1.8	1.71	2.14	1.55	0.61	0.7	0.77	0.55	0.42
ENSMUSG00000004933 Matk	megakaryocyte-associated tyrosine kinase [Source:MGI Symbol;Acc:MGI:99259]	7.321088704	2.872058205	1.14E-29	5.18E-28	yes	up	1.096	6.314	0.51	1.62	0.88	1.32	1.15	3.88	6.58	5.6	6.57	8.94
ENSMUSG00000013974 Mcemp1	mast cell expressed membrane protein 1 [Source:MGI Symbol;Acc:MGI:1916439]	3.785242256	1.920385636	3.63E-83	8.53E-81	yes	up	18.85	60.496	19.24	19.08	19.33	16.22	20.38	51.06	66.5	63.59	58.91	62.42
ENSMUSG00000050164 Mchr1	melanin-concentrating hormone receptor 1 [Source:MGI Symbol;Acc:MGI:2180756]	0.337843626	-1.565572459	0.002759126	0.00967569	yes	down	0.556	0.158	0.71	0.54	0.25	0.91	0.37	0.13	0.14	0.12	0.23	0.17
ENSMUSG00000026669 Mcm10	minichromosome maintenance 10 replication initiation factor [Source:MGI Symbol;Acc:MGI:1917274]	2.183972216	1.126954503	0.000209384	0.000951044	yes	up	0.804	1.34	0.82	0.3	1.05	0.85	1	1.75	1.19	1.07	1.07	1.62
ENSMUSG00000011008 Mcoln2	mucolipin 2 [Source:MGI Symbol;Acc:MGI:1915529]	6.617635442	2.726315817	3.76E-30	1.76E-28	yes	up	1.194	3.638	2.58	0.81	1.63	0.63	0.32	3.32	3.21	3.85	3.69	4.12
ENSMUSG00000036853 Mcoln3	mucolipin 3 [Source:MGI Symbol;Acc:MGI:1890500]	5.09503788	2.349092872	8.15E-43	6.28E-41	yes	up	5.272	20.596	5.74	3.62	8.26	3.82	4.92	18.61	22.37	21.76	24.11	16.13
ENSMUSG00000061068 Mcpt4	mast cell protease 4 [Source:MGI Symbol;Acc:MGI:96940]	0.228054131	-2.132551789	0.000396438	0.001697004	yes	down	1.93	0.37	1.36	4.15	2.58	1.25	0.31	0.48	0.39	0.28	0.36	0.34
ENSMUSG00000027994 Mcub	mitochondrial calcium uniporter dominant negative beta subunit [Source:MGI Symbol;Acc:MGI:1914065]	2.100611728	1.070809523	4.67E-17	9.69E-16	yes	up	13.508	22.048	13.91	11.27	12.87	12.29	17.2	18.83	20.9	21.1	23.14	26.27
ENSMUSG00000027239 Mdk	midkine [Source:MGI Symbol;Acc:MGI:96949]	2.942618347	1.557100439	8.95E-14	1.38E-12	yes	up	9.72	24.054	8.76	9.84	13.63	7.68	8.69	21.49	18.75	24.38	21.8	33.85
ENSMUSG00000030621 Me3	malic enzyme 3, NADP(+)-dependent, mitochondrial [Source:MGI Symbol;Acc:MGI:1916679]	0.425812862	-1.231708568	7.96E-05	0.000393506	yes	down	2.786	1.214	1.72	4.2	2.31	2.39	3.31	0.94	0.65	1.44	2.11	0.93
ENSMUSG00000029659 Medag	mesenteric estrogen dependent adipogenesis [Source:MGI Symbol;Acc:MGI:1917967]	0.398493275	-1.327372717	4.26E-12	5.57E-11	yes	down	11.456	3.814	9.74	14.86	11.06	9.48	12.14	2.55	3.22	5.59	3.87	3.84
ENSMUSG00000079033 Mef2b	myocyte enhancer factor 2B [Source:MGI Symbol;Acc:MGI:104526]	4.189673083	2.066837676	0.00306611	0.010625178	yes	up	0.226	0.764	0.06	0.32	0.29	0.3	0.16	1.83	0.31	0.4	0.38	0.9
ENSMUSG00000022534 Mefv	Mediterranean fever [Source:MGI Symbol;Acc:MGI:1859396]	4.116591013	2.041450123	1.62E-06	1.07E-05	yes	up	0.672	2.312	0.72	0.82	0.37	1.32	0.13	3.7	1.66	1.9	1.6	2.7
ENSMUSG00000036466 Megf11	multiple EGF-like-domains 11 [Source:MGI Symbol;Acc:MGI:1920951]	4.294140946	2.102369545	4.60E-13	6.59E-12	yes	up	0.172	0.64	0.17	0.22	0.12	0.15	0.2	0.59	0.58	0.79	0.44	0.8
ENSMUSG00000035683 Melk	maternal embryonic leucine zipper kinase [Source:MGI Symbol;Acc:MGI:106924]	2.365722665	1.242280956	5.40E-07	3.85E-06	yes	up	0.876	1.742	0.66	0.89	1.2	0.88	0.75	2.06	1.41	1.82	1.27	2.15
ENSMUSG00000039208 Metrnl	meteorin, glial cell differentiation regulator-like [Source:MGI Symbol;Acc:MGI:2384806]	3.041622085	1.604840912	2.37E-42	1.79E-40	yes	up	12.574	33.202	12.33	13.38	13.21	10.93	13.02	27.1	34.32	30.95	38.98	34.66
ENSMUSG00000030116 Mfap5	microfibrillar associated protein 5 [Source:MGI Symbol;Acc:MGI:1354387]	2.04717463	1.033634174	4.58E-14	7.29E-13	yes	up	35.128	62.318	31.77	39.76	39.58	31.13	33.4	43.68	63.96	63.17	65.04	75.74
ENSMUSG00000030605 Mfge8	milk fat globule-EGF factor 8 protein [Source:MGI	2.504281397	1.324396682	2.74E-46	2.43E-44	yes	up	196.28	403.41	202.25	178.34	180.45	222.36	198	376.05	437.81	334.15	475.85	393.19

		Symbol;Acc:MGI:102768]																		
ENSMUSG00000034854	Mfsd12	major facilitator superfamily domain containing 12 [Source:MGI Symbol;Acc:MGI:3604804]	4.44955416	2.153660787	3.32E-125	2.29E-122	yes	up	8.13	30.634	6.94	8.06	8.26	8.02	9.37	29.33	32.34	28.65	30.44	32.41
ENSMUSG00000037336	Mfsd2b	major facilitator superfamily domain containing 2B [Source:MGI Symbol;Acc:MGI:3583946]	0.434415666	-1.202851963	1.08E-05	6.26E-05	yes	down	2.488	0.86	2.87	3.93	2.07	2.09	1.48	0.7	1.12	0.86	0.87	0.75
ENSMUSG00000029490	Mfsd7a	major facilitator superfamily domain containing 7A [Source:MGI Symbol;Acc:MGI:2442629]	3.561356331	1.832426792	6.53E-17	1.34E-15	yes	up	1.552	4.658	0.85	2.07	1.63	1.91	1.3	4.86	6.09	3.57	4.24	4.53
ENSMUSG00000068587	Mgam	maltase-glucoamylase [Source:MGI Symbol;Acc:MGI:1203495]	0.413779266	-1.273066739	0.005950935	0.019147418	yes	down	0.57	0.312	0.45	1.14	0.36	0.39	0.51	0.99	0.1	0.15	0.18	0.14
ENSMUSG00000040950	Mgl2	macrophage galactose N-acetyl-galactosamine specific lectin 2 [Source:MGI Symbol;Acc:MGI:2385729]	0.462049838	-1.113879622	1.64E-08	1.46E-07	yes	down	24.92	10.168	21.79	32	25.69	20.6	24.52	9.07	6.5	15.61	7.56	12.1
ENSMUSG00000040987	Mill2	MHC I like leukocyte 2 [Source:MGI Symbol;Acc:MGI:2179989]	0.388671335	-1.363377384	4.03E-30	1.87E-28	yes	down	26.86	9.268	30.5	24.13	26.56	24.74	28.37	7.76	10.87	12.06	9.34	6.31
ENSMUSG00000040528	Milr1	mast cell immunoglobulin like receptor 1 [Source:MGI Symbol;Acc:MGI:2685731]	3.535703724	1.821997389	7.23E-20	1.81E-18	yes	up	4.474	12.174	4.87	6.03	4.86	3.2	3.41	12.12	12.09	9.8	11.56	15.3
ENSMUSG00000065601	Mir146	microRNA 146 [Source:MGI Symbol;Acc:MGI:2676831]	2.814959931	1.493114387	3.47E-08	2.93E-07	yes	up	1.058	2.494	1.48	0.6	1.19	1.26	0.76	3.87	2.43	2.11	1.75	2.31
ENSMUSG00000097418	Mir155hg	Mir155 host gene (non-protein coding) [Source:MGI Symbol;Acc:MGI:5477161]	3.394048026	1.763006979	0.008460591	0.026025856	yes	up	0.358	1.012	0.3	0.25	0.56	0.58	0.1	2.22	1.36	0.28	0.3	0.9
ENSMUSG00000047534	Mis18bp1	MIS18 binding protein 1 [Source:MGI Symbol;Acc:MGI:2145099]	2.969422207	1.570182238	1.51E-06	1.01E-05	yes	up	0.576	1.348	0.59	0.34	0.71	0.42	0.82	1.44	1.83	0.6	0.8	2.07
ENSMUSG00000035158	Mitf	melanogenesis associated transcription factor [Source:MGI Symbol;Acc:MGI:104554]	2.35251867	1.234206173	1.95E-36	1.19E-34	yes	up	6.142	11.404	6.77	6.63	6.85	5.24	5.22	9.16	13.03	11.54	10.49	12.8
ENSMUSG00000031004	Mki67	antigen identified by monoclonal antibody Ki 67 [Source:MGI Symbol;Acc:MGI:106035]	3.324222404	1.733016908	4.26E-12	5.57E-11	yes	up	5.388	11.99	4.62	8.68	5.66	3.57	4.41	15.71	10.82	9.93	8.08	15.41
ENSMUSG00000005373	Mlxipl	MLX interacting protein-like [Source:MGI Symbol;Acc:MGI:1927999]	0.350213992	-1.513691372	0.000589386	0.002425689	yes	down	1.332	0.578	0.63	1.36	1.78	1.51	1.38	0.9	0.33	0.76	0.86	0.04
ENSMUSG00000049723	Mmp12	matrix metallopeptidase 12 [Source:MGI Symbol;Acc:MGI:97005]	50.20214869	5.649677209	3.04E-117	1.72E-114	yes	up	7.96	347.612	7	11.95	9.7	4.5	6.65	398.72	333.61	346.39	400.18	259.16
ENSMUSG00000050578	Mmp13	matrix metallopeptidase 13 [Source:MGI Symbol;Acc:MGI:1340026]	4.820887135	2.269298654	4.32E-20	1.11E-18	yes	up	0.702	2.836	1.09	0.66	0.63	0.54	0.59	3.21	2.11	2.56	2.95	3.35
ENSMUSG00000000957	Mmp14	matrix metallopeptidase 14 (membrane-inserted) [Source:MGI Symbol;Acc:MGI:101900]	2.548827789	1.349833901	8.23E-41	5.80E-39	yes	up	34.002	72.738	33.86	35.19	34.96	32.08	33.92	62.35	72.25	76.86	63.96	88.27
ENSMUSG00000025355	Mmp19	matrix metallopeptidase 19 [Source:MGI Symbol;Acc:MGI:1927899]	5.041068614	2.333729591	5.46E-21	1.47E-19	yes	up	13.228	54.466	14.79	14.29	14.62	6.86	15.58	43.19	67.67	50.06	57.1	54.31
ENSMUSG00000031740	Mmp2	matrix metallopeptidase 2 [Source:MGI Symbol;Acc:MGI:97009]	3.353353776	1.745604694	8.67E-38	5.54E-36	yes	up	45.52	125.372	41.77	48.91	52.44	35.31	49.17	89.31	140.85	135.87	117.85	142.98
ENSMUSG00000023903	Mmp25	matrix metallopeptidase 25 [Source:MGI Symbol;Acc:MGI:2443938]	2.798971269	1.484896678	3.41E-06	2.17E-05	yes	up	0.602	1.376	0.51	0.67	0.74	0.48	0.61	1.21	0.91	1.58	0.7	2.48
ENSMUSG00000043613	Mmp3	matrix metallopeptidase 3 [Source:MGI Symbol;Acc:MGI:97010]	0.493691585	-1.01831804	0.00455718	0.015114845	yes	down	41.798	17.646	43.86	43.4	40.56	15.25	65.92	12.63	20.78	22.01	15.45	17.36
ENSMUSG00000005800	Mmp8	matrix metallopeptidase 8 [Source:MGI Symbol;Acc:MGI:1202395]	3.451757061	1.787330929	5.34E-14	8.42E-13	yes	up	3.372	9.798	3.34	4.76	2.82	2.99	2.95	16.23	9.03	6	9.69	8.04

ENSMUSG00000052396 Mogat2	monoacylglycerol O-acyltransferase 2 [Source:MGI Symbol;Acc:MGI:2663253]	0.077883365	-3.682540968	0.011814533	0.034622338	yes	down	0.132	0.006	0.08	0.16	0.04	0.21	0.17	0	0.03	0	0	0
ENSMUSG00000046805 Mpeg1	macrophage expressed gene 1 [Source:MGI Symbol;Acc:MGI:1333743]	8.369144329	3.065080128	2.04E-156	2.56E-153	yes	up	83.788	593.312	75.11	90.64	105.23	64.34	83.62	556.09	598.83	551.22	644.1	616.32
ENSMUSG00000006389 Mpl	myeloproliferative leukemia virus oncogene [Source:MGI Symbol;Acc:MGI:97076]	0.364132163	-1.457465918	0.001949612	0.007117779	yes	down	0.76	0.2	0.58	1.22	0.74	0.78	0.48	0.13	0.35	0.1	0.28	0.14
ENSMUSG00000052373 Mpp3	membrane protein, palmitoylated 3 (MAGUK p55 subfamily member 3) [Source:MGI Symbol;Acc:MGI:1328354]	0.248689926	-2.007580029	6.71E-08	5.45E-07	yes	down	3.036	0.742	3.16	3.04	1.85	5.54	1.59	0.95	1.11	0.65	0.63	0.37
ENSMUSG00000056569 Mpz	myelin protein zero [Source:MGI Symbol;Acc:MGI:103177]	0.189802356	-2.397430197	0.005266308	0.017184711	yes	down	12.298	2.434	3.13	12.63	37.05	2.04	6.64	0.86	3.55	2.65	3.65	1.46
ENSMUSG00000026712 Mrc1	mannose receptor, C type 1 [Source:MGI Symbol;Acc:MGI:97142]	2.164353704	1.113936287	1.73E-06	1.15E-05	yes	up	28.404	51.696	27.27	30.62	36.01	16.11	32.01	43.95	59.67	53.03	54.96	46.87
ENSMUSG00000039395 Mreg	melanoregulin [Source:MGI Symbol;Acc:MGI:2151839]	4.022069294	2.007937937	2.78E-30	1.31E-28	yes	up	2.56	8.674	2.03	2.95	2.45	3.09	2.28	11.44	8.56	6.73	7.85	8.79
ENSMUSG00000048965 Mrgpre	MAS-related GPR, member E [Source:MGI Symbol;Acc:MGI:2441884]	3.076087268	1.621096433	2.02E-18	4.60E-17	yes	up	1.188	3.08	1.36	1.1	0.85	1.53	1.1	3.81	3.18	2.62	2.7	3.09
ENSMUSG00000099398 Ms4a14	membrane-spanning 4-domains, subfamily A, member 14 [Source:MGI Symbol;Acc:MGI:2686122]	5.722592527	2.516668885	4.25E-16	7.99E-15	yes	up	0.31	1.506	0.23	0.53	0.22	0.27	0.3	2.06	1.81	1.54	0.91	1.21
ENSMUSG00000101389 Ms4a4a	membrane-spanning 4-domains, subfamily A, member 4A [Source:MGI Symbol;Acc:MGI:3643932]	2.27402177	1.185246065	5.65E-08	4.64E-07	yes	up	8.484	16.278	8.38	10.57	7.5	7.67	8.3	25.17	13.18	14.23	13.99	14.82
ENSMUSG00000079419 Ms4a6c	membrane-spanning 4-domains, subfamily A, member 6C [Source:MGI Symbol;Acc:MGI:2385644]	3.800690899	1.926261699	1.18E-30	5.63E-29	yes	up	18.536	58.008	12.94	22.99	18.98	23.1	14.67	76.17	46.85	58.31	42.93	65.78
ENSMUSG00000024679 Ms4a6d	membrane-spanning 4-domains, subfamily A, member 6D [Source:MGI Symbol;Acc:MGI:1916024]	8.565045553	3.098460919	3.05E-84	7.43E-82	yes	up	9.87	71.71	10.17	11.33	11.16	6.11	10.58	85.2	66.62	61.73	65.6	79.4
ENSMUSG00000024672 Ms4a7	membrane-spanning 4-domains, subfamily A, member 7 [Source:MGI Symbol;Acc:MGI:1918846]	13.6802612	3.774023871	2.06E-45	1.75E-43	yes	up	6.454	75.278	6.31	8.74	9.05	3.75	4.42	75.29	72.41	66.27	73.17	89.25
ENSMUSG00000024730 Ms4a8a	membrane-spanning 4-domains, subfamily A, member 8A [Source:MGI Symbol;Acc:MGI:1927657]	2.352022223	1.233901691	1.22E-07	9.59E-07	yes	up	17.232	34.532	16.46	14.11	25.21	8.26	22.12	31.78	43.07	38.3	33.78	25.73
ENSMUSG00000025044 Msr1	macrophage scavenger receptor 1 [Source:MGI Symbol;Acc:MGI:98257]	12.01107095	3.586292887	4.18E-113	1.99E-110	yes	up	2.916	24.634	4.02	3.28	2.68	2.22	2.38	21.2	24.06	22.58	30.95	24.38
ENSMUSG00000075705 Msrb1	methionine sulfoxide reductase B1 [Source:MGI Symbol;Acc:MGI:1351642]	2.248074162	1.168689629	1.73E-39	1.17E-37	yes	up	79.684	150.908	76.03	89.89	83.22	75.81	73.47	132.67	159.13	137.35	168.4	156.99
ENSMUSG00000065947 mt-Nd4l	mitochondrially encoded NADH dehydrogenase 4L [Source:MGI Symbol;Acc:MGI:102497]	0.417680128	-1.259529589	1.15E-05	6.62E-05	yes	down	273.28	100.18	307.97	361.18	220.24	206.29	270.72	187.48	75.06	57.61	69.89	110.86
ENSMUSG00000064339 mt-Rnr2	mitochondrially encoded 16S rRNA [Source:MGI Symbol;Acc:MGI:102492]	0.413476934	-1.274121245	1.07E-16	2.17E-15	yes	down	644.822	225.024	774.27	664.4	727.68	490.27	567.49	221.2	196	306.62	234.97	166.33
ENSMUSG00000019992 Mtfr2	mitochondrial fission regulator 2 [Source:MGI	4.388585786	2.133756109	1.31E-05	7.45E-05	yes	up	2.962	0.986	13.65	0.28	0.56	0.2	0.12	1.59	0.48	1.22	0.64	1

	Symbol;Acc:MGI:1919054]																		
ENSMUSG00000042784 Muc1	mucin 1, transmembrane [Source:MGI Symbol;Acc:MGI:97231]	2.081825228	1.057848958	2.11E-14	3.44E-13	yes	up	112.036	196.742	107.02	137.96	110.91	85.71	118.58	148.84	196.76	219.92	203.62	214.57
ENSMUSG00000109564 Muc16	mucin 16 [Source:MGI Symbol;Acc:MGI:1920982]	0.363936796	-1.458240173	0.002329689	0.008339227	yes	down	7.908	3.462	7.41	10.49	7.53	7.19	6.92	2.15	3.44	2.43	8.69	0.6
ENSMUSG00000073408 Mucl3	mucin like 3 [Source:MGI Symbol;Acc:MGI:2685476]	3.604045944	1.849617402	1.82E-08	1.60E-07	yes	up	0.426	1.31	0.28	0.39	0.42	0.35	0.69	1.14	1.38	1.29	0.91	1.83
ENSMUSG00000042515 Mum1l1	melanoma associated antigen (mutated) 1-like 1 [Source:MGI Symbol;Acc:MGI:2445062]	0.439495983	-1.186078115	2.75E-08	2.37E-07	yes	down	1.898	0.688	1.77	1.91	1.74	2.16	1.91	0.68	0.48	0.74	0.91	0.63
ENSMUSG00000042485 Mustn1	musculoskeletal, embryonic nuclear protein 1 [Source:MGI Symbol;Acc:MGI:1913425]	0.458641597	-1.124560886	4.49E-19	1.07E-17	yes	down	30.596	11.866	28.87	36.27	27	32.72	28.12	9.41	13.73	13.14	11.13	11.92
ENSMUSG00000006517 Mvd	mevalonate (diphospho) decarboxylase [Source:MGI Symbol;Acc:MGI:2179327]	2.72668258	1.447146762	2.90E-28	1.23E-26	yes	up	10.2	23.528	8.34	12.27	11.62	8.89	9.88	20.08	27.46	23.02	21.28	25.8
ENSMUSG00000000386 Mx1	MX dynamin-like GTPase 1 [Source:MGI Symbol;Acc:MGI:97243]	2.529696334	1.338964213	1.27E-05	7.22E-05	yes	up	1.316	2.912	0.97	1.62	1.24	1.76	0.99	3.09	3.01	3.61	1.07	3.78
ENSMUSG00000021485 Mxd3	Max dimerization protein 3 [Source:MGI Symbol;Acc:MGI:104987]	2.060780929	1.043191147	0.00282387	0.00988493	yes	up	1.638	2.814	1.37	1.46	2.73	1.03	1.6	2.5	2.12	3.54	1.61	4.3
ENSMUSG00000039110 Mycbpap	MYCBP associated protein [Source:MGI Symbol;Acc:MGI:2388726]	0.436485578	-1.195994108	4.22E-08	3.53E-07	yes	down	7.65	3.12	9.08	7.91	6.48	7.76	7.02	1.73	4.07	5.06	1.84	2.9
ENSMUSG00000037169 Mycn	v-myc avian myelocytomatosis viral related oncogene, neuroblastoma derived [Source:MGI Symbol;Acc:MGI:97357]	2.425713983	1.278409452	0.000673002	0.002735334	yes	up	0.468	0.93	0.48	0.35	0.63	0.44	0.44	0.41	0.77	1.1	0.85	1.52
ENSMUSG00000020900 Myh10	myosin, heavy polypeptide 10, non-muscle [Source:MGI Symbol;Acc:MGI:1930780]	0.405123569	-1.303566077	2.77E-43	2.17E-41	yes	down	85.43	30.948	85.59	85.57	78.52	106.74	70.73	28.44	32.42	27.01	34.7	32.17
ENSMUSG00000018830 Myh11	myosin, heavy polypeptide 11, smooth muscle [Source:MGI Symbol;Acc:MGI:102643]	0.414178521	-1.271675356	3.41E-08	2.89E-07	yes	down	38.996	13.56	36.2	54.97	25.03	41.67	37.11	10.47	14.36	17.6	10.47	14.9
ENSMUSG00000053093 Myh7	myosin, heavy polypeptide 7, cardiac muscle, beta [Source:MGI Symbol;Acc:MGI:2155600]	0.464989905	-1.1047287	4.67E-10	4.91E-09	yes	down	13.662	4.438	12.54	13.37	15.88	11.84	14.68	4.14	3.64	5.82	3.62	4.97
ENSMUSG00000059741 Myl3	myosin, light polypeptide 3 [Source:MGI Symbol;Acc:MGI:97268]	0.149998674	-2.736978344	0.001651478	0.006134962	yes	down	2.418	0.332	0.82	5.82	1.05	3.39	1.01	0.27	0	1.1	0	0.29
ENSMUSG00000067818 Myl9	myosin, light polypeptide 9, regulatory [Source:MGI Symbol;Acc:MGI:2138915]	0.483640198	-1.047993935	3.80E-05	0.000198385	yes	down	111.786	45.4	101.01	177.06	62.87	120.67	97.32	36.54	38.71	60.74	42.31	48.7
ENSMUSG00000039057 Myo16	myosin XVI [Source:MGI Symbol;Acc:MGI:2685951]	2.814411415	1.492833239	0.000984128	0.003863627	yes	up	0.112	0.234	0.07	0.19	0.06	0.14	0.1	0.24	0.18	0.19	0.22	0.34
ENSMUSG00000024300 Myo1f	myosin IF [Source:MGI Symbol;Acc:MGI:107711]	4.861568937	2.281421979	3.19E-117	1.75E-114	yes	up	14.188	57.7	12.02	16.55	15.84	14.02	12.51	52.18	61.83	58.14	52.7	63.65
ENSMUSG00000020437 Myo1g	myosin IG [Source:MGI Symbol;Acc:MGI:1927091]	2.102975793	1.072432244	4.09E-23	1.26E-21	yes	up	18.244	29.878	19.48	17.94	18.21	20.58	15.01	26.82	32.02	30.27	25.92	34.36
ENSMUSG00000042064 Myo3b	myosin IIIB [Source:MGI Symbol;Acc:MGI:2448580]	3.796142111	1.924534002	5.59E-09	5.25E-08	yes	up	0.154	0.644	0.12	0.17	0.16	0.16	0.16	0.51	0.57	0.43	0.7	1.01
ENSMUSG00000034593 Myo5a	myosin VA [Source:MGI Symbol;Acc:MGI:105976]	4.561205791	2.189415263	2.40E-144	2.14E-141	yes	up	11.106	34.41	11.36	13.82	12.23	7.83	10.29	32.11	34.18	36.8	34.78	34.18
ENSMUSG00000030761 Myo7a	myosin VIIA [Source:MGI Symbol;Acc:MGI:104510]	2.32009809	1.214185801	6.46E-21	1.73E-19	yes	up	10.478	20.954	11.56	8.35	11.07	8.45	12.96	21.18	24.58	19.34	18.36	21.31
ENSMUSG00000020542 Myocd	myocardin [Source:MGI Symbol;Acc:MGI:2137495]	0.463503125	-1.10934903	0.000902444	0.00357704	yes	down	2.318	0.714	0.85	4.7	3.58	1.77	0.69	0.96	0.59	1.49	0.25	0.28

ENSMUSG00000048612 Myof	myoferlin [Source:MGI Symbol;Acc:MGI:1919192]	2.087235963	1.061593707	1.02E-34	5.85E-33	yes	up	66.27	112.63	71.78	62.53	68.81	59.75	68.48	96.79	122.46	110.12	104.25	129.53
ENSMUSG00000041794 Myrip	myosin VIIA and Rab interacting protein [Source:MGI Symbol;Acc:MGI:2384407]	0.3417448	-1.549008709	2.91E-05	0.000156051	yes	down	24.742	5.96	49.9	10.08	22.54	14.94	26.25	9.44	7.04	3.76	5.16	4.4
ENSMUSG00000024353 Mzb1	marginal zone B and B1 cell-specific protein 1 [Source:MGI Symbol;Acc:MGI:1917066]	8.421251485	3.074034649	3.07E-16	5.89E-15	yes	up	16.442	117.926	9.41	24.5	25.81	11.23	11.26	175.63	93.28	95.69	84.28	140.75
ENSMUSG00000029413 Naaa	N-acylethanolamine acid amidase [Source:MGI Symbol;Acc:MGI:1914361]	2.252079289	1.171257621	2.28E-20	5.95E-19	yes	up	58.228	101.358	47.71	73.09	64.03	41.75	64.56	77.95	114.04	102.38	103.86	108.56
ENSMUSG00000026107 Nabp1	nucleic acid binding protein 1 [Source:MGI Symbol;Acc:MGI:1923258]	2.169546237	1.117393333	2.05E-21	5.65E-20	yes	up	21.134	36.54	19.34	21.67	26.66	18.61	19.39	35.7	37.87	29.49	41.24	38.4
ENSMUSG00000001751 Naglu	alpha-N-acetylglucosaminidase (Sanfilippo disease IIIB) [Source:MGI Symbol;Acc:MGI:1351641]	2.315315969	1.21120909	7.19E-29	3.17E-27	yes	up	9.416	18.454	9.99	10.66	7.65	9.61	9.17	16.3	21.21	16.77	17.81	20.18
ENSMUSG00000048217 Nags	N-acetylglutamate synthase [Source:MGI Symbol;Acc:MGI:2387600]	10.04325856	3.328155525	1.48E-12	2.02E-11	yes	up	0.134	1.048	0.12	0.21	0.13	0.12	0.09	0.79	1	1.05	1.09	1.31
ENSMUSG00000021640 Naip1	NLR family, apoptosis inhibitory protein 1 [Source:MGI Symbol;Acc:MGI:1298223]	6.767975073	2.758724255	6.58E-08	5.35E-07	yes	up	0.1	0.398	0.28	0.1	0.06	0	0.06	0.34	0.65	0.32	0.32	0.36
ENSMUSG00000078945 Naip2	NLR family, apoptosis inhibitory protein 2 [Source:MGI Symbol;Acc:MGI:1298226]	4.215633582	2.075749475	4.44E-59	5.69E-57	yes	up	5.306	18.902	4.79	6.25	6.17	3.73	5.59	18.03	19.6	17.89	18.73	20.26
ENSMUSG00000071203 Naip5	NLR family, apoptosis inhibitory protein 5 [Source:MGI Symbol;Acc:MGI:1298220]	3.313496453	1.728354375	7.23E-52	7.55E-50	yes	up	1.958	5.422	1.87	2.24	2.14	1.95	1.59	5.26	6.15	5.59	5.21	4.9
ENSMUSG00000078942 Naip6	NLR family, apoptosis inhibitory protein 6 [Source:MGI Symbol;Acc:MGI:1298222]	3.174851845	1.66668927	2.03E-19	4.96E-18	yes	up	0.892	2.386	0.64	0.74	0.8	1.16	1.12	2.64	2.89	2.19	2.22	1.99
ENSMUSG00000055430 Nap1l5	nucleosome assembly protein 1-like 5 [Source:MGI Symbol;Acc:MGI:1923555]	0.359040215	-1.477782651	0.000593852	0.002442936	yes	down	1.392	0.452	1.52	1.21	1.32	1.78	1.13	0.76	0.26	0.41	0.27	0.56
ENSMUSG00000022762 Ncam2	neural cell adhesion molecule 2 [Source:MGI Symbol;Acc:MGI:97282]	0.292762545	-1.772197104	0.002683258	0.009440553	yes	down	0.19	0.068	0.2	0.2	0.23	0.12	0.2	0.04	0.09	0.01	0.05	0.15
ENSMUSG00000038252 Ncapd2	non-SMC condensin I complex, subunit D2 [Source:MGI Symbol;Acc:MGI:1915548]	2.037779678	1.026998078	8.87E-12	1.12E-10	yes	up	4.854	9.356	6.22	3.75	5.49	4.06	4.75	12.27	9.42	6.54	5.97	12.58
ENSMUSG00000015880 Ncapg	non-SMC condensin I complex, subunit G [Source:MGI Symbol;Acc:MGI:1930197]	3.863369397	1.949859628	4.89E-12	6.34E-11	yes	up	0.716	2.096	0.58	0.62	1.01	0.65	0.72	2.67	1.44	1.78	1.36	3.23
ENSMUSG00000042029 Ncapg2	non-SMC condensin II complex, subunit G2 [Source:MGI Symbol;Acc:MGI:1923294]	2.841809565	1.50680988	2.63E-24	8.72E-23	yes	up	2.932	6.94	2.28	4.37	2.68	3.07	2.26	7.67	7.46	5.68	5.12	8.77
ENSMUSG00000015950 Ncf1	neutrophil cytosolic factor 1 [Source:MGI Symbol;Acc:MGI:97283]	5.767660523	2.527986252	2.17E-104	8.61E-102	yes	up	23.394	110.702	24.38	26.81	25.38	18.06	22.34	88.8	126.83	109.58	105.75	122.55
ENSMUSG00000026480 Ncf2	neutrophil cytosolic factor 2 [Source:MGI Symbol;Acc:MGI:97284]	3.792643347	1.92320371	1.19E-86	3.16E-84	yes	up	20.118	65.004	26.45	20.65	18.73	18.26	16.5	57.58	76.36	61.25	71.2	58.63
ENSMUSG00000071715 Ncf4	neutrophil cytosolic factor 4 [Source:MGI Symbol;Acc:MGI:109186]	3.233829119	1.693243446	3.04E-40	2.11E-38	yes	up	10.136	28.026	10.25	10.37	12.67	9.8	7.59	32.9	28.1	23.28	26.41	29.44

ENSMUSG00000022488 Nckap1l	NCK associated protein 1 like [Source:MGI Symbol;Acc:MGI:1926063]	4.020328454	2.007313372	3.29E-138	2.71E-135	yes	up	16.038	54.32	15.19	17.7	16.87	16.12	14.31	54.12	55.02	48.98	52.11	61.37
ENSMUSG00000049690 Nckap5	NCK-associated protein 5 [Source:MGI Symbol;Acc:MGI:2686394]	0.351171131	-1.509753847	3.80E-41	2.69E-39	yes	down	57.54	18.87	78.77	46.2	68.23	51.7	42.8	15.72	18.94	18.9	22.71	18.08
ENSMUSG00000056234 Ncoa4	nuclear receptor coactivator 4 [Source:MGI Symbol;Acc:MGI:1350932]	2.083561561	1.059051726	2.06E-41	1.49E-39	yes	up	56.196	103.676	49.32	57.44	66.37	53.82	54.03	92.01	102.18	97.36	115.71	111.12
ENSMUSG00000062524 Ncr1	natural cytotoxicity triggering receptor 1 [Source:MGI Symbol;Acc:MGI:1336212]	0.408710004	-1.290850538	3.94E-08	3.31E-07	yes	down	4.868	1.692	5.53	4.31	3.68	5.42	5.4	1.37	2.25	1.28	1.39	2.17
ENSMUSG00000024056 Ndc80	NDC80 kinetochore complex component [Source:MGI Symbol;Acc:MGI:1914302]	2.708741501	1.437622722	4.91E-09	4.64E-08	yes	up	1.328	3.058	1.33	1.08	0.89	1.95	1.39	4.04	2.83	2.29	2.23	3.9
ENSMUSG00000040138 Ndp	Norrie disease (pseudoglioma) (human) [Source:MGI Symbol;Acc:MGI:102570]	0.250956256	-1.994492183	0.016535916	0.046195301	yes	down	0.24	0.05	0.21	0.26	0.18	0.25	0.3	0.09	0.08	0.05	0.03	0
ENSMUSG00000004558 Ndrg2	N-myc downstream regulated gene 2 [Source:MGI Symbol;Acc:MGI:1352498]	0.458733617	-1.12427146	2.80E-27	1.11E-25	yes	down	57.396	23.214	44.79	55.63	55.2	81.94	49.42	17.67	22.43	25.47	17.19	33.31
ENSMUSG00000026950 Neb	nebulin [Source:MGI Symbol;Acc:MGI:97292]	3.022422551	1.595705371	1.40E-08	1.26E-07	yes	up	0.478	1.112	0.45	0.25	0.48	0.33	0.88	1.15	1.74	1.41	0.73	0.53
ENSMUSG00000035121 Neil2	nei like 2 (E. coli) [Source:MGI Symbol;Acc:MGI:2686058]	2.416912617	1.273165314	0.000564216	0.002332086	yes	up	0.69	1.548	0.43	0.29	0.51	0.94	1.28	1.34	2.01	2.16	1.51	0.72
ENSMUSG00000039396 Neil3	nei like 3 (E. coli) [Source:MGI Symbol;Acc:MGI:2384588]	2.36259913	1.240374863	0.001306347	0.00496746	yes	up	0.608	4.866	0.9	0.58	0.59	0.35	0.62	1.89	1.63	19.07	0.48	1.26
ENSMUSG00000026749 Nek6	NIMA (never in mitosis gene a)-related expressed kinase 6 [Source:MGI Symbol;Acc:MGI:1891638]	2.338297179	1.225458297	1.99E-52	2.11E-50	yes	up	11.182	27.204	10.39	12.95	10.79	11.25	10.53	26.94	26.9	26.15	28.8	27.23
ENSMUSG00000058099 Nfam1	Nfat activating molecule with ITAM motif 1 [Source:MGI Symbol;Acc:MGI:1921289]	3.119641849	1.64138041	7.50E-88	2.03E-85	yes	up	10.162	29.45	9.91	10.75	9.69	10.49	9.97	28.61	27.95	28.2	33.73	28.76
ENSMUSG00000027544 Nfatc2	nuclear factor of activated T cells, cytoplasmic, calcineurin dependent 2 [Source:MGI Symbol;Acc:MGI:102463]	2.079850891	1.056480102	4.50E-08	3.75E-07	yes	up	2.114	3.676	1.81	2.51	2.28	1.8	2.17	4.69	3.17	4.02	2.23	4.27
ENSMUSG00000029832 Nfe2l3	nuclear factor, erythroid derived 2, like 3 [Source:MGI Symbol;Acc:MGI:1339958]	0.401543135	-1.316373119	3.75E-08	3.16E-07	yes	down	2.144	0.704	2.43	2.5	1.5	2.56	1.73	0.74	0.83	0.68	0.68	0.59
ENSMUSG00000021025 Nfkbia	nuclear factor of kappa light polypeptide gene enhancer in B cells inhibitor, alpha [Source:MGI Symbol;Acc:MGI:104741]	0.494129822	-1.017037967	3.30E-19	7.99E-18	yes	down	205.05	85.188	227.49	172.45	185.39	187.49	252.43	79.04	85.58	106.15	81.51	73.66
ENSMUSG00000023947 Nfkbie	nuclear factor of kappa light polypeptide gene enhancer in B cells inhibitor, epsilon [Source:MGI Symbol;Acc:MGI:1194908]	2.758723662	1.464000951	8.08E-16	1.48E-14	yes	up	10.432	10.544	33.19	4.66	6.8	3.89	3.62	11.98	9.95	10.36	8.02	12.41
ENSMUSG00000021032 Ngb	neuroglobin [Source:MGI Symbol;Acc:MGI:2151886]	0.402174465	-1.31410661	2.31E-05	0.000125975	yes	down	2.798	0.976	2.59	2.28	2.91	3.9	2.31	0.85	0.8	1.3	1.3	0.63
ENSMUSG00000044231 Nhlrc1	NHL repeat containing 1 [Source:MGI Symbol;Acc:MGI:2145264]	0.459394872	-1.122193343	6.01E-08	4.91E-07	yes	down	4.522	1.75	3.97	4.95	3.4	6.17	4.12	2.15	1.64	1.72	1.3	1.94
ENSMUSG00000090113 Nhlrc4	NHL repeat containing 4 [Source:MGI Symbol;Acc:MGI:3687200]	0.480776495	-1.05656173	0.006996656	0.022074305	yes	down	7.752	4.33	2.08	8.85	2.14	16.23	9.46	5.1	4.57	8.31	0.98	2.69
ENSMUSG00000027574 Nkain4	Na+/K+ transporting ATPase interacting 4 [Source:MGI Symbol;Acc:MGI:1915372]	0.411024954	-1.282702111	1.26E-17	2.72E-16	yes	down	44.846	15.688	47.67	45.61	45.28	47.44	38.23	13.42	15.13	11.15	20.81	17.93

ENSMUSG00000041309 Nkx6-2	NK6 homeobox 2 [Source:MGI Symbol;Acc:MGI:1352738]	0.09068948	-3.462920983	0.004270524	0.014245724	yes	down	0.28	0.03	0.23	0.24	0.33	0.19	0.41	0.15	0	0	0	0
ENSMUSG00000049871 Nlrc3	NLR family, CARD domain containing 3 [Source:MGI Symbol;Acc:MGI:2444070]	2.177056508	1.122378855	4.67E-08	3.88E-07	yes	up	1.296	2.496	1	1.02	1.55	1.7	1.21	3.05	2.57	2.32	1.69	2.85
ENSMUSG00000039193 Nlrc4	NLR family, CARD domain containing 4 [Source:MGI Symbol;Acc:MGI:3036243]	2.920489917	1.546210404	2.67E-10	2.88E-09	yes	up	0.882	2.17	0.89	1.46	0.95	0.63	0.48	2.44	2.21	1.77	2.31	2.12
ENSMUSG00000074151 Nlrc5	NLR family, CARD domain containing 5 [Source:MGI Symbol;Acc:MGI:3612191]	2.192336321	1.132469136	6.12E-13	8.64E-12	yes	up	12.396	26.866	10.3	9.27	14.74	12.7	14.97	29.89	26.66	18.38	20.55	38.85
ENSMUSG00000069830 Nlrp1a	NLR family, pyrin domain containing 1A [Source:MGI Symbol;Acc:MGI:2684861]	4.711268171	2.236115454	2.46E-11	2.97E-10	yes	up	0.224	0.894	0.34	0.17	0.23	0.25	0.13	1.04	1.19	0.66	0.64	0.94
ENSMUSG00000070390 Nlrp1b	NLR family, pyrin domain containing 1B [Source:MGI Symbol;Acc:MGI:3582959]	3.463708096	1.792317352	4.60E-12	5.99E-11	yes	up	5.1	7.586	2.67	0.38	7.36	8.29	6.8	10.79	5.98	2.71	15.7	2.75
ENSMUSG00000092528 Nlrp1c-ps	NLR family, pyrin domain containing 1C, pseudogene [Source:MGI Symbol;Acc:MGI:3582962]	4.346145606	2.119736509	2.51E-06	1.62E-05	yes	up	0.21	2.802	0.22	0.33	0.13	0.26	0.11	3.83	4.89	1.08	1.84	2.37
ENSMUSG00000032691 Nlrp3	NLR family, pyrin domain containing 3 [Source:MGI Symbol;Acc:MGI:2653833]	2.233773666	1.159483014	2.39E-17	5.05E-16	yes	up	2.432	4.128	2.09	3.19	2.22	2.16	2.5	4.47	4.49	3.77	3.62	4.29
ENSMUSG00000046242 Nme9	NME/NM23 family member 9 [Source:MGI Symbol;Acc:MGI:4359686]	0.457163725	-1.129217163	4.70E-06	2.90E-05	yes	down	16.09	6.438	15.76	20.08	11.36	13.63	19.62	4.48	6.59	11.6	3.98	5.54
ENSMUSG00000067786 Nnat	neuronatin [Source:MGI Symbol;Acc:MGI:104716]	2.287338544	1.193669912	1.59E-13	2.40E-12	yes	up	13.046	25.644	11.71	9.59	15.18	13.3	15.45	22.08	28.6	27.17	18.58	31.79
ENSMUSG00000020826 Nos2	nitric oxide synthase 2, inducible [Source:MGI Symbol;Acc:MGI:97361]	5.276479752	2.399575744	2.17E-17	4.62E-16	yes	up	1.276	5.408	1.06	2.22	1.08	1.04	0.98	4.93	4.15	4.49	3.87	9.6
ENSMUSG00000031257 Nox1	NADPH oxidase 1 [Source:MGI Symbol;Acc:MGI:2450016]	0.238884548	-2.06561456	0.003970994	0.013363107	yes	down	0.828	0.186	1.13	0.73	0.4	1.41	0.47	0.55	0.02	0.05	0.02	0.29
ENSMUSG00000036805 Noxa1	NADPH oxidase activator 1 [Source:MGI Symbol;Acc:MGI:2449980]	2.287569905	1.193815831	0.007054802	0.022231555	yes	up	18.106	2.412	86.4	0.64	0.68	2.21	0.6	0.82	3.27	2.4	2.04	3.53
ENSMUSG00000026077 Npas2	neuronal PAS domain protein 2 [Source:MGI Symbol;Acc:MGI:109232]	0.250074178	-1.999571997	1.60E-16	3.19E-15	yes	down	8.496	1.794	8.76	7.89	7.51	8.35	9.97	1.51	3.23	1.73	1.69	0.81
ENSMUSG00000021242 Npc2	NPC intracellular cholesterol transporter 2 [Source:MGI Symbol;Acc:MGI:1915213]	2.007285593	1.005245895	4.19E-17	8.73E-16	yes	up	388.592	658.82	333.89	475.7	396.04	303.13	434.2	599.41	659.17	657.08	737.95	640.49
ENSMUSG00000022206 Npr3	natriuretic peptide receptor 3 [Source:MGI Symbol;Acc:MGI:97373]	0.273486039	-1.870460909	4.80E-08	3.98E-07	yes	down	262.142	59.134	370.34	183.65	285.48	228.34	242.9	77.99	67.79	30.77	100.94	18.18
ENSMUSG00000029819 Npy	neuropeptide Y [Source:MGI Symbol;Acc:MGI:97374]	1143.258511	10.15893594	8.62E-27	3.33E-25	yes	up	0.044	53.838	0	0	0.22	0	0	78.7	33.65	41.07	45.34	70.43
ENSMUSG00000003849 Nqo1	NAD(P)H dehydrogenase, quinone 1 [Source:MGI Symbol;Acc:MGI:103187]	0.394861925	-1.340579832	4.20E-11	4.92E-10	yes	down	7.126	2.378	5.36	8.49	6.97	8.74	6.07	2.29	3.11	2.01	2.61	1.87
ENSMUSG00000021775 Nr1d2	nuclear receptor subfamily 1, group D, member 2 [Source:MGI Symbol;Acc:MGI:2449205]	2.410306354	1.269216527	4.28E-08	3.58E-07	yes	up	14.07	29.3	11.61	13.9	20.98	9.82	14.04	32.51	23.28	29.52	32.78	28.41
ENSMUSG00000023034 Nr4a1	nuclear receptor subfamily 4, group A, member 1 [Source:MGI Symbol;Acc:MGI:1352454]	0.099360369	-3.331185665	3.91E-27	1.54E-25	yes	down	166.278	16.92	162.61	265.9	87.14	174.11	141.63	15.79	16.57	23.97	12.26	16.01

ENSMUSG00000026826	Nr4a2	nuclear receptor subfamily 4, group A, member 2 [Source:MGI Symbol;Acc:MGI:1352456]	0.427060824	-1.227486534	5.04E-14	7.96E-13	yes	down	7.646	5.254	5.93	6.59	3.81	5.82	16.08	17.77	2.76	1.9	1.93	1.91
ENSMUSG00000028341	Nr4a3	nuclear receptor subfamily 4, group A, member 3 [Source:MGI Symbol;Acc:MGI:1352457]	0.322288381	-1.633575915	0.00011204	0.00053679	yes	down	6.8	1.918	8.84	9.06	3.11	2.57	10.42	1.87	1.57	2.45	2.07	1.63
ENSMUSG00000026398	Nr5a2	nuclear receptor subfamily 5, group A, member 2 [Source:MGI Symbol;Acc:MGI:1346834]	2.663659119	1.413409466	9.56E-05	0.000464783	yes	up	0.348	0.826	0.3	0.23	0.65	0.3	0.26	0.5	1.35	0.71	0.77	0.8
ENSMUSG00000032311	Nrg4	neuregulin 4 [Source:MGI Symbol;Acc:MGI:1933833]	2.861765182	1.516905299	2.57E-06	1.66E-05	yes	up	1.296	2.838	1.02	1.26	2.65	0.92	0.63	2.48	3.35	3.02	3.22	2.12
ENSMUSG00000053310	Nrgn	neurogranin [Source:MGI Symbol;Acc:MGI:1927184]	0.424895906	-1.234818653	0.000147815	0.000693601	yes	down	12.706	4.438	9.05	27.62	7.25	9.58	10.03	6.67	4.25	2.92	4.04	4.31
ENSMUSG00000025969	Nrp2	neuropilin 2 [Source:MGI Symbol;Acc:MGI:1100492]	2.397504815	1.261533713	2.28E-82	5.31E-80	yes	up	18.702	37.526	17.48	19.25	18.23	20.45	18.1	32.94	37.95	38.53	38.02	40.19
ENSMUSG00000033768	Nrxn2	neurexin II [Source:MGI Symbol;Acc:MGI:1096362]	0.300373547	-1.735170332	2.88E-17	6.08E-16	yes	down	8.02	1.54	7.14	7.65	8.61	8.55	8.15	1.93	2.41	1.3	0.66	1.4
ENSMUSG00000066392	Nrxn3	neurexin III [Source:MGI Symbol;Acc:MGI:1096389]	13.94036762	3.801196701	0.001625083	0.006045297	yes	up	0.008	5.532	0	0	0.02	0	0.02	0.19	0.59	0.02	26.8	0.06
ENSMUSG00000062510	Nsl1	NSL1, MIS12 kinetochore complex component [Source:MGI Symbol;Acc:MGI:2685830]	2.252818221	1.171730908	1.95E-05	0.000107978	yes	up	0.566	1.662	0.35	0.45	0.71	0.37	0.95	1.96	1.06	2.18	1.11	2
ENSMUSG00000049107	Ntf3	neurotrophin 3 [Source:MGI Symbol;Acc:MGI:97380]	0.405987581	-1.300492499	3.06E-07	2.27E-06	yes	down	4.652	1.392	4.85	5.19	6.46	3.44	3.32	1.63	1.26	1.22	1.31	1.54
ENSMUSG00000031851	Ntpcr	nucleoside-triphosphatase, cancer-related [Source:MGI Symbol;Acc:MGI:1913816]	2.053503339	1.038087293	3.68E-15	6.43E-14	yes	up	10.138	18.968	10.5	10.23	9.25	9.16	11.55	15.78	20.04	19.62	19.05	20.35
ENSMUSG00000055254	Ntrk2	neurotrophic tyrosine kinase, receptor, type 2 [Source:MGI Symbol;Acc:MGI:97384]	2.183395495	1.12657348	0.00242071	0.008629716	yes	up	5	8.108	2.44	6.68	6.22	2.1	7.56	6.68	6.89	9.2	6.66	11.11
ENSMUSG00000026683	Nuf2	NUF2, NDC80 kinetochore complex component [Source:MGI Symbol;Acc:MGI:1914227]	2.318439387	1.213154009	1.81E-06	1.19E-05	yes	up	1.89	3.772	2.27	1.59	2.25	1.13	2.21	4.18	4.44	2.71	2.65	4.88
ENSMUSG00000061356	Nuggc	nuclear GTPase, germinal center associated [Source:MGI Symbol;Acc:MGI:2685446]	22.22822458	4.474320816	2.80E-19	6.81E-18	yes	up	0.086	1.522	0	0.16	0.07	0.16	0.04	2.25	0.99	1.36	1.02	1.99
ENSMUSG00000027306	Nusap1	nucleolar and spindle associated protein 1 [Source:MGI Symbol;Acc:MGI:2675669]	2.092313034	1.065098711	1.34E-06	9.04E-06	yes	up	2.412	4.44	2.74	1.44	3.01	1.89	2.98	4.36	5.25	3.26	3.74	5.59
ENSMUSG00000021396	Nxnl2	nucleoredoxin-like 2 [Source:MGI Symbol;Acc:MGI:1922374]	0.419399345	-1.253603485	0.016078576	0.045123678	yes	down	1.114	0.352	0.54	1.27	1.3	1.31	1.15	0.17	0.3	0.44	0.17	0.68
ENSMUSG00000047592	Nxpe5	neurexophilin and PC-esterase domain family, member 5 [Source:MGI Symbol;Acc:MGI:3584036]	8.953204145	3.162404082	1.59E-20	4.20E-19	yes	up	0.248	1.842	0.13	0.41	0.2	0.25	0.25	1.14	1.78	2.22	2.07	2
ENSMUSG00000046178	Nxph1	neurexophilin 1 [Source:MGI Symbol;Acc:MGI:107492]	0.320621095	-1.641058747	0.003719965	0.012599872	yes	down	0.628	0.176	0.89	0.69	0.21	0.58	0.77	0.13	0.14	0.04	0.17	0.4
ENSMUSG00000052776	Oas1a	2'-5' oligoadenylate synthetase 1A [Source:MGI Symbol;Acc:MGI:2180860]	2.189324313	1.130485682	1.39E-14	2.32E-13	yes	up	11.352	21.114	10.09	11.74	12.06	9.2	13.67	19.14	19.68	22.95	18.78	25.02
ENSMUSG00000032661	Oas3	2'-5' oligoadenylate synthetase 3 [Source:MGI	3.162306518	1.660977213	1.34E-17	2.88E-16	yes	up	1.276	3.362	1.31	1.12	1.2	1.49	1.26	3.92	2.29	3.32	2.76	4.52

		Symbol;Acc:MGI:2180850]																	
ENSMUSG00000047394	Odf3b	outer dense fiber of sperm tails 3B [Source:MGI Symbol;Acc:MGI:1917363]	0.489930645	-1.029350559	4.89E-05	0.00025022	yes	down	14.722	5.616	10.99	15.53	11.76	16.8	18.53	3.43	6.41	9.01	3.21	6.02
ENSMUSG00000045620	Odf3l1	outer dense fiber of sperm tails 3-like 1 [Source:MGI Symbol;Acc:MGI:2681875]	10.96232304	3.45448165	0.009758144	0.029400734	yes	up	0.038	0.348	0	0	0	0	0.19	0.06	0.79	0.35	0.31	0.23
ENSMUSG00000021390	Ogn	osteoglycin [Source:MGI Symbol;Acc:MGI:109278]	0.388063088	-1.365636882	2.95E-21	8.05E-20	yes	down	262.424	85.858	284.43	266.54	288.19	250.65	222.31	97.82	96.03	91.34	93.49	50.61
ENSMUSG00000032172	Olfm2	olfactomedin 2 [Source:MGI Symbol;Acc:MGI:3045350]	0.307239854	-1.702562724	1.08E-16	2.18E-15	yes	down	9.69	2.52	12.84	7.73	9.84	9.84	8.2	1.71	2.55	2.11	3.64	2.59
ENSMUSG00000046618	Olfml2a	olfactomedin-like 2A [Source:MGI Symbol;Acc:MGI:2444741]	0.474451672	-1.075666954	2.23E-29	9.93E-28	yes	down	14.64	5.86	14.62	12.88	13.05	18.04	14.61	5.35	6.24	5.8	5.73	6.18
ENSMUSG00000045392	Olfr1033	olfactory receptor 1033 [Source:MGI Symbol;Acc:MGI:3030867]	0.466759916	-1.099247423	3.87E-09	3.71E-08	yes	down	4.168	1.4	6.24	3.92	4.86	2.67	3.15	1.19	1.95	1.55	1.07	1.24
ENSMUSG00000090675	Olfr111	olfactory receptor 111 [Source:MGI Symbol;Acc:MGI:2177494]	21.76681033	4.444058108	0.001667804	0.006191312	yes	up	0.188	1.246	0	0	0	0	0.94	1.51	1.09	1.2	0.83	1.6
ENSMUSG00000057503	Olfr1459	olfactory receptor 1459 [Source:MGI Symbol;Acc:MGI:3031293]	2.346100182	1.23026462	0.004296784	0.014322167	yes	up	0.09	0.174	0.06	0.11	0.13	0.07	0.08	0.15	0.27	0.1	0.15	0.2
ENSMUSG00000111611	Olfr157	olfactory receptor 157 [Source:MGI Symbol;Acc:MGI:1352684]	24.34522305	4.605566814	0.005857815	0.018873273	yes	up	0	0.102	0	0	0	0	0	0.24	0.03	0.15	0	0.09
ENSMUSG00000060112	Olfr60	olfactory receptor 60 [Source:MGI Symbol;Acc:MGI:1333881]	8.67287351	3.116510069	0.000675092	0.002742368	yes	up	0.024	9.412	0	0	0.02	0.08	0.02	41.57	4.63	0.23	0.38	0.25
ENSMUSG00000035932	Olfr750	olfactory receptor 750 [Source:MGI Symbol;Acc:MGI:3030584]	0.28916613	-1.790029518	0.000632953	0.002582458	yes	down	3.22	1.01	3.78	2.01	2.19	2.84	5.28	0.29	1.75	0.77	1.67	0.57
ENSMUSG00000030162	Olr1	oxidized low density lipoprotein (lectin-like) receptor 1 [Source:MGI Symbol;Acc:MGI:1261434]	7.70569705	2.945925467	1.17E-55	1.35E-53	yes	up	6.532	36.752	5.06	8.53	10.01	2.74	6.32	33.89	40.31	34.98	39	35.58
ENSMUSG00000048368	Omd	osteomodulin [Source:MGI Symbol;Acc:MGI:1350918]	0.287898839	-1.796366123	1.90E-13	2.85E-12	yes	down	4.542	0.956	4.52	4.28	4.44	4.76	4.71	0.58	1.21	0.98	1.28	0.73
ENSMUSG00000045991	Onecut2	one cut domain, family member 2 [Source:MGI Symbol;Acc:MGI:1891408]	21.21186864	4.406799814	0.009068886	0.027613486	yes	up	0	0.012	0	0	0	0	0	0.02	0	0.01	0.02	0.01
ENSMUSG00000032346	Ooep	oocyte expressed protein [Source:MGI Symbol;Acc:MGI:1915218]	0.485462977	-1.04256682	0.014378829	0.040959963	yes	down	0.956	0.39	1.27	0.85	0.69	0.94	1.03	0.34	0.47	0.54	0.08	0.52
ENSMUSG00000041857	Oosp1	oocyte secreted protein 1 [Source:MGI Symbol;Acc:MGI:2149290]	9.31122171	3.218970474	0.001140578	0.004405954	yes	up	1.638	5.58	0	1.2	6.77	0	0.22	5.2	4.9	2.01	12.4	3.39
ENSMUSG00000039196	Orm1	orosomucoid 1 [Source:MGI Symbol;Acc:MGI:97443]	12.77801845	3.675592223	0.000101104	0.000489101	yes	up	2.756	29.826	0.36	6.52	5.34	0	1.56	28.99	27.13	29.13	25.73	38.15
ENSMUSG00000061540	Orm2	orosomucoid 2 [Source:MGI Symbol;Acc:MGI:97444]	30.91305151	4.950144168	8.50E-07	5.89E-06	yes	up	0.384	2.374	0	1.61	0	0	0.31	3.27	1.13	3.63	0.31	3.53
ENSMUSG00000058755	Osm	oncostatin M [Source:MGI Symbol;Acc:MGI:104749]	2.009597458	1.006906544	0.0001379	0.000650499	yes	up	2.454	3.876	3.13	3.56	2.6	1.31	1.67	2.67	3.27	5.4	3.7	4.34
ENSMUSG00000020787	P2rx1	purinergic receptor P2X, ligand-gated ion channel, 1 [Source:MGI Symbol;Acc:MGI:1098235]	0.402937108	-1.31137342	0.000115736	0.000553262	yes	down	2.016	0.712	1.53	2.9	2.5	2.12	1.03	0.41	0.8	0.76	0.85	0.74
ENSMUSG00000054293	P2ry10b	purinergic receptor P2Y, G-protein coupled 10B [Source:MGI Symbol;Acc:MGI:2441814]	2.570849414	1.362245107	2.00E-13	2.99E-12	yes	up	2.752	6.518	2.24	1.78	4.35	3.79	1.6	4.43	9.68	6.81	6.28	5.39
ENSMUSG00000036362	P2ry13	purinergic receptor P2Y, G-protein coupled 13 [Source:MGI Symbol;Acc:MGI:1921441]	2.315516777	1.21133421	2.47E-09	2.41E-08	yes	up	2.582	5.042	2.92	3.14	2.72	1.51	2.62	5.45	4.65	4.67	4.19	6.25
ENSMUSG00000036381	P2ry14	purinergic receptor P2Y, G-protein coupled, 14 [Source:MGI	2.620500271	1.389842258	7.10E-38	4.56E-36	yes	up	14.006	32.376	13.45	15.98	15.72	10.94	13.94	29.16	34.02	29.23	32.53	36.94

	Symbol;Acc:MGI:2155705]																		
ENSMUSG00000048779 P2ry6	pyrimidinergic receptor P2Y, G-protein coupled, 6 [Source:MGI Symbol;Acc:MGI:2673874]	3.931037952	1.974910292	3.84E-56	4.57E-54	yes	up	8.906	30.814	7.02	9.85	11.32	8.28	8.06	28.18	33.43	29.83	27.75	34.88
ENSMUSG00000051048 P4ha3	procollagen-proline, 2-oxoglutarate 4-dioxygenase (proline 4-hydroxylase), alpha polypeptide III [Source:MGI Symbol;Acc:MGI:2444049]	4.046245803	2.016583964	1.10E-12	1.52E-11	yes	up	0.906	3.214	1	0.88	0.96	0.84	0.85	1.86	3.06	3.36	2.38	5.41
ENSMUSG00000040276 Pacsin1	protein kinase C and casein kinase substrate in neurons 1 [Source:MGI Symbol;Acc:MGI:1345181]	3.165985498	1.662654647	5.46E-08	4.50E-07	yes	up	1.088	1.582	0.6	0.58	3.09	0.49	0.68	1.01	1.91	1.77	1.7	1.52
ENSMUSG00000031284 Pak3	p21 (RAC1) activated kinase 3 [Source:MGI Symbol;Acc:MGI:1339656]	0.412401615	-1.277878111	0.004120919	0.013809045	yes	down	0.306	0.16	0.23	0.56	0.12	0.25	0.37	0.32	0.15	0.1	0.14	0.09
ENSMUSG00000014301 Pam16	presequence translocase-asssociated motor 16 homolog (S. cerevisiae) [Source:MGI Symbol;Acc:MGI:1913699]	2.350450449	1.232937267	0.008309599	0.025620267	yes	up	0.87	2.656	0.31	1.54	1.55	0.62	0.33	3.19	3.66	2.86	1.21	2.36
ENSMUSG00000032278 Paqr5	progestin and adipoQ receptor family member V [Source:MGI Symbol;Acc:MGI:1921340]	0.396664926	-1.334007256	0.014630138	0.041554248	yes	down	0.618	0.208	0.64	0.35	0.2	0.51	1.39	0.09	0.45	0.2	0.05	0.25
ENSMUSG00000035365 Parpbp	PARP1 binding protein [Source:MGI Symbol;Acc:MGI:1922567]	2.713493833	1.44015163	7.02E-05	0.000350294	yes	up	0.468	0.876	0.34	0.42	0.26	0.91	0.41	0.59	0.74	1.54	0.43	1.08
ENSMUSG00000022439 Parvg	parvin, gamma [Source:MGI Symbol;Acc:MGI:2158329]	2.852881903	1.512420027	1.26E-44	1.04E-42	yes	up	8.148	20.352	7.23	9.21	9.06	7.7	7.54	17.58	20.55	18.96	19.63	25.04
ENSMUSG00000022033 Pbk	PDZ binding kinase [Source:MGI Symbol;Acc:MGI:1289156]	2.982195553	1.576374863	2.87E-08	2.47E-07	yes	up	1.708	4.636	1.69	1.36	2.28	1.67	1.54	4.7	4.92	2.53	3.52	7.51
ENSMUSG00000050505 Pcdh20	protocadherin 20 [Source:MGI Symbol;Acc:MGI:2443376]	0.167845487	-2.574794351	8.72E-10	8.94E-09	yes	down	0.514	0.084	0.39	0.67	0.54	0.42	0.55	0.11	0.03	0.05	0.11	0.12
ENSMUSG00000029108 Pcdh7	protocadherin 7 [Source:MGI Symbol;Acc:MGI:1860487]	2.226653541	1.154877098	6.13E-11	7.06E-10	yes	up	5.116	8.208	3.96	8.58	4.07	3.78	5.19	6.24	7.91	8.59	10.13	8.17
ENSMUSG00000103255 Pcdhac1	protocadherin alpha subfamily C, 1 [Source:MGI Symbol;Acc:MGI:1891442]	0.384321491	-1.379614441	0.000896611	0.003556553	yes	down	0.71	0.232	0.61	0.59	0.83	0.9	0.62	0.21	0.35	0.05	0.42	0.13
ENSMUSG00000102697 Pcdhac2	protocadherin alpha subfamily C, 2 [Source:MGI Symbol;Acc:MGI:1891443]	0.353196777	-1.501455915	1.78E-15	3.20E-14	yes	down	5.072	1.518	4.74	4.68	5.15	6.05	4.74	0.74	2.02	1.78	1.55	1.5
ENSMUSG00000104346 Pcdhga3	protocadherin gamma subfamily A, 3 [Source:MGI Symbol;Acc:MGI:1935215]	0.463319048	-1.109922099	7.48E-07	5.23E-06	yes	down	2.042	0.796	1.31	2.22	2.83	1.86	1.99	0.63	0.82	0.7	0.77	1.06
ENSMUSG00000103897 Pcdhga8	protocadherin gamma subfamily A, 8 [Source:MGI Symbol;Acc:MGI:1935221]	0.460168119	-1.11976706	7.18E-06	4.29E-05	yes	down	1.92	0.746	2.25	1.43	2.08	2.35	1.49	0.59	0.57	0.66	1.27	0.64
ENSMUSG00000027513 Pck1	phosphoenolpyruvate carboxykinase 1, cytosolic [Source:MGI Symbol;Acc:MGI:97501]	0.015285248	-6.031716222	0.012470629	0.036272033	yes	down	13.508	0.2	0.25	24.96	38.42	0	3.91	0	0	0.9	0	0.1
ENSMUSG00000040204 Pclaf	PCNA clamp associated factor [Source:MGI Symbol;Acc:MGI:1915276]	3.378729154	1.756480706	2.37E-10	2.57E-09	yes	up	3.654	8.352	4.04	4.81	4.82	1.78	2.82	10.73	6.29	7.72	4.01	13.01
ENSMUSG00000061601 Pclo	piccolo (presynaptic cytomatrix protein) [Source:MGI Symbol;Acc:MGI:1349390]	0.391500956	-1.352912265	4.25E-11	4.98E-10	yes	down	9.142	2.778	0.84	0.81	42.05	0.95	1.06	0.31	0.35	12.69	0.29	0.25

ENSMUSG00000060212	Pcnx2	pecanex homolog 2 [Source:MGI Symbol;Acc:MGI:2445010]	0.276186335	-1.856286152	1.02E-06	6.98E-06	yes	down	3.532	0.212	14.65	0.86	0.75	0.63	0.77	0.17	0.44	0.16	0.17	0.12
ENSMUSG00000015354	Pcolce2	procollagen C-endopeptidase enhancer 2 [Source:MGI Symbol;Acc:MGI:1923727]	0.410570884	-1.284296773	1.92E-38	1.27E-36	yes	down	122.53	34.94	129.27	101.84	129.09	144.41	108.04	28.63	34.46	33.54	49.78	28.29
ENSMUSG00000030513	Pcsk6	proprotein convertase subtilisin/kexin type 6 [Source:MGI Symbol;Acc:MGI:102897]	0.456313467	-1.131902864	4.89E-17	1.01E-15	yes	down	5.914	2.472	5.87	6.56	5.91	6.03	5.2	1.78	3.54	2.4	2.7	1.94
ENSMUSG00000026285	Pdcd1	programmed cell death 1 [Source:MGI Symbol;Acc:MGI:104879]	5.671579407	2.503750549	1.42E-21	3.92E-20	yes	up	1.214	5.832	1.4	0.92	1.29	0.81	1.65	6.7	5.21	4.91	3.95	8.39
ENSMUSG00000016498	Pdcd1lg2	programmed cell death 1 ligand 2 [Source:MGI Symbol;Acc:MGI:1930125]	6.28449124	2.651795956	1.79E-10	1.96E-09	yes	up	0.376	1.916	0.48	0.36	0.4	0.53	0.11	2.73	0.95	2	1.69	2.21
ENSMUSG00000041741	Pde3a	phosphodiesterase 3A, cGMP inhibited [Source:MGI Symbol;Acc:MGI:1860764]	0.475638856	-1.072061517	5.13E-24	1.67E-22	yes	down	23.968	9.584	26.92	22.49	22.56	27.88	19.99	10.28	9.93	8.34	11.23	8.14
ENSMUSG00000021684	Pde8b	phosphodiesterase 8B [Source:MGI Symbol;Acc:MGI:2443999]	0.471135577	-1.085785816	1.89E-18	4.31E-17	yes	down	19.964	8.364	20.8	18.68	17.35	25.02	17.97	7.36	8.79	8.12	10.13	7.42
ENSMUSG00000041119	Pde9a	phosphodiesterase 9A [Source:MGI Symbol;Acc:MGI:1277179]	0.455950667	-1.133050359	8.69E-13	1.21E-11	yes	down	45.586	17.544	55.11	36.15	40.33	41.62	54.72	13.23	21.4	15.48	21.72	15.89
ENSMUSG00000028019	Pdgfc	platelet-derived growth factor, C polypeptide [Source:MGI Symbol;Acc:MGI:1859631]	3.150311743	1.655494599	1.59E-12	2.17E-11	yes	up	2.03	5.346	2.45	2.47	2.12	0.85	2.26	4.36	5.24	5.42	6.45	5.26
ENSMUSG00000029231	Pdgfra	platelet derived growth factor receptor, alpha polypeptide [Source:MGI Symbol;Acc:MGI:97530]	0.492887033	-1.020671067	1.30E-21	3.60E-20	yes	down	98.138	40.628	104.19	79.4	91.12	119.4	96.58	39.41	51.01	38.23	40.13	34.36
ENSMUSG00000031595	Pdgfrl	platelet-derived growth factor receptor-like [Source:MGI Symbol;Acc:MGI:1916047]	2.365900273	1.242389263	1.00E-06	6.87E-06	yes	up	4.844	8.808	3.18	5.99	3.48	3.91	7.66	5.62	10.31	9.79	7.94	10.38
ENSMUSG00000019577	Pdk4	pyruvate dehydrogenase kinase, isoenzyme 4 [Source:MGI Symbol;Acc:MGI:1351481]	0.215191133	-2.216309463	2.79E-14	4.49E-13	yes	down	20.618	3.83	17.23	28.78	15.72	20.8	20.56	3.56	4.85	5.95	2.8	1.99
ENSMUSG00000031636	Pdlim3	PDZ and LIM domain 3 [Source:MGI Symbol;Acc:MGI:1859274]	0.409697446	-1.287369196	8.45E-08	6.76E-07	yes	down	15.006	5.462	14.08	22.82	7.88	14.55	15.7	4.03	5.33	7.25	6.35	4.35
ENSMUSG00000116165	Pdxp	pyridoxal (pyridoxine, vitamin B6) phosphatase [Source:MGI Symbol;Acc:MGI:1919282]	2.893388747	1.532760172	0.002956195	0.010299313	yes	up	0.386	0.95	0.38	0	0.28	0.71	0.56	1.29	0.75	0.85	0.92	0.94
ENSMUSG00000027400	Pdyn	prodynorphin [Source:MGI Symbol;Acc:MGI:97535]	13.50370329	3.755283204	0.003654373	0.012406794	yes	up	0.01	0.122	0	0	0.03	0	0.02	0.14	0.13	0.17	0.02	0.15
ENSMUSG00000028957	Per3	period circadian clock 3 [Source:MGI Symbol;Acc:MGI:1277134]	2.573268038	1.36360174	2.47E-16	4.82E-15	yes	up	3.506	7.276	3.11	2.79	3.72	4.47	3.44	8.06	5.3	7.22	7.4	8.4
ENSMUSG00000029373	Pf4	platelet factor 4 [Source:MGI Symbol;Acc:MGI:1888711]	2.765879383	1.467738244	3.98E-10	4.22E-09	yes	up	45.592	106.422	36.2	86.16	29.22	36.23	40.15	85.11	98.26	123.72	127.93	97.09
ENSMUSG00000073678	Pgap1	post-GPI attachment to proteins 1 [Source:MGI Symbol;Acc:MGI:2443342]	2.44074948	1.287324224	1.29E-28	5.56E-27	yes	up	2.328	4.76	2.01	2.38	2.62	2.63	2	5.46	4.38	4.15	5.17	4.64
ENSMUSG00000030413	Pglyrp1	peptidoglycan recognition protein 1 [Source:MGI Symbol;Acc:MGI:1345092]	2.675596707	1.419860675	0.010107455	0.030299727	yes	up	17.896	40.364	12.89	35.11	11.54	11.62	18.32	35.75	24.87	94.35	13.8	33.05
ENSMUSG00000041731	Pgm5	phosphoglucomutase 5 [Source:MGI Symbol;Acc:MGI:1925668]	0.387829876	-1.36650415	2.55E-18	5.78E-17	yes	down	16.87	5.786	14.25	20.67	13.49	20.4	15.54	4.88	5.91	8.65	5.11	4.38

ENSMUSG00000036062 Phf24	PHD finger protein 24 [Source:MGI Symbol;Acc:MGI:2140712]	0.270409918	-1.88678003	1.27E-29	5.74E-28	yes	down	4.186	1.384	3.8	4.29	3.87	4.05	4.92	0.55	2.89	0.89	1.18	1.41
ENSMUSG00000067780 Pi15	peptidase inhibitor 15 [Source:MGI Symbol;Acc:MGI:1934659]	0.361577644	-1.467622616	4.20E-33	2.27E-31	yes	down	8.424	2.556	10.01	9.13	6.79	8.66	7.53	2.32	3.05	2.82	2.08	2.51
ENSMUSG00000024011 Pi16	peptidase inhibitor 16 [Source:MGI Symbol;Acc:MGI:1921366]	0.40394268	-1.307777508	0.002394853	0.008546604	yes	down	16.148	5.662	12.18	21.69	11.56	13.56	21.75	3.42	4.67	12.51	1.46	6.25
ENSMUSG00000030329 Pianp	PILR alpha associated neural protein [Source:MGI Symbol;Acc:MGI:2441908]	17.31873981	4.114262052	2.14E-95	6.55E-93	yes	up	1.094	12.25	0.43	1.7	1.04	1.7	0.6	8.63	11.31	12.5	17.75	11.06
ENSMUSG00000041064 Pif1	PIF1 5'-to-3' DNA helicase [Source:MGI Symbol;Acc:MGI:2143057]	3.679670286	1.879576501	0.000309253	0.001358279	yes	up	0.208	0.9	0.24	0.14	0.21	0.38	0.07	1.21	0.73	0.25	0.73	1.58
ENSMUSG00000010136 Pifo	primary cilia formation [Source:MGI Symbol;Acc:MGI:1923670]	0.424002105	-1.237856668	1.74E-09	1.73E-08	yes	down	19.61	7.052	17.25	20.01	15.23	19.03	26.53	7.38	7.3	9.05	4.93	6.6
ENSMUSG00000042433 Pih1h3b	PIH1 domain containing 3B [Source:MGI Symbol;Acc:MGI:3607720]	0.445408428	-1.166799239	0.005092302	0.016670246	yes	down	1.6	0.602	1.4	2.29	1.24	1.01	2.06	0.52	0.71	0.82	0.2	0.76
ENSMUSG00000025017 Pik3ap1	phosphoinositide-3-kinase adaptor protein 1 [Source:MGI Symbol;Acc:MGI:1933177]	3.43659019	1.780977822	1.28E-66	2.05E-64	yes	up	20.674	60.238	19.89	19.4	24.73	17.44	21.91	51.81	67.64	61.27	59.76	60.71
ENSMUSG00000030228 Pik3c2g	phosphatidylinositol-4-phosphate 3-kinase catalytic subunit type 2 gamma [Source:MGI Symbol;Acc:MGI:1203730]	0.229439977	-2.12381131	1.12E-05	6.45E-05	yes	down	0.88	0.14	1.26	0.45	0.56	0.62	1.51	0.08	0.17	0.26	0.12	0.07
ENSMUSG00000039936 Pik3cd	phosphatidylinositol-4,5-bisphosphate 3-kinase catalytic subunit delta [Source:MGI Symbol;Acc:MGI:1098211]	2.048446662	1.034530328	6.79E-46	5.91E-44	yes	up	13.198	21.644	13.83	11.66	16.18	13.9	10.42	21.15	21.75	22.95	20.13	22.24
ENSMUSG00000020573 Pik3cg	phosphatidylinositol-4,5-bisphosphate 3-kinase catalytic subunit gamma [Source:MGI Symbol;Acc:MGI:1353576]	3.586908151	1.842740803	1.58E-73	3.23E-71	yes	up	4.422	13.376	4.08	5.15	4.81	4.11	3.96	14.93	13.07	13.44	11.94	13.5
ENSMUSG00000020901 Pik3r5	phosphoinositide-3-kinase regulatory subunit 5 [Source:MGI Symbol;Acc:MGI:2443588]	3.465720682	1.793155386	5.46E-89	1.52E-86	yes	up	11.444	34.404	10.68	11.1	9.21	13.09	13.14	33.18	38.28	30.99	35.3	34.27
ENSMUSG00000046245 Pilra	paired immunoglobin-like type 2 receptor alpha [Source:MGI Symbol;Acc:MGI:2450529]	2.764866604	1.467209877	3.49E-24	1.14E-22	yes	up	6.834	15.648	7.23	6.48	6.78	6.23	7.45	11.83	18.7	15	12.92	19.79
ENSMUSG00000066684 Pilrb1	paired immunoglobin-like type 2 receptor beta 1 [Source:MGI Symbol;Acc:MGI:2450532]	2.123518576	1.086456729	1.33E-05	7.56E-05	yes	up	3.56	5.76	4.59	3.25	3.05	2.87	4.04	7.81	5.97	2.55	6.81	5.66
ENSMUSG00000066682 Pilrb2	paired immunoglobin-like type 2 receptor beta 2 [Source:MGI Symbol;Acc:MGI:2450535]	3.418010531	1.773156842	9.28E-18	2.02E-16	yes	up	3.568	10.278	4.49	4.27	3	3.22	2.86	6.85	9.72	8.82	14.14	11.86
ENSMUSG00000035828 Pim3	proviral integration site 3 [Source:MGI Symbol;Acc:MGI:1355297]	0.404903728	-1.30434917	2.63E-26	9.84E-25	yes	down	71.194	24.734	75.39	77.57	64.38	69.41	69.22	28.21	25.04	25.85	27.03	17.54
ENSMUSG00000020808 Pimreg	PICALM interacting mitotic regulator [Source:MGI Symbol;Acc:MGI:1924434]	4.340574907	2.117886139	1.47E-08	1.31E-07	yes	up	1.238	4.324	0.58	1.26	1.18	0.79	2.38	4.42	5.27	1.66	4.09	6.18
ENSMUSG00000031379 Pir	pirin [Source:MGI Symbol;Acc:MGI:1916906]	0.498952044	-1.003026935	1.44E-14	2.39E-13	yes	down	24.54	10.73	26	28.8	20.21	26.42	21.27	11.07	10.33	12.02	9.28	10.95
ENSMUSG00000089942 Pira2	paired-Ig-like receptor A2 [Source:MGI Symbol;Acc:MGI:1195970]	5.353897192	2.420589436	3.99E-47	3.58E-45	yes	up	3.938	17.182	3.32	4.78	3.86	2.35	5.38	14.01	19.41	15.95	20.23	16.31
ENSMUSG00000058818 Pirb	paired Ig-like receptor B [Source:MGI Symbol;Acc:MGI:894311]	5.608253694	2.487551612	3.86E-117	2.07E-114	yes	up	12.872	59.516	9.65	15.17	14.19	11.97	13.38	57.69	64.27	54.65	53.92	67.05
ENSMUSG00000034416 Pkd1l2	polycystic kidney disease 1 like 2 [Source:MGI	28.35334316	4.825446949	0.000440584	0.001867684	yes	up	0	0.066	0	0	0	0	0	0.03	0.01	0.18	0.06	0.05

		Symbol;Acc:MGI:2664668]																		
ENSMUSG00000024247	Pkdcc	protein kinase domain containing, cytoplasmic [Source:MGI Symbol;Acc:MGI:2147077]	2.039881908	1.028485634	2.12E-12	2.86E-11	yes	up	5.85	10.49	6.26	6.56	7.96	3.79	4.68	11.34	7.86	12.01	11.03	10.21
ENSMUSG00000052496	Pkdrej	polycystin (PKD) family receptor for egg jelly [Source:MGI Symbol;Acc:MGI:1338786]	0.312035424	-1.680218274	0.000794545	0.00317818	yes	down	0.27	0.07	0.17	0.33	0.25	0.25	0.35	0.07	0.07	0.06	0.14	0.01
ENSMUSG00000043760	Pkhd1	polycystic kidney and hepatic disease 1 [Source:MGI Symbol;Acc:MGI:2155808]	2.488350833	1.315189905	0.00382552	0.01291618	yes	up	0.078	0.144	0.19	0.08	0.04	0.05	0.03	0.07	0.05	0.22	0.29	0.09
ENSMUSG00000038725	Pkhd1l1	polycystic kidney and hepatic disease 1-like 1 [Source:MGI Symbol;Acc:MGI:2183153]	0.358835903	-1.478603848	5.64E-06	3.43E-05	yes	down	3.416	1.202	3.87	2.9	4.53	2.73	3.05	1.77	0.75	1.29	1.63	0.57
ENSMUSG00000019876	Pkib	protein kinase inhibitor beta, cAMP dependent, testis specific [Source:MGI Symbol;Acc:MGI:101937]	3.121287261	1.642141139	9.71E-20	2.42E-18	yes	up	3.384	9.616	4.03	3.38	3.26	3.71	2.54	7.34	6.16	14.11	13.52	6.95
ENSMUSG00000035934	Pknox2	Pbx/knotted 1 homeobox 2 [Source:MGI Symbol;Acc:MGI:2445415]	0.459918435	-1.12055007	6.58E-05	0.000330451	yes	down	19.056	7.282	24.29	14.68	22.36	13.65	20.3	9.1	7.98	7.15	5.75	6.43
ENSMUSG00000002847	Pla1a	phospholipase A1 member A [Source:MGI Symbol;Acc:MGI:1934677]	4.263948976	2.092190174	1.14E-05	6.56E-05	yes	up	1.958	7.06	2.13	1.33	4.08	0.61	1.64	5.18	6.86	4.51	7.32	11.43
ENSMUSG00000031903	Pla2g15	phospholipase A2, group XV [Source:MGI Symbol;Acc:MGI:2178076]	3.227251913	1.690306196	1.19E-61	1.61E-59	yes	up	21.824	59.336	22.31	19.09	24.6	20.23	22.89	48.84	64.27	54.66	69.3	59.61
ENSMUSG00000041202	Pla2g2d	phospholipase A2, group IID [Source:MGI Symbol;Acc:MGI:1341796]	14.90670622	3.89788961	3.55E-100	1.27E-97	yes	up	2.646	35.694	1.44	2.22	4.43	2.15	2.99	38.34	33.94	36.92	37.03	32.24
ENSMUSG00000033847	Pla2g4c	phospholipase A2, group IVC (cytosolic, calcium-independent) [Source:MGI Symbol;Acc:MGI:1196403]	3.981860949	1.993442841	0.005375661	0.01750658	yes	up	0.064	0.22	0	0.14	0.04	0.07	0.07	0.24	0.12	0.42	0.1	0.22
ENSMUSG00000023913	Pla2g7	phospholipase A2, group VII (platelet-activating factor acetylhydrolase, plasma) [Source:MGI Symbol;Acc:MGI:1351327]	9.557809088	3.256679951	1.40E-45	1.20E-43	yes	up	12.98	102.568	11.3	17.57	11.65	12.78	11.6	142.31	81.53	79.42	92.48	117.1
ENSMUSG00000054580	Pla2r1	phospholipase A2 receptor 1 [Source:MGI Symbol;Acc:MGI:102468]	0.444203284	-1.170708037	9.34E-28	3.82E-26	yes	down	7.948	3.278	7.88	8.06	7.15	9.66	6.99	3.01	4.18	3.03	3.18	2.99
ENSMUSG00000051413	Plagl2	pleiomorphic adenoma gene-like 2 [Source:MGI Symbol;Acc:MGI:1933165]	2.135025712	1.094253444	3.08E-37	1.93E-35	yes	up	5.722	10.252	4.88	5.82	6.53	5.97	5.41	9.49	10.48	10.05	10.16	11.08
ENSMUSG00000086807	Platr21	pluripotency associated transcript 21 [Source:MGI Symbol;Acc:MGI:1925776]	0.234573486	-2.091888139	1.39E-05	7.88E-05	yes	down	2.292	0.422	2.47	2.32	2.66	2.58	1.43	0.5	0.18	0.91	0.23	0.29
ENSMUSG00000097448	Platr22	pluripotency associated transcript 22 [Source:MGI Symbol;Acc:MGI:4439804]	0.339775723	-1.557345319	0.000532251	0.002214198	yes	down	1.398	5.12	1.15	1.55	1.27	2.23	0.79	24.08	0.28	0.14	0.44	0.66
ENSMUSG00000030214	Plbd1	phospholipase B domain containing 1 [Source:MGI Symbol;Acc:MGI:1914107]	2.00193681	1.001396437	1.26E-15	2.28E-14	yes	up	36.242	60.818	30.47	41.08	42.04	33.55	34.07	67.83	55.15	67.41	47.59	66.11
ENSMUSG00000024998	Plce1	phospholipase C, epsilon 1 [Source:MGI Symbol;Acc:MGI:1921305]	0.43717909	-1.193703697	9.86E-22	2.76E-20	yes	down	12.008	4.27	12.32	11.93	10.92	13.58	11.29	3.72	4.28	4.04	5.83	3.48
ENSMUSG00000034330	Plcg2	phospholipase C, gamma 2 [Source:MGI Symbol;Acc:MGI:97616]	2.665802999	1.41457017	1.70E-41	1.24E-39	yes	up	9.256	20.77	8.97	9.6	9.51	9.88	8.32	22.37	18.68	20.57	17.55	24.68
ENSMUSG00000038910	Plcl2	phospholipase C-like 2 [Source:MGI Symbol;Acc:MGI:1352756]	2.773659759	1.471790825	1.53E-42	1.17E-40	yes	up	4.026	9.396	4.4	4.56	4	4.06	3.11	9.94	9.33	9.61	9.31	8.79

ENSMUSG00000064247	Plcxd1	phosphatidylinositol-specific phospholipase C, X domain containing 1 [Source:MGI Symbol;Acc:MGI:2685422]	2.002923208	1.002107109	0.000836543	0.003333097	yes	up	0.792	1.352	0.8	1.03	0.37	1.13	0.63	1.06	0.84	1.77	1.2	1.89
ENSMUSG00000003363	Pld3	phospholipase D family, member 3 [Source:MGI Symbol;Acc:MGI:1333782]	5.074908035	2.343381679	2.48E-154	2.79E-151	yes	up	54.928	229.79	54.63	54.22	59.56	49.02	57.21	207.23	244.59	198.34	247.77	251.02
ENSMUSG00000052160	Pld4	phospholipase D family, member 4 [Source:MGI Symbol;Acc:MGI:2144765]	3.069648947	1.618073675	3.59E-41	2.56E-39	yes	up	26.04	64.376	24.41	28.89	27.04	27.46	22.4	62.05	63.45	62.12	53.41	80.85
ENSMUSG00000020120	Plek	pleckstrin [Source:MGI Symbol;Acc:MGI:1860485]	3.095263346	1.63006216	4.59E-46	4.05E-44	yes	up	28.686	74.912	28.16	36.29	31.26	23.15	24.57	79.97	72.17	70.97	73.26	78.19
ENSMUSG00000028917	Plekhm2	pleckstrin homology domain containing, family M (with RUN domain) member 2 [Source:MGI Symbol;Acc:MGI:1916832]	2.250165781	1.170031296	4.88E-41	3.45E-39	yes	up	15.342	35.096	14.27	13.42	18.67	13.51	16.84	26.87	43.95	38.87	33.02	32.77
ENSMUSG00000050721	Plekho2	pleckstrin homology domain containing, family O member 2 [Source:MGI Symbol;Acc:MGI:2143132]	2.351976564	1.233873685	4.67E-36	2.82E-34	yes	up	16.538	32.56	14.64	16.44	18.88	18	14.73	31.92	30.59	28.48	32.99	38.82
ENSMUSG00000028494	Plin2	perilipin 2 [Source:MGI Symbol;Acc:MGI:87920]	3.171824057	1.665312746	2.35E-41	1.69E-39	yes	up	116.842	308.198	121.91	111.12	133.55	93.44	124.19	279.23	361.65	241.19	374.23	284.69
ENSMUSG00000002831	Plin4	perilipin 4 [Source:MGI Symbol;Acc:MGI:1929709]	0.227964385	-2.133119645	0.000616655	0.002522394	yes	down	4.28	0.806	2.12	5.65	8.63	1.84	3.16	0.64	0.59	2.02	0.28	0.5
ENSMUSG00000030867	Plk1	polo like kinase 1 [Source:MGI Symbol;Acc:MGI:97621]	3.415394503	1.77205223	4.07E-10	4.31E-09	yes	up	1.544	3.854	1.05	0.76	1.7	1.78	2.43	4.44	2.97	3.84	2.55	5.47
ENSMUSG00000031425	Plp1	proteolipid protein (myelin) 1 [Source:MGI Symbol;Acc:MGI:97623]	0.460108924	-1.119952657	0.000447143	0.001892867	yes	down	1.354	0.6	1.34	2.24	0.71	1.53	0.95	0.8	0.44	0.69	0.55	0.52
ENSMUSG00000021759	Plpp1	phospholipid phosphatase 1 [Source:MGI Symbol;Acc:MGI:108412]	0.485911094	-1.041235724	7.53E-16	1.39E-14	yes	down	162.59	67.384	184.52	157.04	152.9	140.25	178.24	51.63	87.51	54.95	73.75	69.08
ENSMUSG00000028517	Plpp3	phospholipid phosphatase 3 [Source:MGI Symbol;Acc:MGI:1915166]	0.480847947	-1.056347336	2.44E-26	9.17E-25	yes	down	138.712	56.166	136.27	111.59	134.7	175.4	135.6	59.27	58.95	58.92	52.51	51.18
ENSMUSG00000034845	Plvap	plasmalemma vesicle associated protein [Source:MGI Symbol;Acc:MGI:1890497]	0.454578613	-1.137398286	7.10E-18	1.56E-16	yes	down	362.038	139.154	442.8	274.51	343.69	406.05	343.14	121.22	169.16	109.62	167.66	128.11
ENSMUSG00000031385	Plxnb3	plexin B3 [Source:MGI Symbol;Acc:MGI:2154240]	2.160395475	1.111295432	0.003720069	0.012599872	yes	up	0.296	0.57	0.09	0.56	0.27	0.18	0.38	0.31	0.47	0.67	0.71	0.69
ENSMUSG00000052468	Pmp2	peripheral myelin protein 2 [Source:MGI Symbol;Acc:MGI:102667]	0.045663126	-4.452826563	0.010682805	0.031766585	yes	down	0.2	0	0.26	0.16	0.54	0	0.04	0	0	0	0	0
ENSMUSG00000036833	Pnpla7	patatin-like phospholipase domain containing 7 [Source:MGI Symbol;Acc:MGI:2385325]	3.773689349	1.915975665	7.31E-131	5.59E-128	yes	up	44.6	71.076	41.51	37.92	66.38	48.32	28.87	67.02	85.16	62.72	75	65.48
ENSMUSG00000012889	Podnl1	podocan-like 1 [Source:MGI Symbol;Acc:MGI:2685352]	2.341429511	1.227389606	0.000255322	0.001142255	yes	up	0.956	1.828	0.65	1.81	0.75	0.89	0.68	2.19	1.68	1.22	0.89	3.16
ENSMUSG00000002588	Pon1	paraoxonase 1 [Source:MGI Symbol;Acc:MGI:103295]	0.143064371	-2.805263672	1.61E-69	2.90E-67	yes	down	80.598	10.412	92.13	70.75	71.99	81.72	86.4	13.56	8.78	13.3	7.54	8.88
ENSMUSG00000032053	Pou2af1	POU domain, class 2, associating factor 1 [Source:MGI Symbol;Acc:MGI:105086]	3.050577436	1.609082353	3.72E-19	8.98E-18	yes	up	10.086	25.676	9.68	11.44	11.84	10.86	6.61	35.84	21.66	23.31	18.26	29.31
ENSMUSG00000008496	Pou2f2	POU domain, class 2, transcription factor 2 [Source:MGI Symbol;Acc:MGI:101897]	2.973048168	1.571942837	9.86E-16	1.80E-14	yes	up	2.008	4.534	1.4	2.89	1.94	2.76	1.05	5.35	4.71	3.64	3.88	5.09
ENSMUSG00000000440	Pparg	peroxisome proliferator activated receptor gamma [Source:MGI Symbol;Acc:MGI:97747]	2.647444385	1.404600378	8.45E-12	1.07E-10	yes	up	10.732	24.688	9.7	11.28	17.04	5.56	10.08	18.31	26.89	27.71	26.41	24.12

ENSMUSG00000029167 Ppargc1a	peroxisome proliferative activated receptor, gamma, coactivator 1 alpha [Source:MGI Symbol;Acc:MGI:1342774]	0.462995103	-1.110931159	2.50E-06	1.62E-05	yes	down	2.348	0.67	1.46	4.11	1.14	2.7	2.33	0.81	0.63	0.96	0.47	0.48
ENSMUSG00000033871 Ppargc1b	peroxisome proliferative activated receptor, gamma, coactivator 1 beta [Source:MGI Symbol;Acc:MGI:2444934]	2.53145518	1.339966941	6.98E-05	0.000348656	yes	up	0.334	0.724	0.21	0.34	0.3	0.39	0.43	0.76	1.07	0.67	0.39	0.73
ENSMUSG00000029372 Ppbp	pro-platelet basic protein [Source:MGI Symbol;Acc:MGI:1888712]	0.221087484	-2.17731074	1.42E-17	3.06E-16	yes	down	38.262	7.158	36.45	66.85	27.42	36.51	24.08	8.38	9.18	4.1	8.07	6.06
ENSMUSG00000053825 Ppfia2	protein tyrosine phosphatase, receptor type, f polypeptide (PTPRF), interacting protein (liprin), alpha 2 [Source:MGI Symbol;Acc:MGI:2443834]	0.341095149	-1.551753857	0.00128234	0.004889192	yes	down	0.24	0.074	0.3	0.22	0.37	0.12	0.19	0.06	0.1	0.06	0.11	0.04
ENSMUSG00000026458 Ppfia4	protein tyrosine phosphatase, receptor type, f polypeptide (PTPRF), interacting protein (liprin), alpha 4 [Source:MGI Symbol;Acc:MGI:1915757]	3.630486246	1.860162787	1.10E-29	5.02E-28	yes	up	4.168	9.966	5.04	3.24	6.3	4.12	2.14	11	9.22	9.82	7.42	12.37
ENSMUSG00000078451 Ppil6	peptidylprolyl isomerase (cyclophilin)-like 6 [Source:MGI Symbol;Acc:MGI:1920325]	0.443331741	-1.173541438	0.00014208	0.000668451	yes	down	11.718	4.252	9.68	12.4	11.18	12.02	13.31	1.96	3.94	7.55	2.4	5.41
ENSMUSG00000030402 Ppm1n	protein phosphatase, Mg2+/Mn2+ dependent, 1N (putative) [Source:MGI Symbol;Acc:MGI:2142330]	23.18420893	4.535070596	9.52E-06	5.55E-05	yes	up	0.022	0.634	0	0	0	0.04	0.07	0.36	0.47	0.7	0.69	0.95
ENSMUSG00000037166 Ppp1r14a	protein phosphatase 1, regulatory inhibitor subunit 14A [Source:MGI Symbol;Acc:MGI:1931139]	0.437228121	-1.193541903	1.62E-08	1.44E-07	yes	down	42.314	15.436	32.04	30.99	34	48.5	66.04	22.06	14.67	14.98	9.37	16.1
ENSMUSG00000022490 Ppp1r1a	protein phosphatase 1, regulatory inhibitor subunit 1A [Source:MGI Symbol;Acc:MGI:1889595]	0.259873678	-1.944117581	0.000403025	0.001723478	yes	down	1.834	0.438	0.69	2.68	2.61	1.26	1.93	0.19	0.39	1.02	0.24	0.35
ENSMUSG00000035179 Ppp1r32	protein phosphatase 1, regulatory subunit 32 [Source:MGI Symbol;Acc:MGI:1915002]	0.449520076	-1.153542544	0.004636093	0.01534094	yes	down	2.906	0.954	2.39	5.18	1.44	2.18	3.34	0.61	1.46	1.26	0.24	1.2
ENSMUSG00000024500 Ppp2r2b	protein phosphatase 2, regulatory subunit B, beta [Source:MGI Symbol;Acc:MGI:1920180]	0.29615776	-1.755562205	8.17E-10	8.38E-09	yes	down	2.364	0.602	2.24	3.26	1.53	2.61	2.18	0.61	0.62	0.78	0.38	0.62
ENSMUSG00000029120 Ppp2r2c	protein phosphatase 2, regulatory subunit B, gamma [Source:MGI Symbol;Acc:MGI:2442660]	0.382302281	-1.387214288	2.85E-06	1.83E-05	yes	down	2.62	0.844	3.55	2.47	1.87	3.47	1.74	0.32	1.13	0.87	1.2	0.7
ENSMUSG00000021209 Ppp4r4	protein phosphatase 4, regulatory subunit 4 [Source:MGI Symbol;Acc:MGI:1921771]	0.459249336	-1.122650459	2.11E-06	1.38E-05	yes	down	3.67	1.672	3.45	3.39	3.84	5.2	2.47	1.68	2.05	1.42	2.44	0.77
ENSMUSG00000038943 Prc1	protein regulator of cytokinesis 1 [Source:MGI Symbol;Acc:MGI:1858961]	2.527735923	1.337845751	1.08E-10	1.22E-09	yes	up	4.38	8.578	4.26	3.71	5.73	3.48	4.72	8.79	7.47	8.2	6.67	11.76
ENSMUSG00000061119 Prcp	prolylcarboxypeptidase (angiotensinase C) [Source:MGI Symbol;Acc:MGI:1919711]	2.1909388	1.131549186	8.50E-38	5.45E-36	yes	up	31.916	59.024	33.44	34.48	33.8	28.09	29.77	53.27	59.26	56.73	61.12	64.74
ENSMUSG00000027073 Prg2	proteoglycan 2, bone marrow [Source:MGI Symbol;Acc:MGI:103294]	8.855700376	3.146606411	8.88E-24	2.85E-22	yes	up	2.612	20.046	2.34	4.7	2.56	0.57	2.89	24.87	20.26	13.42	20.53	21.15

ENSMUSG00000006014 Prg4	proteoglycan 4 (megakaryocyte stimulating factor, articular superficial zone protein) [Source:MGI Symbol;Acc:MGI:1891344]	2.213846177	1.146554984	0.000529565	0.002204309	yes	up	0.982	1.686	1.04	0.53	1.38	0.89	1.07	1.13	2.17	0.89	3.4	0.84
ENSMUSG00000041669 Prima1	proline rich membrane anchor 1 [Source:MGI Symbol;Acc:MGI:1926097]	0.043358232	-4.527550248	0.009274784	0.0281703	yes	down	0.39	0	0.1	0.2	0.58	1.07	0	0	0	0	0	0
ENSMUSG00000006542 Prkag3	protein kinase, AMP-activated, gamma 3 non-catalytic subunit [Source:MGI Symbol;Acc:MGI:1891343]	0.391808027	-1.351781139	1.19E-09	1.20E-08	yes	down	3.012	1.038	2.57	2.72	2.66	3.77	3.34	0.6	1.43	0.85	1.16	1.15
ENSMUSG00000052889 Prkcb	protein kinase C, beta [Source:MGI Symbol;Acc:MGI:97596]	2.563340709	1.358025249	3.53E-20	9.14E-19	yes	up	11.472	25.254	11.01	14.39	11.4	14.3	6.26	26.52	21.48	24.63	26.32	27.32
ENSMUSG00000021948 Prkcd	protein kinase C, delta [Source:MGI Symbol;Acc:MGI:97598]	2.169740222	1.117522322	4.06E-34	2.28E-32	yes	up	55.578	101.466	53.22	46.82	56.88	64.27	56.7	106.47	111.71	95.11	86.54	107.5
ENSMUSG00000029334 Prkg2	protein kinase, cGMP-dependent, type II [Source:MGI Symbol;Acc:MGI:108173]	0.242108403	-2.046274942	8.20E-31	3.95E-29	yes	down	9.32	2.07	10.95	6.45	10.27	9.89	9.04	2.45	3.09	1.79	1.88	1.14
ENSMUSG00000027338 Prnd	prion like protein doppel [Source:MGI Symbol;Acc:MGI:1346999]	3.00685678	1.588256152	3.42E-10	3.65E-09	yes	up	1.452	3.57	1.74	1.42	1.92	1.11	1.07	2.01	4.04	2.66	4.21	4.93
ENSMUSG00000027611 Procr	protein C receptor, endothelial [Source:MGI Symbol;Acc:MGI:104596]	7.058941434	2.819451852	4.38E-62	6.09E-60	yes	up	5.244	28.272	7.17	5.17	4.93	4.93	4.02	31.9	24.38	24.46	25.17	35.45
ENSMUSG00000030069 Prok2	prokineticin 2 [Source:MGI Symbol;Acc:MGI:1354178]	68.93776643	6.107222651	1.11E-07	8.77E-07	yes	up	0	0.65	0	0	0	0	0	0.94	0.87	0.23	0.34	0.87
ENSMUSG00000027376 Prom2	prominin 2 [Source:MGI Symbol;Acc:MGI:2138997]	0.150895116	-2.728381985	2.67E-07	2.00E-06	yes	down	5.868	0.058	0.44	0.2	27.4	0.65	0.65	0.02	0.1	0.02	0.1	0.05
ENSMUSG00000031445 Proz	protein Z, vitamin K-dependent plasma glycoprotein [Source:MGI Symbol;Acc:MGI:1860488]	2.30126611	1.202427821	3.06E-05	0.000163143	yes	up	1.782	3.474	1.34	2.83	1.5	0.7	2.54	3.31	3.84	3.75	3.57	2.9
ENSMUSG00000020493 Prr11	proline rich 11 [Source:MGI Symbol;Acc:MGI:2444496]	2.367408487	1.243308659	6.67E-07	4.70E-06	yes	up	0.964	1.934	0.55	0.74	1.07	1.24	1.22	2.31	1.8	1.4	1.74	2.42
ENSMUSG00000043795 Prr33	proline rich 33 [Source:MGI Symbol;Acc:MGI:3642289]	2.310134075	1.207976585	7.34E-05	0.000365058	yes	up	0.992	1.94	0.99	0.68	0.89	1.43	0.97	2.65	2.68	1.55	1.08	1.74
ENSMUSG00000036106 Prr5	proline rich 5 (renal) [Source:MGI Symbol;Acc:MGI:1924714]	2.202830675	1.139358604	2.82E-11	3.37E-10	yes	up	4.83	8.778	4.85	4.55	4.31	5.67	4.77	11.53	7.64	9.12	7.43	8.17
ENSMUSG00000079654 Prrt4	proline-rich transmembrane protein 4 [Source:MGI Symbol;Acc:MGI:2141677]	0.38103432	-1.392007148	1.29E-17	2.79E-16	yes	down	3.712	1.196	3.51	3.01	4.26	4.35	3.43	1.24	1.18	1.06	1.45	1.05
ENSMUSG00000006179 Prss16	protease, serine 16 (thymus) [Source:MGI Symbol;Acc:MGI:1859181]	3.26963926	1.709131472	0.003448921	0.011780239	yes	up	0.308	0.662	0.09	0.37	0.34	0.1	0.64	0.69	1.03	0.59	0.56	0.44
ENSMUSG00000039405 Prss23	protease, serine 23 [Source:MGI Symbol;Acc:MGI:1923703]	0.439334412	-1.186608587	1.69E-31	8.36E-30	yes	down	106.374	38.008	122.18	92.48	106.18	104	107.03	33.49	45.3	31.61	48.18	31.46
ENSMUSG00000024124 Prss30	protease, serine 30 [Source:MGI Symbol;Acc:MGI:1353645]	2.9026082	1.537349847	0.000408929	0.001745592	yes	up	0.796	1.844	0.59	0.86	0.94	0.72	0.87	0.48	2.91	2.21	1.45	2.17
ENSMUSG00000033491 Prss35	protease, serine 35 [Source:MGI Symbol;Acc:MGI:2444800]	2.267100542	1.180848373	0.004146089	0.013882526	yes	up	0.468	0.854	0.46	0.89	0.29	0.31	0.39	0.29	1.36	1.03	0.64	0.95
ENSMUSG00000057729 Prtn3	proteinase 3 [Source:MGI Symbol;Acc:MGI:893580]	0.220818529	-2.179066858	0.013456426	0.038682259	yes	down	1.614	0.244	0.79	1.14	2.32	3.12	0.7	0	0.41	0.08	0.31	0.42
ENSMUSG00000039126 Prune2	prune homolog 2 [Source:MGI Symbol;Acc:MGI:1925004]	3.206402561	1.680955566	3.70E-22	1.07E-20	yes	up	4	11.16	1.78	2.85	10.59	1.75	3.03	7.95	9.86	15.84	12.71	9.44
ENSMUSG00000004207 Psap	prosaposin [Source:MGI Symbol;Acc:MGI:97783]	5.00737757	2.324055243	6.20E-235	3.32E-231	yes	up	575.78	2443.082	562.63	586.41	615.42	547.97	566.47	2240.95	2528.05	2186.74	2519.74	2739.93
ENSMUSG00000026979 Psd4	pleckstrin and Sec7 domain containing 4 [Source:MGI Symbol;Acc:MGI:2674093]	2.343588889	1.228719515	4.12E-33	2.23E-31	yes	up	8.652	18.436	8.31	8.48	10.05	9.07	7.35	19.31	20.02	16.29	16.25	20.31
ENSMUSG00000024338 Psmb8	proteasome (prosome, macropain) subunit, beta type 8 (large	2.260030535	1.176342265	6.01E-22	1.71E-20	yes	up	109.21	209.484	94.81	104.53	120.65	123.75	102.31	193.72	185.7	204.47	183.49	280.04

	multifunctional peptidase 7) [Source:MGI Symbol;Acc:MGI:1346527]																			
ENSMUSG00000096727 Psmb9	proteasome (prosome, macropain) subunit, beta type 9 (large multifunctional peptidase 2) [Source:MGI Symbol;Acc:MGI:1346526]	2.103291327	1.072648692	4.25E-15	7.38E-14	yes	up	36.384	62.76	32.33	34.7	38.77	39.39	36.73	61.98	54.28	56.01	53.77	87.76
ENSMUSG00000068744 Psrc1	proline/serine-rich coiled-coil 1 [Source:MGI Symbol;Acc:MGI:1913099]	3.814285103	1.931412683	5.55E-17	1.14E-15	yes	up	1.55	4.78	1.03	1.17	2.43	1.49	1.63	4.53	4.82	3.67	5.82	5.06
ENSMUSG00000032322 Pstpip1	proline-serine-threonine phosphatase-interacting protein 1 [Source:MGI Symbol;Acc:MGI:1321396]	2.8886441	1.530392466	3.86E-32	2.00E-30	yes	up	6.718	16.43	6.67	8.44	6.85	7.01	4.62	16.18	17.72	15.31	14.44	18.5
ENSMUSG00000025429 Pstpip2	proline-serine-threonine phosphatase-interacting protein 2 [Source:MGI Symbol;Acc:MGI:1335088]	2.301024707	1.202276474	1.00E-15	1.83E-14	yes	up	4.206	8.118	3.66	5.63	3.32	4.52	3.9	7.66	7.9	8.17	7.78	9.08
ENSMUSG00000056529 Ptafr	platelet-activating factor receptor [Source:MGI Symbol;Acc:MGI:106066]	6.12765158	2.615334268	2.55E-84	6.27E-82	yes	up	2.8	14.05	3.17	3.51	2.79	2.14	2.39	14.05	13.16	13.78	12.89	16.37
ENSMUSG00000037759 Ptger2	prostaglandin E receptor 2 (subtype EP2) [Source:MGI Symbol;Acc:MGI:97794]	2.762965776	1.466217692	1.44E-20	3.81E-19	yes	up	2.11	4.826	1.74	2.33	1.98	2.8	1.7	5.07	5.58	3.71	5.09	4.68
ENSMUSG00000039942 Ptger4	prostaglandin E receptor 4 (subtype EP4) [Source:MGI Symbol;Acc:MGI:104311]	2.874730796	1.523426861	3.81E-15	6.65E-14	yes	up	2.616	6.354	1.79	3.01	3.49	2.09	2.7	7.31	5.8	6.13	4.81	7.72
ENSMUSG00000028036 Ptgfr	prostaglandin F receptor [Source:MGI Symbol;Acc:MGI:97796]	0.389061922	-1.361928305	8.53E-23	2.59E-21	yes	down	4.606	1.714	4.8	6.16	3.45	4.42	4.2	1.79	2.06	2.13	1.43	1.16
ENSMUSG00000043017 Ptgir	prostaglandin I receptor (IP) [Source:MGI Symbol;Acc:MGI:99535]	6.788791686	2.763154817	4.28E-97	1.41E-94	yes	up	2.002	10.866	1.8	2.06	2.57	2.19	1.39	9.47	10.65	10.4	11.67	12.14
ENSMUSG00000032492 Pth1r	parathyroid hormone 1 receptor [Source:MGI Symbol;Acc:MGI:97801]	2.003407433	1.002455852	6.93E-07	4.85E-06	yes	up	1.94	3.034	1.91	1.65	1.97	1.55	2.62	2.59	2.68	2.56	3.44	3.9
ENSMUSG00000059456 Ptk2b	PTK2 protein tyrosine kinase 2 beta [Source:MGI Symbol;Acc:MGI:104908]	2.82191733	1.496675724	2.37E-73	4.79E-71	yes	up	17.64	42.71	17.45	17.55	18.86	18.05	16.29	45.28	41.63	42.18	38.46	46
ENSMUSG00000029838 Ptn	pleiotrophin [Source:MGI Symbol;Acc:MGI:97804]	0.3377923	-1.565791651	3.34E-05	0.000176322	yes	down	2.286	0.652	1.91	2.88	2.37	2.2	2.07	0.33	0.45	0.96	0.33	1.19
ENSMUSG00000030854 Ptpn5	protein tyrosine phosphatase, non-receptor type 5 [Source:MGI Symbol;Acc:MGI:97807]	2.842594437	1.507208279	0.001749655	0.006464908	yes	up	0.24	0.468	0.3	0.49	0.08	0.21	0.12	0.5	0.34	0.51	0.48	0.51
ENSMUSG00000004266 Ptpn6	protein tyrosine phosphatase, non-receptor type 6 [Source:MGI Symbol;Acc:MGI:96055]	2.592443634	1.374312622	9.33E-48	8.57E-46	yes	up	37.756	83.566	37.38	40.06	41.99	36.54	32.81	79.52	79.62	80.82	78.05	99.82
ENSMUSG00000031506 Ptpn7	protein tyrosine phosphatase, non-receptor type 7 [Source:MGI Symbol;Acc:MGI:2156893]	4.982729042	2.316936123	6.95E-67	1.14E-64	yes	up	3.366	14.852	3.22	2.88	4.07	3.59	3.07	16.11	14.1	11.24	14.59	18.22
ENSMUSG00000026395 Ptprc	protein tyrosine phosphatase, receptor type, C [Source:MGI Symbol;Acc:MGI:97810]	2.346854656	1.230728497	3.06E-45	2.59E-43	yes	up	56.534	113.16	56.23	60.32	59.04	61.93	45.15	124.92	113.89	117.11	105.08	104.8
ENSMUSG00000030223 Ptpro	protein tyrosine phosphatase, receptor type, O [Source:MGI Symbol;Acc:MGI:1097152]	3.132982841	1.647536868	3.49E-33	1.90E-31	yes	up	3.918	10.494	3.29	4.31	2.96	4.94	4.09	9.02	11.85	9.95	8.52	13.13

ENSMUSG00000097993 Ptprv	protein tyrosine phosphatase, receptor type, V [Source:MGI Symbol;Acc:MGI:108027]	3.591807251	1.844709932	1.21E-05	6.93E-05	yes	up	0.768	1.416	0.2	0.22	3.21	0.12	0.09	2.39	2.73	1.31	0.17	0.48
ENSMUSG00000053746 Ptrh1	peptidyl-tRNA hydrolase 1 homolog [Source:MGI Symbol;Acc:MGI:1913779]	2.19785926	1.136099006	3.49E-07	2.56E-06	yes	up	5.364	8.424	5.91	7.34	4.56	4.83	4.18	10.01	7.02	7.96	4.71	12.42
ENSMUSG00000027832 Ptx3	pentraxin related gene [Source:MGI Symbol;Acc:MGI:104641]	3.689102508	1.883269877	1.28E-07	9.97E-07	yes	up	0.576	1.67	0.91	0.75	0.39	0.43	0.4	1.7	1.31	1.49	1.44	2.41
ENSMUSG00000109713 Pvrig	poliovirus receptor related immunoglobulin domain containing [Source:MGI Symbol;Acc:MGI:5596028]	4.341100686	2.118060884	8.57E-07	5.93E-06	yes	up	0.566	2.092	0.22	0.54	0.53	1.16	0.38	2.06	2.61	1.77	1.74	2.28
ENSMUSG00000021411 Pxdc1	PX domain containing 1 [Source:MGI Symbol;Acc:MGI:1914145]	0.496602679	-1.009836052	7.77E-15	1.32E-13	yes	down	191.986	78.92	233.66	154.5	191.45	213.86	166.46	96.05	82.29	76.59	85.69	53.98
ENSMUSG00000030793 Pycard	PYD and CARD domain containing [Source:MGI Symbol;Acc:MGI:1931465]	3.529507098	1.819466723	2.66E-42	1.99E-40	yes	up	13.056	38.762	14.28	13.74	12.19	12.21	12.86	38.58	31.14	36.81	37.45	49.83
ENSMUSG00000025140 Pycr1	pyrroline-5-carboxylate reductase 1 [Source:MGI Symbol;Acc:MGI:2384795]	2.352928719	1.234457615	3.33E-07	2.45E-06	yes	up	1.944	3.746	1.74	1.12	2.22	2.55	2.09	2.98	5.48	2.42	2.4	5.45
ENSMUSG00000032648 Pygm	muscle glycogen phosphorylase [Source:MGI Symbol;Acc:MGI:97830]	0.429115297	-1.220562764	7.97E-11	9.07E-10	yes	down	11.992	4.606	10.55	15.9	10.12	13.88	9.51	2.71	5.12	5.52	4.95	4.73
ENSMUSG00000030359 Pzp	PZP, alpha-2-macroglobulin like [Source:MGI Symbol;Acc:MGI:87854]	0.218116128	-2.196831647	0.003760696	0.012731437	yes	down	0.5	0.028	0.4	0.29	0.16	1.29	0.36	0.03	0	0.04	0.06	0.01
ENSMUSG00000019832 Rab32	RAB32, member RAS oncogene family [Source:MGI Symbol;Acc:MGI:1915094]	4.119885161	2.042604124	1.43E-47	1.30E-45	yes	up	11.768	40.75	9.67	13.64	15.29	8.16	12.08	40.32	41.47	38.8	41.33	41.83
ENSMUSG00000024663 Rab3il1	RAB3A interacting protein (rabin3)-like 1 [Source:MGI Symbol;Acc:MGI:1922010]	2.331358581	1.221170919	1.50E-14	2.48E-13	yes	up	5.87	11.204	5.02	7.17	6.63	5.66	4.87	9.88	9.07	11.65	11.63	13.79
ENSMUSG00000052688 Rab7b	RAB7B, member RAS oncogene family [Source:MGI Symbol;Acc:MGI:2442295]	3.187137772	1.672261384	3.42E-44	2.79E-42	yes	up	10.462	22.458	7.7	11.64	8.07	16.09	8.81	21.97	21.77	23.33	22.44	22.78
ENSMUSG00000033220 Rac2	Rac family small GTPase 2 [Source:MGI Symbol;Acc:MGI:97846]	2.292836763	1.197133647	2.42E-33	1.34E-31	yes	up	41.44	67.05	53.36	25.2	76.31	34.4	17.93	115.99	82.21	43.63	41.71	51.71
ENSMUSG00000027323 Rad51	RAD51 recombinase [Source:MGI Symbol;Acc:MGI:97890]	2.326613938	1.21823184	4.37E-06	2.71E-05	yes	up	1.144	2.766	1.18	1.38	1.34	0.68	1.14	1.44	1.64	1.8	2.64	6.31
ENSMUSG00000028702 Rad54l	RAD54 like (S. cerevisiae) [Source:MGI Symbol;Acc:MGI:894697]	2.278413091	1.188029341	0.000265501	0.001184082	yes	up	0.698	1.298	0.81	0.62	0.92	0.72	0.42	1.75	1.15	1.19	0.63	1.77
ENSMUSG00000078452 Raet1d	retinoic acid early transcript delta [Source:MGI Symbol;Acc:MGI:1861032]	12.31461693	3.622299845	5.63E-05	0.000284948	yes	up	0.058	0.576	0.06	0	0.06	0.17	0	0.35	0.79	0.37	0.8	0.57
ENSMUSG00000036894 Rap2b	RAP2B, member of RAS oncogene family [Source:MGI Symbol;Acc:MGI:1921262]	2.467537649	1.303072097	6.04E-19	1.44E-17	yes	up	2.34	4.86	2.23	1.84	3	2.44	2.19	5.74	4.31	4.09	5.61	4.55
ENSMUSG00000038020 Rapgefl1	Rap guanine nucleotide exchange factor (GEF)-like 1 [Source:MGI Symbol;Acc:MGI:3611446]	0.478152244	-1.064458047	0.000552457	0.002288475	yes	down	1.026	0.356	0.86	1.4	0.52	0.99	1.36	0.23	0.27	0.46	0.38	0.44
ENSMUSG00000004952 Rasa4	RAS p21 protein activator 4 [Source:MGI Symbol;Acc:MGI:1858600]	2.622642611	1.391021224	2.67E-28	1.13E-26	yes	up	8.204	15.942	7.89	9.25	9.51	7.64	6.73	16.26	18.15	14.32	12.36	18.62
ENSMUSG00000029602 Rasal1	RAS protein activator like 1 (GAP1 like) [Source:MGI	2.384120583	1.253457206	0.011350165	0.033458381	yes	up	0.408	0.41	0.14	0.44	0.36	0.32	0.78	0.75	0.44	0.11	0.34	0.41

	Symbol;Acc:MGI:1330842]																		
ENSMUSG00000052142 Rasal3	RAS protein activator like 3 [Source:MGI Symbol;Acc:MGI:2444128]	2.580072871	1.367411813	1.48E-15	2.67E-14	yes	up	6.832	13.664	5.62	6.78	9.32	7.58	4.86	19.6	12.06	14.53	7.18	14.95
ENSMUSG00000049892 Rasd1	RAS, dexamethasone-induced 1 [Source:MGI Symbol;Acc:MGI:1270848]	0.212943477	-2.231457559	0.000156826	0.0007314	yes	down	11.47	2.066	11.85	11.69	9.58	9.13	15.1	1.11	2.04	5.65	0.48	1.05
ENSMUSG00000034472 Rasd2	RASD family, member 2 [Source:MGI Symbol;Acc:MGI:1922391]	2.369085795	1.244330446	5.77E-07	4.09E-06	yes	up	1.336	2.666	1.19	1.9	1.63	0.85	1.11	1.81	2.28	3.29	2.96	2.99
ENSMUSG00000089809 Rasgef1b	RasGEF domain family, member 1B [Source:MGI Symbol;Acc:MGI:2443755]	0.422700808	-1.242291223	1.14E-24	3.88E-23	yes	down	45.73	16.658	50.3	46.69	32.35	45.69	53.62	16.28	19.51	15	16.73	15.77
ENSMUSG00000027347 Rasgrp1	RAS guanyl releasing protein 1 [Source:MGI Symbol;Acc:MGI:1314635]	2.037330394	1.026679961	5.77E-14	9.05E-13	yes	up	3.518	6.106	3.4	4.07	2.95	4.38	2.79	6.58	5.47	5.66	5.69	7.13
ENSMUSG00000042129 Rassf4	Ras association (RalGDS/AF-6) domain family member 4 [Source:MGI Symbol;Acc:MGI:2386853]	3.83789809	1.940316403	4.67E-63	6.67E-61	yes	up	12.09	38.342	10.91	13.31	12.88	9.87	13.48	36.15	41.53	38.62	37.03	38.38
ENSMUSG00000035275 Raver2	ribonucleoprotein, PTB-binding 2 [Source:MGI Symbol;Acc:MGI:2443623]	0.419849025	-1.252057457	4.66E-26	1.72E-24	yes	down	25.288	8.734	23.41	23.59	25.57	30.69	23.18	8.28	10.35	7.08	10.15	7.81
ENSMUSG00000025576 Rbfox3	RNA binding protein, fox-1 homolog (C. elegans) 3 [Source:MGI Symbol;Acc:MGI:106368]	0.360561214	-1.47168388	8.22E-05	0.000405498	yes	down	2.8	0.604	2.18	1.54	2.45	4.72	3.11	0.75	1.46	0.25	0.35	0.21
ENSMUSG00000024990 Rbp4	retinol binding protein 4, plasma [Source:MGI Symbol;Acc:MGI:97879]	0.493216704	-1.019706433	9.92E-07	6.79E-06	yes	down	27.518	11	22.59	34.2	26.7	21.21	32.89	6.85	11.93	15.04	8.07	13.11
ENSMUSG00000039601 Rcan2	regulator of calcineurin 2 [Source:MGI Symbol;Acc:MGI:1858219]	0.454280852	-1.138343599	3.53E-28	1.48E-26	yes	down	18.146	6.984	19.53	19.8	15.66	19.97	15.77	6.93	7.51	6.34	7.83	6.31
ENSMUSG00000022106 Rcbtb2	regulator of chromosome condensation (RCC1) and BTB (POZ) domain containing protein 2 [Source:MGI Symbol;Acc:MGI:1917200]	2.224205577	1.153290138	1.57E-32	8.32E-31	yes	up	29.458	77.75	29.72	35.4	32.34	22.61	27.22	87.12	80.59	68.43	87.94	64.67
ENSMUSG00000049353 Rd3	retinal degeneration 3 [Source:MGI Symbol;Acc:MGI:1921273]	0.389657693	-1.359720794	1.06E-08	9.67E-08	yes	down	3	1.044	2.33	3.77	2.51	3.48	2.91	1.39	1.43	0.97	0.87	0.56
ENSMUSG00000028476 Reck	reversion-inducing-cysteine-rich protein with kazal motifs [Source:MGI Symbol;Acc:MGI:1855698]	0.431802733	-1.211555721	1.35E-27	5.46E-26	yes	down	32.328	11.696	33.56	27.34	31.46	40.67	28.61	11.41	12.6	10.8	13.8	9.87
ENSMUSG00000008318 Relt	RELT tumor necrosis factor receptor [Source:MGI Symbol;Acc:MGI:2443373]	2.32424028	1.216759222	3.41E-11	4.03E-10	yes	up	4.11	10.476	1.93	3.31	2.3	8.66	4.35	10.08	7	11.72	9.32	14.26
ENSMUSG00000022176 Rem2	rad and gem related GTP binding protein 2 [Source:MGI Symbol;Acc:MGI:2155260]	0.481321028	-1.054928642	0.001216319	0.004663208	yes	down	1.836	1.3	1.33	2.51	2.27	1.47	1.6	0.58	1.68	2.98	0.59	0.67
ENSMUSG00000061100 Retnla	resistin like alpha [Source:MGI Symbol;Acc:MGI:1888504]	3.143461858	1.652354258	8.85E-06	5.20E-05	yes	up	112.62	302.878	59.07	158.66	86.29	69.65	189.43	295.68	280.6	354.78	206.81	376.52
ENSMUSG00000039316 Rftn1	raftlin lipid raft linker 1 [Source:MGI Symbol;Acc:MGI:1923688]	2.202776386	1.139323048	3.08E-50	3.01E-48	yes	up	20.182	34.78	23.17	20	23.38	16.14	18.22	35.02	37.52	31	31.18	39.18
ENSMUSG00000022018 Rgcc	regulator of cell cycle [Source:MGI Symbol;Acc:MGI:1913464]	0.484759854	-1.04465787	0.007893799	0.024500521	yes	down	231.686	95.372	182.79	152.48	180.25	186.4	456.51	144.69	74.54	114.31	58.59	84.73
ENSMUSG00000026358 Rgs1	regulator of G-protein signaling 1 [Source:MGI Symbol;Acc:MGI:1354694]	2.803639661	1.487300938	2.83E-07	2.11E-06	yes	up	29.052	60.388	23.89	42.24	38.76	14.66	25.71	55.51	49.81	102.11	48.58	45.93

ENSMUSG00000030844 Rgs10	regulator of G-protein signalling 10 [Source:MGI Symbol;Acc:MGI:1915115]	2.283512184	1.191254488	8.02E-25	2.74E-23	yes	up	34.702	67.126	29.38	40.6	37.26	31.38	34.89	58.12	67.7	68.92	64.45	76.44
ENSMUSG00000026475 Rgs16	regulator of G-protein signaling 16 [Source:MGI Symbol;Acc:MGI:108407]	11.12783026	3.476100413	6.39E-09	5.96E-08	yes	up	0.426	3.96	0.29	0.95	0.65	0.07	0.17	4.9	2.3	3.03	3.55	6.02
ENSMUSG00000037627 Rgs22	regulator of G-protein signalling 22 [Source:MGI Symbol;Acc:MGI:3613651]	0.415849457	-1.265866746	3.36E-06	2.13E-05	yes	down	4.768	1.928	3.27	4.8	3.03	6.76	5.98	2.15	1.61	3.22	0.74	1.92
ENSMUSG00000021219 Rgs6	regulator of G-protein signaling 6 [Source:MGI Symbol;Acc:MGI:1354730]	0.363466538	-1.460105544	1.80E-22	5.36E-21	yes	down	11.664	19.804	30.97	7.42	6.26	7.05	6.62	1.65	1.78	35.88	58.04	1.67
ENSMUSG00000042671 Rgs8	regulator of G-protein signaling 8 [Source:MGI Symbol;Acc:MGI:108408]	19.7142136	4.301164257	0.004238969	0.014153688	yes	up	0.004	0.08	0	0.02	0	0	0	0.06	0	0.03	0.03	0.28
ENSMUSG00000020599 Rgs9	regulator of G-protein signaling 9 [Source:MGI Symbol;Acc:MGI:1338824]	0.468097988	-1.09511753	8.33E-09	7.67E-08	yes	down	11.356	4.456	13.2	8.38	10.96	13.23	11.01	5.74	5.38	3.07	3.34	4.75
ENSMUSG00000030324 Rho	rhodopsin [Source:MGI Symbol;Acc:MGI:97914]	0.284635435	-1.812812816	0.011800615	0.034586283	yes	down	0.174	0.076	0.05	0.34	0.11	0.13	0.24	0.15	0.02	0.14	0.03	0.04
ENSMUSG00000029204 Rhoh	ras homolog family member H [Source:MGI Symbol;Acc:MGI:1921984]	2.288253446	1.194246853	1.60E-09	1.59E-08	yes	up	3.34	6.342	2.94	3.73	3.81	3.68	2.54	8.49	5.16	5.39	4.82	7.85
ENSMUSG00000025257 Ribc1	RIB43A domain with coiled-coils 1 [Source:MGI Symbol;Acc:MGI:1913861]	0.376501459	-1.409272641	1.92E-05	0.000106715	yes	down	2.848	0.908	3.77	2.93	2.23	1.89	3.42	1.05	0.83	1.23	0.44	0.99
ENSMUSG00000022431 Ribc2	RIB43A domain with coiled-coils 2 [Source:MGI Symbol;Acc:MGI:1914997]	0.271912087	-1.878787814	0.015713675	0.044244552	yes	down	0.55	0.14	0.36	0.69	0.64	0.14	0.92	0.31	0.2	0.1	0.09	0
ENSMUSG00000028139 Riiad1	regulatory subunit of type II PKA R-subunit (RIIa) domain containing 1 [Source:MGI Symbol;Acc:MGI:1913603]	0.484468238	-1.04552601	0.000233641	0.001054868	yes	down	19.692	8.056	17.18	25.28	13.26	22.19	20.55	5.27	7.97	13.78	5.09	8.17
ENSMUSG00000029401 Rilpl2	Rab interacting lysosomal protein-like 2 [Source:MGI Symbol;Acc:MGI:1933112]	3.156988228	1.658548881	5.35E-51	5.38E-49	yes	up	15.672	41.68	15.6	14.58	15.03	16.7	16.45	42.86	41.21	32.98	43.92	47.43
ENSMUSG00000029420 Rimbp2	RIMS binding protein 2 [Source:MGI Symbol;Acc:MGI:2443235]	6.901482357	2.786906269	0.010840164	0.032123005	yes	up	0.046	0.046	0	0.18	0.04	0.01	0	0.03	0.03	0.04	0.06	0.07
ENSMUSG00000032890 Rims3	regulating synaptic membrane exocytosis 3 [Source:MGI Symbol;Acc:MGI:2443331]	4.263592377	2.092069515	5.07E-20	1.29E-18	yes	up	0.58	2.138	0.51	0.7	0.57	0.49	0.63	2.11	2.73	1.57	2.69	1.59
ENSMUSG00000045232 Rln3	relaxin 3 [Source:MGI Symbol;Acc:MGI:2158015]	9.392001538	3.231432645	2.50E-05	0.000135869	yes	up	0.268	2.086	0.34	0.18	0.52	0.3	0	2.46	2.27	1.05	1.29	3.36
ENSMUSG00000047222 Rnase2a	ribonuclease, RNase A family, 2A (liver, eosinophil-derived neurotoxin) [Source:MGI Symbol;Acc:MGI:1890465]	131.8669538	7.042939256	2.95E-11	3.52E-10	yes	up	0	2.888	0	0	0	0	0	1.99	4.7	3.61	1.36	2.78
ENSMUSG00000059606 Rnase2b	ribonuclease, RNase A family, 2B (liver, eosinophil-derived neurotoxin) [Source:MGI Symbol;Acc:MGI:1858598]	7.094516212	2.826704308	3.22E-07	2.37E-06	yes	up	0.492	2.928	0.28	0.74	0.58	0.62	0.24	2.64	3.88	3.92	2.86	1.34
ENSMUSG00000021880 Rnase6	ribonuclease, RNase A family, 6 [Source:MGI	2.574765152	1.364440848	1.18E-38	7.86E-37	yes	up	15.878	34.404	12.42	18.54	18.17	12.66	17.6	38.99	34.8	35.41	31.41	31.41

		Symbol;Acc:MGI:1925666]																		
ENSMUSG00000095687	Rnaset2a	ribonuclease T2A [Source:MGI Symbol;Acc:MGI:1915445]	2.658883702	1.410820676	0.016579045	0.046291633	yes	up	8.738	20.692	3.93	5.53	6.6	7.57	20.06	44	15.57	20.2	7.27	16.42
ENSMUSG00000010086	Rnf112	ring finger protein 112 [Source:MGI Symbol;Acc:MGI:106611]	0.026888403	-5.216872136	3.89E-05	0.000202594	yes	down	0.164	0	0.13	0.11	0.22	0.29	0.07	0	0	0	0	0
ENSMUSG00000031438	Rnf128	ring finger protein 128 [Source:MGI Symbol;Acc:MGI:1914139]	4.097676092	2.034805949	5.56E-35	3.22E-33	yes	up	4.894	15.984	3.96	7.04	4.24	4.16	5.07	16.75	14.96	16.27	16.74	15.2
ENSMUSG00000048234	Rnf149	ring finger protein 149 [Source:MGI Symbol;Acc:MGI:2677438]	3.435670041	1.780591488	5.45E-62	7.54E-60	yes	up	18.41	53.898	16.9	21.67	17.12	16.67	19.69	59.8	51.33	50.39	53.41	54.56
ENSMUSG00000021720	Rnf180	ring finger protein 180 [Source:MGI Symbol;Acc:MGI:1919066]	5.682434985	2.506509273	5.95E-52	6.28E-50	yes	up	1.628	6.95	1.96	1.91	1.69	1.32	1.26	5.49	7.76	6.75	6.95	7.8
ENSMUSG00000032850	Rnft2	ring finger protein, transmembrane 2 [Source:MGI Symbol;Acc:MGI:2442859]	0.3756818	-1.412416869	0.002368886	0.008463808	yes	down	0.506	0.25	0.19	0.9	0.19	0.87	0.38	0.11	0.22	0.26	0.33	0.33
ENSMUSG00000029608	Rph3a	rabphilin 3A [Source:MGI Symbol;Acc:MGI:102788]	11.41311038	3.512620113	6.75E-08	5.48E-07	yes	up	0.05	0.47	0	0.11	0.13	0	0.01	0.24	0.56	0.5	0.48	0.57
ENSMUSG00000025665	Rps6ka6	ribosomal protein S6 kinase polypeptide 6 [Source:MGI Symbol;Acc:MGI:1914321]	0.335826756	-1.574210916	2.62E-07	1.96E-06	yes	down	1.078	0.494	1.34	0.99	1.02	1.23	0.81	0.39	0.21	0.58	0.96	0.33
ENSMUSG00000041984	Rptn	repetin [Source:MGI Symbol;Acc:MGI:1099055]	0.254327658	-1.975239733	1.87E-05	0.000103769	yes	down	0.896	0.194	1.25	0.71	0.5	0.74	1.28	0.24	0.25	0.35	0.08	0.05
ENSMUSG00000031880	Rrad	Ras-related associated with diabetes [Source:MGI Symbol;Acc:MGI:1930943]	2.483332868	1.312277655	1.22E-08	1.11E-07	yes	up	4.15	8.826	2.88	5.41	4.45	3.18	4.83	9.42	8.28	11.04	5.23	10.16
ENSMUSG00000020649	Rrm2	ribonucleotide reductase M2 [Source:MGI Symbol;Acc:MGI:98181]	2.222519984	1.152196391	3.22E-07	2.37E-06	yes	up	4.794	8.906	4.11	5.08	7.28	3.87	3.63	11.54	7.96	6.62	6.53	11.88
ENSMUSG00000009070	Rsph14	radial spoke head homolog 14 (Chlamydomonas) [Source:MGI Symbol;Acc:MGI:1918486]	0.414621508	-1.270133138	0.010891316	0.032238896	yes	down	5.692	1.642	2.57	10.17	2.76	6.25	6.71	0.88	2.32	3.23	0.22	1.56
ENSMUSG00000028871	Rspo1	R-spondin 1 [Source:MGI Symbol;Acc:MGI:2183426]	0.391650744	-1.352360397	1.53E-18	3.52E-17	yes	down	15.45	4.47	16.67	13.15	16.91	16.48	14.04	3.34	5.45	4.16	5.35	4.05
ENSMUSG00000032852	Rspo4	R-spondin 4 [Source:MGI Symbol;Acc:MGI:1924467]	0.164805485	-2.601163835	9.54E-05	0.000463728	yes	down	0.39	0.052	0.34	0.47	0.29	0.44	0.41	0.09	0.04	0.09	0	0.04
ENSMUSG00000034959	Rubcnl	RUN and cysteine rich domain containing beclin 1 interacting protein like [Source:MGI Symbol;Acc:MGI:2685590]	2.065333661	1.046374872	9.63E-07	6.61E-06	yes	up	8.748	3.122	1.25	1.93	36.9	2.24	1.42	3.71	2.24	3.07	3.11	3.48
ENSMUSG00000006575	Rundc3a	RUN domain containing 3A [Source:MGI Symbol;Acc:MGI:1858752]	2.424852747	1.27789714	1.49E-07	1.15E-06	yes	up	1.112	2.184	1.07	0.86	1.12	0.87	1.64	1.68	2.22	2.37	2.29	2.36
ENSMUSG00000039153	Runx2	runt related transcription factor 2 [Source:MGI Symbol;Acc:MGI:99829]	2.074410277	1.052701259	1.34E-16	2.68E-15	yes	up	2.742	6.228	4.15	1.85	2.5	3.1	2.11	6.75	5.21	5.75	5.39	8.04
ENSMUSG00000070691	Runx3	runt related transcription factor 3 [Source:MGI Symbol;Acc:MGI:102672]	2.358549116	1.237899645	5.71E-13	8.10E-12	yes	up	2.298	4.64	1.94	1.62	2.89	2.71	2.33	6.04	3.63	4.69	3.51	5.33
ENSMUSG00000028133	Rwdd3	RWD domain containing 3 [Source:MGI Symbol;Acc:MGI:1920420]	0.375980292	-1.411271052	3.12E-05	0.000165603	yes	down	3.56	1.084	3.72	4.92	1.85	3.05	4.26	1.06	1.06	1.61	0.62	1.07
ENSMUSG00000030592	Ryr1	ryanodine receptor 1, skeletal muscle [Source:MGI Symbol;Acc:MGI:99659]	2.205139161	1.140869704	0.007257463	0.02278637	yes	up	0.082	0.152	0.02	0.09	0.14	0.1	0.06	0.08	0.23	0.17	0.17	0.11
ENSMUSG00000001020	S100a4	S100 calcium binding protein A4 [Source:MGI Symbol;Acc:MGI:1330282]	2.678119049	1.421220093	6.59E-22	1.87E-20	yes	up	125.09	283.752	109.4	150.62	135.12	140.75	89.56	288.54	266.07	305.02	215.88	343.25
ENSMUSG00000045092	S1pr1	sphingosine-1-phosphate receptor 1 [Source:MGI	0.421431261	-1.246630761	2.70E-37	1.70E-35	yes	down	266.804	94.772	271.31	295.62	218.67	313.88	234.54	101.77	103.07	85.66	96.71	86.65

	Symbol;Acc:MGI:1096355]																	
ENSMUSG00000045087 S1pr5	sphingosine-1-phosphate receptor 5 [Source:MGI Symbol;Acc:MGI:2150641]	0.377479744	-1.405528867	3.50E-06	2.22E-05	yes	down	1.42	0.454	1.09	1.48	1.09	1.9	1.54	0.64	0.43	0.37	0.47	0.36
ENSMUSG00000040026 Saa3	serum amyloid A 3 [Source:MGI Symbol;Acc:MGI:98223]	133.7161361	7.063029762	2.01E-48	1.88E-46	yes	up	6.942	793.924	13.61	6.45	6.6	2.3	5.75	938.56	535.95	378.28	809.5	1307.33
ENSMUSG00000031101 Sash3	SAM and SH3 domain containing 3 [Source:MGI Symbol;Acc:MGI:1921381]	2.353874114	1.235037167	5.43E-31	2.64E-29	yes	up	13.888	27.486	14.28	12.97	15.63	15.62	10.94	30.48	25.59	24.75	26.88	29.73
ENSMUSG00000069835 Sat2	spermidine/spermine N1-acetyl transferase 2 [Source:MGI Symbol;Acc:MGI:1916465]	0.467176351	-1.09796085	0.004181993	0.013983069	yes	down	2.944	1.136	2.67	5.22	2.17	2.47	2.19	0.87	1.23	1	0.93	1.65
ENSMUSG00000030433 Sbk2	SH3-binding domain kinase family, member 2 [Source:MGI Symbol;Acc:MGI:2685925]	5.057737816	2.338492252	0.012420782	0.036173986	yes	up	0.712	4.198	0.24	2.5	0.57	0.25	0	3.4	3.77	2.99	5.61	5.22
ENSMUSG00000085272 Sbk3	SH3 domain binding kinase family, member 3 [Source:MGI Symbol;Acc:MGI:2685924]	0.215167909	-2.216465171	0.013527735	0.038859074	yes	down	1.702	0.37	0.72	5.62	0.32	1.13	0.72	0.15	0.13	0.87	0.21	0.49
ENSMUSG00000046056 Sbsn	suprabasin [Source:MGI Symbol;Acc:MGI:2446326]	0.432645935	-1.208741248	2.68E-16	5.20E-15	yes	down	13.318	4.15	13.02	12.58	12.42	15.05	13.52	3.74	4.38	4.02	4.5	4.11
ENSMUSG00000032719 Sbspon	somatomedin B and thrombospondin, type 1 domain containing [Source:MGI Symbol;Acc:MGI:2684952]	0.22205776	-2.170993108	0.000183568	0.000843806	yes	down	1.222	0.226	1.1	3.26	0.57	0.58	0.6	0.07	0.22	0.42	0.08	0.34
ENSMUSG00000040722 Scamp5	secretory carrier membrane protein 5 [Source:MGI Symbol;Acc:MGI:1928948]	2.558211054	1.355135293	4.37E-17	9.09E-16	yes	up	3.058	6.178	2.33	3.58	3.14	2.76	3.48	6.77	6.17	5.08	6.86	6.01
ENSMUSG00000022123 Scel	sciellin [Source:MGI Symbol;Acc:MGI:1891228]	0.498347126	-1.004777086	4.28E-14	6.80E-13	yes	down	37.774	15.924	46.83	37.78	30.4	40.81	33.05	17.19	17.81	13.41	18.34	12.87
ENSMUSG00000023236 Scg5	secretogranin V [Source:MGI Symbol;Acc:MGI:98289]	0.278944181	-1.841951638	0.003093456	0.010706118	yes	down	0.838	0.198	0.77	0.79	1.25	0.83	0.55	0.21	0.1	0.25	0.38	0.05
ENSMUSG00000024653 Scgb1a1	secretoglobin, family 1A, member 1 (uteroglobin) [Source:MGI Symbol;Acc:MGI:98919]	0.336683977	-1.570533029	2.08E-36	1.27E-34	yes	down	137137.026	39228.444	149176.64	105381.17	125342.03	155562.13	150223.16	44942.39	36521.76	46239.58	35235.5	33202.99
ENSMUSG00000038801 Scgb1c1	secretoglobin, family 1C, member 1 [Source:MGI Symbol;Acc:MGI:2655401]	0.379732468	-1.396944738	2.05E-11	2.49E-10	yes	down	43.486	14.114	50.75	33.36	37.88	41.92	53.52	12.98	14.07	15	15.76	12.76
ENSMUSG00000057135 Scimp	SLP adaptor and CSK interacting membrane protein [Source:MGI Symbol;Acc:MGI:3610314]	3.25320913	1.701863568	8.26E-28	3.39E-26	yes	up	5.53	14.918	5.13	5.07	6.63	4.33	6.49	13.92	14.83	14.86	14.95	16.03
ENSMUSG00000019194 Scn1b	sodium channel, voltage-gated, type I, beta [Source:MGI Symbol;Acc:MGI:98247]	2.335357759	1.223643576	3.13E-14	5.02E-13	yes	up	8.8	17.056	6.54	9.49	10.14	7.3	10.53	15.29	15.09	18.96	17.25	18.69
ENSMUSG00000057182 Scn3a	sodium channel, voltage-gated, type III, alpha [Source:MGI Symbol;Acc:MGI:98249]	0.335236615	-1.576748365	2.08E-22	6.17E-21	yes	down	6.004	1.9	6.29	6.33	5.44	8.09	3.87	1.54	2	2.99	1.75	1.22
ENSMUSG00000032511 Scn5a	sodium channel, voltage-gated, type V, alpha [Source:MGI Symbol;Acc:MGI:98251]	0.389452567	-1.360480469	5.39E-07	3.84E-06	yes	down	2.628	0.7	1.8	2	2.37	4.58	2.39	0.54	0.78	1.35	0.38	0.45

ENSMUSG00000091780	Sco2	SCO2 cytochrome c oxidase assembly protein [Source:MGI Symbol;Acc:MGI:3818630]	2.120545328	1.084435322	3.83E-05	0.000199932	yes	up	2.764	4.92	1.77	2.52	3.41	1.93	4.19	4.44	5.67	4.49	4.44	5.56
ENSMUSG00000000278	Scpep1	serine carboxypeptidase 1 [Source:MGI Symbol;Acc:MGI:1921867]	2.076334113	1.054038613	1.49E-31	7.41E-30	yes	up	38.884	68.258	35.91	42.71	38.99	35.6	41.21	61.94	63.61	69.12	72.13	74.49
ENSMUSG00000007279	Scube2	signal peptide, CUB domain, EGF-like 2 [Source:MGI Symbol;Acc:MGI:1928765]	0.43718259	-1.193692144	2.59E-12	3.47E-11	yes	down	21.928	8.41	23.76	16.43	19.51	30.97	18.97	9.49	10.28	7.69	9.53	5.06
ENSMUSG00000022769	Sdf2l1	stromal cell-derived factor 2-like 1 [Source:MGI Symbol;Acc:MGI:2149842]	2.795690471	1.483204639	8.21E-22	2.32E-20	yes	up	29.046	68.594	29.95	21.28	40.06	24.22	29.72	72.35	64.72	77.7	55.95	72.25
ENSMUSG00000024516	Sec11c	SEC11 homolog C, signal peptidase complex subunit [Source:MGI Symbol;Acc:MGI:1913536]	2.052537369	1.037408488	1.24E-19	3.07E-18	yes	up	63.682	112.102	57.34	76.95	64.48	60.43	59.21	133.62	105.26	112.8	105.8	103.03
ENSMUSG00000026582	Sele	selectin, endothelial cell [Source:MGI Symbol;Acc:MGI:98278]	0.460490023	-1.118758195	0.009224649	0.028049847	yes	down	1.114	0.43	1.19	1.15	0.55	2.01	0.67	0.41	0.31	0.52	0.75	0.16
ENSMUSG00000068877	Selenbp2	selenium binding protein 2 [Source:MGI Symbol;Acc:MGI:104859]	0.434914224	-1.201197201	1.19E-05	6.82E-05	yes	down	7.226	3.64	3.5	6.22	9.46	9.03	7.92	6.41	2.82	3.35	2.99	2.63
ENSMUSG00000048163	Selplg	selectin, platelet (p-selectin) ligand [Source:MGI Symbol;Acc:MGI:106689]	3.188389284	1.672827785	1.57E-103	5.99E-101	yes	up	58.39	154.42	56.85	66.62	60.51	59.22	48.75	132.87	168.61	152.08	151.4	167.14
ENSMUSG00000028064	Sema4a	sema domain, immunoglobulin domain (Ig), transmembrane domain (TM) and short cytoplasmic domain, (semaphorin) 4A [Source:MGI Symbol;Acc:MGI:107560]	2.094462744	1.066580222	2.53E-26	9.46E-25	yes	up	19.018	34.112	18.05	21.63	20.42	17.61	17.38	30.96	32.33	34.68	32.17	40.42
ENSMUSG00000021451	Sema4d	sema domain, immunoglobulin domain (Ig), transmembrane domain (TM) and short cytoplasmic domain, (semaphorin) 4D [Source:MGI Symbol;Acc:MGI:109244]	3.43577156	1.780634117	4.53E-72	8.90E-70	yes	up	13.044	31.95	9.66	10.76	23.39	12.25	9.16	32.84	28.87	29.67	28.15	40.22
ENSMUSG00000061947	Serpina10	serine (or cysteine) peptidase inhibitor, clade A (alpha-1 antiproteinase, antitrypsin), member 10 [Source:MGI Symbol;Acc:MGI:2667725]	4.731354099	2.242253138	1.17E-07	9.18E-07	yes	up	0.396	1.514	0.07	0.63	0.42	0.16	0.7	2.43	1.07	1.34	1.43	1.3
ENSMUSG00000066361	Serpina3c	serine (or cysteine) peptidase inhibitor, clade A, member 3C [Source:MGI Symbol;Acc:MGI:102848]	0.224565468	-2.154791997	1.35E-53	1.49E-51	yes	down	10.986	2.086	11.33	10.17	12.25	10.97	10.21	2.24	2.52	1.91	2.15	1.61
ENSMUSG00000066363	Serpina3f	serine (or cysteine) peptidase inhibitor, clade A, member 3F [Source:MGI Symbol;Acc:MGI:2182838]	3.464635682	1.792703656	7.15E-06	4.28E-05	yes	up	4.43	13.042	5.58	3.46	3.59	2.49	7.03	16.5	9.47	7.56	10.03	21.65
ENSMUSG00000041481	Serpina3g	serine (or cysteine) peptidase inhibitor, clade A, member 3G [Source:MGI Symbol;Acc:MGI:105046]	4.562901415	2.189951484	1.22E-13	1.87E-12	yes	up	12.398	52.464	12.04	11.54	13.61	8.19	16.61	63.09	37.11	39.05	44.25	78.82
ENSMUSG00000092572	Serpinb10	serine (or cysteine) peptidase inhibitor, clade B (ovalbumin), member 10 [Source:MGI Symbol;Acc:MGI:2138648]	0.422928508	-1.241514284	0.002625996	0.009269545	yes	down	3.056	1.094	2.17	3.06	2.32	5.37	2.36	1.7	1.27	0.24	1.23	1.03
ENSMUSG00000051029	Serpinb1b	serine (or cysteine) peptidase inhibitor, clade B, member 1b [Source:MGI Symbol;Acc:MGI:2445361]	0.367140599	-1.445595438	0.001305737	0.004966022	yes	down	1.414	0.396	1.42	2.11	0.78	1.65	1.11	0.24	0.62	0.5	0.42	0.2

ENSMUSG00000021403	Serpinb9b	serine (or cysteine) peptidase inhibitor, clade B, member 9b [Source:MGI Symbol;Acc:MGI:894668]	0.409784894	-1.287061291	0.002531092	0.00897151	yes	down	0.562	0.218	0.66	0.87	0.57	0.36	0.35	0.2	0.32	0.24	0.1	0.23
ENSMUSG00000031548	Sfrp1	secreted frizzled-related protein 1 [Source:MGI Symbol;Acc:MGI:892014]	2.039924614	1.028515838	6.91E-13	9.70E-12	yes	up	8.032	13.87	6.82	9.79	9.04	6.02	8.49	11.44	13.24	13.21	16.35	15.11
ENSMUSG00000035296	Sgcg	sarcoglycan, gamma (dystrophin-associated glycoprotein) [Source:MGI Symbol;Acc:MGI:1346524]	0.288864959	-1.791532889	0.000258277	0.001154753	yes	down	1.82	0.55	1.04	2.96	1.89	1.71	1.5	0.65	0.45	1.39	0.17	0.09
ENSMUSG00000028524	Sgip1	SH3-domain GRB2-like (endophilin) interacting protein 1 [Source:MGI Symbol;Acc:MGI:1920344]	0.440565083	-1.182572937	1.28E-18	2.97E-17	yes	down	12.456	6.84	11.04	15.62	11.29	11.88	12.45	5.25	8.84	2.26	8.21	9.64
ENSMUSG00000023940	Sgo1	shugoshin 1 [Source:MGI Symbol;Acc:MGI:1919665]	2.109920563	1.077188684	3.67E-05	0.000192387	yes	up	1	0.86	0.39	0.95	0.61	2.45	0.6	0.79	0.97	0.94	0.71	0.89
ENSMUSG00000026039	Sgo2a	shugoshin 2A [Source:MGI Symbol;Acc:MGI:1098767]	2.301612908	1.202645217	8.87E-06	5.21E-05	yes	up	0.602	1.18	0.34	0.49	0.99	0.49	0.7	1.19	1.19	1.07	0.93	1.52
ENSMUSG00000020097	Sgpl1	sphingosine phosphate lyase 1 [Source:MGI Symbol;Acc:MGI:1261415]	2.371748646	1.245951123	1.52E-76	3.23E-74	yes	up	32.058	64.244	29.5	32.57	34.88	34.33	29.01	62.16	58.46	62.4	61.34	76.86
ENSMUSG00000005057	Sh2b2	SH2B adaptor protein 2 [Source:MGI Symbol;Acc:MGI:1345171]	2.92016714	1.546050946	1.13E-14	1.90E-13	yes	up	1.684	4.118	1.69	1.67	2.01	1.45	1.6	4.67	4.35	3.14	3.18	5.25
ENSMUSG00000102418	Sh2d1b1	SH2 domain containing 1B1 [Source:MGI Symbol;Acc:MGI:1349420]	2.601346004	1.379258305	4.55E-12	5.92E-11	yes	up	5.298	12.594	5.33	5.17	8.18	2.96	4.85	10.5	14.86	13.47	13.51	10.63
ENSMUSG00000073494	Sh2d1b2	SH2 domain containing 1B2 [Source:MGI Symbol;Acc:MGI:3622649]	2.891326757	1.531731661	4.56E-06	2.82E-05	yes	up	0.56	1.254	0.93	0.41	0.62	0.39	0.45	1.22	2.11	1.19	0.87	0.88
ENSMUSG00000040666	Sh3bgr	SH3-binding domain glutamic acid-rich protein [Source:MGI Symbol;Acc:MGI:1354740]	0.43440406	-1.202890507	0.001322213	0.005021549	yes	down	9.456	3.638	7.15	20.94	8.2	6.59	4.4	1.54	7.54	4.19	2.57	2.35
ENSMUSG00000028843	Sh3bgrl3	SH3 domain binding glutamic acid-rich protein-like 3 [Source:MGI Symbol;Acc:MGI:1920973]	2.172253035	1.119192166	1.18E-37	7.53E-36	yes	up	215.652	395.026	203.86	241.96	219.31	215.3	197.83	348.59	428.62	365.21	371.23	461.48
ENSMUSG00000022436	Sh3bp1	SH3-domain binding protein 1 [Source:MGI Symbol;Acc:MGI:104603]	2.297358071	1.199975736	1.66E-15	2.99E-14	yes	up	13.248	25.45	9.34	14.67	15.34	10.89	16	24.69	29.45	27.24	19.25	26.62
ENSMUSG00000054520	Sh3bp2	SH3-domain binding protein 2 [Source:MGI Symbol;Acc:MGI:1346349]	3.50839143	1.810809718	2.73E-64	4.12E-62	yes	up	7.018	19.736	5.4	7.38	5.99	9.79	6.53	19.67	21.72	16.75	18.09	22.45
ENSMUSG00000028488	Sh3gl2	SH3-domain GRB2-like 2 [Source:MGI Symbol;Acc:MGI:700009]	0.403789613	-1.308324296	7.43E-06	4.42E-05	yes	down	9.232	0.94	6.17	2.64	2.3	33.41	1.64	0.75	0.94	1.38	1.05	0.58
ENSMUSG00000040711	Sh3pxd2b	SH3 and PX domains 2B [Source:MGI Symbol;Acc:MGI:2442062]	2.257436021	1.1746851	4.81E-44	3.88E-42	yes	up	9.618	18.448	10.39	10.07	8.85	8.68	10.1	18.79	18.53	17.49	17.79	19.64
ENSMUSG00000022322	Shcbp1	Shc SH2-domain binding protein 1 [Source:MGI Symbol;Acc:MGI:1338802]	2.113481687	1.079621612	0.000836397	0.003333097	yes	up	1.124	1.492	0.55	0.8	1.31	1.97	0.99	1.71	1.85	0.9	1.18	1.82
ENSMUSG00000022494	Shisa9	shisa family member 9 [Source:MGI Symbol;Acc:MGI:1919805]	0.286491081	-1.80343787	0.000192269	0.000880965	yes	down	0.504	0.118	0.8	0.59	0.19	0.51	0.43	0.11	0.18	0.06	0.09	0.15
ENSMUSG00000091722	Siah3	siah E3 ubiquitin protein ligase family member 3 [Source:MGI Symbol;Acc:MGI:2685758]	19.20319034	4.263274109	0.002162421	0.007791117	yes	up	0.002	0.058	0	0.01	0	0	0	0.01	0.05	0.1	0.02	0.11
ENSMUSG00000027322	Siglec1	sialic acid binding Ig-like lectin 1, sialoadhesin [Source:MGI Symbol;Acc:MGI:99668]	5.108643339	2.352940217	1.41E-40	9.89E-39	yes	up	3.616	16.222	3.89	3.65	3.9	1.91	4.73	13.41	19.5	15.8	17.05	15.35
ENSMUSG00000030474	Siglece	sialic acid binding Ig-like lectin E [Source:MGI Symbol;Acc:MGI:1932475]	2.704520633	1.435372904	4.08E-25	1.43E-23	yes	up	4.662	11.278	5.11	5.14	5.05	4.36	3.65	11.45	12.2	10.25	9.69	12.8

ENSMUSG00000039013	Siglecf	sialic acid binding Ig-like lectin F [Source:MGI Symbol;Acc:MGI:2681107]	3.337793081	1.738894521	2.04E-18	4.64E-17	yes	up	11.234	29.638	10.05	12.74	15.64	5.56	12.18	21.24	36.49	29.48	34.21	26.77
ENSMUSG00000024042	Sik1	salt inducible kinase 1 [Source:MGI Symbol;Acc:MGI:104754]	0.370584806	-1.432124361	9.27E-26	3.35E-24	yes	down	79.244	24.006	83.63	97.76	59.07	83.08	72.68	24.36	25.13	30.7	19.58	20.26
ENSMUSG00000037902	Sirpa	signal-regulatory protein alpha [Source:MGI Symbol;Acc:MGI:108563]	3.627593925	1.859012969	7.29E-106	3.12E-103	yes	up	48.048	155.988	47.67	44.03	52.58	42.94	53.02	146.48	162.55	160.31	142.09	168.51
ENSMUSG00000095788	Sirpb1a	signal-regulatory protein beta 1A [Source:MGI Symbol;Acc:MGI:2444824]	5.501378703	2.459793218	1.28E-96	4.15E-94	yes	up	6.702	31.052	6.04	8.36	7.61	5.44	6.06	29.91	35.06	29.88	30.99	29.42
ENSMUSG00000095028	Sirpb1b	signal-regulatory protein beta 1B [Source:MGI Symbol;Acc:MGI:3779828]	4.611083737	2.205105865	4.97E-46	4.36E-44	yes	up	8.512	30.18	6.35	11.14	11.22	8.38	5.47	25.8	33.51	27.3	34.77	29.52
ENSMUSG00000074677	Sirpb1c	signal-regulatory protein beta 1C [Source:MGI Symbol;Acc:MGI:3807521]	10.50206847	3.392601601	1.05E-207	3.76E-204	yes	up	10.914	97.554	9.33	10.6	12.64	9.68	12.32	83.96	95.31	89.23	108.01	111.26
ENSMUSG00000028460	Sit1	suppression inducing transmembrane adaptor 1 [Source:MGI Symbol;Acc:MGI:1889342]	2.925533605	1.54869979	1.70E-06	1.13E-05	yes	up	0.968	2.422	1.06	1.03	1.03	0.86	0.86	2.91	3.06	2.36	1.47	2.31
ENSMUSG00000036223	Ska1	spindle and kinetochore associated complex subunit 1 [Source:MGI Symbol;Acc:MGI:1913718]	2.439304941	1.286470123	0.009288329	0.028187452	yes	up	0.378	0.828	0.25	0.37	0.48	0.2	0.59	1.3	0.89	0.14	0.62	1.19
ENSMUSG00000021965	Ska3	spindle and kinetochore associated complex subunit 3 [Source:MGI Symbol;Acc:MGI:3041235]	2.292235441	1.196755234	0.000173436	0.000801015	yes	up	0.674	1.262	0.45	0.92	0.46	0.54	1	1.64	1.14	1.06	1.05	1.42
ENSMUSG00000070868	Skint3	selection and upkeep of intraepithelial T cells 3 [Source:MGI Symbol;Acc:MGI:3045331]	0.328501843	-1.606026632	2.09E-05	0.000115269	yes	down	1.698	0.468	1.69	2.08	1.43	2.36	0.93	0.24	0.32	0.74	0.76	0.28
ENSMUSG00000022372	Sla	src-like adaptor [Source:MGI Symbol;Acc:MGI:104295]	2.425320684	1.278175518	3.41E-37	2.12E-35	yes	up	26.95	56.17	24.54	32.89	26.57	24.53	26.22	59.23	59.43	61.4	45.83	54.96
ENSMUSG00000015314	Slamf6	SLAM family member 6 [Source:MGI Symbol;Acc:MGI:1353620]	2.744555918	1.456572733	2.06E-16	4.05E-15	yes	up	3.44	8.82	2.21	3.05	4.21	5.29	2.44	10.59	8.4	7.03	6.7	11.38
ENSMUSG00000038179	Slamf7	SLAM family member 7 [Source:MGI Symbol;Acc:MGI:1922595]	9.07290208	3.181564088	1.20E-98	4.01E-96	yes	up	13.008	72.824	6.5	33.47	8.63	10.19	6.25	80.69	58.76	66.74	69.95	87.98
ENSMUSG00000053318	Slamf8	SLAM family member 8 [Source:MGI Symbol;Acc:MGI:1921998]	9.456234078	3.241265748	2.98E-38	1.95E-36	yes	up	2.206	17.572	2.22	2.12	2.69	1.76	2.24	20.88	12.14	13.23	12.29	29.32
ENSMUSG00000026548	Slamf9	SLAM family member 9 [Source:MGI Symbol;Acc:MGI:1923692]	3.594816428	1.8459181	8.97E-32	4.55E-30	yes	up	9.76	29.742	7.99	10.96	11.42	7.69	10.74	22.01	32.01	28.96	29.47	36.26
ENSMUSG00000058921	Slc10a5	solute carrier family 10 (sodium/bile acid cotransporter family), member 5 [Source:MGI Symbol;Acc:MGI:2685251]	0.477047849	-1.067794116	4.26E-05	0.000220656	yes	down	1.584	0.634	1.54	1.61	1.11	2.08	1.58	0.47	0.41	0.93	0.74	0.62
ENSMUSG00000029321	Slc10a6	solute carrier family 10 (sodium/bile acid cotransporter family), member 6 [Source:MGI Symbol;Acc:MGI:1923000]	0.458876226	-1.123823032	5.50E-23	1.69E-21	yes	down	31.638	12.794	33.43	27.09	29.79	31.95	35.93	12.19	15.49	12.17	12.89	11.23
ENSMUSG00000026177	Slc11a1	solute carrier family 11 (proton-coupled divalent metal ion transporters), member 1 [Source:MGI Symbol;Acc:MGI:1345275]	6.815950589	2.768914877	1.04E-89	2.94E-87	yes	up	10.688	61.654	14.86	12.08	7.23	8.15	11.12	62.69	64.23	49.25	50.94	81.16
ENSMUSG00000017740	Slc12a5	solute carrier family 12, member 5 [Source:MGI Symbol;Acc:MGI:1862037]	0.428374037	-1.223057051	2.29E-05	0.000125028	yes	down	1.042	0.344	1.48	0.87	1.04	1.1	0.72	0.38	0.46	0.19	0.51	0.18

ENSMUSG00000018459 Slc13a3	solute carrier family 13 (sodium-dependent dicarboxylate transporter), member 3 [Source:MGI Symbol;Acc:MGI:2149635]	2.768496904	1.469102909	1.61E-08	1.43E-07	yes	up	0.842	2.002	0.6	0.5	0.8	0.92	1.39	1.79	2.27	1.51	2.11	2.33
ENSMUSG00000024737 Slc15a3	solute carrier family 15, member 3 [Source:MGI Symbol;Acc:MGI:1929691]	4.096759313	2.034483136	1.12E-52	1.20E-50	yes	up	15.07	46.274	17.54	11.61	13.86	7.88	24.46	42.86	47.61	42.88	48.69	49.33
ENSMUSG00000040938 Slc16a11	solute carrier family 16 (monocarboxylic acid transporters), member 11 [Source:MGI Symbol;Acc:MGI:2663709]	0.480317057	-1.057941052	2.39E-06	1.55E-05	yes	down	23.446	7.924	10.63	19.67	15.03	45.77	26.13	11.37	5.67	10.51	4.23	7.84
ENSMUSG00000045775 Slc16a5	solute carrier family 16 (monocarboxylic acid transporters), member 5 [Source:MGI Symbol;Acc:MGI:2443515]	4.513105958	2.17412065	1.25E-07	9.77E-07	yes	up	0.292	1.798	0.26	0.36	0.09	0.39	0.36	1.82	1.82	0.86	3.64	0.85
ENSMUSG00000041920 Slc16a6	solute carrier family 16 (monocarboxylic acid transporters), member 6 [Source:MGI Symbol;Acc:MGI:2144585]	2.359659625	1.23857877	4.70E-11	5.48E-10	yes	up	3.24	6.178	3	3.87	3.82	3.86	1.65	6.23	7.7	6.64	4.4	5.92
ENSMUSG00000061742 Slc22a12	solute carrier family 22 (organic anion/cation transporter), member 12 [Source:MGI Symbol;Acc:MGI:1195269]	0.038572044	-4.696300606	1.05E-07	8.35E-07	yes	down	2.316	0.06	3.31	1.71	2.17	1.21	3.18	0.05	0.11	0	0.14	0
ENSMUSG00000022366 Slc22a22	solute carrier family 22 (organic cation transporter), member 22 [Source:MGI Symbol;Acc:MGI:2446114]	0.053972551	-4.211630324	0.008803363	0.026921722	yes	down	0.166	0.032	0	0.14	0.1	0.56	0.03	0	0	0	0.16	0
ENSMUSG00000023828 Slc22a3	solute carrier family 22 (organic cation transporter), member 3 [Source:MGI Symbol;Acc:MGI:1333817]	0.390149466	-1.357901168	8.99E-11	1.02E-09	yes	down	2.372	7.736	1.79	1.94	2.51	2.96	2.66	0.67	35.8	0.8	0.75	0.66
ENSMUSG00000020334 Slc22a4	solute carrier family 22 (organic cation transporter), member 4 [Source:MGI Symbol;Acc:MGI:1353479]	2.73585922	1.451993995	1.61E-06	1.07E-05	yes	up	0.896	2.428	0.62	1.1	1.35	0.55	0.86	3.19	2.7	1.61	2.51	2.13
ENSMUSG00000063873 Slc24a3	solute carrier family 24 (sodium/potassium/calcium exchanger), member 3 [Source:MGI Symbol;Acc:MGI:2137513]	0.454630966	-1.137232144	9.74E-30	4.45E-28	yes	down	9.87	3.814	8.53	9.85	10.52	10.81	9.64	3.33	4.13	3.77	3.8	4.04
ENSMUSG00000040740 Slc25a34	solute carrier family 25, member 34 [Source:MGI Symbol;Acc:MGI:2686215]	0.199406995	-2.326212073	7.29E-05	0.000362871	yes	down	0.87	0.148	0.84	0.76	1.29	0.24	1.22	0.12	0.25	0.23	0	0.14
ENSMUSG00000037636 Slc25a43	solute carrier family 25, member 43 [Source:MGI Symbol;Acc:MGI:2684854]	2.896078201	1.534100559	7.69E-08	6.19E-07	yes	up	1.296	3.166	1.57	1.11	1.12	1.62	1.06	2.51	3.27	3.77	2.75	3.53
ENSMUSG00000001225 Slc26a3	solute carrier family 26, member 3 [Source:MGI Symbol;Acc:MGI:107181]	0.106771732	-3.22739835	0.010003078	0.030045712	yes	down	0.26	0.032	0.07	0.15	0.02	0.1	0.96	0	0.02	0.14	0	0
ENSMUSG00000020651 Slc26a4	solute carrier family 26, member 4 [Source:MGI Symbol;Acc:MGI:1346029]	5.568100725	2.477185309	1.32E-09	1.33E-08	yes	up	1.598	7.538	0.97	3.5	1.36	0.7	1.46	6.65	6.43	7.77	7.62	9.22
ENSMUSG00000024600 Slc27a6	solute carrier family 27 (fatty acid transporter), member 6 [Source:MGI Symbol;Acc:MGI:3036230]	3.077261409	1.621647004	8.69E-26	3.16E-24	yes	up	3.16	8.236	2.98	3	3.3	3.12	3.4	5.87	8.78	7.28	9.76	9.49
ENSMUSG00000020100 Slc29a3	solute carrier family 29 (nucleoside transporters), member 3 [Source:MGI Symbol;Acc:MGI:1918529]	2.670436099	1.417075362	4.61E-33	2.49E-31	yes	up	22.51	34.704	10.56	48.98	23.19	13.79	16.03	41.3	49.18	24.66	17.79	40.59

ENSMUSG00000036067 Slc2a6	solute carrier family 2 (facilitated glucose transporter), member 6 [Source:MGI Symbol;Acc:MGI:2443286]	3.15295013	1.656702351	2.89E-13	4.22E-12	yes	up	1.826	4.706	1.98	1.76	2.39	2.02	0.98	3.28	4.37	5.08	4.65	6.15
ENSMUSG00000037771 Slc32a1	solute carrier family 32 (GABA vesicular transporter), member 1 [Source:MGI Symbol;Acc:MGI:1194488]	28.830421	4.849519999	0.000619843	0.002533311	yes	up	0	0.1	0	0	0	0	0	0.08	0.19	0.11	0.1	0.02
ENSMUSG00000049922 Slc35c1	solute carrier family 35, member C1 [Source:MGI Symbol;Acc:MGI:2443301]	2.106748449	1.075018063	2.32E-37	1.47E-35	yes	up	9.422	16.832	8.34	9.79	9.01	10.62	9.35	15.05	17.42	16.91	17.67	17.11
ENSMUSG00000038602 Slc35f1	solute carrier family 35, member F1 [Source:MGI Symbol;Acc:MGI:2139810]	0.083527831	-3.581599221	0.005523572	0.017909587	yes	down	0.118	0.008	0.04	0.35	0.09	0.1	0.01	0	0	0	0.04	0
ENSMUSG00000057060 Slc35f3	solute carrier family 35, member F3 [Source:MGI Symbol;Acc:MGI:2444426]	0.483727762	-1.047732757	0.007142297	0.022470904	yes	down	0.948	0.388	0.82	0.72	1.34	1.04	0.82	0.46	0.48	0.54	0.32	0.14
ENSMUSG00000020261 Slc36a1	solute carrier family 36 (proton/amino acid symporter), member 1 [Source:MGI Symbol;Acc:MGI:2445299]	3.156060716	1.65812496	1.37E-41	1.00E-39	yes	up	5.278	14.4	5.5	4	5.78	5.13	5.98	14.41	16.92	13.56	11.97	15.14
ENSMUSG00000032122 Slc37a2	solute carrier family 37 (glycerol-3-phosphate transporter), member 2 [Source:MGI Symbol;Acc:MGI:1929693]	7.497837201	2.906474501	9.95E-148	9.69E-145	yes	up	1.442	10.07	1.41	1.27	1.59	1.41	1.53	9.82	9.09	10.37	9.48	11.59
ENSMUSG00000022464 Slc38a4	solute carrier family 38, member 4 [Source:MGI Symbol;Acc:MGI:1916604]	0.290289263	-1.784436881	2.78E-21	7.61E-20	yes	down	11.918	2.918	15.99	8.83	12.56	10.8	11.41	2.67	3.92	2.23	3.72	2.05
ENSMUSG00000031170 Slc38a5	solute carrier family 38, member 5 [Source:MGI Symbol;Acc:MGI:2148066]	0.457559145	-1.127969854	1.59E-13	2.40E-12	yes	down	21.744	8.39	21.88	18.35	20.25	28.23	20.01	6.28	8.94	7.14	11.13	8.46
ENSMUSG00000072572 Slc39a2	solute carrier family 39 (zinc transporter), member 2 [Source:MGI Symbol;Acc:MGI:2684326]	5.836668553	2.545145144	5.92E-20	1.50E-18	yes	up	2.31	11.36	2.08	2.28	3.91	0.7	2.58	8.58	13.11	11.33	10.3	13.48
ENSMUSG00000025993 Slc40a1	solute carrier family 40 (iron-regulated transporter), member 1 [Source:MGI Symbol;Acc:MGI:1315204]	2.035574578	1.025436079	2.47E-15	4.40E-14	yes	up	5.058	8.528	4.34	5.15	6.17	4.87	4.76	9.51	7.93	8.3	7.87	9.03
ENSMUSG00000010122 Slc47a1	solute carrier family 47, member 1 [Source:MGI Symbol;Acc:MGI:1914723]	0.203823303	-2.294609091	0.000632067	0.002579824	yes	down	4.634	0.176	0.84	1.65	0.38	19.53	0.77	0	0.09	0.49	0.1	0.2
ENSMUSG00000006574 Slc4a1	solute carrier family 4 (anion exchanger), member 1 [Source:MGI Symbol;Acc:MGI:109393]	0.459726325	-1.121152815	0.002620284	0.009253959	yes	down	0.966	0.336	0.72	0.31	0.96	1.37	1.47	0.44	0.2	0.24	0.46	0.34
ENSMUSG00000011034 Slc5a1	solute carrier family 5 (sodium/glucose cotransporter), member 1 [Source:MGI Symbol;Acc:MGI:107678]	2.970762062	1.57083306	9.68E-12	1.22E-10	yes	up	1.496	3.742	1.51	2.47	1.19	1.21	1.1	2.94	2.82	4	4.74	4.21
ENSMUSG00000041644 Slc5a12	solute carrier family 5 (sodium/glucose cotransporter), member 12 [Source:MGI Symbol;Acc:MGI:2138890]	0.195391677	-2.355559077	6.52E-23	1.99E-21	yes	down	2.828	0.432	3.07	2.97	2.6	2.83	2.67	0.44	0.27	0.67	0.47	0.31
ENSMUSG00000030109 Slc6a12	solute carrier family 6 (neurotransmitter transporter, betaine/GABA), member 12 [Source:MGI Symbol;Acc:MGI:95628]	74.4597124	6.218388139	2.97E-32	1.56E-30	yes	up	0.438	5.084	0.28	0	0.12	1.79	0	4.45	7.55	3.25	4.72	5.45

ENSMUSG00000036814 Slc6a20a	solute carrier family 6 (neurotransmitter transporter), member 20A [Source:MGI Symbol;Acc:MGI:2143217]	3.525969184	1.818019866	5.95E-36	3.56E-34	yes	up	4.85	10.856	2.53	5.56	9.2	3.38	3.58	9.81	12.71	10.32	11.04	10.4
ENSMUSG00000030096 Slc6a6	solute carrier family 6 (neurotransmitter transporter, taurine), member 6 [Source:MGI Symbol;Acc:MGI:98488]	2.03831743	1.027378742	3.26E-63	4.76E-61	yes	up	39.27	69.172	36.62	39.84	39.39	40.31	40.19	68.1	72.61	67.41	64.57	73.17
ENSMUSG00000030495 Slc7a10	solute carrier family 7 (cationic amino acid transporter, y+ system), member 10 [Source:MGI Symbol;Acc:MGI:1858261]	0.226310453	-2.143624875	2.65E-23	8.33E-22	yes	down	10.376	2.864	11.86	8.11	9.2	12.62	10.09	4.4	2.88	4.27	1.8	0.97
ENSMUSG00000027737 Slc7a11	solute carrier family 7 (cationic amino acid transporter, y+ system), member 11 [Source:MGI Symbol;Acc:MGI:1347355]	12.4580496	3.639006316	2.51E-67	4.17E-65	yes	up	0.358	3.064	0.3	0.48	0.36	0.42	0.23	3.94	2.41	2.62	2.96	3.39
ENSMUSG00000069072 Slc7a14	solute carrier family 7 (cationic amino acid transporter, y+ system), member 14 [Source:MGI Symbol;Acc:MGI:3040688]	0.147529413	-2.760925483	0.002658572	0.00936139	yes	down	0.078	0.014	0.05	0.06	0.1	0.04	0.14	0.01	0.05	0	0.01	0
ENSMUSG00000020600 Slc7a15	solute carrier family 7 (cationic amino acid transporter, y+ system), member 15 [Source:MGI Symbol;Acc:MGI:3045351]	0.405189451	-1.303331482	3.19E-05	0.000169092	yes	down	13.326	4.62	15.37	11.55	13.12	8.97	17.62	5.02	5.21	3.49	6.99	2.39
ENSMUSG00000031596 Slc7a2	solute carrier family 7 (cationic amino acid transporter, y+ system), member 2 [Source:MGI Symbol;Acc:MGI:99828]	3.327942155	1.734630357	1.21E-61	1.62E-59	yes	up	11.666	34.082	11.96	11.45	12.29	10.42	12.21	28.13	32.15	32.09	35.2	42.84
ENSMUSG00000022180 Slc7a8	solute carrier family 7 (cationic amino acid transporter, y+ system), member 8 [Source:MGI Symbol;Acc:MGI:1355323]	3.09035251	1.627771413	9.60E-27	3.69E-25	yes	up	1.582	4.216	1.48	1.58	1.87	1.27	1.71	3.86	4.84	3.83	3.75	4.8
ENSMUSG00000032754 Slc8b1	solute carrier family 8 (sodium/lithium/calcium exchanger), member B1 [Source:MGI Symbol;Acc:MGI:2180781]	2.630915268	1.395564785	1.21E-31	6.01E-30	yes	up	11.676	26.288	11.11	8.66	12.44	12.49	13.68	23.63	31.01	25.09	27.05	24.66
ENSMUSG00000026065 Slc9a4	solute carrier family 9 (sodium/hydrogen exchanger), member 4 [Source:MGI Symbol;Acc:MGI:105074]	6.941603872	2.795269039	5.23E-90	1.49E-87	yes	up	2.852	16.61	2.39	2.58	2.88	3.07	3.34	15.23	18.53	17.09	17.36	14.84
ENSMUSG00000037341 Slc9a7	solute carrier family 9 (sodium/hydrogen exchanger), member 7 [Source:MGI Symbol;Acc:MGI:2444530]	2.200177609	1.13761999	6.24E-13	8.80E-12	yes	up	3.028	5.754	2.49	3.68	3.05	2.79	3.13	4.99	6.64	6.92	4.74	5.48
ENSMUSG00000037994 Slc9b2	solute carrier family 9, subfamily B (NHA2, cation proton antiporter 2), member 2 [Source:MGI Symbol;Acc:MGI:2140077]	2.854968901	1.51347503	0.002881486	0.010061944	yes	up	0.226	0.426	0.07	0.12	0.27	0.28	0.39	0.22	0.84	0.15	0.42	0.5
ENSMUSG00000079262 Slco1a6	solute carrier organic anion transporter family, member 1a6 [Source:MGI Symbol;Acc:MGI:1351906]	0.22212436	-2.170560474	8.75E-13	1.22E-11	yes	down	2.53	0.488	3.14	2.25	2.35	1.77	3.14	0.53	0.33	0.62	0.78	0.18
ENSMUSG00000030737 Slco2b1	solute carrier organic anion transporter family, member 2b1 [Source:MGI Symbol;Acc:MGI:1351872]	2.038253397	1.027333419	8.85E-08	7.07E-07	yes	up	9.968	16.536	8.52	10.56	13.19	6.41	11.16	9.69	21.31	20.48	16.06	15.14
ENSMUSG00000038963 Slco4a1	solute carrier organic anion transporter family, member 4a1 [Source:MGI Symbol;Acc:MGI:1351866]	2.160693749	1.111494603	0.000158639	0.000738887	yes	up	0.546	1.11	0.37	0.57	0.51	0.54	0.74	1.02	1.47	1.07	1.04	0.95
ENSMUSG00000072621 Slfn10-ps	schlafen 10, pseudogene [Source:MGI Symbol;Acc:MGI:3512288]	4.129162683	2.04584926	6.06E-11	6.98E-10	yes	up	0.322	1.09	0.18	0.39	0.22	0.38	0.44	1.2	1.28	0.71	1.08	1.18

ENSMUSG00000072620 Slfn2	schlafen 2 [Source:MGI Symbol;Acc:MGI:1313258]	2.305359012	1.204991438	3.83E-30	1.79E-28	yes	up	44.644	86.67	40.43	49.63	47.19	47.89	38.08	99.02	79.5	77.9	79.09	97.84
ENSMUSG00000035208 Slfn8	schlafen 8 [Source:MGI Symbol;Acc:MGI:2672859]	2.878729282	1.525432123	3.07E-34	1.73E-32	yes	up	3.57	8.622	3.62	3.95	3.99	3.73	2.56	9.71	8.08	7.15	8.9	9.27
ENSMUSG00000069793 Slfn9	schlafen 9 [Source:MGI Symbol;Acc:MGI:2445121]	2.473878589	1.306774699	8.63E-15	1.46E-13	yes	up	1.276	2.654	1.18	1.1	1.68	1.42	1	2.51	2.69	2.24	2.51	3.32
ENSMUSG00000025020 Slit1	slit guidance ligand 1 [Source:MGI Symbol;Acc:MGI:1315203]	17.13377705	4.098771316	0.002188794	0.007876862	yes	up	0.022	0.06	0	0	0	0	0.11	0.06	0.01	0.04	0.05	0.14
ENSMUSG00000056427 Slit3	slit guidance ligand 3 [Source:MGI Symbol;Acc:MGI:1315202]	0.451909651	-1.145893727	3.50E-26	1.30E-24	yes	down	29.172	11.192	30.85	27.87	30.09	25.94	31.11	10.04	13.19	13.31	10.3	9.12
ENSMUSG00000017002 Slpi	secretory leukocyte peptidase inhibitor [Source:MGI Symbol;Acc:MGI:109297]	15.70342259	3.973007126	1.17E-54	1.32E-52	yes	up	44.532	574.084	45.13	58.7	43.62	26.34	48.87	665.02	548.96	450.6	553.4	652.44
ENSMUSG00000036867 Smad6	SMAD family member 6 [Source:MGI Symbol;Acc:MGI:1336883]	0.377238484	-1.406451236	1.17E-23	3.72E-22	yes	down	67.578	21.53	68.5	62.89	62.93	78.34	65.23	31.36	25.63	14.59	18.87	17.2
ENSMUSG00000085763 Smc2os	structural maintenance of chromosomes 2, opposite strand [Source:MGI Symbol;Acc:MGI:1919898]	0.081583378	-3.615580943	0.013252968	0.038230815	yes	down	0.556	0.028	0.52	0.18	0.72	0.61	0.75	0	0	0	0	0.14
ENSMUSG00000078439 Smim24	small integral membrane protein 24 [Source:MGI Symbol;Acc:MGI:1919523]	2.634947909	1.397774441	4.15E-10	4.39E-09	yes	up	3.47	7.756	2.43	3.73	4.05	2.87	4.27	6.69	7.74	7.22	7.92	9.21
ENSMUSG00000045709 Smkr-ps	smal lysine rich protein 1, pseudogene [Source:MGI Symbol;Acc:MGI:1923669]	0.442009217	-1.177851641	0.000440052	0.001865798	yes	down	6.638	2.606	8.25	7.57	3.73	5.75	7.89	1.72	3.09	2.24	2.15	3.83
ENSMUSG00000023886 Smoc2	SPARC related modular calcium binding 2 [Source:MGI Symbol;Acc:MGI:1929881]	0.469318203	-1.091361677	2.28E-09	2.24E-08	yes	down	11.086	4.306	10.13	10.38	13.29	9.47	12.16	3.43	4.01	6.69	3.73	3.67
ENSMUSG00000028885 Smpdl3b	sphingomyelin phosphodiesterase, acid-like 3B [Source:MGI Symbol;Acc:MGI:1916022]	4.112644879	2.040066503	6.60E-31	3.20E-29	yes	up	3.31	11.472	3.4	4.37	2.88	3.54	2.36	8.23	12.11	12.18	10.77	14.07
ENSMUSG00000081752 Sms-ps	spermine synthase, pseudogene [Source:MGI Symbol;Acc:MGI:3705601]	2.174625726	1.120767121	0.003602012	0.012250386	yes	up	4.166	7.772	2.5	2.24	4.56	2.82	8.71	10.12	10.93	6.61	3.1	8.1
ENSMUSG00000025889 Snca	synuclein, alpha [Source:MGI Symbol;Acc:MGI:1277151]	0.265532683	-1.913038649	9.44E-18	2.05E-16	yes	down	18.486	4.15	17.32	17.04	15.96	26.35	15.76	3.71	3.64	2.3	5.82	5.28
ENSMUSG00000025909 Sntg1	syntrophin, gamma 1 [Source:MGI Symbol;Acc:MGI:1918346]	0.392772313	-1.348234858	2.82E-10	3.04E-09	yes	down	9.39	1.598	5.73	5.38	27.12	3.41	5.31	1.31	2.36	1.49	1.85	0.98
ENSMUSG00000044772 Sntn	sentan, cilia apical structure protein [Source:MGI Symbol;Acc:MGI:3045373]	0.434805453	-1.201558061	2.76E-05	0.000148583	yes	down	14.526	5.334	13.99	18.63	8.81	13.76	17.44	5.7	4.29	9.14	2.43	5.11
ENSMUSG00000038301 Snx10	sorting nexin 10 [Source:MGI Symbol;Acc:MGI:1919232]	2.564523776	1.358690947	6.45E-32	3.31E-30	yes	up	16.274	35.13	16.74	18.29	19.72	12.36	14.26	34.75	32.34	34.72	34.57	39.27
ENSMUSG00000031662 Snx20	sorting nexin 20 [Source:MGI Symbol;Acc:MGI:1918857]	3.499333813	1.807080295	4.74E-36	2.85E-34	yes	up	7.458	21.684	6.75	8.86	8.21	8.06	5.41	23.48	21.21	20.64	18.09	25
ENSMUSG00000013611 Snx31	sorting nexin 31 [Source:MGI Symbol;Acc:MGI:1913946]	0.106145499	-3.235884903	4.77E-05	0.000244324	yes	down	0.608	0.058	0.89	0.3	0.67	0.86	0.32	0	0	0	0.17	0.12
ENSMUSG00000027423 Snx5	sorting nexin 5 [Source:MGI Symbol;Acc:MGI:1916428]	2.179267301	1.123843163	6.84E-52	7.18E-50	yes	up	68.378	125.69	70.87	69.66	75.03	62.81	63.52	136.53	123.83	120.42	121.69	125.98
ENSMUSG00000026600 Soat1	sterol O-acyltransferase 1 [Source:MGI Symbol;Acc:MGI:104665]	2.261989771	1.177592405	1.72E-81	3.83E-79	yes	up	43.85	91.724	41.42	46.24	45.33	46	40.26	88.04	95.36	95.01	88.5	91.71
ENSMUSG00000038037 Socs1	suppressor of cytokine signaling 1 [Source:MGI Symbol;Acc:MGI:1354910]	4.798530042	2.262592526	7.09E-07	4.96E-06	yes	up	0.284	1.152	0.09	0.39	0.14	0.46	0.34	1.74	0.68	1.26	0.51	1.57

ENSMUSG00000025006	Sorbs1	sorbin and SH3 domain containing 1 [Source:MGI Symbol;Acc:MGI:700014]	0.424865618	-1.234921494	5.80E-44	4.67E-42	yes	down	38.514	13.524	43.1	39.71	40.21	32.65	36.9	13.79	12.38	12.78	13.18	15.49
ENSMUSG00000022091	Sorbs3	sorbin and SH3 domain containing 3 [Source:MGI Symbol;Acc:MGI:700013]	0.492405214	-1.022082056	6.87E-23	2.09E-21	yes	down	109.666	45.9	128.51	88.23	103.54	110.7	117.35	42.32	54.03	43.25	48.32	41.58
ENSMUSG00000036169	Sostdc1	sclerostin domain containing 1 [Source:MGI Symbol;Acc:MGI:1913292]	5.59907489	2.485188477	0.009288058	0.028187452	yes	up	0.118	0.55	0	0.28	0.28	0.03	0	1.45	0.09	0.36	0.2	0.65
ENSMUSG00000105265	Sox2ot	SOX2 overlapping transcript (non-protein coding) [Source:MGI Symbol;Acc:MGI:2444112]	0.492859743	-1.02075095	0.011092411	0.032797884	yes	down	10.426	4.066	5.05	13.6	8.51	9.37	15.6	5.02	5.62	4.87	1.86	2.96
ENSMUSG00000051910	Sox6	SRY (sex determining region Y)-box 6 [Source:MGI Symbol;Acc:MGI:98368]	0.321148053	-1.638689545	1.77E-16	3.51E-15	yes	down	14.866	0.774	3.95	6.39	1.51	3.01	59.47	0.82	0.33	0.58	0.64	1.5
ENSMUSG00000070031	Sp140	Sp140 nuclear body protein [Source:MGI Symbol;Acc:MGI:3702467]	2.878653989	1.525394389	9.37E-50	9.12E-48	yes	up	13.364	33.512	15.11	13.81	12.83	12.67	12.4	36.84	31.71	32.2	30.64	36.17
ENSMUSG00000075304	Sp5	trans-acting transcription factor 5 [Source:MGI Symbol;Acc:MGI:1927715]	2.744414645	1.45649847	7.49E-05	0.000371819	yes	up	0.574	1.34	0.52	0.27	0.6	0.54	0.94	1.79	1.31	1.18	0.79	1.63
ENSMUSG00000001948	Spa17	sperm autoantigenic protein 17 [Source:MGI Symbol;Acc:MGI:1333778]	0.478150847	-1.064462263	1.17E-06	7.96E-06	yes	down	22.47	9.006	16.74	30.48	16.88	23.79	24.46	9.84	9.72	11.13	6.53	7.81
ENSMUSG00000053153	Spag16	sperm associated antigen 16 [Source:MGI Symbol;Acc:MGI:1913972]	0.44560261	-1.166170413	7.42E-08	5.98E-07	yes	down	7.348	2.772	7.5	8.24	6.21	5.94	8.85	2.62	2.2	4.66	1.56	2.82
ENSMUSG00000002055	Spag5	sperm associated antigen 5 [Source:MGI Symbol;Acc:MGI:1927470]	2.336192482	1.224159145	8.23E-06	4.88E-05	yes	up	0.922	2.012	1	0.64	0.92	0.58	1.47	1.86	1.7	2.39	1.45	2.66
ENSMUSG00000005233	Spc25	SPC25, NDC80 kinetochore complex component, homolog (S. cerevisiae) [Source:MGI Symbol;Acc:MGI:1913692]	2.322046544	1.21539689	1.34E-07	1.04E-06	yes	up	2.872	5.24	3.34	3.08	3.31	2.84	1.79	5.67	5.17	3.59	5.06	6.71
ENSMUSG00000069910	Spdl1	spindle apparatus coiled-coil protein 1 [Source:MGI Symbol;Acc:MGI:1917635]	3.879891529	1.956016319	2.23E-09	2.19E-08	yes	up	0.616	1.68	0.45	1	0.6	0.6	0.43	1.88	2.73	1.41	0.82	1.56
ENSMUSG00000073208	Speer4c	spermatogenesis associated glutamate (E)-rich protein 4C [Source:MGI Symbol;Acc:MGI:1914023]	3.73755495	1.90209479	0.01448249	0.041200453	yes	up	0.54	2.506	0.27	1.54	0	0.06	0.83	3.74	1.16	4.57	1.39	1.67
ENSMUSG00000002111	Spi1	spleen focus forming virus (SFFV) proviral integration oncogene [Source:MGI Symbol;Acc:MGI:98282]	3.52896718	1.819246013	8.80E-45	7.36E-43	yes	up	23.88	67.99	21.19	24.72	29.98	24.21	19.3	66.47	71	72.63	60.91	68.94
ENSMUSG00000004359	Spic	Spi-C transcription factor (Spi-1/PU.1 related) [Source:MGI Symbol;Acc:MGI:1341168]	3.903192562	1.964654639	1.17E-05	6.72E-05	yes	up	0.35	1.092	0.07	0.25	0.71	0.44	0.28	1.34	1.09	1.27	0.76	1
ENSMUSG00000053030	Spink2	serine peptidase inhibitor, Kazal type 2 [Source:MGI Symbol;Acc:MGI:1917232]	3.948205509	1.981197086	7.31E-11	8.36E-10	yes	up	3.272	10.97	4.28	3.4	2.12	3.59	2.97	6.2	13.34	14.01	10.58	10.72
ENSMUSG00000037379	Spon2	spondin 2, extracellular matrix protein [Source:MGI Symbol;Acc:MGI:1923724]	0.244334838	-2.033068512	7.29E-08	5.89E-07	yes	down	35.598	7.692	34.74	35.59	36.18	34.29	37.19	4.14	16	5.77	4.52	8.03
ENSMUSG00000029304	Spp1	secreted phosphoprotein 1 [Source:MGI Symbol;Acc:MGI:98389]	7.34656944	2.877070725	2.75E-174	4.53E-171	yes	up	185.614	1154.988	163.73	209.3	171.62	170.11	213.31	1233.76	1084.53	1030.56	1212.5	1213.59
ENSMUSG00000068893	Sprr2a2	small proline-rich protein 2A2 [Source:MGI Symbol;Acc:MGI:3845026]	2.834816968	1.503255589	0.009614363	0.029041071	yes	up	0.812	1.696	1.49	0.7	0.7	0.12	1.05	1.34	0.72	1.98	1.87	2.57

ENSMUSG00000046997 Spsb4	splA/ryanodine receptor domain and SOCS box containing 4 [Source:MGI Symbol;Acc:MGI:2183445]	3.266028164	1.707537232	0.001013543	0.003964571	yes	up	0.254	0.552	0.12	0.19	0.18	0.46	0.32	0.52	0.5	0.54	0.36	0.84
ENSMUSG00000021061 Sptb	spectrin beta, erythrocytic [Source:MGI Symbol;Acc:MGI:98387]	0.366541026	-1.44795341	4.60E-11	5.37E-10	yes	down	0.99	0.308	1.01	1.2	0.7	1.25	0.79	0.24	0.34	0.4	0.26	0.3
ENSMUSG00000033740 St18	suppression of tumorigenicity 18 [Source:MGI Symbol;Acc:MGI:2446700]	11.18977092	3.484108596	3.01E-11	3.59E-10	yes	up	0.078	0.784	0.05	0.06	0.09	0	0.19	1.69	0.67	0.48	0.34	0.74
ENSMUSG00000052544 St6galnac3	ST6 (alpha-N-acetyl-neuraminyl-2,3-beta-galactosyl-1,3)-N-acetylgalactosaminide alpha-2,6-sialyltransferase 3 [Source:MGI Symbol;Acc:MGI:1341828]	0.443541108	-1.172860274	3.13E-16	6.00E-15	yes	down	13.05	4.636	16.08	10.2	13.53	13.21	12.23	2.76	5.35	4.78	4.63	5.66
ENSMUSG00000039037 St6galnac5	ST6 (alpha-N-acetyl-neuraminyl-2,3-beta-galactosyl-1,3)-N-acetylgalactosaminide alpha-2,6-sialyltransferase 5 [Source:MGI Symbol;Acc:MGI:1349471]	0.45684073	-1.130236813	0.00075202	0.00302661	yes	down	0.42	0.16	0.44	0.33	0.5	0.43	0.4	0.21	0.08	0.16	0.14	0.21
ENSMUSG00000003418 St8sia6	ST8 alpha-N-acetyl-neuraminide alpha-2,8-sialyltransferase 6 [Source:MGI Symbol;Acc:MGI:2386797]	2.104813839	1.073692639	0.013858826	0.039631542	yes	up	1.474	2.672	0.99	2.8	1.45	1	1.13	3.18	1.68	4.66	1.44	2.4
ENSMUSG00000035459 Stab2	stabilin 2 [Source:MGI Symbol;Acc:MGI:2178743]	2.396782257	1.261098849	1.64E-10	1.81E-09	yes	up	0.69	1.502	0.6	1.53	0.34	0.5	0.48	1.7	1.22	1.47	1.36	1.76
ENSMUSG00000017400 Stac2	SH3 and cysteine rich domain 2 [Source:MGI Symbol;Acc:MGI:2144518]	20.64165644	4.367486843	3.25E-188	8.70E-185	yes	up	1.052	18.29	0.88	1.45	1.03	0.81	1.09	15.47	21.62	17.89	19.34	17.13
ENSMUSG00000029254 Stap1	signal transducing adaptor family member 1 [Source:MGI Symbol;Acc:MGI:1926193]	2.183600272	1.126708782	3.79E-11	4.45E-10	yes	up	4.22	6.128	2.33	5.64	6.32	4.01	2.8	7.53	7.25	5.93	4.53	5.4
ENSMUSG00000026104 Stat1	signal transducer and activator of transcription 1 [Source:MGI Symbol;Acc:MGI:103063]	2.125909428	1.088080134	1.28E-13	1.96E-12	yes	up	29.81	57.522	33.73	27.63	33.09	26.73	27.87	65.81	44.44	50.94	45.96	80.46
ENSMUSG00000014813 Stc1	stanniocalcin 1 [Source:MGI Symbol;Acc:MGI:109131]	0.435601736	-1.198918391	3.05E-08	2.61E-07	yes	down	3.29	1.21	3.65	4.44	2.18	3.4	2.78	0.76	1.16	1.51	1.31	1.31
ENSMUSG00000031027 Stk33	serine/threonine kinase 33 [Source:MGI Symbol;Acc:MGI:2152419]	0.401055301	-1.318126913	3.05E-08	2.61E-07	yes	down	9.62	3.286	11.65	9.64	7.52	8.64	10.65	2.88	3.72	5	1.69	3.14
ENSMUSG00000027581 Stmn3	stathmin-like 3 [Source:MGI Symbol;Acc:MGI:1277137]	0.069551589	-3.845772722	0.01229159	0.035838896	yes	down	0.26	0.012	0	0.36	0.28	0.42	0.24	0.06	0	0	0	0
ENSMUSG00000063529 Stmnd1	stathmin domain containing 1 [Source:MGI Symbol;Acc:MGI:2686420]	0.390242827	-1.35755598	1.59E-08	1.41E-07	yes	down	12.808	4.232	11.95	14.85	8.59	12.33	16.32	3.07	3.84	6.79	3.07	4.39
ENSMUSG00000033855 Ston1	stonin 1 [Source:MGI Symbol;Acc:MGI:1924307]	0.469991155	-1.089294489	1.31E-30	6.20E-29	yes	down	59.938	21.694	68.83	51.34	72.82	51.56	55.14	22.89	24.99	19.34	22.62	18.63
ENSMUSG00000028801 Stpg1	sperm tail PG rich repeat containing 1 [Source:MGI Symbol;Acc:MGI:1926056]	0.49652877	-1.010050781	0.017032995	0.04737789	yes	down	15.222	0.692	1	1.07	12.83	0.61	60.6	0.81	1.21	0.64	0.31	0.49
ENSMUSG00000028327 Stra6l	STRA6-like [Source:MGI Symbol;Acc:MGI:1921402]	35.45021479	5.147722464	1.80E-30	8.55E-29	yes	up	0.242	7.412	0.07	0.54	0.29	0.06	0.25	7.96	7.21	7.07	7.63	7.19
ENSMUSG00000046314 Stxbp6	syntaxin binding protein 6 (amisyn) [Source:MGI	0.471581875	-1.084419826	1.22E-19	3.03E-18	yes	down	14.732	5.784	14.57	14.36	14.79	16.67	13.27	6.6	7.03	4.92	5.83	4.54

		Symbol;Acc:MGI:2384963]																		
ENSMUSG00000019178	Styxl1	serine/threonine/tyrosine interacting-like 1 [Source:MGI Symbol;Acc:MGI:1923821]	0.411420364	-1.281314891	0.006175623	0.019775291	yes	down	1.684	0.456	2.12	2.01	0.87	1.67	1.75	0.31	0.47	0.81	0.15	0.54
ENSMUSG00000006800	Sulf2	sulfatase 2 [Source:MGI Symbol;Acc:MGI:1919293]	2.044461923	1.031721194	2.05E-11	2.49E-10	yes	up	21.174	34.612	25.45	18.82	22.95	14.34	24.31	26.29	40.28	38.75	33.04	34.7
ENSMUSG00000029273	Sult1d1	sulfotransferase family 1D, member 1 [Source:MGI Symbol;Acc:MGI:1926341]	0.13827187	-2.854420414	0.015879585	0.044652993	yes	down	10.602	1.416	3.1	31.67	2.15	2.76	13.33	0.18	0.58	5.1	0.07	1.15
ENSMUSG00000021384	Susd3	sushi domain containing 3 [Source:MGI Symbol;Acc:MGI:1913579]	2.202236307	1.138969283	1.85E-09	1.83E-08	yes	up	4.54	8.104	3.03	4.37	6.45	6.19	2.66	9.6	7.86	7.43	7.02	8.61
ENSMUSG00000022340	Sybu	syntabulin (syntaxin-interacting) [Source:MGI Symbol;Acc:MGI:2442392]	0.359874676	-1.474433512	9.80E-09	8.95E-08	yes	down	10.194	9.768	1.79	2.03	43.22	1.84	2.09	46.2	0.35	0.92	0.77	0.6
ENSMUSG00000021457	Syk	spleen tyrosine kinase [Source:MGI Symbol;Acc:MGI:99515]	2.945205038	1.558368074	1.33E-100	4.93E-98	yes	up	18.682	46.914	19.01	18.25	18.62	20.75	16.78	50	50.02	44.05	43.35	47.15
ENSMUSG00000030554	Synm	synemin, intermediate filament protein [Source:MGI Symbol;Acc:MGI:2661187]	0.443094334	-1.174314218	6.63E-23	2.02E-21	yes	down	21.49	7.988	21.99	18.96	22.19	25.15	19.16	8.17	9.14	8.24	8.76	5.63
ENSMUSG00000056296	Synpr	synaptoporin [Source:MGI Symbol;Acc:MGI:1919253]	0.39479208	-1.340835048	0.006510952	0.020734358	yes	down	2.47	0.48	1.11	6.54	1.21	1.97	1.52	0.42	0.35	0.99	0.47	0.17
ENSMUSG00000049303	Syt12	synaptotagmin XII [Source:MGI Symbol;Acc:MGI:2159601]	2.508209873	1.32665807	0.000227284	0.001028201	yes	up	0.414	1.032	0.36	0.45	0.47	0.23	0.56	0.66	1.39	0.52	1.57	1.02
ENSMUSG00000058420	Syt17	synaptotagmin XVII [Source:MGI Symbol;Acc:MGI:104966]	0.437886695	-1.191370481	2.13E-06	1.39E-05	yes	down	2.498	0.914	2.49	1.84	2.53	3.65	1.98	0.87	0.9	0.82	1.22	0.76
ENSMUSG00000026452	Syt2	synaptotagmin II [Source:MGI Symbol;Acc:MGI:99666]	4.669473758	2.22325997	0.000259058	0.001157762	yes	up	0.128	0.472	0.04	0.2	0.33	0	0.07	1.17	0.21	0.25	0.55	0.18
ENSMUSG00000058159	T2	brachyury 2 [Source:MGI Symbol;Acc:MGI:104658]	0.406547456	-1.298504329	0.000667707	0.002715977	yes	down	4.926	2.322	2.24	0.84	4.95	1.69	14.91	0.69	8.67	1.14	0.56	0.55
ENSMUSG00000020081	Tacr2	tachykinin receptor 2 [Source:MGI Symbol;Acc:MGI:98477]	0.044633194	-4.48573914	0.000522336	0.002178026	yes	down	0.26	0.008	0.39	0.34	0	0.26	0.31	0.02	0	0.02	0	0
ENSMUSG00000032085	Tagln	transgelin [Source:MGI Symbol;Acc:MGI:106012]	0.380075218	-1.395643135	4.02E-20	1.03E-18	yes	down	160.568	51.376	157.59	224.88	109.26	159.03	152.08	47.78	51	60.17	42.43	55.5
ENSMUSG00000037321	Tap1	transporter 1, ATP-binding cassette, sub-family B (MDR/TAP) [Source:MGI Symbol;Acc:MGI:98483]	2.358711601	1.237999031	3.51E-16	6.69E-15	yes	up	29.946	57.736	30.66	26.92	28.81	34.05	29.29	65.18	46.3	48.06	47.85	81.29
ENSMUSG00000053338	Tarm1	T cell-interacting, activating receptor on myeloid cells 1 [Source:MGI Symbol;Acc:MGI:2442280]	7.665615183	2.938401576	2.46E-08	2.13E-07	yes	up	0.362	2.346	0.24	0.6	0.05	0.68	0.24	3.52	1.7	0.74	2.16	3.61
ENSMUSG00000028950	Tas1r1	taste receptor, type 1, member 1 [Source:MGI Symbol;Acc:MGI:1927505]	5.905039546	2.561946721	0.009156081	0.027861131	yes	up	0.03	0.148	0.02	0.05	0	0.06	0.02	0.17	0.07	0.3	0.02	0.18
ENSMUSG00000001670	Tat	tyrosine aminotransferase [Source:MGI Symbol;Acc:MGI:98487]	0.154465746	-2.694641147	5.57E-06	3.39E-05	yes	down	1.72	0.226	1.21	2.21	0.98	2.18	2.02	0.61	0.33	0.07	0.12	0
ENSMUSG00000020096	Tbata	thymus, brain and testes associated [Source:MGI Symbol;Acc:MGI:1923820]	0.056075004	-4.156498373	1.25E-10	1.40E-09	yes	down	8.628	0.11	2.06	2.73	1.32	33.94	3.09	0	0.39	0.08	0	0.08
ENSMUSG00000031709	Tbc1d9	TBC1 domain family, member 9 [Source:MGI Symbol;Acc:MGI:1918560]	2.426920355	1.279126764	7.36E-49	7.01E-47	yes	up	35.166	180.6	46.16	32.01	53.1	33.59	10.97	85.3	255.55	205.23	136.9	220.02
ENSMUSG00000039233	Tbce	tubulin-specific chaperone E [Source:MGI Symbol;Acc:MGI:1917680]	0.395093721	-1.339733175	9.85E-08	7.83E-07	yes	down	102.354	42.96	122.71	99.3	91.4	88.42	109.94	47.29	49.07	40.34	49	29.1

ENSMUSG00000087516 Tbx3os1	T-box 3, opposite strand 1 [Source:MGI Symbol;Acc:MGI:3780472]	0.344715632	-1.536521374	4.29E-06	2.67E-05	yes	down	3.814	0.864	5.42	1.54	2.61	4.11	5.39	0.44	1.06	0.87	1.24	0.71
ENSMUSG00000029925 Tbxas1	thromboxane A synthase 1, platelet [Source:MGI Symbol;Acc:MGI:98497]	6.992025812	2.805710511	8.77E-65	1.36E-62	yes	up	8.128	41.818	11.62	9.38	7.42	4.38	7.84	32.68	48.02	36.58	46.71	45.1
ENSMUSG00000006642 Tcf23	transcription factor 23 [Source:MGI Symbol;Acc:MGI:1934960]	0.48332412	-1.048937101	0.002958236	0.010304747	yes	down	2.266	0.934	2.56	1.9	1.67	1.11	4.09	1.32	1.06	0.59	1.24	0.46
ENSMUSG00000001750 Tcirg1	T cell, immune regulator 1, ATPase, H+ transporting, lysosomal V0 protein A3 [Source:MGI Symbol;Acc:MGI:1350931]	2.910489007	1.541261569	9.62E-52	1.00E-49	yes	up	26.448	64.376	26.01	27.29	26.75	30.17	22.02	59.3	91.73	56.92	52.5	61.43
ENSMUSG00000076749 Tcrg-C1	T cell receptor gamma, constant 1 [Source:MGI Symbol;Acc:MGI:98625]	2.474993757	1.307424886	8.20E-07	5.69E-06	yes	up	3.72	7.668	3.85	4.02	3.8	4.28	2.65	7.81	6.62	6.36	4.88	12.67
ENSMUSG00000076752 Tcrg-C2	T-cell receptor gamma, constant 2 [Source:MGI Symbol;Acc:MGI:98626]	3.740230516	1.903127188	2.46E-12	3.30E-11	yes	up	2.224	6.828	2.01	1.74	2.36	2.4	2.61	5.68	6.84	4.69	6.18	10.75
ENSMUSG00000076757 Tcrg-C4	T cell receptor gamma, constant 4 [Source:MGI Symbol;Acc:MGI:98628]	2.331641755	1.221346143	0.000586467	0.002417159	yes	up	1.232	2.436	2.42	0.68	1.63	0.78	0.65	2.04	2.15	2.04	2.82	3.13
ENSMUSG00000029217 Tec	tec protein tyrosine kinase [Source:MGI Symbol;Acc:MGI:98662]	2.481212243	1.311045149	1.81E-28	7.76E-27	yes	up	5.924	12.144	5.78	6.81	6.37	5.47	5.19	13.08	10.82	11.82	10.86	14.14
ENSMUSG00000049537 Tecrl	trans-2,3-enoyl-CoA reductase-like [Source:MGI Symbol;Acc:MGI:2444966]	0.246040052	-2.02303491	8.97E-08	7.16E-07	yes	down	1.674	0.352	1.32	1.79	1.81	1.02	2.43	0.24	0.38	0.55	0.43	0.16
ENSMUSG00000037466 Tedc1	tubulin epsilon and delta complex 1 [Source:MGI Symbol;Acc:MGI:2144738]	2.507279848	1.32612303	8.51E-05	0.000418833	yes	up	0.584	1.232	0.66	0.87	0.37	0.68	0.34	1.3	1.23	1.19	0.52	1.92
ENSMUSG00000024175 Tekt4	tektin 4 [Source:MGI Symbol;Acc:MGI:1919090]	0.462163368	-1.113525183	1.55E-06	1.03E-05	yes	down	10.044	3.742	12.96	9.57	8.33	8.81	10.55	4.28	2.92	5.22	2.53	3.76
ENSMUSG00000032265 Tent5a	terminal nucleotidyltransferase 5A [Source:MGI Symbol;Acc:MGI:2670964]	2.201011356	1.138166589	6.61E-27	2.57E-25	yes	up	4.43	8.214	3.68	5.08	5	4.3	4.09	8.75	8.64	8.23	8.07	7.38
ENSMUSG00000046694 Tent5b	terminal nucleotidyltransferase 5B [Source:MGI Symbol;Acc:MGI:2140500]	0.449046825	-1.155062202	8.87E-11	1.00E-09	yes	down	13.948	5.304	14.39	14.58	11.41	11.31	18.05	5.37	4.99	6.73	3.79	5.64
ENSMUSG00000021611 Tert	telomerase reverse transcriptase [Source:MGI Symbol;Acc:MGI:1202709]	2.693205548	1.429324342	0.000617108	0.00252358	yes	up	0.438	1.116	0.43	0.21	0.95	0.23	0.37	1.74	0.75	0.85	0.41	1.83
ENSMUSG00000034833 Tespa1	thymocyte expressed, positive selection associated 1 [Source:MGI Symbol;Acc:MGI:1914846]	2.320329736	1.214329837	4.71E-05	0.000241467	yes	up	1.776	2.844	1.14	1.36	1.73	3.65	1	3.56	1.99	2.98	3.15	2.54
ENSMUSG00000021359 Tfap2a	transcription factor AP-2, alpha [Source:MGI Symbol;Acc:MGI:104671]	3.254879739	1.70260424	0.00053303	0.002217008	yes	up	0.28	0.414	0.29	0.62	0.21	0.17	0.11	0.19	0.54	0.56	0.45	0.33
ENSMUSG00000029553 Tfec	transcription factor EC [Source:MGI Symbol;Acc:MGI:1333760]	5.144867263	2.363133856	6.79E-33	3.62E-31	yes	up	5.056	21.226	4.66	6.35	6.82	2.98	4.47	16.48	22.69	20.37	25.47	21.12
ENSMUSG00000024032 Tff1	trefoil factor 1 [Source:MGI Symbol;Acc:MGI:88135]	55.94156711	5.805848764	2.45E-06	1.59E-05	yes	up	0	1.174	0	0	0	0	0	1.8	0.22	1.2	1.08	1.57
ENSMUSG00000022797 Tfrc	transferrin receptor [Source:MGI Symbol;Acc:MGI:98822]	0.493441583	-1.019048797	5.63E-14	8.85E-13	yes	down	28.27	11.778	28.48	37.87	24.82	27.95	22.23	12.28	10.43	10.12	12.86	13.2
ENSMUSG00000035493 Tgfbi	transforming growth factor, beta induced [Source:MGI	2.604496096	1.381004274	8.83E-26	3.21E-24	yes	up	41.734	92.114	34.3	53.66	41.53	35.78	43.4	81.2	91.73	85.79	84.23	117.62

		Symbol;Acc:MGI:99959]																	
ENSMUSG00000007613	Tgfbr1	transforming growth factor, beta receptor I [Source:MGI Symbol;Acc:MGI:98728]	2.510653715	1.328063057	1.32E-59	1.71E-57	yes	up	24.474	52.192	24.83	25.33	28.36	21.19	22.66	53.2	54.56	50.8	51.93	50.47
ENSMUSG00000029287	Tgfbr3	transforming growth factor, beta receptor III [Source:MGI Symbol;Acc:MGI:104637]	0.387320226	-1.368401255	2.92E-44	2.38E-42	yes	down	34.834	11.482	39.71	28.23	35.37	33.36	37.5	10.91	12.23	11.91	12.17	10.19
ENSMUSG00000022218	Tgm1	transglutaminase 1, K polypeptide [Source:MGI Symbol;Acc:MGI:98730]	2.17697227	1.122323031	3.96E-05	0.000206005	yes	up	1.794	3.52	2	2.53	1.24	0.9	2.3	2.92	4.55	3.05	2.56	4.52
ENSMUSG00000078922	Tgtp1	T cell specific GTPase 1 [Source:MGI Symbol;Acc:MGI:98734]	4.761500089	2.25141616	5.70E-10	5.94E-09	yes	up	4.842	18.61	3.11	6.8	5.6	3.14	5.56	25.16	10.52	17.83	11.48	28.06
ENSMUSG00000040152	Thbs1	thrombospondin 1 [Source:MGI Symbol;Acc:MGI:98737]	0.363612837	-1.459524962	3.68E-05	0.0001927	yes	down	95.51	29.458	108.06	174.95	51.72	63.92	78.9	23.82	37.73	22.68	31.78	31.28
ENSMUSG00000021702	Thbs4	thrombospondin 4 [Source:MGI Symbol;Acc:MGI:1101779]	5.074782226	2.343345913	4.32E-06	2.68E-05	yes	up	0.27	0.984	0.06	0.46	0.41	0.32	0.1	0.44	0.85	1.68	0.74	1.21
ENSMUSG00000037731	Themis2	thymocyte selection associated family member 2 [Source:MGI Symbol;Acc:MGI:2446213]	3.607251907	1.850900175	1.43E-67	2.40E-65	yes	up	9.108	27.776	9.57	11	8.45	8.07	8.45	26.76	28.43	25.51	25.57	32.61
ENSMUSG00000021779	Thrb	thyroid hormone receptor beta [Source:MGI Symbol;Acc:MGI:98743]	0.487765388	-1.035740709	4.13E-15	7.17E-14	yes	down	5.226	1.754	5.54	4.18	5.42	6.71	4.28	1.82	1.65	1.91	1.82	1.57
ENSMUSG00000035686	Thrsp	thyroid hormone responsive [Source:MGI Symbol;Acc:MGI:109126]	0.191659217	-2.383384717	0.013418414	0.038588528	yes	down	23.658	3.814	4.05	60.06	39.67	2.43	12.08	1.54	2.49	11.93	1.15	1.96
ENSMUSG00000056130	Ticam2	toll-like receptor adaptor molecule 2 [Source:MGI Symbol;Acc:MGI:3040056]	6.419190829	2.68239145	1.42E-34	8.13E-33	yes	up	1.77	9.63	1.34	2.18	3.04	1.03	1.26	9.16	9.56	8.98	9.96	10.49
ENSMUSG00000046591	Ticrr	TOPBP1-interacting checkpoint and replication regulator [Source:MGI Symbol;Acc:MGI:1924261]	3.021754996	1.595386691	9.85E-07	6.75E-06	yes	up	0.166	0.428	0.12	0.24	0.16	0.19	0.12	0.38	0.28	0.48	0.46	0.54
ENSMUSG00000046688	Tifa	TRAF-interacting protein with forkhead-associated domain [Source:MGI Symbol;Acc:MGI:2182965]	3.195598963	1.676086366	1.33E-33	7.44E-32	yes	up	6.58	17.394	6.06	7.21	8.65	5.87	5.11	21.04	16.98	14.86	16.17	17.92
ENSMUSG00000049625	Tifab	TRAF-interacting protein with forkhead-associated domain, family member B [Source:MGI Symbol;Acc:MGI:2385852]	3.905235673	1.965409615	1.57E-69	2.85E-67	yes	up	6.5	20.94	5.61	7.29	7.73	6.41	5.46	18.96	21.71	20.86	20.12	23.05
ENSMUSG00000047819	Tigd4	tigger transposable element derived 4 [Source:MGI Symbol;Acc:MGI:2685264]	0.085226857	-3.552548053	0.00845839	0.026022825	yes	down	0.084	0.006	0.13	0.08	0.04	0.06	0.11	0	0	0.03	0	0
ENSMUSG00000071552	Tigit	T cell immunoreceptor with Ig and ITIM domains [Source:MGI Symbol;Acc:MGI:3642260]	5.575706943	2.479154736	1.24E-24	4.18E-23	yes	up	3.576	14.676	4.29	2.38	4.79	2.53	3.89	21.69	12.29	13.4	10.86	15.14
ENSMUSG00000055546	Timd4	T cell immunoglobulin and mucin domain containing 4 [Source:MGI Symbol;Acc:MGI:2445125]	0.393280143	-1.346370748	0.00176679	0.006520715	yes	down	1.02	0.342	0.75	1.35	1.5	0.65	0.85	0.16	0.27	0.53	0.5	0.25
ENSMUSG00000001131	Timp1	tissue inhibitor of metalloproteinase 1 [Source:MGI Symbol;Acc:MGI:98752]	2.031267634	1.022380337	7.65E-07	5.34E-06	yes	up	12.296	21.37	12.99	11.33	15.07	7.47	14.62	17.51	19.44	23.84	19.69	26.37
ENSMUSG00000025574	Tk1	thymidine kinase 1 [Source:MGI Symbol;Acc:MGI:98763]	2.065209082	1.046287848	7.13E-07	4.99E-06	yes	up	4.34	7.498	4.83	3.16	5.73	3.7	4.28	7.43	8.15	5.93	5.43	10.55

ENSMUSG00000052698 Tln2	talin 2 [Source:MGI Symbol;Acc:MGI:1917799]	0.485489327	-1.042488515	3.37E-37	2.10E-35	yes	down	7.596	3.422	7.85	7.51	7.31	8.02	7.29	2.73	3.04	3.36	4.69	3.29
ENSMUSG00000044827 Tlr1	toll-like receptor 1 [Source:MGI Symbol;Acc:MGI:1341295]	3.12256382	1.642731058	3.71E-09	3.56E-08	yes	up	2.242	6.088	1.77	3.25	1.76	3.02	1.41	7.66	6.01	4.98	3.1	8.69
ENSMUSG00000051969 Tlr11	toll-like receptor 11 [Source:MGI Symbol;Acc:MGI:3045226]	4.92040429	2.298776861	6.45E-13	9.08E-12	yes	up	0.65	2.65	0.53	0.7	1.14	0.67	0.21	3.59	2.11	3.06	1.97	2.52
ENSMUSG00000062545 Tlr12	toll-like receptor 12 [Source:MGI Symbol;Acc:MGI:3045221]	4.748911154	2.247596765	1.18E-12	1.63E-11	yes	up	0.84	3.346	1.27	0.42	0.46	1.46	0.59	3.07	2.61	3.76	2.35	4.94
ENSMUSG00000033777 Tlr13	toll-like receptor 13 [Source:MGI Symbol;Acc:MGI:3045213]	5.686254039	2.507478554	2.95E-165	4.31E-162	yes	up	5.33	25.558	5.17	6.26	5.15	5.06	5.01	26.95	25.74	22.99	25.03	27.08
ENSMUSG00000027995 Tlr2	toll-like receptor 2 [Source:MGI Symbol;Acc:MGI:1346060]	4.637838879	2.213452701	6.87E-60	8.97E-58	yes	up	11.768	46.272	10.97	13.63	13.52	7.88	12.84	42.53	45.86	50.05	43.86	49.06
ENSMUSG00000051498 Tlr6	toll-like receptor 6 [Source:MGI Symbol;Acc:MGI:1341296]	3.568917111	1.835486395	2.16E-20	5.65E-19	yes	up	2.142	7.35	2.79	0.78	2.04	4.39	0.71	10.15	7.46	4.29	10.44	4.41
ENSMUSG00000044583 Tlr7	toll-like receptor 7 [Source:MGI Symbol;Acc:MGI:2176882]	5.983831212	2.581069481	5.07E-184	1.09E-180	yes	up	5.712	33.004	4.87	6.46	7.01	5.2	5.02	28.25	47.63	29.93	31.77	27.44
ENSMUSG00000040522 Tlr8	toll-like receptor 8 [Source:MGI Symbol;Acc:MGI:2176887]	7.20731163	2.849461226	7.95E-126	5.78E-123	yes	up	9.31	53.812	7.94	9.95	9.1	6.12	13.44	53.58	55.1	50.18	57.66	52.54
ENSMUSG00000045322 Tlr9	toll-like receptor 9 [Source:MGI Symbol;Acc:MGI:1932389]	2.927872236	1.5498526	9.96E-18	2.16E-16	yes	up	2.03	5.268	1.7	1.86	2.42	2.32	1.85	6.31	4.83	5.4	3.36	6.44
ENSMUSG00000079625 Tm4sf19	transmembrane 4 L six family member 19 [Source:MGI Symbol;Acc:MGI:3645933]	35.59815175	5.153730433	4.59E-15	7.94E-14	yes	up	0.08	2.42	0	0.19	0	0.11	0.1	2.85	2.08	2.2	1.53	3.44
ENSMUSG00000018919 Tm4sf5	transmembrane 4 superfamily member 5 [Source:MGI Symbol;Acc:MGI:1922854]	5.137134415	2.360963823	0.000108096	0.000520219	yes	up	0.724	2.124	0.41	0.39	0.42	0	2.4	1.87	1.35	2.22	0.92	4.26
ENSMUSG00000038623 Tm6sf1	transmembrane 6 superfamily member 1 [Source:MGI Symbol;Acc:MGI:1933209]	2.083412503	1.058948513	3.48E-29	1.55E-27	yes	up	28.88	50.682	28.04	26.04	36.97	24.08	29.27	49.02	52.06	54.02	49.27	49.04
ENSMUSG00000042066 Tmcc2	transmembrane and coiled-coil domains 2 [Source:MGI Symbol;Acc:MGI:1916125]	0.391309991	-1.353616149	1.55E-44	1.28E-42	yes	down	125.83	41.342	139.55	122.43	118.34	141.18	107.65	42.89	46.53	33.49	44.78	39.02
ENSMUSG00000026109 Tmeff2	transmembrane protein with EGF-like and two follistatin-like domains 2 [Source:MGI Symbol;Acc:MGI:1861735]	0.301105624	-1.73165844	1.19E-18	2.78E-17	yes	down	6.174	22.538	7.01	6.58	5.24	7.3	4.74	1.19	106.67	1.26	2.33	1.24
ENSMUSG00000069763 Tmem100	transmembrane protein 100 [Source:MGI Symbol;Acc:MGI:1915138]	0.448687555	-1.156216925	6.67E-16	1.24E-14	yes	down	537.112	203.14	628.54	515.91	543.82	530.82	466.47	214.98	233.11	144.58	267.5	155.53
ENSMUSG00000034947 Tmem106a	transmembrane protein 106A [Source:MGI Symbol;Acc:MGI:1922056]	3.119933762	1.6415154	9.78E-42	7.25E-40	yes	up	15.076	44.182	13.72	18.75	16.77	12.27	13.87	38.48	45.38	41.02	41.92	54.11
ENSMUSG00000046456 Tmem150b	transmembrane protein 150B [Source:MGI Symbol;Acc:MGI:2679718]	2.417297616	1.273395108	7.41E-05	0.000367886	yes	up	1.28	2.446	1.2	1.73	1.36	1.13	0.98	1.53	4.56	1.93	1.97	2.24
ENSMUSG00000056498 Tmem154	transmembrane protein 154 [Source:MGI Symbol;Acc:MGI:2444725]	2.354453407	1.235392173	4.10E-25	1.43E-23	yes	up	10.472	21.01	12.64	10.09	12	7.42	10.21	21.34	22.62	19.69	21.63	19.77
ENSMUSG00000024349 Tmem173	transmembrane protein 173 [Source:MGI Symbol;Acc:MGI:1919762]	3.423321772	1.775396904	1.03E-44	8.55E-43	yes	up	13.72	31.738	11.65	15.59	12.72	17.81	10.83	37.06	26.55	30.24	25.21	39.63
ENSMUSG00000049526 Tmem202	transmembrane protein 202 [Source:MGI Symbol;Acc:MGI:1921143]	3.557705581	1.830947125	8.04E-05	0.000397144	yes	up	0.664	1.818	0.41	1.26	1.01	0.28	0.36	2.43	1.9	1.23	1.68	1.85
ENSMUSG00000036502 Tmem255a	transmembrane protein 255A [Source:MGI Symbol;Acc:MGI:3045722]	0.393013175	-1.347350417	0.002574607	0.009110673	yes	down	0.802	0.292	0.49	0.67	0.96	1.34	0.55	0.18	0.2	0.12	0.8	0.16
ENSMUSG00000045917 Tmem268	transmembrane protein 268 [Source:MGI Symbol;Acc:MGI:1913920]	2.342983822	1.228346992	9.72E-27	3.73E-25	yes	up	7.176	17.222	7.18	9.75	6.46	5.17	7.32	14.64	23.58	13.94	17.02	16.93
ENSMUSG00000059900 Tmem40	transmembrane protein 40 [Source:MGI Symbol;Acc:MGI:2137870]	0.353268591	-1.501162608	7.86E-06	4.67E-05	yes	down	2.96	0.898	3.25	3.14	2.88	2.67	2.86	0.95	0.74	1.14	0.5	1.16
ENSMUSG00000025666 Tmem47	transmembrane protein 47 [Source:MGI Symbol;Acc:MGI:2177570]	0.491658745	-1.02427079	6.59E-22	1.87E-20	yes	down	19.342	8.124	19.22	21.01	20.32	19.76	16.4	7.19	9.41	7.04	9.71	7.27
ENSMUSG00000040616 Tmem51	transmembrane protein 51 [Source:MGI Symbol;Acc:MGI:2384874]	2.087243247	1.061598742	9.59E-24	3.07E-22	yes	up	10.066	17.616	8.35	9.65	11.08	10.42	10.83	16.85	17.99	18.39	17.82	17.03

ENSMUSG00000010307	Tmem86a	transmembrane protein 86A [Source:MGI Symbol;Acc:MGI:1915143]	4.133189886	2.047255645	1.55E-38	1.03E-36	yes	up	6.798	23.758	6.04	5.07	8.99	7	6.89	18.4	25.12	21.02	23.98	30.27
ENSMUSG00000037129	Tmprss13	transmembrane protease, serine 13 [Source:MGI Symbol;Acc:MGI:2682935]	0.262173977	-1.931403604	3.36E-07	2.46E-06	yes	down	1.51	0.332	1.22	1.66	1.02	2.02	1.63	0.18	0.28	0.75	0.23	0.22
ENSMUSG00000036019	Tmtc2	transmembrane and tetratricopeptide repeat containing 2 [Source:MGI Symbol;Acc:MGI:1914057]	0.480314382	-1.057949087	9.37E-13	1.30E-11	yes	down	24.64	10.124	28.29	31.52	22.77	24.84	15.78	7.61	9.26	11.14	11.27	11.34
ENSMUSG00000024401	Tnf	tumor necrosis factor [Source:MGI Symbol;Acc:MGI:104798]	4.459663745	2.156934936	9.18E-05	0.000448121	yes	up	0.234	0.816	0.25	0.27	0.31	0.17	0.17	1.38	0.58	0.87	0.29	0.96
ENSMUSG00000021281	Tnfaip2	tumor necrosis factor, alpha-induced protein 2 [Source:MGI Symbol;Acc:MGI:104960]	3.298887965	1.721979783	1.68E-47	1.52E-45	yes	up	22.534	50.604	21.6	30.11	27.26	15.01	18.69	47.6	51.46	48.13	46.52	59.31
ENSMUSG00000053475	Tnfaip6	tumor necrosis factor alpha induced protein 6 [Source:MGI Symbol;Acc:MGI:1195266]	0.466836389	-1.099011075	0.009975736	0.029976198	yes	down	1.726	0.68	2.24	1.92	2.33	0.83	1.31	0.24	0.45	0.67	0.87	1.17
ENSMUSG00000062210	Tnfaip8	tumor necrosis factor, alpha-induced protein 8 [Source:MGI Symbol;Acc:MGI:2147191]	2.174609255	1.120756193	1.47E-21	4.07E-20	yes	up	26.28	47.236	31.42	28.9	28.05	18.74	24.29	51.86	44.94	51.26	42.31	45.81
ENSMUSG00000044469	Tnfaip8l1	tumor necrosis factor, alpha-induced protein 8-like 1 [Source:MGI Symbol;Acc:MGI:1913693]	2.021727432	1.015588507	2.77E-05	0.000149174	yes	up	1.746	2.89	1.1	2.03	1.57	1.92	2.11	1.88	3.34	3.14	3.09	3
ENSMUSG00000013707	Tnfaip8l2	tumor necrosis factor, alpha-induced protein 8-like 2 [Source:MGI Symbol;Acc:MGI:1917019]	4.17326103	2.061175162	7.65E-72	1.46E-69	yes	up	10.688	37.592	10.32	12.15	11.92	10.28	8.77	37.31	40.85	32.63	40.11	37.06
ENSMUSG00000026321	Tnfrsf11a	tumor necrosis factor receptor superfamily, member 11a, NFKB activator [Source:MGI Symbol;Acc:MGI:1314891]	2.174748129	1.120848323	4.65E-15	8.03E-14	yes	up	2.228	3.684	1.69	2.2	2.69	1.7	2.86	3.09	4.18	3.79	3.24	4.12
ENSMUSG00000010142	Tnfrsf13b	tumor necrosis factor receptor superfamily, member 13b [Source:MGI Symbol;Acc:MGI:1889411]	2.726798806	1.447208256	2.78E-16	5.38E-15	yes	up	13.424	25.68	9.77	19.35	11.59	15.56	10.85	33.98	25.88	20.73	18.29	29.52
ENSMUSG00000022496	Tnfrsf17	tumor necrosis factor receptor superfamily, member 17 [Source:MGI Symbol;Acc:MGI:1343050]	5.693428362	2.509297649	1.63E-14	2.68E-13	yes	up	1.506	7.222	0.34	2.32	1.96	1.37	1.54	6.88	7.08	7.85	7.84	6.46
ENSMUSG00000041954	Tnfrsf18	tumor necrosis factor receptor superfamily, member 18 [Source:MGI Symbol;Acc:MGI:894675]	2.235734034	1.160748574	1.36E-08	1.23E-07	yes	up	4.168	7.778	3.95	4.36	3.34	4.01	5.18	6.72	7.52	9.18	6.13	9.34
ENSMUSG00000028599	Tnfrsf1b	tumor necrosis factor receptor superfamily, member 1b [Source:MGI Symbol;Acc:MGI:1314883]	5.281831901	2.401038387	0	0	yes	up	22.264	97.054	21.6	22.25	22.79	21.66	23.02	91.61	99.23	93.7	99.92	100.81
ENSMUSG00000037613	Tnfrsf23	tumor necrosis factor receptor superfamily, member 23 [Source:MGI Symbol;Acc:MGI:1930269]	4.007467596	2.002690855	3.57E-35	2.07E-33	yes	up	2.266	6.772	2.1	2.49	2.66	1.78	2.3	7.52	7.35	6.17	6.23	6.59
ENSMUSG00000045362	Tnfrsf26	tumor necrosis factor receptor superfamily, member 26 [Source:MGI Symbol;Acc:MGI:2651928]	5.103372934	2.351451072	8.50E-45	7.14E-43	yes	up	1.404	6.018	1.59	1.72	1.31	1.14	1.26	7.24	6	5.59	6.31	4.95
ENSMUSG00000029075	Tnfrsf4	tumor necrosis factor receptor superfamily, member 4 [Source:MGI	3.093552011	1.62926429	9.26E-07	6.38E-06	yes	up	2.184	5.568	2.3	1.64	0.65	2.46	3.87	5.51	5.58	6.08	3.43	7.24

		Symbol;Acc:MGI:104512]																		
ENSMUSG00000028602	Tnfrsf8	tumor necrosis factor receptor superfamily, member 8 [Source:MGI Symbol;Acc:MGI:99908]	4.751935231	2.248515172	3.73E-09	3.58E-08	yes	up	0.324	1.382	0.24	0.43	0.24	0.56	0.15	1.14	1.05	1.06	2.05	1.61
ENSMUSG00000028965	Tnfrsf9	tumor necrosis factor receptor superfamily, member 9 [Source:MGI Symbol;Acc:MGI:1101059]	8.297470475	3.052671591	1.77E-35	1.04E-33	yes	up	1.162	9.022	0.76	1.69	0.6	1.33	1.43	9.35	9.86	7.66	8.21	10.03
ENSMUSG00000022015	Tnfsf11	tumor necrosis factor (ligand) superfamily, member 11 [Source:MGI Symbol;Acc:MGI:1100089]	2.457312188	1.297081156	0.001855115	0.0068076	yes	up	0.38	0.784	0.36	0.28	0.77	0.34	0.15	0.91	0.66	0.84	0.66	0.85
ENSMUSG00000089669	Tnfsf13	tumor necrosis factor (ligand) superfamily, member 13 [Source:MGI Symbol;Acc:MGI:1916833]	2.151105896	1.105078548	1.16E-23	3.69E-22	yes	up	19.704	36.016	21.63	16.94	20.07	17.74	22.14	31.81	36.05	33.99	39.68	38.55
ENSMUSG00000028362	Tnfsf8	tumor necrosis factor (ligand) superfamily, member 8 [Source:MGI Symbol;Acc:MGI:88328]	2.769002979	1.469366606	6.27E-10	6.50E-09	yes	up	0.966	2.548	1.09	0.98	1.01	1.15	0.6	4.73	1.77	2.09	1.63	2.52
ENSMUSG00000044162	Tnip3	TNFAIP3 interacting protein 3 [Source:MGI Symbol;Acc:MGI:3041165]	2.755431498	1.462278261	2.82E-08	2.43E-07	yes	up	1.406	4.01	0.88	2.41	1.26	0.93	1.55	3.99	3.59	5.24	2.6	4.63
ENSMUSG00000031097	Tnni2	troponin I, skeletal, fast 2 [Source:MGI Symbol;Acc:MGI:105070]	2.133017369	1.092895713	0.001633152	0.006073205	yes	up	6.366	12.738	4.94	10.12	3.38	2.61	10.78	5.01	17.83	17.62	10.55	12.68
ENSMUSG00000064179	Tnnt1	troponin T1, skeletal, slow [Source:MGI Symbol;Acc:MGI:1333868]	0.393916132	-1.344039594	0.002673538	0.009407903	yes	down	2.938	1.396	1.8	3.32	3.79	3.07	2.71	2.2	0.99	0.94	0.82	2.03
ENSMUSG00000061723	Tnnt3	troponin T3, skeletal, fast [Source:MGI Symbol;Acc:MGI:109550]	0.236402982	-2.08067986	0.006654094	0.021152452	yes	down	3.828	0.726	1.61	10.58	0.81	3.51	2.63	0.95	0.32	1.67	0	0.69
ENSMUSG00000015829	Tnr	tenascin R [Source:MGI Symbol;Acc:MGI:99516]	0.084270095	-3.568835442	1.59E-06	1.06E-05	yes	down	0.186	0.008	0.04	0.06	0.12	0.21	0.5	0	0.01	0.02	0.01	0
ENSMUSG00000037003	Tns2	tensin 2 [Source:MGI Symbol;Acc:MGI:2387586]	0.498857463	-1.003300439	9.02E-30	4.13E-28	yes	down	132.542	56.08	141.09	109.57	139.66	143.16	129.23	47.36	57.67	55.72	61.32	58.33
ENSMUSG00000017607	Tns4	tensin 4 [Source:MGI Symbol;Acc:MGI:2144377]	4.528210947	2.178941168	3.65E-19	8.83E-18	yes	up	1.066	2.422	1.09	0.92	0.86	0.49	1.97	1.82	2.77	2.39	1.95	3.18
ENSMUSG00000092200	Tnxa	tenascin XA (pseudogene) [Source:MGI Symbol;Acc:MGI:2148489]	0.407084936	-1.296598257	0.003462491	0.011819047	yes	down	8.738	3.008	11.08	7.23	9.5	6.41	9.47	1.39	3.93	3.7	3.59	2.43
ENSMUSG00000020914	Top2a	topoisomerase (DNA) II alpha [Source:MGI Symbol;Acc:MGI:98790]	2.87096999	1.52153825	4.96E-09	4.69E-08	yes	up	7.202	18.57	7.54	4.69	10.26	5.12	8.4	20.67	16.42	16.93	11.84	26.99
ENSMUSG00000041272	Tox	thymocyte selection-associated high mobility group box [Source:MGI Symbol;Acc:MGI:2181659]	2.025824214	1.018508993	5.44E-06	3.32E-05	yes	up	1.032	1.884	1.01	0.71	1.3	0.95	1.19	2.42	1.48	2.1	1.46	1.96
ENSMUSG00000000296	Tpd52l1	tumor protein D52-like 1 [Source:MGI Symbol;Acc:MGI:1298386]	0.432439397	-1.209430129	0.007077345	0.022289475	yes	down	2.178	0.86	1.92	4.26	1.56	1.26	1.89	0.76	0.62	0.67	1.44	0.81
ENSMUSG00000028464	Tpm2	tropomyosin 2, beta [Source:MGI Symbol;Acc:MGI:98810]	0.401760536	-1.315592235	3.29E-11	3.90E-10	yes	down	77.066	26.088	68.25	124.36	49.66	66.78	76.28	22.32	23.16	30.81	23	31.15
ENSMUSG00000033825	Tpsb2	tryptase beta 2 [Source:MGI Symbol;Acc:MGI:96942]	0.0676585	-3.885584993	0.001711662	0.00634313	yes	down	0.794	0.048	0	2.71	0.65	0.31	0.3	0	0.07	0.05	0.12	0
ENSMUSG00000027469	Tpx2	TPX2, microtubule-associated [Source:MGI Symbol;Acc:MGI:1919369]	2.617759064	1.388332319	1.98E-10	2.17E-09	yes	up	2.402	5.236	2.57	2.01	3.27	1.72	2.44	5.33	5.03	4.53	3.49	7.8
ENSMUSG00000076928	Trac	T cell receptor alpha constant [Source:MGI Symbol;Acc:MGI:4439838]	2.435207467	1.284044687	7.97E-12	1.01E-10	yes	up	16.45	37.438	15.69	16.87	18.01	17.28	14.4	44.35	29.87	40.04	26.99	45.94
ENSMUSG00000037318	Traf3ip3	TRAF3 interacting protein 3 [Source:MGI Symbol;Acc:MGI:2441706]	2.076762076	1.054335944	5.41E-09	5.09E-08	yes	up	8.688	16.322	8.74	10.79	9.66	8.39	5.86	21.73	12.04	17.97	10.86	19.01
ENSMUSG00000044528	Tram1l1	translocation associated membrane protein 1-like 1 [Source:MGI Symbol;Acc:MGI:2443503]	0.428646297	-1.222140415	0.017330396	0.048107293	yes	down	0.28	0.102	0.46	0.22	0.17	0.27	0.28	0.04	0.13	0.09	0.08	0.17
ENSMUSG00000096653	Trav15d-1-dv6d-1	T cell receptor alpha variable 15D-1-DV6D-1 [Source:MGI	10.83740387	3.437947292	0.009237126	0.028075829	yes	up	0.158	1.708	0	0	0.45	0.34	0	1.91	1.24	1.01	2.63	1.75

	Symbol;Acc:MGI:3651288]																		
ENSMUSG00000094766 Trav7-4	T cell receptor alpha variable 7-4 [Source:MGI Symbol;Acc:MGI:3649611]	12.45244096	3.638356666	0.007072799	0.022281607	yes	up	0.132	1.51	0.35	0	0	0.31	0	1.55	2.82	0.61	0.3	2.27
ENSMUSG00000076471 Trbv14	T cell receptor beta, variable 14 [Source:MGI Symbol;Acc:MGI:98587]	3.131839392	1.64701023	0.017366573	0.048178769	yes	up	2.652	7.172	2.53	4.81	1.32	1.13	3.47	6.27	17.59	4.48	3.36	4.16
ENSMUSG00000076476 Trbv20	T cell receptor beta, variable 20 [Source:MGI Symbol;Acc:MGI:98589]	5.752074612	2.52408239	0.010748023	0.031911779	yes	up	1.102	5.314	1.74	2.86	0.91	0	0	3.98	4.33	3.46	3.92	10.88
ENSMUSG00000107486 Trbv4	T cell receptor beta, variable 10 [Source:MGI Symbol;Acc:MGI:98584]	3.658785559	1.871364862	0.012846171	0.037212714	yes	up	1.194	3.816	2.58	0.56	1.07	0.92	0.84	5.5	3.77	1.58	3.62	4.61
ENSMUSG00000104876 Trdc	T cell receptor delta, constant region [Source:MGI Symbol;Acc:MGI:98612]	2.495282196	1.319202982	8.67E-06	5.11E-05	yes	up	2.958	6.428	2.65	4.17	3.13	2.23	2.61	6.04	4.08	5.11	5.06	11.85
ENSMUSG00000076873 Trdv5	T cell receptor delta variable 5 [Source:MGI Symbol;Acc:MGI:3704133]	25.61673795	4.679014869	0.006486913	0.02067738	yes	up	0	1.488	0	0	0	0	0	4.35	0.61	0	0.33	2.15
ENSMUSG00000023992 Trem2	triggering receptor expressed on myeloid cells 2 [Source:MGI Symbol;Acc:MGI:1913150]	28.41156504	4.828406399	7.64E-49	7.24E-47	yes	up	7.47	196.86	3.96	11.99	10.59	3.36	7.45	163.25	207.24	153.34	228.07	232.4
ENSMUSG00000023993 Treml1	triggering receptor expressed on myeloid cells-like 1 [Source:MGI Symbol;Acc:MGI:1918576]	0.149770313	-2.739176411	8.60E-12	1.09E-10	yes	down	3.86	0.482	2.48	7.18	3.31	3.3	3.03	0.42	0.48	0.45	0.52	0.54
ENSMUSG00000073400 Trim10	tripartite motif-containing 10 [Source:MGI Symbol;Acc:MGI:1338757]	0.372316322	-1.42539923	0.004819996	0.015868567	yes	down	0.908	0.282	1.3	0.56	0.99	1.36	0.33	0.18	0.14	0.39	0.18	0.52
ENSMUSG00000050747 Trim15	tripartite motif-containing 15 [Source:MGI Symbol;Acc:MGI:1916347]	0.412615714	-1.277129331	0.016318107	0.045670226	yes	down	0.76	0.266	0.38	1.29	0.78	0.8	0.55	0.13	0.34	0.47	0.09	0.3
ENSMUSG00000032013 Trim29	tripartite motif-containing 29 [Source:MGI Symbol;Acc:MGI:1919419]	5.644962427	2.496963978	9.71E-31	4.66E-29	yes	up	3.038	11.756	2.89	4.69	3.04	1.32	3.25	9.1	15.76	12.59	10.35	10.98
ENSMUSG00000034317 Trim59	tripartite motif-containing 59 [Source:MGI Symbol;Acc:MGI:1914199]	2.075301224	1.053320755	1.24E-07	9.74E-07	yes	up	3.244	5.156	3.47	3.29	3.8	3.68	1.98	6.77	4.24	4.39	5.24	5.14
ENSMUSG00000021569 Trip13	thyroid hormone receptor interactor 13 [Source:MGI Symbol;Acc:MGI:1916966]	2.320662805	1.214536913	0.000123842	0.000589115	yes	up	0.908	1.358	0.84	1.48	0.75	0.75	0.72	1.58	1.09	1.39	1.19	1.54
ENSMUSG00000055963 Triqk	triple QxxK/R motif containing [Source:MGI Symbol;Acc:MGI:3650048]	0.385176909	-1.376406878	4.57E-09	4.34E-08	yes	down	6.106	2.176	4.62	10.29	4.8	5.57	5.25	2.92	1.72	2.06	2.56	1.62
ENSMUSG00000039620 Trmt9b	tRNA methyltransferase 9B [Source:MGI Symbol;Acc:MGI:2442328]	0.355594683	-1.491694341	0.000152959	0.000715859	yes	down	6.962	0.408	1.24	0.83	1.59	4.13	27.02	0.33	0.41	0.56	0.28	0.46
ENSMUSG00000025272 Tro	trophinin [Source:MGI Symbol;Acc:MGI:1928994]	0.274452002	-1.86537423	6.10E-08	4.98E-07	yes	down	1.432	0.438	1.92	1.14	1.28	1.24	1.58	0.44	0.35	1.02	0.07	0.31
ENSMUSG00000032783 Troap	trophinin associated protein [Source:MGI Symbol;Acc:MGI:1925983]	2.536944609	1.34309202	0.000540603	0.002245454	yes	up	0.684	1.516	0.33	0.28	0.98	0.33	1.5	1.15	1.29	1.64	0.78	2.72
ENSMUSG00000027748 Trpc4	transient receptor potential cation channel, subfamily C, member 4 [Source:MGI Symbol;Acc:MGI:109525]	59.62384093	5.897817411	6.35E-13	8.94E-12	yes	up	0.014	2.574	0	0	0.04	0	0.03	8.06	1.85	1.27	0.38	1.31
ENSMUSG00000009292 Trpm2	transient receptor potential cation channel, subfamily M, member 2 [Source:MGI Symbol;Acc:MGI:1351901]	5.170740514	2.370370907	1.02E-27	4.15E-26	yes	up	1.478	5.554	1.74	2.37	1.25	1.25	0.78	6.93	3.7	3.26	4.76	9.12
ENSMUSG00000018507 Trpv2	transient receptor potential cation channel, subfamily V, member 2 [Source:MGI Symbol;Acc:MGI:1341836]	2.997696411	1.583854283	6.38E-69	1.14E-66	yes	up	14.862	40.688	15.65	12.87	17.85	12.98	14.96	35.52	43.23	39.51	41.39	43.79
ENSMUSG00000027857 Tshb	thyroid stimulating hormone, beta subunit [Source:MGI	0.407180133	-1.296260924	0.011290369	0.033305549	yes	down	0.588	0.518	0.5	0.41	0.56	0.98	0.49	1.12	1.15	0.15	0.16	0.01

		Symbol;Acc:MGI:98848]																		
ENSMUSG00000031893	Tsnaxip1	translin-associated factor X (Tsnax) interacting protein 1 [Source:MGI Symbol;Acc:MGI:1919486]	0.492817746	-1.020873887	0.000387512	0.001663119	yes	down	2.968	1.934	2.49	2.53	1.53	2.92	5.37	1.87	3.42	2.61	0.51	1.26
ENSMUSG00000039691	Tspan10	tetraspanin 10 [Source:MGI Symbol;Acc:MGI:2384781]	69.52567169	6.119473872	1.68E-08	1.49E-07	yes	up	0.008	0.644	0.04	0	0	0	0	0.74	0.23	0.55	0.74	0.96
ENSMUSG00000029669	Tspan12	tetraspanin 12 [Source:MGI Symbol;Acc:MGI:1889818]	0.44613033	-1.164462862	3.63E-32	1.88E-30	yes	down	55.916	20.99	54.76	60.25	53.44	61.02	50.11	18.84	21.39	17.8	25.79	21.13
ENSMUSG00000001763	Tspan33	tetraspanin 33 [Source:MGI Symbol;Acc:MGI:1919012]	2.147118697	1.102401948	8.23E-06	4.88E-05	yes	up	1.402	2.544	0.93	1.57	1.35	1.87	1.29	2.51	1.72	2.89	2.27	3.33
ENSMUSG00000025511	Tspan4	tetraspanin 4 [Source:MGI Symbol;Acc:MGI:1928097]	2.119748702	1.083893242	1.41E-16	2.81E-15	yes	up	30.952	54.264	26.74	29.38	30.1	39.7	28.84	46.26	42.67	53.93	65.5	62.96
ENSMUSG00000058254	Tspan7	tetraspanin 7 [Source:MGI Symbol;Acc:MGI:1298407]	0.440169701	-1.183868254	1.25E-24	4.23E-23	yes	down	694.754	258.05	803.29	568.16	651.43	787.48	663.41	249.97	295.92	205.79	309.32	229.25
ENSMUSG00000069581	Tspear	thrombospondin type laminin G domain and EAR repeats [Source:MGI Symbol;Acc:MGI:2671932]	0.052941514	-4.239456724	0.013586864	0.038994409	yes	down	0.158	0	0	0.23	0.13	0.08	0.35	0	0	0	0	0
ENSMUSG00000103711	Tstd1	thiosulfate sulfurtransferase (rhodanese)-like domain containing 1 [Source:MGI Symbol;Acc:MGI:3648482]	0.430707865	-1.215218426	0.000478458	0.0020127	yes	down	8.802	3.174	7.74	9.78	8.04	11.62	6.83	1.49	2.99	4.8	3.75	2.84
ENSMUSG00000015599	Ttbk1	tau tubulin kinase 1 [Source:MGI Symbol;Acc:MGI:2147036]	2.673507237	1.418733582	0.001032814	0.004034057	yes	up	0.074	0.178	0.1	0.09	0.08	0.06	0.04	0.21	0.24	0.1	0.11	0.23
ENSMUSG00000039021	Ttc16	tetratricopeptide repeat domain 16 [Source:MGI Symbol;Acc:MGI:2443048]	0.427126647	-1.22726419	1.16E-05	6.69E-05	yes	down	7.146	1.326	21.65	3.49	2.32	4.26	4.01	1.28	1.32	2.23	0.7	1.1
ENSMUSG00000037101	Ttc29	tetratricopeptide repeat domain 29 [Source:MGI Symbol;Acc:MGI:1920551]	0.423033702	-1.241155491	1.77E-07	1.36E-06	yes	down	15.532	5.83	16.11	15.81	14.76	14.93	16.05	5.15	6.09	9.66	2.62	5.63
ENSMUSG00000046637	Ttc34	tetratricopeptide repeat domain 34 [Source:MGI Symbol;Acc:MGI:2445205]	0.490012691	-1.02910898	2.88E-05	0.000154662	yes	down	1.55	0.61	1.6	1.7	1.32	1.24	1.89	0.51	0.57	0.86	0.52	0.59
ENSMUSG00000046782	Ttc6	tetratricopeptide repeat domain 6 [Source:MGI Symbol;Acc:MGI:2684915]	0.490071145	-1.028936889	6.10E-05	0.000307407	yes	down	1.47	0.494	1.57	1.45	1.59	1.82	0.92	0.41	0.53	0.68	0.56	0.29
ENSMUSG00000038379	Ttk	Ttk protein kinase [Source:MGI Symbol;Acc:MGI:1194921]	4.363613243	2.125523238	5.77E-08	4.74E-07	yes	up	0.362	1.336	0.32	0.23	0.48	0.26	0.52	1.76	2.11	0.95	0.49	1.37
ENSMUSG00000038756	Ttll6	tubulin tyrosine ligase-like family, member 6 [Source:MGI Symbol;Acc:MGI:2683461]	0.483038221	-1.049790747	4.48E-05	0.000231235	yes	down	1.648	0.674	1.71	1.21	1.33	2	1.99	0.55	0.79	0.96	0.47	0.6
ENSMUSG00000034714	Ttyh2	tweety family member 2 [Source:MGI Symbol;Acc:MGI:2157091]	2.251633952	1.170972308	7.88E-26	2.87E-24	yes	up	4.594	8.634	5.03	4.17	4.36	4.75	4.66	7.52	9.24	7.62	10.01	8.78
ENSMUSG00000016255	Tubb1	tubulin, beta 1 class VI [Source:MGI Symbol;Acc:MGI:107814]	0.244757572	-2.030574603	1.61E-24	5.39E-23	yes	down	17.146	3.548	15.18	23.4	15.17	18.45	13.53	3.96	4.02	2.07	3.88	3.81
ENSMUSG00000062591	Tubb4a	tubulin, beta 4A class IVA [Source:MGI Symbol;Acc:MGI:107848]	0.373931634	-1.419153568	2.80E-21	7.66E-20	yes	down	13.912	4.388	15.83	11.99	10.76	18.19	12.79	3.74	4.86	4.2	4.21	4.93
ENSMUSG00000050908	Tvp23a	trans-golgi network vesicle protein 23A [Source:MGI Symbol;Acc:MGI:3665441]	2.373144523	1.246799962	0.000789916	0.003161434	yes	up	0.616	3.578	0.4	0.75	0.95	0.74	0.24	1.79	1.5	0.83	0.91	12.86
ENSMUSG00000030579	Tyrobp	TYRO protein tyrosine kinase binding protein [Source:MGI Symbol;Acc:MGI:1277211]	5.43392161	2.441993753	2.82E-214	1.21E-210	yes	up	152.726	705.066	142.31	176.46	157.36	140.83	146.67	680.27	724.7	666.33	725.03	729

ENSMUSG00000042345 Ubash3a	ubiquitin associated and SH3 domain containing, A [Source:MGI Symbol;Acc:MGI:1926074]	2.16639286	1.115294889	3.57E-06	2.26E-05	yes	up	5.434	2.536	21.93	1.59	1.27	1.69	0.69	3.04	1.4	2.51	3.05	2.68
ENSMUSG00000032020 Ubash3b	ubiquitin associated and SH3 domain containing, B [Source:MGI Symbol;Acc:MGI:1920078]	2.185145318	1.127729226	1.02E-14	1.72E-13	yes	up	3.33	5.824	3.61	2.33	4.57	2.57	3.57	6.65	5.72	4.96	4.73	7.06
ENSMUSG00000035186 Ubd	ubiquitin D [Source:MGI Symbol;Acc:MGI:1344410]	28.14495124	4.814804244	1.89E-09	1.86E-08	yes	up	0.388	9.042	0.82	0.38	0.66	0.08	0	11.86	3.72	6.34	4.61	18.68
ENSMUSG00000001403 Ube2c	ubiquitin-conjugating enzyme E2C [Source:MGI Symbol;Acc:MGI:1915862]	2.66175768	1.412379238	3.09E-10	3.31E-09	yes	up	7.624	17.208	8.34	4.3	8.27	7.62	9.59	19.1	14.61	14.9	13.53	23.9
ENSMUSG00000026429 Ube2t	ubiquitin-conjugating enzyme E2T [Source:MGI Symbol;Acc:MGI:1914446]	2.966251712	1.568641028	4.69E-06	2.89E-05	yes	up	1.204	3.024	0.86	1.17	1.39	0.56	2.04	1.66	3.19	3.38	3.53	3.36
ENSMUSG00000032942 Ucp3	uncoupling protein 3 (mitochondrial, proton carrier) [Source:MGI Symbol;Acc:MGI:1099787]	2.549477821	1.350201787	1.03E-08	9.35E-08	yes	up	2.446	5.496	2.27	1.78	3.17	1.54	3.47	4.93	7.1	6.12	4.6	4.73
ENSMUSG00000032854 Ugt8a	UDP galactosyltransferase 8A [Source:MGI Symbol;Acc:MGI:109522]	0.355122313	-1.493612085	0.00135682	0.005141128	yes	down	0.616	0.18	0.42	0.51	0.7	0.8	0.65	0.13	0.12	0.26	0.04	0.35
ENSMUSG00000001228 Uhrf1	ubiquitin-like, containing PHD and RING finger domains, 1 [Source:MGI Symbol;Acc:MGI:1338889]	2.77025937	1.470021057	1.55E-09	1.54E-08	yes	up	2.154	4.832	1.81	2.1	2.26	2.42	2.18	5.49	2.97	3.71	3.54	8.45
ENSMUSG00000079685 Ulbp1	UL16 binding protein 1 [Source:MGI Symbol;Acc:MGI:1925027]	2.116644229	1.081778798	1.38E-11	1.70E-10	yes	up	3.972	5.864	4.39	2.65	7.85	3.05	1.92	10.1	4.57	3.63	3.38	7.64
ENSMUSG00000002058 Unc119	unc-119 lipid binding chaperone [Source:MGI Symbol;Acc:MGI:1328357]	2.448539366	1.291921391	1.69E-42	1.29E-40	yes	up	18.854	37.408	18.31	21.82	20.02	17.25	16.87	33.61	39.07	37.69	40.79	35.88
ENSMUSG00000021198 Unc79	unc-79 homolog [Source:MGI Symbol;Acc:MGI:2684729]	0.396855798	-1.333313211	0.001287058	0.004905435	yes	down	0.726	0.306	0.64	1.1	0.62	0.55	0.72	0.05	0.3	0.41	0.14	0.63
ENSMUSG00000055567 Unc80	unc-80, NALCN activator [Source:MGI Symbol;Acc:MGI:2652882]	0.293813166	-1.767029049	0.010640988	0.031664282	yes	down	0.114	0.098	0.06	0.29	0.09	0.09	0.04	0.12	0.15	0	0.11	0.11
ENSMUSG00000036908 Unc93b1	unc-93 homolog B1, TLR signaling regulator [Source:MGI Symbol;Acc:MGI:1859307]	2.866133707	1.519105914	5.47E-70	1.00E-67	yes	up	52.828	124.034	51.51	55.43	57.49	47.13	52.58	109.15	126.71	121.04	110.47	152.8
ENSMUSG00000006313 Upk1a	uroplakin 1A [Source:MGI Symbol;Acc:MGI:98911]	0.170188045	-2.554798396	1.60E-06	1.06E-05	yes	down	1.89	0.274	1.35	1.02	1.92	3.34	1.82	0.22	0.61	0.08	0.09	0.37
ENSMUSG00000022435 Upk3a	uroplakin 3A [Source:MGI Symbol;Acc:MGI:98914]	0.187684475	-2.413618774	0.003033627	0.010527978	yes	down	0.87	0.138	0.73	1.51	0.6	0.7	0.81	0.06	0.28	0	0	0.35
ENSMUSG00000042985 Upk3b	uroplakin 3B [Source:MGI Symbol;Acc:MGI:2140882]	0.336388894	-1.571798018	1.25E-08	1.13E-07	yes	down	32.388	9.22	35.68	28.44	33.43	32.24	32.15	7.82	9.18	9.53	15.19	4.38
ENSMUSG00000035713 Usp35	ubiquitin specific peptidase 35 [Source:MGI Symbol;Acc:MGI:2685339]	2.152553012	1.106048768	2.19E-10	2.39E-09	yes	up	2.33	3.82	2.08	1.83	2.87	2.76	2.11	3.12	4.51	4.71	2.76	4
ENSMUSG00000037568 Vash2	vasohibin 2 [Source:MGI Symbol;Acc:MGI:2444826]	5.104162046	2.351674132	1.35E-05	7.67E-05	yes	up	0.12	0.52	0	0.27	0.07	0.07	0.19	0.6	0.54	0.59	0.54	0.33
ENSMUSG00000034116 Vav1	vav 1 oncogene [Source:MGI Symbol;Acc:MGI:98923]	3.322796561	1.732397967	3.94E-67	6.49E-65	yes	up	10.7	30.272	9.54	10.32	11.55	13.29	8.8	32.61	27.58	28.82	28.74	33.61
ENSMUSG00000091243 Vgll3	vestigial like family member 3 [Source:MGI Symbol;Acc:MGI:1920819]	0.448739715	-1.156049221	9.47E-26	3.41E-24	yes	down	15.034	5.706	15.19	14.87	15.99	15.84	13.28	4.6	8.25	5.12	5.37	5.19
ENSMUSG00000026728 Vim	vimentin [Source:MGI Symbol;Acc:MGI:98932]	2.048500553	1.034568282	1.46E-24	4.91E-23	yes	up	872.504	1587.944	871.49	869.27	914.9	778.28	928.58	1155.74	1795.94	1550.05	1598.76	1839.23
ENSMUSG00000011171 Vipr2	vasoactive intestinal peptide receptor 2 [Source:MGI Symbol;Acc:MGI:107166]	0.337160578	-1.568492235	2.14E-16	4.20E-15	yes	down	14.55	4.152	19.33	8.78	16.33	15.89	12.42	3.62	4.29	3.12	6.07	3.66

ENSMUSG00000037440	Vnn1	vanin 1 [Source:MGI Symbol;Acc:MGI:108395]	2.618846927	1.388931736	4.57E-22	1.31E-20	yes	up	10.034	22.136	13.47	7.2	10.44	11.64	7.42	22.81	19.73	23.58	19.85	24.71
ENSMUSG00000020010	Vnn3	vanin 3 [Source:MGI Symbol;Acc:MGI:1347055]	3.367521756	1.751687266	3.08E-28	1.30E-26	yes	up	6.162	17.482	5.57	5.21	8.95	5.78	5.3	14.87	16.16	19.81	16.41	20.16
ENSMUSG00000049598	Vsig8	V-set and immunoglobulin domain containing 8 [Source:MGI Symbol;Acc:MGI:3642995]	12.15600347	3.603597086	0.000166094	0.000769699	yes	up	0.048	0.446	0	0	0	0	0.24	0.26	0.64	0.48	0.39	0.46
ENSMUSG00000054459	Vsnl1	visinin-like 1 [Source:MGI Symbol;Acc:MGI:1349453]	0.478277861	-1.064079082	5.97E-09	5.58E-08	yes	down	13.32	5.376	14.45	14.79	11.66	16.38	9.32	3.68	6.2	4.32	6.95	5.73
ENSMUSG00000031165	Was	Wiskott-Aldrich syndrome [Source:MGI Symbol;Acc:MGI:105059]	2.328996933	1.21970874	8.02E-25	2.74E-23	yes	up	10.35	20.398	10.3	12.97	10.01	9.38	9.09	21.41	19.18	19.9	18.42	23.08
ENSMUSG00000051506	Wdfy4	WD repeat and FYVE domain containing 4 [Source:MGI Symbol;Acc:MGI:3584510]	2.483480757	1.312363568	1.22E-42	9.34E-41	yes	up	7.738	17.632	7.13	7.77	9.08	6.89	7.82	15.89	20.92	19.75	14.64	16.96
ENSMUSG00000104301	Wdr49	WD repeat domain 49 [Source:MGI Symbol;Acc:MGI:3645287]	0.31423448	-1.670086603	0.014123969	0.040298284	yes	down	6.054	0.566	17.68	1.85	6.2	2.54	2	0.25	0.94	0.29	0.75	0.6
ENSMUSG00000055235	Wdr86	WD repeat domain 86 [Source:MGI Symbol;Acc:MGI:1915466]	2.427161535	1.279270127	0.000447629	0.001894549	yes	up	0.56	0.954	0.32	0.51	0.83	0.92	0.22	0.77	0.79	1.19	1.05	0.97
ENSMUSG00000042845	Wfdc12	WAP four-disulfide core domain 12 [Source:MGI Symbol;Acc:MGI:2183434]	24.59660214	4.620387125	6.13E-08	5.01E-07	yes	up	0.108	2.362	0.12	0	0	0.11	0.31	1.02	1.32	2.42	2.85	4.2
ENSMUSG00000070530	Wfdc16	WAP four-disulfide core domain 16 [Source:MGI Symbol;Acc:MGI:2670994]	0.069470624	-3.847453132	0.013847938	0.039610986	yes	down	0.272	0.012	0.36	0.22	0.22	0.56	0	0.06	0	0	0	0
ENSMUSG00000069792	Wfdc17	WAP four-disulfide core domain 17 [Source:MGI Symbol;Acc:MGI:3649773]	21.41302117	4.420416455	4.36E-57	5.34E-55	yes	up	22.126	402.422	12.9	32.15	29.83	15.48	20.27	439.21	408.6	263.24	466.31	434.75
ENSMUSG00000000983	Wfdc18	WAP four-disulfide core domain 18 [Source:MGI Symbol;Acc:MGI:107506]	21.72384692	4.441207697	0.002499141	0.00888473	yes	up	0	0.826	0	0	0	0	0	1.08	1.19	0.39	1.07	0.4
ENSMUSG00000051748	Wfdc21	WAP four-disulfide core domain 21 [Source:MGI Symbol;Acc:MGI:1913357]	7.501512676	2.907181544	2.81E-24	9.31E-23	yes	up	15.942	101.72	9.37	29.13	16.59	6.54	18.08	72.41	115.87	113.22	111.76	95.34
ENSMUSG00000074595	Wfdc6a	WAP four-disulfide core domain 6A [Source:MGI Symbol;Acc:MGI:2684968]	0.385655085	-1.37461696	0.004342615	0.014452439	yes	down	2.59	0.828	4.46	2.28	2.45	1.22	2.54	0.64	0.57	1.29	0.82	0.82
ENSMUSG00000070531	Wfdc6b	WAP four-disulfide core domain 6B [Source:MGI Symbol;Acc:MGI:3575430]	0.220176447	-2.18326795	7.45E-06	4.43E-05	yes	down	2.298	0.44	2.72	1.72	1.82	2.33	2.9	0.52	0.83	0.47	0.25	0.13
ENSMUSG00000037989	Wnk2	WNK lysine deficient protein kinase 2 [Source:MGI Symbol;Acc:MGI:1922857]	0.351464787	-1.50854794	0.013292226	0.038328574	yes	down	0.342	0.088	0.25	0.67	0.1	0.42	0.27	0.14	0.06	0.16	0.04	0.04
ENSMUSG00000010797	Wnt2	wingless-type MMTV integration site family, member 2 [Source:MGI Symbol;Acc:MGI:98954]	0.307925481	-1.69934684	1.71E-09	1.70E-08	yes	down	8.608	2.224	7.55	8.72	7.58	14.09	5.1	1.7	1.74	1.31	3.9	2.47
ENSMUSG00000027840	Wnt2b	wingless-type MMTV integration site family, member 2B [Source:MGI Symbol;Acc:MGI:1261834]	0.314308371	-1.6697474	6.98E-16	1.29E-14	yes	down	2.986	2.156	2.43	2.62	3.58	2.87	3.43	7.85	0.72	0.88	0.57	0.76
ENSMUSG00000016458	Wt1	Wilms tumor 1 homolog [Source:MGI Symbol;Acc:MGI:98968]	0.302676478	-1.724151533	1.67E-06	1.10E-05	yes	down	2.48	0.596	2.03	2.73	3.45	1.81	2.38	0.63	0.23	0.83	0.93	0.36

ENSMUSG00000026573 Xcl1	chemokine (C motif) ligand 1 [Source:MGI Symbol;Acc:MGI:104593]	8.070122651	3.0125906	6.74E-07	4.74E-06	yes	up	0.966	6.588	0.75	1.83	0.51	0.65	1.09	11.38	4.93	4.81	1.7	10.12
ENSMUSG00000060509 Xcr1	chemokine (C motif) receptor 1 [Source:MGI Symbol;Acc:MGI:1346338]	4.427264617	2.146415606	1.43E-23	4.51E-22	yes	up	1.192	4.456	0.86	1.45	1.82	0.66	1.17	4.31	4.13	4.45	4.58	4.81
ENSMUSG00000031362 Xlr4c	X-linked lymphocyte-regulated 4C [Source:MGI Symbol;Acc:MGI:3574099]	2.325554882	1.217574987	0.005610277	0.018171461	yes	up	1.664	3.238	1.44	2.38	0.76	3.37	0.37	3.68	1.77	3.26	2.25	5.23
ENSMUSG00000037005 Xpnpep2	X-prolyl aminopeptidase (aminopeptidase P) 2, membrane-bound [Source:MGI Symbol;Acc:MGI:2180001]	0.260637179	-1.939885201	3.56E-22	1.03E-20	yes	down	7.104	1.22	6.25	10.06	4.01	10.98	4.22	2.05	0.93	1.17	1.1	0.85
ENSMUSG00000030657 Xylt1	xylosyltransferase 1 [Source:MGI Symbol;Acc:MGI:2451073]	3.140400893	1.65094874	1.73E-56	2.07E-54	yes	up	1.526	4.034	1.49	1.7	1.49	1.6	1.35	4.41	4.18	4.08	4	3.5
ENSMUSG00000041774 Ydjc	YdjC homolog (bacterial) [Source:MGI Symbol;Acc:MGI:1916351]	2.563963033	1.358375461	0.000338447	0.001470817	yes	up	0.932	2.192	0.7	0.83	1.25	0.41	1.47	1.45	3.13	2.21	1.77	2.4
ENSMUSG00000027514 Zbp1	Z-DNA binding protein 1 [Source:MGI Symbol;Acc:MGI:1927449]	2.815819459	1.493554836	7.97E-16	1.47E-14	yes	up	9.864	25.308	10.11	9.85	11.09	8.34	9.93	28.16	23.12	20.47	21.83	32.96
ENSMUSG00000066687 Zbtb16	zinc finger and BTB domain containing 16 [Source:MGI Symbol;Acc:MGI:103222]	0.381602335	-1.389858095	0.000406586	0.001736973	yes	down	51.424	16.33	74.38	58.68	51.42	13.86	58.78	20.02	16.65	10.78	17.51	16.69
ENSMUSG00000039981 Zc3h12d	zinc finger CCCH type containing 12D [Source:MGI Symbol;Acc:MGI:3045313]	2.047371573	1.033772958	1.33E-12	1.83E-11	yes	up	1.97	3.388	2.08	1.94	2.01	2.33	1.49	3.53	3.41	3.86	2.83	3.31
ENSMUSG00000027520 Zdbf2	zinc finger, DBF-type containing 2 [Source:MGI Symbol;Acc:MGI:1921134]	0.316868943	-1.658041831	0.000369836	0.001594927	yes	down	0.12	0.032	0.14	0.15	0.05	0.16	0.1	0.03	0.02	0.04	0.04	0.03
ENSMUSG00000044786 Zfp36	zinc finger protein 36 [Source:MGI Symbol;Acc:MGI:99180]	0.424158889	-1.237323298	8.09E-06	4.80E-05	yes	down	134.278	48.688	120.46	197.27	99.42	144.56	109.68	45.27	44.34	78.29	33.92	41.62
ENSMUSG00000000552 Zfp385a	zinc finger protein 385A [Source:MGI Symbol;Acc:MGI:1352495]	2.807745377	1.48941211	1.16E-28	5.03E-27	yes	up	5.338	12.328	4.08	5.78	6.8	4.78	5.25	10.9	14.39	12.14	11.43	12.78
ENSMUSG00000062518 Zfp534	zinc finger protein 534 [Source:MGI Symbol;Acc:MGI:3650650]	0.060442657	-4.0482891	0.011111339	0.032849315	yes	down	0.096	0.004	0.13	0.02	0.25	0.08	0	0.02	0	0	0	0
ENSMUSG00000062794 Zfp599	zinc finger protein 599 [Source:MGI Symbol;Acc:MGI:2679006]	2.574483759	1.364283169	0.00957542	0.028935684	yes	up	0.172	0.368	0.13	0.33	0.06	0.17	0.17	0.7	0.31	0.23	0.19	0.41
ENSMUSG00000022987 Zfp641	zinc finger protein 641 [Source:MGI Symbol;Acc:MGI:2442788]	0.446096507	-1.164572243	1.46E-15	2.64E-14	yes	down	6.9	2.536	7.97	6.7	6.02	7.93	5.88	2.61	3.18	2.16	2.61	2.12
ENSMUSG00000044081 Zfp85os	zinc finger protein 85, opposite strand [Source:MGI Symbol;Acc:MGI:1915521]	0.322524512	-1.632519284	0.005082317	0.0166401	yes	down	0.46	0.126	0.67	0.3	0.31	0.37	0.65	0.04	0.08	0.06	0.29	0.16
ENSMUSG00000067916 Zfp991	zinc finger protein 991 [Source:MGI Symbol;Acc:MGI:3701604]	2.830189084	1.500898442	1.09E-20	2.91E-19	yes	up	4.266	10.308	5	3.37	5.38	2.87	4.71	9.44	8.61	11.4	10.03	12.06
ENSMUSG00000070605 Zfp992	zinc finger protein 992 [Source:MGI Symbol;Acc:MGI:3700963]	3.110778204	1.637275535	1.04E-16	2.11E-15	yes	up	3.214	5.89	1.13	7.35	1.32	4.13	2.14	3.47	8.19	9.1	4.99	3.7
ENSMUSG00000063245 Zfp993	zinc finger protein 993 [Source:MGI Symbol;Acc:MGI:3713585]	2.867771532	1.519930093	4.16E-08	3.48E-07	yes	up	1.814	3.904	2	2.14	2.54	1.43	0.96	2.57	5.83	4.34	2.45	4.33
ENSMUSG00000035877 Zhx3	zinc fingers and homeoboxes 3 [Source:MGI Symbol;Acc:MGI:2444772]	0.495271881	-1.013707382	2.55E-34	1.45E-32	yes	down	15.702	5.212	19.73	18.13	14.49	13.75	12.41	6.09	5.42	5.6	4.28	4.67
ENSMUSG00000002266 Zim1	zinc finger, imprinted 1 [Source:MGI Symbol;Acc:MGI:1341879]	0.148237537	-2.754017283	0.00109478	0.004248622	yes	down	0.122	0.036	0.07	0.04	0.12	0.07	0.31	0.07	0.02	0.09	0	0
ENSMUSG00000037492 Zmat4	zinc finger, matrin type 4 [Source:MGI Symbol;Acc:MGI:2443497]	0.454540195	-1.137520217	0.000779584	0.003122418	yes	down	0.878	0.336	0.8	0.95	0.5	1.16	0.98	0.15	0.45	0.36	0.48	0.24
ENSMUSG00000070806 Zmynd12	zinc finger, MYND domain containing 12 [Source:MGI Symbol;Acc:MGI:2140259]	0.472871718	-1.080479238	0.000202474	0.000922714	yes	down	2.554	0.99	2.26	2.4	2.77	2.65	2.69	1	0.58	1.23	0.88	1.26

ENSMUSG00000040829	Zmynd15	zinc finger, MYND-type containing 15 [Source:MGI Symbol;Acc:MGI:3603821]	3.128964882	1.645685466	1.64E-34	9.35E-33	yes	up	3.952	10.898	3.37	4.09	4.08	3.48	4.74	10.94	12.29	11.18	7.4	12.68
ENSMUSG00000084350	Znf41-ps	ZNF41, pseudogene [Source:MGI Symbol;Acc:MGI:1917255]	2.74722782	1.457976554	1.88E-18	4.31E-17	yes	up	2.996	6.048	2.46	4.16	3.55	1.77	3.04	6.65	5.57	6.42	6.23	5.37
ENSMUSG00000036086	Zranb3	zinc finger, RAN-binding domain containing 3 [Source:MGI Symbol;Acc:MGI:1918362]	9.200726967	3.201747856	6.93E-151	7.42E-148	yes	up	1.574	15.954	1.21	1.49	1.72	2.38	1.07	16.19	16.15	17.83	14.76	14.84

Table S4-2. 811 differentially expressed genes between Mel and LPS group.

Gene_id	Gene name	Gene description	FC(mel/lps)	Log2FC(mel/lps)	Pvalue	Padjust	Significant	Regulate	mel_3	mel_5	mel_6	mel_8	lps_1	lps_3	lps_4	lps_5	lps_6	mel	lps
ENSMUSG00000099764	Rps10-ps2	ribosomal protein S10, pseudogene 2 [Source:MGI Symbol;Acc:MGI:3645627]	55.55555556	5.795859283	1.76617E-05	0.000228136	yes	up	0	2.15	2.82	4.98	0.03	0.04	0	0	0	2.602	0.014
ENSMUSG00000115729	Gm48957	predicted gene, 48957 [Source:MGI Symbol;Acc:MGI:6118287]	30.3030303	4.921390165	0.001028549	0.006895155	yes	up	0.14	0.21	0	0.42	0	0	0	0	0	0.182	0

ENSMUSG00000038155	Gstp2	glutathione S-transferase, pi 2 [Source:MGI Symbol;Acc:MGI:95864]	27.02702703	4.756330919	0.002303289	0.013341297	yes	up	0.43	0.23	0	2.42	0	0	0.24	0	0	0.834	0.048
ENSMUSG00000085055	Gm15958	predicted gene 15958 [Source:MGI Symbol;Acc:MGI:3801839]	25	4.64385619	0.00150044	0.009371894	yes	up	0.48	0.83	4.31	0.08	0	0.04	0	0	0	1.14	0.008
ENSMUSG00000108932	Gm31463	predicted gene, 31463 [Source:MGI Symbol;Acc:MGI:5590622]	16.66666667	4.058893689	0.000894837	0.006153364	yes	up	0.12	0.42	0.12	0.38	0	0.04	0	0	0.04	0.288	0.016
ENSMUSG00000020081	Tacr2	tachykinin receptor 2 [Source:MGI Symbol;Acc:MGI:98477]	16.12903226	4.011587974	0.0007115	0.005110299	yes	up	0.16	0.21	0.1	0.25	0.02	0	0.02	0	0	0.168	0.008
ENSMUSG00000095497	Igkv1-122	immunoglobulin kappa chain variable 1-122 [Source:MGI Symbol;Acc:MGI:4439722]	15.625	3.965784285	1.56129E-11	9.52285E-10	yes	up	149.88	124.44	437.86	54.38	9.49	8.63	22.77	16.68	13.68	242.186	14.25
ENSMUSG00000030730	Atp2a1	ATPase, Ca++ transporting, cardiac muscle, fast twitch 1 [Source:MGI Symbol;Acc:MGI:105058]	13.69863014	3.775959726	2.69972E-07	6.03763E-06	yes	up	0.03	1.85	1.45	0.93	0.4	0.04	0.11	0.02	0.06	0.964	0.126
ENSMUSG00000053182	Gm609	predicted gene 609 [Source:MGI Symbol;Acc:MGI:2685455]	13.33333333	3.736965594	0.002755576	0.015376961	yes	up	1.86	0.62	0.4	0.35	0	0	0.03	0.2	0	0.66	0.046
ENSMUSG00000032268	Tmprss5	transmembrane protease, serine 5 (spinesin) [Source:MGI Symbol;Acc:MGI:1933407]	12.98701299	3.698997744	0.001453328	0.009137037	yes	up	0.07	0.3	0.24	3.16	0.04	0.11	0	0	0	0.802	0.03
ENSMUSG00000049235	Gm7324	predicted gene 7324 [Source:MGI Symbol;Acc:MGI:3646999]	9.900990099	3.307572802	0.006987568	0.032490838	yes	up	0.13	0.3	0.65	0.69	0.14	0	0	0	0.26	0.64	0.08
ENSMUSG00000079597	Gm5483	predicted gene 5483 [Source:MGI Symbol;Acc:MGI:3645124]	9.345794393	3.224317298	0.00085668	0.005927038	yes	up	8.88	3.54	0.79	6.38	0.42	0	0	1.16	0.36	4.214	0.388
ENSMUSG00000061742	Slc22a12	solute carrier family 22 (organic anion/cation transporter), member 12 [Source:MGI Symbol;Acc:MGI:1195269]	9.259259259	3.210896782	0.002488327	0.014184314	yes	up	0.68	0.83	0.43	0.15	0.05	0.11	0	0.14	0	0.428	0.06
ENSMUSG00000015829	Tnr	tenascin R [Source:MGI Symbol;Acc:MGI:99516]	8.771929825	3.13289427	5.14032E-05	0.000565965	yes	up	0.02	0.07	0.08	0.28	0	0.01	0.02	0.01	0	0.098	0.008
ENSMUSG00000113346	Eprn	ephemeron, early developmental lncRNA [Source:MGI Symbol;Acc:MGI:3583897]	8.403361345	3.070966521	0.004704357	0.023785478	yes	up	0.18	0.32	0.51	0.15	0.1	0.09	0	0	0	0.334	0.038
ENSMUSG00000089837	Npcd	neuronal pentraxin chromo domain [Source:MGI Symbol;Acc:MGI:3845555]	8.26446281	3.046921047	0.003852659	0.020163755	yes	up	0.25	0.81	1.07	0.43	0	0	0	0.39	0	0.654	0.078
ENSMUSG00000020096	Tbata	thymus, brain and testes associated [Source:MGI Symbol;Acc:MGI:1923820]	7.874015748	2.977099598	0.000274471	0.002318999	yes	up	0.89	0.82	1.39	1.1	0	0.39	0.08	0	0.08	0.874	0.11
ENSMUSG00000110588	Gm45774	predicted gene 45774 [Source:MGI Symbol;Acc:MGI:5804889]	7.692307692	2.943416472	0.00284225	0.015750835	yes	up	2.14	0.15	0.06	0.04	0.04	0.13	0.11	0.04	0	0.538	0.064
ENSMUSG00000045238	A730035I17Rik	RIKEN cDNA A730035I17 gene [Source:MGI Symbol;Acc:MGI:3696858]	7.462686567	2.899695094	0.008937645	0.039348985	yes	up	0.53	0	0.66	0.31	0.07	0.07	0.03	0	0	0.336	0.034
ENSMUSG00000073386	9830107B12Rik	RIKEN cDNA 9830107B12 gene [Source:MGI Symbol;Acc:MGI:3608415]	6.329113924	2.662003536	0.00346854	0.018566005	yes	up	0.29	0.39	0.08	0.13	0.12	0	0	0.06	0.03	0.206	0.042
ENSMUSG00000098773	Gm27179	predicted gene 27179 [Source:MGI Symbol;Acc:MGI:5521022]	6.211180124	2.634867407	0.005576851	0.027212906	yes	up	0.12	0.25	0.14	0.24	0.02	0.05	0	0	0.05	0.156	0.024
ENSMUSG00000102060	1700061E17Rik	RIKEN cDNA 1700061E17 gene [Source:MGI Symbol;Acc:MGI:3642196]	5.988023952	2.582079992	0.005220748	0.0258788	yes	up	0	4.35	8.35	4.05	0.75	2.02	0.24	0	1.44	5.906	0.89
ENSMUSG00000117592	AC162182.1	novel transcript, antisense to Pi16	5.882352941	2.556393349	0.00121556	0.007911435	yes	up	0.39	0.34	0.19	0.75	0.23	0.02	0.29	0	0.04	0.38	0.116
ENSMUSG00000030359	Pzp	PZP, alpha-2-macroglobulin like [Source:MGI Symbol;Acc:MGI:87854]	5.649717514	2.498178735	0.000397904	0.003166881	yes	up	0.11	0.41	0.15	1.01	0.03	0	0.04	0.06	0.01	0.648	0.028
ENSMUSG00000052188	Gm14964	predicted gene 14964 [Source:MGI Symbol;Acc:MGI:3641621]	5.586592179	2.481968507	9.25414E-09	3.03413E-07	yes	up	2.58	5.2	2.93	6.15	1.75	0.56	0.38	1.49	0.63	4.604	0.962
ENSMUSG00000028940	Hes2	hes family bHLH transcription factor 2 [Source:MGI Symbol;Acc:MGI:1098624]	5.586592179	2.481968507	1.18013E-06	2.17183E-05	yes	up	2.82	1.16	1.24	2.68	0.46	0.12	0.12	0.23	0	1.888	0.186
ENSMUSG00000035448	Ccr3	chemokine (C-C motif) receptor 3 [Source:MGI Symbol;Acc:MGI:104616]	5.524861878	2.465938398	8.0365E-17	1.36642E-14	yes	up	1.3	1.38	1.85	1.52	0.13	0.29	0.2	0.48	0.2	1.522	0.26
ENSMUSG00000027350	Chgb	chromogranin B [Source:MGI Symbol;Acc:MGI:88395]	5.208333333	2.380821784	0.000454236	0.003539024	yes	up	0.3	0.37	0.35	0.47	0	0.06	0.17	0	0.09	0.356	0.064
ENSMUSG00000022651	Retnlg	resistin like gamma [Source:MGI Symbol;Acc:MGI:2667763]	4.716981132	2.23786383	1.28041E-19	3.41673E-17	yes	up	111.03	72.53	82.77	73.19	27.32	11.53	8.92	21.87	13.9	83.804	16.708
ENSMUSG00000055547	Apobec4	apolipoprotein B mRNA editing enzyme, catalytic polypeptide-like 4 (putative) [Source:MGI Symbol;Acc:MGI:1918531]	4.694835681	2.231074664	0.000880323	0.006066587	yes	up	0.54	0.9	0.3	0.62	0.04	0.04	0.14	0.23	0.11	0.546	0.112

ENSMUSG00000038418	Egr1	early growth response 1 [Source:MGI Symbol;Acc:MGI:95295]	4.694835681	2.231074664	0.002326114	0.013446493	yes	up	122.77	11.36	11.93	10.25	6.81	6.5	11.99	5.95	6.05	37.318	7.46
ENSMUSG00000117921	AC113491.1	novel transcript	4.651162791	2.217591435	0.00771498	0.035158424	yes	up	0.71	0.64	0.92	0.59	0	0.38	0.13	0	0.1	0.592	0.122
ENSMUSG00000020848	Doc2b	double C2, beta [Source:MGI Symbol;Acc:MGI:1100497]	4.385964912	2.13289427	1.32888E-05	0.000179801	yes	up	0.85	12.56	0.62	0.43	0.17	0.12	0.26	0.24	0.1	2.952	0.178
ENSMUSG00000075330	A930003A15Rik	RIKEN cDNA A930003A15 gene [Source:MGI Symbol;Acc:MGI:1915412]	4.310344828	2.10780329	7.7926E-05	0.000805255	yes	up	0.92	3.3	3.55	1.23	1.01	0.36	0.42	0.37	0.43	2.144	0.518
ENSMUSG00000089989	Gm45713	predicted gene 45713 [Source:MGI Symbol;Acc:MGI:5804828]	4.166666667	2.058893689	0.008271297	0.037043459	yes	up	5.95	3.56	3.25	6.02	0	2.36	1.13	0.95	0.84	4.444	1.056
ENSMUSG00000116579	Gm49701	predicted gene, 49701 [Source:MGI Symbol;Acc:MGI:6215160]	4.149377593	2.052894948	0.002187613	0.012779146	yes	up	1.67	1.07	0.69	2.9	0.73	0.58	0.47	0.15	0.12	1.692	0.41
ENSMUSG00000018924	Alox15	arachidonate 15-lipoxygenase [Source:MGI Symbol;Acc:MGI:87997]	4.098360656	2.035046947	9.09624E-14	8.7383E-12	yes	up	2.14	1.99	1.61	2.23	0.18	1.38	0.56	0.49	0.59	2.038	0.64
ENSMUSG00000054966	Lmntd1	lamin tail domain containing 1 [Source:MGI Symbol;Acc:MGI:1921321]	4.016064257	2.005782353	5.26669E-09	1.81668E-07	yes	up	1.66	1.37	2.75	1.61	0.42	0.58	0.34	1.1	0.43	2.156	0.574
ENSMUSG00000112388	AI463170	expressed sequence AI463170 [Source:MGI Symbol;Acc:MGI:2144839]	3.984063745	1.994240731	0.007514502	0.03441624	yes	up	2.18	2.38	1.78	2.82	1.7	0.07	0.07	0.35	0.67	2.5	0.572
ENSMUSG00000055370	Gm9968	predicted gene 9968 [Source:MGI Symbol;Acc:MGI:3642146]	3.95256917	1.98279071	0.000367264	0.002962318	yes	up	0.43	0.14	0.39	0.42	0.15	0.03	0.05	0.11	0.07	0.346	0.082
ENSMUSG00000028003	Lrat	lecithin-retinol acyltransferase (phosphatidylcholine-retinol-O-acyltransferase) [Source:MGI Symbol;Acc:MGI:1891259]	3.90625	1.965784285	3.53754E-12	2.48962E-10	yes	up	12.39	11.62	8.85	13.78	1.99	3.61	1.9	3.57	1.87	10.67	2.588
ENSMUSG00000114858	Gm5790	predicted gene 5790 [Source:MGI Symbol;Acc:MGI:3779518]	3.90625	1.965784285	0.000440694	0.003446095	yes	up	1.71	1.9	1.09	1.77	0.15	0.12	0.89	0.08	0.63	1.532	0.374
ENSMUSG00000114277	Gm48583	predicted gene, 48583 [Source:MGI Symbol;Acc:MGI:6098152]	3.875968992	1.954557029	0.007058378	0.032740854	yes	up	129.17	60.59	60.79	113.4	41.79	0	0	26.97	39.46	90.13	21.644
ENSMUSG00000070531	Wfdc6b	WAP four-disulfide core domain 6B [Source:MGI Symbol;Acc:MGI:3575430]	3.861003861	1.948975997	4.53829E-05	0.000507828	yes	up	1.57	1.24	2.09	1.84	0.52	0.83	0.47	0.25	0.13	1.768	0.44
ENSMUSG00000111163	Gm46136	predicted gene, 46136 [Source:MGI Symbol;Acc:MGI:5825773]	3.861003861	1.948975997	0.001321267	0.008475962	yes	up	0.08	0.14	0.11	0.88	0.03	0.03	0.02	0.01	0.04	0.256	0.026
ENSMUSG00000021198	Unc79	unc-79 homolog [Source:MGI Symbol;Acc:MGI:2684729]	3.6900369	1.883635243	7.29355E-06	0.000108042	yes	up	0.66	1.37	0.79	2.03	0.05	0.3	0.41	0.14	0.63	1.228	0.306
ENSMUSG00000029248	Thegl	theg spermatid protein like [Source:MGI Symbol;Acc:MGI:1919118]	3.558718861	1.831357964	0.001801386	0.0108734	yes	up	0.95	0.78	1.14	0.7	0.36	0.17	0.22	0.15	0.1	0.742	0.2
ENSMUSG00000046337	Fam178b	family with sequence similarity 178, member B [Source:MGI Symbol;Acc:MGI:3026913]	3.521126761	1.816037165	2.15992E-13	1.8863E-11	yes	up	6.05	6.43	5.8	9.74	1.53	47.9	1.91	1.68	2.15	7.2	11.034
ENSMUSG00000035932	Olfr750	olfactory receptor 750 [Source:MGI Symbol;Acc:MGI:3030584]	3.521126761	1.816037165	0.000499692	0.003812782	yes	up	0.98	3.09	5.61	1.98	0.29	1.75	0.77	1.67	0.57	3.464	1.01
ENSMUSG00000023236	Scg5	secretogranin V [Source:MGI Symbol;Acc:MGI:98289]	3.521126761	1.816037165	0.009903438	0.042691218	yes	up	0.4	0.6	0.78	1.68	0.21	0.1	0.25	0.38	0.05	0.744	0.198
ENSMUSG00000020524	Gria1	glutamate receptor, ionotropic, AMPA1 (alpha 1) [Source:MGI Symbol;Acc:MGI:95808]	3.460207612	1.790858602	1.36135E-09	5.40405E-08	yes	up	0.92	1.92	1.14	1.37	0.22	0.41	0.54	0.32	0.25	1.26	0.348
ENSMUSG00000027713	1810062G17Rik	RIKEN cDNA 1810062G17 gene [Source:MGI Symbol;Acc:MGI:1919532]	3.460207612	1.790858602	0.00480189	0.024202183	yes	up	1.45	0.71	1.2	1.56	0.08	0.14	0.87	0.16	0.36	1.194	0.322
ENSMUSG00000097722	Gm26841	predicted gene, 26841 [Source:MGI Symbol;Acc:MGI:5477335]	3.460207612	1.790858602	0.005878811	0.028393562	yes	up	0.37	0.53	0.41	0.15	0.05	0.21	0.05	0.06	0.21	0.456	0.116
ENSMUSG00000062345	Serpinb2	serine (or cysteine) peptidase inhibitor, clade B, member 2 [Source:MGI Symbol;Acc:MGI:97609]	3.460207612	1.790858602	0.009913849	0.042716928	yes	up	0.11	7.44	1.12	0.73	0.19	0.05	0.03	0.27	0.11	1.936	0.13
ENSMUSG00000025064	Col17a1	collagen, type XVII, alpha 1 [Source:MGI Symbol;Acc:MGI:88450]	3.436426117	1.780908942	3.23875E-08	9.27364E-07	yes	up	0.75	0.61	1.34	2.44	0.1	1.08	0.29	0.18	0.32	1.172	0.394
ENSMUSG00000113900	Gm40437	predicted gene, 40437 [Source:MGI Symbol;Acc:MGI:5623322]	3.424657534	1.775959726	0.002607555	0.014730088	yes	up	1.65	4.61	4.68	3.64	0.18	1.82	0.32	1.42	1	3.424	0.948
ENSMUSG00000027376	Prom2	prominin 2 [Source:MGI Symbol;Acc:MGI:2138997]	3.412969283	1.77102743	0.002153037	0.012630932	yes	up	0.13	0.3	0.2	0.11	0.02	0.1	0.02	0.1	0.05	0.192	0.058
ENSMUSG00000030495	Slc7a10	solute carrier family 7 (cationic amino acid transporter, y+ system), member 10 [Source:MGI Symbol;Acc:MGI:1858261]	3.401360544	1.76611194	1.85355E-13	1.64871E-11	yes	up	4.58	8.5	6.66	8.11	4.4	2.88	4.27	1.8	0.97	7.404	2.864

ENSMUSG00000050505	Pcdh20	protocadherin 20 [Source:MGI Symbol;Acc:MGI:2443376]	3.367003367	1.751465164	0.002998907	0.016466988	yes	up	0.35	0.36	0.26	0.06	0.11	0.03	0.05	0.11	0.12	0.264	0.084
ENSMUSG00000115946	Mirt2	myocardial infraction associated transcript 2 [Source:MGI Symbol;Acc:MGI:3642813]	3.367003367	1.751465164	0.010546393	0.044858943	yes	up	2.71	0.59	0.47	1.08	0.63	0.27	0.04	0.15	0.23	1.1	0.264
ENSMUSG00000029601	Iqcd	IQ motif containing D [Source:MGI Symbol;Acc:MGI:1922982]	3.344481605	1.74178261	7.07966E-06	0.000105443	yes	up	1.61	1.89	1.4	1.66	0.26	0.46	0.51	0.28	0.5	1.462	0.402
ENSMUSG00000111167	Gm39321	predicted gene, 39321 [Source:MGI Symbol;Acc:MGI:5622206]	3.311258278	1.727379545	0.002533464	0.014383998	yes	up	1.78	0.6	0.64	1.36	0.46	0.38	0.2	0.16	0.1	0.918	0.26
ENSMUSG00000006056	Calcoco2	calcium binding and coiled-coil domain 2 [Source:MGI Symbol;Acc:MGI:1343177]	3.311258278	1.727379545	0.004335994	0.022244979	yes	up	0.47	0.82	0.61	0.71	0.29	0.19	0.4	0.07	0.07	0.592	0.204
ENSMUSG00000020131	Pcsk4	proprotein convertase subtilisin/kexin type 4 [Source:MGI Symbol;Acc:MGI:97514]	3.289473684	1.717856771	0.001792655	0.010830903	yes	up	0.82	1.31	0.33	1.32	0.18	0.32	0.15	0.29	0.2	0.802	0.228
ENSMUSG00000090002	Gm16006	predicted gene 16006 [Source:MGI Symbol;Acc:MGI:3801853]	3.215434084	1.685013515	0.002489435	0.014184314	yes	up	4.83	2.32	4.89	2.75	0.72	0.67	1.79	0.18	1.68	3.504	1.008
ENSMUSG00000027380	Acoxl	acyl-Coenzyme A oxidase-like [Source:MGI Symbol;Acc:MGI:1921371]	3.184713376	1.671163536	9.70238E-11	4.87989E-09	yes	up	48.83	38.89	53.15	50.11	6.4	13.28	14.38	13.67	9.06	48.47	11.358
ENSMUSG00000109305	1810010D01Rik	RIKEN cDNA 1810010D01 gene [Source:MGI Symbol;Acc:MGI:1916283]	3.174603175	1.666576266	0.001165143	0.007635026	yes	up	1.72	6.29	3.62	2.2	0.74	0.23	0.51	0.41	0.39	3.31	0.456
ENSMUSG00000002769	Gnmt	glycine N-methyltransferase [Source:MGI Symbol;Acc:MGI:1202304]	3.095975232	1.63039393	6.65502E-11	3.49352E-09	yes	up	6.2	7.55	5.54	6.97	1.79	1.41	1.76	2.71	1.86	6.264	1.906
ENSMUSG00000085407	1700095J03Rik	RIKEN cDNA 1700095J03 gene [Source:MGI Symbol;Acc:MGI:1921543]	3.095975232	1.63039393	0.004220524	0.021732511	yes	up	0.4	0.49	1.15	0.52	0.16	0.18	0.3	0.16	0.06	0.574	0.172
ENSMUSG00000090576	Gm17055	predicted gene 17055 [Source:MGI Symbol;Acc:MGI:4937882]	3.086419753	1.625934282	0.002997796	0.016465597	yes	up	0.42	0.21	0.51	0.27	0.08	0.23	0.14	0.05	0.1	0.38	0.12
ENSMUSG00000030693	Klk10	kallikrein related-peptidase 10 [Source:MGI Symbol;Acc:MGI:1916790]	3.067484663	1.61705613	0.001655282	0.010115435	yes	up	0.68	1.78	0.96	1.79	0.14	0.3	0.46	0.27	0.64	1.172	0.362
ENSMUSG00000036766	Dner	delta/notch-like EGF repeat containing [Source:MGI Symbol;Acc:MGI:2152889]	3.067484663	1.61705613	0.005375654	0.026509868	yes	up	0.21	0.43	0.2	0.24	0.03	0.1	0.16	0.03	0.06	0.252	0.076
ENSMUSG00000099762	Gm21149	predicted gene, 21149 [Source:MGI Symbol;Acc:MGI:5434504]	3.058103976	1.612637459	0.001121894	0.007400299	yes	up	1.96	4.73	5.25	5.95	2.37	0.53	1.86	0.15	1.4	4.156	1.262
ENSMUSG00000023993	Treml1	triggering receptor expressed on myeloid cells-like 1 [Source:MGI Symbol;Acc:MGI:1918576]	3.03030303	1.59946207	6.37158E-05	0.000680852	yes	up	1.38	2.28	1.46	1.27	0.42	0.48	0.45	0.52	0.54	1.568	0.482
ENSMUSG00000019892	Lrriq1	leucine-rich repeats and IQ motif containing 1 [Source:MGI Symbol;Acc:MGI:1922228]	3.021148036	1.595096878	4.85697E-12	3.3447E-10	yes	up	1.77	1.78	2.15	5.15	0.45	0.44	0.87	0.81	0.4	2.36	0.594
ENSMUSG00000071036	Gm10309	predicted gene 10309 [Source:MGI Symbol;Acc:MGI:3641941]	3.003003003	1.586405918	0.000493617	0.003778807	yes	up	0.75	0.99	0.83	0.52	0.2	0.35	0.32	0.09	0.19	0.722	0.23
ENSMUSG00000106139	Gm30648	predicted gene, 30648 [Source:MGI Symbol;Acc:MGI:5589807]	2.985074627	1.577766999	0.00061303	0.004524837	yes	up	0.82	0.62	0.96	1.6	0.03	1.03	0.17	0.42	0.31	1.182	0.392
ENSMUSG00000063130	Calml3	calmodulin-like 3 [Source:MGI Symbol;Acc:MGI:1917655]	2.985074627	1.577766999	0.007392077	0.033985156	yes	up	1.14	3.48	1.17	6.12	0.22	0.44	2.32	0.65	0.57	2.682	0.84
ENSMUSG00000078552	Dcdc2b	doublecortin domain containing 2b [Source:MGI Symbol;Acc:MGI:2686212]	2.96735905	1.569179503	1.0553E-05	0.000148321	yes	up	4.74	6.05	6.25	7.16	1.54	2.85	3.79	0.81	1.45	5.672	2.088
ENSMUSG00000045259	Klhdc9	kelch domain containing 9 [Source:MGI Symbol;Acc:MGI:1916124]	2.96735905	1.569179503	0.000133216	0.001264564	yes	up	1.46	1.31	0.79	1.66	0.25	0.3	0.5	0.37	0.53	1.216	0.39
ENSMUSG00000047671	Tctex1d4	Tctex1 domain containing 4 [Source:MGI Symbol;Acc:MGI:3045358]	2.915451895	1.543719518	2.20741E-11	1.30898E-09	yes	up	21.02	24.45	24.14	27.77	4.27	6.09	10.31	4.92	8.19	22.298	6.756
ENSMUSG00000041644	Slc5a12	solute carrier family 5 (sodium/glucose cotransporter), member 12 [Source:MGI Symbol;Acc:MGI:2138890]	2.906976744	1.53951953	6.42657E-07	1.28485E-05	yes	up	0.72	1.65	1.43	1.78	0.44	0.27	0.67	0.47	0.31	1.328	0.432
ENSMUSG00000046159	Chrm3	cholinergic receptor, muscarinic 3, cardiac [Source:MGI Symbol;Acc:MGI:88398]	2.906976744	1.53951953	1.1112E-05	0.00015437	yes	up	1.3	1.92	1.05	1.19	0.47	0.14	0.54	0.13	0.44	1.332	0.344
ENSMUSG00000016255	Tubb1	tubulin, beta 1 class VI [Source:MGI Symbol;Acc:MGI:107814]	2.898550725	1.535331733	2.88716E-11	1.65092E-09	yes	up	6.58	13.75	13.22	9.69	3.96	4.02	2.07	3.88	3.81	10.892	3.548
ENSMUSG00000038292	Ccdc155	coiled-coil domain containing 155 [Source:MGI Symbol;Acc:MGI:2687329]	2.898550725	1.535331733	1.67853E-05	0.000219825	yes	up	0.63	1.32	1.27	0.78	0.27	0.25	0.3	0.44	0.42	1.01	0.336
ENSMUSG00000042750	Bex2	brain expressed X-linked 2 [Source:MGI Symbol;Acc:MGI:1338017]	2.808988764	1.490050854	2.00727E-10	9.56966E-09	yes	up	11.89	14.79	15.78	16.65	2.9	4.71	6.12	7.55	3.32	14.724	4.92
ENSMUSG00000087231	E230016M11Rik	RIKEN cDNA E230016M11 gene [Source:MGI Symbol;Acc:MGI:2443524]	2.808988764	1.490050854	0.012018232	0.049807226	yes	up	0.54	0.73	1.21	1.78	0.02	0.59	0.63	0.3	1.18	1.504	0.544
ENSMUSG00000039037	St6galnac5	ST6 (alpha-N-acetyl-neuraminyl-2,3-beta-galactosyl-1,3)-N-acetylgalactosaminide alpha-2,6-sialyltransferase	2.78551532	1.477944251	1.72633E-06	3.03736E-05	yes	up	0.51	0.42	0.55	0.61	0.21	0.08	0.16	0.14	0.21	0.51	0.16

		5 [Source:MGI Symbol;Acc:MGI:1349471]																	
ENSMUSG00000056203	Tas2r135	taste receptor, type 2, member 135 [Source:MGI Symbol;Acc:MGI:2681302]	2.78551532	1.477944251	0.000205307	0.001812757	yes	up	1.15	0.59	0.9	0.78	0.11	0.18	0.46	0.35	0.11	0.776	0.242
ENSMUSG00000071392	Ect2l	epithelial cell transforming sequence 2 oncogene-like [Source:MGI Symbol;Acc:MGI:3641723]	2.754820937	1.461958547	4.70843E-07	9.72721E-06	yes	up	3.46	4.17	2.41	4.42	2.27	0.94	1.61	0.68	1.07	3.496	1.314
ENSMUSG00000052769	Gm9889	predicted gene 9889 [Source:MGI Symbol;Acc:MGI:3642568]	2.754820937	1.461958547	0.0001517	0.001410071	yes	up	1	1.1	1.21	0.89	0.27	0.32	0.28	0.38	0.47	1	0.344
ENSMUSG00000028773	Fabp3	fatty acid binding protein 3, muscle and heart [Source:MGI Symbol;Acc:MGI:95476]	2.754820937	1.461958547	0.006137468	0.029347729	yes	up	12.03	33.34	30	15.42	4.82	3.61	15.59	5.09	5.29	20.084	6.88
ENSMUSG00000006345	Ggt1	gamma-glutamyltransferase 1 [Source:MGI Symbol;Acc:MGI:95706]	2.747252747	1.457989644	0.000577782	0.004306021	yes	up	1.44	2.56	0.94	1.54	0.65	0.37	1.04	0.36	0.61	1.532	0.606
ENSMUSG00000021055	Esr2	estrogen receptor 2 (beta) [Source:MGI Symbol;Acc:MGI:109392]	2.739726027	1.454031631	1.90251E-06	3.30367E-05	yes	up	2.01	0.7	1.18	0.84	0.45	0.54	0.32	0.41	0.38	1.196	0.42
ENSMUSG00000102674	8030442B05Rik	RIKEN cDNA 8030442B05 gene [Source:MGI Symbol;Acc:MGI:1924793]	2.739726027	1.454031631	4.9664E-05	0.000549019	yes	up	1.62	1.12	1.69	1.38	0.84	0.37	0.17	0.57	0.37	1.354	0.464
ENSMUSG00000092586	Ly6g6c	lymphocyte antigen 6 complex, locus G6C [Source:MGI Symbol;Acc:MGI:2148930]	2.732240437	1.450084446	0.003345246	0.017998378	yes	up	1.65	0.55	2.05	1.93	0.66	0.8	0.99	0.44	0.21	1.614	0.62
ENSMUSG00000108256	Gm43923	predicted gene, 43923 [Source:MGI Symbol;Acc:MGI:5690315]	2.72479564	1.446148032	0.005302933	0.026191582	yes	up	0.46	0.42	0.38	0.51	0.04	0.37	0.09	0.11	0.12	0.418	0.146
ENSMUSG00000070806	Zmynd12	zinc finger, MYND domain containing 12 [Source:MGI Symbol;Acc:MGI:2140259]	2.702702703	1.434402824	0.000311197	0.002574946	yes	up	1.57	5.11	2.54	3.61	1	0.58	1.23	0.88	1.26	2.846	0.99
ENSMUSG00000107811	Gm44000	predicted gene, 44000 [Source:MGI Symbol;Acc:MGI:5690392]	2.702702703	1.434402824	0.001364763	0.008696911	yes	up	2.14	2.2	2.95	2.77	0.5	0.54	2.21	0.41	0.43	2.376	0.818
ENSMUSG00000078639	Gm12695	predicted gene 12695 [Source:MGI Symbol;Acc:MGI:3650206]	2.69541779	1.430508908	0.001858273	0.011160674	yes	up	1.41	3.01	1.57	3.43	0.87	1	2.13	0.57	0.71	2.346	1.056
ENSMUSG00000054181	A930012O16Rik	RIKEN cDNA A930012O16 gene [Source:MGI Symbol;Acc:MGI:2442255]	2.69541779	1.430508908	0.006477745	0.030714934	yes	up	0.6	0.68	0.72	0.62	0.2	0.34	0.05	0.35	0.18	0.644	0.224
ENSMUSG00000026579	F5	coagulation factor V [Source:MGI Symbol;Acc:MGI:88382]	2.688172043	1.426625474	2.00002E-12	1.48364E-10	yes	up	1.71	1.4	1.6	1.4	0.58	0.55	0.77	0.43	0.32	1.518	0.53
ENSMUSG00000047361	Gm973	predicted gene 973 [Source:MGI Symbol;Acc:MGI:2685819]	2.688172043	1.426625474	1.98955E-06	3.43135E-05	yes	up	4.16	8.18	8.37	11.74	2.97	1.54	4.88	2.2	1.56	7.362	2.63
ENSMUSG00000034212	Ankmy1	ankyrin repeat and MYND domain containing 1 [Source:MGI Symbol;Acc:MGI:3045261]	2.688172043	1.426625474	0.002102413	0.012374596	yes	up	0.89	1.34	1.09	1.22	0.21	0.66	0.62	0.07	0.21	1.03	0.354
ENSMUSG00000027793	Ccna1	cyclin A1 [Source:MGI Symbol;Acc:MGI:108042]	2.673796791	1.418889825	0.007821836	0.035493842	yes	up	0.55	1.05	1.01	0.63	0.35	0.31	0.36	0.11	0.29	0.776	0.284
ENSMUSG00000059824	Dbp	D site albumin promoter binding protein [Source:MGI Symbol;Acc:MGI:94866]	2.666666667	1.415037499	3.47245E-07	7.51309E-06	yes	up	63.7	73.9	67.54	79.09	21.9	12.11	24.46	26.15	35.87	68.544	24.098
ENSMUSG00000002588	Pon1	paraoxonase 1 [Source:MGI Symbol;Acc:MGI:103295]	2.659574468	1.411195433	3.07072E-11	1.7455E-09	yes	up	20.71	34.42	27.41	33.31	13.56	8.78	13.3	7.54	8.88	27.088	10.412
ENSMUSG00000104376	Gm37516	predicted gene, 37516 [Source:MGI Symbol;Acc:MGI:5610744]	2.659574468	1.411195433	1.83005E-09	7.04625E-08	yes	up	4.22	5.59	6.54	5.43	1.93	1.9	1.29	2.76	1.38	5.122	1.852
ENSMUSG00000060268	Armh1	armadillo-like helical domain containing 1 [Source:MGI Symbol;Acc:MGI:2686507]	2.659574468	1.411195433	5.83753E-07	1.18059E-05	yes	up	2.64	3.2	2.61	4.07	1.14	1.18	1.48	0.64	0.76	2.936	1.04
ENSMUSG00000085696	Hoxaas3	Hoxa cluster antisense RNA 3 [Source:MGI Symbol;Acc:MGI:1919878]	2.652519894	1.407363571	0.008902755	0.039258348	yes	up	1.49	1.5	2.85	2.23	0.93	0.43	0.41	1.13	0.84	2.354	0.748
ENSMUSG00000037973	Itprid1	ITPR interacting domain containing 1 [Source:MGI Symbol;Acc:MGI:2685304]	2.624671916	1.392137097	7.38109E-08	1.9138E-06	yes	up	2.76	3.7	2.68	4.53	1.45	1.16	1.88	0.58	1.04	3.462	1.222
ENSMUSG00000115536	Gm32857	predicted gene, 32857 [Source:MGI Symbol;Acc:MGI:5592016]	2.617801047	1.388355457	7.10421E-06	0.000105727	yes	up	4.8	6.35	7.3	9.28	3.79	1.21	2.11	1.89	1.85	6.726	2.17
ENSMUSG00000009070	Rsph14	radial spoke head homolog 14 (Chlamydomonas) [Source:MGI Symbol;Acc:MGI:1918486]	2.617801047	1.388355457	0.000874863	0.006033292	yes	up	4.38	7.93	4.33	5.89	0.88	2.32	3.23	0.22	1.56	5.318	1.642
ENSMUSG00000020562	Efcab10	EF-hand calcium binding domain 10 [Source:MGI Symbol;Acc:MGI:1922290]	2.617801047	1.388355457	0.002903325	0.016027009	yes	up	5.78	5.98	7.01	10.91	1.69	1.44	5.06	1.03	2.17	6.428	2.278
ENSMUSG00000092572	Serpinb10	serine (or cysteine) peptidase inhibitor, clade B (ovalbumin), member 10 [Source:MGI Symbol;Acc:MGI:2138648]	2.604166667	1.380821784	0.000226574	0.001964425	yes	up	1.73	2.58	2.88	4.34	1.7	1.27	0.24	1.23	1.03	2.732	1.094
ENSMUSG00000060402	Chst8	carbohydrate sulfotransferase 8 [Source:MGI Symbol;Acc:MGI:1916197]	2.597402597	1.377069649	4.21763E-06	6.64753E-05	yes	up	2.65	4.17	2.05	1.94	1.05	0.83	0.91	1.39	2.23	2.958	1.282

ENSMUSG00000050675	Gp1ba	glycoprotein 1b, alpha polypeptide [Source:MGI Symbol;Acc:MGI:1333744]	2.577319588	1.365871442	2.44429E-05	0.000299313	yes	up	0.57	1.1	1.38	1.1	0.4	0.3	0.45	0.25	0.42	1.032	0.364
ENSMUSG00000106416	Gm5857	predicted gene 5857 [Source:MGI Symbol;Acc:MGI:3644956]	2.577319588	1.365871442	0.005794129	0.028097577	yes	up	4.05	7.63	4.76	3	1.97	2.05	1.1	2.19	1.14	4.534	1.69
ENSMUSG00000111556	Gm19299	predicted gene, 19299 [Source:MGI Symbol;Acc:MGI:5011484]	2.570694087	1.36215794	0.000708763	0.005094452	yes	up	0.77	1.16	1.09	0.63	0.21	0.38	0.41	0.24	0.42	0.92	0.332
ENSMUSG00000070868	Skint3	selection and upkeep of intraepithelial T cells 3 [Source:MGI Symbol;Acc:MGI:3045331]	2.564102564	1.358453971	0.000663883	0.004844356	yes	up	1.89	0.65	1.23	1.69	0.24	0.32	0.74	0.76	0.28	1.286	0.468
ENSMUSG00000032033	Barx2	BarH-like homeobox 2 [Source:MGI Symbol;Acc:MGI:109617]	2.557544757	1.354759487	0.00025153	0.002150708	yes	up	0.94	2.73	1.69	1.96	0.38	0.74	0.94	0.35	0.88	1.784	0.658
ENSMUSG00000032118	Fez1	fasciculation and elongation protein zeta 1 (zygin I) [Source:MGI Symbol;Acc:MGI:2670976]	2.551020408	1.351074441	0.011118556	0.046758106	yes	up	0.9	0.43	1.11	1.48	0.15	0.3	0.51	0.74	0.09	0.988	0.358
ENSMUSG00000037157	Il22ra1	interleukin 22 receptor, alpha 1 [Source:MGI Symbol;Acc:MGI:2663588]	2.544529262	1.347398782	0.002398911	0.013769101	yes	up	0.59	1.19	0.83	1.05	0.43	0.14	0.68	0.19	0.18	0.884	0.324
ENSMUSG00000112129	Pbld1	phenazine biosynthesis-like protein domain containing 1 [Source:MGI Symbol;Acc:MGI:1915621]	2.538071066	1.343732465	0.000121082	0.001166673	yes	up	1.15	1.64	1.26	2.68	0.43	1.04	0.28	0.83	0.26	1.778	0.568
ENSMUSG00000029757	Dync1i1	dynein cytoplasmic 1 intermediate chain 1 [Source:MGI Symbol;Acc:MGI:107743]	2.538071066	1.343732465	0.000267335	0.002266683	yes	up	1.34	2.81	2	3.08	0.7	0.4	1.96	0.44	0.97	2.11	0.894
ENSMUSG00000035179	Ppp1r32	protein phosphatase 1, regulatory subunit 32 [Source:MGI Symbol;Acc:MGI:1915002]	2.538071066	1.343732465	0.000843825	0.005852852	yes	up	1.85	2.67	1.99	5.87	0.61	1.46	1.26	0.24	1.2	2.878	0.954
ENSMUSG00000087057	Gm11730	predicted gene 11730 [Source:MGI Symbol;Acc:MGI:3650941]	2.538071066	1.343732465	0.000942609	0.006424388	yes	up	5.47	2.31	4.2	1.46	0.72	0.93	1.09	2.02	1.61	3.356	1.274
ENSMUSG00000097260	Gm5706	predicted gene 5706 [Source:MGI Symbol;Acc:MGI:3643223]	2.53164557	1.340075442	4.83181E-08	1.32809E-06	yes	up	0.66	0.63	1.08	0.75	0.18	0.3	0.71	0.36	0.21	0.762	0.352
ENSMUSG00000048373	Fgfbp1	fibroblast growth factor binding protein 1 [Source:MGI Symbol;Acc:MGI:1096350]	2.518891688	1.332789088	1.70456E-13	1.52324E-11	yes	up	12.94	20.77	17.24	20.27	7.72	7.42	6.79	6.55	4.46	17.71	6.588
ENSMUSG00000078137	Ankrd63	ankyrin repeat domain 63 [Source:MGI Symbol;Acc:MGI:2686183]	2.512562814	1.329159664	3.61783E-09	1.30412E-07	yes	up	2.11	4.16	3.83	3.43	1.43	1.6	1.04	1.75	0.8	3.534	1.324
ENSMUSG00000029372	Ppbp	pro-platelet basic protein [Source:MGI Symbol;Acc:MGI:1888712]	2.506265664	1.325539348	5.49876E-07	1.12153E-05	yes	up	9.26	21.25	29.32	18.89	8.38	9.18	4.1	8.07	6.06	19.092	7.158
ENSMUSG00000045087	S1pr5	sphingosine-1-phosphate receptor 5 [Source:MGI Symbol;Acc:MGI:2150641]	2.5	1.321928095	1.90974E-06	3.31153E-05	yes	up	1.4	1.06	1.13	1.06	0.64	0.43	0.37	0.47	0.36	1.206	0.454
ENSMUSG00000092849	Gm24327	predicted gene, 24327 [Source:MGI Symbol;Acc:MGI:5454104]	2.493765586	1.318325858	0.002484468	0.014168622	yes	up	1.35	1.67	3.08	3.57	0.13	0.77	1.3	1.76	0.96	2.634	0.984
ENSMUSG00000053093	Myh7	myosin, heavy polypeptide 7, cardiac muscle, beta [Source:MGI Symbol;Acc:MGI:2155600]	2.487562189	1.314732593	7.80041E-10	3.26513E-08	yes	up	15.99	8.09	13.96	15.02	4.14	3.64	5.82	3.62	4.97	12.686	4.438
ENSMUSG00000045467	Ttll13	tubulin tyrosine ligase-like family, member 13 [Source:MGI Symbol;Acc:MGI:1920845]	2.487562189	1.314732593	0.000939903	0.006408217	yes	up	0.64	2.18	0.61	2.92	0.32	0.42	0.19	0.79	0.07	1.444	0.358
ENSMUSG00000026828	Galnt5	polypeptide N-acetylgalactosaminyltransferase 5 [Source:MGI Symbol;Acc:MGI:2179403]	2.481389578	1.311148256	0.001244378	0.008058051	yes	up	0.52	1.13	1.09	1	0.28	0.17	0.61	0.19	0.35	0.838	0.32
ENSMUSG00000035615	Frmpd1	FERM and PDZ domain containing 1 [Source:MGI Symbol;Acc:MGI:2446274]	2.469135802	1.304006187	1.69569E-15	2.27828E-13	yes	up	2.02	1.5	1.55	1.6	0.56	0.65	1.05	0.65	0.62	1.724	0.706
ENSMUSG00000100094	1810008I18Rik	RIKEN cDNA 1810008I18 gene [Source:MGI Symbol;Acc:MGI:1920875]	2.463054187	1.300448367	8.34401E-07	1.60905E-05	yes	up	2	2.39	3.04	3.62	0.82	1.19	1.8	0.66	1.46	2.554	1.186
ENSMUSG00000034936	Arl4d	ADP-ribosylation factor-like 4D [Source:MGI Symbol;Acc:MGI:1933155]	2.463054187	1.300448367	0.000277551	0.002339876	yes	up	77.23	135.4	95.55	33.08	37.4	39.11	39.47	33.14	23.86	89.842	34.596
ENSMUSG00000025665	Rps6ka6	ribosomal protein S6 kinase polypeptide 6 [Source:MGI Symbol;Acc:MGI:1914321]	2.457002457	1.2968993	0.000182244	0.001643876	yes	up	0.6	0.58	0.74	1.26	0.39	0.21	0.58	0.96	0.33	0.944	0.494
ENSMUSG00000053153	Spag16	sperm associated antigen 16 [Source:MGI Symbol;Acc:MGI:1913972]	2.450980392	1.293358943	2.05579E-08	6.22014E-07	yes	up	6.04	8.41	7.04	10.04	2.62	2.2	4.66	1.56	2.82	7.404	2.772
ENSMUSG00000067591	Klra3	killer cell lectin-like receptor, subfamily A, member 3 [Source:MGI Symbol;Acc:MGI:101905]	2.450980392	1.293358943	0.001503371	0.0093841	yes	up	1.56	1.69	1.07	1.35	0.27	0.79	0.68	0.61	0.34	1.382	0.538
ENSMUSG00000044772	Sntn	sentan, cilia apical structure protein [Source:MGI Symbol;Acc:MGI:3045373]	2.444987775	1.289827252	4.43665E-05	0.000498779	yes	up	8.46	18.56	19.6	14.38	5.7	4.29	9.14	2.43	5.11	13.894	5.334
ENSMUSG00000042269	Fam92b	family with sequence similarity 92, member B [Source:MGI Symbol;Acc:MGI:3588213]	2.433090024	1.282789701	3.26665E-06	5.30085E-05	yes	up	6.33	9.53	10.49	8.68	2.31	2.9	4.29	2.14	2.87	7.972	2.902
ENSMUSG00000034532	Fbxo16	F-box protein 16 [Source:MGI Symbol;Acc:MGI:1354706]	2.421307506	1.275786313	1.57404E-06	2.80278E-05	yes	up	2.56	2.29	3.96	3.3	1.14	0.21	1.04	1.72	0.94	2.736	1.01
ENSMUSG00000035694	Caps2	calcyphosphine 2 [Source:MGI Symbol;Acc:MGI:2441980]	2.415458937	1.272297327	0.000131852	0.001255339	yes	up	0.51	0.95	0.91	1.19	0.44	0.51	0.33	0.35	0.26	0.94	0.378

ENSMUSG00000055022	Cntn1	contactin 1 [Source:MGI Symbol;Acc:MGI:105980]	2.415458937	1.272297327	0.007743498	0.035254934	yes	up	0.16	0.37	0.58	0.81	0.38	0.16	0.14	0.13	0.34	0.548	0.23
ENSMUSG00000057265	Bbof1	basal body orientation factor 1 [Source:MGI Symbol;Acc:MGI:1920123]	2.409638554	1.268816758	1.52473E-06	2.72508E-05	yes	up	4.1	8.33	10.58	5.07	2.38	2.94	2.95	2.11	1.39	6.306	2.354
ENSMUSG00000030054	Gp9	glycoprotein 9 (platelet) [Source:MGI Symbol;Acc:MGI:1860137]	2.403846154	1.265344567	0.000355952	0.002892939	yes	up	3.05	3.56	3.39	2.46	1.92	1.47	1.45	1	0.55	3.272	1.278
ENSMUSG00000063529	Stmnd1	stathmin domain containing 1 [Source:MGI Symbol;Acc:MGI:2686420]	2.398081535	1.261880711	2.86545E-07	6.36461E-06	yes	up	7.66	12.89	11.37	13.89	3.07	3.84	6.79	3.07	4.39	10.812	4.232
ENSMUSG00000050157	Gm867	predicted gene 867 [Source:MGI Symbol;Acc:MGI:2685713]	2.398081535	1.261880711	1.55465E-05	0.000205713	yes	up	11.45	13.75	13.68	18.32	5.29	3.98	7.37	2.18	6.3	13.11	5.024
ENSMUSG00000034664	Itga2b	integrin alpha 2b [Source:MGI Symbol;Acc:MGI:96601]	2.392344498	1.258425153	9.51085E-11	4.79612E-09	yes	up	3.81	4.91	5.3	4.73	2.78	3.01	1.54	4.28	1.49	4.71	2.62
ENSMUSG00000026691	Fmo3	flavin containing monooxygenase 3 [Source:MGI Symbol;Acc:MGI:1100496]	2.392344498	1.258425153	0.000168113	0.001537346	yes	up	22.16	42.75	33.09	39.42	18.54	9.92	17.48	7.97	9.68	32.486	12.718
ENSMUSG00000041617	Ccdc74a	coiled-coil domain containing 74A [Source:MGI Symbol;Acc:MGI:1919565]	2.392344498	1.258425153	0.000792731	0.005558661	yes	up	3.44	3.81	4.64	4.31	0.79	3.37	2.79	1	0.95	3.828	1.78
ENSMUSG00000023034	Nr4a1	nuclear receptor subfamily 4, group A, member 1 [Source:MGI Symbol;Acc:MGI:1352454]	2.392344498	1.258425153	0.004520895	0.023027558	yes	up	59.46	19.56	25.73	21.8	15.79	16.57	23.97	12.26	16.01	38.536	16.92
ENSMUSG00000096141	Dnah7a	dynein, axonemal, heavy chain 7A [Source:MGI Symbol;Acc:MGI:2685838]	2.386634845	1.254977851	5.05798E-09	1.7573E-07	yes	up	2.47	2.47	4.72	3.88	1.61	1.78	2.65	2.55	3.05	3.374	2.328
ENSMUSG00000087001	Gm15475	predicted gene 15475 [Source:MGI Symbol;Acc:MGI:3705288]	2.380952381	1.251538767	0.001553961	0.009620344	yes	up	1.47	0.85	1.08	1.37	0.17	0.22	0.3	0.16	0.4	1.126	0.25
ENSMUSG00000022790	Igsf11	immunoglobulin superfamily, member 11 [Source:MGI Symbol;Acc:MGI:2388477]	2.375296912	1.248107862	0.000582508	0.00433284	yes	up	0.5	1.02	0.58	0.85	0.23	0.13	0.47	0.23	0.31	0.808	0.274
ENSMUSG00000029212	Gabrb1	gamma-aminobutyric acid (GABA) A receptor, subunit beta 1 [Source:MGI Symbol;Acc:MGI:95619]	2.375296912	1.248107862	0.005015884	0.025031214	yes	up	0.04	0.08	0.11	0.11	0.02	0.03	0.03	0.04	0.03	0.086	0.03
ENSMUSG00000106951	5930430L01Rik	RIKEN cDNA 5930430L01 gene [Source:MGI Symbol;Acc:MGI:2443110]	2.369668246	1.244685096	1.6855E-05	0.000220146	yes	up	1.45	0.89	2.15	1.3	0.49	0.59	0.59	0.41	0.69	1.318	0.554
ENSMUSG00000021751	Acox2	acyl-Coenzyme A oxidase 2, branched chain [Source:MGI Symbol;Acc:MGI:1934852]	2.364066194	1.241270432	1.15613E-11	7.21195E-10	yes	up	5.75	9.62	8.22	8.68	3.67	3.7	4.11	2.2	2.59	7.71	3.254
ENSMUSG00000090379	Gm8229	predicted gene 8229 [Source:MGI Symbol;Acc:MGI:3646979]	2.364066194	1.241270432	2.42471E-06	4.07219E-05	yes	up	7.44	10.48	8.3	9.26	4.24	3.64	5.69	1.52	3.01	9.24	3.62
ENSMUSG00000049690	Nckap5	NCK-associated protein 5 [Source:MGI Symbol;Acc:MGI:2686394]	2.358490566	1.23786383	2.79492E-31	4.13068E-28	yes	up	34.44	41.83	52.41	43.66	15.72	18.94	18.9	22.71	18.08	45.104	18.87
ENSMUSG00000039883	Lrrc17	leucine rich repeat containing 17 [Source:MGI Symbol;Acc:MGI:1921761]	2.358490566	1.23786383	1.37973E-06	2.48909E-05	yes	up	2.13	3.44	2.82	3.57	0.63	0.96	1.36	1.42	1.39	2.888	1.152
ENSMUSG00000046182	Gsg1l	GSG1-like [Source:MGI Symbol;Acc:MGI:2685483]	2.358490566	1.23786383	1.72613E-05	0.000224537	yes	up	1.08	1.13	1.94	1.37	0.19	0.64	0.61	0.82	0.43	1.406	0.538
ENSMUSG00000045193	Cirbp	cold inducible RNA binding protein [Source:MGI Symbol;Acc:MGI:893588]	2.352941176	1.234465254	2.65126E-17	4.71653E-15	yes	up	46.09	54.3	52.84	62	21.32	30.97	17.83	32.78	26.78	53.992	25.936
ENSMUSG00000023999	Kif6	kinesin family member 6 [Source:MGI Symbol;Acc:MGI:1098238]	2.352941176	1.234465254	0.000512691	0.003893408	yes	up	1.44	2.69	1.62	2.62	0.66	0.48	1.37	0.3	0.74	1.89	0.71
ENSMUSG00000068762	Gstm6	glutathione S-transferase, mu 6 [Source:MGI Symbol;Acc:MGI:1309467]	2.341920375	1.227692025	2.10092E-05	0.000263307	yes	up	3.32	3.14	5.04	2.37	1.54	1.5	1.68	1.03	1.25	3.396	1.4
ENSMUSG00000099553	Gm29538	predicted gene 29538 [Source:MGI Symbol;Acc:MGI:5580244]	2.336448598	1.224317298	0.00103255	0.006919556	yes	up	12.14	8.29	13.07	17.81	3.59	5.79	6.43	1.67	5.9	12.064	4.676
ENSMUSG00000045709	Smkr-ps	smal lysine rich protein 1, pseudogene [Source:MGI Symbol;Acc:MGI:1923669]	2.331002331	1.220950447	2.25913E-05	0.000279669	yes	up	6.42	7.31	6.04	7.19	1.72	3.09	2.24	2.15	3.83	6.252	2.606
ENSMUSG00000078810	Gp6	glycoprotein 6 (platelet) [Source:MGI Symbol;Acc:MGI:1889810]	2.331002331	1.220950447	0.0009775	0.006612923	yes	up	0.16	0.32	0.46	1.21	0.18	0.11	0.15	0.15	0.32	0.52	0.182
ENSMUSG00000019027	Dnah1	dynein, axonemal, heavy chain 1 [Source:MGI Symbol;Acc:MGI:107721]	2.325581395	1.217591435	2.12933E-07	4.88197E-06	yes	up	1.95	1.92	1.64	1.76	0.43	0.69	1.24	0.5	0.74	1.788	0.72
ENSMUSG00000003477	Inmt	indolethylamine N-methyltransferase [Source:MGI Symbol;Acc:MGI:102963]	2.320185615	1.214240226	5.21899E-18	1.02319E-15	yes	up	1091.44	1540.51	1301.22	1365.2	469.53	629.01	499.01	729.52	399.67	1340.04	545.348
ENSMUSG00000046242	Nme9	NME/NM23 family member 9 [Source:MGI Symbol;Acc:MGI:4359686]	2.314814815	1.210896782	1.25631E-07	3.07111E-06	yes	up	13.5	18.32	16.22	17.72	4.48	6.59	11.6	3.98	5.54	15.544	6.438
ENSMUSG00000029334	Prkg2	protein kinase, cGMP-dependent, type II [Source:MGI Symbol;Acc:MGI:108173]	2.309468822	1.20756107	9.96349E-12	6.31778E-10	yes	up	5.36	5.32	6.1	5.83	2.45	3.09	1.79	1.88	1.14	6.124	2.07
ENSMUSG00000038987	Cfap157	cilia and flagella associated protein 157 [Source:MGI Symbol;Acc:MGI:2447809]	2.309468822	1.20756107	3.4161E-06	5.50617E-05	yes	up	3.75	5.41	3.65	5.98	1.29	2.38	2.84	1.27	1.6	4.602	1.876

ENSMUSG00000036395	Glb1l2	galactosidase, beta 1-like 2 [Source:MGI Symbol;Acc:MGI:2388283]	2.304147465	1.204233052	2.95907E-20	8.74656E-18	yes	up	10.68	14.3	12.8	12.66	4.21	6.21	5.68	4.15	3.62	12.64	4.774
ENSMUSG00000021534	1700001L19Rik	RIKEN cDNA 1700001L19 gene [Source:MGI Symbol;Acc:MGI:1916565]	2.304147465	1.204233052	0.000133752	0.001267152	yes	up	6.62	7.23	6.77	12.54	4.56	2.19	4.68	1.42	3.19	7.748	3.208
ENSMUSG00000028967	Errfi1	ERBB receptor feedback inhibitor 1 [Source:MGI Symbol;Acc:MGI:1921405]	2.304147465	1.204233052	0.000190299	0.001706116	yes	up	232.58	139.78	104.44	71.56	65.8	56.48	76.72	60.17	39.22	146.514	59.678
ENSMUSG00000067616	Klk11	kallikrein related-peptidase 11 [Source:MGI Symbol;Acc:MGI:1929977]	2.298850575	1.200912694	0.003570845	0.018978324	yes	up	1.28	3.03	1.42	3.12	1.11	0.66	1.04	0.89	0.81	1.962	0.902
ENSMUSG00000029228	Lnx1	ligand of numb-protein X 1 [Source:MGI Symbol;Acc:MGI:1278335]	2.288329519	1.194294815	7.92084E-06	0.000115993	yes	up	1.84	1.64	2.53	42.24	42.13	0.66	0.6	0.39	0.86	9.982	8.928
ENSMUSG00000019178	Styxl1	serine/threonine/tyrosine interacting-like 1 [Source:MGI Symbol;Acc:MGI:1923821]	2.288329519	1.194294815	0.011319423	0.047453649	yes	up	1.47	1.71	0.95	1.32	0.31	0.47	0.81	0.15	0.54	1.248	0.456
ENSMUSG00000025330	Padi4	peptidyl arginine deiminase, type IV [Source:MGI Symbol;Acc:MGI:1338898]	2.283105023	1.190997225	0.000444178	0.003470516	yes	up	5.11	3.25	24.47	3.26	2.84	2.49	1.53	3.01	4.11	10.258	2.796
ENSMUSG00000020787	P2rx1	purinergic receptor P2X, ligand-gated ion channel, 1 [Source:MGI Symbol;Acc:MGI:1098235]	2.277904328	1.187707155	2.65789E-05	0.000321777	yes	up	1.43	1.42	1.96	1.7	0.41	0.8	0.76	0.85	0.74	1.564	0.712
ENSMUSG00000031027	Stk33	serine/threonine kinase 33 [Source:MGI Symbol;Acc:MGI:2152419]	2.272727273	1.184424571	1.78118E-06	3.12243E-05	yes	up	6.36	8.72	9.81	9.17	2.88	3.72	5	1.69	3.14	7.95	3.286
ENSMUSG00000031786	Drc7	dynein regulatory complex subunit 7 [Source:MGI Symbol;Acc:MGI:2685616]	2.272727273	1.184424571	5.6911E-06	8.65742E-05	yes	up	6.63	8.15	6.91	7.61	2.71	3.04	4.56	1.12	3.15	6.786	2.916
ENSMUSG00000061802	Armc4	armadillo repeat containing 4 [Source:MGI Symbol;Acc:MGI:1922184]	2.272727273	1.184424571	3.92202E-05	0.000448001	yes	up	1.88	2.78	2.57	5.39	1.02	0.92	1.76	0.62	0.96	2.916	1.056
ENSMUSG00000097056	Gm4262	predicted gene 4262 [Source:MGI Symbol;Acc:MGI:3782439]	2.272727273	1.184424571	0.00024026	0.002069078	yes	up	1.15	1.62	2.43	1.74	0.93	0.54	0.86	0.38	0.75	1.676	0.692
ENSMUSG00000037541	Shank2	SH3 and multiple ankyrin repeat domains 2 [Source:MGI Symbol;Acc:MGI:2671987]	2.267573696	1.181149439	1.45741E-08	4.59789E-07	yes	up	1.85	2.34	2.29	2.8	0.67	0.98	1.93	0.79	0.73	2.288	1.02
ENSMUSG00000052974	Cyp2f2	cytochrome P450, family 2, subfamily f, polypeptide 2 [Source:MGI Symbol;Acc:MGI:88608]	2.267573696	1.181149439	1.25648E-07	3.07111E-06	yes	up	1122.31	1761.51	1507.73	1658.72	609.66	558.41	786.99	418.66	556.56	1431.032	586.056
ENSMUSG00000031893	Tsnaxip1	translin-associated factor X (Tsnax) interacting protein 1 [Source:MGI Symbol;Acc:MGI:1919486]	2.267573696	1.181149439	7.07968E-06	0.000105443	yes	up	2.36	3.13	2.56	2.89	1.87	3.42	2.61	0.51	1.26	2.562	1.934
ENSMUSG00000090291	Lrrc10b	leucine rich repeat containing 10B [Source:MGI Symbol;Acc:MGI:2685551]	2.267573696	1.181149439	3.60084E-05	0.000416514	yes	up	7.98	10.16	7.65	8.47	2.55	2.55	6.28	1.62	3.74	8.148	3.348
ENSMUSG00000070877	Ldlrad1	low density lipoprotein receptor class A domain containing 1 [Source:MGI Symbol;Acc:MGI:3652166]	2.262443439	1.177881725	4.14977E-09	1.47102E-07	yes	up	12.39	15.41	14.19	14.86	4.3	5.58	8.57	3.35	5.32	13.62	5.424
ENSMUSG00000039661	Dusp26	dual specificity phosphatase 26 (putative) [Source:MGI Symbol;Acc:MGI:1914209]	2.262443439	1.177881725	0.002339967	0.013508921	yes	up	1.09	0.83	0.81	1.6	0.6	0.93	0.56	0.6	0.58	1.04	0.654
ENSMUSG00000023828	Slc22a3	solute carrier family 22 (organic cation transporter), member 3 [Source:MGI Symbol;Acc:MGI:1333817]	2.257336343	1.174621396	3.59594E-09	1.29866E-07	yes	up	1.78	2.07	1.91	1.51	0.67	35.8	0.8	0.75	0.66	1.894	7.736
ENSMUSG00000029811	Aoc1	amine oxidase, copper-containing 1 [Source:MGI Symbol;Acc:MGI:1923757]	2.257336343	1.174621396	8.3326E-05	0.000848312	yes	up	3.21	5.4	3.1	7.23	0.58	1.84	2.95	1.71	1.95	4.614	1.806
ENSMUSG00000002012	Pnck	pregnancy upregulated non-ubiquitously expressed CaM kinase [Source:MGI Symbol;Acc:MGI:1347357]	2.252252252	1.171368418	0.000115889	0.001124537	yes	up	2.11	1.98	2.36	2.26	0.55	1.29	0.75	0.88	1.11	2.154	0.916
ENSMUSG00000019767	Ccdc170	coiled-coil domain containing 170 [Source:MGI Symbol;Acc:MGI:2685067]	2.247191011	1.168122759	4.94207E-06	7.62055E-05	yes	up	4.2	5.41	4.43	5.64	1.75	1.89	3.28	0.77	2.34	4.762	2.006
ENSMUSG00000073208	Speer4c	spermatogenesis associated glutamate (E)-rich protein 4C [Source:MGI Symbol;Acc:MGI:1914023]	2.247191011	1.168122759	0.00553261	0.027034086	yes	up	8.95	5.61	3.74	3.68	3.74	1.16	4.57	1.39	1.67	5.11	2.506
ENSMUSG00000053111	Fank1	fibronectin type 3 and ankyrin repeat domains 1 [Source:MGI Symbol;Acc:MGI:1914180]	2.242152466	1.164884385	3.28477E-05	0.000385504	yes	up	4.07	4.06	3.59	3.31	1.14	1.33	2.59	0.77	1.64	3.572	1.494
ENSMUSG00000101337	Dnah7c	dynein, axonemal, heavy chain 7C [Source:MGI Symbol;Acc:MGI:3639762]	2.242152466	1.164884385	5.27022E-05	0.00057795	yes	up	7.8	1.11	9.51	1.44	0.22	0.26	0.62	0.19	0.28	4.108	0.314
ENSMUSG00000078451	Ppil6	peptidylprolyl isomerase (cyclophilin)-like 6 [Source:MGI Symbol;Acc:MGI:1920325]	2.242152466	1.164884385	0.000162738	0.001500875	yes	up	10.15	10.6	9.41	12.07	1.96	3.94	7.55	2.4	5.41	9.958	4.252
ENSMUSG00000074406	Zfp628	zinc finger protein 628 [Source:MGI Symbol;Acc:MGI:2665174]	2.242152466	1.164884385	0.000195644	0.001744273	yes	up	3.35	2.81	2.99	3.44	0.57	1.91	2.29	0.56	1.24	3.136	1.314
ENSMUSG00000031553	Adam3	a disintegrin and metallopeptidase domain 3 (cyritestin) [Source:MGI Symbol;Acc:MGI:102518]	2.242152466	1.164884385	0.000484186	0.003721652	yes	up	3.09	2.14	2.64	2.96	1.58	1.81	0.32	1.54	0.5	2.74	1.15
ENSMUSG00000025576	Rbfox3	RNA binding protein, fox-1 homolog (C. elegans) 3 [Source:MGI Symbol;Acc:MGI:106368]	2.242152466	1.164884385	0.005390759	0.026550285	yes	up	0.55	6.51	0.76	1.41	0.75	1.46	0.25	0.35	0.21	2.052	0.604
ENSMUSG00000074217	2210011C24Rik	RIKEN cDNA 2210011C24 gene [Source:MGI Symbol;Acc:MGI:1917384]	2.237136465	1.161653263	3.13891E-14	3.29551E-12	yes	up	12.86	13.03	9.47	13.15	4.02	5.13	6.23	4.58	4.22	12.448	4.836

ENSMUSG00000032915	Adgre4	adhesion G protein-coupled receptor E4 [Source:MGI Symbol;Acc:MGI:1196464]	2.237136465	1.161653263	1.20602E-09	4.82735E-08	yes	up	5	3.32	5.32	4.67	3.08	1.6	1.5	1.78	1.9	4.818	1.972
ENSMUSG00000062524	Ncr1	natural cytotoxicity triggering receptor 1 [Source:MGI Symbol;Acc:MGI:1336212]	2.237136465	1.161653263	1.88891E-06	3.2843E-05	yes	up	3.15	4.07	5.57	3.4	1.37	2.25	1.28	1.39	2.17	4.02	1.692
ENSMUSG00000029182	1700001C02Rik	RIKEN cDNA 1700001C02 gene [Source:MGI Symbol;Acc:MGI:1922684]	2.232142857	1.158429363	3.80575E-05	0.000437059	yes	up	9.62	13.2	11.08	10.03	4.61	4.69	5.94	2.19	4.2	10.216	4.326
ENSMUSG00000085368	Gm16188	predicted gene 16188 [Source:MGI Symbol;Acc:MGI:3802067]	2.232142857	1.158429363	0.010134612	0.04345376	yes	up	0.86	1.82	6.87	1.06	0.22	0.73	1.58	0.57	0.41	2.83	0.702
ENSMUSG00000022122	Ednrb	endothelin receptor type B [Source:MGI Symbol;Acc:MGI:102720]	2.227171492	1.15521265	2.04698E-14	2.27333E-12	yes	up	22.55	29.41	32.27	33.07	10.97	16.34	9.45	15.97	10.35	29.196	12.616
ENSMUSG00000047953	Gp5	glycoprotein 5 (platelet) [Source:MGI Symbol;Acc:MGI:1096363]	2.227171492	1.15521265	0.001632303	0.010010036	yes	up	0.58	1.42	1.56	1.02	0.51	0.77	0.4	0.26	0.48	1.148	0.484
ENSMUSG00000074673	Ttll9	tubulin tyrosine ligase-like family, member 9 [Source:MGI Symbol;Acc:MGI:1913589]	2.222222222	1.152003093	3.14815E-05	0.000372447	yes	up	2.02	3.51	2.51	2.44	0.95	1.11	1.4	1.11	1.09	2.42	1.132
ENSMUSG00000070306	Ccdc153	coiled-coil domain containing 153 [Source:MGI Symbol;Acc:MGI:2448587]	2.222222222	1.152003093	3.68725E-05	0.000425228	yes	up	113.9	166.09	147.62	191.12	45.11	62.43	99.62	39.14	49.34	143.664	59.128
ENSMUSG00000041460	Cacna2d4	calcium channel, voltage-dependent, alpha 2/delta subunit 4 [Source:MGI Symbol;Acc:MGI:2442632]	2.2172949	1.148800661	5.70499E-09	1.96082E-07	yes	up	2.2	2.84	4.69	2.95	0.92	1.99	0.83	1.64	1.1	3.194	1.296
ENSMUSG00000011305	Plin5	perilipin 5 [Source:MGI Symbol;Acc:MGI:1914218]	2.2172949	1.148800661	0.000991889	0.006693773	yes	up	1.74	3.62	2.35	2.64	0.75	1.13	1.84	0.61	0.74	2.376	1.014
ENSMUSG00000022759	Lrrc74b	leucine rich repeat containing 74B [Source:MGI Symbol;Acc:MGI:1921935]	2.212389381	1.145605322	2.48647E-07	5.60054E-06	yes	up	5.81	9.15	7.99	8.75	3.13	2.45	4.34	1.77	3.36	7.43	3.01
ENSMUSG00000068587	Mgam	maltase-glucoamylase [Source:MGI Symbol;Acc:MGI:1203495]	2.212389381	1.145605322	0.006462653	0.030658505	yes	up	0.71	0.32	0.47	0.28	0.99	0.1	0.15	0.18	0.14	0.446	0.312
ENSMUSG00000032680	6820408C15Rik	RIKEN cDNA 6820408C15 gene [Source:MGI Symbol;Acc:MGI:3045333]	2.207505519	1.142417045	2.8255E-05	0.000338655	yes	up	8.59	9.03	8.61	9.14	2.43	4.5	6.91	2.74	2.84	8.192	3.884
ENSMUSG00000020686	Gas2l2	growth arrest-specific 2 like 2 [Source:MGI Symbol;Acc:MGI:3652048]	2.207505519	1.142417045	0.001514587	0.009437007	yes	up	2.05	3.09	2.03	2.33	0.84	0.59	2.01	0.56	0.71	2.22	0.942
ENSMUSG00000025257	Ribc1	RIB43A domain with coiled-coils 1 [Source:MGI Symbol;Acc:MGI:1913861]	2.202643172	1.139235797	8.80819E-05	0.000887424	yes	up	2.09	1.99	2.22	2.49	1.05	0.83	1.23	0.44	0.99	2.136	0.908
ENSMUSG00000034112	Atp2c2	ATPase, Ca++ transporting, type 2C, member 2 [Source:MGI Symbol;Acc:MGI:1916297]	2.192982456	1.13289427	0.000379157	0.003035307	yes	up	2.29	3.44	2.76	3.49	0.89	1.04	2.39	0.69	0.88	2.746	1.178
ENSMUSG00000017737	Mmp9	matrix metallopeptidase 9 [Source:MGI Symbol;Acc:MGI:97011]	2.192982456	1.13289427	0.003927033	0.020458264	yes	up	22.77	5.95	7.85	8.56	7.16	2.98	4.06	4.98	4.13	10.358	4.662
ENSMUSG00000073077	Gm7173	predicted gene 7173 [Source:MGI Symbol;Acc:MGI:3645063]	2.188183807	1.12973393	0.00016612	0.001524195	yes	up	0.97	1.57	1.2	0.88	14.19	0.7	0.31	0.66	0.68	1.248	3.308
ENSMUSG00000110427	4933406B17Rik	RIKEN cDNA 4933406B17 gene [Source:MGI Symbol;Acc:MGI:1918290]	2.188183807	1.12973393	0.000253849	0.002169572	yes	up	0.66	0.79	0.7	0.38	0.18	0.54	0.33	0.13	0.44	0.674	0.324
ENSMUSG00000046093	Hpcal4	hippocalcin-like 4 [Source:MGI Symbol;Acc:MGI:2157521]	2.183406114	1.126580497	4.44995E-05	0.000499982	yes	up	1.75	3.71	3.24	1.42	0.85	0.68	1.15	1.08	1.68	2.608	1.088
ENSMUSG00000042567	Nek10	NIMA (never in mitosis gene a)- related kinase 10 [Source:MGI Symbol;Acc:MGI:2685128]	2.183406114	1.126580497	0.000352339	0.00286721	yes	up	0.64	1.19	0.77	1.09	0.32	0.25	0.88	0.31	0.42	0.848	0.436
ENSMUSG00000075325	Gm13582	predicted gene 13582 [Source:MGI Symbol;Acc:MGI:3642495]	2.183406114	1.126580497	0.000356917	0.002898328	yes	up	0.67	0.8	1.31	1.22	0.32	0.3	0.3	0.54	0.62	0.978	0.416
ENSMUSG00000085171	D830026I12Rik	RIKEN cDNA D830026I12 gene [Source:MGI Symbol;Acc:MGI:2442531]	2.183406114	1.126580497	0.000532315	0.004026523	yes	up	1.21	2.19	2.5	1.95	0.79	1.09	0.72	0.43	0.96	1.876	0.798
ENSMUSG00000028294	Cfap206	cilia and flagella associated protein 206 [Source:MGI Symbol;Acc:MGI:1916579]	2.178649237	1.123433941	1.33034E-08	4.2458E-07	yes	up	13.29	15.81	13.79	17.47	4.48	5.6	10.56	4.46	6.21	14.78	6.262
ENSMUSG00000039391	Ccdc81	coiled-coil domain containing 81 [Source:MGI Symbol;Acc:MGI:1918134]	2.178649237	1.123433941	2.56569E-05	0.000312585	yes	up	2.79	4.4	3.72	5.5	0.98	2.06	2.26	1.06	2.25	3.924	1.722
ENSMUSG00000117042	2700054A10Rik	RIKEN cDNA 2700054A10 gene [Source:NCBI gene;Acc:72578]	2.178649237	1.123433941	0.000568211	0.004249528	yes	up	0.9	0.78	0.98	1.33	0.46	0.26	0.36	0.18	0.44	0.962	0.34
ENSMUSG00000032515	Csrnp1	cysteine-serine-rich nuclear protein 1 [Source:MGI Symbol;Acc:MGI:2387989]	2.178649237	1.123433941	0.000971701	0.00658529	yes	up	37.32	41.45	21.68	13.87	14.6	12.43	18.53	10.72	12.08	31.352	13.672
ENSMUSG00000117916	AC141882.2	novel transcript	2.173913043	1.120294234	7.89934E-09	2.63033E-07	yes	up	4.68	5.68	4.96	6.08	1.86	2.6	2.56	1.9	2.12	5.002	2.208
ENSMUSG00000072473	1700024G13Rik	RIKEN cDNA 1700024G13 gene [Source:MGI Symbol;Acc:MGI:1914335]	2.173913043	1.120294234	1.37312E-07	3.32683E-06	yes	up	14.58	15.05	14.74	16.46	5.29	8.35	8.23	3.96	5.53	14.52	6.272
ENSMUSG00000054161	Fam83e	family with sequence similarity 83, member E [Source:MGI Symbol;Acc:MGI:1921063]	2.173913043	1.120294234	0.000380133	0.003041857	yes	up	1.52	2.44	2.25	3.1	0.84	1.01	2.37	0.66	0.78	2.462	1.132

ENSMUSG00000057606	Colq	collagen-like tail subunit (single strand of homotrimer) of asymmetric acetylcholinesterase [Source:MGI Symbol;Acc:MGI:1338761]	2.169197397	1.117161344	4.64564E-09	1.62285E-07	yes	up	3.73	3.44	3.76	5	2.3	1.62	10.8	1.11	1.72	3.914	3.51
ENSMUSG00000043164	Tmem212	transmembrane protein 212 [Source:MGI Symbol;Acc:MGI:2685410]	2.169197397	1.117161344	1.08629E-06	2.01847E-05	yes	up	42.61	62.74	47.68	67.75	15.71	23.02	33.93	19.53	19.5	50.868	22.338
ENSMUSG00000078588	Ccdc24	coiled-coil domain containing 24 [Source:MGI Symbol;Acc:MGI:2685874]	2.169197397	1.117161344	1.10613E-06	2.04939E-05	yes	up	3.64	3.5	3.17	5.25	2.2	1.91	3.21	1.45	1.15	4.008	1.984
ENSMUSG00000021061	Sptb	spectrin beta, erythrocytic [Source:MGI Symbol;Acc:MGI:98387]	2.169197397	1.117161344	1.14616E-05	0.000158562	yes	up	0.7	0.94	0.9	0.53	0.24	0.34	0.4	0.26	0.3	0.754	0.308
ENSMUSG00000037627	Rgs22	regulator of G-protein signalling 22 [Source:MGI Symbol;Acc:MGI:3613651]	2.169197397	1.117161344	5.64751E-05	0.000614066	yes	up	3.19	4.11	3.05	4.28	2.15	1.61	3.22	0.74	1.92	3.454	1.928
ENSMUSG00000001027	Scn4a	sodium channel, voltage-gated, type IV, alpha [Source:MGI Symbol;Acc:MGI:98250]	2.169197397	1.117161344	0.000179812	0.00162575	yes	up	0.34	0.28	0.37	0.33	0.19	0.2	0.13	0.09	0.15	0.358	0.152
ENSMUSG00000097343	9030407P20Rik	RIKEN cDNA 9030407P20 gene [Source:MGI Symbol;Acc:MGI:1918818]	2.169197397	1.117161344	0.000251106	0.00214804	yes	up	1.51	1.22	1.75	1.26	0.64	0.53	0.65	0.87	0.33	1.408	0.604
ENSMUSG00000022123	Scel	sciellin [Source:MGI Symbol;Acc:MGI:1891228]	2.164502165	1.114035243	1.92919E-15	2.53874E-13	yes	up	26.15	31.55	45	42.33	17.19	17.81	13.41	18.34	12.87	36.786	15.924
ENSMUSG00000073650	Catip	ciliogenesis associated TTC17 interacting protein [Source:MGI Symbol;Acc:MGI:2685062]	2.164502165	1.114035243	3.62502E-06	5.78949E-05	yes	up	4.64	6.08	4.94	6.42	3	3.48	3.32	2.82	2.57	5.188	3.038
ENSMUSG00000022783	Spag6l	sperm associated antigen 6-like [Source:MGI Symbol;Acc:MGI:1354388]	2.164502165	1.114035243	8.6841E-06	0.000125003	yes	up	9.61	11.81	13.84	12.2	3.43	3.76	8.39	2.72	4.3	10.756	4.52
ENSMUSG00000035828	Pim3	proviral integration site 3 [Source:MGI Symbol;Acc:MGI:1355297]	2.159827214	1.110915901	2.65944E-10	1.23122E-08	yes	up	62.05	53.86	61.49	32.24	28.21	25.04	25.85	27.03	17.54	53.864	24.734
ENSMUSG00000049154	Fam183b	family with sequence similarity 183, member B [Source:MGI Symbol;Acc:MGI:1922679]	2.159827214	1.110915901	4.5721E-06	7.13597E-05	yes	up	39.51	62.28	60.56	66.44	21.63	21.47	36.7	14.43	21.46	53.194	23.138
ENSMUSG00000057715	A830018L16Rik	RIKEN cDNA A830018L16 gene [Source:MGI Symbol;Acc:MGI:2444149]	2.159827214	1.110915901	6.7333E-05	0.000712373	yes	up	1.52	1.6	1.4	1.01	0.79	0.15	0.53	0.26	0.46	1.298	0.438
ENSMUSG00000024500	Ppp2r2b	protein phosphatase 2, regulatory subunit B, beta [Source:MGI Symbol;Acc:MGI:1920180]	2.155172414	1.10780329	0.000504307	0.003841892	yes	up	0.87	1.84	0.98	1.56	0.61	0.62	0.78	0.38	0.62	1.336	0.602
ENSMUSG00000024972	Lgals12	lectin, galactose binding, soluble 12 [Source:MGI Symbol;Acc:MGI:1929094]	2.155172414	1.10780329	0.008256416	0.036994057	yes	up	0.99	1.17	1.28	0.75	0.25	0.26	0.86	0.2	0.58	0.956	0.43
ENSMUSG00000010136	Pifo	primary cilia formation [Source:MGI Symbol;Acc:MGI:1923670]	2.145922747	1.10159814	3.79747E-07	8.00888E-06	yes	up	13.72	18.88	17.3	19.89	7.38	7.3	9.05	4.93	6.6	16.108	7.052
ENSMUSG00000032023	Jhy	junctional cadherin complex regulator [Source:MGI Symbol;Acc:MGI:1918239]	2.145922747	1.10159814	0.007369377	0.033905134	yes	up	0.83	1.4	1.56	1.27	0.5	0.25	29.37	0.25	0.48	1.436	6.17
ENSMUSG00000041660	Bbox1	butyrobetaine (gamma), 2-oxoglutarate dioxygenase 1 (gamma-butyrobetaine hydroxylase) [Source:MGI Symbol;Acc:MGI:1891372]	2.145922747	1.10159814	0.01069336	0.045343529	yes	up	1.25	3.36	1.8	2.23	0.22	0.66	2.79	0.12	0.98	2.072	0.954
ENSMUSG00000047844	Bex4	brain expressed X-linked 4 [Source:MGI Symbol;Acc:MGI:3606746]	2.141327623	1.098505545	3.11009E-09	1.13614E-07	yes	up	27.95	51.24	46.67	41.55	19.1	19.45	16.94	21.47	12.55	40.592	17.902
ENSMUSG00000033731	3300002A11Rik	RIKEN cDNA 3300002A11 gene [Source:MGI Symbol;Acc:MGI:1919073]	2.141327623	1.098505545	7.18634E-06	0.000106788	yes	up	6.56	17.67	16.1	12.55	5.38	4.51	8.54	3.16	2.9	13.638	4.898
ENSMUSG00000027530	Fabp12	fatty acid binding protein 12 [Source:MGI Symbol;Acc:MGI:1922747]	2.141327623	1.098505545	0.000422021	0.003321707	yes	up	3.58	6.11	5.82	3.54	3.39	3.54	3.24	2.18	0.87	4.67	2.644
ENSMUSG00000031831	Dnaaf1	dynein, axonemal assembly factor 1 [Source:MGI Symbol;Acc:MGI:1915520]	2.141327623	1.098505545	0.00146978	0.009213337	yes	up	2.16	4.72	2.99	3.26	0.96	1.21	2.51	0.58	1.38	3	1.328
ENSMUSG00000029168	Dpysl5	dihydropyrimidinase-like 5 [Source:MGI Symbol;Acc:MGI:1929772]	2.136752137	1.095419565	2.24869E-06	3.80988E-05	yes	up	1.17	2.62	2.32	2.24	1.01	1.21	0.67	0.94	0.73	2.164	0.912
ENSMUSG00000048029	Eno4	enolase 4 [Source:MGI Symbol;Acc:MGI:2441717]	2.136752137	1.095419565	2.77225E-06	4.56412E-05	yes	up	4.83	5.97	6.14	6.87	2.74	2	3.66	1.46	2.44	5.558	2.46
ENSMUSG00000066361	Serpina3c	serine (or cysteine) peptidase inhibitor, clade A, member 3C [Source:MGI Symbol;Acc:MGI:102848]	2.132196162	1.092340172	1.62922E-06	2.89835E-05	yes	up	2.64	6.1	4.16	5.38	2.24	2.52	1.91	2.15	1.61	4.718	2.086
ENSMUSG00000050625	Ccdc121	coiled-coil domain containing 121 [Source:MGI Symbol;Acc:MGI:2685601]	2.132196162	1.092340172	0.000255274	0.002178847	yes	up	2.99	4.55	5.48	6.8	1.22	2.33	3.17	1.09	2.48	4.69	2.058
ENSMUSG00000039004	Bmp6	bone morphogenetic protein 6 [Source:MGI Symbol;Acc:MGI:88182]	2.127659574	1.089267338	4.45478E-26	3.46464E-23	yes	up	52.3	63.59	67.57	59.61	23.78	33.03	23.89	30.93	28.45	63.42	28.016
ENSMUSG00000104213	Ighd	immunoglobulin heavy constant delta [Source:MGI Symbol;Acc:MGI:96447]	2.127659574	1.089267338	5.11676E-14	5.12022E-12	yes	up	34.12	25.34	32.59	36.89	14.77	18.4	17.16	12.47	17.64	35.19	16.088

ENSMUSG00000020014	Cfap54	cilia and flagella associated protein 54 [Source:MGI Symbol;Acc:MGI:1922208]	2.127659574	1.089267338	8.85016E-06	0.000127179	yes	up	2.59	2.63	3	3.68	1.29	1.06	2.37	0.62	1.06	2.816	1.28
ENSMUSG00000022340	Sybu	syntabulin (syntaxin-interacting) [Source:MGI Symbol;Acc:MGI:2442392]	2.127659574	1.089267338	0.000112304	0.001092501	yes	up	0.96	1.35	1.08	1.72	46.2	0.35	0.92	0.77	0.6	1.314	9.768
ENSMUSG00000021090	Lrrc9	leucine rich repeat containing 9 [Source:MGI Symbol;Acc:MGI:1925507]	2.127659574	1.089267338	0.002976496	0.016353279	yes	up	0.29	0.4	0.5	0.53	0.43	0.17	0.2	0.11	0.39	0.452	0.26
ENSMUSG00000055003	Lrtm2	leucine-rich repeats and transmembrane domains 2 [Source:MGI Symbol;Acc:MGI:2141485]	2.123142251	1.086201035	2.08722E-06	3.56912E-05	yes	up	4.08	4.31	4	5.3	1.84	1.83	0.93	3.12	1.68	4.498	1.88
ENSMUSG00000046491	C1qtnf2	C1q and tumor necrosis factor related protein 2 [Source:MGI Symbol;Acc:MGI:1916433]	2.118644068	1.083141235	4.88759E-13	3.96225E-11	yes	up	15.54	14.22	20.09	17.45	7.26	8.99	6.34	8.92	8.01	16.894	7.904
ENSMUSG00000037101	Ttc29	tetratricopeptide repeat domain 29 [Source:MGI Symbol;Acc:MGI:1920551]	2.118644068	1.083141235	6.96926E-06	0.000104041	yes	up	9.05	12.2	14.72	14.62	5.15	6.09	9.66	2.62	5.63	12.606	5.83
ENSMUSG00000059108	Ifitm6	interferon induced transmembrane protein 6 [Source:MGI Symbol;Acc:MGI:2686976]	2.118644068	1.083141235	0.007554423	0.034549662	yes	up	14.33	4.54	10.67	13.9	9.99	3.32	2.3	4.77	2.61	10.48	4.598
ENSMUSG00000028488	Sh3gl2	SH3-domain GRB2-like 2 [Source:MGI Symbol;Acc:MGI:700009]	2.114164905	1.080087911	6.34304E-07	1.27212E-05	yes	up	1.59	1.74	2.05	2.57	0.75	0.94	1.38	1.05	0.58	2.072	0.94
ENSMUSG00000053626	Tll1	tolloid-like [Source:MGI Symbol;Acc:MGI:106923]	2.114164905	1.080087911	9.86124E-07	1.85931E-05	yes	up	3.07	1.79	2.48	2.37	2.16	1.99	2.69	1.29	0.92	2.382	1.81
ENSMUSG00000010797	Wnt2	wingless-type MMTV integration site family, member 2 [Source:MGI Symbol;Acc:MGI:98954]	2.114164905	1.080087911	2.91666E-05	0.000348061	yes	up	3.93	5.59	4.72	4.38	1.7	1.74	1.31	3.9	2.47	4.994	2.224
ENSMUSG00000025089	Gfra1	glial cell line derived neurotrophic factor family receptor alpha 1 [Source:MGI Symbol;Acc:MGI:1100842]	2.109704641	1.077041036	4.51818E-09	1.58409E-07	yes	up	5.52	8.78	6.5	7.64	3.3	4.9	5.09	2.34	7.43	6.826	4.612
ENSMUSG00000024039	Cbs	cystathionine beta-synthase [Source:MGI Symbol;Acc:MGI:88285]	2.109704641	1.077041036	5.19498E-05	0.000571003	yes	up	2.63	3.23	3.49	2.67	1.02	0.92	1.24	0.79	0.73	2.748	0.94
ENSMUSG00000035539	Ccdc180	coiled-coil domain containing 180 [Source:MGI Symbol;Acc:MGI:2685871]	2.109704641	1.077041036	0.000137384	0.001299639	yes	up	2.38	3.77	3.44	4.71	1.81	1.58	3.03	0.67	1.81	3.394	1.78
ENSMUSG00000037005	Xpnpep2	X-prolyl aminopeptidase (aminopeptidase P) 2, membrane-bound [Source:MGI Symbol;Acc:MGI:2180001]	2.100840336	1.070966521	6.48094E-09	2.21563E-07	yes	up	1.86	2.16	2.41	1.92	2.05	0.93	1.17	1.1	0.85	2.452	1.22
ENSMUSG00000024653	Scgb1a1	secretoglobin, family 1A, member 1 (uteroglobin) [Source:MGI Symbol;Acc:MGI:98919]	2.096436059	1.067938829	2.64876E-14	2.81163E-12	yes	up	75339.49	105492.36	94630.88	95904.05	44942.39	36521.76	46239.58	35235.5	33202.99	87575.162	39228.444
ENSMUSG00000019945	Cabcoco1	ciliary associated calcium binding coiled-coil 1 [Source:MGI Symbol;Acc:MGI:1920537]	2.096436059	1.067938829	0.000626648	0.004616482	yes	up	5.18	5.26	4.74	7.77	1.72	1.87	2.98	1.67	3.71	5.112	2.39
ENSMUSG00000086610	Gm15408	predicted gene 15408 [Source:MGI Symbol;Acc:MGI:3705101]	2.096436059	1.067938829	0.009948805	0.042829127	yes	up	1.15	1.05	1.22	1.14	0.32	0.42	0.37	0.81	0.42	1.042	0.468
ENSMUSG00000075225	Ccdc162	coiled-coil domain containing 162 [Source:MGI Symbol;Acc:MGI:1923223]	2.092050209	1.064917477	7.9059E-06	0.000115951	yes	up	5.71	7.28	5.64	7.39	3.14	2.45	5.81	1.86	2.41	6.33	3.134
ENSMUSG00000027296	Itpka	inositol 1,4,5-trisphosphate 3-kinase A [Source:MGI Symbol;Acc:MGI:1333822]	2.092050209	1.064917477	7.39604E-05	0.000769773	yes	up	4.6	6.93	4.45	5.56	2.09	1.86	3.83	1.41	3.35	5.422	2.508
ENSMUSG00000000320	Alox12	arachidonate 12-lipoxygenase [Source:MGI Symbol;Acc:MGI:87998]	2.079002079	1.055891201	3.58876E-07	7.68682E-06	yes	up	2.32	3.21	4.33	3.05	1.49	1.36	1.03	1.99	1.69	3.258	1.512
ENSMUSG00000040938	Slc16a11	solute carrier family 16 (monocarboxylic acid transporters), member 11 [Source:MGI Symbol;Acc:MGI:2663709]	2.074688797	1.052894948	4.0567E-07	8.4996E-06	yes	up	20.64	27.55	12.07	24.51	11.37	5.67	10.51	4.23	7.84	21.338	7.924
ENSMUSG00000029838	Ptn	pleiotrophin [Source:MGI Symbol;Acc:MGI:97804]	2.074688797	1.052894948	0.009124265	0.040033	yes	up	1.4	1.66	1.04	1.77	0.33	0.45	0.96	0.33	1.19	1.444	0.652
ENSMUSG00000025150	Cbr2	carbonyl reductase 2 [Source:MGI Symbol;Acc:MGI:107200]	2.070393375	1.049904906	9.01277E-18	1.71448E-15	yes	up	3123.27	4116.29	3633.37	4106.63	1330.85	1577.06	1961.38	1558.71	1784.59	3611.756	1642.518
ENSMUSG00000026109	Tmeff2	transmembrane protein with EGF-like and two follistatin-like domains 2 [Source:MGI Symbol;Acc:MGI:1861735]	2.070393375	1.049904906	4.95364E-07	1.01791E-05	yes	up	2.47	2.97	4.24	3.72	1.19	106.67	1.26	2.33	1.24	3.464	22.538
ENSMUSG00000006542	Prkag3	protein kinase, AMP-activated, gamma 3 non-catalytic subunit [Source:MGI Symbol;Acc:MGI:1891343]	2.070393375	1.049904906	7.02542E-07	1.38725E-05	yes	up	2.35	1.96	2.09	2.04	0.6	1.43	0.85	1.16	1.15	2.114	1.038
ENSMUSG00000025176	Hoga1	4-hydroxy-2-oxoglutarate aldolase 1 [Source:MGI Symbol;Acc:MGI:1914682]	2.070393375	1.049904906	3.01772E-05	0.000359006	yes	up	3.17	3.28	3.35	3.98	1.03	1.92	1.98	2.28	0.71	3.518	1.584
ENSMUSG00000091415	Ak9	adenylate kinase 9 [Source:MGI Symbol;Acc:MGI:2685080]	2.070393375	1.049904906	7.86374E-05	0.000809245	yes	up	1.25	1.31	1.47	3.76	0.45	0.71	9.93	0.35	0.47	1.738	2.382
ENSMUSG00000030321	Efcab12	EF-hand calcium binding domain 12 [Source:MGI Symbol;Acc:MGI:2681834]	2.066115702	1.046921047	0.00102207	0.0068589	yes	up	1.06	1.78	1.42	2.02	0.89	0.79	1.28	0.35	0.82	1.708	0.826

ENSMUSG00000027886	1700013F07Rik	RIKEN cDNA 1700013F07 gene [Source:MGI Symbol;Acc:MGI:1922754]	2.066115702	1.046921047	0.001531592	0.009521883	yes	up	6.08	7.74	6.94	6.47	3.75	4.36	4.3	1.05	1.98	6.448	3.088
ENSMUSG00000054422	Fabp1	fatty acid binding protein 1, liver [Source:MGI Symbol;Acc:MGI:95479]	2.066115702	1.046921047	0.004840822	0.024340935	yes	up	7.69	6.09	11.37	10.39	4.37	4.72	2.22	7.71	1.76	9.182	4.156
ENSMUSG00000019278	Dpep1	dipeptidase 1 [Source:MGI Symbol;Acc:MGI:94917]	2.06185567	1.043943348	5.50197E-14	5.42099E-12	yes	up	67.65	96.07	90.37	83.38	40.9	44.93	43.14	41.62	25.93	86.3	39.304
ENSMUSG00000084989	Crocc2	ciliary rootlet coiled-coil, rootletin family member 2 [Source:MGI Symbol;Acc:MGI:3045962]	2.06185567	1.043943348	0.003809218	0.020029148	yes	up	0.69	1.11	0.84	0.73	0.26	0.29	0.62	0.13	0.44	0.76	0.348
ENSMUSG00000026581	Sell	selectin, lymphocyte [Source:MGI Symbol;Acc:MGI:98279]	2.057613169	1.040971781	2.61728E-10	1.21463E-08	yes	up	38.33	20.69	29.65	32.62	19.73	12.7	11.15	13.21	12.76	30.41	13.91
ENSMUSG00000038370	Pcp4l1	Purkinje cell protein 4-like 1 [Source:MGI Symbol;Acc:MGI:1913675]	2.057613169	1.040971781	1.50914E-08	4.75328E-07	yes	up	53.91	63.08	49.65	72.34	15.6	30.26	35.78	20.7	31.33	58.352	26.734
ENSMUSG00000027792	Bche	butyrylcholinesterase [Source:MGI Symbol;Acc:MGI:894278]	2.044989775	1.03209363	0.000324425	0.002667167	yes	up	1.35	1.01	1.3	2.02	0.44	0.79	0.84	0.51	0.3	1.374	0.576
ENSMUSG00000038354	Ankrd35	ankyrin repeat domain 35 [Source:MGI Symbol;Acc:MGI:2442590]	2.044989775	1.03209363	0.009584583	0.041671726	yes	up	0.6	0.81	0.34	0.96	0.3	0.68	0.42	0.1	0.28	0.646	0.356
ENSMUSG00000031618	Nr3c2	nuclear receptor subfamily 3, group C, member 2 [Source:MGI Symbol;Acc:MGI:99459]	2.040816327	1.029146346	6.20637E-21	2.12934E-18	yes	up	5.43	6.28	5.73	5.57	2.44	2.46	2.98	2.87	2.64	5.652	2.678
ENSMUSG00000030325	Klrb1c	killer cell lectin-like receptor subfamily B member 1C [Source:MGI Symbol;Acc:MGI:107538]	2.040816327	1.029146346	2.47161E-06	4.13649E-05	yes	up	4.11	5.17	6.95	4.34	1.93	1.85	2.1	2.74	1.73	4.788	2.07
ENSMUSG00000036019	Tmtc2	transmembrane and tetratricopeptide repeat containing 2 [Source:MGI Symbol;Acc:MGI:1914057]	2.036659878	1.02620507	2.21927E-18	4.79088E-16	yes	up	19.39	21.16	29.19	21.17	7.61	9.26	11.14	11.27	11.34	22.806	10.124
ENSMUSG00000033029	1700088E04Rik	RIKEN cDNA 1700088E04 gene [Source:MGI Symbol;Acc:MGI:1920774]	2.036659878	1.02620507	1.80081E-11	1.08122E-09	yes	up	28.43	26.99	24.33	21.73	9.58	11.42	12.31	9.05	12.5	24.472	10.972
ENSMUSG00000039963	Ccdc40	coiled-coil domain containing 40 [Source:MGI Symbol;Acc:MGI:2443893]	2.036659878	1.02620507	2.02172E-08	6.14611E-07	yes	up	14.4	13.8	12.59	14.65	6.91	5.66	9.25	3.96	5.41	12.646	6.238
ENSMUSG00000052131	Akr1b7	aldo-keto reductase family 1, member B7 [Source:MGI Symbol;Acc:MGI:101918]	2.036659878	1.02620507	0.001904549	0.011374604	yes	up	1.72	1.76	2.63	2.31	1.09	1.29	0.59	1.29	0.51	2.074	0.954
ENSMUSG00000025934	Gsta3	glutathione S-transferase, alpha 3 [Source:MGI Symbol;Acc:MGI:95856]	2.032520325	1.023269779	1.5833E-11	9.62656E-10	yes	up	135.31	195.01	178.23	173.05	88.2	68.94	78.11	91.44	50.66	167.076	75.47
ENSMUSG00000047394	Odf3b	outer dense fiber of sperm tails 3B [Source:MGI Symbol;Acc:MGI:1917363]	2.032520325	1.023269779	7.6416E-05	0.000792649	yes	up	16.24	12.97	9.81	12.25	3.43	6.41	9.01	3.21	6.02	11.982	5.616
ENSMUSG00000064280	Ccdc146	coiled-coil domain containing 146 [Source:MGI Symbol;Acc:MGI:1922422]	2.032520325	1.023269779	0.000227832	0.001971774	yes	up	5.41	6	5.61	6.74	2.74	1.58	4.58	1.12	2.96	5.518	2.596
ENSMUSG00000017309	Cd300lg	CD300 molecule like family member G [Source:MGI Symbol;Acc:MGI:1289168]	2.028397566	1.020340448	4.18292E-08	1.15969E-06	yes	up	7.9	10.76	7.88	5.9	3.32	3.88	3.25	4.97	2.69	7.96	3.622
ENSMUSG00000026649	Cfap126	cilia and flagella associated protein 126 [Source:MGI Symbol;Acc:MGI:1922722]	2.028397566	1.020340448	5.64767E-07	1.14703E-05	yes	up	29.54	42.95	38.9	45.39	18.06	19.57	26.26	14.12	15.59	37.086	18.72
ENSMUSG00000025936	Gm4956	predicted gene 4956 [Source:MGI Symbol;Acc:MGI:3647976]	2.024291498	1.017417053	1.70917E-08	5.29651E-07	yes	up	52.26	75.58	89.79	71.25	38.1	34.68	26.94	43.77	22.1	72.108	33.118
ENSMUSG00000030125	Lrrc23	leucine rich repeat containing 23 [Source:MGI Symbol;Acc:MGI:1315192]	2.024291498	1.017417053	4.77547E-07	9.84453E-06	yes	up	32	37.21	41.11	40.25	8.54	10.97	22.4	10.17	16.6	34.498	13.736
ENSMUSG00000039543	Cfap70	cilia and flagella associated protein 70 [Source:MGI Symbol;Acc:MGI:1923920]	2.024291498	1.017417053	0.000126977	0.001219265	yes	up	3.24	4.25	4.64	7.88	1.59	1.51	5.88	1.83	1.56	5.334	2.474
ENSMUSG00000050520	Cldn8	claudin 8 [Source:MGI Symbol;Acc:MGI:1859286]	2.024291498	1.017417053	0.000808362	0.005649715	yes	up	1.23	1.31	1.47	1.92	1.04	0.86	0.42	0.64	0.4	1.454	0.672
ENSMUSG00000057182	Scn3a	sodium channel, voltage-gated, type III, alpha [Source:MGI Symbol;Acc:MGI:98249]	2.02020202	1.01449957	3.59373E-11	2.01301E-09	yes	up	2.67	2.72	4.24	3.99	1.54	2	2.99	1.75	1.22	3.59	1.9
ENSMUSG00000033053	1700028P14Rik	RIKEN cDNA 1700028P14 gene [Source:MGI Symbol;Acc:MGI:1914733]	2.02020202	1.01449957	0.000844249	0.005853681	yes	up	5.52	4.93	5.71	5.91	1.94	1.96	4.48	1.77	1.98	5.25	2.426
ENSMUSG00000055489	Ano5	anoctamin 5 [Source:MGI Symbol;Acc:MGI:3576659]	2.02020202	1.01449957	0.000964286	0.006548895	yes	up	0.4	0.39	0.56	0.48	0.22	26.05	0.17	0.3	0.16	0.548	5.38
ENSMUSG00000045036	Tmem232	transmembrane protein 232 [Source:MGI Symbol;Acc:MGI:2685786]	2.02020202	1.01449957	0.009498224	0.041362053	yes	up	2.48	4.39	2.66	3.92	0.87	1.4	3.17	0.63	1.35	3.112	1.484
ENSMUSG00000097726	9530036O11Rik	RIKEN cDNA 9530036O11Rik [Source:MGI Symbol;Acc:MGI:2444607]	2.016129032	1.011587974	7.5969E-05	0.000788543	yes	up	3.5	5.15	11.59	2.64	1.03	0.93	2.46	1.78	4.21	5.448	2.082
ENSMUSG00000006235	Epor	erythropoietin receptor [Source:MGI Symbol;Acc:MGI:95408]	2.016129032	1.011587974	0.000370224	0.002980858	yes	up	2.8	3.05	2.26	3.67	0.6	2.18	0.52	1.47	0.78	2.7	1.11
ENSMUSG00000030087	Klf15	Kruppel-like factor 15 [Source:MGI Symbol;Acc:MGI:1929988]	2.012072435	1.008682243	2.86921E-07	6.36561E-06	yes	up	21.84	41.84	27.65	18.27	12.62	12.38	14.91	11.07	11.91	26.73	12.578

ENSMUSG00000005983	1700037C18Rik	RIKEN cDNA 1700037C18 gene [Source:MGI Symbol;Acc:MGI:1920511]	2.012072435	1.008682243	4.33665E-05	0.000488681	yes	up	6.92	5.56	4.94	4.27	1.39	2.83	2.36	1.54	1.95	4.888	2.014
ENSMUSG00000029727	Cyp3a13	cytochrome P450, family 3, subfamily a, polypeptide 13 [Source:MGI Symbol;Acc:MGI:88610]	2.012072435	1.008682243	0.000203764	0.00180245	yes	up	1.85	3.3	2.11	3.52	1.58	2.1	0.8	1.82	0.73	2.784	1.406
ENSMUSG00000010492	Uckl1os	uridine-cytidine kinase 1-like 1, opposite strand [Source:MGI Symbol;Acc:MGI:3801877]	2.008032129	1.005782353	4.67413E-05	0.000520905	yes	up	7.73	8.68	9.24	10.47	4.68	3.56	7.14	2.48	3.62	8.586	4.296
ENSMUSG00000045613	Chrm2	cholinergic receptor, muscarinic 2, cardiac [Source:MGI Symbol;Acc:MGI:88397]	2.004008016	1.002888279	1.15135E-08	3.69914E-07	yes	up	2.91	5.69	4.67	4.64	2.18	2.03	2.41	1.58	2.17	4.436	2.074
ENSMUSG00000028354	Fmn2	formin 2 [Source:MGI Symbol;Acc:MGI:1859252]	2.004008016	1.002888279	2.17329E-06	3.70501E-05	yes	up	2.32	2.3	4.16	3.57	1.83	1.73	1.37	1.56	1.17	3.35	1.532
ENSMUSG00000005131	4930550C14Rik	RIKEN cDNA 4930550C14 gene [Source:MGI Symbol;Acc:MGI:1922561]	2.004008016	1.002888279	0.001678582	0.010241535	yes	up	2.23	0.92	1.54	2.61	0.62	1.86	0.44	1.52	0.71	1.818	1.03
ENSMUSG00000040471	Ggt6	gamma-glutamyltransferase 6 [Source:MGI Symbol;Acc:MGI:1918772]	2.004008016	1.002888279	0.004549702	0.023143615	yes	up	2.1	2.86	2.63	1.91	0.5	1	1.78	0.84	1.33	2.144	1.09
ENSMUSG00000057337	Chst3	carbohydrate sulfotransferase 3 [Source:MGI Symbol;Acc:MGI:1858224]	2	1	1.29757E-20	4.24826E-18	yes	up	10.15	8.48	10.5	10.7	5.78	4.62	4.35	4.15	5.13	10.348	4.806
ENSMUSG00000022262	Dnah5	dynein, axonemal, heavy chain 5 [Source:MGI Symbol;Acc:MGI:107718]	2	1	0.005415251	0.026636769	yes	up	4.69	6.43	5.8	6.96	2.15	2.15	5.4	1.36	2.4	5.732	2.692
ENSMUSG00000020288	Ahsa2	AHA1, activator of heat shock protein ATPase 2 [Source:MGI Symbol;Acc:MGI:1916133]	0.499750125	-1.000721167	1.51059E-17	2.76408E-15	yes	down	14.06	12.78	13.65	10.39	27.35	27.23	24.66	27.19	20.35	13.484	25.356
ENSMUSG00000102700	Gm38312	predicted gene, 38312 [Source:MGI Symbol;Acc:MGI:5611540]	0.499750125	-1.000721167	0.000300912	0.002507119	yes	down	2.76	2.64	2.02	3.59	8.48	6.86	3.82	2.79	4.7	2.848	5.33
ENSMUSG00000030162	Olr1	oxidized low density lipoprotein (lectin-like) receptor 1 [Source:MGI Symbol;Acc:MGI:1261434]	0.499251123	-1.002162421	1.09386E-11	6.91327E-10	yes	down	21.62	27.76	21.87	21.54	33.89	40.31	34.98	39	35.58	23.04	36.752
ENSMUSG00000033031	Cip2a	cell proliferation regulating inhibitor of protein phosphatase 2A [Source:MGI Symbol;Acc:MGI:2146335]	0.499001996	-1.002882509	2.00021E-05	0.000252995	yes	down	1.65	0.76	1.58	1.5	3.5	3.21	3.16	2.2	3.75	1.496	3.164
ENSMUSG00000051220	Ercc6l	excision repair cross-complementing rodent repair deficiency complementation group 6 like [Source:MGI Symbol;Acc:MGI:2654144]	0.498753117	-1.003602237	1.29322E-05	0.000175844	yes	down	0.42	0.38	0.62	0.73	1.35	0.87	1.12	0.82	0.93	0.546	1.018
ENSMUSG00000031551	Ido1	indoleamine 2,3-dioxygenase 1 [Source:MGI Symbol;Acc:MGI:96416]	0.498007968	-1.005759269	0.006997109	0.032527331	yes	down	0.87	0.91	1.44	0.62	2.9	1.42	1	1.45	3.19	1.024	1.992
ENSMUSG00000006014	Prg4	proteoglycan 4 (megakaryocyte stimulating factor, articular superficial zone protein) [Source:MGI Symbol;Acc:MGI:1891344]	0.49776008	-1.006477564	0.005219177	0.0258788	yes	down	1.12	3.37	0.52	0.4	1.13	2.17	0.89	3.4	0.84	2.232	1.686
ENSMUSG00000005470	Asf1b	anti-silencing function 1B histone chaperone [Source:MGI Symbol;Acc:MGI:1914179]	0.497265042	-1.007913082	1.00618E-07	2.52045E-06	yes	down	2.22	3.55	5.12	3.12	5.9	4.09	3.99	5.27	10.97	4.098	6.044
ENSMUSG00000031266	Gla	galactosidase, alpha [Source:MGI Symbol;Acc:MGI:1347344]	0.496770989	-1.009347172	1.75341E-20	5.52265E-18	yes	down	15.19	9.78	15	10.83	24.81	29.24	20.5	27.74	23.35	12.82	25.128
ENSMUSG00000104350	Gm38244	predicted gene, 38244 [Source:MGI Symbol;Acc:MGI:5611472]	0.49652433	-1.010063683	0.006217699	0.029694422	yes	down	0.76	0.32	0.45	0.78	1.83	1.43	0.69	0.58	1.15	0.606	1.136
ENSMUSG00000032122	Slc37a2	solute carrier family 37 (glycerol-3-phosphate transporter), member 2 [Source:MGI Symbol;Acc:MGI:1929693]	0.496031746	-1.011495639	3.73798E-24	2.1763E-21	yes	down	6.49	4.59	6.33	5.77	9.82	9.09	10.37	9.48	11.59	5.946	10.07
ENSMUSG00000076752	Tcrg-C2	T-cell receptor gamma, constant 2 [Source:MGI Symbol;Acc:MGI:98626]	0.495540139	-1.012926174	7.80064E-05	0.000805255	yes	down	4.34	2.92	3.3	2.76	5.68	6.84	4.69	6.18	10.75	3.548	6.828
ENSMUSG00000112148	Lilrb4a	leukocyte immunoglobulin-like receptor, subfamily B, member 4A [Source:MGI Symbol;Acc:MGI:102701]	0.4952947	-1.013640911	2.02153E-05	0.000255021	yes	down	46.73	24.23	32.74	25.6	70.8	75.02	51.09	49.18	63.06	32.37	61.83
ENSMUSG00000089774	Slc5a3	solute carrier family 5 (inositol transporters), member 3 [Source:MGI Symbol;Acc:MGI:1858226]	0.494804552	-1.015069322	2.78857E-10	1.28174E-08	yes	down	1.24	1	0.85	1.28	2.52	2.29	2.14	2.43	1.52	1.152	2.18
ENSMUSG00000061013	Mkx	mohawk homeobox [Source:MGI Symbol;Acc:MGI:2687286]	0.494315373	-1.01649632	0.001774201	0.01074114	yes	down	1.12	1.2	0.28	2.55	0.7	2.24	0.53	1.11	0.91	1.106	1.098
ENSMUSG00000032024	Clmp	CXADR-like membrane protein [Source:MGI Symbol;Acc:MGI:1918816]	0.49382716	-1.017921908	1.22274E-25	8.70091E-23	yes	down	10.35	7.54	11.53	9.8	18.65	19.66	16.84	18.6	17.93	9.98	18.336
ENSMUSG00000079553	Kifc1	kinesin family member C1 [Source:MGI Symbol;Acc:MGI:109596]	0.493583416	-1.018634174	9.72323E-06	0.000138277	yes	down	1.63	1.14	1.81	1.53	3.61	3.21	2.64	2.22	4.47	1.714	3.23
ENSMUSG00000112349	Gm48132	predicted gene, 48132 [Source:MGI Symbol;Acc:MGI:6097493]	0.493583416	-1.018634174	0.001993525	0.011828782	yes	down	0.5	0.43	0.36	0.71	1.33	1.26	0.69	0.44	0.81	0.48	0.906

ENSMUSG00000017002 Slpi	secretory leukocyte peptidase inhibitor [Source:MGI Symbol;Acc:MGI:109297]	0.492368291 -1.02219024	5.03798E-13 4.06701E-11 yes	down	400.24	302.93	247.76	282.18	665.02	548.96	450.6	553.4	652.44	306.208	574.084
ENSMUSG00000033213 AA467197	expressed sequence AA467197 [Source:MGI Symbol;Acc:MGI:3034182]	0.492368291 -1.02219024	6.13828E-12 4.13806E-10 yes	down	35.81	31.99	37.39	29.38	55.56	77.16	51.9	72.33	88.34	35.954	69.058
ENSMUSG00000027496 Aurka	aurora kinase A [Source:MGI Symbol;Acc:MGI:894678]	0.492125984 -1.022900402	2.08801E-06 3.56912E-05 yes	down	2.96	1.57	2.02	2.27	5.1	3.08	3.37	3.27	5.63	2.25	4.09
ENSMUSG00000027737 Slc7a11	solute carrier family 7 (cationic amino acid transporter, y+ system), member 11 [Source:MGI Symbol;Acc:MGI:1347355]	0.491883915 -1.023610215	6.04946E-10 2.58285E-08 yes	down	1.7	1.53	1.71	1.22	3.94	2.41	2.62	2.96	3.39	1.626	3.064
ENSMUSG00000026630 Batf3	basic leucine zipper transcription factor, ATF-like 3 [Source:MGI Symbol;Acc:MGI:1925491]	0.491883915 -1.023610215	1.92612E-06 3.33694E-05 yes	down	5.53	5.62	4.84	5.04	9.71	14.4	9.85	7.78	11.24	5.288	10.596
ENSMUSG00000099398 Ms4a14	membrane-spanning 4-domains, subfamily A, member 14 [Source:MGI Symbol;Acc:MGI:2686122]	0.491400491 -1.025028794	7.52571E-05 0.000782123 yes	down	1	0.56	0.76	0.6	2.06	1.81	1.54	0.91	1.21	0.792	1.506
ENSMUSG00000020808 Pimreg	PICALM interacting mitotic regulator [Source:MGI Symbol;Acc:MGI:1924434]	0.491400491 -1.025028794	0.00749432 0.034332691 yes	down	2.35	0.9	3.1	2.12	4.42	5.27	1.66	4.09	6.18	2.384	4.324
ENSMUSG00000025001 Hells	helicase, lymphoid specific [Source:MGI Symbol;Acc:MGI:106209]	0.490436488 -1.027861775	3.02406E-05 0.000359316 yes	down	0.99	0.53	1.25	1.95	4.61	17.06	1.85	2.05	1.81	1.33	5.476
ENSMUSG00000041449 Serpina3h	serine (or cysteine) peptidase inhibitor, clade A, member 3H [Source:MGI Symbol;Acc:MGI:2182839]	0.490436488 -1.027861775	0.005720886 0.027804704 yes	down	0.73	0.93	0.75	0.42	1.46	1.46	1.4	0.84	1.57	0.696	1.346
ENSMUSG00000094689 Ighv1-81	immunoglobulin heavy variable 1-81 [Source:MGI Symbol;Acc:MGI:4439635]	0.490436488 -1.027861775	0.006933053 0.032331248 yes	down	273.62	592.18	411.65	290.95	723.81	1545.75	802.69	450.66	621.66	427.87	828.914
ENSMUSG00000017499 Cdc6	cell division cycle 6 [Source:MGI Symbol;Acc:MGI:1345150]	0.489955904 -1.029276182	6.79779E-05 0.000716827 yes	down	0.62	0.92	0.57	0.56	1.33	0.87	1.17	0.96	1.59	0.792	1.184
ENSMUSG00000050967 Creg2	cellular repressor of E1A-stimulated genes 2 [Source:MGI Symbol;Acc:MGI:1928333]	0.487804878 -1.03562391	2.25439E-11 1.33272E-09 yes	down	2.02	1.6	2.13	1.29	3.67	3.11	2.92	4.19	3.13	1.768	3.404
ENSMUSG00000029915 Clec5a	C-type lectin domain family 5, member a [Source:MGI Symbol;Acc:MGI:1345151]	0.48709206 -1.037733627	3.98693E-12 2.77539E-10 yes	down	13.76	7.23	13.87	11.61	18.9	17.83	19.77	17.58	29.12	12.02	20.64
ENSMUSG00000074476 Spc24	SPC24, NDC80 kinetochore complex component, homolog (S. cerevisiae) [Source:MGI Symbol;Acc:MGI:1914879]	0.486381323 -1.039840265	3.68154E-07 7.84184E-06 yes	down	2.49	2.36	2.32	3.46	10.15	3.68	4.97	5.91	6.79	2.59	6.3
ENSMUSG00000030346 Rad51ap1	RAD51 associated protein 1 [Source:MGI Symbol;Acc:MGI:1098224]	0.486381323 -1.039840265	0.006132402 0.029338108 yes	down	2.09	0.72	0.87	1.83	1.58	2.62	5.22	1.88	4.38	1.282	3.136
ENSMUSG00000024679 Ms4a6d	membrane-spanning 4-domains, subfamily A, member 6D [Source:MGI Symbol;Acc:MGI:1916024]	0.485436893 -1.042644337	1.18874E-19 3.2168E-17 yes	down	40.03	37.19	36.88	31.58	85.2	66.62	61.73	65.6	79.4	37.008	71.71
ENSMUSG00000078853 Igtp	interferon gamma induced GTPase [Source:MGI Symbol;Acc:MGI:107729]	0.484966052 -1.044044333	7.10493E-05 0.00074594 yes	down	23.18	25.82	36.05	23.73	60.61	35.89	37.85	43.91	80.42	26.958	51.736
ENSMUSG00000026177 Slc11a1	solute carrier family 11 (proton-coupled divalent metal ion transporters), member 1 [Source:MGI Symbol;Acc:MGI:1345275]	0.484730974 -1.044743821	9.81375E-12 6.26417E-10 yes	down	27.06	28.49	29.91	22.92	62.69	64.23	49.25	50.94	81.16	28.84	61.654
ENSMUSG00000050957 Insl6	insulin-like 6 [Source:MGI Symbol;Acc:MGI:1351595]	0.484496124 -1.045442971	5.56678E-05 0.000607007 yes	down	7.5	4.6	3.44	4.21	9.14	8.78	7.77	8.97	10.85	4.67	9.102
ENSMUSG00000095794 Igkv6-17	immunoglobulin kappa variable 6-17 [Source:MGI Symbol;Acc:MGI:1330833]	0.484261501 -1.046141782	0.000947622 0.006444839 yes	down	3337.69	2689.57	1264.03	1848.98	4638.63	3991.36	4015.69	3157.77	4778.19	2099.312	4116.328
ENSMUSG00000069793 Slfn9	schlafen 9 [Source:MGI Symbol;Acc:MGI:2445121]	0.483792937 -1.047538389	2.32717E-11 1.36734E-09 yes	down	1.2	0.85	1.58	1.91	2.51	2.69	2.24	2.51	3.32	1.412	2.654
ENSMUSG00000027306 Nusap1	nucleolar and spindle associated protein 1 [Source:MGI Symbol;Acc:MGI:2675669]	0.483792937 -1.047538389	4.92937E-08 1.35297E-06 yes	down	1.97	1.51	3	2.39	4.36	5.25	3.26	3.74	5.59	2.318	4.44
ENSMUSG00000005233 Spc25	SPC25, NDC80 kinetochore complex component, homolog (S. cerevisiae) [Source:MGI Symbol;Acc:MGI:1913692]	0.483792937 -1.047538389	1.91232E-05 0.000243967 yes	down	2.55	1.45	3.47	3.23	5.67	5.17	3.59	5.06	6.71	2.736	5.24
ENSMUSG00000002985 Apoe	apolipoprotein E [Source:MGI Symbol;Acc:MGI:88057]	0.483091787 -1.049630768	5.24347E-13 4.21518E-11 yes	down	4547.03	4330.79	3736.5	2903.83	8534.9	6570.84	7534.35	7042.04	8044.14	3827.57	7545.254
ENSMUSG00000052271 Bhlha15	basic helix-loop-helix family, member a15 [Source:MGI Symbol;Acc:MGI:891976]	0.482858522 -1.050327554	1.44717E-07 3.48865E-06 yes	down	1.88	1.66	1.34	1.57	4.14	2.41	2.87	2.38	3.54	1.58	3.068
ENSMUSG00000027996 Sfrp2	secreted frizzled-related protein 2 [Source:MGI Symbol;Acc:MGI:108078]	0.482858522 -1.050327554	0.004047182 0.020964817 yes	down	3.21	2.65	0.53	1.54	3.21	5.01	4.86	3.84	2.18	1.938	3.82

ENSMUSG00000028362	Tnfsf8	tumor necrosis factor (ligand) superfamily, member 8 [Source:MGI Symbol;Acc:MGI:88328]	0.482625483	-1.051024003	1.30678E-05	0.000177185	yes	down	16.59	0.99	1.25	0.72	4.73	1.77	2.09	1.63	2.52	4.164	2.548
ENSMUSG00000107094	Gm43294	predicted gene 43294 [Source:MGI Symbol;Acc:MGI:5663431]	0.481927711	-1.053111336	0.001482352	0.009277011	yes	down	0.69	0.47	0.37	0.28	1.22	0.73	0.85	1.06	0.68	0.466	0.908
ENSMUSG00000078881	Gm14434	predicted gene 14434 [Source:MGI Symbol;Acc:MGI:3702417]	0.481695568	-1.053806444	0.002342716	0.013512641	yes	down	16.25	14.63	1.86	21.17	45.59	50.53	24.11	6.8	37.57	15.428	32.92
ENSMUSG00000051048	P4ha3	procollagen-proline, 2-oxoglutarate 4-dioxygenase (proline 4-hydroxylase), alpha polypeptide III [Source:MGI Symbol;Acc:MGI:2444049]	0.481463649	-1.054501216	0.000398065	0.003166881	yes	down	1.57	0.87	1.99	1.41	1.86	3.06	3.36	2.38	5.41	1.59	3.214
ENSMUSG00000047678	Gpr82	G protein-coupled receptor 82 [Source:MGI Symbol;Acc:MGI:2441734]	0.481463649	-1.054501216	0.002582648	0.014608878	yes	down	0.63	0.29	0.3	0.45	0.93	0.74	0.96	0.58	0.61	0.394	0.764
ENSMUSG00000033491	Prss35	protease, serine 35 [Source:MGI Symbol;Acc:MGI:2444800]	0.480307397	-1.057970069	0.003689691	0.019496707	yes	down	0.32	0.53	0.4	0.63	0.29	1.36	1.03	0.64	0.95	0.444	0.854
ENSMUSG00000105160	A530030E21Rik	RIKEN cDNA A530030E21 gene [Source:MGI Symbol;Acc:MGI:2444617]	0.479846449	-1.059355278	0.009960519	0.042869947	yes	down	1.72	0.72	1.02	1.12	4.43	1.35	1.82	1.74	1.27	1.092	2.122
ENSMUSG00000045763	Basp1	brain abundant, membrane attached signal protein 1 [Source:MGI Symbol;Acc:MGI:1917600]	0.479616307	-1.060047384	4.04959E-08	1.12761E-06	yes	down	7.94	8.05	6.96	8.17	20.97	12.61	17.12	9.04	17.73	7.95	15.494
ENSMUSG00000074141	Il4i1	interleukin 4 induced 1 [Source:MGI Symbol;Acc:MGI:109552]	0.479386385	-1.060739158	0.0002829	0.002377668	yes	down	3.96	2.44	4.27	3.39	7.93	4.04	9.46	2.45	10.36	3.664	6.848
ENSMUSG00000104116	Gm37296	predicted gene, 37296 [Source:MGI Symbol;Acc:MGI:5610524]	0.479386385	-1.060739158	0.011431854	0.047831057	yes	down	0.21	0.14	0.25	0.15	0.48	0.24	0.28	0.45	0.37	0.186	0.364
ENSMUSG00000094546	Ighv1-26	immunoglobulin heavy variable 1-26 [Source:MGI Symbol;Acc:MGI:4439641]	0.4784689	-1.063502942	0.001444591	0.00909837	yes	down	955.45	819.56	451.58	932.57	2765.11	1269.92	1965.26	1541.35	1049.28	877.248	1718.184
ENSMUSG00000049723	Mmp12	matrix metallopeptidase 12 [Source:MGI Symbol;Acc:MGI:97005]	0.477326969	-1.066950244	1.00454E-06	1.88848E-05	yes	down	221.31	136.93	216.61	108.44	398.72	333.61	346.39	400.18	259.16	179.632	347.612
ENSMUSG00000069910	Spdl1	spindle apparatus coiled-coil protein 1 [Source:MGI Symbol;Acc:MGI:1917635]	0.477326969	-1.066950244	0.000669457	0.004880177	yes	down	0.61	0.74	1.3	0.79	1.88	2.73	1.41	0.82	1.56	0.842	1.68
ENSMUSG00000023505	Cdca3	cell division cycle associated 3 [Source:MGI Symbol;Acc:MGI:1315198]	0.476644423	-1.069014678	1.17752E-05	0.00016241	yes	down	3.51	1.83	4.31	2.66	4.84	4.75	3.47	3.67	7.08	3.138	4.762
ENSMUSG00000039395	Mreg	melanoregulin [Source:MGI Symbol;Acc:MGI:2151839]	0.475963827	-1.071076162	5.35718E-09	1.84458E-07	yes	down	3.9	3.85	3.99	3.65	11.44	8.56	6.73	7.85	8.79	4.416	8.674
ENSMUSG00000037568	Vash2	vasohibin 2 [Source:MGI Symbol;Acc:MGI:2444826]	0.475963827	-1.071076162	0.002338528	0.013504698	yes	down	0.45	0.28	0.2	0.22	0.6	0.54	0.59	0.54	0.33	0.334	0.52
ENSMUSG00000089716	Gm6264	predicted gene 6264 [Source:MGI Symbol;Acc:MGI:3779580]	0.475737393	-1.071762669	5.15686E-08	1.40812E-06	yes	down	8.87	4.84	8.81	6.12	17.24	9.66	10.75	11.37	13.6	7.224	12.524
ENSMUSG00000104563	Gm43041	predicted gene 43041 [Source:MGI Symbol;Acc:MGI:5663178]	0.475285171	-1.073134705	0.000118654	0.00114615	yes	down	1.51	0.67	1.54	1.34	3.93	3.17	2.21	1.97	1.59	1.322	2.574
ENSMUSG00000096954	Gdap10	ganglioside-induced differentiation-associated-protein 10 [Source:MGI Symbol;Acc:MGI:1338008]	0.474833808	-1.074505436	0.001275396	0.008242244	yes	down	0.36	0.31	0.39	0.4	1.14	0.71	0.75	0.26	0.63	0.354	0.698
ENSMUSG00000016239	Lonrf3	LON peptidase N-terminal domain and ring finger 3 [Source:MGI Symbol;Acc:MGI:1921615]	0.473709143	-1.077926579	8.1312E-11	4.18619E-09	yes	down	2.54	2.61	3.07	3.18	7.11	6.63	6.48	4.44	4.93	3.07	5.918
ENSMUSG00000044201	Cdc25c	cell division cycle 25C [Source:MGI Symbol;Acc:MGI:88350]	0.473484848	-1.078609835	0.010158402	0.043536331	yes	down	0.76	0.22	0.67	0.45	1.1	1	2.07	0.4	0.61	0.46	1.036
ENSMUSG00000024397	Aif1	allograft inflammatory factor 1 [Source:MGI Symbol;Acc:MGI:1343098]	0.473260767	-1.079292767	3.93914E-09	1.40936E-07	yes	down	15.55	12.86	15.21	13.8	27.33	22.59	29.45	22.68	43.64	14.82	29.138
ENSMUSG00000026864	Hspa5	heat shock protein 5 [Source:MGI Symbol;Acc:MGI:95835]	0.473036897	-1.079975377	2.63354E-54	5.05982E-50	yes	down	230.56	201.07	246	226.24	478.73	442.74	433.92	495.61	380.35	228.344	446.27
ENSMUSG00000055116	Arntl	aryl hydrocarbon receptor nuclear translocator-like [Source:MGI Symbol;Acc:MGI:1096381]	0.473036897	-1.079975377	9.05306E-09	2.97836E-07	yes	down	4.87	3.98	4.16	3.91	8.25	12.93	8.06	8.72	5.61	4.536	8.714
ENSMUSG00000058435	Btnl4	butyrophilin-like 4 [Source:MGI Symbol;Acc:MGI:1932036]	0.472143532	-1.082702589	0.008472541	0.037735522	yes	down	1.18	0.22	0.74	0.41	1.13	1.41	0.86	1.06	2.18	0.682	1.328
ENSMUSG00000027331	Knstrn	kinetochore-localized astrin/SPAG5 binding [Source:MGI Symbol;Acc:MGI:1289298]	0.471031559	-1.086104371	1.08246E-05	0.000150924	yes	down	4.6	1.77	6.27	9.16	27.95	68.35	7.18	7	18.5	5.096	25.796
ENSMUSG00000001517	Foxm1	forkhead box M1 [Source:MGI Symbol;Acc:MGI:1347487]	0.469483568	-1.09085343	5.64262E-08	1.50781E-06	yes	down	1.19	1.5	1.29	1.47	2.88	3.91	3.09	2.33	4.52	1.53	3.346
ENSMUSG00000072082	Ccnf	cyclin F [Source:MGI Symbol;Acc:MGI:102551]	0.469483568	-1.09085343	3.02024E-05	0.000359083	yes	down	1.04	0.93	0.92	1.41	2.48	1.75	1.79	1.28	2.76	1.148	2.012
ENSMUSG00000030786	Itgam	integrin alpha M [Source:MGI Symbol;Acc:MGI:96607]	0.469263257	-1.091530593	1.28775E-16	2.11466E-14	yes	down	24.05	17.19	20.99	16.9	39.39	34.2	30.57	37.34	49.64	19.718	38.228

ENSMUSG00000028031	Dkk2	dickkopf WNT signaling pathway inhibitor 2 [Source:MGI Symbol;Acc:MGI:1890663]	0.467726848	-1.096261853	0.000410461	0.003246677	yes	down	0.45	0.2	0.47	0.42	0.46	0.74	0.94	0.63	1.07	0.386	0.768
ENSMUSG00000092517	Art2a-ps	ADP-ribosyltransferase 2a, pseudogene [Source:MGI Symbol;Acc:MGI:107546]	0.467726848	-1.096261853	0.001115401	0.007365453	yes	down	0.34	0.47	0.72	0.57	1.72	1.33	1.34	0.4	1.53	0.452	1.264
ENSMUSG00000028270	Gbp2	guanylate binding protein 2 [Source:MGI Symbol;Acc:MGI:102772]	0.467508181	-1.096936483	1.6345E-06	2.90345E-05	yes	down	45.04	41.57	55.63	45.78	105.86	68.28	68.17	90.57	146.8	48.04	95.936
ENSMUSG00000021569	Trip13	thyroid hormone receptor interactor 13 [Source:MGI Symbol;Acc:MGI:1916966]	0.467508181	-1.096936483	0.000207743	0.001831746	yes	down	0.42	0.57	0.75	0.92	1.58	1.09	1.39	1.19	1.54	0.718	1.358
ENSMUSG00000037544	Dlgap5	DLG associated protein 5 [Source:MGI Symbol;Acc:MGI:2183453]	0.466853408	-1.09895848	4.96786E-06	7.64803E-05	yes	down	0.98	1.28	1.52	1.23	3.2	2.27	2.05	2.17	5.35	1.294	3.008
ENSMUSG00000052005	Gm9864	predicted gene 9864 [Source:MGI Symbol;Acc:MGI:3708663]	0.465983225	-1.101650076	0.000147765	0.001380196	yes	down	1.7	0.89	1.55	1.52	3.41	3.12	2.98	1.84	1.78	1.302	2.626
ENSMUSG00000050578	Mmp13	matrix metallopeptidase 13 [Source:MGI Symbol;Acc:MGI:1340026]	0.465983225	-1.101650076	0.001065348	0.00709727	yes	down	0.83	0.38	1.93	1.65	3.21	2.11	2.56	2.95	3.35	1.44	2.836
ENSMUSG00000009292	Trpm2	transient receptor potential cation channel, subfamily M, member 2 [Source:MGI Symbol;Acc:MGI:1351901]	0.465766185	-1.102322191	0.00114465	0.00752641	yes	down	4.25	1.74	1.81	1.89	6.93	3.7	3.26	4.76	9.12	2.288	5.554
ENSMUSG00000037474	Dtl	denticleless E3 ubiquitin protein ligase [Source:MGI Symbol;Acc:MGI:1924093]	0.465116279	-1.10433666	5.9347E-06	8.98529E-05	yes	down	0.52	0.63	0.45	0.89	1.32	0.94	1.01	1.21	1.56	0.602	1.208
ENSMUSG00000072844	G530011O06Rik	RIKEN cDNA G530011O06 gene [Source:MGI Symbol;Acc:MGI:3603513]	0.464900046	-1.105007525	0.007545111	0.034523509	yes	down	0.64	0.5	0.28	0.83	1.47	0.87	0.84	0.44	1.53	0.514	1.03
ENSMUSG00000051235	Gen1	GEN1, Holliday junction 5' flap endonuclease [Source:MGI Symbol;Acc:MGI:2443149]	0.464468184	-1.10634832	0.000290292	0.002427059	yes	down	0.16	0.5	0.42	0.42	1.51	27.35	0.47	0.47	0.92	0.368	6.144
ENSMUSG00000035273	Hpse	heparanase [Source:MGI Symbol;Acc:MGI:1343124]	0.464037123	-1.107687869	3.82517E-11	2.12407E-09	yes	down	9.04	6.96	7.89	6.15	18.84	12.34	10.92	14.43	19.76	7.556	15.258
ENSMUSG00000024353	Mzb1	marginal zone B and B1 cell-specific protein 1 [Source:MGI Symbol;Acc:MGI:1917066]	0.463821892	-1.108357178	9.80641E-05	0.000971189	yes	down	69.59	74.51	49.11	46.58	175.63	93.28	95.69	84.28	140.75	57.72	117.926
ENSMUSG00000096452	Ighv1-77	immunoglobulin heavy variable 1-77 [Source:MGI Symbol;Acc:MGI:4439670]	0.463821892	-1.108357178	0.01065187	0.045207506	yes	down	122	232.39	119.76	81.43	324.19	194.06	520.22	134.81	276.3	143.342	289.916
ENSMUSG00000093896	Ighv1-76	immunoglobulin heavy variable 1-76 [Source:MGI Symbol;Acc:MGI:4439737]	0.46339203	-1.109694865	0.002142778	0.012574584	yes	down	382.06	564.21	161.52	496.95	1155.16	640.43	726.24	705.42	623.21	375.002	770.092
ENSMUSG00000023992	Trem2	triggering receptor expressed on myeloid cells 2 [Source:MGI Symbol;Acc:MGI:1913150]	0.463177397	-1.110363243	1.5595E-15	2.12501E-13	yes	down	130.84	89.23	105.57	76.04	163.25	207.24	153.34	228.07	232.4	100.022	196.86
ENSMUSG00000029380	Cxcl1	chemokine (C-X-C motif) ligand 1 [Source:MGI Symbol;Acc:MGI:108068]	0.462320851	-1.113033665	0.008363544	0.037360794	yes	down	0.94	0.83	1.16	0.86	2.33	4.57	1.43	1.63	1.8	1.142	2.352
ENSMUSG00000026779	Mastl	microtubule associated serine/threonine kinase-like [Source:MGI Symbol;Acc:MGI:1914371]	0.461893764	-1.114367025	0.000549976	0.004135693	yes	down	0.21	0.19	0.54	0.45	1	0.61	0.53	0.5	1.48	0.356	0.824
ENSMUSG00000039396	Neil3	nei like 3 (E. coli) [Source:MGI Symbol;Acc:MGI:2384588]	0.461893764	-1.114367025	0.002736676	0.015293703	yes	down	0.55	0.37	0.93	0.62	1.89	1.63	19.07	0.48	1.26	0.614	4.866
ENSMUSG00000115148	Gm49125	predicted gene, 49125 [Source:MGI Symbol;Acc:MGI:6118530]	0.460617227	-1.118359726	0.005495831	0.026907742	yes	down	1.42	0.73	0.9	1.04	2.35	3.19	0.94	0.93	2.67	0.986	2.016
ENSMUSG00000051517	Arhgef39	Rho guanine nucleotide exchange factor (GEF) 39 [Source:MGI Symbol;Acc:MGI:3036286]	0.459558824	-1.121678557	0.002019674	0.011958088	yes	down	0.95	0.65	0.97	0.73	7.83	4.69	1.31	1.64	3.21	0.99	3.736
ENSMUSG00000035270	Impg2	interphotoreceptor matrix proteoglycan 2 [Source:MGI Symbol;Acc:MGI:3044955]	0.458926113	-1.123666196	0.003818968	0.020050731	yes	down	0.11	0.06	0.07	0.21	0.29	0.26	0.27	0.22	0.15	0.118	0.238
ENSMUSG00000081058	Hist2h3c2	histone cluster 2, H3c2 [Source:MGI Symbol;Acc:MGI:2448357]	0.458085204	-1.12631213	0.000246346	0.002111084	yes	down	1.28	2.01	1.77	1.95	3.56	2.66	3.52	4.14	2.4	1.592	3.256
ENSMUSG00000092438	Gm18734	predicted gene, 18734 [Source:MGI Symbol;Acc:MGI:5010919]	0.457665904	-1.12763328	0.001107015	0.007316503	yes	down	11.29	4.4	3.68	5.54	24.99	25.46	22.02	7.89	18.68	8.67	19.808
ENSMUSG00000038379	Ttk	Ttk protein kinase [Source:MGI Symbol;Acc:MGI:1194921]	0.457456542	-1.128293401	0.001153112	0.007561345	yes	down	0.73	0.37	0.86	0.59	1.76	2.11	0.95	0.49	1.37	0.626	1.336
ENSMUSG00000098318	Lockd	lncRNA downstream of Cdkn1b [Source:MGI Symbol;Acc:MGI:1915081]	0.457247371	-1.12895322	0.00812034	0.036546287	yes	down	0.42	0.09	0.24	0.05	0.38	0.16	0.25	0.19	0.29	0.196	0.254
ENSMUSG00000032113	Chek1	checkpoint kinase 1 [Source:MGI Symbol;Acc:MGI:1202065]	0.456829603	-1.130271955	0.006583848	0.031072334	yes	down	0.32	0.59	0.99	0.37	1.6	0.72	0.75	0.47	1.22	0.982	0.952
ENSMUSG00000100029	D7Ertd128e	DNA segment, Chr 7, ERATO Doi 128, expressed [Source:MGI Symbol;Acc:MGI:1098716]	0.456412597	-1.131589484	0.011602901	0.04837694	yes	down	0.87	0.72	0.6	0.63	1.47	2.3	1.42	2.78	0.87	0.86	1.768
ENSMUSG00000078921	Tgtp2	T cell specific GTPase 2 [Source:MGI Symbol;Acc:MGI:3710083]	0.455373406	-1.134878054	1.12971E-05	0.000156715	yes	down	26.91	31.53	46.69	30.69	82.23	43.64	60.94	49.6	96.86	33.088	66.654
ENSMUSG00000022034	Esco2	establishment of sister chromatid cohesion N-acetyltransferase 2 [Source:MGI Symbol;Acc:MGI:1919238]	0.455373406	-1.134878054	3.88019E-05	0.000444279	yes	down	0.54	0.52	0.69	0.99	1.55	1.41	1.1	0.82	2	0.696	1.376

ENSMUSG00000048480	Cxcr1	chemokine (C-X-C motif) receptor 1 [Source:MGI Symbol;Acc:MGI:2448715]	0.455373406	-1.134878054	0.010884144	0.046010355	yes	down	3.59	0.78	0.57	0.96	2.83	4.16	1.68	4.07	3.43	1.552	3.234
ENSMUSG00000026683	Nuf2	NUF2, NDC80 kinetochore complex component [Source:MGI Symbol;Acc:MGI:1914227]	0.454959054	-1.136191386	1.23896E-05	0.000169908	yes	down	1.47	0.9	2.43	2.13	4.18	4.44	2.71	2.65	4.88	1.846	3.772
ENSMUSG00000109105	Gm44873	predicted gene 44873 [Source:MGI Symbol;Acc:MGI:5753449]	0.454959054	-1.136191386	0.005968291	0.028717448	yes	down	0.11	0.29	0.1	0.29	0.68	0.44	0.38	0.32	0.5	0.22	0.464
ENSMUSG00000099583	Hist1h3d	histone cluster 1, H3d [Source:MGI Symbol;Acc:MGI:2448322]	0.454959054	-1.136191386	0.007835006	0.035518395	yes	down	0.47	0.48	0.28	0.65	1.16	1.52	0.65	0.74	0.74	0.464	0.962
ENSMUSG00000111619	Gm48348	predicted gene, 48348 [Source:MGI Symbol;Acc:MGI:6097812]	0.454545455	-1.137503524	2.85195E-05	0.000341611	yes	down	3.5	1.41	2.11	2.75	8.39	6.27	4.2	3.67	3.37	2.534	5.18
ENSMUSG00000030867	Plk1	polo like kinase 1 [Source:MGI Symbol;Acc:MGI:97621]	0.454338937	-1.138159145	9.0319E-07	1.72324E-05	yes	down	2.08	1.25	2.13	1.85	4.44	2.97	3.84	2.55	5.47	1.972	3.854
ENSMUSG00000030361	Klrb1a	killer cell lectin-like receptor subfamily B member 1A [Source:MGI Symbol;Acc:MGI:107540]	0.454132607	-1.138814469	0.000294398	0.002457112	yes	down	1.13	1.28	1.56	1.07	1.2	3.76	2.78	2.02	2.52	1.238	2.456
ENSMUSG00000042254	Cilp	cartilage intermediate layer protein, nucleotide pyrophosphohydrolase [Source:MGI Symbol;Acc:MGI:2444507]	0.453103761	-1.14208663	7.62117E-07	1.48958E-05	yes	down	2.12	2.36	2.37	2.65	4.55	4.12	7.18	2.33	6.26	2.368	4.888
ENSMUSG00000015656	Hspa8	heat shock protein 8 [Source:MGI Symbol;Acc:MGI:105384]	0.452693526	-1.143393419	9.42249E-41	4.52586E-37	yes	down	932.33	774.15	888.99	838.11	1994.62	1742.7	1867.55	2030.42	1684.47	906.124	1863.952
ENSMUSG00000046591	Ticrr	TOPBP1-interacting checkpoint and replication regulator [Source:MGI Symbol;Acc:MGI:1924261]	0.452693526	-1.143393419	5.83088E-05	0.000631148	yes	down	0.21	0.3	0.17	0.15	0.38	0.28	0.48	0.46	0.54	0.234	0.428
ENSMUSG00000029379	Cxcl3	chemokine (C-X-C motif) ligand 3 [Source:MGI Symbol;Acc:MGI:3037818]	0.451875282	-1.146003451	0.000192601	0.001722738	yes	down	1.91	2.04	5.03	2.06	5.36	5.87	8.77	4	5.88	2.858	5.976
ENSMUSG00000113491	Gm19221	predicted gene, 19221 [Source:MGI Symbol;Acc:MGI:5011406]	0.451875282	-1.146003451	0.001323614	0.00847969	yes	down	1.21	0.69	0.56	0.76	2.15	2.74	1.57	0.91	1.87	0.89	1.848
ENSMUSG00000031891	Hsd11b2	hydroxysteroid 11-beta dehydrogenase 2 [Source:MGI Symbol;Acc:MGI:104720]	0.450856628	-1.149259365	0.004597839	0.023345211	yes	down	0.36	0.24	0.4	0.61	1.04	1.12	1.37	0.54	1.13	0.51	1.04
ENSMUSG00000048327	Ckap2l	cytoskeleton associated protein 2-like [Source:MGI Symbol;Acc:MGI:1917716]	0.45045045	-1.150559677	3.96286E-09	1.41521E-07	yes	down	1.5	1.44	1.66	2.23	4.34	3.86	3.29	2.49	4.67	1.81	3.73
ENSMUSG00000028068	Iqgap3	IQ motif containing GTPase activating protein 3 [Source:MGI Symbol;Acc:MGI:3028642]	0.45045045	-1.150559677	2.24918E-05	0.000278977	yes	down	1	0.48	1.06	0.87	1.7	2.06	1.24	1.67	2.95	0.864	1.924
ENSMUSG00000050908	Tvp23a	trans-golgi network vesicle protein 23A [Source:MGI Symbol;Acc:MGI:3665441]	0.450247636	-1.151209393	0.000789141	0.005541584	yes	down	0.69	0.46	0.65	0.58	1.79	1.5	0.83	0.91	12.86	0.644	3.578
ENSMUSG00000001403	Ube2c	ubiquitin-conjugating enzyme E2C [Source:MGI Symbol;Acc:MGI:1915862]	0.449640288	-1.153156788	4.74942E-10	2.08811E-08	yes	down	6.55	7.72	9.72	8.94	19.1	14.61	14.9	13.53	23.9	8.254	17.208
ENSMUSG00000105646	Gm30211	predicted gene, 30211 [Source:MGI Symbol;Acc:MGI:5589370]	0.449640288	-1.153156788	0.004902672	0.024557016	yes	down	140.74	172.39	89.33	57.78	290.72	190.9	317.84	179.08	187.61	117.592	233.23
ENSMUSG00000040133	Gpr176	G protein-coupled receptor 176 [Source:MGI Symbol;Acc:MGI:2685858]	0.449438202	-1.153805336	1.83527E-07	4.30538E-06	yes	down	1.55	1.74	1.13	0.91	3.51	2.3	2.31	2.41	3.47	1.334	2.8
ENSMUSG00000027469	Tpx2	TPX2, microtubule-associated [Source:MGI Symbol;Acc:MGI:1919369]	0.449236298	-1.154453593	4.49301E-08	1.24029E-06	yes	down	2.43	1.61	2.57	3.17	5.33	5.03	4.53	3.49	7.8	2.704	5.236
ENSMUSG00000012443	Kif11	kinesin family member 11 [Source:MGI Symbol;Acc:MGI:1098231]	0.449034576	-1.155101558	2.25335E-09	8.52904E-08	yes	down	1.94	1.34	1.94	1.65	4.59	3.68	3.53	2.56	5.14	1.88	3.9
ENSMUSG00000004098	Col5a3	collagen, type V, alpha 3 [Source:MGI Symbol;Acc:MGI:1858212]	0.448229494	-1.157690514	2.52526E-06	4.21161E-05	yes	down	0.86	0.46	0.7	1.03	0.94	2.31	1.97	1.54	1.86	0.824	1.724
ENSMUSG00000096506	CR974586.5	-	0.447227191	-1.160920188	0.005379482	0.026521939	yes	down	2	2.02	1.88	1.4	2.63	1.38	4.96	2.15	3.73	1.662	2.97
ENSMUSG00000030329	Pianp	PILR alpha associated neural protein [Source:MGI Symbol;Acc:MGI:2441908]	0.446827525	-1.162210036	5.19759E-14	5.14749E-12	yes	down	6.87	4.17	4.29	10.2	8.63	11.31	12.5	17.75	11.06	6.17	12.25
ENSMUSG00000047534	Mis18bp1	MIS18 binding protein 1 [Source:MGI Symbol;Acc:MGI:2145099]	0.446827525	-1.162210036	0.000178698	0.001617832	yes	down	0.58	0.3	0.65	0.44	1.44	1.83	0.6	0.8	2.07	0.52	1.348
ENSMUSG00000024056	Ndc80	NDC80 kinetochore complex component [Source:MGI Symbol;Acc:MGI:1914302]	0.446627959	-1.162854528	1.80551E-06	3.15644E-05	yes	down	1.21	1.04	1.3	1.66	4.04	2.83	2.29	2.23	3.9	1.474	3.058
ENSMUSG00000020493	Prr11	proline rich 11 [Source:MGI Symbol;Acc:MGI:2444496]	0.446229362	-1.164142649	3.01747E-06	4.93401E-05	yes	down	0.8	0.49	1.42	0.83	2.31	1.8	1.4	1.74	2.42	0.93	1.934
ENSMUSG00000095127	Ighv1-82	immunoglobulin heavy variable 1-82 [Source:MGI Symbol;Acc:MGI:4439671]	0.446229362	-1.164142649	0.00594415	0.028637152	yes	down	1597.85	2229.38	879.78	1041.15	3518.93	3614.34	1203.05	2944.31	2651.98	1285.288	2786.522
ENSMUSG00000062380	Tubb3	tubulin, beta 3 class III [Source:MGI Symbol;Acc:MGI:107813]	0.445434298	-1.166715445	0.000268816	0.00227623	yes	down	0.96	0.61	0.63	0.63	2.3	1.45	0.75	1.84	1.99	0.774	1.666

ENSMUSG00000048922	Cdca2	cell division cycle associated 2 [Source:MGI Symbol;Acc:MGI:1919787]	0.444839858	-1.168642036	0.000149986	0.001396166	yes	down	1.17	0.57	1.31	0.66	1.87	1.74	2.71	1.08	3.91	1.016	2.262
ENSMUSG00000104713	Gbp6	guanylate binding protein 6 [Source:MGI Symbol;Acc:MGI:2140937]	0.444247001	-1.170566057	1.72481E-07	4.07612E-06	yes	down	17.67	14.52	23.15	12.85	38.23	30.81	24.27	33.97	51.28	17.448	35.712
ENSMUSG00000030109	Slc6a12	solute carrier family 6 (neurotransmitter transporter, betaine/GABA), member 12 [Source:MGI Symbol;Acc:MGI:95628]	0.443852641	-1.171847314	6.30272E-06	9.49526E-05	yes	down	2.55	2.59	2.66	1.46	4.45	7.55	3.25	4.72	5.45	2.324	5.084
ENSMUSG00000107962	Gm43980	predicted gene, 43980 [Source:MGI Symbol;Acc:MGI:5690372]	0.443852641	-1.171847314	0.002676322	0.015030877	yes	down	2.01	1.11	1.37	2.11	4.95	5.05	1.58	1.38	2.69	1.484	3.13
ENSMUSG00000113146	Gm47406	predicted gene, 47406 [Source:MGI Symbol;Acc:MGI:6096338]	0.443852641	-1.171847314	0.004199586	0.021655032	yes	down	0.48	0.35	1.19	0.69	1.09	1.23	1.79	1.45	1.21	0.646	1.354
ENSMUSG00000038179	Slamf7	SLAM family member 7 [Source:MGI Symbol;Acc:MGI:1922595]	0.443262411	-1.173767068	2.66834E-27	2.69825E-24	yes	down	35.25	36.31	30.72	29.59	80.69	58.76	66.74	69.95	87.98	31.966	72.824
ENSMUSG00000079227	Ccr5	chemokine (C-C motif) receptor 5 [Source:MGI Symbol;Acc:MGI:107182]	0.443066017	-1.174406419	1.79419E-27	2.02775E-24	yes	down	9.03	6.77	12.99	10.4	18.76	19.28	17.73	18.55	24.44	9.772	19.752
ENSMUSG00000062545	Tlr12	toll-like receptor 12 [Source:MGI Symbol;Acc:MGI:3045221]	0.442673749	-1.175684271	3.53093E-07	7.59683E-06	yes	down	1.5	1.21	1.57	1.27	3.07	2.61	3.76	2.35	4.94	1.42	3.346
ENSMUSG00000023272	Creld2	cysteine-rich with EGF-like domains 2 [Source:MGI Symbol;Acc:MGI:1923987]	0.442477876	-1.176322773	3.89893E-25	2.497E-22	yes	down	42.02	51.03	40.75	40.06	107.24	82.95	90.71	86.74	91.79	43.216	91.886
ENSMUSG00000042029	Ncapg2	non-SMC condensin II complex, subunit G2 [Source:MGI Symbol;Acc:MGI:1923294]	0.442477876	-1.176322773	9.93621E-20	2.72721E-17	yes	down	4.84	2.43	3.26	2.91	7.67	7.46	5.68	5.12	8.77	3.452	6.94
ENSMUSG00000096410	Ighv1-19	immunoglobulin heavy variable V1-19 [Source:MGI Symbol;Acc:MGI:4439779]	0.442282176	-1.176960992	7.59015E-05	0.000788268	yes	down	1698.36	1624.31	813.46	1780.23	5264.26	2310.61	2533.22	2817.98	3599.98	1549.896	3305.21
ENSMUSG00000097213	Gm26745	predicted gene, 26745 [Source:MGI Symbol;Acc:MGI:5477239]	0.441891295	-1.178236585	0.00360898	0.019138651	yes	down	3.84	2.98	8.17	4.09	14.3	5.68	11	9.37	8.43	4.632	9.756
ENSMUSG00000036887	C1qa	complement component 1, q subcomponent, alpha polypeptide [Source:MGI Symbol;Acc:MGI:88223]	0.441111601	-1.180784391	7.03515E-22	2.70333E-19	yes	down	156.59	133.12	134.03	118.76	334.08	244.31	262.93	261.62	361.65	137.838	292.918
ENSMUSG00000034438	Gbp8	guanylate-binding protein 8 [Source:MGI Symbol;Acc:MGI:1923324]	0.439367311	-1.186500558	4.05093E-09	1.44131E-07	yes	down	7.12	3.74	7.93	5.55	17.26	9.17	11.86	11.06	17.02	6.26	13.274
ENSMUSG00000030677	Kif22	kinesin family member 22 [Source:MGI Symbol;Acc:MGI:109233]	0.438981563	-1.187767747	5.22203E-07	1.06962E-05	yes	down	1.94	1.69	2.16	1.84	5.72	4.67	3.62	2.77	7.2	2.024	4.796
ENSMUSG00000020897	Aurkb	aurora kinase B [Source:MGI Symbol;Acc:MGI:107168]	0.438981563	-1.187767747	5.30343E-06	8.13206E-05	yes	down	2.29	1.57	2.49	2.12	6.07	3.91	3.19	3.02	7.88	2.538	4.814
ENSMUSG00000117239	AC166110.3	G protein-coupled receptor 31, D17Leh66b region (Gpr31b) pseudogene	0.438212095	-1.190298792	0.000494902	0.003782219	yes	down	0.48	0.3	0.19	0.28	0.88	0.38	0.77	0.53	0.99	0.326	0.71
ENSMUSG00000066363	Serpina3f	serine (or cysteine) peptidase inhibitor, clade A, member 3F [Source:MGI Symbol;Acc:MGI:2182838]	0.438212095	-1.190298792	0.001397722	0.008871634	yes	down	9.33	4.84	7.62	3.81	16.5	9.47	7.56	10.03	21.65	6.092	13.042
ENSMUSG00000037872	Ackr1	atypical chemokine receptor 1 (Duffy blood group) [Source:MGI Symbol;Acc:MGI:1097689]	0.438212095	-1.190298792	0.001428137	0.009021202	yes	down	1.93	1.51	0.83	0.93	2.89	2.57	1.52	2.98	2.53	1.15	2.498
ENSMUSG00000030144	Clec4d	C-type lectin domain family 4, member d [Source:MGI Symbol;Acc:MGI:1298389]	0.438020149	-1.19093086	5.9683E-07	1.20387E-05	yes	down	72.41	59.84	66.46	33.09	162.45	103.66	93.75	121.8	107.49	56.658	117.83
ENSMUSG00000028111	Ctsk	cathepsin K [Source:MGI Symbol;Acc:MGI:107823]	0.437636761	-1.192194165	4.66106E-22	1.86569E-19	yes	down	129.91	100.44	145.67	154.42	220	338.03	351.41	317.11	296.22	141.08	304.554
ENSMUSG00000036596	Cpz	carboxypeptidase Z [Source:MGI Symbol;Acc:MGI:88487]	0.437636761	-1.192194165	1.67212E-05	0.000219294	yes	down	0.61	1.41	0.65	1.58	1.75	2.38	2.45	2.29	2.65	1.072	2.304
ENSMUSG00000100658	F730311O21Rik	RIKEN cDNA F730311O21 gene [Source:MGI Symbol;Acc:MGI:3643355]	0.435919791	-1.197865391	0.001021129	0.006854981	yes	down	0.32	0.2	0.31	0.42	1.22	0.7	0.83	0.3	0.39	0.322	0.688
ENSMUSG00000028678	Kif2c	kinesin family member 2C [Source:MGI Symbol;Acc:MGI:1921054]	0.43554007	-1.199122642	7.84019E-06	0.000115163	yes	down	1.22	0.54	1.45	1.27	2.53	2.2	1.99	1.42	3.29	1.128	2.286
ENSMUSG00000103928	Gm37893	predicted gene, 37893 [Source:MGI Symbol;Acc:MGI:5611121]	0.434027778	-1.204140717	0.001122004	0.007400299	yes	down	0.88	0.41	0.46	0.67	1.69	2.21	0.97	0.73	0.77	0.59	1.274
ENSMUSG00000030117	Gdf3	growth differentiation factor 3 [Source:MGI Symbol;Acc:MGI:95686]	0.433651344	-1.205392513	0.001936437	0.011529213	yes	down	0.63	0.2	0.36	0.54	1.26	0.55	1.11	0.64	1.08	0.434	0.928
ENSMUSG00000048216	Gpr85	G protein-coupled receptor 85 [Source:MGI Symbol;Acc:MGI:1927851]	0.433087917	-1.207268173	0.000173396	0.001574409	yes	down	0.56	0.44	0.65	0.63	0.82	1.96	0.86	1.34	0.92	0.602	1.18
ENSMUSG00000045328	Cenpe	centromere protein E [Source:MGI Symbol;Acc:MGI:1098230]	0.432713111	-1.20851726	2.10988E-10	1.00091E-08	yes	down	0.65	0.6	0.83	1.02	2.03	2.91	2.9	1.06	1.69	0.78	2.118
ENSMUSG00000089936	Gm16199	predicted gene 16199 [Source:MGI Symbol;Acc:MGI:3801797]	0.432713111	-1.20851726	0.007827985	0.035497358	yes	down	19.5	4.23	23.5	21.53	25.15	27.48	44.74	66.03	30.04	18.386	38.688

ENSMUSG00000000562	Adora3	adenosine A3 receptor [Source:MGI Symbol;Acc:MGI:104847]	0.432338954	-1.209765266	2.57116E-06	4.28112E-05	yes	down	0.62	0.84	1.3	0.93	2.91	2.18	1.24	2.05	2.64	0.95	2.204
ENSMUSG00000099137	Gm10603	predicted gene 10603 [Source:MGI Symbol;Acc:MGI:3642592]	0.432338954	-1.209765266	0.012069842	0.049977988	yes	down	0.17	0.11	0.5	0.08	0.41	0.39	0.38	0.32	0.14	0.188	0.328
ENSMUSG00000032586	Traip	TRAF-interacting protein [Source:MGI Symbol;Acc:MGI:1096377]	0.431965443	-1.211012193	0.000864472	0.005968059	yes	down	0.77	0.3	1.05	0.25	1.66	0.72	0.69	0.67	1.13	0.594	0.974
ENSMUSG00000110018	5430437J10Rik	RIKEN cDNA 5430437J10 gene [Source:MGI Symbol;Acc:MGI:1918682]	0.431406385	-1.212880566	0.011865714	0.049273011	yes	down	0.71	0.58	0.27	0.49	0.49	1.94	1.04	1.08	1.38	0.492	1.186
ENSMUSG00000024791	Cdca5	cell division cycle associated 5 [Source:MGI Symbol;Acc:MGI:1915099]	0.43047783	-1.215989154	0.000399168	0.003171715	yes	down	0.8	0.98	0.82	0.69	2.86	1.74	1.12	1.11	3.09	0.858	1.984
ENSMUSG00000109713	Pvrig	poliovirus receptor related immunoglobulin domain containing [Source:MGI Symbol;Acc:MGI:5596028]	0.430292599	-1.216610069	0.004938394	0.024695828	yes	down	1.52	0.52	0.54	0.38	2.06	2.61	1.77	1.74	2.28	0.962	2.092
ENSMUSG00000094335	Igkv1-117	immunoglobulin kappa variable 1-117 [Source:MGI Symbol;Acc:MGI:4439721]	0.429368828	-1.21971064	5.62513E-05	0.00061198	yes	down	1713.99	1232.47	1389.08	756.01	3297.19	3702.42	2749.5	1481.68	1956.56	1216.084	2637.47
ENSMUSG00000033182	Kbtbd12	kelch repeat and BTB (POZ) domain containing 12 [Source:MGI Symbol;Acc:MGI:1918481]	0.428632662	-1.222186307	0.007804042	0.035429835	yes	down	1.22	3.99	0.42	0.58	0.62	0.89	0.67	0.89	0.61	1.292	0.736
ENSMUSG00000038943	Prc1	protein regulator of cytokinesis 1 [Source:MGI Symbol;Acc:MGI:1858961]	0.428265525	-1.22342255	2.51868E-09	9.43303E-08	yes	down	3.21	2.33	4.29	4.82	8.79	7.47	8.2	6.67	11.76	3.904	8.578
ENSMUSG00000027386	Fbln7	fibulin 7 [Source:MGI Symbol;Acc:MGI:1917620]	0.427899016	-1.224657734	0.010841334	0.045869752	yes	down	0.57	0.25	0.16	0.23	0.17	0.59	0.97	0.54	1.01	0.3	0.656
ENSMUSG00000036905	C1qb	complement component 1, q subcomponent, beta polypeptide [Source:MGI Symbol;Acc:MGI:88224]	0.427350427	-1.22650853	2.68613E-22	1.2002E-19	yes	down	193.01	143.59	158.32	135.66	368.2	297.41	314.16	301.89	440.75	156.994	344.482
ENSMUSG00000074802	Gas2l3	growth arrest-specific 2 like 3 [Source:MGI Symbol;Acc:MGI:1918780]	0.426985482	-1.227741076	2.44259E-14	2.64147E-12	yes	down	1.13	0.9	1.3	1.25	2.97	2.42	2.09	2.11	2.82	1.188	2.482
ENSMUSG00000021700	Rab3c	RAB3C, member RAS oncogene family [Source:MGI Symbol;Acc:MGI:1914545]	0.426985482	-1.227741076	7.25381E-12	4.82241E-10	yes	down	1.36	0.74	1.07	1.66	2.49	1.81	1.89	2.27	2.33	1.166	2.158
ENSMUSG00000022838	Eaf2	ELL associated factor 2 [Source:MGI Symbol;Acc:MGI:2146616]	0.42625746	-1.230203013	6.41985E-05	0.000684461	yes	down	6.98	12.46	5.45	8.26	37.24	10.66	12.71	8.43	12.39	7.358	16.286
ENSMUSG00000029675	Eln	elastin [Source:MGI Symbol;Acc:MGI:95317]	0.426075841	-1.230817842	6.43067E-05	0.000684881	yes	down	114.85	58.4	106.24	119.82	172.2	380.59	247.85	117.49	291.69	110.284	241.964
ENSMUSG00000026628	Atf3	activating transcription factor 3 [Source:MGI Symbol;Acc:MGI:109384]	0.425894378	-1.231432408	3.8677E-14	3.95266E-12	yes	down	21.08	7.27	11.77	14.3	29.65	26.59	32.98	29.84	30.56	13.896	29.924
ENSMUSG00000076612	Ighg2c	immunoglobulin heavy constant gamma 2C [Source:MGI Symbol;Acc:MGI:2686979]	0.425713069	-1.232046713	0.010077282	0.043285227	yes	down	2697.02	2175.51	594.02	773.37	4703.31	3148.1	2135.17	4010.23	2464.97	1465.768	3292.356
ENSMUSG00000093955	Ighv1-34	immunoglobulin heavy variable 1-34 [Source:MGI Symbol;Acc:MGI:4439659]	0.425531915	-1.232660757	0.000361092	0.002922352	yes	down	1303.32	1589.45	505.5	872.86	3925.49	2216.32	2169.01	2071.9	2284.88	1129.02	2533.52
ENSMUSG00000065601	Mir146	microRNA 146 [Source:MGI Symbol;Acc:MGI:2676831]	0.425350915	-1.233274539	0.000130109	0.001241201	yes	down	1.44	0.3	1.13	1.29	3.87	2.43	2.11	1.75	2.31	1.152	2.494
ENSMUSG00000003779	Kif20a	kinesin family member 20A [Source:MGI Symbol;Acc:MGI:1201682]	0.424808836	-1.23511432	1.99363E-06	3.4353E-05	yes	down	1.9	1.5	1.96	1.58	4.55	3.97	2.85	3	6.42	2.046	4.158
ENSMUSG00000096459	Ighv9-3	immunoglobulin heavy variable V9-3 [Source:MGI Symbol;Acc:MGI:3642720]	0.423190859	-1.240619629	0.00026714	0.002266035	yes	down	274.56	458.38	214.54	366.85	977.6	512.82	414.96	637.88	997.97	315.598	708.246
ENSMUSG00000022769	Sdf2l1	stromal cell-derived factor 2-like 1 [Source:MGI Symbol;Acc:MGI:2149842]	0.422654269	-1.242450074	3.42024E-26	2.98696E-23	yes	down	31.89	31.91	28.24	26.98	72.35	64.72	77.7	55.95	72.25	30.906	68.594
ENSMUSG00000041431	Ccnb1	cyclin B1 [Source:MGI Symbol;Acc:MGI:88302]	0.422475708	-1.243059706	6.24955E-06	9.44709E-05	yes	down	1.24	1.17	2.86	2.56	5.73	2.87	3.54	3.24	8.18	2.194	4.712
ENSMUSG00000105606	Igkv2-109	immunoglobulin kappa variable 2-109 [Source:MGI Symbol;Acc:MGI:3642626]	0.42158516	-1.24610401	0.00128959	0.008311607	yes	down	310.35	170.53	334.39	209.49	749.73	202.99	436.49	730.73	855.93	270.3	595.174
ENSMUSG00000105504	Gbp5	guanylate binding protein 5 [Source:MGI Symbol;Acc:MGI:2429943]	0.421052632	-1.247927513	1.98781E-10	9.50045E-09	yes	down	8.95	6.9	10.8	9.04	24.07	12.66	17.08	15.73	30.62	9.032	20.032
ENSMUSG00000026822	Lcn2	lipocalin 2 [Source:MGI Symbol;Acc:MGI:96757]	0.420168067	-1.250961574	2.77449E-28	3.55375E-25	yes	down	427.87	356.86	425.72	376.11	851.9	848.84	1022.01	765.53	1158.36	417.648	929.328
ENSMUSG00000108825	Gm45838	predicted gene 45838 [Source:MGI Symbol;Acc:MGI:5804953]	0.419815281	-1.252173413	0.001214394	0.007906526	yes	down	0.6	0.25	0.37	0.48	1.43	1.23	0.84	0.39	0.79	0.418	0.936
ENSMUSG00000002847	Pla1a	phospholipase A1 member A [Source:MGI Symbol;Acc:MGI:1934677]	0.419463087	-1.253384236	6.95177E-07	1.37695E-05	yes	down	4.61	3.83	2.53	5.82	5.18	6.86	4.51	7.32	11.43	4.018	7.06
ENSMUSG00000036896	C1qc	complement component 1, q subcomponent, C chain [Source:MGI Symbol;Acc:MGI:88225]	0.419287212	-1.253989266	3.57896E-22	1.52806E-19	yes	down	117.5	101.88	96.84	81.79	236.42	198.45	209.65	193.01	296.18	101.06	226.742
ENSMUSG00000035455	Fignl1	fidgetin-like 1 [Source:MGI Symbol;Acc:MGI:1890648]	0.419111484	-1.254594043	8.4008E-06	0.000121631	yes	down	0.89	0.66	1.02	0.66	2.14	1.16	1.57	1.26	2.78	0.8	1.782

ENSMUSG00000019942	Cdk1	cyclin-dependent kinase 1 [Source:MGI Symbol;Acc:MGI:88351]	0.417188152	-1.261229909	3.57185E-08	1.00856E-06	yes	down	8.29	5.92	9.09	7.55	20.3	17.62	15.4	16.53	29.93	8.88	19.956
ENSMUSG00000024989	Cep55	centrosomal protein 55 [Source:MGI Symbol;Acc:MGI:1921357]	0.416666667	-1.263034406	9.83154E-07	1.85553E-05	yes	down	1.45	0.91	1.96	2.11	4.88	2.57	3.37	2.39	4.98	1.704	3.638
ENSMUSG00000031262	Cenpi	centromere protein I [Source:MGI Symbol;Acc:MGI:2147897]	0.416666667	-1.263034406	1.63236E-05	0.000214665	yes	down	0.72	0.56	0.77	0.66	1.64	1.1	0.79	0.94	1.64	0.748	1.222
ENSMUSG00000022385	Gtse1	G two S phase expressed protein 1 [Source:MGI Symbol;Acc:MGI:1352755]	0.415627598	-1.266636643	3.32872E-05	0.000389684	yes	down	0.69	0.55	1.21	0.61	1.81	1.93	1.46	1.44	3.2	0.874	1.968
ENSMUSG00000055978	Fut2	fucosyltransferase 2 [Source:MGI Symbol;Acc:MGI:109374]	0.415282392	-1.267835392	0.001279185	0.008258395	yes	down	0.27	0.21	0.21	0.47	0.29	0.89	1.27	0.64	1.19	0.274	0.856
ENSMUSG00000068101	Cenpm	centromere protein M [Source:MGI Symbol;Acc:MGI:1913820]	0.414421881	-1.270827916	0.002167839	0.012698381	yes	down	0.79	0.86	1.44	0.89	7.59	2.05	2.34	1.08	6.28	0.868	3.868
ENSMUSG00000028327	Stra6l	STRA6-like [Source:MGI Symbol;Acc:MGI:1921402]	0.414250207	-1.271425676	1.04996E-18	2.37328E-16	yes	down	4.43	2.42	3.22	2.66	7.96	7.21	7.07	7.63	7.19	3.29	7.412
ENSMUSG00000086196	Gm13571	predicted gene 13571 [Source:MGI Symbol;Acc:MGI:3652223]	0.414078675	-1.272023189	0.000670643	0.004884405	yes	down	0.42	0.46	0.87	0.83	1.02	1.68	1.13	1.31	1.31	0.572	1.29
ENSMUSG00000115230	AU022793	expressed sequence AU022793 [Source:MGI Symbol;Acc:MGI:2146144]	0.413907285	-1.272620455	3.11656E-07	6.83543E-06	yes	down	2.71	1.21	3.34	2.49	3.47	6.51	4.05	3.32	6.46	2.43	4.762
ENSMUSG00000029414	Kntc1	kinetochore associated 1 [Source:MGI Symbol;Acc:MGI:2673709]	0.413052458	-1.275603079	1.93334E-07	4.50793E-06	yes	down	0.61	0.25	0.36	0.57	2.63	1.53	3.87	1.15	1.63	0.496	2.162
ENSMUSG00000022322	Shcbp1	Shc SH2-domain binding protein 1 [Source:MGI Symbol;Acc:MGI:1338802]	0.413052458	-1.275603079	0.000207609	0.001831398	yes	down	1.3	0.25	1.34	2.61	1.71	1.85	0.9	1.18	1.82	1.276	1.492
ENSMUSG00000039055	Eme1	essential meiotic structure-specific endonuclease 1 [Source:MGI Symbol;Acc:MGI:3576783]	0.412881916	-1.276198865	0.002975235	0.016351027	yes	down	0.19	0.48	0.42	0.25	0.87	0.67	0.56	0.34	1.01	0.392	0.69
ENSMUSG00000006179	Prss16	protease, serine 16 (thymus) [Source:MGI Symbol;Acc:MGI:1859181]	0.412201154	-1.27857955	0.00346899	0.018566005	yes	down	0.67	0.22	0.17	0.27	0.69	1.03	0.59	0.56	0.44	0.314	0.662
ENSMUSG00000071005	Ccl19	chemokine (C-C motif) ligand 19 [Source:MGI Symbol;Acc:MGI:1346316]	0.412031314	-1.279174108	0.000169257	0.001544532	yes	down	7.64	6.85	3.64	4.81	16.42	8.95	11.38	6.41	22.08	6.204	13.048
ENSMUSG00000025877	Hk3	hexokinase 3 [Source:MGI Symbol;Acc:MGI:2670962]	0.411861614	-1.279768422	1.85633E-10	8.9164E-09	yes	down	10.5	5.08	3.97	4.66	11.66	13.04	8.49	10.12	18.54	6.134	12.37
ENSMUSG00000061356	Nuggc	nuclear GTPase, germinal center associated [Source:MGI Symbol;Acc:MGI:2685446]	0.411692054	-1.28036249	0.00015234	0.001415335	yes	down	1.05	0.78	0.44	0.55	2.25	0.99	1.36	1.02	1.99	0.68	1.522
ENSMUSG00000117764	AC157784.1	novel transcript, sense intronic to Ccbe1	0.410677618	-1.283921772	2.4173E-06	4.06331E-05	yes	down	1.74	1.66	0.71	1.64	4	2.56	2.44	3.72	2.39	1.308	3.022
ENSMUSG00000100750	Gm29084	predicted gene 29084 [Source:MGI Symbol;Acc:MGI:5579790]	0.410340583	-1.285106251	1.51964E-07	3.64505E-06	yes	down	3.62	2.45	2.45	2.26	8.05	7.09	6.09	3.36	5.44	2.63	6.006
ENSMUSG00000026077	Npas2	neuronal PAS domain protein 2 [Source:MGI Symbol;Acc:MGI:109232]	0.409668169	-1.287472295	1.82262E-05	0.000234391	yes	down	0.55	1.28	0.73	0.96	1.51	3.23	1.73	1.69	0.81	0.84	1.794
ENSMUSG00000095335	Igkv3-5	immunoglobulin kappa chain variable 3-5 [Source:MGI Symbol;Acc:MGI:1330854]	0.407830343	-1.293958979	0.011066204	0.046595437	yes	down	427.58	1206.11	196.41	820.61	1776.58	1261.46	1719.8	950.91	1310.04	597.262	1403.758
ENSMUSG00000068794	Col28a1	collagen, type XXVIII, alpha 1 [Source:MGI Symbol;Acc:MGI:2685312]	0.407664085	-1.294547234	0.006761045	0.031682914	yes	down	3.37	1.13	1.55	2.38	1.16	5.85	3.42	5.59	6.72	1.972	4.548
ENSMUSG00000095589	Ighv1-55	immunoglobulin heavy variable 1-55 [Source:MGI Symbol;Acc:MGI:4439716]	0.407331976	-1.295723025	0.000158004	0.0014623	yes	down	794.06	1366.14	500.43	658.24	2070.1	1543.95	1996.84	1239.32	2320.56	781.098	1834.154
ENSMUSG00000029814	Igf2bp3	insulin-like growth factor 2 mRNA binding protein 3 [Source:MGI Symbol;Acc:MGI:1890359]	0.406834825	-1.297484916	0.001890909	0.011310722	yes	down	2.01	0.08	0.14	0.66	3.16	0.45	0.73	1.94	0.85	0.744	1.426
ENSMUSG00000001228	Uhrf1	ubiquitin-like, containing PHD and RING finger domains, 1 [Source:MGI Symbol;Acc:MGI:1338889]	0.406173842	-1.299830762	3.86092E-08	1.07976E-06	yes	down	2.55	2.49	2.3	3.17	5.49	2.97	3.71	3.54	8.45	2.576	4.832
ENSMUSG00000102813	Gm37795	predicted gene, 37795 [Source:MGI Symbol;Acc:MGI:5611023]	0.405515004	-1.3021728	0.000939418	0.006408217	yes	down	0.72	0.3	0.31	0.44	1.54	1.66	0.9	0.45	1.11	0.492	1.132
ENSMUSG00000004951	Hspb1	heat shock protein 1 [Source:MGI Symbol;Acc:MGI:96240]	0.405350628	-1.302757716	1.27867E-05	0.000174358	yes	down	55.51	58.45	43.07	46.41	216.48	85.23	129.59	107.36	85	54.104	124.732
ENSMUSG00000056531	Ccdc18	coiled-coil domain containing 18 [Source:MGI Symbol;Acc:MGI:1922974]	0.405350628	-1.302757716	0.000854446	0.005913714	yes	down	0.16	0.05	0.17	0.13	0.42	0.3	0.28	0.18	0.32	0.122	0.3
ENSMUSG00000028718	Stil	Scl/Tal1 interrupting locus [Source:MGI Symbol;Acc:MGI:107477]	0.404367165	-1.30626224	8.90244E-05	0.000895511	yes	down	0.14	0.32	0.27	0.4	0.59	1.09	0.72	0.43	0.86	0.35	0.738
ENSMUSG00000044708	Kcnj10	potassium inwardly-rectifying channel, subfamily J, member 10 [Source:MGI Symbol;Acc:MGI:1194504]	0.404203719	-1.3068455	9.59464E-12	6.18597E-10	yes	down	0.83	0.56	0.69	1.02	2.1	1.71	1.9	1.3	2.15	0.794	1.832
ENSMUSG00000025930	Msc	musculin [Source:MGI Symbol;Acc:MGI:1333884]	0.404203719	-1.3068455	0.000420561	0.003312929	yes	down	0.46	0.86	1.18	0.64	3.83	2.15	2.3	1.48	2.92	1.014	2.536

ENSMUSG00000039521	Foxp3	forkhead box P3 [Source:MGI Symbol;Acc:MGI:1891436]	0.40371417	-1.308593869	1.7557E-11	1.05975E-09	yes	down	1.15	0.82	1.22	1.14	2.43	2.12	3.82	2.27	2.68	1.168	2.664
ENSMUSG00000068129	Cst7	cystatin F (leukocystatin) [Source:MGI Symbol;Acc:MGI:1298217]	0.403551251	-1.309176187	0.000163044	0.001501713	yes	down	3.71	4.47	4.45	3.38	13.25	5.2	7.73	3.96	17.6	4.122	9.548
ENSMUSG00000076514	Igkv17-121	immunoglobulin kappa variable 17-121 [Source:MGI Symbol;Acc:MGI:3647671]	0.402252615	-1.313826296	0.004887198	0.024523705	yes	down	454.43	589.48	471.02	177.48	2007.52	854.03	520.99	651.82	715.42	400.218	949.956
ENSMUSG00000021965	Ska3	spindle and kinetochore associated complex subunit 3 [Source:MGI Symbol;Acc:MGI:3041235]	0.401767778	-1.31556623	2.60797E-05	0.000316332	yes	down	0.88	0.35	0.92	0.4	1.64	1.14	1.06	1.05	1.42	0.648	1.262
ENSMUSG00000094694	Ighv1-9	immunoglobulin heavy variable V1-9 [Source:MGI Symbol;Acc:MGI:4439621]	0.401123145	-1.317882883	0.001059333	0.007066999	yes	down	678.14	401.11	166.1	321.83	1498.79	674.43	1136.52	694.05	813.56	407.946	963.47
ENSMUSG00000020330	Hmmr	hyaluronan mediated motility receptor (RHAMM) [Source:MGI Symbol;Acc:MGI:104667]	0.400160064	-1.321350901	1.84305E-11	1.09971E-09	yes	down	0.74	0.83	1.3	1.1	2.33	2.31	1.92	1.87	2.92	0.962	2.27
ENSMUSG00000021322	Aoah	acyloxyacyl hydrolase [Source:MGI Symbol;Acc:MGI:1350928]	0.399680256	-1.32308179	7.80696E-14	7.57551E-12	yes	down	5.47	6.94	7.04	4.39	15.32	12.68	10.43	12.25	18.12	5.848	13.76
ENSMUSG00000032315	Cyp1a1	cytochrome P450, family 1, subfamily a, polypeptide 1 [Source:MGI Symbol;Acc:MGI:88588]	0.399042298	-1.325386415	0.006555562	0.030976886	yes	down	23.32	32.62	19.79	10.08	56.25	25.42	84.14	19.85	77.01	22.244	52.534
ENSMUSG00000071552	Tigit	T cell immunoreceptor with Ig and ITIM domains [Source:MGI Symbol;Acc:MGI:3642260]	0.398406375	-1.327687364	2.2712E-10	1.07215E-08	yes	down	6.5	8.69	5.77	5.69	21.69	12.29	13.4	10.86	15.14	6.782	14.676
ENSMUSG00000023913	Pla2g7	phospholipase A2, group VII (platelet-activating factor acetylhydrolase, plasma) [Source:MGI Symbol;Acc:MGI:1351327]	0.398089172	-1.328836464	2.82218E-08	8.20312E-07	yes	down	63.9	39.57	44.65	32.48	142.31	81.53	79.42	92.48	117.1	44.534	102.568
ENSMUSG00000076522	Igkv16-104	immunoglobulin kappa variable 16-104 [Source:MGI Symbol;Acc:MGI:2685913]	0.398089172	-1.328836464	0.006350071	0.030206465	yes	down	340.96	152.16	388.15	253.13	980.35	628.42	488.86	500.77	238.78	242.35	567.436
ENSMUSG00000076596	Igkv3-10	immunoglobulin kappa variable 3-10 [Source:MGI Symbol;Acc:MGI:1330821]	0.39745628	-1.331131922	0.003599497	0.019093633	yes	down	158.82	199.33	73.42	66.34	345.33	212.57	471.36	143.83	282.52	121.254	291.122
ENSMUSG00000029641	Rasl11a	RAS-like, family 11, member A [Source:MGI Symbol;Acc:MGI:1916145]	0.39698293	-1.332851122	9.62158E-23	4.62149E-20	yes	down	13.92	7.72	10.86	12.66	25.79	30.6	23.2	28.49	26.42	11.398	26.9
ENSMUSG00000096336	Igkv1-135	immunoglobulin kappa variable 1-135 [Source:MGI Symbol;Acc:MGI:3819952]	0.39698293	-1.332851122	0.009846088	0.042520318	yes	down	494.65	921.83	327.33	544.84	1300.99	568.12	1497.95	595.27	2129.08	507.574	1218.282
ENSMUSG00000027715	Ccna2	cyclin A2 [Source:MGI Symbol;Acc:MGI:108069]	0.396825397	-1.333423734	2.06013E-12	1.51807E-10	yes	down	2.62	2.62	3.31	3.22	9.69	6.48	6.58	4.91	9.11	3.124	7.354
ENSMUSG00000027323	Rad51	RAD51 recombinase [Source:MGI Symbol;Acc:MGI:97890]	0.396353547	-1.335140206	2.7694E-07	6.17985E-06	yes	down	1.44	0.45	0.9	0.84	1.44	1.64	1.8	2.64	6.31	1.154	2.766
ENSMUSG00000062148	Ear6	eosinophil-associated, ribonuclease A family, member 6 [Source:MGI Symbol;Acc:MGI:1890463]	0.394788788	-1.340847077	0.000302571	0.00251767	yes	down	2.83	0.97	1.76	3.8	7.86	8.46	4.5	3.27	6.3	2.562	6.078
ENSMUSG00000103983	Gm20045	predicted gene, 20045 [Source:MGI Symbol;Acc:MGI:5012230]	0.394788788	-1.340847077	0.000343357	0.002800047	yes	down	0.35	0.07	0.25	0.21	0.8	0.55	0.4	0.32	0.5	0.216	0.514
ENSMUSG00000020914	Top2a	topoisomerase (DNA) II alpha [Source:MGI Symbol;Acc:MGI:98790]	0.394011032	-1.343692069	4.25258E-17	7.36079E-15	yes	down	7.53	5.28	8.29	7.16	20.67	16.42	16.93	11.84	26.99	7.51	18.57
ENSMUSG00000092528	Nlrp1c-ps	NLR family, pyrin domain containing 1C, pseudogene [Source:MGI Symbol;Acc:MGI:3582962]	0.393700787	-1.344828497	0.000713701	0.00512228	yes	down	0.65	0.69	2.29	0.16	3.83	4.89	1.08	1.84	2.37	0.844	2.802
ENSMUSG00000040204	Pclaf	PCNA clamp associated factor [Source:MGI Symbol;Acc:MGI:1915276]	0.392772977	-1.348232419	1.19179E-07	2.94318E-06	yes	down	3.28	4.08	3.02	3.06	10.73	6.29	7.72	4.01	13.01	3.518	8.352
ENSMUSG00000076564	Igkv12-46	immunoglobulin kappa variable 12-46 [Source:MGI Symbol;Acc:MGI:4439773]	0.39231071	-1.349931373	0.000818	0.005704622	yes	down	439.06	841.61	960.88	1377.4	1579.64	2823.27	2941.08	1347.46	1056.18	824.23	1949.526
ENSMUSG00000035373	Ccl7	chemokine (C-C motif) ligand 7 [Source:MGI Symbol;Acc:MGI:99512]	0.39231071	-1.349931373	0.004483315	0.02285432	yes	down	1.53	0.99	1.31	1.37	3.29	1.44	3.8	0.96	4.62	1.188	2.822
ENSMUSG00000054072	Iigp1	interferon inducible GTPase 1 [Source:MGI Symbol;Acc:MGI:1926259]	0.391389432	-1.353323291	1.65787E-09	6.46099E-08	yes	down	25.47	16.62	31.69	23.79	67.07	43.67	47.21	46.49	80.75	24.094	57.038
ENSMUSG00000028217	Cdh17	cadherin 17 [Source:MGI Symbol;Acc:MGI:1095414]	0.391083301	-1.354452161	0.001140438	0.007503845	yes	down	0.25	0.27	0.42	0.2	1.23	0.43	0.36	0.5	0.64	0.264	0.632
ENSMUSG00000024672	Ms4a7	membrane-spanning 4-domains, subfamily A, member 7 [Source:MGI Symbol;Acc:MGI:1918846]	0.390472472	-1.356707253	9.38207E-37	2.57511E-33	yes	down	31.01	26.71	35.02	25.78	75.29	72.41	66.27	73.17	89.25	30.628	75.278
ENSMUSG00000117964	AC119228.1	novel transcript, antisense to Cd274	0.390320062	-1.357270476	1.75443E-05	0.000227443	yes	down	0.88	1.16	0.39	1.33	1.99	2.87	3.01	0.54	1.93	1.1	2.068
ENSMUSG00000017716	Birc5	baculoviral IAP repeat-containing 5 [Source:MGI Symbol;Acc:MGI:1203517]	0.389559797	-1.360083296	5.58466E-11	2.96404E-09	yes	down	3.14	4.01	5.29	3.96	10.3	8.26	7.93	9.95	13.26	4.348	9.94
ENSMUSG00000090877	Hspa1b	heat shock protein 1B [Source:MGI Symbol;Acc:MGI:99517]	0.3894081	-1.360645202	0.002555402	0.014491423	yes	down	13.56	6.31	5.7	12.06	29.58	34.13	37.98	44.03	20.74	13.96	33.292

ENSMUSG00000117001	AC124237.1	Friend virus susceptibility 1 (Fv1) pseudogene	0.38925652	-1.36120689	0.011725263	0.048761359	yes	down	1.49	0.13	0.92	1.12	3.91	1.46	1.16	0.9	2.35	0.814	1.956
ENSMUSG00000015880	Ncapg	non-SMC condensin I complex, subunit G [Source:MGI Symbol;Acc:MGI:1930197]	0.387446726	-1.367930141	6.50686E-07	1.29685E-05	yes	down	1.04	0.65	0.92	0.69	2.67	1.44	1.78	1.36	3.23	0.9	2.096
ENSMUSG00000028873	Cdca8	cell division cycle associated 8 [Source:MGI Symbol;Acc:MGI:1196274]	0.386100386	-1.372952098	1.14416E-11	7.16049E-10	yes	down	2.49	5.13	3.63	2.7	9.53	7.33	5.86	5.2	9.21	3.404	7.426
ENSMUSG00000033576	Apol6	apolipoprotein L 6 [Source:MGI Symbol;Acc:MGI:1919189]	0.38595137	-1.373509016	0.000146987	0.00137625	yes	down	0.87	1.1	0.49	0.9	2.91	1.41	2.1	0.65	2.99	0.746	2.012
ENSMUSG00000095630	Igkv6-23	immunoglobulin kappa variable 6-23 [Source:MGI Symbol;Acc:MGI:3711980]	0.385059684	-1.376846014	0.00323791	0.017533815	yes	down	1975.25	3145.37	951.52	2503.18	5094.66	10102.2	4389.38	3068.63	2752.2	2036.738	5081.414
ENSMUSG00000021702	Thbs4	thrombospondin 4 [Source:MGI Symbol;Acc:MGI:1101779]	0.384319754	-1.379620962	0.002383209	0.013688666	yes	down	0.38	0.06	0.6	0.61	0.44	0.85	1.68	0.74	1.21	0.408	0.984
ENSMUSG00000016498	Pdcd1lg2	programmed cell death 1 ligand 2 [Source:MGI Symbol;Acc:MGI:1930125]	0.383288616	-1.383496944	0.000289132	0.002419468	yes	down	0.69	1.28	0.46	0.44	2.73	0.95	2	1.69	2.21	0.77	1.916
ENSMUSG00000026605	Cenpf	centromere protein F [Source:MGI Symbol;Acc:MGI:1313302]	0.382848392	-1.385154897	6.56242E-11	3.46384E-09	yes	down	1.34	1.07	0.94	0.69	3.26	2.36	1.35	2.08	2.06	0.968	2.222
ENSMUSG00000076556	Igkv4-57	immunoglobulin kappa variable 4-57 [Source:MGI Symbol;Acc:MGI:2685035]	0.382848392	-1.385154897	0.000437716	0.003429788	yes	down	626.87	612.27	227.2	401.01	1403.44	1188.23	640.45	770.65	1734.52	445.092	1147.458
ENSMUSG00000027326	Knl1	kinetochore scaffold 1 [Source:MGI Symbol;Acc:MGI:1923714]	0.382409178	-1.386810946	2.3231E-10	1.08863E-08	yes	down	0.43	0.4	0.64	0.51	1.43	0.87	0.72	1.27	1.48	0.508	1.154
ENSMUSG00000097855	A930007I19Rik	RIKEN cDNA A930007I19 gene [Source:MGI Symbol;Acc:MGI:1925029]	0.382262997	-1.387362541	7.62336E-12	4.99889E-10	yes	down	6.21	2	2.8	23.59	7.88	9.08	6.35	28.12	5.92	7.21	11.47
ENSMUSG00000033952	Aspm	abnormal spindle microtubule assembly [Source:MGI Symbol;Acc:MGI:1334448]	0.382262997	-1.387362541	6.91628E-08	1.80793E-06	yes	down	0.52	0.33	0.62	0.72	1.54	0.94	1.08	0.95	1.9	0.948	1.282
ENSMUSG00000038751	Ptk6	PTK6 protein tyrosine kinase 6 [Source:MGI Symbol;Acc:MGI:99683]	0.381242852	-1.391217808	0.007169269	0.033167146	yes	down	0.24	0.12	0.07	0.12	0.17	0.67	0.39	0.22	0.2	0.132	0.33
ENSMUSG00000028175	Depdc1a	DEP domain containing 1a [Source:MGI Symbol;Acc:MGI:1923381]	0.379506641	-1.397802962	0.000132512	0.001258507	yes	down	0.62	0.29	0.47	1.04	1.71	1.61	0.97	0.89	2.48	0.702	1.532
ENSMUSG00000108132	Gm44175	predicted gene, 44175 [Source:MGI Symbol;Acc:MGI:5690567]	0.379218809	-1.398897571	0.009417547	0.041094558	yes	down	0.12	0.06	0.08	0.1	0.39	0.36	0.1	0.2	0.09	0.094	0.228
ENSMUSG00000024598	Fbn2	fibrillin 2 [Source:MGI Symbol;Acc:MGI:95490]	0.377358491	-1.40599236	0.008914695	0.039279316	yes	down	0.01	0.03	0.05	0.09	0.29	0.11	0.25	0.05	0.06	0.046	0.152
ENSMUSG00000095519	Ighv1-66	immunoglobulin heavy variable 1-66 [Source:MGI Symbol;Acc:MGI:4439825]	0.377216145	-1.40653667	0.000674384	0.004904601	yes	down	525.48	552.75	249.05	301.58	1594.2	902.87	683.24	580.56	1849.55	445.878	1122.084
ENSMUSG00000045273	Cenph	centromere protein H [Source:MGI Symbol;Acc:MGI:1349448]	0.376789751	-1.408168371	0.002409645	0.013817356	yes	down	0.67	0.62	0.2	0.31	1.48	0.81	1.43	0.72	1.32	0.448	1.152
ENSMUSG00000091575	2010016I18Rik	RIKEN cDNA 2010016I18 gene [Source:MGI Symbol;Acc:MGI:1916456]	0.376647834	-1.408711861	1.02109E-05	0.000144464	yes	down	2.03	1.61	1.34	1.49	5.94	6.51	4.26	1.55	4.23	1.838	4.498
ENSMUSG00000096498	Ighv2-5	immunoglobulin heavy variable 2-5 [Source:MGI Symbol;Acc:MGI:4439517]	0.375798572	-1.41196851	0.011448259	0.047889265	yes	down	163.69	452.54	126.94	255.46	629.11	686.98	1322.42	238.38	529.03	229.678	681.184
ENSMUSG00000041202	Pla2g2d	phospholipase A2, group IID [Source:MGI Symbol;Acc:MGI:1341796]	0.3756574	-1.412510571	5.28409E-23	2.67166E-20	yes	down	20.12	13.12	11.75	11.86	38.34	33.94	36.92	37.03	32.24	14.196	35.694
ENSMUSG00000040552	C3ar1	complement component 3a receptor 1 [Source:MGI Symbol;Acc:MGI:1097680]	0.375516335	-1.413052429	1.91457E-24	1.14952E-21	yes	down	7.49	5.76	7.52	6.42	16.47	13.62	14.29	15.61	20.28	6.434	16.054
ENSMUSG00000095642	Ighv14-3	immunoglobulin heavy variable V14-3 [Source:MGI Symbol;Acc:MGI:4439764]	0.375516335	-1.413052429	0.001602322	0.009867119	yes	down	873.45	676.33	411.4	259.2	1180.85	1681.49	1416.03	843.06	1040.66	481.084	1232.418
ENSMUSG00000070524	Fcrlb	Fc receptor-like B [Source:MGI Symbol;Acc:MGI:3576487]	0.374111485	-1.418459838	0.007648618	0.034938875	yes	down	1.35	0.55	0.05	0.65	2.08	1.38	1.87	0.74	1.91	0.634	1.596
ENSMUSG00000095200	Ighv1-7	immunoglobulin heavy variable V1-7 [Source:MGI Symbol;Acc:MGI:3704122]	0.373971578	-1.418999465	0.00025459	0.002174942	yes	down	165.02	248.42	106.96	170.83	489.15	940.31	345.77	297.47	464.22	199.218	507.384
ENSMUSG00000097709	2810429I04Rik	RIKEN cDNA 2810429I04 gene [Source:MGI Symbol;Acc:MGI:1924187]	0.37327361	-1.421694581	0.00076228	0.005392375	yes	down	0.61	0.17	0.15	0.46	0.69	1.58	2.04	0.88	1.05	0.3	1.248
ENSMUSG00000025355	Mmp19	matrix metallopeptidase 19 [Source:MGI Symbol;Acc:MGI:1927899]	0.373134328	-1.422233001	2.9128E-22	1.2719E-19	yes	down	31.32	17.32	20.42	21.39	43.19	67.67	50.06	57.1	54.31	22.328	54.466
ENSMUSG00000009092	Derl3	Der1-like domain family, member 3 [Source:MGI Symbol;Acc:MGI:1917627]	0.372856078	-1.423309237	7.41778E-11	3.85183E-09	yes	down	14.83	11.46	6.92	7.55	35.41	19.19	21.28	14.04	26.65	9.456	23.314
ENSMUSG00000090231	Cfb	complement factor B [Source:MGI Symbol;Acc:MGI:105975]	0.372162263	-1.425996321	1.51376E-22	7.09361E-20	yes	down	92.31	71.29	73.13	101.65	196.39	205.92	210.21	170.64	350.68	86.43	226.768
ENSMUSG00000045312	Lhfpl2	lipoma HMGIC fusion partner-like 2 [Source:MGI Symbol;Acc:MGI:2145236]	0.37037037	-1.432959407	9.67116E-32	1.54843E-28	yes	down	16.74	10.15	14.92	12.96	41.24	33.7	31.44	40.61	36.29	14.424	36.656

ENSMUSG00000108348	Gm42372	predicted gene, 42372 [Source:MGI Symbol;Acc:MGI:5625257]	0.368867577	-1.438825113	0.000268341	0.002273212	yes	down	0.04	0.13	0.11	0.16	0.31	0.25	0.34	0.23	0.36	0.118	0.298
ENSMUSG00000076733	Ighv8-13	immunoglobulin heavy variable 8-13 [Source:MGI Symbol;Acc:MGI:4439734]	0.368459838	-1.440420721	0.002465769	0.014074514	yes	down	11.51	18.94	7.06	12.29	33.2	35.5	16.45	18.74	36.74	10.83	28.126
ENSMUSG00000046179	E2f8	E2F transcription factor 8 [Source:MGI Symbol;Acc:MGI:1922038]	0.367782273	-1.443076151	1.11898E-11	7.02579E-10	yes	down	0.68	0.59	0.93	0.96	2.31	2.33	1.74	1.4	2.38	0.804	2.032
ENSMUSG00000009731	Kcnd1	potassium voltage-gated channel, Shal-related family, member 1 [Source:MGI Symbol;Acc:MGI:96671]	0.367511944	-1.444136957	2.57152E-05	0.000313096	yes	down	0.13	0.08	0.12	0.26	0.31	0.36	0.39	0.33	0.42	0.142	0.362
ENSMUSG00000044162	Tnip3	TNFAIP3 interacting protein 3 [Source:MGI Symbol;Acc:MGI:3041165]	0.36589828	-1.45048546	5.83018E-08	1.54931E-06	yes	down	1.73	0.78	1.53	1.5	3.99	3.59	5.24	2.6	4.63	1.366	4.01
ENSMUSG00000047420	Fam180a	family with sequence similarity 180, member A [Source:MGI Symbol;Acc:MGI:3039626]	0.36589828	-1.45048546	9.97754E-08	2.50259E-06	yes	down	3.33	1.98	3.03	1.55	8.69	4.93	3.85	6.76	9.16	2.614	6.678
ENSMUSG00000092021	Gbp11	guanylate binding protein 11 [Source:MGI Symbol;Acc:MGI:3646307]	0.36589828	-1.45048546	0.00620245	0.029636327	yes	down	0.33	0.36	0.2	0.14	0.55	0.15	0.49	0.66	0.52	0.242	0.474
ENSMUSG00000028965	Tnfrsf9	tumor necrosis factor receptor superfamily, member 9 [Source:MGI Symbol;Acc:MGI:1101059]	0.365497076	-1.45206823	8.41391E-18	1.61656E-15	yes	down	4.27	2.26	3.59	3.16	9.35	9.86	7.66	8.21	10.03	3.216	9.022
ENSMUSG00000097194	9330175E14Rik	RIKEN cDNA 9330175E14 gene [Source:MGI Symbol;Acc:MGI:2443913]	0.365363537	-1.452595435	8.64325E-05	0.000872179	yes	down	0.49	0.32	0.7	0.36	1.77	0.9	1.19	0.54	0.96	0.522	1.072
ENSMUSG00000019992	Mtfr2	mitochondrial fission regulator 2 [Source:MGI Symbol;Acc:MGI:1919054]	0.364298725	-1.456806149	0.001213387	0.007902647	yes	down	0.43	0.45	0.19	0.4	1.59	0.48	1.22	0.64	1	0.438	0.986
ENSMUSG00000031004	Mki67	antigen identified by monoclonal antibody Ki 67 [Source:MGI Symbol;Acc:MGI:106035]	0.364033491	-1.45785691	1.8038E-16	2.93699E-14	yes	down	4.6	3.28	5.87	4.44	15.71	10.82	9.93	8.08	15.41	5.388	11.99
ENSMUSG00000038508	Gdf15	growth differentiation factor 15 [Source:MGI Symbol;Acc:MGI:1346047]	0.36350418	-1.45995614	0.000214245	0.00187873	yes	down	1.42	0.71	0.76	0.32	3.39	2.2	1.87	1.57	3.25	0.944	2.456
ENSMUSG00000026228	Htr2b	5-hydroxytryptamine (serotonin) receptor 2B [Source:MGI Symbol;Acc:MGI:109323]	0.362450163	-1.464145457	3.76261E-07	7.95433E-06	yes	down	2.66	1.36	2.35	1.01	5.41	5.89	3.19	4.55	4.39	1.71	4.686
ENSMUSG00000044966	Fbxo48	F-box protein 48 [Source:MGI Symbol;Acc:MGI:2442569]	0.362318841	-1.464668267	0.001277846	0.008255295	yes	down	0.2	0.19	0.18	0.43	0.76	0.77	0.92	0.65	0.43	0.304	0.706
ENSMUSG00000048217	Nags	N-acetylglutamate synthase [Source:MGI Symbol;Acc:MGI:2387600]	0.357909807	-1.482332021	6.32246E-05	0.000675979	yes	down	0.61	0.41	0.55	0.2	0.79	1	1.05	1.09	1.31	0.408	1.048
ENSMUSG00000020649	Rrm2	ribonucleotide reductase M2 [Source:MGI Symbol;Acc:MGI:98181]	0.357525921	-1.483880254	4.54703E-13	3.71754E-11	yes	down	2.62	2.38	4.64	3.47	11.54	7.96	6.62	6.53	11.88	3.314	8.906
ENSMUSG00000002992	Apoc2	apolipoprotein C-II [Source:MGI Symbol;Acc:MGI:88054]	0.356252227	-1.489029064	7.40365E-08	1.91707E-06	yes	down	7.93	7.43	5.4	4.66	20.01	11.31	18.33	10.97	23.59	6.396	16.842
ENSMUSG00000102962	Gm37944	predicted gene, 37944 [Source:MGI Symbol;Acc:MGI:5611172]	0.355998576	-1.490056624	0.004758441	0.02403816	yes	down	141.62	98.58	19.4	41.63	280.53	197.88	133.93	134.88	301.65	78.304	209.774
ENSMUSG00000029657	Hsph1	heat shock 105kDa/110kDa protein 1 [Source:MGI Symbol;Acc:MGI:105053]	0.354609929	-1.495695163	1.97256E-11	1.17334E-09	yes	down	35.99	26.81	26.37	24.89	99.67	71.06	93.39	71.29	52.04	30.518	77.49
ENSMUSG00000025105	Bnc1	basonuclin 1 [Source:MGI Symbol;Acc:MGI:1097164]	0.353731871	-1.499271883	1.07973E-05	0.000150762	yes	down	0.28	0.35	0.25	0.34	0.9	0.74	0.86	1.28	0.3	0.308	0.816
ENSMUSG00000059674	Cdh24	cadherin-like 24 [Source:MGI Symbol;Acc:MGI:1928330]	0.35335689	-1.500802053	0.001346986	0.008595033	yes	down	0.25	0.77	0.7	0.42	0.61	1.92	0.27	0.23	0.35	0.454	0.676
ENSMUSG00000008461	Fut1	fucosyltransferase 1 [Source:MGI Symbol;Acc:MGI:109375]	0.351864884	-1.506906555	8.9789E-05	0.000901786	yes	down	0.75	0.74	0.19	0.35	1.71	1	1.11	1.12	1.31	0.464	1.25
ENSMUSG00000022033	Pbk	PDZ binding kinase [Source:MGI Symbol;Acc:MGI:1289156]	0.351493849	-1.508428653	8.48682E-07	1.62569E-05	yes	down	0.87	0.9	2.15	2.49	4.7	4.92	2.53	3.52	7.51	1.774	4.636
ENSMUSG00000038390	Gpr162	G protein-coupled receptor 162 [Source:MGI Symbol;Acc:MGI:1315214]	0.350754121	-1.511468039	4.2935E-13	3.54039E-11	yes	down	1.77	1.55	1.75	1.42	4.72	3.39	4	3.34	6.81	1.616	4.452
ENSMUSG00000090942	F830016B08Rik	RIKEN cDNA F830016B08 gene [Source:MGI Symbol;Acc:MGI:3588218]	0.348553503	-1.520547968	5.17666E-08	1.40913E-06	yes	down	2.07	1.3	2.49	1.32	6.65	4.04	3.5	3.26	5.6	1.712	4.61
ENSMUSG00000045232	Rln3	relaxin 3 [Source:MGI Symbol;Acc:MGI:2158015]	0.347826087	-1.523561956	0.007933059	0.035854592	yes	down	0.54	0.67	0.28	1.32	2.46	2.27	1.05	1.29	3.36	0.78	2.086
ENSMUSG00000016283	H2-M2	histocompatibility 2, M region locus 2 [Source:MGI Symbol;Acc:MGI:95914]	0.346740638	-1.528071165	1.64975E-23	9.05621E-21	yes	down	67.3	38.16	62.03	53.61	157.72	154.22	158.76	153	218.23	62.302	168.386
ENSMUSG00000109251	E230032D23Rik	RIKEN cDNA E230032D23 gene [Source:MGI Symbol;Acc:MGI:3041205]	0.346620451	-1.528571319	0.000292717	0.002444139	yes	down	3.4	1.4	3.59	4.27	6.7	7.71	8.47	6.25	7.03	2.762	7.232
ENSMUSG00000022586	Ly6i	lymphocyte antigen 6 complex, locus I [Source:MGI Symbol;Acc:MGI:1888480]	0.345901072	-1.531568609	4.40343E-06	6.9042E-05	yes	down	37.79	35.12	42.51	35.21	102.77	59.85	110.62	48.07	175.66	36.85	99.394
ENSMUSG00000095117	Ighv8-9	immunoglobulin heavy variable V8-9 [Source:MGI Symbol;Acc:MGI:3644475]	0.344946533	-1.535555333	0.00017232	0.001566433	yes	down	240.71	322.12	114.74	127.23	642.22	398.26	605.35	366.53	508.86	180.842	504.244

ENSMUSG00000035352	Ccl12	chemokine (C-C motif) ligand 12 [Source:MGI Symbol;Acc:MGI:108224]	0.344708721	-1.536550296	1.03948E-05	0.000146514	yes	down	4.65	6.85	6.64	8.41	22.67	8.56	17.63	10.13	20.37	5.918	15.872
ENSMUSG00000076525	Igkv1-99	immunoglobulin kappa variable 1-99 [Source:MGI Symbol;Acc:MGI:4439724]	0.343878955	-1.540027269	0.001860203	0.011168774	yes	down	137.11	233.55	74.37	320.83	510.19	225.85	696.52	299.59	894.81	193.738	525.392
ENSMUSG00000038037	Socs1	suppressor of cytokine signaling 1 [Source:MGI Symbol;Acc:MGI:1354910]	0.343760743	-1.540523296	5.38909E-05	0.000589974	yes	down	0.39	0.31	0.34	0.34	1.74	0.68	1.26	0.51	1.57	0.416	1.152
ENSMUSG00000053030	Spink2	serine peptidase inhibitor, Kazal type 2 [Source:MGI Symbol;Acc:MGI:1917232]	0.343524562	-1.541514839	8.87322E-08	2.24909E-06	yes	down	5.13	4.16	4.65	3.11	6.2	13.34	14.01	10.58	10.72	3.99	10.97
ENSMUSG00000029816	Gpnmb	glycoprotein (transmembrane) nmb [Source:MGI Symbol;Acc:MGI:1934765]	0.342465753	-1.545968369	2.26231E-12	1.64643E-10	yes	down	155.72	90.42	100.03	69.62	319.63	291.57	212.22	304.02	300.49	103.412	285.586
ENSMUSG00000026475	Rgs16	regulator of G-protein signaling 16 [Source:MGI Symbol;Acc:MGI:108407]	0.342114266	-1.547449829	4.11122E-09	1.46005E-07	yes	down	1.87	1.16	1.62	1.31	4.9	2.3	3.03	3.55	6.02	1.45	3.96
ENSMUSG00000047222	Rnase2a	ribonuclease, RNase A family, 2A (liver, eosinophil-derived neurotoxin) [Source:MGI Symbol;Acc:MGI:1890465]	0.340367597	-1.554834396	0.002877215	0.015903317	yes	down	2.07	0.69	0.6	1.38	1.99	4.7	3.61	1.36	2.78	1.042	2.888
ENSMUSG00000039196	Orm1	orosomucoid 1 [Source:MGI Symbol;Acc:MGI:97443]	0.339558574	-1.558267634	6.48706E-24	3.66576E-21	yes	down	10.84	10.39	9.46	10.04	28.99	27.13	29.13	25.73	38.15	10.806	29.826
ENSMUSG00000026452	Syt2	synaptotagmin II [Source:MGI Symbol;Acc:MGI:99666]	0.338180588	-1.564134243	0.000389123	0.003103451	yes	down	0.18	0.08	0.06	0.08	1.17	0.21	0.25	0.55	0.18	0.108	0.472
ENSMUSG00000000982	Ccl3	chemokine (C-C motif) ligand 3 [Source:MGI Symbol;Acc:MGI:98260]	0.337952011	-1.565109697	4.16286E-08	1.15579E-06	yes	down	5.04	4.62	11.65	4.85	19.04	17.53	13.37	15.23	15.35	5.826	16.104
ENSMUSG00000036086	Zranb3	zinc finger, RAN-binding domain containing 3 [Source:MGI Symbol;Acc:MGI:1918362]	0.3354579	-1.575796375	1.35576E-25	9.30293E-23	yes	down	5.57	2.61	5.75	5.25	16.19	16.15	17.83	14.76	14.84	5.254	15.954
ENSMUSG00000029304	Spp1	secreted phosphoprotein 1 [Source:MGI Symbol;Acc:MGI:98389]	0.333333333	-1.584962501	1.64244E-51	1.57781E-47	yes	down	477.32	288.91	413.06	420.92	1233.76	1084.53	1030.56	1212.5	1213.59	413.418	1154.988
ENSMUSG00000102555	6430511E19Rik	RIKEN cDNA 6430511E19 gene [Source:MGI Symbol;Acc:MGI:2443259]	0.333222259	-1.585443319	0.002325784	0.013446493	yes	down	0.12	0.09	0.16	0.26	0.32	0.45	0.52	0.14	0.38	0.132	0.362
ENSMUSG00000026039	Sgo2a	shugoshin 2A [Source:MGI Symbol;Acc:MGI:1098767]	0.333111259	-1.585923977	1.29595E-12	9.95963E-11	yes	down	0.44	0.35	0.44	0.56	1.19	1.19	1.07	0.93	1.52	0.418	1.18
ENSMUSG00000070427	Il18bp	interleukin 18 binding protein [Source:MGI Symbol;Acc:MGI:1333800]	0.332005312	-1.59072177	9.72578E-13	7.56524E-11	yes	down	10.42	5.46	10.62	5.34	24.74	17.65	19.27	17.77	41.19	8.186	24.124
ENSMUSG00000044313	Mab21l3	mab-21-like 3 (C. elegans) [Source:MGI Symbol;Acc:MGI:2446273]	0.331564987	-1.592636429	0.000109952	0.001072885	yes	down	0.39	0.41	0.2	0.29	0.94	0.92	0.91	0.29	1.06	0.292	0.824
ENSMUSG00000085913	Gm15601	predicted gene 15601 [Source:MGI Symbol;Acc:MGI:3783048]	0.33101622	-1.595026184	5.17799E-08	1.40913E-06	yes	down	0.72	0.88	0.55	0.48	2.11	2.02	1.23	0.71	1.69	0.614	1.552
ENSMUSG00000027004	Frzb	frizzled-related protein [Source:MGI Symbol;Acc:MGI:892032]	0.329489292	-1.601696516	0.006081727	0.029175584	yes	down	0.03	0.18	0.15	0.17	0.32	0.42	0.69	0.1	0.54	0.126	0.414
ENSMUSG00000096490	Igkv10-94	immunoglobulin kappa variable 10-94 [Source:MGI Symbol;Acc:MGI:3646140]	0.328947368	-1.604071324	0.003511873	0.018753088	yes	down	881.99	882.91	188.45	307.85	2524.27	3178.12	956.63	1514.63	1010.47	622.328	1836.824
ENSMUSG00000067455	Hist1h4j	histone cluster 1, H4j [Source:MGI Symbol;Acc:MGI:2448436]	0.323834197	-1.626672753	0.000776162	0.005472444	yes	down	0.19	0.66	0.61	0.83	1.64	1.42	1.5	2.24	1.96	0.614	1.752
ENSMUSG00000036223	Ska1	spindle and kinetochore associated complex subunit 1 [Source:MGI Symbol;Acc:MGI:1913718]	0.322061192	-1.634593268	0.009429485	0.041127965	yes	down	0.22	0.14	0.19	0.81	1.3	0.89	0.14	0.62	1.19	0.302	0.828
ENSMUSG00000067577	A430093F15Rik	RIKEN cDNA A430093F15 gene [Source:MGI Symbol;Acc:MGI:2685520]	0.321336761	-1.63784206	0.010806549	0.045742725	yes	down	0.53	0.25	0.14	0.94	0.58	0.22	0.67	0.55	2.38	0.51	0.88
ENSMUSG00000027832	Ptx3	pentraxin related gene [Source:MGI Symbol;Acc:MGI:104641]	0.317762949	-1.653977179	0.000182892	0.001647398	yes	down	0.37	0.06	0.59	0.85	1.7	1.31	1.49	1.44	2.41	0.584	1.67
ENSMUSG00000041064	Pif1	PIF1 5'-to-3' DNA helicase [Source:MGI Symbol;Acc:MGI:2143057]	0.316055626	-1.6617496	0.000600966	0.004446034	yes	down	0.98	0.59	0.15	2.42	1.21	0.73	0.25	0.73	1.58	0.88	0.9
ENSMUSG00000035042	Ccl5	chemokine (C-C motif) ligand 5 [Source:MGI Symbol;Acc:MGI:98262]	0.315457413	-1.66448284	3.24677E-09	1.17698E-07	yes	down	217.12	150.14	217.78	165.16	691.81	475.81	423.16	420.1	957.52	200.784	593.68
ENSMUSG00000086712	AI427809	expressed sequence AI427809 [Source:MGI Symbol;Acc:MGI:2140270]	0.315258512	-1.665392771	1.77565E-09	6.86431E-08	yes	down	1.04	0.8	0.76	0.8	2.27	2.25	1.52	2.57	2.44	0.908	2.21
ENSMUSG00000106905	Gm42741	predicted gene 42741 [Source:MGI Symbol;Acc:MGI:5662878]	0.315159155	-1.665847521	0.009453314	0.041213188	yes	down	0.07	0.28	0.1	0.08	0.3	0.48	0.3	0.4	0.19	0.11	0.334
ENSMUSG00000015709	Arnt2	aryl hydrocarbon receptor nuclear translocator 2 [Source:MGI Symbol;Acc:MGI:107188]	0.314267756	-1.669933836	6.75433E-08	1.77041E-06	yes	down	0.9	0.22	0.27	0.36	25.48	0.76	0.51	1.16	1.07	0.412	5.796
ENSMUSG00000094930	Igkv6-25	immunoglobulin kappa chain variable 6-25 [Source:MGI Symbol;Acc:MGI:4439867]	0.313873195	-1.671746267	0.00289281	0.015980323	yes	down	995.68	1148.3	338	935.33	4224.71	1131.88	1042.91	1503.51	3400.04	741.636	2260.61

ENSMUSG00000104927	Gm43388	predicted gene 43388 [Source:MGI Symbol;Acc:MGI:5663525]	0.312109863	-1.679874148	0.000387339	0.003091794	yes	down	0.4	0.34	0.46	0.37	1.37	1.08	1.6	0.42	1.14	0.374	1.122
ENSMUSG00000076552	Igkv4-61	immunoglobulin kappa chain variable 4-61 [Source:MGI Symbol;Acc:MGI:4439819]	0.311720698	-1.681674142	0.001012602	0.006807252	yes	down	232.36	508.8	144.26	187.85	1755.49	654.65	359.27	468.79	607.97	237.534	769.234
ENSMUSG00000070304	Scn2b	sodium channel, voltage-gated, type II, beta [Source:MGI Symbol;Acc:MGI:106921]	0.311623559	-1.68212379	0.000422194	0.003321707	yes	down	0.18	0.15	0.06	0.07	0.58	0.18	0.43	0.42	0.19	0.12	0.36
ENSMUSG00000056043	Rgs9bp	regulator of G-protein signalling 9 binding protein [Source:MGI Symbol;Acc:MGI:2384418]	0.309981401	-1.689746438	0.003804323	0.020008883	yes	down	0.03	0.03	0.04	0.06	0.25	0.04	0.09	0.18	0.09	0.042	0.13
ENSMUSG00000108064	Gm44423	predicted gene, 44423 [Source:MGI Symbol;Acc:MGI:5690815]	0.309119011	-1.693765712	7.96078E-05	0.000816607	yes	down	0.37	0.18	0.15	0.19	0.63	0.99	0.55	0.29	0.6	0.2	0.612
ENSMUSG00000111340	Gm47171	predicted gene, 47171 [Source:MGI Symbol;Acc:MGI:6095951]	0.30835646	-1.697329024	0.002527123	0.014356481	yes	down	0.15	0.12	0.14	0.18	0.77	1.01	0.49	0.08	0.35	0.18	0.54
ENSMUSG00000053318	Slamf8	SLAM family member 8 [Source:MGI Symbol;Acc:MGI:1921998]	0.307125307	-1.7031007	1.19263E-13	1.11233E-11	yes	down	6.67	4.78	6.32	5.36	20.88	12.14	13.23	12.29	29.32	5.846	17.572
ENSMUSG00000074934	Grem1	gremlin 1, DAN family BMP antagonist [Source:MGI Symbol;Acc:MGI:1344337]	0.3066544	-1.705314441	1.19482E-05	0.000164324	yes	down	0.29	0.12	0.36	0.62	0.67	0.7	1.67	0.76	0.98	0.32	0.956
ENSMUSG00000076577	Igkv8-30	immunoglobulin kappa chain variable 8-30 [Source:MGI Symbol;Acc:MGI:3642250]	0.304878049	-1.713695815	3.03087E-05	0.00035982	yes	down	472.25	829.31	475.81	376.46	2763.85	1617.31	1275.24	610.87	1937.35	525.374	1640.924
ENSMUSG00000108897	Gm44861	predicted gene 44861 [Source:MGI Symbol;Acc:MGI:5753437]	0.304136253	-1.717210299	0.001478114	0.009253506	yes	down	0.4	0.34	0.71	0.16	0.85	1.97	0.88	1.48	0.5	0.362	1.136
ENSMUSG00000079362	Gm43302	predicted gene 43302 [Source:MGI Symbol;Acc:MGI:5663439]	0.303674461	-1.719402512	0.000686099	0.004970597	yes	down	2.5	0.6	0.4	1.63	3.54	2.06	2.97	1.84	4.35	1.192	2.952
ENSMUSG00000025279	Dnase1l3	deoxyribonuclease 1-like 3 [Source:MGI Symbol;Acc:MGI:1314633]	0.302297461	-1.725959235	5.02126E-12	3.44548E-10	yes	down	2.05	1.58	1.49	1.43	7.62	3.63	3.43	3.67	8.17	1.716	5.304
ENSMUSG00000094993	Igkv4-51	immunoglobulin kappa chain variable 4-51 [Source:MGI Symbol;Acc:MGI:5009829]	0.302023558	-1.727267011	0.009691645	0.042042353	yes	down	36.48	100.04	42.07	43.45	558.18	60.37	282.68	222.5	158.38	82.95	256.422
ENSMUSG00000106219	5830416I19Rik	RIKEN cDNA 5830416I19 gene [Source:MGI Symbol;Acc:MGI:1922007]	0.301114122	-1.731617723	9.36525E-06	0.000133972	yes	down	0.22	0.2	0.22	0.17	0.48	0.7	0.54	0.42	0.75	0.186	0.578
ENSMUSG00000105338	Gm43802	predicted gene 43802 [Source:MGI Symbol;Acc:MGI:5663939]	0.299043062	-1.741574847	0.000754572	0.005349667	yes	down	4.32	0	2.5	4.31	10.75	7.73	6.53	7.72	6.7	2.526	7.886
ENSMUSG00000085174	Gm16206	predicted gene 16206 [Source:MGI Symbol;Acc:MGI:3801870]	0.29489826	-1.761710784	0.005197561	0.025803807	yes	down	0.17	0.08	0.08	0.06	0.61	0.44	0.13	0.32	0.28	0.112	0.356
ENSMUSG00000102302	Gm38190	predicted gene, 38190 [Source:MGI Symbol;Acc:MGI:5611418]	0.294204178	-1.765110362	0.001664588	0.010169069	yes	down	0.28	0.04	0.11	0.06	0.73	0.35	0.29	0.12	0.33	0.116	0.364
ENSMUSG00000024402	Lta	lymphotoxin A [Source:MGI Symbol;Acc:MGI:104797]	0.291970803	-1.776103988	8.32512E-05	0.000848312	yes	down	0.68	0.25	0.61	0.41	2.07	0.71	0.91	1.71	1.65	0.442	1.41
ENSMUSG00000046031	Calhm6	calcium homeostasis modulator family member 6 [Source:MGI Symbol;Acc:MGI:2443082]	0.291460216	-1.778629126	7.17467E-07	1.41237E-05	yes	down	4.71	2.68	2.31	2.24	10.23	5.18	6.63	5.2	16.61	2.716	8.77
ENSMUSG00000085295	4930430E12Rik	RIKEN cDNA 4930430E12 gene [Source:MGI Symbol;Acc:MGI:1918889]	0.290866783	-1.781569545	3.94021E-19	9.46291E-17	yes	down	2.3	1.43	2.88	1.44	7.37	5.56	4.64	6.04	7.66	1.984	6.254
ENSMUSG00000061578	Ksr2	kinase suppressor of ras 2 [Source:MGI Symbol;Acc:MGI:3610315]	0.289435601	-1.788685711	1.81412E-07	4.26619E-06	yes	down	0.14	0.05	0.09	0.06	0.3	0.31	0.17	0.37	0.22	0.086	0.274
ENSMUSG00000022303	Dcstamp	dendrocyte expressed seven transmembrane protein [Source:MGI Symbol;Acc:MGI:1923016]	0.2891845	-1.789937869	2.82681E-16	4.45176E-14	yes	down	2.19	1.38	1.82	1.34	8.08	5.51	8.49	5.01	16.47	2.11	8.712
ENSMUSG00000076614	Ighg1	immunoglobulin heavy constant gamma 1 (G1m marker) [Source:MGI Symbol;Acc:MGI:96446]	0.288018433	-1.795766948	0.009975186	0.042923461	yes	down	1040.83	316.09	88.23	138.2	2440	772.12	756.92	1001.49	2623.31	468.684	1518.768
ENSMUSG00000044309	Apol7c	apolipoprotein L 7c [Source:MGI Symbol;Acc:MGI:1920912]	0.285632676	-1.807767062	3.17618E-12	2.24353E-10	yes	down	1.61	1.91	1.39	1.83	6.26	5.11	5.34	2.72	9.34	1.71	5.754
ENSMUSG00000076563	Igkv5-48	immunoglobulin kappa variable 5-48 [Source:MGI Symbol;Acc:MGI:3642817]	0.284900285	-1.811471031	9.19536E-07	1.74748E-05	yes	down	284.22	172.23	260.85	224.42	643.63	417.54	1018.87	1263.71	353.87	226.31	739.524
ENSMUSG00000028197	Col24a1	collagen, type XXIV, alpha 1 [Source:MGI Symbol;Acc:MGI:1918605]	0.280583614	-1.833497337	1.44659E-09	5.70705E-08	yes	down	0.61	0.21	0.72	0.62	1.85	2	3.18	2.76	2.46	0.64	2.45
ENSMUSG00000095565	Ighv2-9-1	immunoglobulin heavy variable 2-9-1 [Source:MGI Symbol;Acc:MGI:4439519]	0.279251606	-1.840362519	0.000637305	0.004677063	yes	down	431.41	311.02	249.9	87.47	1985.54	1310.26	623.16	1347.07	602.98	345.664	1173.802
ENSMUSG00000095583	Ighv14-2	immunoglobulin heavy variable 14-2 [Source:MGI Symbol;Acc:MGI:4439607]	0.279173646	-1.840765337	0.000943769	0.006430011	yes	down	134.43	104.39	33.71	83.16	321.76	96.48	467.64	305.46	150.66	79.034	268.4
ENSMUSG00000093861	Igkv1-110	immunoglobulin kappa variable 1-110 [Source:MGI Symbol;Acc:MGI:4439558]	0.278086763	-1.846393021	0.002329786	0.013462311	yes	down	427.93	662.07	69.85	226.03	1371.62	983.91	1661.25	395.64	1501.46	341.122	1182.776
ENSMUSG00000112843	Gm46224	predicted gene, 46224 [Source:MGI Symbol;Acc:MGI:5825861]	0.27739251	-1.849999259	0.002089934	0.012320926	yes	down	0.39	0.34	0.4	0.31	1.65	1.59	0.28	0.78	0.78	0.298	1.016

ENSMUSG00000078922	Tgtp1	T cell specific GTPase 1 [Source:MGI Symbol;Acc:MGI:98734]	0.274876306	-1.863145543	5.79085E-10	2.48903E-08	yes	down	5.82	4.35	5.5	5.35	25.16	10.52	17.83	11.48	28.06	5.502	18.61
ENSMUSG00000079800	AC125149.3	-	0.272479564	-1.875780063	1.89028E-05	0.000241958	yes	down	1.15	0.39	1.08	0.48	9.13	5	3.99	2.4	3.42	0.998	4.788
ENSMUSG00000040264	Gbp2b	guanylate binding protein 2b [Source:MGI Symbol;Acc:MGI:95666]	0.272405339	-1.876173114	3.21697E-05	0.000378956	yes	down	0.15	0.34	0.11	0.2	1.06	0.79	0.39	0.39	1.09	0.216	0.744
ENSMUSG00000103657	Gm37204	predicted gene, 37204 [Source:MGI Symbol;Acc:MGI:5610432]	0.272331155	-1.876566059	0.005269894	0.026059493	yes	down	0.11	0.03	0.05	0.01	0.16	0.13	0.12	0.16	0.14	0.042	0.142
ENSMUSG00000018930	Ccl4	chemokine (C-C motif) ligand 4 [Source:MGI Symbol;Acc:MGI:98261]	0.27203482	-1.878136767	4.23469E-06	6.66036E-05	yes	down	1.23	0.96	2.06	3.64	7.5	4.52	4.5	7.37	5.86	1.726	5.95
ENSMUSG00000102496	Gm36989	predicted gene, 36989 [Source:MGI Symbol;Acc:MGI:5610217]	0.271296799	-1.882056071	0.008235003	0.03693439	yes	down	0.27	0.02	0.25	0.08	0.71	0.51	0.41	0.24	0.3	0.124	0.434
ENSMUSG00000094075	Ighv1-80	immunoglobulin heavy variable 1-80 [Source:MGI Symbol;Acc:MGI:4439738]	0.27005131	-1.88869455	0.00160044	0.00986185	yes	down	460.95	440.69	116.21	176.35	773.27	1872.71	425.46	1311.74	461.52	266.23	968.94
ENSMUSG00000097423	Gm26522	predicted gene, 26522 [Source:MGI Symbol;Acc:MGI:5477016]	0.269759914	-1.890252115	0.001752582	0.010625546	yes	down	0.18	0.25	0.22	0.28	1.71	0.83	0.75	0.16	0.44	0.224	0.778
ENSMUSG00000050439	Enthd1	ENTH domain containing 1 [Source:MGI Symbol;Acc:MGI:2686088]	0.268384326	-1.897627674	0.008822386	0.038939699	yes	down	0.1	0.05	0.03	0.14	0.22	0.55	0.17	0.16	0.18	0.074	0.256
ENSMUSG00000086907	Gm15298	predicted gene 15298 [Source:MGI Symbol;Acc:MGI:3705276]	0.267022697	-1.904965719	0.010479343	0.044671175	yes	down	0.23	0.26	0.06	0.11	1.49	0.54	0.17	0.73	0.29	0.186	0.644
ENSMUSG00000094797	Igkv6-15	immunoglobulin kappa variable 6-15 [Source:MGI Symbol;Acc:MGI:1330831]	0.266453504	-1.90804429	0.000148691	0.00138747	yes	down	500.07	513.2	241	226.1	2285.09	833.08	1728.16	639.76	617.22	341.052	1220.662
ENSMUSG00000075555	Gm10863	predicted gene 10863 [Source:MGI Symbol;Acc:MGI:3641623]	0.265463233	-1.913416033	0.007685256	0.035081212	yes	down	0.15	0.51	0.46	0.1	1.51	1.11	0.66	0.51	1.02	0.26	0.962
ENSMUSG00000103133	Gm37303	predicted gene, 37303 [Source:MGI Symbol;Acc:MGI:5610531]	0.263435195	-1.924479992	0.001875558	0.011236389	yes	down	0.18	0.04	0.11	0.12	0.46	0.79	0.36	0.27	0.62	0.142	0.5
ENSMUSG00000083396	Gm15542	predicted gene 15542 [Source:MGI Symbol;Acc:MGI:3782990]	0.263365815	-1.924859999	0.004404202	0.022546745	yes	down	0.53	0.44	0.27	0	2.25	1.55	0.67	0.68	0.36	0.358	1.102
ENSMUSG00000030124	Lag3	lymphocyte-activation gene 3 [Source:MGI Symbol;Acc:MGI:106588]	0.263019463	-1.926758532	5.02476E-11	2.70217E-09	yes	down	1.46	1.26	1.05	1.05	5.7	2.62	3.7	2.84	7.26	1.244	4.424
ENSMUSG00000095130	Ighv1-39	immunoglobulin heavy variable 1-39 [Source:MGI Symbol;Acc:MGI:4439888]	0.26157468	-1.9347052	1.07887E-05	0.000150751	yes	down	151.15	142.48	166.1	166.06	841.01	799.64	647.85	146.46	292.61	152.672	545.514
ENSMUSG00000055170	Ifng	interferon gamma [Source:MGI Symbol;Acc:MGI:107656]	0.259538022	-1.945982186	0.000311177	0.002574946	yes	down	0.45	0.15	0.72	0.6	2.68	2.64	2.4	1.5	2.42	0.446	2.328
ENSMUSG00000090675	Olfr111	olfactory receptor 111 [Source:MGI Symbol;Acc:MGI:2177494]	0.258732212	-1.950468414	1.44389E-05	0.000192783	yes	down	0.71	0.11	0.3	0.26	1.51	1.09	1.2	0.83	1.6	0.342	1.246
ENSMUSG00000024401	Tnf	tumor necrosis factor [Source:MGI Symbol;Acc:MGI:104798]	0.254582485	-1.97379493	0.000439406	0.003439944	yes	down	0.21	0.2	0.07	0.21	1.38	0.58	0.87	0.29	0.96	0.234	0.816
ENSMUSG00000104164	Gm38248	predicted gene, 38248 [Source:MGI Symbol;Acc:MGI:5611476]	0.254323499	-1.975263322	9.23751E-06	0.000132349	yes	down	0.13	0.08	0.08	0.14	0.71	0.61	0.31	0.13	0.38	0.118	0.428
ENSMUSG00000104452	Ighv8-8	immunoglobulin heavy variable 8-8 [Source:MGI Symbol;Acc:MGI:3815333]	0.253936008	-1.977463111	0.000165217	0.001518087	yes	down	516.76	707.74	204.27	264.97	2000.59	1304.77	1542.33	792.04	1264.29	361.784	1380.804
ENSMUSG00000035385	Ccl2	chemokine (C-C motif) ligand 2 [Source:MGI Symbol;Acc:MGI:98259]	0.253742705	-1.978561749	9.98658E-06	0.000141603	yes	down	0.97	0.53	1.28	0.48	3.69	2.24	3.37	1.7	5.57	0.922	3.314
ENSMUSG00000076695	Ighv1-18	immunoglobulin heavy variable V1-18 [Source:MGI Symbol;Acc:MGI:4439780]	0.25220681	-1.987320866	0.000496569	0.003791966	yes	down	275.87	355.29	85.8	101.81	754.74	302.18	601.64	832.91	900.45	175.854	678.384
ENSMUSG00000096573	1700009J07Rik	RIKEN cDNA 1700009J07 gene [Source:MGI Symbol;Acc:MGI:1914938]	0.250752257	-1.99566541	0.004899956	0.024557016	yes	down	0.33	0.07	0.08	0.32	0.24	0.75	0.78	0.86	0.73	0.206	0.672
ENSMUSG00000022367	Has2	hyaluronan synthase 2 [Source:MGI Symbol;Acc:MGI:107821]	0.245459008	-2.02644598	0.000309316	0.002566012	yes	down	0.16	0.01	0.11	0.04	0.26	0.3	44.24	0.16	0.63	0.092	9.118
ENSMUSG00000032796	Lama1	laminin, alpha 1 [Source:MGI Symbol;Acc:MGI:99892]	0.239923225	-2.059355278	5.66774E-14	5.52762E-12	yes	down	0.49	0.23	0.31	1.35	1.84	3.53	0.74	1.91	1.97	0.53	1.998
ENSMUSG00000080115	Eef1akmt3	EEF1A lysine methyltransferase 3 [Source:MGI Symbol;Acc:MGI:3645330]	0.23923445	-2.063502942	1.68113E-05	0.000219874	yes	down	0.19	0.08	0.1	0.12	0.54	0.39	0.73	0.26	0.42	0.122	0.468
ENSMUSG00000097484	Gm26807	predicted gene, 26807 [Source:MGI Symbol;Acc:MGI:5477301]	0.239120038	-2.064193062	0.004122105	0.021289784	yes	down	0.03	0	0.05	0.07	0.05	0.33	0.23	0.07	0.23	0.046	0.182
ENSMUSG00000101903	Gm29291	predicted gene 29291 [Source:MGI Symbol;Acc:MGI:5579997]	0.234796901	-2.09051473	0.006885673	0.032141507	yes	down	0.52	0.26	0.55	0.14	1.7	1.44	0.64	0.93	1.15	0.294	1.172
ENSMUSG00000019874	Fabp7	fatty acid binding protein 7, brain [Source:MGI Symbol;Acc:MGI:101916]	0.234411627	-2.092883966	3.6255E-08	1.01986E-06	yes	down	1.62	0.45	1.86	0.59	5.77	6.1	3.14	5.37	4.75	1.27	5.026

ENSMUSG00000079625	Tm4sf19	transmembrane 4 L six family member 19 [Source:MGI Symbol;Acc:MGI:3645933]	0.233372229	-2.099295204	2.25568E-07	5.13488E-06	yes	down	0.14	0.47	0.93	0.62	2.85	2.08	2.2	1.53	3.44	0.608	2.42
ENSMUSG00000076555	Igkv4-57-1	immunoglobulin kappa variable 4-57-1 [Source:MGI Symbol;Acc:MGI:2686264]	0.232720503	-2.103329777	0.000492538	0.00377619	yes	down	55.5	47.27	12.2	47.16	367.59	235.05	76.24	331.77	199.64	59.718	242.058
ENSMUSG00000040026	Saa3	serum amyloid A 3 [Source:MGI Symbol;Acc:MGI:98223]	0.229937917	-2.120683709	2.46049E-10	1.14463E-08	yes	down	244.61	211.13	189.13	136.36	938.56	535.95	378.28	809.5	1307.33	193.39	793.924
ENSMUSG00000046623	Gjb4	gap junction protein, beta 4 [Source:MGI Symbol;Acc:MGI:95722]	0.226911731	-2.139796897	0.003146684	0.017107312	yes	down	0.32	0.08	0	0.22	0.34	0.59	0.74	0.72	0.32	0.132	0.542
ENSMUSG00000115855	Gm34643	predicted gene, 34643 [Source:MGI Symbol;Acc:MGI:5593802]	0.225988701	-2.145677455	0.004811725	0.024239032	yes	down	0.14	0	0.12	0	0.47	0.3	0.14	0.32	0.23	0.07	0.292
ENSMUSG00000096464	Ighv2-2	immunoglobulin heavy variable 2-2 [Source:MGI Symbol;Acc:MGI:4439894]	0.223613596	-2.160920188	0.000504901	0.003844892	yes	down	104.29	163.61	39.05	152.9	377.8	389.17	875.93	105.43	369.94	100.452	423.654
ENSMUSG00000117743	AC129188.1	novel transcript, antisense to Spindoc	0.222866057	-2.165751184	0.001718155	0.010453109	yes	down	0.04	0.21	0.13	0.09	0.18	0.82	0.41	0.71	0.41	0.12	0.506
ENSMUSG00000029608	Rph3a	rabphilin 3A [Source:MGI Symbol;Acc:MGI:102788]	0.222123501	-2.170566057	1.01181E-07	2.53007E-06	yes	down	0.13	0.04	0.16	0.26	0.24	0.56	0.5	0.48	0.57	0.138	0.47
ENSMUSG00000076536	Igkv4-86	immunoglobulin kappa variable 4-86 [Source:MGI Symbol;Acc:MGI:2685305]	0.221336875	-2.175684271	0.00980246	0.042381954	yes	down	9.33	8.4	3.55	2.12	14.59	89.98	20.56	54.79	3.34	8.43	36.652
ENSMUSG00000105096	Gbp10	guanylate-binding protein 10 [Source:MGI Symbol;Acc:MGI:4359647]	0.218866273	-2.191878443	4.40138E-11	2.39557E-09	yes	down	1.68	0.89	1.21	0.5	4.66	4.23	1.74	3.99	4.95	1.042	3.914
ENSMUSG00000034855	Cxcl10	chemokine (C-X-C motif) ligand 10 [Source:MGI Symbol;Acc:MGI:1352450]	0.215192597	-2.216299645	3.89615E-07	8.19002E-06	yes	down	7.35	3.7	3.55	4.53	30.89	9.11	18.07	11.81	37.2	5.02	21.416
ENSMUSG00000083178	Gm12187	predicted gene 12187 [Source:MGI Symbol;Acc:MGI:3651944]	0.209511837	-2.254896336	0.009704565	0.042079398	yes	down	0.04	0.21	0.04	0.05	0.77	0.29	0.42	0.05	0.43	0.086	0.392
ENSMUSG00000048215	A630023P12Rik	RIKEN cDNA A630023P12 gene [Source:MGI Symbol;Acc:MGI:2445162]	0.208811861	-2.259724436	0.003817561	0.020050731	yes	down	0.13	0.15	0.05	0.11	5.28	1.5	8.21	0.11	0.65	0.212	3.15
ENSMUSG00000096499	Ighv1-5	immunoglobulin heavy variable V1-5 [Source:MGI Symbol;Acc:MGI:3704121]	0.208073242	-2.264836648	0.001407071	0.008916242	yes	down	96.4	68.33	25.08	197.97	227.83	248.88	731.29	449.97	141.97	80.82	359.988
ENSMUSG00000103847	Gm20056	predicted gene, 20056 [Source:MGI Symbol;Acc:MGI:5012241]	0.207168013	-2.271126827	0.000192224	0.001720167	yes	down	0.6	0.2	2.53	1.33	8.96	2.41	3.74	4.54	3.65	1.056	4.66
ENSMUSG00000068606	Gm4841	predicted gene 4841 [Source:MGI Symbol;Acc:MGI:3643814]	0.204834084	-2.287472295	1.55007E-08	4.87423E-07	yes	down	0.69	0.11	0.33	0.21	1.79	0.95	1.19	1.25	2.37	0.332	1.51
ENSMUSG00000085885	Gm11906	predicted gene 11906 [Source:MGI Symbol;Acc:MGI:3705114]	0.204415372	-2.290424404	2.57881E-06	4.28975E-05	yes	down	0.24	0.1	0.2	0.09	1.08	0.93	0.74	0.38	1.75	0.216	0.976
ENSMUSG00000091405	Hist2h4	histone cluster 2, H4 [Source:MGI Symbol;Acc:MGI:2140113]	0.202224469	-2.305970521	0.00027128	0.002294063	yes	down	0.1	0.44	0.04	0.3	1.43	1.6	0.47	0.4	0.76	0.198	0.932
ENSMUSG00000076583	Igkv8-24	immunoglobulin kappa chain variable 8-24 [Source:MGI Symbol;Acc:MGI:4947958]	0.202101859	-2.3068455	8.07927E-08	2.06419E-06	yes	down	144.31	167.01	128.57	132.63	398.9	446.74	270.27	1242	681.6	129.06	607.902
ENSMUSG00000041324	Inhba	inhibin beta-A [Source:MGI Symbol;Acc:MGI:96570]	0.201491034	-2.311212455	3.75618E-25	2.48853E-22	yes	down	1.02	0.72	1.97	0.93	7.29	4.57	4.95	4.8	8.02	1.194	5.926
ENSMUSG00000024032	Tff1	trefoil factor 1 [Source:MGI Symbol;Acc:MGI:88135]	0.200360649	-2.319328904	0.007318525	0.033687307	yes	down	0.33	0.44	0	0	1.8	0.22	1.2	1.08	1.57	0.246	1.174
ENSMUSG00000029371	Cxcl5	chemokine (C-X-C motif) ligand 5 [Source:MGI Symbol;Acc:MGI:1096868]	0.197355437	-2.341131829	0.005389578	0.026550285	yes	down	0.04	0	0.55	0.26	0.29	0.43	3.1	0.65	0.29	0.206	0.952
ENSMUSG00000094194	Ighv5-16	immunoglobulin heavy variable 5-16 [Source:MGI Symbol;Acc:MGI:4439556]	0.196386489	-2.348232419	0.000452623	0.003527893	yes	down	40.36	104.18	37.89	45.89	273.56	26.07	318.21	295.44	585.33	62.196	299.722
ENSMUSG00000096670	Ighv2-6	immunoglobulin heavy variable 2-6 [Source:MGI Symbol;Acc:MGI:4439518]	0.195427008	-2.355298233	2.75913E-06	4.54641E-05	yes	down	12.67	47.17	43.78	11.09	86.11	216.56	219.9	145.97	98.25	31.79	153.358
ENSMUSG00000084918	Gm12708	predicted gene 12708 [Source:MGI Symbol;Acc:MGI:3652178]	0.195388824	-2.355580147	0.001532679	0.009523455	yes	down	0.12	0.06	0.31	0.15	0.46	0.61	0.99	3.29	0.85	0.182	1.24
ENSMUSG00000104080	Gm38279	predicted gene, 38279 [Source:MGI Symbol;Acc:MGI:5611507]	0.194855807	-2.35952117	0.000464491	0.003599945	yes	down	0.25	0.4	0.12	0.15	0.74	1.52	0.56	0.63	0.94	0.184	0.878
ENSMUSG00000061540	Orm2	orosomucoid 2 [Source:MGI Symbol;Acc:MGI:97444]	0.193162063	-2.372116318	0.000777918	0.005478789	yes	down	0.48	0.66	0.69	0.62	3.27	1.13	3.63	0.31	3.53	0.49	2.374
ENSMUSG00000029607	Ankrd61	ankyrin repeat domain 61 [Source:MGI Symbol;Acc:MGI:1913979]	0.192086055	-2.380175312	0.001985956	0.011791154	yes	down	0.11	0.08	0.04	0.12	0.31	1.06	0.25	0.27	0.15	0.09	0.408
ENSMUSG00000060112	Olfr60	olfactory receptor 60 [Source:MGI Symbol;Acc:MGI:1333881]	0.191975427	-2.381006437	0.001591723	0.009817815	yes	down	5.9	0	0.03	0.07	41.57	4.63	0.23	0.38	0.25	1.214	9.412
ENSMUSG00000002190	Clgn	calmegin [Source:MGI Symbol;Acc:MGI:107472]	0.191277735	-2.386259141	0.007452416	0.034205274	yes	down	0	0	0.1	0.04	0.55	0.09	0.27	0.05	0.26	0.036	0.244

ENSMUSG00000009185	Ccl8	chemokine (C-C motif) ligand 8 [Source:MGI Symbol;Acc:MGI:101878]	0.187652468	-2.413864833	5.41696E-15	6.46435E-13	yes	down	37.31	29.32	32.59	27.52	209.68	94.36	144.1	87.91	223.49	30.44	151.908
ENSMUSG00000035186	Ubd	ubiquitin D [Source:MGI Symbol;Acc:MGI:1344410]	0.186081131	-2.425996321	1.29901E-08	4.15966E-07	yes	down	2.88	1.86	0.97	1.48	11.86	3.72	6.34	4.61	18.68	1.79	9.042
ENSMUSG00000076543	Igkv4-74	immunoglobulin kappa variable 4-74 [Source:MGI Symbol;Acc:MGI:3779447]	0.18446781	-2.438559007	0.000478781	0.003686771	yes	down	48.19	184.67	125.88	202	1251.78	596.53	76.33	1193.62	266.83	132.37	677.018
ENSMUSG00000094051	Ighv1-36	immunoglobulin heavy variable 1-36 [Source:MGI Symbol;Acc:MGI:4439639]	0.183891136	-2.443076151	0.003118977	0.016974322	yes	down	15.53	6.59	7.22	14.41	171.17	15.23	5.85	38.31	23.87	9.996	50.886
ENSMUSG00000114399	Gm47994	predicted gene, 47994 [Source:MGI Symbol;Acc:MGI:6097290]	0.179953212	-2.47430624	0.010565217	0.044929065	yes	down	0	0	0	0.31	1.01	0.56	0.46	0.81	0.66	0.138	0.7
ENSMUSG00000024842	Cabp4	calcium binding protein 4 [Source:MGI Symbol;Acc:MGI:1920910]	0.178603322	-2.48516918	0.001474222	0.009234268	yes	down	0.02	0.06	0.03	0.08	0.3	0.08	0.22	0.18	0.45	0.038	0.246
ENSMUSG00000027748	Trpc4	transient receptor potential cation channel, subfamily C, member 4 [Source:MGI Symbol;Acc:MGI:109525]	0.173580976	-2.526319258	1.85607E-06	3.236E-05	yes	down	0.54	0.14	0.15	0.05	8.06	1.85	1.27	0.38	1.31	0.18	2.574
ENSMUSG00000095007	Igkv12-41	immunoglobulin kappa chain variable 12-41 [Source:MGI Symbol;Acc:MGI:4439772]	0.173400381	-2.527821022	0.004833447	0.02432295	yes	down	97.74	56.11	21.3	35.06	223.32	49.21	18.36	689.23	273.24	44.754	250.672
ENSMUSG00000102301	Ighv8-2	immunoglobulin heavy variable V8-2 [Source:MGI Symbol;Acc:MGI:3647474]	0.16903313	-2.564622052	0.005522662	0.026992342	yes	down	1.11	1.11	0.61	1.9	0.62	10.69	11.91	1.22	4.52	1.06	5.792
ENSMUSG00000063388	BC023105	cDNA sequence BC023105 [Source:MGI Symbol;Acc:MGI:2384767]	0.167757088	-2.575554373	5.18824E-08	1.40992E-06	yes	down	0.5	0.05	0.56	0.54	4.3	1.33	1.95	2.07	3.54	0.482	2.638
ENSMUSG00000030402	Ppm1n	protein phosphatase, Mg2+/Mn2+ dependent, 1N (putative) [Source:MGI Symbol;Acc:MGI:2142330]	0.16305234	-2.616592951	0.00024301	0.002089015	yes	down	0.07	0.18	0.09	0	0.36	0.47	0.7	0.69	0.95	0.152	0.634
ENSMUSG00000031594	Fgl1	fibrinogen-like protein 1 [Source:MGI Symbol;Acc:MGI:102795]	0.162284972	-2.623398682	0.000746582	0.005302799	yes	down	0.22	0.06	0.23	0.36	0.12	1.32	2.51	0.83	0.5	0.186	1.056
ENSMUSG00000029819	Npy	neuropeptide Y [Source:MGI Symbol;Acc:MGI:97374]	0.160051216	-2.643394453	9.42111E-19	2.15485E-16	yes	down	16.49	6.96	6.29	6.05	78.7	33.65	41.07	45.34	70.43	9.168	53.838
ENSMUSG00000039691	Tspan10	tetraspanin 10 [Source:MGI Symbol;Acc:MGI:2384781]	0.158957241	-2.653289362	0.000276017	0.002330015	yes	down	0.35	0.26	0.03	0	0.74	0.23	0.55	0.74	0.96	0.134	0.644
ENSMUSG00000069305	Hist1h4n	histone cluster 1, H4n [Source:MGI Symbol;Acc:MGI:4843992]	0.155472637	-2.685267407	0.002676346	0.015030877	yes	down	0	1.34	0	1.5	3.34	2.85	4.82	0.68	8.57	0.724	4.052
ENSMUSG00000095197	Ighv1-59	immunoglobulin heavy variable V1-59 [Source:MGI Symbol;Acc:MGI:3644474]	0.153350713	-2.70509322	0.001735109	0.010539567	yes	down	99.98	43.12	67.71	28.13	827.72	23.24	130.59	559.04	84.47	52.726	325.012
ENSMUSG00000110368	Gm45518	predicted gene 45518 [Source:MGI Symbol;Acc:MGI:5791354]	0.151057402	-2.726831217	0.00413263	0.021338412	yes	down	0.08	0	0	0.02	0.24	0.51	0.08	0.13	0.09	0.032	0.21
ENSMUSG00000039716	Dock3	dedicator of cyto-kinesis 3 [Source:MGI Symbol;Acc:MGI:2429763]	0.149633398	-2.740495874	1.38405E-05	0.000185308	yes	down	0.03	0	0.01	0.37	0.29	24.92	0.09	0.21	0.43	0.088	5.188
ENSMUSG00000022126	Acod1	aconitate decarboxylase 1 [Source:MGI Symbol;Acc:MGI:103206]	0.148104265	-2.755314904	2.83913E-15	3.66095E-13	yes	down	0.91	0.39	0.95	0.53	4.09	2.92	3.08	2.44	9.08	0.686	4.322
ENSMUSG00000045225	Olfr1152	olfactory receptor 1152 [Source:MGI Symbol;Acc:MGI:3030986]	0.14594279	-2.776525151	0.009210397	0.040346411	yes	down	0.02	0	0	0	0.07	0.07	0.05	0.02	0.04	0.008	0.05
ENSMUSG00000110141	Gm45684	predicted gene 45684 [Source:MGI Symbol;Acc:MGI:5791520]	0.145793847	-2.777998256	0.002196607	0.012804435	yes	down	0.11	0	0	0.08	0.84	0.33	0.25	0.08	0.61	0.068	0.422
ENSMUSG00000108447	Gm44567	predicted gene 44567 [Source:MGI Symbol;Acc:MGI:5753143]	0.144196107	-2.793895883	0.007126688	0.033001944	yes	down	0.04	0.02	0.02	0.02	0.17	0.2	0.06	0.02	0.22	0.02	0.134
ENSMUSG00000094345	Igkv14-126	immunoglobulin kappa variable 14-126 [Source:MGI Symbol;Acc:MGI:3643131]	0.139256371	-2.844184763	0.000760574	0.00538427	yes	down	29.58	42.49	49.54	15.98	665.58	23.3	36.02	108.29	249.44	32.01	216.526
ENSMUSG00000105954	Gm42793	predicted gene 42793 [Source:MGI Symbol;Acc:MGI:5662930]	0.138734739	-2.849599011	0.003472713	0.018569786	yes	down	0.14	0	0.05	0.05	1.23	0.19	0.24	0.1	0.53	0.068	0.458
ENSMUSG00000113553	Gm40932	predicted gene, 40932 [Source:MGI Symbol;Acc:MGI:5623817]	0.135722041	-2.881273061	0.005835512	0.028248348	yes	down	0.44	0	0	0	3.76	1.27	0.42	1.4	3.64	0.3	2.098
ENSMUSG00000020017	Hal	histidine ammonia lyase [Source:MGI Symbol;Acc:MGI:96010]	0.13568521	-2.881664619	0.003937113	0.020503134	yes	down	0	0	0.01	0.18	0.12	0.19	0.07	0.07	0.07	0.072	0.104
ENSMUSG00000097248	Gm2694	predicted gene 2694 [Source:MGI Symbol;Acc:MGI:3780864]	0.130089762	-2.942420668	0.002696423	0.015099499	yes	down	0	0	0	0.29	1.56	1.48	0.51	2.24	2.88	0.238	1.734
ENSMUSG00000087077	Gm12480	predicted gene 12480 [Source:MGI Symbol;Acc:MGI:3651952]	0.129785853	-2.945794957	0.007438681	0.034158551	yes	down	0.1	0.17	0	0.2	1.07	0.11	2.52	0.12	0.51	0.114	0.866
ENSMUSG00000028262	Clca3a2	chloride channel accessory 3A2 [Source:MGI Symbol;Acc:MGI:1931471]	0.12909889	-2.953451501	0.006646117	0.031304695	yes	down	0.01	0.29	0	0	0.19	0	0.41	0.09	0.28	0.068	0.194
ENSMUSG00000027718	Il21	interleukin 21 [Source:MGI Symbol;Acc:MGI:1890474]	0.127356088	-2.973060173	0.000649499	0.004750221	yes	down	0.07	0.07	0	0.04	0.77	0.02	0.28	0.19	0.25	0.04	0.302

ENSMUSG00000062393	Dgkk	diacylglycerol kinase kappa [Source:MGI Symbol;Acc:MGI:3580254]	0.123365408	-3.018990175	0.001784142	0.010789648	yes	down	0.01	0.01	0	0.02	0.1	0.03	0.11	0.01	0.07	0.01	0.064
ENSMUSG00000052613	Pcdh15	protocadherin 15 [Source:MGI Symbol;Acc:MGI:1891428]	0.120351426	-3.054674857	0.005925979	0.028563932	yes	down	0.03	0.01	0.02	0	0.24	0	0.02	0.03	0.16	0.014	0.09
ENSMUSG00000097418	Mir155hg	Mir155 host gene (non-protein coding) [Source:MGI Symbol;Acc:MGI:5477161]	0.11778563	-3.085764554	9.10044E-05	0.000912435	yes	down	0.14	0.28	0.15	0.05	2.22	1.36	0.28	0.3	0.9	0.124	1.012
ENSMUSG00000103738	Gm37652	predicted gene, 37652 [Source:MGI Symbol;Acc:MGI:5610880]	0.117192078	-3.093053048	0.008418573	0.037545507	yes	down	0.03	0	0	0.06	0.19	0.2	0	0.22	0.23	0.024	0.168
ENSMUSG00000063887	Nlgn1	neuroligin 1 [Source:MGI Symbol;Acc:MGI:2179435]	0.116049669	-3.107185684	0.003374059	0.018133092	yes	down	0.02	0	0.03	0	0.01	0.16	0.03	0.15	0.13	0.01	0.096
ENSMUSG00000105607	Gm43513	predicted gene 43513 [Source:MGI Symbol;Acc:MGI:5663650]	0.109853894	-3.186342081	0.008138572	0.03661976	yes	down	0	0.12	0.06	0	0.38	0.47	0.12	0.25	0.35	0.036	0.314
ENSMUSG00000029417	Cxcl9	chemokine (C-X-C motif) ligand 9 [Source:MGI Symbol;Acc:MGI:1352449]	0.108896875	-3.198965546	3.71883E-09	1.33551E-07	yes	down	6.99	4.47	3	3.8	59.77	15.44	19.86	17.89	78.33	4.446	38.258
ENSMUSG00000079455	Gm16026	predicted pseudogene 16026 [Source:MGI Symbol;Acc:MGI:3783243]	0.100462126	-3.315276387	0.007145249	0.03307195	yes	down	0.23	0	0.1	0	0.54	0.58	0.09	0.2	0.76	0.066	0.434
ENSMUSG00000085894	Gm15832	predicted gene 15832 [Source:MGI Symbol;Acc:MGI:3834078]	0.096711799	-3.370164281	1.3566E-06	2.45428E-05	yes	down	0.14	0.32	0	0.33	3.69	1.56	0.86	0.81	0.92	0.158	1.568
ENSMUSG00000104761	Gm43511	predicted gene 43511 [Source:MGI Symbol;Acc:MGI:5663648]	0.089718285	-3.478454153	0.000675623	0.004911365	yes	down	0.1	0	0	0.02	0.46	0.18	0.32	0.07	0.18	0.024	0.242
ENSMUSG00000093966	Trav4-3	T cell receptor alpha variable 4-3 [Source:MGI Symbol;Acc:MGI:4440478]	0.08914245	-3.487743583	0.00084848	0.005876658	yes	down	0	1.08	0	0.41	2.01	1.83	2.83	3.55	5.88	0.298	3.22
ENSMUSG00000027762	Sucnr1	succinate receptor 1 [Source:MGI Symbol;Acc:MGI:1934135]	0.087573343	-3.513364409	0.010462035	0.044618663	yes	down	0	0	0	0.12	0.08	0.04	0.67	0.15	0.25	0.024	0.238
ENSMUSG00000071311	Gpr31b	G protein-coupled receptor 31, D17Leh66b region [Source:MGI Symbol;Acc:MGI:1354372]	0.086028906	-3.539034703	0.002189077	0.012781119	yes	down	0	0	0.05	0.1	0.45	0.48	0.17	0.15	0.3	0.03	0.31
ENSMUSG00000027400	Pdyn	prodynorphin [Source:MGI Symbol;Acc:MGI:97535]	0.06932409	-3.850499414	0.002341583	0.013510158	yes	down	0.02	0.02	0	0	0.14	0.13	0.17	0.02	0.15	0.008	0.122
ENSMUSG00000095737	Igkv11-125	immunoglobulin kappa variable 11-125 [Source:MGI Symbol;Acc:MGI:3642338]	0.061716966	-4.018189049	4.53061E-15	5.50928E-13	yes	down	18.16	21.13	7.07	26.89	269.04	162.96	122.2	227.58	426.27	15.738	241.61
ENSMUSG00000060183	Cxcl11	chemokine (C-X-C motif) ligand 11 [Source:MGI Symbol;Acc:MGI:1860203]	0.058180126	-4.103329777	0.007723893	0.03518235	yes	down	0	0.07	0	0	0.11	0	0.05	0.04	0.65	0.014	0.17
ENSMUSG00000037771	Slc32a1	solute carrier family 32 (GABA vesicular transporter), member 1 [Source:MGI Symbol;Acc:MGI:1194488]	0.04842615	-4.368069877	0.001056086	0.007047785	yes	down	0	0	0	0	0.08	0.19	0.11	0.1	0.02	0.004	0.1
ENSMUSG00000042671	Rgs8	regulator of G-protein signaling 8 [Source:MGI Symbol;Acc:MGI:108408]	0.047560164	-4.394102512	0.002969974	0.016336134	yes	down	0	0.01	0	0	0.06	0	0.03	0.03	0.28	0.002	0.08
ENSMUSG00000102286	Gm37004	predicted gene, 37004 [Source:MGI Symbol;Acc:MGI:5610232]	0.031694716	-4.979613827	0.008234487	0.03693439	yes	down	0	0	0	0	0	0	22.25	42.34	2.34	0	13.386
ENSMUSG00000105242	Gm9236	predicted gene 9236 [Source:MGI Symbol;Acc:MGI:3645301]	0.01619643	-5.948180312	2.28358E-07	5.17998E-06	yes	down	0	0.86	0	0	10.74	9.53	7.9	13.9	51.07	0.346	18.628
ENSMUSG00000093906	Igkv9-129	immunoglobulin kappa variable 9-129 [Source:MGI Symbol;Acc:MGI:3525017]	0.013302295	-6.232181058	4.17957E-06	6.59837E-05	yes	down	1.76	0	0	0	55.91	91.33	2.36	5.17	20.39	0.474	35.032

Table S4-3　360 differentially expressed genes between con+LPS+mel+luz and con+LPS+mel.

Gene_id	Gene name	Gene description	FC(luz/mel)	Log2FC(luz/mel)	Pvalue	Padjust	Significant	Regulate	luz	mel	luz_1	luz_2	luz_3	luz_7	luz_8	mel_1	mel_3	mel_5	mel_6	mel_8
ENSMUSG00000102099	1700011B04Rik	RIKEN cDNA 1700011B04 gene [Source:MGI Symbol;Acc:MGI:1922894]	1000	9.965784285	1.32E-04	2.01E-03	yes	up	14.726	0	58.82	0.17	12.17	0	2.47	0	0	0	0	0
ENSMUSG00000071311	Gpr31b	G protein-coupled receptor 31, D17Leh66b region [Source:MGI Symbol;Acc:MGI:1354372]	45.45454545	5.506352666	2.34E-06	7.63E-05	yes	up	1.254	0.03	4	0.68	0.82	0.1	0.67	0	0	0	0.05	0.1
ENSMUSG00000030680	Pagr1a	PAXIP1 associated glutamate rich protein 1A [Source:MGI Symbol;Acc:MGI:1914528]	28.57142857	4.836501268	1.55E-03	1.40E-02	yes	up	6.114	0.732	4.04	12.86	0	11.84	1.83	0	0.43	2.18	0.52	0.53
ENSMUSG00000022126	Acod1	aconitate decarboxylase 1 [Source:MGI Symbol;Acc:MGI:103206]	21.73913043	4.442222329	3.72E-12	1.11E-09	yes	up	14.244	0.686	42.14	8.2	11.45	5.02	4.41	0.65	0.91	0.39	0.95	0.53
ENSMUSG00000048498	Cd300e	CD300E molecule [Source:MGI Symbol;Acc:MGI:2387602]	14.49275362	3.857259828	1.72E-06	5.94E-05	yes	up	33.82	2.426	101.12	3.54	41.44	4.5	18.5	2.64	2.61	1.63	2.65	2.6
ENSMUSG00000076536	Igkv4-86	immunoglobulin kappa variable 4-86 [Source:MGI Symbol;Acc:MGI:2685305]	14.49275362	3.857259828	9.12E-06	2.27E-04	yes	up	109.796	8.43	67.04	29.14	26.52	355.78	70.5	18.75	9.33	8.4	3.55	2.12
ENSMUSG00000063234	Gpr84	G protein-coupled receptor 84 [Source:MGI Symbol;Acc:MGI:1934129]	13.51351351	3.756330919	1.19E-07	6.92E-06	yes	up	1.896	0.144	6.34	0.63	1.43	0.36	0.72	0.09	0.23	0.13	0.13	0.14
ENSMUSG00000016529	Il10	interleukin 10 [Source:MGI Symbol;Acc:MGI:96537]	11.62790698	3.53951953	2.49E-03	2.01E-02	yes	up	3.916	0.35	12.43	0	5.34	0.7	1.11	0.65	0.36	0.36	0.23	0.15
ENSMUSG00000105373	Gm42429	predicted gene 42429 [Source:MGI Symbol;Acc:MGI:5662566]	11.49425287	3.522840789	5.74E-03	3.81E-02	yes	up	2.94	0.308	4	0	0	4.79	5.91	0.93	0.16	0	0	0.45
ENSMUSG00000111792	Gm33858	predicted gene, 33858 [Source:MGI Symbol;Acc:MGI:5593017]	11.11111111	3.473931188	1.16E-04	1.82E-03	yes	up	1.296	0.12	3.84	0.9	1.34	0.17	0.23	0.04	0.04	0.25	0	0.27
ENSMUSG00000018930	Ccl4	chemokine (C-C motif) ligand 4 [Source:MGI Symbol;Acc:MGI:98261]	9.615384615	3.265344567	3.80E-09	4.06E-07	yes	up	15.912	1.726	42	5.14	13.68	4.65	14.09	0.74	1.23	0.96	2.06	3.64
ENSMUSG00000024401	Tnf	tumor necrosis factor [Source:MGI Symbol;Acc:MGI:104798]	9.523809524	3.251538767	8.57E-08	5.25E-06	yes	up	2.044	0.234	4.82	1.19	2.85	0.56	0.8	0.48	0.21	0.2	0.07	0.21
ENSMUSG00000030142	Clec4e	C-type lectin domain family 4, member e [Source:MGI Symbol;Acc:MGI:1861232]	9.174311927	3.19759996	1.37E-06	4.97E-05	yes	up	61.014	6.728	174.27	16.28	74.74	15.82	23.96	9.7	8.42	5.06	6.07	4.39
ENSMUSG00000041754	Trem3	triggering receptor expressed on myeloid cells 3 [Source:MGI Symbol;Acc:MGI:1930003]	8.130081301	3.023269779	2.73E-05	5.60E-04	yes	up	14.042	1.788	38.58	2.41	17.19	5.59	6.44	1.27	3.64	1.17	1.58	1.28
ENSMUSG00000110018	5430437J10Rik	RIKEN cDNA 5430437J10 gene [Source:MGI Symbol;Acc:MGI:1918682]	8.064516129	3.011587974	1.86E-07	1.01E-05	yes	up	4.042	0.492	9.87	2.5	5.28	1.88	0.68	0.41	0.71	0.58	0.27	0.49
ENSMUSG00000033227	Wnt6	wingless-type MMTV integration site family, member 6 [Source:MGI Symbol;Acc:MGI:98960]	7.751937984	2.954557029	3.52E-04	4.45E-03	yes	up	1.51	0.188	4.18	0.47	2.13	0.07	0.7	0.43	0.13	0.19	0.03	0.16
ENSMUSG00000051682	Treml4	triggering receptor expressed on myeloid cells-like 4 [Source:MGI Symbol;Acc:MGI:1923239]	7.575757576	2.921390165	3.27E-05	6.48E-04	yes	up	50.548	6.48	145.88	8.74	65.41	10.93	21.78	7.18	7.39	4.67	6.65	6.51
ENSMUSG00000032915	Adgre4	adhesion G protein-coupled receptor E4 [Source:MGI Symbol;Acc:MGI:1196464]	7.352941176	2.878321443	8.95E-06	2.24E-04	yes	up	32.348	4.818	83.53	6.71	46.57	7.75	17.18	5.78	5	3.32	5.32	4.67
ENSMUSG00000076512	Igkv9-123	immunoglobulin kappa variable 9-123 [Source:MGI Symbol;Acc:MGI:3643848]	7.352941176	2.878321443	3.56E-03	2.66E-02	yes	up	57.614	9.7	6.19	4.27	54.03	75.7	147.88	16.96	7.38	12.89	9.21	2.06
ENSMUSG00000094322	Ighv9-4	immunoglobulin heavy variable 9-4 [Source:MGI Symbol;Acc:MGI:3646379]	7.299270073	2.867752202	5.87E-03	3.88E-02	yes	up	182.25	21.206	688.48	27.51	107.79	71.88	15.59	10.49	11.37	63.53	9	11.64
ENSMUSG00000117239	AC166110.3	G protein-coupled receptor 31, D17Leh66b region (Gpr31b) pseudogene	7.194244604	2.846843212	5.02E-06	1.39E-04	yes	up	2.248	0.326	6.57	0.91	1.87	0.98	0.91	0.38	0.48	0.3	0.19	0.28
ENSMUSG00000044162	Tnip3	TNFAIP3 interacting protein 3 [Source:MGI Symbol;Acc:MGI:3041165]	6.993006993	2.805912948	1.16E-06	4.37E-05	yes	up	8.892	1.366	23.8	3.33	10.64	2.85	3.84	1.29	1.73	0.78	1.53	1.5
ENSMUSG00000101389	Ms4a4a	membrane-spanning 4-domains, subfamily A, member 4A [Source:MGI Symbol;Acc:MGI:3643932]	6.802721088	2.76611194	2.65E-05	5.44E-04	yes	up	70.536	10.818	207.82	14.05	73.53	25.39	31.89	9.14	9.63	10.57	13.46	11.29

ENSMUSG00000115869	Gm31814	predicted gene, 31814 [Source:MGI Symbol;Acc:MGI:5590973]	6.622516556	2.727379545	1.90E-05	4.11E-04	yes	up	15.462	1.282	49.16	4.74	11.29	6.54	5.58	0.39	1.58	1.02	2.96	0.46
ENSMUSG00000016498	Pdcd1lg2	programmed cell death 1 ligand 2 [Source:MGI Symbol;Acc:MGI:1930125]	5.747126437	2.522840789	1.25E-06	4.64E-05	yes	up	4.264	0.77	12.07	2.16	3.45	1.4	2.24	0.98	0.69	1.28	0.46	0.44
ENSMUSG00000029819	Npy	neuropeptide Y [Source:MGI Symbol;Acc:MGI:97374]	5.555555556	2.473931188	1.82E-04	2.64E-03	yes	up	48.804	9.168	14.01	130.68	25.42	58.79	15.12	10.05	16.49	6.96	6.29	6.05
ENSMUSG00000108847	B830042I05Rik	RIKEN cDNA B830042I05 gene [Source:MGI Symbol;Acc:MGI:1925942]	5.405405405	2.434402824	4.94E-04	5.80E-03	yes	up	0.73	0.142	2.13	0.26	0.58	0.32	0.36	0.21	0.14	0.1	0.18	0.08
ENSMUSG00000002992	Apoc2	apolipoprotein C-II [Source:MGI Symbol;Acc:MGI:88054]	5.235602094	2.388355457	8.23E-20	1.07E-16	yes	up	32.108	6.396	48.89	22.08	40.59	25.66	23.32	6.56	7.93	7.43	5.4	4.66
ENSMUSG00000115855	Gm34643	predicted gene, 34643 [Source:MGI Symbol;Acc:MGI:5593802]	5.18134715	2.373327247	6.37E-03	4.11E-02	yes	up	0.344	0.07	0.78	0.28	0.21	0.4	0.05	0.09	0.14	0	0.12	0
ENSMUSG00000092517	Art2a-ps	ADP-ribosyltransferase 2a, pseudogene [Source:MGI Symbol;Acc:MGI:107546]	5.050505051	2.336427665	7.89E-05	1.33E-03	yes	up	2.03	0.452	5.35	1.61	1.19	1.18	0.82	0.16	0.34	0.47	0.72	0.57
ENSMUSG00000074141	Il4i1	interleukin 4 induced 1 [Source:MGI Symbol;Acc:MGI:109552]	4.95049505	2.307572802	1.06E-06	4.01E-05	yes	up	16.178	3.664	42.41	12.73	10.82	8.42	6.51	4.26	3.96	2.44	4.27	3.39
ENSMUSG00000027004	Frzb	frizzled-related protein [Source:MGI Symbol;Acc:MGI:892032]	4.926108374	2.300448367	1.53E-07	8.58E-06	yes	up	0.53	0.126	0.66	0.47	0.44	0.41	0.67	0.1	0.03	0.18	0.15	0.17
ENSMUSG00000029417	Cxcl9	chemokine (C-X-C motif) ligand 9 [Source:MGI Symbol;Acc:MGI:1352449]	4.854368932	2.279283757	5.40E-10	7.97E-08	yes	up	20.652	4.446	28.73	31.99	10.9	19.52	12.12	3.97	6.99	4.47	3	3.8
ENSMUSG00000004359	Spic	Spi-C transcription factor (Spi-1/PU.1 related) [Source:MGI Symbol;Acc:MGI:1341168]	4.807692308	2.265344567	8.26E-07	3.30E-05	yes	up	3.576	0.916	8.96	2.33	3.39	1.23	1.97	0.68	0.51	1.25	0.72	1.42
ENSMUSG00000035186	Ubd	ubiquitin D [Source:MGI Symbol;Acc:MGI:1344410]	4.761904762	2.251538767	4.66E-07	2.15E-05	yes	up	8.19	1.79	14.56	11.79	2.35	8.47	3.78	1.76	2.88	1.86	0.97	1.48
ENSMUSG00000104686	Gm42815	predicted gene 42815 [Source:MGI Symbol;Acc:MGI:5662952]	4.761904762	2.251538767	2.85E-04	3.79E-03	yes	up	0.918	0.192	1.17	1.62	0.73	0.51	0.56	0.11	0.18	0.41	0	0.26
ENSMUSG00000094051	Ighv1-36	immunoglobulin heavy variable 1-36 [Source:MGI Symbol;Acc:MGI:4439639]	4.716981132	2.23786383	5.05E-06	1.40E-04	yes	up	44.73	9.996	20.85	68.31	83.55	12.5	38.44	6.23	15.53	6.59	7.22	14.41
ENSMUSG00000009185	Ccl8	chemokine (C-C motif) ligand 8 [Source:MGI Symbol;Acc:MGI:101878]	4.62962963	2.210896782	4.05582E-25	3.30566E-21	yes	up	133.956	30.44	192.39	152.82	97.39	147.21	79.97	25.46	37.31	29.32	32.59	27.52
ENSMUSG00000034855	Cxcl10	chemokine (C-X-C motif) ligand 10 [Source:MGI Symbol;Acc:MGI:1352450]	4.608294931	2.204233052	6.31E-07	2.67E-05	yes	up	21.986	5.02	48.75	20.27	16.55	15.52	8.84	5.97	7.35	3.7	3.55	4.53
ENSMUSG00000114432	Gm49391	predicted gene, 49391 [Source:MGI Symbol;Acc:MGI:6121619]	4.587155963	2.19759996	5.63E-03	3.76E-02	yes	up	1.014	0.248	0.44	1.12	0.4	0.57	2.54	0	0.07	0.34	0.49	0.34
ENSMUSG00000034387	Ssu2	ssu-2 homolog (C. elegans) [Source:MGI Symbol;Acc:MGI:2443733]	4.566210046	2.190997225	1.06E-03	1.05E-02	yes	up	1.502	0.344	3.68	0.95	1.48	0.4	1	0.17	0.37	0.39	0.26	0.53
ENSMUSG00000095583	Ighv14-2	immunoglobulin heavy variable 14-2 [Source:MGI Symbol;Acc:MGI:4439607]	4.545454545	2.184424571	1.38E-05	3.22E-04	yes	up	345.874	79.034	549.56	189.19	282.86	201.95	505.81	39.48	134.43	104.39	33.71	83.16
ENSMUSG00000093838	Ighv3-1	immunoglobulin heavy variable 3-1 [Source:MGI Symbol;Acc:MGI:4439534]	4.545454545	2.184424571	8.02E-04	8.46E-03	yes	up	415.068	94.096	277.56	395.57	497.92	381.62	522.67	105.37	169.85	153.63	27.95	13.68
ENSMUSG00000076733	Ighv8-13	immunoglobulin heavy variable 8-13 [Source:MGI Symbol;Acc:MGI:4439734]	4.504504505	2.171368418	1.41E-04	2.14E-03	yes	up	46.264	10.83	14.45	94.01	17.8	39.05	66.01	4.35	11.51	18.94	7.06	12.29
ENSMUSG00000117694	Gm28285	novel protein, identical to matrin 3 (Matr3)	4.504504505	2.171368418	7.51E-04	8.02E-03	yes	up	2.81	0.472	2.89	4.89	4.21	0.58	1.48	0.22	0.54	0.74	0.61	0.25
ENSMUSG00000103847	Gm20056	predicted gene, 20056 [Source:MGI Symbol;Acc:MGI:5012241]	4.385964912	2.13289427	2.86E-04	3.80E-03	yes	up	4.312	1.056	4.34	6.34	5.67	2.05	3.16	0.62	0.6	0.2	2.53	1.33
ENSMUSG00000096719	Mrgpra2b	MAS-related GPR, member A2B [Source:MGI Symbol;Acc:MGI:3033098]	4.385964912	2.13289427	3.08E-03	2.38E-02	yes	up	1.362	0.336	0.77	0.36	1.72	3.23	0.73	0	0.7	0.42	0.24	0.32
ENSMUSG00000045087	S1pr5	sphingosine-1-phosphate receptor 5 [Source:MGI Symbol;Acc:MGI:2150641]	4.310344828	2.10780329	1.15E-04	1.80E-03	yes	up	4.98	1.206	11.51	2.04	6.57	1.22	3.56	1.38	1.4	1.06	1.13	1.06
ENSMUSG00000089722	Cd300ld5	CD300 molecule like family member D5 [Source:MGI Symbol;Acc:MGI:3702661]	4.273504274	2.095419565	7.07E-06	1.83E-04	yes	up	24.184	5.888	59.31	12.32	22.91	12.71	13.67	5.14	7.28	5.1	6.65	5.27
ENSMUSG00000019874	Fabp7	fatty acid binding protein 7, brain [Source:MGI Symbol;Acc:MGI:101916]	4.237288136	2.083141235	2.52E-05	5.25E-04	yes	up	5.064	1.27	3.26	10.8	3.74	5.41	2.11	1.83	1.62	0.45	1.86	0.59
ENSMUSG00000027748	Trpc4	transient receptor potential cation channel, subfamily C, member 4 [Source:MGI Symbol;Acc:MGI:109525]	4.237288136	2.083141235	7.19E-04	7.73E-03	yes	up	1.186	0.18	0.99	2.86	0.45	1.31	0.32	0.02	0.54	0.14	0.15	0.05
ENSMUSG00000027338	Prnd	prion like protein doppel [Source:MGI Symbol;Acc:MGI:1346999]	4.201680672	2.070966521	6.16E-03	4.01E-02	yes	up	14.8	3.592	40.26	5.15	21.19	3.08	4.32	4.79	5	1.09	2.41	4.67
ENSMUSG00000049848	Ceacam19	carcinoembryonic antigen-related cell adhesion molecule 19 [Source:MGI Symbol;Acc:MGI:2443001]	4.149377593	2.052894948	7.08E-04	7.66E-03	yes	up	0.35	0.088	0.57	0.31	0.54	0.25	0.08	0.09	0.14	0.01	0.14	0.06

ENSMUSG00000027737	Slc7a11	solute carrier family 7 (cationic amino acid transporter, y+ system), member 11 [Source:MGI Symbol;Acc:MGI:1347355]	4.098360656	2.035046947	1.00E-05	2.48E-04	yes	up	6.298	1.626	13.47	4.53	8.16	2.24	3.09	1.97	1.7	1.53	1.71	1.22
ENSMUSG00000060600	Eno3	enolase 3, beta muscle [Source:MGI Symbol;Acc:MGI:95395]	4.016064257	2.005782353	7.38E-04	7.90E-03	yes	up	31.628	9.464	82.17	10.28	38.1	11.8	15.79	10.53	6.95	10.81	9.24	9.79
ENSMUSG00000034641	Cd300ld	CD300 molecule like family member d [Source:MGI Symbol;Acc:MGI:2442358]	4	2	3.91E-05	7.46E-04	yes	up	38.64	10.156	92.43	17.18	40.02	19.15	24.42	10.41	14.67	6.3	11.17	8.23
ENSMUSG00000079625	Tm4sf19	transmembrane 4 L six family member 19 [Source:MGI Symbol;Acc:MGI:3645933]	3.95256917	1.98279071	2.57E-06	8.24E-05	yes	up	2.268	0.608	3.65	2.23	2.47	1.61	1.38	0.88	0.14	0.47	0.93	0.62
ENSMUSG00000023913	Pla2g7	phospholipase A2, group VII (platelet-activating factor acetylhydrolase, plasma) [Source:MGI Symbol;Acc:MGI:1351327]	3.921568627	1.971430848	3.81E-09	4.06E-07	yes	up	167.23	44.534	314.68	132.01	185.32	104.4	99.74	42.07	63.9	39.57	44.65	32.48
ENSMUSG00000093861	Igkv1-110	immunoglobulin kappa variable 1-110 [Source:MGI Symbol;Acc:MGI:4439558]	3.861003861	1.948975997	3.89E-04	4.82E-03	yes	up	1271.274	341.122	900.35	1430.04	1085.79	1870.45	1069.74	319.73	427.93	662.07	69.85	226.03
ENSMUSG00000041653	Pnpla3	patatin-like phospholipase domain containing 3 [Source:MGI Symbol;Acc:MGI:2151796]	3.846153846	1.943416472	3.30E-03	2.50E-02	yes	up	0.764	0.214	1.75	0.69	0.98	0.23	0.17	0.13	0.16	0.3	0.23	0.25
ENSMUSG00000076552	Igkv4-61	immunoglobulin kappa chain variable 4-61 [Source:MGI Symbol;Acc:MGI:4439819]	3.816793893	1.932361283	2.36E-03	1.93E-02	yes	up	1039.416	237.534	299.2	400.37	2785.7	603.16	1108.65	114.4	232.36	508.8	144.26	187.85
ENSMUSG00000036067	Slc2a6	solute carrier family 2 (facilitated glucose transporter), member 6 [Source:MGI Symbol;Acc:MGI:2443286]	3.787878788	1.921390165	5.79E-11	1.19E-08	yes	up	10.486	3.106	18.46	7.82	11.45	8.28	6.42	2.66	5.1	2.31	2.84	2.62
ENSMUSG00000061540	Orm2	orosomucoid 2 [Source:MGI Symbol;Acc:MGI:97444]	3.745318352	1.905088353	4.86E-03	3.36E-02	yes	up	1.73	0.49	1.02	1.89	1.71	3.5	0.53	0	0.48	0.66	0.69	0.62
ENSMUSG00000049037	Clec4a1	C-type lectin domain family 4, member a1 [Source:MGI Symbol;Acc:MGI:3036291]	3.717472119	1.894321922	5.15E-06	1.42E-04	yes	up	42.61	12.01	94.59	18.16	47.38	25.95	26.97	12.73	11.95	11.05	12.19	12.13
ENSMUSG00000022534	Mefv	Mediterranean fever [Source:MGI Symbol;Acc:MGI:1859396]	3.6900369	1.883635243	2.08E-09	2.49E-07	yes	up	4.594	1.324	8.23	3.23	4.86	4.52	2.13	1.81	1.66	0.76	1.03	1.36
ENSMUSG00000079014	Serpina3i	serine (or cysteine) peptidase inhibitor, clade A, member 3I [Source:MGI Symbol;Acc:MGI:2182841]	3.663003663	1.873027144	4.27E-05	7.99E-04	yes	up	2.81	0.888	9.09	1.38	0.68	1.13	1.77	1.22	0.41	0.05	1.91	0.85
ENSMUSG00000079022	Col22a1	collagen, type XXII, alpha 1 [Source:MGI Symbol;Acc:MGI:1916950]	3.663003663	1.873027144	3.91E-04	4.85E-03	yes	up	0.146	0.04	0.12	0.16	0.19	0.23	0.03	0.05	0.04	0.04	0.02	0.05
ENSMUSG00000036931	Nfkbid	nuclear factor of kappa light polypeptide gene enhancer in B cells inhibitor, delta [Source:MGI Symbol;Acc:MGI:3041243]	3.636363636	1.862496476	2.49E-03	2.02E-02	yes	up	5.758	1.52	16.14	2.31	5.29	1.95	3.1	2.52	1.8	1.14	1.51	0.63
ENSMUSG00000069607	Cd300ld3	CD300 molecule like family member D3 [Source:MGI Symbol;Acc:MGI:2687214]	3.610108303	1.852042119	3.41E-04	4.33E-03	yes	up	62.556	17.344	162.81	25.96	58.91	27.99	37.11	19.19	18.33	14.09	17.81	17.3
ENSMUSG00000073412	Lst1	leukocyte specific transcript 1 [Source:MGI Symbol;Acc:MGI:1096324]	3.584229391	1.841662973	3.06E-09	3.53E-07	yes	up	126.194	37.156	256.3	58.69	140.26	84.64	91.08	50.95	35.52	28.46	34.42	36.43
ENSMUSG00000030268	Bcat1	branched chain aminotransferase 1, cytosolic [Source:MGI Symbol;Acc:MGI:104861]	3.584229391	1.841662973	4.49E-03	3.17E-02	yes	up	0.962	0.206	0.11	0.62	1.24	2.5	0.34	0.27	0.26	0.31	0.01	0.18
ENSMUSG00000079419	Ms4a6c	membrane-spanning 4-domains, subfamily A, member 6C [Source:MGI Symbol;Acc:MGI:2385644]	3.50877193	1.810966176	2.34E-05	4.92E-04	yes	up	122.72	35.476	273.05	63.17	135.49	67.97	73.92	41.43	39.26	28.56	34.93	33.2
ENSMUSG00000063388	BC023105	cDNA sequence BC023105 [Source:MGI Symbol;Acc:MGI:2384767]	3.50877193	1.810966176	1.40E-03	1.30E-02	yes	up	1.59	0.482	1.64	3.47	0.9	1.33	0.61	0.76	0.5	0.05	0.56	0.54
ENSMUSG00000030117	Gdf3	growth differentiation factor 3 [Source:MGI Symbol;Acc:MGI:95686]	3.484320557	1.800877358	6.62E-05	1.15E-03	yes	up	1.434	0.434	2.82	1.12	1.67	0.44	1.12	0.44	0.63	0.2	0.36	0.54
ENSMUSG00000109675	Nxpe1-ps	neurexophilin and PC-esterase domain family, member 1, pseudogene [Source:MGI Symbol;Acc:MGI:3646632]	3.436426117	1.780908942	1.97E-05	4.25E-04	yes	up	13.894	1.76	11.01	4.92	6.98	3.21	43.35	1.41	1.32	1.15	1.97	2.95
ENSMUSG00000095682	Igkv3-1	immunoglobulin kappa variable 3-1 [Source:MGI Symbol;Acc:MGI:1330851]	3.424657534	1.775959726	1.64E-03	1.46E-02	yes	up	401.116	123.378	457.4	166.57	863.25	337.52	180.84	62.17	77.47	183.57	74.49	219.19
ENSMUSG00000085885	Gm11906	predicted gene 11906 [Source:MGI Symbol;Acc:MGI:3705114]	3.424657534	1.775959726	1.72E-03	1.51E-02	yes	up	0.696	0.216	1.33	0.5	0.96	0.16	0.53	0.45	0.24	0.1	0.2	0.09
ENSMUSG00000085786	Gm15987	predicted gene 15987 [Source:MGI Symbol;Acc:MGI:3801849]	3.412969283	1.77102743	1.59E-08	1.35E-06	yes	up	24.58	7.342	44.18	12.92	33.85	15.44	16.51	7.99	7.18	5.44	8.96	7.14
ENSMUSG00000026177	Slc11a1	solute carrier family 11 (proton-coupled divalent metal ion transporters), member 1 [Source:MGI Symbol;Acc:MGI:1345275]	3.355704698	1.746615764	1.30E-07	7.48E-06	yes	up	81.616	28.84	145.54	76.39	90.05	45.31	50.79	35.82	27.06	28.49	29.91	22.92
ENSMUSG00000040026	Saa3	serum amyloid A 3 [Source:MGI Symbol;Acc:MGI:98223]	3.25732899	1.703689439	5.85E-25	3.31E-21	yes	up	600.65	193.39	736.76	550.59	690.76	576.88	448.26	185.72	244.61	211.13	189.13	136.36
ENSMUSG00000104452	Ighv8-8	immunoglobulin heavy variable 8-8 [Source:MGI Symbol;Acc:MGI:3815333]	3.25732899	1.703689439	2.16E-03	1.80E-02	yes	up	1124.396	361.784	453.34	938.21	1273.81	1389.07	1567.55	115.18	516.76	707.74	204.27	264.97
ENSMUSG00000114608	Gm36161	predicted gene, 36161 [Source:MGI Symbol;Acc:MGI:5595320]	3.154574132	1.657445255	6.15E-05	1.08E-03	yes	up	25.37	8.474	56.36	13.58	27.36	14.44	15.11	10.03	9.49	5.79	8.08	8.98

ENSMUSG00000040441	Slc26a10	solute carrier family 26, member 10 [Source:MGI Symbol;Acc:MGI:2143920]	3.105590062	1.634867407	5.64E-05	1.00E-03	yes	up	1.166	0.394	2.12	0.91	1.47	0.91	0.42	0.45	0.21	0.64	0.36	0.31
ENSMUSG00000066684	Pilrb1	paired immunoglobin-like type 2 receptor beta 1 [Source:MGI Symbol;Acc:MGI:2450532]	3.058103976	1.612637459	1.02E-06	3.94E-05	yes	up	14.442	5.16	28.54	8.17	16.67	9.98	8.85	5.62	6.94	3.06	4.41	5.77
ENSMUSG00000024210	Ip6k3	inositol hexaphosphate kinase 3 [Source:MGI Symbol;Acc:MGI:3045325]	3.058103976	1.612637459	4.97E-03	3.43E-02	yes	up	4.692	1.576	11.88	2.22	4.6	1.55	3.21	1.35	1.6	2.68	1.38	0.87
ENSMUSG00000048621	Gm6377	predicted gene 6377 [Source:MGI Symbol;Acc:MGI:3647255]	3.048780488	1.60823228	6.09E-03	3.98E-02	yes	up	5.276	1.606	14.74	2.06	4.79	1.38	3.41	2.35	1.38	1.17	1.78	1.35
ENSMUSG00000026678	Rgs5	regulator of G-protein signaling 5 [Source:MGI Symbol;Acc:MGI:1098434]	3.039513678	1.603840511	4.64E-04	5.54E-03	yes	up	12.004	4.124	27.64	6.48	13.83	5.21	6.86	5.52	3.39	4.34	3.91	3.46
ENSMUSG00000038352	Arl5c	ADP-ribosylation factor-like 5C [Source:MGI Symbol;Acc:MGI:3028577]	3.021148036	1.595096878	3.54E-08	2.57E-06	yes	up	17.258	6.244	30.78	13.65	23.16	9.37	9.33	8.51	5.34	4.29	6.19	6.89
ENSMUSG00000094797	Igkv6-15	immunoglobulin kappa variable 6-15 [Source:MGI Symbol;Acc:MGI:1330831]	3.003003003	1.586405918	6.97E-05	1.20E-03	yes	up	970.092	341.052	699.07	1000.3	682.96	1297.66	1170.47	224.89	500.07	513.2	241	226.1
ENSMUSG00000097855	A930007I19Rik	RIKEN cDNA A930007I19 gene [Source:MGI Symbol;Acc:MGI:1925029]	2.994011976	1.582079992	5.86E-06	1.57E-04	yes	up	8.548	7.21	15.51	14.36	6.18	2.8	3.89	1.45	6.21	2	2.8	23.59
ENSMUSG00000048031	Fcrl5	Fc receptor-like 5 [Source:MGI Symbol;Acc:MGI:3053558]	2.994011976	1.582079992	3.14E-03	2.41E-02	yes	up	2.91	0.438	2.87	9.98	0.46	0.83	0.41	0.52	0.26	0.69	0.22	0.5
ENSMUSG00000030638	Sh3gl3	SH3-domain GRB2-like 3 [Source:MGI Symbol;Acc:MGI:700011]	2.976190476	1.573466862	1.51E-03	1.38E-02	yes	up	2.3	0.89	5.38	1.74	1.98	1.08	1.32	0.35	0.99	1.14	0.85	1.12
ENSMUSG00000022322	Shcbp1	Shc SH2-domain binding protein 1 [Source:MGI Symbol;Acc:MGI:1338802]	2.96735905	1.569179503	4.24E-06	1.21E-04	yes	up	1.994	1.276	2.56	1.68	2.64	1.3	1.79	0.88	1.3	0.25	1.34	2.61
ENSMUSG00000029254	Stap1	signal transducing adaptor family member 1 [Source:MGI Symbol;Acc:MGI:1926193]	2.949852507	1.560642822	2.22E-04	3.11E-03	yes	up	15.468	14.504	35.54	11.31	14.8	7.48	8.21	6.02	5.91	3.93	5.09	51.57
ENSMUSG00000010461	Eya4	EYA transcriptional coactivator and phosphatase 4 [Source:MGI Symbol;Acc:MGI:1337104]	2.941176471	1.556393349	3.31E-06	9.97E-05	yes	up	0.386	0.132	0.49	0.41	0.38	0.32	0.33	0.07	0.15	0.1	0.19	0.15
ENSMUSG00000028885	Smpdl3b	sphingomyelin phosphodiesterase, acid-like 3B [Source:MGI Symbol;Acc:MGI:1916022]	2.941176471	1.556393349	2.85E-04	3.79E-03	yes	up	25.466	9.068	53.73	14.49	33.25	10.08	15.78	9.88	8.59	8.38	10.46	8.03
ENSMUSG00000022367	Has2	hyaluronan synthase 2 [Source:MGI Symbol;Acc:MGI:107821]	2.93255132	1.552156356	5.33E-03	3.61E-02	yes	up	0.258	0.092	0.33	0.32	0.29	0.23	0.12	0.14	0.16	0.01	0.11	0.04
ENSMUSG00000059108	Ifitm6	interferon induced transmembrane protein 6 [Source:MGI Symbol;Acc:MGI:2686976]	2.915451895	1.543719518	3.58E-07	1.74E-05	yes	up	28.632	10.48	38.54	15.49	36.37	32.87	19.89	8.96	14.33	4.54	10.67	13.9
ENSMUSG00000062148	Ear6	eosinophil-associated, ribonuclease A family, member 6 [Source:MGI Symbol;Acc:MGI:1890463]	2.906976744	1.53951953	6.20E-05	1.08E-03	yes	up	7.036	2.562	8.33	8.73	7.86	7.75	2.51	3.45	2.83	0.97	1.76	3.8
ENSMUSG00000049723	Mmp12	matrix metallopeptidase 12 [Source:MGI Symbol;Acc:MGI:97005]	2.898550725	1.535331733	1.10E-08	9.96E-07	yes	up	490.372	179.632	513.68	652.91	633.7	342.55	309.02	214.87	221.31	136.93	216.61	108.44
ENSMUSG00000069609	Cd300ld4	CD300 molecule like family member D4 [Source:MGI Symbol;Acc:MGI:3702658]	2.873563218	1.522840789	1.28E-04	1.98E-03	yes	up	2.106	1.654	1.67	2.63	2.21	1.9	2.12	2.63	1.34	1.94	0.27	2.09
ENSMUSG00000041064	Pif1	PIF1 5'-to-3' DNA helicase [Source:MGI Symbol;Acc:MGI:2143057]	2.849002849	1.510457064	1.32E-05	3.12E-04	yes	up	0.832	0.88	0.7	1.23	0.4	1.01	0.82	0.26	0.98	0.59	0.15	2.42
ENSMUSG00000050377	Il31ra	interleukin 31 receptor A [Source:MGI Symbol;Acc:MGI:2180511]	2.83286119	1.502259911	3.03E-03	2.35E-02	yes	up	0.33	0.196	0.16	0.23	0.72	0.26	0.28	0.39	0.08	0.25	0.12	0.14
ENSMUSG00000109350	Gm44805	predicted gene 44805 [Source:MGI Symbol;Acc:MGI:5753381]	2.777777778	1.473931188	5.11E-07	2.29E-05	yes	up	2.35	0.942	2.46	2.45	2.32	2.05	2.47	1.05	1.27	0.84	1.08	0.47
ENSMUSG00000024679	Ms4a6d	membrane-spanning 4-domains, subfamily A, member 6D [Source:MGI Symbol;Acc:MGI:1916024]	2.777777778	1.473931188	3.31E-05	6.54E-04	yes	up	98.538	37.008	207.02	68.98	100.01	54.68	62	39.36	40.03	37.19	36.88	31.58
ENSMUSG00000105747	Igkv13-64	immunoglobulin kappa chain variable 13-64 [Source:MGI Symbol;Acc:MGI:5009834]	2.777777778	1.473931188	2.49E-03	2.02E-02	yes	up	140.834	61.992	69.03	285.28	84.23	157.66	107.97	13.02	131.37	66.67	35.79	63.11
ENSMUSG00000035385	Ccl2	chemokine (C-C motif) ligand 2 [Source:MGI Symbol;Acc:MGI:98259]	2.770083102	1.469929258	2.93E-04	3.87E-03	yes	up	2.394	0.922	2.54	2.59	3.28	1.89	1.67	1.35	0.97	0.53	1.28	0.48
ENSMUSG00000086712	AI427809	expressed sequence AI427809 [Source:MGI Symbol;Acc:MGI:2140270]	2.762430939	1.465938398	4.93E-06	1.37E-04	yes	up	2.416	0.908	1.5	3.76	3.1	2.83	0.89	1.14	1.04	0.8	0.76	0.8
ENSMUSG00000096844	Igkv6-14	immunoglobulin kappa variable 6-14 [Source:MGI Symbol;Acc:MGI:1330830]	2.762430939	1.465938398	1.55E-03	1.40E-02	yes	up	169.828	65.06	182.62	193.47	79.74	269.13	124.18	29.56	119.67	71.84	58.76	45.47
ENSMUSG00000076596	Igkv3-10	immunoglobulin kappa variable 3-10 [Source:MGI Symbol;Acc:MGI:1330821]	2.762430939	1.465938398	4.98E-03	3.43E-02	yes	up	317.448	121.254	395.59	189.08	104.64	440.36	457.57	108.36	158.82	199.33	73.42	66.34
ENSMUSG00000035352	Ccl12	chemokine (C-C motif) ligand 12 [Source:MGI Symbol;Acc:MGI:108224]	2.754820937	1.461958547	1.04E-07	6.25E-06	yes	up	15.376	5.918	17.04	13.65	13.04	17.66	15.49	3.04	4.65	6.85	6.64	8.41
ENSMUSG00000047592	Nxpe5	neurexophilin and PC-esterase domain family, member 5 [Source:MGI Symbol;Acc:MGI:3584036]	2.754820937	1.461958547	1.39E-05	3.24E-04	yes	up	2.644	0.934	5.07	1.54	2.84	2.5	1.27	0.91	0.94	1.01	0.96	0.85

ENSMUSG00000026039	Sgo2a	shugoshin 2A [Source:MGI Symbol;Acc:MGI:1098767]	2.747252747	1.457989644	1.69E-10	2.86E-08	yes	up	1.096	0.418	1.23	1.18	1.38	0.79	0.9	0.3	0.44	0.35	0.44	0.56
ENSMUSG00000030786	Itgam	integrin alpha M [Source:MGI Symbol;Acc:MGI:96607]	2.739726027	1.454031631	3.72E-14	2.04E-11	yes	up	50.09	19.718	80.24	44.04	54.63	38.17	33.37	19.46	24.05	17.19	20.99	16.9
ENSMUSG00000068101	Cenpm	centromere protein M [Source:MGI Symbol;Acc:MGI:1913820]	2.739726027	1.454031631	3.64E-04	4.56E-03	yes	up	3.708	0.868	3.32	4.88	1.08	3.88	5.38	0.36	0.79	0.86	1.44	0.89
ENSMUSG00000095117	Ighv8-9	immunoglobulin heavy variable V8-9 [Source:MGI Symbol;Acc:MGI:3644475]	2.739726027	1.454031631	1.57E-03	1.41E-02	yes	up	473.814	180.842	266.58	581.77	347.25	519.94	653.53	99.41	240.71	322.12	114.74	127.23
ENSMUSG00000020826	Nos2	nitric oxide synthase 2, inducible [Source:MGI Symbol;Acc:MGI:97361]	2.732240437	1.450084446	9.93E-05	1.60E-03	yes	up	8.766	3.404	17.62	7.01	8.69	5.43	5.08	3.55	5.2	2.02	2.9	3.35
ENSMUSG00000026981	Il1rn	interleukin 1 receptor antagonist [Source:MGI Symbol;Acc:MGI:96547]	2.72479564	1.446148032	8.00E-07	3.23E-05	yes	up	48.942	20.338	69.6	45.95	68.18	31.28	29.7	23.98	25.69	13.11	23.44	15.47
ENSMUSG00000015854	Cd5l	CD5 antigen-like [Source:MGI Symbol;Acc:MGI:1334419]	2.7100271	1.438307279	7.44E-03	4.64E-02	yes	up	1.34	0.506	2.55	0.98	1.6	1.25	0.32	0.67	0.34	1.02	0.16	0.34
ENSMUSG00000051457	Spn	sialophorin [Source:MGI Symbol;Acc:MGI:98384]	2.702702703	1.434402824	4.11E-04	5.04E-03	yes	up	48.836	19.544	110.23	28.82	49.89	25.29	29.95	20.73	20.5	16.81	19.61	20.07
ENSMUSG00000095609	Gm21188	predicted gene, 21188 [Source:MGI Symbol;Acc:MGI:5434543]	2.69541779	1.430508908	2.43E-04	3.35E-03	yes	up	20.924	7.97	41.02	15.61	24.75	11.57	11.67	8.02	9.42	5.24	8.36	8.81
ENSMUSG00000109036	2210406H18Rik	RIKEN cDNA 2210406H18 gene [Source:MGI Symbol;Acc:MGI:1917383]	2.680965147	1.422752464	4.10E-04	5.02E-03	yes	up	2.86	1.112	5.09	1.35	3.3	2.54	2.02	0.92	1.75	1.29	0.87	0.73
ENSMUSG00000070304	Scn2b	sodium channel, voltage-gated, type II, beta [Source:MGI Symbol;Acc:MGI:106921]	2.673796791	1.418889825	6.85E-04	7.46E-03	yes	up	0.3	0.12	0.27	0.34	0.38	0.33	0.18	0.14	0.18	0.15	0.06	0.07
ENSMUSG00000023045	Soat2	sterol O-acyltransferase 2 [Source:MGI Symbol;Acc:MGI:1332226]	2.666666667	1.415037499	2.18E-03	1.81E-02	yes	up	0.752	0.298	0.99	0.65	0.38	0.96	0.78	0.29	0.55	0.19	0.1	0.36
ENSMUSG00000038508	Gdf15	growth differentiation factor 15 [Source:MGI Symbol;Acc:MGI:1346047]	2.638522427	1.399730246	4.83E-04	5.70E-03	yes	up	2.43	0.944	4.18	1.82	2.33	1.97	1.85	1.51	1.42	0.71	0.76	0.32
ENSMUSG00000044201	Cdc25c	cell division cycle 25C [Source:MGI Symbol;Acc:MGI:88350]	2.604166667	1.380821784	1.79E-03	1.56E-02	yes	up	0.878	0.46	1.07	1.59	0.6	0.54	0.59	0.2	0.76	0.22	0.67	0.45
ENSMUSG00000034773	BC030867	cDNA sequence BC030867 [Source:MGI Symbol;Acc:MGI:2387601]	2.604166667	1.380821784	2.59E-03	2.08E-02	yes	up	0.594	0.192	0.89	0.51	0.62	0.36	0.59	0.33	0.12	0.16	0.1	0.25
ENSMUSG00000029304	Spp1	secreted phosphoprotein 1 [Source:MGI Symbol;Acc:MGI:98389]	2.597402597	1.377069649	7.14E-08	4.55E-06	yes	up	1015.136	413.418	793.13	1708.74	928.71	902.74	742.36	466.88	477.32	288.91	413.06	420.92
ENSMUSG00000022876	Samsn1	SAM domain, SH3 domain and nuclear localization signals, 1 [Source:MGI Symbol;Acc:MGI:1914992]	2.597402597	1.377069649	1.30E-04	1.99E-03	yes	up	37.648	15.258	74.36	20.25	43.85	20.21	29.57	16.96	14.64	12.86	18.06	13.77
ENSMUSG00000027398	Il1b	interleukin 1 beta [Source:MGI Symbol;Acc:MGI:96543]	2.597402597	1.377069649	5.36E-03	3.63E-02	yes	up	83.908	33.794	179.6	40.48	80.21	67.92	51.33	37.6	62.27	18.55	23.22	27.33
ENSMUSG00000086196	Gm13571	predicted gene 13571 [Source:MGI Symbol;Acc:MGI:3652223]	2.583979328	1.369594529	6.75E-04	7.40E-03	yes	up	1.394	0.572	0.82	2.01	1.52	1.03	1.59	0.28	0.42	0.46	0.87	0.83
ENSMUSG00000022303	Dcstamp	dendrocyte expressed seven transmembrane protein [Source:MGI Symbol;Acc:MGI:1923016]	2.570694087	1.36215794	5.60E-07	2.46E-05	yes	up	6.122	2.11	3.57	8.67	9.07	6.26	3.04	3.82	2.19	1.38	1.82	1.34
ENSMUSG00000030346	Rad51ap1	RAD51 associated protein 1 [Source:MGI Symbol;Acc:MGI:1098224]	2.564102564	1.358453971	3.40E-06	1.02E-04	yes	up	1.84	1.282	2.24	2.34	2.18	1.23	1.21	0.9	2.09	0.72	0.87	1.83
ENSMUSG00000015852	Fcrls	Fc receptor-like S, scavenger receptor [Source:MGI Symbol;Acc:MGI:1933397]	2.557544757	1.354759487	1.19E-07	6.92E-06	yes	up	20.396	9.052	35.84	15.4	25.92	10.85	13.97	10.26	10	6.74	7.66	10.6
ENSMUSG00000029414	Kntc1	kinetochore associated 1 [Source:MGI Symbol;Acc:MGI:2673709]	2.544529262	1.347398782	5.82E-10	8.36E-08	yes	up	2.16	0.496	1.46	3.21	1.17	1.37	3.59	0.69	0.61	0.25	0.36	0.57
ENSMUSG00000022033	Pbk	PDZ binding kinase [Source:MGI Symbol;Acc:MGI:1289156]	2.544529262	1.347398782	1.59E-05	3.57E-04	yes	up	3.942	1.774	5.47	4.07	4.78	2.62	2.77	2.46	0.87	0.9	2.15	2.49
ENSMUSG00000000982	Ccl3	chemokine (C-C motif) ligand 3 [Source:MGI Symbol;Acc:MGI:98260]	2.544529262	1.347398782	5.30E-05	9.56E-04	yes	up	13.992	5.826	22.25	11.99	10.37	10.09	15.26	2.97	5.04	4.62	11.65	4.85
ENSMUSG00000094335	Igkv1-117	immunoglobulin kappa variable 1-117 [Source:MGI Symbol;Acc:MGI:4439721]	2.538071066	1.343732465	5.63E-07	2.46E-05	yes	up	2907.31	1216.084	4217.02	2484.48	2940.47	2989.12	1905.46	988.87	1713.99	1232.47	1389.08	756.01
ENSMUSG00000031444	F10	coagulation factor X [Source:MGI Symbol;Acc:MGI:103107]	2.538071066	1.343732465	2.24E-03	1.85E-02	yes	up	39.964	18.294	91.21	20.35	47.83	18.12	22.31	22.12	16.28	15.68	19.02	18.37
ENSMUSG00000046031	Calhm6	calcium homeostasis modulator family member 6 [Source:MGI Symbol;Acc:MGI:2443082]	2.525252525	1.336427665	1.49E-05	3.41E-04	yes	up	6.53	2.716	5.79	9.63	4.84	7.52	4.87	1.64	4.71	2.68	2.31	2.24
ENSMUSG00000042349	Ikbke	inhibitor of kappaB kinase epsilon [Source:MGI Symbol;Acc:MGI:1929612]	2.518891688	1.332789088	9.65E-05	1.57E-03	yes	up	20.102	7.444	42.95	18.35	22.72	8.24	8.25	7.74	7.77	6.78	7.4	7.53
ENSMUSG00000038807	Rap1gap2	RAP1 GTPase activating protein 2 [Source:MGI Symbol;Acc:MGI:3028623]	2.506265664	1.325539348	1.72E-03	1.51E-02	yes	up	8.514	3.76	17.99	5.38	9.61	3.73	5.86	3.95	4.19	3.37	3.92	3.37

ENSMUSG00000019942	Cdk1	cyclin-dependent kinase 1 [Source:MGI Symbol;Acc:MGI:88351]	2.5	1.321928095	3.38E-09	3.79E-07	yes	up	17.842	8.88	27.72	14.69	17.43	14.94	14.43	13.55	8.29	5.92	9.09	7.55
ENSMUSG00000004709	Cd244a	CD244 molecule A [Source:MGI Symbol;Acc:MGI:109294]	2.5	1.321928095	3.35E-06	1.00E-04	yes	up	8.222	3.504	13.6	3.69	9	8.65	6.17	3.81	4.41	2.24	3.41	3.65
ENSMUSG00000085295	4930430E12Rik	RIKEN cDNA 4930430E12 gene [Source:MGI Symbol;Acc:MGI:1918889]	2.493765586	1.318325858	3.36E-12	1.04E-09	yes	up	4.8	1.984	5.89	5.03	3.75	4.58	4.75	1.87	2.3	1.43	2.88	1.44
ENSMUSG00000071715	Ncf4	neutrophil cytosolic factor 4 [Source:MGI Symbol;Acc:MGI:109186]	2.487562189	1.314732593	1.39E-09	1.79E-07	yes	up	50.606	19.48	83.72	43.34	47.8	49.08	29.09	23.75	20.47	15.37	17.01	20.8
ENSMUSG00000030124	Lag3	lymphocyte-activation gene 3 [Source:MGI Symbol;Acc:MGI:106588]	2.487562189	1.314732593	2.92E-07	1.49E-05	yes	up	2.94	1.244	2.51	4.57	2.33	3.27	2.02	1.4	1.46	1.26	1.05	1.05
ENSMUSG00000008496	Pou2f2	POU domain, class 2, transcription factor 2 [Source:MGI Symbol;Acc:MGI:101897]	2.487562189	1.314732593	1.52E-04	2.27E-03	yes	up	8.708	3.612	17.95	7.02	6.91	5.44	6.22	3.62	4.11	2.02	4.31	4
ENSMUSG00000024672	Ms4a7	membrane-spanning 4-domains, subfamily A, member 7 [Source:MGI Symbol;Acc:MGI:1918846]	2.481389578	1.311148256	3.20067E-27	5.42738E-23	yes	up	73.296	30.628	82.6	82.46	73.73	73.05	54.64	34.62	31.01	26.71	35.02	25.78
ENSMUSG00000022148	Fyb	FYN binding protein [Source:MGI Symbol;Acc:MGI:1346327]	2.481389578	1.311148256	2.22E-10	3.73E-08	yes	up	48.588	23.742	77.24	42.02	44.06	44.15	35.47	24.08	31.71	18.84	23.94	20.14
ENSMUSG00000049988	Lrrc25	leucine rich repeat containing 25 [Source:MGI Symbol;Acc:MGI:2445284]	2.481389578	1.311148256	8.04E-07	3.24E-05	yes	up	14.906	6.442	27.57	10.07	16.13	10.61	10.15	5.01	8.66	4.85	6.89	6.8
ENSMUSG00000028031	Dkk2	dickkopf WNT signaling pathway inhibitor 2 [Source:MGI Symbol;Acc:MGI:1890663]	2.481389578	1.311148256	9.26E-04	9.48E-03	yes	up	0.906	0.386	1.11	0.67	1.72	0.71	0.32	0.39	0.45	0.2	0.47	0.42
ENSMUSG00000083773	Gm13394	predicted gene 13394 [Source:MGI Symbol;Acc:MGI:3651848]	2.475247525	1.307572802	2.15E-04	3.03E-03	yes	up	2.694	1.164	2.52	2.53	2.47	3.85	2.1	0.69	0.77	1.5	1.76	1.1
ENSMUSG00000035373	Ccl7	chemokine (C-C motif) ligand 7 [Source:MGI Symbol;Acc:MGI:99512]	2.475247525	1.307572802	1.60E-03	1.44E-02	yes	up	2.716	1.188	1.93	2.37	2.24	4.82	2.22	0.74	1.53	0.99	1.31	1.37
ENSMUSG00000026228	Htr2b	5-hydroxytryptamine (serotonin) receptor 2B [Source:MGI Symbol;Acc:MGI:109323]	2.469135802	1.304006187	4.83E-06	1.35E-04	yes	up	4.058	1.71	3.89	3.92	4.77	3.65	4.06	1.17	2.66	1.36	2.35	1.01
ENSMUSG00000035004	Igsf6	immunoglobulin superfamily, member 6 [Source:MGI Symbol;Acc:MGI:1891393]	2.463054187	1.300448367	2.04E-09	2.49E-07	yes	up	43.238	18.48	74.86	34.61	47.88	31.22	27.62	18.38	22.04	14.23	20.74	17.01
ENSMUSG00000053318	Slamf8	SLAM family member 8 [Source:MGI Symbol;Acc:MGI:1921998]	2.450980392	1.293358943	5.46E-12	1.52E-09	yes	up	13.582	5.846	14.72	19.71	10.72	13.75	9.01	6.1	6.67	4.78	6.32	5.36
ENSMUSG00000030474	Siglece	sialic acid binding Ig-like lectin E [Source:MGI Symbol;Acc:MGI:1932475]	2.43902439	1.286304185	3.06E-06	9.42E-05	yes	up	19.516	8.146	40.77	11.48	19.44	12.85	13.04	7.7	9.65	7.32	8.48	7.58
ENSMUSG00000060044	Tmem26	transmembrane protein 26 [Source:MGI Symbol;Acc:MGI:2143537]	2.43902439	1.286304185	1.08E-04	1.71E-03	yes	up	1.914	0.79	3.71	1.38	2.05	1.01	1.42	1.01	1.08	0.43	0.57	0.86
ENSMUSG00000037872	Ackr1	atypical chemokine receptor 1 (Duffy blood group) [Source:MGI Symbol;Acc:MGI:1097689]	2.43902439	1.286304185	3.08E-04	4.02E-03	yes	up	2.668	1.15	3.47	2.27	2.35	2.74	2.51	0.55	1.93	1.51	0.83	0.93
ENSMUSG00000030144	Clec4d	C-type lectin domain family 4, member d [Source:MGI Symbol;Acc:MGI:1298389]	2.433090024	1.282789701	4.80E-12	1.40E-09	yes	up	129.49	56.658	146.02	145.94	149.83	110.86	94.8	51.49	72.41	59.84	66.46	33.09
ENSMUSG00000039055	Eme1	essential meiotic structure-specific endonuclease 1 [Source:MGI Symbol;Acc:MGI:3576783]	2.433090024	1.282789701	2.57E-03	2.06E-02	yes	up	0.812	0.392	0.75	0.95	0.69	0.83	0.84	0.62	0.19	0.48	0.42	0.25
ENSMUSG00000026683	Nuf2	NUF2, NDC80 kinetochore complex component [Source:MGI Symbol;Acc:MGI:1914227]	2.421307506	1.275786313	5.07E-07	2.29E-05	yes	up	4.156	1.846	5.33	4.23	5.1	2.81	3.31	2.3	1.47	0.9	2.43	2.13
ENSMUSG00000021384	Susd3	sushi domain containing 3 [Source:MGI Symbol;Acc:MGI:1913579]	2.421307506	1.275786313	7.81E-07	3.17E-05	yes	up	12.396	5.074	22.31	9.24	13.55	8.51	8.37	3.87	5.52	5.61	4.99	5.38
ENSMUSG00000033213	AA467197	expressed sequence AA467197 [Source:MGI Symbol;Acc:MGI:3034182]	2.398081535	1.261880711	1.79E-22	3.79E-19	yes	up	81.702	35.954	87.22	99.48	82.23	75.64	63.94	45.2	35.81	31.99	37.39	29.38
ENSMUSG00000047945	Marcksl1	MARCKS-like 1 [Source:MGI Symbol;Acc:MGI:97143]	2.392344498	1.258425153	3.27E-08	2.39E-06	yes	up	29.508	12.974	48.76	20.87	37.92	21.82	18.17	13.57	14.75	10.97	11.5	14.08
ENSMUSG00000028068	Iqgap3	IQ motif containing GTPase activating protein 3 [Source:MGI Symbol;Acc:MGI:3028642]	2.386634845	1.254977851	8.27E-06	2.09E-04	yes	up	2.164	0.864	3.25	2.02	2.66	0.93	1.96	0.91	1	0.48	1.06	0.87
ENSMUSG00000028718	Stil	Scl/Tal1 interrupting locus [Source:MGI Symbol;Acc:MGI:107477]	2.386634845	1.254977851	2.35E-05	4.94E-04	yes	up	0.94	0.35	0.79	0.78	0.73	1.35	1.05	0.62	0.14	0.32	0.27	0.4
ENSMUSG00000026271	Gpr35	G protein-coupled receptor 35 [Source:MGI Symbol;Acc:MGI:1929509]	2.386634845	1.254977851	6.77E-04	7.41E-03	yes	up	12.158	12.776	25.89	7.91	11.72	8.83	6.44	6.28	6.19	42.49	4.67	4.25
ENSMUSG00000026955	Sapcd2	suppressor APC domain containing 2 [Source:MGI Symbol;Acc:MGI:1919330]	2.375296912	1.248107862	1.63E-03	1.46E-02	yes	up	0.994	0.554	0.84	1.2	0.72	0.75	1.46	0.79	0.37	1.09	0.43	0.09
ENSMUSG00000117732	Hdhd2	novel protein	2.369668246	1.244685096	8.41E-04	8.79E-03	yes	up	0.838	0.374	0.48	0.83	0.63	1.24	1.01	0.61	0.29	0.39	0.22	0.36
ENSMUSG00000045273	Cenph	centromere protein H [Source:MGI Symbol;Acc:MGI:1349448]	2.369668246	1.244685096	3.96E-03	2.89E-02	yes	up	1.014	0.448	1.02	1.25	0.86	0.98	0.96	0.44	0.67	0.62	0.2	0.31

ENSMUSG00000029816	Gpnmb	glycoprotein (transmembrane) nmb [Source:MGI Symbol;Acc:MGI:1934765]	2.364066194	1.241270432	1.43E-12	5.17E-10	yes	up	236.046	103.412	261.78	296.98	244.95	198.72	177.8	101.27	155.72	90.42	100.03	69.62
ENSMUSG00000029322	Plac8	placenta-specific 8 [Source:MGI Symbol;Acc:MGI:2445289]	2.364066194	1.241270432	1.05E-04	1.67E-03	yes	up	235.986	105.58	410.49	140.42	289.61	200.02	139.39	118.38	109.91	66.89	123.18	109.54
ENSMUSG00000023800	Tiam2	T cell lymphoma invasion and metastasis 2 [Source:MGI Symbol;Acc:MGI:1344338]	2.364066194	1.241270432	2.60E-04	3.52E-03	yes	up	4.298	1.528	9.29	2.01	5.48	2.56	2.15	1.19	1.64	1.27	1.64	1.9
ENSMUSG00000023078	Cxcl13	chemokine (C-X-C motif) ligand 13 [Source:MGI Symbol;Acc:MGI:1888499]	2.352941176	1.234465254	1.81E-06	6.19E-05	yes	up	11.58	5.206	15.27	10.46	10.74	14.84	6.59	7.94	3.97	5.54	3.59	4.99
ENSMUSG00000005413	Hmox1	heme oxygenase 1 [Source:MGI Symbol;Acc:MGI:96163]	2.352941176	1.234465254	3.80E-06	1.11E-04	yes	up	121.776	54.384	207.54	102.84	140.48	81.4	76.62	63.35	60.68	47.11	52.97	47.81
ENSMUSG00000029581	Fscn1	fascin actin-bundling protein 1 [Source:MGI Symbol;Acc:MGI:1352745]	2.336448598	1.224317298	5.77E-10	8.36E-08	yes	up	28.14	15.234	47.4	22.46	29.16	21.58	20.1	17.17	17.07	15.17	13.18	13.58
ENSMUSG00000009731	Kcnd1	potassium voltage-gated channel, Shal-related family, member 1 [Source:MGI Symbol;Acc:MGI:96671]	2.331002331	1.220950447	5.69E-04	6.46E-03	yes	up	0.312	0.142	0.41	0.31	0.27	0.28	0.29	0.12	0.13	0.08	0.12	0.26
ENSMUSG00000026398	Nr5a2	nuclear receptor subfamily 5, group A, member 2 [Source:MGI Symbol;Acc:MGI:1346834]	2.325581395	1.217591435	3.28E-03	2.49E-02	yes	up	1.34	0.804	2.91	0.74	1.35	0.64	1.06	1.5	0.56	0.31	0.58	1.07
ENSMUSG00000027331	Knstrn	kinetochore-localized astrin/SPAG5 binding [Source:MGI Symbol;Acc:MGI:1289298]	2.320185615	1.214240226	7.24E-07	2.99E-05	yes	up	7.542	5.096	7.32	9.27	9.97	6.36	4.79	3.68	4.6	1.77	6.27	9.16
ENSMUSG00000028965	Tnfrsf9	tumor necrosis factor receptor superfamily, member 9 [Source:MGI Symbol;Acc:MGI:1101059]	2.314814815	1.210896782	9.14E-12	2.25E-09	yes	up	7.44	3.216	9.95	9.02	7.4	6.12	4.71	2.8	4.27	2.26	3.59	3.16
ENSMUSG00000095200	Ighv1-7	immunoglobulin heavy variable V1-7 [Source:MGI Symbol;Acc:MGI:3704122]	2.314814815	1.210896782	3.66E-03	2.72E-02	yes	up	431.484	199.218	183.16	467.43	387.06	405.99	713.78	304.86	165.02	248.42	106.96	170.83
ENSMUSG00000055254	Ntrk2	neurotrophic tyrosine kinase, receptor, type 2 [Source:MGI Symbol;Acc:MGI:97384]	2.309468822	1.20756107	3.26E-08	2.39E-06	yes	up	13.886	5.826	22.81	9.11	21.9	7.01	8.6	6.16	5.61	4.22	7.13	6.01
ENSMUSG00000068129	Cst7	cystatin F (leukocystatin) [Source:MGI Symbol;Acc:MGI:1298217]	2.309468822	1.20756107	1.31E-06	4.80E-05	yes	up	9.036	4.122	9.31	13.67	5	7.87	9.33	4.6	3.71	4.47	4.45	3.38
ENSMUSG00000045136	Tubb2b	tubulin, beta 2B class IIB [Source:MGI Symbol;Acc:MGI:1920960]	2.309468822	1.20756107	1.15E-05	2.77E-04	yes	up	7.65	3.714	11.8	5.13	9.72	5.37	6.23	4	2.42	2.23	4.23	5.69
ENSMUSG00000028874	Fgr	FGR proto-oncogene, Src family tyrosine kinase [Source:MGI Symbol;Acc:MGI:95527]	2.309468822	1.20756107	4.67E-04	5.56E-03	yes	up	40.608	19.078	79.06	26.55	44.02	27.91	25.5	21.44	21.85	11.99	17.76	22.35
ENSMUSG00000073705	Cenps	centromere protein S [Source:MGI Symbol;Acc:MGI:1917178]	2.309468822	1.20756107	5.55E-03	3.73E-02	yes	up	1.634	1.032	2.63	1.53	1.29	1.84	0.88	0.75	1.29	0.28	1.56	1.28
ENSMUSG00000046245	Pilra	paired immunoglobin-like type 2 receptor alpha [Source:MGI Symbol;Acc:MGI:2450529]	2.304147465	1.204233052	5.60E-05	9.97E-04	yes	up	37.086	15.556	61.72	25.11	50.6	25.83	22.17	16.78	14.7	13.73	15.96	16.61
ENSMUSG00000020493	Prr11	proline rich 11 [Source:MGI Symbol;Acc:MGI:2444496]	2.298850575	1.200912694	3.06E-06	9.42E-05	yes	up	2.008	0.93	2.61	1.57	2.54	1.49	1.83	1.11	0.8	0.49	1.42	0.83
ENSMUSG00000098318	Lockd	lncRNA downstream of Cdkn1b [Source:MGI Symbol;Acc:MGI:1915081]	2.298850575	1.200912694	2.28E-03	1.88E-02	yes	up	0.276	0.196	0.33	0.2	0.37	0.24	0.24	0.18	0.42	0.09	0.24	0.05
ENSMUSG00000038943	Prc1	protein regulator of cytokinesis 1 [Source:MGI Symbol;Acc:MGI:1858961]	2.293577982	1.19759996	9.06E-08	5.53E-06	yes	up	8.35	3.904	11.46	8.23	8.99	5.26	7.81	4.87	3.21	2.33	4.29	4.82
ENSMUSG00000102715	Gm6209	predicted gene 6209 [Source:MGI Symbol;Acc:MGI:3643374]	2.277904328	1.187707155	1.06E-07	6.34E-06	yes	up	7.744	3.114	8.52	7.41	8.67	7.58	6.54	4.16	3.27	2.75	3.26	2.13
ENSMUSG00000105504	Gbp5	guanylate binding protein 5 [Source:MGI Symbol;Acc:MGI:2429943]	2.272727273	1.184424571	2.95E-08	2.21E-06	yes	up	19.568	9.032	34	21.39	14.62	13.35	14.48	9.47	8.95	6.9	10.8	9.04
ENSMUSG00000062380	Tubb3	tubulin, beta 3 class III [Source:MGI Symbol;Acc:MGI:107813]	2.272727273	1.184424571	5.05E-03	3.46E-02	yes	up	1.576	0.774	3.75	0.89	1.28	1.05	0.91	1.04	0.96	0.61	0.63	0.63
ENSMUSG00000024397	Aif1	allograft inflammatory factor 1 [Source:MGI Symbol;Acc:MGI:1343098]	2.262443439	1.177881725	1.68E-15	1.30E-12	yes	up	31.968	14.82	31.98	38.33	28.65	36.38	24.5	16.68	15.55	12.86	15.21	13.8
ENSMUSG00000039476	Prrx2	paired related homeobox 2 [Source:MGI Symbol;Acc:MGI:98218]	2.262443439	1.177881725	2.19E-03	1.82E-02	yes	up	1.868	0.656	2.75	1.84	1.4	2.13	1.22	0.36	0.65	0.7	0.61	0.96
ENSMUSG00000028197	Col24a1	collagen, type XXIV, alpha 1 [Source:MGI Symbol;Acc:MGI:1918605]	2.262443439	1.177881725	2.33E-03	1.92E-02	yes	up	1.44	0.64	1.5	2.53	1.41	1.06	0.7	1.04	0.61	0.21	0.72	0.62
ENSMUSG00000078922	Tgtp1	T cell specific GTPase 1 [Source:MGI Symbol;Acc:MGI:98734]	2.257336343	1.174621396	1.56E-03	1.41E-02	yes	up	11.778	5.502	17.48	17.87	4.26	12.32	6.96	6.49	5.82	4.35	5.5	5.35
ENSMUSG00000016283	H2-M2	histocompatibility 2, M region locus 2 [Source:MGI Symbol;Acc:MGI:95914]	2.252252252	1.171368418	1.31E-04	2.00E-03	yes	up	132.978	62.302	80.9	239.11	118.23	119.26	107.39	90.41	67.3	38.16	62.03	53.61
ENSMUSG00000102051	I830127L07Rik	RIKEN cDNA I830127L07 gene [Source:MGI Symbol;Acc:MGI:3612406]	2.252252252	1.171368418	4.64E-04	5.54E-03	yes	up	4.49	2.004	3.8	3.52	4.15	6.62	4.36	3	2.08	0.74	2.14	2.06
ENSMUSG00000052889	Prkcb	protein kinase C, beta [Source:MGI Symbol;Acc:MGI:97596]	2.247191011	1.168122759	3.78E-08	2.68E-06	yes	up	43.582	19.094	74.07	39.46	45.02	29.02	30.34	20.53	23.53	16.8	16.65	17.96

ENSMUSG00000005667	Mthfd2	methylenetetrahydrofolate dehydrogenase (NAD+ dependent), methenyltetrahydrofolate cyclohydrolase [Source:MGI Symbol;Acc:MGI:1338850]	2.247191011	1.168122759	3.00E-07	1.51E-05	yes	up	9.322	4.272	15.74	7.26	11.07	6.23	6.31	4.17	4.5	4.23	4.22	4.24
ENSMUSG00000030109	Slc6a12	solute carrier family 6 (neurotransmitter transporter, betaine/GABA), member 12 [Source:MGI Symbol;Acc:MGI:95628]	2.247191011	1.168122759	2.59E-04	3.52E-03	yes	up	4.916	2.324	5.93	7.37	4.94	3.94	2.4	2.36	2.55	2.59	2.66	1.46
ENSMUSG00000033952	Aspm	abnormal spindle microtubule assembly [Source:MGI Symbol;Acc:MGI:1334448]	2.242152466	1.164884385	2.99E-07	1.51E-05	yes	up	1.8	0.948	4.39	1.69	1.02	0.93	0.97	2.55	0.52	0.33	0.62	0.72
ENSMUSG00000061578	Ksr2	kinase suppressor of ras 2 [Source:MGI Symbol;Acc:MGI:3610315]	2.242152466	1.164884385	1.22E-03	1.17E-02	yes	up	0.176	0.086	0.15	0.26	0.15	0.13	0.19	0.09	0.14	0.05	0.09	0.06
ENSMUSG00000032218	Ccnb2	cyclin B2 [Source:MGI Symbol;Acc:MGI:88311]	2.237136465	1.161653263	1.44E-08	1.24E-06	yes	up	7.09	3.342	8.37	6.95	9.32	5.27	5.54	4.29	2.87	2.73	4.21	2.61
ENSMUSG00000074934	Grem1	gremlin 1, DAN family BMP antagonist [Source:MGI Symbol;Acc:MGI:1344337]	2.237136465	1.161653263	4.01E-03	2.91E-02	yes	up	0.666	0.32	0.53	0.71	1.1	0.6	0.39	0.21	0.29	0.12	0.36	0.62
ENSMUSG00000020178	Adora2a	adenosine A2a receptor [Source:MGI Symbol;Acc:MGI:99402]	2.232142857	1.158429363	2.97E-04	3.91E-03	yes	up	8.686	3.312	19.73	5.02	9.42	4.18	5.08	4.06	3.83	1.71	4.05	2.91
ENSMUSG00000024791	Cdca5	cell division cycle associated 5 [Source:MGI Symbol;Acc:MGI:1915099]	2.227171492	1.15521265	5.50E-07	2.44E-05	yes	up	1.888	0.858	2.32	1.55	1.96	1.77	1.84	1	0.8	0.98	0.82	0.69
ENSMUSG00000025279	Dnase1l3	deoxyribonuclease 1-like 3 [Source:MGI Symbol;Acc:MGI:1314633]	2.227171492	1.15521265	1.36E-05	3.19E-04	yes	up	3.612	1.716	2.04	5.57	2.25	4.95	3.25	2.03	2.05	1.58	1.49	1.43
ENSMUSG00000028602	Tnfrsf8	tumor necrosis factor receptor superfamily, member 8 [Source:MGI Symbol;Acc:MGI:99908]	2.227171492	1.15521265	4.86E-05	8.90E-04	yes	up	1.776	0.792	2.76	2.04	1.39	1.64	1.05	1.03	0.92	0.39	0.71	0.91
ENSMUSG00000029814	Igf2bp3	insulin-like growth factor 2 mRNA binding protein 3 [Source:MGI Symbol;Acc:MGI:1890359]	2.227171492	1.15521265	4.23E-03	3.04E-02	yes	up	1.444	0.744	0.34	6	0.29	0.32	0.27	0.83	2.01	0.08	0.14	0.66
ENSMUSG00000024675	Ms4a4c	membrane-spanning 4-domains, subfamily A, member 4C [Source:MGI Symbol;Acc:MGI:1927656]	2.2172949	1.148800661	2.17E-03	1.81E-02	yes	up	54.946	22.574	131.37	46.25	41.18	24.86	31.07	34.94	25.59	8.9	24.89	18.55
ENSMUSG00000104835	Gm5547	predicted gene 5547 [Source:MGI Symbol;Acc:MGI:3648206]	2.212389381	1.145605322	2.99E-06	9.30E-05	yes	up	9.6	4.37	10.03	12.63	5.68	9.02	10.64	4.04	4.17	5.98	4.22	3.44
ENSMUSG00000027715	Ccna2	cyclin A2 [Source:MGI Symbol;Acc:MGI:108069]	2.207505519	1.142417045	5.12E-12	1.46E-09	yes	up	6.528	3.124	9.22	6.53	6.62	4.85	5.42	3.85	2.62	2.62	3.31	3.22
ENSMUSG00000026605	Cenpf	centromere protein F [Source:MGI Symbol;Acc:MGI:1313302]	2.202643172	1.139235797	2.64E-08	2.03E-06	yes	up	2.672	0.968	1.95	1.48	4.48	4.14	1.31	0.8	1.34	1.07	0.94	0.69
ENSMUSG00000006403	Adamts4	a disintegrin-like and metallopeptidase (reprolysin type) with thrombospondin type 1 motif, 4 [Source:MGI Symbol;Acc:MGI:1339949]	2.202643172	1.139235797	5.91E-03	3.90E-02	yes	up	1.116	0.54	1.91	0.63	1.92	0.58	0.54	0.77	0.64	0.3	0.38	0.61
ENSMUSG00000117628	AC154492.2	Novel transcript, sense intronic to Rhoq	2.192982456	1.13289427	8.26E-04	8.66E-03	yes	up	1.006	0.482	1.51	1.1	1.08	0.72	0.62	0.53	0.41	0.49	0.48	0.5
ENSMUSG00000070868	Skint3	selection and upkeep of intraepithelial T cells 3 [Source:MGI Symbol;Acc:MGI:3045331]	2.183406114	1.126580497	2.92E-03	2.28E-02	yes	up	2.648	1.286	4.54	1.17	3.97	1.5	2.06	0.97	1.89	0.65	1.23	1.69
ENSMUSG00000021322	Aoah	acyloxyacyl hydrolase [Source:MGI Symbol;Acc:MGI:1350928]	2.178649237	1.123433941	3.17E-13	1.35E-10	yes	up	11.66	5.848	14.49	12.06	11.45	9.4	10.9	5.4	5.47	6.94	7.04	4.39
ENSMUSG00000051378	Kif18b	kinesin family member 18B [Source:MGI Symbol;Acc:MGI:2446979]	2.178649237	1.123433941	2.29E-06	7.54E-05	yes	up	1.422	0.692	1.62	1.37	1.18	1.02	1.92	0.87	0.64	0.6	0.56	0.79
ENSMUSG00000070427	Il18bp	interleukin 18 binding protein [Source:MGI Symbol;Acc:MGI:1333800]	2.173913043	1.120294234	6.91E-07	2.86E-05	yes	up	17.288	8.186	15.03	28.5	12.08	17.24	13.59	9.09	10.42	5.46	10.62	5.34
ENSMUSG00000038811	Gngt2	guanine nucleotide binding protein (G protein), gamma transducing activity polypeptide 2 [Source:MGI Symbol;Acc:MGI:893584]	2.173913043	1.120294234	9.96E-04	1.00E-02	yes	up	260.814	134.162	498.56	175.41	298.1	160.35	171.65	148.64	133.2	109.06	143.59	136.32
ENSMUSG00000024798	Htr7	5-hydroxytryptamine (serotonin) receptor 7 [Source:MGI Symbol;Acc:MGI:99841]	2.173913043	1.120294234	2.66E-03	2.12E-02	yes	up	1.326	0.658	2.81	1.1	1.08	0.63	1.01	0.75	0.52	0.85	0.71	0.46
ENSMUSG00000096459	Ighv9-3	immunoglobulin heavy variable V9-3 [Source:MGI Symbol;Acc:MGI:3642720]	2.169197397	1.117161344	3.18E-03	2.43E-02	yes	up	644.394	315.598	358.79	970.87	353.58	714.36	824.37	263.66	274.56	458.38	214.54	366.85
ENSMUSG00000027469	Tpx2	TPX2, microtubule-associated [Source:MGI Symbol;Acc:MGI:1919369]	2.159827214	1.110915901	5.50E-09	5.62E-07	yes	up	5.15	2.704	6.64	4.83	5.47	3.57	5.24	3.74	2.43	1.61	2.57	3.17
ENSMUSG00000030830	Itgal	integrin alpha L [Source:MGI Symbol;Acc:MGI:96606]	2.159827214	1.110915901	3.98E-04	4.90E-03	yes	up	89.428	43.524	161.17	63.83	102.96	54.47	64.71	51.43	46.76	36.22	42.31	40.9
ENSMUSG00000020330	Hmmr	hyaluronan mediated motility receptor (RHAMM) [Source:MGI Symbol;Acc:MGI:104667]	2.150537634	1.104697379	1.09E-07	6.52E-06	yes	up	2.034	0.962	2.21	2.57	2.18	1.38	1.83	0.84	0.74	0.83	1.3	1.1

ENSMUSG00000040204	Pclaf	PCNA clamp associated factor [Source:MGI Symbol;Acc:MGI:1915276]	2.150537634	1.104697379	4.17E-07	1.98E-05	yes	up	7.15	3.518	9.52	8.73	4.76	9.13	3.61	4.15	3.28	4.08	3.02	3.06
ENSMUSG00000097352	C920009B18Rik	RIKEN cDNA C920009B18 gene [Source:MGI Symbol;Acc:MGI:3583961]	2.150537634	1.104697379	7.04E-04	7.64E-03	yes	up	2.382	1.24	3.46	3.78	2.23	1.61	0.83	1.85	1.93	0.55	1.15	0.72
ENSMUSG00000020914	Top2a	topoisomerase (DNA) II alpha [Source:MGI Symbol;Acc:MGI:98790]	2.141327623	1.098505545	2.42E-19	2.93E-16	yes	up	15.886	7.51	18.16	17.3	14.86	15.49	13.62	9.29	7.53	5.28	8.29	7.16
ENSMUSG00000051855	Mest	mesoderm specific transcript [Source:MGI Symbol;Acc:MGI:96968]	2.141327623	1.098505545	9.32E-07	3.64E-05	yes	up	6.054	3.16	9.49	4.39	7.04	3.9	5.45	4.26	2.73	2.33	3.25	3.23
ENSMUSG00000039196	Orm1	orosomucoid 1 [Source:MGI Symbol;Acc:MGI:97443]	2.141327623	1.098505545	3.47E-05	6.79E-04	yes	up	21.844	10.806	12.49	34.77	19.5	28.65	13.81	13.3	10.84	10.39	9.46	10.04
ENSMUSG00000036896	C1qc	complement component 1, q subcomponent, C chain [Source:MGI Symbol;Acc:MGI:88225]	2.136752137	1.095419565	3.11E-22	5.86E-19	yes	up	206.208	101.06	234.6	224.78	218.54	183.67	169.45	107.29	117.5	101.88	96.84	81.79
ENSMUSG00000036086	Zranb3	zinc finger, RAN-binding domain containing 3 [Source:MGI Symbol;Acc:MGI:1918362]	2.136752137	1.095419565	9.27E-07	3.63E-05	yes	up	11.548	5.254	8.6	20.24	10.19	11.42	7.29	7.09	5.57	2.61	5.75	5.25
ENSMUSG00000041431	Ccnb1	cyclin B1 [Source:MGI Symbol;Acc:MGI:88302]	2.136752137	1.095419565	2.93E-06	9.12E-05	yes	up	4.12	2.194	4.75	4.36	3.71	3.56	4.22	3.14	1.24	1.17	2.86	2.56
ENSMUSG00000031004	Mki67	antigen identified by monoclonal antibody Ki 67 [Source:MGI Symbol;Acc:MGI:106035]	2.132196162	1.092340172	2.04E-15	1.50E-12	yes	up	9.594	5.388	10.4	11.64	9.4	7.79	8.74	8.75	4.6	3.28	5.87	4.44
ENSMUSG00000066363	Serpina3f	serine (or cysteine) peptidase inhibitor, clade A, member 3F [Source:MGI Symbol;Acc:MGI:2182838]	2.132196162	1.092340172	3.79E-05	7.28E-04	yes	up	12.36	6.092	18.51	12.64	8.54	14.19	7.92	4.86	9.33	4.84	7.62	3.81
ENSMUSG00000024174	Pot1b	protection of telomeres 1B [Source:MGI Symbol;Acc:MGI:1920086]	2.132196162	1.092340172	3.31E-03	2.51E-02	yes	up	15.224	8.13	30.46	9.56	18.67	8.14	9.29	10.61	7.09	5.26	9.69	8
ENSMUSG00000033777	Tlr13	toll-like receptor 13 [Source:MGI Symbol;Acc:MGI:3045213]	2.127659574	1.089267338	6.70E-11	1.31E-08	yes	up	34.092	16.834	46.05	28.08	38.98	25.98	31.37	12.96	21.95	15.1	18.2	15.96
ENSMUSG00000015880	Ncapg	non-SMC condensin I complex, subunit G [Source:MGI Symbol;Acc:MGI:1930197]	2.127659574	1.089267338	3.22E-06	9.78E-05	yes	up	1.758	0.9	2.34	2.13	1.62	1.54	1.16	1.2	1.04	0.65	0.92	0.69
ENSMUSG00000066682	Pilrb2	paired immunoglobin-like type 2 receptor beta 2 [Source:MGI Symbol;Acc:MGI:2450535]	2.127659574	1.089267338	4.45E-05	8.25E-04	yes	up	23.828	12.346	44.45	18.75	25.15	18.39	12.4	11.87	19.55	7.5	12.97	9.84
ENSMUSG00000036887	C1qa	complement component 1, q subcomponent, alpha polypeptide [Source:MGI Symbol;Acc:MGI:88223]	2.123142251	1.086201035	1.85E-24	7.09E-21	yes	up	280.322	137.838	304.86	298.5	296.74	274.55	226.96	146.69	156.59	133.12	134.03	118.76
ENSMUSG00000097804	Gm16685	predicted gene, 16685 [Source:MGI Symbol;Acc:MGI:4439609]	2.123142251	1.086201035	2.77E-06	8.79E-05	yes	up	1.504	0.87	1.25	2.09	1.54	1.5	1.14	0.81	0.93	0.87	0.9	0.84
ENSMUSG00000021965	Ska3	spindle and kinetochore associated complex subunit 3 [Source:MGI Symbol;Acc:MGI:3041235]	2.123142251	1.086201035	5.28E-04	6.08E-03	yes	up	1.164	0.648	0.95	1.27	1.08	1.47	1.05	0.69	0.88	0.35	0.92	0.4
ENSMUSG00000031551	Ido1	indoleamine 2,3-dioxygenase 1 [Source:MGI Symbol;Acc:MGI:96416]	2.123142251	1.086201035	3.09E-03	2.38E-02	yes	up	2.104	1.024	3.8	2.58	1.13	1.39	1.62	1.28	0.87	0.91	1.44	0.62
ENSMUSG00000112023	Lilr4b	leukocyte immunoglobulin-like receptor, subfamily B, member 4B [Source:MGI Symbol;Acc:MGI:102702]	2.118644068	1.083141235	3.72E-04	4.64E-03	yes	up	35.5	16.212	52.79	36.75	46.53	24.32	17.11	17.42	19.31	13.85	19.55	10.93
ENSMUSG00000055541	Lair1	leukocyte-associated Ig-like receptor 1 [Source:MGI Symbol;Acc:MGI:105492]	2.114164905	1.080087911	1.41E-10	2.42E-08	yes	up	37.856	17.658	55.98	36.47	38	27.56	31.27	19.09	20.51	13.51	19.19	15.99
ENSMUSG00000021451	Sema4d	sema domain, immunoglobulin domain (Ig), transmembrane domain (TM) and short cytoplasmic domain, (semaphorin) 4D [Source:MGI Symbol;Acc:MGI:109244]	2.114164905	1.080087911	2.08E-08	1.69E-06	yes	up	45.43	24.054	81.96	39.99	41.46	34.77	28.97	23.57	27.32	19.71	22.97	26.7
ENSMUSG00000052336	Cx3cr1	chemokine (C-X3-C motif) receptor 1 [Source:MGI Symbol;Acc:MGI:1333815]	2.109704641	1.077041036	6.40E-07	2.70E-05	yes	up	30.374	18.15	51.97	26.91	27.16	23.43	22.4	17.32	15.71	15.81	18.79	23.12
ENSMUSG00000024989	Cep55	centrosomal protein 55 [Source:MGI Symbol;Acc:MGI:1921357]	2.109704641	1.077041036	2.07E-06	6.89E-05	yes	up	3.218	1.704	4.23	3.71	3.03	2.46	2.66	2.09	1.45	0.91	1.96	2.11
ENSMUSG00000036905	C1qb	complement component 1, q subcomponent, beta polypeptide [Source:MGI Symbol;Acc:MGI:88224]	2.100840336	1.070966521	3.45E-23	8.35E-20	yes	up	313.26	156.994	350.3	337.97	330.58	287.9	259.55	154.39	193.01	143.59	158.32	135.66
ENSMUSG00000002068	Ccne1	cyclin E1 [Source:MGI Symbol;Acc:MGI:88316]	2.100840336	1.070966521	1.31E-04	2.00E-03	yes	up	2.272	1.576	1.74	4.21	1.76	2.36	1.29	1.02	1.6	2.54	1.8	0.92
ENSMUSG00000020649	Rrm2	ribonucleotide reductase M2 [Source:MGI Symbol;Acc:MGI:98181]	2.092050209	1.064917477	7.65E-11	1.47E-08	yes	up	6.48	3.314	6.57	7.95	6.02	5.93	5.93	3.46	2.62	2.38	4.64	3.47
ENSMUSG00000019122	Ccl9	chemokine (C-C motif) ligand 9 [Source:MGI Symbol;Acc:MGI:104533]	2.092050209	1.064917477	2.66E-08	2.04E-06	yes	up	97.6	49.136	134.91	67.39	127.59	82.5	75.61	54.66	47.5	52.52	45.92	45.08
ENSMUSG00000027326	Knl1	kinetochore scaffold 1 [Source:MGI Symbol;Acc:MGI:1923714]	2.087682672	1.061902439	3.41E-09	3.80E-07	yes	up	1.264	0.508	1.23	0.97	2.48	0.82	0.82	0.56	0.43	0.4	0.64	0.51
ENSMUSG00000021624	Cd180	CD180 antigen [Source:MGI Symbol;Acc:MGI:1194924]	2.083333333	1.058893689	6.05E-10	8.55E-08	yes	up	22.696	11.274	28.59	31.69	17.06	18.37	17.77	11.38	11.75	8.59	12.53	12.12

ENSMUSG00000037095	Lrg1	leucine-rich alpha-2-glycoprotein 1 [Source:MGI Symbol;Acc:MGI:1924155]	2.083333333	1.058893689	5.78E-05	1.02E-03	yes	up	155.002	78.324	130.49	170.94	151.45	210.19	111.94	97.78	90.31	96.05	51.6	55.88
ENSMUSG00000041324	Inhba	inhibin beta-A [Source:MGI Symbol;Acc:MGI:96570]	2.083333333	1.058893689	1.14E-04	1.80E-03	yes	up	2.824	1.194	2.27	5.27	1.88	3.21	1.49	1.33	1.02	0.72	1.97	0.93
ENSMUSG00000001403	Ube2c	ubiquitin-conjugating enzyme E2C [Source:MGI Symbol;Acc:MGI:1915862]	2.079002079	1.055891201	1.14E-10	2.08E-08	yes	up	16.256	8.254	20.45	17.65	13.59	12.55	17.04	8.34	6.55	7.72	9.72	8.94
ENSMUSG00000040552	C3ar1	complement component 3a receptor 1 [Source:MGI Symbol;Acc:MGI:1097680]	2.079002079	1.055891201	2.09E-09	2.49E-07	yes	up	12.72	6.434	15.53	16.98	8.84	12.02	10.23	4.98	7.49	5.76	7.52	6.42
ENSMUSG00000023505	Cdca3	cell division cycle associated 3 [Source:MGI Symbol;Acc:MGI:1315198]	2.079002079	1.055891201	5.65E-07	2.46E-05	yes	up	4.598	3.138	5.27	4.58	4.69	2.91	5.54	3.38	3.51	1.83	4.31	2.66
ENSMUSG00000024621	Csf1r	colony stimulating factor 1 receptor [Source:MGI Symbol;Acc:MGI:1339758]	2.079002079	1.055891201	4.73E-04	5.62E-03	yes	up	165.084	74.482	306.87	116.54	191.63	98.39	111.99	78	73.11	72.29	79.47	69.54
ENSMUSG00000039396	Neil3	nei like 3 (E. coli) [Source:MGI Symbol;Acc:MGI:2384588]	2.079002079	1.055891201	1.10E-03	1.08E-02	yes	up	1.238	0.614	1.64	1.37	1.43	0.62	1.13	0.6	0.55	0.37	0.93	0.62
ENSMUSG00000001131	Timp1	tissue inhibitor of metalloproteinase 1 [Source:MGI Symbol;Acc:MGI:98752]	2.074688797	1.052894948	9.60E-05	1.56E-03	yes	up	24.16	12.164	26.54	18.12	42.95	16.61	16.58	10.54	15.12	9.18	9.28	16.7
ENSMUSG00000028175	Depdc1a	DEP domain containing 1a [Source:MGI Symbol;Acc:MGI:1923381]	2.074688797	1.052894948	1.67E-03	1.48E-02	yes	up	1.264	0.702	1.98	1.38	1.44	0.7	0.82	1.09	0.62	0.29	0.47	1.04
ENSMUSG00000054871	Tmem158	transmembrane protein 158 [Source:MGI Symbol;Acc:MGI:1919559]	2.070393375	1.049904906	1.97E-03	1.69E-02	yes	up	1.302	0.666	0.89	1.2	1.75	1.37	1.3	0.33	0.58	0.64	0.87	0.91
ENSMUSG00000031138	F9	coagulation factor IX [Source:MGI Symbol;Acc:MGI:88384]	2.070393375	1.049904906	2.69E-03	2.14E-02	yes	up	0.682	0.346	0.64	1.15	0.47	0.52	0.63	0.42	0.38	0.34	0.37	0.22
ENSMUSG00000057191	AB124611	cDNA sequence AB124611 [Source:MGI Symbol;Acc:MGI:3043001]	2.066115702	1.046921047	1.13E-09	1.50E-07	yes	up	33.03	16.794	45.98	27.86	36.86	32.33	22.12	17.85	20.25	12.79	16.9	16.18
ENSMUSG00000094689	Ighv1-81	immunoglobulin heavy variable 1-81 [Source:MGI Symbol;Acc:MGI:4439635]	2.066115702	1.046921047	1.05E-03	1.04E-02	yes	up	830.484	427.87	736.19	1021.41	492.44	1056.15	846.23	570.95	273.62	592.18	411.65	290.95
ENSMUSG00000035273	Hpse	heparanase [Source:MGI Symbol;Acc:MGI:1343124]	2.06185567	1.043943348	9.58E-19	1.08E-15	yes	up	14.798	7.556	15.92	16.2	15.87	14.02	11.98	7.74	9.04	6.96	7.89	6.15
ENSMUSG00000043740	B430306N03Rik	RIKEN cDNA B430306N03 gene [Source:MGI Symbol;Acc:MGI:2443478]	2.06185567	1.043943348	1.11E-03	1.09E-02	yes	up	2.712	1.398	4.72	2.29	2.55	2.09	1.91	0.91	2.66	0.99	1.26	1.17
ENSMUSG00000089929	Bcl2a1b	B cell leukemia/lymphoma 2 related protein A1b [Source:MGI Symbol;Acc:MGI:1278326]	2.057613169	1.040971781	1.30E-08	1.15E-06	yes	up	158.114	80.914	241.95	148.5	178.63	101.73	119.76	89.3	91.16	65.76	88.74	69.61
ENSMUSG00000025877	Hk3	hexokinase 3 [Source:MGI Symbol;Acc:MGI:2670962]	2.057613169	1.040971781	5.56E-08	3.71E-06	yes	up	11.176	6.134	14.9	13.96	10.84	8.25	7.93	6.46	10.5	5.08	3.97	4.66
ENSMUSG00000003779	Kif20a	kinesin family member 20A [Source:MGI Symbol;Acc:MGI:1201682]	2.057613169	1.040971781	5.39E-06	1.47E-04	yes	up	3.578	2.046	4.26	3.75	3.58	2.72	3.58	3.29	1.9	1.5	1.96	1.58
ENSMUSG00000045763	Basp1	brain abundant, membrane attached signal protein 1 [Source:MGI Symbol;Acc:MGI:1917600]	2.05338809	1.038006323	2.52E-16	2.25E-13	yes	up	15.45	7.95	12.87	17.59	16.87	16.86	13.06	8.63	7.94	8.05	6.96	8.17
ENSMUSG00000055978	Fut2	fucosyltransferase 2 [Source:MGI Symbol;Acc:MGI:109374]	2.05338809	1.038006323	3.81E-03	2.81E-02	yes	up	0.782	0.274	0.83	1.06	0.48	0.42	1.12	0.21	0.27	0.21	0.21	0.47
ENSMUSG00000040751	Lat2	linker for activation of T cells family, member 2 [Source:MGI Symbol;Acc:MGI:1926479]	2.049180328	1.035046947	1.04E-20	1.60E-17	yes	up	30.122	14.744	33.63	34.91	31.5	24.33	26.24	18.98	14.72	10.19	14.94	14.89
ENSMUSG00000079553	Kifc1	kinesin family member C1 [Source:MGI Symbol;Acc:MGI:109596]	2.049180328	1.035046947	9.90E-06	2.45E-04	yes	up	3.312	1.714	4.53	3.47	3.72	2.08	2.76	2.46	1.63	1.14	1.81	1.53
ENSMUSG00000062248	Cks2	CDC28 protein kinase regulatory subunit 2 [Source:MGI Symbol;Acc:MGI:1913447]	2.049180328	1.035046947	3.73E-05	7.17E-04	yes	up	22.956	11.786	36.62	19.02	24.56	19	15.58	12.04	14.95	9.66	14.95	7.33
ENSMUSG00000020641	Rsad2	radical S-adenosyl methionine domain containing 2 [Source:MGI Symbol;Acc:MGI:1929628]	2.049180328	1.035046947	2.44E-04	3.35E-03	yes	up	6.108	3.14	12.57	6.12	4.17	3.18	4.5	3.3	3.14	2.39	3.49	3.38
ENSMUSG00000050914	Ankrd37	ankyrin repeat domain 37 [Source:MGI Symbol;Acc:MGI:3603344]	2.049180328	1.035046947	3.18E-03	2.43E-02	yes	up	9.134	4.388	18.88	6.3	7.04	6.17	7.28	3.72	4.64	3.15	5.77	4.66
ENSMUSG00000050967	Creg2	cellular repressor of E1A-stimulated genes 2 [Source:MGI Symbol;Acc:MGI:1928333]	2.044989775	1.03209363	2.36E-13	1.03E-10	yes	up	3.432	1.768	3.75	3.79	3.58	3.09	2.95	1.8	2.02	1.6	2.13	1.29
ENSMUSG00000030677	Kif22	kinesin family member 22 [Source:MGI Symbol;Acc:MGI:109233]	2.044989775	1.03209363	1.39E-06	4.99E-05	yes	up	4.128	2.024	5.56	4.09	5.24	2.56	3.19	2.49	1.94	1.69	2.16	1.84
ENSMUSG00000069792	Wfdc17	WAP four-disulfide core domain 17 [Source:MGI Symbol;Acc:MGI:3649773]	2.040816327	1.029146346	2.09E-11	4.78E-09	yes	up	428.47	221.682	527.77	389.62	430.81	425.52	368.63	219.17	309.21	174.93	228.74	176.36
ENSMUSG00000017716	Birc5	baculoviral IAP repeat-containing 5 [Source:MGI Symbol;Acc:MGI:1203517]	2.040816327	1.029146346	4.60E-07	2.13E-05	yes	up	8.558	4.348	12.13	8.16	7.98	7.71	6.81	5.34	3.14	4.01	5.29	3.96
ENSMUSG00000099974	Bcl2a1d	B cell leukemia/lymphoma 2 related protein A1d [Source:MGI Symbol;Acc:MGI:1278325]	2.040816327	1.029146346	3.04E-06	9.41E-05	yes	up	87.568	45.334	147.41	65.77	106.26	55.89	62.51	46.05	53.32	36.7	49.14	41.46

ENSMUSG00000048142	Nat8l	N-acetyltransferase 8-like [Source:MGI Symbol;Acc:MGI:2447776]	2.040816327	1.029146346	1.47E-03	1.34E-02	yes	up	0.746	0.382	1.21	0.46	0.92	0.61	0.53	0.48	0.39	0.45	0.42	0.17
ENSMUSG00000058715	Fcer1g	Fc receptor, IgE, high affinity I, gamma polypeptide [Source:MGI Symbol;Acc:MGI:95496]	2.032520325	1.023269779	7.43E-10	1.02E-07	yes	up	604.082	313.104	903.2	484.92	700.04	465.72	466.53	349.9	358.7	259.08	315.52	282.32
ENSMUSG00000020437	Myo1g	myosin IG [Source:MGI Symbol;Acc:MGI:1927091]	2.032520325	1.023269779	5.99E-04	6.73E-03	yes	up	54.566	27.438	92.22	37.2	66.57	34.82	42.02	29.49	28.74	21.05	30.15	27.76
ENSMUSG00000039187	Fanci	Fanconi anemia, complementation group I [Source:MGI Symbol;Acc:MGI:2384790]	2.028397566	1.020340448	4.89E-04	5.76E-03	yes	up	0.608	0.358	0.65	0.77	0.67	0.33	0.62	0.65	0.39	0.24	0.24	0.27
ENSMUSG00000015709	Arnt2	aryl hydrocarbon receptor nuclear translocator 2 [Source:MGI Symbol;Acc:MGI:107188]	2.028397566	1.020340448	1.57E-03	1.42E-02	yes	up	0.702	0.412	0.63	0.76	0.65	1.16	0.31	0.31	0.9	0.22	0.27	0.36
ENSMUSG00000002111	Spi1	spleen focus forming virus (SFFV) proviral integration oncogene [Source:MGI Symbol;Acc:MGI:98282]	2.024291498	1.017417053	1.36E-08	1.19E-06	yes	up	109.046	59.834	155.69	93.75	128.74	87.28	79.77	62.97	65.88	50.19	56	64.13
ENSMUSG00000040907	Atp1a3	ATPase, Na+/K+ transporting, alpha 3 polypeptide [Source:MGI Symbol;Acc:MGI:88107]	2.024291498	1.017417053	3.02E-08	2.24E-06	yes	up	21.918	11.348	26.29	23.4	24.03	19.84	16.03	10.15	16.08	11.92	11.11	7.48
ENSMUSG00000027009	Itga4	integrin alpha 4 [Source:MGI Symbol;Acc:MGI:96603]	2.024291498	1.017417053	8.14E-04	8.57E-03	yes	up	34.678	17.96	66.06	27.81	34.88	19.19	25.45	17.75	20.79	15.53	19.07	16.66
ENSMUSG00000026475	Rgs16	regulator of G-protein signaling 16 [Source:MGI Symbol;Acc:MGI:108407]	2.024291498	1.017417053	8.55E-04	8.92E-03	yes	up	2.802	1.45	3.81	4.3	2.48	2.34	1.08	1.29	1.87	1.16	1.62	1.31
ENSMUSG00000020120	Plek	pleckstrin [Source:MGI Symbol;Acc:MGI:1860485]	2.024291498	1.017417053	8.93E-04	9.23E-03	yes	up	112.236	57.568	210.08	97.36	111.69	64.84	77.21	69.14	56.44	41.31	62.25	58.7
ENSMUSG00000071714	Csf2rb2	colony stimulating factor 2 receptor, beta 2, low-affinity (granulocyte-macrophage) [Source:MGI Symbol;Acc:MGI:1339760]	2.012072435	1.008682243	6.11E-11	1.23E-08	yes	up	25.06	13.884	32.12	30.27	25.62	20.24	17.05	13.82	15.66	12.62	16.4	10.92
ENSMUSG00000037474	Dtl	denticleless E3 ubiquitin protein ligase [Source:MGI Symbol;Acc:MGI:1924093]	2.012072435	1.008682243	5.27E-04	6.08E-03	yes	up	1.15	0.602	1.59	1.58	1.14	0.65	0.79	0.52	0.52	0.63	0.45	0.89
ENSMUSG00000076563	Igkv5-48	immunoglobulin kappa variable 5-48 [Source:MGI Symbol;Acc:MGI:3642817]	2.012072435	1.008682243	3.82E-03	2.81E-02	yes	up	416.168	226.31	317.42	227.22	310.74	615.14	610.32	189.83	284.22	172.23	260.85	224.42
ENSMUSG00000031438	Rnf128	ring finger protein 128 [Source:MGI Symbol;Acc:MGI:1914139]	2.008032129	1.005782353	2.51E-14	1.47E-11	yes	up	18.584	9.788	19.74	20.94	22.53	15.48	14.23	8.97	11.51	9.38	9.49	9.59
ENSMUSG00000023992	Trem2	triggering receptor expressed on myeloid cells 2 [Source:MGI Symbol;Acc:MGI:1913150]	2.008032129	1.005782353	4.99E-13	1.97E-10	yes	up	191.768	100.022	209.23	216.97	216.75	153.72	162.17	98.43	130.84	89.23	105.57	76.04
ENSMUSG00000058818	Pirb	paired Ig-like receptor B [Source:MGI Symbol;Acc:MGI:894311]	2.008032129	1.005782353	8.27E-09	8.09E-07	yes	up	74.386	41.32	115.6	69.03	79.88	56.57	50.85	42.26	49.67	34.29	39.88	40.5
ENSMUSG00000027514	Zbp1	Z-DNA binding protein 1 [Source:MGI Symbol;Acc:MGI:1927449]	2.008032129	1.005782353	1.80E-07	9.86E-06	yes	up	27.44	14.878	38.17	33.8	21.22	24.61	19.4	16.34	18.2	11.4	16.08	12.37
ENSMUSG00000034310	Tmem132d	transmembrane protein 132D [Source:MGI Symbol;Acc:MGI:3044963]	0.5	-1	2.82E-15	1.99E-12	yes	down	3.438	4.652	0.99	3.02	1.85	4.5	6.83	4.9	3.62	2.23	4.23	8.28
ENSMUSG00000079105	C7	complement component 7 [Source:MGI Symbol;Acc:MGI:88235]	0.497512438	-1.007195501	4.27E-16	3.62E-13	yes	down	72.14	153.488	60.66	69.67	78.49	59.35	92.53	155.75	146.49	154	145.39	165.81
ENSMUSG00000101625	Gm29371	predicted gene 29371 [Source:MGI Symbol;Acc:MGI:5580077]	0.496277916	-1.010779839	1.60E-03	1.44E-02	yes	down	1.012	2.134	1.03	0.88	1.26	1.03	0.86	1.2	4.37	2.02	1.31	1.77
ENSMUSG00000011171	Vipr2	vasoactive intestinal peptide receptor 2 [Source:MGI Symbol;Acc:MGI:107166]	0.487329435	-1.037030731	2.14E-11	4.84E-09	yes	down	3.55	8.864	2.43	4.92	3.21	3.54	3.65	11.18	9.1	7.52	8.84	7.68
ENSMUSG00000102697	Pcdhac2	protocadherin alpha subfamily C, 2 [Source:MGI Symbol;Acc:MGI:1891443]	0.487329435	-1.037030731	2.90E-07	1.49E-05	yes	down	1.372	2.982	0.76	1.65	1.34	1.25	1.86	2.88	2.86	3.08	3.31	2.78
ENSMUSG00000025064	Col17a1	collagen, type XVII, alpha 1 [Source:MGI Symbol;Acc:MGI:88450]	0.487329435	-1.037030731	7.41E-04	7.93E-03	yes	down	0.476	1.172	0.22	0.31	0.38	0.89	0.58	0.72	0.75	0.61	1.34	2.44
ENSMUSG00000021055	Esr2	estrogen receptor 2 (beta) [Source:MGI Symbol;Acc:MGI:109392]	0.481695568	-1.053806444	5.27E-03	3.59E-02	yes	down	0.53	1.196	0.2	0.65	0.45	0.51	0.84	1.25	2.01	0.7	1.18	0.84
ENSMUSG00000047844	Bex4	brain expressed X-linked 4 [Source:MGI Symbol;Acc:MGI:3606746]	0.481000481	-1.055889758	8.75E-07	3.48E-05	yes	down	18.474	40.592	15.25	13.29	22.91	16.3	24.62	35.55	27.95	51.24	46.67	41.55
ENSMUSG00000021090	Lrrc9	leucine rich repeat containing 9 [Source:MGI Symbol;Acc:MGI:1925507]	0.477326969	-1.066950244	4.48E-03	3.17E-02	yes	down	0.194	0.452	0.1	0.12	0.17	0.32	0.26	0.54	0.29	0.4	0.5	0.53
ENSMUSG00000020427	Igfbp3	insulin-like growth factor binding protein 3 [Source:MGI Symbol;Acc:MGI:96438]	0.470588235	-1.087462841	6.52E-13	2.51E-10	yes	down	14.628	32.958	15.3	11.44	16.09	16.09	14.22	26.24	26.67	31.19	37.6	43.09
ENSMUSG00000032356	Rasgrf1	RAS protein-specific guanine nucleotide-releasing factor 1 [Source:MGI Symbol;Acc:MGI:99694]	0.465983225	-1.101650076	7.22E-03	4.52E-02	yes	down	0.198	0.688	0.11	0.34	0.08	0.17	0.29	0.67	0.46	1.14	0.69	0.48
ENSMUSG00000115536	Gm32857	predicted gene, 32857 [Source:MGI Symbol;Acc:MGI:5592016]	0.461680517	-1.115033243	4.47E-05	8.28E-04	yes	down	3.308	6.726	3.46	2.72	2.8	4.59	2.97	5.9	4.8	6.35	7.3	9.28

ENSMUSG00000052229	Gpr17	G protein-coupled receptor 17 [Source:MGI Symbol;Acc:MGI:3584514]	0.460617227	-1.118359726	4.54E-03	3.20E-02	yes	down	0.128	0.298	0.1	0.17	0.04	0.17	0.16	0.36	0.26	0.24	0.39	0.24
ENSMUSG00000106139	Gm30648	predicted gene, 30648 [Source:MGI Symbol;Acc:MGI:5589807]	0.454959054	-1.136191386	3.41E-03	2.56E-02	yes	down	0.366	1.182	0.15	0.49	0.59	0.35	0.25	1.91	0.82	0.62	0.96	1.6
ENSMUSG00000029168	Dpysl5	dihydropyrimidinase-like 5 [Source:MGI Symbol;Acc:MGI:1929772]	0.454338937	-1.138159145	1.08E-04	1.71E-03	yes	down	0.984	2.164	0.47	0.63	0.91	1.13	1.78	2.47	1.17	2.62	2.32	2.24
ENSMUSG00000086709	Gm16263	predicted gene 16263 [Source:MGI Symbol;Acc:MGI:3826536]	0.453514739	-1.140778656	1.84E-04	2.66E-03	yes	down	0.714	2.81	0.47	0.6	0.5	1.69	0.31	1.36	1	2.19	7.76	1.74
ENSMUSG00000037157	Il22ra1	interleukin 22 receptor, alpha 1 [Source:MGI Symbol;Acc:MGI:2663588]	0.453103761	-1.14208663	4.14E-03	2.98E-02	yes	down	0.376	0.884	0.17	0.31	0.25	0.52	0.63	0.76	0.59	1.19	0.83	1.05
ENSMUSG00000029334	Prkg2	protein kinase, cGMP-dependent, type II [Source:MGI Symbol;Acc:MGI:108173]	0.451875282	-1.146003451	4.62E-08	3.18E-06	yes	down	2.104	6.124	1.01	2.31	2.7	2.29	2.21	8.01	5.36	5.32	6.1	5.83
ENSMUSG00000019894	Slc6a15	solute carrier family 6 (neurotransmitter transporter), member 15 [Source:MGI Symbol;Acc:MGI:2143484]	0.451059991	-1.14860877	4.25E-07	2.00E-05	yes	down	0.812	1.656	1.07	0.48	0.65	0.93	0.93	1.62	1.45	1.47	1.9	1.84
ENSMUSG00000099338	2810030D12Rik	RIKEN cDNA 2810030D12 gene [Source:MGI Symbol;Acc:MGI:1925585]	0.447828034	-1.158983251	4.98E-03	3.43E-02	yes	down	1.45	3.898	0.79	1.66	0.73	2.01	2.06	4.99	2.02	3.16	6.35	2.97
ENSMUSG00000020431	Adcy1	adenylate cyclase 1 [Source:MGI Symbol;Acc:MGI:99677]	0.447627574	-1.159629186	1.09E-03	1.07E-02	yes	down	0.078	0.16	0.03	0.05	0.08	0.07	0.16	0.15	0.2	0.17	0.1	0.18
ENSMUSG00000054422	Fabp1	fatty acid binding protein 1, liver [Source:MGI Symbol;Acc:MGI:95479]	0.438404209	-1.189666447	1.77E-05	3.89E-04	yes	down	3.756	9.182	4.15	3.29	3.56	4.26	3.52	10.37	7.69	6.09	11.37	10.39
ENSMUSG00000039661	Dusp26	dual specificity phosphatase 26 (putative) [Source:MGI Symbol;Acc:MGI:1914209]	0.430663221	-1.215367972	2.10E-03	1.77E-02	yes	down	0.53	1.04	0.48	0.71	0.56	0.57	0.33	0.87	1.09	0.83	0.81	1.6
ENSMUSG00000029120	Ppp2r2c	protein phosphatase 2, regulatory subunit B, gamma [Source:MGI Symbol;Acc:MGI:2442660]	0.42973786	-1.218471211	2.89E-08	2.18E-06	yes	down	0.576	1.418	0.45	0.59	0.78	0.59	0.47	1.91	1.4	1.29	1.38	1.11
ENSMUSG00000057182	Scn3a	sodium channel, voltage-gated, type III, alpha [Source:MGI Symbol;Acc:MGI:98249]	0.423011844	-1.241230036	6.17E-15	4.03E-12	yes	down	1.8	3.59	3.07	1.84	1.19	1.47	1.43	4.33	2.67	2.72	4.24	3.99
ENSMUSG00000026828	Galnt5	polypeptide N-acetylgalactosaminyltransferase 5 [Source:MGI Symbol;Acc:MGI:2179403]	0.422654269	-1.242450074	4.02E-04	4.94E-03	yes	down	0.34	0.838	0.35	0.37	0.21	0.43	0.34	0.45	0.52	1.13	1.09	1
ENSMUSG00000046337	Fam178b	family with sequence similarity 178, member B [Source:MGI Symbol;Acc:MGI:3026913]	0.422119038	-1.244278199	3.05E-06	9.42E-05	yes	down	2.842	7.2	1.92	3.03	2.52	2.38	4.36	7.98	6.05	6.43	5.8	9.74
ENSMUSG00000027380	Acoxl	acyl-Coenzyme A oxidase-like [Source:MGI Symbol;Acc:MGI:1921371]	0.419815281	-1.252173413	2.94E-23	8.30E-20	yes	down	19.162	48.47	16.34	18.66	24.38	17.75	18.68	51.37	48.83	38.89	53.15	50.11
ENSMUSG00000031340	Gabre	gamma-aminobutyric acid (GABA) A receptor, subunit epsilon [Source:MGI Symbol;Acc:MGI:1330235]	0.418235048	-1.25761413	8.53E-05	1.41E-03	yes	down	4.672	7.614	0.19	5.78	2.85	11.7	2.84	25.99	0.4	8.11	3.13	0.44
ENSMUSG00000020892	Aloxe3	arachidonate lipoxygenase 3 [Source:MGI Symbol;Acc:MGI:1345140]	0.407996736	-1.293370484	4.75E-04	5.64E-03	yes	down	0.248	1.35	0.16	0.53	0.24	0.09	0.22	1.5	0.75	2.03	0.62	1.85
ENSMUSG00000108256	Gm43923	predicted gene, 43923 [Source:MGI Symbol;Acc:MGI:5690315]	0.404694456	-1.305095011	1.84E-03	1.59E-02	yes	down	0.162	0.418	0.11	0.23	0.2	0.07	0.2	0.32	0.46	0.42	0.38	0.51
ENSMUSG00000113342	AA414992	expressed sequence AA414992 [Source:MGI Symbol;Acc:MGI:2142939]	0.401606426	-1.316145742	5.12E-04	5.96E-03	yes	down	0.61	1.6	0.26	0.68	0.76	0.8	0.55	1.32	3.18	0.91	1.03	1.56
ENSMUSG00000029212	Gabrb1	gamma-aminobutyric acid (GABA) A receptor, subunit beta 1 [Source:MGI Symbol;Acc:MGI:95619]	0.400801603	-1.319039816	4.10E-03	2.96E-02	yes	down	0.03	0.086	0.04	0.03	0.03	0.04	0.01	0.09	0.04	0.08	0.11	0.11
ENSMUSG00000031494	Cd209a	CD209a antigen [Source:MGI Symbol;Acc:MGI:2157942]	0.397140588	-1.332278283	3.96E-07	1.89E-05	yes	down	3.716	10.17	1.81	3.28	3.13	5.43	4.93	12.13	7.55	8.16	11.7	11.31
ENSMUSG00000112458	Gm40773	predicted gene, 40773 [Source:MGI Symbol;Acc:MGI:5623658]	0.394166338	-1.343123519	1.04E-03	1.04E-02	yes	down	0.65	1.714	0.68	0.3	1.17	0.29	0.81	1.26	1.81	1.95	1.27	2.28
ENSMUSG00000107023	Gm42715	predicted gene 42715 [Source:MGI Symbol;Acc:MGI:5662852]	0.393391031	-1.34596403	1.42E-04	2.15E-03	yes	down	3.738	4.366	3.53	6.11	0.98	4.93	3.14	1.96	3.89	4.9	6	5.08
ENSMUSG00000117775	AC132307.1	novel transcript, sense intronic to Zfp532	0.383582662	-1.382390583	5.20E-03	3.55E-02	yes	down	0.162	0.452	0.18	0.27	0.11	0.12	0.13	0.61	0.27	0.17	0.67	0.54
ENSMUSG00000051111	Sv2c	synaptic vesicle glycoprotein 2c [Source:MGI Symbol;Acc:MGI:1922459]	0.377500944	-1.405447844	1.84E-04	2.66E-03	yes	down	0.104	0.314	0.05	0.2	0.08	0.13	0.06	0.33	0.39	0.21	0.23	0.41
ENSMUSG00000073733	Rsg1	REM2 and RAB-like small GTPase 1 [Source:MGI Symbol;Acc:MGI:1923416]	0.37593985	-1.411426246	2.62E-05	5.41E-04	yes	down	1.438	5.236	1.26	0.71	0.76	3.28	1.18	3.95	2.33	3.45	13.51	2.94
ENSMUSG00000054966	Lmntd1	lamin tail domain containing 1 [Source:MGI Symbol;Acc:MGI:1921321]	0.37327361	-1.421694581	1.38E-04	2.10E-03	yes	down	0.842	2.156	0.12	1.28	1.55	0.43	0.83	3.39	1.66	1.37	2.75	1.61
ENSMUSG00000060988	Galnt13	polypeptide N-acetylgalactosaminyltransferase 13 [Source:MGI Symbol;Acc:MGI:2139447]	0.372717108	-1.423847055	2.63E-05	5.43E-04	yes	down	0.27	0.598	0.34	0.13	0.16	0.4	0.32	0.69	0.51	0.58	0.42	0.79
ENSMUSG00000032172	Olfm2	olfactomedin 2 [Source:MGI Symbol;Acc:MGI:3045350]	0.370233247	-1.43349364	1.12E-06	4.23E-05	yes	down	1.326	3.76	0.66	1.66	1.89	1.14	1.28	4.05	5.44	2.14	3.45	3.72

ENSMUSG00000042750	Bex2	brain expressed X-linked 2 [Source:MGI Symbol;Acc:MGI:1338017]	0.36101083	-1.469885976	8.74E-09	8.34E-07	yes	down	5.008	14.724	3.77	3.45	6.18	3.32	8.32	14.51	11.89	14.79	15.78	16.65
ENSMUSG00000109115	Gm44669	predicted gene 44669 [Source:MGI Symbol;Acc:MGI:5753245]	0.352360817	-1.504874589	1.29E-03	1.22E-02	yes	down	0.21	0.628	0.23	0.09	0.25	0.2	0.28	0.45	0.8	0.52	0.42	0.95
ENSMUSG00000052188	Gm14964	predicted gene 14964 [Source:MGI Symbol;Acc:MGI:3641621]	0.346860909	-1.527570837	1.47E-05	3.40E-04	yes	down	1.462	4.604	0.91	1.07	1.56	2.01	1.76	6.16	2.58	5.2	2.93	6.15
ENSMUSG00000057606	Colq	collagen-like tail subunit (single strand of homotrimer) of asymmetric acetylcholinesterase [Source:MGI Symbol;Acc:MGI:1338761]	0.342348511	-1.546462358	5.71E-07	2.47E-05	yes	down	1.264	3.914	0.36	2.02	1.05	1.52	1.37	3.64	3.73	3.44	3.76	5
ENSMUSG00000073680	Tmem88b	transmembrane protein 88B [Source:MGI Symbol;Acc:MGI:2444329]	0.338294993	-1.563646269	6.96E-03	4.40E-02	yes	down	0.072	0.224	0.09	0.06	0.1	0.01	0.1	0.15	0.22	0.32	0.31	0.12
ENSMUSG00000020524	Gria1	glutamate receptor, ionotropic, AMPA1 (alpha 1) [Source:MGI Symbol;Acc:MGI:95808]	0.287604257	-1.797843067	7.54E-08	4.76E-06	yes	down	0.35	1.26	0.26	0.45	0.4	0.51	0.13	0.95	0.92	1.92	1.14	1.37
ENSMUSG00000107134	Gm42528	predicted gene 42528 [Source:MGI Symbol;Acc:MGI:5662665]	0.256147541	-1.964953053	2.18E-05	4.63E-04	yes	down	0.344	1.426	0.02	0.3	0.55	0.29	0.56	1.46	1.69	1.08	1.81	1.09
ENSMUSG00000028940	Hes2	hes family bHLH transcription factor 2 [Source:MGI Symbol;Acc:MGI:1098624]	0.239808153	-2.060047384	3.57E-09	3.93E-07	yes	down	0.274	1.888	0.26	0.11	0.37	0.16	0.47	1.54	2.82	1.16	1.24	2.68
ENSMUSG00000064225	Paqr9	progestin and adipoQ receptor family member IX [Source:MGI Symbol;Acc:MGI:1922802]	0.223613596	-2.160920188	5.71E-03	3.79E-02	yes	down	0.024	0.108	0.03	0.06	0.02	0.01	0	0.05	0.02	0.15	0.22	0.1
ENSMUSG00000089989	Gm45713	predicted gene 45713 [Source:MGI Symbol;Acc:MGI:5804828]	0.216497077	-2.207580546	6.08E-03	3.98E-02	yes	down	1.164	4.444	0	0.84	2.96	0.47	1.55	3.44	5.95	3.56	3.25	6.02
ENSMUSG00000101678	Gm29609	predicted gene 29609 [Source:MGI Symbol;Acc:MGI:5580315]	0.16204829	-2.625504295	3.53E-03	2.64E-02	yes	down	0.308	0.796	0.18	0.34	1.02	0	0	0.13	0.74	1.84	0.51	0.76
ENSMUSG00000031665	Sall1	spalt like transcription factor 1 [Source:MGI Symbol;Acc:MGI:1889585]	0.150647785	-2.730748629	2.74E-05	5.60E-04	yes	down	0.024	0.168	0	0.03	0.04	0.02	0.03	0.15	0.12	0.29	0.16	0.12
ENSMUSG00000095497	Igkv1-122	immunoglobulin kappa chain variable 1-122 [Source:MGI Symbol;Acc:MGI:4439722]	0.09827044	-3.347098671	5.11E-07	2.29E-05	yes	down	22.256	242.186	33.85	36.55	6.1	24.68	10.1	444.37	149.88	124.44	437.86	54.38
