# Supplementary material for: An Integrative Transcriptomic and Metabolomic Study Revealed That Melatonin Plays a Protective Role in Chronic Lung Inflammation by Reducing Necroptosis
Source: Front Immunol. 2021 May 4;12:668002. doi: 10.3389/fimmu.2021.668002 (PMC8129533; doi:10.3389/fimmu.2021.668002)
Supplement: Supplementary Figure 1 — Score plots of PLS-DA based on the metabolic profile of COPD. (A) The plot of PLS-DA scores showing almost complete separation of Mel (red circles), Luz (blue triangles), LPS (green rhombi), and Con (gray squares). The classification parameters were R2X (cum) = 0.65, R2Y (cum) = 0.871, and Q2 (cum) = 0.536. (B) Validation model of PLS-DA. The R2 and Q2 intercept values were 0.4356 and −0.4317, respectively, after 200 permutations. [file DataSheet_1.zip › Table S5.pdf]

## Table S5-1. Integrative metabolic pathways enrichment analyses between LPS vs Con group.

| Metabolite name | Total | Expected | Hits | Raw p | -LOG10(p) | FDR | Impact | matched_features |
|---|---|---|---|---|---|---|---|---|
| Drug metabolism - cytochrome P450 | 39 | 3.1069 | 14 | 6.60E-07 | 14.23 | 5.55E-05 | 0.23684 | mmu:14262; mmu:226564; mmu:55990; mmu:14858; mmu:14859; mmu:14863; mmu:14864; mmu:14867; mmu:14869; mmu:54486; mmu:68312; mmu:11670; mmu:56847; mmu:11761 |
| Glycerolipid metabolism | 35 | 2.7883 | 11 | 4.69E-05 | 9.9672 | 0.0019704 | 0.70588 | mmu:15450; mmu:16891; mmu:67717; mmu:19012; mmu:67916; mmu:233549; mmu:11997; mmu:72535; mmu:16956; mmu:11605; mmu:331374 |
| Glutathione metabolism | 56 | 4.4612 | 14 | 7.50E-05 | 9.4982 | 0.0020996 | 0.50909 | cpd:C00025; cpd:C00097; cpd:C00077; mmu:14598; mmu:14776; mmu:14858; mmu:14859; mmu:14863; mmu:14864; mmu:14867; mmu:14869; mmu:54486; mmu:68312; mmu:20135 |
| Phenylalanine metabolism | 24 | 1.9119 | 8 | 0.00033839 | 7.9913 | 0.0071061 | 1.0435 | cpd:C00079; cpd:C00082; mmu:11670; mmu:56847; mmu:13195; mmu:11754; mmu:234724; mmu:14204 |
| Ether lipid metabolism | 39 | 3.1069 | 10 | 0.00066744 | 7.3121 | 0.011213 | 0.47368 | mmu:27226; mmu:19012; mmu:67916; mmu:18782; mmu:232889; mmu:104759; mmu:18807; mmu:270084; mmu:320981; mmu:22239 |
| Mucin type O-glycan biosynthesis | 22 | 1.7526 | 7 | 0.0011082 | 6.805 | 0.015515 | 0.33333 | mmu:56336; mmu:108150; mmu:207839; mmu:241391; mmu:271786; mmu:78754; mmu:218476 |
| Drug metabolism - other enzymes | 69 | 5.4969 | 13 | 0.0024703 | 6.0034 | 0.029644 | 0.23529 | mmu:21877; mmu:13897; mmu:234564; mmu:234671; mmu:110006; mmu:20135; mmu:14858; mmu:14859; mmu:14863; mmu:14864; mmu:14867; mmu:14869; mmu:68312 |
| Alanine, aspartate and glutamate metabolism | 61 | 4.8595 | 11 | 0.0074388 | 4.901 | 0.076956 | 0.7 | cpd:C01042; cpd:C00049; cpd:C00152; cpd:C00041; cpd:C00025; cpd:C00158; mmu:11484; mmu:14204; mmu:11898; mmu:14584; mmu:53320 |
| Phenylalanine, tyrosine and tryptophan biosynthesis | 11 | 0.87631 | 4 | 0.0082453 | 4.7981 | 0.076956 | 2.4 | cpd:C00079; cpd:C00082; mmu:234724; mmu:14204 |
| Aminoacyl-tRNA biosynthesis | 74 | 5.8952 | 12 | 0.012384 | 4.3914 | 0.10402 | 0.17808 | cpd:C00152; cpd:C00079; cpd:C00097; cpd:C00049; cpd:C00065; cpd:C00073; cpd:C00041; cpd:C00407; cpd:C00188; cpd:C00082; cpd:C00148; cpd:C00025 |
| Sphingolipid metabolism | 58 | 4.6205 | 10 | 0.014416 | 4.2394 | 0.10782 | 0.40351 | cpd:C00065; cpd:C00346; mmu:19012; mmu:67916; mmu:11605; mmu:22239; mmu:12091; mmu:230379; mmu:20397; mmu:56386 |
| Pantothenate and CoA biosynthesis | 34 | 2.7086 | 7 | 0.015403 | 4.1732 | 0.10782 | 0.42424 | cpd:C00049; cpd:C00097; cpd:C00099; mmu:18605; mmu:22361; mmu:26464; mmu:12035 |
| Arginine biosynthesis | 27 | 2.1509 | 6 | 0.016967 | 4.0765 | 0.10963 | 0.69231 | cpd:C00025; cpd:C00049; cpd:C00077; mmu:18126; mmu:217214; mmu:11898 |
| Glycosaminoglycan degradation | 44 | 3.5052 | 8 | 0.020451 | 3.8897 | 0.12271 | 0.55814 | mmu:15442; mmu:75612; mmu:50917; mmu:12091; mmu:15211; mmu:15212; mmu:27419; mmu:110006 |

| | | | | | | | |
|---|---|---|---|---|---|---|---|
| Glycosphingolipid biosynthesis - globo and isoglobo series | 31 | 2.4696 | 6 | 0.032358 | 3.4309 | 0.17396 | 0.8 | mmu:93961; mmu:15211; mmu:15212; mmu:14343; mmu:11605; mmu:227671 |
| Neomycin, kanamycin and gentamicin biosynthesis | 4 | 0.31866 | 2 | 0.034025 | 3.3806 | 0.17396 | 1.3333 | cpd:C00031; mmu:212032 |
| Glycerophospholipid metabolism | 86 | 6.8512 | 12 | 0.037071 | 3.2949 | 0.17396 | 0.36471 | cpd:C00346; cpd:C00189; mmu:270084; mmu:18782; mmu:232889; mmu:331374; mmu:19012; mmu:67916; mmu:104759; mmu:18807; mmu:241274; mmu:192654 |
| Histidine metabolism | 32 | 2.5493 | 6 | 0.037278 | 3.2894 | 0.17396 | 0.29032 | cpd:C00025; cpd:C00049; mmu:72535; mmu:11670; mmu:56847; mmu:11484 |
| Linoleic acid metabolism | 17 | 1.3543 | 4 | 0.04078 | 3.1996 | 0.17654 | 0.5 | mmu:226143; mmu:11687; mmu:18782; mmu:232889 |
| Arginine and proline metabolism | 78 | 6.2138 | 11 | 0.042033 | 3.1693 | 0.17654 | 0.55844 | cpd:C01157; cpd:C00148; cpd:C00025; cpd:C00077; mmu:18126; mmu:67092; mmu:72535; mmu:69215; mmu:67432; mmu:209027; mmu:320452 |
| Arachidonic acid metabolism | 79 | 6.2935 | 11 | 0.045548 | 3.089 | 0.18219 | 0.48718 | mmu:226143; mmu:72054; mmu:14776; mmu:11684; mmu:11687; mmu:18782; mmu:232889; mmu:17001; mmu:14598; mmu:21391; mmu:54486 |
| Glycolysis or Gluconeogenesis | 61 | 4.8595 | 9 | 0.049304 | 3.0098 | 0.18825 | 0.5 | cpd:C00267; mmu:72535; mmu:11670; mmu:56847; mmu:13807; mmu:226265; mmu:11676; mmu:212032; mmu:18534 |
| beta-Alanine metabolism | 44 | 3.5052 | 7 | 0.055978 | 2.8828 | 0.20444 | 0.65116 | cpd:C00099; cpd:C00049; mmu:11754; mmu:72535; mmu:11670; mmu:56847; mmu:80911 |
| Valine, leucine and isoleucine biosynthesis | 12 | 0.95597 | 3 | 0.06406 | 2.7479 | 0.22421 | 0.90909 | cpd:C00188; cpd:C00407; mmu:12035 |
| Butanoate metabolism | 29 | 2.3103 | 5 | 0.075366 | 2.5854 | 0.25323 | 0.21429 | cpd:C00025; mmu:15360; mmu:117147; mmu:20216; mmu:272428 |
| alpha-Linolenic acid metabolism | 22 | 1.7526 | 4 | 0.092029 | 2.3857 | 0.29732 | 0.66667 | mmu:80911; mmu:235674; mmu:18782; mmu:232889 |
| Galactose metabolism | 51 | 4.0629 | 7 | 0.10626 | 2.2418 | 0.33059 | 1.2 | cpd:C00267; cpd:C00031; mmu:11605; mmu:232714; mmu:11997; mmu:212032; mmu:12091 |
| Glycine, serine and threonine metabolism | 72 | 5.7358 | 9 | 0.11446 | 2.1675 | 0.34339 | 0.56338 | cpd:C00065; cpd:C00188; cpd:C00097; mmu:12411; mmu:67092; mmu:26912; mmu:11656; mmu:11754; mmu:14711 |
| Ubiquinone and other terpenoid-quinone biosynthesis | 17 | 1.3543 | 3 | 0.14829 | 1.9086 | 0.42953 | 0.4375 | cpd:C00082; mmu:234724; mmu:18104 |
| Riboflavin metabolism | 9 | 0.71698 | 2 | 0.157 | 1.8515 | 0.43858 | 0.5 | mmu:18605; mmu:11433 |
| Glycosphingolipid biosynthesis - ganglio series | 47 | 3.7442 | 6 | 0.16655 | 1.7925 | 0.43858 | 0.34783 | mmu:12091; mmu:15211; mmu:15212; mmu:14421; mmu:20447; mmu:26938 |
| Starch and sucrose metabolism | 37 | 2.9476 | 5 | 0.16708 | 1.7893 | 0.43858 | 0.77778 | cpd:C00031; mmu:212032; mmu:232714; mmu:18605; mmu:19309 |
| Nitrogen metabolism | 10 | 0.79665 | 2 | 0.18642 | 1.6797 | 0.47453 | 0.44444 | cpd:C00025; mmu:71934 |
| Metabolism of xenobiotics by cytochrome P450 | 117 | 9.3208 | 12 | 0.21704 | 1.5277 | 0.53622 | 0.28448 | mmu:14858; mmu:14859; mmu:14863; mmu:14864; mmu:14867; mmu:54486; mmu:68312; mmu:11670; mmu:56847; mmu:14869; mmu:15483; mmu:13107 |
| Tyrosine metabolism | 88 | 7.0105 | 9 | 0.26399 | 1.3319 | 0.63095 | 0.54023 | cpd:C00355; cpd:C00082; mmu:11670; mmu:56847; mmu:11754; mmu:13195; mmu:234724; mmu:14204; mmu:11761 |
| Retinol metabolism | 44 | 3.5052 | 5 | 0.27041 | 1.3078 | 0.63095 | 0.34884 | mmu:11668; mmu:19378; mmu:11761; mmu:79235; mmu:226143 |

| | | | | | | | | |
|---|---|---|---|---|---|---|---|---|
| Thiamine metabolism | 14 | 1.1153 | 2 | 0.30821 | 1.177 | 0.69971 | 0.23077 | cpd:C00097; mmu:66566 |
| Cysteine and methionine metabolism | 71 | 5.6562 | 7 | 0.33517 | 1.0931 | 0.7219 | 0.4 | cpd:C00065; cpd:C00073; cpd:C00097; mmu:234724; mmu:12411; mmu:14204; mmu:12035 |
| Fructose and mannose metabolism | 37 | 2.9476 | 4 | 0.34015 | 1.0784 | 0.7219 | 0.55556 | cpd:C00267; mmu:11997; mmu:212032; mmu:11676 |
| Lysine degradation | 49 | 3.9036 | 5 | 0.35104 | 1.0469 | 0.7219 | 0.3125 | cpd:C00956; mmu:30956; mmu:170442; mmu:72535; mmu:269132 |
| Tryptophan metabolism | 84 | 6.6918 | 8 | 0.35236 | 1.0431 | 0.7219 | 0.40964 | cpd:C00780; cpd:C05635; mmu:14204; mmu:13195; mmu:70789; mmu:72535; mmu:11761; mmu:21743 |
| Taurine and hypotaurine metabolism | 16 | 1.2746 | 2 | 0.36839 | 0.99863 | 0.73677 | 0.2 | cpd:C00097; mmu:14598 |
| Porphyrin and chlorophyll metabolism | 53 | 4.2222 | 5 | 0.41653 | 0.87579 | 0.81369 | 0.25 | cpd:C00025; mmu:11656; mmu:110006; mmu:15368; mmu:109778 |
| Glycosaminoglycan biosynthesis - chondroitin sulfate / dermatan sulfate | 18 | 1.434 | 2 | 0.42644 | 0.85229 | 0.81411 | 0.35294 | mmu:234356; mmu:233781 |
| Nicotinate and nicotinamide metabolism | 43 | 3.4256 | 4 | 0.45176 | 0.79461 | 0.84249 | 0.30952 | cpd:C00049; mmu:18605; mmu:12182; mmu:11761 |
| Amino sugar and nucleotide sugar metabolism | 81 | 6.4528 | 7 | 0.47004 | 0.75494 | 0.84249 | 0.25 | cpd:C00267; mmu:26384; mmu:245847; mmu:14584; mmu:212032; mmu:15211; mmu:15212 |
| Pentose and glucuronate interconversions | 32 | 2.5493 | 3 | 0.47538 | 0.74365 | 0.84249 | 0.51613 | cpd:C00379; mmu:110006; mmu:11997 |
| Purine metabolism | 169 | 13.463 | 14 | 0.48143 | 0.731 | 0.84249 | 0.32738 | cpd:C00366; cpd:C00147; mmu:20135; mmu:54611; mmu:218461; mmu:104111; mmu:11513; mmu:432530; mmu:215446; mmu:66566; mmu:11821; mmu:14544; mmu:18585; mmu:18605 |
| Vitamin B6 metabolism | 21 | 1.673 | 2 | 0.50795 | 0.67738 | 0.87076 | 0.4 | mmu:57028; mmu:11761 |
| Valine, leucine and isoleucine degradation | 88 | 7.0105 | 7 | 0.56084 | 0.57831 | 0.89506 | 0.28736 | cpd:C00407; mmu:15360; mmu:12035; mmu:235674; mmu:14204; mmu:11761; mmu:72535 |
| Phosphonate and phosphinate metabolism | 10 | 0.79665 | 1 | 0.56474 | 0.57139 | 0.89506 | 0.11111 | cpd:C03557 |
| Synthesis and degradation of ketone bodies | 10 | 0.79665 | 1 | 0.56474 | 0.57139 | 0.89506 | 0.33333 | mmu:15360 |
| D-Glutamine and D-glutamate metabolism | 10 | 0.79665 | 1 | 0.56474 | 0.57139 | 0.89506 | 0.33333 | cpd:C00025 |
| Inositol phosphate metabolism | 69 | 5.4969 | 5 | 0.65508 | 0.42299 | 1 | 0.10294 | mmu:234779; mmu:74055; mmu:18707; mmu:30955; mmu:18705 |
| Glyoxylate and dicarboxylate metabolism | 56 | 4.4612 | 4 | 0.66441 | 0.40885 | 1 | 0.23636 | cpd:C00158; cpd:C00065; cpd:C00025; mmu:67432 |
| Pyruvate metabolism | 45 | 3.5849 | 3 | 0.70891 | 0.34403 | 1 | 0.27273 | mmu:18534; mmu:109264; mmu:72535 |
| Lipoic acid metabolism | 15 | 1.195 | 1 | 0.71323 | 0.33795 | 1 | 0.14286 | mmu:117147 |
| Phosphatidylinositol signaling system | 74 | 5.8952 | 5 | 0.71566 | 0.33456 | 1 | 0.23288 | mmu:234779; mmu:74055; mmu:18707; mmu:18705; mmu:331374 |
| Ascorbate and aldarate metabolism | 17 | 1.3543 | 1 | 0.75738 | 0.27789 | 1 | 0.25 | mmu:72535 |
| Sulfur metabolism | 18 | 1.434 | 1 | 0.77685 | 0.25251 | 1 | 0.11765 | mmu:20342 |
| Selenocompound metabolism | 35 | 2.7883 | 2 | 0.78172 | 0.24626 | 1 | 0.14706 | cpd:C00041; mmu:21743 |

| | | | | | | | | |
|---|---|---|---|---|---|---|---|---|
| Terpenoid backbone biosynthesis | 36 | 2.8679 | 2 | 0.79497 | 0.22945 | 1 | 0.14286 | mmu:15360; mmu:192156 |
| Citrate cycle (TCA cycle) | 42 | 3.3459 | 2 | 0.86051 | 0.15023 | 1 | 0.19512 | cpd:C00158; mmu:18534 |
| Steroid biosynthesis | 82 | 6.5325 | 4 | 0.90401 | 0.10092 | 1 | 0.19753 | cpd:C05437; mmu:13360; mmu:16889; mmu:20652 |
| Propanoate metabolism | 48 | 3.8239 | 2 | 0.90642 | 0.098256 | 1 | 0.12766 | cpd:C00099; mmu:80911 |
| Pyrimidine metabolism | 99 | 7.8868 | 5 | 0.90796 | 0.096553 | 1 | 0.27551 | cpd:C00099; mmu:20135; mmu:215446; mmu:21877; mmu:18605 |
| Fatty acid elongation | 75 | 5.9748 | 3 | 0.94675 | 0.054721 | 1 | 0.081081 | mmu:66775; mmu:26897; mmu:54325 |
| Glycosphingolipid biosynthesis - lacto and neolacto series | 102 | 8.1258 | 4 | 0.96907 | 0.031422 | 1 | 0.37624 | mmu:14345; mmu:14347; mmu:14343; mmu:93961 |
| Fatty acid degradation | 102 | 8.1258 | 4 | 0.96907 | 0.031422 | 1 | 0.73267 | mmu:80911; mmu:235674; mmu:72535; mmu:94180 |
| Pentose phosphate pathway | 47 | 3.7442 | 1 | 0.98058 | 0.019609 | 1 | 0.086957 | mmu:11676 |
| Biosynthesis of unsaturated fatty acids | 47 | 3.7442 | 1 | 0.98058 | 0.019609 | 1 | 0.47826 | mmu:26897 |
| Folate biosynthesis | 60 | 4.7799 | 1 | 0.99357 | 0.0064532 | 1 | 0.067797 | mmu:11997 |
| Steroid hormone biosynthesis | 175 | 13.941 | 5 | 0.99898 | 0.0010186 | 1 | 0.1954 | mmu:13079; mmu:226143; mmu:56448; mmu:15483; mmu:15485 |
| Primary bile acid biosynthesis | 90 | 7.1698 | 1 | 0.99951 | 0.00049027 | 1 | 0.022472 | mmu:12642 |
| Fatty acid biosynthesis | 129 | 10.277 | 1 | 0.99998 | 1.64E-05 | 1 | 0.03125 | mmu:94180 |

Table S5-2. Annotation of metabolites and genes between LPS vs Con group.

| Gene | Entrez | Symbol | Name | Metabolite KEGG ID | Name |
|---|---|---|---|---|---|
| Tnfrsf1b | 21938 | Tnfrsf1b | "tumor necrosis factor receptor superfamily, member 1b" | C01601 | Pelargonic acid |
| Cybb | 13058 | Cybb | "cytochrome b-245, beta polypeptide" | C00152 | L-Asparagine |
| Gpnmb | 93695 | Gpnmb | glycoprotein (transmembrane) nmb | C00490 | Itaconic acid |
| Psap | 19156 | Psap | prosaposin | C00065 | L-Serine |
| Tyrobp | 22177 | Tyrobp | TYRO protein tyrosine kinase binding protein | C01835 | Maltotriose |
| Sirpb1c | 100038947 | Sirpb1c | signal-regulatory protein beta 1C | C03557 | Ciliatine |
| Ctsd | 13033 | Ctsd | cathepsin D | C00073 | L-Methionine |
| Stac2 | 217154 | Stac2 | SH3 and cysteine rich domain 2 | C00079 | L-Phenylalanine |
| Man2b1 | 17159 | Man2b1 | "mannosidase 2, alpha B1" | C00188 | L-Threonine |
| Tlr7 | 170743 | Tlr7 | toll-like receptor 7 | C05437 | Zymosterol intermediate 2 |
| Ctsb | 13030 | Ctsb | cathepsin B | C00366 | Uric acid |
| Fcgr3 | 14131 | Fcgr3 | "Fc receptor, IgG, low affinity III" | C12535 | Tranexamic Acid |
| Spp1 | 20750 | Spp1 | secreted phosphoprotein 1 | C00407 | L-Isoleucine |
| Ctss | 13040 | Ctss | cathepsin S | C05635 | 5-Hydroxyindoleacetic acid |
| Tlr13 | 279572 | Tlr13 | toll-like receptor 13 | C01157 | 4-Hydroxyproline |
| Ftl1 | 14325 | Ftl1 | ferritin light polypeptide 1 | C00425 | Dehydroascorbic acid |
| Mpeg1 | 17476 | Mpeg1 | macrophage expressed gene 1 | C00148 | L-Proline |
| Itgb2 | 16414 | Itgb2 | integrin beta 2 | C08243 | Melezitose |
| Pld3 | 18807 | Pld3 | "phospholipase D family, member 3" | C00031 | D-Glucose |
| Zranb3 | 226409 | Zranb3 | "zinc finger, RAN-binding domain containing 3" | C03661 | 1-Kestose |
| Hvcn1 | 74096 | Hvcn1 | hydrogen voltage-gated channel 1 | C00082 | L-Tyrosine |
| Slc37a2 | 56857 | Slc37a2 | "solute carrier family 37 (glycerol-3-phosphate transporter), member 2" | C00041 | L-Alanine |
| Lhfpl2 | 218454 | Lhfpl2 | lipoma HMGIC fusion partner-like 2 | C01042 | N-Acetyl-L-aspartic acid |
| Myo5a | 17918 | Myo5a | myosin VA | C03139 | Guanidinosuccinic acid |
| Laptm5 | 16792 | Laptm5 | lysosomal-associated protein transmembrane 5 | C02896 | Putrescine |
| Nckap1l | 105855 | Nckap1l | NCK associated protein 1 like | C00049 | L-Aspartic acid |
| Clec4d | 17474 | Clec4d | "C-type lectin domain family 4, member d" | C00189 | Ethanolamine |
| Pnpla7 | 241274 | Pnpla7 | patatin-like phospholipase domain containing 7 | C00263 | L-Homoserine |

| | | | | | |
|---|---|---|---|---|---|
| Ctsz | 64138 | Ctsz | cathepsin Z | C00077 | Ornithine |
| Tlr8 | 170744 | Tlr8 | toll-like receptor 8 | C00158 | Citric acid |
| Mfsd12 | 73822 | Mfsd12 | major facilitator superfamily domain containing 12 | C00355 | L-Dopa |
| Fcer1g | 14127 | Fcer1g | "Fc receptor, IgE, high affinity I, gamma polypeptide" | C06562 | Catechin |
| Apobec1 | 11810 | Apobec1 | "apolipoprotein B mRNA editing enzyme, catalytic polypeptide 1" | C00956 | Aminoadipic acid |
| Apoe | 11816 | Apoe | apolipoprotein E | C00267 | Alpha-D-Glucose |
| Bhlhe41 | 79362 | Bhlhe41 | "basic helix-loop-helix family, member e41" | C00346 | O-Phosphoethanolamine |
| Csf2rb | 12983 | Csf2rb | "colony stimulating factor 2 receptor, beta, low-affinity (granulocyte-macrophage)" | C00025 | L-Glutamic acid |
| Lpcat2 | 270084 | Lpcat2 | lysophosphatidylcholine acyltransferase 2 | C00780 | Serotonin |
| Mmp12 | 17381 | Mmp12 | matrix metallopeptidase 12 | C00147 | Adenine |
| Myo1f | 17916 | Myo1f | myosin IF | C00099 | Beta-Alanine |
| Pirb | 18733 | Pirb | paired Ig-like receptor B | C00097 | L-Cysteine |
| Glipr1 | 73690 | Glipr1 | GLI pathogenesis-related 1 (glioma) | C01770 | Gamma-Butyrolactone |
| Fam20c | 80752 | Fam20c | "family with sequence similarity 20, member C" | C00379 | D-Xylitol |
| Lipa | 16889 | Lipa | lysosomal acid lipase A | | |
| Cd68 | 12514 | Cd68 | CD68 antigen | | |
| Msr1 | 20288 | Msr1 | macrophage scavenger receptor 1 | | |
| Fcgr2b | 14130 | Fcgr2b | "Fc receptor, IgG, low affinity IIb" | | |
| Ly9 | 17085 | Ly9 | lymphocyte antigen 9 | | |
| Cd200r1 | 57781 | Cd200r1 | CD200 receptor 1 | | |
| Cyth4 | 72318 | Cyth4 | cytohesin 4 | | |
| Sirpa | 19261 | Sirpa | signal-regulatory protein alpha | | |
| H2-M2 | 14990 | H2-M2 | "histocompatibility 2, M region locus 2" | | |
| Cd300c2 | 140497 | Cd300c2 | CD300C molecule 2 | | |
| Csf2rb2 | 12984 | Csf2rb2 | "colony stimulating factor 2 receptor, beta 2, low-affinity (granulocyte-macrophage)" | | |
| Ncf1 | 17969 | Ncf1 | neutrophil cytosolic factor 1 | | |
| Cd84 | 12523 | Cd84 | CD84 antigen | | |

| | | | |
|---|---|---|---|
| Selplg | 20345 | Selplg | "selectin, platelet (p-selectin) ligand" |
| Hexa | 15211 | Hexa | hexosaminidase A |
| Syk | 20963 | Syk | spleen tyrosine kinase |
| Cyba | 13057 | Cyba | "cytochrome b-245, alpha polypeptide" |
| Pla2g2d | 18782 | Pla2g2d | "phospholipase A2, group IID" |
| Clmp | 71566 | Clmp | CXADR-like membrane protein |
| Itgax | 16411 | Itgax | integrin alpha X |
| Cd274 | 60533 | Cd274 | CD274 antigen |
| Slamf7 | 75345 | Slamf7 | SLAM family member 7 |
| Ptgir | 19222 | Ptgir | prostaglandin I receptor (IP) |
| Sirpb1a | 320832 | Sirpb1a | signal-regulatory protein beta 1A |
| Gm49339 | NA | NA | NA |
| Cfb | 14962 | Cfb | complement factor B |
| Gm2a | 14667 | Gm2a | GM2 ganglioside activator protein |
| Pianp | 319352 | Pianp | PILR alpha associated neural protein |
| Ccr1 | 12768 | Ccr1 | chemokine (C-C motif) receptor 1 |
| Csf1r | 12978 | Csf1r | colony stimulating factor 1 receptor |
| Ccr5 | 12774 | Ccr5 | chemokine (C-C motif) receptor 5 |
| Slc9a4 | 110895 | Slc9a4 | "solute carrier family 9 (sodium/hydrogen exchanger), member 4" |
| Slc11a1 | 18173 | Slc11a1 | "solute carrier family 11 (proton-coupled divalent metal ion transporters), member 1" |
| Pik3r5 | 320207 | Pik3r5 | phosphoinositide-3-kinase regulatory subunit 5 |
| Clec4n | 56620 | Clec4n | "C-type lectin domain family 4, member n" |
| Nfam1 | 74039 | Nfam1 | Nfat activating molecule with ITAM motif 1 |
| Kynu | 70789 | Kynu | kynureninase |

| | | | |
|---|---|---|---|
| Ncf2 | 17970 | Ncf2 | neutrophil cytosolic factor 2 |
| Dock2 | 94176 | Dock2 | dedicator of cyto-kinesis 2 |
| Lrp12 | 239393 | Lrp12 | low density lipoprotein-related protein 12 |
| Fn1 | 14268 | Fn1 | fibronectin 1 |
| Ear1 | 13586 | Ear1 | "eosinophil-associated, ribonuclease A family, member 1" |
| Ctsa | 19025 | Ctsa | cathepsin A |
| Ptafr | 19204 | Ptafr | platelet-activating factor receptor |
| Ms4a6d | 68774 | Ms4a6d | "membrane-spanning 4-domains, subfamily A, member 6D" |
| Itgal | 16408 | Itgal | integrin alpha L |
| Mcemp1 | 69189 | Mcemp1 | mast cell expressed membrane protein 1 |
| Nrp2 | 18187 | Nrp2 | neuropilin 2 |
| Fcgr1 | 14129 | Fcgr1 | "Fc receptor, IgG, high affinity I" |
| Ctsc | 13032 | Ctsc | cathepsin C |
| Clec4a2 | 26888 | Clec4a2 | "C-type lectin domain family 4, member a2" |
| Soat1 | 20652 | Soat1 | sterol O-acyltransferase 1 |
| Clec7a | 56644 | Clec7a | "C-type lectin domain family 7, member a" |
| Clec4a3 | 73149 | Clec4a3 | "C-type lectin domain family 4, member a3" |
| C3ar1 | 12267 | C3ar1 | complement component 3a receptor 1 |
| Sgpl1 | 20397 | Sgpl1 | sphingosine phosphate lyase 1 |
| Lair1 | 52855 | Lair1 | leukocyte-associated Ig-like receptor 1 |
| Gusb | 110006 | Gusb | "glucuronidase, beta" |
| Pik3cg | 30955 | Pik3cg | "phosphatidylinositol-4,5-bisphosphate 3-kinase catalytic subunit gamma" |
| Ptk2b | 19229 | Ptk2b | PTK2 protein tyrosine kinase 2 beta |
| Adgre1 | 13733 | Adgre1 | adhesion G protein-coupled receptor E1 |
| Fpr2 | 14289 | Fpr2 | formyl peptide receptor 2 |

| | | | |
|---|---|---|---|
| Sema4d | 20354 | Sema4d | "sema domain, immunoglobulin domain (Ig), transmembrane domain (TM) and short cytoplasmic domain, (semaphorin) 4D" |
| Edem1 | 192193 | Edem1 | "ER degradation enhancer, mannosidase alpha-like 1" |
| Cd200r4 | 239849 | Cd200r4 | CD200 receptor 4 |
| Tnfaip8l2 | 69769 | Tnfaip8l2 | "tumor necrosis factor, alpha-induced protein 8-like 2" |
| Ctsk | 13038 | Ctsk | cathepsin K |
| Cotl1 | 72042 | Cotl1 | coactosin-like 1 (Dictyostelium) |
| Lgals3 | 16854 | Lgals3 | "lectin, galactose binding, soluble 3" |
| Ly86 | 17084 | Ly86 | lymphocyte antigen 86 |
| Unc93b1 | 54445 | Unc93b1 | "unc-93 homolog B1, TLR signaling regulator" |
| Tifab | 212937 | Tifab | "TRAF-interacting protein with forkhead-associated domain, family member B" |
| Trpv2 | 22368 | Trpv2 | "transient receptor potential cation channel, subfamily V, member 2" |
| Capg | 12332 | Capg | "capping protein (actin filament), gelsolin-like" |
| Cd300lf | 246746 | Cd300lf | CD300 molecule like family member F |
| Grn | 14824 | Grn | granulin |
| Csf2ra | 12982 | Csf2ra | "colony stimulating factor 2 receptor, alpha, low-affinity (granulocyte-macrophage)" |
| Dpep2 | 319446 | Dpep2 | dipeptidase 2 |
| Itgam | 16409 | Itgam | integrin alpha M |
| Creg2 | 263764 | Creg2 | cellular repressor of E1A-stimulated genes 2 |
| Themis2 | 230787 | Themis2 | thymocyte selection associated family member 2 |
| Slc7a11 | 26570 | Slc7a11 | "solute carrier family 7 (cationic amino acid transporter, y+ system), member 11" |
| Vav1 | 22324 | Vav1 | vav 1 oncogene |
| Ptpn7 | 320139 | Ptpn7 | "protein tyrosine phosphatase, non-receptor type 7" |
| Gns | 75612 | Gns | glucosamine (N-acetyl)-6-sulfatase |
| Pik3ap1 | 83490 | Pik3ap1 | phosphoinositide-3-kinase adaptor protein 1 |

| | | | |
|---|---|---|---|
| Gnpda1 | 26384 | Gnpda1 | glucosamine-6-phosphate deaminase 1 |
| Tbxas1 | 21391 | Tbxas1 | "thromboxane A synthase 1, platelet" |
| Lcp1 | 18826 | Lcp1 | lymphocyte cytosolic protein 1 |
| B2m | 12010 | B2m | beta-2 microglobulin |
| Cd300a | 217303 | Cd300a | CD300A molecule |
| Sh3bp2 | 24055 | Sh3bp2 | SH3-domain binding protein 2 |
| Hmox1 | 15368 | Hmox1 | heme oxygenase 1 |
| Gla | 11605 | Gla | "galactosidase, alpha" |
| Arhgap25 | 232201 | Arhgap25 | Rho GTPase activating protein 25 |
| Cd22 | 12483 | Cd22 | CD22 antigen |
| Slc6a6 | 21366 | Slc6a6 | "solute carrier family 6 (neurotransmitter transporter, taurine), member 6" |
| Gm15931 | 112136069 | Gm15931 | predicted gene 15931 |
| Itgb5 | 16419 | Itgb5 | integrin beta 5 |
| Rassf4 | 213391 | Rassf4 | Ras association (RalGDS/AF-6) domain family member 4 |
| Enpp1 | 18605 | Enpp1 | ectonucleotide pyrophosphatase/phosphodiesterase 1 |
| Cxcl16 | 66102 | Cxcl16 | chemokine (C-X-C motif) ligand 16 |
| C1qc | 12262 | C1qc | "complement component 1, q subcomponent, C chain" |
| Procr | 19124 | Procr | "protein C receptor, endothelial" |
| Rnf149 | 67702 | Rnf149 | ring finger protein 149 |
| Bst1 | 12182 | Bst1 | bone marrow stromal cell antigen 1 |
| Pla2g15 | 192654 | Pla2g15 | "phospholipase A2, group XV" |
| Slc7a2 | 11988 | Slc7a2 | "solute carrier family 7 (cationic amino acid transporter, y+ system), member 2" |
| Atp6v0c | 11984 | Atp6v0c | "ATPase, H+ transporting, lysosomal V0 subunit C" |
| Lgmn | 19141 | Lgmn | legumain |
| Arl11 | 219144 | Arl11 | ADP-ribosylation factor-like 11 |

| | | | |
|---|---|---|---|
| Lcp2 | 16822 | Lcp2 | lymphocyte cytosolic protein 2 |
| Tlr2 | 24088 | Tlr2 | toll-like receptor 2 |
| Tgfbr1 | 21812 | Tgfbr1 | "transforming growth factor, beta receptor I" |
| Amz1 | 231842 | Amz1 | archaelysin family metallopeptidase 1 |
| Naip2 | 17948 | Naip2 | "NLR family, apoptosis inhibitory protein 2" |
| Ly75 | 17076 | Ly75 | lymphocyte antigen 75 |
| Apobr | 171504 | Apobr | apolipoprotein B receptor |
| H2-K1 | 14972 | H2-K1 | "histocompatibility 2, K1, K region" |
| Apbb1ip | 54519 | Apbb1ip | "amyloid beta (A4) precursor protein-binding, family B, member 1 interacting protein" |
| Cd53 | 12508 | Cd53 | CD53 antigen |
| Galnt7 | 108150 | Galnt7 | polypeptide N-acetylgalactosaminyltransferase 7 |
| Cd52 | 23833 | Cd52 | CD52 antigen |
| Wfdc17 | 100034251 | Wfdc17 | WAP four-disulfide core domain 17 |
| Ifi30 | 65972 | Ifi30 | interferon gamma inducible protein 30 |
| Hk3 | 212032 | Hk3 | hexokinase 3 |
| Xylt1 | 233781 | Xylt1 | xylosyltransferase 1 |
| P2ry6 | 233571 | P2ry6 | "pyrimidinergic receptor P2Y, G-protein coupled, 6" |
| Fcgr4 | 246256 | Fcgr4 | "Fc receptor, IgG, low affinity IV" |
| F10 | 14058 | F10 | coagulation factor X |
| Ebi3 | 50498 | Ebi3 | Epstein-Barr virus induced gene 3 |
| Creg1 | 433375 | Creg1 | cellular repressor of E1A-stimulated genes 1 |
| Cstb | 13014 | Cstb | cystatin B |
| Olr1 | 108078 | Olr1 | oxidized low density lipoprotein (lectin-like) receptor 1 |
| Acpp | 56318 | Acpp | "acid phosphatase, prostate" |

| | | | |
|---|---|---|---|
| Arhgap30 | 226652 | Arhgap30 | Rho GTPase activating protein 30 |
| Col24a1 | 71355 | Col24a1 | "collagen, type XXIV, alpha 1" |
| Slpi | 20568 | Slpi | secretory leukocyte peptidase inhibitor |
| Il1rn | 16181 | Il1rn | interleukin 1 receptor antagonist |
| Arhgap9 | 216445 | Arhgap9 | Rho GTPase activating protein 9 |
| Igsf6 | 80719 | Igsf6 | "immunoglobulin superfamily, member 6" |
| Cd48 | 12506 | Cd48 | CD48 antigen |
| Adap1 | 231821 | Adap1 | ArfGAP with dual PH domains 1 |
| Slc15a3 | 65221 | Slc15a3 | "solute carrier family 15, member 3" |
| Nek6 | 59126 | Nek6 | NIMA (never in mitosis gene a)-related expressed kinase 6 |
| Rnf180 | 71816 | Rnf180 | ring finger protein 180 |
| Snx5 | 69178 | Snx5 | sorting nexin 5 |
| Naip5 | 17951 | Naip5 | "NLR family, apoptosis inhibitory protein 5" |
| Tcirg1 | 27060 | Tcirg1 | "T cell, immune regulator 1, ATPase, H+ transporting, lysosomal V0 protein A3" |
| Kcnab2 | 16498 | Kcnab2 | "potassium voltage-gated channel, shaker-related subfamily, beta member 2" |
| Bcl2a1a | 12044 | Bcl2a1a | B cell leukemia/lymphoma 2 related protein A1a |
| Clec12a | 232413 | Clec12a | "C-type lectin domain family 12, member a" |
| Irf8 | 15900 | Irf8 | interferon regulatory factor 8 |
| Dock10 | 210293 | Dock10 | dedicator of cytokinesis 10 |
| Rilpl2 | 80291 | Rilpl2 | Rab interacting lysosomal protein-like 2 |
| Acp5 | 11433 | Acp5 | "acid phosphatase 5, tartrate resistant" |
| Fxyd5 | 18301 | Fxyd5 | FXYD domain-containing ion transport regulator 5 |
| Abhd12 | 76192 | Abhd12 | abhydrolase domain containing 12 |
| Cd300lb | 217304 | Cd300lb | CD300 molecule like family member B |
| Rftn1 | 76438 | Rftn1 | raftlin lipid raft linker 1 |

| | | | |
|---|---|---|---|
| Sp140 | 434484 | Sp140 | Sp140 nuclear body protein |
| Evi2a | 14017 | Evi2a | ecotropic viral integration site 2a |
| Clec5a | 23845 | Clec5a | "C-type lectin domain family 5, member a" |
| Atp6v1b2 | 11966 | Atp6v1b2 | "ATPase, H+ transporting, lysosomal V1 subunit B2" |
| Tbc1d9 | 71310 | Tbc1d9 | "TBC1 domain family, member 9" |
| Trem2 | 83433 | Trem2 | triggering receptor expressed on myeloid cells 2 |
| Ddhd1 | 114874 | Ddhd1 | DDHD domain containing 1 |
| Cd33 | 12489 | Cd33 | CD33 antigen |
| Saa3 | 20210 | Saa3 | serum amyloid A 3 |
| B4galnt1 | 14421 | B4galnt1 | "beta-1,4-N-acetyl-galactosaminyl transferase 1" |
| Edem2 | 108687 | Edem2 | "ER degradation enhancer, mannosidase alpha-like 2" |
| Lgals3bp | 19039 | Lgals3bp | "lectin, galactoside-binding, soluble, 3 binding protein" |
| Ptpn6 | 15170 | Ptpn6 | "protein tyrosine phosphatase, non-receptor type 6" |
| Rab32 | 67844 | Rab32 | "RAB32, member RAS oncogene family" |
| Tnfaip2 | 21928 | Tnfaip2 | "tumor necrosis factor, alpha-induced protein 2" |
| Blnk | 17060 | Blnk | B cell linker |
| Glb1 | 12091 | Glb1 | "galactosidase, beta 1" |
| Pira2 | 18725 | Pira2 | paired-Ig-like receptor A2 |
| Mafb | 16658 | Mafb | "v-maf musculoaponeurotic fibrosarcoma oncogene family, protein B (avian)" |
| Mfge8 | 17304 | Mfge8 | milk fat globule-EGF factor 8 protein |
| Cd63 | 12512 | Cd63 | CD63 antigen |
| Plek | 56193 | Plek | pleckstrin |
| Sirpb1b | 668101 | Sirpb1b | signal-regulatory protein beta 1B |
| AU020206 | 100504230 | AU020206 | expressed sequence AU020206 |
| Pik3cd | 18707 | Pik3cd | "phosphatidylinositol-4,5-bisphosphate 3-kinase catalytic subunit delta" |

| | | | |
|---|---|---|---|
| Gnptab | 432486 | Gnptab | "N-acetylglucosamine-1-phosphate transferase, alpha and beta subunits" |
| Ass1 | 11898 | Ass1 | argininosuccinate synthetase 1 |
| Evl | 14026 | Evl | Ena-vasodilator stimulated phosphoprotein |
| Pla2g7 | 27226 | Pla2g7 | "phospholipase A2, group VII (platelet-activating factor acetylhydrolase, plasma)" |
| Hacd4 | 66775 | Hacd4 | 3-hydroxyacyl-CoA dehydratase 4 |
| Ms4a7 | 109225 | Ms4a7 | "membrane-spanning 4-domains, subfamily A, member 7" |
| Ptprc | 19264 | Ptprc | "protein tyrosine phosphatase, receptor type, C" |
| Malt1 | 240354 | Malt1 | MALT1 paracaspase |
| Tnfrsf26 | 244237 | Tnfrsf26 | "tumor necrosis factor receptor superfamily, member 26" |
| Spi1 | 20375 | Spi1 | spleen focus forming virus (SFFV) proviral integration oncogene |
| Tmem173 | NA | NA | NA |
| Parvg | 64099 | Parvg | "parvin, gamma" |
| Ifi207 | 226691 | Ifi207 | interferon activated gene 207 |
| Hexb | 15212 | Hexb | hexosaminidase B |
| Rab7b | 226421 | Rab7b | "RAB7B, member RAS oncogene family" |
| Kcnn4 | 16534 | Kcnn4 | "potassium intermediate/small conductance calcium-activated channel, subfamily N, member 4" |
| Sh3pxd2b | 268396 | Sh3pxd2b | SH3 and PX domains 2B |
| Bcl2a1b | 12045 | Bcl2a1b | B cell leukemia/lymphoma 2 related protein A1b |
| Gbgt1 | 227671 | Gbgt1 | "globoside alpha-1,3-N-acetylgalactosaminyltransferase 1" |
| Klhl6 | 239743 | Klhl6 | kelch-like 6 |
| Alox5ap | 11690 | Alox5ap | arachidonate 5-lipoxygenase activating protein |
| Mapkapk3 | 102626 | Mapkapk3 | mitogen-activated protein kinase-activated protein kinase 3 |
| Card11 | 108723 | Card11 | "caspase recruitment domain family, member 11" |
| Igsf8 | 140559 | Igsf8 | "immunoglobulin superfamily, member 8" |

| | | | |
|---|---|---|---|
| Hcls1 | 15163 | Hcls1 | hematopoietic cell specific Lyn substrate 1 |
| Cd74 | 16149 | Cd74 | "CD74 antigen (invariant polypeptide of major histocompatibility complex, class II antigen-associated)" |
| Mcoln3 | 171166 | Mcoln3 | mucolipin 3 |
| Wdfy4 | 545030 | Wdfy4 | WD repeat and FYVE domain containing 4 |
| B4galt5 | 56336 | B4galt5 | "UDP-Gal:betaGlcNAc beta 1,4-galactosyltransferase, polypeptide 5" |
| Plcl2 | 224860 | Plcl2 | phospholipase C-like 2 |
| Unc119 | 22248 | Unc119 | unc-119 lipid binding chaperone |
| Ly6c2 | 100041546 | Ly6c2 | "lymphocyte antigen 6 complex, locus C2" |
| Metrnl | 210029 | Metrnl | "meteorin, glial cell differentiation regulator-like" |
| Atp6v0d2 | 242341 | Atp6v0d2 | "ATPase, H+ transporting, lysosomal V0 subunit D2" |
| Pycard | 66824 | Pycard | PYD and CARD domain containing |
| Dpp7 | 83768 | Dpp7 | dipeptidylpeptidase 7 |
| Tmem106a | 217203 | Tmem106a | transmembrane protein 106A |
| Arhgap19 | 71085 | Arhgap19 | Rho GTPase activating protein 19 |
| Abcg1 | 11307 | Abcg1 | ATP binding cassette subfamily G member 1 |
| Slc36a1 | 215335 | Slc36a1 | "solute carrier family 36 (proton/amino acid symporter), member 1" |
| Plcg2 | 234779 | Plcg2 | "phospholipase C, gamma 2" |
| Ncoa4 | 27057 | Ncoa4 | nuclear receptor coactivator 4 |
| Chil3 | 12655 | Chil3 | chitinase-like 3 |
| AI662270 | 100043636 | AI662270 | expressed sequence AI662270 |
| Plin2 | 11520 | Plin2 | perilipin 2 |
| Dcstamp | 75766 | Dcstamp | dendrocyte expressed seven transmembrane protein |
| Pld4 | 104759 | Pld4 | "phospholipase D family, member 4" |
| Plekhm2 | 69582 | Plekhm2 | "pleckstrin homology domain containing, family M (with RUN domain) member 2" |

| | | | |
|---|---|---|---|
| Mmp14 | 17387 | Mmp14 | matrix metallopeptidase 14 (membrane-inserted) |
| Siglec1 | 20612 | Siglec1 | "sialic acid binding Ig-like lectin 1, sialoadhesin" |
| Cd180 | 17079 | Cd180 | CD180 antigen |
| Ncf4 | 17972 | Ncf4 | neutrophil cytosolic factor 4 |
| Cd44 | 12505 | Cd44 | CD44 antigen |
| Fth1 | 14319 | Fth1 | ferritin heavy polypeptide 1 |
| C1qb | 12260 | C1qb | "complement component 1, q subcomponent, beta polypeptide" |
| Msrb1 | 27361 | Msrb1 | methionine sulfoxide reductase B1 |
| Cib2 | 56506 | Cib2 | calcium and integrin binding family member 2 |
| Galns | 50917 | Galns | galactosamine (N-acetyl)-6-sulfate sulfatase |
| Dmxl2 | 235380 | Dmxl2 | Dmx-like 2 |
| H2-D1 | 14964 | H2-D1 | "histocompatibility 2, D region locus 1" |
| Rnase6 | 78416 | Rnase6 | "ribonuclease, RNase A family, 6" |
| Tmem86a | 67893 | Tmem86a | transmembrane protein 86A |
| 1-Mar | NA | NA | NA |
| Igf1 | 16000 | Igf1 | insulin-like growth factor 1 |
| Slamf8 | 74748 | Slamf8 | SLAM family member 8 |
| Foxp3 | 20371 | Foxp3 | forkhead box P3 |
| Gsap | 212167 | Gsap | gamma-secretase activating protein |
| Axl | 26362 | Axl | AXL receptor tyrosine kinase |
| P2ry14 | 140795 | P2ry14 | "purinergic receptor P2Y, G-protein coupled, 14" |
| Prcp | 72461 | Prcp | prolylcarboxypeptidase (angiotensinase C) |
| Mmp2 | 17390 | Mmp2 | matrix metallopeptidase 2 |
| Sh3bgrl3 | 73723 | Sh3bgrl3 | SH3 domain binding glutamic acid-rich protein-like 3 |
| H2-Aa | 14960 | H2-Aa | "histocompatibility 2, class II antigen A, alpha" |

| | | | |
|---|---|---|---|
| Slc35c1 | 228368 | Slc35c1 | "solute carrier family 35, member C1" |
| Gas2l3 | 237436 | Gas2l3 | growth arrest-specific 2 like 3 |
| Plagl2 | 54711 | Plagl2 | pleiomorphic adenoma gene-like 2 |
| Sla | 20491 | Sla | src-like adaptor |
| Adcy7 | 11513 | Adcy7 | adenylate cyclase 7 |
| Mitf | 17342 | Mitf | melanogenesis associated transcription factor |
| Ighv1-72 | 619916 | Ighv1-72 | immunoglobulin heavy variable 1-72 |
| Plekho2 | 102595 | Plekho2 | "pleckstrin homology domain containing, family O member 2" |
| Snx20 | 71607 | Snx20 | sorting nexin 20 |
| Lyz2 | 17105 | Lyz2 | lysozyme 2 |
| Slc6a20a | 102680 | Slc6a20a | "solute carrier family 6 (neurotransmitter transporter), member 20A" |
| Dnase2a | 13423 | Dnase2a | deoxyribonuclease II alpha |
| AI467606 | 101602 | AI467606 | expressed sequence AI467606 |
| Tnfrsf9 | 21942 | Tnfrsf9 | "tumor necrosis factor receptor superfamily, member 9" |
| Ltbp2 | 16997 | Ltbp2 | latent transforming growth factor beta binding protein 2 |
| Tnfrsf23 | 79201 | Tnfrsf23 | "tumor necrosis factor receptor superfamily, member 23" |
| Rnf128 | 66889 | Rnf128 | ring finger protein 128 |
| Myof | 226101 | Myof | myoferlin |
| Ticam2 | 225471 | Ticam2 | toll-like receptor adaptor molecule 2 |
| Ighv1-62-2 | 238448 | Ighv1-62-2 | immunoglobulin heavy variable 1-62-2 |
| Zmynd15 | 574428 | Zmynd15 | "zinc finger, MYND-type containing 15" |
| 5830432E09Rik | 67765 | 5830432E09Rik | RIKEN cDNA 5830432E09 gene |
| Slfn8 | 276950 | Slfn8 | schlafen 8 |
| Prkcd | 18753 | Prkcd | "protein kinase C, delta" |
| Tifa | 211550 | Tifa | TRAF-interacting protein with forkhead-associated domain |

| | | | |
|---|---|---|---|
| Daglb | 231871 | Daglb | "diacylglycerol lipase, beta" |
| Derl3 | 70377 | Derl3 | "Der1-like domain family, member 3" |
| Rac2 | 19354 | Rac2 | Rac family small GTPase 2 |
| Galnt6 | 207839 | Galnt6 | polypeptide N-acetylgalactosaminyltransferase 6 |
| Fmn1 | 14260 | Fmn1 | formin 1 |
| Irf5 | 27056 | Irf5 | interferon regulatory factor 5 |
| Ptpro | 19277 | Ptpro | "protein tyrosine phosphatase, receptor type, O" |
| Fermt3 | 108101 | Fermt3 | fermitin family member 3 |
| Psd4 | 215632 | Psd4 | pleckstrin and Sec7 domain containing 4 |
| Slc29a3 | 71279 | Slc29a3 | "solute carrier family 29 (nucleoside transporters), member 3" |
| Fgr | 14191 | Fgr | "FGR proto-oncogene, Src family tyrosine kinase" |
| Igkc | 16071 | Igkc | immunoglobulin kappa constant |
| Tfec | 21426 | Tfec | transcription factor EC |
| 4930430E12Rik | 71639 | 4930430E12Rik | RIKEN cDNA 4930430E12 gene |
| Rcbtb2 | 105670 | Rcbtb2 | regulator of chromosome condensation (RCC1) and BTB (POZ) domain containing protein 2 |
| Ighv8-8 | 780938 | Ighv8-8 | immunoglobulin heavy variable 8-8 |
| Slc6a12 | 14411 | Slc6a12 | "solute carrier family 6 (neurotransmitter transporter, betaine/GABA), member 12" |
| Kcnj10 | 16513 | Kcnj10 | "potassium inwardly-rectifying channel, subfamily J, member 10" |
| Pstpip1 | 19200 | Pstpip1 | proline-serine-threonine phosphatase-interacting protein 1 |
| Ighv1-77 | 619994 | Ighv1-77 | immunoglobulin heavy variable 1-77 |
| Snx10 | 71982 | Snx10 | sorting nexin 10 |
| Cyp4v3 | 102294 | Cyp4v3 | "cytochrome P450, family 4, subfamily v, polypeptide 3" |
| Slamf9 | 98365 | Slamf9 | SLAM family member 9 |
| Lpar5 | 381810 | Lpar5 | lysophosphatidic acid receptor 5 |

| | | | |
|---|---|---|---|
| Ch25h | 12642 | Ch25h | cholesterol 25-hydroxylase |
| Aif1 | 11629 | Aif1 | allograft inflammatory factor 1 |
| Il10ra | 16154 | Il10ra | "interleukin 10 receptor, alpha" |
| Ccdc88b | 78317 | Ccdc88b | coiled-coil domain containing 88B |
| Slc8b1 | 170756 | Slc8b1 | "solute carrier family 8 (sodium/lithium/calcium exchanger), member B1" |
| Scpep1 | 74617 | Scpep1 | serine carboxypeptidase 1 |
| Gm26584 | 105244982 | Gm26584 | "predicted gene, 26584" |
| Btk | 12229 | Btk | Bruton agammaglobulinemia tyrosine kinase |
| Sash3 | 74131 | Sash3 | SAM and SH3 domain containing 3 |
| AI504432 | 229694 | AI504432 | expressed sequence AI504432 |
| Smpdl3b | 100340 | Smpdl3b | "sphingomyelin phosphodiesterase, acid-like 3B" |
| Dnase1l3 | 13421 | Dnase1l3 | deoxyribonuclease 1-like 3 |
| Trim29 | 72169 | Trim29 | tripartite motif-containing 29 |
| Ighv8-9 | 432709 | Ighv8-9 | immunoglobulin heavy variable V8-9 |
| Ms4a6c | 73656 | Ms4a6c | "membrane-spanning 4-domains, subfamily A, member 6C" |
| Stra6l | 74152 | Stra6l | STRA6-like |
| H2-Eb1 | 14969 | H2-Eb1 | "histocompatibility 2, class II antigen E beta" |
| Ap3s1 | 11777 | Ap3s1 | "adaptor-related protein complex 3, sigma 1 subunit" |
| Mreg | 381269 | Mreg | melanoregulin |
| Ifi204 | 15951 | Ifi204 | interferon activated gene 204 |
| Mcoln2 | 68279 | Mcoln2 | mucolipin 2 |
| Slfn2 | 20556 | Slfn2 | schlafen 2 |
| Arhgdib | 11857 | Arhgdib | "Rho, GDP dissociation inhibitor (GDI) beta" |
| Mag | 17136 | Mag | myelin-associated glycoprotein |
| Ppfia4 | 68507 | Ppfia4 | "protein tyrosine phosphatase, receptor type, f polypeptide (PTPRF), interacting |

| | | | |
|---|---|---|---|
| | | | protein (liprin), alpha 4" |
| Matk | 17179 | Matk | megakaryocyte-associated tyrosine kinase |
| Cybc1 | 217370 | Cybc1 | cytochrome b 245 chaperone 1 |
| Ighv1-52 | 382696 | Ighv1-52 | immunoglobulin heavy variable 1-52 |
| H2-Ab1 | 14961 | H2-Ab1 | "histocompatibility 2, class II antigen A, beta 1" |
| Tm6sf1 | 107769 | Tm6sf1 | transmembrane 6 superfamily member 1 |
| Klhdc4 | 234825 | Klhdc4 | kelch domain containing 4 |
| Naglu | 27419 | Naglu | alpha-N-acetylglucosaminidase (Sanfilippo disease IIIB) |
| Acod1 | 16365 | Acod1 | aconitate decarboxylase 1 |
| Lilr4b | 14727 | Lilr4b | "leukocyte immunoglobulin-like receptor, subfamily B, member 4B" |
| Casp1 | 12362 | Casp1 | caspase 1 |
| Fes | 14159 | Fes | feline sarcoma oncogene |
| Igkv6-17 | 667865 | Igkv6-17 | immunoglobulin kappa variable 6-17 |
| Gm6264 | 621835 | Gm6264 | predicted gene 6264 |
| Zfp385a | 29813 | Zfp385a | zinc finger protein 385A |
| Pgap1 | 241062 | Pgap1 | post-GPI attachment to proteins 1 |
| AA467197 | 433470 | AA467197 | expressed sequence AA467197 |
| Atp1a3 | 232975 | Atp1a3 | "ATPase, Na+/K+ transporting, alpha 3 polypeptide" |
| Aprt | 11821 | Aprt | adenine phosphoribosyl transferase |
| Tec | 21682 | Tec | tec protein tyrosine kinase |
| Igkv4-55 | 385253 | Igkv4-55 | immunoglobulin kappa variable 4-55 |
| Rasa4 | 54153 | Rasa4 | RAS p21 protein activator 4 |
| Camk1d | 227541 | Camk1d | calcium/calmodulin-dependent protein kinase ID |
| Mvd | 192156 | Mvd | mevalonate (diphospho) decarboxylase |
| Vnn3 | 26464 | Vnn3 | vanin 3 |

| | | | |
|---|---|---|---|
| Arrb2 | 216869 | Arrb2 | "arrestin, beta 2" |
| Cd300ld5 | 100043125 | Cd300ld5 | CD300 molecule like family member D5 |
| Igkv12-46 | 692245 | Igkv12-46 | immunoglobulin kappa variable 12-46 |
| Lpxn | 107321 | Lpxn | leupaxin |
| Scimp | 327957 | Scimp | SLP adaptor and CSK interacting membrane protein |
| Trpm2 | 28240 | Trpm2 | "transient receptor potential cation channel, subfamily M, member 2" |
| Igha | 238447 | Igha | immunoglobulin heavy constant alpha |
| Hpgds | 54486 | Hpgds | hematopoietic prostaglandin D synthase |
| Clcn7 | 26373 | Clcn7 | "chloride channel, voltage-sensitive 7" |
| Emp3 | 13732 | Emp3 | epithelial membrane protein 3 |
| Gpr35 | 64095 | Gpr35 | G protein-coupled receptor 35 |
| Lrrc25 | 211228 | Lrrc25 | leucine rich repeat containing 25 |
| Batf | 53314 | Batf | "basic leucine zipper transcription factor, ATF-like" |
| Bhlha15 | 17341 | Bhlha15 | "basic helix-loop-helix family, member a15" |
| Clec4e | 56619 | Clec4e | "C-type lectin domain family 4, member e" |
| Tent5a | 212943 | Tent5a | terminal nucleotidyltransferase 5A |
| Iqgap2 | 544963 | Iqgap2 | IQ motif containing GTPase activating protein 2 |
| Gm5431 | 432555 | Gm5431 | predicted gene 5431 |
| Npy | 109648 | Npy | neuropeptide Y |
| Slc7a8 | 50934 | Slc7a8 | "solute carrier family 7 (cationic amino acid transporter, y+ system), member 8" |
| Tmem268 | 230279 | Tmem268 | transmembrane protein 268 |
| Fmnl1 | 57778 | Fmnl1 | formin-like 1 |
| Gpr68 | 238377 | Gpr68 | G protein-coupled receptor 68 |
| Lcn2 | 16819 | Lcn2 | lipocalin 2 |
| Ighv1-34 | 628614 | Ighv1-34 | immunoglobulin heavy variable 1-34 |

| | | | |
|---|---|---|---|
| Igkv8-19 | 232065 | Igkv8-19 | immunoglobulin kappa variable 8-19 |
| Sema4a | 20351 | Sema4a | "sema domain, immunoglobulin domain (Ig), transmembrane domain (TM) and short cytoplasmic domain, (semaphorin) 4A" |
| H2-DMb1 | 14999 | H2-DMb1 | "histocompatibility 2, class II, locus Mb1" |
| Havcr2 | 171285 | Havcr2 | hepatitis A virus cellular receptor 2 |
| AU022793 | 105976 | AU022793 | expressed sequence AU022793 |
| Hpse | 15442 | Hpse | heparanase |
| Egfem1 | 75740 | Egfem1 | EGF-like and EMI domain containing 1 |
| Cx3cr1 | 13051 | Cx3cr1 | chemokine (C-X3-C motif) receptor 1 |
| Aim2 | 383619 | Aim2 | absent in melanoma 2 |
| Ttyh2 | 117160 | Ttyh2 | tweety family member 2 |
| Slc27a6 | 225579 | Slc27a6 | "solute carrier family 27 (fatty acid transporter), member 6" |
| Tgfbi | 21810 | Tgfbi | "transforming growth factor, beta induced" |
| Ccl9 | 20308 | Ccl9 | chemokine (C-C motif) ligand 9 |
| Ifi211 | 381308 | Ifi211 | interferon activated gene 211 |
| Lpl | 16956 | Lpl | lipoprotein lipase |
| Cyp4f18 | 72054 | Cyp4f18 | "cytochrome P450, family 4, subfamily f, polypeptide 18" |
| Bhlhe40 | 20893 | Bhlhe40 | "basic helix-loop-helix family, member e40" |
| Gm21188 | 100861753 | Gm21188 | "predicted gene, 21188" |
| Gm15922 | NA | NA | NA |
| Ighv1-82 | 100775175 | Ighv1-82 | immunoglobulin heavy variable 1-82 |
| Cfp | 18636 | Cfp | complement factor properdin |
| Siglece | 83382 | Siglece | sialic acid binding Ig-like lectin E |
| Tmem154 | 320782 | Tmem154 | transmembrane protein 154 |
| Gpr65 | 14744 | Gpr65 | G-protein coupled receptor 65 |

| | | | |
|---|---|---|---|
| Igkv6-20 | 108024 | Igkv6-20 | immunoglobulin kappa variable 6-20 |
| Evi2b | 216984 | Evi2b | ecotropic viral integration site 2b |
| H2-Q7 | 15018 | H2-Q7 | "histocompatibility 2, Q region locus 7" |
| Was | 22376 | Was | Wiskott-Aldrich syndrome |
| Rgs10 | 67865 | Rgs10 | regulator of G-protein signalling 10 |
| Abca1 | 11303 | Abca1 | "ATP-binding cassette, sub-family A (ABC1), member 1" |
| 1700017B05Rik | 74211 | 1700017B05Rik | RIKEN cDNA 1700017B05 gene |
| Tigit | 100043314 | Tigit | T cell immunoreceptor with Ig and ITIM domains |
| Vim | 22352 | Vim | vimentin |
| Htr2b | 15559 | Htr2b | 5-hydroxytryptamine (serotonin) receptor 2B |
| Ncapg2 | 76044 | Ncapg2 | "non-SMC condensin II complex, subunit G2" |
| Wfdc21 | 66107 | Wfdc21 | WAP four-disulfide core domain 21 |
| Aoah | 27052 | Aoah | acyloxyacyl hydrolase |
| Fam111a | 107373 | Fam111a | "family with sequence similarity 111, member A" |
| Map4k1 | 26411 | Map4k1 | mitogen-activated protein kinase kinase kinase kinase 1 |
| Pilra | 231805 | Pilra | paired immunoglobin-like type 2 receptor alpha |
| Fabp5 | 16592 | Fabp5 | "fatty acid binding protein 5, epidermal" |
| C1qa | 12259 | C1qa | "complement component 1, q subcomponent, alpha polypeptide" |
| Fyb | 23880 | Fyb | FYN binding protein |
| Cd86 | 12524 | Cd86 | CD86 antigen |
| Gpr162 | 14788 | Gpr162 | G protein-coupled receptor 162 |
| Prg2 | 19074 | Prg2 | "proteoglycan 2, bone marrow" |
| Tmem51 | 214359 | Tmem51 | transmembrane protein 51 |
| Ighv1-5 | 668469 | Ighv1-5 | immunoglobulin heavy variable V1-5 |
| Tnfsf13 | 69583 | Tnfsf13 | "tumor necrosis factor (ligand) superfamily, member 13" |

| | | | |
|---|---|---|---|
| Chil1 | 12654 | Chil1 | chitinase-like 1 |
| Ccl6 | 20305 | Ccl6 | chemokine (C-C motif) ligand 6 |
| Xcr1 | 23832 | Xcr1 | chemokine (C motif) receptor 1 |
| Cbln1 | 12404 | Cbln1 | cerebellin 1 precursor protein |
| Ciita | 12265 | Ciita | class II transactivator |
| Gpr176 | 381413 | Gpr176 | G protein-coupled receptor 176 |
| Cd1d1 | 12479 | Cd1d1 | CD1d1 antigen |
| Acox3 | 80911 | Acox3 | "acyl-Coenzyme A oxidase 3, pristanoyl" |
| H2-M3 | 14991 | H2-M3 | "histocompatibility 2, M region locus 3" |
| Ighv2-5 | 638605 | Ighv2-5 | immunoglobulin heavy variable 2-5 |
| Cxcl3 | 330122 | Cxcl3 | chemokine (C-X-C motif) ligand 3 |
| Ighv1-81 | 668591 | Ighv1-81 | immunoglobulin heavy variable 1-81 |
| Blvra | 109778 | Blvra | biliverdin reductase A |
| Myo1g | 246177 | Myo1g | myosin IG |
| Cd37 | 12493 | Cd37 | CD37 antigen |
| Igkv8-30 | 384419 | Igkv8-30 | immunoglobulin kappa chain variable 8-30 |
| Itgae | 16407 | Itgae | "integrin alpha E, epithelial-associated" |
| Aldoc | 11676 | Aldoc | "aldolase C, fructose-bisphosphate" |
| Bcl2a1d | 12047 | Bcl2a1d | B cell leukemia/lymphoma 2 related protein A1d |
| Igkv19-93 | 692161 | Igkv19-93 | immunoglobulin kappa chain variable 19-93 |
| Gna15 | 14676 | Gna15 | "guanine nucleotide binding protein, alpha 15" |
| Ctse | 13034 | Ctse | cathepsin E |
| Ighv1-66 | 380824 | Ighv1-66 | immunoglobulin heavy variable 1-66 |
| Clec4a1 | 269799 | Clec4a1 | "C-type lectin domain family 4, member a1" |
| Prune2 | 353211 | Prune2 | prune homolog 2 |

| | | | |
|---|---|---|---|
| Vnn1 | 22361 | Vnn1 | vanin 1 |
| H2-DMa | 14998 | H2-DMa | "histocompatibility 2, class II, locus DMa" |
| Creld2 | 76737 | Creld2 | cysteine-rich with EGF-like domains 2 |
| Psmb8 | 16913 | Psmb8 | "proteasome (prosome, macropain) subunit, beta type 8 (large multifunctional peptidase 7)" |
| S100a4 | 20198 | S100a4 | S100 calcium binding protein A4 |
| Jchain | 16069 | Jchain | immunoglobulin joining chain |
| Sdf2l1 | 64136 | Sdf2l1 | stromal cell-derived factor 2-like 1 |
| Irak3 | 73914 | Irak3 | interleukin-1 receptor-associated kinase 3 |
| Bub1b | 12236 | Bub1b | "BUB1B, mitotic checkpoint serine/threonine kinase" |
| Cpxm1 | 56264 | Cpxm1 | carboxypeptidase X 1 (M14 family) |
| Ighv1-80 | 668589 | Ighv1-80 | immunoglobulin heavy variable 1-80 |
| Lilra5 | 232801 | Lilra5 | "leukocyte immunoglobulin-like receptor, subfamily A (with TM domain), member 5" |
| Pdcd1 | 18566 | Pdcd1 | programmed cell death 1 |
| Tnfaip8 | 106869 | Tnfaip8 | "tumor necrosis factor, alpha-induced protein 8" |
| Nabp1 | 109019 | Nabp1 | nucleic acid binding protein 1 |
| Ighv1-9 | 668478 | Ighv1-9 | immunoglobulin heavy variable V1-9 |
| Cd4 | 12504 | Cd4 | CD4 antigen |
| Gatm | 67092 | Gatm | glycine amidinotransferase (L-arginine:glycine amidinotransferase) |
| AW112010 | 107350 | AW112010 | expressed sequence AW112010 |
| A930007I19Rik | 77779 | A930007I19Rik | RIKEN cDNA A930007I19 gene |
| Ly6i | 57248 | Ly6i | "lymphocyte antigen 6 complex, locus I" |
| Lat2 | 56743 | Lat2 | "linker for activation of T cells family, member 2" |
| Mmp19 | 58223 | Mmp19 | matrix metallopeptidase 19 |
| Ccr2 | 12772 | Ccr2 | chemokine (C-C motif) receptor 2 |

| | | | |
|---|---|---|---|
| Myo7a | 17921 | Myo7a | myosin VIIA |
| Calhm6 | 215900 | Calhm6 | calcium homeostasis modulator family member 6 |
| Klra2 | 16633 | Klra2 | "killer cell lectin-like receptor, subfamily A, member 2" |
| Zfp991 | 666532 | Zfp991 | zinc finger protein 991 |
| Ptger2 | 19217 | Ptger2 | prostaglandin E receptor 2 (subtype EP2) |
| Nxpe5 | 381680 | Nxpe5 | "neurexophilin and PC-esterase domain family, member 5" |
| Cd300ld3 | 382551 | Cd300ld3 | CD300 molecule like family member D3 |
| D330045A20Rik | NA | NA | NA |
| Igkv13-64 | 628564 | Igkv13-64 | immunoglobulin kappa chain variable 13-64 |
| Tlr6 | 21899 | Tlr6 | toll-like receptor 6 |
| Naaa | 67111 | Naaa | N-acylethanolamine acid amidase |
| Cxcr3 | 12766 | Cxcr3 | chemokine (C-X-C motif) receptor 3 |
| Ear2 | 13587 | Ear2 | "eosinophil-associated, ribonuclease A family, member 2" |
| Gm8995 | 668139 | Gm8995 | predicted gene 8995 |
| Igkv3-11 | 667917 | Igkv3-11 | immunoglobulin kappa variable 3-11 |
| Prkcb | 18751 | Prkcb | "protein kinase C, beta" |
| Igkv6-13 | 667899 | Igkv6-13 | immunoglobulin kappa variable 6-13 |
| Gm11427 | 435271 | Gm11427 | predicted gene 11427 |
| Mmp13 | 17386 | Mmp13 | matrix metallopeptidase 13 |
| Rims3 | 242662 | Rims3 | regulating synaptic membrane exocytosis 3 |
| Card9 | 332579 | Card9 | "caspase recruitment domain family, member 9" |
| Adcy3 | 104111 | Adcy3 | adenylate cyclase 3 |
| Slc39a2 | 214922 | Slc39a2 | "solute carrier family 39 (zinc transporter), member 2" |
| Ighv3-6 | 780829 | Ighv3-6 | immunoglobulin heavy variable 3-6 |
| Milr1 | 380732 | Milr1 | mast cell immunoglobulin like receptor 1 |

| | | | |
|---|---|---|---|
| Cnr2 | 12802 | Cnr2 | cannabinoid receptor 2 (macrophage) |
| Cd3e | 12501 | Cd3e | "CD3 antigen, epsilon polypeptide" |
| Pkib | 18768 | Pkib | "protein kinase inhibitor beta, cAMP dependent, testis specific" |
| B3gnt7 | 227327 | B3gnt7 | "UDP-GlcNAc:betaGal beta-1,3-N-acetylglucosaminyltransferase 7" |
| Sec11c | 66286 | Sec11c | "SEC11 homolog C, signal peptidase complex subunit" |
| Il21r | 60504 | Il21r | interleukin 21 receptor |
| Atp8b4 | 241633 | Atp8b4 | "ATPase, class I, type 8B, member 4" |
| Apoc1 | 11812 | Apoc1 | apolipoprotein C-I |
| Igkv16-104 | 381778 | Igkv16-104 | immunoglobulin kappa variable 16-104 |
| Naip6 | 17952 | Naip6 | "NLR family, apoptosis inhibitory protein 6" |
| Ikbke | 56489 | Ikbke | inhibitor of kappaB kinase epsilon |
| Nuggc | 100503545 | Nuggc | "nuclear GTPase, germinal center associated" |
| Ccr10 | 12777 | Ccr10 | chemokine (C-C motif) receptor 10 |
| Lilrb4a | 14728 | Lilrb4a | "leukocyte immunoglobulin-like receptor, subfamily B, member 4A" |
| Tns4 | 217169 | Tns4 | tensin 4 |
| Pou2af1 | 18985 | Pou2af1 | "POU domain, class 2, associating factor 1" |
| Ighv1-63 | 780956 | Ighv1-63 | immunoglobulin heavy variable V1-63 |
| Rap2b | 74012 | Rap2b | "RAP2B, member of RAS oncogene family" |
| Hc | 15139 | Hc | hemolytic complement |
| Csmd1 | 94109 | Csmd1 | CUB and Sushi multiple domains 1 |
| Ighv1-76 | 100775174 | Ighv1-76 | immunoglobulin heavy variable 1-76 |
| H2-Q6 | 110557 | H2-Q6 | "histocompatibility 2, Q region locus 6" |
| Ahnak2 | 100041194 | Ahnak2 | AHNAK nucleoprotein 2 |
| Ighv2-9-1 | 791089 | Ighv2-9-1 | immunoglobulin heavy variable 2-9-1 |
| Znf41-ps | 70005 | Znf41-ps | "ZNF41, pseudogene" |

| | | | |
|---|---|---|---|
| Mrgpre | 244238 | Mrgpre | "MAS-related GPR, member E" |
| Siglecf | 233186 | Siglecf | sialic acid binding Ig-like lectin F |
| Dbp | 13170 | Dbp | D site albumin promoter binding protein |
| AC157784.1 | NA | NA | NA |
| Ccl5 | 20304 | Ccl5 | chemokine (C-C motif) ligand 5 |
| Hdac9 | 79221 | Hdac9 | histone deacetylase 9 |
| Flt3 | 14255 | Flt3 | FMS-like tyrosine kinase 3 |
| Ighv1-55 | 780932 | Ighv1-55 | immunoglobulin heavy variable 1-55 |
| Igkv11-125 | 243428 | Igkv11-125 | immunoglobulin kappa variable 11-125 |
| Igkv6-25 | 381784 | Igkv6-25 | immunoglobulin kappa chain variable 6-25 |
| Ctsh | 13036 | Ctsh | cathepsin H |
| Gas7 | 14457 | Gas7 | growth arrest specific 7 |
| Fgd3 | 30938 | Fgd3 | "FYVE, RhoGEF and PH domain containing 3" |
| Ighv1-78 | 213570 | Ighv1-78 | immunoglobulin heavy variable 1-78 |
| Pilrb2 | 545812 | Pilrb2 | paired immunoglobin-like type 2 receptor beta 2 |
| Tlr9 | 81897 | Tlr9 | toll-like receptor 9 |
| Best1 | 24115 | Best1 | bestrophin 1 |
| Iglv1 | 16142 | Iglv1 | immunoglobulin lambda variable 1 |
| Oas3 | 246727 | Oas3 | 2'-5' oligoadenylate synthetase 3 |
| Id2 | 15902 | Id2 | inhibitor of DNA binding 2 |
| Cxcl9 | 17329 | Cxcl9 | chemokine (C-X-C motif) ligand 9 |
| Il18bp | 16068 | Il18bp | interleukin 18 binding protein |
| Nos2 | 18126 | Nos2 | "nitric oxide synthase 2, inducible" |
| Nlrp3 | 216799 | Nlrp3 | "NLR family, pyrin domain containing 3" |
| Lacc1 | 210808 | Lacc1 | laccase domain containing 1 |

| | | | |
|---|---|---|---|
| Gda | 14544 | Gda | guanine deaminase |
| Gm4841 | 225594 | Gm4841 | predicted gene 4841 |
| Npc2 | 67963 | Npc2 | NPC intracellular cholesterol transporter 2 |
| Scamp5 | 56807 | Scamp5 | secretory carrier membrane protein 5 |
| Cd3g | 12502 | Cd3g | "CD3 antigen, gamma polypeptide" |
| Mcub | 66815 | Mcub | mitochondrial calcium uniporter dominant negative beta subunit |
| Psrc1 | 56742 | Psrc1 | proline/serine-rich coiled-coil 1 |
| Mfsd7a | 243197 | Mfsd7a | major facilitator superfamily domain containing 7A |
| AB124611 | 382062 | AB124611 | cDNA sequence AB124611 |
| Kcna3 | 16491 | Kcna3 | "potassium voltage-gated channel, shaker-related subfamily, member 3" |
| B430306N03Rik | 320148 | B430306N03Rik | RIKEN cDNA B430306N03 gene |
| Il7r | 16197 | Il7r | interleukin 7 receptor |
| Zfp992 | 433791 | Zfp992 | zinc finger protein 992 |
| Igkv5-43 | 381783 | Igkv5-43 | immunoglobulin kappa chain variable 5-43 |
| Ighv1-4 | 432702 | Ighv1-4 | immunoglobulin heavy variable 1-4 |
| Fgd2 | 26382 | Fgd2 | "FYVE, RhoGEF and PH domain containing 2" |
| Ksr2 | 333050 | Ksr2 | kinase suppressor of ras 2 |
| Runx2 | 12393 | Runx2 | runt related transcription factor 2 |
| Tspan4 | 64540 | Tspan4 | tetraspanin 4 |
| Arid3a | 13496 | Arid3a | AT rich interactive domain 3A (BRIGHT-like) |
| Igkv3-4 | 626347 | Igkv3-4 | immunoglobulin kappa variable 3-4 |
| Slamf6 | 30925 | Slamf6 | SLAM family member 6 |
| Per3 | 18628 | Per3 | period circadian clock 3 |
| Ighg2c | 404711 | Ighg2c | immunoglobulin heavy constant gamma 2C |
| Tnfrsf13b | 57916 | Tnfrsf13b | "tumor necrosis factor receptor superfamily, member 13b" |

| | | | |
|---|---|---|---|
| Ighv1-53 | 780931 | Ighv1-53 | immunoglobulin heavy variable 1-53 |
| Mzb1 | 69816 | Mzb1 | marginal zone B and B1 cell-specific protein 1 |
| Ighv1-75 | 622728 | Ighv1-75 | immunoglobulin heavy variable 1-75 |
| B4galt6 | 56386 | B4galt6 | "UDP-Gal:betaGlcNAc beta 1,4-galactosyltransferase, polypeptide 6" |
| Fabp7 | 12140 | Fabp7 | "fatty acid binding protein 7, brain" |
| Tap1 | 21354 | Tap1 | "transporter 1, ATP-binding cassette, sub-family B (MDR/TAP)" |
| Ighv1-58 | 780939 | Ighv1-58 | immunoglobulin heavy variable 1-58 |
| Igkv10-94 | 667550 | Igkv10-94 | immunoglobulin kappa variable 10-94 |
| Ms4a14 | 383435 | Ms4a14 | "membrane-spanning 4-domains, subfamily A, member 14" |
| Gm13212 | 433801 | Gm13212 | predicted gene 13212 |
| C5ar1 | 12273 | C5ar1 | complement component 5a receptor 1 |
| Btnl4 | 632126 | Btnl4 | butyrophilin-like 4 |
| Zbp1 | 58203 | Zbp1 | Z-DNA binding protein 1 |
| Nfkbie | 18037 | Nfkbie | "nuclear factor of kappa light polypeptide gene enhancer in B cells inhibitor, epsilon" |
| Arhgap11a | 228482 | Arhgap11a | Rho GTPase activating protein 11A |
| Pou2f2 | 18987 | Pou2f2 | "POU domain, class 2, transcription factor 2" |
| Pstpip2 | 19201 | Pstpip2 | proline-serine-threonine phosphatase-interacting protein 2 |
| Iglc2 | 110786 | Iglc2 | immunoglobulin lambda constant 2 |
| Plbd1 | 66857 | Plbd1 | phospholipase B domain containing 1 |
| Ighv1-54 | 211331 | Ighv1-54 | immunoglobulin heavy variable V1-54 |
| Gm6209 | 621304 | Gm6209 | predicted gene 6209 |
| Rasal3 | 320484 | Rasal3 | RAS protein activator like 3 |
| Sh3bp1 | 20401 | Sh3bp1 | SH3-domain binding protein 1 |
| Insl6 | 27356 | Insl6 | insulin-like 6 |

| | | | |
|---|---|---|---|
| Igkv1-99 | 434028 | Igkv1-99 | immunoglobulin kappa variable 1-99 |
| Ccl8 | 20307 | Ccl8 | chemokine (C-C motif) ligand 8 |
| Iglv3 | 404743 | Iglv3 | immunoglobulin lambda variable 3 |
| Slc40a1 | 53945 | Slc40a1 | "solute carrier family 40 (iron-regulated transporter), member 1" |
| Kif11 | 16551 | Kif11 | kinesin family member 11 |
| Igkv3-12 | 667914 | Igkv3-12 | immunoglobulin kappa variable 3-12 |
| Ighv1-18 | 629871 | Ighv1-18 | immunoglobulin heavy variable V1-18 |
| Egr2 | 13654 | Egr2 | early growth response 2 |
| Ntpcr | 66566 | Ntpcr | "nucleoside-triphosphatase, cancer-related" |
| Apol7c | 108956 | Apol7c | apolipoprotein L 7c |
| Ptger4 | 19219 | Ptger4 | prostaglandin E receptor 4 (subtype EP4) |
| Psmb9 | 16912 | Psmb9 | "proteasome (prosome, macropain) subunit, beta type 9 (large multifunctional peptidase 2)" |
| Gm36161 | 102639975 | Gm36161 | "predicted gene, 36161" |
| Tm4sf19 | 277203 | Tm4sf19 | transmembrane 4 L six family member 19 |
| Tnfrsf11a | 21934 | Tnfrsf11a | "tumor necrosis factor receptor superfamily, member 11a, NFKB activator" |
| Igkv2-109 | 628268 | Igkv2-109 | immunoglobulin kappa variable 2-109 |
| Elovl1 | 54325 | Elovl1 | "elongation of very long chain fatty acids (FEN1/Elo2, SUR4/Elo3, yeast)-like 1" |
| Ipcef1 | 320495 | Ipcef1 | interaction protein for cytohesin exchange factors 1 |
| Slfn9 | 237886 | Slfn9 | schlafen 9 |
| Gpr160 | 71862 | Gpr160 | G protein-coupled receptor 160 |
| Fgl2 | 14190 | Fgl2 | fibrinogen-like protein 2 |
| Ubash3b | 72828 | Ubash3b | "ubiquitin associated and SH3 domain containing, B" |
| Dhcr7 | 13360 | Dhcr7 | 7-dehydrocholesterol reductase |
| Espl1 | 105988 | Espl1 | "extra spindle pole bodies 1, separase" |

| | | | |
|---|---|---|---|
| Igkv8-24 | 677858 | Igkv8-24 | immunoglobulin kappa chain variable 8-24 |
| Sh2b2 | 23921 | Sh2b2 | SH2B adaptor protein 2 |
| Clu | 12759 | Clu | clusterin |
| Ighv1-69 | 619833 | Ighv1-69 | immunoglobulin heavy variable 1-69 |
| Oas1a | 246730 | Oas1a | 2'-5' oligoadenylate synthetase 1A |
| Ighv1-26 | 629884 | Ighv1-26 | immunoglobulin heavy variable 1-26 |
| Rab3il1 | 74760 | Rab3il1 | RAB3A interacting protein (rabin3)-like 1 |
| Tnfrsf17 | 21935 | Tnfrsf17 | "tumor necrosis factor receptor superfamily, member 17" |
| Cd72 | 12517 | Cd72 | CD72 antigen |
| Lag3 | 16768 | Lag3 | lymphocyte-activation gene 3 |
| Muc1 | 17829 | Muc1 | "mucin 1, transmembrane" |
| F830016B08Rik | 240328 | F830016B08Rik | RIKEN cDNA F830016B08 gene |
| Kcnn3 | 140493 | Kcnn3 | "potassium intermediate/small conductance calcium-activated channel, subfamily N, member 3" |
| Epsti1 | 108670 | Epsti1 | epithelial stromal interaction 1 (breast) |
| Scn1b | 20266 | Scn1b | "sodium channel, voltage-gated, type I, beta" |
| Batf3 | 381319 | Batf3 | "basic leucine zipper transcription factor, ATF-like 3" |
| Kif2c | 73804 | Kif2c | kinesin family member 2C |
| Mfap5 | 50530 | Mfap5 | microfibrillar associated protein 5 |
| Jaml | 270152 | Jaml | junction adhesion molecule like |
| Mmp8 | 17394 | Mmp8 | matrix metallopeptidase 8 |
| Irf4 | 16364 | Irf4 | interferon regulatory factor 4 |
| Gpr55 | 227326 | Gpr55 | G protein-coupled receptor 55 |
| Rasgrp1 | 19419 | Rasgrp1 | RAS guanyl releasing protein 1 |
| Knl1 | 76464 | Knl1 | kinetochore scaffold 1 |

| | | | |
|---|---|---|---|
| Ighv2-2 | 777686 | Ighv2-2 | immunoglobulin heavy variable 2-2 |
| Mdk | 17242 | Mdk | midkine |
| Gbp8 | 76074 | Gbp8 | guanylate-binding protein 8 |
| Lax1 | 240754 | Lax1 | lymphocyte transmembrane adaptor 1 |
| Ighv5-16 | 633568 | Ighv5-16 | immunoglobulin heavy variable 5-16 |
| Serpina3g | 20715 | Serpina3g | "serine (or cysteine) peptidase inhibitor, clade A, member 3G" |
| Stat1 | 20846 | Stat1 | signal transducer and activator of transcription 1 |
| C130026I21Rik | 620078 | C130026I21Rik | RIKEN cDNA C130026I21 gene |
| H2-Eb2 | 381091 | H2-Eb2 | "histocompatibility 2, class II antigen E beta2" |
| Crlf2 | 57914 | Crlf2 | cytokine receptor-like factor 2 |
| Ltc4s | 17001 | Ltc4s | leukotriene C4 synthase |
| Nnat | 18111 | Nnat | neuronatin |
| Arsg | 74008 | Arsg | arylsulfatase G |
| Gm1966 | 434223 | Gm1966 | predicted gene 1966 |
| Gm15601 | NA | NA | NA |
| P2ry10b | 213438 | P2ry10b | "purinergic receptor P2Y, G-protein coupled 10B" |
| Igkv4-72 | 385109 | Igkv4-72 | immunoglobulin kappa chain variable 4-72 |
| Il4i1 | 14204 | Il4i1 | interleukin 4 induced 1 |
| Slc2a6 | 227659 | Slc2a6 | "solute carrier family 2 (facilitated glucose transporter), member 6" |
| Igkv15-103 | 692169 | Igkv15-103 | immunoglobulin kappa chain variable 15-103 |
| Atp10a | 11982 | Atp10a | "ATPase, class V, type 10A" |
| Eaf2 | 106389 | Eaf2 | ELL associated factor 2 |
| Cd300ld | 217305 | Cd300ld | CD300 molecule like family member d |
| Cd5 | 12507 | Cd5 | CD5 antigen |
| Cenpf | 108000 | Cenpf | centromere protein F |

| | | | |
|---|---|---|---|
| Gm10521 | 100038548 | Gm10521 | predicted gene 10521 |
| Ear6 | 93719 | Ear6 | "eosinophil-associated, ribonuclease A family, member 6" |
| Megf11 | 214058 | Megf11 | multiple EGF-like-domains 11 |
| Gm30211 | 102632036 | Gm30211 | "predicted gene, 30211" |
| Runx3 | 12399 | Runx3 | runt related transcription factor 3 |
| Fcho1 | 74015 | Fcho1 | FCH domain only 1 |
| Nlrc5 | 434341 | Nlrc5 | "NLR family, CARD domain containing 5" |
| Slc9a7 | 236727 | Slc9a7 | "solute carrier family 9 (sodium/hydrogen exchanger), member 7" |
| Trpc4 | 22066 | Trpc4 | "transient receptor potential cation channel, subfamily C, member 4" |
| Tlr11 | 239081 | Tlr11 | toll-like receptor 11 |
| Sfrp1 | 20377 | Sfrp1 | secreted frizzled-related protein 1 |
| Fscn1 | 14086 | Fscn1 | fascin actin-bundling protein 1 |
| Cxcl10 | 15945 | Cxcl10 | chemokine (C-X-C motif) ligand 10 |
| Cideb | 12684 | Cideb | "cell death-inducing DNA fragmentation factor, alpha subunit-like effector B" |
| Gpsm3 | 106512 | Gpsm3 | "G-protein signalling modulator 3 (AGS3-like, C. elegans)" |
| P4ha3 | 320452 | P4ha3 | "procollagen-proline, 2-oxoglutarate 4-dioxygenase (proline 4-hydroxylase), alpha polypeptide III" |
| C920009B18Rik | 606736 | C920009B18Rik | RIKEN cDNA C920009B18 gene |
| Ighv2-3 | 238412 | Ighv2-3 | immunoglobulin heavy variable 2-3 |
| Fcrls | 80891 | Fcrls | "Fc receptor-like S, scavenger receptor" |
| Tlr12 | 384059 | Tlr12 | toll-like receptor 12 |
| Zc3h12d | 237256 | Zc3h12d | zinc finger CCCH type containing 12D |
| Nags | 217214 | Nags | N-acetylglutamate synthase |
| Igkv12-44 | 545851 | Igkv12-44 | immunoglobulin kappa variable 12-44 |
| Igkv17-121 | 667435 | Igkv17-121 | immunoglobulin kappa variable 17-121 |

| | | | |
|---|---|---|---|
| Pdgfc | 54635 | Pdgfc | "platelet-derived growth factor, C polypeptide" |
| Arnt2 | 11864 | Arnt2 | aryl hydrocarbon receptor nuclear translocator 2 |
| Pkdcc | 106522 | Pkdcc | "protein kinase domain containing, cytoplasmic" |
| Ighv1-64 | 380823 | Ighv1-64 | immunoglobulin heavy variable 1-64 |
| Dok3 | 27261 | Dok3 | docking protein 3 |
| Tcrg-C2 | NA | NA | NA |
| Cd14 | 12475 | Cd14 | CD14 antigen |
| AC166110.3 | NA | NA | NA |
| Cdc20 | 107995 | Cdc20 | cell division cycle 20 |
| Btnl6 | 624681 | Btnl6 | butyrophilin-like 6 |
| Adora2b | 11541 | Adora2b | adenosine A2b receptor |
| Dna2 | 327762 | Dna2 | DNA replication helicase/nuclease 2 |
| Armc7 | 276905 | Armc7 | armadillo repeat containing 7 |
| Mki67 | 17345 | Mki67 | antigen identified by monoclonal antibody Ki 67 |
| Igkv1-117 | 16098 | Igkv1-117 | immunoglobulin kappa variable 1-117 |
| Sh2d1b1 | 26904 | Sh2d1b1 | SH2 domain containing 1B1 |
| H2-Q5 | 15016 | H2-Q5 | "histocompatibility 2, Q region locus 5" |
| Nlrp1b | 637515 | Nlrp1b | "NLR family, pyrin domain containing 1B" |
| Ncapg | 54392 | Ncapg | "non-SMC condensin I complex, subunit G" |
| Bend6 | 320705 | Bend6 | BEN domain containing 6 |
| Gpr157 | 269604 | Gpr157 | G protein-coupled receptor 157 |
| Cd6 | 12511 | Cd6 | CD6 antigen |
| Amdhd2 | 245847 | Amdhd2 | amidohydrolase domain containing 2 |
| Adora3 | 11542 | Adora3 | adenosine A3 receptor |
| Emilin2 | 246707 | Emilin2 | elastin microfibril interfacer 2 |

| | | | |
|---|---|---|---|
| Car13 | 71934 | Car13 | carbonic anhydrase 13 |
| Cep55 | 74107 | Cep55 | centrosomal protein 55 |
| Ccnb1 | 268697 | Ccnb1 | cyclin B1 |
| Inhba | 16323 | Inhba | inhibin beta-A |
| Kif20a | 19348 | Kif20a | kinesin family member 20A |
| Igkv4-74 | 236047 | Igkv4-74 | immunoglobulin kappa variable 4-74 |
| Trac | 100101484 | Trac | T cell receptor alpha constant |
| Pparg | 19016 | Pparg | peroxisome proliferator activated receptor gamma |
| Ighv1-22 | 780883 | Ighv1-22 | immunoglobulin heavy variable 1-22 |
| Ncapd2 | 68298 | Ncapd2 | "non-SMC condensin I complex, subunit D2" |
| Igkv6-23 | 637227 | Igkv6-23 | immunoglobulin kappa variable 6-23 |
| Slc5a1 | 20537 | Slc5a1 | "solute carrier family 5 (sodium/glucose cotransporter), member 1" |
| E2f8 | 108961 | E2f8 | E2F transcription factor 8 |
| Cd80 | 12519 | Cd80 | CD80 antigen |
| Ighv1-59 | 432708 | Ighv1-59 | immunoglobulin heavy variable V1-59 |
| Igkv1-135 | 243420 | Igkv1-135 | immunoglobulin kappa variable 1-135 |
| Ulbp1 | 77777 | Ulbp1 | UL16 binding protein 1 |
| Cxcr6 | 80901 | Cxcr6 | chemokine (C-X-C motif) receptor 6 |
| Ankrd34b | 218440 | Ankrd34b | ankyrin repeat domain 34B |
| 5830415G21Rik | 76009 | 5830415G21Rik | RIKEN cDNA 5830415G21 gene |
| Ikzf2 | 22779 | Ikzf2 | IKAROS family zinc finger 2 |
| Igkv9-120 | 434025 | Igkv9-120 | immunoglobulin kappa chain variable 9-120 |
| Sulf2 | 72043 | Sulf2 | sulfatase 2 |
| Lipn | 70166 | Lipn | "lipase, family member N" |
| Ccna2 | 12428 | Ccna2 | cyclin A2 |

| | | | |
|---|---|---|---|
| Nlrp1a | 195046 | Nlrp1a | "NLR family, pyrin domain containing 1A" |
| Gpr85 | 64450 | Gpr85 | G protein-coupled receptor 85 |
| Igkv3-10 | 667924 | Igkv3-10 | immunoglobulin kappa variable 3-10 |
| Gm13571 | 102636093 | Gm13571 | predicted gene 13571 |
| Ighv5-6 | 777780 | Ighv5-6 | immunoglobulin heavy variable 5-6 |
| Fpr3 | 14294 | Fpr3 | formyl peptide receptor 3 |
| Prr5 | 109270 | Prr5 | proline rich 5 (renal) |
| Rnase2a | 93726 | Rnase2a | "ribonuclease, RNase A family, 2A (liver, eosinophil-derived neurotoxin)" |
| St18 | 240690 | St18 | suppression of tumorigenicity 18 |
| Gm26573 | NA | NA | NA |
| Cd276 | 102657 | Cd276 | CD276 antigen |
| Hebp1 | 15199 | Hebp1 | heme binding protein 1 |
| Relt | 320100 | Relt | RELT tumor necrosis factor receptor |
| AI427809 | 381524 | AI427809 | expressed sequence AI427809 |
| Stap1 | 56792 | Stap1 | signal transducing adaptor family member 1 |
| Igkv4-68 | 384515 | Igkv4-68 | immunoglobulin kappa variable 4-68 |
| Slc16a6 | 104681 | Slc16a6 | "solute carrier family 16 (monocarboxylic acid transporters), member 6" |
| Ighv4-1 | 780802 | Ighv4-1 | immunoglobulin heavy variable 4-1 |
| Ighv6-3 | 629845 | Ighv6-3 | immunoglobulin heavy variable 6-3 |
| Slfn10-ps | 237887 | Slfn10-ps | "schlafen 10, pseudogene" |
| Pcdh7 | 54216 | Pcdh7 | protocadherin 7 |
| Fut7 | 14347 | Fut7 | fucosyltransferase 7 |
| Spink2 | 69982 | Spink2 | "serine peptidase inhibitor, Kazal type 2" |
| Apoc2 | 11813 | Apoc2 | apolipoprotein C-II |
| Fut4 | 14345 | Fut4 | fucosyltransferase 4 |

| | | | |
|---|---|---|---|
| Ighv14-3 | 238418 | Ighv14-3 | immunoglobulin heavy variable V14-3 |
| Ltb | 16994 | Ltb | lymphotoxin B |
| Igkv12-38 | 620050 | Igkv12-38 | immunoglobulin kappa chain variable 12-38 |
| Mab21l3 | 242125 | Mab21l3 | mab-21-like 3 |
| Prc1 | 233406 | Prc1 | protein regulator of cytokinesis 1 |
| Ighv5-4 | 777688 | Ighv5-4 | immunoglobulin heavy variable 5-4 |
| Gdf3 | 14562 | Gdf3 | growth differentiation factor 3 |
| Ighv10-1 | 380808 | Ighv10-1 | immunoglobulin heavy variable 10-1 |
| Ighv7-4 | 632654 | Ighv7-4 | immunoglobulin heavy variable 7-4 |
| Evi2 | 101488212 | Evi2 | ecotropic viral integration site 2 |
| Ighv6-6 | 238427 | Ighv6-6 | immunoglobulin heavy variable 6-6 |
| Stab2 | 192188 | Stab2 | stabilin 2 |
| F7 | 14068 | F7 | coagulation factor VII |
| B3gnt8 | 232984 | B3gnt8 | "UDP-GlcNAc:betaGal beta-1,3-N-acetylglucosaminyltransferase 8" |
| Ighv5-9-1 | 641178 | Ighv5-9-1 | immunoglobulin heavy variable 5-9-1 |
| Birc5 | 11799 | Birc5 | baculoviral IAP repeat-containing 5 |
| Pdcd1lg2 | 58205 | Pdcd1lg2 | programmed cell death 1 ligand 2 |
| Eno2 | 13807 | Eno2 | "enolase 2, gamma neuronal" |
| Igkv5-39 | 620017 | Igkv5-39 | immunoglobulin kappa variable 5-39 |
| Tpx2 | 72119 | Tpx2 | "TPX2, microtubule-associated" |
| Igkv4-57-1 | 384514 | Igkv4-57-1 | immunoglobulin kappa variable 4-57-1 |
| Usp35 | 244144 | Usp35 | ubiquitin specific peptidase 35 |
| Pclaf | 68026 | Pclaf | PCNA clamp associated factor |
| Ighv9-2 | 629818 | Ighv9-2 | immunoglobulin heavy variable V9-2 |
| Nlrc4 | 268973 | Nlrc4 | "NLR family, CARD domain containing 4" |

| | | | |
|---|---|---|---|
| Htr7 | 15566 | Htr7 | 5-hydroxytryptamine (serotonin) receptor 7 |
| Ube2c | 68612 | Ube2c | ubiquitin-conjugating enzyme E2C |
| Hcar2 | 80885 | Hcar2 | hydroxycarboxylic acid receptor 2 |
| Prnd | 26434 | Prnd | prion like protein doppel |
| Chek2 | 50883 | Chek2 | checkpoint kinase 2 |
| Cdk1 | 12534 | Cdk1 | cyclin-dependent kinase 1 |
| Pf4 | 56744 | Pf4 | platelet factor 4 |
| Plk1 | 18817 | Plk1 | polo like kinase 1 |
| Smim24 | 72273 | Smim24 | small integral membrane protein 24 |
| Ckap2l | 70466 | Ckap2l | cytoskeleton associated protein 2-like |
| 5830416I19Rik | 74757 | 5830416I19Rik | RIKEN cDNA 5830416I19 gene |
| Igkv1-133 | 628027 | Igkv1-133 | immunoglobulin kappa variable 1-133 |
| Ccnf | 12449 | Ccnf | cyclin F |
| Foxm1 | 14235 | Foxm1 | forkhead box M1 |
| Tgtp1 | 21822 | Tgtp1 | T cell specific GTPase 1 |
| Tnfsf8 | 21949 | Tnfsf8 | "tumor necrosis factor (ligand) superfamily, member 8" |
| Adam8 | 11501 | Adam8 | a disintegrin and metallopeptidase domain 8 |
| Gm44805 | 105886299 | Gm44805 | predicted gene 44805 |
| Gtse1 | 29870 | Gtse1 | G two S phase expressed protein 1 |
| Gpr34 | 23890 | Gpr34 | G protein-coupled receptor 34 |
| Gm37944 | NA | NA | NA |
| Igkv4-61 | 546244 | Igkv4-61 | immunoglobulin kappa chain variable 4-61 |
| Gcsam | 14525 | Gcsam | "germinal center associated, signaling and motility" |
| Ect2 | 13605 | Ect2 | ect2 oncogene |
| Ankrd55 | 77318 | Ankrd55 | ankyrin repeat domain 55 |

| | | | |
|---|---|---|---|
| Clip4 | 78785 | Clip4 | "CAP-GLY domain containing linker protein family, member 4" |
| Gdf6 | 242316 | Gdf6 | growth differentiation factor 6 |
| Slc26a4 | 23985 | Slc26a4 | "solute carrier family 26, member 4" |
| Igkv14-100 | 243439 | Igkv14-100 | immunoglobulin kappa chain variable 14-100 |
| Ighv1-7 | 668474 | Ighv1-7 | immunoglobulin heavy variable V1-7 |
| Marcksl1 | 17357 | Marcksl1 | MARCKS-like 1 |
| Gm47242 | NA | NA | NA |
| Hmmr | 15366 | Hmmr | hyaluronan mediated motility receptor (RHAMM) |
| Uhrf1 | 18140 | Uhrf1 | "ubiquitin-like, containing PHD and RING finger domains, 1" |
| Rhoh | 74734 | Rhoh | ras homolog family member H |
| Aspm | 12316 | Aspm | abnormal spindle microtubule assembly |
| Ighv1-71 | 619886 | Ighv1-71 | immunoglobulin heavy variable 1-71 |
| Susd3 | 66329 | Susd3 | sushi domain containing 3 |
| Ubd | 24108 | Ubd | ubiquitin D |
| Icos | 54167 | Icos | inducible T cell co-stimulator |
| Spdl1 | 70385 | Spdl1 | spindle apparatus coiled-coil protein 1 |
| P2ry13 | 74191 | P2ry13 | "purinergic receptor P2Y, G-protein coupled 13" |
| Gm9236 | 668552 | Gm9236 | predicted gene 9236 |
| 5430427O19Rik | 71398 | 5430427O19Rik | RIKEN cDNA 5430427O19 gene |
| Il12rb2 | 16162 | Il12rb2 | "interleukin 12 receptor, beta 2" |
| Il27ra | 50931 | Il27ra | "interleukin 27 receptor, alpha" |
| Cenpe | 229841 | Cenpe | centromere protein E |
| Tlr1 | 21897 | Tlr1 | toll-like receptor 1 |
| Kifc1 | 100502766 | Kifc1 | kinesin family member C1 |
| Tnfrsf8 | 21941 | Tnfrsf8 | "tumor necrosis factor receptor superfamily, member 8" |

| | | | |
|---|---|---|---|
| Igkv17-127 | 243433 | Igkv17-127 | immunoglobulin kappa variable 17-127 |
| Fam167b | 230766 | Fam167b | "family with sequence similarity 167, member B" |
| Acsbg1 | 94180 | Acsbg1 | acyl-CoA synthetase bubblegum family member 1 |
| Ighv8-5 | 640506 | Ighv8-5 | immunoglobulin heavy variable V8-5 |
| Igkv1-110 | 381777 | Igkv1-110 | immunoglobulin kappa variable 1-110 |
| Ftl1-ps1 | 634386 | Ftl1-ps1 | "ferritin light polypeptide 1, pseudogene 1" |
| Ndc80 | 67052 | Ndc80 | NDC80 kinetochore complex component |
| Top2a | 21973 | Top2a | topoisomerase (DNA) II alpha |
| Asf1b | 66929 | Asf1b | anti-silencing function 1B histone chaperone |
| Gm5547 | 433637 | Gm5547 | predicted gene 5547 |
| Gm5150 | 381484 | Gm5150 | predicted gene 5150 |
| Traf3ip3 | 215243 | Traf3ip3 | TRAF3 interacting protein 3 |
| Myo3b | 329421 | Myo3b | myosin IIIB |
| Gpr141 | 353346 | Gpr141 | G protein-coupled receptor 141 |
| Ighv3-1 | 780803 | Ighv3-1 | immunoglobulin heavy variable 3-1 |
| Igkv4-57 | 235952 | Igkv4-57 | immunoglobulin kappa variable 4-57 |
| Rgs16 | 19734 | Rgs16 | regulator of G-protein signaling 16 |
| Gm14548 | 100038909 | Gm14548 | predicted gene 14548 |
| Fpr1 | 14293 | Fpr1 | formyl peptide receptor 1 |
| Ighv1-12 | 629860 | Ighv1-12 | immunoglobulin heavy variable V1-12 |
| Ighv8-13 | 100775172 | Ighv8-13 | immunoglobulin heavy variable 8-13 |
| 1110038B12Rik | 68763 | 1110038B12Rik | RIKEN cDNA 1110038B12 gene |
| Ucp3 | 22229 | Ucp3 | "uncoupling protein 3 (mitochondrial, proton carrier)" |
| Iigp1 | 60440 | Iigp1 | interferon inducible GTPase 1 |
| Krt78 | 332131 | Krt78 | keratin 78 |

| | | | |
|---|---|---|---|
| Rrad | 56437 | Rrad | Ras-related associated with diabetes |
| Igkv6-15 | 108022 | Igkv6-15 | immunoglobulin kappa variable 6-15 |
| E230032D23Rik | 414103 | E230032D23Rik | RIKEN cDNA E230032D23 gene |
| Tnfrsf18 | 21936 | Tnfrsf18 | "tumor necrosis factor receptor superfamily, member 18" |
| Neb | 17996 | Neb | nebulin |
| Pimreg | 109212 | Pimreg | PICALM interacting mitotic regulator |
| E230016K23Rik | 100504464 | E230016K23Rik | RIKEN cDNA E230016K23 gene |
| B3galt5 | 93961 | B3galt5 | "UDP-Gal:betaGlcNAc beta 1,3-galactosyltransferase, polypeptide 5" |
| Slc13a3 | 114644 | Slc13a3 | "solute carrier family 13 (sodium-dependent dicarboxylate transporter), member 3" |
| Tspan10 | 208634 | Tspan10 | tetraspanin 10 |
| Fcrlb | 435653 | Fcrlb | Fc receptor-like B |
| Ccl12 | 20293 | Ccl12 | chemokine (C-C motif) ligand 12 |
| Mucl3 | 268949 | Mucl3 | mucin like 3 |
| Cdca8 | 52276 | Cdca8 | cell division cycle associated 8 |
| Gm38248 | NA | NA | NA |
| Tarm1 | 245126 | Tarm1 | "T cell-interacting, activating receptor on myeloid cells 1" |
| Tnip3 | 414084 | Tnip3 | TNFAIP3 interacting protein 3 |
| Cd200r3 | 74603 | Cd200r3 | CD200 receptor 3 |
| Igkv3-9 | 667928 | Igkv3-9 | immunoglobulin kappa variable 3-9 |
| Pbk | 52033 | Pbk | PDZ binding kinase |
| Gimap7 | 231932 | Gimap7 | "GTPase, IMAP family member 7" |
| Depdc1a | 76131 | Depdc1a | DEP domain containing 1a |
| Igkv13-84 | 692152 | Igkv13-84 | immunoglobulin kappa chain variable 13-84 |
| Cks1b | 54124 | Cks1b | CDC28 protein kinase 1b |
| Cxcr1 | 227288 | Cxcr1 | chemokine (C-X-C motif) receptor 1 |

| | | | |
|---|---|---|---|
| Mir146 | 387164 | Mir146 | microRNA 146 |
| Cgas | 214763 | Cgas | cyclic GMP-AMP synthase |
| Ighv1-19 | 382692 | Ighv1-19 | immunoglobulin heavy variable V1-19 |
| Cacna1e | 12290 | Cacna1e | "calcium channel, voltage-dependent, R type, alpha 1E subunit" |
| Ighv9-3 | 780825 | Ighv9-3 | immunoglobulin heavy variable V9-3 |
| Zfp993 | 100503000 | Zfp993 | zinc finger protein 993 |
| Nr1d2 | 353187 | Nr1d2 | "nuclear receptor subfamily 1, group D, member 2" |
| Col6a4 | 68553 | Col6a4 | "collagen, type VI, alpha 4" |
| Knstrn | 51944 | Knstrn | kinetochore-localized astrin/SPAG5 binding |
| Ighv5-12 | 668395 | Ighv5-12 | immunoglobulin heavy variable 5-12 |
| Gm20460 | NA | NA | NA |
| Nfatc2 | 18019 | Nfatc2 | "nuclear factor of activated T cells, cytoplasmic, calcineurin dependent 2" |
| Nlrc3 | 268857 | Nlrc3 | "NLR family, CARD domain containing 3" |
| Ighv10-3 | 380809 | Ighv10-3 | immunoglobulin heavy variable V10-3 |
| Gm19142 | 100418330 | Gm19142 | "predicted gene, 19142" |
| Gm26807 | NA | NA | NA |
| Pacsin1 | 23969 | Pacsin1 | protein kinase C and casein kinase substrate in neurons 1 |
| Ms4a4a | 666907 | Ms4a4a | "membrane-spanning 4-domains, subfamily A, member 4A" |
| Ttk | 22137 | Ttk | Ttk protein kinase |
| Wfdc12 | 192200 | Wfdc12 | WAP four-disulfide core domain 12 |
| Ces2c | 234671 | Ces2c | carboxylesterase 2C |
| Naip1 | 17940 | Naip1 | "NLR family, apoptosis inhibitory protein 1" |
| Gpr33 | 14762 | Gpr33 | G protein-coupled receptor 33 |
| Rph3a | 19894 | Rph3a | rabphilin 3A |
| Ccne1 | 12447 | Ccne1 | cyclin E1 |

| | | | |
|---|---|---|---|
| Igkv6-32 | 434039 | Igkv6-32 | immunoglobulin kappa variable 6-32 |
| Slc25a43 | 194744 | Slc25a43 | "solute carrier family 25, member 43" |
| Il1a | 16175 | Il1a | interleukin 1 alpha |
| Ly96 | 17087 | Ly96 | lymphocyte antigen 96 |
| Cd177 | 68891 | Cd177 | CD177 antigen |
| Slco2b1 | 101488 | Slco2b1 | "solute carrier organic anion transporter family, member 2b1" |
| Dlgap5 | 218977 | Dlgap5 | DLG associated protein 5 |
| Cacna1h | 58226 | Cacna1h | "calcium channel, voltage-dependent, T type, alpha 1H subunit" |
| Prok2 | 50501 | Prok2 | prokineticin 2 |
| Serpina10 | 217847 | Serpina10 | "serine (or cysteine) peptidase inhibitor, clade A (alpha-1 antiproteinase, antitrypsin), member 10" |
| A2m | 232345 | A2m | alpha-2-macroglobulin |
| Ms4a8a | 64381 | Ms4a8a | "membrane-spanning 4-domains, subfamily A, member 8A" |
| Il7 | 16196 | Il7 | interleukin 7 |
| Igkv10-96 | 692165 | Igkv10-96 | immunoglobulin kappa variable 10-96 |
| Trim59 | 66949 | Trim59 | tripartite motif-containing 59 |
| Slc16a5 | 217316 | Slc16a5 | "solute carrier family 16 (monocarboxylic acid transporters), member 5" |
| AC168977.1 | NA | NA | NA |
| Ighv1-84 | 434609 | Ighv1-84 | immunoglobulin heavy variable 1-84 |
| Ptx3 | 19288 | Ptx3 | pentraxin related gene |
| Spc25 | 66442 | Spc25 | "SPC25, NDC80 kinetochore complex component, homolog (S. cerevisiae)" |
| Igkv8-28 | 434040 | Igkv8-28 | immunoglobulin kappa variable 8-28 |
| Rundc3a | 51799 | Rundc3a | RUN domain containing 3A |
| AC125149.3 | NA | NA | NA |
| Adgrg5 | 382045 | Adgrg5 | adhesion G protein-coupled receptor G5 |

| | | | |
|---|---|---|---|
| Ankrd37 | 654824 | Ankrd37 | ankyrin repeat domain 37 |
| Gm12551 | 100040388 | Gm12551 | predicted gene 12551 |
| Gpr15 | 71223 | Gpr15 | G protein-coupled receptor 15 |
| Gm26917 | NA | NA | NA |
| Igkv3-5 | 667940 | Igkv3-5 | immunoglobulin kappa chain variable 3-5 |
| Ighv1-20 | 668497 | Ighv1-20 | immunoglobulin heavy variable V1-20 |
| Ighv6-4 | 628398 | Ighv6-4 | immunoglobulin heavy variable V6-4 |
| Rgs1 | 50778 | Rgs1 | regulator of G-protein signaling 1 |
| Gm37787 | NA | NA | NA |
| Iqgap3 | 404710 | Iqgap3 | IQ motif containing GTPase activating protein 3 |
| Cilp | 214425 | Cilp | "cartilage intermediate layer protein, nucleotide pyrophosphohydrolase" |
| Ighv15-2 | 780864 | Ighv15-2 | immunoglobulin heavy variable V15-2 |
| Gpr141b | 319293 | Gpr141b | G protein-coupled receptor 141B |
| Rnase2b | 54159 | Rnase2b | "ribonuclease, RNase A family, 2B (liver, eosinophil-derived neurotoxin)" |
| Rrm2 | 20135 | Rrm2 | ribonucleotide reductase M2 |
| Gm20056 | 100504089 | Gm20056 | "predicted gene, 20056" |
| Pycr1 | 209027 | Pycr1 | pyrroline-5-carboxylate reductase 1 |
| Gm49792 | NA | NA | NA |
| Cdca2 | 108912 | Cdca2 | cell division cycle associated 2 |
| Ptrh1 | 329384 | Ptrh1 | peptidyl-tRNA hydrolase 1 homolog |
| Fxyd4 | 108017 | Fxyd4 | FXYD domain-containing ion transport regulator 4 |
| Igkv14-130 | 628072 | Igkv14-130 | immunoglobulin kappa variable 14-130 |
| Igkv4-80 | 545848 | Igkv4-80 | immunoglobulin kappa variable 4-80 |
| Igkv9-124 | 243431 | Igkv9-124 | immunoglobulin kappa chain variable 9-124 |
| Igkv4-91 | 434033 | Igkv4-91 | immunoglobulin kappa chain variable 4-91 |

| | | | |
|---|---|---|---|
| Gbp5 | 229898 | Gbp5 | guanylate binding protein 5 |
| Gm45606 | NA | NA | NA |
| D7Ertd128e | 52222 | D7Ertd128e | "DNA segment, Chr 7, ERATO Doi 128, expressed" |
| Melk | 17279 | Melk | maternal embryonic leucine zipper kinase |
| 2210406H18Rik | 70133 | 2210406H18Rik | RIKEN cDNA 2210406H18 gene |
| Ccl20 | 20297 | Ccl20 | chemokine (C-C motif) ligand 20 |
| Lama1 | 16772 | Lama1 | "laminin, alpha 1" |
| Ighg2b | 16016 | Ighg2b | immunoglobulin heavy constant gamma 2B |
| Rasd2 | 75141 | Rasd2 | "RASD family, member 2" |
| Gm5063 | 271374 | Gm5063 | predicted gene 5063 |
| Gm49508 | NA | NA | NA |
| 5830411N06Rik | 244234 | 5830411N06Rik | RIKEN cDNA 5830411N06 gene |
| Cacna1s | 12292 | Cacna1s | "calcium channel, voltage-dependent, L type, alpha 1S subunit" |
| Dscam | 13508 | Dscam | DS cell adhesion molecule |
| Ighg1 | 16017 | Ighg1 | immunoglobulin heavy constant gamma 1 (G1m marker) |
| Prr11 | 270906 | Prr11 | proline rich 11 |
| Celsr3 | 107934 | Celsr3 | "cadherin, EGF LAG seven-pass G-type receptor 3" |
| Xcl1 | 16963 | Xcl1 | chemokine (C motif) ligand 1 |
| Pth1r | 19228 | Pth1r | parathyroid hormone 1 receptor |
| Socs1 | 12703 | Socs1 | suppressor of cytokine signaling 1 |
| Tk1 | 21877 | Tk1 | thymidine kinase 1 |
| Ccr6 | 12458 | Ccr6 | chemokine (C-C motif) receptor 6 |
| Gm47507 | NA | NA | NA |
| Igkv4-59 | 667683 | Igkv4-59 | immunoglobulin kappa variable 4-59 |
| Timp1 | 21857 | Timp1 | tissue inhibitor of metalloproteinase 1 |

| | | | |
|---|---|---|---|
| Cip2a | 224171 | Cip2a | cell proliferation regulating inhibitor of protein phosphatase 2A |
| Izumo1r | 64931 | Izumo1r | "IZUMO1 receptor, JUNO" |
| Tcrg-C1 | NA | NA | NA |
| Orm2 | 18406 | Orm2 | orosomucoid 2 |
| Kif18b | 70218 | Kif18b | kinesin family member 18B |
| Pvrig | 102640920 | Pvrig | poliovirus receptor related immunoglobulin domain containing |
| Tnfrsf4 | 22163 | Tnfrsf4 | "tumor necrosis factor receptor superfamily, member 4" |
| Ighv14-2 | 668421 | Ighv14-2 | immunoglobulin heavy variable 14-2 |
| Il9r | 16199 | Il9r | interleukin 9 receptor |
| Rubcnl | 271221 | Rubcnl | RUN and cysteine rich domain containing beclin 1 interacting protein like |
| Ticrr | 77011 | Ticrr | TOPBP1-interacting checkpoint and replication regulator |
| Ighv9-1 | 195180 | Ighv9-1 | immunoglobulin heavy variable 9-1 |
| Cks2 | 66197 | Cks2 | CDC28 protein kinase regulatory subunit 2 |
| Pdgfrl | 68797 | Pdgfrl | platelet-derived growth factor receptor-like |
| Igkv4-90 | 434034 | Igkv4-90 | immunoglobulin kappa chain variable 4-90 |
| C1qtnf3 | 81799 | C1qtnf3 | C1q and tumor necrosis factor related protein 3 |
| Cst7 | 13011 | Cst7 | cystatin F (leukocystatin) |
| BC023105 | 667597 | BC023105 | cDNA sequence BC023105 |
| Ccl3 | 20302 | Ccl3 | chemokine (C-C motif) ligand 3 |
| Nusap1 | 108907 | Nusap1 | nucleolar and spindle associated protein 1 |
| Haspin | 14841 | Haspin | histone H3 associated protein kinase |
| Adora1 | 11539 | Adora1 | adenosine A1 receptor |
| Mis18bp1 | 217653 | Mis18bp1 | MIS18 binding protein 1 |
| Iglc3 | 110787 | Iglc3 | immunoglobulin lambda constant 3 |
| Fkbp1b | 14226 | Fkbp1b | FK506 binding protein 1b |

| | | | |
|---|---|---|---|
| Igkv1-88 | 692154 | Igkv1-88 | immunoglobulin kappa chain variable 1-88 |
| Gm20045 | 100504072 | Gm20045 | "predicted gene, 20045" |
| Slc22a4 | 30805 | Slc22a4 | "solute carrier family 22 (organic cation transporter), member 4" |
| Mefv | 54483 | Mefv | Mediterranean fever |
| Sit1 | 54390 | Sit1 | suppression inducing transmembrane adaptor 1 |
| Mrc1 | 17533 | Mrc1 | "mannose receptor, C type 1" |
| Ighv1-61 | 674018 | Ighv1-61 | immunoglobulin heavy variable 1-61 |
| Ighv2-6 | 630486 | Ighv2-6 | immunoglobulin heavy variable 2-6 |
| Nuf2 | 66977 | Nuf2 | "NUF2, NDC80 kinetochore complex component" |
| Apol7e | 666348 | Apol7e | apolipoprotein L 7e |
| BE692007 | 100504727 | BE692007 | expressed sequence BE692007 |
| Ighe | 380792 | Ighe | Immunoglobulin heavy constant epsilon |
| Cdca3 | 14793 | Cdca3 | cell division cycle associated 3 |
| Cdkn2a | 12578 | Cdkn2a | cyclin dependent kinase inhibitor 2A |
| Tff1 | 21784 | Tff1 | trefoil factor 1 |
| Art2a-ps | NA | NA | NA |
| Nlrp1c-ps | 627984 | Nlrp1c-ps | "NLR family, pyrin domain containing 1C, pseudogene" |
| Ccdc80 | 67896 | Ccdc80 | coiled-coil domain containing 80 |
| Gm37795 | NA | NA | NA |
| Nrg4 | 83961 | Nrg4 | neuregulin 4 |
| Ighv1-39 | 780891 | Ighv1-39 | immunoglobulin heavy variable 1-39 |
| Dtl | 76843 | Dtl | denticleless E3 ubiquitin protein ligase |
| Ighv8-12 | 780960 | Ighv8-12 | immunoglobulin heavy variable V8-12 |
| Igkv3-2 | 626583 | Igkv3-2 | immunoglobulin kappa variable 3-2 |
| Mmp25 | 240047 | Mmp25 | matrix metallopeptidase 25 |

| | | | |
|---|---|---|---|
| A130077B15Rik | 319272 | A130077B15Rik | RIKEN cDNA A130077B15 gene |
| Ubash3a | 328795 | Ubash3a | "ubiquitin associated and SH3 domain containing, A" |
| AC119228.1 | NA | NA | NA |
| Cdh17 | 12557 | Cdh17 | cadherin 17 |
| Thbs4 | 21828 | Thbs4 | thrombospondin 4 |
| Il12rb1 | 16161 | Il12rb1 | "interleukin 12 receptor, beta 1" |
| Igkv2-137 | 692187 | Igkv2-137 | immunoglobulin kappa chain variable 2-137 |
| Ighv1-85 | 668597 | Ighv1-85 | immunoglobulin heavy variable 1-85 |
| Rad51 | 19361 | Rad51 | RAD51 recombinase |
| AC109138.8 | NA | NA | NA |
| Sh2d1b2 | 545378 | Sh2d1b2 | SH2 domain containing 1B2 |
| Cdca5 | 67849 | Cdca5 | cell division cycle associated 5 |
| Ube2t | 67196 | Ube2t | ubiquitin-conjugating enzyme E2T |
| Ifng | 15978 | Ifng | interferon gamma |
| Igkv8-27 | 434041 | Igkv8-27 | immunoglobulin kappa chain variable 8-27 |
| Lipf | 67717 | Lipf | "lipase, gastric" |
| Cdc6 | 23834 | Cdc6 | cell division cycle 6 |
| Gm43388 | NA | NA | NA |
| Aurkb | 20877 | Aurkb | aurora kinase B |
| Bhlhe22 | 59058 | Bhlhe22 | "basic helix-loop-helix family, member e22" |
| Tox | 252838 | Tox | thymocyte selection-associated high mobility group box |
| Gm47705 | NA | NA | NA |
| Crmp1 | 12933 | Crmp1 | collapsin response mediator protein 1 |
| Fkbp11 | 66120 | Fkbp11 | FK506 binding protein 11 |
| Gbp2 | 14469 | Gbp2 | guanylate binding protein 2 |

| | | | |
|---|---|---|---|
| Clec4b1 | 69810 | Clec4b1 | "C-type lectin domain family 4, member b1" |
| Igkv9-129 | 692182 | Igkv9-129 | immunoglobulin kappa variable 9-129 |
| Gbp10 | 626578 | Gbp10 | guanylate-binding protein 10 |
| Gm14461 | 329436 | Gm14461 | predicted gene 14461 |
| Gm2694 | 100040294 | Gm2694 | predicted gene 2694 |
| Ighv5-9 | 544896 | Ighv5-9 | immunoglobulin heavy variable 5-9 |
| Serpina3f | 238393 | Serpina3f | "serine (or cysteine) peptidase inhibitor, clade A, member 3F" |
| Brca1 | 12189 | Brca1 | "breast cancer 1, early onset" |
| F9 | 14071 | F9 | coagulation factor IX |
| Gm5086 | 328314 | Gm5086 | predicted gene 5086 |
| Spag5 | 54141 | Spag5 | sperm associated antigen 5 |
| Tspan33 | 232670 | Tspan33 | tetraspanin 33 |
| Gm44511 | NA | NA | NA |
| Trdc | 100123473 | Trdc | "T cell receptor delta, constant region" |
| Retnla | 57262 | Retnla | resistin like alpha |
| Sgo2a | 68549 | Sgo2a | shugoshin 2A |
| Gm40932 | 105245484 | Gm40932 | "predicted gene, 40932" |
| Ighv1-47 | 629915 | Ighv1-47 | immunoglobulin heavy variable 1-47 |
| Igkv5-48 | 619846 | Igkv5-48 | immunoglobulin kappa variable 5-48 |
| Ppm1n | 232941 | Ppm1n | "protein phosphatase, Mg2+/Mn2+ dependent, 1N (putative)" |
| Ccnb2 | 12442 | Ccnb2 | cyclin B2 |
| Il21 | 60505 | Il21 | interleukin 21 |
| Pla1a | 85031 | Pla1a | phospholipase A1 member A |
| Spic | 20728 | Spic | Spi-C transcription factor (Spi-1/PU.1 related) |
| Gm44861 | NA | NA | NA |

| | | | |
|---|---|---|---|
| B4galnt4 | 330671 | B4galnt4 | "beta-1,4-N-acetyl-galactosaminyl transferase 4" |
| 9330175E14Rik | 320377 | 9330175E14Rik | RIKEN cDNA 9330175E14 gene |
| Ptprv | 13924 | Ptprv | "protein tyrosine phosphatase, receptor type, V" |
| Clspn | 269582 | Clspn | claspin |
| Mx1 | 17857 | Mx1 | MX dynamin-like GTPase 1 |
| Mtfr2 | 71804 | Mtfr2 | mitochondrial fission regulator 2 |
| Pilrb1 | 170741 | Pilrb1 | paired immunoglobin-like type 2 receptor beta 1 |
| Vash2 | 226841 | Vash2 | vasohibin 2 |
| CR974586.5 | NA | NA | NA |
| Igkv2-112 | 381776 | Igkv2-112 | immunoglobulin kappa variable 2-112 |
| 4933430I17Rik | 214106 | 4933430I17Rik | RIKEN cDNA 4933430I17 gene |
| Fam83f | 213956 | Fam83f | "family with sequence similarity 83, member F" |
| Gm49125 | NA | NA | NA |
| Lrg1 | 76905 | Lrg1 | leucine-rich alpha-2-glycoprotein 1 |
| 2700046A07Rik | 78449 | 2700046A07Rik | RIKEN cDNA 2700046A07 gene |
| Nsl1 | 381318 | Nsl1 | "NSL1, MIS12 kinetochore complex component" |
| Dusp13 | 27389 | Dusp13 | dual specificity phosphatase 13 |
| Gm47389 | NA | NA | NA |
| Kif22 | 110033 | Kif22 | kinesin family member 22 |
| Ighv8-2 | 629849 | Ighv8-2 | immunoglobulin heavy variable V8-2 |
| Gm37468 | NA | NA | NA |
| Igkv4-51 | 619806 | Igkv4-51 | immunoglobulin kappa chain variable 4-51 |
| Ackr1 | 13349 | Ackr1 | atypical chemokine receptor 1 (Duffy blood group) |
| Kbtbd6 | 432879 | Kbtbd6 | kelch repeat and BTB (POZ) domain containing 6 |
| Rln3 | 212108 | Rln3 | relaxin 3 |

| | | | |
|---|---|---|---|
| Ccr4 | 12773 | Ccr4 | chemokine (C-C motif) receptor 4 |
| Tnfaip8l1 | 66443 | Tnfaip8l1 | "tumor necrosis factor, alpha-induced protein 8-like 1" |
| Gm46224 | 108167810 | Gm46224 | "predicted gene, 46224" |
| Gm48898 | NA | NA | NA |
| Igkv4-79 | 213684 | Igkv4-79 | immunoglobulin kappa variable 4-79 |
| Gm34643 | 102637961 | Gm34643 | "predicted gene, 34643" |
| Gm9821 | 100310873 | Gm9821 | predicted gene 9821 |
| Proz | 66901 | Proz | "protein Z, vitamin K-dependent plasma glycoprotein" |
| Gm34425 | 102637674 | Gm34425 | "predicted gene, 34425" |
| Kbtbd12 | 74589 | Kbtbd12 | kelch repeat and BTB (POZ) domain containing 12 |
| Gm37199 | NA | NA | NA |
| Lta | 16992 | Lta | lymphotoxin A |
| Sgo1 | 72415 | Sgo1 | shugoshin 1 |
| Sco2 | 100126824 | Sco2 | SCO2 cytochrome c oxidase assembly protein |
| Tgm1 | 21816 | Tgm1 | "transglutaminase 1, K polypeptide" |
| Gm15448 | 100041146 | Gm15448 | predicted gene 15448 |
| Kif5c | 16574 | Kif5c | kinesin family member 5C |
| 1600010M07Rik | 69781 | 1600010M07Rik | RIKEN cDNA 1600010M07 gene |
| Lst1 | 16988 | Lst1 | leukocyte specific transcript 1 |
| Cd163l1 | 244233 | Cd163l1 | CD163 molecule-like 1 |
| Tespa1 | 67596 | Tespa1 | "thymocyte expressed, positive selection associated 1" |
| Gm26522 | NA | NA | NA |
| Igkv4-58 | 381831 | Igkv4-58 | immunoglobulin kappa variable 4-58 |
| Igkv6-14 | 667881 | Igkv6-14 | immunoglobulin kappa variable 6-14 |
| Aurka | 20878 | Aurka | aurora kinase A |

| | | | |
|---|---|---|---|
| Gbp2b | 14468 | Gbp2b | guanylate binding protein 2b |
| Raet1d | 56554 | Raet1d | retinoic acid early transcript delta |
| Col28a1 | 213945 | Col28a1 | "collagen, type XXVIII, alpha 1" |
| Chl1 | 12661 | Chl1 | cell adhesion molecule L1-like |
| Icam5 | 15898 | Icam5 | "intercellular adhesion molecule 5, telencephalin" |
| Kif5a | 16572 | Kif5a | kinesin family member 5A |
| Exosc6 | 72544 | Exosc6 | exosome component 6 |
| Ppargc1b | 170826 | Ppargc1b | "peroxisome proliferative activated receptor, gamma, coactivator 1 beta" |
| Parpbp | 75317 | Parpbp | PARP1 binding protein |
| D6Ertd527e | 52372 | D6Ertd527e | "DNA segment, Chr 6, ERATO Doi 527, expressed" |
| Kcnd1 | 16506 | Kcnd1 | "potassium voltage-gated channel, Shal-related family, member 1" |
| Igkv8-21 | 620400 | Igkv8-21 | immunoglobulin kappa variable 8-21 |
| Prr33 | 677289 | Prr33 | proline rich 33 |
| Tmem150b | 330460 | Tmem150b | transmembrane protein 150B |
| Gm18734 | 100417645 | Gm18734 | "predicted gene, 18734" |
| Ccl19 | 24047 | Ccl19 | chemokine (C-C motif) ligand 19 |
| Sp5 | 64406 | Sp5 | trans-acting transcription factor 5 |
| Gm16712 | 100504333 | Gm16712 | "predicted gene, 16712" |
| Ighv1-36 | 629897 | Ighv1-36 | immunoglobulin heavy variable 1-36 |
| Tmem202 | 73893 | Tmem202 | transmembrane protein 202 |
| Gm15964 | 100503130 | Gm15964 | predicted gene 15964 |
| Tedc1 | 104732 | Tedc1 | tubulin epsilon and delta complex 1 |
| Gm44423 | NA | NA | NA |
| D930019O06Rik | 425051 | D930019O06Rik | RIKEN cDNA D930019O06 |
| Enthd1 | 383075 | Enthd1 | ENTH domain containing 1 |

| | | | |
|---|---|---|---|
| Ccl2 | 20296 | Ccl2 | chemokine (C-C motif) ligand 2 |
| Tnf | 21926 | Tnf | tumor necrosis factor |
| Nr5a2 | 26424 | Nr5a2 | "nuclear receptor subfamily 5, group A, member 2" |
| Ighv1-74 | 100775173 | Ighv1-74 | immunoglobulin heavy variable V1-74 |
| Orm1 | 18405 | Orm1 | orosomucoid 1 |
| Gm17275 | 100504581 | Gm17275 | "predicted gene, 17275" |
| Tm4sf5 | 75604 | Tm4sf5 | transmembrane 4 superfamily member 5 |
| Apol7b | 278679 | Apol7b | apolipoprotein L 7b |
| Chil4 | 104183 | Chil4 | chitinase-like 4 |
| Grik3 | 14807 | Grik3 | "glutamate receptor, ionotropic, kainate 3" |
| 2310040G24Rik | 381792 | 2310040G24Rik | RIKEN cDNA 2310040G24 gene |
| Ighv5-17 | 780794 | Ighv5-17 | immunoglobulin heavy variable 5-17 |
| Trip13 | 69716 | Trip13 | thyroid hormone receptor interactor 13 |
| 4930512J16Rik | 75131 | 4930512J16Rik | RIKEN cDNA 4930512J16 gene |
| Osm | 18413 | Osm | oncostatin M |
| Gfi1 | 14581 | Gfi1 | growth factor independent 1 transcription repressor |
| Gm45838 | NA | NA | NA |
| Gm42418 | NA | NA | NA |
| Ighv7-3 | 629822 | Ighv7-3 | immunoglobulin heavy variable 7-3 |
| Gm28177 | NA | NA | NA |
| Cd300ld4 | 100043123 | Cd300ld4 | CD300 molecule like family member D4 |
| Slco4a1 | 108115 | Slco4a1 | "solute carrier organic anion transporter family, member 4a1" |
| Igkv8-34 | 620126 | Igkv8-34 | immunoglobulin kappa variable 8-34 |
| Ccr8 | 12776 | Ccr8 | chemokine (C-C motif) receptor 8 |
| Igkv12-41 | 619960 | Igkv12-41 | immunoglobulin kappa chain variable 12-41 |

| | | | |
|---|---|---|---|
| Vsig8 | 240916 | Vsig8 | V-set and immunoglobulin domain containing 8 |
| Ska3 | 219114 | Ska3 | spindle and kinetochore associated complex subunit 3 |
| Igkv13-85 | 434036 | Igkv13-85 | immunoglobulin kappa chain variable 13-85 |
| Gm4873 | 232047 | Gm4873 | predicted gene 4873 |
| D630003M21Rik | 228846 | D630003M21Rik | RIKEN cDNA D630003M21 gene |
| Ighv9-4 | 636260 | Ighv9-4 | immunoglobulin heavy variable 9-4 |
| AC132148.1 | NA | NA | NA |
| Gm42372 | 105247240 | Gm42372 | "predicted gene, 42372" |
| Aicda | 11628 | Aicda | activation-induced cytidine deaminase |
| AC163635.1 | NA | NA | NA |
| Bcl2l15 | 229672 | Bcl2l15 | BCLl2-like 15 |
| Efna3 | 13638 | Efna3 | ephrin A3 |
| Igkv14-111 | 545847 | Igkv14-111 | immunoglobulin kappa variable 14-111 |
| 2310031A07Rik | 69621 | 2310031A07Rik | RIKEN cDNA 2310031A07 gene |
| Mcm10 | 70024 | Mcm10 | minichromosome maintenance 10 replication initiation factor |
| Igkv18-36 | 620088 | Igkv18-36 | immunoglobulin kappa chain variable 18-36 |
| Syt12 | 171180 | Syt12 | synaptotagmin XII |
| Bsn | 12217 | Bsn | bassoon |
| Gm36723 | 328066 | Gm36723 | "predicted gene, 36723" |
| Gm35037 | 102638484 | Gm35037 | "predicted gene, 35037" |
| Ighv1-50 | 780929 | Ighv1-50 | immunoglobulin heavy variable 1-50 |
| Podnl1 | 244550 | Podnl1 | podocan-like 1 |
| Syt2 | 20980 | Syt2 | synaptotagmin II |
| Rad54l | 19366 | Rad54l | RAD54 like (S. cerevisiae) |
| Cdk5r1 | 12569 | Cdk5r1 | "cyclin-dependent kinase 5, regulatory subunit 1 (p35)" |

| | | | |
|---|---|---|---|
| 4930503B20Rik | 75015 | 4930503B20Rik | RIKEN cDNA 4930503B20 gene |
| Fam83d | 71878 | Fam83d | "family with sequence similarity 83, member D" |
| Gm17230 | 100417294 | Gm17230 | predicted gene 17230 |
| Igkv4-77 | 667621 | Igkv4-77 | immunoglobulin kappa variable 4-77 |
| Dppa3 | 73708 | Dppa3 | developmental pluripotency-associated 3 |
| Fignl1 | 60530 | Fignl1 | fidgetin-like 1 |
| Gm45441 | NA | NA | NA |
| Pif1 | 208084 | Pif1 | PIF1 5'-to-3' DNA helicase |
| Csprs | 114564 | Csprs | component of Sp100-rs |
| Fam180a | 208164 | Fam180a | "family with sequence similarity 180, member A" |
| 5830418P13Rik | 100529079 | 5830418P13Rik | RIKEN cDNA 5830418P13 gene |
| Gm10612 | 102638502 | Gm10612 | predicted gene 10612 |
| Igkv10-95 | 434031 | Igkv10-95 | immunoglobulin kappa variable 10-95 |
| Ceacam19 | 319930 | Ceacam19 | carcinoembryonic antigen-related cell adhesion molecule 19 |
| Gpr82 | 319200 | Gpr82 | G protein-coupled receptor 82 |
| Ydjc | 69101 | Ydjc | YdjC homolog (bacterial) |
| Il12b | 16160 | Il12b | interleukin 12b |
| Kntc1 | 208628 | Kntc1 | kinetochore associated 1 |
| Gm32857 | 102635555 | Gm32857 | "predicted gene, 32857" |
| Krt79 | 223917 | Krt79 | keratin 79 |
| Gm47815 | 115487448 | Gm47815 | "predicted gene, 47815" |
| H2-T-ps | 667803 | H2-T-ps | "histocompatibility 2, T region locus, pseudogene" |
| Gm13241 | 433790 | Gm13241 | predicted gene 13241 |
| Prss30 | 30943 | Prss30 | "protease, serine 30" |
| Cdc25c | 12532 | Cdc25c | cell division cycle 25C |

| | | | |
|---|---|---|---|
| Pkd1l2 | 76645 | Pkd1l2 | polycystic kidney disease 1 like 2 |
| Fam89b | 17826 | Fam89b | "family with sequence similarity 89, member B" |
| Gm5421 | 432438 | Gm5421 | predicted gene 5421 |
| Wdr86 | 269633 | Wdr86 | WD repeat domain 86 |
| Gm34084 | 102637213 | Gm34084 | "predicted gene, 34084" |
| Itih4 | 16427 | Itih4 | "inter alpha-trypsin inhibitor, heavy chain 4" |
| Fndc9 | 320116 | Fndc9 | fibronectin type III domain containing 9 |
| Clgn | 12745 | Clgn | calmegin |
| Ctla4 | 12477 | Ctla4 | cytotoxic T-lymphocyte-associated protein 4 |
| A530030E21Rik | 320731 | A530030E21Rik | RIKEN cDNA A530030E21 gene |
| Prg4 | 96875 | Prg4 | "proteoglycan 4 (megakaryocyte stimulating factor, articular superficial zone protein)" |
| Tfap2a | 21418 | Tfap2a | "transcription factor AP-2, alpha" |
| Troap | 78733 | Troap | trophinin associated protein |
| Dpep2 | 319446 | Dpep2 | dipeptidase 2 |
| Neil2 | 382913 | Neil2 | nei like 2 (E. coli) |
| BC051142 | 407788 | BC051142 | cDNA sequence BC051142 |
| Tcrg-C4 | NA | NA | NA |
| Tert | 21752 | Tert | telomerase reverse transcriptase |
| Slc32a1 | 22348 | Slc32a1 | "solute carrier family 32 (GABA vesicular transporter), member 1" |
| Gm26745 | NA | NA | NA |
| Gpr84 | 80910 | Gpr84 | G protein-coupled receptor 84 |
| Mycn | 18109 | Mycn | "v-myc avian myelocytomatosis viral related oncogene, neuroblastoma derived" |
| Olfr60 | 18361 | Olfr60 | olfactory receptor 60 |
| Gm15513 | NA | NA | NA |

| | | | |
|---|---|---|---|
| Dkk2 | 56811 | Dkk2 | dickkopf WNT signaling pathway inhibitor 2 |
| Ajm1 | 381353 | Ajm1 | apical junction component 1 |
| Ckap2 | 80986 | Ckap2 | cytoskeleton associated protein 2 |
| Exo1 | 26909 | Exo1 | exonuclease 1 |
| Gcnt4 | 218476 | Gcnt4 | "glucosaminyl (N-acetyl) transferase 4, core 2 (beta-1,6-N-acetylglucosaminyltransferase)" |
| Gm44206 | NA | NA | NA |
| Grem1 | 23892 | Grem1 | "gremlin 1, DAN family BMP antagonist" |
| Tvp23a | 383103 | Tvp23a | trans-golgi network vesicle protein 23A |
| Gm16278 | NA | NA | NA |
| Atcay | 16467 | Atcay | "ataxia, cerebellar, Cayman type" |
| Shcbp1 | 20419 | Shcbp1 | Shc SH2-domain binding protein 1 |
| Plcxd1 | 403178 | Plcxd1 | "phosphatidylinositol-specific phospholipase C, X domain containing 1" |
| Gm10138 | 102640369 | Gm10138 | predicted gene 10138 |
| Fut1 | 14343 | Fut1 | fucosyltransferase 1 |
| Esco2 | 71988 | Esco2 | establishment of sister chromatid cohesion N-acetyltransferase 2 |
| E330020D12Rik | 626058 | E330020D12Rik | Riken cDNA E330020D12 gene |
| Dgkk | 331374 | Dgkk | diacylglycerol kinase kappa |
| Myo16 | 244281 | Myo16 | myosin XVI |
| Ighv7-1 | 780799 | Ighv7-1 | immunoglobulin heavy variable 7-1 |
| Awat1 | 245533 | Awat1 | acyl-CoA wax alcohol acyltransferase 1 |
| Spsb4 | 211949 | Spsb4 | splA/ryanodine receptor domain and SOCS box containing 4 |
| Ttbk1 | 106763 | Ttbk1 | tau tubulin kinase 1 |
| Lockd | 381822 | Lockd | lncRNA downstream of Cdkn1b |
| Gm43511 | NA | NA | NA |

| | | | |
|---|---|---|---|
| Gm47171 | NA | NA | NA |
| Gm45705 | NA | NA | NA |
| Fcor | 100503924 | Fcor | Foxo1 corepressor |
| Oosp1 | 170834 | Oosp1 | oocyte secreted protein 1 |
| Ceacam10 | 26366 | Ceacam10 | carcinoembryonic antigen-related cell adhesion molecule 10 |
| Ighv14-1 | 780801 | Ighv14-1 | immunoglobulin heavy variable 14-1 |
| AC133103.1 | NA | NA | NA |
| Gm28884 | NA | NA | NA |
| Gm13304 | 100504346 | Gm13304 | predicted gene 13304 |
| Neil3 | 234258 | Neil3 | nei like 3 (E. coli) |
| Gm28286 | NA | NA | NA |
| Gm7609 | 665378 | Gm7609 | predicted pseudogene 7609 |
| 1300014J16Rik | 74160 | 1300014J16Rik | RIKEN cDNA 1300014J16 gene |
| H2afx | NA | NA | NA |
| Gm5345 | 384808 | Gm5345 | predicted gene 5345 |
| Gm39458 | 105243571 | Gm39458 | "predicted gene, 39458" |
| Cela1 | 109901 | Cela1 | "chymotrypsin-like elastase family, member 1" |
| Galr3 | 14429 | Galr3 | galanin receptor 3 |
| 2610300M13Rik | 72484 | 2610300M13Rik | RIKEN cDNA 2610300M13 gene |
| Gm30329 | 102632189 | Gm30329 | "predicted gene, 30329" |
| Nrxn3 | 18191 | Nrxn3 | neurexin III |
| Tnni2 | 21953 | Tnni2 | "troponin I, skeletal, fast 2" |
| Aldh1b1 | 72535 | Aldh1b1 | "aldehyde dehydrogenase 1 family, member B1" |
| Igkv4-71 | 434586 | Igkv4-71 | immunoglobulin kappa chain variable 4-71 |
| Olfr111 | 545205 | Olfr111 | olfactory receptor 111 |

| | | | |
|---|---|---|---|
| Ptpn5 | 19259 | Ptpn5 | "protein tyrosine phosphatase, non-receptor type 5" |
| C530008M17Rik | 320827 | C530008M17Rik | RIKEN cDNA C530008M17 gene |
| Tnfsf11 | 21943 | Tnfsf11 | "tumor necrosis factor (ligand) superfamily, member 11" |
| Gm9864 | NA | NA | NA |
| 1700071M16Rik | 73504 | 1700071M16Rik | RIKEN cDNA 1700071M16 gene |
| Il17rb | 50905 | Il17rb | interleukin 17 receptor B |
| Igkv14-126 | 628127 | Igkv14-126 | immunoglobulin kappa variable 14-126 |
| Siah3 | 380918 | Siah3 | siah E3 ubiquitin protein ligase family member 3 |
| Gm11906 | NA | NA | NA |
| Gm49254 | NA | NA | NA |
| Slit1 | 20562 | Slit1 | slit guidance ligand 1 |
| Btnl5-ps | 81497 | Btnl5-ps | "butyrophilin-like 5, pseudogene" |
| 2010016I18Rik | 69206 | 2010016I18Rik | RIKEN cDNA 2010016I18 gene |
| Igkv4-92 | 384410 | Igkv4-92 | immunoglobulin kappa variable 4-92 |
| Gm26530 | NA | NA | NA |
| Cdhr1 | 170677 | Cdhr1 | cadherin-related family member 1 |
| Igkv3-7 | 108005 | Igkv3-7 | immunoglobulin kappa variable 3-7 |
| Ntrk2 | 18212 | Ntrk2 | "neurotrophic tyrosine kinase, receptor, type 2" |
| Gm11110 | 100169874 | Gm11110 | predicted gene 11110 |
| Gm48128 | NA | NA | NA |
| Wfdc18 | 14038 | Wfdc18 | WAP four-disulfide core domain 18 |
| Hist1h1d | NA | NA | NA |
| 9330020H09Rik | 100048895 | 9330020H09Rik | RIKEN cDNA 9330020H09 gene |
| Dock3 | 208869 | Dock3 | dedicator of cyto-kinesis 3 |
| Gm29216 | NA | NA | NA |

| | | | |
|---|---|---|---|
| Gm26902 | NA | NA | NA |
| Bcat1 | 12035 | Bcat1 | "branched chain aminotransferase 1, cytosolic" |
| Mxd3 | 17121 | Mxd3 | Max dimerization protein 3 |
| 2900089D17Rik | 67277 | 2900089D17Rik | RIKEN cDNA 2900089D17 gene |
| Basp1 | 70350 | Basp1 | "brain abundant, membrane attached signal protein 1" |
| AC134555.1 | NA | NA | NA |
| Gm6311 | 622313 | Gm6311 | predicted gene 6311 |
| Gm26549 | NA | NA | NA |
| Slc9b2 | 97086 | Slc9b2 | "solute carrier family 9, subfamily B (NHA2, cation proton antiporter 2), member 2" |
| Cyp21a1 | 13079 | Cyp21a1 | "cytochrome P450, family 21, subfamily a, polypeptide 1" |
| Angptl3 | 30924 | Angptl3 | angiopoietin-like 3 |
| Pdxp | 57028 | Pdxp | "pyridoxal (pyridoxine, vitamin B6) phosphatase" |
| Gm31718 | 102634034 | Gm31718 | "predicted gene, 31718" |
| Dok6 | 623279 | Dok6 | docking protein 6 |
| Mef2b | 17259 | Mef2b | myocyte enhancer factor 2B |
| Gm47071 | NA | NA | NA |
| Gm16206 | 102632669 | Gm16206 | predicted gene 16206 |
| Gm36989 | NA | NA | NA |
| Gm8229 | 666675 | Gm8229 | predicted gene 8229 |
| Kifc5b | 16580 | Kifc5b | kinesin family member C5B |
| Clnk | 27278 | Clnk | cytokine-dependent hematopoietic cell linker |
| Gm3608 | NA | NA | NA |
| Cerkl | 228094 | Cerkl | ceramide kinase-like |
| Prss16 | 54373 | Prss16 | "protease, serine 16 (thymus)" |
| Ighv1-37 | 668517 | Ighv1-37 | immunoglobulin heavy variable 1-37 |

| | | | |
|---|---|---|---|
| 2010008C14Rik | 69859 | 2010008C14Rik | RIKEN cDNA 2010008C14 gene |
| Sms-ps | 671878 | Sms-ps | "spermine synthase, pseudogene" |
| Gm31814 | 102634162 | Gm31814 | "predicted gene, 31814" |
| Pdyn | 18610 | Pdyn | prodynorphin |
| AW822252 | 331578 | AW822252 | expressed sequence AW822252 |
| 4930432E11Rik | 243900 | 4930432E11Rik | RIKEN cDNA 4930432E11 gene |
| Plxnb3 | 140571 | Plxnb3 | plexin B3 |
| Chek1 | 12649 | Chek1 | checkpoint kinase 1 |
| Pkhd1 | 241035 | Pkhd1 | polycystic kidney and hepatic disease 1 |
| Gm45867 | NA | NA | NA |
| Frzb | 20378 | Frzb | frizzled-related protein |
| Cd200r2 | 271375 | Cd200r2 | Cd200 receptor 2 |
| 9930022D16Rik | 320509 | 9930022D16Rik | RIKEN cDNA 9930022D16 gene |
| Prss35 | 244954 | Prss35 | "protease, serine 35" |
| Gm13391 | 102632465 | Gm13391 | predicted gene 13391 |
| Gm7535 | 665187 | Gm7535 | predicted gene 7535 |
| Rgs8 | 67792 | Rgs8 | regulator of G-protein signaling 8 |
| Olfr1459 | 258684 | Olfr1459 | olfactory receptor 1459 |
| Dyrk4 | 101320 | Dyrk4 | dual-specificity tyrosine-(Y)-phosphorylation regulated kinase 4 |
| Aadac | 67758 | Aadac | arylacetamide deacetylase |
| Gm29291 | NA | NA | NA |
| Krt20 | 66809 | Krt20 | keratin 20 |
| Gm37893 | NA | NA | NA |
| Il17a | 16171 | Il17a | interleukin 17A |
| A230028O05Rik | 319487 | A230028O05Rik | RIKEN cDNA A230028O05 gene |

| | | | |
|---|---|---|---|
| Gm43909 | NA | NA | NA |
| Gm33251 | 102636079 | Gm33251 | "predicted gene, 33251" |
| Gm44198 | NA | NA | NA |
| Cnbd2 | 70873 | Cnbd2 | cyclic nucleotide binding domain containing 2 |
| Apba2 | 11784 | Apba2 | "amyloid beta (A4) precursor protein-binding, family A, member 2" |
| Gm48562 | NA | NA | NA |
| Gm28100 | NA | NA | NA |
| 1700009J07Rik | 75188 | 1700009J07Rik | RIKEN cDNA 1700009J07 gene |
| Impg2 | 224224 | Impg2 | interphotoreceptor matrix proteoglycan 2 |
| Pla2g4c | 232889 | Pla2g4c | "phospholipase A2, group IVC (cytosolic, calcium-independent)" |
| Gm12537 | 100041342 | Gm12537 | predicted gene 12537 |
| C230014O12Rik | 329387 | C230014O12Rik | RIKEN cDNA C230014O12 gene |
| Xlr4c | 72891 | Xlr4c | X-linked lymphocyte-regulated 4C |
| Gm44154 | NA | NA | NA |
| Hist2h3c2 | NA | NA | NA |
| Olfr157 | 100040268 | Olfr157 | olfactory receptor 157 |
| Gm16350 | 102638701 | Gm16350 | predicted gene 16350 |
| Ankle1 | 234396 | Ankle1 | ankyrin repeat and LEM domain containing 1 |
| Hmgb1-ps8 | 667280 | Hmgb1-ps8 | "high mobility group box 1, pseudogene 8" |
| Gzmk | 14945 | Gzmk | granzyme K |
| Creb3l3 | 208677 | Creb3l3 | cAMP responsive element binding protein 3-like 3 |
| Trdv5 | 626545 | Trdv5 | T cell receptor delta variable 5 |
| Gm44739 | NA | NA | NA |
| Krt83 | 100126226 | Krt83 | keratin 83 |
| Gm45218 | NA | NA | NA |

| | | | |
|---|---|---|---|
| Dok5 | 76829 | Dok5 | docking protein 5 |
| Ascl2 | 17173 | Ascl2 | achaete-scute family bHLH transcription factor 2 |
| Gm6236 | 100534370 | Gm6236 | predicted gene 6236 |
| E030037K01Rik | NA | NA | NA |
| Noxa1 | 241275 | Noxa1 | NADPH oxidase activator 1 |
| Trav7-4 | 386450 | Trav7-4 | T cell receptor alpha variable 7-4 |
| Fam181a | 100504156 | Fam181a | "family with sequence similarity 181, member A" |
| Ryr1 | 20190 | Ryr1 | "ryanodine receptor 1, skeletal muscle" |
| 4933417C20Rik | 71128 | 4933417C20Rik | RIKEN cDNA 4933417C20 gene |
| Gm38832 | 105242682 | Gm38832 | "predicted gene, 38832" |
| Gm16685 | 102634900 | Gm16685 | "predicted gene, 16685" |
| Gm15645 | 626055 | Gm15645 | predicted gene 15645 |
| Gm48210 | NA | NA | NA |
| Fhl5 | 57756 | Fhl5 | four and a half LIM domains 5 |
| H60b | 667281 | H60b | histocompatibility 60b |
| 9430064I24Rik | 100327266 | 9430064I24Rik | RIKEN cDNA 9430064I24 gene |
| Gm37696 | NA | NA | NA |
| Kcnj9 | 16524 | Kcnj9 | "potassium inwardly-rectifying channel, subfamily J, member 9" |
| Gpr31a | 625031 | Gpr31a | "G protein-coupled receptor 31, D17Leh66a region" |
| Gm39323 | 105243388 | Gm39323 | "predicted gene, 39323" |
| Pam16 | 66449 | Pam16 | presequence translocase-asssociated motor 16 homolog (S. cerevisiae) |
| Gm26546 | NA | NA | NA |
| Igf2bp3 | 140488 | Igf2bp3 | insulin-like growth factor 2 mRNA binding protein 3 |
| Cenpk | 60411 | Cenpk | centromere protein K |
| Mir155hg | 100653389 | Mir155hg | Mir155 host gene (non-protein coding) |

| | | | |
|---|---|---|---|
| Cst6 | 73720 | Cst6 | cystatin E/M |
| Chodl | 246048 | Chodl | chondrolectin |
| Igkv3-3 | 667946 | Igkv3-3 | immunoglobulin kappa variable 3-3 |
| Gnat2 | 14686 | Gnat2 | "guanine nucleotide binding protein, alpha transducing 2" |
| Gm38335 | NA | NA | NA |
| Gm26536 | 102639417 | Gm26536 | "predicted gene, 26536" |
| Onecut2 | 225631 | Onecut2 | "one cut domain, family member 2" |
| Tas1r1 | 110326 | Tas1r1 | "taste receptor, type 1, member 1" |
| Igkv4-60 | 385277 | Igkv4-60 | immunoglobulin kappa variable 4-60 |
| Trav15d-1-dv6d-1 | 436468 | Trav15d-1-dv6d-1 | T cell receptor alpha variable 15D-1-DV6D-1 |
| Ighv1-56 | 382695 | Ighv1-56 | immunoglobulin heavy variable 1-56 |
| Sostdc1 | 66042 | Sostdc1 | sclerostin domain containing 1 |
| Ska1 | 66468 | Ska1 | spindle and kinetochore associated complex subunit 1 |
| Cenpm | 66570 | Cenpm | centromere protein M |
| Gm45250 | NA | NA | NA |
| AC126937.1 | NA | NA | NA |
| A330040F15Rik | 74333 | A330040F15Rik | RIKEN cDNA A330040F15 gene |
| Zfp599 | 235048 | Zfp599 | zinc finger protein 599 |
| Il23r | 209590 | Il23r | interleukin 23 receptor |
| Sprr2a2 | 100303744 | Sprr2a2 | small proline-rich protein 2A2 |
| Gm20100 | 100504175 | Gm20100 | "predicted gene, 20100" |
| Odf3l1 | 382075 | Odf3l1 | outer dense fiber of sperm tails 3-like 1 |
| 2410017I17Rik | 675325 | 2410017I17Rik | RIKEN cDNA 2410017I17 gene |
| Gm44175 | NA | NA | NA |
| Gm37004 | 115489463 | Gm37004 | "predicted gene, 37004" |

| | | | |
|---|---|---|---|
| Gm17484 | 100503003 | Gm17484 | "predicted gene, 17484" |
| Pglyrp1 | 21946 | Pglyrp1 | peptidoglycan recognition protein 1 |
| Igkv4-69 | 628541 | Igkv4-69 | immunoglobulin kappa variable 4-69 |
| Afp | 11576 | Afp | alpha fetoprotein |
| Duox1 | 99439 | Duox1 | dual oxidase 1 |
| Eme1 | 268465 | Eme1 | essential meiotic structure-specific endonuclease 1 |
| Trbv20 | 100124685 | Trbv20 | "T cell receptor beta, variable 20" |
| Rimbp2 | 231760 | Rimbp2 | RIMS binding protein 2 |
| Ighv1-15 | 629865 | Ighv1-15 | immunoglobulin heavy variable 1-15 |
| Hist1h4j | NA | NA | NA |
| Gm16071 | 100384881 | Gm16071 | predicted gene 16071 |
| Dnmt3aos | 100038522 | Dnmt3aos | "DNA methyltransferase 3A, opposite strand" |
| Rasal1 | 19415 | Rasal1 | RAS protein activator like 1 (GAP1 like) |
| Gm49774 | NA | NA | NA |
| Gm13622 | NA | NA | NA |
| Gjb4 | 14621 | Gjb4 | "gap junction protein, beta 4" |
| Cited4 | 56222 | Cited4 | "Cbp/p300-interacting transactivator, with Glu/Asp-rich carboxy-terminal domain, 4" |
| Ighv14-4 | 629826 | Ighv14-4 | immunoglobulin heavy variable 14-4 |
| Gm6904 | NA | NA | NA |
| Eya4 | 14051 | Eya4 | EYA transcriptional coactivator and phosphatase 4 |
| Gm48627 | NA | NA | NA |
| Gm45059 | NA | NA | NA |
| Gm45684 | NA | NA | NA |
| Cryba4 | 12959 | Cryba4 | "crystallin, beta A4" |

| | | | |
|---|---|---|---|
| Sbk2 | 381836 | Sbk2 | "SH3-binding domain kinase family, member 2" |
| Eef1akmt3 | 100504608 | Eef1akmt3 | EEF1A lysine methyltransferase 3 |
| Gm26486 | NA | NA | NA |
| Galnt9 | 231605 | Galnt9 | polypeptide N-acetylgalactosaminyltransferase 9 |
| Gm10143 | 100038753 | Gm10143 | predicted gene 10143 |
| Trbv4 | 100124660 | Trbv4 | "T cell receptor beta, variable 10" |
| Il27 | 246779 | Il27 | interleukin 27 |
| Gm37648 | NA | NA | NA |
| Gm13290 | 545646 | Gm13290 | predicted gene 13290 |
| Ighv7-2 | 780800 | Ighv7-2 | immunoglobulin heavy variable 7-2 |
| Hsd17b1 | 15485 | Hsd17b1 | hydroxysteroid (17-beta) dehydrogenase 1 |
| Ccl1 | 20290 | Ccl1 | chemokine (C-C motif) ligand 1 |
| Fcamr | 64435 | Fcamr | "Fc receptor, IgA, IgM, high affinity" |
| Gm16170 | 100861855 | Gm16170 | predicted gene 16170 |
| G730013B05Rik | 619312 | G730013B05Rik | RIKEN cDNA G730013B05 gene |
| Gm13270 | 105244084 | Gm13270 | predicted gene 13270 |
| St8sia6 | 241230 | St8sia6 | "ST8 alpha-N-acetyl-neuraminide alpha-2,8-sialyltransferase 6" |
| Hist2h4 | NA | NA | NA |
| Speer4c | 100502967 | Speer4c | spermatogenesis associated glutamate (E)-rich protein 4C |
| Ly6k | 76486 | Ly6k | "lymphocyte antigen 6 complex, locus K" |
| 5430437J10Rik | 71432 | 5430437J10Rik | RIKEN cDNA 5430437J10 gene |
| Gm19510 | 100503021 | Gm19510 | "predicted gene, 19510" |
| Gm48065 | NA | NA | NA |
| Gm42967 | NA | NA | NA |
| Fgl1 | 234199 | Fgl1 | fibrinogen-like protein 1 |

| | | | |
|---|---|---|---|
| Gm49502 | NA | NA | NA |
| Gm37168 | 102634085 | Gm37168 | "predicted gene, 37168" |
| B230317F23Rik | 320383 | B230317F23Rik | RIKEN cDNA B230317F23 gene |
| Gm20658 | 102633213 | Gm20658 | predicted gene 20658 |
| Gm15542 | 100043546 | Gm15542 | predicted gene 15542 |
| 1700061G19Rik | NA | NA | NA |
| AC154707.1 | NA | NA | NA |
| Gm43513 | NA | NA | NA |
| Gm6445 | 623688 | Gm6445 | predicted gene 6445 |
| Lipc | 15450 | Lipc | "lipase, hepatic" |
| Gm32089 | 102634530 | Gm32089 | "predicted gene, 32089" |
| Rnaset2a | 100037283 | Rnaset2a | ribonuclease T2A |
| Gm1070 | 381785 | Gm1070 | predicted gene 1070 |
| Dmrta1 | 242523 | Dmrta1 | doublesex and mab-3 related transcription factor like family A1 |
| Ighv11-2 | 780818 | Ighv11-2 | immunoglobulin heavy variable V11-2 |
| AC124108.1 | NA | NA | NA |
| Gm48604 | NA | NA | NA |
| Trbv14 | 100124678 | Trbv14 | "T cell receptor beta, variable 14" |
| Ighv1-62-3 | 668549 | Ighv1-62-3 | immunoglobulin heavy variable 1-62-3 |
| Gm28306 | NA | NA | NA |
| Gm47882 | NA | NA | NA |
| Gm10518 | 100040914 | Gm10518 | predicted gene 10518 |
| Gm2564 | 100040035 | Gm2564 | predicted gene 2564 |
| Cacng5 | 140723 | Cacng5 | "calcium channel, voltage-dependent, gamma subunit 5" |
| Gm12187 | 100502678 | Gm12187 | predicted gene 12187 |

| | | | |
|---|---|---|---|
| Igfbp3 | 16009 | Igfbp3 | insulin-like growth factor binding protein 3 |
| Gstm2 | 14863 | Gstm2 | "glutathione S-transferase, mu 2" |
| Gria1 | 14799 | Gria1 | "glutamate receptor, ionotropic, AMPA1 (alpha 1)" |
| Fibin | 67606 | Fibin | fin bud initiation factor homolog (zebrafish) |
| Pon1 | 18979 | Pon1 | paraoxonase 1 |
| Id1 | 15901 | Id1 | "inhibitor of DNA binding 1, HLH protein" |
| Arl4d | 80981 | Arl4d | ADP-ribosylation factor-like 4D |
| Colq | 382864 | Colq | collagen-like tail subunit (single strand of homotrimer) of asymmetric acetylcholinesterase |
| Klf15 | 66277 | Klf15 | Kruppel-like factor 15 |
| Kcna2 | 16490 | Kcna2 | "potassium voltage-gated channel, shaker-related subfamily, member 2" |
| Serpina3c | 16625 | Serpina3c | "serine (or cysteine) peptidase inhibitor, clade A, member 3C" |
| Igfbp6 | 16012 | Igfbp6 | insulin-like growth factor binding protein 6 |
| Crispld2 | 78892 | Crispld2 | cysteine-rich secretory protein LCCL domain containing 2 |
| Cyp4b1 | 13120 | Cyp4b1 | "cytochrome P450, family 4, subfamily b, polypeptide 1" |
| Cavin2 | 20324 | Cavin2 | caveolae associated 2 |
| Hpgd | 15446 | Hpgd | hydroxyprostaglandin dehydrogenase 15 (NAD) |
| Tmcc2 | 68875 | Tmcc2 | transmembrane and coiled-coil domains 2 |
| Tgfbr3 | 21814 | Tgfbr3 | "transforming growth factor, beta receptor III" |
| Sorbs1 | 20411 | Sorbs1 | sorbin and SH3 domain containing 1 |
| Myh10 | 77579 | Myh10 | "myosin, heavy polypeptide 10, non-muscle" |
| Cdkn1a | 12575 | Cdkn1a | cyclin-dependent kinase inhibitor 1A (P21) |
| Fermt2 | 218952 | Fermt2 | fermitin family member 2 |
| Limch1 | 77569 | Limch1 | LIM and calponin homology domains 1 |
| Nckap5 | 210356 | Nckap5 | NCK-associated protein 5 |

| | | | |
|---|---|---|---|
| Fzd4 | 14366 | Fzd4 | frizzled class receptor 4 |
| Errfi1 | 74155 | Errfi1 | ERBB receptor feedback inhibitor 1 |
| Bex4 | 406217 | Bex4 | brain expressed X-linked 4 |
| Klf9 | 16601 | Klf9 | Kruppel-like factor 9 |
| Pcolce2 | 76477 | Pcolce2 | procollagen C-endopeptidase enhancer 2 |
| Foxf1 | 15227 | Foxf1 | forkhead box F1 |
| 9330159F19Rik | 212448 | 9330159F19Rik | RIKEN cDNA 9330159F19 gene |
| S1pr1 | 13609 | S1pr1 | sphingosine-1-phosphate receptor 1 |
| Tln2 | 70549 | Tln2 | talin 2 |
| Bmp6 | 12161 | Bmp6 | bone morphogenetic protein 6 |
| Glb1l2 | 244757 | Glb1l2 | "galactosidase, beta 1-like 2" |
| Hecw2 | 329152 | Hecw2 | "HECT, C2 and WW domain containing E3 ubiquitin protein ligase 2" |
| Scgb1a1 | 22287 | Scgb1a1 | "secretoglobin, family 1A, member 1 (uteroglobin)" |
| Gsta3 | 14859 | Gsta3 | "glutathione S-transferase, alpha 3" |
| Faim2 | 72393 | Faim2 | Fas apoptotic inhibitory molecule 2 |
| Arrdc3 | 105171 | Arrdc3 | arrestin domain containing 3 |
| Hopx | 74318 | Hopx | HOP homeobox |
| Gsn | 227753 | Gsn | gelsolin |
| Zhx3 | 320799 | Zhx3 | zinc fingers and homeoboxes 3 |
| Efnb1 | 13641 | Efnb1 | ephrin B1 |
| Pi15 | 94227 | Pi15 | peptidase inhibitor 15 |
| Fgfr3 | 14184 | Fgfr3 | fibroblast growth factor receptor 3 |
| Amph | 218038 | Amph | amphiphysin |
| Bex2 | 12069 | Bex2 | brain expressed X-linked 2 |
| Tspan12 | 269831 | Tspan12 | tetraspanin 12 |

| | | | |
|---|---|---|---|
| Efemp1 | 216616 | Efemp1 | epidermal growth factor-containing fibulin-like extracellular matrix protein 1 |
| Col13a1 | 12817 | Col13a1 | "collagen, type XIII, alpha 1" |
| Efnb2 | 13642 | Efnb2 | ephrin B2 |
| Prss23 | 76453 | Prss23 | "protease, serine 23" |
| Inmt | 21743 | Inmt | indolethylamine N-methyltransferase |
| Cyp2d22 | 56448 | Cyp2d22 | "cytochrome P450, family 2, subfamily d, polypeptide 22" |
| Egfr | 13649 | Egfr | epidermal growth factor receptor |
| Prkg2 | 19092 | Prkg2 | "protein kinase, cGMP-dependent, type II" |
| Ston1 | 77057 | Ston1 | stonin 1 |
| Mill2 | 243864 | Mill2 | MHC I like leukocyte 2 |
| Gm5706 | 435657 | Gm5706 | predicted gene 5706 |
| Ednrb | 13618 | Ednrb | endothelin receptor type B |
| Tns2 | 209039 | Tns2 | tensin 2 |
| Slc24a3 | 94249 | Slc24a3 | "solute carrier family 24 (sodium/potassium/calcium exchanger), member 3" |
| Phf24 | 230085 | Phf24 | PHD finger protein 24 |
| Galnt15 | 78754 | Galnt15 | polypeptide N-acetylgalactosaminyltransferase 15 |
| Olfml2a | 241327 | Olfml2a | olfactomedin-like 2A |
| Cdc42bpg | 240505 | Cdc42bpg | CDC42 binding protein kinase gamma (DMPK-like) |
| Rcan2 | 53901 | Rcan2 | regulator of calcineurin 2 |
| Alas2 | 11656 | Alas2 | "aminolevulinic acid synthase 2, erythroid" |
| Pla2r1 | 18779 | Pla2r1 | phospholipase A2 receptor 1 |
| Hsd11b1 | 15483 | Hsd11b1 | hydroxysteroid 11-beta dehydrogenase 1 |
| Reck | 53614 | Reck | reversion-inducing-cysteine-rich protein with kazal motifs |
| Adamts15 | 235130 | Adamts15 | "a disintegrin-like and metallopeptidase (reprolysin type) with thrombospondin type 1 motif, 15" |

| | | | |
|---|---|---|---|
| Lipg | 16891 | Lipg | "lipase, endothelial" |
| Fam198b | NA | NA | NA |
| Ndrg2 | 29811 | Ndrg2 | N-myc downstream regulated gene 2 |
| Cst8 | 13012 | Cst8 | cystatin 8 (cystatin-related epididymal spermatogenic) |
| Gata2 | 14461 | Gata2 | GATA binding protein 2 |
| Nr4a1 | 15370 | Nr4a1 | "nuclear receptor subfamily 4, group A, member 1" |
| Ltbp4 | 108075 | Ltbp4 | latent transforming growth factor beta binding protein 4 |
| Ces1g | 12623 | Ces1g | carboxylesterase 1G |
| Cldn10 | 58187 | Cldn10 | claudin 10 |
| Epas1 | 13819 | Epas1 | endothelial PAS domain protein 1 |
| Abca5 | 217265 | Abca5 | "ATP-binding cassette, sub-family A (ABC1), member 5" |
| Plpp3 | 67916 | Plpp3 | phospholipid phosphatase 3 |
| Pim3 | 223775 | Pim3 | proviral integration site 3 |
| Ecm2 | 407800 | Ecm2 | "extracellular matrix protein 2, female organ and adipocyte specific" |
| Slit3 | 20564 | Slit3 | slit guidance ligand 3 |
| Raver2 | 242570 | Raver2 | "ribonucleoprotein, PTB-binding 2" |
| Csgalnact1 | 234356 | Csgalnact1 | chondroitin sulfate N-acetylgalactosaminyltransferase 1 |
| Sik1 | 17691 | Sik1 | salt inducible kinase 1 |
| Vgll3 | 73569 | Vgll3 | vestigial like family member 3 |
| Cdkn1c | 12577 | Cdkn1c | cyclin-dependent kinase inhibitor 1C (P57) |
| Gnao1 | 14681 | Gnao1 | "guanine nucleotide binding protein, alpha O" |
| Lrat | 79235 | Lrat | lecithin-retinol acyltransferase (phosphatidylcholine-retinol-O-acyltransferase) |
| Dpysl5 | 65254 | Dpysl5 | dihydropyrimidinase-like 5 |
| Adgrl3 | 319387 | Adgrl3 | adhesion G protein-coupled receptor L3 |
| Rasgef1b | 320292 | Rasgef1b | "RasGEF domain family, member 1B" |

| | | | |
|---|---|---|---|
| Tspan7 | 21912 | Tspan7 | tetraspanin 7 |
| Atp1a2 | 98660 | Atp1a2 | "ATPase, Na+/K+ transporting, alpha 2 polypeptide" |
| Tubb1 | 545486 | Tubb1 | "tubulin, beta 1 class VI" |
| Cyp2f2 | 13107 | Cyp2f2 | "cytochrome P450, family 2, subfamily f, polypeptide 2" |
| Lims2 | 225341 | Lims2 | LIM and senescent cell antigen like domains 2 |
| Aldh1a1 | 11668 | Aldh1a1 | "aldehyde dehydrogenase family 1, subfamily A1" |
| Colgalt2 | 269132 | Colgalt2 | collagen beta(1-O)galactosyltransferase 2 |
| Pde3a | 54611 | Pde3a | "phosphodiesterase 3A, cGMP inhibited" |
| Abi3bp | 320712 | Abi3bp | "ABI gene family, member 3 (NESH) binding protein" |
| Smad6 | 17130 | Smad6 | SMAD family member 6 |
| Slc7a10 | 53896 | Slc7a10 | "solute carrier family 7 (cationic amino acid transporter, y+ system), member 10" |
| Eda | 13607 | Eda | ectodysplasin-A |
| Slc10a6 | 75750 | Slc10a6 | "solute carrier family 10 (sodium/bile acid cotransporter family), member 6" |
| Slc5a12 | 241612 | Slc5a12 | "solute carrier family 5 (sodium/glucose cotransporter), member 12" |
| Synm | 233335 | Synm | "synemin, intermediate filament protein" |
| Sorbs3 | 20410 | Sorbs3 | sorbin and SH3 domain containing 3 |
| Aox1 | 11761 | Aox1 | aldehyde oxidase 1 |
| Edil3 | 13612 | Edil3 | EGF-like repeats and discoidin I-like domains 3 |
| Ptgfr | 19220 | Ptgfr | prostaglandin F receptor |
| Cd300lg | 52685 | Cd300lg | CD300 molecule like family member G |
| a | 50518 | a | nonagouti |
| Rgs6 | 50779 | Rgs6 | regulator of G-protein signaling 6 |
| Scn3a | 20269 | Scn3a | "sodium channel, voltage-gated, type III, alpha" |
| Apln | 30878 | Apln | apelin |
| Gpm6a | 234267 | Gpm6a | glycoprotein m6a |

| | | | |
|---|---|---|---|
| F3 | 14066 | F3 | coagulation factor III |
| Xpnpep2 | 170745 | Xpnpep2 | "X-prolyl aminopeptidase (aminopeptidase P) 2, membrane-bound" |
| Fxyd1 | 56188 | Fxyd1 | FXYD domain-containing ion transport regulator 1 |
| Tmem47 | 192216 | Tmem47 | transmembrane protein 47 |
| Col12a1 | 12816 | Col12a1 | "collagen, type XII, alpha 1" |
| Plce1 | 74055 | Plce1 | "phospholipase C, epsilon 1" |
| Pdgfra | 18595 | Pdgfra | "platelet derived growth factor receptor, alpha polypeptide" |
| Adamts5 | 23794 | Adamts5 | "a disintegrin-like and metallopeptidase (reprolysin type) with thrombospondin type 1 motif, 5 (aggrecanase-2)" |
| Gm9917 | 100038755 | Gm9917 | predicted gene 9917 |
| Slc38a4 | 69354 | Slc38a4 | "solute carrier family 38, member 4" |
| Tubb4a | 22153 | Tubb4a | "tubulin, beta 4A class IVA" |
| Ogn | 18295 | Ogn | osteoglycin |
| Dpep1 | 13479 | Dpep1 | dipeptidase 1 |
| Gm14964 | 100008567 | Gm14964 | predicted gene 14964 |
| Gzmb | 14939 | Gzmb | granzyme B |
| Tagln | 21345 | Tagln | transgelin |
| Fkbp14 | 231997 | Fkbp14 | FK506 binding protein 14 |
| Akap12 | 83397 | Akap12 | A kinase (PRKA) anchor protein (gravin) 12 |
| Gm4956 | 241041 | Gm4956 | predicted gene 4956 |
| Gstm3 | 14864 | Gstm3 | "glutathione S-transferase, mu 3" |
| Stxbp6 | 217517 | Stxbp6 | syntaxin binding protein 6 (amisyn) |
| Fmo2 | 55990 | Fmo2 | flavin containing monooxygenase 2 |
| Nfkbia | 18035 | Nfkbia | "nuclear factor of kappa light polypeptide gene enhancer in B cells inhibitor, alpha" |
| Cldn5 | 12741 | Cldn5 | claudin 5 |

| | | | |
|---|---|---|---|
| Gdpd2 | 71584 | Gdpd2 | glycerophosphodiester phosphodiesterase domain containing 2 |
| Hoxb5 | 15413 | Hoxb5 | homeobox B5 |
| G0s2 | 14373 | G0s2 | G0/G1 switch gene 2 |
| Mustn1 | 66175 | Mustn1 | "musculoskeletal, embryonic nuclear protein 1" |
| Aass | 30956 | Aass | aminoadipate-semialdehyde synthase |
| Tmeff2 | 56363 | Tmeff2 | transmembrane protein with EGF-like and two follistatin-like domains 2 |
| Krt80 | 74127 | Krt80 | keratin 80 |
| Sgip1 | 73094 | Sgip1 | SH3-domain GRB2-like (endophilin) interacting protein 1 |
| Rspo1 | 192199 | Rspo1 | R-spondin 1 |
| Pde8b | 218461 | Pde8b | phosphodiesterase 8B |
| Eng | 13805 | Eng | endoglin |
| Pgm5 | 226041 | Pgm5 | phosphoglucomutase 5 |
| Acer2 | 230379 | Acer2 | alkaline ceramidase 2 |
| Mapt | 17762 | Mapt | microtubule-associated protein tau |
| Col17a1 | 12821 | Col17a1 | "collagen, type XVII, alpha 1" |
| Bche | 12038 | Bche | butyrylcholinesterase |
| Ankrd29 | 225187 | Ankrd29 | ankyrin repeat domain 29 |
| Plvap | 84094 | Plvap | plasmalemma vesicle associated protein |
| Igfbp5 | 16011 | Igfbp5 | insulin-like growth factor binding protein 5 |
| Snca | 20617 | Snca | "synuclein, alpha" |
| Nkain4 | 58237 | Nkain4 | Na+/K+ transporting ATPase interacting 4 |
| Prrt4 | 101359 | Prrt4 | proline-rich transmembrane protein 4 |
| Klf4 | 16600 | Klf4 | Kruppel-like factor 4 (gut) |
| Ppbp | 57349 | Ppbp | pro-platelet basic protein |
| Adamts9 | 101401 | Adamts9 | "a disintegrin-like and metallopeptidase (reprolysin type) with thrombospondin |

| | | | |
|---|---|---|---|
| | | | type 1 motif, 9" |
| Nrxn2 | 18190 | Nrxn2 | neurexin II |
| Pcsk6 | 18553 | Pcsk6 | proprotein convertase subtilisin/kexin type 6 |
| mt-Rnr2 | 17725 | mt-Rnr2 | "16S rRNA, mitochondrial" |
| Olfm2 | 244723 | Olfm2 | olfactomedin 2 |
| C7 | 109828 | C7 | complement component 7 |
| Npas2 | 18143 | Npas2 | neuronal PAS domain protein 2 |
| Akap5 | 238276 | Akap5 | A kinase (PRKA) anchor protein 5 |
| Sox6 | 20679 | Sox6 | SRY (sex determining region Y)-box 6 |
| Vipr2 | 22355 | Vipr2 | vasoactive intestinal peptide receptor 2 |
| Cnga2 | 12789 | Cnga2 | cyclic nucleotide gated channel alpha 2 |
| Hmgcs2 | 15360 | Hmgcs2 | 3-hydroxy-3-methylglutaryl-Coenzyme A synthase 2 |
| Sbsn | 282619 | Sbsn | suprabasin |
| Klrb1c | 17059 | Klrb1c | killer cell lectin-like receptor subfamily B member 1C |
| St6galnac3 | 20447 | St6galnac3 | "ST6 (alpha-N-acetyl-neuraminyl-2,3-beta-galactosyl-1,3)-N-acetylgalactosaminide alpha-2,6-sialyltransferase 3" |
| Grhl1 | 195733 | Grhl1 | grainyhead like transcription factor 1 |
| Gm29371 | 102635830 | Gm29371 | predicted gene 29371 |
| Hbb-bs | 100503605 | Hbb-bs | "hemoglobin, beta adult s chain" |
| Cacna2d4 | 319734 | Cacna2d4 | "calcium channel, voltage-dependent, alpha 2/delta subunit 4" |
| 1810010H24Rik | 69066 | 1810010H24Rik | RIKEN cDNA 1810010H24 gene |
| Tmem100 | 67888 | Tmem100 | transmembrane protein 100 |
| Wnt2b | 22414 | Wnt2b | "wingless-type MMTV integration site family, member 2B" |
| Plpp1 | 19012 | Plpp1 | phospholipid phosphatase 1 |
| Fgfbp1 | 14181 | Fgfbp1 | fibroblast growth factor binding protein 1 |

| | | | |
|---|---|---|---|
| Zfp641 | 239652 | Zfp641 | zinc finger protein 641 |
| Pcdhac2 | 353237 | Pcdhac2 | "protocadherin alpha subfamily C, 2" |
| Arntl | 11865 | Arntl | aryl hydrocarbon receptor nuclear translocator-like |
| Ces1e | 13897 | Ces1e | carboxylesterase 1E |
| Gm35853 | 102639573 | Gm35853 | "predicted gene, 35853" |
| Csrnp1 | 215418 | Csrnp1 | cysteine-serine-rich nuclear protein 1 |
| Thrb | 21834 | Thrb | thyroid hormone receptor beta |
| Gm12840 | NA | NA | NA |
| Gfra1 | 14585 | Gfra1 | glial cell line derived neurotrophic factor family receptor alpha 1 |
| Pxdc1 | 66895 | Pxdc1 | PX domain containing 1 |
| Agtr1a | 11607 | Agtr1a | "angiotensin II receptor, type 1a" |
| Hba-a2 | 110257 | Hba-a2 | "hemoglobin alpha, adult chain 2" |
| Aldh1a2 | 19378 | Aldh1a2 | "aldehyde dehydrogenase family 1, subfamily A2" |
| Chrdl1 | 83453 | Chrdl1 | chordin-like 1 |
| Pir | 69656 | Pir | pirin |
| Cckar | 12425 | Cckar | cholecystokinin A receptor |
| Fam107a | 268709 | Fam107a | "family with sequence similarity 107, member A" |
| Pdk4 | 27273 | Pdk4 | "pyruvate dehydrogenase kinase, isoenzyme 4" |
| Igf2 | 16002 | Igf2 | insulin-like growth factor 2 |
| Scel | 64929 | Scel | sciellin |
| Map3k6 | 53608 | Map3k6 | mitogen-activated protein kinase kinase kinase 6 |
| Nr4a2 | 18227 | Nr4a2 | "nuclear receptor subfamily 4, group A, member 2" |
| Tfrc | 22042 | Tfrc | transferrin receptor |
| Klhl13 | 67455 | Klhl13 | kelch-like 13 |
| Gprc6a | 210198 | Gprc6a | "G protein-coupled receptor, family C, group 6, member A" |

| | | | |
|---|---|---|---|
| Slc38a5 | 209837 | Slc38a5 | "solute carrier family 38, member 5" |
| Enho | 69638 | Enho | energy homeostasis associated |
| Iyd | 70337 | Iyd | iodotyrosine deiodinase |
| Omd | 27047 | Omd | osteomodulin |
| Hba-a1 | 15122 | Hba-a1 | "hemoglobin alpha, adult chain 1" |
| Cdh13 | 12554 | Cdh13 | cadherin 13 |
| Gm9889 | 791369 | Gm9889 | predicted gene 9889 |
| Egr1 | 13653 | Egr1 | early growth response 1 |
| AC161438.1 | NA | NA | NA |
| Ccn1 | 16007 | Ccn1 | cellular communication network factor 1 |
| Gm2415 | NA | NA | NA |
| Dcdc2a | 195208 | Dcdc2a | doublecortin domain containing 2a |
| Pde9a | 18585 | Pde9a | phosphodiesterase 9A |
| Slco1a6 | 28254 | Slco1a6 | "solute carrier organic anion transporter family, member 1a6" |
| Tmtc2 | 278279 | Tmtc2 | transmembrane and tetratricopeptide repeat containing 2 |
| Gzma | 14938 | Gzma | granzyme A |
| D630045J12Rik | 330286 | D630045J12Rik | RIKEN cDNA D630045J12 gene |
| Aard | 239435 | Aard | alanine and arginine rich domain containing protein |
| Arhgef9 | 236915 | Arhgef9 | CDC42 guanine nucleotide exchange factor (GEF) 9 |
| 1200007C13Rik | 74119 | 1200007C13Rik | RIKEN cDNA 1200007C13 gene |
| Scube2 | 56788 | Scube2 | "signal peptide, CUB domain, EGF-like 2" |
| E030013I19Rik | 338535 | E030013I19Rik | RIKEN cDNA E030013I19 gene |
| Gabrp | 216643 | Gabrp | "gamma-aminobutyric acid (GABA) A receptor, pi" |
| Medag | 70717 | Medag | mesenteric estrogen dependent adipogenesis |
| Hbb-bt | 101488143 | Hbb-bt | "hemoglobin, beta adult t chain" |

| | | | |
|---|---|---|---|
| Itga2b | 16399 | Itga2b | integrin alpha 2b |
| Ces1f | 234564 | Ces1f | carboxylesterase 1F |
| Treml1 | 71326 | Treml1 | triggering receptor expressed on myeloid cells-like 1 |
| Ifitm10 | 320802 | Ifitm10 | interferon induced transmembrane protein 10 |
| Kif21a | 16564 | Kif21a | kinesin family member 21A |
| Ier2 | 15936 | Ier2 | immediate early response 2 |
| Gm49417 | NA | NA | NA |
| Des | 13346 | Des | desmin |
| Cytl1 | 231162 | Cytl1 | cytokine-like 1 |
| Scgb1c1 | 338417 | Scgb1c1 | "secretoglobin, family 1C, member 1" |
| Fam71f2 | 245884 | Fam71f2 | "family with sequence similarity 71, member F2" |
| Gm37516 | NA | NA | NA |
| Chst4 | 26887 | Chst4 | carbohydrate sulfotransferase 4 |
| Cpa3 | 12873 | Cpa3 | "carboxypeptidase A3, mast cell" |
| C1qtnf2 | 69183 | C1qtnf2 | C1q and tumor necrosis factor related protein 2 |
| Tpm2 | 22004 | Tpm2 | "tropomyosin 2, beta" |
| Fign | 60344 | Fign | fidgetin |
| Nqo1 | 18104 | Nqo1 | "NAD(P)H dehydrogenase, quinone 1" |
| Pclo | 26875 | Pclo | piccolo (presynaptic cytomatrix protein) |
| Dusp8 | 18218 | Dusp8 | dual specificity phosphatase 8 |
| Sptb | 20741 | Sptb | "spectrin beta, erythrocytic" |
| Fmn2 | 54418 | Fmn2 | formin 2 |
| Hif3a | 53417 | Hif3a | "hypoxia inducible factor 3, alpha subunit" |
| Cldn15 | 60363 | Cldn15 | claudin 15 |
| Adamtsl5 | 66548 | Adamtsl5 | ADAMTS-like 5 |

| | | | |
|---|---|---|---|
| Lmntd1 | 74071 | Lmntd1 | lamin tail domain containing 1 |
| B430010I23Rik | 78849 | B430010I23Rik | RIKEN cDNA B430010I23 gene |
| Alox12 | 11684 | Alox12 | arachidonate 12-lipoxygenase |
| Cyp4f15 | 106648 | Cyp4f15 | "cytochrome P450, family 4, subfamily f, polypeptide 15" |
| Pygm | 19309 | Pygm | muscle glycogen phosphorylase |
| Fosb | 14282 | Fosb | FBJ osteosarcoma oncogene B |
| Tent5b | 100342 | Tent5b | terminal nucleotidyltransferase 5B |
| Slc22a3 | 20519 | Slc22a3 | "solute carrier family 22 (organic cation transporter), member 3" |
| Lrriq1 | 74978 | Lrriq1 | leucine-rich repeats and IQ motif containing 1 |
| Gjb6 | 14623 | Gjb6 | "gap junction protein, beta 6" |
| Tbata | 65971 | Tbata | "thymus, brain and testes associated" |
| Esm1 | 71690 | Esm1 | endothelial cell-specific molecule 1 |
| Masp1 | 17174 | Masp1 | mannan-binding lectin serine peptidase 1 |
| Ctla2b | 13025 | Ctla2b | cytotoxic T lymphocyte-associated protein 2 beta |
| Gm21980 | NA | NA | NA |
| Ankrd63 | 383787 | Ankrd63 | ankyrin repeat domain 63 |
| Sntg1 | 71096 | Sntg1 | "syntrophin, gamma 1" |
| Gm30648 | 102632621 | Gm30648 | "predicted gene, 30648" |
| Adm | 11535 | Adm | adrenomedullin |
| Hrct1 | 100039781 | Hrct1 | histidine rich carboxyl terminus 1 |
| Gpx2 | 14776 | Gpx2 | glutathione peroxidase 2 |
| Evpl | 14027 | Evpl | envoplakin |
| Myh7 | 140781 | Myh7 | "myosin, heavy polypeptide 7, cardiac muscle, beta" |
| Enpp6 | 320981 | Enpp6 | ectonucleotide pyrophosphatase/phosphodiesterase 6 |
| Kcne4 | 57814 | Kcne4 | "potassium voltage-gated channel, Isk-related subfamily, gene 4" |

| | | | |
|---|---|---|---|
| Eno4 | 226265 | Eno4 | enolase 4 |
| Cpne5 | 240058 | Cpne5 | copine V |
| Bmpr1b | 12167 | Bmpr1b | "bone morphogenetic protein receptor, type 1B" |
| Iqcd | 75732 | Iqcd | IQ motif containing D |
| Fras1 | 231470 | Fras1 | Fraser extracellular matrix complex subunit 1 |
| Gnmt | 14711 | Gnmt | glycine N-methyltransferase |
| Ppp2r2b | 72930 | Ppp2r2b | "protein phosphatase 2, regulatory subunit B, beta" |
| Pcdh20 | 219257 | Pcdh20 | protocadherin 20 |
| Fabp1 | 14080 | Fabp1 | "fatty acid binding protein 1, liver" |
| Prkag3 | 241113 | Prkag3 | "protein kinase, AMP-activated, gamma 3 non-catalytic subunit" |
| Dnah12 | 110083 | Dnah12 | "dynein, axonemal, heavy chain 12" |
| Doc2b | 13447 | Doc2b | "double C2, beta" |
| Adam3 | 11497 | Adam3 | a disintegrin and metallopeptidase domain 3 (cyritestin) |
| Fat3 | 270120 | Fat3 | FAT atypical cadherin 3 |
| Wnt2 | 22413 | Wnt2 | "wingless-type MMTV integration site family, member 2" |
| Pifo | 100503311 | Pifo | primary cilia formation |
| Lrrc74b | 74685 | Lrrc74b | leucine rich repeat containing 74B |
| Asgr1 | 11889 | Asgr1 | asialoglycoprotein receptor 1 |
| Esr2 | 13983 | Esr2 | estrogen receptor 2 (beta) |
| Gm2115 | 100039239 | Gm2115 | predicted gene 2115 |
| Esyt3 | 272636 | Esyt3 | extended synaptotagmin-like protein 3 |
| Smoc2 | 64074 | Smoc2 | SPARC related modular calcium binding 2 |
| 8030442B05Rik | 77543 | 8030442B05Rik | RIKEN cDNA 8030442B05 gene |
| Chrm2 | 243764 | Chrm2 | "cholinergic receptor, muscarinic 2, cardiac" |
| Gm13387 | 329358 | Gm13387 | predicted gene 13387 |

| | | | |
|---|---|---|---|
| Ccdc171 | 320226 | Ccdc171 | coiled-coil domain containing 171 |
| Olfr1033 | 258571 | Olfr1033 | olfactory receptor 1033 |
| Itprid1 | 232016 | Itprid1 | ITPR interacting domain containing 1 |
| Arxes2 | 76976 | Arxes2 | adipocyte-related X-chromosome expressed sequence 2 |
| Triqk | 208820 | Triqk | triple QxxK/R motif containing |
| Gm30928 | 102632993 | Gm30928 | "predicted gene, 30928" |
| Ccdc187 | 329366 | Ccdc187 | coiled-coil domain containing 187 |
| Gm49767 | NA | NA | NA |
| Gm41442 | 105246099 | Gm41442 | "predicted gene, 41442" |
| Vsnl1 | 26950 | Vsnl1 | visinin-like 1 |
| Chst8 | 68947 | Chst8 | carbohydrate sulfotransferase 8 |
| Gal | 14419 | Gal | galanin and GMAP prepropeptide |
| Lyve1 | 114332 | Lyve1 | lymphatic vessel endothelial hyaluronan receptor 1 |
| Rgs9 | 19739 | Rgs9 | regulator of G-protein signaling 9 |
| Sybu | 319613 | Sybu | syntabulin (syntaxin-interacting) |
| Rd3 | 74023 | Rd3 | retinal degeneration 3 |
| Ccdc153 | 270150 | Ccdc153 | coiled-coil domain containing 153 |
| Upk3b | 100647 | Upk3b | uroplakin 3B |
| Gstm6 | 14867 | Gstm6 | "glutathione S-transferase, mu 6" |
| Stmnd1 | 380842 | Stmnd1 | stathmin domain containing 1 |
| Ppp1r14a | 68458 | Ppp1r14a | "protein phosphatase 1, regulatory inhibitor subunit 14A" |
| Mgl2 | 216864 | Mgl2 | macrophage galactose N-acetyl-galactosamine specific lectin 2 |
| Lrrc26 | 227618 | Lrrc26 | leucine rich repeat containing 26 |
| Gm15475 | NA | NA | NA |
| Acoxl | 74121 | Acoxl | acyl-Coenzyme A oxidase-like |

| | | | |
|---|---|---|---|
| Lrrc23 | 16977 | Lrrc23 | leucine rich repeat containing 23 |
| Fmo3 | 14262 | Fmo3 | flavin containing monooxygenase 3 |
| Hes2 | 15206 | Hes2 | hes family bHLH transcription factor 2 |
| Dnah1 | 110084 | Dnah1 | "dynein, axonemal, heavy chain 1" |
| Adra1a | 11549 | Adra1a | "adrenergic receptor, alpha 1a" |
| Adrb3 | 11556 | Adrb3 | "adrenergic receptor, beta 3" |
| Mum1l1 | NA | NA | NA |
| 9030622O22Rik | 100043820 | 9030622O22Rik | RIKEN cDNA 9030622O22 gene |
| 2610027K06Rik | 69909 | 2610027K06Rik | RIKEN cDNA 2610027K06 gene |
| Gstm7 | 68312 | Gstm7 | "glutathione S-transferase, mu 7" |
| Entpd3 | 215446 | Entpd3 | ectonucleoside triphosphate diphosphohydrolase 3 |
| Stk33 | 117229 | Stk33 | serine/threonine kinase 33 |
| Stc1 | 20855 | Stc1 | stanniocalcin 1 |
| Avpr1a | 54140 | Avpr1a | arginine vasopressin receptor 1A |
| A730046J19Rik | 319764 | A730046J19Rik | RIKEN cDNA A730046J19 gene |
| Fam183b | 75429 | Fam183b | "family with sequence similarity 183, member B" |
| Myh11 | 17880 | Myh11 | "myosin, heavy polypeptide 11, smooth muscle" |
| Nfe2l3 | 18025 | Nfe2l3 | "nuclear factor, erythroid derived 2, like 3" |
| Gm32014 | 102634431 | Gm32014 | "predicted gene, 32014" |
| Bex1 | 19716 | Bex1 | brain expressed X-linked 1 |
| Ncr1 | 17086 | Ncr1 | natural cytotoxicity triggering receptor 1 |
| 3300002A11Rik | 100503812 | 3300002A11Rik | RIKEN cDNA 3300002A11 gene |
| Mycbpap | 104601 | Mycbpap | MYCBP associated protein |
| Lrrc17 | 74511 | Lrrc17 | leucine rich repeat containing 17 |
| Gfpt2 | 14584 | Gfpt2 | glutamine fructose-6-phosphate transaminase 2 |

| | | | |
|---|---|---|---|
| Npr3 | 18162 | Npr3 | natriuretic peptide receptor 3 |
| Gabra3 | 14396 | Gabra3 | "gamma-aminobutyric acid (GABA) A receptor, subunit alpha 3" |
| B530045E10Rik | 320966 | B530045E10Rik | RIKEN cDNA B530045E10 gene |
| Arsj | 271970 | Arsj | arylsulfatase J |
| Angptl4 | 57875 | Angptl4 | angiopoietin-like 4 |
| Cadm2 | 239857 | Cadm2 | cell adhesion molecule 2 |
| Cebpd | 12609 | Cebpd | "CCAAT/enhancer binding protein (C/EBP), delta" |
| Nhlrc1 | 105193 | Nhlrc1 | NHL repeat containing 1 |
| Tro | 56191 | Tro | trophinin |
| Mpp3 | 13384 | Mpp3 | "membrane protein, palmitoylated 3 (MAGUK p55 subfamily member 3)" |
| Spon2 | 100689 | Spon2 | "spondin 2, extracellular matrix protein" |
| Spag16 | 66722 | Spag16 | sperm associated antigen 16 |
| Enkur | 71233 | Enkur | "enkurin, TRPC channel interacting protein" |
| Pdlim3 | 53318 | Pdlim3 | PDZ and LIM domain 3 |
| Tecrl | 243078 | Tecrl | "trans-2,3-enoyl-CoA reductase-like" |
| Tbce | 70430 | Tbce | tubulin-specific chaperone E |
| Adcy1 | 432530 | Adcy1 | adenylate cyclase 1 |
| Slc22a12 | 20521 | Slc22a12 | "solute carrier family 22 (organic anion/cation transporter), member 12" |
| Gm36283 | 102640148 | Gm36283 | "predicted gene, 36283" |
| 5033421B08Rik | 75990 | 5033421B08Rik | RIKEN cDNA 5033421B08 gene |
| H2-Q1 | 15006 | H2-Q1 | "histocompatibility 2, Q region locus 1" |
| Gm11730 | 102637059 | Gm11730 | predicted gene 11730 |
| Best3 | 382427 | Best3 | bestrophin 3 |
| Ldlrad1 | 546840 | Ldlrad1 | low density lipoprotein receptor class A domain containing 1 |
| Gm973 | 381260 | Gm973 | predicted gene 973 |

| | | | |
|---|---|---|---|
| Cfap161 | 75556 | Cfap161 | cilia and flagella associated protein 161 |
| Ttc29 | 73301 | Ttc29 | tetratricopeptide repeat domain 29 |
| Chrm3 | 12671 | Chrm3 | "cholinergic receptor, muscarinic 3, cardiac" |
| Ddit4 | 74747 | Ddit4 | DNA-damage-inducible transcript 4 |
| Hoga1 | 67432 | Hoga1 | 4-hydroxy-2-oxoglutarate aldolase 1 |
| Atp6v0a4 | 140494 | Atp6v0a4 | "ATPase, H+ transporting, lysosomal V0 subunit A4" |
| Acsm1 | 117147 | Acsm1 | acyl-CoA synthetase medium-chain family member 1 |
| AA414992 | 102402 | AA414992 | expressed sequence AA414992 |
| Ddc | 13195 | Ddc | dopa decarboxylase |
| H60c | 670558 | H60c | histocompatibility 60c |
| Acaa1b | 235674 | Acaa1b | acetyl-Coenzyme A acyltransferase 1B |
| Rps6ka6 | 67071 | Rps6ka6 | ribosomal protein S6 kinase polypeptide 6 |
| Prom2 | 192212 | Prom2 | prominin 2 |
| Kif27 | 75050 | Kif27 | kinesin family member 27 |
| Gp5 | 14729 | Gp5 | glycoprotein 5 (platelet) |
| Btg2 | 12227 | Btg2 | BTG anti-proliferation factor 2 |
| 4933406B17Rik | 71040 | 4933406B17Rik | RIKEN cDNA 4933406B17 gene |
| Ntf3 | 18205 | Ntf3 | neurotrophin 3 |
| Tmprss13 | 214531 | Tmprss13 | "transmembrane protease, serine 13" |
| Cldn8 | 54420 | Cldn8 | claudin 8 |
| Gp9 | 54368 | Gp9 | glycoprotein 9 (platelet) |
| Armh4 | 67419 | Armh4 | armadillo-like helical domain containing 4 |
| AC138228.3 | NA | NA | NA |
| Dync1i1 | 13426 | Dync1i1 | dynein cytoplasmic 1 intermediate chain 1 |
| Acsm3 | 20216 | Acsm3 | acyl-CoA synthetase medium-chain family member 3 |

| | | | |
|---|---|---|---|
| Scn5a | 20271 | Scn5a | "sodium channel, voltage-gated, type V, alpha" |
| Fam92b | 436062 | Fam92b | "family with sequence similarity 92, member B" |
| Gm24327 | NA | NA | NA |
| Pcdhga3 | 93711 | Pcdhga3 | "protocadherin gamma subfamily A, 3" |
| C130046K22Rik | 399609 | C130046K22Rik | RIKEN cDNA C130046K22 gene |
| Cfap206 | 69329 | Cfap206 | cilia and flagella associated protein 206 |
| Armc3 | 70882 | Armc3 | armadillo repeat containing 3 |
| Rbp4 | 19662 | Rbp4 | "retinol binding protein 4, plasma" |
| Pcnx2 | 270109 | Pcnx2 | pecanex homolog 2 |
| Spa17 | 20686 | Spa17 | sperm autoantigenic protein 17 |
| AC122252.3 | NA | NA | NA |
| Acta2 | 11475 | Acta2 | "actin, alpha 2, smooth muscle, aorta" |
| Carmn | 328968 | Carmn | cardiac mesoderm enhancer-associated non-coding RNA |
| D830026I12Rik | 319682 | D830026I12Rik | RIKEN cDNA D830026I12 gene |
| Crym | 12971 | Crym | "crystallin, mu" |
| Tekt4 | 71840 | Tekt4 | tektin 4 |
| Aldh3a1 | 11670 | Aldh3a1 | "aldehyde dehydrogenase family 3, subfamily A1" |
| Tnr | 21960 | Tnr | tenascin R |
| Upk1a | 109637 | Upk1a | uroplakin 1A |
| Wt1 | 22431 | Wt1 | Wilms tumor 1 homolog |
| Gm17473 | 100503767 | Gm17473 | "predicted gene, 17473" |
| Galnt13 | 271786 | Galnt13 | polypeptide N-acetylgalactosaminyltransferase 13 |
| BC030870 | NA | NA | NA |
| Lrtm2 | 211187 | Lrtm2 | leucine-rich repeats and transmembrane domains 2 |
| Ppp4r4 | 74521 | Ppp4r4 | "protein phosphatase 4, regulatory subunit 4" |

| | | | |
|---|---|---|---|
| Syt17 | 110058 | Syt17 | synaptotagmin XVII |
| Slc16a11 | 216867 | Slc16a11 | "solute carrier family 16 (monocarboxylic acid transporters), member 11" |
| Ppargc1a | 19017 | Ppargc1a | "peroxisome proliferative activated receptor, gamma, coactivator 1 alpha" |
| Lpar4 | 78134 | Lpar4 | lysophosphatidic acid receptor 4 |
| Ppp2r2c | 269643 | Ppp2r2c | "protein phosphatase 2, regulatory subunit B, gamma" |
| 2010001K21Rik | 69829 | 2010001K21Rik | RIKEN cDNA 2010001K21 gene |
| Gm14226 | 100462662 | Gm14226 | predicted gene 14226 |
| Il22ra2 | 237310 | Il22ra2 | "interleukin 22 receptor, alpha 2" |
| Gm10309 | 100038711 | Gm10309 | predicted gene 10309 |
| Ccdc170 | 100504234 | Ccdc170 | coiled-coil domain containing 170 |
| Rgs22 | 626596 | Rgs22 | regulator of G-protein signalling 22 |
| Ccdc162 | 75973 | Ccdc162 | coiled-coil domain containing 162 |
| Gsta2 | 14858 | Gsta2 | "glutathione S-transferase, alpha 2 (Yc2)" |
| S1pr5 | 94226 | S1pr5 | sphingosine-1-phosphate receptor 5 |
| Gm13582 | 100038493 | Gm13582 | predicted gene 13582 |
| Ccdc85a | 216613 | Ccdc85a | coiled-coil domain containing 85A |
| Ccdc180 | 381522 | Ccdc180 | coiled-coil domain containing 180 |
| Cbs | 12411 | Cbs | cystathionine beta-synthase |
| Ccdc184 | 239650 | Ccdc184 | coiled-coil domain containing 184 |
| Tbx3os1 | 100039542 | Tbx3os1 | "T-box 3, opposite strand 1" |
| Lnx1 | 16924 | Lnx1 | ligand of numb-protein X 1 |
| Ccdc3 | 74186 | Ccdc3 | coiled-coil domain containing 3 |
| Cntfr | 12804 | Cntfr | ciliary neurotrophic factor receptor |
| Nme9 | 623534 | Nme9 | NME/NM23 family member 9 |
| 4833427G06Rik | 235345 | 4833427G06Rik | RIKEN cDNA 4833427G06 gene |

| | | | |
|---|---|---|---|
| Iqca | 74918 | Iqca | IQ motif containing with AAA domain |
| Tat | 234724 | Tat | tyrosine aminotransferase |
| Pkhd1l1 | 192190 | Pkhd1l1 | polycystic kidney and hepatic disease 1-like 1 |
| Gm5144 | 381002 | Gm5144 | predicted gene 5144 |
| Gsg1l | 269994 | Gsg1l | GSG1-like |
| Dusp1 | 19252 | Dusp1 | dual specificity phosphatase 1 |
| Acot1 | 26897 | Acot1 | acyl-CoA thioesterase 1 |
| Gm9910 | 791371 | Gm9910 | predicted gene 9910 |
| Pcdhga8 | 93716 | Pcdhga8 | "protocadherin gamma subfamily A, 8" |
| Map3k7cl | 224419 | Map3k7cl | Map3k7 C-terminal like |
| Sh3gl2 | 20404 | Sh3gl2 | SH3-domain GRB2-like 2 |
| Wfdc6b | 433502 | Wfdc6b | WAP four-disulfide core domain 6B |
| Tmem40 | 94346 | Tmem40 | transmembrane protein 40 |
| Zfp36 | 22695 | Zfp36 | zinc finger protein 36 |
| Gm6634 | 625901 | Gm6634 | predicted gene 6634 |
| Lrrn4 | 320974 | Lrrn4 | leucine rich repeat neuronal 4 |
| Atp13a4 | 224079 | Atp13a4 | ATPase type 13A4 |
| Azgp1 | 12007 | Azgp1 | "alpha-2-glycoprotein 1, zinc" |
| 1810008I18Rik | 100503969 | 1810008I18Rik | RIKEN cDNA 1810008I18 gene |
| Mfsd2b | 432628 | Mfsd2b | major facilitator superfamily domain containing 2B |
| Pik3c2g | 18705 | Pik3c2g | phosphatidylinositol-4-phosphate 3-kinase catalytic subunit type 2 gamma |
| mt-Nd4l | 17720 | mt-Nd4l | "NADH dehydrogenase 4L, mitochondrial" |
| Ttc16 | 338348 | Ttc16 | tetratricopeptide repeat domain 16 |
| Selenbp2 | 20342 | Selenbp2 | selenium binding protein 2 |
| Gm48228 | NA | NA | NA |

| | | | |
|---|---|---|---|
| Abra | 223513 | Abra | actin-binding Rho activating protein |
| Dnah7c | 100101919 | Dnah7c | "dynein, axonemal, heavy chain 7C" |
| Platr21 | 78526 | Platr21 | pluripotency associated transcript 21 |
| Aspa | 11484 | Aspa | aspartoacylase |
| Cd209b | 69165 | Cd209b | CD209b antigen |
| Kcna1 | 16485 | Kcna1 | "potassium voltage-gated channel, shaker-related subfamily, member 1" |
| Catip | 241112 | Catip | ciliogenesis associated TTC17 interacting protein |
| Rptn | 20129 | Rptn | repetin |
| A730049H05Rik | 74516 | A730049H05Rik | RIKEN cDNA A730049H05 gene |
| Ribc1 | 66611 | Ribc1 | RIB43A domain with coiled-coils 1 |
| D630029K05Rik | 103175 | D630029K05Rik | RIKEN cDNA D630029K05 gene |
| Skint3 | 195564 | Skint3 | selection and upkeep of intraepithelial T cells 3 |
| Fez1 | 235180 | Fez1 | fasciculation and elongation protein zeta 1 (zygin I) |
| Chrd | 12667 | Chrd | chordin |
| Slc12a5 | 57138 | Slc12a5 | "solute carrier family 12, member 5" |
| Ngb | 64242 | Ngb | neuroglobin |
| Alb | 11657 | Alb | albumin |
| Gm19522 | 100503040 | Gm19522 | "predicted gene, 19522" |
| Sntn | 218739 | Sntn | "sentan, cilia apical structure protein" |
| Agr3 | 403205 | Agr3 | anterior gradient 3 |
| Ttc34 | 242800 | Ttc34 | tetratricopeptide repeat domain 34 |
| Cfap58 | 381229 | Cfap58 | cilia and flagella associated protein 58 |
| Myrip | 245049 | Myrip | myosin VIIA and Rab interacting protein |
| Gm15541 | 105242887 | Gm15541 | predicted gene 15541 |
| Gp6 | 243816 | Gp6 | glycoprotein 6 (platelet) |

| | | | |
|---|---|---|---|
| Grip1 | 74053 | Grip1 | glutamate receptor interacting protein 1 |
| Gm26671 | 102635136 | Gm26671 | "predicted gene, 26671" |
| Rwdd3 | 66568 | Rwdd3 | RWD domain containing 3 |
| Crocc2 | 381284 | Crocc2 | "ciliary rootlet coiled-coil, rootletin family member 2" |
| Fam178b | 381337 | Fam178b | "family with sequence similarity 178, member B" |
| Slc7a15 | 328059 | Slc7a15 | "solute carrier family 7 (cationic amino acid transporter, y+ system), member 15" |
| Cfap54 | 380654 | Cfap54 | cilia and flagella associated protein 54 |
| Cttnbp2 | 30785 | Cttnbp2 | cortactin binding protein 2 |
| Ptn | 19242 | Ptn | pleiotrophin |
| 2610203C22Rik | 72481 | 2610203C22Rik | RIKEN cDNA 2610203C22 gene |
| Gmnc | 239789 | Gmnc | geminin coiled-coil domain containing |
| Thbs1 | 21825 | Thbs1 | thrombospondin 1 |
| Myl9 | 98932 | Myl9 | "myosin, light polypeptide 9, regulatory" |
| Rnf112 | 22671 | Rnf112 | ring finger protein 112 |
| Lgals12 | 56072 | Lgals12 | "lectin, galactose binding, soluble 12" |
| Gstp2 | 14869 | Gstp2 | "glutathione S-transferase, pi 2" |
| Slc10a5 | 241877 | Slc10a5 | "solute carrier family 10 (sodium/bile acid cotransporter family), member 5" |
| Ttll6 | 237930 | Ttll6 | "tubulin tyrosine ligase-like family, member 6" |
| Snx31 | 66696 | Snx31 | sorting nexin 31 |
| Actg2 | 11468 | Actg2 | "actin, gamma 2, smooth muscle, enteric" |
| Odf3b | 70113 | Odf3b | outer dense fiber of sperm tails 3B |
| Gpr179 | 217143 | Gpr179 | G protein-coupled receptor 179 |
| F5 | 14067 | F5 | coagulation factor V |
| Itih2 | 16425 | Itih2 | "inter-alpha trypsin inhibitor, heavy chain 2" |
| Fsip1 | 71313 | Fsip1 | fibrous sheath-interacting protein 1 |

| | | | |
|---|---|---|---|
| Ttc6 | 70846 | Ttc6 | tetratricopeptide repeat domain 6 |
| Cd300e | 217306 | Cd300e | CD300E molecule |
| Cdh19 | 227485 | Cdh19 | "cadherin 19, type 2" |
| Pknox2 | 208076 | Pknox2 | Pbx/knotted 1 homeobox 2 |
| Cntn1 | 12805 | Cntn1 | contactin 1 |
| Folh1 | 53320 | Folh1 | folate hydrolase 1 |
| Slc25a34 | 384071 | Slc25a34 | "solute carrier family 25, member 34" |
| Itgad | 381924 | Itgad | "integrin, alpha D" |
| AC174800.1 | NA | NA | NA |
| Galnt5 | 241391 | Galnt5 | polypeptide N-acetylgalactosaminyltransferase 5 |
| Me3 | 109264 | Me3 | "malic enzyme 3, NADP(+)-dependent, mitochondrial" |
| Ccdc81 | 70884 | Ccdc81 | coiled-coil domain containing 81 |
| Gm45774 | NA | NA | NA |
| Rbfox3 | 52897 | Rbfox3 | "RNA binding protein, fox-1 homolog (C. elegans) 3" |
| Gp1ba | 14723 | Gp1ba | "glycoprotein 1b, alpha polypeptide" |
| Agt | 11606 | Agt | "angiotensinogen (serpin peptidase inhibitor, clade A, member 8)" |
| Iqcn | 637079 | Iqcn | IQ motif containing N |
| 6820408C15Rik | 228778 | 6820408C15Rik | RIKEN cDNA 6820408C15 gene |
| Rspo4 | 228770 | Rspo4 | R-spondin 4 |
| Clec12b | 71183 | Clec12b | "C-type lectin domain family 12, member B" |
| Gm7173 | NA | NA | NA |
| Btg4 | 56057 | Btg4 | BTG anti-proliferation factor 4 |
| Gm41253 | 105245865 | Gm41253 | "predicted gene, 41253" |
| Gm44899 | NA | NA | NA |
| A530013C23Rik | 329562 | A530013C23Rik | RIKEN cDNA A530013C23 gene |

| | | | |
|---|---|---|---|
| Nr4a3 | 18124 | Nr4a3 | "nuclear receptor subfamily 4, group A, member 3" |
| A730020E08Rik | 100504262 | A730020E08Rik | RIKEN cDNA A730020E08 gene |
| P2rx1 | 18436 | P2rx1 | "purinergic receptor P2X, ligand-gated ion channel, 1" |
| CT010496.1 | NA | NA | NA |
| C430049B03Rik | 72575 | C430049B03Rik | RIKEN cDNA C430049B03 gene |
| Gabre | 14404 | Gabre | "gamma-aminobutyric acid (GABA) A receptor, subunit epsilon" |
| Dab1 | 13131 | Dab1 | disabled 1 |
| Ppil6 | 73075 | Ppil6 | peptidylprolyl isomerase (cyclophilin)-like 6 |
| Fank1 | 66930 | Fank1 | fibronectin type 3 and ankyrin repeat domains 1 |
| Nrgn | 64011 | Nrgn | neurogranin |
| Trmt9b | 319582 | Trmt9b | tRNA methyltransferase 9B |
| Ect2l | 100045792 | Ect2l | epithelial cell transforming sequence 2 oncogene-like |
| Ccl17 | 20295 | Ccl17 | chemokine (C-C motif) ligand 17 |
| Rasd1 | 19416 | Rasd1 | "RAS, dexamethasone-induced 1" |
| Klra8 | 16639 | Klra8 | "killer cell lectin-like receptor, subfamily A, member 8" |
| Gm4777 | 212399 | Gm4777 | predicted gene 4777 |
| Gm28040 | NA | NA | NA |
| Klra4 | 16635 | Klra4 | "killer cell lectin-like receptor, subfamily A, member 4" |
| Fendrr | 68790 | Fendrr | Foxf1 adjacent non-coding developmental regulatory RNA |
| Cnksr2 | 245684 | Cnksr2 | connector enhancer of kinase suppressor of Ras 2 |
| Gm26841 | NA | NA | NA |
| Gm4262 | 100043147 | Gm4262 | predicted gene 4262 |
| Sbspon | 226866 | Sbspon | "somatomedin B and thrombospondin, type 1 domain containing" |
| Shisa9 | 72555 | Shisa9 | shisa family member 9 |
| Cnn1 | 12797 | Cnn1 | calponin 1 |

| | | | |
|---|---|---|---|
| 4933406C10Rik | 74076 | 4933406C10Rik | RIKEN cDNA 4933406C10 gene |
| 1700001C02Rik | NA | NA | NA |
| Zmynd12 | 332934 | Zmynd12 | "zinc finger, MYND domain containing 12" |
| 1700095J03Rik | 74293 | 1700095J03Rik | RIKEN cDNA 1700095J03 gene |
| Riiad1 | 66353 | Riiad1 | regulatory subunit of type II PKA R-subunit (RIIa) domain containing 1 |
| Gm14421 | 102639713 | Gm14421 | predicted gene 14421 |
| Kif6 | 319991 | Kif6 | kinesin family member 6 |
| 2010204K13Rik | 68355 | 2010204K13Rik | RIKEN cDNA 2010204K13 gene |
| 4933403O08Rik | 71030 | 4933403O08Rik | RIKEN cDNA 4933403O08 gene |
| A830018L16Rik | 320492 | A830018L16Rik | RIKEN cDNA A830018L16 gene |
| Sgcg | 24053 | Sgcg | "sarcoglycan, gamma (dystrophin-associated glycoprotein)" |
| Adgrv1 | 110789 | Adgrv1 | adhesion G protein-coupled receptor V1 |
| 9130230L23Rik | 231253 | 9130230L23Rik | RIKEN cDNA 9130230L23 gene |
| Grik4 | 110637 | Grik4 | "glutamate receptor, ionotropic, kainate 4" |
| Crispld1 | 83691 | Crispld1 | cysteine-rich secretory protein LCCL domain containing 1 |
| Dnah6 | 330355 | Dnah6 | "dynein, axonemal, heavy chain 6" |
| Gdf2 | 12165 | Gdf2 | growth differentiation factor 2 |
| Gm14416 | 100504247 | Gm14416 | predicted gene 14416 |
| AC096777.1 | NA | NA | NA |
| Eprn | 432777 | Eprn | "ephemeron, early developmental lncRNA" |
| 9330175M20Rik | 442821 | 9330175M20Rik | RIKEN cDNA 9330175M20 gene |
| Gm13816 | 105244235 | Gm13816 | predicted gene 13816 |
| Angpt4 | 11602 | Angpt4 | angiopoietin 4 |
| Gpr17 | 574402 | Gpr17 | G protein-coupled receptor 17 |
| Zdbf2 | 73884 | Zdbf2 | "zinc finger, DBF-type containing 2" |

| | | | |
|---|---|---|---|
| Gm26878 | 102636330 | Gm26878 | "predicted gene, 26878" |
| Lrrc10b | 278795 | Lrrc10b | leucine rich repeat containing 10B |
| Tsnaxip1 | 72236 | Tsnaxip1 | translin-associated factor X (Tsnax) interacting protein 1 |
| 1810062G17Rik | 72282 | 1810062G17Rik | RIKEN cDNA 1810062G17 gene |
| Mcpt4 | 17227 | Mcpt4 | mast cell protease 4 |
| Ppp1r1a | 58200 | Ppp1r1a | "protein phosphatase 1, regulatory inhibitor subunit 1A" |
| Zbtb16 | 235320 | Zbtb16 | zinc finger and BTB domain containing 16 |
| Ggt1 | 14598 | Ggt1 | gamma-glutamyltransferase 1 |
| Gm9899 | 100502829 | Gm9899 | predicted gene 9899 |
| Smkr-ps | 76419 | Smkr-ps | "smal lysine rich protein 1, pseudogene" |
| Plp1 | 18823 | Plp1 | proteolipid protein (myelin) 1 |
| Cyp2c23 | 226143 | Cyp2c23 | "cytochrome P450, family 2, subfamily c, polypeptide 23" |
| Gm47732 | NA | NA | NA |
| Hoxaas3 | 72628 | Hoxaas3 | Hoxa cluster antisense RNA 3 |
| 4930443O20Rik | 74687 | 4930443O20Rik | RIKEN cDNA 4930443O20 gene |
| Armc4 | 74934 | Armc4 | armadillo repeat containing 4 |
| Fbxo16 | 50759 | Fbxo16 | F-box protein 16 |
| Gm281 | 238939 | Gm281 | predicted gene 281 |
| Tstd1 | 226654 | Tstd1 | thiosulfate sulfurtransferase (rhodanese)-like domain containing 1 |
| Daw1 | 71227 | Daw1 | dynein assembly factor with WDR repeat domains 1 |
| Ccdc152 | 100039139 | Ccdc152 | coiled-coil domain containing 152 |
| Lrrc6 | 54562 | Lrrc6 | leucine rich repeat containing 6 (testis) |
| Clec4g | 75863 | Clec4g | "C-type lectin domain family 4, member g" |
| Gm45826 | NA | NA | NA |
| Tacr2 | 21337 | Tacr2 | tachykinin receptor 2 |

| | | | |
|---|---|---|---|
| Gm7008 | 629820 | Gm7008 | predicted gene 7008 |
| Platr22 | 100503474 | Platr22 | pluripotency associated transcript 22 |
| Gm48957 | NA | NA | NA |
| Gm2274 | 100039503 | Gm2274 | predicted gene 2274 |
| Rapgefl1 | 268480 | Rapgefl1 | Rap guanine nucleotide exchange factor (GEF)-like 1 |
| Gm15958 | 100503546 | Gm15958 | predicted gene 15958 |
| Gm12709 | 100504717 | Gm12709 | predicted gene 12709 |
| Fmo4 | 226564 | Fmo4 | flavin containing monooxygenase 4 |
| Mlxipl | 58805 | Mlxipl | MLX interacting protein-like |
| Nap1l5 | 58243 | Nap1l5 | nucleosome assembly protein 1-like 5 |
| Dnaaf1 | 68270 | Dnaaf1 | "dynein, axonemal assembly factor 1" |
| Dio2 | 13371 | Dio2 | "deiodinase, iodothyronine, type II" |
| Dnm3os | 474332 | Dnm3os | "dynamin 3, opposite strand" |
| Gm15657 | NA | NA | NA |
| Plin4 | 57435 | Plin4 | perilipin 4 |
| Gm10030 | 791282 | Gm10030 | predicted gene 10030 |
| 1700013F07Rik | 75504 | 1700013F07Rik | RIKEN cDNA 1700013F07 gene |
| Slc47a1 | 67473 | Slc47a1 | "solute carrier family 47, member 1" |
| Olfr750 | 404319 | Olfr750 | olfactory receptor 750 |
| Gm36169 | 102639986 | Gm36169 | "predicted gene, 36169" |
| Cfap44 | 212517 | Cfap44 | cilia and flagella associated protein 44 |
| T2 | 21331 | T2 | brachyury 2 |
| Lrrc4c | 241568 | Lrrc4c | leucine rich repeat containing 4C |
| Gm16853 | 330902 | Gm16853 | "predicted gene, 16853" |
| Gm29103 | NA | NA | NA |

| | | | |
|---|---|---|---|
| Atp2c2 | 69047 | Atp2c2 | "ATPase, Ca++ transporting, type 2C, member 2" |
| Gm35330 | 102638869 | Gm35330 | "predicted gene, 35330" |
| Col20a1 | 73368 | Col20a1 | "collagen, type XX, alpha 1" |
| Gm11827 | 100503518 | Gm11827 | predicted gene 11827 |
| Gcat | 26912 | Gcat | glycine C-acetyltransferase (2-amino-3-ketobutyrate-coenzyme A ligase) |
| St6galnac5 | 26938 | St6galnac5 | "ST6 (alpha-N-acetyl-neuraminyl-2,3-beta-galactosyl-1,3)-N-acetylgalactosaminide alpha-2,6-sialyltransferase 5" |
| E330018M18Rik | 414120 | E330018M18Rik | RIKEN cDNA E330018M18 gene |
| Hs3st6 | 328779 | Hs3st6 | heparan sulfate (glucosamine) 3-O-sulfotransferase 6 |
| Zmat4 | 320158 | Zmat4 | "zinc finger, matrin type 4" |
| G930009F23Rik | 100038726 | G930009F23Rik | RIKEN cDNA G930009F23 gene |
| Pkdrej | 18766 | Pkdrej | polycystin (PKD) family receptor for egg jelly |
| Fos | 14281 | Fos | FBJ osteosarcoma oncogene |
| Pcdhac1 | 353236 | Pcdhac1 | "protocadherin alpha subfamily C, 1" |
| Myocd | 214384 | Myocd | myocardin |
| Kctd8 | 243043 | Kctd8 | potassium channel tetramerisation domain containing 8 |
| A330023F24Rik | 320977 | A330023F24Rik | RIKEN cDNA A330023F24 gene |
| Gm9951 | 791287 | Gm9951 | predicted gene 9951 |
| Gm10699 | 100038645 | Gm10699 | predicted gene 10699 |
| Gpr88 | 64378 | Gpr88 | G-protein coupled receptor 88 |
| Gm15883 | 102632321 | Gm15883 | predicted gene 15883 |
| Gm13597 | 100039683 | Gm13597 | predicted gene 13597 |
| Gm36660 | 102640645 | Gm36660 | "predicted gene, 36660" |
| Gm10600 | 100042472 | Gm10600 | predicted gene 10600 |
| Gm9968 | 791329 | Gm9968 | predicted gene 9968 |

| | | | |
|---|---|---|---|
| 4732491K20Rik | 224523 | 4732491K20Rik | RIKEN cDNA 4732491K20 gene |
| Ccdc74a | 72315 | Ccdc74a | coiled-coil domain containing 74A |
| 1700001L19Rik | 69315 | 1700001L19Rik | RIKEN cDNA 1700001L19 gene |
| Zim1 | 22776 | Zim1 | "zinc finger, imprinted 1" |
| 2010310C07Rik | 72131 | 2010310C07Rik | RIKEN cDNA 2010310C07 gene |
| Gm10741 | 102642038 | Gm10741 | predicted gene 10741 |
| Gm19299 | 100502636 | Gm19299 | "predicted gene, 19299" |
| Kcnma1 | 16531 | Kcnma1 | "potassium large conductance calcium-activated channel, subfamily M, alpha member 1" |
| Ly6g6c | 68468 | Ly6g6c | "lymphocyte antigen 6 complex, locus G6C" |
| Grik2 | 14806 | Grik2 | "glutamate receptor, ionotropic, kainate 2 (beta 2)" |
| Adra1b | 11548 | Adra1b | "adrenergic receptor, alpha 1b" |
| Hydin | 244653 | Hydin | "HYDIN, axonemal central pair apparatus protein" |
| Rem2 | 140743 | Rem2 | rad and gem related GTP binding protein 2 |
| Gm49395 | NA | NA | NA |
| Atp13a5 | 268878 | Atp13a5 | ATPase type 13A5 |
| Ppfia2 | 327814 | Ppfia2 | "protein tyrosine phosphatase, receptor type, f polypeptide (PTPRF), interacting protein (liprin), alpha 2" |
| Unc79 | 217843 | Unc79 | unc-79 homolog |
| Gm49704 | NA | NA | NA |
| Serpinb1b | 282663 | Serpinb1b | "serine (or cysteine) peptidase inhibitor, clade B, member 1b" |
| Cxxc4 | 319478 | Cxxc4 | CXXC finger 4 |
| Sh3bgr | 50795 | Sh3bgr | SH3-binding domain glutamic acid-rich protein |
| Ugt8a | 22239 | Ugt8a | UDP galactosyltransferase 8A |
| Igsf11 | 207683 | Igsf11 | "immunoglobulin superfamily, member 11" |
| Gm13546 | 100042926 | Gm13546 | predicted gene 13546 |

| | | | |
|---|---|---|---|
| 4930550C14Rik | 75311 | 4930550C14Rik | RIKEN cDNA 4930550C14 gene |
| Ebf2 | 13592 | Ebf2 | early B cell factor 2 |
| Gm16263 | 102638835 | Gm16263 | predicted gene 16263 |
| Gm14473 | 668982 | Gm14473 | predicted gene 14473 |
| Gm45470 | 108167529 | Gm45470 | predicted gene 45470 |
| Cpb1 | 76703 | Cpb1 | carboxypeptidase B1 (tissue) |
| 1700012B09Rik | 69325 | 1700012B09Rik | RIKEN cDNA 1700012B09 gene |
| Abca14 | 67928 | Abca14 | "ATP-binding cassette, sub-family A (ABC1), member 14" |
| Myl3 | 17897 | Myl3 | "myosin, light polypeptide 3" |
| Tpsb2 | 17229 | Tpsb2 | tryptase beta 2 |
| Hepacam | 72927 | Hepacam | hepatocyte cell adhesion molecule |
| 2810030D12Rik | 78335 | 2810030D12Rik | RIKEN cDNA 2810030D12 gene |
| 1700028P14Rik | 67483 | 1700028P14Rik | RIKEN cDNA 1700028P14 gene |
| Timd4 | 276891 | Timd4 | T cell immunoglobulin and mucin domain containing 4 |
| C030037D09Rik | 193280 | C030037D09Rik | RIKEN cDNA C030037D09 gene |
| CR974586.1 | NA | NA | NA |
| Bves | 23828 | Bves | blood vessel epicardial substance |
| Gm43618 | NA | NA | NA |
| Gm16499 | 100049166 | Gm16499 | predicted gene 16499 |
| Gm38708 | 105242443 | Gm38708 | "predicted gene, 38708" |
| Kcnd3 | 56543 | Kcnd3 | "potassium voltage-gated channel, Shal-related family, member 3" |
| Dnah10 | 56087 | Dnah10 | "dynein, axonemal, heavy chain 10" |
| Col6a5 | 665033 | Col6a5 | "collagen, type VI, alpha 5" |
| Mpl | 17480 | Mpl | myeloproliferative leukemia virus oncogene |
| Gm44421 | NA | NA | NA |

| | | | |
|---|---|---|---|
| Draxin | 70433 | Draxin | dorsal inhibitory axon guidance protein |
| Il11 | 16156 | Il11 | interleukin 11 |
| Gpr3 | 14748 | Gpr3 | G-protein coupled receptor 3 |
| Dner | 227325 | Dner | delta/notch-like EGF repeat containing |
| Ankef1 | 319196 | Ankef1 | ankyrin repeat and EF-hand domain containing 1 |
| D230017M19Rik | 320933 | D230017M19Rik | RIKEN cDNA D230017M19 gene |
| Gfra3 | 14587 | Gfra3 | glial cell line derived neurotrophic factor family receptor alpha 3 |
| Fam205c | 277773 | Fam205c | "family with sequence similarity 205, member C" |
| Caps2 | 353025 | Caps2 | calcyphosphine 2 |
| Lgals2 | 107753 | Lgals2 | "lectin, galactose-binding, soluble 2" |
| Muc16 | 73732 | Muc16 | mucin 16 |
| Ccdc42 | 276920 | Ccdc42 | coiled-coil domain containing 42 |
| Gm42791 | NA | NA | NA |
| Gm49701 | NA | NA | NA |
| Rnft2 | 269695 | Rnft2 | "ring finger protein, transmembrane 2" |
| 6030407O03Rik | 109198 | 6030407O03Rik | RIKEN cDNA 6030407O03 gene |
| Pi16 | 74116 | Pi16 | peptidase inhibitor 16 |
| Il12a | 16159 | Il12a | interleukin 12a |
| Ccdc150 | 78016 | Ccdc150 | coiled-coil domain containing 150 |
| Fbxo47 | 72973 | Fbxo47 | F-box protein 47 |
| Serpinb9b | 20706 | Serpinb9b | "serine (or cysteine) peptidase inhibitor, clade B, member 9b" |
| Cox8b | 12869 | Cox8b | cytochrome c oxidase subunit 8B |
| 5930403N24Rik | 100040846 | 5930403N24Rik | RIKEN cDNA 5930403N24 gene |
| Tmem255a | 245386 | Tmem255a | transmembrane protein 255A |
| Slc4a1 | 20533 | Slc4a1 | "solute carrier family 4 (anion exchanger), member 1" |

| | | | |
|---|---|---|---|
| Gm13655 | 667828 | Gm13655 | predicted gene 13655 |
| Serpinb10 | 241197 | Serpinb10 | "serine (or cysteine) peptidase inhibitor, clade B (ovalbumin), member 10" |
| Slc7a14 | 241919 | Slc7a14 | "solute carrier family 7 (cationic amino acid transporter, y+ system), member 14" |
| Tnnt1 | 21955 | Tnnt1 | "troponin T1, skeletal, slow" |
| Ncam2 | 17968 | Ncam2 | neural cell adhesion molecule 2 |
| C920006O11Rik | 320295 | C920006O11Rik | RIKEN cDNA C920006O11 gene |
| Gp1bb | 14724 | Gp1bb | "glycoprotein Ib, beta polypeptide" |
| Efcab10 | 75040 | Efcab10 | EF-hand calcium binding domain 10 |
| Dnah9 | 237806 | Dnah9 | "dynein, axonemal, heavy chain 9" |
| Gm48878 | NA | NA | NA |
| Gm5628 | 434585 | Gm5628 | predicted gene 5628 |
| Mchr1 | 207911 | Mchr1 | melanin-concentrating hormone receptor 1 |
| Gm14412 | 102640477 | Gm14412 | predicted gene 14412 |
| Tcf23 | 69852 | Tcf23 | transcription factor 23 |
| Upk3a | 22270 | Upk3a | uroplakin 3A |
| Scg5 | 20394 | Scg5 | secretogranin V |
| Akap6 | 238161 | Akap6 | A kinase (PRKA) anchor protein 6 |
| Ccdc146 | 75172 | Ccdc146 | coiled-coil domain containing 146 |
| Akr1b7 | 11997 | Akr1b7 | "aldo-keto reductase family 1, member B7" |
| Klb | 83379 | Klb | klotho beta |
| Tnxa | 100043024 | Tnxa | tenascin XA (pseudogene) |
| Apold1 | 381823 | Apold1 | apolipoprotein L domain containing 1 |
| Cma1 | 17228 | Cma1 | "chymase 1, mast cell" |
| Bcas3os2 | 78420 | Bcas3os2 | breast carcinoma amplified sequence 3 opposite strand 2 |
| Nxph1 | 18231 | Nxph1 | neurexophilin 1 |

| | | | |
|---|---|---|---|
| Pzp | 11287 | Pzp | "PZP, alpha-2-macroglobulin like" |
| Gm13307 | 102631509 | Gm13307 | predicted gene 13307 |
| Bbox1 | 170442 | Bbox1 | "butyrobetaine (gamma), 2-oxoglutarate dioxygenase 1 (gamma-butyrobetaine hydroxylase)" |
| Acsm5 | 272428 | Acsm5 | acyl-CoA synthetase medium-chain family member 5 |
| Aldh1a3 | 56847 | Aldh1a3 | "aldehyde dehydrogenase family 1, subfamily A3" |
| Nox1 | 237038 | Nox1 | NADPH oxidase 1 |
| Dcdc2b | 100504491 | Dcdc2b | doublecortin domain containing 2b |
| Pak3 | 18481 | Pak3 | p21 (RAC1) activated kinase 3 |
| Hebp2 | 56016 | Hebp2 | heme binding protein 2 |
| Sat2 | 69215 | Sat2 | spermidine/spermine N1-acetyl transferase 2 |
| Nkx6-2 | 14912 | Nkx6-2 | NK6 homeobox 2 |
| Gm47036 | NA | NA | NA |
| Wfdc6a | 209351 | Wfdc6a | WAP four-disulfide core domain 6A |
| Gm15737 | 102633179 | Gm15737 | predicted gene 15737 |
| Gm44153 | NA | NA | NA |
| Mmp3 | 17392 | Mmp3 | matrix metallopeptidase 3 |
| Ankmy1 | 241158 | Ankmy1 | ankyrin repeat and MYND domain containing 1 |
| A630014C17Rik | 399633 | A630014C17Rik | RIKEN cDNA A630014C17 gene |
| Ppp1r32 | 67752 | Ppp1r32 | "protein phosphatase 1, regulatory subunit 32" |
| Alox15 | 11687 | Alox15 | arachidonate 15-lipoxygenase |
| Trim10 | 19824 | Trim10 | tripartite motif-containing 10 |
| Gm45805 | 115486981 | Gm45805 | predicted gene 45805 |
| E230001N04Rik | 320187 | E230001N04Rik | RIKEN cDNA E230001N04 gene |
| Gm13568 | NA | NA | NA |

| | | | |
|---|---|---|---|
| 4632432E15Rik | 74046 | 4632432E15Rik | RIKEN cDNA 4632432E15 gene |
| Klra9 | 16640 | Klra9 | "killer cell lectin-like receptor subfamily A, member 9" |
| Lrrc9 | 78257 | Lrrc9 | leucine rich repeat containing 9 |
| Zfp85os | 68271 | Zfp85os | "zinc finger protein 85, opposite strand" |
| Pih1h3b | 331537 | Pih1h3b | PIH1 domain containing 3B |
| Gm3248 | 100041279 | Gm3248 | predicted gene 3248 |
| Gm20755 | 626410 | Gm20755 | "predicted gene, 20755" |
| Mpz | 17528 | Mpz | myelin protein zero |
| Gata4 | 14463 | Gata4 | GATA binding protein 4 |
| Gm37359 | 105244462 | Gm37359 | "predicted gene, 37359" |
| Gm5790 | 544928 | Gm5790 | predicted gene 5790 |
| Slc35f1 | 215085 | Slc35f1 | "solute carrier family 35, member F1" |
| Ccl24 | 56221 | Ccl24 | chemokine (C-C motif) ligand 24 |
| Gm35533 | 102639153 | Gm35533 | "predicted gene, 35533" |
| Gm43123 | NA | NA | NA |
| Ccdc155 | 384619 | Ccdc155 | coiled-coil domain containing 155 |
| Gm10416 | 667213 | Gm10416 | predicted pseudogene 10416 |
| Mgam | 232714 | Mgam | maltase-glucoamylase |
| Grip2 | 243547 | Grip2 | glutamate receptor interacting protein 2 |
| Gm16188 | NA | NA | NA |
| D630024D03Rik | 414116 | D630024D03Rik | RIKEN cDNA D630024D03 gene |
| Styxl1 | 76571 | Styxl1 | serine/threonine/tyrosine interacting-like 1 |
| 9330159M07Rik | 319673 | 9330159M07Rik | RIKEN cDNA 9330159M07 gene |
| Synpr | 72003 | Synpr | synaptoporin |
| Asb14 | 142687 | Asb14 | ankyrin repeat and SOCS box-containing 14 |

| | | | |
|---|---|---|---|
| Tnnt3 | 21957 | Tnnt3 | "troponin T3, skeletal, fast" |
| Calcb | 116903 | Calcb | "calcitonin-related polypeptide, beta" |
| Gm44000 | NA | NA | NA |
| Chst9 | 71367 | Chst9 | carbohydrate (N-acetylgalactosamine 4-0) sulfotransferase 9 |
| Gm32926 | 102635639 | Gm32926 | "predicted gene, 32926" |
| Nhlrc4 | 621239 | Nhlrc4 | NHL repeat containing 4 |
| 1500035N22Rik | 70258 | 1500035N22Rik | RIKEN cDNA 1500035N22 gene |
| Gm45447 | NA | NA | NA |
| Tpd52l1 | 21987 | Tpd52l1 | tumor protein D52-like 1 |
| AC132460.3 | NA | NA | NA |
| Gm1667 | 381564 | Gm1667 | predicted gene 1667 |
| Slc35f3 | 210027 | Slc35f3 | "solute carrier family 35, member F3" |
| A330076H08Rik | 320026 | A330076H08Rik | RIKEN cDNA A330076H08 gene |
| 1700086O06Rik | 73516 | 1700086O06Rik | RIKEN cDNA 1700086O06 gene |
| Cfap73 | 546886 | Cfap73 | cilia and flagella associated protein 73 |
| Gm48500 | NA | NA | NA |
| Crb2 | 241324 | Crb2 | crumbs family member 2 |
| Gm48552 | NA | NA | NA |
| Alpk3 | 116904 | Alpk3 | alpha-kinase 3 |
| 8430426J06Rik | 78102 | 8430426J06Rik | RIKEN cDNA 8430426J06 gene |
| Gm16006 | 105245957 | Gm16006 | predicted gene 16006 |
| Dcdc5 | 329482 | Dcdc5 | doublecortin domain containing 5 |
| Cfap221 | 226356 | Cfap221 | cilia and flagella associated protein 221 |
| Gm17055 | NA | NA | NA |
| Il31ra | 218624 | Il31ra | interleukin 31 receptor A |

| | | | |
|---|---|---|---|
| Rgcc | 66214 | Rgcc | regulator of cell cycle |
| Aoc3 | 11754 | Aoc3 | "amine oxidase, copper containing 3" |
| A930012O16Rik | 227923 | A930012O16Rik | RIKEN cDNA A930012O16 gene |
| Acr | 11434 | Acr | acrosin prepropeptide |
| Tigd4 | 403175 | Tigd4 | tigger transposable element derived 4 |
| 9230112J17Rik | 100504561 | 9230112J17Rik | RIKEN cDNA 9230112J17 gene |
| Gm29538 | 102636980 | Gm29538 | predicted gene 29538 |
| Slc22a22 | 210463 | Slc22a22 | "solute carrier family 22 (organic cation transporter), member 22" |
| Gm12295 | 327946 | Gm12295 | predicted gene 12295 |
| Gm17066 | NA | NA | NA |
| Lncppara | 102800312 | Lncppara | long noncoding RNA near Ppara |
| Sele | 20339 | Sele | "selectin, endothelial cell" |
| Prima1 | 170952 | Prima1 | proline rich membrane anchor 1 |
| Ivl | 16447 | Ivl | involucrin |
| Kcnip4 | 80334 | Kcnip4 | Kv channel interacting protein 4 |
| Tnfaip6 | 21930 | Tnfaip6 | tumor necrosis factor alpha induced protein 6 |
| Slc26a3 | 13487 | Slc26a3 | "solute carrier family 26, member 3" |
| 4930412L05Rik | 73941 | 4930412L05Rik | RIKEN cDNA 4930412L05 gene |
| Ces1b | 382044 | Ces1b | carboxylesterase 1B |
| A730035I17Rik | 102637492 | A730035I17Rik | RIKEN cDNA A730035I17 gene |
| Apob | 238055 | Apob | apolipoprotein B |
| Has1 | 15116 | Has1 | hyaluronan synthase 1 |
| Dnmt3c | NA | NA | NA |
| Esrrg | 26381 | Esrrg | estrogen-related receptor gamma |
| Gm47715 | 108167801 | Gm47715 | "predicted gene, 47715" |

| | | | |
|---|---|---|---|
| Unc80 | 329178 | Unc80 | "unc-80, NALCN activator" |
| Pmp2 | 18857 | Pmp2 | peripheral myelin protein 2 |
| Gm34907 | 102638313 | Gm34907 | "predicted gene, 34907" |
| Itgb1bp2 | 26549 | Itgb1bp2 | integrin beta 1 binding protein 2 |
| Adam21 | 56622 | Adam21 | a disintegrin and metallopeptidase domain 21 |
| 9530051G07Rik | 319781 | 9530051G07Rik | RIKEN cDNA 9530051G07 gene |
| Rsph14 | 71236 | Rsph14 | radial spoke head homolog 14 (Chlamydomonas) |
| Sox2ot | 320478 | Sox2ot | SOX2 overlapping transcript (non-protein coding) |
| Zfp534 | 100503584 | Zfp534 | zinc finger protein 534 |
| Lsamp | 268890 | Lsamp | limbic system-associated membrane protein |
| Gm12474 | 545557 | Gm12474 | predicted gene 12474 |
| Tshb | 22094 | Tshb | "thyroid stimulating hormone, beta subunit" |
| Gm15912 | 115488138 | Gm15912 | predicted gene 15912 |
| Il22ra1 | 230828 | Il22ra1 | "interleukin 22 receptor, alpha 1" |
| Kcna5 | 16493 | Kcna5 | "potassium voltage-gated channel, shaker-related subfamily, member 5" |
| 1700041G16Rik | 73299 | 1700041G16Rik | RIKEN cDNA 1700041G16 gene |
| Rho | 212541 | Rho | rhodopsin |
| Mogat2 | 233549 | Mogat2 | monoacylglycerol O-acyltransferase 2 |
| Gm7312 | 640803 | Gm7312 | predicted gene 7312 |
| Gm10644 | 100126034 | Gm10644 | predicted gene 10644 |
| Gm15287 | NA | NA | NA |
| Stmn3 | 20262 | Stmn3 | stathmin-like 3 |
| Gm3417 | 100041586 | Gm3417 | predicted gene 3417 |
| Pck1 | 18534 | Pck1 | "phosphoenolpyruvate carboxykinase 1, cytosolic" |
| Ica1l | 70375 | Ica1l | islet cell autoantigen 1-like |

| | | | |
|---|---|---|---|
| Gabrq | 57249 | Gabrq | "gamma-aminobutyric acid (GABA) A receptor, subunit theta" |
| Calml3 | 70405 | Calml3 | calmodulin-like 3 |
| Gm36026 | 102639799 | Gm36026 | "predicted gene, 36026" |
| Smc2os | 72648 | Smc2os | "structural maintenance of chromosomes 2, opposite strand" |
| Wnk2 | 75607 | Wnk2 | WNK lysine deficient protein kinase 2 |
| Efhc2 | 74405 | Efhc2 | EF-hand domain (C-terminal) containing 2 |
| Gm30003 | 102631739 | Gm30003 | "predicted gene, 30003" |
| Thrsp | 21835 | Thrsp | thyroid hormone responsive |
| Cyp3a57 | 622127 | Cyp3a57 | "cytochrome P450, family 3, subfamily a, polypeptide 57" |
| Prtn3 | 19152 | Prtn3 | proteinase 3 |
| Sbk3 | 381835 | Sbk3 | "SH3 domain binding kinase family, member 3" |
| BC035947 | 269211 | BC035947 | cDNA sequence BC035947 |
| Cdh26 | 381409 | Cdh26 | cadherin-like 26 |
| Tspear | 252974 | Tspear | thrombospondin type laminin G domain and EAR repeats |
| Fam151a | 230579 | Fam151a | "family with sequence simliarity 151, member A" |
| Gm35394 | 102638959 | Gm35394 | "predicted gene, 35394" |
| Gm14569 | 101055983 | Gm14569 | predicted gene 14569 |
| Wfdc16 | 277345 | Wfdc16 | WAP four-disulfide core domain 16 |
| Gm49601 | NA | NA | NA |
| Wdr49 | 213248 | Wdr49 | WD repeat domain 49 |
| Ooep | 67968 | Ooep | oocyte expressed protein |
| Gm16464 | 672495 | Gm16464 | predicted gene 16464 |
| Gm49397 | NA | NA | NA |
| Paqr5 | 74090 | Paqr5 | progestin and adipoQ receptor family member V |
| Gm18703 | 100417591 | Gm18703 | "predicted gene, 18703" |

| | | | |
|---|---|---|---|
| Gm26910 | 102632394 | Gm26910 | "predicted gene, 26910" |
| Gm31105 | 102633223 | Gm31105 | "predicted gene, 31105" |
| AC125110.1 | NA | NA | NA |
| 5033426O07Rik | 76001 | 5033426O07Rik | RIKEN cDNA 5033426O07 gene |
| Bcas3os1 | 71489 | Bcas3os1 | "breast carcinoma amplified sequence 3, opposite strand 1" |
| Col6a6 | 245026 | Col6a6 | "collagen, type VI, alpha 6" |
| Il6 | 16193 | Il6 | interleukin 6 |
| 3110070M22Rik | 67304 | 3110070M22Rik | RIKEN cDNA 3110070M22 gene |
| Atp8b5 | 320571 | Atp8b5 | "ATPase, class I, type 8B, member 5" |
| Ribc2 | 67747 | Ribc2 | RIB43A domain with coiled-coils 2 |
| Gm8206 | 666637 | Gm8206 | predicted gene 8206 |
| Sult1d1 | 53315 | Sult1d1 | "sulfotransferase family 1D, member 1" |
| Lrrc52 | 240899 | Lrrc52 | leucine rich repeat containing 52 |
| Nxnl2 | 75124 | Nxnl2 | nucleoredoxin-like 2 |
| Lrrc39 | 109245 | Lrrc39 | leucine rich repeat containing 39 |
| Cd163 | 93671 | Cd163 | CD163 antigen |
| Gm13301 | 101056185 | Gm13301 | predicted gene 13301 |
| Trim15 | 69097 | Trim15 | tripartite motif-containing 15 |
| Ndp | 17986 | Ndp | Norrie disease (pseudoglioma) (human) |
| Crybb3 | 12962 | Crybb3 | "crystallin, beta B3" |
| A430108G06Rik | 319650 | A430108G06Rik | RIKEN cDNA A430108G06 gene |
| Stpg1 | 78806 | Stpg1 | sperm tail PG rich repeat containing 1 |
| Gm30382 | 102632254 | Gm30382 | "predicted gene, 30382" |
| Tram1l1 | 229801 | Tram1l1 | translocation associated membrane protein 1-like 1 |
| Gm48780 | NA | NA | NA |

| | | | |
|---|---|---|---|
| Gm44626 | NA | NA | NA |
| Gm15217 | 100041724 | Gm15217 | predicted gene 15217 |
| 8430419K02Rik | 74518 | 8430419K02Rik | RIKEN cDNA 8430419K02 gene |
| 1700061E17Rik | 100038395 | 1700061E17Rik | RIKEN cDNA 1700061E17 gene |

## Table S5-3. Integrative metabolic pathways enrichment analyses between Mel vs LPS group.

| Metabolite name | Total | Expected | Hits | Raw p | -LOG10(p) | FDR | Impact | matched_features |
|---|---|---|---|---|---|---|---|---|
| Aminoacyl-tRNA biosynthesis | 74 | 2.1409 | 11 | 5.21E-06 | 5.283 | 0.00043784 | 0.16438 | cpd:C00152; cpd:C00079; cpd:C00049; cpd:C00065; cpd:C00073; cpd:C00041; cpd:C00407; cpd:C00188; cpd:C00082; cpd:C00148; cpd:C00025 |
| Glutathione metabolism | 56 | 1.6201 | 8 | 0.00015265 | 3.8163 | 0.0064112 | 0.4 | cpd:C00025; cpd:C00077; mmu:14598; mmu:71522; mmu:14859; mmu:14867; mmu:14869; mmu:20135 |
| Alanine, aspartate and glutamate metabolism | 61 | 1.7648 | 8 | 0.00028189 | 3.5499 | 0.007893 | 0.58333 | cpd:C01042; cpd:C00049; cpd:C00152; cpd:C00041; cpd:C00025; cpd:C00334; cpd:C00438; mmu:14204 |
| Galactose metabolism | 51 | 1.4755 | 7 | 0.00052445 | 3.2803 | 0.011014 | 1.18 | cpd:C00267; cpd:C00031; cpd:C00794; mmu:11605; mmu:232714; mmu:11997; mmu:212032 |
| Arginine and proline metabolism | 78 | 2.2566 | 8 | 0.0015148 | 2.8197 | 0.025448 | 0.37662 | cpd:C00334; cpd:C01157; cpd:C00148; cpd:C00025; cpd:C00077; mmu:76507; mmu:67432; mmu:320452 |
| Phenylalanine, tyrosine and tryptophan biosynthesis | 11 | 0.31824 | 3 | 0.0032383 | 2.4897 | 0.045337 | 1.6 | cpd:C00079; cpd:C00082; mmu:14204 |
| Neomycin, kanamycin and gentamicin biosynthesis | 4 | 0.11572 | 2 | 0.0047676 | 2.3217 | 0.057211 | 1.3333 | cpd:C00031; mmu:212032 |
| Arginine biosynthesis | 27 | 0.78113 | 4 | 0.0068133 | 2.1666 | 0.071539 | 0.42308 | cpd:C00025; cpd:C00049; cpd:C00077; mmu:217214 |
| Tryptophan metabolism | 84 | 2.4302 | 7 | 0.0096096 | 2.0173 | 0.089689 | 0.31325 | cpd:C00780; cpd:C05635; mmu:15930; mmu:14204; mmu:13076; mmu:76507; mmu:21743 |
| Linoleic acid metabolism | 17 | 0.49182 | 3 | 0.011784 | 1.9287 | 0.095638 | 0.5 | mmu:13113; mmu:11687; mmu:18782 |
| Histidine metabolism | 32 | 0.92579 | 4 | 0.012524 | 1.9023 | 0.095638 | 0.22581 | cpd:C00025; cpd:C00049; mmu:15109; mmu:76507 |
| Fructose and mannose metabolism | 37 | 1.0704 | 4 | 0.020644 | 1.6852 | 0.14451 | 0.38889 | cpd:C00794; cpd:C00267; mmu:11997; mmu:212032 |
| Drug metabolism - cytochrome P450 | 39 | 1.1283 | 4 | 0.024624 | 1.6086 | 0.15911 | 0.078947 | mmu:14262; mmu:14859; mmu:14867; mmu:14869 |
| Phenylalanine metabolism | 24 | 0.69434 | 3 | 0.030367 | 1.5176 | 0.1822 | 0.43478 | cpd:C00079; cpd:C00082; mmu:14204 |
| Valine, leucine and isoleucine biosynthesis | 12 | 0.34717 | 2 | 0.045156 | 1.3453 | 0.25288 | 0.18182 | cpd:C00188; cpd:C00407 |
| Butanoate metabolism | 29 | 0.83899 | 3 | 0.049501 | 1.3054 | 0.25988 | 0.21429 | cpd:C01089; cpd:C00334; cpd:C00025 |
| Glycosphingolipid biosynthesis - globo and isoglobo series | 31 | 0.89686 | 3 | 0.058468 | 1.2331 | 0.2889 | 0.26667 | mmu:14343; mmu:14344; mmu:11605 |
| Arachidonic acid metabolism | 79 | 2.2855 | 5 | 0.07579 | 1.1204 | 0.32974 | 0.15385 | mmu:11684; mmu:11687; mmu:18782; mmu:14598; mmu:12409 |
| Taurine and hypotaurine metabolism | 16 | 0.46289 | 2 | 0.076261 | 1.1177 | 0.32974 | 0.13333 | mmu:14598; mmu:71522 |
| Glycerolipid metabolism | 35 | 1.0126 | 3 | 0.078511 | 1.1051 | 0.32974 | 0.23529 | mmu:11997; mmu:11605; mmu:331374 |
| Starch and sucrose metabolism | 37 | 1.0704 | 3 | 0.089522 | 1.0481 | 0.35809 | 0.66667 | cpd:C00031; mmu:212032; mmu:232714 |
| Metabolism of xenobiotics by cytochrome P450 | 117 | 3.3849 | 6 | 0.11934 | 0.92322 | 0.45565 | 0.49138 | mmu:13076; mmu:14859; mmu:14867; mmu:14869; mmu:12409; mmu:13107 |
| Retinol metabolism | 44 | 1.273 | 3 | 0.13263 | 0.87735 | 0.47863 | 0.30233 | mmu:79235; mmu:13113; mmu:13076 |
| Drug metabolism - other enzymes | 69 | 1.9962 | 4 | 0.13675 | 0.86407 | 0.47863 | 0.058824 | mmu:20135; mmu:14859; mmu:14867; mmu:14869 |
| Cysteine and methionine metabolism | 71 | 2.0541 | 4 | 0.1474 | 0.83151 | 0.49379 | 0.18571 | cpd:C00065; cpd:C00073; mmu:12411; mmu:14204 |
| Glycine, serine and threonine metabolism | 72 | 2.083 | 4 | 0.15284 | 0.81576 | 0.49379 | 0.38028 | cpd:C00065; cpd:C00188; mmu:12411; mmu:14711 |

| | | | | | | | | |
|---|---|---|---|---|---|---|---|---|
| Glyoxylate and dicarboxylate metabolism | 56 | 1.6201 | 3 | 0.21908 | 0.6594 | 0.65691 | 0.16364 | cpd:C00065; cpd:C00025; mmu:67432 |
| Pentose and glucuronate interconversions | 32 | 0.92579 | 2 | 0.23627 | 0.62659 | 0.65691 | 0.45161 | cpd:C00379; mmu:11997 |
| Phosphonate and phosphinate metabolism | 10 | 0.28931 | 1 | 0.25483 | 0.59375 | 0.65691 | 0.11111 | cpd:C03557 |
| Synthesis and degradation of ketone bodies | 10 | 0.28931 | 1 | 0.25483 | 0.59375 | 0.65691 | 0.22222 | cpd:C01089 |
| Nitrogen metabolism | 10 | 0.28931 | 1 | 0.25483 | 0.59375 | 0.65691 | 0.22222 | cpd:C00025 |
| D-Glutamine and D-glutamate metabolism | 10 | 0.28931 | 1 | 0.25483 | 0.59375 | 0.65691 | 0.33333 | cpd:C00025 |
| Glycolysis or Gluconeogenesis | 61 | 1.7648 | 3 | 0.25807 | 0.58826 | 0.65691 | 0.23333 | cpd:C00267; mmu:226265; mmu:212032 |
| Selenocompound metabolism | 35 | 1.0126 | 2 | 0.26886 | 0.57048 | 0.66424 | 0.14706 | cpd:C00041; mmu:21743 |
| Ether lipid metabolism | 39 | 1.1283 | 2 | 0.31229 | 0.50545 | 0.74949 | 0.15789 | mmu:27226; mmu:18782 |
| Ubiquinone and other terpenoid-quinone biosynthesis | 17 | 0.49182 | 1 | 0.39395 | 0.40456 | 0.91921 | 0.0625 | cpd:C00082 |
| alpha-Linolenic acid metabolism | 22 | 0.63648 | 1 | 0.47731 | 0.3212 | 1 | 0.095238 | mmu:18782 |
| Mucin type O-glycan biosynthesis | 22 | 0.63648 | 1 | 0.47731 | 0.3212 | 1 | 0.14286 | mmu:241391 |
| Sphingolipid metabolism | 58 | 1.678 | 2 | 0.50555 | 0.29623 | 1 | 0.052632 | cpd:C00065; mmu:11605 |
| Pyrimidine metabolism | 99 | 2.8642 | 3 | 0.55288 | 0.25737 | 1 | 0.27551 | cpd:C00438; mmu:20135; mmu:633979 |
| Pantothenate and CoA biosynthesis | 34 | 0.98365 | 1 | 0.63404 | 0.19788 | 1 | 0.030303 | cpd:C00049 |
| Phosphatidylinositol signaling system | 74 | 2.1409 | 2 | 0.63947 | 0.19418 | 1 | 0.054795 | mmu:331374; mmu:228550 |
| Amino sugar and nucleotide sugar metabolism | 81 | 2.3434 | 2 | 0.68862 | 0.16202 | 1 | 0.175 | cpd:C00267; mmu:212032 |
| Nicotinate and nicotinamide metabolism | 43 | 1.244 | 1 | 0.72023 | 0.14253 | 1 | 0.02381 | cpd:C00049 |
| Glycerophospholipid metabolism | 86 | 2.4881 | 2 | 0.72033 | 0.14247 | 1 | 0.070588 | mmu:18782; mmu:331374 |
| beta-Alanine metabolism | 44 | 1.273 | 1 | 0.72847 | 0.13759 | 1 | 0.046512 | cpd:C00049 |
| Glycosaminoglycan degradation | 44 | 1.273 | 1 | 0.72847 | 0.13759 | 1 | 0.13953 | mmu:15442 |
| Valine, leucine and isoleucine degradation | 88 | 2.5459 | 2 | 0.73224 | 0.13534 | 1 | 0.057471 | cpd:C00407; mmu:14204 |
| Tyrosine metabolism | 88 | 2.5459 | 2 | 0.73224 | 0.13534 | 1 | 0.14943 | cpd:C00082; mmu:14204 |
| Purine metabolism | 169 | 4.8893 | 4 | 0.73326 | 0.13474 | 1 | 0.26786 | cpd:C00366; cpd:C00147; mmu:20135; mmu:633979 |
| Glycosphingolipid biosynthesis - ganglio series | 47 | 1.3597 | 1 | 0.75179 | 0.1239 | 1 | 0.043478 | mmu:26938 |
| Lysine degradation | 49 | 1.4176 | 1 | 0.76623 | 0.11564 | 1 | 0.041667 | mmu:170442 |
| Porphyrin and chlorophyll metabolism | 53 | 1.5333 | 1 | 0.79267 | 0.10091 | 1 | 0.019231 | cpd:C00025 |
| Glycosphingolipid biosynthesis - lacto and neolacto series | 102 | 2.9509 | 2 | 0.80427 | 0.094596 | 1 | 0.13861 | mmu:14343; mmu:14344 |
| Folate biosynthesis | 60 | 1.7358 | 1 | 0.83203 | 0.079863 | 1 | 0.067797 | mmu:11997 |

| | | | | | | | | |
|---|---|---|---|---|---|---|---|---|
| Inositol phosphate metabolism | 69 | 1.9962 | 1 | 0.87198 | 0.059494 | 1 | 0.029412 | mmu:228550 |
| Steroid hormone biosynthesis | 175 | 5.0629 | 3 | 0.89329 | 0.049006 | 1 | 0.08046 | mmu:13076; mmu:13113; mmu:15484 |
| Steroid biosynthesis | 82 | 2.3723 | 1 | 0.91369 | 0.039202 | 1 | 0.049383 | cpd:C05437 |
| Primary bile acid biosynthesis | 90 | 2.6038 | 1 | 0.93236 | 0.030418 | 1 | 0.044944 | mmu:93732 |

Table S5-4. Annotation of metabolites and genes between Mel vs LPS group.

| Gene | Entrez | Symbol | Name | Metabolite KEGG ID | Name |
|---|---|---|---|---|---|
| Nckap5 | 210356 | Nckap5 | NCK-associated protein 5 | C03557 | Ciliatine |
| Bmp6 | 12161 | Bmp6 | bone morphogenetic protein 6 | C00556 | Benzyl alcohol |
| Nr3c2 | 110784 | Nr3c2 | "nuclear receptor subfamily 3, group C, member 2" | C00490 | Itaconic acid |
| Chst3 | 53374 | Chst3 | carbohydrate sulfotransferase 3 | C00152 | L-Asparagine |
| Glb1l2 | 244757 | Glb1l2 | "galactosidase, beta 1-like 2" | C02226 | Citraconic acid |
| Retnlg | 245195 | Retnlg | resistin like gamma | C03661 | 1-Kestose |
| Tmtc2 | 278279 | Tmtc2 | transmembrane and tetratricopeptide repeat containing 2 | C00267 | Alpha-D-Glucose |
| Inmt | 21743 | Inmt | indolethylamine N-methyltransferase | C01835 | Maltotriose |
| Cbr2 | 12409 | Cbr2 | carbonyl reductase 2 | C00031 | D-Glucose |
| Cirbp | 12696 | Cirbp | cold inducible RNA binding protein | C08243 | Melezitose |
| Ccr3 | 12771 | Ccr3 | chemokine (C-C motif) receptor 3 | C00065 | L-Serine |
| Frmpd1 | 666060 | Frmpd1 | FERM and PDZ domain containing 1 | C00073 | L-Methionine |
| Scel | 64929 | Scel | sciellin | C00438 | Ureidosuccinic acid |
| Ednrb | 13618 | Ednrb | endothelin receptor type B | C00263 | L-Homoserine |
| Scgb1a1 | 22287 | Scgb1a1 | "secretoglobin, family 1A, member 1 (uteroglobin)" | C00188 | L-Threonine |
| 2210011C24Rik | NA | NA | NA | C00407 | L-Isoleucine |
| Ighd | 380797 | Ighd | immunoglobulin heavy constant delta | C00148 | L-Proline |
| Dpep1 | 13479 | Dpep1 | dipeptidase 1 | C00082 | L-Tyrosine |
| Alox15 | 11687 | Alox15 | arachidonate 15-lipoxygenase | C05635 | 5-Hydroxyindoleacetic acid |
| Fgfbp1 | 14181 | Fgfbp1 | fibroblast growth factor binding protein 1 | C00041 | L-Alanine |
| Slc7a10 | 53896 | Slc7a10 | "solute carrier family 7 (cationic amino acid transporter, y+ system), member 10" | C00079 | L-Phenylalanine |
| Fam178b | 381337 | Fam178b | "family with sequence similarity 178, member B" | C00077 | Ornithine |
| C1qtnf2 | 69183 | C1qtnf2 | C1q and tumor necrosis factor related protein 2 | C00379 | D-Xylitol |
| F5 | 14067 | F5 | coagulation factor V | C01089 | (R)-3-Hydroxybutyric acid |
| Lrat | 79235 | Lrat | lecithin-retinol acyltransferase (phosphatidylcholine-retinol-O-acyltransferase) | C00147 | Adenine |
| Lrriq1 | 74978 | Lrriq1 | leucine-rich repeats and IQ motif containing 1 | C00025 | L-Glutamic acid |
| Prkg2 | 19092 | Prkg2 | "protein kinase, cGMP-dependent, type II" | C05629 | Hydrocinnamic acid |

| | | | |
|---|---|---|---|
| Acox2 | 93732 | Acox2 | "acyl-Coenzyme A oxidase 2, branched chain" |
| Igkv1-122 | 434024 | Igkv1-122 | immunoglobulin kappa chain variable 1-122 |
| Gsta3 | 14859 | Gsta3 | "glutathione S-transferase, alpha 3" |
| 1700088E04Rik | 27660 | 1700088E04Rik | RIKEN cDNA 1700088E04 gene |
| Tctex1d4 | 242646 | Tctex1d4 | Tctex1 domain containing 4 |
| Tubb1 | 545486 | Tubb1 | "tubulin, beta 1 class VI" |
| Pon1 | 18979 | Pon1 | paraoxonase 1 |
| Scn3a | 20269 | Scn3a | "sodium channel, voltage-gated, type III, alpha" |
| Gnmt | 14711 | Gnmt | glycine N-methyltransferase |
| Itga2b | 16399 | Itga2b | integrin alpha 2b |
| Acoxl | 74121 | Acoxl | acyl-Coenzyme A oxidase-like |
| Bex2 | 12069 | Bex2 | brain expressed X-linked 2 |
| Sell | 20343 | Sell | "selectin, lymphocyte" |
| Pim3 | 223775 | Pim3 | proviral integration site 3 |
| Myh7 | 140781 | Myh7 | "myosin, heavy polypeptide 7, cardiac muscle, beta" |
| Adgre4 | 52614 | Adgre4 | adhesion G protein-coupled receptor E4 |
| Gria1 | 14799 | Gria1 | "glutamate receptor, ionotropic, AMPA1 (alpha 1)" |
| Gm37516 | NA | NA | NA |
| Bex4 | 406217 | Bex4 | brain expressed X-linked 4 |
| Slc22a3 | 20519 | Slc22a3 | "solute carrier family 22 (organic cation transporter), member 3" |
| Ankrd63 | 383787 | Ankrd63 | ankyrin repeat domain 63 |
| Ldlrad1 | 546840 | Ldlrad1 | low density lipoprotein receptor class A domain containing 1 |
| Gfra1 | 14585 | Gfra1 | glial cell line derived neurotrophic factor family receptor alpha 1 |
| Colq | 382864 | Colq | collagen-like tail subunit (single strand of homotrimer) of asymmetric acetylcholinesterase |
| Dnah7a | 627872 | Dnah7a | "dynein, axonemal, heavy chain 7A" |
| Lmntd1 | 74071 | Lmntd1 | lamin tail domain containing 1 |
| Cacna2d4 | 319734 | Cacna2d4 | "calcium channel, voltage-dependent, alpha 2/delta subunit 4" |

| | |
|---|---|
| C00780 | Serotonin |
| C00366 | Uric acid |
| C05437 | Zymosterol intermediate 2 |
| C00049 | L-Aspartic acid |
| C00794 | Sorbitol |
| C01042 | N-Acetyl-L-aspartic acid |
| C00334 | Gamma-Aminobutyric acid |
| C01157 | 4-Hydroxyproline |
| C06562 | Catechin |

| | | | |
|---|---|---|---|
| Xpnpep2 | 170745 | Xpnpep2 | "X-prolyl aminopeptidase (aminopeptidase P) 2, membrane-bound" |
| AC141882.2 | NA | NA | NA |
| Gm14964 | 100008567 | Gm14964 | predicted gene 14964 |
| Chrm2 | 243764 | Chrm2 | "cholinergic receptor, muscarinic 2, cardiac" |
| Cfap206 | 69329 | Cfap206 | cilia and flagella associated protein 206 |
| Shank2 | 210274 | Shank2 | SH3 and multiple ankyrin repeat domains 2 |
| Pcp4l1 | 66425 | Pcp4l1 | Purkinje cell protein 4-like 1 |
| Gm4956 | 241041 | Gm4956 | predicted gene 4956 |
| Ccdc40 | 207607 | Ccdc40 | coiled-coil domain containing 40 |
| Spag16 | 66722 | Spag16 | sperm associated antigen 16 |
| Col17a1 | 12821 | Col17a1 | "collagen, type XVII, alpha 1" |
| Cd300lg | 52685 | Cd300lg | CD300 molecule like family member G |
| Gm5706 | 435657 | Gm5706 | predicted gene 5706 |
| Itprid1 | 232016 | Itprid1 | ITPR interacting domain containing 1 |
| Nme9 | 623534 | Nme9 | NME/NM23 family member 9 |
| Cyp2f2 | 13107 | Cyp2f2 | "cytochrome P450, family 2, subfamily f, polypeptide 2" |
| 1700024G13Rik | 67085 | 1700024G13Rik | RIKEN cDNA 1700024G13 gene |
| Dnah1 | 110084 | Dnah1 | "dynein, axonemal, heavy chain 1" |
| Lrrc74b | 74685 | Lrrc74b | leucine rich repeat containing 74B |
| Atp2a1 | 11937 | Atp2a1 | "ATPase, Ca++ transporting, cardiac muscle, fast twitch 1" |
| Stmnd1 | 380842 | Stmnd1 | stathmin domain containing 1 |
| Klf15 | 66277 | Klf15 | Kruppel-like factor 15 |
| Dbp | 13170 | Dbp | D site albumin promoter binding protein |
| Alox12 | 11684 | Alox12 | arachidonate 12-lipoxygenase |
| Pifo | 100503311 | Pifo | primary cilia formation |

| | | | |
|---|---|---|---|
| Slc16a11 | 216867 | Slc16a11 | "solute carrier family 16 (monocarboxylic acid transporters), member 11" |
| Ect2l | 100045792 | Ect2l | epithelial cell transforming sequence 2 oncogene-like |
| Lrrc23 | 16977 | Lrrc23 | leucine rich repeat containing 23 |
| Tmeff2 | 56363 | Tmeff2 | transmembrane protein with EGF-like and two follistatin-like domains 2 |
| Ppbp | 57349 | Ppbp | pro-platelet basic protein |
| Cfap126 | 75472 | Cfap126 | cilia and flagella associated protein 126 |
| Armh1 | 381544 | Armh1 | armadillo-like helical domain containing 1 |
| Sh3gl2 | 20404 | Sh3gl2 | SH3-domain GRB2-like 2 |
| Slc5a12 | 241612 | Slc5a12 | "solute carrier family 5 (sodium/glucose cotransporter), member 12" |
| Prkag3 | 241113 | Prkag3 | "protein kinase, AMP-activated, gamma 3 non-catalytic subunit" |
| 1810008I18Rik | 100503969 | 1810008I18Rik | RIKEN cDNA 1810008I18 gene |
| Tll1 | 21892 | Tll1 | tolloid-like |
| Tmem212 | 208613 | Tmem212 | transmembrane protein 212 |
| Ccdc24 | 381546 | Ccdc24 | coiled-coil domain containing 24 |
| Hes2 | 15206 | Hes2 | hes family bHLH transcription factor 2 |
| Lrrc17 | 74511 | Lrrc17 | leucine rich repeat containing 17 |
| Bbof1 | 72873 | Bbof1 | basal body orientation factor 1 |
| Fbxo16 | 50759 | Fbxo16 | F-box protein 16 |
| Serpina3c | 16625 | Serpina3c | "serine (or cysteine) peptidase inhibitor, clade A, member 3C" |
| St6galnac5 | 26938 | St6galnac5 | "ST6 (alpha-N-acetyl-neuraminyl-2,3-beta-galactosyl-1,3)-N-acetylgalactosaminide alpha-2,6-sialyltransferase 5" |
| Stk33 | 117229 | Stk33 | serine/threonine kinase 33 |
| Ncr1 | 17086 | Ncr1 | natural cytotoxicity triggering receptor 1 |
| Esr2 | 13983 | Esr2 | estrogen receptor 2 (beta) |
| S1pr5 | 94226 | S1pr5 | sphingosine-1-phosphate receptor 5 |
| Gm973 | 381260 | Gm973 | predicted gene 973 |

| | | | |
|---|---|---|---|
| Lrtm2 | 211187 | Lrtm2 | leucine-rich repeats and transmembrane domains 2 |
| Fmn2 | 54418 | Fmn2 | formin 2 |
| Dpysl5 | 65254 | Dpysl5 | dihydropyrimidinase-like 5 |
| Gm8229 | 666675 | Gm8229 | predicted gene 8229 |
| Klrb1c | 17059 | Klrb1c | killer cell lectin-like receptor subfamily B member 1C |
| Eno4 | 226265 | Eno4 | enolase 4 |
| Fam92b | 436062 | Fam92b | "family with sequence similarity 92, member B" |
| Cfap157 | 227736 | Cfap157 | cilia and flagella associated protein 157 |
| Catip | 241112 | Catip | ciliogenesis associated TTC17 interacting protein |
| Chst8 | 68947 | Chst8 | carbohydrate sulfotransferase 8 |
| Fam183b | 75429 | Fam183b | "family with sequence similarity 183, member B" |
| Ccdc170 | 100504234 | Ccdc170 | coiled-coil domain containing 170 |
| Drc7 | 330830 | Drc7 | dynein regulatory complex subunit 7 |
| Ttc29 | 73301 | Ttc29 | tetratricopeptide repeat domain 29 |
| Iqcd | 75732 | Iqcd | IQ motif containing D |
| Tsnaxip1 | 72236 | Tsnaxip1 | translin-associated factor X (Tsnax) interacting protein 1 |
| Gm32857 | 102635555 | Gm32857 | "predicted gene, 32857" |
| 3300002A11Rik | 100503812 | 3300002A11Rik | RIKEN cDNA 3300002A11 gene |
| Unc79 | 217843 | Unc79 | unc-79 homolog |
| Ccdc162 | 75973 | Ccdc162 | coiled-coil domain containing 162 |
| Lnx1 | 16924 | Lnx1 | ligand of numb-protein X 1 |
| Spag6l | 50525 | Spag6l | sperm associated antigen 6-like |
| Cfap54 | 380654 | Cfap54 | cilia and flagella associated protein 54 |
| Dcdc2b | 100504491 | Dcdc2b | doublecortin domain containing 2b |
| Chrm3 | 12671 | Chrm3 | "cholinergic receptor, muscarinic 3, cardiac" |

| | | | |
|---|---|---|---|
| Sptb | 20741 | Sptb | "spectrin beta, erythrocytic" |
| Doc2b | 13447 | Doc2b | "double C2, beta" |
| Gm867 | 333670 | Gm867 | predicted gene 867 |
| Ccdc155 | 384619 | Ccdc155 | coiled-coil domain containing 155 |
| 5930430L01Rik | 319982 | 5930430L01Rik | RIKEN cDNA 5930430L01 gene |
| Gsg1l | 269994 | Gsg1l | GSG1-like |
| Rps10-ps2 | 666274 | Rps10-ps2 | "ribosomal protein S10, pseudogene 2" |
| Gstm6 | 14867 | Gstm6 | "glutathione S-transferase, mu 6" |
| Smkr-ps | 76419 | Smkr-ps | "smal lysine rich protein 1, pseudogene" |
| Gp1ba | 14723 | Gp1ba | "glycoprotein 1b, alpha polypeptide" |
| Ccdc81 | 70884 | Ccdc81 | coiled-coil domain containing 81 |
| P2rx1 | 18436 | P2rx1 | "purinergic receptor P2X, ligand-gated ion channel, 1" |
| 6820408C15Rik | 228778 | 6820408C15Rik | RIKEN cDNA 6820408C15 gene |
| Wnt2 | 22413 | Wnt2 | "wingless-type MMTV integration site family, member 2" |
| Hoga1 | 67432 | Hoga1 | 4-hydroxy-2-oxoglutarate aldolase 1 |
| Ttll9 | 74711 | Ttll9 | "tubulin tyrosine ligase-like family, member 9" |
| Fank1 | 66930 | Fank1 | fibronectin type 3 and ankyrin repeat domains 1 |
| Lrrc10b | 278795 | Lrrc10b | leucine rich repeat containing 10B |
| Ccdc153 | 270150 | Ccdc153 | coiled-coil domain containing 153 |
| 1700001C02Rik | NA | NA | NA |
| Armc4 | 74934 | Armc4 | armadillo repeat containing 4 |
| 1700037C18Rik | 73261 | 1700037C18Rik | RIKEN cDNA 1700037C18 gene |
| Sntn | 218739 | Sntn | "sentan, cilia apical structure protein" |
| Hpcal4 | 170638 | Hpcal4 | hippocalcin-like 4 |
| Wfdc6b | 433502 | Wfdc6b | WAP four-disulfide core domain 6B |

| | | | |
|---|---|---|---|
| Uckl1os | 100271841 | Uckl1os | "uridine-cytidine kinase 1-like 1, opposite strand" |
| 8030442B05Rik | 77543 | 8030442B05Rik | RIKEN cDNA 8030442B05 gene |
| Tnr | 21960 | Tnr | tenascin R |
| Cbs | 12411 | Cbs | cystathionine beta-synthase |
| Dnah7c | 100101919 | Dnah7c | "dynein, axonemal, heavy chain 7C" |
| Rgs22 | 626596 | Rgs22 | regulator of G-protein signalling 22 |
| Treml1 | 71326 | Treml1 | triggering receptor expressed on myeloid cells-like 1 |
| A830018L16Rik | 320492 | A830018L16Rik | RIKEN cDNA A830018L16 gene |
| Itpka | 228550 | Itpka | "inositol 1,4,5-trisphosphate 3-kinase A" |
| 9530036O11Rik | 654796 | 9530036O11Rik | RIKEN cDNA 9530036O11Rik |
| Odf3b | 70113 | Odf3b | outer dense fiber of sperm tails 3B |
| A930003A15Rik | 68162 | A930003A15Rik | RIKEN cDNA A930003A15 gene |
| Ak9 | 633979 | Ak9 | adenylate kinase 9 |
| Aoc1 | 76507 | Aoc1 | "amine oxidase, copper-containing 1" |
| Ribc1 | 66611 | Ribc1 | RIB43A domain with coiled-coils 1 |
| Sybu | 319613 | Sybu | syntabulin (syntaxin-interacting) |
| Pnck | 93843 | Pnck | pregnancy upregulated non-ubiquitously expressed CaM kinase |
| Pbld1 | 68371 | Pbld1 | phenazine biosynthesis-like protein domain containing 1 |
| Cfap70 | 76670 | Cfap70 | cilia and flagella associated protein 70 |
| Caps2 | 353025 | Caps2 | calcyphosphine 2 |
| Klhdc9 | 68874 | Klhdc9 | kelch domain containing 9 |
| 1700001L19Rik | 69315 | 1700001L19Rik | RIKEN cDNA 1700001L19 gene |
| Ccdc180 | 381522 | Ccdc180 | coiled-coil domain containing 180 |
| Gm9889 | 791369 | Gm9889 | predicted gene 9889 |
| Ppil6 | 73075 | Ppil6 | peptidylprolyl isomerase (cyclophilin)-like 6 |

| | | | |
|---|---|---|---|
| Gm7173 | NA | NA | NA |
| Fmo3 | 14262 | Fmo3 | flavin containing monooxygenase 3 |
| Scn4a | 110880 | Scn4a | "sodium channel, voltage-gated, type IV, alpha" |
| Rps6ka6 | 67071 | Rps6ka6 | ribosomal protein S6 kinase polypeptide 6 |
| Errfi1 | 74155 | Errfi1 | ERBB receptor feedback inhibitor 1 |
| Zfp628 | 232816 | Zfp628 | zinc finger protein 628 |
| Cyp3a13 | 13113 | Cyp3a13 | "cytochrome P450, family 3, subfamily a, polypeptide 13" |
| Tas2r135 | 387512 | Tas2r135 | "taste receptor, type 2, member 135" |
| Serpinb10 | 241197 | Serpinb10 | "serine (or cysteine) peptidase inhibitor, clade B (ovalbumin), member 10" |
| Ccdc146 | 75172 | Ccdc146 | coiled-coil domain containing 146 |
| Gm4262 | 100043147 | Gm4262 | predicted gene 4262 |
| 9030407P20Rik | 100504389 | 9030407P20Rik | RIKEN cDNA 9030407P20 gene |
| Barx2 | 12023 | Barx2 | BarH-like homeobox 2 |
| 4933406B17Rik | 71040 | 4933406B17Rik | RIKEN cDNA 4933406B17 gene |
| Ccdc121 | 403180 | Ccdc121 | coiled-coil domain containing 121 |
| Dync1i1 | 13426 | Dync1i1 | dynein cytoplasmic 1 intermediate chain 1 |
| Tbata | 65971 | Tbata | "thymus, brain and testes associated" |
| Arl4d | 80981 | Arl4d | ADP-ribosylation factor-like 4D |
| Zmynd12 | 332934 | Zmynd12 | "zinc finger, MYND domain containing 12" |
| Bche | 12038 | Bche | butyrylcholinesterase |
| Nek10 | 674895 | Nek10 | NIMA (never in mitosis gene a)- related kinase 10 |
| Gp9 | 54368 | Gp9 | glycoprotein 9 (platelet) |
| Gm13582 | 100038493 | Gm13582 | predicted gene 13582 |
| Gm9968 | 791329 | Gm9968 | predicted gene 9968 |
| Epor | 13857 | Epor | erythropoietin receptor |

| | | | |
|---|---|---|---|
| Atp2c2 | 69047 | Atp2c2 | "ATPase, Ca++ transporting, type 2C, member 2" |
| Fam83e | 73813 | Fam83e | "family with sequence similarity 83, member E" |
| Pzp | 11287 | Pzp | "PZP, alpha-2-macroglobulin like" |
| Fabp12 | 75497 | Fabp12 | fatty acid binding protein 12 |
| Gm5790 | 544928 | Gm5790 | predicted gene 5790 |
| Padi4 | 18602 | Padi4 | "peptidyl arginine deiminase, type IV" |
| Chgb | 12653 | Chgb | chromogranin B |
| Adam3 | 11497 | Adam3 | a disintegrin and metallopeptidase domain 3 (cyritestin) |
| Gm10309 | 100038711 | Gm10309 | predicted gene 10309 |
| Olfr750 | 404319 | Olfr750 | olfactory receptor 750 |
| Ppp2r2b | 72930 | Ppp2r2b | "protein phosphatase 2, regulatory subunit B, beta" |
| Kif6 | 319991 | Kif6 | kinesin family member 6 |
| D830026I12Rik | 319682 | D830026I12Rik | RIKEN cDNA D830026I12 gene |
| 2700054A10Rik | 72578 | 2700054A10Rik | RIKEN cDNA 2700054A10 gene |
| Ggt1 | 14598 | Ggt1 | gamma-glutamyltransferase 1 |
| Igsf11 | 207683 | Igsf11 | "immunoglobulin superfamily, member 11" |
| Gm30648 | 102632621 | Gm30648 | "predicted gene, 30648" |
| Cabcoco1 | 73287 | Cabcoco1 | ciliary associated calcium binding coiled-coil 1 |
| Skint3 | 195564 | Skint3 | selection and upkeep of intraepithelial T cells 3 |
| Gm19299 | 100502636 | Gm19299 | "predicted gene, 19299" |
| Tacr2 | 21337 | Tacr2 | tachykinin receptor 2 |
| Ccdc74a | 72315 | Ccdc74a | coiled-coil domain containing 74A |
| Cldn8 | 54420 | Cldn8 | claudin 8 |
| Ppp1r32 | 67752 | Ppp1r32 | "protein phosphatase 1, regulatory subunit 32" |
| 1700028P14Rik | 67483 | 1700028P14Rik | RIKEN cDNA 1700028P14 gene |

| | | | |
|---|---|---|---|
| Gm5483 | NA | NA | NA |
| Rsph14 | 71236 | Rsph14 | radial spoke head homolog 14 (Chlamydomonas) |
| Apobec4 | 71281 | Apobec4 | "apolipoprotein B mRNA editing enzyme, catalytic polypeptide-like 4 (putative)" |
| Gm31463 | 102633702 | Gm31463 | "predicted gene, 31463" |
| Ttll13 | 269954 | Ttll13 | "tubulin tyrosine ligase-like family, member 13" |
| Gm11730 | 102637059 | Gm11730 | predicted gene 11730 |
| Ano5 | 233246 | Ano5 | anoctamin 5 |
| Csrnp1 | 215418 | Csrnp1 | cysteine-serine-rich nuclear protein 1 |
| Gp6 | 243816 | Gp6 | glycoprotein 6 (platelet) |
| Plin5 | 66968 | Plin5 | perilipin 5 |
| Efcab12 | 212516 | Efcab12 | EF-hand calcium binding domain 12 |
| Gm48957 | NA | NA | NA |
| Gm29538 | 102636980 | Gm29538 | predicted gene 29538 |
| Gm21149 | 100861702 | Gm21149 | "predicted gene, 21149" |
| 1810010D01Rik | NA | NA | NA |
| AC162182.1 | NA | NA | NA |
| Galnt5 | 241391 | Galnt5 | polypeptide N-acetylgalactosaminyltransferase 5 |
| Gm46136 | 108167692 | Gm46136 | "predicted gene, 46136" |
| Gm44000 | NA | NA | NA |
| Tmprss5 | 80893 | Tmprss5 | "transmembrane protease, serine 5 (spinesin)" |
| Dnaaf1 | 68270 | Dnaaf1 | "dynein, axonemal assembly factor 1" |
| Gm15958 | 100503546 | Gm15958 | predicted gene 15958 |
| Klra3 | 16634 | Klra3 | "killer cell lectin-like receptor, subfamily A, member 3" |
| Gas2l2 | 237891 | Gas2l2 | growth arrest-specific 2 like 2 |
| 1700013F07Rik | 75504 | 1700013F07Rik | RIKEN cDNA 1700013F07 gene |

| | | | |
|---|---|---|---|
| Gm15475 | NA | NA | NA |
| Gp5 | 14729 | Gp5 | glycoprotein 5 (platelet) |
| Klk10 | 69540 | Klk10 | kallikrein related-peptidase 10 |
| 4930550C14Rik | 75311 | 4930550C14Rik | RIKEN cDNA 4930550C14 gene |
| Pcsk4 | 18551 | Pcsk4 | proprotein convertase subtilisin/kexin type 4 |
| Thegl | 71868 | Thegl | theg spermatid protein like |
| Gm12695 | 620779 | Gm12695 | predicted gene 12695 |
| Akr1b7 | 11997 | Akr1b7 | "aldo-keto reductase family 1, member B7" |
| Ankmy1 | 241158 | Ankmy1 | ankyrin repeat and MYND domain containing 1 |
| Prom2 | 192212 | Prom2 | prominin 2 |
| Gm49701 | NA | NA | NA |
| Gstp2 | 14869 | Gstp2 | "glutathione S-transferase, pi 2" |
| Egr1 | 13653 | Egr1 | early growth response 1 |
| Dusp26 | 66959 | Dusp26 | dual specificity phosphatase 26 (putative) |
| Il22ra1 | 230828 | Il22ra1 | "interleukin 22 receptor, alpha 1" |
| Gm24327 | NA | NA | NA |
| Gm16006 | 105245957 | Gm16006 | predicted gene 16006 |
| Slc22a12 | 20521 | Slc22a12 | "solute carrier family 22 (organic anion/cation transporter), member 12" |
| Gm39321 | 105243385 | Gm39321 | "predicted gene, 39321" |
| Gm40437 | 105244915 | Gm40437 | "predicted gene, 40437" |
| Gm609 | 208166 | Gm609 | predicted gene 609 |
| Gm45774 | NA | NA | NA |
| Efcab10 | 75040 | Efcab10 | EF-hand calcium binding domain 10 |
| Lrrc9 | 78257 | Lrrc9 | leucine rich repeat containing 9 |
| Gm17055 | NA | NA | NA |

| | | | |
|---|---|---|---|
| Pcdh20 | 219257 | Pcdh20 | protocadherin 20 |
| Ly6g6c | 68468 | Ly6g6c | "lymphocyte antigen 6 complex, locus G6C" |
| 9830107B12Rik | 328829 | 9830107B12Rik | RIKEN cDNA 9830107B12 gene |
| Klk11 | 56538 | Klk11 | kallikrein related-peptidase 11 |
| Crocc2 | 381284 | Crocc2 | "ciliary rootlet coiled-coil, rootletin family member 2" |
| Npcd | 504193 | Npcd | neuronal pentraxin chromo domain |
| Mmp9 | 17395 | Mmp9 | matrix metallopeptidase 9 |
| 1700095J03Rik | 74293 | 1700095J03Rik | RIKEN cDNA 1700095J03 gene |
| Calcoco2 | 76815 | Calcoco2 | calcium binding and coiled-coil domain 2 |
| Nr4a1 | 15370 | Nr4a1 | "nuclear receptor subfamily 4, group A, member 1" |
| Ggt6 | 71522 | Ggt6 | gamma-glutamyltransferase 6 |
| Eprn | 432777 | Eprn | "ephemeron, early developmental lncRNA" |
| 1810062G17Rik | 72282 | 1810062G17Rik | RIKEN cDNA 1810062G17 gene |
| Fabp1 | 14080 | Fabp1 | "fatty acid binding protein 1, liver" |
| Gabrb1 | 14400 | Gabrb1 | "gamma-aminobutyric acid (GABA) A receptor, subunit beta 1" |
| 1700061E17Rik | 100038395 | 1700061E17Rik | RIKEN cDNA 1700061E17 gene |
| Gm43923 | NA | NA | NA |
| Dner | 227325 | Dner | delta/notch-like EGF repeat containing |
| Rbfox3 | 52897 | Rbfox3 | "RNA binding protein, fox-1 homolog (C. elegans) 3" |
| Dnah5 | 110082 | Dnah5 | "dynein, axonemal, heavy chain 5" |
| Speer4c | 100502967 | Speer4c | spermatogenesis associated glutamate (E)-rich protein 4C |
| Gm27179 | NA | NA | NA |
| Gm5857 | 545572 | Gm5857 | predicted gene 5857 |
| Gm26841 | NA | NA | NA |
| Fabp3 | 14077 | Fabp3 | "fatty acid binding protein 3, muscle and heart" |

| | | | |
|---|---|---|---|
| Mgam | 232714 | Mgam | maltase-glucoamylase |
| A930012O16Rik | 227923 | A930012O16Rik | RIKEN cDNA A930012O16 gene |
| Gm7324 | 652988 | Gm7324 | predicted gene 7324 |
| Gm48583 | NA | NA | NA |
| Jhy | 70989 | Jhy | junctional cadherin complex regulator |
| Calml3 | 70405 | Calml3 | calmodulin-like 3 |
| AI463170 | 100504549 | AI463170 | expressed sequence AI463170 |
| Ifitm6 | 213002 | Ifitm6 | interferon induced transmembrane protein 6 |
| AC113491.1 | NA | NA | NA |
| Cntn1 | 12805 | Cntn1 | contactin 1 |
| Ccna1 | 12427 | Ccna1 | cyclin A1 |
| Lgals12 | 56072 | Lgals12 | "lectin, galactose binding, soluble 12" |
| Gm45713 | NA | NA | NA |
| Hoxaas3 | 72628 | Hoxaas3 | Hoxa cluster antisense RNA 3 |
| A730035I17Rik | 102637492 | A730035I17Rik | RIKEN cDNA A730035I17 gene |
| Ptn | 19242 | Ptn | pleiotrophin |
| Tmem232 | 381107 | Tmem232 | transmembrane protein 232 |
| Ankrd35 | 213121 | Ankrd35 | ankyrin repeat domain 35 |
| Scg5 | 20394 | Scg5 | secretogranin V |
| Serpinb2 | 18788 | Serpinb2 | "serine (or cysteine) peptidase inhibitor, clade B, member 2" |
| Gm15408 | 100503307 | Gm15408 | predicted gene 15408 |
| Gm16188 | NA | NA | NA |
| Mirt2 | 100038659 | Mirt2 | myocardial infraction associated transcript 2 |
| Bbox1 | 170442 | Bbox1 | "butyrobetaine (gamma), 2-oxoglutarate dioxygenase 1 (gamma-butyrobetaine hydroxylase)" |
| Fez1 | 235180 | Fez1 | fasciculation and elongation protein zeta 1 (zygin I) |

| | | | |
|---|---|---|---|
| Styxl1 | 76571 | Styxl1 | serine/threonine/tyrosine interacting-like 1 |
| E230016M11Rik | 320172 | E230016M11Rik | RIKEN cDNA E230016M11 gene |
| Hspa5 | 14828 | Hspa5 | heat shock protein 5 |
| Spp1 | 20750 | Spp1 | secreted phosphoprotein 1 |
| Hspa8 | 15481 | Hspa8 | heat shock protein 8 |
| Ms4a7 | 109225 | Ms4a7 | "membrane-spanning 4-domains, subfamily A, member 7" |
| Lhfpl2 | 218454 | Lhfpl2 | lipoma HMGIC fusion partner-like 2 |
| Lcn2 | 16819 | Lcn2 | lipocalin 2 |
| Ccr5 | 12774 | Ccr5 | chemokine (C-C motif) receptor 5 |
| Slamf7 | 75345 | Slamf7 | SLAM family member 7 |
| Sdf2l1 | 64136 | Sdf2l1 | stromal cell-derived factor 2-like 1 |
| Clmp | 71566 | Clmp | CXADR-like membrane protein |
| Zranb3 | 226409 | Zranb3 | "zinc finger, RAN-binding domain containing 3" |
| Inhba | 16323 | Inhba | inhibin beta-A |
| Creld2 | 76737 | Creld2 | cysteine-rich with EGF-like domains 2 |
| C3ar1 | 12267 | C3ar1 | complement component 3a receptor 1 |
| Slc37a2 | 56857 | Slc37a2 | "solute carrier family 37 (glycerol-3-phosphate transporter), member 2" |
| Orm1 | 18405 | Orm1 | orosomucoid 1 |
| H2-M2 | 14990 | H2-M2 | "histocompatibility 2, M region locus 2" |
| Pla2g2d | 18782 | Pla2g2d | "phospholipase A2, group IID" |
| Rasl11a | 68895 | Rasl11a | "RAS-like, family 11, member A" |
| Cfb | 14962 | Cfb | complement factor B |
| C1qb | 12260 | C1qb | "complement component 1, q subcomponent, beta polypeptide" |
| Mmp19 | 58223 | Mmp19 | matrix metallopeptidase 19 |
| C1qc | 12262 | C1qc | "complement component 1, q subcomponent, C chain" |

| | | | |
|---|---|---|---|
| Ctsk | 13038 | Ctsk | cathepsin K |
| C1qa | 12259 | C1qa | "complement component 1, q subcomponent, alpha polypeptide" |
| Gla | 11605 | Gla | "galactosidase, alpha" |
| Ncapg2 | 76044 | Ncapg2 | "non-SMC condensin II complex, subunit G2" |
| Ms4a6d | 68774 | Ms4a6d | "membrane-spanning 4-domains, subfamily A, member 6D" |
| 4930430E12Rik | 71639 | 4930430E12Rik | RIKEN cDNA 4930430E12 gene |
| Npy | 109648 | Npy | neuropeptide Y |
| Stra6l | 74152 | Stra6l | STRA6-like |
| Tnfrsf9 | 21942 | Tnfrsf9 | "tumor necrosis factor receptor superfamily, member 9" |
| Ahsa2 | 268390 | Ahsa2 | "AHA1, activator of heat shock protein ATPase 2" |
| Top2a | 21973 | Top2a | topoisomerase (DNA) II alpha |
| Itgam | 16409 | Itgam | integrin alpha M |
| Mki67 | 17345 | Mki67 | antigen identified by monoclonal antibody Ki 67 |
| Dcstamp | 75766 | Dcstamp | dendrocyte expressed seven transmembrane protein |
| Trem2 | 83433 | Trem2 | triggering receptor expressed on myeloid cells 2 |
| Acod1 | 16365 | Acod1 | aconitate decarboxylase 1 |
| Igkv11-125 | 243428 | Igkv11-125 | immunoglobulin kappa variable 11-125 |
| Ccl8 | 20307 | Ccl8 | chemokine (C-C motif) ligand 8 |
| Gas2l3 | 237436 | Gas2l3 | growth arrest-specific 2 like 3 |
| Atf3 | 11910 | Atf3 | activating transcription factor 3 |
| Pianp | 319352 | Pianp | PILR alpha associated neural protein |
| Lama1 | 16772 | Lama1 | "laminin, alpha 1" |
| Aoah | 27052 | Aoah | acyloxyacyl hydrolase |
| Slamf8 | 74748 | Slamf8 | SLAM family member 8 |
| Gpr162 | 14788 | Gpr162 | G protein-coupled receptor 162 |

| | | | |
|---|---|---|---|
| Rrm2 | 20135 | Rrm2 | ribonucleotide reductase M2 |
| Slpi | 20568 | Slpi | secretory leukocyte peptidase inhibitor |
| Apoe | 11816 | Apoe | apolipoprotein E |
| Il18bp | 16068 | Il18bp | interleukin 18 binding protein |
| Sgo2a | 68549 | Sgo2a | shugoshin 2A |
| Ccna2 | 12428 | Ccna2 | cyclin A2 |
| Gpnmb | 93695 | Gpnmb | glycoprotein (transmembrane) nmb |
| Apol7c | 108956 | Apol7c | apolipoprotein L 7c |
| Clec5a | 23845 | Clec5a | "C-type lectin domain family 5, member a" |
| Dnase1l3 | 13421 | Dnase1l3 | deoxyribonuclease 1-like 3 |
| AA467197 | 433470 | AA467197 | expressed sequence AA467197 |
| Rab3c | 67295 | Rab3c | "RAB3C, member RAS oncogene family" |
| A930007I19Rik | 77779 | A930007I19Rik | RIKEN cDNA A930007I19 gene |
| Kcnj10 | 16513 | Kcnj10 | "potassium inwardly-rectifying channel, subfamily J, member 10" |
| Slc11a1 | 18173 | Slc11a1 | "solute carrier family 11 (proton-coupled divalent metal ion transporters), member 1" |
| Olr1 | 108078 | Olr1 | oxidized low density lipoprotein (lectin-like) receptor 1 |
| E2f8 | 108961 | E2f8 | E2F transcription factor 8 |
| Cdca8 | 52276 | Cdca8 | cell division cycle associated 8 |
| Foxp3 | 20371 | Foxp3 | forkhead box P3 |
| Hmmr | 15366 | Hmmr | hyaluronan mediated motility receptor (RHAMM) |
| Hsph1 | 15505 | Hsph1 | heat shock 105kDa/110kDa protein 1 |
| Creg2 | 263764 | Creg2 | cellular repressor of E1A-stimulated genes 2 |
| Slfn9 | 237886 | Slfn9 | schlafen 9 |
| Hpse | 15442 | Hpse | heparanase |
| Gbp10 | 626578 | Gbp10 | guanylate-binding protein 10 |

| | | | |
|---|---|---|---|
| Lag3 | 16768 | Lag3 | lymphocyte-activation gene 3 |
| Birc5 | 11799 | Birc5 | baculoviral IAP repeat-containing 5 |
| Cenpf | 108000 | Cenpf | centromere protein F |
| Derl3 | 70377 | Derl3 | "Der1-like domain family, member 3" |
| Lonrf3 | 74365 | Lonrf3 | LON peptidase N-terminal domain and ring finger 3 |
| Hk3 | 212032 | Hk3 | hexokinase 3 |
| Gbp5 | 229898 | Gbp5 | guanylate binding protein 5 |
| Cenpe | 229841 | Cenpe | centromere protein E |
| Tigit | 100043314 | Tigit | T cell immunoreceptor with Ig and ITIM domains |
| Knl1 | 76464 | Knl1 | kinetochore scaffold 1 |
| Saa3 | 20210 | Saa3 | serum amyloid A 3 |
| Slc5a3 | 53881 | Slc5a3 | "solute carrier family 5 (inositol transporters), member 3" |
| Ube2c | 68612 | Ube2c | ubiquitin-conjugating enzyme E2C |
| Tgtp1 | 21822 | Tgtp1 | T cell specific GTPase 1 |
| Slc7a11 | 26570 | Slc7a11 | "solute carrier family 7 (cationic amino acid transporter, y+ system), member 11" |
| Col24a1 | 71355 | Col24a1 | "collagen, type XXIV, alpha 1" |
| Iigp1 | 60440 | Iigp1 | interferon inducible GTPase 1 |
| AI427809 | 381524 | AI427809 | expressed sequence AI427809 |
| Kif11 | 16551 | Kif11 | kinesin family member 11 |
| Prc1 | 233406 | Prc1 | protein regulator of cytokinesis 1 |
| Ccl5 | 20304 | Ccl5 | chemokine (C-C motif) ligand 5 |
| Cxcl9 | 17329 | Cxcl9 | chemokine (C-X-C motif) ligand 9 |
| Aif1 | 11629 | Aif1 | allograft inflammatory factor 1 |
| Ckap2l | 70466 | Ckap2l | cytoskeleton associated protein 2-like |
| Gbp8 | 76074 | Gbp8 | guanylate-binding protein 8 |

| | | | |
|---|---|---|---|
| Rgs16 | 19734 | Rgs16 | regulator of G-protein signaling 16 |
| Mreg | 381269 | Mreg | melanoregulin |
| Arntl | 11865 | Arntl | aryl hydrocarbon receptor nuclear translocator-like |
| Ubd | 24108 | Ubd | ubiquitin D |
| Gm4841 | 225594 | Gm4841 | predicted gene 4841 |
| Pla2g7 | 27226 | Pla2g7 | "phospholipase A2, group VII (platelet-activating factor acetylhydrolase, plasma)" |
| Cdk1 | 12534 | Cdk1 | cyclin-dependent kinase 1 |
| Fabp7 | 12140 | Fabp7 | "fatty acid binding protein 7, brain" |
| Uhrf1 | 18140 | Uhrf1 | "ubiquitin-like, containing PHD and RING finger domains, 1" |
| Basp1 | 70350 | Basp1 | "brain abundant, membrane attached signal protein 1" |
| Ccl3 | 20302 | Ccl3 | chemokine (C-C motif) ligand 3 |
| Tpx2 | 72119 | Tpx2 | "TPX2, microtubule-associated" |
| Nusap1 | 108907 | Nusap1 | nucleolar and spindle associated protein 1 |
| Gm6264 | 621835 | Gm6264 | predicted gene 6264 |
| Gm15601 | NA | NA | NA |
| F830016B08Rik | 240328 | F830016B08Rik | RIKEN cDNA F830016B08 gene |
| BC023105 | 667597 | BC023105 | cDNA sequence BC023105 |
| Foxm1 | 14235 | Foxm1 | forkhead box M1 |
| Tnip3 | 414084 | Tnip3 | TNFAIP3 interacting protein 3 |
| Arnt2 | 11864 | Arnt2 | aryl hydrocarbon receptor nuclear translocator 2 |
| Aspm | 12316 | Aspm | abnormal spindle microtubule assembly |
| Apoc2 | 11813 | Apoc2 | apolipoprotein C-II |
| Igkv8-24 | 677858 | Igkv8-24 | immunoglobulin kappa chain variable 8-24 |
| Spink2 | 69982 | Spink2 | "serine peptidase inhibitor, Kazal type 2" |
| Fam180a | 208164 | Fam180a | "family with sequence similarity 180, member A" |

| | | | |
|---|---|---|---|
| Asf1b | 66929 | Asf1b | anti-silencing function 1B histone chaperone |
| Rph3a | 19894 | Rph3a | rabphilin 3A |
| Pclaf | 68026 | Pclaf | PCNA clamp associated factor |
| Bhlha15 | 17341 | Bhlha15 | "basic helix-loop-helix family, member a15" |
| Gm29084 | NA | NA | NA |
| Gbp6 | 100702 | Gbp6 | guanylate binding protein 6 |
| Ksr2 | 333050 | Ksr2 | kinase suppressor of ras 2 |
| Gpr176 | 381413 | Gpr176 | G protein-coupled receptor 176 |
| Kntc1 | 208628 | Kntc1 | kinetochore associated 1 |
| Tm4sf19 | 277203 | Tm4sf19 | transmembrane 4 L six family member 19 |
| Gm9236 | 668552 | Gm9236 | predicted gene 9236 |
| Rad51 | 19361 | Rad51 | RAD51 recombinase |
| AU022793 | 105976 | AU022793 | expressed sequence AU022793 |
| Tlr12 | 384059 | Tlr12 | toll-like receptor 12 |
| Spc24 | 67629 | Spc24 | "SPC24, NDC80 kinetochore complex component, homolog (S. cerevisiae)" |
| Htr2b | 15559 | Htr2b | 5-hydroxytryptamine (serotonin) receptor 2B |
| Cxcl10 | 15945 | Cxcl10 | chemokine (C-X-C motif) ligand 10 |
| Kif22 | 110033 | Kif22 | kinesin family member 22 |
| Clec4d | 17474 | Clec4d | "C-type lectin domain family 4, member d" |
| Ncapg | 54392 | Ncapg | "non-SMC condensin I complex, subunit G" |
| Pla1a | 85031 | Pla1a | phospholipase A1 member A |
| Calhm6 | 215900 | Calhm6 | calcium homeostasis modulator family member 6 |
| Cilp | 214425 | Cilp | "cartilage intermediate layer protein, nucleotide pyrophosphohydrolase" |
| Pbk | 52033 | Pbk | PDZ binding kinase |
| Plk1 | 18817 | Plk1 | polo like kinase 1 |

| | | | |
|---|---|---|---|
| Igkv5-48 | 619846 | Igkv5-48 | immunoglobulin kappa variable 5-48 |
| Cep55 | 74107 | Cep55 | centrosomal protein 55 |
| Mmp12 | 17381 | Mmp12 | matrix metallopeptidase 12 |
| Gm15832 | NA | NA | NA |
| Gbp2 | 14469 | Gbp2 | guanylate binding protein 2 |
| Ndc80 | 67052 | Ndc80 | NDC80 kinetochore complex component |
| Trpc4 | 22066 | Trpc4 | "transient receptor potential cation channel, subfamily C, member 4" |
| Batf3 | 381319 | Batf3 | "basic leucine zipper transcription factor, ATF-like 3" |
| Kif20a | 19348 | Kif20a | kinesin family member 20A |
| Aurka | 20878 | Aurka | aurora kinase A |
| AC157784.1 | NA | NA | NA |
| Col5a3 | 53867 | Col5a3 | "collagen, type V, alpha 3" |
| Adora3 | 11542 | Adora3 | adenosine A3 receptor |
| Gm11906 | NA | NA | NA |
| Ighv2-6 | 630486 | Ighv2-6 | immunoglobulin heavy variable 2-6 |
| Prr11 | 270906 | Prr11 | proline rich 11 |
| Igkv9-129 | 692182 | Igkv9-129 | immunoglobulin kappa variable 9-129 |
| Ccl4 | 20303 | Ccl4 | chemokine (C-C motif) ligand 4 |
| Ly6i | 57248 | Ly6i | "lymphocyte antigen 6 complex, locus I" |
| Dlgap5 | 218977 | Dlgap5 | DLG associated protein 5 |
| Aurkb | 20877 | Aurkb | aurora kinase B |
| Dtl | 76843 | Dtl | denticleless E3 ubiquitin protein ligase |
| Ccnb1 | 268697 | Ccnb1 | cyclin B1 |
| Slc6a12 | 14411 | Slc6a12 | "solute carrier family 6 (neurotransmitter transporter, betaine/GABA), member 12" |
| Kif2c | 73804 | Kif2c | kinesin family member 2C |

| | | | |
|---|---|---|---|
| Fignl1 | 60530 | Fignl1 | fidgetin-like 1 |
| Gm38248 | NA | NA | NA |
| 5830416I19Rik | 74757 | 5830416I19Rik | RIKEN cDNA 5830416I19 gene |
| Kifc1 | 100502766 | Kifc1 | kinesin family member C1 |
| Ccl2 | 20296 | Ccl2 | chemokine (C-C motif) ligand 2 |
| 2010016I18Rik | 69206 | 2010016I18Rik | RIKEN cDNA 2010016I18 gene |
| Ccl12 | 20293 | Ccl12 | chemokine (C-C motif) ligand 12 |
| Ighv1-39 | 780891 | Ighv1-39 | immunoglobulin heavy variable 1-39 |
| Bnc1 | 12173 | Bnc1 | basonuclin 1 |
| Knstrn | 51944 | Knstrn | kinetochore-localized astrin/SPAG5 binding |
| Tgtp2 | 100039796 | Tgtp2 | T cell specific GTPase 2 |
| Cdca3 | 14793 | Cdca3 | cell division cycle associated 3 |
| Grem1 | 23892 | Grem1 | "gremlin 1, DAN family BMP antagonist" |
| Nuf2 | 66977 | Nuf2 | "NUF2, NDC80 kinetochore complex component" |
| Hspb1 | 15507 | Hspb1 | heat shock protein 1 |
| Ercc6l | 236930 | Ercc6l | excision repair cross-complementing rodent repair deficiency complementation group 6 like |
| Tnfsf8 | 21949 | Tnfsf8 | "tumor necrosis factor (ligand) superfamily, member 8" |
| Dock3 | 208869 | Dock3 | dedicator of cyto-kinesis 3 |
| Olfr111 | 545205 | Olfr111 | olfactory receptor 111 |
| Cenpi | 102920 | Cenpi | centromere protein I |
| Cpz | 242939 | Cpz | carboxypeptidase Z |
| Eef1akmt3 | 100504608 | Eef1akmt3 | EEF1A lysine methyltransferase 3 |
| AC119228.1 | NA | NA | NA |
| Npas2 | 18143 | Npas2 | neuronal PAS domain protein 2 |
| AC125149.3 | NA | NA | NA |

| | | | |
|---|---|---|---|
| Spc25 | 66442 | Spc25 | "SPC25, NDC80 kinetochore complex component, homolog (S. cerevisiae)" |
| Cip2a | 224171 | Cip2a | cell proliferation regulating inhibitor of protein phosphatase 2A |
| Lilrb4a | 14728 | Lilrb4a | "leukocyte immunoglobulin-like receptor, subfamily B, member 4A" |
| Iqgap3 | 404710 | Iqgap3 | IQ motif containing GTPase activating protein 3 |
| Kcnd1 | 16506 | Kcnd1 | "potassium voltage-gated channel, Shal-related family, member 1" |
| Ska3 | 219114 | Ska3 | spindle and kinetochore associated complex subunit 3 |
| Gm48348 | NA | NA | NA |
| Ccnf | 12449 | Ccnf | cyclin F |
| Hells | 15201 | Hells | "helicase, lymphoid specific" |
| Igkv8-30 | 384419 | Igkv8-30 | immunoglobulin kappa chain variable 8-30 |
| Gbp2b | 14468 | Gbp2b | guanylate binding protein 2b |
| Gtse1 | 29870 | Gtse1 | G two S phase expressed protein 1 |
| Esco2 | 71988 | Esco2 | establishment of sister chromatid cohesion N-acetyltransferase 2 |
| Socs1 | 12703 | Socs1 | suppressor of cytokine signaling 1 |
| Insl6 | 27356 | Insl6 | insulin-like 6 |
| Igkv1-117 | 16098 | Igkv1-117 | immunoglobulin kappa variable 1-117 |
| Ticrr | 77011 | Ticrr | TOPBP1-interacting checkpoint and replication regulator |
| Nags | 217214 | Nags | N-acetylglutamate synthase |
| Eaf2 | 106389 | Eaf2 | ELL associated factor 2 |
| Eln | 13717 | Eln | elastin |
| Cdc6 | 23834 | Cdc6 | cell division cycle 6 |
| Igtp | 16145 | Igtp | interferon gamma induced GTPase |
| Ms4a14 | 383435 | Ms4a14 | "membrane-spanning 4-domains, subfamily A, member 14" |
| Ighv1-19 | 382692 | Ighv1-19 | immunoglobulin heavy variable V1-19 |
| Tcrg-C2 | NA | NA | NA |

| | | | |
|---|---|---|---|
| Gm44423 | NA | NA | NA |
| Lta | 16992 | Lta | lymphotoxin A |
| 9330175E14Rik | 320377 | 9330175E14Rik | RIKEN cDNA 9330175E14 gene |
| Stil | 20460 | Stil | Scl/Tal1 interrupting locus |
| Fut1 | 14343 | Fut1 | fucosyltransferase 1 |
| Mir155hg | 100653389 | Mir155hg | Mir155 host gene (non-protein coding) |
| Mzb1 | 69816 | Mzb1 | marginal zone B and B1 cell-specific protein 1 |
| Mab21l3 | 242125 | Mab21l3 | mab-21-like 3 |
| Gm43041 | NA | NA | NA |
| Mir146 | 387164 | Mir146 | microRNA 146 |
| Depdc1a | 76131 | Depdc1a | DEP domain containing 1a |
| Apol6 | 71939 | Apol6 | apolipoprotein L 6 |
| Gm9864 | NA | NA | NA |
| Igkv6-15 | 108022 | Igkv6-15 | immunoglobulin kappa variable 6-15 |
| Cdca2 | 108912 | Cdca2 | cell division cycle associated 2 |
| Nuggc | 100503545 | Nuggc | "nuclear GTPase, germinal center associated" |
| Ighv1-55 | 780932 | Ighv1-55 | immunoglobulin heavy variable 1-55 |
| Cst7 | 13011 | Cst7 | cystatin F (leukocystatin) |
| Ighv8-8 | 780938 | Ighv8-8 | immunoglobulin heavy variable 8-8 |
| Ccl19 | 24047 | Ccl19 | chemokine (C-C motif) ligand 19 |
| Ighv8-9 | 432709 | Ighv8-9 | immunoglobulin heavy variable V8-9 |
| Gpr85 | 64450 | Gpr85 | G protein-coupled receptor 85 |
| Mis18bp1 | 217653 | Mis18bp1 | MIS18 binding protein 1 |
| Ptx3 | 19288 | Ptx3 | pentraxin related gene |
| Gm20056 | 100504089 | Gm20056 | "predicted gene, 20056" |

| | | | |
|---|---|---|---|
| Cxcl3 | 330122 | Cxcl3 | chemokine (C-X-C motif) ligand 3 |
| Shcbp1 | 20419 | Shcbp1 | Shc SH2-domain binding protein 1 |
| Trip13 | 69716 | Trip13 | thyroid hormone receptor interactor 13 |
| Gdf15 | 23886 | Gdf15 | growth differentiation factor 15 |
| Ppm1n | 232941 | Ppm1n | "protein phosphatase, Mg2+/Mn2+ dependent, 1N (putative)" |
| Hist2h3c2 | NA | NA | NA |
| Ighv1-7 | 668474 | Ighv1-7 | immunoglobulin heavy variable V1-7 |
| Ighv9-3 | 780825 | Ighv9-3 | immunoglobulin heavy variable V9-3 |
| Gm42372 | 105247240 | Gm42372 | "predicted gene, 42372" |
| Tubb3 | 22152 | Tubb3 | "tubulin, beta 3 class III" |
| Hist2h4 | NA | NA | NA |
| Tspan10 | 208634 | Tspan10 | tetraspanin 10 |
| Il4i1 | 14204 | Il4i1 | interleukin 4 induced 1 |
| Pdcd1lg2 | 58205 | Pdcd1lg2 | programmed cell death 1 ligand 2 |
| Gen1 | 209334 | Gen1 | "GEN1, Holliday junction 5' flap endonuclease" |
| E230032D23Rik | 414103 | E230032D23Rik | RIKEN cDNA E230032D23 gene |
| Klrb1a | 17057 | Klrb1a | killer cell lectin-like receptor subfamily B member 1A |
| Gm38312 | NA | NA | NA |
| Ear6 | 93719 | Ear6 | "eosinophil-associated, ribonuclease A family, member 6" |
| Has2 | 15117 | Has2 | hyaluronan synthase 2 |
| Ifng | 15978 | Ifng | interferon gamma |
| Gm20045 | 100504072 | Gm20045 | "predicted gene, 20045" |
| Ighv1-34 | 628614 | Ighv1-34 | immunoglobulin heavy variable 1-34 |
| Gm43388 | NA | NA | NA |
| Syt2 | 20980 | Syt2 | synaptotagmin II |

| | | | |
|---|---|---|---|
| P4ha3 | 320452 | P4ha3 | "procollagen-proline, 2-oxoglutarate 4-dioxygenase (proline 4-hydroxylase), alpha polypeptide III" |
| Cdca5 | 67849 | Cdca5 | cell division cycle associated 5 |
| Dkk2 | 56811 | Dkk2 | dickkopf WNT signaling pathway inhibitor 2 |
| Msc | 17681 | Msc | musculin |
| Scn2b | 72821 | Scn2b | "sodium channel, voltage-gated, type II, beta" |
| Igkv4-57 | 235952 | Igkv4-57 | immunoglobulin kappa variable 4-57 |
| Tnf | 21926 | Tnf | tumor necrosis factor |
| Ighv5-16 | 633568 | Ighv5-16 | immunoglobulin heavy variable 5-16 |
| Gm38279 | NA | NA | NA |
| Igkv4-74 | 236047 | Igkv4-74 | immunoglobulin kappa variable 4-74 |
| Igkv4-57-1 | 384514 | Igkv4-57-1 | immunoglobulin kappa variable 4-57-1 |
| AC166110.3 | NA | NA | NA |
| Ighv1-18 | 629871 | Ighv1-18 | immunoglobulin heavy variable V1-18 |
| Ighv2-2 | 777686 | Ighv2-2 | immunoglobulin heavy variable 2-2 |
| Mastl | 67121 | Mastl | microtubule associated serine/threonine kinase-like |
| Pif1 | 208084 | Pif1 | PIF1 5'-to-3' DNA helicase |
| Ighv2-9-1 | 791089 | Ighv2-9-1 | immunoglobulin heavy variable 2-9-1 |
| Il21 | 60505 | Il21 | interleukin 21 |
| Spdl1 | 70385 | Spdl1 | spindle apparatus coiled-coil protein 1 |
| Gm13571 | 102636093 | Gm13571 | predicted gene 13571 |
| Ighv1-66 | 380824 | Ighv1-66 | immunoglobulin heavy variable 1-66 |
| Gm43511 | NA | NA | NA |
| Gm43302 | NA | NA | NA |
| Nlrp1c-ps | 627984 | Nlrp1c-ps | "NLR family, pyrin domain containing 1C, pseudogene" |
| Fgl1 | 234199 | Fgl1 | fibrinogen-like protein 1 |

| | | | |
|---|---|---|---|
| Gm43802 | NA | NA | NA |
| Igkv14-126 | 628127 | Igkv14-126 | immunoglobulin kappa variable 14-126 |
| 2810429I04Rik | 76937 | 2810429I04Rik | RIKEN cDNA 2810429I04 gene |
| Hist1h4j | NA | NA | NA |
| Orm2 | 18406 | Orm2 | orosomucoid 2 |
| Tvp23a | 383103 | Tvp23a | trans-golgi network vesicle protein 23A |
| Igkv12-46 | 692245 | Igkv12-46 | immunoglobulin kappa variable 12-46 |
| Trav4-3 | 100126471 | Trav4-3 | T cell receptor alpha variable 4-3 |
| Ccdc18 | 73254 | Ccdc18 | coiled-coil domain containing 18 |
| Traip | 22036 | Traip | TRAF-interacting protein |
| Gm37795 | NA | NA | NA |
| Ighv14-2 | 668421 | Ighv14-2 | immunoglobulin heavy variable 14-2 |
| Igkv6-17 | 667865 | Igkv6-17 | immunoglobulin kappa variable 6-17 |
| Igkv4-61 | 546244 | Igkv4-61 | immunoglobulin kappa chain variable 4-61 |
| F730311O21Rik | 622304 | F730311O21Rik | RIKEN cDNA F730311O21 gene |
| Slc32a1 | 22348 | Slc32a1 | "solute carrier family 32 (GABA vesicular transporter), member 1" |
| Ighv1-9 | 668478 | Ighv1-9 | immunoglobulin heavy variable V1-9 |
| Mmp13 | 17386 | Mmp13 | matrix metallopeptidase 13 |
| Gm18734 | 100417645 | Gm18734 | "predicted gene, 18734" |
| Art2a-ps | NA | NA | NA |
| Gm37893 | NA | NA | NA |
| Cdh17 | 12557 | Cdh17 | cadherin 17 |
| Trpm2 | 28240 | Trpm2 | "transient receptor potential cation channel, subfamily M, member 2" |
| Ttk | 22137 | Ttk | Ttk protein kinase |
| Mtfr2 | 71804 | Mtfr2 | mitochondrial fission regulator 2 |

| | | | |
|---|---|---|---|
| Gm45838 | NA | NA | NA |
| Gdap10 | 100504486 | Gdap10 | ganglioside-induced differentiation-associated-protein 10 |
| Fbxo48 | 319701 | Fbxo48 | F-box protein 48 |
| Fut2 | 14344 | Fut2 | fucosyltransferase 2 |
| Igkv2-109 | 628268 | Igkv2-109 | immunoglobulin kappa variable 2-109 |
| Gm19221 | 100418453 | Gm19221 | "predicted gene, 19221" |
| Cdh24 | 239096 | Cdh24 | cadherin-like 24 |
| Serpina3f | 238393 | Serpina3f | "serine (or cysteine) peptidase inhibitor, clade A, member 3F" |
| Ighv1-5 | 668469 | Ighv1-5 | immunoglobulin heavy variable V1-5 |
| Ackr1 | 13349 | Ackr1 | atypical chemokine receptor 1 (Duffy blood group) |
| Ighv1-26 | 629884 | Ighv1-26 | immunoglobulin heavy variable 1-26 |
| Cabp4 | 73660 | Cabp4 | calcium binding protein 4 |
| Gm44861 | NA | NA | NA |
| Gm43294 | NA | NA | NA |
| Gm12708 | 105244624 | Gm12708 | predicted gene 12708 |
| Olfr60 | 18361 | Olfr60 | olfactory receptor 60 |
| Ighv1-80 | 668589 | Ighv1-80 | immunoglobulin heavy variable 1-80 |
| Ighv14-3 | 238418 | Ighv14-3 | immunoglobulin heavy variable V14-3 |
| Gm38190 | NA | NA | NA |
| AC129188.1 | NA | NA | NA |
| Ighv1-59 | 432708 | Ighv1-59 | immunoglobulin heavy variable V1-59 |
| Gm26522 | NA | NA | NA |
| Mkx | 210719 | Mkx | mohawk homeobox |
| Dgkk | 331374 | Dgkk | diacylglycerol kinase kappa |
| Igkv1-99 | 434028 | Igkv1-99 | immunoglobulin kappa variable 1-99 |

| | | | |
|---|---|---|---|
| Gm37303 | NA | NA | NA |
| Igf2bp3 | 140488 | Igf2bp3 | insulin-like growth factor 2 mRNA binding protein 3 |
| Gdf3 | 14562 | Gdf3 | growth differentiation factor 3 |
| Ankrd61 | 66729 | Ankrd61 | ankyrin repeat domain 61 |
| Gm48132 | NA | NA | NA |
| Arhgef39 | 230098 | Arhgef39 | Rho guanine nucleotide exchange factor (GEF) 39 |
| Gm46224 | 108167810 | Gm46224 | "predicted gene, 46224" |
| Ighv1-76 | 100775174 | Ighv1-76 | immunoglobulin heavy variable 1-76 |
| Cenpm | 66570 | Cenpm | centromere protein M |
| Gpr31b | 436440 | Gpr31b | "G protein-coupled receptor 31, D17Leh66b region" |
| Gm45684 | NA | NA | NA |
| 6430511E19Rik | 320056 | 6430511E19Rik | RIKEN cDNA 6430511E19 gene |
| Igkv1-110 | 381777 | Igkv1-110 | immunoglobulin kappa variable 1-110 |
| Vash2 | 226841 | Vash2 | vasohibin 2 |
| Pdyn | 18610 | Pdyn | prodynorphin |
| Gm14434 | 668039 | Gm14434 | predicted gene 14434 |
| Thbs4 | 21828 | Thbs4 | thrombospondin 4 |
| Cenph | 26886 | Cenph | centromere protein H |
| Ighv8-13 | 100775172 | Ighv8-13 | immunoglobulin heavy variable 8-13 |
| Gm47171 | NA | NA | NA |
| Hspa1b | 15511 | Hspa1b | heat shock protein 1B |
| Gpr82 | 319200 | Gpr82 | G protein-coupled receptor 82 |
| Hist1h4n | 319161 | Hist1h4n | "histone cluster 1, H4n" |
| Gm43980 | NA | NA | NA |
| Gm2694 | 100040294 | Gm2694 | predicted gene 2694 |

| | | | |
|---|---|---|---|
| Neil3 | 234258 | Neil3 | nei like 3 (E. coli) |
| Rnase2a | 93726 | Rnase2a | "ribonuclease, RNase A family, 2A (liver, eosinophil-derived neurotoxin)" |
| Igkv6-25 | 381784 | Igkv6-25 | immunoglobulin kappa chain variable 6-25 |
| Rgs8 | 67792 | Rgs8 | regulator of G-protein signaling 8 |
| Eme1 | 268465 | Eme1 | essential meiotic structure-specific endonuclease 1 |
| Ighv1-36 | 629897 | Ighv1-36 | immunoglobulin heavy variable 1-36 |
| Gjb4 | 14621 | Gjb4 | "gap junction protein, beta 4" |
| Igkv6-23 | 637227 | Igkv6-23 | immunoglobulin kappa variable 6-23 |
| Nlgn1 | 192167 | Nlgn1 | neuroligin 1 |
| Prss16 | 54373 | Prss16 | "protease, serine 16 (thymus)" |
| Gm42793 | NA | NA | NA |
| Igkv10-94 | 667550 | Igkv10-94 | immunoglobulin kappa variable 10-94 |
| Igkv3-10 | 667924 | Igkv3-10 | immunoglobulin kappa variable 3-10 |
| Gm26745 | NA | NA | NA |
| Prss35 | 244954 | Prss35 | "protease, serine 35" |
| Rgs9bp | 243923 | Rgs9bp | regulator of G-protein signalling 9 binding protein |
| A630023P12Rik | 231603 | A630023P12Rik | RIKEN cDNA A630023P12 gene |
| Impg2 | 224224 | Impg2 | interphotoreceptor matrix proteoglycan 2 |
| Hal | 15109 | Hal | histidine ammonia lyase |
| Sfrp2 | 20319 | Sfrp2 | secreted frizzled-related protein 2 |
| Gm26807 | NA | NA | NA |
| Gm45518 | NA | NA | NA |
| Gm47406 | NA | NA | NA |
| Gm15542 | 100043546 | Gm15542 | predicted gene 15542 |
| Ccl7 | 20306 | Ccl7 | chemokine (C-C motif) ligand 7 |

| | | | |
|---|---|---|---|
| Hsd11b2 | 15484 | Hsd11b2 | hydroxysteroid 11-beta dehydrogenase 2 |
| Gm37944 | NA | NA | NA |
| Gm34643 | 102637961 | Gm34643 | "predicted gene, 34643" |
| Igkv12-41 | 619960 | Igkv12-41 | immunoglobulin kappa chain variable 12-41 |
| Igkv17-121 | 667435 | Igkv17-121 | immunoglobulin kappa variable 17-121 |
| 1700009J07Rik | 75188 | 1700009J07Rik | RIKEN cDNA 1700009J07 gene |
| Gm30211 | 102632036 | Gm30211 | "predicted gene, 30211" |
| Pvrig | 102640920 | Pvrig | poliovirus receptor related immunoglobulin domain containing |
| Gm16206 | 102632669 | Gm16206 | predicted gene 16206 |
| Prg4 | 96875 | Prg4 | "proteoglycan 4 (megakaryocyte stimulating factor, articular superficial zone protein)" |
| Gm37204 | NA | NA | NA |
| CR974586.5 | NA | NA | NA |
| Cxcl5 | 20311 | Cxcl5 | chemokine (C-X-C motif) ligand 5 |
| Gm49125 | NA | NA | NA |
| Ighv8-2 | 629849 | Ighv8-2 | immunoglobulin heavy variable V8-2 |
| Serpina3h | 546546 | Serpina3h | "serine (or cysteine) peptidase inhibitor, clade A, member 3H" |
| Gm40932 | 105245484 | Gm40932 | "predicted gene, 40932" |
| Pcdh15 | 11994 | Pcdh15 | protocadherin 15 |
| Ighv1-82 | 100775175 | Ighv1-82 | immunoglobulin heavy variable 1-82 |
| Gm44873 | NA | NA | NA |
| Frzb | 20378 | Frzb | frizzled-related protein |
| Rad51ap1 | 19362 | Rad51ap1 | RAD51 associated protein 1 |
| Gbp11 | 634650 | Gbp11 | guanylate binding protein 11 |
| Gm38244 | NA | NA | NA |
| Igkv16-104 | 381778 | Igkv16-104 | immunoglobulin kappa variable 16-104 |

| | | | |
|---|---|---|---|
| Cyp1a1 | 13076 | Cyp1a1 | "cytochrome P450, family 1, subfamily a, polypeptide 1" |
| Chek1 | 12649 | Chek1 | checkpoint kinase 1 |
| Clca3a2 | 80797 | Clca3a2 | chloride channel accessory 3A2 |
| Col28a1 | 213945 | Col28a1 | "collagen, type XXVIII, alpha 1" |
| Gm29291 | NA | NA | NA |
| Ighv1-81 | 668591 | Ighv1-81 | immunoglobulin heavy variable 1-81 |
| Ido1 | 15930 | Ido1 | "indoleamine 2,3-dioxygenase 1" |
| Gm44567 | NA | NA | NA |
| Gm16026 | 101056250 | Gm16026 | predicted pseudogene 16026 |
| Ptk6 | 20459 | Ptk6 | PTK6 protein tyrosine kinase 6 |
| Tff1 | 21784 | Tff1 | trefoil factor 1 |
| Gm12480 | 100041303 | Gm12480 | predicted gene 12480 |
| Clgn | 12745 | Clgn | calmegin |
| Pimreg | 109212 | Pimreg | PICALM interacting mitotic regulator |
| G530011O06Rik | 654820 | G530011O06Rik | RIKEN cDNA G530011O06 gene |
| Fcrlb | 435653 | Fcrlb | Fc receptor-like B |
| Gm10863 | 100041655 | Gm10863 | predicted gene 10863 |
| Cxcl11 | 56066 | Cxcl11 | chemokine (C-X-C motif) ligand 11 |
| Kbtbd12 | 74589 | Kbtbd12 | kelch repeat and BTB (POZ) domain containing 12 |
| Gm16199 | NA | NA | NA |
| Hist1h3d | NA | NA | NA |
| Rln3 | 212108 | Rln3 | relaxin 3 |
| Lockd | 381822 | Lockd | lncRNA downstream of Cdkn1b |
| Gm43513 | NA | NA | NA |
| Gm37004 | 115489463 | Gm37004 | "predicted gene, 37004" |

| | | | |
|---|---|---|---|
| Gm36989 | NA | NA | NA |
| Cxcl1 | 14825 | Cxcl1 | chemokine (C-X-C motif) ligand 1 |
| Gm37652 | NA | NA | NA |
| Btnl4 | 632126 | Btnl4 | butyrophilin-like 4 |
| Enthd1 | 383075 | Enthd1 | ENTH domain containing 1 |
| Fbn2 | 14119 | Fbn2 | fibrillin 2 |
| Olfr1152 | 258103 | Olfr1152 | olfactory receptor 1152 |
| Gm44175 | NA | NA | NA |
| Ska1 | 66468 | Ska1 | spindle and kinetochore associated complex subunit 1 |
| Gm42741 | NA | NA | NA |
| Igkv4-51 | 619806 | Igkv4-51 | immunoglobulin kappa chain variable 4-51 |
| Gm12187 | 100502678 | Gm12187 | predicted gene 12187 |
| Igkv4-86 | 243451 | Igkv4-86 | immunoglobulin kappa variable 4-86 |
| Igkv1-135 | 243420 | Igkv1-135 | immunoglobulin kappa variable 1-135 |
| A530030E21Rik | 320731 | A530030E21Rik | RIKEN cDNA A530030E21 gene |
| Ighg1 | 16017 | Ighg1 | immunoglobulin heavy constant gamma 1 (G1m marker) |
| Ighg2c | 404711 | Ighg2c | immunoglobulin heavy constant gamma 2C |
| Cdc25c | 12532 | Cdc25c | cell division cycle 25C |
| Sucnr1 | 84112 | Sucnr1 | succinate receptor 1 |
| Gm15298 | NA | NA | NA |
| Gm47994 | NA | NA | NA |
| Ighv1-77 | 619994 | Ighv1-77 | immunoglobulin heavy variable 1-77 |
| A430093F15Rik | 403202 | A430093F15Rik | RIKEN cDNA A430093F15 gene |
| Fbln7 | 70370 | Fbln7 | fibulin 7 |
| Cxcr1 | 227288 | Cxcr1 | chemokine (C-X-C motif) receptor 1 |

| | | | |
|---|---|---|---|
| Igkv3-5 | 667940 | Igkv3-5 | immunoglobulin kappa chain variable 3-5 |
| Gm37296 | NA | NA | NA |
| Ighv2-5 | 638605 | Ighv2-5 | immunoglobulin heavy variable 2-5 |
| D7Ertd128e | 52222 | D7Ertd128e | "DNA segment, Chr 7, ERATO Doi 128, expressed" |
| AC124237.1 | NA | NA | NA |
| 5430437J10Rik | 71432 | 5430437J10Rik | RIKEN cDNA 5430437J10 gene |
| Gm10603 | 100038497 | Gm10603 | predicted gene 10603 |

## Table S5-5. Integrative metabolic pathways enrichment analyses between Luz vs Mel group.

| Metabolite name | Total | Expected | Hits | Raw p | -LOG10(p) | FDR | Impact | matched_features |
|---|---|---|---|---|---|---|---|---|
| Aminoacyl-tRNA biosynthesis | 74 | 1.2721 | 9 | 2.57E-06 | 5.5902 | 0.00021582 | 0.13699 | cpd:C00152; cpd:C00079; cpd:C00065; cpd:C00073; cpd:C00041; cpd:C00407; cpd:C00188; cpd:C00082; cpd:C00148 |
| Alanine, aspartate and glutamate metabolism | 61 | 1.0486 | 7 | 5.74E-05 | 4.2411 | 0.0024108 | 0.28333 | cpd:C01042; cpd:C00152; cpd:C00041; cpd:C00158; cpd:C00438; mmu:269642; mmu:14204 |
| Phenylalanine, tyrosine and tryptophan biosynthesis | 11 | 0.1891 | 3 | 0.0007075 | 3.1503 | 0.019574 | 1.6 | cpd:C00079; cpd:C00082; mmu:14204 |
| Valine, leucine and isoleucine biosynthesis | 12 | 0.20629 | 3 | 0.00093208 | 3.0305 | 0.019574 | 0.90909 | cpd:C00188; cpd:C00407; mmu:12035 |
| Neomycin, kanamycin and gentamicin biosynthesis | 4 | 0.068763 | 2 | 0.0016931 | 2.7713 | 0.028444 | 1.3333 | cpd:C00031; mmu:212032 |
| Phenylalanine metabolism | 24 | 0.41258 | 3 | 0.0074296 | 2.129 | 0.10401 | 0.43478 | cpd:C00079; cpd:C00082; mmu:14204 |
| Cysteine and methionine metabolism | 71 | 1.2205 | 4 | 0.031845 | 1.497 | 0.38214 | 0.2 | cpd:C00065; cpd:C00073; mmu:14204; mmu:12035 |
| Arginine and proline metabolism | 78 | 1.3409 | 4 | 0.042898 | 1.3676 | 0.45043 | 0.20779 | cpd:C01157; cpd:C00148; cpd:C00077; mmu:18126 |
| Mucin type O-glycan biosynthesis | 22 | 0.3782 | 2 | 0.053625 | 1.2706 | 0.4667 | 0.14286 | mmu:241391; mmu:271786 |
| Galactose metabolism | 51 | 0.87673 | 3 | 0.05556 | 1.2552 | 0.4667 | 0.18 | cpd:C00031; cpd:C01697; mmu:212032 |
| Arginine biosynthesis | 27 | 0.46415 | 2 | 0.077238 | 1.1122 | 0.58982 | 0.26923 | cpd:C00077; mmu:18126 |
| Starch and sucrose metabolism | 37 | 0.63606 | 2 | 0.13181 | 0.88004 | 0.92269 | 0.41667 | cpd:C00031; mmu:212032 |
| Tryptophan metabolism | 84 | 1.444 | 3 | 0.17345 | 0.76083 | 1 | 0.18072 | cpd:C00780; mmu:15930; mmu:14204 |
| Valine, leucine and isoleucine degradation | 88 | 1.5128 | 3 | 0.19083 | 0.71935 | 1 | 0.1954 | cpd:C00407; mmu:12035; mmu:14204 |
| Glutathione metabolism | 56 | 0.96268 | 2 | 0.25013 | 0.60184 | 1 | 0.090909 | cpd:C00077; mmu:20135 |
| Glyoxylate and dicarboxylate metabolism | 56 | 0.96268 | 2 | 0.25013 | 0.60184 | 1 | 0.10909 | cpd:C00158; cpd:C00065 |
| Ubiquinone and other terpenoid-quinone biosynthesis | 17 | 0.29224 | 1 | 0.25605 | 0.59167 | 1 | 0.0625 | cpd:C00082 |
| Glycolysis or Gluconeogenesis | 61 | 1.0486 | 2 | 0.28241 | 0.54913 | 1 | 0.13333 | mmu:13808; mmu:212032 |
| Purine metabolism | 169 | 2.9052 | 4 | 0.33031 | 0.48108 | 1 | 0.1131 | cpd:C00212; cpd:C00366; mmu:20135; mmu:432530 |
| Glycine, serine and threonine metabolism | 72 | 1.2377 | 2 | 0.35291 | 0.45233 | 1 | 0.26761 | cpd:C00065; cpd:C00188 |
| Glycosphingolipid biosynthesis - globo and isoglobo series | 31 | 0.53291 | 1 | 0.41783 | 0.379 | 1 | 0.13333 | mmu:14344 |
| One carbon pool by folate | 31 | 0.53291 | 1 | 0.41783 | 0.379 | 1 | 0.26667 | mmu:17768 |
| Pantothenate and CoA biosynthesis | 34 | 0.58449 | 1 | 0.44773 | 0.34898 | 1 | 0.12121 | mmu:12035 |
| Tyrosine metabolism | 88 | 1.5128 | 2 | 0.45106 | 0.34577 | 1 | 0.14943 | cpd:C00082; mmu:14204 |
| Selenocompound metabolism | 35 | 0.60168 | 1 | 0.45736 | 0.33974 | 1 | 0.029412 | cpd:C00041 |
| Glycerolipid metabolism | 35 | 0.60168 | 1 | 0.45736 | 0.33974 | 1 | 0.17647 | mmu:116939 |
| Fructose and mannose metabolism | 37 | 0.63606 | 1 | 0.47614 | 0.32227 | 1 | 0.11111 | mmu:212032 |
| Ether lipid metabolism | 39 | 0.67044 | 1 | 0.49428 | 0.30603 | 1 | 0.052632 | mmu:27226 |

| | | | | | | | | |
|---|---|---|---|---|---|---|---|---|
| Pyrimidine metabolism | 99 | 1.7019 | 2 | 0.51378 | 0.28922 | 1 | 0.071429 | cpd:C00438; mmu:20135 |
| Citrate cycle (TCA cycle) | 42 | 0.72201 | 1 | 0.52034 | 0.28371 | 1 | 0.14634 | cpd:C00158 |
| Glycosaminoglycan degradation | 44 | 0.75639 | 1 | 0.53698 | 0.27004 | 1 | 0.13953 | mmu:15442 |
| Pyruvate metabolism | 45 | 0.77358 | 1 | 0.54509 | 0.26353 | 1 | 0.045455 | cpd:C00256 |
| Propanoate metabolism | 48 | 0.82516 | 1 | 0.5686 | 0.24519 | 1 | 0.042553 | cpd:C05984 |
| Porphyrin and chlorophyll metabolism | 53 | 0.91111 | 1 | 0.60517 | 0.21812 | 1 | 0.11538 | mmu:15368 |
| Sphingolipid metabolism | 58 | 0.99706 | 1 | 0.6387 | 0.1947 | 1 | 0.017544 | cpd:C00065 |
| Drug metabolism - other enzymes | 69 | 1.1862 | 1 | 0.703 | 0.15304 | 1 | 0.029412 | mmu:20135 |
| Phosphatidylinositol signaling system | 74 | 1.2721 | 1 | 0.7284 | 0.13763 | 1 | 0.054795 | mmu:271424 |
| Amino sugar and nucleotide sugar metabolism | 81 | 1.3925 | 1 | 0.76042 | 0.11895 | 1 | 0.125 | mmu:212032 |
| Steroid biosynthesis | 82 | 1.4096 | 1 | 0.76469 | 0.11652 | 1 | 0.049383 | mmu:223920 |
| Glycerophospholipid metabolism | 86 | 1.4784 | 1 | 0.78101 | 0.10734 | 1 | 0.035294 | cpd:C00189 |
| Glycosphingolipid biosynthesis - lacto and neolacto series | 102 | 1.7535 | 1 | 0.83595 | 0.077818 | 1 | 0.13861 | mmu:14344 |

## Table S5-6. Annotation of metabolites and genes between Luz vs Mel group.

| Gene | Entrez | Symbol | Name | Metabolite KEGG ID | Name |
|---|---|---|---|---|---|
| Ms4a7 | 109225 | Ms4a7 | "membrane-spanning 4-domains, subfamily A, member 7" | C00490 | Itaconic acid |
| Ccl8 | 20307 | Ccl8 | chemokine (C-C motif) ligand 8 | C01835 | Maltotriose |
| Saa3 | 20210 | Saa3 | serum amyloid A 3 | C02226 | Citraconic acid |
| C1qa | 12259 | C1qa | "complement component 1, q subcomponent, alpha polypeptide" | C00152 | L-Asparagine |
| C1qb | 12260 | C1qb | "complement component 1, q subcomponent, beta polypeptide" | C05984 | 2-Hydroxybutyric acid |
| AA467197 | 433470 | AA467197 | expressed sequence AA467197 | C00366 | Uric acid |
| C1qc | 12262 | C1qc | "complement component 1, q subcomponent, C chain" | C02896 | Putrescine |
| Lat2 | 56743 | Lat2 | "linker for activation of T cells family, member 2" | C01042 | N-Acetyl-L-aspartic acid |
| Apoc2 | 11813 | Apoc2 | apolipoprotein C-II | C00031 | D-Glucose |
| Top2a | 21973 | Top2a | topoisomerase (DNA) II alpha | C03661 | 1-Kestose |
| Hpse | 15442 | Hpse | heparanase | C01770 | Gamma-Butyrolactone |
| Basp1 | 70350 | Basp1 | "brain abundant, membrane attached signal protein 1" | C00065 | L-Serine |
| Aif1 | 11629 | Aif1 | allograft inflammatory factor 1 | C00407 | L-Isoleucine |
| Mki67 | 17345 | Mki67 | antigen identified by monoclonal antibody Ki 67 | C00073 | L-Methionine |
| Rnf128 | 66889 | Rnf128 | ring finger protein 128 | C00148 | L-Proline |
| Itgam | 16409 | Itgam | integrin alpha M | C01157 | 4-Hydroxyproline |
| Creg2 | 263764 | Creg2 | cellular repressor of E1A-stimulated genes 2 | C00079 | L-Phenylalanine |
| Aoah | 27052 | Aoah | acyloxyacyl hydrolase | C06562 | Catechin |
| Trem2 | 83433 | Trem2 | triggering receptor expressed on myeloid cells 2 | C05629 | Hydrocinnamic acid |
| Gpnmb | 93695 | Gpnmb | glycoprotein (transmembrane) nmb | C00188 | L-Threonine |
| 4930430E12Rik | 71639 | 4930430E12Rik | RIKEN cDNA 4930430E12 gene | C00438 | Ureidosuccinic acid |
| Acod1 | 16365 | Acod1 | aconitate decarboxylase 1 | C00158 | Citric acid |
| Clec4d | 17474 | Clec4d | "C-type lectin domain family 4, member d" | C00780 | Serotonin |
| Ccna2 | 12428 | Ccna2 | cyclin A2 | C00077 | Ornithine |
| Slamf8 | 74748 | Slamf8 | SLAM family member 8 | C00082 | L-Tyrosine |
| Tnfrsf9 | 21942 | Tnfrsf9 | "tumor necrosis factor receptor superfamily, member 9" | C00041 | L-Alanine |
| Wfdc17 | 100034251 | Wfdc17 | WAP four-disulfide core domain 17 | C00263 | L-Homoserine |
| Slc2a6 | 227659 | Slc2a6 | "solute carrier family 2 (facilitated glucose transporter), member 6" | C00212 | Adenosine |

| | | | | | |
|---|---|---|---|---|---|
| Csf2rb2 | 12984 | Csf2rb2 | "colony stimulating factor 2 receptor, beta 2, low-affinity (granulocyte-macrophage)" | C03668 | Malic acid |
| Tlr13 | 279572 | Tlr13 | toll-like receptor 13 | C00189 | Ethanolamine |
| Rrm2 | 20135 | Rrm2 | ribonucleotide reductase M2 | C01697 | Galactitol |
| Ube2c | 68612 | Ube2c | ubiquitin-conjugating enzyme E2C | C00256 | L-Lactic acid |
| Lair1 | 52855 | Lair1 | leukocyte-associated Ig-like receptor 1 | C15584 | Phenol |
| Sgo2a | 68549 | Sgo2a | shugoshin 2A | | |
| Fyb | 23880 | Fyb | FYN binding protein | | |
| Cxcl9 | 17329 | Cxcl9 | chemokine (C-X-C motif) ligand 9 | | |
| Fscn1 | 14086 | Fscn1 | fascin actin-bundling protein 1 | | |
| Kntc1 | 208628 | Kntc1 | kinetochore associated 1 | | |
| Cd180 | 17079 | Cd180 | CD180 antigen | | |
| Fcer1g | 14127 | Fcer1g | "Fc receptor, IgE, high affinity I, gamma polypeptide" | | |
| AB124611 | 382062 | AB124611 | cDNA sequence AB124611 | | |
| Ncf4 | 17972 | Ncf4 | neutrophil cytosolic factor 4 | | |
| Igsf6 | 80719 | Igsf6 | "immunoglobulin superfamily, member 6" | | |
| C3ar1 | 12267 | C3ar1 | complement component 3a receptor 1 | | |
| Mefv | 54483 | Mefv | Mediterranean fever | | |
| Lst1 | 16988 | Lst1 | leukocyte specific transcript 1 | | |
| Cdk1 | 12534 | Cdk1 | cyclin-dependent kinase 1 | | |
| Knl1 | 76464 | Knl1 | kinetochore scaffold 1 | | |
| Pla2g7 | 27226 | Pla2g7 | "phospholipase A2, group VII (platelet-activating factor acetylhydrolase, plasma)" | | |
| Ccl4 | 20303 | Ccl4 | chemokine (C-C motif) ligand 4 | | |
| Tpx2 | 72119 | Tpx2 | "TPX2, microtubule-associated" | | |
| Pirb | 18733 | Pirb | paired Ig-like receptor B | | |
| Mmp12 | 17381 | Mmp12 | matrix metallopeptidase 12 | | |
| Bcl2a1b | 12045 | Bcl2a1b | B cell leukemia/lymphoma 2 related protein A1b | | |

| | | | |
|---|---|---|---|
| Spi1 | 20375 | Spi1 | spleen focus forming virus (SFFV) proviral integration oncogene |
| Ccnb2 | 12442 | Ccnb2 | cyclin B2 |
| Gm15987 | 100504591 | Gm15987 | predicted gene 15987 |
| Sema4d | 20354 | Sema4d | "sema domain, immunoglobulin domain (Ig), transmembrane domain (TM) and short cytoplasmic domain, (semaphorin) 4D" |
| Cenpf | 108000 | Cenpf | centromere protein F |
| Ccl9 | 20308 | Ccl9 | chemokine (C-C motif) ligand 9 |
| Gbp5 | 229898 | Gbp5 | guanylate binding protein 5 |
| Atp1a3 | 232975 | Atp1a3 | "ATPase, Na+/K+ transporting, alpha 3 polypeptide" |
| Ntrk2 | 18212 | Ntrk2 | "neurotrophic tyrosine kinase, receptor, type 2" |
| Marcksl1 | 17357 | Marcksl1 | MARCKS-like 1 |
| Arl5c | 217151 | Arl5c | ADP-ribosylation factor-like 5C |
| Prkcb | 18751 | Prkcb | "protein kinase C, beta" |
| Hk3 | 212032 | Hk3 | hexokinase 3 |
| Spp1 | 20750 | Spp1 | secreted phosphoprotein 1 |
| Tnf | 21926 | Tnf | tumor necrosis factor |
| Prc1 | 233406 | Prc1 | protein regulator of cytokinesis 1 |
| Ccl12 | 20293 | Ccl12 | chemokine (C-C motif) ligand 12 |
| Gm6209 | 621304 | Gm6209 | predicted gene 6209 |
| Hmmr | 15366 | Hmmr | hyaluronan mediated motility receptor (RHAMM) |
| Gpr84 | 80910 | Gpr84 | G protein-coupled receptor 84 |
| Fcrls | 80891 | Fcrls | "Fc receptor-like S, scavenger receptor" |
| Slc11a1 | 18173 | Slc11a1 | "solute carrier family 11 (proton-coupled divalent metal ion transporters), member 1" |
| Frzb | 20378 | Frzb | frizzled-related protein |
| Zbp1 | 58203 | Zbp1 | Z-DNA binding protein 1 |
| 5430437J10Rik | 71432 | 5430437J10Rik | RIKEN cDNA 5430437J10 gene |

| | | | |
|---|---|---|---|
| Lag3 | 16768 | Lag3 | lymphocyte-activation gene 3 |
| Aspm | 12316 | Aspm | abnormal spindle microtubule assembly |
| Mthfd2 | 17768 | Mthfd2 | "methylenetetrahydrofolate dehydrogenase (NAD+ dependent), methenyltetrahydrofolate cyclohydrolase" |
| Ifitm6 | 213002 | Ifitm6 | interferon induced transmembrane protein 6 |
| Pclaf | 68026 | Pclaf | PCNA clamp associated factor |
| Birc5 | 11799 | Birc5 | baculoviral IAP repeat-containing 5 |
| Ubd | 24108 | Ubd | ubiquitin D |
| Nuf2 | 66977 | Nuf2 | "NUF2, NDC80 kinetochore complex component" |
| Gm44805 | 105886299 | Gm44805 | predicted gene 44805 |
| Cdca5 | 67849 | Cdca5 | cell division cycle associated 5 |
| Cdca3 | 14793 | Cdca3 | cell division cycle associated 3 |
| Dcstamp | 75766 | Dcstamp | dendrocyte expressed seven transmembrane protein |
| Igkv1-117 | 16098 | Igkv1-117 | immunoglobulin kappa variable 1-117 |
| Cxcl10 | 15945 | Cxcl10 | chemokine (C-X-C motif) ligand 10 |
| Cx3cr1 | 13051 | Cx3cr1 | chemokine (C-X3-C motif) receptor 1 |
| Il18bp | 16068 | Il18bp | interleukin 18 binding protein |
| Knstrn | 51944 | Knstrn | kinetochore-localized astrin/SPAG5 binding |
| Susd3 | 66329 | Susd3 | sushi domain containing 3 |
| Il1rn | 16181 | Il1rn | interleukin 1 receptor antagonist |
| Lrrc25 | 211228 | Lrrc25 | leucine rich repeat containing 25 |
| Spic | 20728 | Spic | Spi-C transcription factor (Spi-1/PU.1 related) |
| Zranb3 | 226409 | Zranb3 | "zinc finger, RAN-binding domain containing 3" |
| Mest | 17294 | Mest | mesoderm specific transcript |
| Pilrb1 | 170741 | Pilrb1 | paired immunoglobin-like type 2 receptor beta 1 |
| Il4i1 | 14204 | Il4i1 | interleukin 4 induced 1 |

| | | | |
|---|---|---|---|
| Tnip3 | 414084 | Tnip3 | TNFAIP3 interacting protein 3 |
| Pdcd1lg2 | 58205 | Pdcd1lg2 | programmed cell death 1 ligand 2 |
| Cst7 | 13011 | Cst7 | cystatin F (leukocystatin) |
| Clec4e | 56619 | Clec4e | "C-type lectin domain family 4, member e" |
| Kif22 | 110033 | Kif22 | kinesin family member 22 |
| Cd300e | 217306 | Cd300e | CD300E molecule |
| Cxcl13 | 55985 | Cxcl13 | chemokine (C-X-C motif) ligand 13 |
| Cep55 | 74107 | Cep55 | centrosomal protein 55 |
| Kif18b | 70218 | Kif18b | kinesin family member 18B |
| Gpr31b | 436440 | Gpr31b | "G protein-coupled receptor 31, D17Leh66b region" |
| Tm4sf19 | 277203 | Tm4sf19 | transmembrane 4 L six family member 19 |
| Gm16685 | 102634900 | Gm16685 | "predicted gene, 16685" |
| Ccnb1 | 268697 | Ccnb1 | cyclin B1 |
| Gm5547 | 433637 | Gm5547 | predicted gene 5547 |
| Bcl2a1d | 12047 | Bcl2a1d | B cell leukemia/lymphoma 2 related protein A1d |
| Siglece | 83382 | Siglece | sialic acid binding Ig-like lectin E |
| Prr11 | 270906 | Prr11 | proline rich 11 |
| Ncapg | 54392 | Ncapg | "non-SMC condensin I complex, subunit G" |
| Eya4 | 14051 | Eya4 | EYA transcriptional coactivator and phosphatase 4 |
| Cd244a | 18106 | Cd244a | CD244 molecule A |
| Rad51ap1 | 19362 | Rad51ap1 | RAD51 associated protein 1 |
| Hmox1 | 15368 | Hmox1 | heme oxygenase 1 |
| Shcbp1 | 20419 | Shcbp1 | Shc SH2-domain binding protein 1 |
| Htr2b | 15559 | Htr2b | 5-hydroxytryptamine (serotonin) receptor 2B |
| AI427809 | 381524 | AI427809 | expressed sequence AI427809 |

| | | | |
|---|---|---|---|
| AC166110.3 | NA | NA | NA |
| Ighv1-36 | 629897 | Ighv1-36 | immunoglobulin heavy variable 1-36 |
| Clec4a1 | 269799 | Clec4a1 | "C-type lectin domain family 4, member a1" |
| Kif20a | 19348 | Kif20a | kinesin family member 20A |
| A930007I19Rik | 77779 | A930007I19Rik | RIKEN cDNA A930007I19 gene |
| Cd300ld5 | 100043125 | Cd300ld5 | CD300 molecule like family member D5 |
| Iqgap3 | 404710 | Iqgap3 | IQ motif containing GTPase activating protein 3 |
| Adgre4 | 52614 | Adgre4 | adhesion G protein-coupled receptor E4 |
| Igkv4-86 | 243451 | Igkv4-86 | immunoglobulin kappa variable 4-86 |
| Kifc1 | 100502766 | Kifc1 | kinesin family member C1 |
| Slc7a11 | 26570 | Slc7a11 | "solute carrier family 7 (cationic amino acid transporter, y+ system), member 11" |
| Tubb2b | 73710 | Tubb2b | "tubulin, beta 2B class IIB" |
| Pif1 | 208084 | Pif1 | PIF1 5'-to-3' DNA helicase |
| Dnase1l3 | 13421 | Dnase1l3 | deoxyribonuclease 1-like 3 |
| Ighv14-2 | 668421 | Ighv14-2 | immunoglobulin heavy variable 14-2 |
| Nxpe5 | 381680 | Nxpe5 | "neurexophilin and PC-esterase domain family, member 5" |
| Calhm6 | 215900 | Calhm6 | calcium homeostasis modulator family member 6 |
| Pbk | 52033 | Pbk | PDZ binding kinase |
| Gm31814 | 102634162 | Gm31814 | "predicted gene, 31814" |
| Nxpe1-ps | 640268 | Nxpe1-ps | "neurexophilin and PC-esterase domain family, member 1, pseudogene" |
| Ms4a6c | 73656 | Ms4a6c | "membrane-spanning 4-domains, subfamily A, member 6C" |
| Stil | 20460 | Stil | Scl/Tal1 interrupting locus |
| Fabp7 | 12140 | Fabp7 | "fatty acid binding protein 7, brain" |
| Ms4a4a | 666907 | Ms4a4a | "membrane-spanning 4-domains, subfamily A, member 4A" |
| Trem3 | 58218 | Trem3 | triggering receptor expressed on myeloid cells 3 |

| | | | |
|---|---|---|---|
| Treml4 | 224840 | Treml4 | triggering receptor expressed on myeloid cells-like 4 |
| Ms4a6d | 68774 | Ms4a6d | "membrane-spanning 4-domains, subfamily A, member 6D" |
| Orm1 | 18405 | Orm1 | orosomucoid 1 |
| Cks2 | 66197 | Cks2 | CDC28 protein kinase regulatory subunit 2 |
| Serpina3f | 238393 | Serpina3f | "serine (or cysteine) peptidase inhibitor, clade A, member 3F" |
| Cd300ld | 217305 | Cd300ld | CD300 molecule like family member d |
| Serpina3i | 628900 | Serpina3i | "serine (or cysteine) peptidase inhibitor, clade A, member 3I" |
| Pilrb2 | 545812 | Pilrb2 | paired immunoglobin-like type 2 receptor beta 2 |
| Tnfrsf8 | 21941 | Tnfrsf8 | "tumor necrosis factor receptor superfamily, member 8" |
| Ccl3 | 20302 | Ccl3 | chemokine (C-C motif) ligand 3 |
| Pilra | 231805 | Pilra | paired immunoglobin-like type 2 receptor alpha |
| Slc26a10 | 216441 | Slc26a10 | "solute carrier family 26, member 10" |
| Lrg1 | 76905 | Lrg1 | leucine-rich alpha-2-glycoprotein 1 |
| Gm36161 | 102639975 | Gm36161 | "predicted gene, 36161" |
| Ear6 | 93719 | Ear6 | "eosinophil-associated, ribonuclease A family, member 6" |
| Gdf3 | 14562 | Gdf3 | growth differentiation factor 3 |
| Igkv6-15 | 108022 | Igkv6-15 | immunoglobulin kappa variable 6-15 |
| Art2a-ps | NA | NA | NA |
| Timp1 | 21857 | Timp1 | tissue inhibitor of metalloproteinase 1 |
| Ikbke | 56489 | Ikbke | inhibitor of kappaB kinase epsilon |
| Nos2 | 18126 | Nos2 | "nitric oxide synthase 2, inducible" |
| Plac8 | 231507 | Plac8 | placenta-specific 8 |
| Tmem26 | 327766 | Tmem26 | transmembrane protein 26 |
| Inhba | 16323 | Inhba | inhibin beta-A |
| S1pr5 | 94226 | S1pr5 | sphingosine-1-phosphate receptor 5 |

| | | | |
|---|---|---|---|
| Gm33858 | 102636917 | Gm33858 | "predicted gene, 33858" |
| Cd300ld4 | 100043123 | Cd300ld4 | CD300 molecule like family member D4 |
| Samsn1 | 67742 | Samsn1 | "SAM domain, SH3 domain and nuclear localization signals, 1" |
| Ccne1 | 12447 | Ccne1 | cyclin E1 |
| H2-M2 | 14990 | H2-M2 | "histocompatibility 2, M region locus 2" |
| 1700011B04Rik | 100503727 | 1700011B04Rik | RIKEN cDNA 1700011B04 gene |
| Ighv8-13 | 100775172 | Ighv8-13 | immunoglobulin heavy variable 8-13 |
| Pou2f2 | 18987 | Pou2f2 | "POU domain, class 2, transcription factor 2" |
| Npy | 109648 | Npy | neuropeptide Y |
| Gm13394 | 100033452 | Gm13394 | predicted gene 13394 |
| Stap1 | 56792 | Stap1 | signal transducing adaptor family member 1 |
| Gm21188 | 100861753 | Gm21188 | "predicted gene, 21188" |
| Rsad2 | 58185 | Rsad2 | radical S-adenosyl methionine domain containing 2 |
| Slc6a12 | 14411 | Slc6a12 | "solute carrier family 6 (neurotransmitter transporter, betaine/GABA), member 12" |
| Tiam2 | 24001 | Tiam2 | T cell lymphoma invasion and metastasis 2 |
| Smpdl3b | 100340 | Smpdl3b | "sphingomyelin phosphodiesterase, acid-like 3B" |
| Gm42815 | NA | NA | NA |
| Gm20056 | 100504089 | Gm20056 | "predicted gene, 20056" |
| Ccl2 | 20296 | Ccl2 | chemokine (C-C motif) ligand 2 |
| Adora2a | 11540 | Adora2a | adenosine A2a receptor |
| Ackr1 | 13349 | Ackr1 | atypical chemokine receptor 1 (Duffy blood group) |
| Cd300ld3 | 382551 | Cd300ld3 | CD300 molecule like family member D3 |
| Wnt6 | 22420 | Wnt6 | "wingless-type MMTV integration site family, member 6" |
| Cenpm | 66570 | Cenpm | centromere protein M |
| Lilr4b | 14727 | Lilr4b | "leukocyte immunoglobulin-like receptor, subfamily B, member 4B" |

| | | | |
|---|---|---|---|
| Igkv1-110 | 381777 | Igkv1-110 | immunoglobulin kappa variable 1-110 |
| Col22a1 | 69700 | Col22a1 | "collagen, type XXII, alpha 1" |
| Itgal | 16408 | Itgal | integrin alpha L |
| 2210406H18Rik | 70133 | 2210406H18Rik | RIKEN cDNA 2210406H18 gene |
| Spn | 20737 | Spn | sialophorin |
| I830127L07Rik | NA | NA | NA |
| Rgs5 | 19737 | Rgs5 | regulator of G-protein signaling 5 |
| Fgr | 14191 | Fgr | "FGR proto-oncogene, Src family tyrosine kinase" |
| Csf1r | 12978 | Csf1r | colony stimulating factor 1 receptor |
| Gdf15 | 23886 | Gdf15 | growth differentiation factor 15 |
| Fanci | 208836 | Fanci | "Fanconi anemia, complementation group I" |
| B830042I05Rik | 78692 | B830042I05Rik | RIKEN cDNA B830042I05 gene |
| Dtl | 76843 | Dtl | denticleless E3 ubiquitin protein ligase |
| Ska3 | 219114 | Ska3 | spindle and kinetochore associated complex subunit 3 |
| Kcnd1 | 16506 | Kcnd1 | "potassium voltage-gated channel, Shal-related family, member 1" |
| Myo1g | 246177 | Myo1g | myosin IG |
| Gm13571 | 102636093 | Gm13571 | predicted gene 13571 |
| Gpr35 | 64095 | Gpr35 | G protein-coupled receptor 35 |
| Scn2b | 72821 | Scn2b | "sodium channel, voltage-gated, type II, beta" |
| C920009B18Rik | 606736 | C920009B18Rik | RIKEN cDNA C920009B18 gene |
| Ceacam19 | 319930 | Ceacam19 | carcinoembryonic antigen-related cell adhesion molecule 19 |
| Trpc4 | 22066 | Trpc4 | "transient receptor potential cation channel, subfamily C, member 4" |
| Eno3 | 13808 | Eno3 | "enolase 3, beta muscle" |
| Gm28285 | NA | NA | NA |
| Ighv3-1 | 780803 | Ighv3-1 | immunoglobulin heavy variable 3-1 |

| | | | |
|---|---|---|---|
| Itga4 | 16401 | Itga4 | integrin alpha 4 |
| AC154492.2 | NA | NA | NA |
| Hdhd2 | 76987 | Hdhd2 | haloacid dehalogenase-like hydrolase domain containing 2 |
| Rgs16 | 19734 | Rgs16 | regulator of G-protein signaling 16 |
| Plek | 56193 | Plek | pleckstrin |
| Dkk2 | 56811 | Dkk2 | dickkopf WNT signaling pathway inhibitor 2 |
| Gngt2 | 14710 | Gngt2 | "guanine nucleotide binding protein (G protein), gamma transducing activity polypeptide 2" |
| Ighv1-81 | 668591 | Ighv1-81 | immunoglobulin heavy variable 1-81 |
| Ssu2 | 243612 | Ssu2 | ssu-2 homolog (C. elegans) |
| Neil3 | 234258 | Neil3 | nei like 3 (E. coli) |
| B430306N03Rik | 320148 | B430306N03Rik | RIKEN cDNA B430306N03 gene |
| Ksr2 | 333050 | Ksr2 | kinase suppressor of ras 2 |
| BC023105 | 667597 | BC023105 | cDNA sequence BC023105 |
| Nat8l | 269642 | Nat8l | N-acetyltransferase 8-like |
| Sh3gl3 | 20408 | Sh3gl3 | SH3-domain GRB2-like 3 |
| Pagr1a | 67278 | Pagr1a | PAXIP1 associated glutamate rich protein 1A |
| Igkv6-14 | 667881 | Igkv6-14 | immunoglobulin kappa variable 6-14 |
| Tgtp1 | 21822 | Tgtp1 | T cell specific GTPase 1 |
| Ighv8-9 | 432709 | Ighv8-9 | immunoglobulin heavy variable V8-9 |
| Arnt2 | 11864 | Arnt2 | aryl hydrocarbon receptor nuclear translocator 2 |
| Ccl7 | 20306 | Ccl7 | chemokine (C-C motif) ligand 7 |
| Sapcd2 | 72080 | Sapcd2 | suppressor APC domain containing 2 |
| Igkv3-1 | 108004 | Igkv3-1 | immunoglobulin kappa variable 3-1 |
| Depdc1a | 76131 | Depdc1a | DEP domain containing 1a |
| Rap1gap2 | 380711 | Rap1gap2 | RAP1 GTPase activating protein 2 |

| | | | |
|---|---|---|---|
| Gm11906 | NA | NA | NA |
| Cdc25c | 12532 | Cdc25c | cell division cycle 25C |
| Tmem158 | 72309 | Tmem158 | transmembrane protein 158 |
| Ighv8-8 | 780938 | Ighv8-8 | immunoglobulin heavy variable 8-8 |
| Ms4a4c | 64380 | Ms4a4c | "membrane-spanning 4-domains, subfamily A, member 4C" |
| Soat2 | 223920 | Soat2 | sterol O-acyltransferase 2 |
| Prrx2 | 20204 | Prrx2 | paired related homeobox 2 |
| F10 | 14058 | F10 | coagulation factor X |
| Lockd | 381822 | Lockd | lncRNA downstream of Cdkn1b |
| Col24a1 | 71355 | Col24a1 | "collagen, type XXIV, alpha 1" |
| Igkv4-61 | 546244 | Igkv4-61 | immunoglobulin kappa chain variable 4-61 |
| Il10 | 16153 | Il10 | interleukin 10 |
| Nfkbid | 243910 | Nfkbid | "nuclear factor of kappa light polypeptide gene enhancer in B cells inhibitor, delta" |
| Igkv13-64 | 628564 | Igkv13-64 | immunoglobulin kappa chain variable 13-64 |
| Eme1 | 268465 | Eme1 | essential meiotic structure-specific endonuclease 1 |
| BC030867 | NA | NA | NA |
| Htr7 | 15566 | Htr7 | 5-hydroxytryptamine (serotonin) receptor 7 |
| F9 | 14071 | F9 | coagulation factor IX |
| Skint3 | 195564 | Skint3 | selection and upkeep of intraepithelial T cells 3 |
| Il31ra | 218624 | Il31ra | interleukin 31 receptor A |
| Mrgpra2b | 235712 | Mrgpra2b | "MAS-related GPR, member A2B" |
| Ido1 | 15930 | Ido1 | "indoleamine 2,3-dioxygenase 1" |
| Fcrl5 | 329693 | Fcrl5 | Fc receptor-like 5 |
| Ighv9-3 | 780825 | Ighv9-3 | immunoglobulin heavy variable V9-3 |
| Ankrd37 | 654824 | Ankrd37 | ankyrin repeat domain 37 |

| | | | |
|---|---|---|---|
| Nr5a2 | 26424 | Nr5a2 | "nuclear receptor subfamily 5, group A, member 2" |
| Pnpla3 | 116939 | Pnpla3 | patatin-like phospholipase domain containing 3 |
| Pot1b | 72836 | Pot1b | protection of telomeres 1B |
| Igkv9-123 | 628144 | Igkv9-123 | immunoglobulin kappa variable 9-123 |
| Ighv1-7 | 668474 | Ighv1-7 | immunoglobulin heavy variable V1-7 |
| Fut2 | 14344 | Fut2 | fucosyltransferase 2 |
| Igkv5-48 | 619846 | Igkv5-48 | immunoglobulin kappa variable 5-48 |
| Cenph | 26886 | Cenph | centromere protein H |
| Grem1 | 23892 | Grem1 | "gremlin 1, DAN family BMP antagonist" |
| Igf2bp3 | 140488 | Igf2bp3 | insulin-like growth factor 2 mRNA binding protein 3 |
| Bcat1 | 12035 | Bcat1 | "branched chain aminotransferase 1, cytosolic" |
| Orm2 | 18406 | Orm2 | orosomucoid 2 |
| Ip6k3 | 271424 | Ip6k3 | inositol hexaphosphate kinase 3 |
| Igkv3-10 | 667924 | Igkv3-10 | immunoglobulin kappa variable 3-10 |
| Tubb3 | 22152 | Tubb3 | "tubulin, beta 3 class III" |
| Has2 | 15117 | Has2 | hyaluronan synthase 2 |
| Il1b | 16176 | Il1b | interleukin 1 beta |
| Cenps | 69928 | Cenps | centromere protein S |
| Gm49391 | NA | NA | NA |
| Gm42429 | NA | NA | NA |
| Ighv9-4 | 636260 | Ighv9-4 | immunoglobulin heavy variable 9-4 |
| Adamts4 | 240913 | Adamts4 | "a disintegrin-like and metallopeptidase (reprolysin type) with thrombospondin type 1 motif, 4" |
| Gm6377 | 622976 | Gm6377 | predicted gene 6377 |
| Prnd | 26434 | Prnd | prion like protein doppel |
| Gm34643 | 102637961 | Gm34643 | "predicted gene, 34643" |

| | | | |
|---|---|---|---|
| Cd5l | 11801 | Cd5l | CD5 antigen-like |
| Acoxl | 74121 | Acoxl | acyl-Coenzyme A oxidase-like |
| C7 | 109828 | C7 | complement component 7 |
| Tmem132d | 243274 | Tmem132d | transmembrane protein 132D |
| Scn3a | 20269 | Scn3a | "sodium channel, voltage-gated, type III, alpha" |
| Igfbp3 | 16009 | Igfbp3 | insulin-like growth factor binding protein 3 |
| Vipr2 | 22355 | Vipr2 | vasoactive intestinal peptide receptor 2 |
| Hes2 | 15206 | Hes2 | hes family bHLH transcription factor 2 |
| Bex2 | 12069 | Bex2 | brain expressed X-linked 2 |
| Ppp2r2c | 269643 | Ppp2r2c | "protein phosphatase 2, regulatory subunit B, gamma" |
| Prkg2 | 19092 | Prkg2 | "protein kinase, cGMP-dependent, type II" |
| Gria1 | 14799 | Gria1 | "glutamate receptor, ionotropic, AMPA1 (alpha 1)" |
| Pcdhac2 | 353237 | Pcdhac2 | "protocadherin alpha subfamily C, 2" |
| Cd209a | 170786 | Cd209a | CD209a antigen |
| Slc6a15 | 103098 | Slc6a15 | "solute carrier family 6 (neurotransmitter transporter), member 15" |
| Igkv1-122 | 434024 | Igkv1-122 | immunoglobulin kappa chain variable 1-122 |
| Colq | 382864 | Colq | collagen-like tail subunit (single strand of homotrimer) of asymmetric acetylcholinesterase |
| Bex4 | 406217 | Bex4 | brain expressed X-linked 4 |
| Olfm2 | 244723 | Olfm2 | olfactomedin 2 |
| Fam178b | 381337 | Fam178b | "family with sequence similarity 178, member B" |
| Gm14964 | 100008567 | Gm14964 | predicted gene 14964 |
| Fabp1 | 14080 | Fabp1 | "fatty acid binding protein 1, liver" |
| Gm42528 | NA | NA | NA |
| Rsg1 | NA | NA | NA |
| Galnt13 | 271786 | Galnt13 | polypeptide N-acetylgalactosaminyltransferase 13 |

| | | | |
|---|---|---|---|
| Sall1 | 58198 | Sall1 | spalt like transcription factor 1 |
| Gm32857 | 102635555 | Gm32857 | "predicted gene, 32857" |
| Gabre | 14404 | Gabre | "gamma-aminobutyric acid (GABA) A receptor, subunit epsilon" |
| Dpysl5 | 65254 | Dpysl5 | dihydropyrimidinase-like 5 |
| Lmntd1 | 74071 | Lmntd1 | lamin tail domain containing 1 |
| Gm42715 | NA | NA | NA |
| Sv2c | 75209 | Sv2c | synaptic vesicle glycoprotein 2c |
| Gm16263 | 102638835 | Gm16263 | predicted gene 16263 |
| Galnt5 | 241391 | Galnt5 | polypeptide N-acetylgalactosaminyltransferase 5 |
| Aloxe3 | 23801 | Aloxe3 | arachidonate lipoxygenase 3 |
| AA414992 | 102402 | AA414992 | expressed sequence AA414992 |
| Col17a1 | 12821 | Col17a1 | "collagen, type XVII, alpha 1" |
| Gm40773 | 105245298 | Gm40773 | "predicted gene, 40773" |
| Adcy1 | 432530 | Adcy1 | adenylate cyclase 1 |
| Gm44669 | NA | NA | NA |
| Gm29371 | 102635830 | Gm29371 | predicted gene 29371 |
| Gm43923 | NA | NA | NA |
| Dusp26 | 66959 | Dusp26 | dual specificity phosphatase 26 (putative) |
| Gm30648 | 102632621 | Gm30648 | "predicted gene, 30648" |
| Gm29609 | 115482680 | Gm29609 | predicted gene 29609 |
| Gabrb1 | 14400 | Gabrb1 | "gamma-aminobutyric acid (GABA) A receptor, subunit beta 1" |
| Il22ra1 | 230828 | Il22ra1 | "interleukin 22 receptor, alpha 1" |
| Lrrc9 | 78257 | Lrrc9 | leucine rich repeat containing 9 |
| Gpr17 | 574402 | Gpr17 | G protein-coupled receptor 17 |
| 2810030D12Rik | 78335 | 2810030D12Rik | RIKEN cDNA 2810030D12 gene |

| | | | |
|---|---|---|---|
| AC132307.1 | NA | NA | NA |
| Esr2 | 13983 | Esr2 | estrogen receptor 2 (beta) |
| Paqr9 | 75552 | Paqr9 | progestin and adipoQ receptor family member IX |
| Gm45713 | NA | NA | NA |
| Tmem88b | 320587 | Tmem88b | transmembrane protein 88B |
| Rasgrf1 | 19417 | Rasgrf1 | RAS protein-specific guanine nucleotide-releasing factor 1 |
