# Supplementary figures and images for: An Integrative Transcriptomic and Metabolomic Study Revealed That Melatonin Plays a Protective Role in Chronic Lung Inflammation by Reducing Necroptosis
Source: Front Immunol. 2021 May 4;12:668002. doi: 10.3389/fimmu.2021.668002 (PMC8129533; doi:10.3389/fimmu.2021.668002)

A

Score (PLS-DA) plot

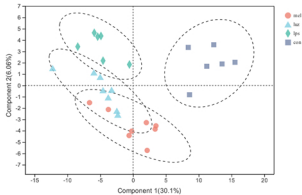

B

Permutation testing

R2=(0.04358), Q2=(0.-0.4317)

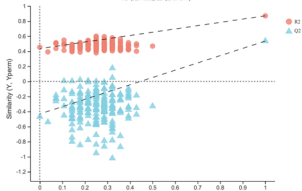

Supplement: Supplementary Figure 1 — Score plots of PLS-DA based on the metabolic profile of COPD. (A) The plot of PLS-DA scores showing almost complete separation of Mel (red circles), Luz (blue triangles), LPS (green rhombi), and Con (gray squares). The classification parameters were R2X (cum) = 0.65, R2Y (cum) = 0.871, and Q2 (cum) = 0.536. (B) Validation model of PLS-DA. The R2 and Q2 intercept values were 0.4356 and −0.4317, respectively, after 200 permutations. [file DataSheet_1.zip › Figure S1.pdf]

A

Expression Profile &amp; VIP of Metabolites

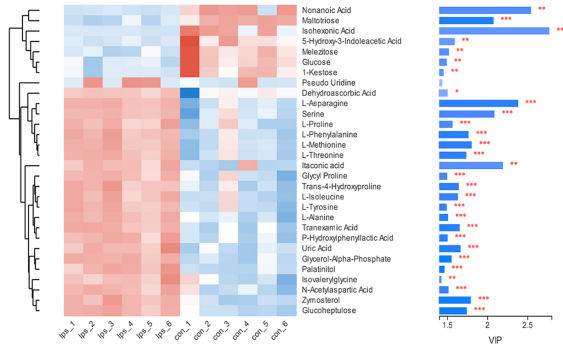

B

Expression Profile &amp; VIP of Metabolites

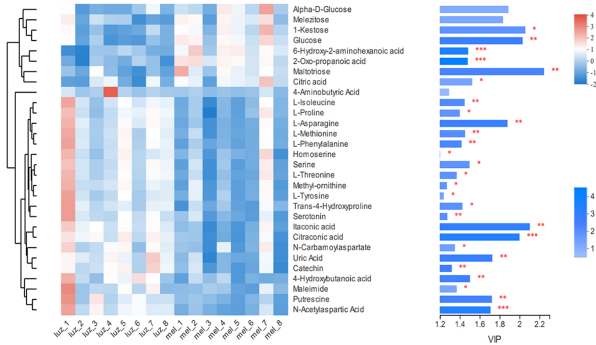

Supplement: Supplementary Figure 1 — Score plots of PLS-DA based on the metabolic profile of COPD. (A) The plot of PLS-DA scores showing almost complete separation of Mel (red circles), Luz (blue triangles), LPS (green rhombi), and Con (gray squares). The classification parameters were R2X (cum) = 0.65, R2Y (cum) = 0.871, and Q2 (cum) = 0.536. (B) Validation model of PLS-DA. The R2 and Q2 intercept values were 0.4356 and −0.4317, respectively, after 200 permutations. [file DataSheet_1.zip › Figure S2.pdf]
